# Both Nitro Groups Are Essential for High Antitubercular Activity of 3,5-Dinitrobenzylsulfanyl Tetrazoles and 1,3,4-Oxadiazoles through the Deazaflavin-Dependent Nitroreductase Activation Pathway

**DOI:** 10.1021/acs.jmedchem.3c00925

**Published:** 2023-12-29

**Authors:** Galina Karabanovich, Viktória Fabiánová, Anthony Vocat, Jan Dušek, Lenka Valášková, Jiřina Stolaříková, Russell R. A. Kitson, Petr Pávek, Kateřina Vávrová, Kamel Djaout, Katarína Mikušová, Alain R. Baulard, Stewart T. Cole, Jana Korduláková, Jaroslav Roh

**Affiliations:** † Charles University, Faculty of Pharmacy in Hradec Králové, Akademika Heyrovského 1203, 50005 Hradec Králové, Czech Republic; ‡ Faculty of Natural Sciences, Department of Biochemistry, Comenius University in Bratislava, Ilkovičova 6, Mlynská dolina, 842 15 Bratislava, Slovakia; § Global Health Institute, École Polytechnique Fédérale de Lausanne, 1015 Lausanne, Switzerland; ∥ Regional Institute of Public Health, Department of Bacteriology and Mycology, Partyzánské náměstí 7, 70200 Ostrava, Czech Republic; ⊥ Univ. Lille, CNRS, Inserm, CHU Lille, Institut Pasteur de Lille, U1019 - UMR 9017 - CIIL - Center for Infection and Immunity of Lille, F-59000 Lille, France

## Abstract

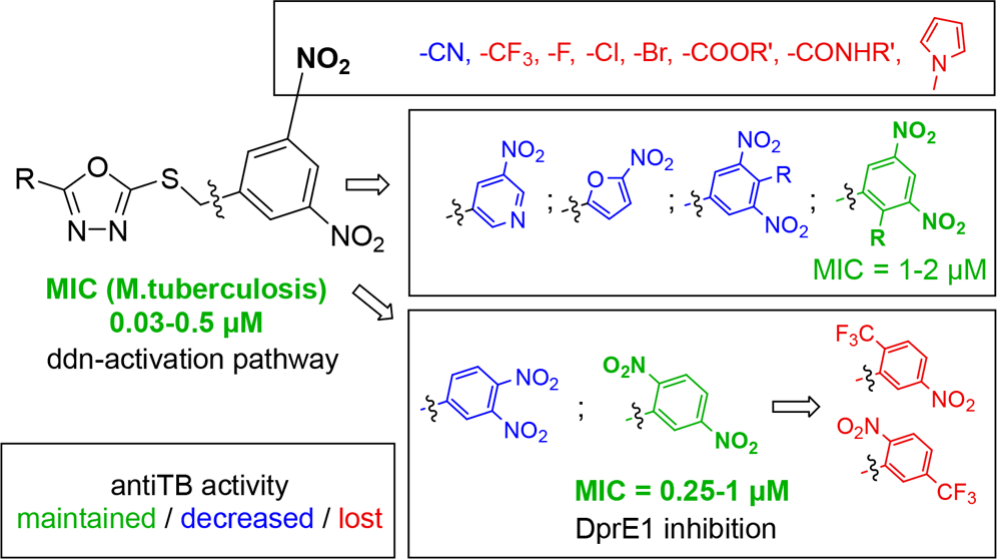

3,5-Dinitrobenzylsulfanyl tetrazoles and 1,3,4-oxadiazoles, previously identified as having high *in vitro* activities against both replicating and nonreplicating mycobacteria and favorable cytotoxicity and genotoxicity profiles were investigated. First we demonstrated that these compounds act in a deazaflavin-dependent nitroreduction pathway and thus *require* a nitro group for their activity. Second, we confirmed the necessity of *both* nitro groups for antimycobacterial activity through extensive structure–activity relationship studies using 32 structural types of analogues, each in a five-membered series. Only the analogues with shifted nitro groups, namely, 2,5-dinitrobenzylsulfanyl oxadiazoles and tetrazoles, maintained high antimycobacterial activity but in this case mainly as a result of DprE1 inhibition. However, these analogues also showed increased toxicity to the mammalian cell line. Thus, *both* nitro groups in 3,5-dinitrobenzylsulfanyl-containing antimycobacterial agents remain essential for their high efficacy, and further efforts should be directed at finding ways to address the possible toxicity and solubility issues, for example, by targeted delivery.

## Introduction

Although tuberculosis (TB) is a curable and preventable disease, it remains among the top causes of death worldwide and indeed recently became the second leading infectious killer after COVID-19. Moreover, the COVID-19 pandemic has reduced the access to TB diagnosis and treatment, which resulted in an increase in TB deaths. Only 6.4 million people newly diagnosed with TB were reported in 2021 from an estimated 10.6 million people who developed the disease, and the number of TB-related deaths increased from 1.4 million in 2019 to 1.6 million in 2021.^1^ The COVID-19 pandemic also reduced the number of people provided with treatment for drug-resistant TB by approximately 15%, and only one in three people with drug-resistant TB received treatment in 2020, with a slight recovery in 2021 (7.5% increase). Globally, approximately 3–4% of newly diagnosed TB cases are classified as multidrug-resistant strains (MDR-TB), and in the case of patients previously treated for TB, the proportion of MDR-TB is higher than 18%. Current therapy for drug-resistant TB has a low success rate (about 60% in 2019) and consists of prolonged multidrug regimens, which can last up to 24 months of taking five or more different anti-TB drugs.^1^ Such treatment regimens have many unpleasant side effects and drug–drug interactions (especially with antiretroviral drugs in the case of HIV co-infection) which cause poor compliance and hamper the coadministration of antiretroviral and anti-TB drugs. This, together with the fact that the most affected regions are those with relatively poor medical care, increases the risk of the formation and spread of MDR and extensively drug-resistant (XDR) strains. Therefore, addressing the availability and effectiveness of treatment for drug-resistant TB remains a major concern, and new, highly efficient, and better-tolerated drugs are needed. Recently, two nitro group-containing agents, delamanid^2^ and pretomanid,^3^ have been approved for the treatment of MDR/XDR-TB. Both these agents have a nitro group-dependent mechanism of action as they are bioreductively activated by deazaflavin-dependent nitroreductase (Ddn) in mycobacteria.^4^ Other compounds with nitro group-dependent antimycobacterial activity are the benzothiazinones,^5^ which are inhibitors of mycobacterial decaprenylphosphoryl-β-d-ribofuranose 2′-oxidase (DprE1).^6,7^ Two benzothiazinone derivatives, BTZ-043 and PBTZ-169 (macozinone), are currently undergoing evaluation in the clinic.^8^

**Figure 1:**
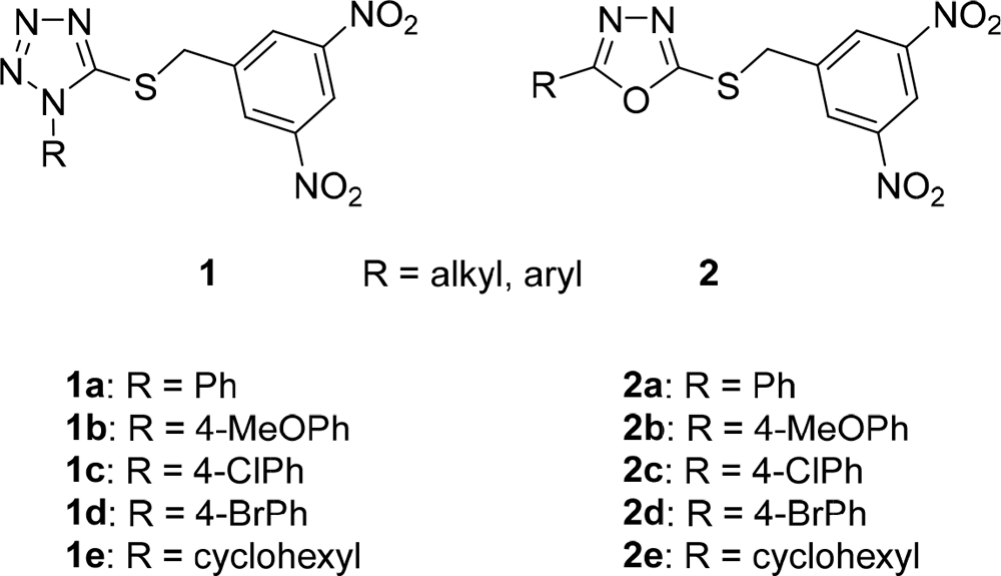
General structure of 3,5-dinitrobenzylsulfanyl tetrazole (**1**) and 1,3,4-oxadiazole (**2**) lead compounds.^12,13^ Compounds **1a**–**e** and **2a**–**e** served as parent and reference compounds in this study.

Our research group has developed several structural types of new antitubercular agents with high and selective antimycobacterial activity. These compounds typically contain a five-membered heterocycle and a 3,5-dinitrophenyl^9−11^ or 3,5-dinitrobenzylsulfanyl moiety.^12,13^ The latter group, 3,5-dinitrobenzylsulfanyl tetrazoles (**1**) and oxadiazoles (**2**) (Figure 1), showed excellent activity against both drug-susceptible and drug-resistant strains. The best oxadiazole derivatives of structure **2** had minimum inhibitory concentrations (MIC) of 0.03 μM against replicating *Mycobacterium tuberculosis* (*M.tb.*) strains and were also highly effective against the nonreplicating *M.tb*. SS18b-Lux strain.^13^ Interestingly, despite the structural similarity to known DprE1 inhibitors,^6,14^ 3,5-dinitrobenzylsulfanyl oxadiazoles **2** did not affect the function of this enzyme, and the actual mechanism of action remained elusive.^13^ The *in vitro* antimycobacterial efficiency of tetrazole derivatives **1** was lower compared to their oxadiazole counterparts; their MIC values reached 1 μM concentration_._^12^ Despite the presence of two nitro groups in the molecules, these lead compounds did not suffer from cytotoxicity to various cell lines, including isolated human hepatocytes, and did not exhibit genotoxicity in several assays.

The results of the above-mentioned studies indicated that the 3,5-dinitrobenzyl moiety is the fragment responsible for high *in vitro* antimycobacterial activity. It was found that 2,4-dinitrobenzyl isomers had substantially lower antimycobacterial activity compared to 3,5-dinitro compounds, and 3-nitro-5-(trifluoromethyl)benzyl or 3-amino-5-nitrobenzyl analogues lost antimycobacterial activity altogether.^12,13,15^ However, the presence of two nitro groups could be the main obstacle to the further development of these potent antimycobacterial agents. Despite the long history of nitro-containing drugs and recent findings of bioreductive activation,^16^ medicinal chemists typically try to avoid nitro groups in drug design due to concerns about toxicity and solubility.

**Figure 2:**
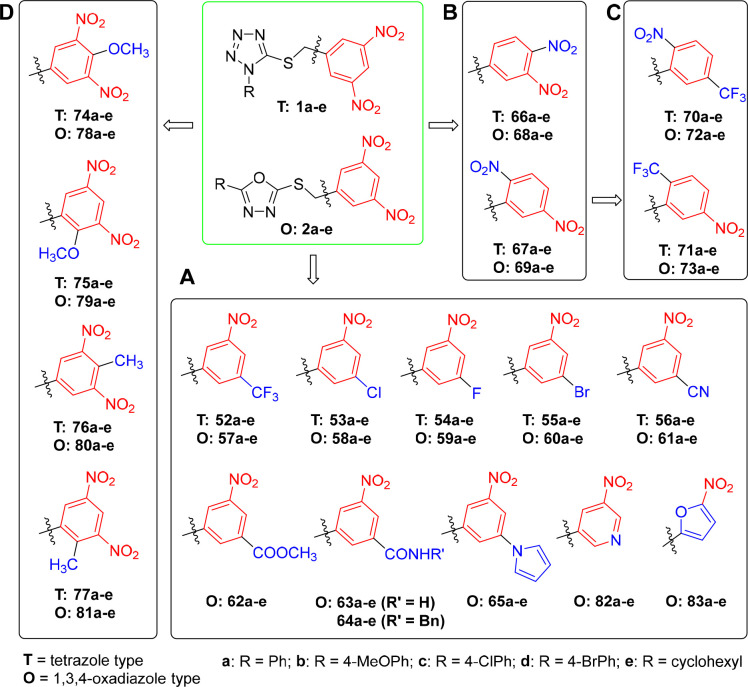
General structures of investigated tetrazole and 1,3,4-oxadiazole derivatives with replaced nitro group (A), with shifted nitro group (B), and their trifluoromethyl analogues (C) or those with methyl- or methoxy-substituted 3,5-dinitrobenzyl moiety (D). Tetrazoles **1a**–**e** and oxadiazoles **2a**–**e** served as the lead compounds in this study.

Therefore, the first aim of this work was to elucidate the mode of action of oxadiazoles **2** to (a) determine whether the presence of a nitro group is essential for antimycobacterial activity and (b) rationalize the design of new analogues. Second, the structure–activity relationships were explored. In Part A (Figure 2A), one nitro group in the two lead compounds 3,5-dinitrobenzylsulfanyl tetrazole (**1**) and oxadiazole (**2**) was replaced by other electron withdrawing groups. Thus, chloro-, fluoro-, bromo-, cyano-, methoxycarbonyl-, carbamoyl-, and pyrrol-1-yl- analogues of tetrazoles **1a**–**e** and/or oxadiazoles **2a**–**e** were prepared, and their *in vitro* antimycobacterial activities were evaluated. Trifluoromethyl analogues were also prepared to complete the series.^13^ The 3,5-dinitrobenzyl moiety was also replaced by a heterocyclic (5-nitropyridin-3-yl)methyl or (5-nitrofuran-2-yl)methyl group.

Parts B and C of the structure–activity relationship study focused on the position of the nitro groups on the benzyl moiety, which appeared to be crucial for the antimycobacterial activity of compounds **1** and **2**. In addition to the previously investigated 2,4-dinitrobenzyl analogues, in this work we shifted just one nitro group of the parent compounds **1a**–**e** and **2a**–**e** and prepared their 3,4- and 2,5-dinitrobenzyl analogues (Figure 2B). As the preliminary experiments showed high *in vitro* antimycobacterial activity of the compounds with the 2,5-dinitrobenzyl moiety, their mononitro analogues with 2-nitro-5-(trifluoromethyl)benzyl and 5-nitro-2-(trifluoromethyl)benzyl groups were also synthesized (Figure 2C).

In part D, a methyl or methoxy group was introduced to the 3,5-dinitrobenzyl moiety to explore the effect of additional substitution and steric hindrance of one or both neighboring nitro groups on the antimycobacterial activity of lead compounds (Figure 2D). Structure–activity relationships with respect to the substituent R on the tetrazole or oxadiazole core have been fully elucidated in our previous studies;^11−13^ thus in this work we selected five lipophilic substituents R (**a**–**e**, Figure 2) and used them in all series prepared and studied in this work to obtain easily comparable results.

## Results and Discussion

### Mode of Action of 3,5-Dinitrobenzylsulfanyl Oxadiazoles **2**

Previously we proved that 3,5-dinitrobenzylsulfanyl oxadiazoles and thiadiazoles do not affect the mycobacterial DprE1 and may target the synthesis of mycobacterial nucleic acids.^13^ To elucidate the mechanism of action of these compounds, mutants of *M.tb*. Erdman resistant to 3,5-dinitrobenzylsulfanyl 1,3,4-oxadiazole T6030 (**11i** in ref (13)) and 1,3,4-thiadiazole T6053 (**14g** in ref (13)) were generated using concentrations 10 times and 20 times higher than their MIC values. Whole genome sequencing followed by bioinformatics analysis showed that all mutant colonies carried a different nonsynonymous single nucleotide polymorphism in the *fgd1* gene (*rv0407*) encoding F_420_-dependent glucose-6-phosphate dehydrogenase (FGD1) (Table 1), similarly as in *M.tb*. mutants resistant to nitroimidazoles pretomanid and delamanid,^17−19^ FDA-approved anti-TB drugs. Mutations in FGD1 disrupt the reduction of cofactor F_420_ to F_420_-H_2_, which inhibits the function of Ddn and blocks the reductive activation of nitroimidazoles.^17^

**Table 1:** Resistance Profile and Mutations in *M.tb.* Erdman Mutants Exhibiting Resistance to 3,5-Dinitrobenzylsulfanyl Oxadiazole T6030 and Thiadiazole T6053

*M.tb*. strain	nucleotide change in *fgd1*	amino acid change in FGD1	T6030/T6053 MIC (μM)
Erdman			0.1
*T6030*-10×	g310c	Gly104Arg	8.1
*T6030*-20×	c949t	Gln317^a^	6.7
*T6053*-10×	g911t	Gly304Val	5.6
*T6053*-20×	c863a	Ser288^a^	3.4

a Truncated protein.

To further confirm that the antimycobacterial activity of compounds T6030 and T6053 rely on the Ddn-activation, we determined the MIC values in Ddn- and FbiC-deficient *M.tb*. mutants. We found that both mutant strains showed resistance to both T6030 and T6053 (>3- and 10-fold increase in MIC values, respectively, compared to wild-type *M.tb*. H37Rv), as well as to pretomanid.

**Figure 3:**
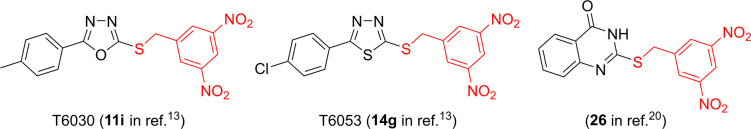
3,5-Dinitrobenzylsulfanyl-substituted oxadiazole T6030, thiadiazole T6053, and previously studied quinazolinone (**26** in ref (21)) whose Ddn-dependent mechanism of antimycobacterial activity was independently proven.

These results indicated that 3,5-dinitrobenzylsulfanyl oxadiazoles **2** are activated in a similar way as nitroimidazoles pretomanid and delamanid and proved that their antimycobacterial activity is nitro group-dependent. This conclusion is in agreement with a recent study of van Calenbergh et al., who experimentally proved that the antimycobacterial activity of closely related quinazolinones bearing the key 3,5-dinitrobenzylsulfanyl group depends on the reductive activation of the 3,5-dinitrobenzyl moiety by Ddn as in the case of the nitroimidazoles (Figure 3).^20^

These findings proved that at least one nitro group must be maintained in the structure of 3,5-dinitrobenzylsulfanyl heterocycles such as tetrazoles **1** and oxadiazoles **2** and drove the design of their mononitro analogues prepared in this work.

### Chemistry Part A

**Scheme 1:**
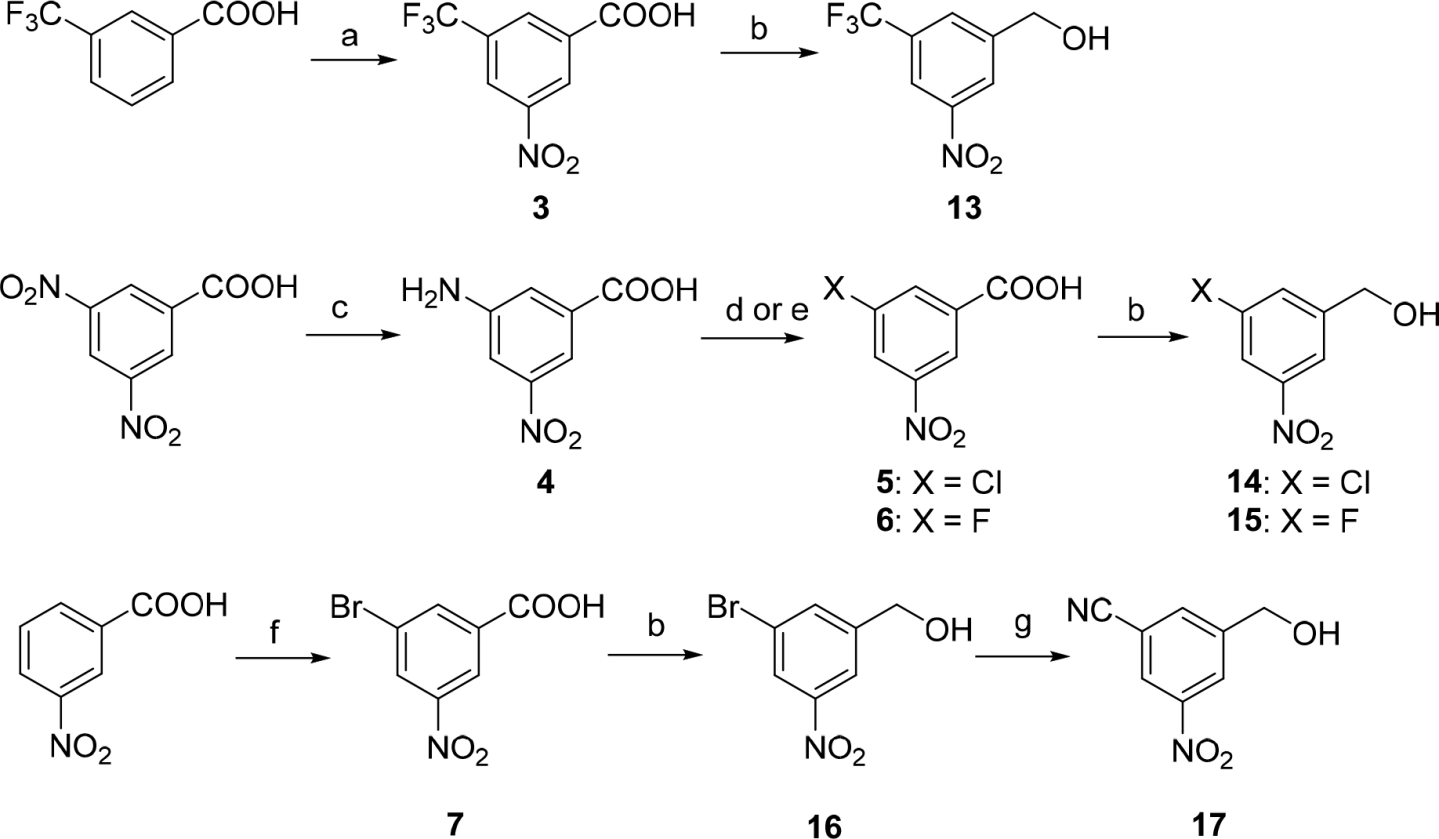
Synthesis of Trifluoromethyl-, Fluoro-, Chloro-, Bromo-, and Cyano-Substituted Nitrobenzyl Alcohols (**13**–**17**) Reagents and conditions: (a) fuming HNO_3_, H_2_SO_4_, 0 °C → rt, overnight, 87%; (b) BH_3_·THF, THF, −20 °C → rt, overnight, 79–98%; (c) Na_2_S·H_2_O, NH_4_Cl, CH_3_OH, reflux, 17 h, 92%; (d) X = Cl: NaNO_2_, CuCl, HCl, H_2_O, −5 °C; 3 h, rt; 30 min, 65 °C; 78%; (e) X = F: NOBF_4_, CH_3_CN, argon, 5 °C; 48 h, rt; 1,2-Cl_2_C_6_H_3_, 170 °C, 40 min, 61%; (f) NBS, H_2_SO_4_, 60 °C, 2 h, 87%; (g) K_4_[Fe(CN)_6_]·3H_2_O, Pd(OAc)_2_, Na_2_CO_3_, DMAC, 120 °C, 6 h, 30%.

**Scheme 2:**
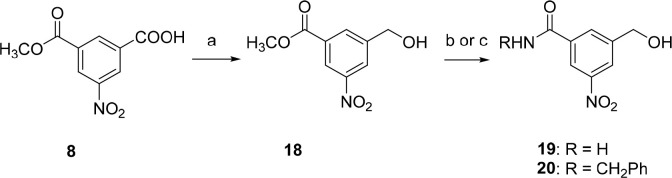
Synthesis of Methyl 3-(Hydroxymethyl)-5-nitrobenzoate (**18**) and 3-(Hydroxymethyl)-5-nitrobenzamides **19** and **20** from 3-Methoxycarbonyl-5-nitrobenzoic Acid (**8**) Reagents and conditions: (a) BH_3_·THF, THF, −20 °C→ rt, overnight, 76%; (b) R = H: NH_3_, CH_3_OH, autoclave reactor, 80 °C, 32 h, 71%; (c) R = CH_2_Ph: PhCH_2_NH_2_, CH_3_OH, autoclave reactor, 120 °C, 40 h, 64%.

Synthesis of the compounds with one trifluoromethyl- (**52a**–**e**, **57a**–**e**), chloro- (**53a**–**e**, **58a**–**e**), fluoro- (**54a**–**e**, **59a**–**e**), bromo- (**55a**–**e**, **60a**–**e**), cyano- (**56a**–**e**, **61a**–**e**), methoxycarbonyl- (**62a**–**e**), carbamoyl- (**63a**–**e**), or *N*-benzylcarbamoyl-group (**64a**–**e**) started with the preparation of the corresponding 3-nitro-5-substituted benzoic acids **3**–**8**, followed by the reduction of the carboxylic acid group using borane in THF (Scheme 1 and 2).^21^

3-Nitro-5-trifluoromethylbenzoic acid (**3**) was obtained by nitration of 3-trifluoromethylbenzoic acid in excellent yield (Scheme 1).^13^ Synthesis of 3-chloro- (**5**) or 3-fluoro-5-nitrobenzoic acid (**6**) started from 3,5-dinitrobenzoic acid. Its reduction by sodium sulfide hydrate in the presence of ammonium chloride provided 3-amino-5-nitrobenzoic acid **4**, which, after diazotization and substitution with chlorine or fluorine gave acids **5** and **6**, respectively. 3-Bromo-5-nitrobenzoic acid (**7**) was prepared via bromination of 5-nitrobenzoic acid with *N*-bromosuccinimide (NBS) in the presence of sulfuric acid in 87% yield.^22^ Final reduction of nitrobenzoic acids **3** and **5**–**7** led to the corresponding benzyl alcohols **13**–**16** in high yields (79–98%). 3-Cyano-5-nitrobenzyl alcohol **17** was prepared from 3-bromo-5-nitrobenzyl alcohol (**16**) by palladium-catalyzed cyanation (Scheme 1).^23^

The synthesis of 3-methoxycarbonyl and 3-carbamoyl 5-nitrobenzyl alcohols (**18**–**20**) started with partial esterification of 5-nitroisophthalic acid. 3-Methoxycarbonyl-5-nitrobenzoic acid (**8**) was obtained in a mixture with dimethyl 5-nitroisophthalate and 5-nitroisophthalic acid and therefore was isolated in modest yield (42%). Nitrobenzoic acid **8** underwent the reduction to provide methyl 3-(hydroxymethyl)-5-nitrobenzoate (**18**) in 76% yield. Aminolysis of methyl benzoate **18** with ammonia or benzylamine in an autoclave resulted in the desired carbamoyl derivatives **19** and **20**, respectively. (Scheme 2).

**Scheme 3:**
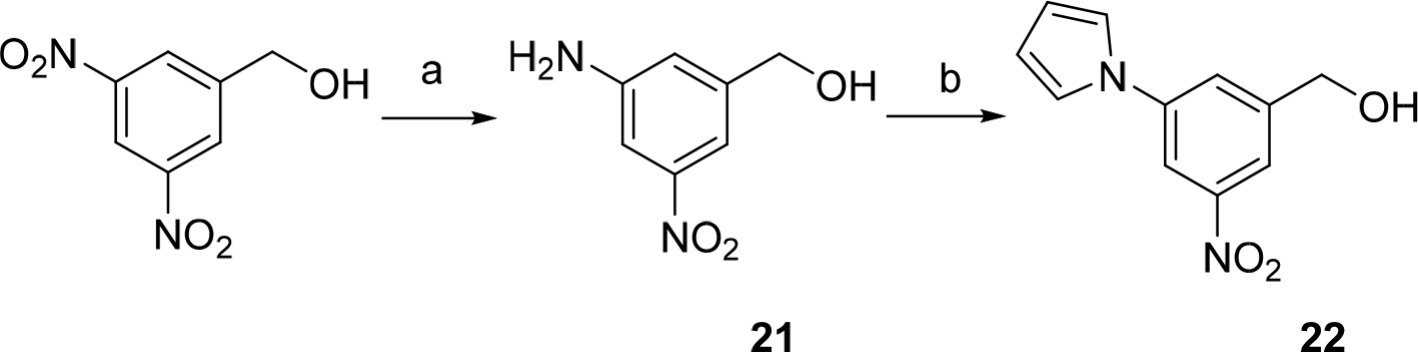
Synthesis of 3-Nitro-5-(1*H*-pyrrol-1-yl)benzyl Alcohol (**22**) Reagents and conditions: (a) Na_2_S·H_2_O, NH_4_Cl, CH_3_OH, reflux, 15 h, 70%; (b) 2,5-dimethoxytetrahydrofuran, THF/CH_3_COOH (2:1), reflux, 24 h, 68%.

The synthetic approach to 3-nitro-5-(1*H*-pyrrol-1-yl)benzyl alcohol (**22**) consisted of two steps. First, 3,5-dinitrobenzyl alcohol was partially reduced by sodium sulfide hydrate in the presence of ammonium chloride in methanol. In the second step, reaction of 3-amino-5-nitrobenzyl alcohol (**21**) with 2,5-dimethoxytetrahydrofuran led to the formation of the pyrrole derivative **22** in 68% yield (Scheme 3).

**Scheme 4:**
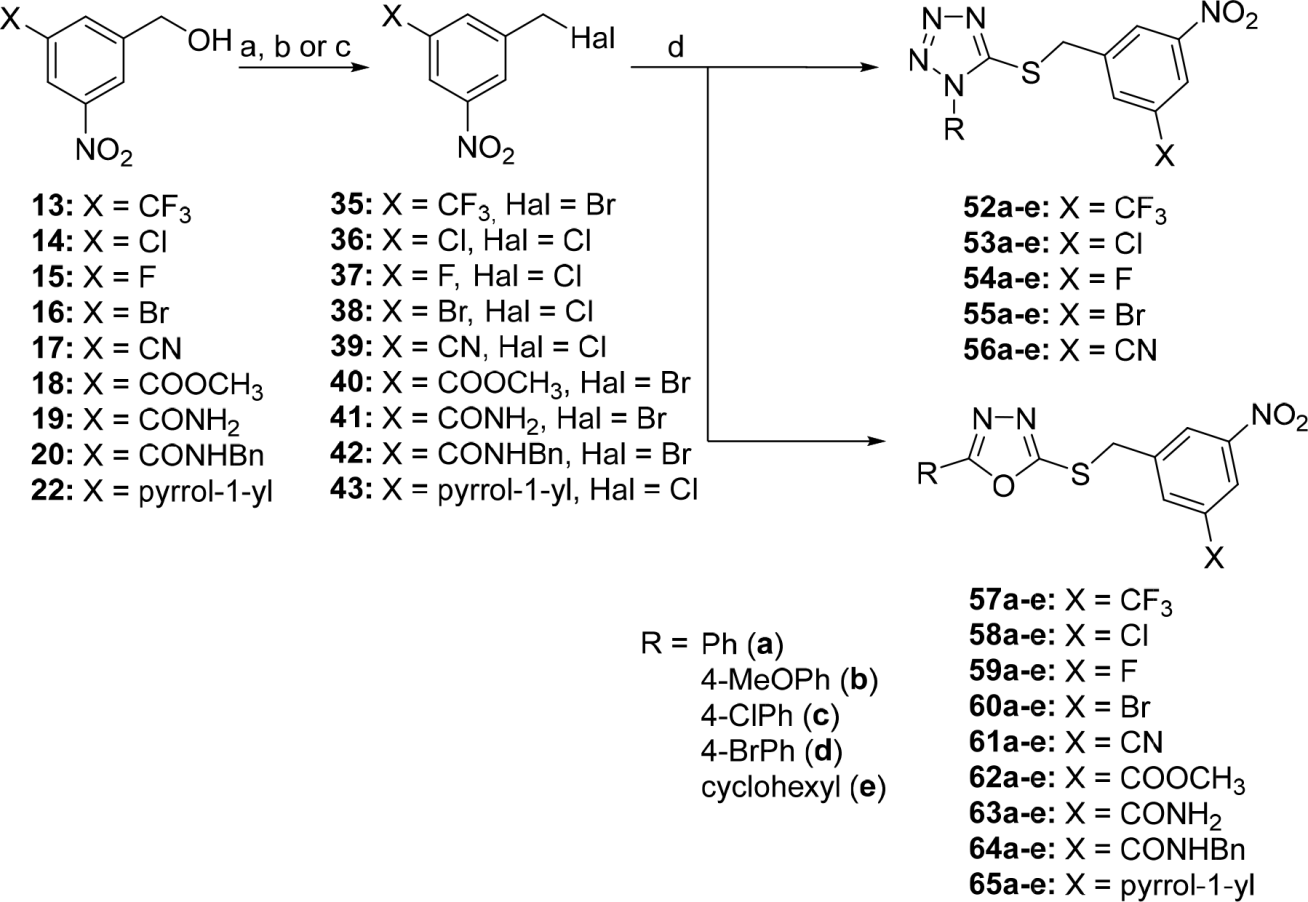
Synthesis of 3-Nitro-5-(trifluoromethyl)benzyl Derivatives **52a**–**e** and **57a**–**e**, 3-Chloro-5-nitrobenzyl Derivatives **53a**–**e** and **58a**–**e**, 3-Fluoro-5-nitrobenzyl Derivatives **54a**–**e** and **59a**–**e**, 3-Bromo-5-nitrobenzyl Derivatives **55a**–**e** and **60a**–**e**, 3-Cyano-5-nitrobenzyl Derivatives **56a**–**e** and **61a**–**e**, 3-Methoxycarbonyl-5-nitrobenzyl Derivatives **62a**–**e**, 3-Carbamoyl-5-nitrobenzyl Derivatives **63a**–**e**, 3-(Benzylcarbamoyl)-5-nitrobenzyl Derivatives **64a**–**e**, and 3-Nitro-5-(1*H*-pyrrol-1-yl)benzyl Derivatives **65a**–**e** Reagents and conditions: (a) for **36**, **37**, and **43**: SOCl_2_, Et_3_N, CH_2_Cl_2_, 0 °C–rt, 3 h, 74–79%; (b) for **38** and **39**: PCl_5_, CHCl_3_, 0 °C–reflux, 24 h, 70–89%; (c) for **35**, **40**–**42**: NBS, Ph_3_P, CH_2_Cl_2_, 0 °C–rt, 30 min–2 h, 40–94%; (d) 1-substituted 1*H*-tetrazole-5-thiol or 5-substituted 1,3,4-oxadiazole-2-thiol, Et_3_N, CH_3_CN, rt, 0.5–1 h or overnight, 53–98%.

Benzyl alcohols **13**–**20** and **22** were further converted to the corresponding benzyl halides **35**–**43**, which were used for the alkylations of the corresponding 1-substituted 1*H*-tetrazole-5-thiols and 5-substituted 1,3,4-oxadiazole-2-thiols (Scheme 4). The alkylation reactions were carried out in acetonitrile using triethylamine as a base, with the final 3-substituted 5-nitrobenzylsulfanyl tetrazoles **52**–**56** and oxadiazoles **57**–**65** obtained in high yields (53–98%).

**Scheme 5:**
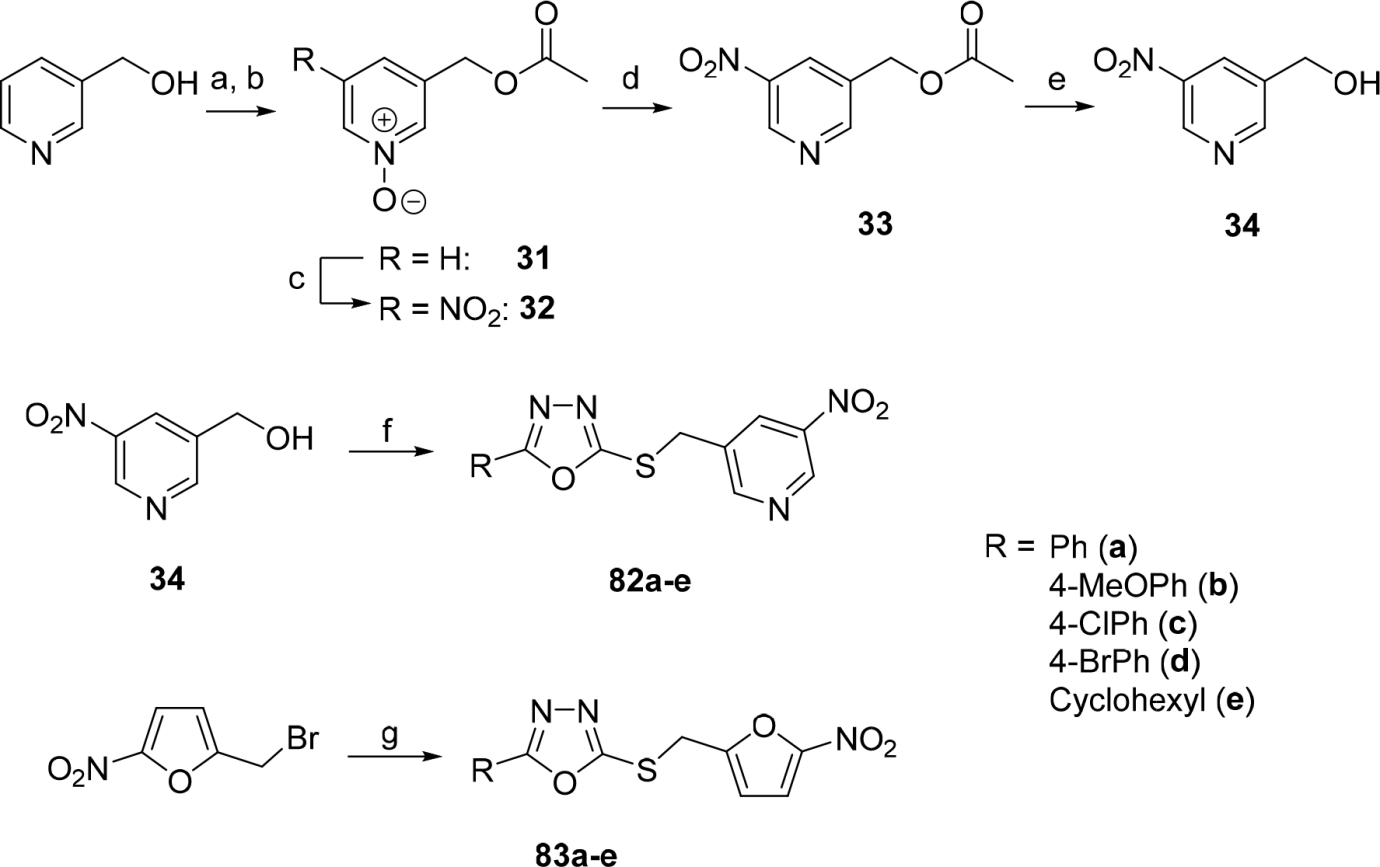
Synthesis 2-Alkyl/Aryl-5-((5-nitropyridin-3-yl)methylsulfanyl)-1,3,4-oxadiazoles **82a**–**e** and 2-Aryl-5-((5-nitrofuran-2-yl)methylsulfanyl)-1,3,4-oxadiazoles **83a**–**e** Reagents and conditions: (a) acetic anhydride, rt, 30 min; 65 °C, 1 h; (b) *m*CPBA, rt, overnight, 75% (two steps); (c) 4-NO_2_PhCOCl, AgNO_3_, CH_2_Cl_2_, −5 °C → reflux, 48 h, 16%; (d) PCl_3_, CH_2_Cl_2_, rt, 1 h, 70%; (e) H_2_SO_4_, H_2_O, THF, 85 °C, 15 h, 95%; (f) 1. SOCl_2_, CH_2_Cl_2_, rt, 5 h; 2. 5-substituted-1,3,4-oxadiazole-2-thiol, Et_3_N, THF, rt, overnight, 48–74%; (g) 5-aryl-1,3,4-oxadiazole-2-thiol, Et_3_N, CH_3_CN, rt, 30 min, 43–73%.

The synthesis of 2-alkyl/aryl-5-((5-nitropyridin-3-yl)methylsulfanyl)-1,3,4-oxadiazoles **82a**–**e** started from commercially available 3-pyridinemethanol, which was converted to 3-acetoxymethylpyridine-*N*-oxide (**31**) via reactions with *m*CPBA in acetic anhydride.^24^ Nitration of *N*-oxide **31** using silver nitrate and 4-nitrobenzoyl chloride in dry dichloromethane gave the nitro-derivative **32** in 16% yield.^25^ The reduction of 3-acetoxymethyl-5-nitropyridine-*N*-oxide (**32**) by PCl_3_ followed by acid hydrolysis resulted in (5-nitropyridin-3-yl)methanol (**34**) in 67% yield over two steps. (5-Nitropyridin-3-yl)methanol **34** was converted to 3-(chloromethyl)-5-nitropyridine hydrochloride, which was directly used for the alkylation of 5-substituted 1,3,4-oxadiazole-2-thiols. The final (5-nitropyridin-3-yl)methylsulfanyl 1,3,4-oxadiazoles **82a**–**e** were obtained in good yield (48–74%). The last series of studied compounds, 2-aryl-5-((5-nitrofuran-2-yl)methylsulfanyl)-1,3,4-oxadiazoles **83a**–**e**, was prepared by the alkylation of 5-aryl-1,3,4-oxadiazole-2-thiols with commercially available 5-(bromomethyl)-2-nitrofuran in the presence of triethylamine in acetonitrile, with the final compounds **83a**–**e** obtained in 43–73% yield (Scheme 5).

### Chemistry Part B

**Scheme 6:**
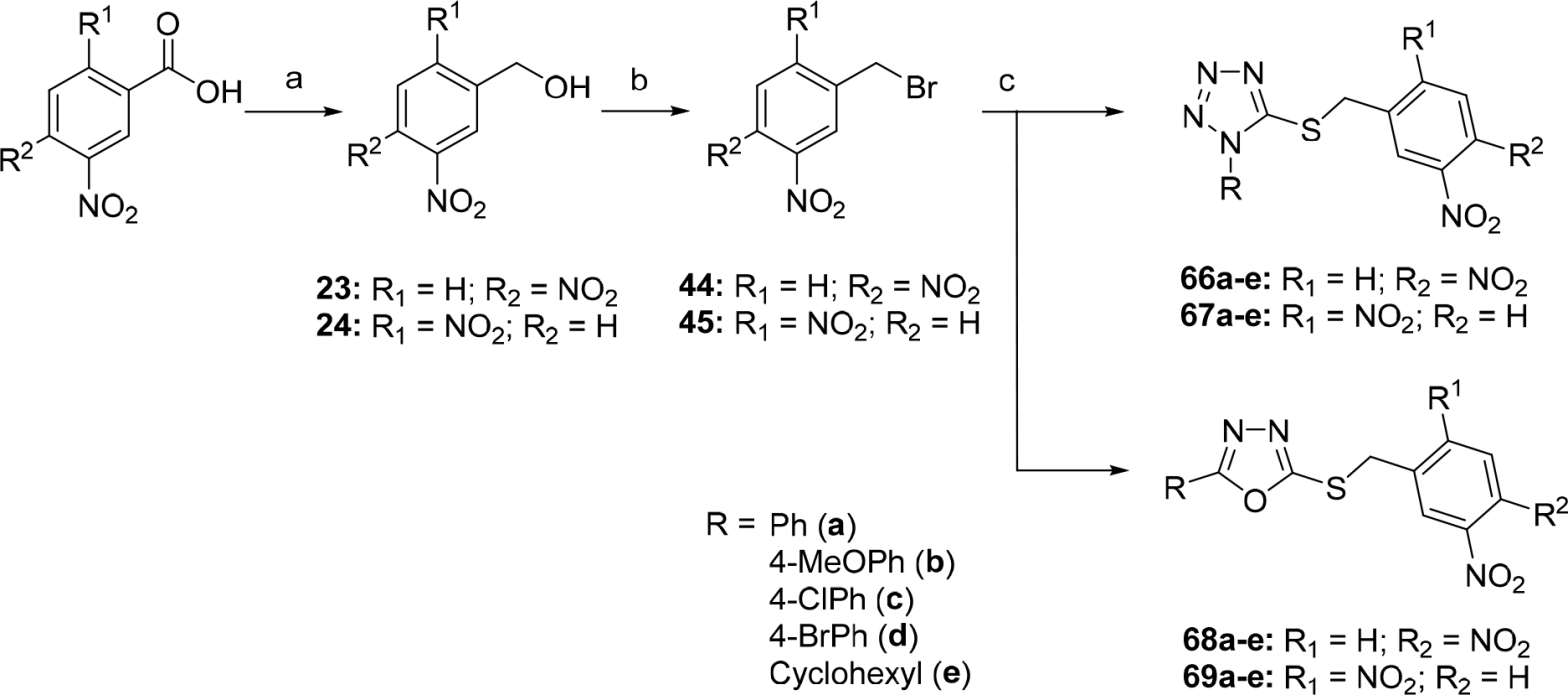
Synthesis of Final 3,4-Dinitrobenzylsulfanyl Tetrazoles **66a**–**e** and Oxadiazoles **68a**–**e** and 2,5-Dinitrobenzylsulfanyl Tetrazoles **67a**–**e** and Oxadiazoles **69a**–**e** Reagents and conditions: (a) BH_3_·THF, THF, −20 °C → rt, overnight, 63–85%; (b) NBS, Ph_3_P, CH_2_Cl_2_, 0 °C → rt, 1 h, 92–93%; (c) 1-substituted 1*H*-tetrazole-5-thiol or 5-substituted 1,3,4-oxadiazole-2-thiol, Et_3_N, CH_3_CN, 0.5–1 h, rt, 61–88%.

The synthesis of compounds with a shifted nitro group (**66**–**69**) is shown in Scheme 6. First, the appropriate dinitro-substituted benzyl alcohols **23** and **24** were prepared via the borane-mediated reductions of commercially available 3,4-dinitro or 2,5-dinitrobenzoic acids, respectively. These benzyl alcohols were converted to the corresponding benzyl bromides **44** and **45** by their reactions with NBS and PPh_3_ in dichloromethane.^24,26^ Benzyl bromides **44** and **45** were used to alkylate 1-substituted 1*H*-tetrazole-5-thiols and 5-substituted 1,3,4-oxadiazole-2-thiols to provide the final compounds of series **66**–**69** in high yields (61–88%). The alkylation was carried out in acetonitrile with triethylamine as a base (Scheme 6).

### Chemistry Part C

**Scheme 7:**
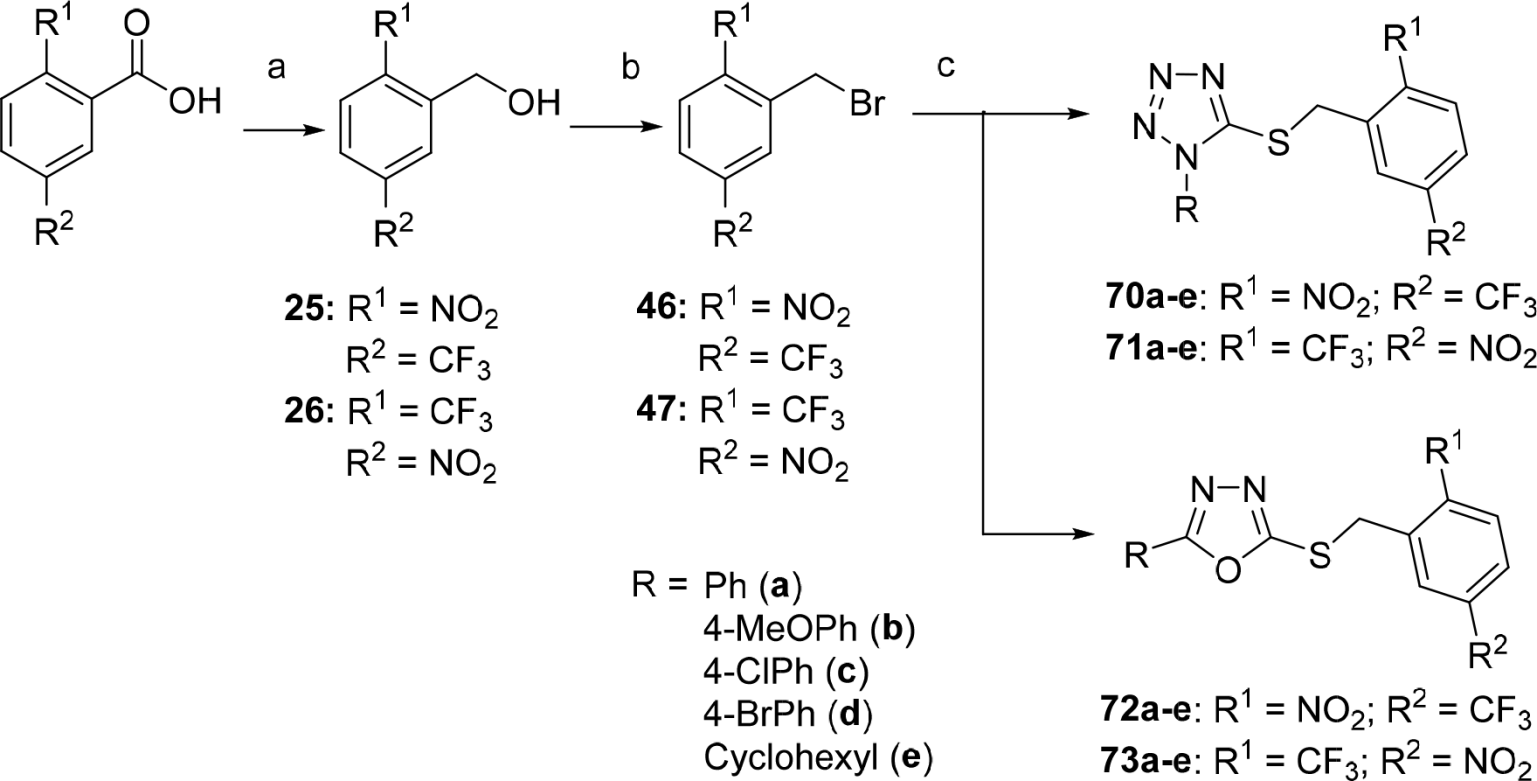
Synthesis of the Series of Final 2-Nitro-5-(trifluoromethyl)benzylsulfanyl Tetrazoles **70a**–**e** and Oxadiazoles **72a**–**e** and 5-Nitro-2-(trifluoromethyl)benzylsulfanyl Tetrazoles **71a**–**e** and Oxadiazoles **73a**–**e** Reagents and conditions: (a) BH_3_·THF, THF, −20 °C → rt, overnight, 93%; (b) NBS, Ph_3_P, CH_2_Cl_2_, 0 °C → rt, 1 h, 78–80%; (c) 1-substituted 1*H*-tetrazole-5-thiol or 5-substituted 1,3,4-oxadiazole-2-thiol, Et_3_N, CH_3_CN, rt, 1–2 h, 67–98%.

To prepare the 2-nitro-5-(trifluoromethyl)benzyl (**70a**–**e** and **72a**–**e**) or 5-nitro-2-(trifluoromethyl)benzyl (**71a**–**e** and **73a**–**e**) derivatives, commercially available 2-nitro-5-(trifluoromethyl) or 5-nitro-2-(trifluoromethyl)benzoic acids were used as the starting materials, respectively. They were first reduced to the benzyl alcohols **25** and **26** and then converted to benzyl bromides **46** and **47** by their reactions with NBS and PPh_3_ in dichloromethane. In the last step of synthesis, alkylations of the corresponding tetrazole-5-thiols or 1,3,4-oxadiazol-2-thiols resulted in the target compounds of series **70**–**73** in high yields (67–98%) (Scheme 7).

### Chemistry Part D

**Scheme 8:**
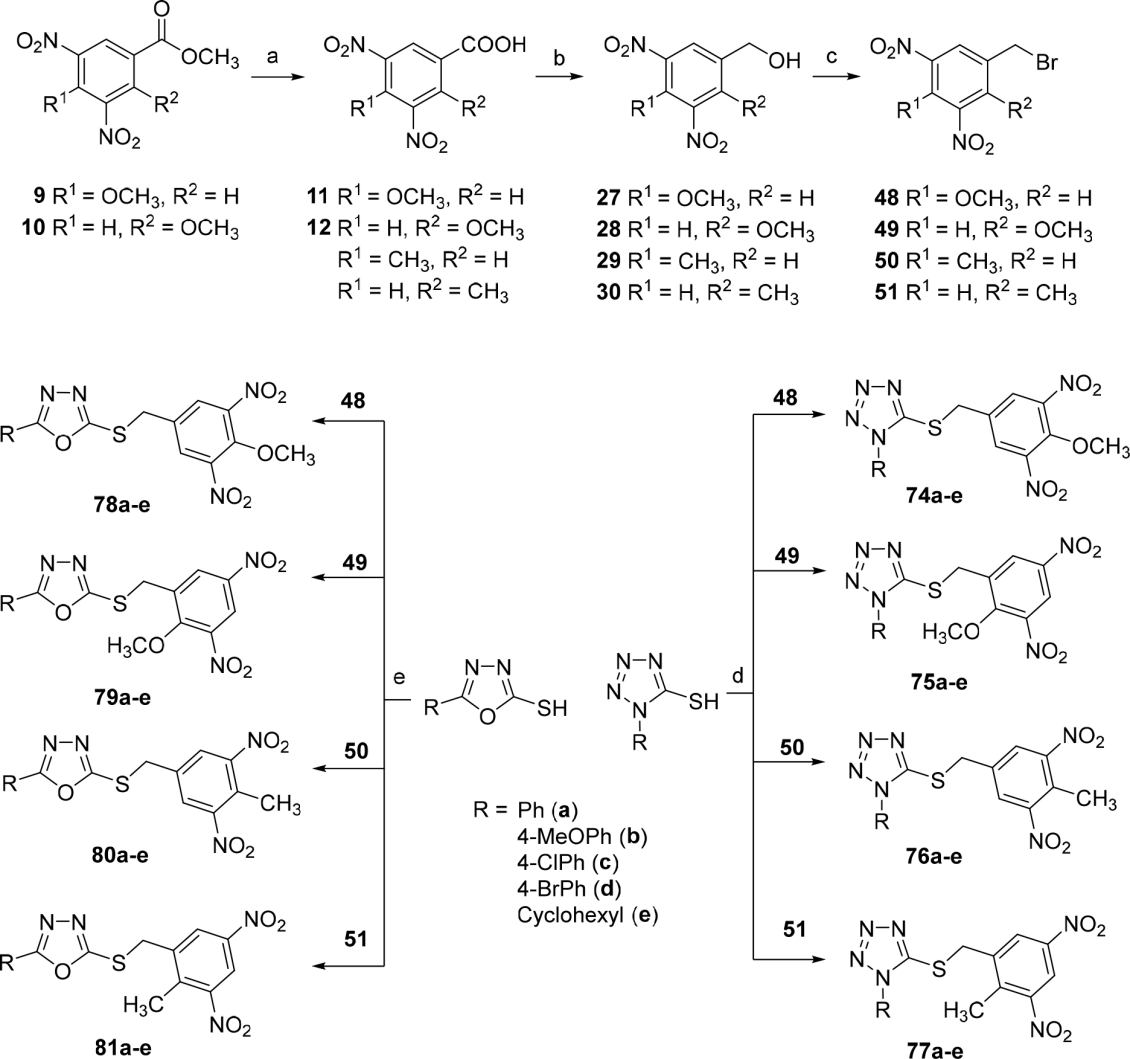
Synthesis of 4-Methoxy- and 2-Methoxy-3,5-dinitrobenzylbromides and 4-Methyl- and 2-Methyl-3,5-dinitrobenzylbromides **48**, **49**, **50**, and **51**, respectively, and Their Use in the Synthesis of Final Tetrazoles of Series **74**–**77** and Oxadiazoles of Series **78**–**81** Reagents and conditions: (a) CH_3_ONa, CH_3_OH, reflux, 2 h, 40–60%; (b) BH_3_·THF, THF, −20 °C → rt, overnight, 71–83%; (c) NBS, Ph_3_P, CH_2_Cl_2_, 0 °C → rt, 1–12 h, 70–85%; (d) Et_3_N, CH_3_CN, 0.5–1 h, rt, 66–95%; (e) Et_3_N, CH_3_CN, 0.5–1 h, rt, 68–91%.

The synthesis of final compounds with an additional methyl or methoxy group on the key 3,5-dinitrobenzyl part of the molecule (**74**–**81**) started from commercially available 2-methyl-, 4-methyl-, 2-hydroxy, or 4-hydroxy-3,5-dinitrobenzoic acids (Scheme 8). 4-Hydroxy-3,5-dinitrobenzoic acid and 3,5-dinitrosalicylic acid were methylated using dimethyl sulfate in the presence of potassium carbonate to obtain methyl esters of methoxy acids **9** and **10**, respectively. These esters were converted to acids **11** and **12** using sodium methoxide.^27^ 4-Methoxy- or 2-methoxy-3,5-dinitrobenzoic acids **11** and **12**, as well as 4-methyl- or 2-methyl-3,5-dinitrobenzoic acids were reduced to the corresponding benzyl alcohols **27**–**30** and then converted to benzyl bromides **48**–**51**.^26^ The alkylation of the corresponding 1-substituted-1*H*-tetrazole-5-thiols or 5-substitued-1,3,4-oxadiazol-2-thiols provided the target tetrazole-based compounds of series **74**–**77** and oxadiazole-based compounds of series **78**–**81** in 66–95% yield (Scheme 8).

### *In Vitro* Antimycobacterial Activity

*In vitro* antimycobacterial activity of all final compounds of series **52**–**83** were evaluated against *M.tb.* CNCTC My 331/88 (H37Rv) and against nontuberculous mycobacterial strains of *M. avium* CNCTC My 330/88 and *M. kansasii* CNCTC My 235/80 and compared with *in vitro* antimycobacterial activity of lead compounds of series **1** and **2**. The antimycobacterial activities of all compounds were evaluated after 7, 14, or 21 days of incubation and are expressed as minimum inhibitory concentration (MIC) in micromolar.

The first aim of this work was to explore the possibility of the replacement of one nitro group for another electron-withdrawing and/or (bio)isosteric group in 3,5-dinitrobenzylsulfanyl tetrazole **1** and/or oxadiazole **2** antitubercular agents (Part A). Therefore, derivatives with trifluoromethyl- (**52a**–**e**, **57a**–**e**), chloro- (**53a**–**e**, **58a**–**e**), fluoro- (**54a**–**e**, **59a**–**e**), bromo- (**55a**–**e**, **60a**–**e**), and cyano- (**56a**–**e**, **61a**–**e**) groups instead of *one* nitro group in the 3,5-dinitrobenzyl part were prepared. In the case of oxadiazole lead compounds **2a**–**e**, which displayed outstanding activities, additional analogues with methoxycarbonyl- (**62a**–**e**), carbamoyl- (**63a**–**e**, **64a**–**e**), and pyrrole (**65a**–**e**) groups were prepared. However, a strong decrease of the antimycobacterial activity was observed in all cases, regardless of the introduced functional group or heterocycle involved; indeed the majority of compounds completely lost their antitubercular activity (Tables 2 and 3). Among the prepared analogues, 3-cyano-5-nitrobenzyl derivatives of series **56** and **61** were slightly effective against *M.tb*. 5-((3-Cyano-5-nitrobenzyl)sulfanyl)-1-(4-chlorophenyl)-1*H*-tetrazole (**56c**) and 2-((3-cyano-5-nitrobenzyl)sulfanyl)-5-(4-methoxyphenyl)-1,3,4-oxadiazole (**61b**) showed the best activities with MIC values of 2 μM and 4 μM, respectively. Nonetheless, these MIC values were substantially lower than those of the parent oxadiazoles **2** or INH.

**Table 2:**
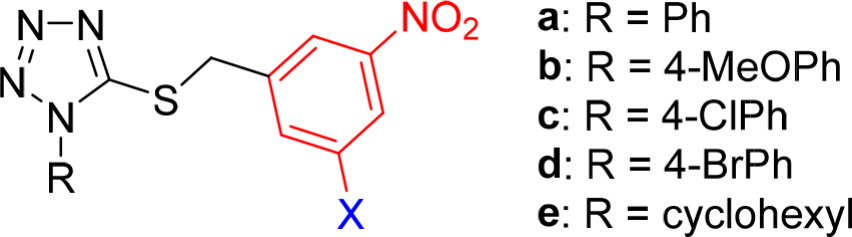
*In Vitro* Antimycobacterial Activities of the Final Tetrazole-Based Compounds of Series **52–56** Expressed as MIC (μM) and Their Comparison with Those of Parent Tetrazoles **1a**–**e**^12^

	X	*M. tuberculosis* My 331/88^a^	*M. avium* My 330/88^a^	*M. kansasii* My 235/80^b^
INH		0.5/1	250/250	250/250/250
pretomanid		0.125/0.25	>32/>32	>32/>32/>32
**1a**	NO_2_	4/4	62/62	2/4/16
**1b**	NO_2_	2/4	16/32	1/4/4
**1c**	NO_2_	1/2	125/125	2/4/4
**1d**	NO_2_	1/1	125/125	1/2/2
**1e**	NO_2_	1/1	16/32	4/4/4
**52a**	CF_3_	32/64	250/250	125/250/250
**52b**	CF_3_	32/64	250/250	125/250/250
**52c**	CF_3_	32/32	250/250	125/250/250
**52d**	CF_3_	64/125	250/250	125/250/250
**52e**	CF_3_	64/64	250/250	64/64/64
**53a**	Cl	64/125	250/250	125/250/250
**53b**	Cl	250/250	250/250	125/250/250
**53c**	Cl	250/250	250/250	250/250/250
**53d**	Cl	250/250	250/250	125/250/250
**53e**	Cl	250/250	250/250	125/250/250
**54a**	F	250/250	250/250	125/250/250
**54b**	F	250/250	250/250	125/250/250
**54c**	F	125/250	250/250	125/250/250
**54d**	F	64/64	250/250	64/125/250
**54e**	F	64/125	250/250	125/250/250
**55a**–**55e**	Br	>250	>250	>250
**56a**	CN	250/250	250/250	250/250/250
**56b**	CN	32/32	250/250	16/32/32
**56c**	CN	2/4	250/250	4/8/16
**56d**	CN	32/32	250/250	16/32/32
**56e**	CN	250/250	250/250	250/250/250

a 14/21 days.

b 7/14/21 days.

**Table 3:**
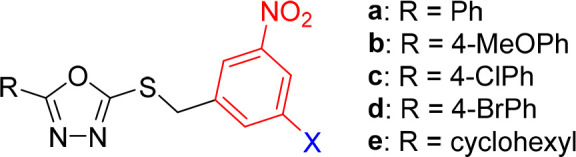
*In Vitro* Antimycobacterial Activities of the Final Oxadiazole-Based Compounds of Series **57**–**65** Expressed as MICs (μM) and Their Comparison with Those of Parent Oxadiazoles **2a**–**e**^13^

	X	*M. tuberculosis* My 331/88^a^	*M. avium* My 330/88^a^	*M. kansasii* My 235/80^b^
INH		0.5/1	250/250	250/250/250
pretomanid		0.125/0.25	>32/>32	>32/>32/>32
**2a**	NO_2_	0.06/0.06	16/32	0.5/1/1
**2b**	NO_2_	0.125/0.125	16/32	0.125/0.25/0.25
**2c**	NO_2_	0.125/0.125	>125/>125	0.125/0.25/0.25
**2d**	NO_2_	0.125/0.125	250/250	0.125/0.25/0.5
**2e**	NO_2_	≤0.03/≤0.03	>32/>32	0.06/0.125/0.25
**57a**	CF_3_	64/64	250/250	32/64/125
**57b**	CF_3_	64/125	250/250	64/64/125
**57c**	CF_3_	250/250	250/250	250/250/250
**57d**	CF_3_	250/250	250/250	250/250/250
**57e**	CF_3_	125/125	250/250	64/125/250
**58a**–**58e**	Cl	>250	>250	>125
**59a**–**59c**	F	250/250	250/250	250/250/250
**59d**	F	250/250	250/250	125/250/250
**59e**	F	125/125	250/250	64/125/125
**60a**	Br	125/250	250/250	64/125/250
**60b**	Br	125/250	250/250	64/125/250
**60c**	Br	125/125	125/125	125/125/125
**60d**	Br	250/250	250/250	250/250/250
**60e**	Br	32/32	250/250	64/64/64
**61a**	CN	16/16	250/250	4/8/2016
**61b**	CN	4/4	250/250	2/4/2008
**61c**	CN	8/16	250/250	4/8/2016
**61d**	CN	250/250	250/250	250/250/250
**61e**	CN	32/32	250/250	16/32/32
**62a**–**62e**	COOCH_3_	>250	>250	>125
**63a**–**63e**	CONH_2_	>250	>250	>125
**64a**–**64e**	CONHBn	>250	>250	>250
**65a**–**65e**	pyrrol-1-yl	>250	>250	>250

a 14/21 days.

b 7/14/21 days.

Another possibility to reduce the number of nitro groups in the lead compounds was the replacement of the 3,5-dinitrobenzyl fragment with heterocyclic (5-nitropyridin-3-yl)methyl and (5-nitrofuran-2-yl)methyl moieties, especially the latter, since the 5-nitrofuran-2-yl group has previously been identified as a key moiety responsible for high antimycobacterial effect of several series of potent anti-TB agents.^28,29^ Thus, oxadiazole-type series **82a**–**e** and **83a**–**e** were prepared. Despite good antimycobacterial activity found with some of the prepared analogues, especially in the case of 5-nitrofuran-2-yl analogue **83e**, lead compounds of series **2** were always in excess of 10 times more active (Table 4).

**Table 4:**
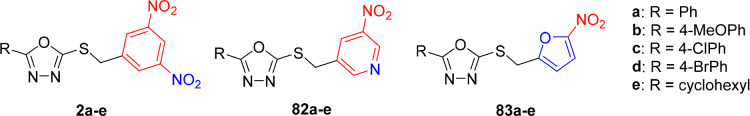
*In Vitro* Antimycobacterial Activities of the Compounds with (5-Nitropyridin-3-yl)methyl (**82a**–**e**) and (5-Nitrofuran-2-yl)methyl (**83a**–**e**) Groups Expressed as MIC (μM) and Their Comparison with Those of Parent Oxadiazoles **2a**–**e**^13^

	*M. tuberculosis* My 331/88^a^	*M. avium* My 330/88^a^	*M. kansasii* My 235/80^b^
INH	0.5/1	250/250	250/250/250
pretomanid	0.125/0.25	>32/>32	>32/>32/>32
**2a**	0.06/0.06	16/32	0.5/1/1
**2b**	0.125/0.125	16/32	0.125/0.25/0.25
**2c**	0.125/0.125	>125/>125	0.125/0.25/0.25
**2d**	0.125/0.125	250/250	0.125/0.25/0.5
**2e**	≤0.03/≤0.03	>32/>32	0.06/0.125/0.25
**82a**	125/250	250/250	125/250/250
**82b**	16/16	250/250	16/16/32
**82c**	4/8	>1000/>1000	8/8/16
**82d**	16/16	>1000/>1000	8/16/16
**82e**	16/16	1000/1000	8/16/16
**83a**	16/32	32/62	32/>32/>32
**83b**	>32/>32	64/64	>32/>32/>32
**83c**	16/32	32/32	>32/>32/>32
**83d**	8/16	16/32	16/16/32
**83e**	0.5/1	32/32	4/8/16

a 14/21 days.

b 7/14/21 days.

Because all the efforts to remove or replace one nitro group in the lead compounds **1** and **2** resulted in substantial decrease of antimycobacterial activity, we decided to explore more deeply the role of the position of both nitro groups in antimycobacterial activity. In our previous work, we proved that 2,4-dinitrobenzyl analogues showed lower antimycobacterial activity compared to their 3,5-dinitro counterparts.^12,13,15^ Therefore, 3,5-dinitro-substituted compounds served as the lead compounds in following studies.^11,30^ Thus, in Part B we focused on the remaining variants with a nitro group in position 3 (or 5), i.e. 2,5-dinitro and 3,4-dinitro analogues. Positive hits could open a new path to further structural modifications and the possibility of nitro group replacement, which was not the case with 3,5-dinitrobenzyl lead compounds. In the case of 3,4-dinitrobenzyl analogues of series **66** and **68**, we found a decrease in antimycobacterial activity when compared to those of the lead compounds of series **1** and **2**. Nonetheless, 2,5-dinitro analogues of series **67** and **69** showed very good activities comparable to that of INH, i.e., comparable to those of lead compounds **1a**–**e** but lower than oxadiazole-based lead compounds **2a**–**e**. Interestingly, activities of 3,4-dinitro and especially 2,5-dinitro analogues were not influenced by the type of the heterocycle. Tetrazole-based and oxadiazole-based compounds **67a**–**e** and **69a**–**e**, respectively, showed very similar activities. As 2,5-dinitrobenzylsulfanyl maintained high antimycobacterial activities, we preliminarily checked the possibility of replacing one nitro group for another electron-withdrawing group: trifluoromethyl. Thus, in Part C, 2-nitro-5-(trifluoromethyl) derivatives **70a**–**e** and **72a**–**e** and 5-nitro-2-(trifluoromethyl) derivatives **71a**–**e** and **73a**–**e** were prepared and evaluated for their antimycobacterial efficacy. Unfortunately, significant decrease of activity or its complete loss was observed for both tetrazole and oxadiazole series, similarly to the case of trifluoromethyl analogues of lead compounds **1** and **2** (Tables 5 and 6).

**Table 5:**
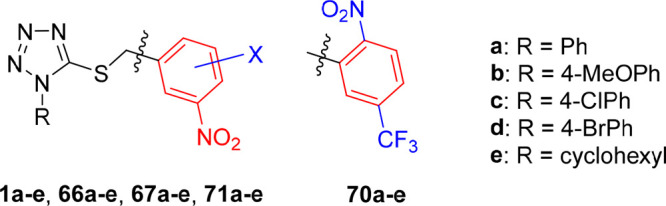
*In Vitro* Antimycobacterial Activities of the Tetrazole-Based Compounds **66a**–**e** and **67a**–**e** with 3,4-Dinitrobenzyl and 2,5-Dinitrobenzyl Groups, Respectively, and Trifluoromethyl Analogues of the Latter with 5-Trifluoromethyl and 2-Trifluoromethyl Groups **70a**–**e** and **71a**–**e**, Respectively, Expressed as MIC (μM) and Their Comparison with Those of Parent Tetrazoles **1a**–**e**^12^

	X	*M. tuberculosis* My 331/88^a^	*M. avium* My 330/88^a^	*M. kansasii* My 235/80^b^
INH		0.5/1	250/250	250/250/250
pretomanid		0.125/0.25	>32/>32	>32/>32/>32
**1a**	3-NO_2_	4/4	62/62	2/4/16
**1b**	3-NO_2_	2/4	16/32	1/4/4
**1c**	3-NO_2_	1/2	125/125	2/4/4
**1d**	3-NO_2_	1/1	125/125	1/2/2
**1e**	3-NO_2_	1/1	16/32	4/4/4
**66a**	4-NO_2_	16/16	32/32	16/32/32
**66b**	4-NO_2_	32/32	125/125	16/32/32
**66c**	4-NO_2_	4/8	250/250	2/4/8
**66d**	4-NO_2_	16/16	250/250	8/16/16
**66e**	4-NO_2_	32/64	>1000/>1000	32/64/125
**67a**	2-NO_2_	1/1	250/250	2/4/4
**67b**	2-NO_2_	0.5/1	250/250	1/1/1
**67c**	2-NO_2_	0.5/1	250/250	1/1/2
**67d**	2-NO_2_	1/1	250/250	1/1/2
**67e**	2-NO_2_	1/1	250/250	1/1/1
**70a**	-	250/250	250/250	125/250/250
**70b**–**70e**	-	250/250	250/250	250/250/250
**71a**	2-CF_3_	32/32	250/250	64/132/250
**71b**	2-CF_3_	8/8	250/250	32/32/32
**71c**	2-CF_3_	250/250	250/250	250/250/250
**71d**	2-CF_3_	250/250	250/250	250/250/250
**71e**	2-CF_3_	32/32	250/250	64/64/64

a 14/21 days.

b 7/14/21 days.

**Table 6:**
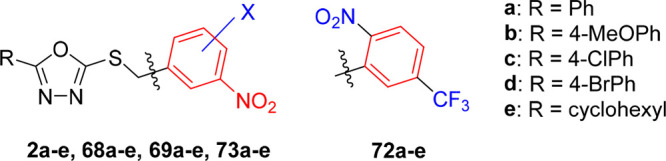
*In Vitro* Antimycobacterial Activities of the Oxadiazole-Based Compounds **68a**–**e** and **69a**–**e** with 3,4-Dinitrobenzyl and 2,5-Dinitrobenzyl Group, Respectively, and Trifluoromethyl Analogues of the Latter with 5-Trifluoromethyl and 2-Trifluoromethyl Groups **72a**–**e** and **73a**–**e**, Respectively, Expressed as MIC (μM) and Their Comparison with Those of Parent Oxadiazoles **2a-e**(13)

	X	*M. tuberculosis* My 331/88^a^	*M. avium* My 330/88^a^	*M. kansasii* My 235/80^b^
INH		0.5/1	250/250	250/250/250
pretomanid		0.125/0.25	>32/>32	>32/>32/>32
**2a**	3-NO_2_	0.06/0.06	16/32	0.5/1/1
**2b**	3-NO_2_	0.125/0.125	16/32	0.125/0.25/0.25
**2c**	3-NO_2_	0.125/0.125	>125/>125	0.125/0.25/0.25
**2d**	3-NO_2_	0.125/0.125	250/250	0.125/0.25/0.5
**2e**	3-NO_2_	≤0.03/≤0.03	>32/>32	0.06/0.125/0.25
**68a**	4-NO_2_	8/16	250/250	16/32/32
**68b**	4-NO_2_	16/16	125/125	16/32/32
**68c**	4-NO_2_	8/8	64/64	8/16/16
**68d**	4-NO_2_	4/8	250/250	2/4/8
**68e**	4-NO_2_	125/250	250/250	250/250/250
**69a**	2-NO_2_	1/1	250/250	1/2/4
**69b**	2-NO_2_	1/1	250/250	1/1/2
**69c**	2-NO_2_	1/1	>1000/>1000	2/2/4
**69d**	2-NO_2_	1/1	250/250	2/4/4
**69e**	2-NO_2_	1/1	>1000/>1000	1/1/1
**72a**	-	250/250	250/250	125/250/250
**72b**–**72e**	-	250/250	250/250	250/250/250
**73a**–**73e**	2-CF_3_	250/250	250/250	250/250/250

a 14/21 days.

b 7/14/21 days.

Another modification of the lead compounds in Part D, i.e., the introduction of a methyl or methoxy group at position 2 or 4 of the 3,5-dinitrobenzyl fragment, caused a slight to significant decrease of antimycobacterial activities (Tables 7 and 8). Antimycobacterial activities of 4-methoxy, 2-methoxy, or 2-methyl-substituted 3,5-dinitrobenzylsulfanyl tetrazoles **74a**–**e**, **75a**–**e**, and **77a**–**e**, respectively, were considerably lower than those of parent compounds **1a**–**e**. However, 4-methyl-3,5-dinitrobenzylsulfanyl analogous **76a**–**e** maintained high efficacy with MIC values of 2–4 μM only slightly lower than those of tetrazoles **1a**–**e**. Moreover, these compounds showed good activity against *M. kansasii* My 235/80 (Table 7).

**Table 7:**
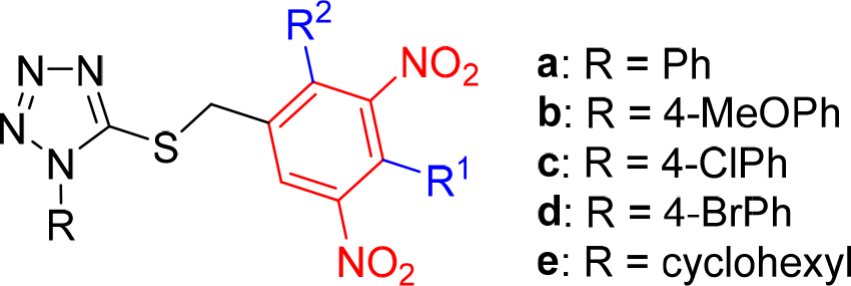
*In Vitro* Antimycobacterial Activities of the 3,5-Dinitrobenzysulfanyl Tetrazoles with Additional Methoxy Group (**74a**–**e** and **75a**–**e**) or Methyl Group (**76a**–**e** and **77a**–**e**) on 3,5-Dinitrophenyl Moiety Expressed as MICs (μM) and Their Comparison with Those of Parent Tetrazoles **1a**–**e**^12^

	R^1^	R^2^	*M. tuberculosis* My 331/88^a^	*M. avium* My 330/88^a^	*M. kansasii* My 235/80^b^
INH			0.5/1	250/250	250/250/250
pretomanid			0.125/0.25	>32/>32	>32/>32/>32
**1a**	H	H	4/4	62/62	2/4/16
**1b**	H	H	2/4	16/32	1/4/4
**1c**	H	H	1/2	125/125	2/4/4
**1d**	H	H	1/1	125/125	1/2/2
**1e**	H	H	1/1	16/32	4/4/4
**74a**	OCH_3_	H	250/250	250/250	250/250/250
**74b**	OCH_3_	H	16/32	250/250	250/250/250
**74c**	OCH_3_	H	16/32	250/250	250/250/250
**74d**	OCH_3_	H	>32/>32	250/250	>32/>32/>32
**74e**	OCH_3_	H	32/32	>1000/>1000	16/32/32
**75a**	H	OCH_3_	>32/>32	250/250	>32/>32/>32
**75b**	H	OCH_3_	16/32	250/250	8/16/32
**75c**	H	OCH_3_	8/16	250/250	8/16/32
**75d**	H	OCH_3_	8/16	250/250	8/16/32
**75e**	H	OCH_3_	32/64	250/250	32/64/125
**76a**	CH_3_	H	4/8	250/250	2/4/8
**76b**	CH_3_	H	4/4	500/500	8/8/16
**76c**	CH_3_	H	2/2	250/250	1/2/4
**76d**	CH_3_	H	2/2	500/500	2/4/4
**76e**	CH_3_	H	4/8	250/250	8/16/32
**77a**	H	CH_3_	>32/>32	250/250	32/>32/>32
**77b**	H	CH_3_	16/16	250/250	8/16/32
**77c**	H	CH_3_	16/32	250/250	8/16/32
**77d**	H	CH_3_	32/32	64/64	32/32/32
**77e**	H	CH_3_	250/250	250/250	250/250/250

a 14/21 days.

b 7/14/21 days.

**Table 8:**
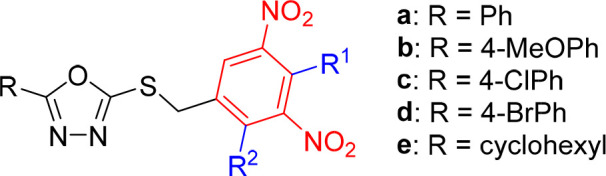
*In Vitro* Antimycobacterial Activities of the 3,5-Dinitrobenzysulfanyl Oxadiazoles with Additional Methoxy Group (**78a**–**e** and **79a**–**e**) or Methyl Group (**80a**–**e** and **81a**–**e**) on 3,5-Dinitrophenyl Moiety Expressed as MICs (μM) and Their Comparison with Those of Parent Oxadiazoles **2a**–**e**^13^

	R^1^	R^2^	*M. tuberculosis* My 331/88^a^	*M. avium* My 330/88^a^	*M. kansasii* My 235/80^b^
INH			0.5/1	250/250	250/250/250
pretomanid			0.125/0.25	>32/>32	>32/>32/>32
**2a**	H	H	0.06/0.06	16/32	0.5/1/1
**2b**	H	H	0.125/0.125	16/32	0.125/0.25/0.25
**2c**	H	H	0.125/0.125	>125/>125	0.125/0.25/0.25
**2d**	H	H	0.125/0.125	250/250	0.125/0.25/0.5
**2e**	H	H	≤0.03/≤0.03	>32/>32	0.06/0.125/0.25
**78a**	OCH_3_	H	16/16	250/250	4/8/16
**78b**	OCH_3_	H	16/16	250/250	4/8/16
**78c**	OCH_3_	H	8/16	250/250	8/16/16
**78d**	OCH_3_	H	250/250	250/250	250/250/250
**78e**	OCH_3_	H	250/250	250/250	250/250/250
**79a**	H	OCH_3_	1/2	250/250	2/8/16
**79b**	H	OCH_3_	2/2	500/500	1/2/4
**79c**	H	OCH_3_	2/2	500/500	1/2/2
**79d**	H	OCH_3_	0.5/1	250/250	2/2/4
**79e**	H	OCH_3_	1/1	250/250	1/1/1
**80a**	CH_3_	H	>32/>32	250/250	32/>32/>32
**80b**	CH_3_	H	250/250	250/250	250/250/250
**80c**	CH_3_	H	32/>32	250/250	16/32/>32
**80d**	CH_3_	H	>32/>32	250/250	>32/>32/>32
**80e**	CH_3_	H	125/250	250/250	250/250/250
**81a**	H	CH_3_	2/2	250/250	1/2/4
**81b**	H	CH_3_	2/4	250/250	2/4/8
**81c**	H	CH_3_	2/2	250/250	2/4/8
**81d**	H	CH_3_	2/2	250/250	2/4/8
**81e**	H	CH_3_	2/2	250/250	8/16/32

a 14/21 days.

b 7/14/21 days.

For methyl- and methoxy-substituted 3,5-dinitrobenzylsulfanyl oxadiazoles **78**–**81**, it was found that the substitution in position 2 is more beneficial, while 2-methoxy and 2-methyl oxadiazoles **79a**–**e** and **81a**–**e**, respectively, were more active compared to their 4-substituted counterparts **78a**–**e** and **80a**–**e** (Table 8). This is the opposite phenomenon than what was found in the tetrazole series, where 4-substituted derivatives **76a**–**e** showed the highest antimycobacterial activities within tetrazole series **74**–**77**. Antimycobacterial activities of oxadiazoles **79a**–**e** and **81a**–**e** were comparable to those of tetrazoles **1a**–**e** and INH but still significantly lower compared to the most efficient 3,5-dinitrobenzylsulfanyl oxadiazoles **2a**–**e** (Table 8).

To further inspect the antimycobacterial activities of the most active derivatives prepared in this study, 14 compounds, tetrazoles **56c**, **67a**, **67b**, **67c**, and **67e** and oxadiazoles **61b**, **69a**, **69b**, **69c**, **69e**, **79a**, **79e**, **81a**, and **81e**, were selected, and their activity against seven clinically isolated MDR/XDR *M.tb*. strains was evaluated (Table 9). The activities of studied compounds against these resistant strains were comparable with those against the standard *M.tb*. strain indicating that these derivatives acted through a Ddn-activation pathway similar to the parent oxadiazoles **2**. Consistently, the highest activities were found in the series of 2,5-dinitrobenzylsulfanyl derivatives **67** and **69**, regardless of the substituent R on the tetrazole or oxadiazole, respectively.

**Table 9:** Antimycobacterial Activities of Compounds **56c**, **61b**, **67a**-**67c**, **67e**, **69a**–**69c**, **69e**, **79a**, **79e**, **81a**, and **81e** and Standard Anti-TB Drugs against Seven Clinically Isolated MDR/XDR-TB Strains Expressed as MIC (μM)^a^

	MDR/XDR *M.tb*. strain						
	Praha 1	Praha 4	Praha 131	9449/2007	234/2005	7357/1998	8666/2010
**56c**	4/8	4/8	4/8	4/8	4/8	4/8	4/8
**61b**	8/8	8/8	8/8	8/8	8/8	8/8	8/8
**67a**	1/2	0.5/1	0.5/1	1/1	1/1	1/1	1/1
**67b**	0.5/1	0.25/0.5	0.25/0.5	0.25/0.5	0.5/1	0.5/1	0.5/1
**67c**	0.5/1	0.25/0.5	0.25/0.5	0.5/1	0.5/1	0.5/1	0.5/0.5
**67e**	1/2	1/1	1/1	1/1	0.5/1	0.5/1	0.5/0.5
**69a**	1/2	0.5/1	0.5/1	0.5/1	0.5/1	0.5/1	0.5/0.5
**69b**	1/1	0.5/1	0.5/1	1/1	0.5/1	0.5/1	0.5/0.5
**69c**	1/1	0.5/1	0.5/1	1/2	0.5/1	0.5/1	0.5/0.5
**69e**	1/1	0.5/1	0.5/1	0.5/1	0.5/1	0.5/1	0.25/0.5
**79a**	2/4	1/2	1/2	2/2	1/2	1/2	1/2
**79e**	1/2	0.5/1	0.5/1	1/1	1/1	1/1	1/1
**81a**	2/4	2/4	2/4	2/4	2/4	2/4	2/4
**81e**	2/4	1/2	2/2	4/4	2/2	1/2	1/2
streptomycin	16 (R)	>32 (R)	>32 (R)	>32 (R)	32 (R)	>32 (R)	>32 (R)
isoniazid	16 (R)	16 (R)	16 (R)	64 (R)	16 (R)	16 (R)	32 (R)
ethambutol	32 (R)	16 (R)	32 (R)	8 (S)	16 (R)	16 (R)	16 (R)
rifampin	>8 (R)	>8 (R)	>8 (R)	>8 (R)	>8 (R)	>8 (R)	>8 (R)
ofloxacin	1 (S)	>16 (R)	16 (R)	2 (S)	0.5 (S)	8 (R)	8 (R)
gentamicin	1 (S)	0.5 (S)	>8 (R)	1 (S)	0.25 (S)	1 (S)	2 (S)
clofazimine	0.5 (R)	0.5 (R)	0.25 (S)	0.125 (S)	0.125 (S)	0.125 (S)	2 (R)
amikacin	0.5 (S)	1 (S)	>32 (R)	0.5 (S)	0.5 (S)	1 (S)	2 (S)
pretomanid	n.d.	n.d.	0.25	0.125	n.d.	n.d.	0.25

a S, Strain susceptible to the given antibiotic drug. R, Strain resistant to the given antibiotic drug. n.d., not determined.

### Mode of Action of 2,5-Dinitrobenzylsulfanyl Tetrazoles **67a**–**e** and Oxadiazoles **69a**–**e**

**Figure 4:**
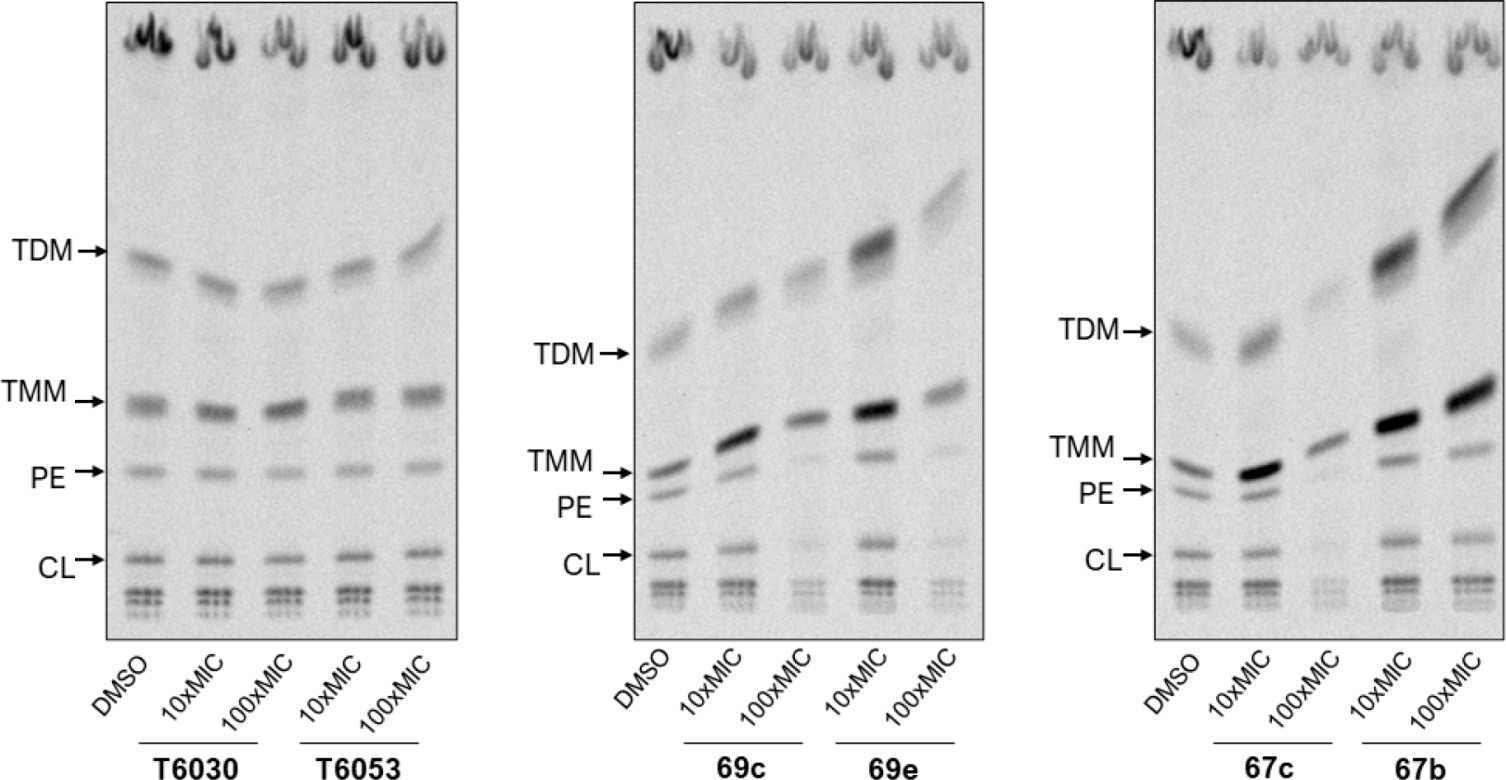
Evaluation of DprE1 inhibition by T6030, T6053, **67b**, **67c**, **69c**, and **69e** using metabolic radiolabeling via TLC analysis of the lipids from radiolabeled *M.tb*. H37Rv. Mycobacteria were co-incubated with [^14^C]acetate and tested compounds at 10 times or 100 times the MIC for 24 h. TMM, trehalose monomycolates; TDM, trehalose dimycolates; PE, phosphatidylethanolamine; CL, cardiolipin.

Due to the very small difference in the structure of 2,5-dinitro- and 3,5-dinitrobenzylsulfanyl derivatives, we first checked whether their mechanism of action is consistent. However, in contrast to the parent 3,5-dinitrobenzylsulfanyl derivatives T6030 and T6053, selected 2,5-dinitrobenzylsulfanyl tetrazoles **67b** and **67c** and oxadiazoles **69c** and **69e** showed the same inhibitory activity against wild-type *M.tb.* H37Rv as against Ddn- and FbiC-deficient mutants indicating that 2,5-dinitro compounds of series **67** and **69** acted via a Ddn-independent pathway. Thus, we turned our attention to DprE1, another important target of nitro-group-containing anti-TB agents including 3,5-dinitrophenyl-containing entities.^11,14^ First, we inspected the effects of 2,5-dinitrobenzylsulfanyl tetrazoles **67b** and **67c** and oxadiazoles **69c** and **69e** on the biosynthesis of lipids of *M.tb*. H37Rv via the [^14^C]acetate radiolabeling experiments in the presence of 10 times or 100 times the MIC of selected compound. The effects of parent T6030 and T6053 were also reassessed (**11i** and **14g** in ref (13), respectively) as the reference. As shown in Figure 4, tetrazole **67b** and oxadiazole **69e** caused accumulation of trehalose monomycolates (TMMs) and trehalose dimycolates (TDMs) in mycobacteria, which is a typical phenomenon for DprE1 inhibitors including BTZ-043.^11^ Treatment of mycobacteria with derivatives **67c** and **69c** led to the accumulation of TMM only. As expected, treatment with 3,5-dinitrobenzylsulfanyl derivatives T6030 and T6053 did not affect the [^14^C]-labeled lipid profiles in mycobacteria (Figure 4). To confirm that the antimycobacterial activity of 2,5-dinitrobenzylsulfanyl heterocycles of series **67** and **69** is related to DprE1 inhibition, we determined their MIC values in *M.tb*. H37Ra overproducing DprE1/2, with BTZ-043, one of the most efficient DprE1 inhibitors, used as a control. As shown in Table 10, the activity of 2,5-dinitrobenzylsulfanyl tetrazole **67b** and oxadiazole **69e** against mycobacteria overproducing DprE1/2 dropped more than 10 times, while the activity of tetrazole **67c** and oxadiazole **69c** was not significantly affected. As expected, the activity of BTZ-043 dropped significantly, while the original 3,5-dinitro compounds T6030 and T6053 showed similar activity regardless of the level of DprE1/2 production.

**Table 10:** Antimycobacterial Activities of Compounds T6030, T6053, **67b**, **67c**, **69c**, and **69e** against *M.tb*. H37Ra Overproducing DprE1/2 Expressed as MIC

	BTZ-043 (ng/mL)	T6030 (μM)	T6053 (μM)	**67b** (μM)	**67c** (μM)	**69c** (μM)	**69e** (μM)
pVV2	1	0.3	0.18	0.125	0.5–1.5	3	0.25–0.5
pVV2-dprE1/2	>30	0.09–0.3	0.18	1.5	1.5	1–3	3

### *In Vitro* Effects of Studied Compounds on Mammalian Cell Viability

The effects of selected final compounds on mammalian cell viability were tested using HepG2 (human hepatocellular carcinoma) cells. In the cases when the IC_50_ exceeded 30 μM, the data are presented as the relative viability at a concentration of 30 μM compared to control vehicle-treated samples (100% viability). All 2,5-dinitrobenzylsulfanyl tetrazoles (**67b**, **67c**, **67e**) and oxadiazoles (**69a**–**c**, **69e**) that showed the highest antimycobacterial activities within compounds in this SAR study showed the highest toxicity/antiproliferative activity to HepG2 cells (Table 11), which was not the case for parent 3,5-dinitrobenzylsulfanyl tetrazoles **1**(12) and mainly oxadiazoles **2**, which did not affect HepG2 cell viability at 50 μM concentrations after 48 h of incubation.^13^

**Table 11:** Viability of HepG2 Cell Line Determined by Viability Cell Assays^a^ after 48 h of Treatment with Compounds **56c**, **61b**, **67b**, **67c**, **67e**, **69a**, **69b**, **69c**, **69e**, **79a**, **79e**, **81a**, and **81e** and Their Selectivity Indices^b^

	IC_50_ (μM)	viability at 30 μM (%)	SI^c^	(MIC for *M.tb.*)
**56c**	>30	92 ± 4	>15	(2)
**61b**	>30	88 ± 6	>7.5	(4)
**67b**	10.18 ± 1.01	33 ± 4	20.4	(0.5)
**67c**	23.28 ± 1.37	51 ± 16	46.6	(0.5)
**67e**	12.47 ± 1.09	35 ± 5	12.5	(1)
**69a**	8.3 ± 0.92	32 ± 9	8.3	(1)
**69b**	5.94 ± 0.77	17 ± 1	5.9	(1)
**69c**	13.41 ± 1.13	29 ± 15	13.4	(1)
**69e**	10.16 ± 1.01	30 ± 12	10.2	(1)
**79a**	>30	93 ± 4	>30	(1)
**79e**	>30	54 ± 2	>30	(1)
**81a**	>30	98 ± 5	>15	(2)
**81e**	>30	55 ± 5	>15	(2)

a CellTiter96 assay.

b Vehicle-treated control viability was set to 100%. SDS-treated cell viability was set to 0%.

c Selectivity index (SI) was calculated using the formula: SI = (IC_50_ for HepG2)/(MIC for *M.tb*).

## Conclusions

The presence of nitro groups has often discouraged further development of hit compounds as drugs, because nitro groups can increase the risk of toxicity (mainly genotoxicity/mutagenicity), decrease the solubility of these compounds, and lead to their rapid metabolization.^16^ However, 3,5-dinitrobenzylsulfanyl-substituted heterocycles have been identified by us and others as readily accessible compounds with excellent antimycobacterial activities and acceptable toxicity profiles.^12,13,15,20^ Here, we first examined the role of the nitro groups in the mode of antimycobacterial action of these compounds. Whole genome sequencing of spontaneously resistant colonies showed that they harbored mutations in the *fgd1* (Rv0407) gene encoding FGD1. Mutations in FGD1 disrupt the reduction of cofactor F_420_ to F_420_-H_2_, which inhibits the function of Ddn and blocks the reductive activation of nitro-group-containing drugs like pretomanid or delamanid.^17^ Decreased activity of 3,5-dinitrobenzylsulfanyl derivatives T6030 and T6053 toward Ddn- and FbiC-deficient *M.tb.* mutants proved that 3,5-dinitrobenzylsulfanyl heterocycles have a nitro-group-dependent mode of action that relies on Ddn-reductive activation. In the second part of this work, we have thoroughly investigated the structure–activity relationships of 3,5-dinitrobenzylsulfanyl tetrazoles and 1,3,4-oxadiazoles to see if we can replace/relocate one of the two nitro groups. Thus, various electron-withdrawing groups were attached instead of one nitro group. Moreover, the isosteric pyrrol-1-yl group, which has been successfully used to replace nitro group in various types of anti-TB agents,^31^ was utilized. Finally, the entire 3,5-dinitrophenyl group was replaced by nitro-substituted heterocyclic groups. However, the majority of the prepared compounds had significantly decreased activity as compared to their parent tetrazole and especially oxadiazole compounds. Thus, in the next step, we investigated the role of the relative position of the two nitro groups to possibly open the way for further structural optimization. We found that 2,5-dinitrobenzylsulfanyl tetrazoles **67a**–**e** and oxadiazoles **69a**–**e** showed consistently high antimycobacterial activity with MIC values around 1 μM against drug-susceptible and also MDR/XDR clinically isolated strains, i.e., activities comparable to those of parent tetrazoles **1a**–**e** but lower compared to oxadiazoles **2a**–**e**. Interestingly, shifting the nitro group from position 3 to position 2 led to a change in the dominant mechanism of antimycobacterial action. 2,5-Dinitrobenzylsulfanyl tetrazoles of series **67** and oxadiazoles of series **69** acted as DprE1 inhibitors as demonstrated by the accumulation of TMMs and TDMs in treated mycobacteria and by decreased activity of these compounds in mycobacteria overproducing DprE1/2. However, all 2,5-dinitro analogues showed significant toxicity to HepG2 cells, which was not the case for the parent 3,5-dinitro compounds. The replacement of one nitro group for a trifluoromethyl group in 2,5-dinitrobenzyl derivatives also led to a significant decrease or complete loss of antimycobacterial activity. The last attempt to modify the structure of compounds **1** and **2** was the introduction of an additional methyl or methoxy substituent adjacent to the 3,5-dinitrophenyl group, which can sterically hinder one or both nitro groups. However, these modifications also led to a significant decrease in antimycobacterial activity.

In conclusion, both nitro groups in 3,5-dinitrobenzylsulfanyl-containing antimycobacterial agents remain essential for their high efficacy. Further efforts should therefore be directed at fine-tuning the activity/toxicity ratios and finding ways to address the solubility issues, for example, by targeted delivery, rather than avoiding nitro groups.

## Experimental Section

### General

The prepared compounds were characterized using ^1^H NMR and ^13^C NMR spectroscopy. The purity of all prepared compounds was >95% as determined using elemental analysis (fluorine-free compounds) or HPLC–HRMS experiments (fluorine-containing compounds and oily compounds). All chemicals used in the syntheses were obtained from Sigma-Aldrich (Schnelldorf, Germany) and PENTA s.r.o. (Prague, Czech Republic) and were used as received. TLC separations were performed on Merck aluminum plates with silica gel 60 F_254_. Merck Kieselgel 60 (0.040–0.063 mm) was used for column chromatography. Melting points were recorded with a Büchi B-545 apparatus (BUCHI Labortechnik AG, Flawil, Switzerland) and are uncorrected. ^1^H and ^13^C NMR spectra were recorded using Varian Mercury Vx BB 300, VNMR S500 NMR (Varian, Palo Alto, CA, USA) or Jeol JNM-ECZ600R (JEOL Ltd., Akishima, Tokyo, Japan) spectrometers. Chemical shifts are reported as δ values in parts per million (ppm) and were indirectly referenced to tetramethylsilane (TMS) via the solvent signal. Elemental analyses were performed on an Automatic Microanalyzer EA1110CE (Fisons Instruments S.p.A., Milano, Italy). HPLC–HRMS (ESI) experiments were performed using an HRMS system Acquity UPLC I-class and a Synapt G2Si Q-TOF mass spectrometer (Waters, Milford, MA, USA).

### General Method for the Synthesis of Final Compounds **52**–**81**, **83**

The corresponding alkyl halide **35**–**51** (1 mmol) was added to the solution of 1-substituted tetrazole-5-thiol or 5-substituted 1,3,4-oxadiazole-2-thiol (1.1 mmol) and triethylamine (1.2 mmol) in acetonitrile (5–10 mL). The reaction mixture was stirred at rt upon complete consumption of alkyl halide as determined by TLC. Then, the solvent was evaporated under reduced pressure, and the residue was dissolved in EtOAc (50 mL) and washed with 5% aqueous Na_2_CO_3_ (2 × 30 mL) and water (1 × 30 mL). The organic phase was separated, dried over anhydrous sodium sulfate, and evaporated under reduced pressure. The crude product was purified using column chromatography (mobile phase: hexane/EtOAc).

#### 1-Alkyl/Aryl-5-((3-nitro-5-(trifluoromethyl)benzyl)sulfanyl)-1*H*-tetrazoles **52a**–**52e**

3-Nitro-5-(trifluoromethyl)benzyl bromide (**35**) was used as the alkylating agent. The reactions were completed in 1 h.

##### 5-((3-Nitro-5-(trifluoromethyl)benzyl)sulfanyl)-1-phenyl-1*H*-tetrazole (**52a**)

Yield: 93% as a yellowish solid; mp 112–113 °C. ^1^H NMR (600 MHz, DMSO*-d*_6_) δ 8.63 (t, *J* = 2.1 Hz, 1H), 8.35 (t, *J* = 2.1 Hz, 1H), 8.31 (t, *J* = 1.9 Hz, 1H), 7.61–7.55 (m, 5H), 4.75 (s, 2H). ^13^C NMR (151 MHz, DMSO*-d*_6_) δ 154.08, 148.63, 141.96, 133.43, 132.75, 131.23, 130.74 (q, *J* = 33.4 Hz), 130.53, 128.48, 125.11, 123.43 (d, *J* = 273.1 Hz), 120.18 (d, *J* = 4.3 Hz), 35.57. HRMS (ESI+) calcd for (C_15_H_10_F_3_N_5_O_2_S + H^+^) *m*/*z*: 382.05801(100%), 383.06136 (16%); found: 382.0588 (100%), 383.0610 (18%).

##### 1-(4-Methoxyphenyl)-5-((3-nitro-5-(trifluoromethyl)benzyl)sulfanyl)-1*H*-tetrazole (**52b**)

Yield: 80% as a yellowish solid; mp 104–105 °C. ^1^H NMR (600 MHz, DMSO*-d*_6_) δ 8.61 (t, *J* = 1.9 Hz, 1H), 8.35 (s, 1H), 8.30 (s, 1H), 7.46 (d, *J* = 9.0 Hz, 2H), 7.10 (d, *J* = 9.0 Hz, 2H), 4.73 (s, 2H), 3.80 (s, 3H). ^13^C NMR (151 MHz, DMSO*-d*_6_) δ 161.18, 154.18, 148.61, 142.04, 132.71 (q, *J* = 3.6 Hz), 130.74 (q, *J* = 33.4 Hz), 128.43, 126.88, 126.01, 123.43 (q, *J* = 273.1 Hz), 120.14 (d, *J* = 3.6 Hz), 115.52, 56.22, 35.52. HRMS (ESI+) calcd for (C_16_H_12_F_3_N_5_O_3_S + H^+^) *m*/*z*: 412.06857 (100%), 413.07193 (17%); found: 412.0688 (100%), 413.0711 (18%).

##### 1-(4-Chlorophenyl)-5-((3-nitro-5-(trifluoromethyl)benzyl)sulfanyl)-1*H*-tetrazole (**52c**)

Yield: 96% as a white solid; mp 125–126 °C. ^1^H NMR (600 MHz, DMSO*-d*_6_) δ 8.62 (t, *J* = 1.9 Hz, 1H), 8.35 (s, 1H), 8.30 (s, 1H), 7.67 (d, *J* = 8.9 Hz, 2H), 7.62 (d, *J* = 8.9 Hz, 2H), 4.75 (s, 2H). ^13^C NMR (151 MHz, DMSO*-d*_6_) δ 154.20, 148.61, 141.94, 135.86, 132.76 (q, J = 3.7 Hz), 132.26, 130.75 (q, *J* = 33.6 Hz), 130.54, 128.46, 127.03, 123.42 (q, *J* = 272.7 Hz), 120.16 (d, *J* = 3.9 Hz), 35.70. HRMS (ESI+) calcd for (C_15_H_9_ClF_3_N_5_O_2_S + H^+^) *m*/*z*: 416.01903 (100%), 418.01608 (32%); found: 416.0197 (100%), 418.0159 (38%).

##### 1-(4-Bromophenyl)-5-((3-nitro-5-(trifluoromethyl)benzyl)sulfanyl)-1*H*-tetrazole (**52d**)

Yield: 62% as a white solid; mp 121–123 °C.^1^H NMR (600 MHz, DMSO*-d*_6_) δ 8.62 (t, *J* = 1.9 Hz, 1H), 8.35 (s, 1H), 8.30 (s, 1H), 7.80 (d, *J* = 8.8 Hz, 2H), 7.55 (d, *J* = 8.7 Hz, 2H), 4.74 (s, 2H). ^13^C NMR (151 MHz, DMSO*-d*_6_) δ 154.15, 148.61, 141.93, 133.50, 132.76 (d, *J* = 3.5 Hz), 132.68, 130.74 (q, *J* = 33.2 Hz), 128.46, 127.18, 124.42, 123.42 (q, *J* = 273.1 Hz), 120.16 (d, *J* = 4.0 Hz), 35.71. HRMS (ESI+) calcd for (C_15_H_9_BrF_3_N_5_O_2_S + H^+^) *m*/*z*: 459.96852 (100%), 461.96647 (97%); found: 461.9672 (100%), 459.9691 (97%).

##### 1-Cyclohexyl-5-((3-nitro-5-(trifluoromethyl)benzyl)sulfanyl)-1*H*-tetrazole (**52e**)

Yield: 91% as a white solid; mp 57–59 °C. ^1^H NMR (600 MHz, DMSO*-d*_6_) δ 8.61 (t, *J* = 1.9 Hz, 1H), 8.35 (s, 1H), 8.28 (s, 1H), 4.73 (s, 2H), 4.21 (tt, *J* = 11.5, 3.9 Hz, 1H), 1.86–1.80 (m, 2H), 1.77–1.72 (m, 2H), 1.70–1.64 (m, 2H), 1.61–1.54 (m, 1H), 1.38–1.30 (m, 2H), 1.22–1.14 (m, 1H). ^13^C NMR (151 MHz, DMSO*-d*_6_) δ 152.09, 148.63, 142.21, 132.64 (d, *J* = 3.6 Hz), 130.80 (q, *J* = 33.5 Hz), 128.35, 123.41 (q, *J* = 272.4 Hz), 120.14 (d, *J* = 4.1 Hz), 58.00, 35.54, 32.13, 24.94, 24.88. HRMS (ESI+) calcd for (C_15_H_16_F_3_N_5_O_2_S + H^+^) *m*/*z*: 388.10496 (100%), 389.10831 (16%); found: 388.1056 (100%), 389.1079 (18%).

#### 1-Alkyl/Aryl-5-((3-chloro-5-nitrobenzyl)sulfanyl)-1*H*-tetrazoles **53a**–**53e**

3-Chloro-5-nitrobenzyl chloride (**36**) was used as the alkylating agent. The reactions were stirred overnight.

##### 5-((3-Chloro-5-nitrobenzyl)sulfanyl)-1-phenyl-1*H*-tetrazole (**53a**)

Yield: 85% as a yellow solid; mp 112–114 °C. ^1^H NMR (600 MHz, DMSO*-d*_6_) δ 8.30 (t, *J* = 1.8 Hz, 1H), 8.14 (t, *J* = 2.1 Hz, 1H), 8.00 (t, *J* = 1.8 Hz, 1H), 7.64–7.54 (m, 5H), 4.67 (s, 2H). ^13^C NMR (151 MHz, DMSO*-d*_6_) δ 154.14, 148.96, 141.85, 135.99, 134.34, 133.44, 131.23, 130.53, 125.12, 123.38, 123.13, 35.56. Elem. Anal. Calcd for C_14_H_10_ClN_5_O_2_S: C, 48.35; H, 2.90; N, 20.14; S, 9.22. Found: C, 48.44; H, 2.60; N, 20.10; S, 9.39.

##### 5-((3-Chloro-5-nitrobenzyl)sulfanyl)-1-(4-methoxyphenyl)-1*H*-tetrazole (**53b**)

Yield: 89% as a white solid; mp 124–126 °C. ^1^H NMR (600 MHz, DMSO*-d*_6_) δ 8.28 (t, *J* = 1.8 Hz, 1H), 8.14 (t, *J* = 2.0 Hz, 1H), 7.98 (t, *J* = 1.8 Hz, 1H), 7.48 (d, *J* = 8.8 Hz, 2H), 7.11 (d, *J* = 9.0 Hz, 2H), 4.64 (s, 2H), 3.80 (s, 3H). ^13^C NMR (151 MHz, DMSO*-d*_6_) δ 161.18, 154.24, 148.95, 141.93, 135.95, 134.32, 126.92, 126.02, 123.33, 123.10, 115.53, 56.23, 35.50. Elem. Anal. Calcd for C_15_H_12_ClN_5_O_3_S: C, 47.69; H, 3.20; N, 18.54; S, 8.49. Found: C, 47.78; H, 2.88; N, 18.71; S, 8.60.

##### 5-((3-Chloro-5-nitrobenzyl)sulfanyl)-1-(4-chlorophenyl)-1*H*-tetrazole (**53c**)

Yield: 80% as a white solid; mp 142–144 °C. ^1^H NMR (500 MHz, DMSO*-d*_6_) δ 8.32 (dd, *J* = 2.1, 1.6 Hz, 1H), 8.17 (t, *J* = 2.0 Hz, 1H), 8.02 (t, *J* = 1.7 Hz, 1H), 7.71 (d, *J* = 8.9 Hz, 2H), 7.66 (d, *J* = 8.8 Hz, 2H), 4.69 (s, 2H). ^13^C NMR (126 MHz, DMSO*-d*_6_) δ 154.16, 148.86, 141.72, 135.90, 135.78, 134.26, 132.18, 130.47, 126.97, 123.28, 123.04, 35.62. Elem. Anal. Calcd for C_14_H_9_Cl_2_N_5_O_2_S: C, 43.99; H, 2.37; N, 18.32; S, 8.39. Found: C, 44.16; H, 2.01; N, 18.49; S, 8.77.

##### 1-(4-Bromophenyl)-5-((3-chloro-5-nitrobenzyl)sulfanyl)-1*H*-tetrazole (**53d**)

Yield: 86% as a yellow solid; mp 155–157 °C. ^1^H NMR (600 MHz, DMSO*-d*_6_) δ 8.28 (t, *J* = 1.8 Hz, 1H), 8.14 (t, *J* = 2.1 Hz, 1H), 7.98 (t, *J* = 1.7 Hz, 1H), 7.81 (d, *J* = 9.0 Hz, 2H), 7.56 (d, *J* = 9.0 Hz, 2H), 4.66 (s, 2H). ^13^C NMR (151 MHz, DMSO*-d*_6_) δ 154.20, 148.95, 141.81, 135.98, 134.34, 133.50, 132.70, 127.21, 124.43, 123.37, 123.12, 35.71. Elem. Anal. Calcd for C_14_H_9_BrClN_5_O_2_S: C, 39.41; H, 2.13; N, 16.41; S, 7.51. Found: C, 39.56; H, 1.70; N, 16.54; S, 7.61.

##### 5-((3-Chloro-5-nitrobenzyl)sulfanyl)-1-cyclohexyl-1*H*-tetrazole (**53e**)

Yield: 81% as a white solid; mp 110–112 °C. ^1^H NMR (600 MHz, DMSO*-d*_6_) δ 8.28 (t, *J* = 1.8 Hz, 1H), 8.15 (t, *J* = 2.1 Hz, 1H), 7.97 (t, *J* = 1.7 Hz, 1H), 4.64 (s, 2H), 4.23–4.18 (m, 1H), 1.86–1.79 (m, 2H), 1.80–1.73 (m, 2H), 1.68 (qd, *J* = 12.4, 3.6 Hz, 2H), 1.65–1.55 (m, 1H), 1.35 (qt, *J* = 12.9, 3.5 Hz, 2H), 1.18 (qt, *J* = 12.8, 3.6 Hz, 1H). ^13^C NMR (151 MHz, DMSO*-d*_6_) δ 152.14, 148.98, 142.09, 135.92, 134.38, 123.25, 123.10, 58.01, 35.57, 32.16, 24.96, 24.91. Elem. Anal. Calcd for C_14_H_16_ClN_5_O_2_S: C, 47.52; H, 4.56; N, 19.79; S, 9.06. Found: C, 47.32; H, 4.25; N, 19.86; S, 9.36.

#### 1-Alkyl/Aryl-5-((3-fluoro-5-nitrobenzyl)sulfanyl)-1*H*-tetrazoles **54a**–**54e**

3-Fluoro-5-nitrobenzyl chloride (**37**) was used as the alkylating agent. The reactions were stirred overnight.

##### 5-((3-Fluoro-5-nitrobenzyl)sulfanyl)-1-phenyl-1*H*-tetrazole (**54a**)

Yield: 79% as a white solid; mp 120–122 °C. ^1^H NMR (600 MHz, DMSO-*d*_6_) δ 8.21 (s, 1H), 7.97 (dt, *J* = 8.6, 2.4 Hz, 1H), 7.81 (dt, *J* = 9.1, 1.9 Hz, 1H), 7.64–7.53 (m, 5H), 4.68 (s, 2H). ^13^C NMR (151 MHz, DMSO-*d*_6_) δ 161.89 (d, *J* = 248.4 Hz), 154.13, 149.10 (d, *J* = 9.6 Hz), 142.16 (d, *J* = 8.3 Hz), 133.46, 131.21, 130.52, 125.10, 123.41 (d, *J* = 22.4 Hz), 120.85 (d, *J* = 2.9 Hz), 110.95 (d, *J* = 26.8 Hz), 35.70. HRMS (ESI+) calcd for (C_14_H_10_FN_5_O_2_S + H^+^) *m*/*z*: 332.0618; found: 332.0622.

##### 5-((3-Fluoro-5-nitrobenzyl)sulfanyl)-1-(4-methoxyphenyl)-1*H*-tetrazole (**54b**)

Yield: 81% as a white solid; mp 113–114 °C. ^1^H NMR (600 MHz, DMSO-*d*_6_) δ 8.19 (t, *J* = 1.8 Hz, 1H), 7.97 (dt, *J* = 8.6, 2.2 Hz, 1H), 7.80 (dt, *J* = 9.1, 2.1 Hz, 1H), 7.48 (d, *J* = 9.0 Hz, 2H), 7.11 (d, *J* = 8.9 Hz, 2H), 4.65 (s, 2H), 3.80 (s, 3H). ^13^C NMR (151 MHz, DMSO-*d*_6_) δ 161.85 (d, *J* = 248.5 Hz), 161.19, 154.26, 149.09 (d, *J* = 9.7 Hz), 142.24 (d, *J* = 8.0 Hz), 126.92, 126.03, 123.37 (d, *J* = 22.4 Hz), 120.82 (d, *J* = 3.0 Hz), 115.54, 110.94 (d, *J* = 26.8 Hz), 56.23, 35.63. HRMS (ESI+) calcd for (C_15_H_12_FN_5_O_3_S + H^+^) *m*/*z*: 362.0723; found: 362.0727.

##### 1-(4-Chlorophenyl)-5-((3-fluoro-5-nitrobenzyl)sulfanyl)-1*H*-tetrazole (**54c**)

Yield: 90% as a white solid; mp 108–110 °C. ^1^H NMR (600 MHz, DMSO-*d*_6_) δ 8.20 (t, *J* = 1.9 Hz, 1H), 7.97 (dt, *J* = 8.6, 2.3 Hz, 1H), 7.80 (dt, *J* = 8.8., 1.9 Hz, 1H), 7.68 (d, *J* = 8.8 Hz, 2H), 7.63 (d, *J* = 8.8 Hz, 2H), 4.67 (s, 2H). ^13^C NMR (151 MHz, DMSO-*d*_6_) δ 161.89 (d, *J* = 248.4 Hz), 154.26, 149.09 (d, *J* = 9.5 Hz), 142.12 (d, *J* = 8.1 Hz), 135.86, 132.28, 130.56, 127.06, 123.40 (d, *J* = 22.4 Hz), 120.85 (d, *J* = 3.0 Hz), 110.95 (d, *J* = 26.8 Hz), 35.82. HRMS (ESI+) calcd for (C_14_H_9_ClFN_5_O_2_S + H^+^) *m*/*z*: 366.0228; found: 366.0232.

##### 1-(4-Bromophenyl)-5-((3-fluoro-5-nitrobenzyl)sulfanyl)-1*H*-tetrazole (**54d**)

Yield: 89% as a white solid; mp 123–125 °C. ^1^H NMR (600 MHz, DMSO-*d*_6_) δ 8.20 (t, *J* = 1.7 Hz, 1H), 7.97 (dt, *J* = 8.6, 2.3 Hz, 1H), 7.83–7.78 (m, 3H), 7.56 (d, *J* = 8.7 Hz, 2H), 4.67 (s, 2H). ^13^C NMR (151 MHz, DMSO-*d*_6_) δ 161.89 (d, *J* = 248.4 Hz), 154.21, 149.09 (d, *J* = 9.5 Hz), 142.12 (d, *J* = 8.0 Hz), 133.51, 132.71, 127.20, 124.42, 123.40 (d, *J* = 22.5 Hz), 120.85 (d, *J* = 3.0 Hz), 110.96 (d, *J* = 26.8 Hz), 35.83. HRMS (ESI+) calcd for (C_14_H_9_BrFN_5_O_2_S + H^+^) *m*/*z*: 409.9718 (100%), 411.9697 (97%); found: 409.9721 (97%), 411.9701 (100%).

##### 1-Cyclohexyl-5-((3-fluoro-5-nitrobenzyl)sulfanyl)-1*H*-tetrazole (**54e**)

Yield: 87% as a white solid; mp 83–85 °C. ^1^H NMR (600 MHz, DMSO-*d*_6_) δ 8.19 (t, *J* = 1.8 Hz, 1H), 7.98 (dt, *J* = 8.6, 2.3 Hz, 1H), 7.79 (dt, *J* = 9.1, 1.9 Hz, 1H), 4.65 (s, 2H), 4.25–4.18 (m, 1H), 1.88–1.82 (m, 2H), 1.79–1.73 (m, 2H), 1.73–1.64 (m, 2H), 1.62–1.57 (m, 1H), 1.40–1.30 (m, 2H), 1.23–1.12 (m, 1H). ^13^C NMR (151 MHz, DMSO-*D*_6_) δ 161.91 (d, *J* = 248.5 Hz), 152.15, 149.13 (d, *J* = 9.6 Hz), 142.41 (d, *J* = 8.0 Hz), 123.34 (d, *J* = 22.4 Hz), 120.74 (d, *J* = 3.1 Hz), 110.94 (d, *J* = 26.7 Hz), 58.01, 35.71, 32.15, 24.96, 24.90. HRMS (ESI+) calcd for (C_14_H_16_FN_5_O_2_S + H^+^) *m*/*z*: 338.1087; found: 338.1092.

#### 1-Alkyl/Aryl-5-((3-bromo-5-nitrobenzyl)sulfanyl)-1*H*-tetrazoles **55a**–**55e**

3-Bromo-5-nitrobenzyl chloride (**38**) was used as the alkylating agent. The reactions were stirred overnight.

##### 5-((3-Bromo-5-nitrobenzyl)sulfanyl)-1-phenyl-1*H*-tetrazole (**55a**)

Yield: 86% as a white solid; mp 100–101 °C. ^1^H NMR (500 MHz, DMSO*-d*_6_): δ 8.37 (t, *J* = 1.8 Hz, 1H), 8.28 (t, *J* = 1.9 Hz, 1H), 8.16 (t, *J* = 1.7 Hz, 1H), 7.53–7.60 (m, 5H), 4.69 (s, 2H). ^13^C NMR (126 MHz, DMSO*-d*_6_): δ 153.77, 148.60, 141.63, 138.48, 133.07, 130.86, 130.16, 125.49, 124.76, 123.36, 121.92, 35.12. Elem. Anal. Calcd for C_14_H_10_BrN_5_O_2_S: C, 42.87; H, 2.57; N, 17.86; S, 8.17. Found: C, 43.09; H, 2.20; N, 18.04; S, 8.18.

##### 5-((3-Bromo-5-nitrobenzyl)sulfanyl)-1-(4-methoxyphenyl)-1*H*-tetrazole (**55b**)

Yield: 96% as a white solid; mp 130–131 °C. ^1^H NMR (600 MHz, DMSO*-d*_6_) δ 8.31 (t, *J* = 1.8 Hz, 1H), 8.24 (t, *J* = 1.9 Hz, 1H), 8.11 (t, *J* = 1.6 Hz, 1H), 7.47 (d, *J* = 9.0 Hz, 2H), 7.11 (d, *J* = 9.0 Hz, 2H), 4.63 (s, 2H), 3.80 (s, 3H). ^13^C NMR (151 MHz, DMSO*-d*_6_) δ 161.19, 154.24, 148.97, 142.08, 138.81, 126.93, 126.02, 125.83, 123.68, 122.28, 115.53, 56.24, 35.44. Elem. Anal. Calcd for C_15_H_12_BrN_5_O_3_S: C, 42.67; H, 2.86; N, 16.59; S, 7.59. Found: C, 42.36; H, 2.55; N, 16.48; S, 7.66.

##### 5-((3-Bromo-5-nitrobenzyl)sulfanyl)-1-(4-chlorophenyl)-1*H*-tetrazole (**55c**)

Yield: 70% as a white solid; mp 182–183 °C. ^1^H NMR (600 MHz, DMSO*-d*_6_) δ 8.32 (t, *J* = 2.1, 1H), 8.24 (t, *J* = 2.0 Hz, 1H), 8.11 (t, *J* = 1.7 Hz, 1H), 7.68 (d, *J* = 8.9 Hz, 2H), 7.63 (d, *J* = 8.9 Hz, 2H), 4.65 (s, 2H). ^13^C NMR (151 MHz, DMSO*-d*_6_) δ 154.25, 148.96, 141.97, 138.85, 135.86, 132.27, 130.55, 127.06, 125.85, 123.72, 122.30, 35.63. Elem. Anal. Calcd for C_14_H_9_BrClN_5_O_2_S: C, 39.41; H, 2.13; N, 16.41; S, 7.51. Found: C, 39.58; H, 1.87; N, 16.55; S, 7.53.

##### 5-((3-Bromo-5-nitrobenzyl)sulfanyl)-1-(4-bromophenyl)-1*H*-tetrazole (**55d**)

Yield: 70% as a yellowish solid; mp 187–188 °C. ^1^H NMR (600 MHz, DMSO*-d*_6_) δ 8.32 (t, *J* = 2.0 Hz, 1H), 8.24 (t, *J* = 2.0 Hz, 1H), 8.11 (t, *J* = 1.7 Hz, 1H), 7.81 (d, *J* = 8.7 Hz, 2H), 7.55 (d, *J* = 8.7 Hz, 2H), 4.65 (s, 2H). ^13^C NMR (151 MHz, DMSO*-d*_6_) δ 154.20, 148.97, 141.96, 138.85, 133.50, 132.70, 127.22, 125.85, 124.43, 123.72, 122.30, 35.65. Elem. Anal. Calcd for C_14_H_9_Br_2_N_5_O_2_S: C, 35.69; H, 1.93; N, 14.87; S, 6.80. Found: C, 35.61; H, 1.71; N, 14.74; S, 6.68.

##### 5-((3-Bromo-5-nitrobenzyl)sulfanyl)-1-cyclohexyl-1*H*-tetrazole (**55e**)

Yield: 88% as a white solid; mp 112–113 °C. ^1^H NMR (600 MHz, DMSO*-d*_6_) δ 8.31 (t, *J* = 1.8 Hz, 1H), 8.25 (t, *J* = 2.0 Hz, 1H), 8.10 (t, *J* = 1.7 Hz, 1H), 4.63 (s, 2H), 4.20 (tt, *J* = 11.5, 3.9 Hz, 1H), 1.86–1.82 (m, 2H), 1.79–1.72 (m, 2H), 1.71–1.65 (m, 2H), 1.63–1.56 (m, 1H), 1.41–1.28 (m, 2H), 1.25–1.11 (m, 1H). ^13^C NMR (151 MHz, DMSO*-d*_6_) δ 152.13, 148.98, 142.24, 138.78, 125.82, 123.59, 122.33, 58.01, 35.52, 32.16, 24.96, 24.92. Elem. Anal. Calcd for C_14_H_16_BrN_5_O_2_S: C, 42.22; H, 4.05; N, 17.58; S, 8.05. Found: C, 42.61; H, 3.91; N, 17.87; S, 8.07.

#### 1-Alkyl/Aryl-((3-cyano-5-nitrobenzyl)sulfanyl)-1*H*-tetrazoles **56a**–**56e**

3-Cyano-5-nitrobenzyl chloride (**39**) was used as the alkylating agent. The reactions were stirred overnight.

##### 5-((3-Cyano-5-nitrobenzyl)sulfanyl)-1-phenyl-1*H*-tetrazole (**56a**)

Yield: 88% as a white solid; mp 140–142 °C. ^1^H NMR (600 MHz, DMSO-*d*_6_) δ 8.63 (t, *J* = 1.9 Hz, 1H), 8.62–8.60 (m, 1H), 8.36 (t, *J* = 1.5 Hz, 1H), 7.64–7.55 (m, 5H), 4.71 (s, 2H). ^13^C NMR (151 MHz, DMSO-*d*_6_) δ 154.07, 148.44, 141.64, 139.39, 133.44, 131.25, 130.54, 129.03, 127.15, 125.13, 117.38, 113.26, 35.32. Elem. Anal. Calcd for C_15_H_10_N_6_O_2_S: C, 53.25; H, 2.98; N, 24.84; S, 9.48. Found: C, 53.11; H, 2.95; N, 24.52; S, 9.87.

##### 5-((3-Cyano-5-nitrobenzyl)sulfanyl)-1-(4-methoxyphenyl)-1*H*-tetrazole (**56b**)

Yield: 75% as a yellowish solid; mp 121–122 °C. ^1^H NMR (600 MHz, DMSO-*d*_6_) δ 8.62–8.60 (m, 2H), 8.34 (t, *J* = 1.5 Hz, 1H), 7.48 (d, *J* = 9.0 Hz, 2H), 7.11 (d, *J* = 9.0 Hz, 2H), 4.68 (s, 2H), 3.80 (s, 3H). ^13^C NMR (151 MHz, DMSO-*d*_6_) δ 161.19, 154.17, 148.42, 141.72, 139.34, 128.98, 127.12, 126.93, 126.01, 117.38, 115.53, 113.24, 56.23, 35.28. Elem. Anal. Calcd for C_16_H_12_N_6_O_3_S: C, 52.17; H, 3.28; N, 22.81; S, 8.70. Found: C, 52.34; H, 3.31; N, 22.6; S, 8.77.

##### 1-(4-Chlorophenyl)-5-((3-cyano-5-nitrobenzyl)sulfanyl)-1*H*-tetrazole (**56c**)

Yield: 90% as a white solid; mp 143–144 °C. ^1^H NMR (600 MHz, DMSO-*d*_6_) δ 8.62 (t, *J* = 1.9 Hz, 1H), 8.61 (t, *J* = 1.8 Hz, 1H), 8.35 (t, *J* = 1.5 Hz, 1H), 7.68 (d, *J* = 8.8 Hz, 2H), 7.64 (d, *J* = 8.8 Hz, 2H), 4.70 (s, 2H). ^13^C NMR (151 MHz, DMSO-*d*_6_) δ 154.20, 148.42, 141.61, 139.37, 135.88, 132.27, 130.57, 129.02, 127.14, 127.07, 117.37, 113.26, 35.44. Elem. Anal. Calcd for C_15_H_9_ClN_6_O_2_S: C, 48.33; H, 2.43; N, 22.54; S, 8.60. Found: C, 48.11; H, 2.17; N, 22.59; S, 8.80.

##### 1-(4-Bromophenyl)-5-((3-cyano-5-nitrobenzyl)sulfanyl)-1*H*-tetrazole (**56d**)

Yield: 70% as a yellow solid; mp 159–160 °C. ^1^H NMR (600 MHz, DMSO-*d*_6_) δ 8.63–8.60 (m, 2H), 8.35 (t, *J* = 1.6 Hz, 1H), 7.82 (d, *J* = 8.7 Hz, 2H), 7.57 (d, *J* = 8.7 Hz, 2H), 4.70 (s, 2H). ^13^C NMR (151 MHz, DMSO-*d*_6_) δ 154.15, 148.42, 141.61, 139.37, 133.52, 132.70, 129.02, 127.23, 127.14, 124.45, 117.37, 113.26, 35.45. Elem. Anal. Calcd for C_15_H_9_BrN_6_O_2_S: C, 43.18; H, 2.17; N, 20.14; S, 7.68. Found: C, 43.39; H, 1.92; N, 20.05; S, 7.62.

##### 5-((3-Cyano-5-nitrobenzyl)sulfanyl)-1-cyclohexyl-1*H*-tetrazole (**56e**)

Yield: 86% as a white solid; mp 121–123 °C. ^1^H NMR (600 MHz, DMSO-*d*_6_) δ 8.63–8.61 (m, 2H), 8.35 (t, *J* = 1.5 Hz, 1H), 4.69 (s, 2H), 4.22 (tt, *J* = 11.5, 3.9 Hz, 1H), 1.89–1.84 (m, 2H), 1.79–1.74 (m, 2H), 1.72–1.64 (m, 2H), 1.62–1.58 (m, 1H), 1.41–1.31 (m, 2H), 1.22–1.14 (m, 1H). ^13^C NMR (151 MHz, DMSO-*d*_6_) δ 152.13, 148.46, 141.86, 139.38, 128.94, 127.15, 117.34, 113.27, 58.02, 35.27, 32.15, 24.96, 24.90. Elem. Anal. Calcd for C_15_H_16_N_6_O_2_S: C, 52.31; H, 4.68; N, 24.40; S, 9.31. Found: C, 52.13; H, 4.55; N, 24.34; S, 9.78.

#### 2-Alkyl/Aryl-5-((3-nitro-5-(trifluoromethyl)benzyl)sulfanyl)-1,3,4-oxadiazoles **57a**–**57e**

3-Nitro-5-(trifluoromethyl)benzyl bromide (**35**) was used as the alkylating agent. The reactions were completed in 1 h.

##### 2-((3-Nitro-5-(trifluoromethyl)benzyl)sulfanyl)-5-phenyl-1,3,4-oxadiazole (**57a**)

Yield: 90% as a yellowish solid; 89–91 °C. ^1^H NMR (600 MHz, DMSO*-d*_6_) δ 8.69 (s, 1H), 8.36 (s, 2H), 7.92–7.87 (m, 2H), 7.60–7.56 (m, 1H), 7.55–7.50 (m, 2H), 4.75 (s, 2H). ^13^C NMR (151 MHz, DMSO*-d*_6_) δ 166.00, 163.33, 148.69, 142.44, 132.65, 130.82 (q, *J* = 33.2 Hz), 129.91, 128.46, 128.38, 126.94, 123.44, 123.44 (q, *J* = 273.1 Hz), 120.20, 34.82. HRMS (ESI+) calcd for (C_16_H_10_F_3_N_3_O_3_S + H^+^) *m*/*z*: 382.04677 (100%), 383.05013 (17%); found: 382.0472 (100%); 383.0498 (18%).

##### 2-(4-Methoxyphenyl)-5-((3-nitro-5-(trifluoromethyl)benzyl)sulfanyl)-1,3,4-oxadiazole (**57b**)

Yield: 82% as a yellowish solid; 105–107 °C. ^1^H NMR (500 MHz, DMSO*-d*_6_) δ 8.71 (t, *J* = 1.9 Hz, 1H), 8.39 (s, 1H), 8.37 (s, 1H), 7.85 (d, *J* = 8.9 Hz, 2H), 7.09 (d, *J* = 8.9 Hz, 2H), 4.76 (s, 2H), 3.83 (s, 3H). ^13^C NMR (126 MHz, DMSO*-d*_6_) δ 165.89, 162.56, 162.39, 148.58, 142.37, 132.53 (q, *J* = 3.5 Hz), 130.73 (q, *J* = 33.2 Hz), 128.73, 128.30, 123.34 (d, *J* = 273.2 Hz), 120.08 (q, *J* = 4.1 Hz), 115.68, 115.25, 55.98, 34.76. HRMS (ESI+) calcd for (C_17_H_12_F_3_N_3_O_4_S + H^+^) *m*/*z*: 412.05734 (100%), 413.06069 (18%); found: 412.0580 (100%), 413.0604 (18%).

##### 2-(4-Chlorophenyl)-5-((3-nitro-5-(trifluoromethyl)benzyl)sulfanyl)-1,3,4-oxadiazole (**57c**)

Yield: 82% as a white solid; mp 118–120 °C. ^1^H NMR (500 MHz, DMSO*-d*_6_) δ 8.71 (t, *J* = 1.9 Hz, 1H), 8.39 (s, 2H), 7.93 (d, *J* = 8.6 Hz, 2H), 7.62 (d, *J* = 8.6 Hz, 2H), 4.78 (s, 2H). ^13^C NMR (126 MHz, DMSO*-d*_6_) δ 165.17, 163.55, 148.58, 142.25, 137.26, 132.57 (q, *J* = 3.6 Hz), 130.73 (q, *J* = 33.3 Hz), 129.97, 128.66, 128.33, 123.34 (q, *J* = 272.8 Hz), 122.25, 120.12 (q, *J* = 3.8 Hz), 34.72. HRMS (ESI+) calcd for (C_16_H_9_ClF_3_N_3_O_3_S + H^+^) *m*/*z*: 416.00780 (100%), 418.00485 (32%); found: 416.0088 (100%), 418.0055 (38%).

##### 2-(4-Bromophenyl)-5-((3-nitro-5-(trifluoromethyl)benzyl)sulfanyl)-1,3,4-oxadiazole (**57d**)

Yield: 83% as a white solid; 126–127 °C. ^1^H NMR (500 MHz, DMSO*-d*_6_) δ 8.72 (t, *J* = 1.9 Hz, 1H), 8.41–8.37 (m, 2H), 7.86 (d, *J* = 8.6 Hz, 2H), 7.77 (d, *J* = 8.6 Hz, 2H), 4.78 (s, 2H). ^13^C NMR (126 MHz, DMSO*-d*_6_) δ 165.29, 163.57, 148.59, 142.25, 132.91, 132.57 (q, *J* = 3.6 Hz), 130.73 (q, *J* = 33.3 Hz), 128.78, 128.34, 126.16, 123.35 (d, *J* = 273.0 Hz), 122.59, 120.13 (d, *J* = 4.0 Hz), 34.72. HRMS (ESI+) calcd for (C_16_H_9_BrF_3_N_3_O_3_S + H^+^) *m*/*z*: 459.95729 (100%), 461.95524 (97%); found: 461.9563 (100%), 459.9581 (97%).

##### 2-Cyclohexyl-5-((3-nitro-5-(trifluoromethyl)benzyl)sulfanyl)-1,3,4-oxadiazole (**57e**)

Yield: 98% as a colorless oil. ^1^H NMR (500 MHz, DMSO*-d*_6_) δ 8.65 (t, *J* = 1.9 Hz, 1H), 8.40 (t, *J* = 1.9 Hz, 1H), 8.32 (s, 1H), 4.68 (s, 2H), 2.91–2.85 (m, 1H), 1.95–1.86 (m, 2H), 1.71–1.58 (m, 3H), 1.47–1.10 (m, 5H). ^13^C NMR (126 MHz, DMSO*-d*_6_) δ 171.44, 162.30, 148.57, 142.42, 132.48 (q, *J* = 3.5 Hz), 130.73 (q, *J* = 33.4 Hz), 128.26, 123.35 (d, *J* = 272.8 Hz), 120.07 (q, *J* = 3.9 Hz), 34.67, 34.60, 29.79, 25.51, 25.05. HRMS (ESI+) calcd for (C_16_H_16_F_3_N_3_O_3_S + H^+^) *m*/*z*: 388.09372 (100%), 389.09708 (17%); found: 388.0941 (100%), 389.0970 (18%).

#### 2-Alkyl/Aryl-5-((3-chloro-5-nitrobenzyl)sulfanyl)-1,3,4-oxadiazoles **58a**–**58e**

3-Chloro-5-nitrobenzyl chloride (**36**) was used as the alkylating agent. The reactions were stirred overnight.

##### 2-((3-Chloro-5-nitrobenzyl)sulfanyl)-5-phenyl-1,3,4-oxadiazole (**58a**)

Yield: 86% as a white solid; mp 95–97 °C. ^1^H NMR (500 MHz, DMSO*-d*_6_) δ 8.39 (t, *J* = 1.8 Hz, 1H), 8.18 (t, *J* = 2.1 Hz, 1H), 8.08 (t, *J* = 1.7 Hz, 1H), 7.96–7.89 (m, 2H), 7.65–7.55 (m, 3H), 4.69 (s, 2H). ^13^C NMR (126 MHz, DMSO*-d*_6_) δ 165.62, 163.03, 148.63, 141.94, 135.63, 134.01, 132.27, 129.58, 126.58, 123.10, 122.99, 122.80, 34.46. Elem. Anal. Calcd for C_15_H_10_ClN_3_O_3_S: C, 51.81; H, 2.90; N, 12.08; S, 9.22. Found: C, 51.44; H, 2.63; N, 12.07; S, 9.24.

##### 2-((3-Chloro-5-nitrobenzyl)sulfanyl)-5-(4-methoxyphenyl)-1,3,4-oxadiazole (**58b**)

Yield: 87% as a yellowish solid; mp 129–131 °C. ^1^H NMR (600 MHz, DMSO*-d*_6_) δ 8.35 (t, *J* = 1.9 Hz, 1H), 8.15 (t, *J* = 2.1 Hz, 1H), 8.04 (t, *J* = 1.7 Hz, 1H), 7.84 (d, *J* = 8.9 Hz, 2H), 7.08 (d, *J* = 8.8 Hz, 2H), 4.64 (s, 2H), 3.80 (s, 3H). ^13^C NMR (151 MHz, DMSO*-d*_6_) δ 165.96, 162.66, 162.53, 149.01, 142.34, 135.96, 134.37, 128.84, 123.32, 123.14, 115.80, 115.39, 56.08, 34.86. Elem. Anal. Calcd for C_16_H_12_ClN_3_O_4_S: C, 50.87; H, 3.20; N, 11.12; S, 8.49. Found: C, 50.58; H, 2.92; N, 11.16; S, 8.88.

##### 2-((3-Chloro-5-nitrobenzyl)sulfanyl)-5-(4-chlorophenyl)-1,3,4-oxadiazole (**58c**)

Yield: 89% as a white solid; mp 140–142 °C. ^1^H NMR (500 MHz, DMSO*-d*_6_) δ 8.39 (t, *J* = 1.9 Hz, 1H), 8.18 (t, *J* = 2.1 Hz, 1H), 8.08 (t, *J* = 1.7 Hz, 1H), 7.94 (d, *J* = 8.7 Hz, 2H), 7.64 (d, *J* = 8.7 Hz, 2H), 4.69 (s, 2H). ^13^C NMR (126 MHz, DMSO*-d*_6_) δ 164.88, 163.33, 148.63, 141.86, 136.98, 135.63, 134.02, 129.74, 128.40, 122.99, 122.82, 122.00, 34.44. Elem. Anal. Calcd for C_15_H_9_Cl_2_N_3_O_3_S: C, 47.14; H, 2.37; N, 10.99; S, 8.39. Found: C, 47.49; H, 2.31; N, 10.79; S, 8.0.

##### 2-(4-Bromophenyl)-5-((3-chloro-5-nitrobenzyl)sulfanyl)-1,3,4-oxadiazole (**58d**)

Yield: 90% as a white solid; mp 132–134 °C. ^1^H NMR (600 MHz, DMSO*-d*_6_) δ 8.35 (t, *J* = 1.9 Hz, 1H), 8.15 (t, *J* = 2.1 Hz, 1H), 8.05 (t, *J* = 1.8 Hz, 1H), 7.83 (d, *J* = 8.6 Hz, 2H), 7.75 (d, *J* = 8.6 Hz, 2H), 4.66 (s, 2H). ^13^C NMR (151 MHz, DMSO*-d*_6_) δ 165.36, 163.71, 149.01, 142.21, 135.98, 134.39, 133.02, 128.87, 126.24, 123.35, 123.18, 122.70, 34.82. Elem. Anal. Calcd for C_15_H_9_BrClN_3_O_3_S: C, 42.23; H, 2.13; N, 9.85; S, 7.51. Found: C, 42.35; H, 1.90; N, 9.86; S, 7.78.

##### 2-((3-Chloro-5-nitrobenzyl)sulfanyl)-5-cyclohexyl-1,3,4-oxadiazole (**58e**)

Yield: 91% as a white solid; mp 78–80 °C. ^1^H NMR (500 MHz, DMSO*-d*_6_) δ 8.31 (t, *J* = 1.8 Hz, 1H), 8.19 (t, *J* = 2.0 Hz, 1H), 8.01 (t, *J* = 1.8 Hz, 1H), 4.59 (s, 2H), 2.90 (tt, *J* = 11.0, 3.7 Hz, 1H), 2.00–1.88 (m, 2H), 1.73–1.57 (m, 3H), 1.49–1.40 (m, 2H), 1.38–1.29 (m, 2H), 1.27–1.17 (m, 1H). ^13^C NMR (126 MHz, DMSO*-d*_6_) δ 171.15, 162.04, 148.61, 142.00, 135.56, 134.00, 122.91, 122.74, 34.42, 34.32, 29.53, 25.25, 24.78. Elem. Anal. Calcd for C_15_H_16_ClN_3_O_3_S: C, 50.92; H, 4.56; N, 11.88; S, 9.06. Found: C, 50.56; H, 4.32; N, 11.91; S, 9.42.

#### 2-Alkyl/Aryl-5-((3-fluoro-5-nitrobenzyl)sulfanyl)-1,3,4-oxadiazoles **59a**–**59e**

3-Fluoro-5-nitrobenzyl chloride (**37**) was used as the alkylating agent. The reactions were stirred overnight.

##### 2-((3-Fluoro-5-nitrobenzyl)sulfanyl)-5-phenyl-1,3,4-oxadiazole (**59a**)

Yield: 87% as a white solid; mp 138–139 °C. ^1^H NMR (600 MHz, DMSO-*d*_6_) δ 8.27 (t, *J* = 1.8 Hz, 1H), 7.99 (dt, *J* = 8.5, 2.3 Hz, 1H), 7.92–7.88 (m, 2H), 7.86 (dt, *J* = 9.2, 2.0 Hz, 1H), 7.59–7.55 (m, 1H), 7.58–7.48 (m, 2H), 4.67 (s, 2H). ^13^C NMR (151 MHz, DMSO-*d*_6_) δ 165.99, 163.39, 161.95 (d, *J* = 248.2 Hz), 149.13 (d, *J* = 9.5 Hz), 142.57 (d, *J* = 8.0 Hz), 132.62, 129.93, 126.94, 123.46 (d, *J* = 4.6 Hz), 123.30, 120.82 (d, *J* = 2.9 Hz), 111.01 (d, *J* = 26.8 Hz), 35.00. HRMS (ESI+) calcd for (C_15_H_10_FN_3_O_3_S + H^+^) *m*/*z*: 332.0505; found: 332.0505.

##### 2-((3-Fluoro-5-nitrobenzyl)sulfanyl)-5-(4-methoxyphenyl)-1,3,4-oxadiazole (**59b**)

Yield: 85% as a white solid; mp 115–117 °C. ^1^H NMR (600 MHz, DMSO-*d*_6_) δ 8.25 (t, *J* = 1.8 Hz, 1H), 7.98 (dt, *J* = 8.6, 2.3 Hz, 1H), 7.88–7.79 (m, 3H),7.07 (d, *J* = 8.7 Hz, 2H), 4.65 (s, 2H), 3.80 (s, 3H). ^13^C NMR (151 MHz, DMSO-*d*_6_) δ 165.96, 162.65, 162.52, 161.92 (d, *J* = 247.9 Hz), 149.13 (d, *J* = 9.5 Hz), 142.61 (d, *J* = 8.2 Hz), 128.82, 123.34 (d, *J* = 22.3 Hz), 120.79 (d, *J* = 2.9 Hz), 115.80, 115.38, 110.98 (d, *J* = 26.8 Hz), 56.06, 35.02. HRMS (ESI+) calcd for (C_16_H_12_FN_3_O_4_S + H^+^) *m*/*z*: 362.0605; found: 362.0618.

##### 2-(4-Chlorophenyl)-5-((3-fluoro-5-nitrobenzyl)sulfanyl)-1,3,4-oxadiazole (**59c**)

Yield: 82% as a white solid; mp 141–142 °C. ^1^H NMR (600 MHz, DMSO-*d*_6_) δ 8.26 (t, *J* = 1.9 Hz, 1H), 7.99 (dt, *J* = 8.6, 2.3 Hz, 1H), 7.91 (d, *J* = 8.6 Hz, 2H), 7.85 (dt, *J* = 9.1, 2.0 Hz, 1H), 7.61 (d, *J* = 8.5 Hz, 2H), 4.67 (s, 2H). ^13^C NMR (151 MHz, DMSO-*d*_6_) δ 165.25, 163.68, 161.94 (d, *J* = 248.3 Hz), 149.13 (d, *J* = 9.3 Hz), 142.49 (d, *J* = 8.1 Hz), 137.34, 130.10, 128.76, 123.37 (d, *J* = 22.4 Hz), 122.37, 120.82 (d, *J* = 3.1 Hz), 111.03 (d, *J* = 26.8 Hz), 34.98. HRMS (ESI+) calcd for (C_15_H_9_ClFN_3_O_3_S + H^+^) *m*/*z*: 366.0115; found: 366.0116.

##### 2-(4-Bromophenyl)-5-((3-fluoro-5-nitrobenzyl)sulfanyl)-1,3,4-oxadiazole (**59d**)

Yield: 87% as a white solid; mp 149–151 °C. ^1^H NMR (500 MHz, DMSO-*d*_6_) δ 8.30 (t, *J* = 1.8 Hz, 1H), 8.03 (dt, *J* = 8.7, 2.3 Hz, 1H), 7.92–7.82 (m, 3H), 7.78 (d, *J* = 8.5 Hz, 2H), 4.70 (s, 2H). ^13^C NMR (126 MHz, DMSO-*d*_6_) δ 165.01, 163.36, 161.58 (d, *J* = 248.3 Hz), 148.77 (d, *J* = 9.6 Hz), 142.14 (d, *J* = 8.1 Hz), 132.68, 128.52, 125.89, 123.04 (d, *J* = 22.4 Hz), 122.35, 120.49 (d, *J* = 3.0 Hz), 110.70 (d, *J* = 26.7 Hz), 34.60. HRMS (ESI+) calcd for (C_15_H_9_BrFN_3_O_3_S + H^+^): 409.9605 (100%), 411.9585 (97%); found: 409.9609 (97%), 411.9590 (100%).

##### 2-Cyclohexyl-5-((3-fluoro-5-nitrobenzyl)sulfanyl)-1,3,4-oxadiazole (**59e**)

Yield: 88% as a white solid; mp 64–66 °C. ^1^H NMR (500 MHz, DMSO-*d*_6_) δ 8.22 (t, *J* = 1.8 Hz, 1H), 8.03 (dt, *J* = 8.7, 2.3 Hz, 1H), 7.86–7.79 (m, 1H), 4.60 (s, 2H), 2.94–2.85 (m, 1H), 1.96–1.87 (m, 2H), 1.73–1.64 (m, 2H), 1.67–1.57 (m, 1H), 1.50–1.40 (m, 2H), 1.42–1.22 (m, 2H), 1.25–1.15 (m, 1H). ^13^C NMR (126 MHz, DMSO-*d*_6_) δ 171.43, 162.35, 161.86 (d, *J* = 248.5 Hz), 149.00 (d, *J* = 9.7 Hz), 142.57 (d, *J* = 7.9 Hz), 123.27 (d, *J* = 22.4 Hz), 120.68 (d, *J* = 2.9 Hz), 110.89 (d, *J* = 26.8 Hz), 34.83, 34.58, 29.79, 25.53, 25.05. HRMS (ESI+) calcd for (C_15_H_16_FN_3_O_3_S + H^+^) *m*/*z*: 338.0975; found: 338.0982.

#### 2-Alkyl/Aryl-5-((3-bromo-5-nitrobenzyl)sulfanyl)-1,3,4-oxadiazoles **60a**–**60e**

3-Bromo-5-nitrobenzyl chloride (**38**) was used as the alkylating agent. The reactions were stirred overnight.

##### 2-((3-Bromo-5-nitrobenzyl)sulfanyl)-5-phenyl-1,3,4-oxadiazole (**60a**)

Yield: 73% as a white solid; mp 100–101 °C. ^1^H NMR (500 MHz, DMSO-*d*_6_) δ 8.43 (t, *J* = 1.8 Hz, 1H), 8.29 (t, *J* = 2.0 Hz, 1H), 8.22 (t, *J* = 1.7 Hz, 1H), 7.96–7.91 (m, 2H), 7.64–7.55 (m, 3H), 4.68 (s, 2H). ^13^C NMR (126 MHz, DMSO-*d*_6_) δ 165.60, 163.01, 148.65, 142.10, 138.47, 132.25, 129.56, 126.58, 125.50, 123.32, 123.09, 121.95, 34.38. Elem. Anal. Calcd for C_15_H_10_BrN_3_O_3_S: C, 45.93; H, 2.57; N, 10.71; S, 8.17. Found: C, 45.95; H, 2.29; N, 10.73; S, 8.38.

##### 2-((3-Bromo-5-nitrobenzyl)sulfanyl)-5-(4-methoxyphenyl)-1,3,4-oxadiazole (**60b**)

Yield: 73% as a white solid; mp 116–118 °C. ^1^H NMR (500 MHz, DMSO-*d*_6_) δ 8.41 (t, *J* = 1.8 Hz, 1H), 8.29 (t, *J* = 2.0 Hz, 1H), 8.20 (t, *J* = 1.7 Hz, 1H), 7.87 (d, *J* = 8.9 Hz, 2H), 7.11 (d, *J* = 8.9 Hz, 2H), 4.66 (s, 2H), 3.84 (s, 3H). ^13^C NMR (126 MHz, DMSO-*d*_6_) δ 165.56, 162.26, 162.14, 148.63, 142.13, 138.44, 128.46, 125.46, 123.28, 121.93, 115.41, 115.00, 55.70, 34.39. Elem. Anal. Calcd for C_16_H_12_BrN_3_O_4_S: C, 45.51; H, 2.86; N, 9.95; S, 7.59. Found: C, 45.90; H, 3.03; N, 9.56; S, 7.20.

##### 2-((3-Bromo-5-nitrobenzyl)sulfanyl)-5-(4-chlorophenyl)-1,3,4-oxadiazole (**60c**)

Yield: 70% as a white solid; mp 157–158 °C. ^1^H NMR (600 MHz, DMSO-*d*_6_) δ 8.39 (t, *J* = 1.8 Hz, 1H), 8.25 (t, *J* = 2.0 Hz, 1H), 8.18 (t, *J* = 1.7 Hz, 1H), 7.91 (d, *J* = 8.6 Hz, 2H), 7.61 (d, *J* = 8.6 Hz, 2H), 4.65 (s, 2H). ^13^C NMR (151 MHz, DMSO-*d*_6_) δ 165.24, 163.69, 149.03, 142.40, 138.85, 137.35, 130.11, 128.77, 125.89, 123.70, 122.37, 122.33, 34.75. Elem. Anal. Calcd for C_15_H_9_BrClN_3_O_3_S: C, 42.23; H, 2.13; N, 9.85; S, 7.51. Found: C, 41.84; H, 1.80; N, 9.78; S, 7.46.

##### 2-((3-Bromo-5-nitrobenzyl)sulfanyl)-5-(4-bromophenyl)-1,3,4-oxadiazole (**60d**)

Yield: 68% as a yellowish solid; mp 152–153 °C. ^1^H NMR (600 MHz, DMSO-*d*_6_) δ 8.39 (t, *J* = 1.8 Hz, 1H), 8.26 (t, *J* = 1.5 Hz, 1H), 8.18 (t, *J* = 1.7 Hz, 1H), 7.85–7.81 (m, 2H), 7.77–7.72 (m, 2H), 4.65 (s, 2H). ^13^C NMR (151 MHz, DMSO-*d*_6_) δ 165.36, 163.71, 149.03, 142.40, 138.85, 133.03, 128.88, 126.24, 125.89, 123.70, 122.71, 122.34, 34.74. Elem. Anal. Calcd for C_15_H_9_Br_2_N_3_O_3_S: C, 38.24; H, 1.93; N, 8.92; S, 6.81. Found: C, 38.3; H, 1.61; N, 8.95; S, 6.95.

##### 2-((3-Bromo-5-nitrobenzyl)sulfanyl)-5-cyclohexyl-1,3,4-oxadiazole (**60e**)

Yield: 75% as a white solid; mp 67–68 °C. ^1^H NMR (600 MHz, DMSO-*d*_6_) δ 8.31 (t, *J* = 1.8 Hz, 1H), 8.26 (t, *J* = 2.0 Hz, 1H), 8.10 (t, *J* = 1.7 Hz, 1H), 4.54 (s, 2H), 2.87 (tt, *J* = 11.0, 3.7 Hz, 1H), 1.92–1.86 (m, 2H), 1.69–1.63 (m, 2H), 1.61–1.54 (m, 1H), 1.46–1.37 (m, 2H), 1.36–1.26 (m, 2H), 1.23–1.17 (m, 1H). ^13^C NMR (151 MHz, DMSO-*d*_6_) δ 171.53, 162.40, 149.00, 142.54, 138.78, 125.83, 123.62, 122.31, 34.71, 34.69, 29.91, 25.61, 25.15. Elem. Anal. Calcd for C_15_H_19_BrN_3_O_3_S: C, 45.24; H, 4.05; N, 10.55; S, 8.05. Found: C, 45.09; H, 3.93; N, 10.50; S, 8.04.

#### 2-Alkyl/Aryl-5-((3-cyano-5-nitrobenzyl)sulfanyl)-1,3,4-oxadiazoles **61a**–**61e**

3-Cyano-5-nitrobenzyl chloride (**39**) was used as the alkylating agent. The reactions were stirred overnight.

##### 2-((3-Cyano-5-nitrobenzyl)sulfanyl)-5-phenyl-1,3,4-oxadiazole (**61a**)

Yield: 79% as a yellowish solid; mp 125–126 °C. ^1^H NMR (600 MHz, DMSO-*d*_6_) δ 8.69 (t, *J* = 2.0 Hz, 1H), 8.62 (t, *J* = 1.8 Hz, 1H), 8.41 (t, *J* = 1.6 Hz, 1H), 7.92–7.87 (m, 2H), 7.61–7.51 (m, 3H), 4.70 (s, 2H). ^13^C NMR (151 MHz, DMSO-*d*_6_) δ 166.00, 163.29, 148.50, 142.06, 139.40, 132.63, 129.93, 128.99, 127.18, 126.95, 123.47, 117.36, 113.32, 34.63. Elem. Anal. Calcd for C_16_H_10_N_4_O_3_S: C, 56.80; H, 2.98; N, 16.56; S, 9.48. Found: C, 56.49; H, 2.80; N, 16.31; S, 9.46.

##### 2-((3-Cyano-5-nitrobenzyl)sulfanyl)-5-(4-methoxyphenyl)-1,3,4-oxadiazole (**61b**)

Yield: 80% as a white solid; mp 147–148 °C. ^1^H NMR (600 MHz, DMSO-*d*_6_) δ 8.68 (t, *J* = 1.9 Hz, 1H), 8.63–8.62 (m, 1H), 8.40 (t, *J* = 1.6 Hz, 1H), 7.83 (d, *J* = 9.0 Hz, 2H), 7.07 (d, *J* = 9.0 Hz, 2H), 4.68 (s, 2H), 3.80 (s, 3H). ^13^C NMR (151 MHz, DMSO-*d*_6_) δ 165.97, 162.65, 162.44, 148.49, 142.09, 139.38, 128.96, 128.84, 127.16, 117.37, 115.80, 115.38, 113.30, 56.07, 34.65. Elem. Anal. Calcd for C_17_H_12_N_4_O_4_S: C, 55.43; H, 3.28; N, 15.21; S, 8.70. Found: C, 55.29; H, 3.10; N, 15.14; S, 8.79.

##### 2-(4-Chlorophenyl)-5-((3-cyano-5-nitrobenzyl)sulfanyl)-1,3,4-oxadiazole (**61c**)

Yield: 77% as a white solid; mp 166–168 °C. ^1^H NMR (500 MHz, DMSO-*d*_6_) δ 8.72 (t, *J* = 1.9 Hz, 1H), 8.66 (t, *J* = 1.8 Hz, 1H), 8.44 (t, *J* = 1.5 Hz, 1H), 7.94 (d, *J* = 8.6 Hz, 2H), 7.64 (d, *J* = 8.6 Hz, 2H), 4.73 (s, 2H). ^13^C NMR (126 MHz, DMSO-*d*_6_) δ 164.88, 163.21, 148.11, 141.59, 139.03, 136.96, 129.72, 128.62, 128.39, 126.83, 122.01, 117.00, 112.94, 34.24. Elem. Anal. Calcd for C_16_H_9_ClN_4_O_3_S: C, 51.55; H, 2.43; N, 15.03; S, 8.60. Found: C, 51.65; H, 2.36; N, 14.87; S, 8.65.

##### 2-(4-Bromophenyl)-5-((3-cyano-5-nitrobenzyl)sulfanyl)-1,3,4-oxadiazole (**61d**)

Yield: 71% as a yellow solid; mp 175–176 °C. ^1^H NMR (500 MHz, CDCl_3_) δ 8.63 (t, *J* = 2.0 Hz, 1H), 8.45 (t, *J* = 1.8 Hz, 1H), 8.19 (t, *J* = 1.6 Hz, 1H), 7.85 (d, *J* = 8.6 Hz, 2H), 7.65 (d, *J* = 8.6 Hz, 2H), 4.61 (s, 2H). ^13^C NMR (126 MHz, CDCl_3_) δ 165.77, 162.47, 148.37, 140.51, 138.00, 132.51, 128.06, 126.77, 126.53, 122.03, 116.19, 114.37, 34.72. Elem. Anal. Calcd for C_16_H_9_BrN_4_O_3_S: C, 46.06; H, 2.17; N, 13.43; S, 7.68. Found: C, 46.45; H, 2.21; N, 13.06; S, 7.29.

##### 2-((3-Cyano-5-nitrobenzyl)sulfanyl)-5-cyclohexyl-1,3,4-oxadiazole (**61e**)

Yield: 91% as a white solid; mp 91–92 °C. ^1^H NMR (600 MHz, DMSO-*d*_6_) δ 8.63–8.61 (m, 2H), 8.34 (t, *J* = 1.6 Hz, 1H), 4.59 (s, 2H), 2.86 (tt, *J* = 10.9, 3.7 Hz, 1H), 1.91–1.85 (m, 2H), 1.70–1.63 (m, 2H), 1.61–1.56 (m, 1H), 1.46–1.36 (m, 2H), 1.36–1.25 (m, 2H), 1.24–1.14 (m, 1H). ^13^C NMR (151 MHz, DMSO-*d*_6_) δ 171.53, 162.33, 148.49, 142.14, 139.35, 128.94, 127.15, 117.32, 113.28, 34.68, 34.56, 29.90, 25.60, 25.14. Elem. Anal. Calcd for C_16_H_16_N_4_O_3_S: C, 55.80; H, 4.68; N, 16.27; S, 9.31. Found: C, 55.68; H, 4.59; N, 16.27; S, 9.61.

#### 2-Alkyl/Aryl-5-((3-(methoxycarbonyl)-5-nitrobenzyl)sulfanyl)-1,3,4-oxadiazoles **62a**–**62e**

Methyl 3-(bromomethyl)-5-nitrobenzoate (**40**) was used as the alkylating agent. The reactions were stirred overnight.

##### 2-((3-(Methoxycarbonyl)-5-nitrobenzyl)sulfanyl)-5-phenyl-1,3,4-oxadiazole (**62a**)

Yield: 98% as a white solid; mp 106–107 °C. ^1^H NMR (500 MHz, DMSO-*d*_6_) δ 8.68 (t, *J* = 2.0 Hz, 1H), 8.53 (t, *J* = 1.6 Hz, 1H), 8.51 (dd, *J* = 2.3, 1.5 Hz, 1H), 7.95–7.89 (m, 2H), 7.63–7.52 (m, 3H), 4.77 (s, 2H), 3.89 (s, 3H). ^13^C NMR (126 MHz, DMSO-*d*_6_) δ 165.58, 164.46, 163.07, 148.09, 141.14, 135.90, 132.27, 131.41, 129.57, 128.36, 126.58, 123.08, 123.06, 53.08, 34.54. Elem. Anal. Calcd for C_17_H_13_N_3_O_5_S: C, 54.98; H, 3.53; H, 11.32; S, 8.63. Found: C, 55.08; H, 3.49; N, 11.23; S, 8.81.

##### 2-((3-(Methoxycarbonyl)-5-nitrobenzyl)sulfanyl)-5-(4-methoxyphenyl)-1,3,4-oxadiazole (**62b**)

Yield: 91% as a yellow solid; mp 107–108 °C. ^1^H NMR (500 MHz, DMSO-*d*_6_) δ 8.66 (t, *J* = 2.0 Hz, 1H), 8.52–8.49 (m, 2H), 7.86 (d, *J* = 8.9 Hz, 2H), 7.09 (d, *J* = 8.9 Hz, 2H), 4.75 (s, 2H), 3.90 (s, 3H), 3.83 (s, 3H). ^13^C NMR (126 MHz, DMSO-*d*_6_) δ 165.55, 164.46, 162.27, 162.21, 148.08, 141.17, 135.87, 131.40, 128.47, 128.33, 123.04, 115.42, 115.00, 55.72, 53.09, 34.56. Elem. Anal. Calcd for C_18_H_15_N_3_O_6_S: C, 53.86; H, 3.77; N, 10.47; S, 7.99. Found: C, 53.85; H, 3.52; N, 10.27; S, 7.98.

##### 2-(4-Chlorophenyl)-5-((3-(methoxycarbonyl)-5-nitrobenzyl)sulfanyl)-1,3,4-oxadiazole (**62c**)

Yield: 86% as a white solid; mp 154–155 °C. ^1^H NMR (500 MHz, DMSO-*d*_6_) δ 8.67 (t, *J* = 1.9 Hz, 1H), 8.52 (t, *J* = 1.6 Hz, 1H), 8.50 (dd, *J* = 2.2, 1.5 Hz, 1H), 7.93 (d, *J* = 8.6 Hz, 2H), 7.63 (d, *J* = 8.7 Hz, 2H), 4.77 (s, 2H), 3.90 (s, 3H). ^13^C NMR (126 MHz, DMSO-*d*_6_) δ 164.84, 164.45, 163.37, 148.07, 141.04, 136.97, 135.90, 131.41, 129.72, 128.39, 128.36, 123.07, 121.98, 53.09, 34.52. Elem. Anal. Calcd for C_17_H_12_ClN_3_O_5_S: C, 50.32; H, 2.98; N, 10.35; S, 7.90. Found: C, 50.33; H, 2.99; N, 10.12; S, 7.91.

##### 2-(4-Bromophenyl)-5-((3-(methoxycarbonyl)-5-nitrobenzyl)sulfanyl)-1,3,4-oxadiazole (**62d**)

Yield: 81% as a yellowish solid; mp 145–146 °C. ^1^H NMR (500 MHz, DMSO-*d*_6_) δ 8.67 (t, *J* = 2.0 Hz, 1H), 8.53 (t, *J* = 1.6 Hz, 1H), 8.51 (dd, *J* = 2.3, 1.5 Hz, 1H), 7.86 (d, *J* = 8.6 Hz, 2H), 7.77 (d, *J* = 8.6 Hz, 2H), 4.77 (s, 2H), 3.90 (s, 3H). ^13^C NMR (126 MHz, DMSO-*d*_6_) δ 164.96, 164.46, 163.39, 148.08, 141.04, 135.90, 132.64, 131.41, 128.50, 128.36, 125.87, 123.07, 122.31, 53.09, 34.52. Elem. Anal. Calcd for C_17_H_12_BrN_3_O_5_S: C, 45.35; H, 2.69; N, 9.33; S, 7.12. Found: C, 45.39; H, 2.52; N, 9.11; S, 7.31.

##### 2-Cyclohexyl-5-((3-(methoxycarbonyl)-5-nitrobenzyl)sulfanyl)-1,3,4-oxadiazole (**62e**)

Yield: 97% as a yellowish oil, which crystallized over time; mp 51–53 °C. ^1^H NMR (500 MHz, DMSO-*d*_6_) δ 8.60 (t, *J* = 2.0 Hz, 1H), 8.51 (dd, *J* = 2.3, 1.5 Hz, 1H), 8.46 (t, *J* = 1.6 Hz, 1H), 4.67 (s, 2H), 3.92 (s, 3H), 2.90–2.85 (m, 1H), 1.93–1.88 (m, 2H), 1.70–1.58 (m, 3H), 1.47–1.18 (m, 5H). ^13^C NMR (126 MHz, DMSO-*d*_6_) δ 171.12, 164.46, 162.10, 148.06, 141.23, 135.84, 131.41, 128.30, 123.01, 53.11, 34.49, 34.32, 29.53, 25.25, 24.80. HRMS (ESI+) calcd for (C_17_H_19_N_3_O_5_S + H^+^) *m*/*z*: 378.11182 (100%); found: 378.1123 (100%).

#### 2-Alkyl/aryl-5-((3-(carbamoyl)-5-nitrobenzyl)sulfanyl)-1,3,4-oxadiazoles **63a**–**63e**

3-(Bromomethyl)-5-nitrobenzamide (**41**) was used as the alkylating agent. The reactions were completed in 30 min. The final products **63a**–**63d** had low solubility. Therefore, upon reaction completion, the solvent was evaporated, and the residue was washed with 5% Na_2_CO_3_ (2 × 15 mL), water (2 × 20 mL), and EtOAc (7 mL) to give the final product. Compound **63e** was purified using column chromatography (mobile phase: hexane/EtOAc, 4:1).

##### 2-((3-(Carbamoyl)-5-nitrobenzyl)sulfanyl)-5-phenyl-1,3,4-oxadiazole (**63a**)

Yield: 94% as a white solid; mp 192–193 °C. ^1^H NMR (600 MHz, DMSO-*d*_6_) δ 8.59 (t, *J* = 1.9 Hz, 1H), 8.53 (t, *J* = 1.9 Hz, 1H), 8.44 (t, *J* = 1.6 Hz, 1H), 8.32 (s, 1H, NH-*H*), 7.91–7.86 (m, 2H), 7.68 (s, 1H, NH-*H*), 7.61–7.50 (m, 3H), 4.71 (s, 2H). ^13^C NMR (151 MHz, DMSO-*d*_6_) δ 166.03, 165.94, 163.47, 148.30, 140.58, 136.50, 135.29, 132.60, 129.93, 126.96, 126.87, 123.45, 121.88, 35.22. Elem. Anal. Calcd for C_16_H_12_N_4_O_4_S: C, 53.93; H, 3.39; N, 15.72; S, 9.0. Found: C, 53.98; H, 3.45; N, 15.76; S, 9.36.

##### 2-((3-(Carbamoyl)-5-nitrobenzyl)sulfanyl)-5-(4-methoxyphenyl)-1,3,4-oxadiazole (**63b**)

Yield: 80% as a white solid; mp 157–158 °C. ^1^H NMR (600 MHz, DMSO-*d*_6_) δ 8.59 (t, *J* = 1.9 Hz, 1H), 8.51 (t, *J* = 1.9 Hz, 1H), 8.43 (t, *J* = 1.6 Hz, 1H), 8.32 (s, 1H, NH-*H*), 7.83 (d, *J* = 8.9 Hz, 2H), 7.68 (s, 1H, NH-*H*), 7.06 (d, *J* = 8.9 Hz, 2H), 4.69 (s, 2H), 3.80 (s, 3H). ^13^C NMR (151 MHz, DMSO-*d*_6_) δ 166.01, 165.90, 162.62, 148.30, 140.61, 136.50, 135.28, 128.85, 126.84, 121.85, 115.80, 115.37, 56.06, 35.24. Elem. Anal. Calcd for C_17_H_14_N_4_O_5_S: C, 52.85; H, 3.65; N, 14.50; S, 8.30. Found: C, 53.24; H, 3.54; N, 14.56; S, 8.48.

##### 2-((3-(Carbamoyl)-5-nitrobenzyl)sulfanyl)-5-(4-chlorophenyl)-1,3,4-oxadiazole (**63c**)

Yield: 73% as a white solid; mp 217–218 °C. ^1^H NMR (600 MHz, DMSO-*d*_6_) δ 8.59 (t, *J* = 1.9 Hz, 1H), 8.53 (t, *J* = 1.9 Hz, 1H), 8.43 (t, *J* = 1.6 Hz, 1H), 8.32 (s, 1H, NH-*H*), 7.91 (d, *J* = 8.6 Hz, 2H), 7.68 (s, 1H, NH-*H*), 7.60 (d, *J* = 8.6 Hz, 2H), 4.71 (s, 2H). ^13^C NMR (151 MHz, DMSO-*d*_6_) δ 166.03, 165.19, 163.77, 148.32, 140.53, 137.31, 136.48, 135.30, 130.09, 128.79, 126.89, 122.36, 121.88, 35.20. Elem. Anal. Calcd for C_16_H_11_ClN_4_O_4_S: C, 49.18; H, 2.84; N, 14.34; S, 8.2. Found: C, 48.79; H, 2.63; N, 14.24; S, 8.0.

##### 2-(4-Bromophenyl)-5-((3-(carbamoyl)-5-nitrobenzyl)sulfanyl)-1,3,4-oxadiazole (**63d**)

Yield: 77% as a white solid; mp 222–223 °C. ^1^H NMR (600 MHz, DMSO-*d*_6_) δ 8.59 (t, *J* = 1.9 Hz, 1H), 8.53 (t, *J* = 2.0 Hz, 1H), 8.43 (t, *J* = 1.6 Hz, 1H), 8.32 (s, 1H, NH-*H*), 7.83 (d, *J* = 8.6 Hz, 2H), 7.74 (d, *J* = 8.6 Hz, 2H), 7.69 (s, 1H, NH-*H*), 4.71 (s, 2H). ^13^C NMR (151 MHz, DMSO-*d*_6_) δ 166.02, 165.30, 163.79, 148.33, 140.53, 136.49, 135.31, 133.01, 128.90, 126.89, 126.20, 122.70, 121.88, 35.20. Elem. Anal. Calcd for C_16_H_11_BrN_4_O_4_S: C, 44.15; H, 2.55; N, 12.87; S, 7.37. Found: C, 43.77; H, 2.57; N, 12.74; S, 7.18.

##### 2-((3-(Carbamoyl)-5-nitrobenzyl)sulfanyl)-5-cyclohexyl-1,3,4-oxadiazole (**63e**)

Yield: 93% as a white solid; mp 119–120 °C. ^1^H NMR (600 MHz, DMSO-*d*_6_) δ 8.58 (t, *J* = 2.0 Hz, 1H), 8.44 (t, *J* = 1.9 Hz, 1H), 8.35 (t, *J* = 1.9 Hz, 1H), 8.30 (s, 1H, NH-*H*), 7.68 (s, 1H, NH-*H*), 4.60 (s, 2H), 2.85 (tt, *J* = 11.0, 3.7 Hz, 1H), 1.93–1.82 (m, 2H), 1.67–1.62 (m, 2H), 1.60–1.54 (m, 1H), 1.45–1.35 (m, 2H), 1.34–1.24 (m, 2H), 1.21–1.13 (m, 1H). ^13^C NMR (151 MHz, DMSO-*d*_6_) δ 171.50, 165.96, 162.43, 148.26, 140.62, 136.49, 135.24, 126.79, 121.80, 35.20, 34.68, 29.87, 25.59, 25.14. Elem. Anal. Calcd for C_16_H_18_N_4_O_4_S: C, 53.03; H, 5.01; N, 15.46; S, 8.85. Found: C, 52.99; H, 5.14; N, 15.09; S, 8.59.

#### 2-Alkyl/Aryl-5-((3-(*N*-benzylcarbamoyl)-5-nitrobenzyl)sulfanyl)-1,3,4-oxadiazoles **64a**–**64e**

*N*-Benzyl-3-(bromomethyl)-5-nitrobenzamide (**42**) was used as the alkylating agent. The reactions were completed in 1 h. The final products **64a**–**64e** had low solubility. Therefore, upon reaction completion, the solvent was evaporated, and the residue was washed with 5% Na_2_CO_3_ (2 × 15 mL), water (2 × 20 mL, and EtOAc (7 mL) to give the final product.

##### 2-((3-(*N*-Benzylcarbamoyl)-5-nitrobenzyl)sulfanyl)-5-phenyl-1,3,4-oxadiazole (**64a**)

Yield: 81% as a white solid; mp 162–163 °C. ^1^H NMR (600 MHz, DMSO-*d*_6_) δ 9.41 (t, *J* = 5.9 Hz, 1H), 8.64 (t, *J* = 1.9 Hz, 1H), 8.55 (t, *J* = 1.9 Hz, 1H), 8.47 (t, *J* = 1.6 Hz, 1H), 7.93–7.86 (m, 2H), 7.60–7.53 (m, 1H), 7.54–7.47 (m, 2H), 7.32–7.25 (m, 4H), 7.25–7.18 (m, 1H), 4.73 (s, 2H), 4.47 (d, *J* = 5.8 Hz, 2H). ^13^C NMR (151 MHz, DMSO-*d*_6_) δ 165.94, 164.36, 163.48, 148.31, 140.71, 139.62, 136.38, 135.20, 132.60, 129.93, 128.88, 127.96, 127.46, 126.96, 126.90, 123.46, 121.66, 43.48, 35.21. Elem. Anal. Calcd for C_23_H_18_N_4_O_4_S: C, 61.87; H, 4.06; N, 12.55; S, 7.18. Found: C, 62.09; H, 4.05; N, 12.69; S, 7.51.

##### 2-((3-(*N*-Benzylcarbamoyl)-5-nitrobenzyl)sulfanyl)-5-(4-methoxyphenyl)-1,3,4-oxadiazole (**64b**)

Yield: 60% as a white solid; mp 169–170 °C. ^1^H NMR (500 MHz, DMSO-*d*_6_) δ 9.44 (t, *J* = 5.9 Hz, 1H), 8.67 (t, *J* = 1.7 Hz, 1H), 8.56 (t, *J* = 2.3 Hz, 1H), 8.49 (t, *J* = 2.0 Hz, 1H), 7.87 (d, *J* = 8.9 Hz, 2H), 7.34–7.30 (m, 4H), 7.28–7.21 (m, 1H), 7.09 (d, *J* = 8.8 Hz, 2H), 4.74 (s, 2H), 4.50 (d, *J* = 5.8 Hz, 2H), 3.83 (s, 3H). ^13^C NMR (126 MHz, DMSO-*d*_6_) δ 165.53, 163.98, 162.24, 147.91, 140.35, 139.24, 135.99, 134.80, 128.50, 128.47, 127.55, 127.07, 126.49, 121.26, 115.43, 115.00, 55.69, 43.09, 34.86. Elem. Anal. Calcd for C_24_H_20_N_4_O_5_S: C, 60.50; H, 4.23; N, 11.76; S, 6.73. Found: C, 60.12; H, 4.22; N, 11.44; S, 6.85.

##### 2-((3-(*N*-Benzylcarbamoyl)-5-nitrobenzyl)sulfanyl)-5-(4-chlorophenyl)-1,3,4-oxadiazole (**64c**)

Yield: 81% as a white solid; mp 165–166 °C. ^1^H NMR (600 MHz, DMSO-*d*_6_) δ 9.40 (t, *J* = 5.9 Hz, 1H), 8.64 (t, *J* = 1.9 Hz, 1H), 8.54 (t, *J* = 1.9 Hz, 1H), 8.47 (t, *J* = 1.6 Hz, 1H), 7.90 (d, *J* = 8.6 Hz, 2H), 7.58 (d, *J* = 8.6 Hz, 2H), 7.31–7.24 (m, 4H), 7.27–7.16 (m, 1H), 4.73 (s, 2H), 4.47 (d, *J* = 5.8 Hz, 2H). ^13^C NMR (151 MHz, DMSO-*d*_6_) δ 165.20, 164.36, 163.77, 148.31, 140.64, 139.62, 137.31, 136.36, 135.21, 130.08, 128.88, 128.77, 127.94, 127.46, 126.90, 122.36, 121.66, 43.48, 35.20. Elem. Anal. Calcd for C_23_H_17_ClN_4_O_4_S: C, 57.44; H, 3.56; N, 11.65; S, 6.67. Found: C, 57.09; H, 3.49; N, 11.64; S, 6.86.

##### 2-((3-(*N*-Benzylcarbamoyl)-5-nitrobenzyl)sulfanyl)-5-(4-bromophenyl)-1,3,4-oxadiazole (**64d**)

Yield: 83% as a white solid; mp 185–186 °C. ^1^H NMR (600 MHz, DMSO-*d*_6_) δ 9.40 (t, *J* = 5.9 Hz, 1H), 8.64 (t, *J* = 1.9 Hz, 1H), 8.54 (t, *J* = 1.9 Hz, 1H), 8.46 (t, *J* = 1.6 Hz, 1H), 7.83 (d, *J* = 8.6 Hz, 2H), 7.72 (d, *J* = 8.6 Hz, 2H), 7.31–7.25 (m, 4H), 7.25–7.18 (m, 1H), 4.73 (s, 2H), 4.47 (d, *J* = 5.9 Hz, 2H). ^13^C NMR (151 MHz, DMSO-*d*_6_) δ 165.31, 164.36, 163.79, 148.31, 140.64, 139.61, 136.36, 135.21, 133.00, 128.88, 127.94, 127.47, 126.91, 126.20, 122.70, 121.66, 43.48, 35.20. Elem. Anal. Calcd for C_23_H_17_BrN_4_O_4_S: C, 52.58; H, 3.26; N, 10.66; S, 6.10. Found: C, 52.21; H, 3.20; N, 10.57; S, 6.46.

##### 2-((3-(*N*-Benzylcarbamoyl)-5-nitrobenzyl)sulfanyl)-5-cyclohexyl-1,3,4-oxadiazole (**64e**)

Yield: 85% as a yellowish solid; mp 100–101 °C. ^1^H NMR (600 MHz, DMSO-*d*_6_) δ 9.40 (t, *J* = 5.9 Hz, 1H), 8.63 (t, *J* = 1.9 Hz, 1H), 8.46 (t, *J* = 2.0 Hz, 1H), 8.39 (t, *J* = 1.7 Hz, 1H), 7.31–7.29 (m, 4H), 7.25–7.18 (m, 1H), 4.61 (s, 2H), 4.47 (d, *J* = 5.8 Hz, 2H), 2.84 (tt, *J* = 10.9, 3.7 Hz, 1H), 1.90–1.83 (m, 2H), 1.68–1.51 (m, 3H), 1.44–1.35 (m, 2H), 1.34–1.21 (m, 2H), 1.21–1.13 (m, 1H). ^13^C NMR (151 MHz, DMSO-*d*_6_) δ 171.49, 164.31, 162.44, 148.26, 140.73, 139.63, 136.37, 135.13, 128.87, 127.96, 127.46, 126.81, 121.59, 43.47, 35.19, 34.67, 29.87, 25.59, 25.13. Elem. Anal. Calcd for C_23_H_24_N_4_O_4_S: C, 61.05; H, 5.35; N, 12.38; S, 7.08. Found: C, 60.80; H, 5.30; N, 12.40; S, 7.46.

#### 2-Alkyl/Aryl-5-((3-nitro-5-(1*H*-pyrrol-1-yl)benzyl)sulfanyl)-1,3,4-oxadiazoles **65a**–**65e**

3-Nitro-5-(1*H*-pyrrol-1-yl)benzyl chloride (**43**) was used as the alkylating agent. The reactions were stirred overnight.

##### 2-((3-Nitro-5-(1*H*-pyrrol-1-yl)benzyl)sulfanyl)-5-phenyl-1,3,4-oxadiazole (**65a**)

Yield: 61% as a yellow solid; mp 110–111 °C. ^1^H NMR (600 MHz, DMSO-*d*_6_) δ 8.25–8.23 (m, 1H), 8.23–8.21 (m, 2H), 7.91–7.86 (m, 2H), 7.60–7.54 (m, 1H), 7.5–7.49 (m, 2H), 7.48 (t, *J* = 2.2 Hz, 2H), 6.30 (t, *J* = 2.2 Hz, 2H), 4.69 (s, 2H). ^13^C NMR (151 MHz, DMSO-*d*_6_) δ 165.99, 163.48, 149.38, 141.66, 141.06, 132.61, 129.90, 126.94, 126.49, 123.47, 120.58, 119.85, 113.38, 112.17, 35.37. Elem. Anal. Calcd for C_19_H_14_N_4_O_3_S: C, 60.31; H, 3.73; N, 14.81; S, 8.47. Found: C, 60.06; H, 3.59; N, 14.82; S, 8.74.

##### 2-(4-Methoxyphenyl)-5-((3-nitro-5-(1*H*-pyrrol-1-yl)benzyl)sulfanyl)-1,3,4-oxadiazole (**65b**)

Yield: 65% as a yellow solid; mp 124–125 °C. ^1^H NMR (600 MHz, DMSO-*d*_6_) δ 8.24 (t, *J* = 2.2 Hz, 1H), 8.22–8.19 (m, 2H), 7.82 (d, *J* = 8.9 Hz, 2H), 7.48 (t, *J* = 2.2 Hz, 2H), 7.03 (d, *J* = 8.9 Hz, 2H), 6.30 (t, *J* = 2.2 Hz, 2H), 4.67 (s, 2H), 3.79 (s, 3H). ^13^C NMR (151 MHz, DMSO-*d*_6_) δ 165.94, 162.62, 149.38, 141.71, 141.04, 128.82, 126.49, 120.55, 119.85, 115.80, 115.35, 113.35, 112.17, 56.06, 35.40. Elem. Anal. Calcd for C_20_H_16_N_4_O_4_S: C, 58.82; H, 3.95; N, 13.72; S, 7.85. Found: C, 58.66; H, 4.06; N, 13.36; S, 7.73.

##### 2-(4-Chlorophenyl)-5-((3-nitro-5-(1*H*-pyrrol-1-yl)benzyl)sulfanyl)-1,3,4-oxadiazole (**65c**)

Yield: 58% as a brownish solid; mp 155–157 °C. ^1^H NMR (600 MHz, DMSO-*d*_6_) δ 8.24 (t, *J* = 2.1 Hz, 1H), 8.23–8.19 (m, 2H), 7.90 (d, *J* = 8.4 Hz, 2H), 7.58 (d, *J* = 8.2 Hz, 2H), 7.48 (t, *J* = 2.2 Hz, 2H), 6.30 (s, 2H), 4.69 (s, 2H). ^13^C NMR (151 MHz, DMSO-*d*_6_) δ 165.25, 163.77, 149.38, 141.59, 141.05, 137.32, 130.07, 128.76, 126.50, 122.37, 120.58, 119.86, 113.39, 112.17, 35.37. Elem. Anal. Calcd for C_19_H_13_ClN_4_O_3_S: C, 55.28; H, 3.17; N, 13.57; S, 7.77. Found: C, 55.03; H, 3.14; N, 13.23; S, 7.55.

##### 2-(4-Bromophenyl)-5-((3-nitro-5-(1*H*-pyrrol-1-yl)benzyl)sulfanyl)-1,3,4-oxadiazole (**65d**)

Yield: 61% as a yellow solid; 156–158 °C. ^1^H NMR (500 MHz, DMSO-*d*_6_) δ 8.24 (t, *J* = 2.1 Hz, 1H), 8.26–8.23 (m, 2H), 7.85 (d, *J* = 8.5 Hz, 2H), 7.75 (d, *J* = 8.4 Hz, 2H), 7.51 (t, *J* = 2.2 Hz, 2H), 6.33 (t, *J* = 2.2 Hz, 2H), 4.72 (s, 2H). ^13^C NMR (126 MHz, DMSO-*d*_6_) δ 164.96, 163.41, 148.99, 141.19, 140.66, 132.60, 128.47, 126.11, 125.82, 122.32, 120.19, 119.47, 113.00, 111.78, 34.97. Elem. Anal. Calcd for C_19_H_13_BrN_4_O_3_S: C, 49.90; H, 2.87; N, 12.25; S, 7.01. Found: C, 50.06; H, 2.66; N, 12.15; S, 7.11.

##### 2-Cyclohexyl-5-((3-nitro-5-(1*H*-pyrrol-1-yl)benzyl)sulfanyl)-1,3,4-oxadiazole (**65e**)

Yield: 53% as a yellow solid; mp 82–84 °C. ^1^H NMR (600 MHz, DMSO-*d*_6_) δ 8.24 (t, *J* = 2.1 Hz, 1H), 8.15–8.11 (m, 2H), 7.48 (t, *J* = 2.2 Hz, 2H), 6.30 (t, *J* = 2.3 Hz, 2H), 4.58 (s, 2H), 2.84 (tt, *J* = 10.9, 3.7 Hz, 1H), 1.89–1.81 (m, 2H), 1.65–1.53 (m, 3H), 1.41–1.34 (m, 2H), 1.30–1.22 (m, 2H), 1.18–1.09 (m, 1H). ^13^C NMR (151 MHz, DMSO-*d*_6_) δ 171.56, 162.45, 149.34, 141.70, 141.05, 126.44, 120.48, 119.84, 113.31, 112.16, 35.37, 34.68, 29.86, 25.58, 25.11. Elem. Anal. Calcd for C_19_H_20_N_4_O_3_S: C, 59.36; H, 5.24; N, 14.57; S, 8.34. Found: C, 58.99; H, 5.48; N, 14.18; S, 8.10.

#### 1-Alkyl/Aryl-5-((3,4-dinitrobenzyl)sulfanyl)-1*H*-tetrazoles **66a**–**66e**

3,4-Dinitrobenzyl bromide (**44**) was used as the alkylating agent. The reactions were completed in 1 h.

##### 5-((3,4-Dinitrobenzyl)sulfanyl)-1-phenyl-1*H*-tetrazole (**66a**)

Yield: 72% as a yellow solid; mp 125–126 °C. ^1^H NMR (600 MHz, DMSO-*d*_6_) δ 8.30 (d, *J* = 1.7 Hz, 1H), 8.16 (d, *J* = 8.3 Hz, 1H), 8.01 (dd, *J* = 8.4, 1.8 Hz, 1H), 7.67–7.53 (m, 5H), 4.72 (s, 2H). ^13^C NMR (151 MHz, DMSO-*d*_6_) δ 154.00, 145.48, 142.46, 141.44, 135.36, 133.43, 131.27, 130.54, 126.49, 126.36, 125.14, 35.43. Elem. Anal. Calcd for C_14_H_10_N_6_O_4_S: C, 46.93; H, 2.81; N, 23.45; S, 8.95. Found: C, 46.90; H, 2.69; N, 23.17; S, 9.14.

##### 5-((3,4-Dinitrobenzyl)sulfanyl)-1-(4-methoxyphenyl)-1*H*-tetrazole (**66b**)

Yield: 78% as a yellow solid; mp 117–118 °C. ^1^H NMR (600 MHz, DMSO-*d*_6_) δ 8.28 (d, *J* = 2.0 Hz, 1H), 8.16 (d, *J* = 8.3 Hz, 1H), 8.00 (dd, *J* = 8.3, 2.0 Hz, 1H), 7.49 (d, *J* = 9.0 Hz, 2H), 7.12 (d, *J* = 9.0 Hz, 2H), 4.68 (s, 2H), 3.81 (s, 3H). ^13^C NMR (151 MHz, DMSO-*d*_6_) δ 161.22, 154.11, 145.56, 142.46, 141.42, 135.32, 126.95, 126.45, 126.36, 126.00, 115.54, 56.24, 35.38. Elem. Anal. Calcd for C_15_H_12_N_6_O_5_S: C, 46.39; H, 3.11; N, 21.64; S, 8.26. Found: C, 46.03; H, 2.98; N, 21.36; S, 8.29.

##### 1-(4-Chlorophenyl)-5-((3,4-dinitrobenzyl)sulfanyl)-1*H*-tetrazole (**66c**)

Yield: 83% as a yellow solid; mp 157–158 °C. ^1^H NMR (600 MHz, DMSO-*d*_6_) δ 8.29 (d, *J* = 1.9 Hz, 1H), 8.16 (d, *J* = 8.3 Hz, 1H), 8.00 (dd, *J* = 8.4, 1.9 Hz, 1H), 7.69 (d, *J* = 8.8 Hz, 2H), 7.64 (d, *J* = 8.8 Hz, 2H), 4.71 (s, 2H). ^13^C NMR (151 MHz, DMSO-*d*_6_) δ 154.12, 145.44, 142.46, 141.44, 135.90, 135.36, 132.26, 130.57, 127.09, 126.48, 126.35, 35.55. Elem. Anal. Calcd for C_14_H_9_ClN_6_O_4_S: C, 42.81; H, 2.31; N, 21.40; S, 8.16. Found: C, 43.18; H, 2.19; N, 21.27; S, 8.29.

##### 1-(4-Bromophenyl)-5-((3,4-dinitrobenzyl)sulfanyl)-1*H*-tetrazole (**66d**)

Yield: 88% as a yellow solid; mp 146–147 °C. ^1^H NMR (600 MHz, DMSO-*d*_6_) δ 8.29 (d, *J* = 1.9 Hz, 1H), 8.16 (d, *J* = 8.4 Hz, 1H), 8.00 (dd, *J* = 8.4, 1.8 Hz, 1H), 7.82 (d, *J* = 8.7 Hz, 2H), 7.57 (d, *J* = 8.8 Hz, 2H), 4.71 (s, 2H). ^13^C NMR (151 MHz, DMSO-*d*_6_) δ 154.07, 145.43, 142.46, 141.44, 135.36, 133.52, 132.68, 127.22, 126.48, 126.35, 124.47, 35.56. Elem. Anal. Calcd for C_14_H_9_BrN_6_O_4_S: C, 38.46; H, 2.07; N, 19.22; S, 7.33. Found: 38.82; H, 1.91; N, 19.33; S, 7.38.

##### 1-Cyclohexyl-5-((3,4-dinitrobenzyl)sulfanyl)-1*H*-tetrazole (**66e**)

Yield: 63% as a yellow solid; mp 117–119 °C. ^1^H NMR (600 MHz, DMSO-*d*_6_) δ 8.29 (d, *J* = 1.9 Hz, 1H), 8.17 (d, *J* = 8.3 Hz, 1H), 7.97 (dd, *J* = 8.3, 1.9 Hz, 1H), 4.69 (s, 2H), 4.21 (tt, *J* = 11.6, 3.9 Hz, 1H), 1.89–1.80 (m, 2H), 1.80–1.72 (m, 2H), 1.72–1.64 (m, 2H), 1.63–1.58 (m, 1H), 1.41–1.29 (m, 2H), 1.24–1.13 (m, 1H). ^13^C NMR (151 MHz, DMSO-*d*_6_) δ 151.99, 145.73, 142.51, 141.39, 135.26, 126.43, 58.04, 35.49, 32.16, 24.96, 24.90. Elem. Anal. Calcd for C_14_H_16_N_6_O_4_S: C, 46.15; H, 4.43; N, 23.06; S, 8.80. Found: C, 46.53; H, 4.35; N, 23.35; S, 9.16.

#### 1-Alkyl/Aryl-5-((2,5-dinitrobenzyl)sulfanyl)-1*H*-tetrazoles **67a**–**67e**

2,5-Dinitrobenzyl bromide (**45**) was used as the alkylating agent. The reactions were completed in 1 h.

##### 5-((2,5-Dinitrobenzyl)sulfanyl)-1-phenyl-1*H*-tetrazole (**67a**)

Yield: 75% as a yellow solid; mp 120–121 °C. ^1^H NMR (500 MHz, DMSO-*d*_6_) δ 8.68 (d, *J* = 2.5 Hz, 1H), 8.34 (dd, *J* = 8.9, 2.6 Hz, 1H), 8.27 (d, *J* = 9.0 Hz, 1H), 7.67–7.56 (m, 5H), 4.93 (s, 2H). ^13^C NMR (126 MHz, DMSO-*d*_6_) δ 153.97, 151.84, 149.76, 134.49, 133.32, 131.17, 130.46, 128.08, 127.28, 125.05, 124.86, 33.69. Elem. Anal. Calcd for C_14_H_10_N_6_O_4_S: C, 46.93; H, 2.81; N, 23.45; S, 8.95. Found: C, 46.70; H, 2.7; N, 23.34; S, 9.25.

##### 5-(2,5-Dinitrobenzyl)sulfanyl)-1-(4-methoxyphenyl)-1*H*-tetrazole (**67b**)

Yield: 85% as a yellow solid; mp 124–125 °C. ^1^H NMR (500 MHz, DMSO-*d*_6_) δ 8.67 (d, *J* = 2.5 Hz, 1H), 8.34 (dd, *J* = 8.9, 2.6 Hz, 1H), 8.27 (d, *J* = 8.9 Hz, 1H), 7.50 (d, *J* = 9.0 Hz, 2H), 7.13 (d, *J* = 9.0 Hz, 2H), 4.90 (s, 2H), 3.83 (s, 3H). ^13^C NMR (126 MHz, DMSO-*d*_6_) δ 161.11, 154.09, 151.81, 149.75, 134.56, 128.03, 127.28, 126.85, 125.88, 124.84, 115.47, 56.15, 33.62. Elem. Anal. Calcd for C_15_H_12_N_6_O_5_S: C, 46.39; H, 3.11; N, 21.64; S, 8.26. Found: C, 46.43; H, 3.03; N, 21.60; S, 8.35.

##### 1-(4-Chlorophenyl)-5-((2,5-dinitrobenzyl)sulfanyl)-1*H*-tetrazole (**67c**)

Yield: 75% as a yellow solid; mp 144–145 °C. ^1^H NMR (500 MHz, DMSO-*d*_6_) δ 8.66 (d, *J* = 2.5 Hz, 1H), 8.35 (dd, *J* = 8.9, 2.5 Hz, 1H), 8.27 (d, *J* = 9.0 Hz, 1H), 7.71 (d, *J* = 8.8 Hz, 2H), 7.65 (d, *J* = 8.7 Hz, 2H), 4.91 (s, 2H). ^13^C NMR (126 MHz, DMSO-*d*_6_) δ 153.77, 151.52, 149.45, 135.50, 134.17, 131.85, 130.19, 127.78, 126.99, 126.71, 124.56, 33.55. Elem. Anal. Calcd for C_14_H_9_ClN_6_O_4_S: C, 42.81; H, 2.31; N, 21.40; S, 8.16. Found: C, 42.88; H, 2.11; N, 21.46; S, 8.25.

##### 1-(4-Bromophenyl)-5-((2,5-dinitrobenzyl)sulfanyl)-1*H*-tetrazole (**67d**)

Yield: 80% as a yellow solid; mp 150–151 °C. ^1^H NMR (600 MHz, DMSO-*d*_6_) δ 8.63 (d, *J* = 2.5 Hz, 1H), 8.31 (dd, *J* = 8.7, 2.5 Hz, 1H), 8.23 (d, *J* = 8.7 Hz, 1H), 7.80 (d, *J* = 8.7 Hz, 2H), 7.54 (d, *J* = 8.7 Hz, 2H), 4.87 (s, 2H). ^13^C NMR (151 MHz, DMSO-*d*_6_) δ 154.10, 151.90, 149.83, 134.55, 133.52, 132.66, 128.17, 127.37, 127.24, 124.94, 124.46, 33.93. Elem. Anal. Calcd for C_14_H_9_BrN_6_O_4_S: C, 38.46; H, 2.07; N, 19.22; S, 7.33. Found: C, 38.33; H, 1.91; N, 19.18; S, 7.19.

##### 1-Cyclohexyl-5-((2,5-dinitrobenzyl)sulfanyl)-1*H*-tetrazole (**67e**)

Yield: 65% as yellowish solid; mp 98–99 °C. ^1^H NMR (600 MHz, DMSO-*d*_6_) δ 8.61 (d, *J* = 2.5 Hz, 1H), 8.32 (dd, *J* = 8.9, 2.5 Hz, 1H), 8.26 (d, *J* = 8.9 Hz, 1H), 4.86 (s, 2H), 4.22 (tt, *J* = 11.5, 3.9 Hz, 1H), 1.88–1.86 (m, 2H), 1.77–1.74 (m, 2H), 1.72–1.66 (m, 2H), 1.62–1.59 (m, 1H), 1.39–1.31 (m, 2H), 1.22–1.18 (m, 1H). ^13^C NMR (151 MHz, DMSO-*d*_6_) δ 152.11, 151.93, 149.80, 134.73, 128.05, 127.42, 124.97, 58.03, 33.77, 32.19, 24.97, 24.89. Elem. Anal. Calcd for C_14_H_16_N_6_O_4_S: C, 46.15; H, 4.43; N, 23.06; S, 8.80. Found: C, 46.33; H, 4.40; N, 23.07; S, 8.90.

#### 2-Alkyl/Aryl-5-((3,4-dinitrobenzyl)sulfanyl)-1,3,4-oxadiazoles **68a**–**68e**

3,4-Dinitrobenzyl bromide (**44**) was used as the alkylating agent. The reactions were completed in 1 h.

##### 2-((3,4-Dinitrobenzyl)sulfanyl)-5-phenyl-1,3,4-oxadiazole (**68a**)

Yield: 71% as a yellow solid; mp 84–85 °C. ^1^H NMR (600 MHz, DMSO-*d*_6_) δ 8.35 (d, *J* = 1.7 Hz, 1H), 8.20 (d, *J* = 8.3 Hz, 1H), 8.05 (dd, *J* = 8.3, 1.9 Hz, 1H), 7.92–7.87 (m, 2H), 7.61–7.51 (m, 3H), 4.71 (s, 2H). ^13^C NMR (151 MHz, DMSO-*d*_6_) δ 166.05, 163.19, 145.85, 142.51, 141.45, 135.31, 132.64, 129.95, 126.97, 126.50, 126.46, 123.47, 34.78. Elem. Anal. Calcd for C_15_H_10_N_4_O_5_S: C, 50.28; H, 2.81; N, 15.64; S, 8.95. Found: C, 50.65; H, 2.66; N, 15.69; S, 9.24.

##### 2-((3,4-Dinitrobenzyl)sulfanyl)-5-(4-methoxyphenyl)-1,3,4-oxadiazole (**68b**)

Yield: 62% as a yellow solid; mp 109–110 °C. ^1^H NMR (600 MHz, DMSO-*d*_6_) δ 8.34 (d, *J* = 1.7 Hz, 1H), 8.19 (d, *J* = 8.3 Hz, 1H), 8.04 (dd, *J* = 8.4, 1.8 Hz, 1H), 7.83 (d, *J* = 8.9 Hz, 2H), 7.08 (d, *J* = 9.0 Hz, 2H), 4.69 (s, 2H), 3.80 (s, 3H). ^13^C NMR (151 MHz, DMSO-*d*_6_) δ 166.01, 162.66, 162.34, 145.90, 142.51, 141.42, 135.28, 128.86, 126.49, 126.43, 115.80, 115.39, 56.08, 34.80. Elem. Anal. Calcd for C_16_H_12_N_4_O_6_S: C, 49.48; H, 3.11; N, 14.43; S, 8.26. Found: C, 49.87; H, 3.10; N, 14.45; S, 8.36.

##### 2-(4-Chlorophenyl)-5-((3,4-dinitrobenzyl)sulfanyl)-1,3,4-oxadiazole (**68c**)

Yield: 61% as a white solid; mp 115–116 °C. ^1^H NMR (600 MHz, DMSO-*d*_6_) δ 8.35 (d, *J* = 1.9 Hz, 1H), 8.19 (d, *J* = 8.3 Hz, 1H), 8.05 (dd, *J* = 8.4, 1.8 Hz, 1H), 7.91 (d, *J* = 8.7 Hz, 2H), 7.61 (d, *J* = 8.6 Hz, 2H), 4.71 (s, 2H). ^13^C NMR (151 MHz, DMSO-*d*_6_) δ 165.30, 163.49, 145.77, 142.50, 141.45, 137.35, 135.32, 130.11, 128.78, 126.49, 126.46, 122.38, 34.75. Elem. Anal. Calcd for C_15_H_9_ClN_4_O_5_S: C, 45.87; H, 2.31; N, 14.26; S, 8.16. Found: C, 45.91; H, 2.13; N, 14.23; S, 8.23.

##### 2-(4-Bromophenyl)-5-((3,4-dinitrobenzyl)sulfanyl)-1,3,4-oxadiazole (**68d**)

Yield: 72% as a yellow solid; mp 145–146 °C. ^1^H NMR (600 MHz, DMSO-*d*_6_) δ 8.34 (d, *J* = 1.9 Hz, 1H), 8.19 (d, *J* = 8.3 Hz, 1H), 8.05 (dd, *J* = 8.4, 1.9 Hz, 1H), 7.83 (d, *J* = 8.6 Hz, 2H), 7.75 (d, *J* = 8.6 Hz, 2H), 4.71 (s, 2H). ^13^C NMR (151 MHz, DMSO-*d*_6_) δ 165.41, 163.51, 145.76, 142.50, 141.45, 135.32, 133.03, 128.89, 126.49, 126.46, 126.24, 122.71, 34.75. Elem. Anal. Calcd for C_15_H_9_BrN_4_O_5_S: C, 41.21; H, 2.07; N, 12.81; S, 7.33. Found: C, 41.44; H, 1.89; N, 12.74; S, 7.34.

##### 2-Cyclohexyl-5-((3,4-dinitrobenzyl)sulfanyl)-1,3,4-oxadiazole (**68e**)

Yield: 66% as a yellowish solid; mp 114–115 °C. ^1^H NMR (600 MHz, DMSO-*d*_6_) δ 8.28 (d, *J* = 1.9 Hz, 1H), 8.18 (d, *J* = 8.3 Hz, 1H), 7.99 (dd, *J* = 8.3, 1.9 Hz, 1H), 4.60 (s, 2H), 2.86 (tt, *J* = 10.9, 3.7 Hz, 1H), 1.91–1.85 (m, 2H), 1.68–1.62 (m, 2H), 1.61–1.54 (m, 1H), 1.45–1.35 (m, 2H), 1.34–1.25 (m, 2H), 1.22–1.14 (m, 1H). ^13^C NMR (151 MHz, DMSO-*d*_6_) δ 171.58, 162.23, 145.90, 142.45, 141.42, 135.29, 126.47, 126.39, 34.72, 34.66, 29.86, 25.60, 25.12. Elem. Anal. Calcd for C_15_H_16_N_4_O_5_S: C, 49.44; H, 4.43; N, 15.38; S, 8.80. Found: C, 49.80; H, 4.35; N, 15.42; S, 9.12.

#### 2-Alkyl/Aryl-5-((2,5-dinitrobenzyl)sulfanyl)-1,3,4-oxadiazoles **69a**–**69e**

2,5-Dinitrobenzyl bromide (**45**) was used as the alkylating agent. The reactions were completed in 1 h.

##### 2-((2,5-Dinitrobenzyl)sulfanyl)-5-phenyl-1,3,4-oxadiazole (**69a**)

Yield: 80% as a yellow solid; mp 141–142 °C. ^1^H NMR (600 MHz, DMSO-*d*_6_) δ 8.69 (d, *J* = 2.5 Hz, 1H), 8.34 (dd, *J* = 8.9, 2.5 Hz, 1H), 8.28 (d, *J* = 8.9 Hz, 1H), 7.94–7.87 (m, 2H), 7.62–7.57 (m, 1H), 7.56–7.51 (m, 2H), 4.88 (s, 2H). ^13^C NMR (151 MHz, DMSO-*d*_6_) δ 166.18, 163.15, 151.78, 149.89, 134.89, 132.67, 129.92, 128.10, 127.54, 127.01, 125.06, 123.47, 33.27. Elem. Anal. Calcd for C_15_H_10_N_4_O_5_S: C, 50.28; H, 2.81; N, 15.64; S, 8.95. Found: C, 49.90; H, 2.58; N, 15.61; S, 9.04.

##### 2-((2,5-Dinitrobenzyl)sulfanyl)-5-(4-methoxyphenyl)-1,3,4-oxadiazole (**69b**)

Yield: 68% as a yellow solid; mp 125–126 °C. ^1^H NMR (600 MHz, DMSO-*d*_6_) δ 8.67 (d, *J* = 2.6 Hz, 1H), 8.34 (dd, *J* = 8.9, 2.5 Hz, 1H), 8.27 (d, *J* = 8.9 Hz, 1H), 7.83 (d, *J* = 8.9 Hz, 2H), 7.07 (d, *J* = 8.9 Hz, 2H), 4.86 (s, 2H), 3.80 (s, 3H). ^13^C NMR (151 MHz, DMSO-*d*_6_) δ 166.15, 162.68, 162.30, 151.78, 149.87, 134.93, 128.89, 128.06, 127.51, 125.03, 115.79, 115.37, 56.07, 33.26. Elem. Anal. Calcd for C_16_H_12_N_4_O_6_S: C, 49.48; H, 3.11; N, 14.43; S, 8.26. Found: C, 49.09; H, 3.02; N, 14.31; S, 8.27.

##### 2-(4-Chlorophenyl)-5-((2,5-dinitrobenzyl)sulfanyl)-1,3,4-oxadiazole (**69c**)

Yield: 71% as a yellow solid; mp 121–122 °C. ^1^H NMR (500 MHz, DMSO-*d*_6_) δ 8.71 (d, *J* = 2.5 Hz, 1H), 8.37 (dd, *J* = 8.9, 2.5 Hz, 1H), 8.31 (d, *J* = 8.9 Hz, 1H), 7.88 (d, *J* = 8.6 Hz, 2H), 7.79 (d, *J* = 8.6 Hz, 2H), 4.92 (s, 2H). ^13^C NMR (126 MHz, DMSO-*d*_6_) δ 165.17, 163.10, 151.40, 149.52, 134.44, 132.65, 128.57, 127.73, 127.17, 125.92, 124.71, 122.33, 32.86. Elem. Anal. Calcd for C_15_H_9_ClN_4_O_5_S: C, 45.87; H, 2.31; N, 14.26; S, 8.16. Found: C, 45.77; H, 2.68; N, 14.32; S, 8.23.

##### 2-(4-Bromophenyl)-5-((2,5-dinitrobenzyl)sulfanyl)-1,3,4-oxadiazole (**69d**)

Yield: 88% as a yellow solid; mp 148–149 °C. ^1^H NMR (600 MHz, DMSO-*d*_6_) δ 8.68 (d, *J* = 2.5 Hz, 1H), 8.34 (dd, *J* = 8.8, 2.5 Hz, 1H), 8.28 (d, *J* = 9.0 Hz, 1H), 7.84 (d, *J* = 8.5 Hz, 2H), 7.75 (d, *J* = 8.5 Hz, 2H), 4.88 (s, 2H). ^13^C NMR (151 MHz, DMSO-*d*_6_) δ 165.54, 163.48, 151.77, 149.89, 134.80, 133.01, 128.92, 128.10, 127.53, 126.29, 125.07, 122.70, 33.24. Elem. Anal. Calcd for C_15_H_9_BrN_4_O_5_S: C, 41.21; H, 2.07; N, 12.81; S, 7.33. Found: C, 40.88; H, 1.88; N, 12.78; S, 7.34.

##### 2-Cyclohexyl-5-((2,5-dinitrobenzyl)sulfanyl)-1,3,4-oxadiazole (**69e**)

Yield: 67% as a yellow solid; mp 100–101 °C. ^1^H NMR (600 MHz, DMSO-*d*_6_) δ 8.59 (d, *J* = 2.6 Hz, 1H), 8.34 (dd, *J* = 8.9, 2.5 Hz, 1H), 8.27 (d, *J* = 9.0 Hz, 1H), 4.78 (s, 2H), 2.85 (tt, *J* = 11.0, 3.7 Hz, 1H), 1.92–1.85 (m, 2H), 1.70–1.53 (m, 3H), 1.45–1.36 (m, 2H), 1.35–1.25 (m, 2H), 1.23–1.16 (m, 1H). ^13^C NMR (151 MHz, DMSO-*d*_6_) δ 171.71, 162.23, 151.79, 149.82, 134.92, 128.03, 127.52, 125.01, 34.70, 33.11, 29.87, 25.61, 25.15. Elem. Anal. Calcd for C_15_H_16_N_4_O_5_S: C, 49.44; H, 4.43; N, 15.38; S, 8.80. Found: C, 49.07; H, 4.36; N, 15.42; S, 9.05.

#### 1-Alkyl/Aryl-5-((2-nitro-5-(trifluoromethyl)benzyl)sulfanyl)-1*H*-tetrazoles **70a**–**70e**

2-Nitro-5-(trifluoromethyl)benzyl bromide (**46**) was used as the alkylating agent. The reactions were completed in 1 h.

##### 5-((2-Nitro-5-(trifluoromethyl)benzyl)sulfanyl)-1-phenyl-1*H*-tetrazole (**70a**)

Yield: 90% as a beige solid; mp 100–101 °C. ^1^H NMR (600 MHz, DMSO-*d*_6_) δ 8.12 (d, *J* = 1.9 Hz, 1H), 8.08 (d, *J* = 8.3 Hz, 1H), 7.99 (dd, *J* = 8.2, 1.9 Hz, 1H), 7.64–7.55 (m, 5H), 4.71 (s, 2H). ^13^C NMR (151 MHz, DMSO-*d*_6_) δ 154.05, 146.90, 144.09, 135.38, 133.43, 131.23, 130.50, 129.32 (d, *J* = 5.1 Hz), 126.27, 125.12, 122.54 (d, *J* = 273.1 Hz), 121.90 (q, *J* = 33.6 Hz), 35.63. HRMS (ESI+) calcd for (C_15_H_10_F_3_N_5_O_2_S + H^+^) *m*/*z*: 382.05801 (100%), 383.06136 (16.2%); found: 382.0584 (100%), 383.0611 (17%).

##### 1-(4-Methoxyphenyl)-5-((2-nitro-5-(trifluoromethyl)benzyl)sulfanyl)-1*H*-tetrazole (**70b**)

Yield: 69% as a white solid; mp 100–101 °C. ^1^H NMR (600 MHz, DMSO-*d*_6_) δ 8.11 (d, *J* = 1.8 Hz, 1H), 8.08 (d, *J* = 8.6 Hz, 1H), 7.97 (dd, *J* = 8.4, 1.9 Hz, 1H), 7.47 (d, *J* = 9.0 Hz, 2H), 7.11 (d, *J* = 9.0 Hz, 2H), 4.68 (s, 2H), 3.80 (s, 3H). ^13^C NMR (151 MHz, DMSO-*d*_6_) δ 161.19, 154.15, 146.88, 144.17, 135.33, 129.29, 126.91, 126.26, 126.01, 122.55 (d, *J* = 273.1 Hz), 121.89 (d, *J* = 33.2 Hz), 115.51 (d, *J* = 15.9 Hz), 56.21, 35.59. HRMS (ESI+) calcd for (C_16_H_12_F_3_N_5_O_3_S + H^+^) *m*/*z*: 412.06857 (100%), 413.07193 (17.3%); found: 412.0688 (100%), 413.0716 (8%).

##### 1-(4-Chlorophenyl)-5-((2-nitro-5-(trifluoromethyl)benzyl)sulfanyl)-1*H*-tetrazole (**70c**)

Yield: 83% as a white solid; mp 127–128 °C. ^1^H NMR (600 MHz, DMSO-*d*_6_) δ 8.11 (d, *J* = 1.9 Hz, 1H), 8.08 (d, *J* = 8.3 Hz, 1H), 7.97 (dd, *J* = 8.4, 1.9 Hz, 1H), 7.68 (d, *J* = 8.8 Hz, 2H), 7.63 (d, *J* = 8.8 Hz, 2H), 4.70 (s, 2H). ^13^C NMR (151 MHz, DMSO-*d*_6_) δ 154.17, 146.90, 144.05, 135.87, 135.38, 132.26, 130.54, 129.33 (d, *J* = 5.4 Hz), 127.06, 126.26, 122.54 (d, *J* = 273.1 Hz), 121.89 (d, *J* = 33.6 Hz), 35.77. HRMS (ESI+) calcd for (C_15_H_9_ClF_3_N_5_O_2_S + H^+^) *m*/*z*: 416.01903 (100%), 418.01609 (32%); found: 416.0193 (100%), 418.0167 (35%).

##### 1-(4-Bromophenyl)-5-((2-nitro-5-(trifluoromethyl)benzyl)sulfanyl)-1*H*-tetrazole (**70d**)

Yield: 88% as a white solid; mp 118–119 °C. ^1^H NMR (500 MHz, DMSO-*d*_6_) δ 8.15 (d, *J* = 1.8 Hz, 1H), 8.12 (d, *J* = 8.3 Hz, 1H), 8.01 (dd, *J* = 8.3, 1.9 Hz, 1H), 7.84 (d, *J* = 8.8 Hz, 2H), 7.59 (d, *J* = 8.8 Hz, 2H), 4.73 (s, 2H). ^13^C NMR (126 MHz, DMSO-*d*_6_) δ 154.04, 146.81, 143.97, 135.30, 133.41, 132.60, 129.25 (q, *J* = 5.2 Hz), 127.13, 126.18, 124.35, 122.46 (q, *J* = 273.2 Hz), 121.81 (q, *J* = 33.4 Hz), 35.69. HRMS (ESI+) calcd for (C_15_H_9_BrF_3_N_5_O_2_S + H^+^) *m*/*z*: 459.96852 (100%), 461.96648 (97.3%); found: 461.9674 (100%), 459.9696 (97%).

##### 1-Cyclohexyl-5-((2-nitro-5-(trifluoromethyl)benzyl)sulfanyl)-1*H*-tetrazole (**70e**)

Yield: 93% as a white solid; mp 158–159 °C. ^1^H NMR (500 MHz, DMSO-*d*_6_) δ 8.15–8.10 (m, 2H), 7.97 (dd, *J* = 8.4, 1.9 Hz, 1H), 4.71 (s, 2H), 4.22 (tt, *J* = 11.5, 3.9 Hz, 1H), 1.90–1.58 (m, 7H), 1.36 (qt, *J* = 12.8, 3.4 Hz, 2H), 1.29–1.12 (m, 1H). ^13^C NMR (126 MHz, DMSO-*d*_6_) δ 151.89, 146.79, 144.27, 135.22, 129.09 (q, *J* = 5.2 Hz), 126.23, 122.44 (q, *J* = 273.0 Hz), 121.85 (q, *J* = 33.4 Hz), 57.92, 35.68, 32.07, 24.86, 24.82. HRMS (ESI+) calcd for (C_15_H_16_F_3_N_5_O_2_S + H^+^) *m*/*z*: 388.10496 (100%), 389.10831 (16.2%); found: 388.1054 (100%), 389.1080 (17%).

#### 1-Alkyl/Aryl-5-((5-nitro-2-(trifluoromethyl)benzyl)sulfanyl)-1*H*-tetrazoles **71a**–**71e**

5-Nitro-2-(trifluoromethyl)benzyl bromide (**47**) was used as the alkylating agent. The reactions were completed in 1 h.

##### 5-((5-Nitro-2-(trifluoromethyl)benzyl)sulfanyl)-1-phenyl-1*H*-tetrazole (**71a**)

Yield: 98% as a yellowish solid; mp 64–65 °C. ^1^H NMR (600 MHz, DMSO-*d*_6_) δ 8.58 (d, *J* = 2.4 Hz, 1H), 8.27 (dd, *J* = 8.6, 2.4, 1H), 8.01 (d, *J* = 8.6 Hz, 1H), 7.63–7.54 (m, 5H), 4.80 (d, *J* = 1.4 Hz, 2H). ^13^C NMR (151 MHz, DMSO-*d*_6_) δ 153.64, 150.40, 137.65, 133.42, 132.86 (d, *J* = 30.5 Hz), 131.24, 130.52, 129.04 (d, *J* = 5.6 Hz), 127.16, 125.18, 124.06, 123.74 (d, *J* = 274.5 Hz), 33.97. HRMS (ESI+) calcd for (C_15_H_10_F_3_N_5_O_2_S + H^+^) *m*/*z*: 382.05801 (100%), 383.06136 (16.2%); found: 382.0589 (100%), 383.0611 (17%).

##### 1-(4-Methoxyphenyl)-5-((5-nitro-2-(trifluoromethyl)benzyl)sulfanyl)-1*H*-tetrazole (**71b**)

Yield: 98% as a white solid; mp 107–108 °C. ^1^H NMR (600 MHz, DMSO-*d*_6_) δ 8.56 (d, *J* = 2.3 Hz, 1H), 8.27 (dd, *J* = 8.6, 2.4 Hz, 1H), 8.01 (d, *J* = 8.7 Hz, 1H), 7.47 (d, *J* = 8.8 Hz, 2H), 7.10 (d, *J* = 9.0 Hz, 2H), 4.77 (s, 2H), 3.79 (s, 3H). ^13^C NMR (151 MHz, DMSO-*d*_6_) δ 161.19, 153.75, 150.38, 137.76, 132.82 (d, *J* = 30.7 Hz), 129.04 (d, *J* = 5.8 Hz), 127.11, 126.94, 125.97, 124.03, 123.73 (d, *J* = 275.3 Hz), 115.52, 56.21, 33.88. HRMS (ESI+) calcd for (C_16_H_12_F_3_N_5_O_3_S + H^+^) *m*/*z*: 412.06857 (100%), 413.07193 (17.3%); found: 412.0688 (100%), 413.0717 (17%).

##### 1-(4-Chlorophenyl)-5-((5-nitro-2-(trifluoromethyl)benzyl)sulfanyl)-1*H*-tetrazole (**71c**)

Yield: 95% as a white solid; mp 144–145 °C. ^1^H NMR (500 MHz, DMSO-*d*_6_) δ 8.60 (d, *J* = 2.3 Hz, 1H), 8.31 (dd, *J* = 8.6, 1,5 Hz, 1H), 8.04 (d, *J* = 8.7 Hz, 1H), 7.70 (d, *J* = 8.9 Hz, 2H), 7.66 (d, *J* = 8.9 Hz, 2H), 4.82 (d, *J* = 1.3 Hz, 2H). ^13^C NMR (126 MHz, DMSO-*d*_6_) δ 153.38, 150.01, 137.29, 135.53, 132.45 (q, *J* = 30.6 Hz), 131.86, 130.17, 128.68 (q, *J* = 5.6 Hz), 126.81, 126.75, 123.68, 123.35 (q, *J* = 274.9 Hz), 33.75 (d, *J* = 2.3 Hz). HRMS (ESI+) calcd for (C_15_H_9_ClF_3_N_5_O_2_S + H^+^) *m*/*z*: 416.01903 (100%), 418.01609 (32%); found: 416.0202 (100%), 418.0172 (38%).

##### 1-(4-Bromophenyl)-5-((5-nitro-2-(trifluoromethyl)benzyl)sulfanyl)-1*H*-tetrazole (**71d**)

Yield: 90% as a white solid; mp 148–150 °C. ^1^H NMR (500 MHz, DMSO-*d*_6_) δ 8.60 (d, *J* = 2.4 Hz, 1H), 8.31 (dd, *J* = 8.6, 1.5 Hz, 1H), 8.04 (d, *J* = 8.7 Hz, 1H), 7.84 (d, *J* = 8.8 Hz, 2H), 7.58 (d, *J* = 8.9 Hz, 2H), 4.82 (d, *J* = 1.3 Hz, 2H). ^13^C NMR (126 MHz, DMSO-*d*_6_) δ 153.62, 150.30, 137.58, 133.42, 132.74 (d, *J* = 30.5 Hz), 132.57, 128.98 (d, *J* = 5.6 Hz), 127.20, 127.10, 124.39, 123.97, 123.64 (d, *J* = 275.1 Hz), 34.05. HRMS (ESI+) calcd for (C_15_H_9_BrF_3_N_5_O_2_S + H^+^) *m*/*z*: 459.96852 (100%), 461.96648 (97.3%); found: 461.9673 (100%), 459.9691 (97%).

##### 1-Cyclohexyl-5-((5-nitro-2-(trifluoromethyl)benzyl)sulfanyl)-1*H*-tetrazole (**71e**)

Yield: 93% as a white solid; mp 83–84 °C. ^1^H NMR (500 MHz, DMSO-*d*_6_) δ 8.59 (d, *J* = 2.4 Hz, 1H), 8.33 (dd, *J* = 8.7, 2.4 Hz, 1H), 8.07 (d, *J* = 8.7 Hz, 1H), 4.81 (d, *J* = 1.2 Hz, 2H), 4.29 (tt, *J* = 11.3, 3.9 Hz, 1H), 1.93–1.85 (m, 2H), 1.82–1.68 (m, 4H), 1.68–1.57 (m, 1H), 1.47–1.30 (m, 2H), 1.28–1.13 (m, 1H). ^13^C NMR (126 MHz, DMSO-*d*_6_) δ 151.62, 150.30, 137.97, 132.68 (d, *J* = 30.8 Hz), 129.07 (q, *J* = 5.5 Hz), 127.01, 123.96, 123.67 (d, *J* = 275.1 Hz), 58.01, 33.89 (d, *J* = 2.2 Hz), 32.15, 24.87, 24.83. HRMS (ESI+) calcd for (C_15_H_16_F_3_N_5_O_2_S + H^+^) *m*/*z*: 388.10496 (100%), 389.10831 (16.2%); found: 388.1058 (100%), 389.1084 (16%).

#### 2-Alkyl/Aryl-5-((2-nitro-5-(trifluoromethyl)benzyl)sulfanyl)-1,3,4-oxadiazoles **72a**–**72e**

2-Nitro-5-(trifluoromethyl)benzyl bromide (**46**) was used as the alkylating agent. The reactions were completed in 1 h.

##### 2-((2-Nitro-5-(trifluoromethyl)benzyl)sulfanyl)-5-phenyl-1,3,4-oxadiazole (**72a**)

Yield: 78% as a white solid; mp 91–92 °C. ^1^H NMR (500 MHz, DMSO-*d*_6_) δ 8.21 (d, *J* = 1.9 Hz, 1H), 8.16 (d, *J* = 8.4 Hz, 1H), 8.07 (dd, *J* = 8.4, 1.9 Hz, 1H), 7.95–7.88 (m, 2H), 7.65–7.53 (m, 3H), 4.74 (s, 2H). ^13^C NMR (126 MHz, DMSO-*d*_6_) δ 165.94, 163.19, 146.82, 144.43, 135.28, 132.55, 129.84, 129.15 (q, *J* = 5.2 Hz), 126.85, 126.33, 123.36, 122.47 (d, *J* = 273.2 Hz), 121.86 (q, *J* = 33.4 Hz), 34.85. HRMS (ESI+) calcd for (C_16_H_10_F_3_N_3_O_3_S + H^+^) *m*/*z*: 382.04677 (100%), 383.05013 (17.3%); found: 382.0478 (100%), 383.0502 (17%).

##### 2-(4-Methoxyphenyl)-5-((2-nitro-5-(trifluoromethyl)benzyl)sulfanyl)-1,3,4-oxadiazole (**72b**)

Yield: 79% as a white solid; mp 112–113 °C. ^1^H NMR (600 MHz, DMSO-*d*_6_) δ 8.15 (d, *J* = 1.8 Hz, 1H), 8.12 (d, *J* = 8.3 Hz, 1H), 8.02 (dd, *J* = 8.4, 1.9 Hz, 1H), 7.82 (d, *J* = 8.9 Hz, 2H), 7.07 (d, *J* = 8.9 Hz, 2H), 4.68 (s, 2H), 3.80 (s, 3H). ^13^C NMR (151 MHz, DMSO-*d*_6_) δ 165.99, 162.65, 162.41, 146.89, 144.53, 135.32, 129.20, 128.82, 126.39, 122.55 (d, *J* = 273.1 Hz), 121.95 (d, *J* = 33.4 Hz), 115.78, 115.65–115.07 (m), 56.07, 34.96. HRMS (ESI+) calcd for (C_17_H_12_F_3_N_3_O_4_S + H^+^) *m*/*z*: 412.05734 (100%), 413.0607 (18.4%); found: 412.0577 (100%), 413.0602 (19%).

##### 2-(4-Chlorophenyl)-5-((2-nitro-5-(trifluoromethyl)benzyl)sulfanyl)-2-phenyl-1,3,4-oxadiazole (**72c**)

Yield: 67% as a white solid; mp 156–157 °C. ^1^H NMR (500 MHz, DMSO-*d*_6_) δ 8.20 (d, *J* = 1.9 Hz, 1H), 8.15 (d, *J* = 8.3 Hz, 1H), 8.07 (dd, *J* = 8.3, 1.9 Hz, 1H), 7.93 (d, *J* = 8.7 Hz, 2H), 7.64 (d, *J* = 8.7 Hz, 2H), 4.74 (s, 2H). ^13^C NMR (126 MHz, DMSO-*d*_6_) δ 165.19, 163.49, 146.81, 144.34, 137.27, 135.29, 130.01, 129.16 (q, *J* = 5.2 Hz), 128.66, 126.31, 122.46 (q, *J* = 273.2 Hz), 122.27, 121.85 (d, *J* = 33.4 Hz), 34.82. HRMS (ESI+) calcd for (C_16_H_9_ClF_3_N_3_O_3_S + H^+^) *m*/*z*: 416.00780 (100%), 418.00485 (32%); found: 416.0085 (100%), 418.0052 (37%).

##### 2-(4-Bromophenyl)-5-((2-nitro-5-(trifluoromethyl)benzyl)sulfanyl)-2-phenyl-1,3,4-oxadiazole (**72d**)

Yield: 71% as a white solid; mp 153–154 °C. ^1^H NMR (500 MHz, DMSO-*d*_6_) δ 8.20 (d, *J* = 1.9 Hz, 1H), 8.15 (d, *J* = 8.3 Hz, 1H), 8.07 (dd, *J* = 8.4, 1.9 Hz, 1H), 7.86 (d, *J* = 8.6 Hz, 2H), 7.78 (d, *J* = 8.6 Hz, 2H), 4.74 (s, 2H). ^13^C NMR (126 MHz, DMSO-*d*_6_) δ 165.31, 163.52, 146.82, 144.33, 135.30, 132.94, 129.16 (q, *J* = 5.2 Hz), 128.77, 126.31, 126.16, 122.61, 122.47 (q, *J* = 272.8 Hz), 121.85 (d, *J* = 33.3 Hz), 34.82. HRMS (ESI+) calcd for (C_16_H_9_BrF_3_N_3_O_3_S + H^+^) *m*/*z*: 459.95729 (100%), 461.95524 (97.3%); found: 461.9561 (100%), 459.9578 (97%).

##### 2-Cyclohexyl-5-((2-nitro-5-(trifluoromethyl)benzyl)sulfanyl)-2-phenyl-1,3,4-oxadiazole (**72e**)

Yield: 69% as a white solid; mp 104–105 °C. ^1^H NMR (500 MHz, DMSO-*d*_6_) δ 8.14 (d, *J* = 8.3 Hz, 1H), 8.13 (d, *J* = 1.9 Hz, 1H), 8.01 (dd, *J* = 8.3, 1.9 Hz, 1H), 4.63 (s, 2H), 2.91–2.85 (m, 1H), 1.95–1.86 (m, 2H), 1.70–1.58 (m, 3H), 1.47–1.27 (m, 4H), 1.27–1.13 (m, 1H). ^13^C NMR (126 MHz, DMSO-*d*_6_) δ 171.47, 162.23, 146.79, 144.46, 135.23, 129.04 (q, *J* = 5.1 Hz), 126.28, 122.46 (q, *J* = 273.2 Hz), 121.81 (q, *J* = 33.3 Hz), 34.81, 34.57, 29.77, 25.51, 25.03. HRMS (ESI+) calcd for (C_16_H_16_F_3_N_3_O_3_S + H^+^) *m*/*z*: 388.09372 (100%), 389.09708 (17.3%); found: 388.0943 (100%), 389.0969 (17%).

#### 2-Alkyl/Aryl-5-((5-nitro-2-(trifluoromethyl)benzyl)sulfanyl)-1,3,4-oxadizaoles **73a**–**73e**

5-Nitro-2-(trifluoromethyl)benzyl bromide (**47**) was used as the alkylating agent. The reactions were completed in 1 h.

##### 2-((5-Nitro-2-(trifluoromethyl)benzyl)sulfanyl)-5-phenyl-1,3,4-oxadiazole (**73a**)

Yield: 92% as a white solid; mp 124–125 °C. ^1^H NMR (500 MHz, DMSO-*d*_6_) δ 8.67 (d, *J* = 2.3 Hz, 1H), 8.35–8.31 (m, 1H), 8.08 (d, *J* = 8.7 Hz, 1H), 7.97–7.91 (m, 2H), 7.65–7.53 (m, 3H), 4.83 (d, *J* = 1.4 Hz, 2H). ^13^C NMR (126 MHz, DMSO-*d*_6_) δ 166.17, 162.67, 150.32, 138.06, 132.79 (q, *J* = 30.5 Hz), 132.63, 129.87, 129.09 (q, *J* = 5.5 Hz), 127.11, 126.90, 124.01, 123.72 (q, *J* = 274.9 Hz), 123.34, 33.19 (d, *J* = 2.2 Hz). HRMS (ESI+) calcd for (C_16_H_10_F_3_N_3_O_3_S + H^+^) *m*/*z*: 382.04677 (100%), 383.05013 (17.3%); found: 382.0470 (100%), 383.0497 (18%).

##### 2-(4-Methoxyphenyl)-5-((5-nitro-2-(trifluoromethyl)benzyl)sulfanyl)-1,3,4-oxadiazole (**73b**)

Yield: 88% as a white solid; mp 134–135 °C. ^1^H NMR (600 MHz, DMSO-*d*_6_) δ 8.62 (d, *J* = 2.4 Hz, 1H), 8.30 (dd, *J* = 8.7, 2.3 Hz, 1H), 8.04 (d, *J* = 8.7 Hz, 1H), 7.83 (d, *J* = 8.9 Hz, 2H), 7.08 (d, *J* = 8.8 Hz, 2H), 4.77 (s, 2H), 3.81 (s, 3H). ^13^C NMR (151 MHz, DMSO-*d*_6_) δ 166.24, 162.73, 161.89, 150.41, 138.24, 132.85 (d, *J* = 31.0 Hz), 129.16 (d, *J* = 5.6 Hz), 128.88, 127.17, 124.06, 123.80 (d, *J* = 275.3 Hz), 115.75, 115.41, 56.08, 33.31. HRMS (ESI+) calcd for (C_17_H_12_F_3_N_3_O_4_S + H^+^) *m*/*z*: 412.05734 (100%), 413.0607 (18.4%); found: 412.0578 (100%), 413.0605 (19%).

##### 2-(4-Chlorophenyl)-5-((5-nitro-2-(trifluoromethyl)benzyl)sulfanyl)-1,3,4-oxadiazole (**73c**)

Yield: 87% as a white solid; mp 101–103 °C. ^1^H NMR (500 MHz, DMSO-*d*_6_) δ 8.66 (d, *J* = 2.4 Hz, 1H), 8.33 (dd, *J* = 8.7, 2.4 Hz, 1H), 8.07 (d, *J* = 8.7 Hz, 1H), 7.94 (d, *J* = 8.6 Hz, 2H), 7.64 (d, *J* = 8.6 Hz, 2H), 4.84 (d, *J* = 1.3 Hz, 2H). ^13^C NMR (126 MHz, DMSO-*d*_6_) δ 165.40, 162.96, 150.32, 137.96, 137.36, 132.79 (q, *J* = 30.8 Hz), 130.04, 129.10 (q, *J* = 5.6 Hz), 128.71, 127.10, 124.03, 123.71 (q, *J* = 274.7 Hz), 122.23, 33.16. HRMS (ESI+) calcd for (C_16_H_9_ClF_3_N_3_O_3_S + H^+^) *m*/*z*: 416.00780 (100%), 418.00485 (32%); found: 416.0082 (100%), 418.0052 (32%).

##### 2-(4-Bromophenyl)-5-((5-nitro-2-(trifluoromethyl)benzyl)sulfanyl)-1,3,4-oxadiazole (**73d**)

Yield: 98% as a white solid; mp 92–93 °C. ^1^H NMR (500 MHz, DMSO-*d*_6_) δ 8.66 (d, *J* = 2.4 Hz, 1H), 8.33 (dd, *J* = 8.6, 2.4 Hz, 1H), 8.07 (d, *J* = 8.7 Hz, 1H), 7.87 (d, *J* = 8.6 Hz, 2H), 7.78 (d, *J* = 8.5 Hz, 2H), 4.83 (s, 2H). ^13^C NMR (126 MHz, DMSO-*d*_6_) δ 165.51, 162.99, 150.32, 137.95, 132.96, 132.79, (q, *J* = 31.0 Hz), 129.10 (q, *J* = 5.5 Hz), 128.81, 127.10, 126.26, 124.03, 123.71 (q, *J* = 275.1 Hz), 122.56, 33.16 (d, *J* = 2.2 Hz). HRMS (ESI+) calcd for (C_16_H_9_BrF_3_N_3_O_3_S + H^+^) *m*/*z*: 459.95729 (100%), 461.95524 (97.3%); found: 461.9558 (100%), 459.9578 (97%).

##### 2-Cyclohexyl-5-((5-nitro-2-(trifluoromethyl)benzyl)sulfanyl)-1,3,4-oxadiazole (**73e**)

Yield: 96% as a white solid; mp 102–103 °C. ^1^H NMR (500 MHz, DMSO-*d*_6_) δ 8.57 (d, *J* = 2.3 Hz, 1H), 8.33 (dd, *J* = 8.6, 2.4 Hz, 1H), 8.07 (d, *J* = 8.7 Hz, 1H), 4.73 (d, *J* = 1.3 Hz, 2H), 2.91 (tt, *J* = 11.0, 3.7 Hz, 1H), 2.00–1.88 (m, 2H), 1.74–1.57 (m, 3H), 1.53–1.17 (m, 5H). ^13^C NMR (126 MHz, DMSO-*d*_6_) δ 171.77, 161.76, 150.27, 138.17, 132.77 (q, *J* = 30.9 Hz), 129.10 (q, *J* = 5.6 Hz), 127.02, 123.96, 123.68 (q, *J* = 274.7 Hz), 34.63, 33.12 (d, *J* = 2.5 Hz), 29.78, 25.53, 25.04. HRMS (ESI+) calcd for (C_16_H_16_F_3_N_3_O_3_S + H^+^) *m*/*z*: 388.09372 (100%), 389.09708 (17.3%); found: 388.0941 (100%), 389.0967 (18%).

#### 1-Alkyl/Aryl-5-((4-methoxy-3,5-dinitrobenzyl)sulfanyl)-1*H*-tetrazoles **74a**–**74e**

4-Methoxy-3,5-dinitrobenzyl bromide (**48**) was used as the alkylating agent. The reactions were completed in 30 min.

##### 5-((4-Methoxy-3,5-dinitrobenzyl)sulfanyl)-1-phenyl-1*H*-tetrazole (**74a**)

Yield: 70% as a yellowish solid; mp 119–120 °C. ^1^H NMR (600 MHz, DMSO-*d*_6_) δ 8.41 (s, 2H), 7.62–7.58 (m, 5H), 4.66 (s, 2H), 3.89 (s, 3H). ^13^C NMR (151 MHz, DMSO-*d*_6_) δ 154.11, 146.22, 144.61, 135.12, 133.46, 131.24, 130.65, 130.54, 125.10, 64.89, 35.00. Elem. Anal. Calcd for C_15_H_12_N_6_O_5_S: C, 46.39; H, 3.11; N, 21.64; S, 8.26. Found: C, 46.76; H, 2.90; N, 21.27; S, 8.30.

##### 5-((4-Methoxy-3,5-dinitrobenzyl)sulfanyl)-1-(4-methoxyphenyl)-1*H*-tetrazole (**74b**)

Yield: 83% as a yellowish solid; mp 107–108 °C. ^1^H NMR (600 MHz, DMSO-*d*_6_) δ 8.40 (s, 2H), 7.50 (d, *J* = 8.8 Hz, 2H), 7.12 (d, *J* = 8.9 Hz, 2H), 4.63 (s, 2H), 3.90 (s, 3H), 3.80 (s, 3H). ^13^C NMR (151 MHz, DMSO-*d*_6_) δ 161.19, 154.22, 146.20, 144.60, 135.21, 130.61, 126.90, 126.03, 115.54, 64.89, 56.24, 34.95. Elem. Anal. Calcd for C_16_H_14_N_6_O_6_S: C, 45.93; H, 3.37; N, 20.09; S, 7.66. Found: C, 46.15; H, 3.58; N, 19.98; S, 7.77.

##### 1-(4-Chlorophenyl)-5-((4-methoxy-3,5-dinitrobenzyl)sulfanyl)-1*H*-tetrazole (**74c**)

Yield: 83% as a white solid; mp 138–139 °C. ^1^H NMR (600 MHz, DMSO-*d*_6_) δ 8.40 (s, 2H), 7.69 (d, *J* = 9.0 Hz, 2H), 7.65 (d, *J* = 9.0 Hz, 2H), 4.65 (s, 2H), 3.90 (s, 3H). ^13^C NMR (151 MHz, DMSO-*d*_6_) δ 154.23, 146.20, 144.59, 135.87, 135.10, 132.29, 130.63, 130.58, 127.03, 64.90, 35.12. Elem. Anal. Calcd for C_15_H_11_ClN_6_O_5_S: C, 42.61; H, 2.62; N, 19.88; S, 7.58. Found: C, 42.64; H, 2.31; N, 19.90; S, 7.73.

##### 1-(4-Bromophenyl)-5-((4-methoxy-3,5-dinitrobenzyl)sulfanyl)-1*H*-tetrazole (**74d**)

Yield: 94% as a white solid; mp 151–153 °C. ^1^H NMR (600 MHz, DMSO-*d*_6_) δ 8.40 (s, 2H), 7.83 (d, *J* = 8.7 Hz, 2H), 7.58 (d, *J* = 8.7 Hz, 2H), 4.65 (s, 2H), 3.90 (s, 3H). ^13^C NMR (151 MHz, DMSO-*d*_6_) δ 154.18, 146.21, 144.60, 135.09, 133.53, 132.71, 130.63, 127.18, 124.43, 64.90, 35.12. Elem. Anal. Calcd for C_15_H_11_BrN_6_O_5_S: C, 38.56; H, 2.37; N, 17.99; S, 6.86. Found: C, 38.20; H, 2.23; N, 17.64; S, 6.72.

##### 1-Cyclohexyl-5-((4-methoxy-3,5-dinitrobenzyl)sulfanyl)-1*H*-tetrazole (**74e**)

Yield: 66% as yellow oil, which crystallized over time; mp 67–69 °C. ^1^H NMR (600 MHz, DMSO-*d*_6_) δ 8.39 (s, 2H), 4.63 (s, 2H), 4.21 (tt, *J* = 11.5, 3.9 Hz, 1H), 3.89 (s, 3H), 1.90–1.84 (m, 2H), 1.79–1.56 (m, 5H), 1.44–1.33 (m, 2H), 1.28–1.19 (m, 1H). ^13^C NMR (126 MHz, DMSO-*d*_6_) δ 152.04, 146.08, 144.55, 135.29, 130.46, 64.84, 57.95, 34.98, 32.09, 24.89, 24.83. Elem. Anal. Calcd for C_15_H_18_N_6_O_5_S: C, 45.68; H, 4.60; N, 21.31; S, 8.13. Found: C, 46.02; H, 4.56; N, 21.08; S, 8.02. HRMS (ESI+) calcd for (C_15_H_18_N_6_O_5_S + H^+^) *m*/*z*: 395.11322 (100%), 396.11657 (16.2%); found: 395.1138 (100%), 396.1158 (17%).

#### 1-Alkyl/Aryl-5-((2-methoxy-3,5-dinitrobenzyl)sulfanyl)-1*H*-tetrazoles **75a**–**75e**

2-Methoxy-3,5-dinitrobenzyl bromide (**49**) was used as the alkylating agent. The reactions were completed in 1 h.

##### 5-((2-Methoxy-3,5-dinitrobenzyl)sulfanyl)-1-phenyl-1*H*-tetrazole (**75a**)

Yield: 87% as a white solid; mp 144–145 °C. ^1^H NMR (500 MHz, DMSO-*d*_6_) δ 8.71 (d, *J* = 2.9 Hz, 1H), 8.69 (d, *J* = 2.9 Hz, 1H), 7.70–7.51 (m, 5H), 4.73 (s, 2H), 3.93 (s, 3H). ^13^C NMR (126 MHz, DMSO-*d*_6_) δ 156.25, 153.64, 142.43, 141.80, 134.59, 133.10, 130.89, 130.18, 129.97, 124.84, 121.43, 63.45, 31.51. Elem. Anal. Calcd for C_15_H_12_N_6_O_5_S: C, 46.39; H, 3.11; N, 21.64; S, 8.26. Found: C, 46.56; H, 3.02; N, 21.74; S, 8.63.

##### 5-((2-Methoxy-3,5-dinitrobenzyl)sulfanyl)-1-(4-methoxyphenyl)-1*H*-tetrazole (**75b**)

Yield: 95% as a beige solid; mp 166–168 °C. ^1^H NMR (500 MHz, DMSO-*d*_6_) δ 8.70 (d, *J* = 2.9 Hz, 1H), 8.67 (d, *J* = 2.9 Hz, 1H), 7.51 (d, *J* = 9.0 Hz, 2H), 7.13 (d, *J* = 9.0 Hz, 2H), 4.70 (s, 2H), 3.92 (s, 3H), 3.83 (s, 3H). ^13^C NMR (126 MHz, DMSO-*d*_6_) δ 160.82, 156.24, 153.75, 142.44, 141.80, 134.68, 129.92, 126.63, 125.67, 121.40, 115.17, 63.46, 55.89, 31.45. Elem. Anal. Calcd for C_15_H_14_N_6_O_6_S: C, 45.93; H, 3.37; N, 20.09; S, 7.66. Found: C, 45.94; H, 3.28; N, 20.03; S, 8.01.

##### 1-(4-Chlorophenyl)-5-((2-methoxy-3,5-dinitrobenzyl)sulfanyl)-1*H*-tetrazole (**75c**)

Yield: 93% as a brownish solid; mp 136–139 °C. ^1^H NMR (500 MHz, DMSO-*d*_6_) δ 8.71 (d, *J* = 2.9 Hz, 1H), 8.68 (d, *J* = 2.8 Hz, 1H), 7.71 (d, *J* = 8.9 Hz, 2H), 7.66 (d, *J* = 8.8 Hz, 2H), 4.72 (s, 2H), 3.93 (s, 3H). ^13^C NMR (126 MHz, DMSO-*d*_6_) δ 156.24, 153.76, 142.41, 141.78, 135.52, 134.57, 131.92, 130.21, 129.97, 126.78, 121.42, 63.46, 31.63. Elem. Anal. Calcd for C_15_H_11_ClN_6_O_5_S: C, 42.61; H, 2.62; N, 19.88; S, 7.58. Found: C, 42.93; H, 2.48; N, 19.97; S, 7.92.

##### 1-(4-Bromophenyl)-5-((2-methoxy-3,5-dinitrobenzyl)sulfanyl)-1*H*-tetrazole (**75d**)

Yield: 80% as a brownish solid; mp 153–155 °C. ^1^H NMR (500 MHz, DMSO-*d*_6_) δ 8.71 (d, *J* = 2.8 Hz, 1H), 8.67 (d, *J* = 2.9 Hz, 1H), 7.84 (d, *J* = 8.7 Hz, 2H), 7.59 (d, *J* = 8.7 Hz, 2H), 4.72 (s, 2H), 3.93 (s, 3H). ^13^C NMR (126 MHz, DMSO-*d*_6_) δ 156.20, 153.66, 142.40, 141.78, 134.54, 133.12, 132.33, 129.92, 126.89, 124.06, 121.36, 63.43, 31.64. Elem. Anal. Calcd for C_15_H_11_BrN_6_O_5_S: C, 38.56; H, 2.37; N, 17.99; S, 6.86. Found: C, 38.72 H, 2.21; N, 17.91; S, 7.08.

##### 1-Cyclohexyl-5-((2-methoxy-3,5-dinitrobenzyl)sulfanyl)-1*H*-tetrazole (**75e**)

Yield: 80% as a yellow solid; mp 96–97 °C. ^1^H NMR (600 MHz, DMSO-*d*_6_) δ 8.69 (d, *J* = 2.9 Hz, 1H), 8.63 (d, *J* = 2.9 Hz, 1H), 4.68 (s, 2H), 4.24 (tt, *J* = 11.5, 3.9 Hz, 1H), 3.93 (s, 3H), 1.92–1.85 (m, 2H), 1.81–1.66 (m, 4H), 1.67–1.56 (m, 1H), 1.40–1.31 (m, 2H), 1.23–1.16 (m, 1H). ^13^C NMR (151 MHz, DMSO-*d*_6_) δ 156.55, 152.10, 142.91, 142.19, 135.25, 130.13, 121.69, 63.84, 58.06, 32.20, 31.85, 24.97, 24.92. Elem. Anal. Calcd for C_15_H_18_N_6_O_5_S: C, 45.68; H, 4.60; N, 21.31; S, 8.13. Found: C, 46.05; H, 4.57; N, 21.25; S, 8.28.

#### 1-Alkyl/Aryl-5-((4-methyl-3,5-dinitrobenzyl)sulfanyl)-1*H*-tetrazoles **76a**–**76e**

4-Methyl-3,5-dinitrobenzyl bromide (**50**) was used as the alkylating agent. The reactions were completed in 1 h.

##### 5-((4-Methyl-3,5-dinitrobenzyl)sulfanyl)-1-phenyl-1*H*-tetrazole (**76a**)

Yield: 70% as a beige solid; mp 112–113 °C. ^1^H NMR (500 MHz, acetone-*d*_6_) δ 8.38 (s, 2H), 7.72–7.61 (m, 5H), 4.85 (s, 2H), 2.52 (s, 3H). ^13^C NMR (126 MHz, acetone-*d*_6_) δ 154.26, 152.39, 139.51, 134.55, 131.46, 130.92, 129.16, 126.70, 125.26, 35.70, 14.74. Elem. Anal. Calcd for C_15_H_12_N_6_O_4_S: C, 48.38; H, 3.25; N, 22.57; S, 8.61. Found: C,48.48; H, 3.50; N, 22.81; S, 8.90.

##### 1-(4-Methoxyphenyl)-5-((4-methyl-3,5-dinitrobenzyl)sulfanyl)-1*H*-tetrazole (**76b**)

Yield: 71% as a white solid; 144–145 °C. ^1^H NMR (600 MHz, DMSO-*d*_6_) δ 8.30 (s, 2H), 7.49 (d, *J* = 8.9 Hz, 2H), 7.11 (d, *J* = 9.0 Hz, 2H), 4.64 (s, 2H), 3.80 (s, 3H), 2.39 (s, 3H). ^13^C NMR (151 MHz, DMSO-*d*_6_) δ 161.19, 154.12, 151.24, 138.89, 128.81, 126.91, 126.02, 125.98, 115.52, 56.23, 35.04, 14.79. Elem. Anal. Calcd for C_16_H_14_N_6_O_5_S: C, 47.76; H, 3.51; N, 20.89; S, 7.97. Found: C, 47.72; H, 3.22; N, 21.09; S, 8.18.

##### 1-(4-Chlorophenyl)-5-((4-methyl-3,5-dinitrobenzyl)sulfanyl(-1*H*-tetrazole (**76c**)

Yield: 94% as a beige solid; mp133–134 °C. ^1^H NMR (600 MHz, DMSO-*d*_6_) δ 8.30 (s, 2H), 7.68 (d, *J* = 8.7 Hz, 2H), 7.63 (d, *J* = 8.9 Hz, 2H), 4.66 (s, 2H), 2.39 (s, 3H). ^13^C NMR (151 MHz, DMSO-*d*_6_) δ 154.12, 151.24, 138.78, 135.86, 132.27, 130.55, 128.83, 127.05, 125.99, 35.24, 14.78. Elem. Anal. Calcd for C_15_H_11_ClN_6_O_4_S: C, 44.29; H, 2.73; N, 20.66; S, 7.88. Found: C, 44.20; H, 2.48; N, 20.67; S, 8.04.

##### 1-(4-Bromophenyl)-5-((4-methyl-3,5-dinitrobenzyl)sulfanyl)-1*H*-tetrazole (**76d**)

Yield: 94% as a white solid; mp 124–125 °C. ^1^H NMR (500 MHz, acetone-*d*_6_) δ 8.35 (s, 2H), 7.85 (d, *J* = 8.9 Hz, 2H), 7.61 (d, *J* = 8.8 Hz, 2H), 4.83 (s, 2H), 2.50 (s, 3H). ^13^C NMR (126 MHz, acetone-*d*_6_) δ 154.25, 152.29, 139.34, 133.97, 133.65, 129.06, 127.07, 126.61, 124.79, 35.71, 14.64. Elem. Anal. Calcd for C_15_H_11_BrN_6_O_4_S: C, 39.93; H, 2.46; N, 18.62; S, 7.10. Found: 40.23; H, 2.31; N, 18.40; S, 7.11.

##### 1-Cyclohexyl-5-((4-methyl-3,5-dinitrobenzyl)sulfanyl)-1*H*-tetrazole (**76e**)

Yield: 73% as a white solid; mp 121–122 °C. ^1^H NMR (600 MHz, DMSO-*d*_6_) δ 8.31 (s, 2H), 4.65 (s, 2H), 4.21 (tt, *J* = 11.5, 3.9 Hz, 1H), 2.39 (s, 3H), 1.90–1.81 (m, 2H), 1.79–1.73 (m, 2H), 1.74–1.64 (m, 2H), 1.65–1.53 (m, 1H), 1.41–1.30 (m, 2H), 1.23–1.13 (m, 1H). ^13^C NMR (151 MHz, DMSO-*d*_6_) δ 152.05, 151.28, 139.02, 128.77, 125.98, 58.02, 35.08, 32.16, 24.97, 24.91, 14.76. Elem. Anal. Calcd for C_15_H_18_N_6_O_4_S: C, 47.61; H, 4.79; N, 22.21; S, 8.47. Found: C, 47.78; H, 4.69; N, 22.26; S, 8.46.

#### 1-Alkyl/Aryl-5-((2-methyl-3,5-dinitrobenzyl)sulfanyl)-1*H*-tetrazoles **77a**–**77e**

2-Methyl-3,5-dinitrobenzyl bromide (**51**) was used as the alkylating agent. The reactions were completed in 1 h.

##### 5-((2-Methyl-3,5-dinitrobenzyl)sulfanyl)-1-phenyl-1*H*-tetrazole (**77a**)

Yield: 76% as a yellow solid; mp 128–129 °C. ^1^H NMR (600 MHz, DMSO-*d*_6_) δ 8.58 (s, 2H), 7.61–7.54 (m, 5H), 4.81 (s, 2H), 2.46 (s, 3H, overlap with solvent). ^13^C NMR (151 MHz, DMSO-*d*_6_) δ 153.68, 151.31, 145.58, 140.13, 138.67, 133.42, 131.23, 130.50, 128.57, 125.16, 119.13, 34.92, 15.56. Elem. Anal. Calcd for C_15_H_12_N_6_O_4_S: C, 48.38; H, 3.25; N, 22.57; S, 8.61. Found: C, 48.19; H, 3.32; N, 22.75; S, 8.82

##### 1-(4-Methoxyphenyl)-5-((2-methyl-3,5-dinitrobenzyl)sulfanyl)-1*H*-tetrazole (**77b**)

Yield: 80% as a white solid; mp 168–168 °C. ^1^H NMR (600 MHz, DMSO-*d*_6_) δ 8.57 (d, *J* = 2.5 Hz, 1H), 8.55 (d, *J* = 2.5 Hz, 1H), 7.46 (d, *J* = 9.0 Hz, 2H), 7.10 (d, *J* = 9.0 Hz, 2H), 4.77 (s, 2H), 3.80 (s, 3H), 2.45 (s, 3H). ^13^C NMR (151 MHz, DMSO-*d*_6_) δ 161.18, 153.76, 151.30, 145.56, 140.21, 138.63, 128.50, 126.93, 126.00, 119.10, 115.50, 56.22, 34.88, 15.55. Elem. Anal. Calcd for C_16_H_14_N_6_O_5_S: C, 47.76; H, 3.51; N, 20.89; S, 7.97. Found: C, 48.09; H, 3.46; N, 20.93; S, 7.95.

##### 1-(4-Chlorophenyl)-5-((2-methyl-3,5-dinitrobenzyl)sulfanyl)-1*H*-tetrazole (**77c**)

Yield: 80% as a yellow solid; mp 131–133 °C. ^1^H NMR (500 MHz, DMSO-*d*_6_) δ 8.61 (d, *J* = 2.5 Hz, 1H), 8.59 (d, *J* = 2.5 Hz, 1H), 7.70 (d, *J* = 8.8 Hz, 2H), 7.65 (d, *J* = 8.8 Hz, 2H), 4.83 (s, 2H), 2.49 (s, 3H). ^13^C NMR (126 MHz, DMSO-*d*_6_) δ 153.39, 150.92, 145.18, 139.72, 138.28, 135.50, 131.86, 130.14, 128.17, 126.70, 118.74, 34.71, 15.19. Elem. Anal. Calcd for C_15_H_11_ClN_6_O_4_S: C, 44.29; H, 2.73; N, 20.66; S, 7.88. Found: C, 43.95; H, 2.45; N, 20.66; S, 7.79.

##### 1-(4-Bromophenyl)-5-((2-methyl-3,5-dinitrobenzyl)sulfanyl)-1*H*-tetrazole (**77d**)

Yield: 94% as a yellow solid; mp 145–146 °C. ^1^H NMR (600 MHz, DMSO-*d*_6_) δ 8.57 (d, *J* = 2.4 Hz, 1H), 8.55 (d, *J* = 2.5 Hz, 1H), 7.80 (d, *J* = 8.7 Hz, 2H), 7.54 (d, *J* = 8.8 Hz, 2H), 4.79 (s, 2H), 2.45 (s, 3H). ^13^C NMR (151 MHz, DMSO-*d*_6_) δ 153.73, 151.30, 145.57, 140.10, 138.67, 133.48, 132.66, 128.55, 127.24, 124.45, 119.12, 35.08, 15.56. Elem. Anal. Calcd for C_15_H_11_BrN_6_O_4_S: C, 39.93; H, 2.46; N, 18.62; S, 7.10. Found: C, 40.27; H, 2.28; N, 18.74; S, 7.15.

##### 1-Cyclohexyl-5-((2-methyl-3,5-dinitrobenzyl)sulfanyl)-1*H*-tetrazole (**77e**)

Yield: 79% as a white solid; mp 97–98 °C. ^1^H NMR (600 MHz, DMSO-*d*_6_) δ 8.59 (d, *J* = 2.4 Hz, 1H), 8.51 (d, *J* = 2.5 Hz, 1H), 4.79 (s, 2H), 4.23 (tt, *J* = 11.5, 3.9 Hz, 1H), 2.51 (s, 3H), 1.88–1.81 (m, 2H), 1.78–1.74 (m, 2H), 1.71–1.64 (m, 2H), 1.62–1.59 (m, 1H), 1.40–1.31 (m, 2H), 1.26–1.09 (m, 1H). ^13^C NMR (151 MHz, DMSO-*d*_6_) δ 151.75, 151.42, 145.56, 140.43, 138.57, 128.32, 119.03, 58.09, 34.90, 32.19, 24.95, 24.92, 15.54. Elem. Anal. Calcd for C_15_H_18_N_6_O_4_S: C, 47.61; H, 4.79; N, 22.21; S, 8.47. Found: C, 47.62; H, 4.57; N, 22.37; S, 8.60.

#### 2-Alkyl/Aryl-5-((4-methoxy-3,5-dinitrobenzyl)sulfanyl)-1,3,4-oxadiazoles **78a**–**78e**

4-Methoxy-3,5-dinitrobenzyl bromide (**48**) was used as the alkylating agent. The reactions were completed in 30 min.

##### 2-((4-Methoxy-3,5-dinitrobenzyl)sulfanyl)-5-phenyl-1,3,4-oxadiazole (**78a**)

Yield: 75% as a yellow solid; mp 99–101 °C. ^1^H NMR (500 MHz, acetone-*d*_6_) δ 8.51 (s, 2H), 8.02–7.98 (m, 2H), 7.65–7.55 (m, 3H), 4.80 (s, 2H), 4.05 (s, 3H). ^13^C NMR (126 MHz, acetone-*d*_6_) δ 166.79, 163.71, 147.15, 145.82, 136.05, 132.71, 130.71, 130.10, 127.31, 124.49, 64.98, 34.91. Elem. Anal. Calcd for C_16_H_12_N_4_O_6_S: C, 49.48; H, 3.11; N, 14.43; S, 8.26. Found: C, 49.52; H, 2.97; N, 14.47; S, 8.61.

##### 2-((4-Methoxy-3,5-dinitrobenzyl)sulfanyl)-5-(4-methoxyphenyl)-1,3,4-oxadiazole (**78b**)

Yield: 70% as a yellowish solid; mp 128–131 °C. ^1^H NMR (500 MHz, DMSO-*d*_6_) δ 8.49 (s, 2H), 7.88 (d, *J* = 9.0 Hz, 2H), 7.11 (d, *J* = 9.0 Hz, 2H), 4.66 (s, 2H), 3.93 (s, 3H), 3.84 (s, 3H). ^13^C NMR (126 MHz, DMSO-*d*_6_) δ 165.59, 162.26, 162.09, 145.89, 144.28, 135.18, 130.24, 128.48, 115.45, 114.99, 64.51, 55.70, 33.96. Elem. Anal. Calcd for: C_17_H_14_N_4_O_7_S: C, 48.80; H, 3.37; N, 13.39; S, 7.66. Found: C, 49.09; H, 3.41; N, 13.35; S, 7.97.

##### 2-(4-Chlorophenyl)-5-((4-methoxy-3,5-dinitrobenzyl)sulfanyl)-1,3,4-oxadiazole (**78c**)

Yield: 81% as a yellow solid; mp 105–107 °C. ^1^H NMR (500 MHz, acetone-*d*_6_) δ 8.50 (s, 2H), 8.01 (d, *J* = 8.9 Hz, 2H), 7.63 (d, *J* = 8.9 Hz, 2H), 4.80 (s, 2H), 4.04 (s, 3H). ^13^C NMR (126 MHz, acetone-*d*_6_) δ 166.02, 164.05, 147.17, 145.80, 138.24, 135.97, 130.72, 130.35, 128.98, 123.27, 64.99, 34.90. Elem. Anal. Calcd for C_16_H_11_ClN_4_O_6_S: C, 45.45; H, 2.62; N, 13.25; S, 7.58. Found: C, 45.80; H, 2.69; N, 13.23; S, 7.93.

##### 2-(4-Bromophenyl)-5-((4-methoxy-3,5-dinitrobenzyl)sulfanyl)-1,3,4-oxadiazole (**78d**)

Yield: 91% as a yellow solid; mp 142–145 °C. ^1^H NMR (500 MHz, DMSO-*d*_6_) δ 8.50 (s, 2H), 7.88 (d, *J* = 8.6 Hz, 2H), 7.78 (d, *J* = 8.6 Hz, 2H), 4.68 (s, 2H), 3.92 (s, 3H). ^13^C NMR (126 MHz, DMSO-*d*_6_) δ 165.02, 163.30, 145.97, 144.30, 135.10, 132.66, 130.34, 128.54, 125.89, 122.38, 64.56, 33.92. Anal. Calcd for C_16_H_11_BrN_4_O_6_S: C, 41.13; H, 2.37; N, 11.99; S, 6.86. Found: C, 41.36; H, 2.26; N, 11.9; S, 7.23.

##### 2-Cyclohexyl-5-((4-methoxy-3,5-dinitrobenzyl)sulfanyl)-1,3,4-oxadiazole (**78e**)

Yield: 75% as a yellow solid; 125–126 °C. ^1^H NMR (600 MHz, DMSO-*d*_6_) δ 8.39 (s, 2H), 4.54 (s, 2H), 3.90 (s, 3H), 2.87 (tt, *J* = 11.1, 3.7 Hz, 1H), 1.92–1.86 (m, 2H), 1.70–1.63 (m, 2H), 1.62–1.55 (m, 1H), 1.46–1.36 (m, 2H), 1.35–1.26 (m, 2H), 1.24–1.16 (m, 1H). ^13^C NMR (151 MHz, DMSO-*d*_6_) δ 171.52, 162.37, 146.24, 144.64, 135.61, 130.58, 64.90, 34.69, 34.25, 29.88, 25.60, 25.15. Anal. Calcd for C_16_H_18_N_4_O_6_S: C, 48.73; H, 4.60; N, 14.21; S, 8.13. Found: C, 49.12; H, 4.67; N, 14.01; S, 8.16.

#### 2-Alkyl/Aryl-5-((2-methoxy-3,5-dinitrobenzyl)sulfanyl)-1,3,4-oxadiazoles **79a**–**79e**

2-Methoxy-3,5-dinitrobenzyl bromide (**49**) was used as the alkylating agent. The reactions were completed in 30 min.

##### 2-((2-Methoxy-3,5-dinitrobenzyl)sulfanyl)-5-phenyl-1,3,4-oxadiazole (**79a**)

Yield: 68% as a yellow solid; mp 92–95 °C. ^1^H NMR (500 MHz, acetone-*d*_6_) δ 8.85 (d, *J* = 2.8 Hz, 1H), 8.72 (d, *J* = 2.8 Hz, 1H), 8.03–7.97 (m, 2H), 7.66–7.54 (m, 3H), 4.82 (s, 2H), 4.15 (s, 3H). ^13^C NMR (126 MHz, acetone-*d*_6_) δ 166.80, 163.73, 157.53, 143.00, 136.07, 132.72, 130.49, 130.11, 127.32, 124.48, 122.06, 63.86, 31.45. Elem. Anal. Calcd for C, 49.48; H, 3.11; N, 14.43; N, 8.26. Found: C, 49.73; H, 3.12; N, 14.48; S, 8.62.

##### 2-((2-Methoxy-3,5-dinitrobenzyl)sulfanyl)-5-(4-methoxyphenyl)-1,3,4-oxadiazole (**79b**)

Yield: 78% as a yellowish solid; mp 115–117 °C. ^1^H NMR (500 MHz, acetone-*d*_6_) δ 8.83 (d, *J* = 2.8 Hz, 1H), 8.72 (d, *J* = 2.9 Hz, 1H), 7.93 (d, *J* = 8.8 Hz, 2H), 7.12 (d, *J* = 8.9 Hz, 2H), 4.79 (s, 2H), 4.14 (s, 3H), 3.91 (s, 3H). ^13^C NMR (126 MHz, acetone-*d*_6_) δ 165.93, 162.63, 162.00, 156.65, 142.60, 142.14, 135.30, 129.60, 128.30, 121.17, 115.93, 114.65, 63.00, 55.07, 30.60. Elem. Anal. Calcd for C_17_H_14_N_4_O_7_S: C, 48.80; H, 3.37; N, 13.39; S, 7.66. Found: C, 48.85; H, 3.27; N, 13.14; S, 7.43.

##### 2-(4-Chlorophenyl)-5-((2-methoxy-3,5-dinitrobenzyl)sulfanyl)-1,3,4-oxadiazole (**79c**)

Yield: 76% as a yellowish solid; 109–112 °C. ^1^H NMR (500 MHz, acetone-*d*_6_) δ 8.84 (d, *J* = 2.8 Hz, 1H), 8.72 (d, *J* = 2.9 Hz, 1H), 8.02 (d, *J* = 8.6 Hz, 2H), 7.63 (d, *J* = 8.6 Hz, 2H), 4.83 (s, 2H), 4.15 (s, 3H). ^13^C NMR (126 MHz, acetone-*d*_6_) δ 166.03, 164.08, 157.52, 143.44, 143.00, 138.25, 136.00, 130.49, 130.36, 128.99, 123.27, 122.08, 63.86, 31.45. Elem. Anal. Calcd for C_16_H_11_ClN_4_O_6_S: C, 45.45; H, 2.62; N, 13.25; S, 7.58. Found: C, 45.59; H, 2.40; N, 13.39; S, 7.65.

##### 2-(4-Bromophenyl)-5-((2-methoxy-3,5-dinitrobenzyl)sulfanyl)-1,3,4-oxadiazole (**79d**)

Yield: 76% as a beige solid; mp 114–116 °C. ^1^H NMR (500 MHz, DMSO-*d*_6_) δ 8.76 (d, *J* = 2.9 Hz, 1H), 8.73 (d, *J* = 2.8 Hz, 1H), 7.89 (d, *J* = 8.6 Hz, 2H), 7.79 (d, *J* = 8.6 Hz, 2H), 4.74 (s, 2H), 3.98 (s, 3H). ^13^C NMR (126 MHz, DMSO-*d*_6_) δ 165.34, 163.43, 156.54, 142.71, 142.11, 135.22, 132.94, 130.24, 128.82, 126.19, 122.62, 121.76, 63.91, 31.09. Anal. Calcd for C_16_H_11_BrN_4_O_6_S: C, 41.13; H, 2.37; N, 11.99; S, 6.86. Found: C, 41.44; H, 2.35; N, 11.92; S, 7.25.

##### 2-Cyclohexyl-5-((2-methoxy-3,5-dinitrobenzyl)sulfanyl)-1,3,4-oxadiazole (**79e**)

Yield: 90% as a yellowish oil. ^1^H NMR (600 MHz, DMSO-*d*_6_) δ 8.69 (d, *J* = 3.0 Hz, 1H), 8.63 (d, *J* = 2.9 Hz, 1H), 4.59 (s, 2H), 3.93 (s, 3H), 2.93–2.80 (m, 1H), 1.95–1.84 (m, 2H), 1.72–1.54 (m, 3H), 1.46–1.37 (m, 2H), 1.35–1.25 (m, 2H), 1.24–1.15 (m, 1H). ^13^C NMR (151 MHz, DMSO-*d*_6_) δ 171.66, 162.25, 156.60, 142.86, 142.18, 135.46, 130.17, 121.74, 63.90, 34.72, 31.06, 29.89, 25.61, 25.15. HRMS (ESI+) calcd for (C_16_H_18_N_4_O_6_S + H^+^) *m*/*z*: 395.10198 (100%), 396.10533 (17.3%); found: 395.1033 (100%), 396.1055 (17%).

#### 2-Alkyl/Aryl-5-((4-methyl-3,5-dinitrobenzyl)sulfanyl)-1,3,4-oxadiazoles **80a**–**80e**

4-Methyl-3,5-dinitrobenzyl bromide (**50**) was used as the alkylating agent. The reactions were completed in 1 h.

##### 2-((4-Methyl-3,5-dinitrobenzyl)sulfanyl)-5-phenyl-1,3,4-oxadiazole (**80a**)

Yield: 86% as a beige solid; mp 116–117 °C. ^1^H NMR (500 MHz, acetone-*d*_6_) δ 8.44 (s, 2H), 8.01–7.97 (m, 2H), 7.64–7.56 (m, 3H), 4.82 (s, 2H), 2.53 (s, 3H). ^13^C NMR (126 MHz, acetone-*d*_6_) δ 166.79, 163.67, 152.35, 139.69, 132.70, 130.09, 129.01, 127.30, 126.69, 124.47, 34.95, 14.67. Elem. Anal. Calcd for C_16_H_12_N_4_O_6_S: C, 51.61; H, 3.25; N, 15.05; S, 8.61. Found: 51.50; H, 3.07; N, 15.12; S, 8.77.

##### 2-(4-Methoxyphenyl)-5-((4-methyl-3,5-dinitrobenzyl)sulfanyl)-1,3,4-oxadiazole (**80b**)

Yield: 70% as a white solid; mp 124–125 °C. ^1^H NMR (600 MHz, DMSO-*d*_6_) δ 8.37 (s, 2H), 7.83 (d, *J* = 9.0 Hz, 2H), 7.07 (d, *J* = 9.0 Hz, 2H), 4.64 (s, 2H), 3.80 (s, 3H), 2.39 (s, 3H). ^13^C NMR (151 MHz, DMSO-*d*_6_) δ 165.98, 162.65, 162.41, 151.32, 139.24, 128.85, 128.82, 126.10, 115.81, 115.36, 56.07, 34.40, 14.83. Elem. Anal. Calcd for C_15_H_16_N_4_O_5_S: C, 50.74; H, 3.51; N, 13.92; S, 7.97. Found: C, 50.64; H, 3.34; N, 13.91; S, 7.79.

##### 2-(4-Chlorophenyl)-5-((4-methyl-3,5-dinitrobenzyl)sulfanyl)-1,3,4-oxadiazole (**80c**)

Yield: 81% as a beige solid; mp 159–160 °C. ^1^H NMR (500 MHz, acetone-*d*_6_) δ 8.43 (s, 2H), 8.00 (d, *J* = 8.6 Hz, 2H), 7.63 (d, *J* = 8.6 Hz, 2H), 4.82 (s, 2H), 2.53 (s, 3H). ^13^C NMR (126 MHz, acetone-*d*_6_) δ 166.02, 164.01, 152.36, 139.63, 138.23, 130.34, 129.02, 128.98, 126.72, 123.26, 34.95, 14.67. Elem. Anal. Calcd for C_16_H_11_ClN_4_O_5_S: C, 47.24; H, 2.73; N, 13.77; S, 7.88. Found: C, 47.31; H, 2.4; N, 13.88; S, 7.89.

##### 2-(4-Bromophenyl)-5-((4-methyl-3,5-dinitrobenzyl)sulfanyl)-1,3,4-oxadiazole (**80d**)

Yield: 75% as a white solid; mp 160–161 °C. ^1^H NMR (600 MHz, DMSO-*d*_6_) δ 8.37 (s, 2H), 7.83 (d, *J* = 8.6 Hz, 2H), 7.75 (d, *J* = 8.6 Hz, 2H), 4.66 (s, 2H), 2.39 (s, 3H). ^13^C NMR (151 MHz, DMSO-*d*_6_) δ 165.39, 163.59, 151.33, 139.14, 133.01, 128.89, 128.85, 126.23, 126.14, 122.72, 34.37, 14.83. Elem. Anal. Calcd for C_16_H_11_BrN_4_O_5_S: C, 42.59; H, 2.46; N, 12.42; S, 7.10. Found: C, 42.58; H, 2.23; N, 12.36; S, 7.22.

##### 2-Cyclohexyl-5-((4-methyl-3,5-dinitrobenzyl)sulfanyl)-1,3,4-oxadiazole (**80e**)

Yield: 70% as a white solid; mp 96–97 °C. ^1^H NMR (600 MHz, DMSO-*d*_6_) δ 8.31 (s, 2H), 4.56 (s, 2H), 2.86 (tt, *J* = 11.0, 3.7 Hz, 1H), 2.40 (s, 3H), 1.92–1.85 (m, 2H), 1.71–1.54 (m, 3H), 1.49–1.36 (m, 2H), 1.36–1.25 (m, 2H), 1.23–1.12 (m, 1H). ^13^C NMR (151 MHz, DMSO-*d*_6_) δ 171.55, 162.30, 151.30, 139.27, 128.80, 126.07, 34.69, 34.33, 29.88, 25.61, 25.16, 14.81. Elem. Anal. Calcd for C_16_H_18_N_4_O_5_S: C, 50.79; H, 4.79; N, 14.81; S, 8.47. Found: C, 50.80; H, 4.63; N, 14.90; S, 8.55.

#### 2-Alkyl/Aryl-5-((2-methyl-3,5-dinitrobenzyl)sulfanyl)-1,3,4-oxadiazoles **81a**–**81e**

2-Methyl-3,5-dinitrobenzyl bromide (**51**) was used as the alkylating agent. The reactions were completed in 1 h.

##### 2-((2-Methyl-3,5-dinitrobenzyl)sulfanyl)-5-phenyl-1,3,4-oxadiazole (**81a**)

Yield: 87% as a yellow solid; mp 93–94 °C. ^1^H NMR (500 MHz, CDCl_3_) δ 8.67 (d, *J* = 2.4 Hz, 1H), 8.58 (d, *J* = 2.4 Hz, 1H), 8.05–7.92 (m, 2H), 7.56–7.45 (m, 3H), 4.70 (s, 2H), 2.69 (s, 3H). ^13^C NMR (126 MHz, CDCl_3_) δ 166.42, 162.12, 151.24, 145.64, 139.06, 138.43, 131.99, 129.11, 128.01, 126.70, 123.15, 119.11, 34.09, 15.72. Elem. Anal. Calcd for C_16_H_12_N_4_O_5_S: C, 51.61; H, 3.25; N, 15.05; S, 8.61. Found: C, 51.62; H, 3.01; N, 15.19; S, 8.74.

##### 2-(4-Methoxyphenyl)-5-((2-methyl-3,5-dinitrobenzyl)sulfanyl)-1,3,4-oxadiazole (**81b**)

Yield: 82% as a yellow solid; mp 118–120 °C. ^1^H NMR (500 MHz, acetone-*d*_6_) δ 8.75 (d, *J* = 2.4 Hz, 1H), 8.59 (d, *J* = 2.4 Hz, 1H), 7.90 (d, *J* = 8.9 Hz, 2H), 7.09 (d, *J* = 8.9 Hz, 2H), 4.90 (s, 2H), 3.89 (s, 3H), 2.70 (s, 3H). ^13^C NMR (126 MHz, acetone-*d*_6_) δ 166.37, 163.07, 162.04, 151.83, 146.07, 140.74, 138.80, 128.72, 128.38, 118.99, 116.31, 115.06, 55.49, 34.22, 15.22. Elem. Anal. Calcd for C_17_H_1_N_4_O_6_S: C, 50.74; H, 3.51; N, 13.92; S, 7.97. Found: C, 50.38; H, 3.36; N, 13.65; S, 8.18.

##### 2-(4-Chlorophenyl)-5-((2-methyl-3,5-dinitrobenzyl)sulfanyl)-1,3,4-oxadiazole (**81c**)

Yield: 75% as a yellow solid; mp 120–121 °C. ^1^H NMR (500 MHz, CDCl_3_) δ 8.67 (d, *J* = 2.3 Hz, 1H), 8.58 (d, *J* = 2.4 Hz, 1H), 7.93 (d, *J* = 8.8 Hz, 2H), 7.49 (d, *J* = 8.7 Hz, 2H), 4.70 (s, 2H), 2.69 (s, 3H). ^13^C NMR (126 MHz, CDCl_3_) δ 165.62, 162.42, 151.26, 145.65, 138.94, 138.41, 138.34, 129.54, 128.02, 127.96, 121.62, 119.16, 34.08, 15.73. Elem. Anal. Calcd for C_16_H_11_ClN_4_O_5_S: C, 47.24; H, 2.73; N, 13.77; S, 7.88. Found: C, 47.41; H, 2.50; N, 13.88; S, 8.26.

##### 2-(4-Bromophenyl)-5-((2-methyl-3,5-dinitrobenzyl)sulfanyl)-1,3,4-oxadiazole (**81d**)

Yield: 79% as a yellow solid; mp 141–142 °C. ^1^H NMR (500 MHz, CDCl_3_) δ 8.67 (d, *J* = 2.4 Hz, 1H), 8.58 (d, *J* = 2.4 Hz, 1H), 7.86 (d, *J* = 8.7 Hz, 2H), 7.65 (d, *J* = 8.7 Hz, 2H), 4.70 (s, 2H), 2.69 (s, 3H). ^13^C NMR (126 MHz, CDCl_3_) δ 165.71, 162.47, 151.26, 145.65, 138.93, 138.41, 132.50, 128.08, 128.02, 126.76, 122.05, 119.16, 34.08, 15.73. Elem. Anal. Calcd for C_16_H_11_BrN_4_O_5_S: C, 42.59; H, 2.46; N, 12.42; S, 7.10. Found: C, 42.23; H, 2.15; N, 12.29; S, 7.11.

##### 2-Cyclohexyl-5-((2-methyl-3,5-dinitrobenzyl)sulfanyl)-1,3,4-oxadiazole (**81e**)

Yield: 75% as a colorless oil. ^1^H NMR (600 MHz, DMSO-*d*_6_) δ 8.60 (d, *J* = 2.4 Hz, 1H), 8.54 (d, *J* = 2.4 Hz, 1H), 4.72 (s, 2H), 2.88–2.84 (m, 1H), 2.50 (s, 3H), 1.93–1.85 (m, 2H), 1.74–1.57 (m, 3H), 1.44–1.37 (m, 2H), 1.34–1.27 (m, 2H), 1.25–1.14 (m, 1H). ^13^C NMR (151 MHz, DMSO-*d*_6_) δ 171.68, 161.98, 151.43, 145.56, 140.68, 138.62, 128.39, 119.09, 34.70, 34.10, 29.88, 25.60, 25.15, 15.57. HRMS (ESI+) calcd for (C_16_H_18_N_4_O_5_S + H^+^) *m*/*z*: 379.10707 (100%), 379.11042 (17.3%); found: 379.1080 (100%), 380.1106 (17%).

#### 2-Alkyl/Aryl-5-((5-nitrofuran-2-yl)methylsulfanyl)-1,3,4-oxadiazoles **83a**–**83e**

Commercially available 5-(bromomethyl)-2-nitrofuran was used as the alkylating agent. The reactions were completed in 30 min.

##### 2-((5-Nitrofuran-2-yl)methylsulfanyl)-5-phenyl-1,3,4-oxadiazole (**83a**)

Yield: 73% as a yellow solid; mp 102–104 °C. ^1^H NMR (500 MHz, acetone-*d*_6_) δ 8.08–8.00 (m, 2H), 7.67–7.56 (m, 3H), 7.48 (d, *J* = 3.7 Hz, 1H), 6.9 (d, *J* = 3.7, 1H), 4.80 (s, 2H). ^13^C NMR (126 MHz, aceton-*D*_6_) δ 166.95, 163.19, 155.07, 132.75, 130.11, 127.37, 124.51, 113.78, 113.73, 29.25. Elem. Anal. Calcd for C_13_H_9_N_3_O_4_S: C, 51.48; H, 2.99; N, 13.85; S, 10.57. Found: C, 51.65; H, 3.02; N, 13.77; S, 10.21.

##### 2-(4-Methoxyphenyl)-5-((5-nitrofuran-2-yl)methylsulfanyl)-1,3,4-oxadiazole (**83b**)

Yield: 56% as a yellow solid; mp 108–110 °C. ^1^H NMR (500 MHz, acetone-*d*_6_) δ 7.97 (d, *J* = 8.4 Hz, 2H), 7.48 (dd, *J* = 3.7, 0.5 Hz, 1H), 7.13 (d, *J* = 8.5 Hz, 2H), 6.88 (dd, *J* = 3.7, 0.6 Hz, 1H), 4.77 (s, 2H), 3.91 (s, 3H). ^13^C NMR (126 MHz, acetone-*d*_6_) δ 166.94, 163.51, 162.31, 155.16, 129.21, 116.82, 115.51, 113.73, 55.93, 29.28. Elem. Anal. Calcd for C_14_H_11_N_3_O_5_S: C, 50.45; H, 3.33; N, 12.61; S, 9.62. Found: C, 50.06; H 3.47; N, 12.50; S 9.24.

##### 2-(4-Chlorophenyl)-5-((5-nitrofuran-2-yl)methylsulfanyl)-1,3,4-oxadiazole (**83c**)

Yield: 61% as a beige solid; mp 125–127 °C. ^1^H NMR (500 MHz, acetone-*d*_6_) δ 8.05 (d, *J* = 8.4 Hz, 2H), 7.64 (d, *J* = 8.4 Hz, 2H), 7.48 (dd, *J* = 3.7, 0.5 Hz, 1H), 6.89 (dd, *J* = 3.8, 0.7 Hz, 1H), 4.80 (s, 2H). ^13^C NMR (126 MHz, acetone-*d*_6_) δ 166.17, 163.53, 155.00, 138.27, 130.36, 129.04, 123.27, 113.81, 113.73, 29.23. Elem. Anal. Calcd for C_13_H_8_ClN_3_O_4_S: C, 46.23; H, 2.39; N, 12.44; S, 9.49. Found: C, 46.46; H, 2.49; N, 12.05; S, 9.88.

##### 2-(4-Bromophenyl)-5-((5-nitrofuran-2-yl)methylsulfanyl)-1,3,4-oxadiazole (**83d**)

Yield: 43% as a white solid; mp 143–145 °C. ^1^H NMR (500 MHz, acetone-*d*_6_) δ 7.98 (d, *J* = 8.3 Hz, 2H), 7.81 (d, *J* = 8.3 Hz, 2H), 7.48 (dd, *J* = 3.7, 0.6 Hz, 1H), 6.89 (dd, *J* = 3.8, 0.6 Hz, 1H), 4.81 (s, 2H). ^13^C NMR (126 MHz, acetone-*d*_6_) δ 166.28, 163.57, 155.00, 133.38, 129.17, 126.71, 123.69, 113.82, 113.73, 29.24. Elem. Anal. Calcd for C_13_H_8_BrN_3_O_4_S: C, 40.86; H, 2.11; N, 10.99; S, 8.39. Found: C, 40.89; H, 1.94; N, 10.92; S, 8.48.

##### 2-Cyclohexyl-5-((5-nitrofuran-2-yl)methylsulfanyl)-1,3,4-oxadiazole (**83e**)

Yield: 57% as a beige oil. ^1^H NMR (600 MHz, acetone-*d*_6_) δ 7.42 (d, *J* = 3.7 Hz, 1H), 6.78 (d, *J* = 3.7 Hz, 1H), 4.64 (s, 2H), 2.91 (tt, *J* = 11.1, 3.7 Hz, 1H), 2.02–1.97 (m, 2H), 1.78–1.72 (m, 2H), 1.67–1.64 (m, 1H), 1.59–1.49 (m, 2H), 1.44–1.35 (m, 2H), 1.32–1.24 (m, 1H). ^13^C NMR (151 MHz, acetone-*d*_6_) δ 171.53, 161.52, 154.40, 112.92, 112.85, 34.93, 29.81, 28.32, 25.45, 25.03. HRMS (ESI+) calcd for (C_13_H_15_N_3_O_4_S + H^+^) *m*/*z*: 310.08560 (100%); found: 310.0860 (100%).

### Synthesis of 2-Alkyl/Aryl-5-((5-nitropyridin-3-yl)methylsulfanyl)-1,3,4-oxadiazoles **82a**–**82e**

Thionyl chloride (0.52 g, 0.32 mL, 4.37 mmol) was added to a stirred solution of 3-hydroxymethyl-5-nitropyrydine **34** (0.17 g, 1.1 mmol) in CH_2_Cl_2_ (5 mL) under argon at −5 °C. The reaction mixture was removed from the cooling bath and stirred for 7 h at rt. Then the reaction mixture was concentrated under reduced pressure, and THF (5 mL) was added to the reaction residue. The resulting suspension was added to a solution of 5-substituted 1,3,4-oxadiazole-2-thiol (1.2 mmol) and Et_3_N (0.33 g, 0.46 mL, 3.3 mmol) in THF (15 mL), and the resulting mixture was stirred at rt overnight. Then, the solvent was evaporated under reduced pressure; the residue was dissolved in EtOAc (50 mL) and washed with 5% aqueous Na_2_CO_3_ (2 × 25 mL) and water (1 × 30 mL). The organic phase was dried over anhydrous sodium sulfate and concentrated under reduced pressure. The crude product was suspended in Et_2_O (15 mL) and filtered off to give final compounds in high purity.

#### 2-((5-Nitropyridin-3-yl)methylsulfanyl)-5-phenyl-1,3,4-oxadiazole (**82a**)

Yield: 63% as a yellowish solid; mp 145–147 °C. ^1^H NMR (600 MHz, DMSO-*d*_6_) δ 9.24 (d, *J* = 2.5 Hz, 1H), 9.06 (d, *J* = 2.0 Hz, 1H), 8.75 (t, *J* = 2.3 Hz, 1H), 7.91–7.89 (m, 2H), 7.61–7.51 (m, 3H), 4.71 (s, 2H). ^13^C NMR (151 MHz, DMSO-*d*_6_) δ 166.02, 163.32, 155.95, 144.60, 144.23, 135.39, 132.64, 132.22, 129.95, 126.96, 123.47, 32.54. Elem. Anal. Calcd for C_14_H_10_N_4_O_3_S: C, 53.50; H, 3.21; N, 17.83; S, 10.2. Found: C, 53.75; H, 3.26; N, 17.42. S, 10.55.

#### 2-(4-Methoxyphenyl)-5-((5-nitropyridin-3-yl)methylsulfanyl)-1,3,4-oxadiazole (**82b**)

Yield: 68% as a yellowish solid; mp 117–118 °C. ^1^H NMR (600 MHz, DMSO-*d*_6_) δ 9.24 (d, *J* = 2.5 Hz, 1H), 9.05 (d, *J* = 1.8 Hz, 1H), 8.73 (t, *J* = 2.3 Hz, 1H), 7.83 (d, *J* = 8.7 Hz, 2H), 7.07 (d, *J* = 8.9 Hz, 2H), 4.69 (s, 2H), 3.80 (s, 3H). ^13^C NMR (151 MHz, DMSO-*d*_6_) δ 165.98, 162.66, 162.46, 155.92, 144.60, 144.22, 135.43, 132.20, 128.85, 115.79, 115.40, 56.08, 32.55. Elem. Anal. Calcd for C_15_H_12_N_4_O_4_S: C, 52.32; H, 3.51; N, 16.27; S, 9.31. Found: C, 52.31; H, 3.40; N, 16.03; N, 9.7.

#### 2-(4-Chlorophenyl)-5-((5-nitropyridin-3-yl)methylsulfanyl)-1,3,4-oxadiazole (**82c**)

Yield: 70% as a white solid; mp 144–145 °C. ^1^H NMR (600 MHz, DMSO-*d*_6_) δ 9.24 (d, *J* = 2.5 Hz, 1H), 9.06 (d, *J* = 2.0 Hz, 1H), 8.74 (t, *J* = 2.2 Hz, 1H), 7.91 (d, *J* = 8.7 Hz, 2H), 7.61 (d, *J* = 8.7 Hz, 2H), 4.71 (s, 2H). ^13^C NMR (151 MHz, DMSO-*d*_6_) δ 165.28, 163.62, 155.95, 144.60, 144.24, 137.36, 135.33, 132.23, 130.12, 128.78, 122.37, 32.52. Elem. Anal. Calcd for C_14_H_9_ClN_4_O_3_S: C, 48.21; H, 2.60; N, 16.06; S, 9.19. Found: C, 48.36; H, 2.48; N, 15.92; S, 9.58.

#### 2-(4-Bromophenyl)-5-((5-nitropyridin-3-yl)methylsulfanyl)-1,3,4-oxadiazole (**82d**)

Yield: 74% as a beige solid; mp 145–147 °C. ^1^H NMR (600 MHz, DMSO-*d*_6_) δ 9.24 (d, *J* = 2.5 Hz, 1H), 9.06 (d, *J* = 2.0 Hz, 1H), 8.74 (t, *J* = 2.3 Hz, 1H), 7.83 (d, *J* = 8.5 Hz, 2H), 7.75 (d, *J* = 8.6 Hz, 2H), 4.71 (s, 2H). ^13^C NMR (151 MHz, DMSO-*d*_6_) δ 165.39, 163.64, 155.95, 144.59, 144.24, 135.31, 133.04, 132.22, 128.88, 126.25, 122.70, 32.52. Elem. Anal. Calcd for C_14_H_9_BrN_4_O_3_S: C, 42.76; H, 2.31; N, 14.25; S, 8.15. Found: C, 43.07; H, 2.23; N, 14.02; S, 8.53.

#### 2-Cyclohexyl-5-((5-nitropyridin-3-yl)methylsulfanyl)-1,3,4-oxadiazole (**82e**)

Yield: 48% as a beige solid; mp 98–99 °C. ^1^H NMR (600 MHz, DMSO-*D*_6_) δ 9.24 (d, *J* = 2.5 Hz, 1H), 9.00 (d, *J* = 1.9 Hz, 1H), 8.68 (t, *J* = 2.3 Hz, 1H), 4.61 (s, 2H), 2.92–2.80 (m, 1H), 1.94–1.84 (m, 2H), 1.71–1.60 (m, 2H), 1.63–1.53 (m, 1H), 1.47–1.37 (m, 2H), 1.37–1.24 (m, 2H), 1.25–1.09 (m, 1H). ^13^C NMR (151 MHz, DMSO-*D*_6_) δ 171.55, 162.37, 155.93, 144.55, 144.18, 135.44, 132.15, 34.66, 32.44, 29.88, 25.61, 25.13. Elem. Anal. Calcd for C_14_H_16_N_4_O_3_S: C, 52.49; H, 5.03; N, 17.49; S, 10.01. Found: 52.66; H, 4.64; N, 17.38; S, 10.38.

### In Vitro Antimycobacterial Assay

The *in vitro* antimycobacterial activities of all compounds were evaluated against *M. tuberculosis* CNCTC My 331/88 (H_37_Rv), *M*. *avium* CNCTC My 330/88, and *M. kansasii* CNCTC My 235/80 from the Czech National Collection of Type Cultures (CNCTC). The *in vitro* antimycobacterial activities of selected compounds were evaluated against clinically isolated drug-resistant strains *M.tb.* 7357/1998, *M.tb.* 234/2005, *M.tb.* 9449/2007, *M.tb.* 8666/2010, *M.tb.* Praha 1, *M.tb.* Praha 4, and *M.tb.* Praha 131. Basic suspensions of the mycobacterial strains were prepared according to a 1.0 McFarland standard. Subsequent dilutions of each strain from the basic suspension were made: *M. tuberculosis*, 10^–3^; *M. avium*, 10^–5^; and *M. kansasii*, 10^–4^. The appropriate dilutions of the strains were prepared, and 0.1 mL of the appropriate solution was added to each well of the microtiter plates containing the compounds. The activities of the compounds were determined via the micromethod for the determination of the MIC in Šula’s semisynthetic medium (SEVAC, Prague). The compounds were dissolved in DMSO and added to the medium at concentrations of 250, 125, 64, 32, 16, 8, 4, 2, 1, 0.5, 0.25, 0.125, 0.06, and 0.03 μM for *M. tuberculosis* and *M. kansasii* strains and at concentrations of 1000, 500, 250, 125, 64, 32, 16, 8, 4, 2, 1 for *M. avium* strain. The MIC values, i.e., the lowest concentration of a substance at which mycobacterial growth inhibition occurred (the concentration that inhibited >99% of the mycobacterial population), were determined after incubation at 37 °C for 14 and 21 days for *M. tuberculosis* and *M. avium* strains and for 7, 14, and 21 days for *M. kansasii* strains. Isoniazid (INH) was used as the standard drug.

### Cell Proliferation/Viability Assay

HepG2 cells were cultivated in DMEM supplemented with 10% fetal bovine serum and sodium pyruvate (1 mM). The viability assay was carried out using the CellTiter 96 Aqueous One Solution Cell Proliferation Assay (Promega) according to the manufacturer’s protocol. Briefly, the cells were seeded onto the 96-well plates at the density of 30 000 cells/well and allowed to attach for 24 h. After that, the cells were treated with the tested compounds that were predissolved in DMSO to a 1000× concentration and then dissolved in cultivation medium to 1× concentration and vehicle control (0.1% DMSO). The cells were treated for 48 h. After that, the reagent was added to the wells, and the plates were incubated in 37 °C, 5% CO_2_ for 1 h. After incubation, the absorbance was measured at 490 nm using the Synergy 2 Biotek plate reader (Biotek, Winooski, VT).

### Isolation and Characterization of *M. tuberculosis* Erdman Mutants Resistant to T6030 or T6053

3,5-Dinitrobenzylsulfanyl oxadiazole mutants of *M. tuberculosis* H37Rv were isolated from 7H9 cultures over 5 passages with increasing concentrations of T6030 or T6053 starting from 2×, 5×, and 10× MIC to final concentrations of 50× and 100× MIC. Single colonies were obtained from three independent cultures by streaking on 7H10 agar plates, and resistance to T6030 and T6053 was measured by REMA. Genomic DNA extraction was performed using the QiaAMP UCP pathogen minikit (Qiagen) as per the manufacturer’s instructions. Whole-genome sequencing was performed using Illumina technology with sequencing libraries prepared using the KAPA HyperPrep kit (Roche) and sequenced on an Illumina HiSeq 2500 instrument. All raw reads were adapter and quality trimmed with Trimmomatic v0.33^32^ and mapped onto the *M. tuberculosis* H37Rv reference genome (RefSeq no. NC_000962.3) using Bowtie2 v2.2.5.^33^ The bamleftalign program from the FreeBayes package v0.9.20–18^34^ was used to left-align indels. Reads with a mapping quality below 8 and duplicate reads were omitted.

### Variant Analysis

Variant calling was done using VarScan v2.3.9^35^ using the following cutoffs: minimum overall coverage of 10 nonduplicated reads, minimum of 5 nonduplicated reads supporting the SNP, base quality score of >15, and an SNP frequency above 30%. The rather low thresholds, especially the SNP frequency, were deliberately chosen to avoid missing potential variants in regions where alignment was difficult or in the case of a mixed population. All putative variants unique to the mutant strains were manually checked by inspecting the alignments.

### Testing Antimycobacterial Activity of the Selected Target Compounds in Ddn- and FbiC-deficient *M.tb*. H37Rv Strains

The parental *M.tb*. H37Rv strain harboring an integrative *gfp*-encoding plasmid carrying hygromycin resistance cassette, as well as the derived Ddn– (DdnL49P) and FbiC– (fbicSTOP, F_420_−) mutant strains were grown shaking (120 rpm) at 37 °C in 7H9 medium supplemented with 10% ADC, 0.05% Tween 80, and hygromycin (40 μg/mL) to early logarithmic phase. Each culture was then diluted to OD_600_ = 0.1 and 2.5 μL was spotted on 7H11-agar plates supplemented with 10% OADC and the tested compounds in concentrations corresponding to 0×, 0.5×, 1×, 3×, 10×, and 30× MIC. The plates were incubated at 37 °C, and the growth was recorded after 12 days.

### Evaluation of the Effects of the Selected Target Compounds on *M.tb*. H37Rv Lipids by [^14^C]Acetate Metabolic Labeling

*M.tb*. H37Rv was grown shaking (120 rpm) at 37 °C in 7H9 medium supplemented with 10% ADC and 0.05% Tween 80 until OD_600_ = 0.18. The culture aliquots (100 μL) were transferred to Eppendorf tubes containing the tested compounds (2 μL of the stock solutions in DMSO) to achieve the final concentrations corresponding to 10× and 100× MIC. At the same time [^14^C]acetate (specific activity: 110 mCi/mmol, American Radiolabeled Chemicals, Inc.) was added at 0.5 μCi/mL, and the cultures were incubated statically at 37 °C for 24 h. The lipids were then extracted with CHCl_3_/CH_3_OH (2:1), analyzed by TLC [Silica Gel TLC plate (Merck), CHCl_3_/CH_3_OH/H_2_O (40:8:1)] as described,^11^ and visualized by Amersham Typhoon 5 phosphorimager (GE Healthcare).

### Examination of DprE1 Target Specificity of the Selected Compounds in *M. tb*. H37Ra

The experiment was performed as recently described, using *M. tb*. H37Ra overproducing DprE1 and DprE2 proteins from *M. tb*. H37Rv.^36^ Briefly, the early log cultures of *M. tb*. H37Ra transformed with pVV2-*dprE1-dprE2* plasmid^37^ and the empty plasmid strain *M. tb*. H37Ra/pVV2 were diluted to OD_600_ = 0.5 and 0.25 with the growth medium, 7H9 medium supplemented with 10% ADC, 0.05% Tween 80, and kanamycin (20 μg/mL). Aliquots of 3 μL of each culture were spotted on 7H11-agar plates supplemented with 10% OADC, kanamycin (20 μg/mL), and the tested compounds at 0×, 0.25×, 0.5×, 1×, 3×, 10×, and 30× MIC. The plates were incubated at 37 °C, and the growth was evaluated after 12 days.

## Supporting Information Available

Synthesis and characterization of intermediate compounds **3**–**51**; Copies of NMR spectra of all final compounds, copies of HRMS spectra of fluorine-containing and/or oily compounds (PDF)
Molecular formula strings and associated biological data (CSV)

## Abbreviations

DMSO: dimethyl sulfoxide
CNCTC: Czech National Collection of Type Cultures
CL: cardiolipin
Ddn: deazaflavin-dependent nitroreductase
DprE1: decaprenylphosphoryl-β-D-ribose 2′-oxidase
FbiC: 7,8-didemethyl-8-hydroxy-5-deazariboflavin (FO) synthase
FGD1: F_420_-dependent glucose-6-phosphate dehydrogenase
HRMS: high-resolution mass spectrometry
INH: isoniazid
*m*CPBA: *meta*-chloroperoxybenzoic acid
MDR: multidrug-resistant
MIC: minimum inhibitory concentration
PE: phosphatidylethanolamine
SDS: sodium dodecyl sulfate
RIF: rifampicin
TB: tuberculosis
TMM: trehalose monomycolates
TDM: trehalose dimycolates
THF: tetrahydrofuran
TLC: thin layer chromatography
XDR: extensively drug-resistant
