# Supplementary material for: Both Nitro Groups Are Essential for High Antitubercular Activity of 3,5-Dinitrobenzylsulfanyl Tetrazoles and 1,3,4-Oxadiazoles through the Deazaflavin-Dependent Nitroreductase Activation Pathway
Source: J Med Chem. 2023 Dec 29;67(1):81–109. doi: 10.1021/acs.jmedchem.3c00925 (PMC10788908; doi:10.1021/acs.jmedchem.3c00925)

Supporting information

# Both nitro groups are essential for high antitubercular activity of 3,5-dinitrobenzylsulfanyl tetrazoles and 1,3,4-oxadiazoles through the deazaflavin-dependent nitroreductase activation pathway

*Galina Karabanovich,[a] Viktória Fabiánová,[b] Anthony Vocat,[c] Jan Dušek,[a] Lenka Valášková,[a] Jiřina Stolaříková,[d] Russell R. A. Kitson,[a] Petr Pávek,[a] Kateřina Vávrová,[a] Kamel Djaout,[e] Katarína Mikušová,[b] Alain R. Baulard,[e] Stewart T. Cole,[c] Jana Korduláková,[b] and Jaroslav Roh [a,]**

[a] Charles University, Faculty of Pharmacy in Hradec Králové, Akademika Heyrovského 1203, 50005 Hradec Králové, Czech Republic

[b] Faculty of Natural Sciences, Department of Biochemistry, Comenius University in Bratislava, Mlynská dolina, Ilkovičova 6, 842 15, Bratislava, Slovakia

[c] Global Health Institute, École Polytechnique Fédérale de Lausanne, 1015 Lausanne, Switzerland

[d] Regional Institute of Public Health, Department of Bacteriology and Mycology, Partyzánské náměstí 7, 70200 Ostrava, Czech Republic

[e] Univ. Lille, CNRS, Inserm, CHU Lille, Institut Pasteur de Lille, U1019 - UMR 9017 - CIIL - Center for Infection and Immunity of Lille, F-59000, Lille, France

*Corresponding author: Jaroslav Roh, email: jaroslav.roh@faf.cuni.cz

Content

1. Synthesis of 3-nitro-5-(trifluoromethyl)benzoic acid (**3**) S5

2. Synthesis of 3-chloro- and 3-fluoro-5-nitrobenzoic acids (**5** and **6**) S5

 2.1. Synthesis of 3-amino-5-nitrobenzoic acid (**4**) S5

 2.2. Synthesis of 3-chloro-5-nitrobenzoic acid (**5**) S6

 2.3. Synthesis of 3-fluoro-5-nitrobenzoic acid (**6**) S7

3. Synthesis of 3-bromo-5-nitrobenzoic acid (**7**) S7

4. Synthesis of 3-(methoxycarbonyl)-5-nitrobenzoic acid (**8**) S8

5. Synthesis of methyl 4-methoxy- and 2-methoxy-3,5-dinitrobenzoates **9** and **10** S9

 5.1. *Methyl 4-methoxy-3,5-dinitrobenzoate* (**9**) S9

 5.2. *Methyl 2-methoxy-3,5-dinitrobenzoate* (**10**) S10

6. Synthesis of 4-methoxy and 2-methoxy-3,5-dinitrobenzoic acids **11** and **12** S10

 6.1. *4-Methoxy-3,5-dinitrobenzoic acid* (**11**) S11

 6.2. *2-Methoxy-3,5-dinitrobenzoic acid* (**12**) S11

7. Synthesis of benzyl alcohols **13-28** S12

 7.1. *3-Nitro-5-(trifluoromethyl)benzyl alcohol* (**13**) S12

 7.2. *3-Chloro-5-nitrobenzyl alcohol* (**14**) S12

 7.3. *3-Fluoro-5-nitrobenzyl alcohol* (**15**) S13

 7.4. *3-Bromo-5-nitrobenzyl alcohol* (**16**) S13

 7.5. *Methyl 3-(hydroxymethyl)-5-nitrobenzoate* (**18**) S13

 7.6. *3,4-Dinitrobenzyl alcohol* (**23**) S14

 7.7. *2,5-Dinitrobenzyl alcohol* (**24**) S14

 7.8. *2-Nitro-5-(trifluoromethyl)benzyl alcohol* (**25**) S14

 7.9. *5-Nitro-2-(trifluoromethyl)benzyl alcohol* (**26**) S15

 7.10. *4-Methoxy-3,5-dinitrobenzyl alcohol* (**27**) S15

7.11. *2-Methoxy-3,5-dinitrobenzyl alcohol* (**28**) S15

7.12. *4-Methyl-3,5-dinitrobenzyl alcohol* (**29**) S15

7.13. *2-Methyl-3,5-dinitrobenzyl alcohol* (**30**) S16

8. Synthesis of 3-cyano-5-nitrobenzyl alcohol (**17**) S16

9. Synthesis of 3-(hydroxymethyl)-5-nitrobenzamides **19** and **20** S17

9.1. Synthesis of 3-(hydroxymethyl)-5-nitrobenzamide (**19**) S17

9.2. *N*-benzyl-3-(hydroxymethyl)-5-nitrobenzamide (**20**) S17

10. Synthesis of 3-nitro-5-(1*H*-pyrrol-1-yl)benzyl alcohol (**22**) S18

10.1. Synthesis of 3-amino-5-nitrobenzyl alcohol (**21**) S18

10.2. Synthesis of 3-nitro-5-(1*H*-pyrrol-1-yl)benzyl alcohol (**22**) S19

11. Synthesis of 3-hydroxymethyl-5-nitropyrydine (**34**) S20

11.1. Synthesis of 3-acetoxymethylpyridine-*N*-oxide (**31**) S20

11.2. Synthesis of 3-acetoxymethyl-5-nitropyridine-*N*-oxide (**32**) S20

11.3. Synthesis of 3-acetoxymethyl-5-nitropyridine (**33**) S21

11.4. Synthesis of 3-hydroxymethyl-5-nitropyrydine (**34**) S21

12. General method for the synthesis of benzyl bromides **35, 40-42, 44-51** S22

12.1. *3-Nitro-5-(trifluoromethyl)benzyl bromide* (**35**) S22

12.2. *Methyl 3-(bromomethyl)-5-nitrobenzoate* (**40**) S22

12.3. *3-(Bromomethyl)-5-nitrobenzamide* (**41**) S23

12.4. *N-Benzyl-3-(bromomethyl)-5-nitrobenzamide* (**42**) S23

12.5. *3,4-Dinitrobenzyl bromide* (**44**) S23

12.6. *2,5-Dinitrobenzyl bromide* (**45**) S24

12.7. *2-Nitro-5-(trifluoromethyl)benzyl bromide* (**46**) S24

12.8. *5-Nitro-2-(trifluoromethyl)benzyl bromide* (**47**) S25

12.9. *4-Methoxy-3,5-dinitrobenzyl bromide* (**48**) S25

12.10. *2-Methoxy-3,5-dinitrobenzyl bromide* (**49**) S25

12.11. *4-Methyl-3,5-dinitrobenzyl bromide* (**50**) S26

12.12. *2-Methyl-3,5-dinitrobenzyl bromide* (**51**) S26

13. Synthesis of benzyl chlorides **38** and **39** S26

13.1. *3-Bromo-5-nitrobenzyl chloride* (**38**) S27

13.2. *3-Cyano-5-nitrobenzyl chloride* (**39**) S27

14. General procedure for the synthesis of benzyl chlorides **36**, **37** and **43** S27

14.1. *3-Chloro-5-nitrobenzyl chloride* (**36**) S27

14.2. *3-Fluoro-5-nitrobenzyl chloride* (**37**) S28

14.3. *3-Nitro-5-(1H-pyrrol-1-yl)benzyl chloride* (**43**) S28

14. HPLC analysis S28

15. References S30

Scans of ^{1}H and ^{13}C NMR spectra of all final compounds; scans of HRMS spectra of fluorine-containing or oily compounds S32

1. Synthesis of 3-nitro-5-(trifluoromethyl)benzoic acid (3)

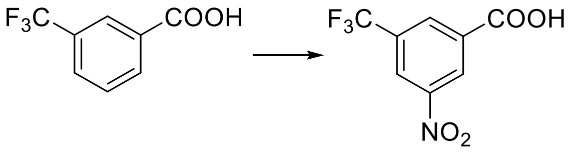

3

Fuming nitric acid (11 mL, 0.26 mol) was added dropwise to ice-cold solution of 3-trifluoromethylbenzoic acid (7 g, 0.037 mol) in 96% sulfuric acid (65 mL, 1.17 mol). The reaction mixture was slowly warmed to room temperature (rt) and stirred overnight. The reaction mixture was then carefully poured into ice (approx. 400 g). The precipitated solid was filtered, washed with water to neutral pH and dried over P_2O_5. Yield: 87% as a white solid; mp 128-129 °C (lit.[1] mp 128-130 °C). ^{1}H NMR (500 MHz, DMSO-*d*$_6$) δ 8.78 (t, *J* = 1.8 Hz, 1H), 8.70 (s, 1H), 8.51 (s, 1H). ^{13}C NMR (126 MHz, DMSO-*d*$_6$) δ 164.74, 148.95, 134.52, 131.60 (q, *J* = 3.8 Hz), 131.29 (q, *J* = 34.1 Hz), 127.86, 124.67 (q, *J* = 3.8 Hz), 123.04 (q, *J* = 273.0 Hz).

2. Synthesis of 3-chloro- and 3-fluoro-5-nitrobenzoic acids (5 and 6)

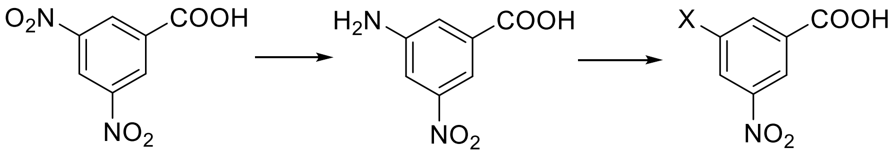

4

5: X = Cl
6: X = F

2.1. Synthesis of 3-amino-5-nitrobenzoic acid (4)

The mixture of sodium sulfide hydrate (60% Na_2S, 9 g, 0.056 mol) and ammonium chloride (7.6 g, 0.142 mol) in methanol (100 mL) was stirred for 20 minutes before 3,5-dinitrobenzoic acid (3 g, 0.014 mol) was added. The reaction mixture was heated to reflux for 17 hours. Upon completion, the reaction mixture was cooled down, filtered, and the filter cake was washed with methanol (30 mL). The filtrate was evaporated under reduced pressure. The residue was mixed with water (150 mL), stirred for 10 min and filtered to give 1.97 g (76%) of 3-amino-5-

nitrobenzoic acid **4**. The aqueous filtrate was extracted with ethyl acetate (2 × 150 mL). The organic layer was separated, washed with water (1 × 150 mL), dried over anhydrous sodium sulfate and concentrated under reduced pressure to give additional 0.42 g (16%) of product **4**. The product was used in next reactions without further purification. (For characterization propose, 3-amino-5-nitrobenzoic acid was purified using column chromatography (hexane/EtOAc/acetic acid, 30:10:1)). Yield: 92% as a yellow solid; mp 208-209 °C (lit.[2] mp 209-210 °C). ^{1}H NMR (500 MHz, DMSO-d_6) δ 13.02 (bs, 1H), 7.74 (dd, J = 2.2, 1.4 Hz, 1H), 7.56 (t, J = 2.3 Hz, 1H), 7.52 (dd, J = 2.3, 1.4 Hz, 1H), 6.09 (bs, 2H). ^{13}C NMR (126 MHz, DMSO-d_6) δ 166.62, 150.84, 149.22, 133.57, 120.45, 110.76, 110.52. Elem. Anal. Calcd. for $C_7H_6N_2O_4$: C, 46.16; H, 3.32; N, 15.38. Found: C, 46.40; H, 3.40; N, 15.27.

2.2. Synthesis of 3-chloro-5-nitrobenzoic acid (5)

3-Chloro-5-nitrobenzoic acid was prepared according to published procedure with some modifications.[3] 3-Amino-5-nitrobenzoic acid (5 g, 0.0275 mol) was dissolved in conc. aqueous HCl (300 mL) and cooled down to -5 °C. A solution of sodium nitrite (13 g, 0.188 mol) in H_2O (200 mL) was added dropwise over 45 minutes and the resulting reaction mixture was stirred for 1 hour at -5 °C. Then a suspension of CuCl (25 g, 0.25 mol) in H_2O (200 mL) was added dropwise to the reaction mixture over 1 hour and the reaction mixture was stirred at rt for 3 hours and heated to 70 °C for additional 30 minutes. Upon completion, the reaction mixture was cooled down and extracted with ethyl acetate (2 × 150 mL). The organic phase was dried over anhydrous sodium sulfate and concentrated under reduced pressure. The product was purified using column chromatography (mobile phase: hexane/EtOAc/acetic acid, 20:10:0.1). Yield: 78% as a yellow solid; mp 129-131 °C (lit.[4] mp 147-148 °C). ^{1}H NMR (600 MHz, DMSO-d_6) δ 13.92 (bs, 1H), 8.47 – 8.45 (m, 2H), 8.24 (t, J = 1.7 Hz, 1H). ^{13}C NMR (151 MHz,

DMSO-d_6) δ 164.94, 149.22, 135.30, 135.11, 134.64, 127.74, 122.94. Elem. Anal. Calcd. for $C_7H_4ClNO_4$: C, 41.71; H, 2.00; N, 6.95. Found: C, 42.10; H, 1.91; N, 6.88.

2.3. Synthesis of 3-fluoro-5-nitrobenzoic acid (6)

3-Fluoro-5-nitrobenzoic acid was prepared according to published procedure with some modifications.[5] 3-Amino-5-nitrobenzoic acid (4.59 g, 0.025 mol) was added in three portions to a solution of nitrosonium tetrafluoroborate (3.4 g, 0.029 mol) in acetonitrile (50 mL) under argon at 5 °C. The reaction mixture was then stirred at rt for 48 hours. 1,2-Dichlorobenzene (50 mL) was added to the mixture after acetonitrile was evaporated under reduced pressure, and then the mixture was heated to 170 °C for 40 minutes. After the reaction mixture was cooled down, dichloromethane (190 mL) was added, and the mixture was extracted with $NaHCO_3$ solution (2 × 150 mL). The aqueous phase was slowly acidified with HCl to pH 2 and extracted with ethyl acetate (3 × 150 mL). The organic phase was dried over anhydrous sodium sulfate and concentrated under reduced pressure. The product was purified using column chromatography (mobile phase: dichloromethane/acetic acid, 99:1). Yield: 61% as a yellow solid; mp 111-112 °C (lit.[6] mp 124-125 °C). ^{1}H NMR (600 MHz, DMSO-d_6) δ 13.89 (bs, 1H), 8.41 – 8.38 (m, 1H), 8.34 – 8.32 (m, 1H), 8.08 – 8.06 (m, 1H). ^{13}C NMR (151 MHz, DMSO-d_6) δ 165.02 (d, J = 2.6 Hz), 162.15 (d, J = 250.0 Hz), 149.38 (d, J = 9.1 Hz), 134.97 (d, J = 7.2 Hz), 122.88 (d, J = 23.1 Hz), 120.43 (d, J = 3.1 Hz), 115.79 (d, J = 27.1 Hz).

3. Synthesis of 3-bromo-5-nitrobenzoic acid (7)

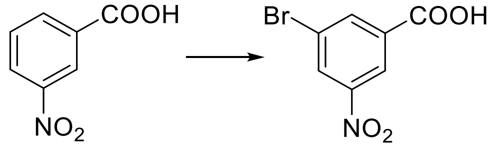

7

3-Nitrobenzoic acid (10 g, 0.06 mol) was suspended in 96% H_2SO_4 (30 mL) and heated to 60 °C. Then NBS (12.78 g, 0.072 mol, 1.2 eq) was added in 3 portions every 15 minutes. The reaction mixture was stirred at 60 °C for an additional 2 hours. Upon completion, the reaction mixture was poured into crushed ice (100 g), the precipitated solid was filtered, washed with water (60 mL) and hexane (40 mL), and finally dried over P_2O_5. Yield: 87% as a white solid; mp 158-160 °C (lit.[7] mp 160-163 °C). R_f of 3-bromo-5-nitrobenzoic acid, 0.25; R_f of 3-nitrobenzoic acid, 0.2. (mobile phase: hexane/EtOAc/acetic acid, 40:10:1). ^{1}H NMR (500 MHz, DMSO-d_6) δ 8.61 (t, J = 2.0 Hz, 1H), 8.54 (dd, J = 2.2, 1.4 Hz, 1H), 8.40 (dd, J = 1.9, 1.4 Hz, 1H). ^{13}C NMR (126 MHz, DMSO-d_6) δ 164.54, 148.89, 137.82, 134.35, 130.14, 122.98, 122.60. Elem. Anal. Calcd. for $C_7H_4BrNO_4$: C, 34.18; H, 1.64; N, 5.69. Found: C, 33.98; H, 1.38; N, 5.57.

4. Synthesis of 3-(methoxycarbonyl)-5-nitrobenzoic acid (8)

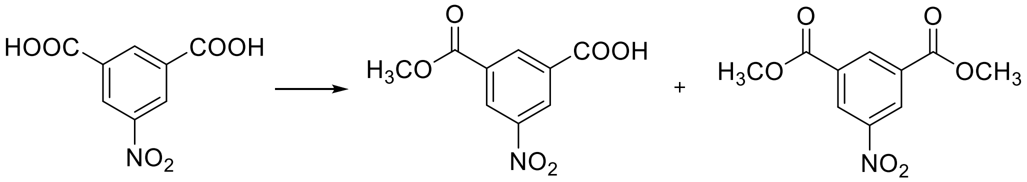

8

A solution of 5-nitroisophthalic acid (5 g, 23.68 mmol) in the mixture of THF and CH_3OH (3:1, 120 mL) with a catalytic amount of H_2SO_4 (0.5 mL) was heated to 60 °C for 48 hours. The reaction was terminated when the amount of dimethyl 5-nitroisophthalate became prevalent according to TLC. However, significant amount of unreacted 5-nitroisophthalic acid was still present in the reaction mixture. The volatiles were evaporated under reduced pressure. The residue was dissolved in EtOAc (150 mL) and washed with water (2 × 75 mL). The organic layer was separated, dried over anhydrous sodium sulfate, and concentrated under reduced pressure. 3-(Methoxycarbonyl)-5-nitrobenzoic acid (**8**) was separated using column

chromatography (mobile phase: hexane/EtOAc/acetic acid, 40:10:1). Yield: 42% as a white solid; mp 174-177 °C (lit.[8] mp 167-169 °C). ^{1}H NMR (600 MHz, DMSO-d_6) δ 8.72 (dd, J = 2.3, 1.5 Hz, 1H), 8.71 (dd, J = 2.3, 1.6 Hz, 1H), 8.66 (t, J = 1.6 Hz, 1H), 3.91 (s, 3H). ^{13}C NMR (151 MHz, DMSO-d_6) δ 165.20, 164.39, 148.76, 135.31, 133.81, 132.31, 128.11, 127.65, 53.61. Elem. Anal. Calcd. for $C_9H_7NO_6$: C, 48.01; H, 3.13; N, 6.22. Found: 48.4; H, 2.92; N, 5.86.

5. Synthesis of methyl 4-methoxy- and 2-methoxy-3,5-dinitrobenzoates 9 and 10

Dimethyl sulfate (1.66 g, 1.25 mL, 13.2 mmol) was added to a suspension of 3,5-dinitrosalicylic acid or 4-hydroxy-3,5-dinitrobenzoic acid (1 g, 4.4 mol) and potassium carbonate (0.51 g, 11 mmol) in acetone (50 mL). The reaction mixture was heated to reflux for 70 hours. Then the reaction mixture was filtered, and filtrate was concentrated under reduced pressure. The crude product was dissolved in EtOAc (50 mL) and washed with 10% Na_2CO_3 (1 × 40 mL), water (2 × 50 mL) and brine (1 × 50 mL). The organic layer was dried over anhydrous sodium sulfate and evaporated under reduced pressure. The product was purified using column chromatography (mobile phase: hexane/EtOAc, 5:1). The filter cake obtained by filtration of reaction mixture was dissolved in H_2O (40 mL), acidified to pH 2 and extracted with EtOAc (2 × 50 mL). The organic layer was dried over anhydrous sodium sulfate, the solvent was evaporated, and the by-products were separated using column chromatography (mobile phase: hexane/EtOAc/acetic acid, 30:10:1).

5.1. *Methyl 4-methoxy-3,5-dinitrobenzoate* (**9**):

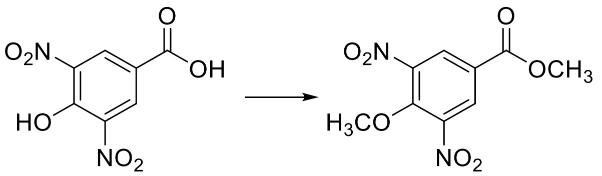

9

Yield: 64% as a yellow solid; mp 37-39 °C (lit.[9] mp 45 °C; lit.[10] mp 53-54 °C) ^{1}H NMR (500 MHz, Acetone-d_6) δ 8.73 (s, 2H), 4.13 (s, 3H), 4.00 (s, 3H). ^{13}C NMR (126 MHz, Acetone-d_6)

δ 163.76, 151.11, 145.84, 130.46, 127.11, 65.15, 53.50. Elem. Anal. Calcd. for $C_9H_8N_2O_7$: C, 42.2; H, 3.15; N, 10.94. Found: C, 42.31; H, 2.93; N, 10.73.

4-Hydroxy-3,5-dinitrobenzoic acid was recovered in 23-55% yield as yellowish solid. The by-product methyl 4-hydroxy-3,5-dinitrobenzoate was obtained in 4% yield. ^{1}H NMR (500 MHz, Acetone-d_6) δ 8.79 (s, 2H), 3.96 (s, 3H). ^{13}C NMR (126 MHz, Acetone-d_6) δ 163.43, 153.16, 139.30, 130.88, 118.40, 52.21.

5.2. *Methyl 2-methoxy-3,5-dinitrobenzoate* (**10**):

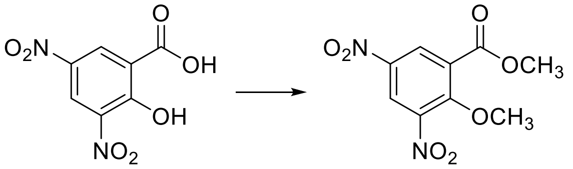

10

Yield: 63% as a yellow solid; mp 67-69 °C (lit.[9] mp 68 °C). ^{1}H NMR (300 MHz, Acetone-d_6) δ 8.89 (d, J = 2.8 Hz, 1H), 8.83 (d, J = 2.9 Hz, 1H), 4.09 (s, 3H), 4.01 (s, 3H). ^{13}C NMR (75 MHz, Acetone-d_6) δ 163.92, 157.88, 145.94, 143.04, 130.55, 128.44, 124.18, 65.11, 53.68. Elem. Anal. Calcd. for $C_9H_8N_2O_7$: C, 42.2; H, 3.15; N, 10.94. Found: C, 42.5; H, 3.07; N, 10.66.

The by-product methyl 2-hydroxy-3,5-dinitrobenzoate was isolated in 20% yield as a yellowish solid. ^{1}H NMR (500 MHz, Acetone-d_6) δ 12.48 (s, 1H), 9.00 (d, J = 2.8 Hz, 1H), 8.94 (d, J = 2.8 Hz, 1H), 4.12 (s, 3H). ^{13}C NMR (126 MHz, Acetone-d_6) δ 169.04, 159.70, 139.44, 139.09, 130.36, 126.91, 117.15, 54.42.

6. Synthesis of 4-methoxy and 2-methoxy-3,5-dinitrobenzoic acids 11 and 12

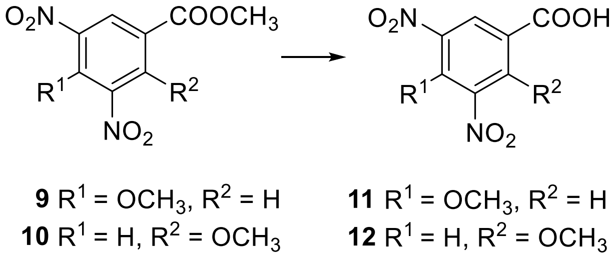

Sodium methoxide (1.33 g, 24.6 mmol) was slowly added to a solution of methyl 2- or 4-methoxy-3,5-dinitrobenzoate (1.25 g, 4.88 mmol) in methanol (40 mL). The reaction mixture was heated to reflux for 2 hours. The volatiles were evaporated under reduced pressure and the residue was dissolved in H_2O (30 mL). The aqueous phase was washed with EtOAc (2 × 30 mL) and acidified with aq. hydrochloric acid to pH 2. The aqueous layer was extracted with EtOAc (2 × 30 mL), the organic layer was separated, dried over anhydrous sodium sulfate, and concentrated under reduced pressure. The product was purified using column chromatography (mobile phase: hexane/EtOAc/acetic acid, 20:10:1).

6.1. *4-Methoxy-3,5-dinitrobenzoic acid* (**11**):

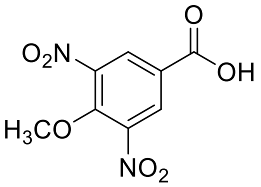

Yield: 40% as a yellow solid; mp 172-174 °C (lit.[11] mp 181 °C). ^{1}H NMR (500 MHz, DMSO-d_6) δ 8.67 (s, 2H), 4.00 (s, 3H). ^{13}C NMR (126 MHz, DMSO-d_6) δ 164.25, 149.91, 144.71, 130.27, 127.31, 64.94. Elem. Anal. Calcd. for $C_8H_6N_2O_7$: C, 39.68; H, 2.50; N, 11.57. Found: C, 39.64; H, 2.25; N, 11.49.

6.2. *2-Methoxy-3,5-dinitrobenzoic acid* (**12**):

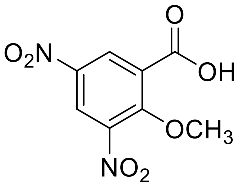

Yield: 60% as a yellow solid; mp 154-156 °C (lit.[12] mp 165-166 °C). ^{1}H NMR (500 MHz, Acetone-d_6) δ 8.89 – 8.86 (m, 2H), 4.12 (s, 3H). ^{13}C NMR (126 MHz, Acetone-d_6) δ 164.13, 158.13, 146.14, 143.06, 130.73, 128.86, 124.10, 65.13. Elem. Anal. Calcd. for $C_8H_6N_2O_7$: C, 39.68; H, 2.50; N, 11.57. Found: C, 40.06; H, 2.54; N, 11.19.

7. Synthesis of benzyl alcohols 13-28

General procedure for the reduction of carboxylic acids 3-12 to corresponding benzyl alcohols 13-16, 18, 23-28.

BH_3.THF (1M solution in THF, 90 mL, 90 mmol, 2 eq.) was added dropwise to a solution of corresponding benzoic acid **3-12** (45 mmol, 1 eq.) in THF (100 mL) under argon at -20 °C. The reaction mixture was stirred at this temperature for 1 hour, then slowly warmed to rt and stirred overnight. Then, the mixture of acetic acid and water (30 mL, 1:1) was carefully added to the reaction mixture followed by the addition of saturated aqueous $NaHCO_3$ (70 mL). The reaction mixture was extracted with EtOAc (2 × 150 mL). The organic layer was separated, washed with water (2 × 150 mL) and brine (1 × 150 mL), dried over anhydrous sodium sulfate, and concentrated under reduced pressure. The product was purified using column chromatography (mobile phase: hexane/EtOAc).

7.1. *3-Nitro-5-(trifluoromethyl)benzyl alcohol* (**13**): 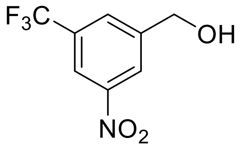

Yield: 90% as a yellow oil [13]. ^1H NMR (500 MHz, $CDCl_3$) δ 8.43 (t, J = 1.8 Hz, 1H), 8.40 (d, J = 1.8 Hz, 1H), 7.98 (t, J = 1.3 Hz, 1H), 4.91 (s, 2H), 2.27 (bs, 1H). ^{13}C NMR (126 MHz, $CDCl_3$) δ 148.45, 144.55, 132.38 (q, J = 33.9 Hz), 128.82 (q, J = 3.5 Hz), 124.34, 122.82 (q, J = 272.9 Hz), 119.60 (q, J = 3.8 Hz), 63.28.

7.2. *3-Chloro-5-nitrobenzyl alcohol* (**14**): 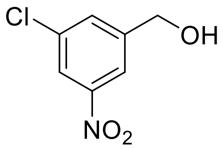

Yield: 79% as a white solid; mp 70-72 °C (lit.[14] mp 78.5 °C). ^1H NMR (500 MHz, DMSO-d_6) δ 8.15 – 8.10 (m, 2H), 7.84 – 7.79 (m, 1H), 5.64 (t, J = 5.8 Hz, 1H), 4.62 (d, J = 5.8, 2H). ^{13}C NMR (126 MHz, DMSO-d_6) δ 148.93, 147.69, 134.20, 132.68, 121.88, 119.92, 61.75.

Elem. Anal. Calcd. for $C_7H_6ClNO_3$: C, 44.82; H, 3.22; N, 7.47. Found: C, 45.21; H, 3.07; N, 7.40.

7.3. *3-Fluoro-5-nitrobenzyl alcohol* (**15**)[15]:

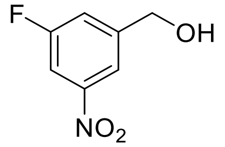

Yield: 82% as a yellow solid; mp 26-28 °C. ^{1}H NMR (500 MHz, DMSO-*d*$_6$) δ 8.07 – 8.02 (m, 1H), 7.95 - 7.93 (m, 1H), 7.66 – 7.59 (m, 1H), 5.64 (t, *J* = 5.8 Hz, 1H), 4.63 (d, *J* = 5.8 Hz, 2H). ^{13}C NMR (126 MHz, DMSO-*d*$_6$) δ 162.06 (d, *J* = 247.9 Hz), 149.04 (d, *J* = 9.5 Hz), 148.24 (d, *J* = 7.2 Hz), 119.94 (d, *J* = 21.9 Hz), 117.26 (d, *J* = 2.8 Hz), 109.59 (d, *J* = 27.1 Hz), 61.85 (d, *J* = 1.8 Hz).

7.4. *3-Bromo-5-nitrobenzyl alcohol* (**16**):

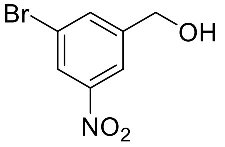

Yield: 98% as a white solid; mp 83-84 °C (lit.[16] mp 89.1-90.1 °C). ^{1}H NMR (600 MHz, DMSO-*d*$_6$) δ 8.21 (t, *J* = 2.0 Hz, 1H), 8.14 – 8.13 (m, 1H), 7.96 – 7.88 (m, 1H), 5.59 (t, *J* = 5.8 Hz, 1H), 4.61 - 4.57 (m, 2H). ^{13}C NMR (151 MHz, DMSO-*d*$_6$) δ 149.09, 147.92, 135.70, 124.73, 122.26, 120.41, 61.78. Elem. Anal. Calcd. for $C_7H_6BrNO_3$: C, 36.23; H, 2.61; N, 6.04. Found: C, 36.45; H, 2.25; N, 6.02.

7.5. *Methyl 3-(hydroxymethyl)-5-nitrobenzoate* (**18**):

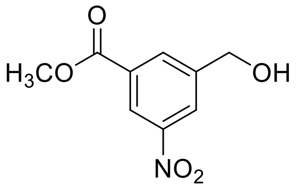

Yield: 76% as a white solid; mp 65-66 °C (lit.[17] mp 75-76 °C). ^{1}H NMR (600 MHz, DMSO-*d*$_6$) δ 8.44 (t, *J* = 2.1 Hz, 1H), 8.36 (dd, *J* = 2.4, 1.3 Hz, 1H), 8.26 – 8.25 (m, 1H), 5.63 (t, *J* = 5.8 Hz, 1H), 4.65 (d, *J* = 5.8 Hz, 2H), 3.88 (s, 3H). ^{13}C NMR (151 MHz, DMSO-*d*$_6$) δ 165.13, 148.45, 146.72, 133.03, 131.49, 125.35, 122.37, 61.94, 53.32. Elem. Anal. Calcd. for $C_9H_9NO_5$: C, 51.19; H, 4.30; N, 6.63. Found: C, 51.43; H, 3.94; N, 6.49.

7.6. *3,4-Dinitrobenzyl alcohol* (**23**): 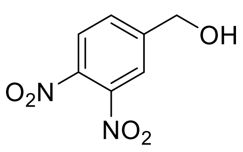

Yield: 85% as a yellow solid; mp 52-53 °C (lit.[18] mp 133-135 °C; lit.[19] mp 55.5-57 °C). ^{1}H NMR (500 MHz, DMSO-*d*$_6$) δ 8.17 (d, *J* = 8.3 Hz, 1H), 8.09 (d, *J* = 1.6 Hz, 1H), 7.86 (dd, *J* = 8.3, 1.7 Hz, 1H), 5.70 (t, *J* = 5.7 Hz, 1H), 4.65 (d, *J* = 5.8 Hz, 2H). ^{13}C NMR (126 MHz, DMSO-*d*$_6$) δ 151.41, 142.76, 140.66, 131.51, 126.13, 122.92, 61.77. Elem. Anal. Calcd. for $C_7H_6N_2O_5$: C, 42.43; H, 3.05; N, 14.14. Found: C, 42.80; H, 2.87; N, 14.01.

7.7. *2,5-Dinitrobenzyl alcohol* (**24**): 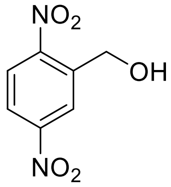

Yield: 63% as an orange solid; mp 86-87 °C (lit.[20] mp 78 °C). ^{1}H NMR (500 MHz, DMSO-*d*$_6$) δ 8.58 (d, *J* = 2.4 Hz, 1H), 8.31 (dd, *J* = 8.9, 2.5 Hz, 1H), 8.28 (d, *J* = 8.9 Hz, 1H), 5.89 (t, *J* = 5.5 Hz, 1H), 4.87 (d, *J* = 5.4 Hz, 2H). ^{13}C NMR (126 MHz, DMSO-*d*$_6$) δ 150.01, 149.93, 140.74, 126.44, 123.32, 123.18, 59.56. Elem. Anal. Calcd. for $C_7H_6N_2O_5$: C, 42.43; H, 3.05; N, 14.14. Found: C, 42.79; H, 2.93; N, 13.94.

7.8. *2-Nitro-5-(trifluoromethyl)benzyl alcohol* (**25**): 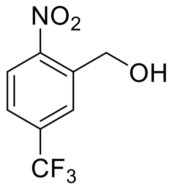

Yield: 93% as a yellow solid; mp 37-38 °C. ^{1}H NMR (500 MHz, $CDCl_3$) δ 7.90 (d, *J* = 8.3 Hz, 1H), 7.85 (d, *J* = 1.8 Hz, 1H), 7.72 (dd, *J* = 8.3, 1.8 Hz, 1H), 4.87 (s, 2H). ^{13}C NMR (126 MHz, $CDCl_3$) δ 147.02, 146.58, 130.38, 125.66 (q, *J* = 5.4 Hz), 125.34, 123.92 (q, *J* = 34.0 Hz), 121.96 (d, *J* = 273.4 Hz), 63.32.

7.9. *5-Nitro-2-(trifluoromethyl)benzyl alcohol* (**26**)[21]:

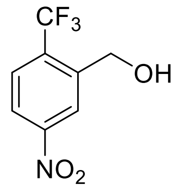

Yield: 93% as a white solid; mp 51-52 °C. ^{1}H NMR (500 MHz, $CDCl_3$) δ 8.74 – 8.58 (m, 1H), 8.24 - 8.22 (m, 1H), 7.85 (d, *J* = 8.5 Hz, 1H), 5.01 (q, *J* = 1.0 Hz, 2H). ^{13}C NMR (126 MHz, $CDCl_3$) δ 150.33, 142.32, 132.03 (q, *J* = 32.2 Hz), 127.24 (q, *J* = 5.7 Hz), 124.32, 122.97, 122.13, 60.29 (d, *J* = 3.4 Hz).

7.10. *4-Methoxy-3,5-dinitrobenzyl alcohol* (**27**):

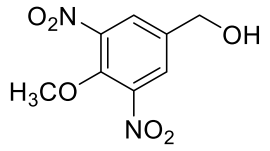

Yield: 71% as a beige solid; mp 59-60 °C (lit.[22] mp 72 °C). ^{1}H NMR (500 MHz, DMSO-d_6) δ 8.22 (s, 2H), 5.68 (t, *J* = 5.7 Hz, 1H), 4.60 (d, *J* = 4.6 Hz, 2H), 3.93 (s, 3H). ^{13}C NMR (126 MHz, DMSO-d_6) δ 145.21, 144.70, 140.82, 127.18, 64.80, 61.27. Elem. Anal. Calcd. for $C_8H_8N_2O_6$: C, 42.11; H, 3.53; N, 12.28. Found: C, 42.12; H, 3.48; N, 12.15.

7.11. *2-Methoxy-3,5-dinitrobenzyl alcohol* (**28**):

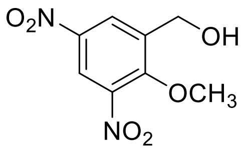

Yield: 83% as a beige solid; mp 67-68 °C. ^{1}H NMR (500 MHz, Acetone-d_6) δ 8.64 (d, *J* = 2.9, 1H), 8.62 (d, *J* = 2.8 Hz, 1H), 4.91 – 4.77 (m, 2H), 4.02 (s, 3H). ^{13}C NMR (126 MHz, Acetone-d_6) δ 155.62, 143.69, 143.62, 141.57, 127.03, 120.16, 63.40, 58.77. Elem. Anal. Calcd. for $C_8H_8N_2O_6$: C, 42.11; H, 3.53; N, 12.28. Found: C, 42.29; H, 3.48; N, 12.12.

7.12. *4-Methyl-3,5-dinitrobenzyl alcohol* (**29**):

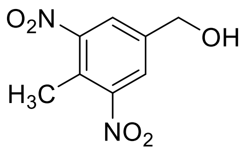

Yield: 75% as a white solid; mp 61-62 °C. ^{1}H NMR (500 MHz, DMSO-d_6) δ 8.14 (s, 2H), 5.67 (t, *J* = 5.7 Hz, 1H), 4.62 (d, *J* = 5.8 Hz, 2H), 2.43 (s, 3H). ^{13}C NMR (126 MHz, DMSO-

d$_6$) δ 151.01, 144.18, 125.25, 124.18, 61.08, 14.27. Elem. Anal. Calcd. for $C_8H_8N_2O_5$: C, 45.29; H, 3.80; N, 13.20. Found: C, 45.67; H, 3.71; N, 13.0.

7.13. *2-Methyl-3,5-dinitrobenzyl alcohol* (**30**): 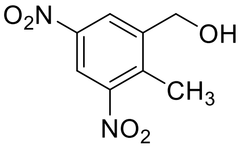

Yield: 73% as a yellow solid; mp 75-77 °C. ^{1}H NMR (500 MHz, DMSO-*d*$_6$) δ 8.58 (d, *J* = 2.5 Hz, 1H), 8.49 (d, *J* = 2.5 Hz, 1H), 5.78 (t, *J* = 5.4 Hz, 1H), 4.68 (d, *J* = 5.3 Hz, 2H), 2.37 (s, 3H). ^{13}C NMR (126 MHz, DMSO-*d*$_6$) δ 150.40, 146.21, 145.56, 136.16, 123.37, 117.38, 60.30, 14.07. Elem. Anal. Calcd. for $C_8H_8N_2O_5$: C, 45.29; H, 3.80; N, 13.20. Found: C, 45.11; H, 3.54; N, 13.1.

8. Synthesis of 3-cyano-5-nitrobenzyl alcohol (17)

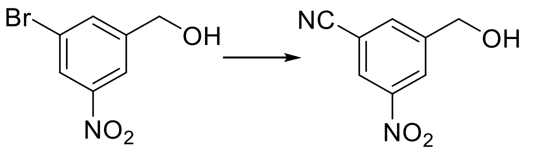

16 **17**

The mixture of 3-bromo-5-nitrobenzyl alcohol **16** (1.5 g, 6.45 mmol), $K_4[Fe(CN)_6]$.3 H_2O (0.6 g, 1.42 mmol, 0.22 eq.), Na_2CO_3 (0.68 g, 6.45 mmol, 1.0 equiv) and $Pd(OAc)_2$ (0.04 g, 0.3 mol %) in *N*,*N*-dimethylacetamide (30 mL) was heated to 120 °C under nitrogen for 6 hours. Then the reaction mixture was cooled down, diluted with EtOAc (70 mL), and filtered. The filtrate was washed with water (2 × 75 mL) and 5% aqueous NH_3 (1 × 50 mL). The organic layer was dried over anhydrous sodium sulfate, and the volatiles were evaporated under reduced pressure. The product was purified using column chromatography (mobile phase: hexane/EtOAc, 5:2). Yield: 30% as a yellowish solid; mp 96-97 °C. ^{1}H NMR (600 MHz, DMSO-*d*$_6$) δ 8.56 (t, *J* = 1.8 Hz, 1H), 8.43 – 8.40 (m, 1H), 8.15 (t, *J* = 1.3 Hz, 1H), 5.68 (t, *J* = 5.7 Hz, 1H), 4.63 (d, *J* = 5.8 Hz, 2H). ^{13}C NMR (151 MHz, DMSO-*d*$_6$) δ 148.54, 147.35, 136.21, 126.13, 125.66, 117.66,

113.07, 61.66. Elem. Anal. Calcd. for $C_8H_6N_2O_3$: C, 53.94; H, 3.39; N, 15.73. Found: C, 54.33; H, 3.29; N, 15.32.

9. Synthesis of 3-(hydroxymethyl)-5-nitrobenzamides 19 and 20

9.1. Synthesis of 3-(hydroxymethyl)-5-nitrobenzamide (19)

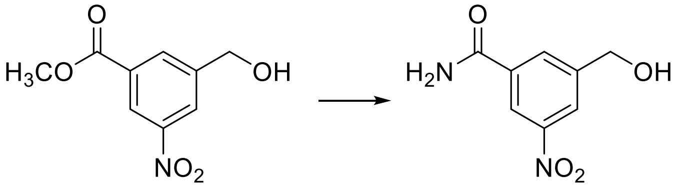

18 **19**

A solution of methyl 3-(hydroxymethyl)-5-nitrobenzoate (**18**) (1.5 g, 7.1 mmol) in methanol (30 mL) was bubbled with a flow of NH_3 for 5 minutes and then heated in an autoclave reactor to 80 °C for 32 hours. The solvent was evaporated under reduced pressure, the residue was mixed with 2M aqueous HCl (20 mL), stirred for 10 min, and filtered. The filter cake was washed with water (50 mL) and EtOAc (7 mL). Yield: 71% as a white solid; mp 167-168 °C. ^{1}H NMR (600 MHz, DMSO-d_6) δ 8.53 (dd, J = 2.3, 1.6 Hz, 1H), 8.30 – 8.25 (m, 2H), 8.22 - 8.21 (m, 1H), 7.63 (s, 1H), 5.57 (t, J = 5.6 Hz, 1H), 4.63 (d, J = 5.7 Hz, 2H). ^{13}C NMR (151 MHz, DMSO-d_6) δ 166.38, 148.38, 145.95, 136.16, 132.11, 123.64, 121.03, 62.23. Elem. Anal. Calcd. for $C_8H_8N_2O_4$: C, 48.98; H, 4.11; N, 14.28. Found: C, 48.95; H, 3.88; N, 14.34.

9.2. *N*-benzyl-3-(hydroxymethyl)-5-nitrobenzamide (20)

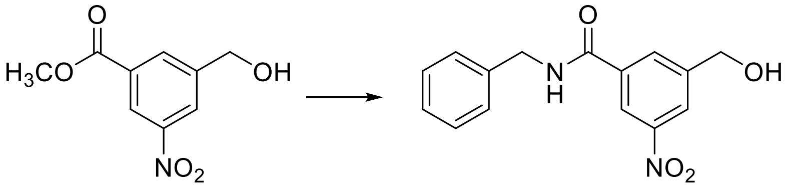

18 **20**

A solution of methyl 3-(hydroxymethyl)-5-nitrobenzoate (**18**) (1.9 g, 9 mmol) and benzylamine (9.64 g, 9.83 mL, 90 mmol) in methanol (40 mL) was heated in autoclave reactor to 120 °C for 40 hours. Upon completion, the volatiles were evaporated under reduced pressure. The residue was dissolved in EtOAc (150 mL) and washed with 2M aqueous HCl (100 mL), water (2 × 100 mL) and brine (1 × 100 mL). The organic layer was dried over anhydrous sodium sulfate and concentrated under reduced pressure. The product was purified using column chromatography (mobile phase: hexane/EtOAc, 2:1). Yield: 64% as a yellowish solid; mp 120-122 °C. ^{1}H NMR (500 MHz, DMSO-d_6) δ 9.42 (t, J = 5.9 Hz, 1H), 8.61 (t, J = 2.0 Hz, 1H), 8.32 (dd, J = 2.4, 1.4 Hz, 1H), 8.29 (t, J = 1.6 Hz, 1H), 7.34 (s, 2H), 7.33 (s, 2H), 7.28 – 7.23 (m, 1H), 5.63 (t, J = 5.7 Hz, 1H), 4.68 (d, J = 5.7 Hz, 2H), 4.51 (d, J = 5.9 Hz, 2H). ^{13}C NMR (126 MHz, DMSO-d_6) δ 164.64, 148.30, 145.99, 139.66, 135.99, 131.84, 128.80, 127.85, 127.35, 123.55, 120.75, 62.13, 43.37. Elem. Anal. Calcd. for $C_{15}H_{14}N_2O_4$: C, 62.93; H, 4.93; N, 9.79. Found: C, 62.87; H, 4.80; N, 9.59.

10. Synthesis of 3-nitro-5-(1*H*-pyrrol-1-yl)benzyl alcohol (22)

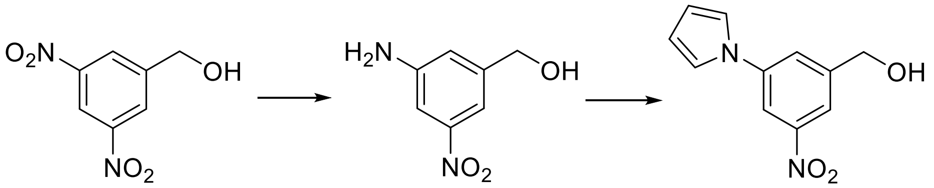

21 **22**

10.1. Synthesis of 3-amino-5-nitrobenzyl alcohol (21):

The mixture of sodium sulfide hydrate (60% Na_2S, 16 g, 0.1 mol) and ammonium chloride (13.5 g, 0.25 mol) in methanol (100 mL) was stirred for 20 minutes before 3,5-dinitrobenzyl alcohol (5 g, 0.025 mol) was added. The reaction mixture was heated to reflux for 15 hours. Upon completion, the reaction mixture was cool down, filtered, and the filter cake was washed with methanol (30 mL). The filtrate was evaporated under reduced pressure and the residue was

dissolved in EtOAc (200 mL) and washed with water (2 × 150 mL). The organic layer was separated, dried over anhydrous sodium sulfate, and concentrated under reduced pressure. The product was purified using column chromatography (mobile phase: hexane/EtOAc, 6:1). Yield: 70% as an orange solid; mp 83-84 °C (lit.[14] mp 91.5 °C). ^{1}H NMR (500 MHz, DMSO-d_6) δ 7.27 (t, J = 1.7 Hz, 1H), 7.24 (t, J = 2.2 Hz, 1H), 6.91 (s, 1H), 5.76 (s, 2H), 5.32 (t, J = 5.8 Hz, 1H), 4.45 (d, J = 5.8 Hz, 2H). ^{13}C NMR (126 MHz, DMSO-d_6) δ 150.07, 148.90, 145.36, 117.61, 107.84, 105.71, 62.35. Elem. Anal. Calcd. for $C_7H_8N_2O_3$: C, 50.00; H, 4.80; N, 16.66. Found: C, 50.18; H, 4.54; N, 16.54.

10.2. Synthesis of 3-nitro-5-(1*H*-pyrrol-1-yl)benzyl alcohol (22):

The mixture of 3-amino-5-nitrobenzyl alcohol **21** (1.5 g, 8.92 mmol) and 2,5-dimethoxytetrahydrofuran (2.35 g, 2.3 mL, 17.78 mmol) in the mixture of acetic acid (15 mL) and THF (30 mL) was heated to reflux for 24 hours. The reaction mixture was cooled down, and the solvent was evaporated under reduced pressure. The residue was disolved in EtOAc (75 mL) and washed with water (2 × 75 mL). The organic layer was separated, dried over anhydrous sodium sulfate, and concentrated under reduced pressure. The product was purified using column chromatography (mobile phase: hexane/EtOAc, 3:1). Yield: 68% as a yellow solid; mp 59-61 °C. ^{1}H NMR (500 MHz, DMSO-d_6) δ 8.21 (t, J = 2.0 Hz, 1H), 8.03 (s, 1H), 7.96 (s, 1H), 7.54 – 7.51 (m, 2H), 6.33 – 6.31 (m, 2H), 5.60 (t, J = 5.6 Hz, 1H), 4.67 (d, J = 5.8 Hz, 2H). ^{13}C NMR (126 MHz, DMSO-d_6) δ 148.99, 146.91, 140.62, 122.92, 119.52, 117.18, 112.05, 111.61, 61.94. Elem. Anal. Calcd. for $C_{11}H_{10}N_2O_3$: C, 60.55; H, 4.62; N, 12.84. Found: C, 60.76; H, 4.47; N, 12.74.

11. Synthesis of 3-hydroxymethyl-5-nitropyrydine (34)

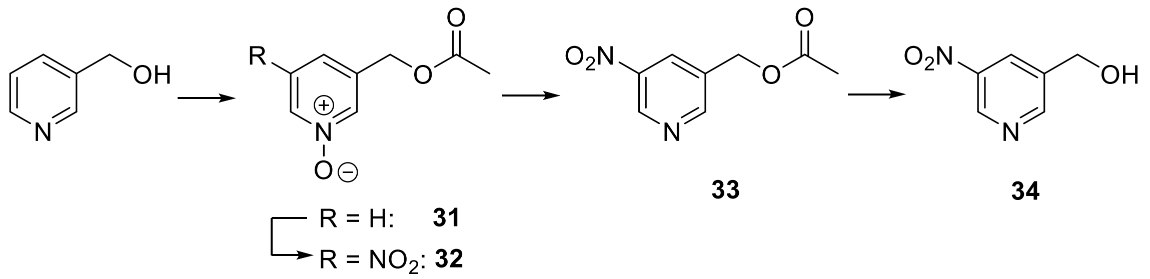

11.1. Synthesis of 3-acetoxymethylpyridine-*N*-oxide (31):

3-Hydroxymethylpyridine (2.2 g, 2 mL, 0.02 mol) was added dropwise to stirred acetic anhydride (5.4 g, 5 mL, 0.053 mol). The reaction mixture was stirred at rt for 30 min and then was heated to 65 °C for 1 hour. Then, the reaction mixture was concentrated under reduced pressure, the residue was dissolved in CH_2Cl_2 (30 mL), and MCPBA (77%, 4.93 g, 0.022 mol) was added. The reaction mixture was stirred at rt overnight and then concentrated under reduced pressure. The product was purified using column chromatography (mobile phase: $CHCl_3/CH_3OH$, 40:1). Yield: 75% as a beige solid; mp 61-63 °C (lit.[23] mp 71.9 °C). ^1H NMR (600 MHz, $CDCl_3$) δ 8.25 - 8.24 (m, 1H), 8.16 - 8.15 (m, 1H), 7.28 – 7.21 (m, 2H), 5.04 (s, 2H), 2.10 (s, 3H). ^{13}C NMR (151 MHz, $CDCl_3$) δ 170.36, 138.79, 138.65, 135.85, 125.94, 125.61, 62.27, 20.76. Elem. Anal. Calcd. for $C_8H_9NO_3$: C, 57.48; H, 5.43; N, 8.38. Found: C, 57.09; H, 5.08; N, 8.48.

11.2. Synthesis of 3-acetoxymethyl-5-nitropyridine-*N*-oxide (32)[24, 25]:

A solution of 4-nitrobenzoyl chloride (2.94 g, 0.0158 mol) in dry CH_2Cl_2 (70 mL) was added dropwise to a solution of 3-acetoxymethylpyridine-*N*-oxide **31** (2.3 g, 0.0138 mol) in dry CH_2Cl_2 (30 mL) under inert atmosphere at 0 °C followed by the addition of powdered $AgNO_3$ (3.74 g, 0.022 mol). The reaction mixture was stirred at rt for 1 hour and then heated to reflux for 48 hours. After cooling to rt, the reaction mixture was filtered, and the filter cake was washed with CH_2Cl_2 (50 mL). The organic filtrate was washed with 5% Na_2CO_3 (2 × 50 mL),

water (1 × 100 mL) and brine (1 × 100 mL), dried over anhydrous sodium sulfate and concentrated. The product was purified using column chromatography (mobile phase: $CHCl_3/CH_3OH$, 30:1). Yield: 16% as a yellowish solid; mp 100-102 °C. ^{1}H NMR (600 MHz, $CDCl_3$) δ 8.90 (t, *J* = 1.7 Hz, 1H), 8.43 (d, *J* = 1.5 Hz, 1H), 7.97 (d, *J* = 1.6 Hz, 1H), 5.13 (s, 2H), 2.15 (s, 3H). ^{13}C NMR (151 MHz, $CDCl_3$) δ 170.14, 146.82, 142.98, 136.43, 134.85, 118.84, 61.58, 20.69. Elem. Anal. Calcd. for $C_8H_8N_2O_5$: C, 45.29; H, 3.80; N, 13.20. Found: C, 44.99; H, 3.63; N, 13.58.

11.3. Synthesis of 3-acetoxymethyl-5-nitropyridine (33)[24]:

A solution of PCl_3 (3.37 g, 2.1 mL, 24.5 mmol) in CH_2Cl_2 (30 mL) was added to a stirred solution of 3-acetoxymethyl-5-nitropyridine-*N*-oxide **32** (1.74 g, 8.2 mmol) in CH_2Cl_2 (40 mL) under inert atmosphere at 0 °C. The reaction mixture was removed from cooling bath and stirred at rt for 1 hour and then poured carefully into saturated aq. Na_2CO_3 (100 mL). The organic layer was separated, washed again with saturated aq. Na_2CO_3 (1 × 75 mL) and then with water (1 × 75 mL) and brine (1 × 75 mL). The solvent was evaporated under reduced pressure to give 3-acetoxymethyl-5-nitropyridine in 70% yield as a yellow oil that was used in the next step without further purification. ^{1}H NMR (600 MHz, $CDCl_3$) δ 9.38 (d, *J* = 2.5 Hz, 1H), 8.90 (d, *J* = 2.0 Hz, 1H), 8.47 (t, *J* = 2.2 Hz, 1H), 5.23 (s, 2H), 2.14 (s, 3H).

11.4. Synthesis of 3-hydroxymethyl-5-nitropyrydine (34)[24]:

A solution of 3-acetoxymethyl-5-nitropyridine **33** (1.13 g, 5.76 mmol) and 2M sulfuric acid (10 mL) in THF (20 mL) was heated to 85 °C for 15 hours. After cooling to rt, the reaction mixture was poured into saturated aq. $NaHCO_3$ (150 mL) and extracted with EtOAc (3 × 75 mL). Organic layer was dried over sodium sulfate and evaporated *in vacuo*. 3-Hydroxymethyl-5-nitropyrydine was obtained as a yellow solid (mp 81-83 °C) in high yield (95%) and in high

purity, therefore was used in the next step without additional purification. ^{1}H NMR (600 MHz, $CDCl_3$) δ 9.30 (d, J = 2.5 Hz, 1H), 8.85 (d, J = 1.9 Hz, 1H), 8.52 (d, J = 2.4 Hz, 1H), 4.89 (s, 2H). ^{13}C NMR (151 MHz, $CDCl_3$) δ 153.15, 144.56, 143.91, 137.87, 129.42, 61.45. Elem. Anal. Calcd. for $C_6H_6N_2O_3$: C, 46.76; H, 3.92; N, 18.18. Found: C, 46.82; H, 3.78; N, 18.57.

12. General method for the synthesis of benzyl bromides 35, 40-42, 44-51

Solution of PPh_3 (3.27 g, 12.47 mmol) in dry CH_2Cl_2 (60 mL) was added dropwise to a stirred solution of corresponding benzyl alcohol **13**, **18-20**, **23-30** (6.16 mmol) and NBS (2.22 g, 12.47 mmol) in dry CH_2Cl_2 (30 mL) under argon at 0 °C. The reaction mixture was stirred for 15 min, then warmed to rt and stirred at rt upon complete consumption of starting material as determined by TLC. The volatiles were evaporated, and the product was purified using column chromatography.

12.1. *3-Nitro-5-(trifluoromethyl)benzyl bromide* (**35**)[26]:

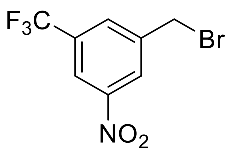

The reaction was completed in 1 hour. The product was purified using column chromatography (mobile phase: hexane/EtOAc, 10:1). Yield: 72% as a brownish oil. ^{1}H NMR (500 MHz, $CDCl_3$) δ 8.48 – 8.46 (m, 1H), 8.45 – 8.43 (m, 1H), 8.02 – 7.97 (m, 1H), 4.59 (s, 2H). ^{13}C NMR (126 MHz, $CDCl_3$) δ 148.56, 141.27, 132.93 (q, J = 34.4 Hz), 131.40 (q, J = 3.5 Hz), 127.00, 122.52 (q, J = 273.3 Hz), 120.54 (q, J = 3.8 Hz), 29.80.

12.2. *Methyl 3-(bromomethyl)-5-nitrobenzoate* (**40**):

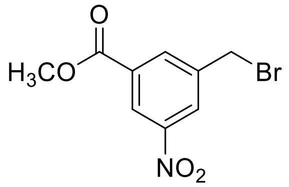

The reaction was completed in 30 min. The product was purified using column chromatography (mobile phase: hexane/EtOAc, 5:1). Yield: 78% as a white solid; mp 102-103 °C. ^{1}H NMR (600 MHz, DMSO-d_6) δ 8.56 (s, 1H), 8.49 (s, 1H), 8.41 (s, 1H), 4.90 (s, 2H), 3.90

(s, 3H). ^{13}C NMR (151 MHz, DMSO-*d$_6$*) δ 164.70, 148.62, 141.99, 136.23, 132.17, 128.56, 123.80, 53.48, 31.96. Elem. Anal. Calcd. for $C_9H_8BrNO_4$: C, 39.44; H, 2.94; N, 5.11. Found: C, 39.55; H, 2.79; N, 5.03.

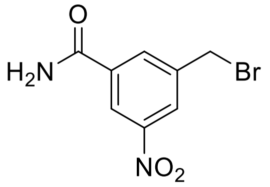

12.3. *3-(Bromomethyl)-5-nitrobenzamide* (**41**):

The reaction was completed in 2 hours. The product was purified using column chromatography (mobile phase: hexane/EtOAc, 3:2). Yield: 40% as a white solid; mp 163-164 °C. ^{1}H NMR (600 MHz, DMSO-*d$_6$*) δ 8.61 (t, *J* = 1.8 Hz, 1H), 8.45 (t, *J* = 1.9 Hz, 1H), 8.36 (t, *J* = 1.6 Hz, 1H), 8.34 (s, 1H), 7.70 (s, 1H), 4.85 (s, 2H). ^{13}C NMR (151 MHz, DMSO-*d$_6$*) δ 165.82, 148.46, 141.21, 136.80, 135.25, 126.79, 122.39, 32.41. Elem. Anal. Calcd. for $C_8H_7BrN_2O_3$: C, 37.09; H, 2.72; N, 10.81. Found: C, 37.48; H, 2.60; N, 10.64.

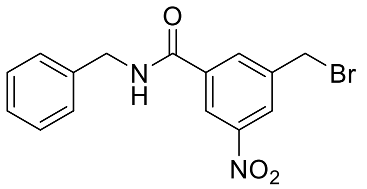

12.4. *N-Benzyl-3-(bromomethyl)-5-nitrobenzamide* (**42**):

The reaction was completed in 2 hours. The product was purified using column chromatography (mobile phase: hexane/EtOAc, 4:1). Yield: 94% as a white solid; mp 138-139 °C. ^{1}H NMR (600 MHz, DMSO-*d$_6$*) δ 9.43 (t, *J* = 5.9 Hz, 1H), 8.65 (dd, *J* = 2.2, 1.6 Hz, 1H), 8.47 (dd, *J* = 2.2, 1.6 Hz, 1H), 8.39 (t, *J* = 1.6 Hz, 1H), 7.32 – 7.29 (m, 4H), 7.26 – 7.19 (m, 1H), 4.86 (s, 2H), 4.48 (d, *J* = 5.9 Hz, 2H). ^{13}C NMR (151 MHz, DMSO-*d$_6$*) δ 164.20, 148.47, 141.31, 139.61, 136.68, 135.12, 128.90, 127.98, 127.48, 126.80, 122.20, 43.50, 32.39. Elem. Anal. Calcd. for $C_{15}H_{13}BrN_2O_3$: C, 51.6; H, 3.75; N, 8.02. Found: C, 51.4; H, 3.54; N, 7.87.

12.5. *3,4-Dinitrobenzyl bromide* (**44**)[27]: 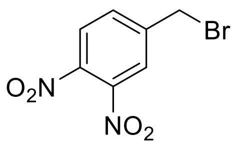

The reaction was completed in 1 hour. The product was purified using column chromatography (mobile phase: hexane/EtOAc, 3:1). Yield: 92% as a yellow solid; mp 45-46 °C. ^{1}H NMR (600 MHz, DMSO-d_6) δ 8.31 (d, J = 2.0 Hz, 1H), 8.21 (d, J = 8.3 Hz, 1H), 8.00 (dd, J = 8.4, 1.9 Hz, 1H), 4.82 (s, 2H). ^{13}C NMR (151 MHz, DMSO-d_6) δ 146.14, 142.66, 141.61, 135.49, 126.86, 126.47, 31.05. Anal. Calcd. for $C_7H_5BrN_2O_4$: C, 32.21; H, 1.93; N, 10.73. Found: C, 32.12; H, 1.65; N, 10.54.

12.6. *2,5-Dinitrobenzyl bromide* (**45**): 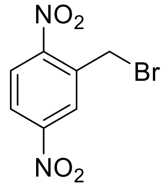

The reaction was completed in 1 hour. The product was purified using column chromatography (mobile phase: hexane/EtOAc, 7:1). Yield: 93% as a beige solid; mp 74-75 °C. ^{1}H NMR (600 MHz, DMSO-d_6) δ 8.64 (d, J = 2.5 Hz, 1H), 8.36 (dd, J = 8.9, 2.6 Hz, 1H), 8.25 (d, J = 9.0 Hz, 1H), 4.98 (s, 2H). ^{13}C NMR (151 MHz, DMSO-d_6) δ 151.77, 149.94, 134.72, 127.95, 127.58, 125.64, 28.53. Elem. Anal. Calcd. for $C_7H_5BrN_2O_4$: C, 32.21; H, 1.93; N, 10.73. Found: C, 32.36; H, 1.67; N, 10.62.

12.7. *2-Nitro-5-(trifluoromethyl)benzyl bromide* (**46**)[28]: 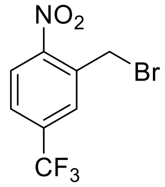

The reaction was completed in 1 hour. The product was purified using column chromatography (mobile phase: hexane/EtOAc, 10:1). Yield: 78% as a yellow oil. ^{1}H NMR (600 MHz, $CDCl_3$) δ 7.87 (d, J = 8.3 Hz, 1H), 7.83 (d, J = 2.0 Hz, 1H), 7.73 (dd, J = 8.3, 2.0

Hz, 1H), 4.51 (s, 2H). NMR (151 MHz, $CDCl_3$) δ 147.59, 143.29, 133.46, 128.60 (q, J = 5.3 Hz), 125.81, 124.45 (d, J = 34.6 Hz), 121.74 (d, J = 273.6 Hz), 29.90.

12.8. *5-Nitro-2-(trifluoromethyl)benzyl bromide* (**47**): 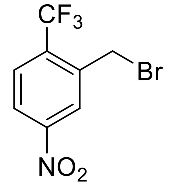

The reaction was completed in 1 hour. The product was purified using column chromatography (mobile phase: hexane/EtOAc, 30:1). Yield: 80% as a white solid; mp 33-34 °C. ^{1}H NMR (500 MHz, $CDCl_3$) δ 8.49 (d, J = 2.2 Hz, 1H), 8.27 (ddd, J = 8.6, 2.3, 1.0 Hz, 1H), 7.88 (d, J = 8.6 Hz, 1H), 4.69 (s, 2H). ^{13}C NMR (126 MHz, $CDCl_3$) δ 150.14, 138.77, 133.42 (d, J = 31.6 Hz), 127.92 (q, J = 5.5 Hz), 127.51, 123.24, 126.86 – 119.55 (m), 26.57 (q, J = 2.8 Hz).

12.9. *4-Methoxy-3,5-dinitrobenzyl bromide* (**48**): 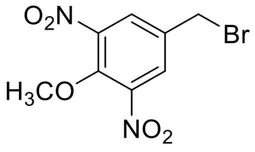

The reaction was completed in 1 hour. The product was purified using column chromatography (mobile phase: hexane/EtOAc, 4:1). Yield: 80% as a yellow solid; mp 68-69 °C. ^{1}H NMR (500 MHz, Acetone-d_6) δ 8.39 (s, 2H), 4.84 (s, 2H), 4.08 (s, 3H). ^{13}C NMR (126 MHz, Acetone-d_6) δ 147.40, 145.99, 136.64, 130.49, 65.03, 30.54. Elem. Anal. Calc. for $C_8H_7BrN_2O_5$: C, 33.01; H, 2.42; N, 9.62. Found: C, 33.38; H, 2.34; N, 9.33.

12.10. *2-Methoxy-3,5-dinitrobenzyl bromide* (**49**): 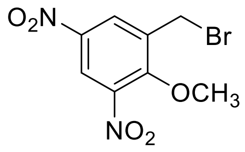

The reaction was completed in 3 hours. The product was purified using column chromatography (mobile phase: hexane/EtOAc, 10:1). Yield: 70% as a beige solid; mp 52-54 °C. ^{1}H NMR (500 MHz, $CDCl_3$) δ 8.69 (d, J = 2.8 Hz, 1H), 8.51 (d, J = 2.8 Hz, 1H), 4.59 (s, 2H), 4.12 (s, 3H). ^{13}C NMR (126 MHz, $CDCl_3$) δ 156.56, 142.59, 142.20, 136.07, 129.63,

121.67, 63.42, 24.76. Elem. Anal. Calc. for $C_8H_7BrN_2O_5$: C, 33.01; H, 2.42; N, 9.62. Found: C, 33.26; H, 2.34; N, 9.44.

12.11. *4-Methyl-3,5-dinitrobenzyl bromide* (**50**): 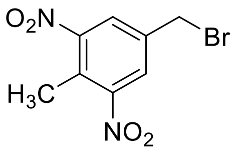

The reaction was completed in 4 hours. The product was purified using column chromatography (mobile phase: hexane/EtOAc, 5:1). Yield: 79% as a beige solid; mp 63-64 °C. ^{1}H NMR (500 MHz, $CDCl_3$) δ 8.03 (s, 2H), 4.50 (s, 2H), 2.57 (s, 3H). ^{13}C NMR (126 MHz, $CDCl_3$) δ 151.62, 138.27, 127.66, 127.12, 29.06, 14.81. Elem. Anal. Calc. for $C_8H_7BrN_2O_4$: C, 34.93; H, 2.57; N, 10.18. Found: C, 34.90; H, 2.27; N, 10.40.

12.12. *2-Methyl-3,5-dinitrobenzyl bromide* (**51**): 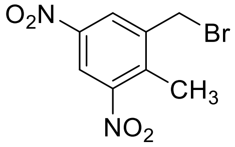

The reaction was completed in 12 hours. The product was purified using column chromatography (mobile phase: hexane/EtOAc, 5:1). Yield: 85% as a yellow solid; mp 39-40 °C. ^{1}H NMR (500 MHz, $CDCl_3$) δ 8.60 (d, J = 2.4 Hz, 1H), 8.43 (d, J = 2.4 Hz, 1H), 4.60 (s, 2H), 2.64 (s, 3H). ^{13}C NMR (126 MHz, $CDCl_3$) δ 151.31, 145.70, 140.79, 138.63, 127.56, 119.38, 28.83, 15.21. Elem. Anal. Calc. for $C_8H_7BrN_2O_4$: C, 34.93; H, 2.57; N, 10.18. Found: C, 35.16; H, 2.36; N, 10.34.

13. Synthesis of benzyl chlorides 38 and 39

PCl_5 (46 mmol) was added to a solution of benzyl alcohols **16** or **17** (5 g, 23 mmol) in chloroform (100 mL) at 0 °C. The resulting solution was warmed to rt and then heated to reflux for 8 hours. Upon completion, the reaction mixture was poured into crashed ice (approx. 125 g) and extracted with CH_2Cl_2 (2 × 100 mL). The combined organic extract was washed with water (2 × 70 mL), concentrated aq. $NaHCO_3$ (2 × 70 mL), and brine (1 × 70 mL). The organic

layer was dried over anhydrous sodium sulfate and concentrated under reduced pressure. The product was purified using column chromatography (mobile phase: hexane/EtOAc, 7:1 – 3:1).

13.1. *3-Bromo-5-nitrobenzyl chloride* (**38**): 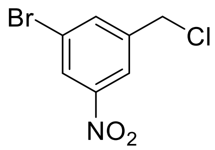

Yield: 70% as a white solid; mp 66-67 °C (lit.[16] mp 74-75.3 °C). ^{1}H NMR (600 MHz, DMSO-*d*$_6$) δ 8.32 (t, *J* = 1.9 Hz, 1H), 8.30 (t, *J* = 1.8 Hz, 1H), 8.12 (t, *J* = 1.7 Hz, 1H), 4.87 (s, 2H). ^{13}C NMR (151 MHz, DMSO-*D*$_6$) δ 149.16, 142.42, 138.42, 126.47, 123.30, 122.60, 44.17. Anal calcd for $C_7H_5BrClNO_2$: C, 33.57; H, 2.01; N, 5.59. Found: C, 33.55; H, 1.67; N, 5.50.

13.2. *3-Cyano-5-nitrobenzyl chloride* (**39**): 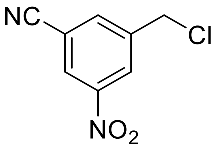

Yield: 89% as a white solid; mp 89-91 °C. ^{1}H NMR (600 MHz, DMSO-*d*$_6$) δ 8.69 – 8.67 (m, 1H), 8.60 (t, *J* = 2.0 Hz, 1H), 8.37 (t, *J* = 1.6 Hz, 1H), 4.91 (s, 2H). ^{13}C NMR (151 MHz, DMSO-*d*$_6$) δ 148.65, 141.96, 139.02, 128.56, 127.77, 117.23, 113.71, 43.93. Elem. Anal. Calc. for $C_8H_5ClN_2O_2$: C, 48.88; H, 2.56; N, 14.25. Found: C, 48.65; H, 2.28; N, 14.30.

14. Synthesis of benzyl chlorides 36, 37 and 43

Thionyl chloride (3 mL, 4.92 g, 41.35 mmol) in CH_2Cl_2 (25 mL) was added dropwise to a stirred solution of benzyl alcohol **14**, **15** or **22** (3 g, 13.75 mmol) and Et_3N (5.8 mL, 4.18 g, 41.35 mmol) in CH_2Cl_2 (50 mL) under argon at 0 °C. The reaction mixture was stirred for 20 min at 0 °C and then at rt for additional 3 hours. The reaction mixture was washed with water (2 × 70 mL) and brine (1 × 70 mL), dried over anhydrous sodium sulfate, concentrated under reduced pressure and the product was purified using column chromatography (mobile phase: hexane/EtOAc, 5:1).

14.1. *3-Chloro-5-nitrobenzyl chloride* (**36**): 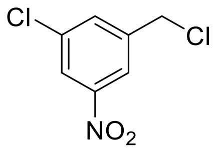

Yield: 74% as a yellow solid; mp 54-56 °C. ^{1}H NMR (600 MHz, DMSO-d_6) δ 8.27 (dd, *J* = 2.1, 1.5 Hz, 1H), 8.21 (t, *J* = 2.0 Hz, 1H), 7.99 (dd, *J* = 2.0, 1.4 Hz, 1H), 4.88 (s, 2H). ^{13}C NMR (151 MHz, DMSO-d_6) δ 149.15, 142.27, 135.56, 134.69, 123.74, 122.93, 44.22. Elem. Anal. Calc. for $C_7H_5Cl_2NO_2$: C, 40.81; H, 2.45; N, 6.80. Found: C, 41.19; H, 2.26; N, 6.71.

14.2. *3-Fluoro-5-nitrobenzyl chloride* (**37**)[15]: 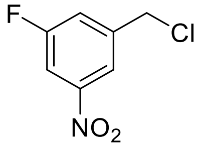

Yield: 75% as a yellow oil. ^{1}H NMR (600 MHz, $CDCl_3$) δ 8.09 – 8.05 (m, 1H), 7.88 (dt, *J* = 8.0, 2.2 Hz, 1H), 7.50 – 7.45 (m, 1H), 4.63 (s, 2H). ^{13}C NMR (151 MHz, $CDCl_3$) δ 162.43 (d, *J* = 252.3 Hz), 149.32 (d, *J* = 8,64 Hz), 141.41 (d, *J* = 7.8 Hz), 121.73 (d, J = 21,72 Hz), 119.28 (d, *J* = 3.4 Hz), 111.30 (d, *J* = 26.6 Hz), 44.07.

14.3. *3-Nitro-5-(1H-pyrrol-1-yl)benzyl chloride* (**43**): 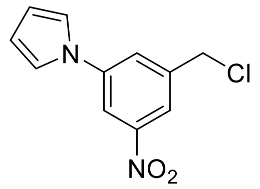

Yield: 79% as a yellow solid; mp 75-77 °C. ^{1}H NMR (500 MHz, DMSO-d_6) δ 8.33 (t, *J* = 2.1 Hz, 1H), 8.18 (t, *J* = 1.9 Hz, 1H), 8.16 (t, *J* = 1.7 Hz, 1H), 7.57 (t, *J* = 2.1 Hz, 2H), 6.34 (t, *J* = 2.2 Hz, 2H), 4.93 (s, 2H). ^{13}C NMR (126 MHz, DMSO-d_6) δ 149.12, 141.46, 140.94, 125.49, 119.82, 119.55, 113.45, 111.85, 44.50. Elem. Anal. Calc. for $C_{11}H_9ClN_2O_2$: C, 55.83; H, 3.83; N, 11.84. Found: C, 55.82; H, 3.77; N, 11.76

HPLC analysis. Compounds **67a-e** and **69a-e** were tested for purity by HPLC on a Shimadzu Prominence HPLC instrument (Shimadzu, Japan) equipped with an SPD-M20A diode array detector, using a LiChroCART 250-4 column packed with LiChrospher 100 RP-18, 5 µm

particles (Merck, Darmstadt, Germany). The mobile phase gradient for the analysis of all compounds is shown in the following table. All tested compounds were dissolved in acetonitrile in a concentration of 10 µg/mL. The flow rate was 1 mL/min at 25°C and the injection volume was 10 µL.

Time (min)	% of H_2O	% of acetonitrile
0	50	50
2	50	50
7	10	90
12	10	90
15	50	50
20	50	50

References

1. Karabanovich, G.; Nemecek, J.; Valaskova, L.; Carazo, A.; Konecna, K.; Stolarikova, J.; Hrabalek, A.; Pavlis, O.; Pavek, P.; Vavrova, K.; Roh, J.; Klimesova, V. S-substituted 3,5-dinitrophenyl 1,3,4-oxadiazole-2-thiols and tetrazole-5-thiols as highly efficient antitubercular agents. *Eur. J. Med. Chem.* **2017,** *126*, 369-383.
2. Piskov, V. B.; Kasperovich, V. P.; Yakovleva, L. M. Synthesis of Δ2-imidazolines in ethylene gylcol. *Chem. Heterocycl. Comp.* **1976,** *12*, 917-923.
3. Donghi, M.; Ferrara, M.; Koch, U.; Narjes, F.; Ontoria Ontoria, J. M.; Summa, V. Quinazoline Derivatives as Antiviral Agents. WO/2007/028789, 2007.
4. Kalfus, K.; Kroupa, J.; Večeřa, M.; Exner, O. Additivity of substituent effects in meta- and para-substituted benzoic acids. *Collect. Czech. Chem. Commun.* **1975,** *40*, 3009-3019.
5. Betts, M. J.; Davies, G. M.; Swain, M. L. Antibiotic compounds. US2005209212 (A1), 2005.
6. Adams, E. S.; Rinehart, K. L. Directed Biosynthesis of 5''-Fluorop Actamycin in Streptomyces pactum. *J. Antibiot.* **1994,** *47*, 1456-1465.
7. Reuter, R.; Wegner, H. A. Switchable 3D networks by light controlled π-stacking of azobenzene macrocycles. *Chem. Comm.* **2013,** *49*, 146-148.
8. Kahnberg, P.; Lager, E.; Rosenberg, C.; Schougaard, J.; Camet, L.; Sterner, O.; Oestergaard Nielsen, E.; Nielsen, M.; Liljefors, T. Refinement and Evaluation of a Pharmacophore Model for Flavone Derivatives Binding to the Benzodiazepine Site of the GABAa-Receptor. *J. Med. Chem.* **2002,** *45*, 4188–4201.
9. Crampton, M. R.; Khan, H. A. The stabilities of Meisenheimer complexes. Part I. Adducts from sodium methoxide and highly activated anisoles in methanol. *J. Chem. Soc., Perkin trans. 2* **1972,** 1173-1177.
10. Chandler, W. D.; Smith, W. M.; Moir, R. Y. The Principal Conformations of some Ortho-Substituted Diphenyl Ethers. *Can. J. Chem.* **1964,** *42*, 2549-2559.
11. Ullmann, F.; Engi, G.; Wosnessensky, N.; Kuhn, E.; Herre, E. Studien über aromatische Verbindungen mit labilem Halogen. *Justus Liebigs Ann. Chem.* **1909,** *366*, 79-118.
12. Hirwe, N. W.; Gavanker, K. D. Derivatives of salicylic acid. *Proc. Indian Acad. Sci. - Section A* **1937,** *5*, 377-380.
13. Karabanovich, G.; Zemanova, J.; Smutny, T.; Szekely, R.; Sarkan, M.; Centarova, I.; Vocat, A.; Pavkova, I.; Conka, P.; Nemecek, J.; Stolarikova, J.; Vejsova, M.; Vavrova, K.; Klimesova, V.; Hrabalek, A.; Pavek, P.; Cole, S. T.; Mikusova, K.; Roh, J. Development of 3,5-Dinitrobenzylsulfanyl-1,3,4-oxadiazoles and Thiadiazoles as Selective Antitubercular Agents Active Against Replicating and Nonreplicating Mycobacterium tuberculosis. *J. Med. Chem.* **2016,** *59*, 2362-2380.
14. Meindl, W. R.; Von Angerer, E.; Schoenenberger, H.; Ruckdeschel, G. Benzylamines: synthesis and evaluation of antimycobacterial properties. *J. Med. Chem.* **1984,** *27*, 1111-1118.
15. Wales, S. M.; Rivinoja, D. J.; Gardiner, M. G.; Bird, M. J.; Meyer, A. G.; Ryan, J. H.; Hyland, C. J. T. Benzoazepine-Fused Isoindolines via Intramolecular (3 + 2)-Cycloadditions of Azomethine Ylides with Dinitroarenes. *Org. Lett.* **2019,** *21*, 4703-4708.

16. Lahiri, S.; Thompson, J. L.; Moore, J. S. Solvophobically Driven π-Stacking of Phenylene Ethynylene Macrocycles and Oligomers. *J. Am. Chem. Soc.* **2000,** *122*, 11315-11319.

17. Watzke, A.; Gutierrez-Rodriguez, M.; Köhn, M.; Wacker, R.; Schroeder, H.; Breinbauer, R.; Kuhlmann, J.; Alexandrov, K.; Niemeyer, C. M.; Goody, R. S.; Waldmann, H. A generic building block for C- and N-terminal protein-labeling and protein-immobilization. *Bioorg. Med. Chem.* **2006,** *14*, 6288-6306.

18. Chen, M. H.; Iakovleva, E.; Kesten, S.; Magano, J.; Rodriguez, D.; Sexton, K. E.; Zhang, J.; Lee, H. T. A Convenient Reduction of Highly Functionalized Aromatic Carboxylic Acids to Alcohols with Borane-THF and Boron Trifluoride-Etherate. *Org. Prep. Proced. Int.* **2002,** *34*, 665-670.

19. Fuchs, R.; Carlton, D. M. Multiple Substituent Effects in the Solvolysis and Thiosulfate Reactions of 4-Substituted α-Chloro-3-nitrotoluenes. *J. Org. Chem.* **1962,** *27*, 1520-1523.

20. Martínez-Viturro, C. M.; Domínguez, D. Synthesis of the antitumoural agent batracylin and related isoindolo[1,2-b]quinazolin-12(10H)-ones. *Tetrahedron Lett.* **2007,** *48*, 1023-1026.

21. Liqiang, C.; Teng, A.; Swati, M. Therapeutic Compounds. US2016376238A1, 2016.

22. Misra, G. S.; Srivastava, S. B. Crossed Cannizzaro Reaction. II. *J. Prakt. Chem.* **1958,** *6*, 170-173.

23. Nagano, H. N., Y.; Hamana, M. The mechanism of the reaction of nicotinic acid 1 oxide with acetic anhydride. *Chem. Pharm. Bull.* **1987,** *35*, 4068-4077.

24. Ashimori, A.; Ono, T.; Uchida, T.; Ohtaki, Y.; Fukaya, C.; Watanabe, M.; Yokoyama, K. Novel 1,4-dihydropyridine calcium antagonists. I. Synthesis and hypotensive activity of 4-(substituted pyridyl)-1,4-dihydropyridine derivatives. *Chem. Pharm. Bull.* **1990,** *38*, 2446-2458.

25. Nakadate, M.; Takano, Y.; Hirayama, T.; Sakaizawa, S.; Hirano, T.; Okamoto, K.; Hirao, K.; Kawamura, T.; Kimura, M. Janovsky Reaction of Nitropyridines. II. Preparation of 5-Nitronicotinic Acid and its Related Compounds. *Chem. Pharm. Bull.* **1965,** *13*, 113-118.

26. Karabanovich, G.; Dušek, J.; Savková, K.; Pavliš, O.; Pávková, I.; Korábečný, J.; Kučera, T.; Kočová Vlčková, H.; Huszár, S.; Konyariková, Z.; Konečná, K.; Jand'ourek, O.; Stolaříková, J.; Korduláková, J.; Vávrová, K.; Pávek, P.; Klimešová, V.; Hrabálek, A.; Mikušová, K.; Roh, J. Development of 3,5-Dinitrophenyl-Containing 1,2,4-Triazoles and Their Trifluoromethyl Analogues as Highly Efficient Antitubercular Agents Inhibiting Decaprenylphosphoryl-β-d-ribofuranose 2′-Oxidase. *J. Med. Chem.* **2019,** *62*, 8115-8139.

27. Lecarme, L.; Prado, E.; De Rache, A.; Nicolau-Travers, M.-L.; Bonnet, R.; Heyden, A. v. D.; Philouze, C.; Gomez, D.; Mergny, J.-L.; Jamet, H.; Defrancq, E.; Jarjayes, O.; Thomas, F. Interaction of Polycationic Ni(II)-Salophen Complexes with G-Quadruplex DNA. *Inorg. Chem.* **2014,** *53*, 12519-12531.

28. McAllister, L. A.; Bechle, B. M.; Dounay, A. B.; Evrard, E.; Gan, X.; Ghosh, S.; Kim, J.-Y.; Parikh, V. D.; Tuttle, J. B.; Verhoest, P. R. A General Strategy for the Synthesis of Cyclic N-Aryl Hydroxamic Acids via Partial Nitro Group Reduction. **2011,** *76*, 3484-3497.

5-((3-Nitro-5-(trifluoromethyl)benzyl)sulfanyl)-1-phenyl-1*H*-tetrazole (**52a**): ^{1}H NMR (600 MHz, DMSO-d_6)

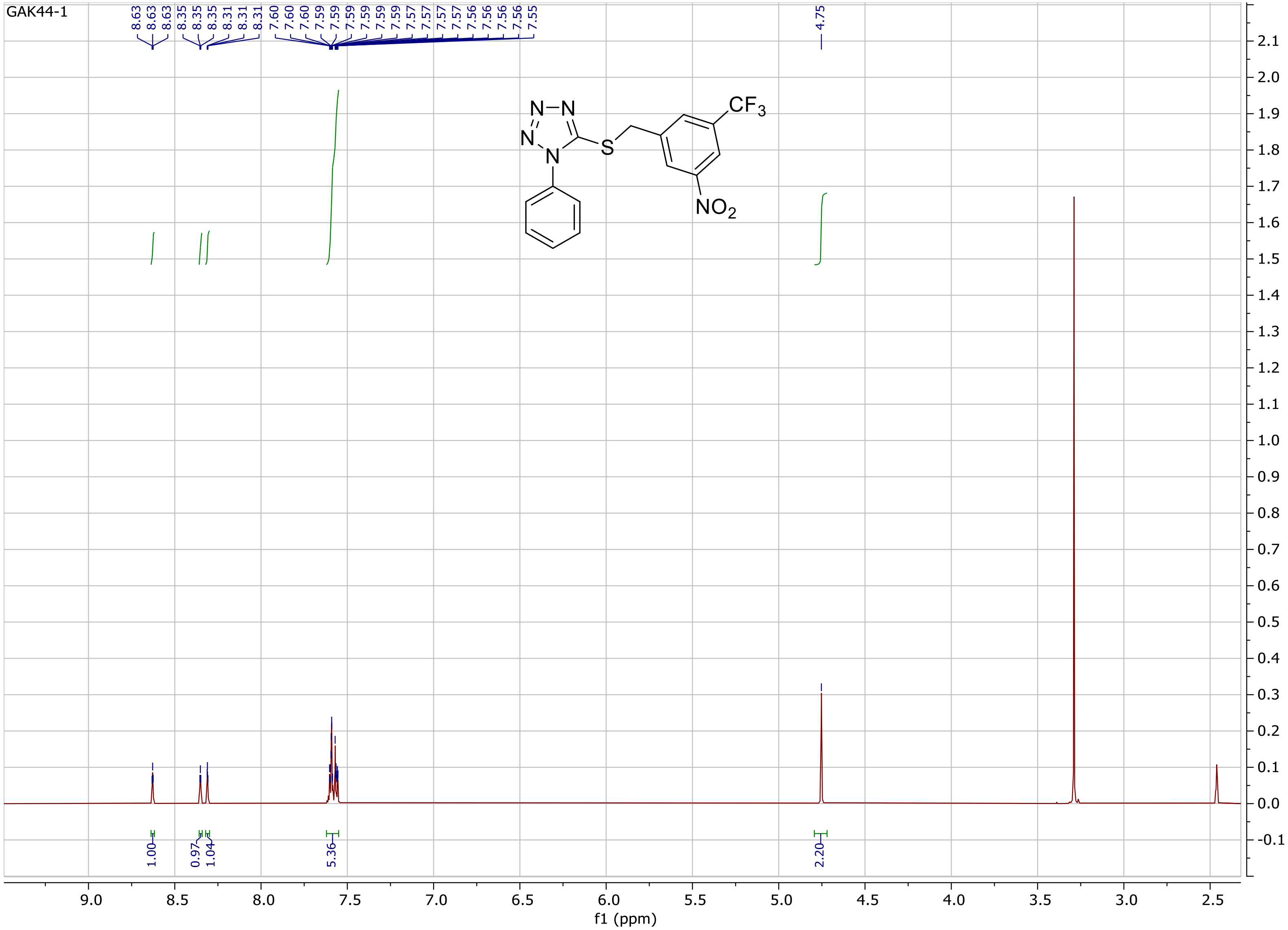

5-((3-Nitro-5-(trifluoromethyl)benzyl)sulfanyl)-1-phenyl-1H-tetrazole (**52a**): ^{13}C NMR (151 MHz, DMSO-d_6)

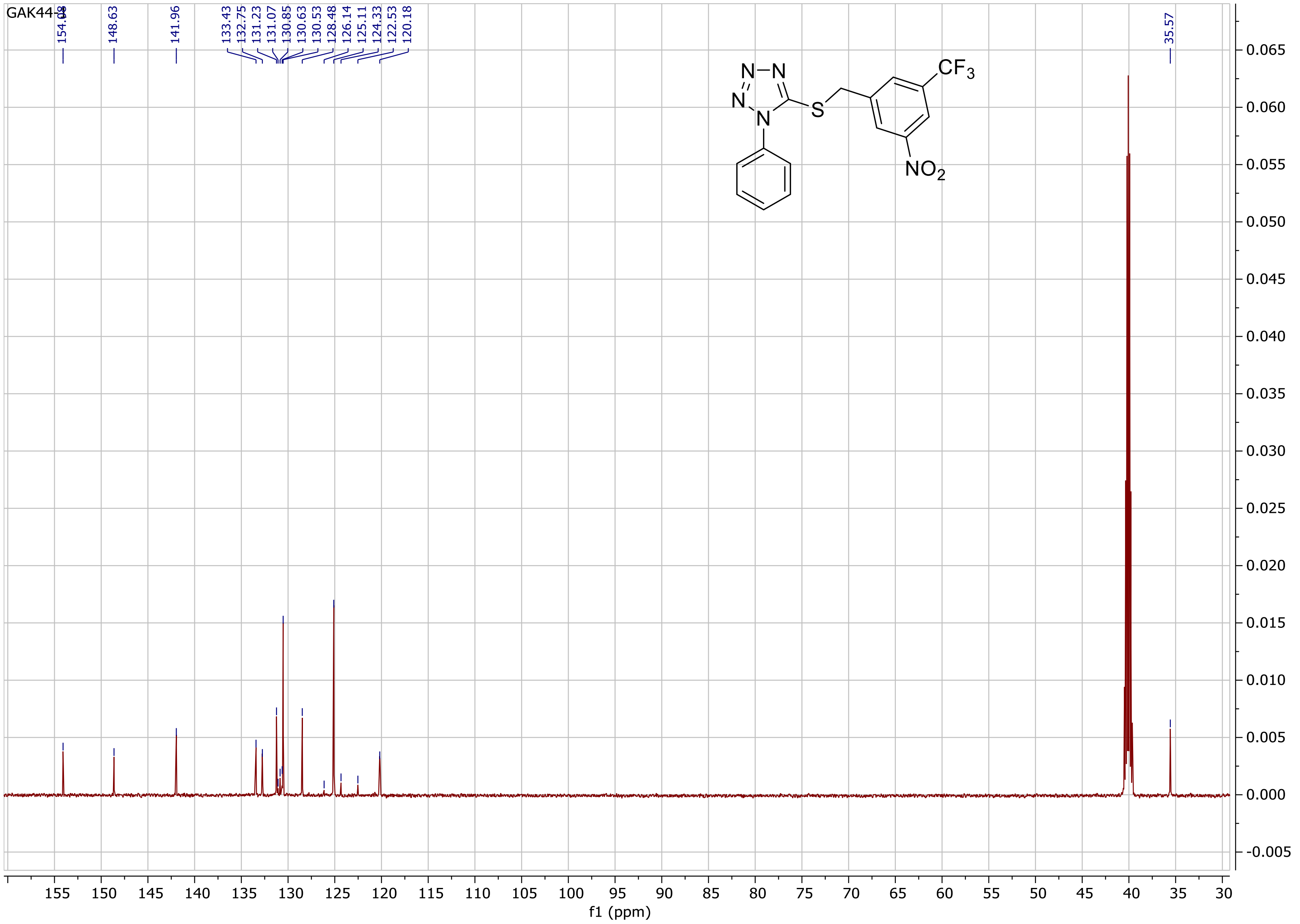

5-((3-Nitro-5-(trifluoromethyl)benzyl)sulfanyl)-1-phenyl-1H-tetrazole (**52a**)

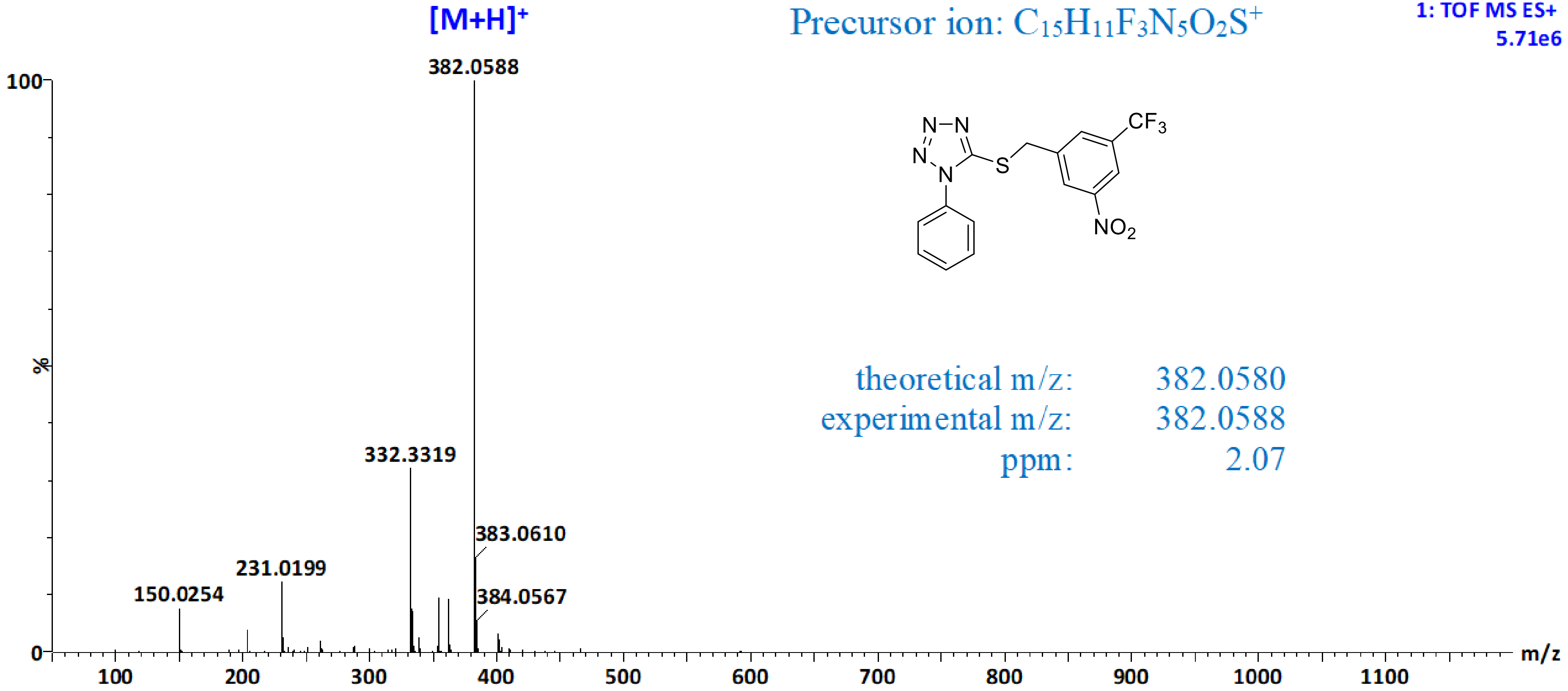

1-(4-Methoxyphenyl)-5-((3-nitro-5-(trifluoromethyl)benzyl)sulfanyl)-1H-tetrazole (**52b**): ^{1}H NMR (600 MHz, DMSO-d_6)

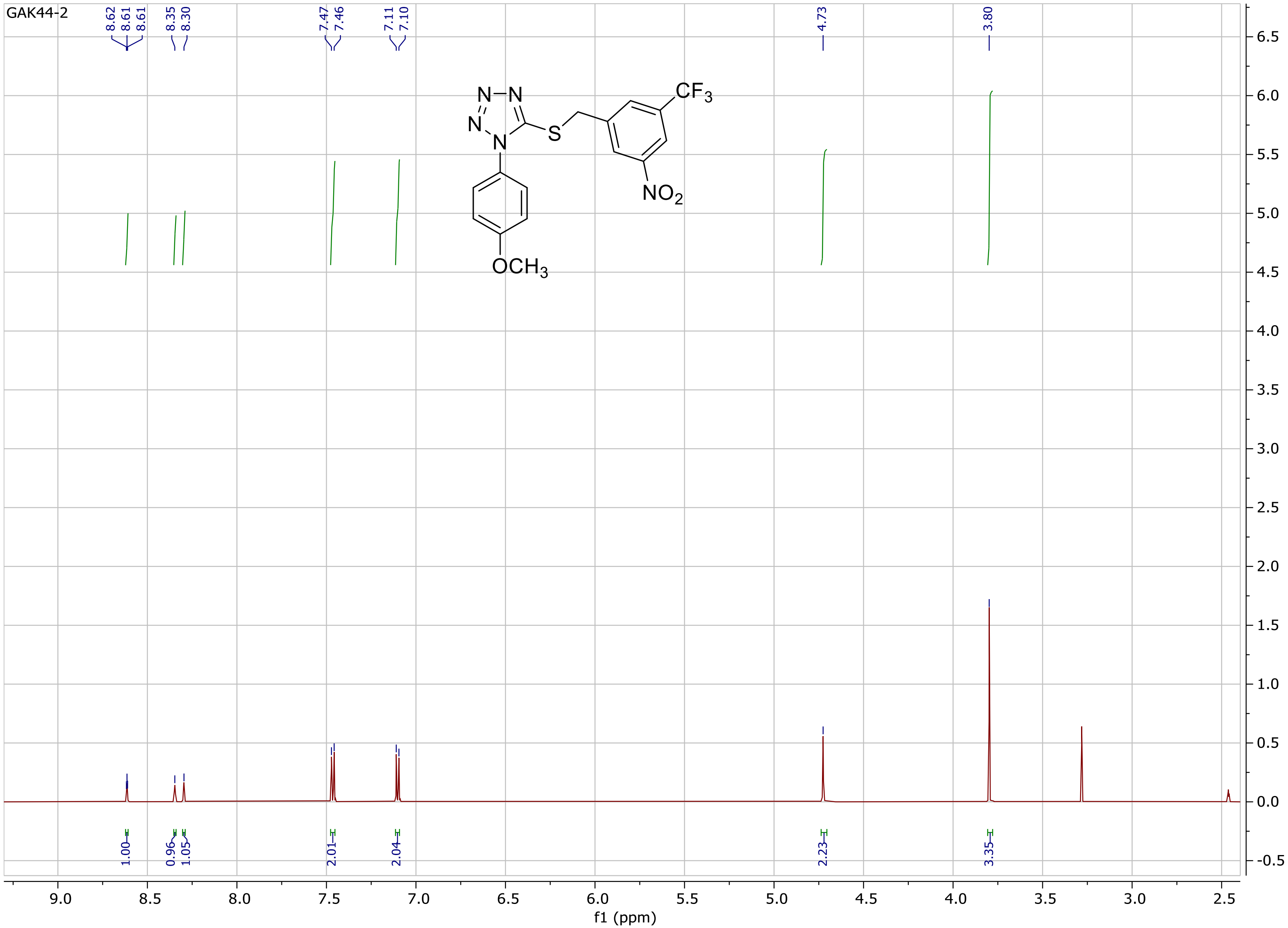

1-(4-Methoxyphenyl)-5-((3-nitro-5-(trifluoromethyl)benzyl)sulfanyl)-1H-tetrazole (**52b**): ^{13}C NMR (151 MHz, DMSO-d_6)

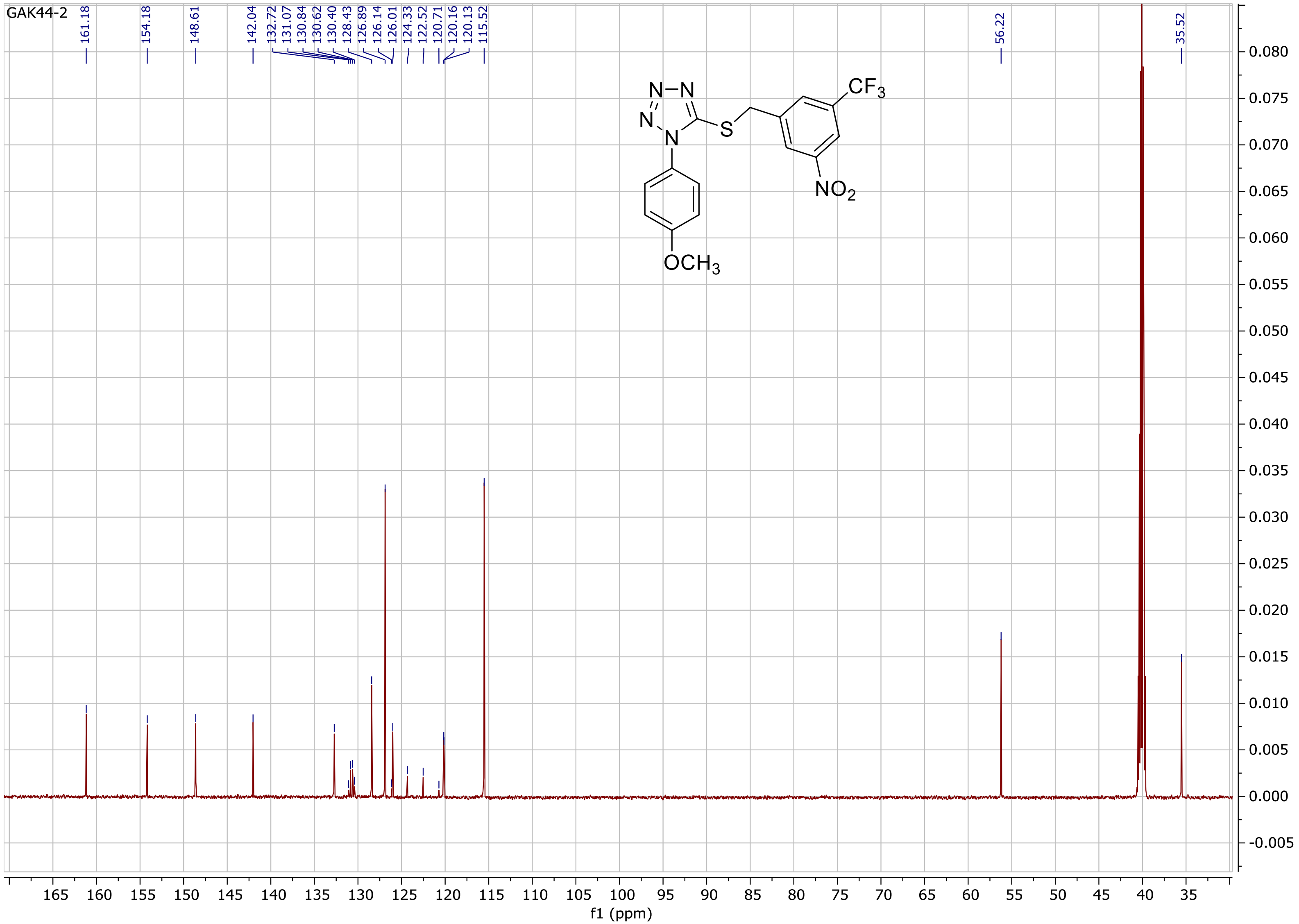

1-(4-Methoxyphenyl)-5-((3-nitro-5-(trifluoromethyl)benzyl)sulfanyl)-1H-tetrazole (**52b**)

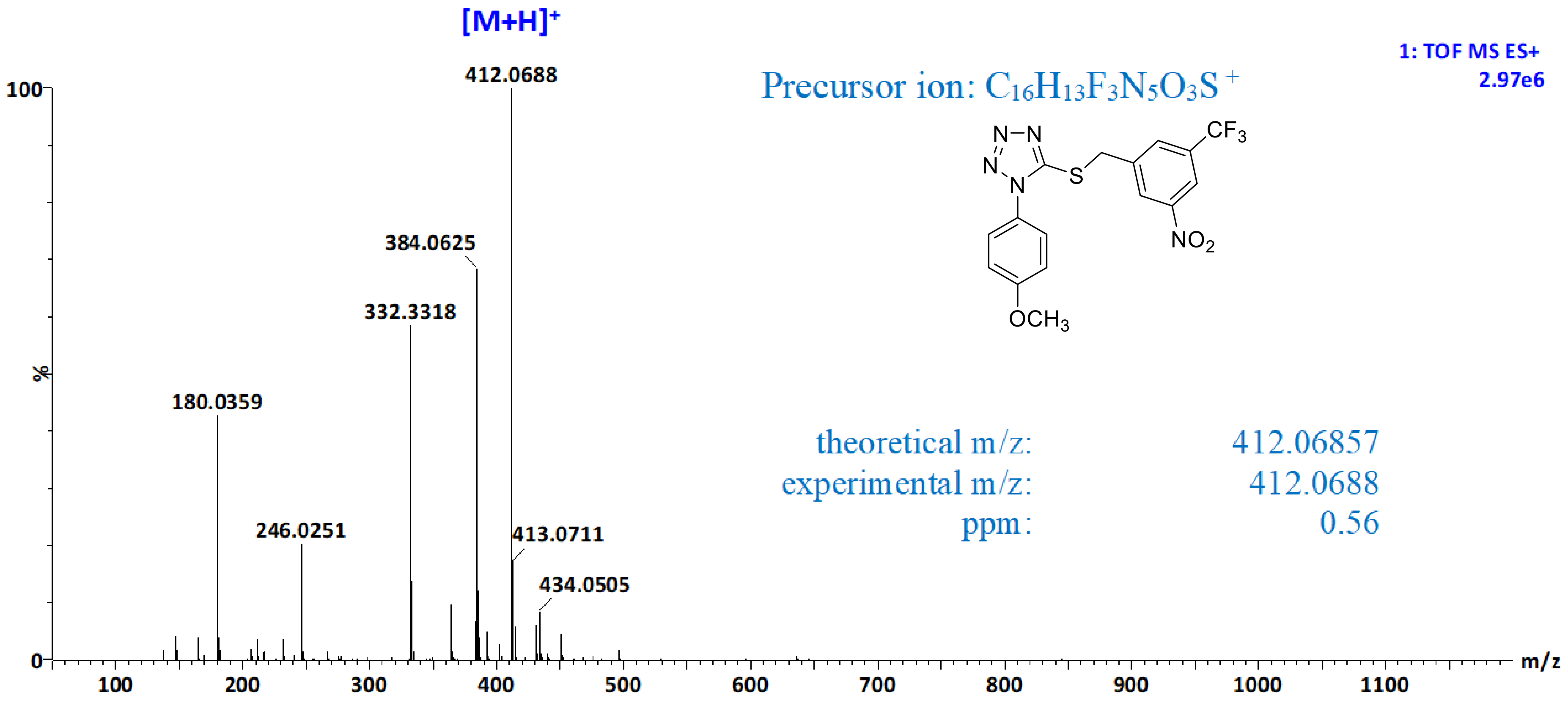

1-(4-Chlorophenyl)-5-((3-nitro-5-(trifluoromethyl)benzyl)sulfanyl)-1H-tetrazole (**52c**): ^{1}H NMR (600 MHz, DMSO-d_6)

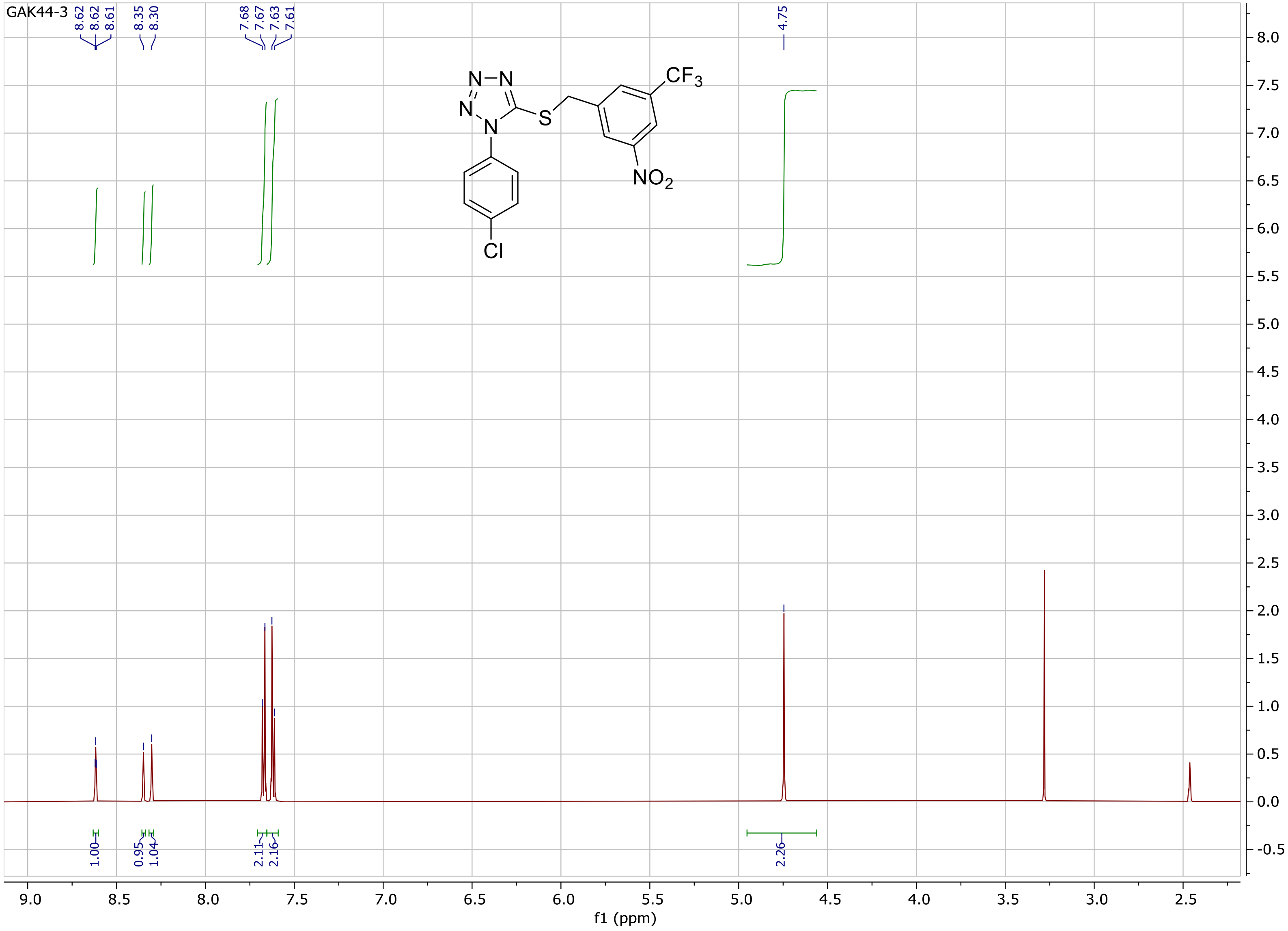

1-(4-Chlorophenyl)-5-((3-nitro-5-(trifluoromethyl)benzyl)sulfanyl)-1H-tetrazole (**52c**): ^{13}C NMR (151 MHz, DMSO-d_6)

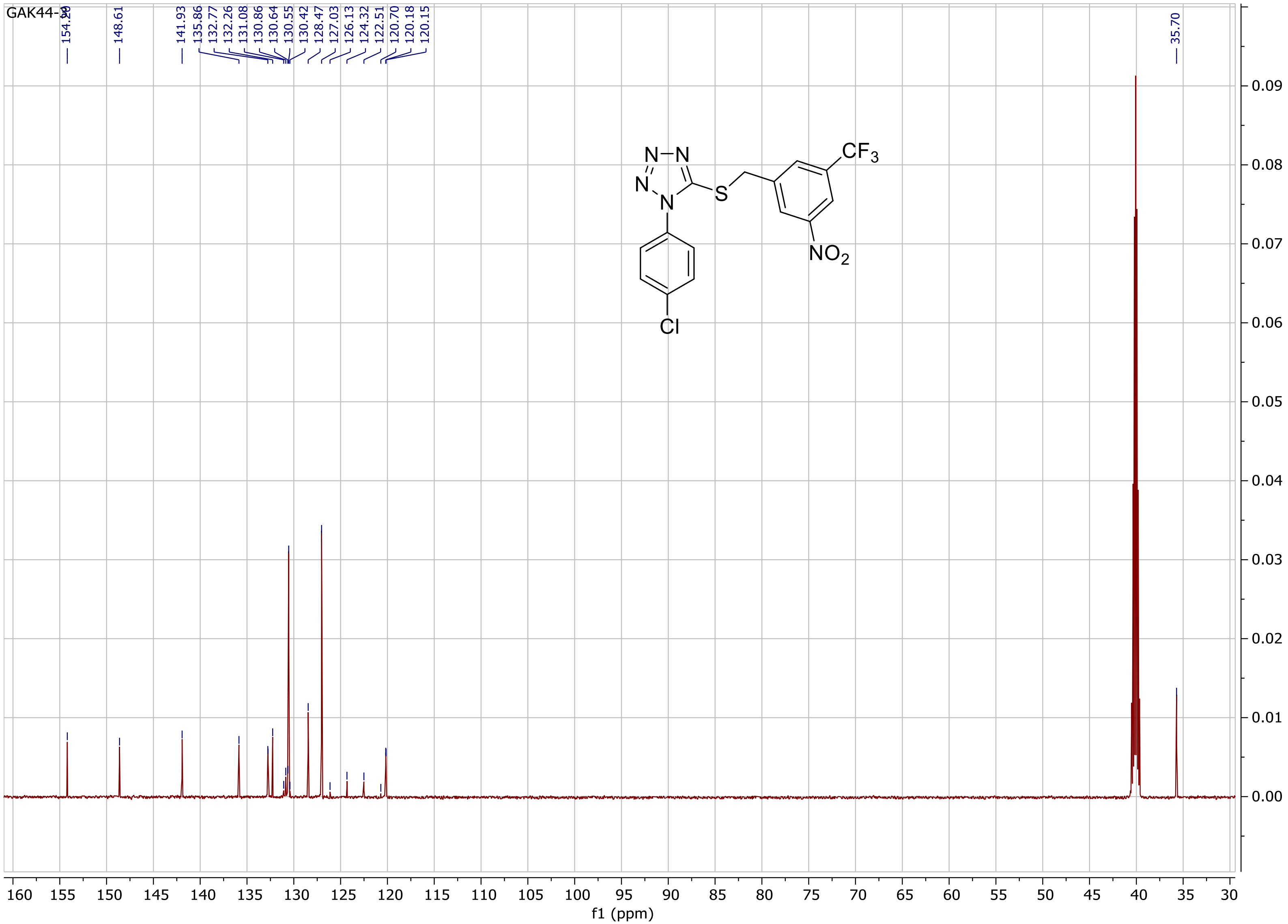

1-(4-Chlorophenyl)-5-((3-nitro-5-(trifluoromethyl)benzyl)sulfanyl)-1H-tetrazole (**52c**)

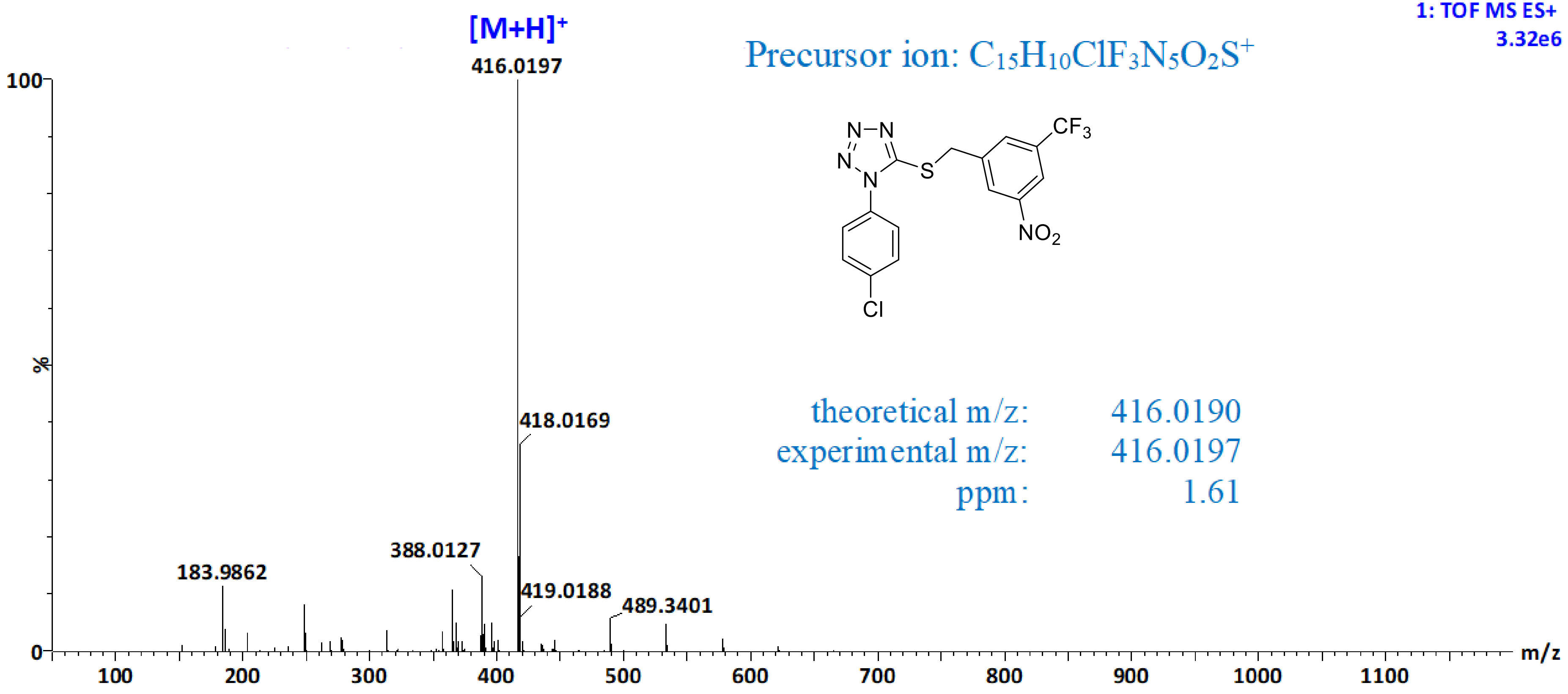

1-(4-Bromophenyl)-5-((3-nitro-5-(trifluoromethyl)benzyl)sulfanyl)-1H-tetrazole (**52d**): ^{1}H NMR (600 MHz, DMSO-d_6)

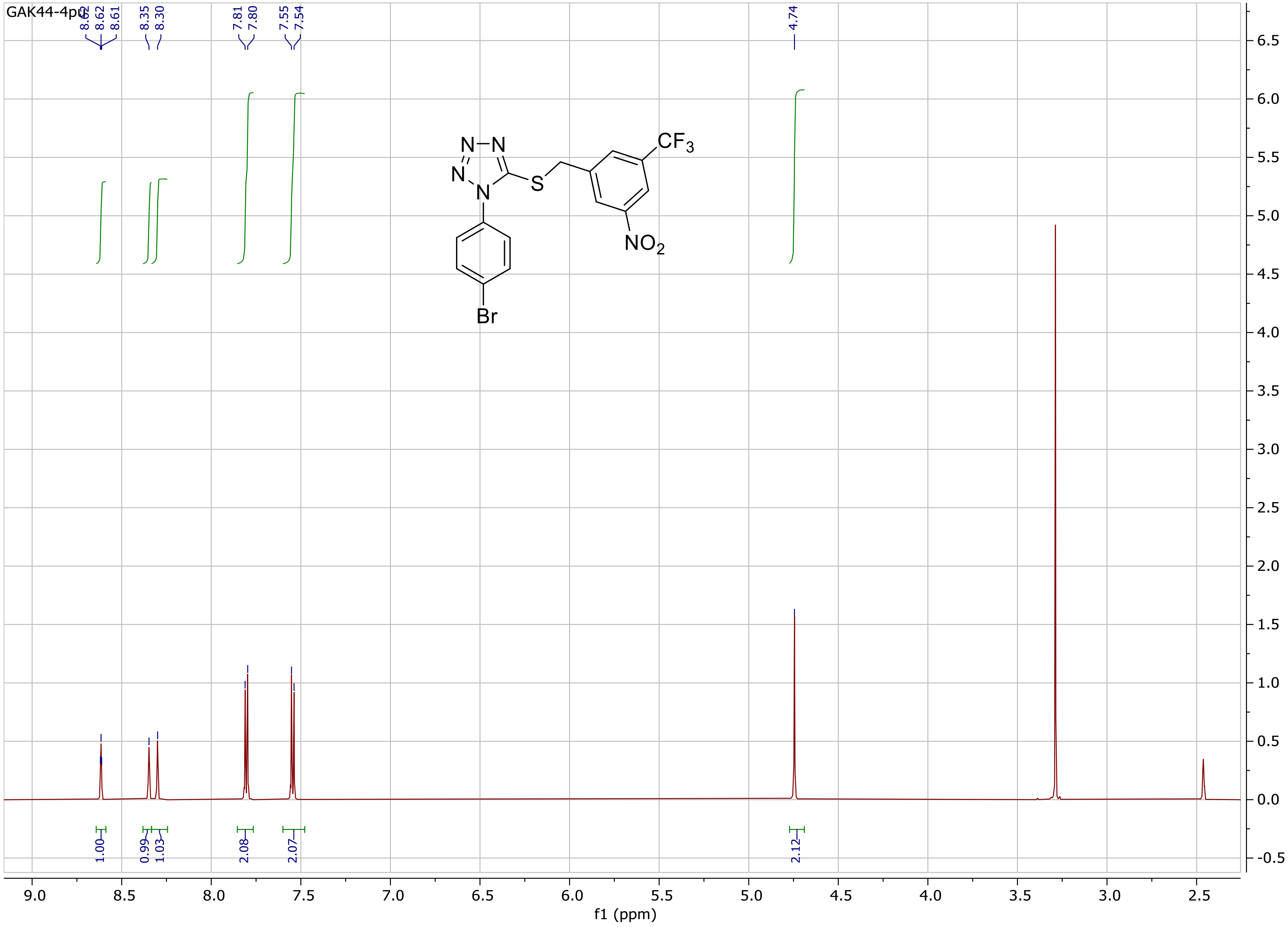

1-(4-Bromophenyl)-5-((3-nitro-5-(trifluoromethyl)benzyl)sulfanyl)-1H-tetrazole (**52d**): ^{13}C NMR (151 MHz, DMSO-d_6)

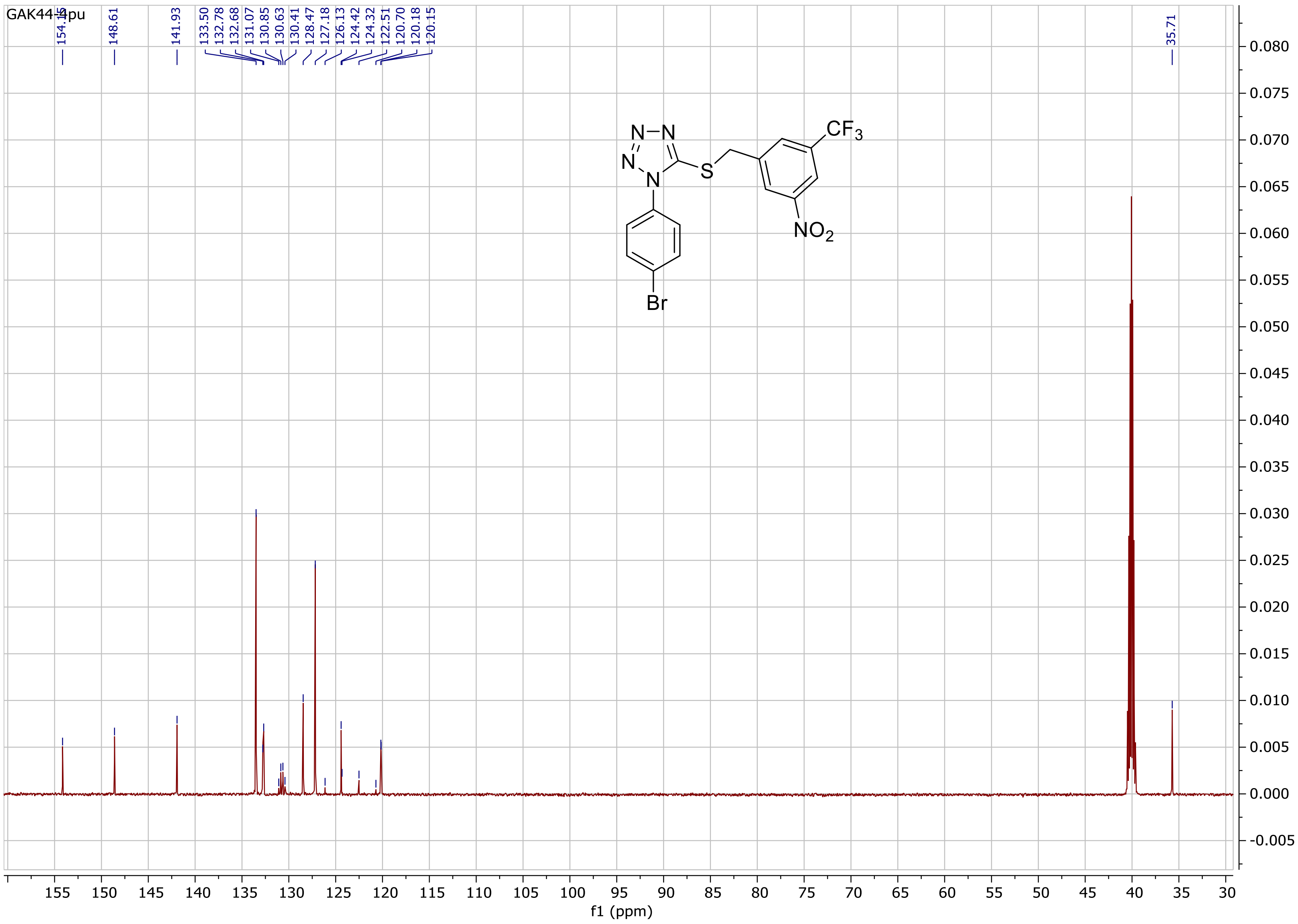

1-(4-Bromophenyl)-5-((3-nitro-5-(trifluoromethyl)benzyl)sulfanyl)-1H-tetrazole (**52d**):

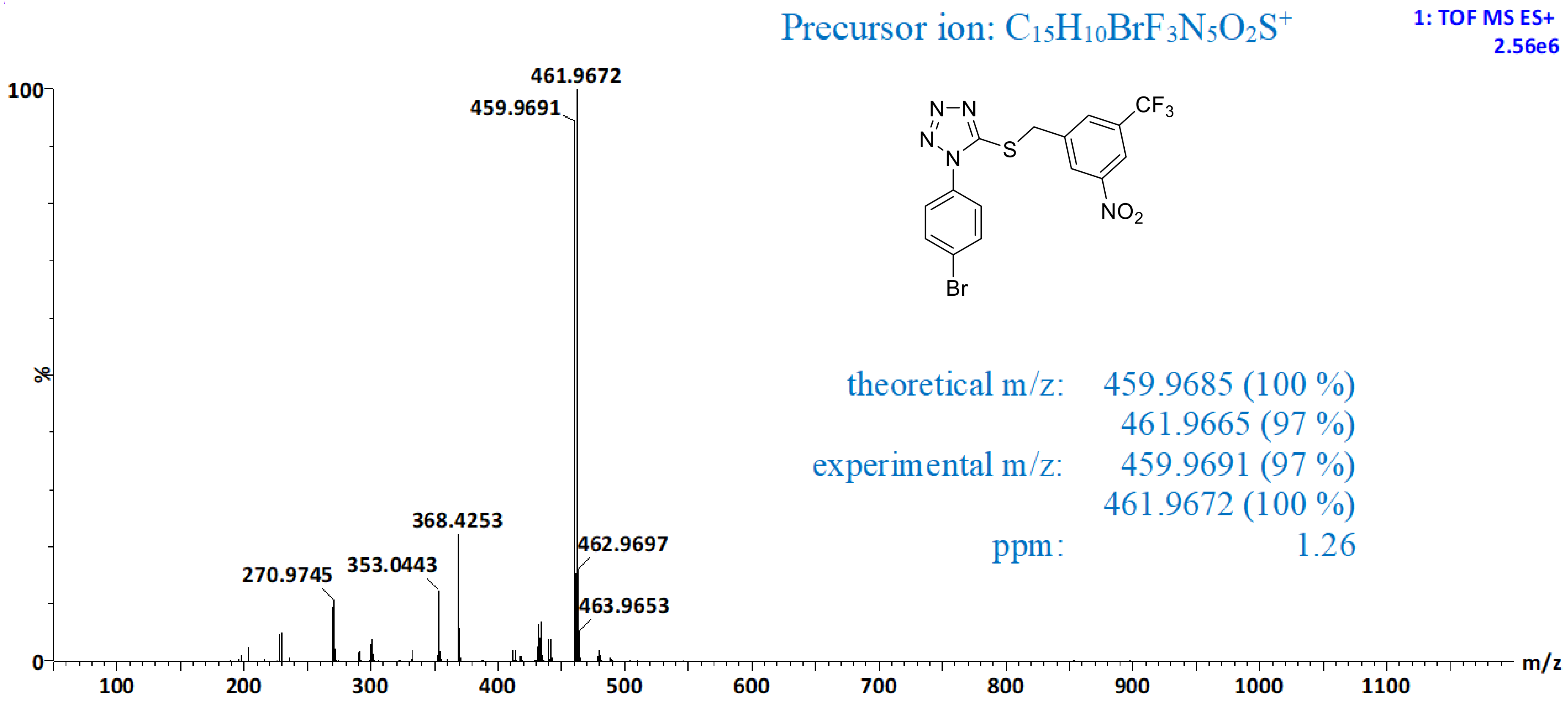

1-Cyclohexyl-5-((3-nitro-5-(trifluoromethyl)benzyl)sulfanyl)-1H-tetrazole (**52e**): ^{1}H NMR (600 MHz, DMSO-d_6)

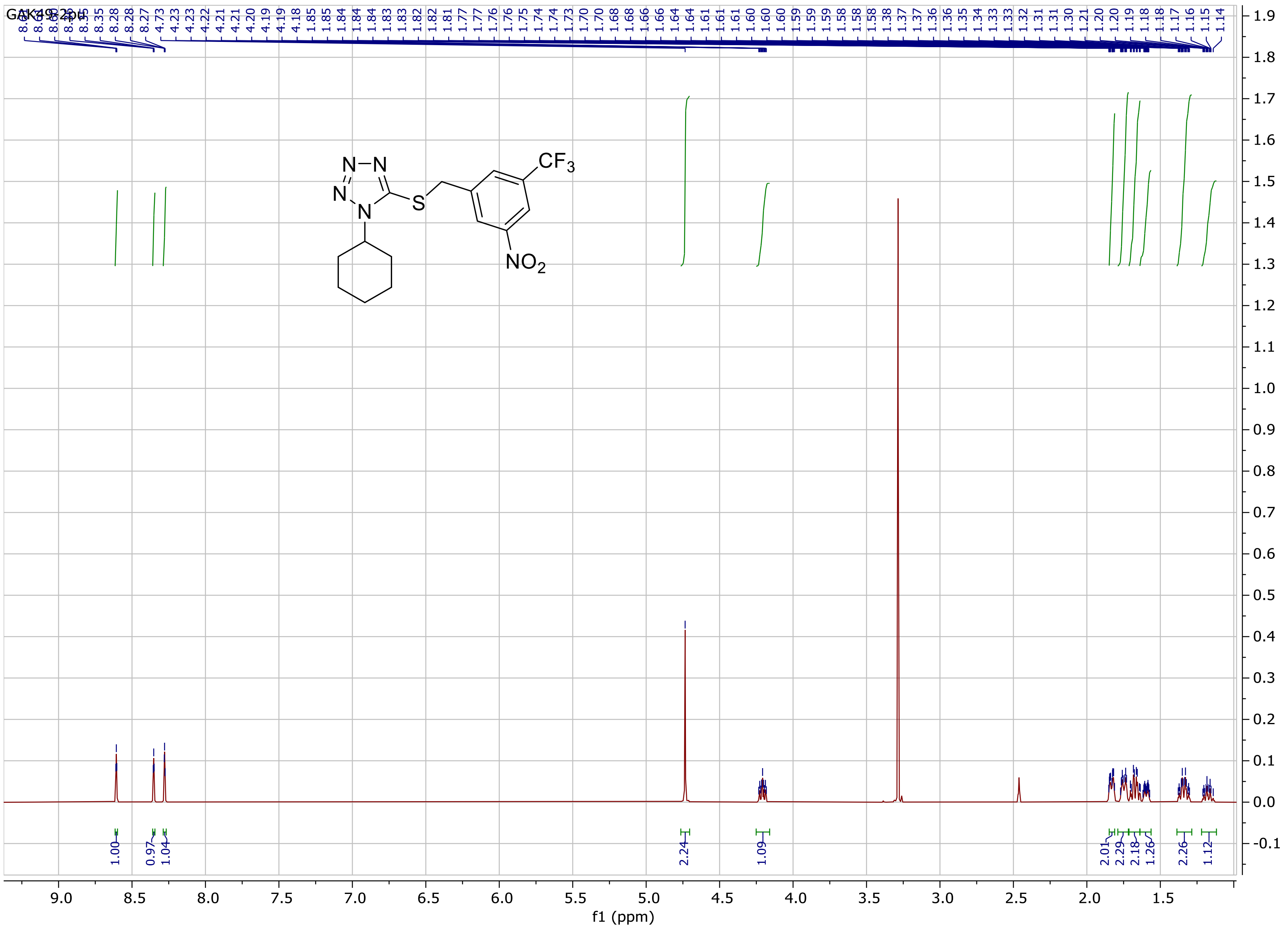

1-Cyclohexyl-5-((3-nitro-5-(trifluoromethyl)benzyl)sulfanyl)-1H-tetrazole (**52e**): ^{13}C NMR (151 MHz, DMSO-d_6)

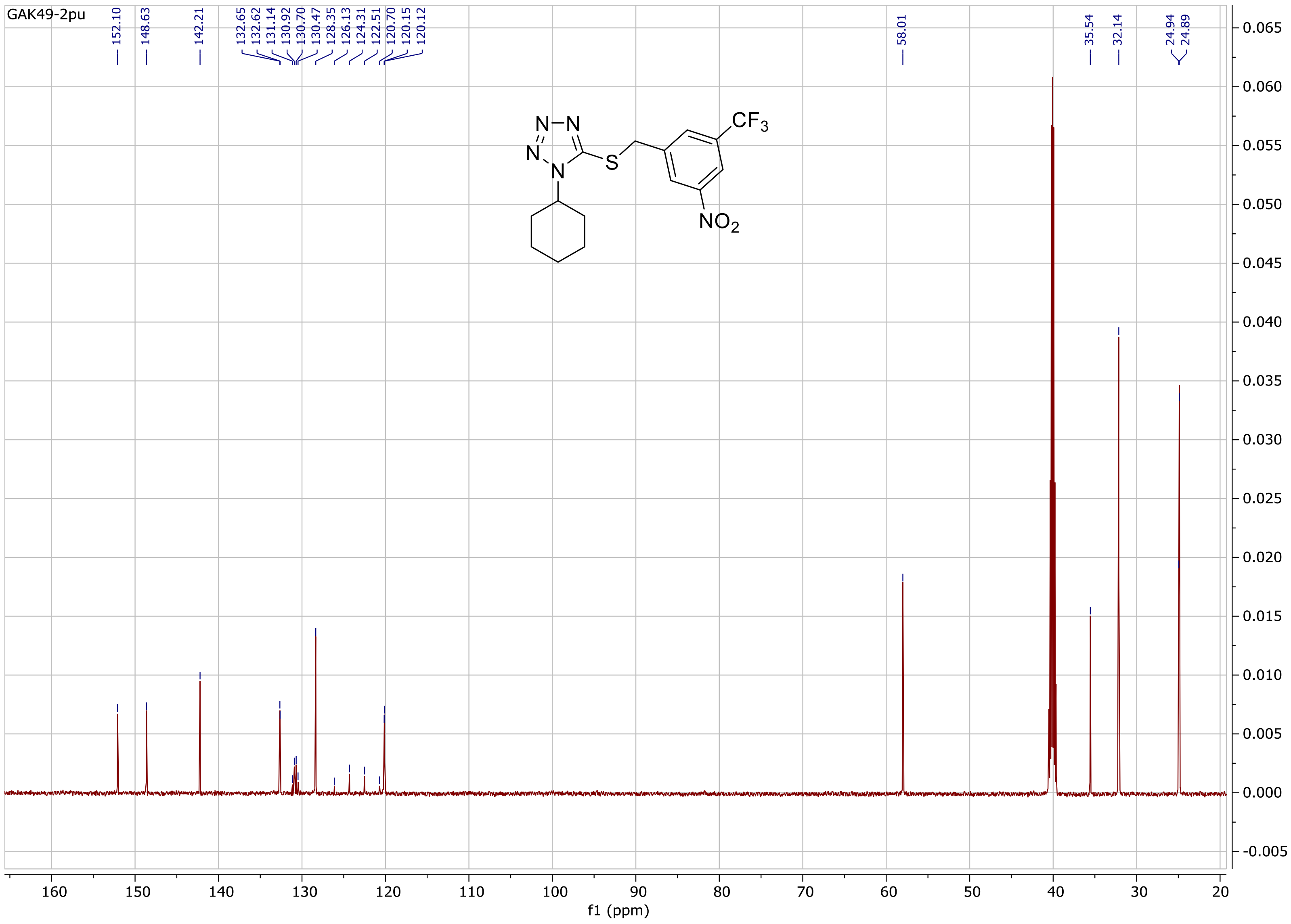

1-Cyclohexyl-5-((3-nitro-5-(trifluoromethyl)benzyl)sulfanyl)-1H-tetrazole (**52e**):

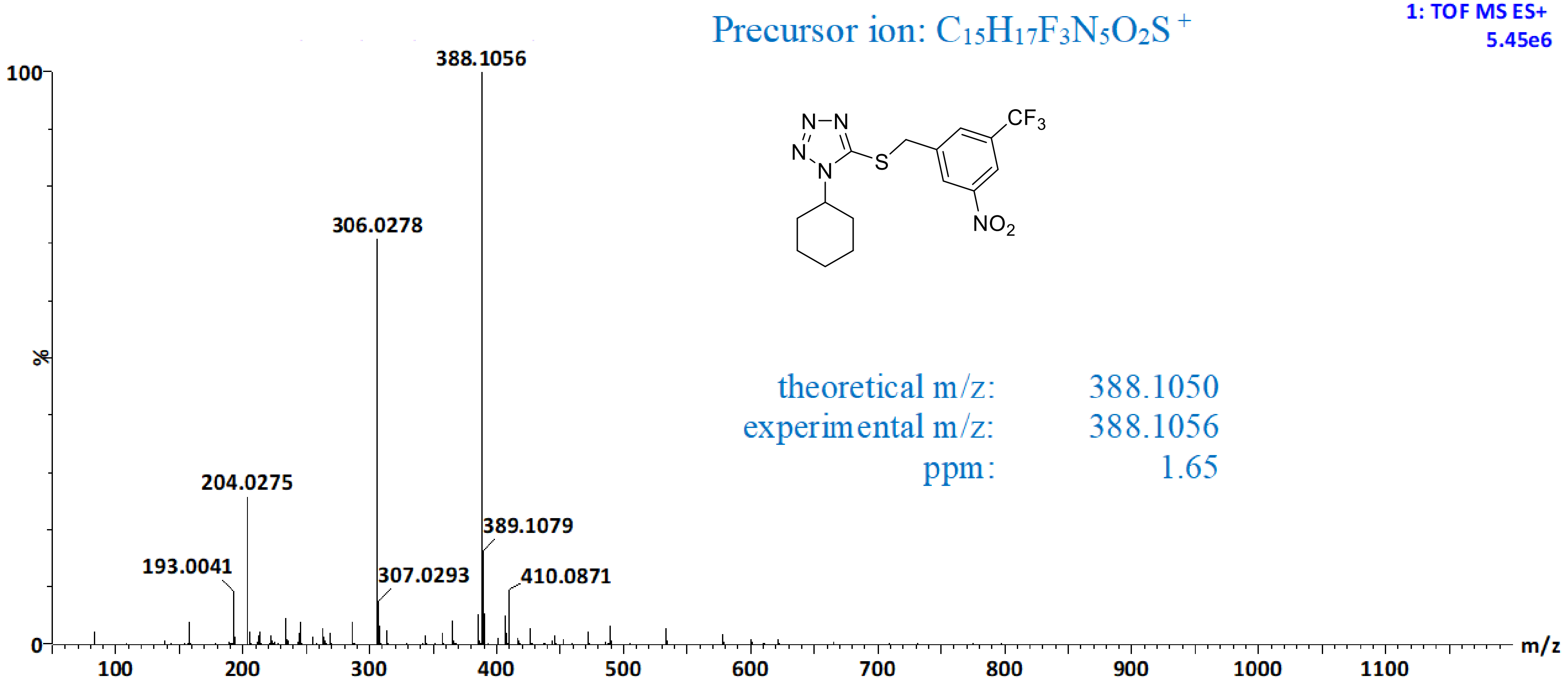

5-((3-Chloro-5-nitrobenzyl)sulfanyl)-1-phenyl-1H-tetrazole (**53a**): ^{1}H NMR (600 MHz, DMSO-d_6)

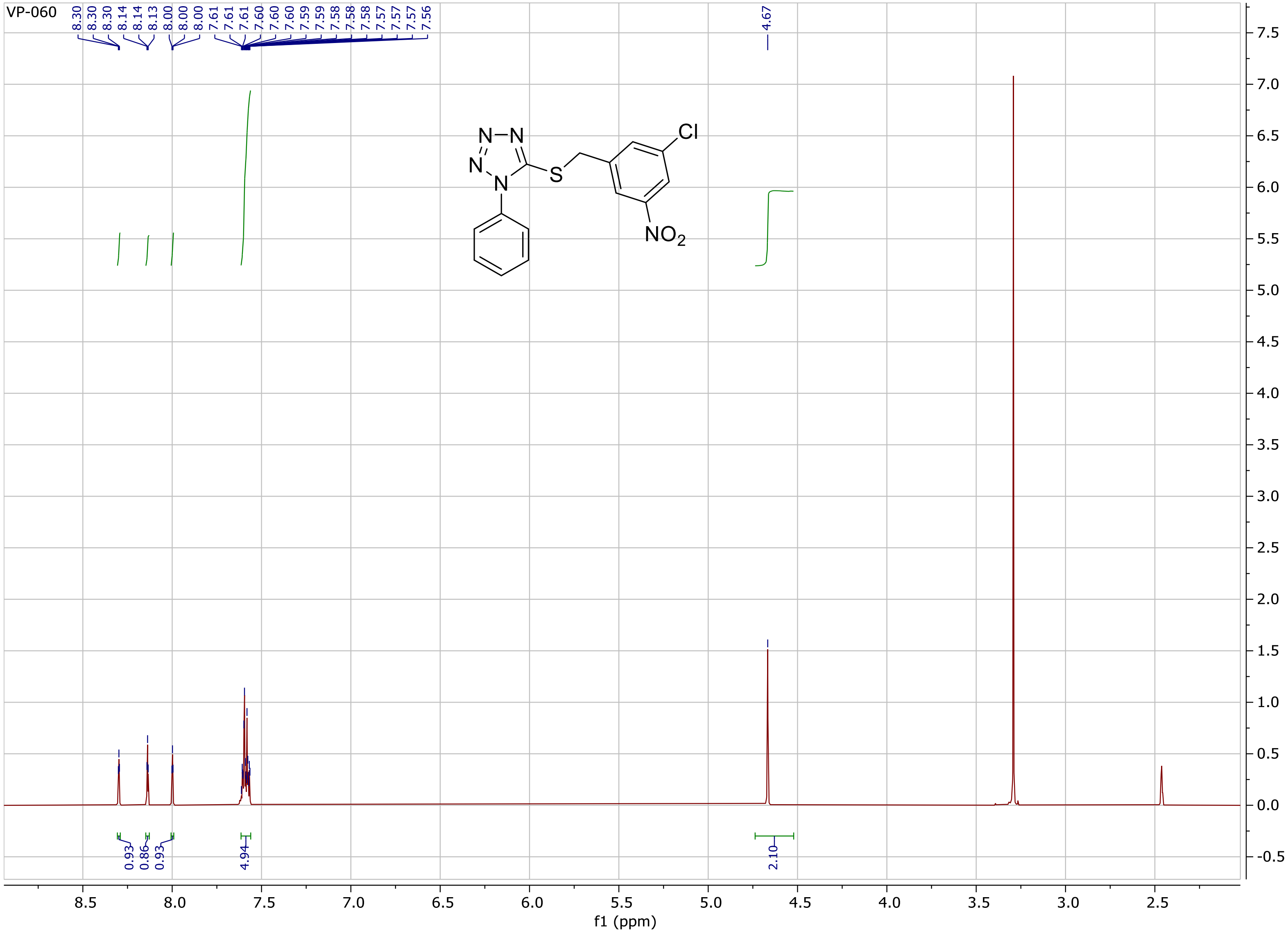

5-((3-Chloro-5-nitrobenzyl)sulfanyl)-1-phenyl-1H-tetrazole (**53a**): ^{13}C NMR (151 MHz, DMSO-d_6)

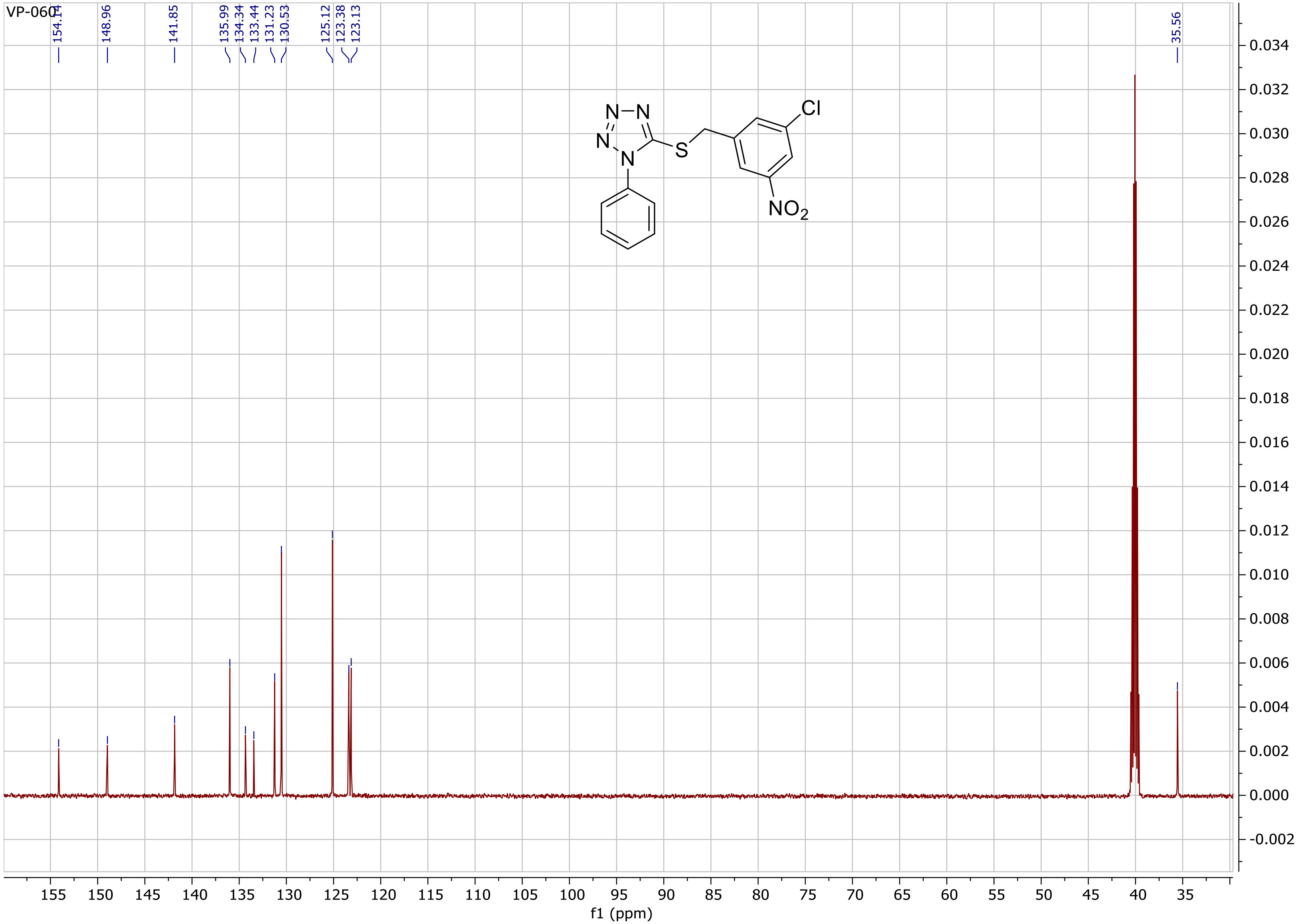

5-((3-Chloro-5-nitrobenzyl)sulfanyl)-1-(4-methoxyphenyl)-1H-tetrazole (**53b**): ^{1}H NMR (600 MHz, DMSO-d_6)

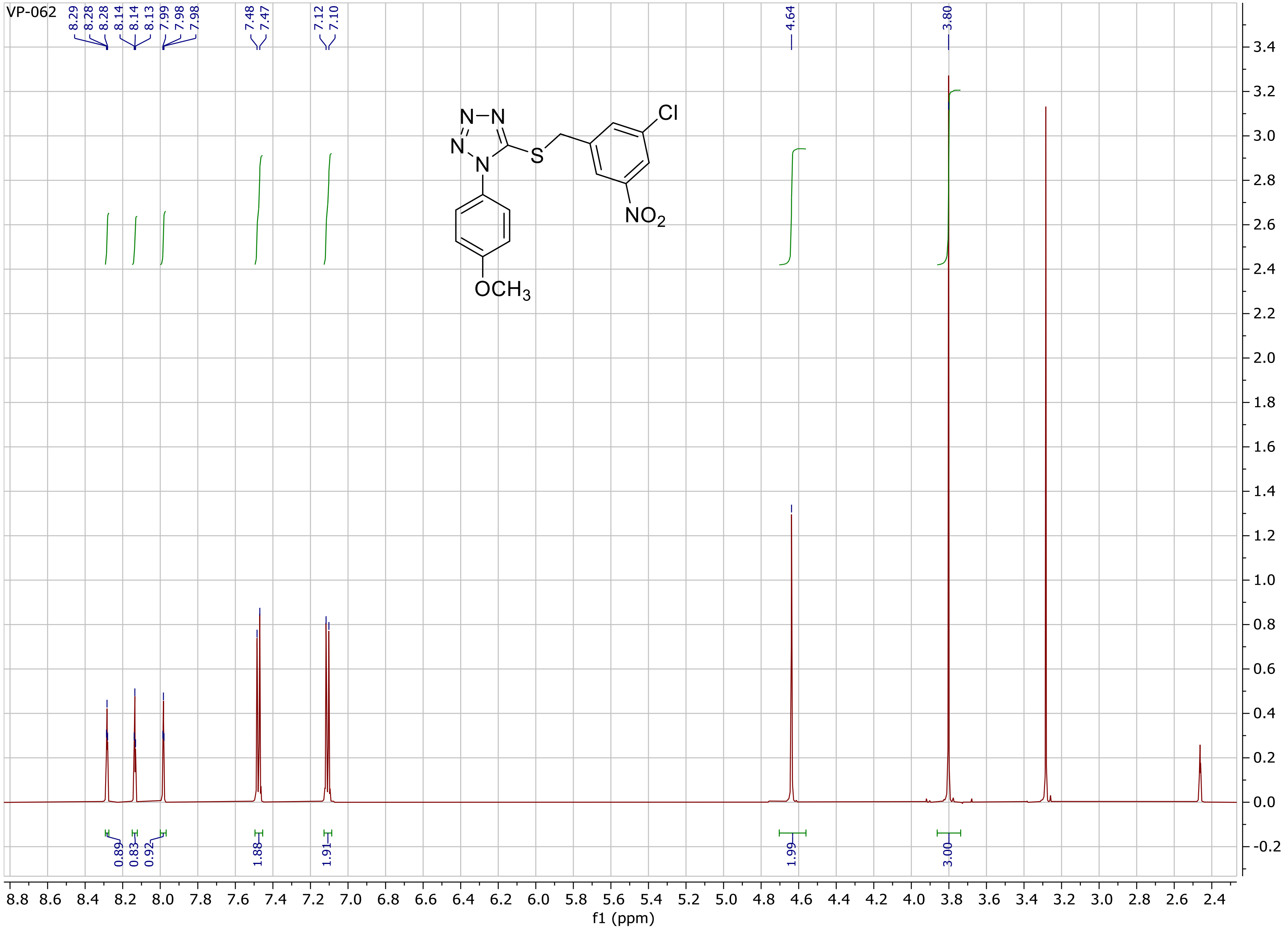

5-((3-Chloro-5-nitrobenzyl)sulfanyl)-1-(4-methoxyphenyl)-1H-tetrazole (**53b**): ^{13}C NMR (151 MHz, DMSO-d_6)

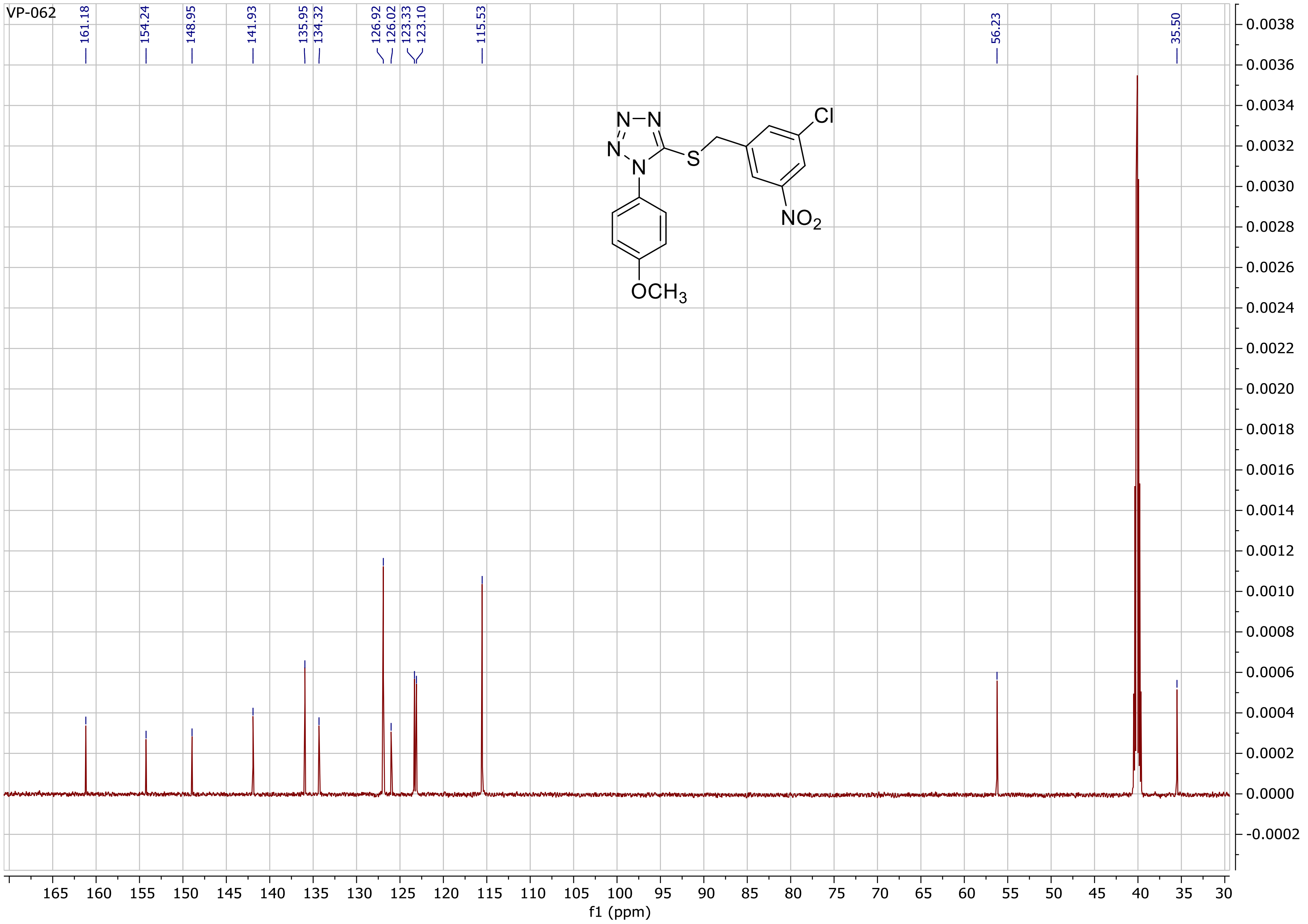

5-((3-Chloro-5-nitrobenzyl)sulfanyl)-1-(4-chlorophenyl)-1H-tetrazole (**53c**): ^{1}H NMR (500 MHz, DMSO-d_6)

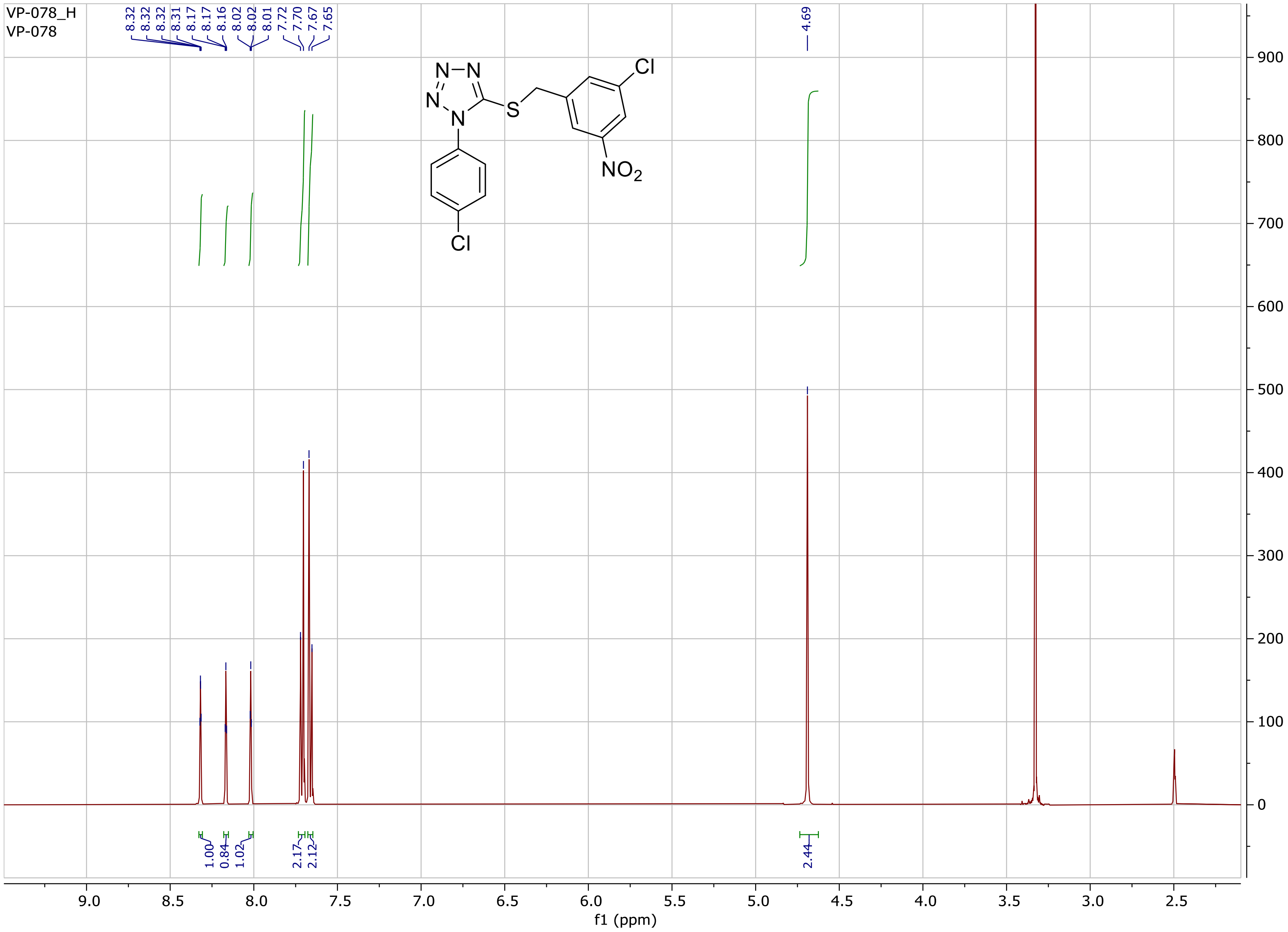

5-((3-Chloro-5-nitrobenzyl)sulfanyl)-1-(4-chlorophenyl)-1H-tetrazole (**53c**): ^{13}C NMR (126 MHz, DMSO-d_6)

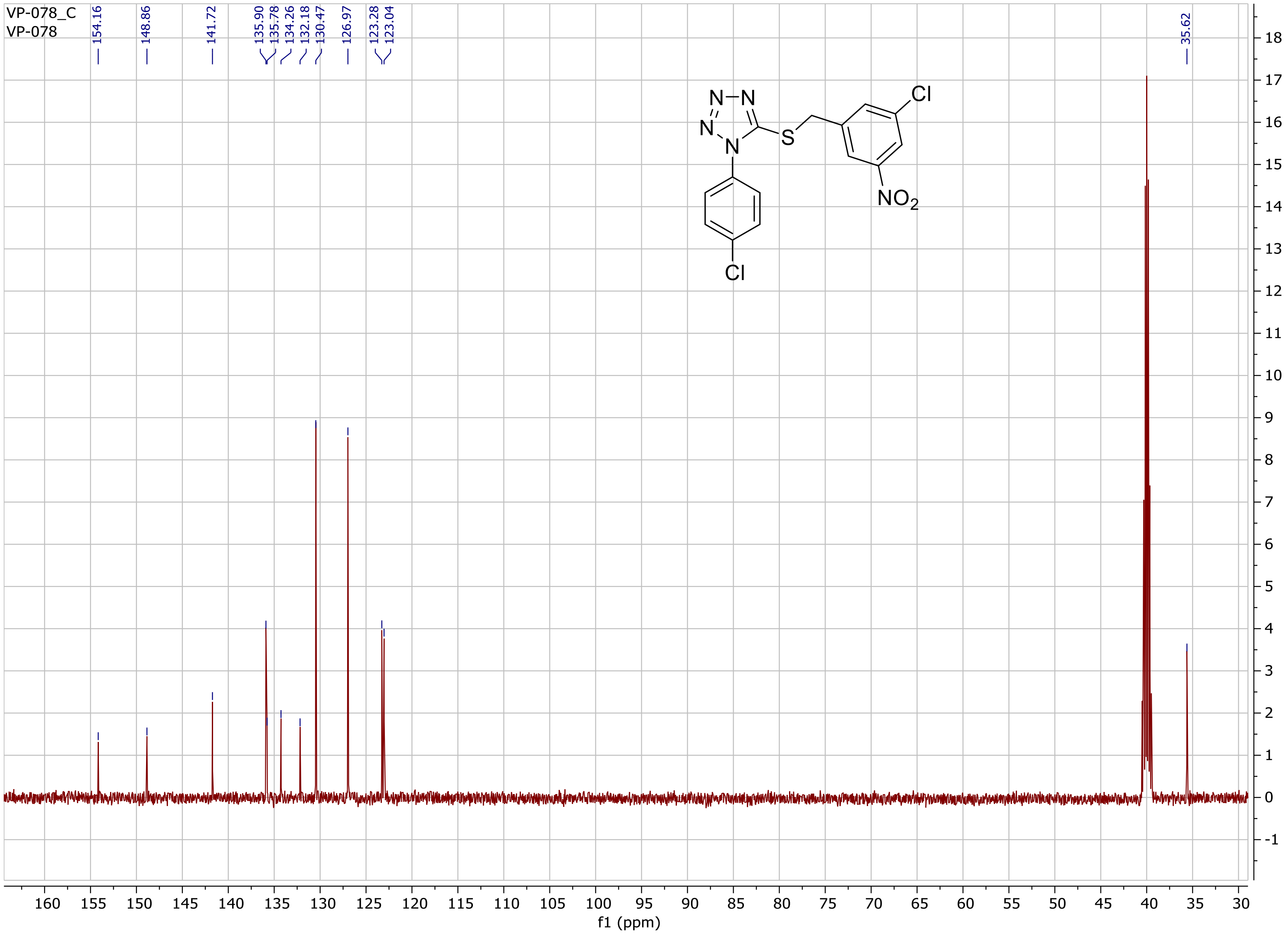

1-(4-Bromophenyl)-5-((3-chloro-5-nitrobenzyl)sulfanyl)-1H-tetrazole (**53d**): ^{1}H NMR (600 MHz, DMSO-d_6)

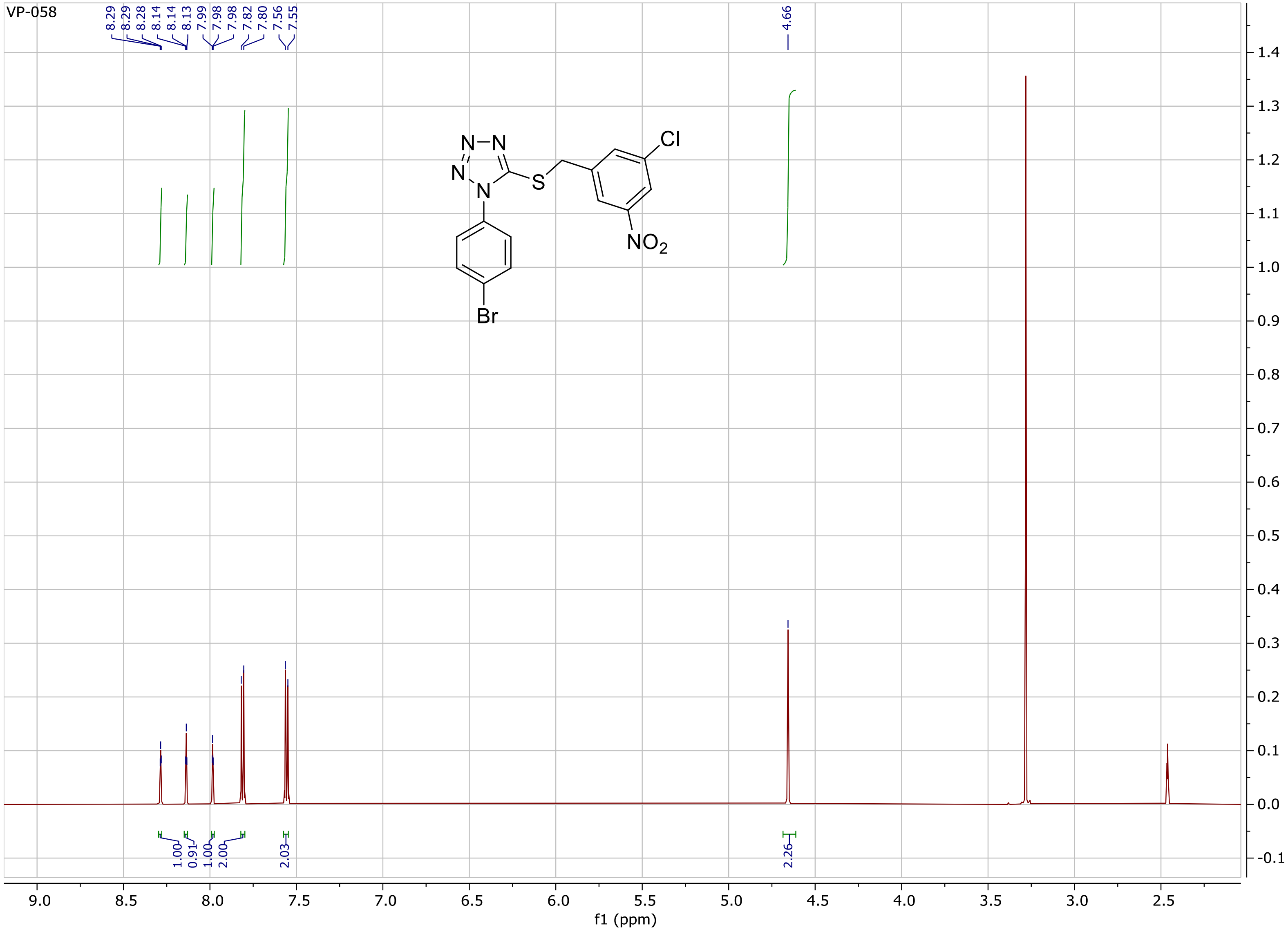

1-(4-Bromophenyl)-5-((3-chloro-5-nitrobenzyl)sulfanyl)-1H-tetrazole (**53d**): ^{13}C NMR (151 MHz, DMSO-d_6)

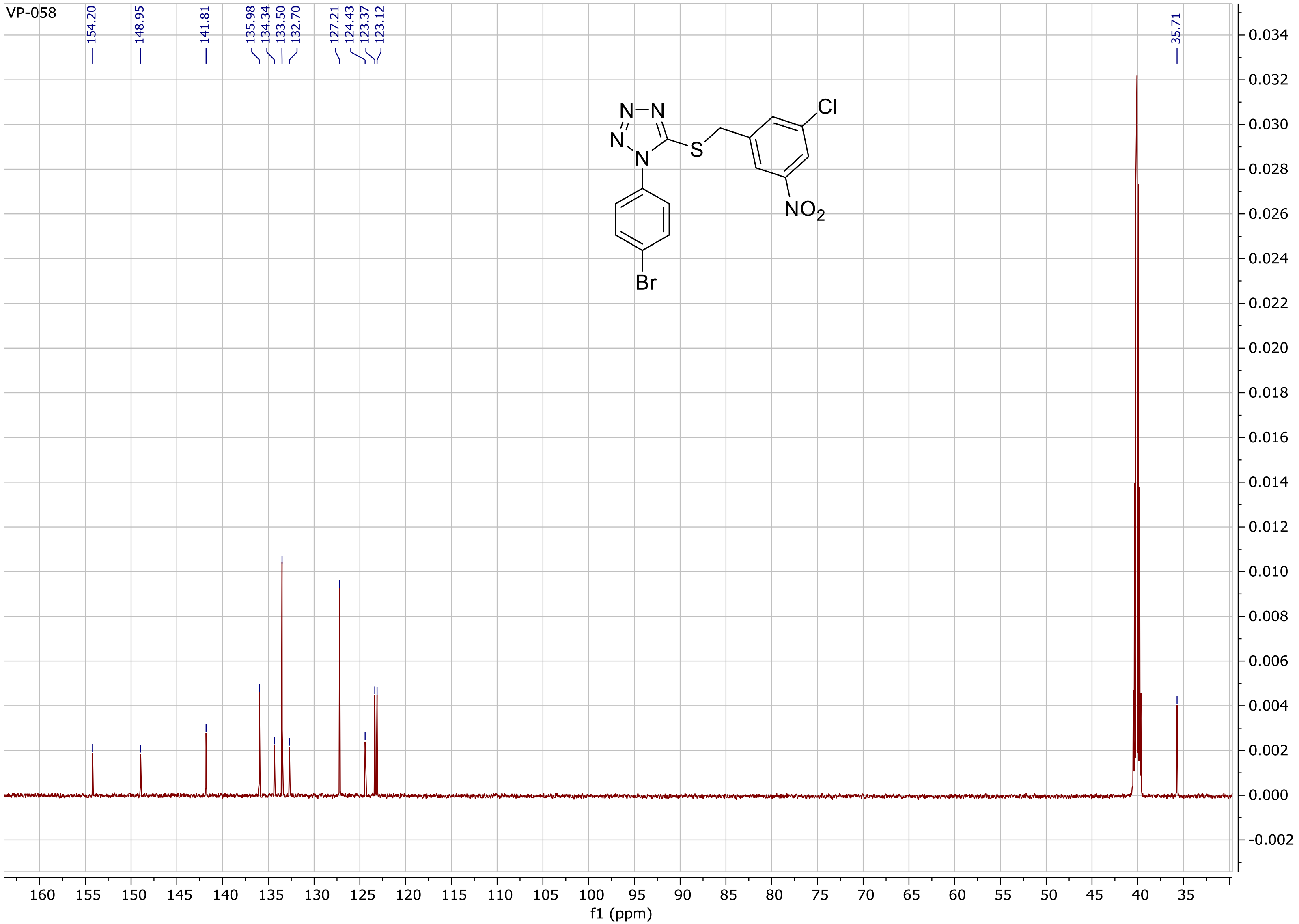

5-((3-Chloro-5-nitrobenzyl)sulfanyl)-1-cyclohexyl-1H-tetrazole (**53e**): ^{1}H NMR (600 MHz, DMSO-d_6)

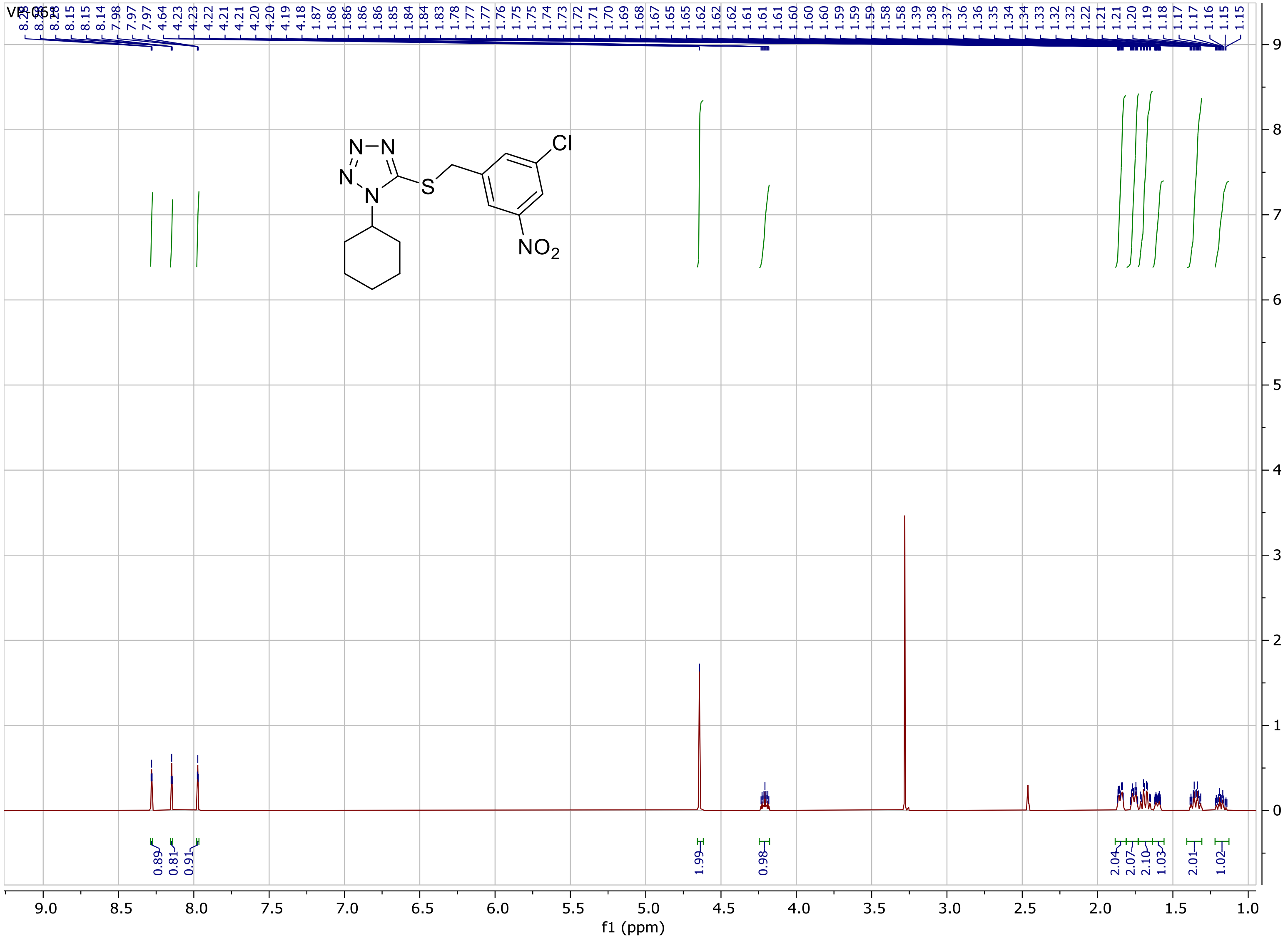

5-((3-Chloro-5-nitrobenzyl)sulfanyl)-1-cyclohexyl-1H-tetrazole (**53e**): ^{13}C NMR (151 MHz, DMSO-d_6)

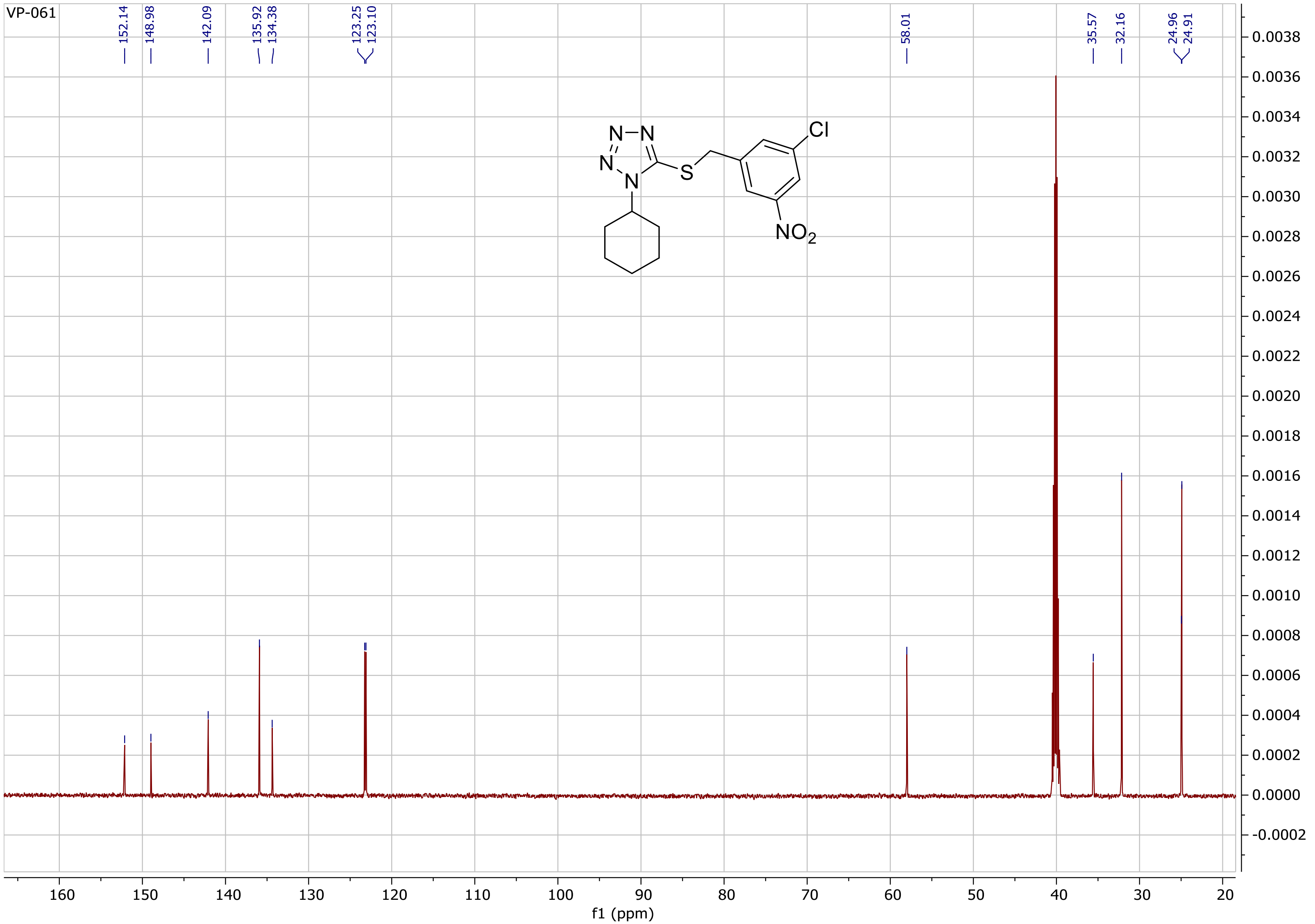

5-((3-Fluoro-5-nitrobenzyl)sulfanyl)-1-phenyl-1H-tetrazole (**54a**): ^{1}H NMR (600 MHz, DMSO-d_6)

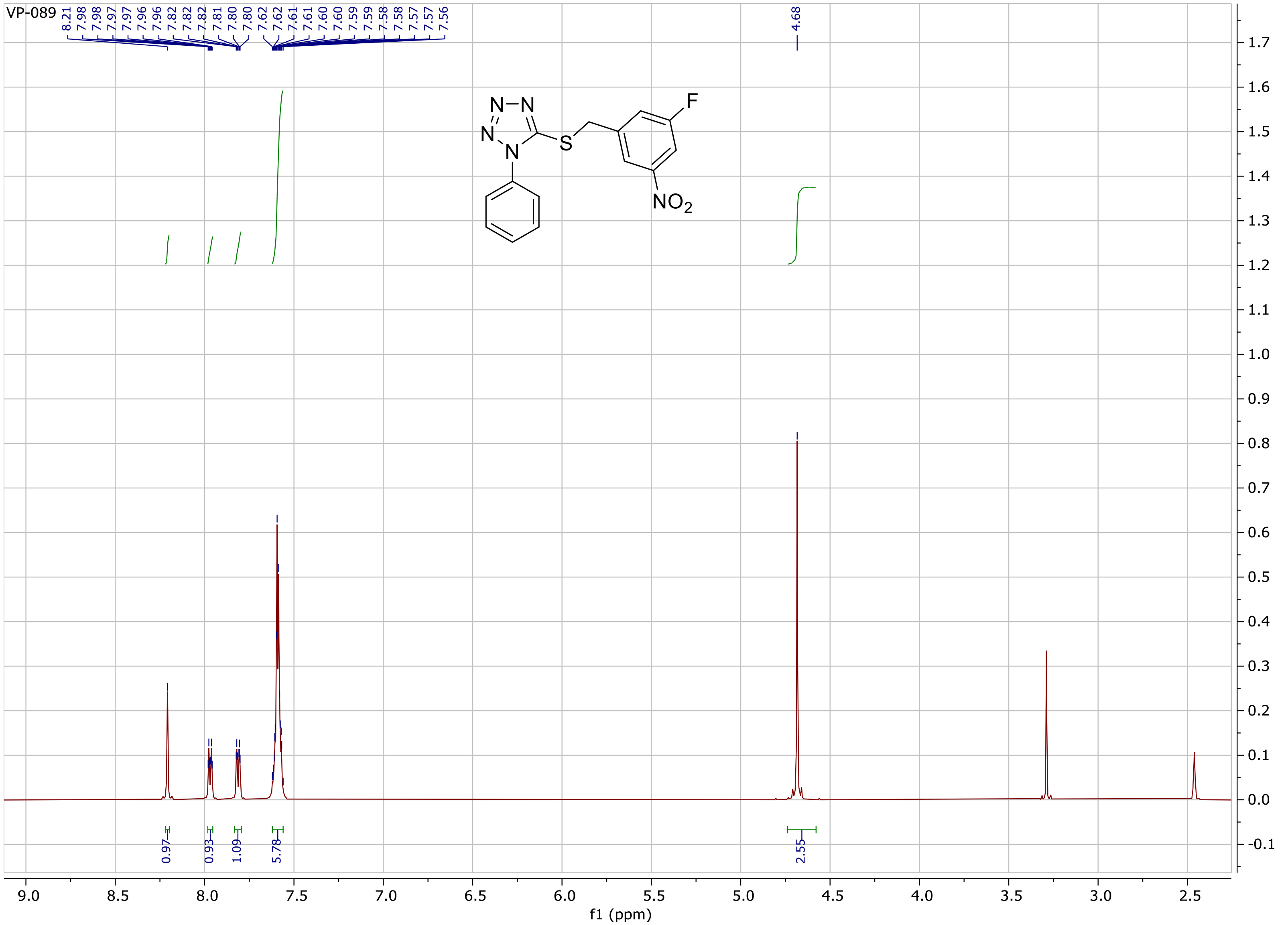

5-((3-Fluoro-5-nitrobenzyl)sulfanyl)-1-phenyl-1H-tetrazole (**54a**): ^{13}C NMR (151 MHz, DMSO-d_6)

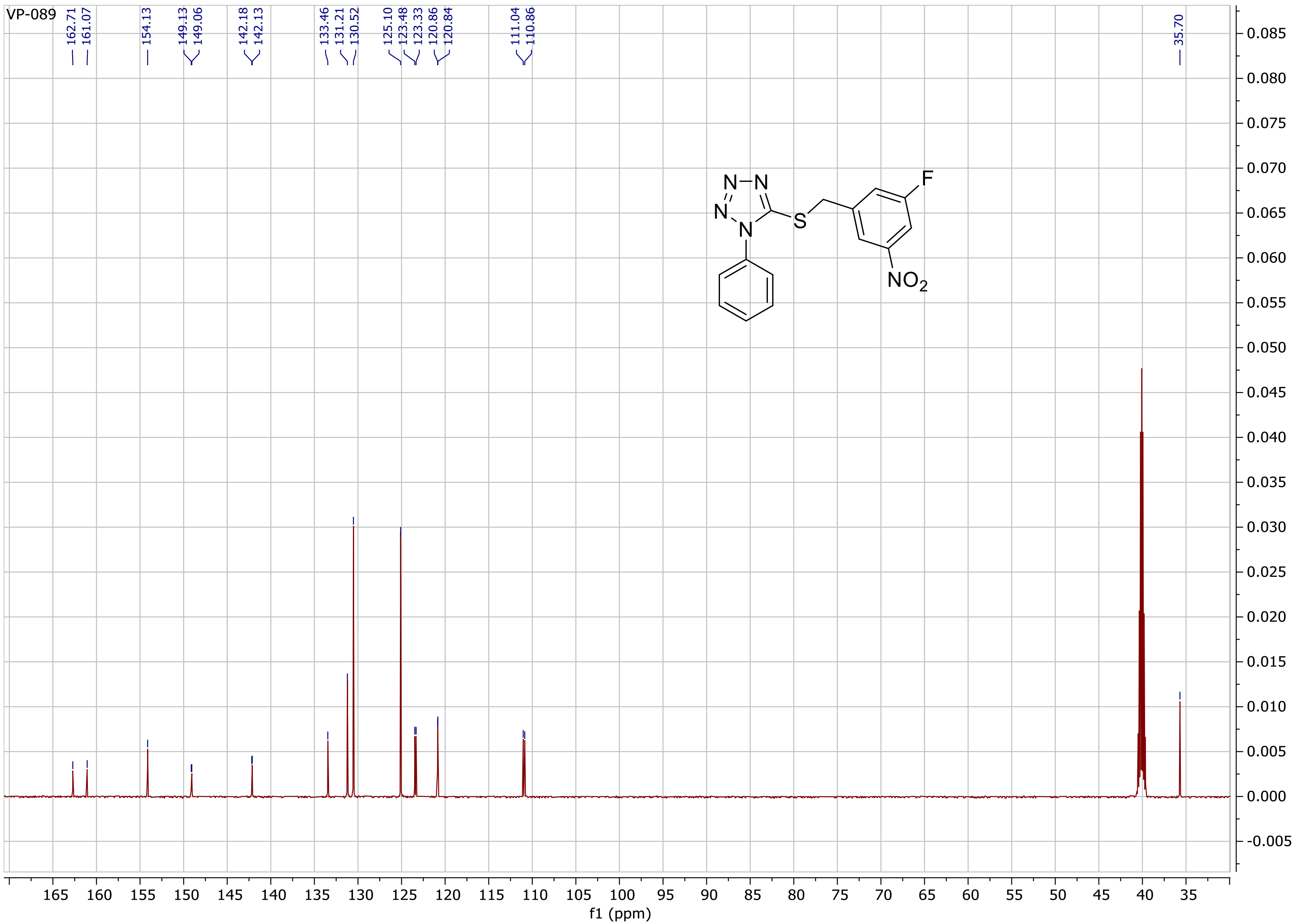

5-((3-Fluoro-5-nitrobenzyl)sulfanyl)-1-phenyl-1H-tetrazole (**54a**):

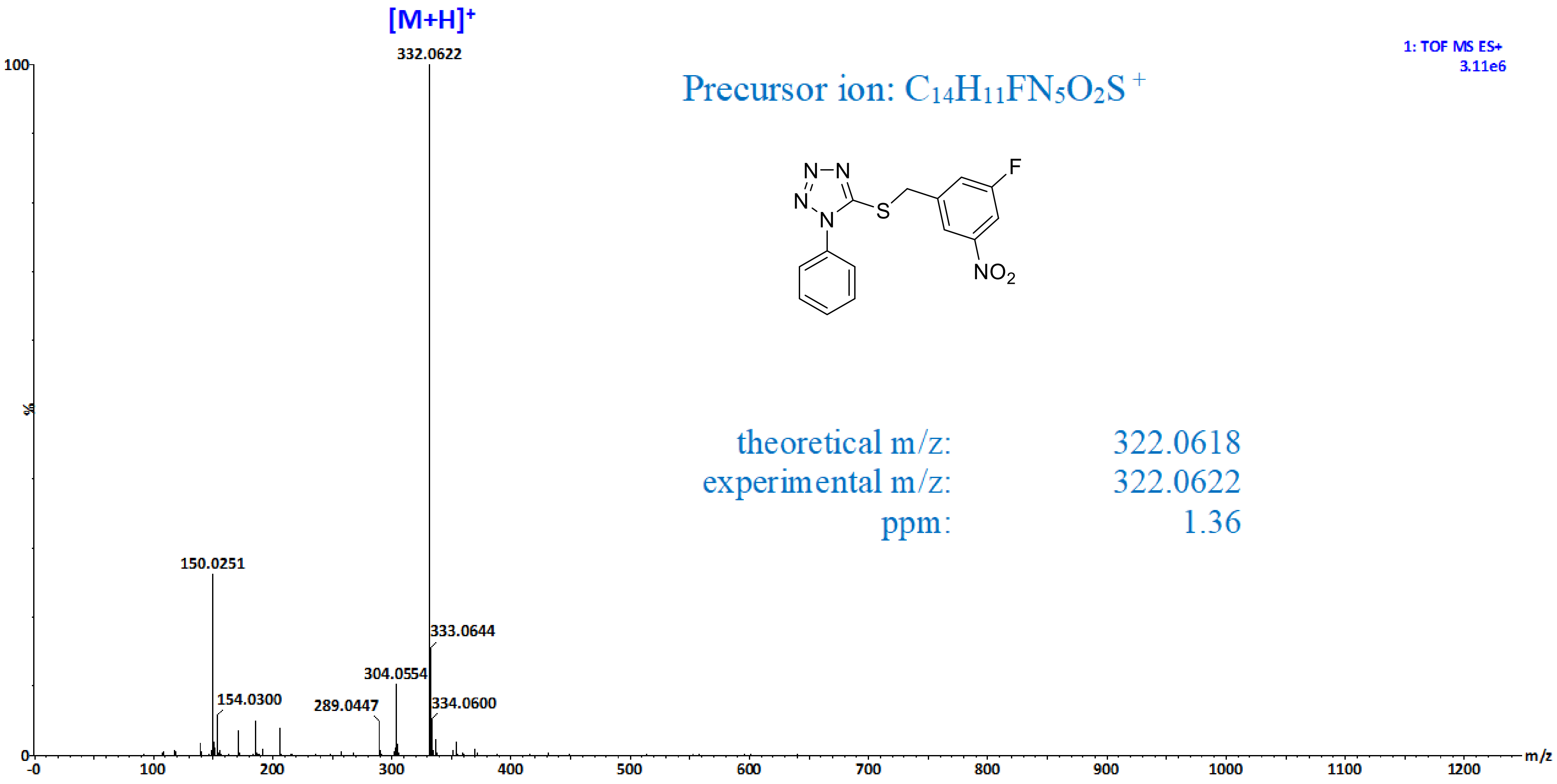

5-((3-Fluoro-5-nitrobenzyl)sulfanyl)-1-(4-methoxyphenyl)-1H-tetrazole (**54b**): ^{1}H NMR (600 MHz, DMSO-d_6)

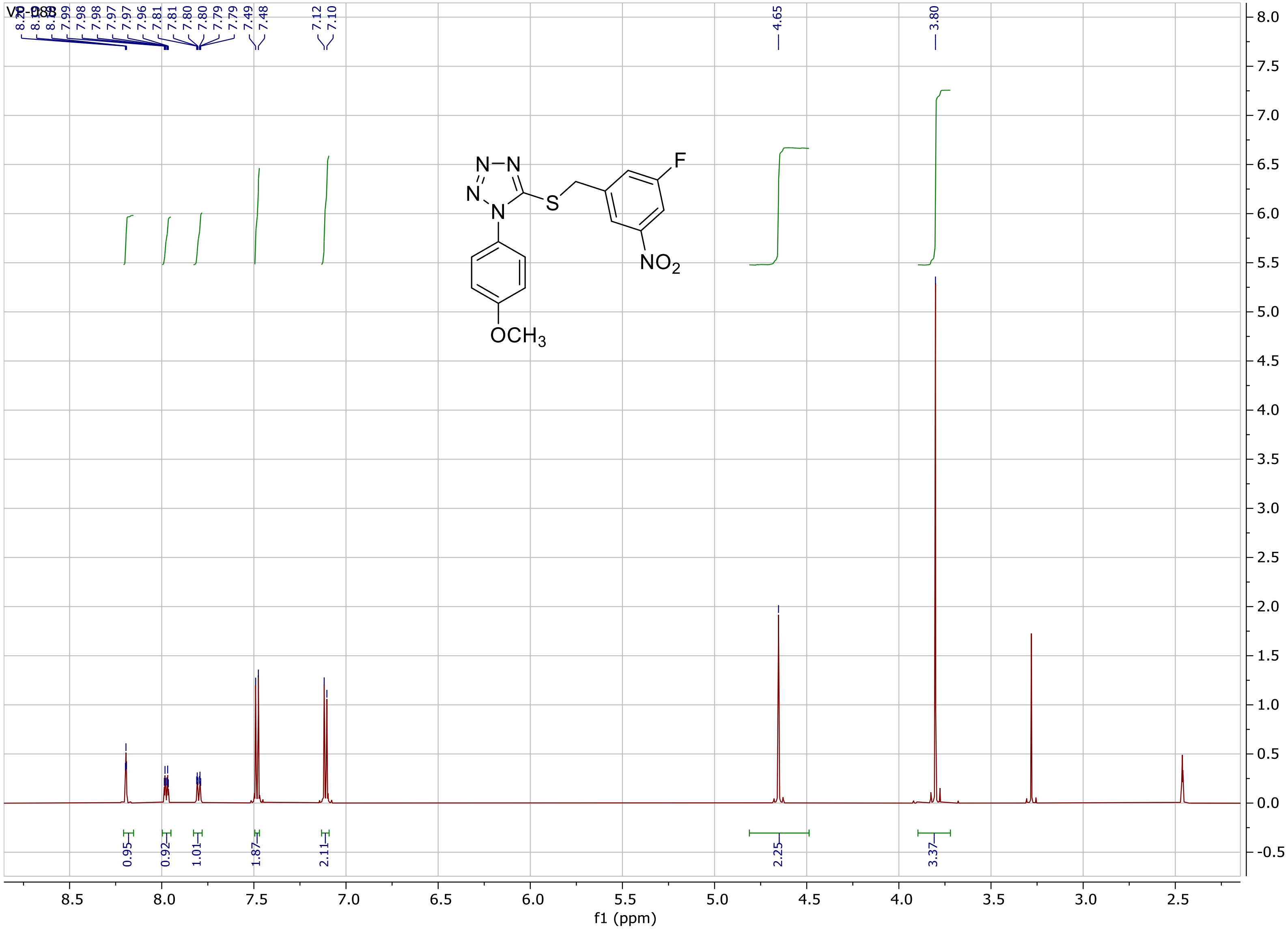

5-((3-Fluoro-5-nitrobenzyl)sulfanyl)-1-(4-methoxyphenyl)-1H-tetrazole (**54b**): ^{13}C NMR (151 MHz, DMSO-d_6)

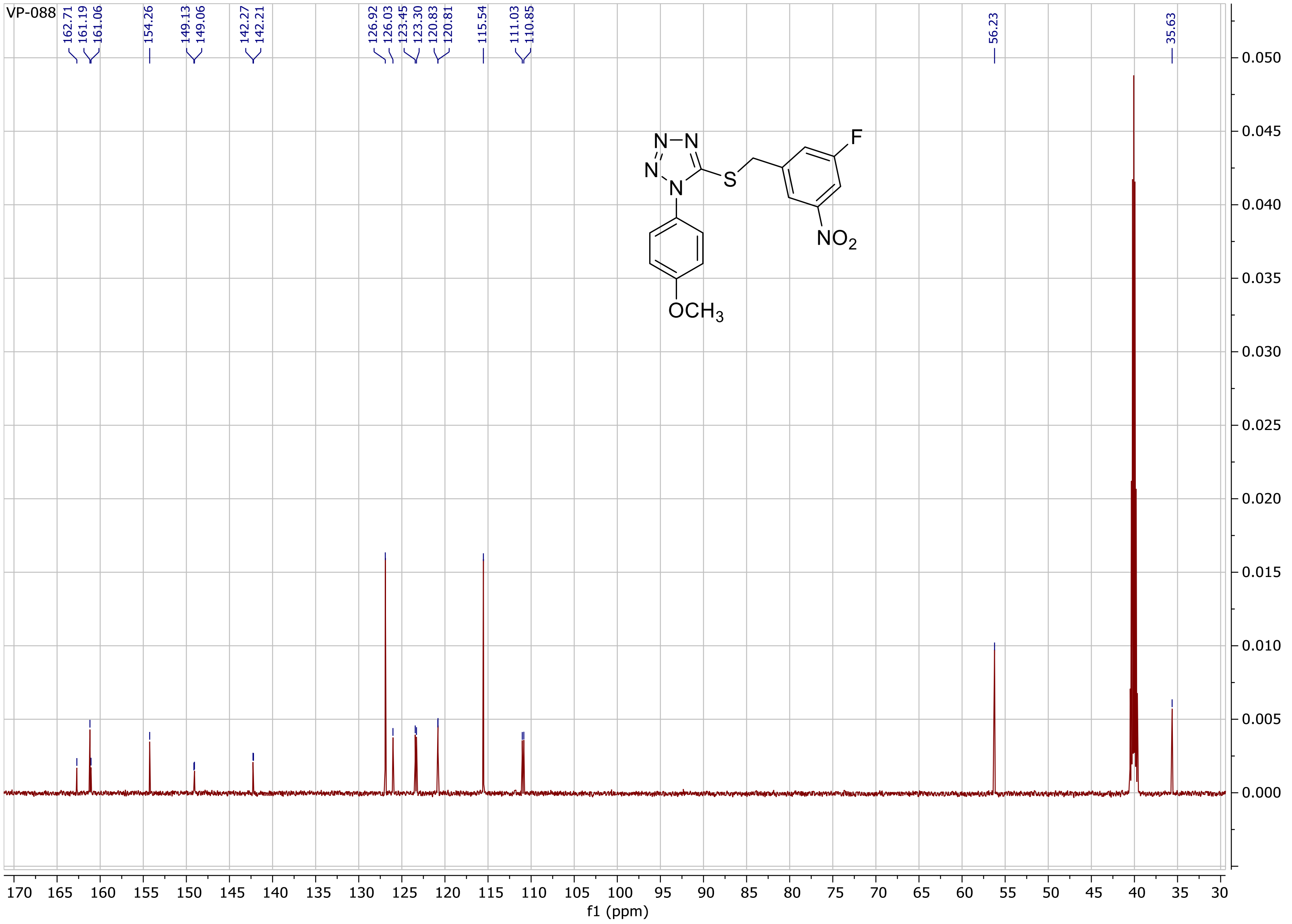

5-((3-Fluoro-5-nitrobenzyl)sulfanyl)-1-(4-methoxyphenyl)-1H-tetrazole (**54b**):

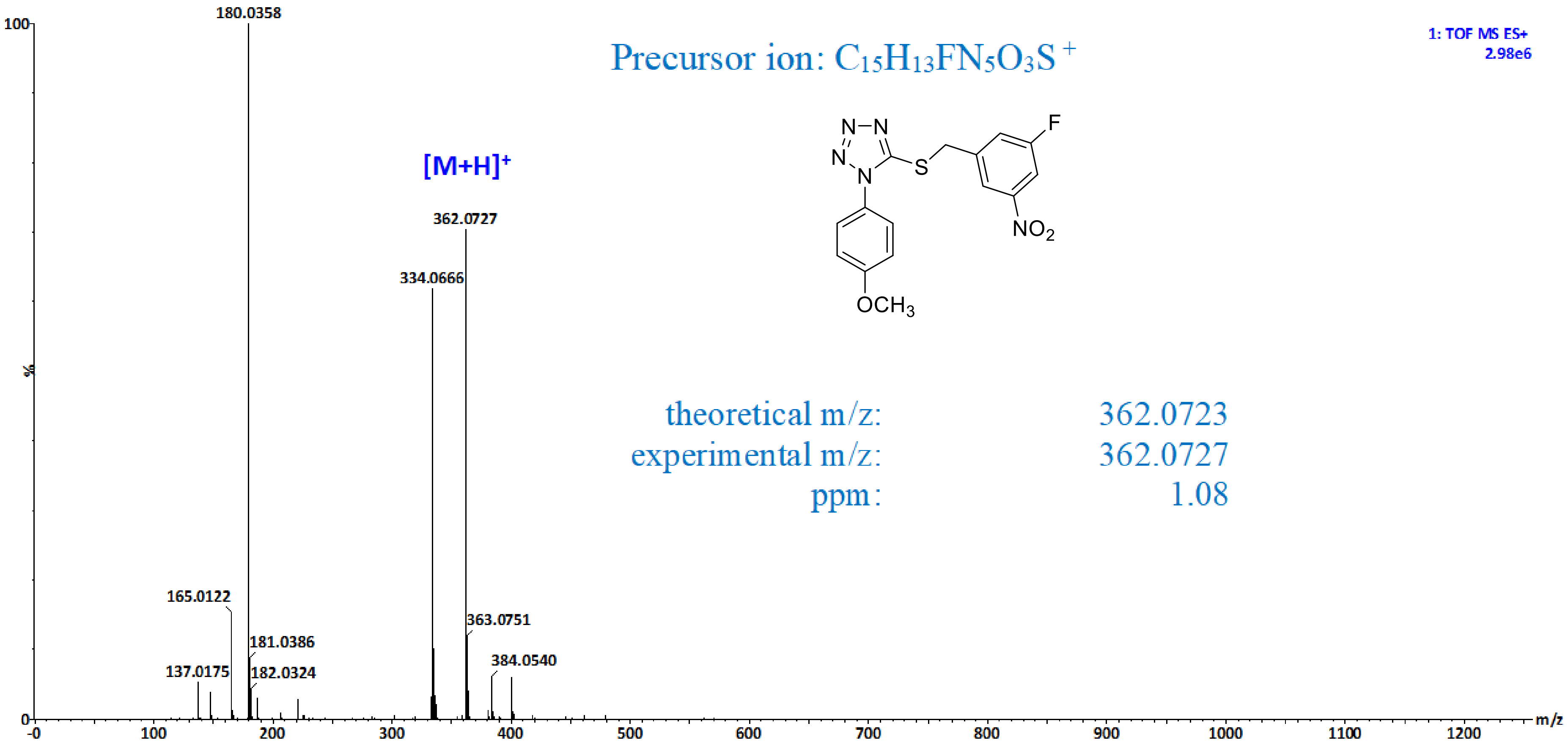

1-(4-Chlorophenyl)-5-((3-fluoro-5-nitrobenzyl)sulfanyl)-1H-tetrazole (**54c**): ^{1}H NMR (600 MHz, DMSO-d_6)

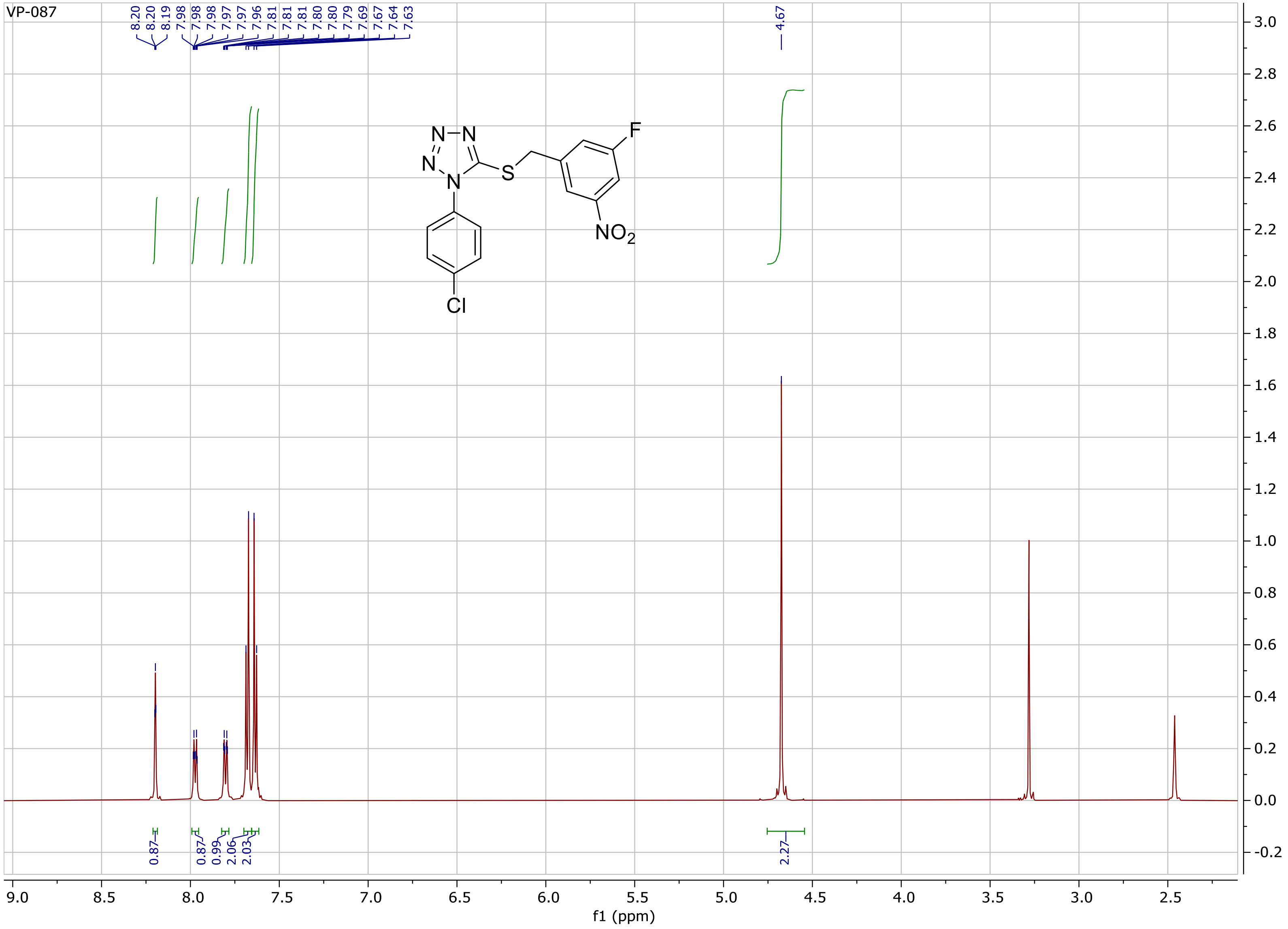

1-(4-Chlorophenyl)-5-((3-fluoro-5-nitrobenzyl)sulfanyl)-1H-tetrazole (**54c**): ^{13}C NMR (151 MHz, DMSO-d_6)

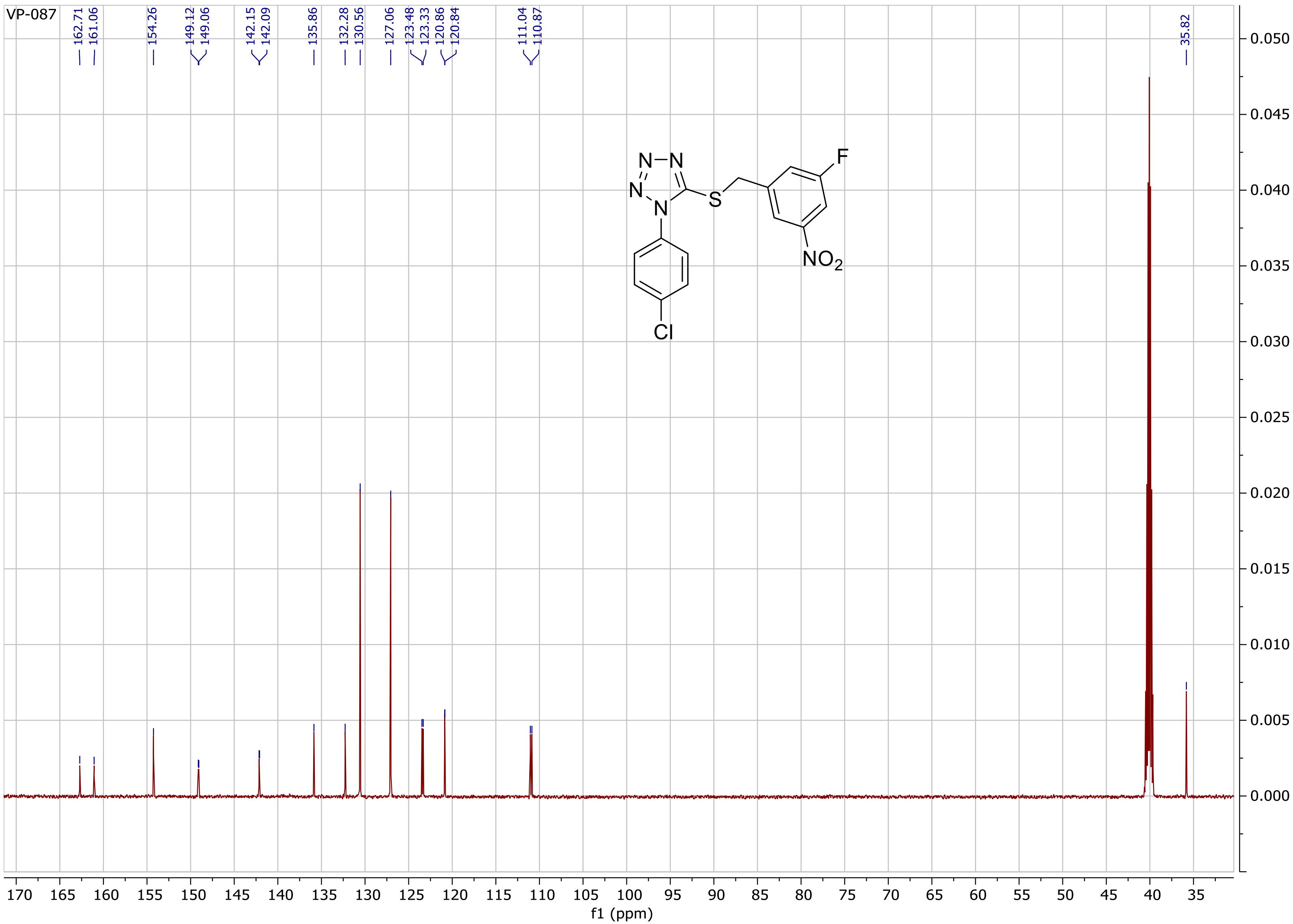

1-(4-Chlorophenyl)-5-((3-fluoro-5-nitrobenzyl)sulfanyl)-1H-tetrazole (**54c**):

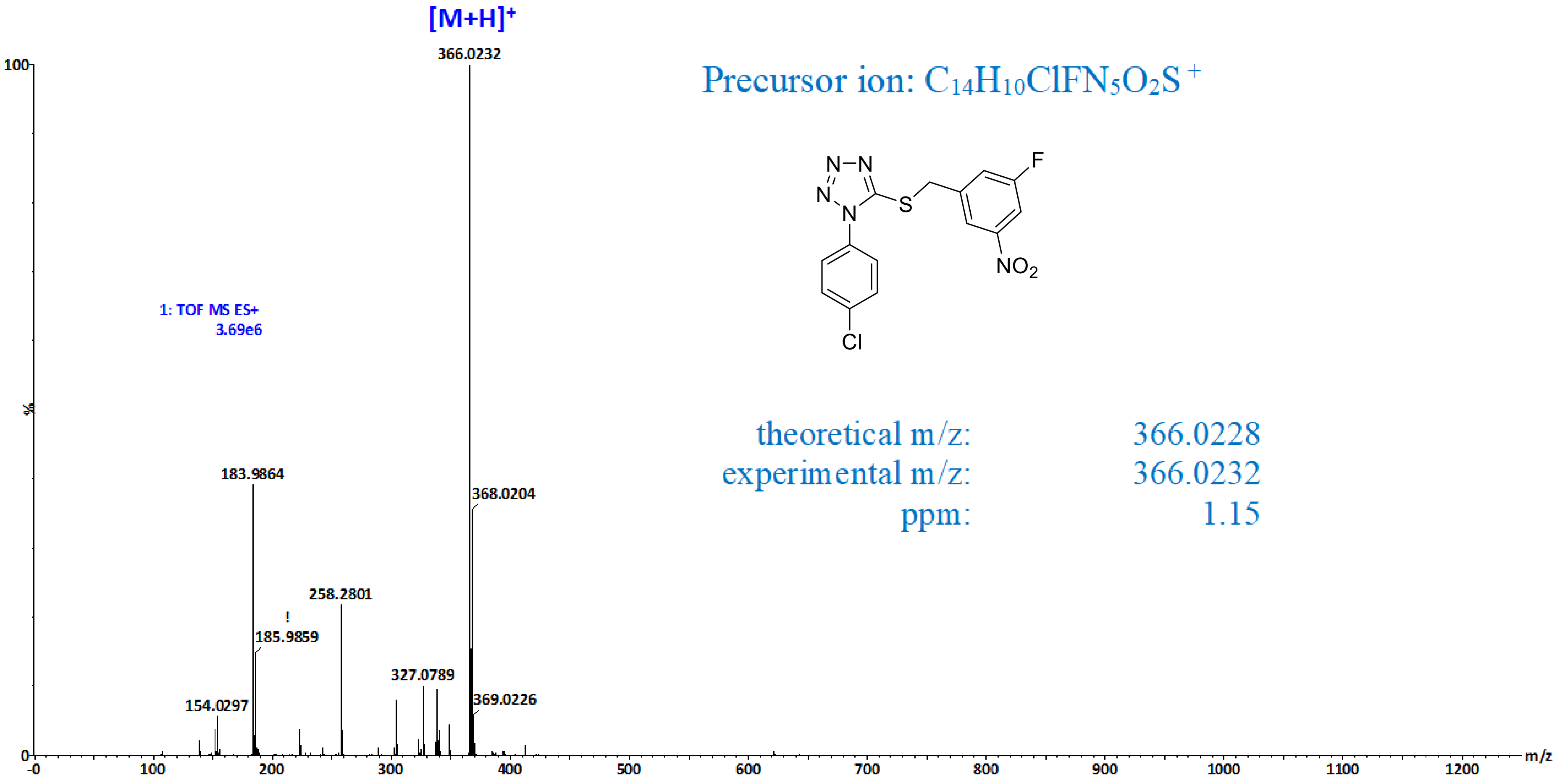

Precursor ion: $C_{14}H_{10}ClFN_5O_2S^+$

theoretical m/z:	366.0228
experimental m/z:	366.0232
ppm:	1.15

1-(4-Bromophenyl)-5-((3-fluoro-5-nitrobenzyl)sulfanyl)-1H-tetrazole (**54d**): ^{1}H NMR (600 MHz, DMSO-d_6)

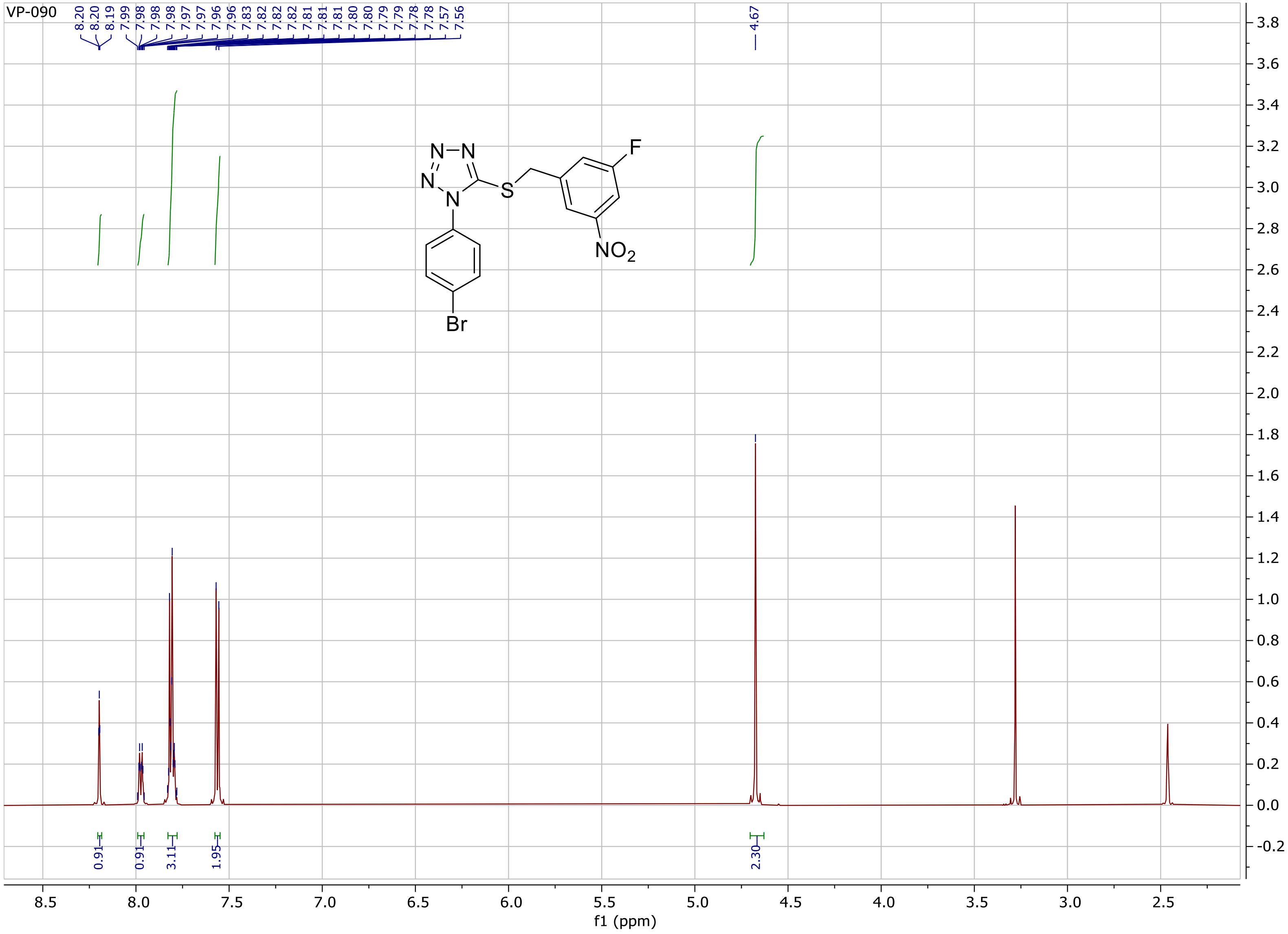

1-(4-Bromophenyl)-5-((3-fluoro-5-nitrobenzyl)sulfanyl)-1H-tetrazole (**54d**): ^{13}C NMR (151 MHz, DMSO-d_6)

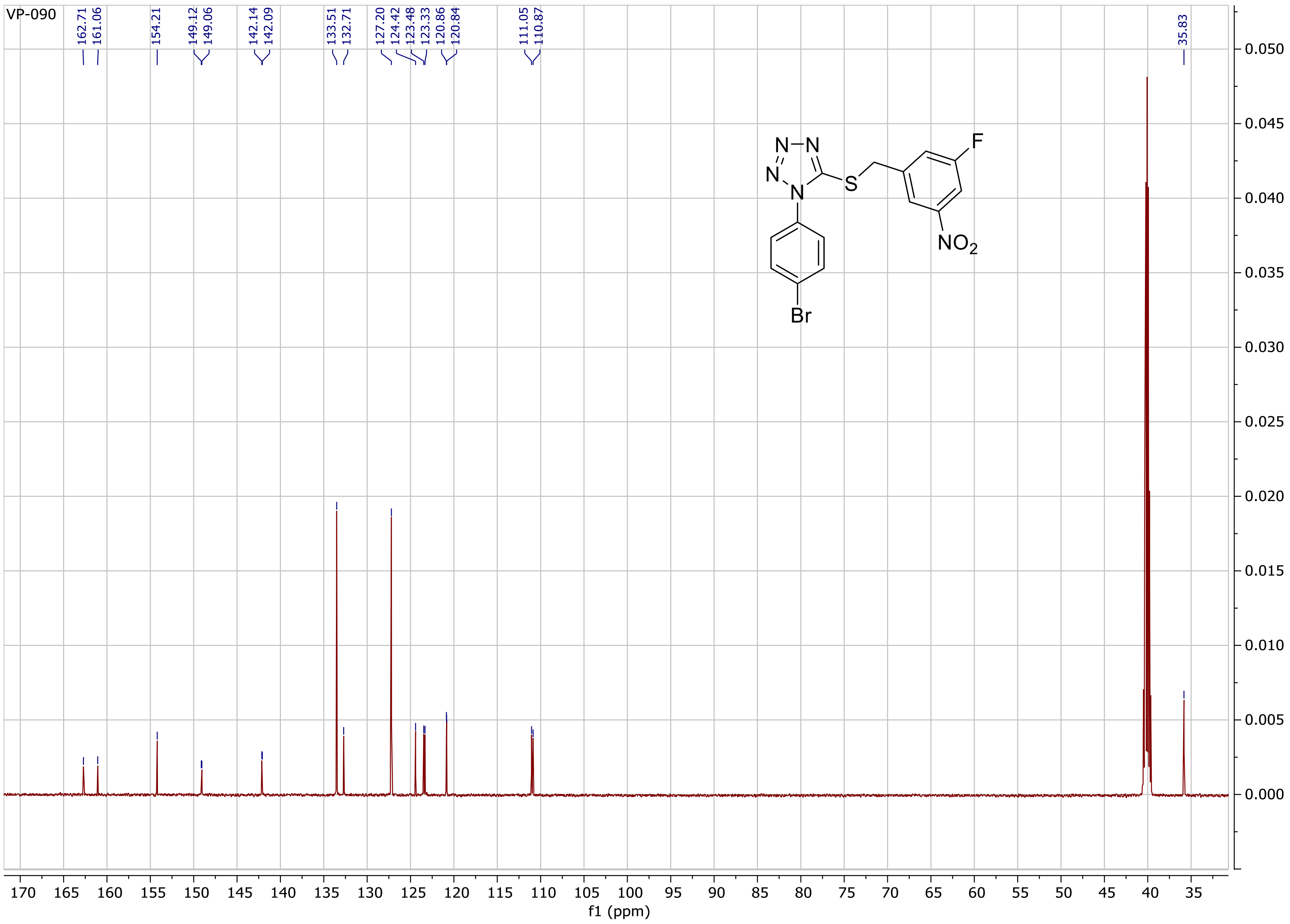

1-(4-Bromophenyl)-5-((3-fluoro-5-nitrobenzyl)sulfanyl)-1H-tetrazole (**54d**):

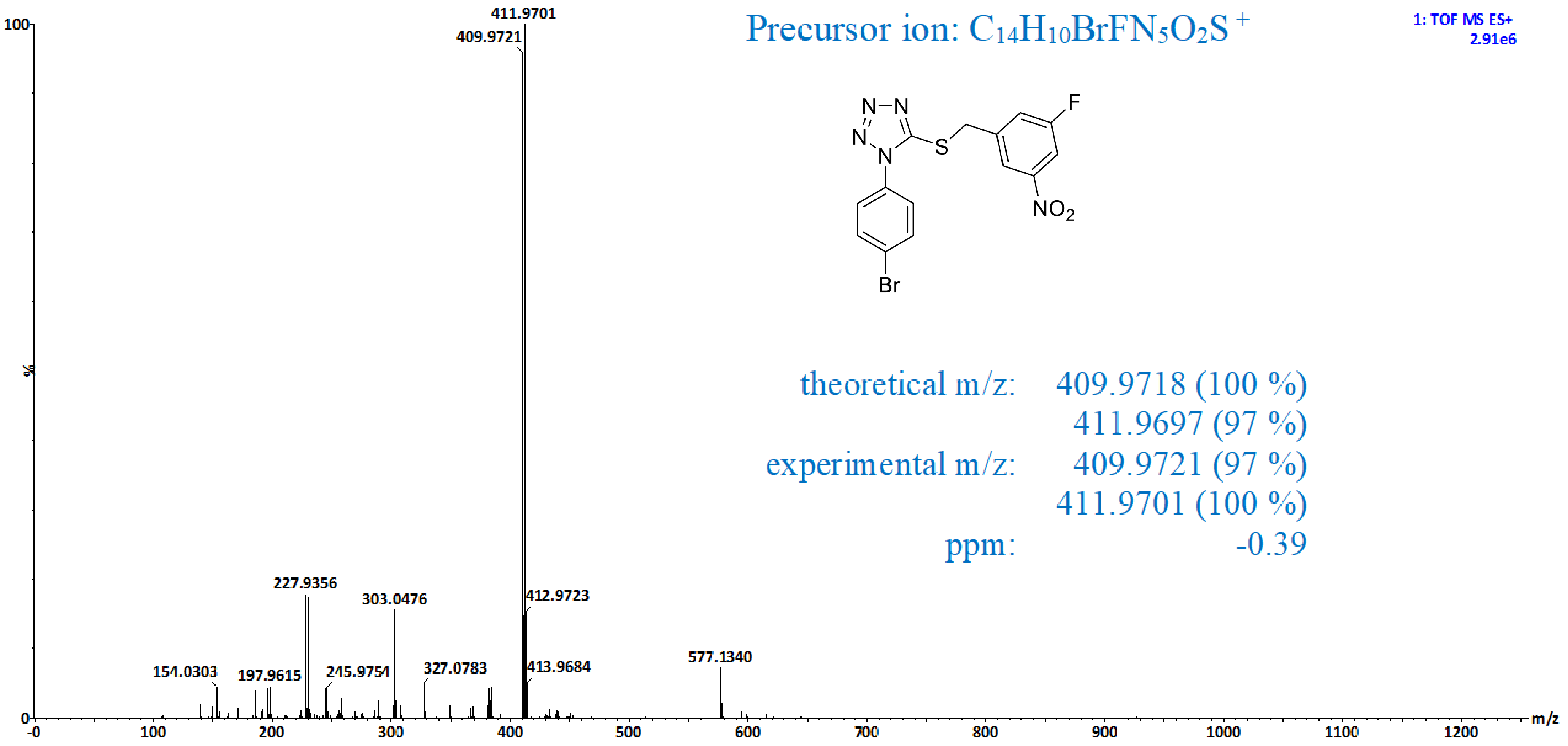

1-Cyclohexyl-5-((3-fluoro-5-nitrobenzyl)sulfanyl)-1H-tetrazole (**54e**): ^{1}H NMR (600 MHz, DMSO-d_6)

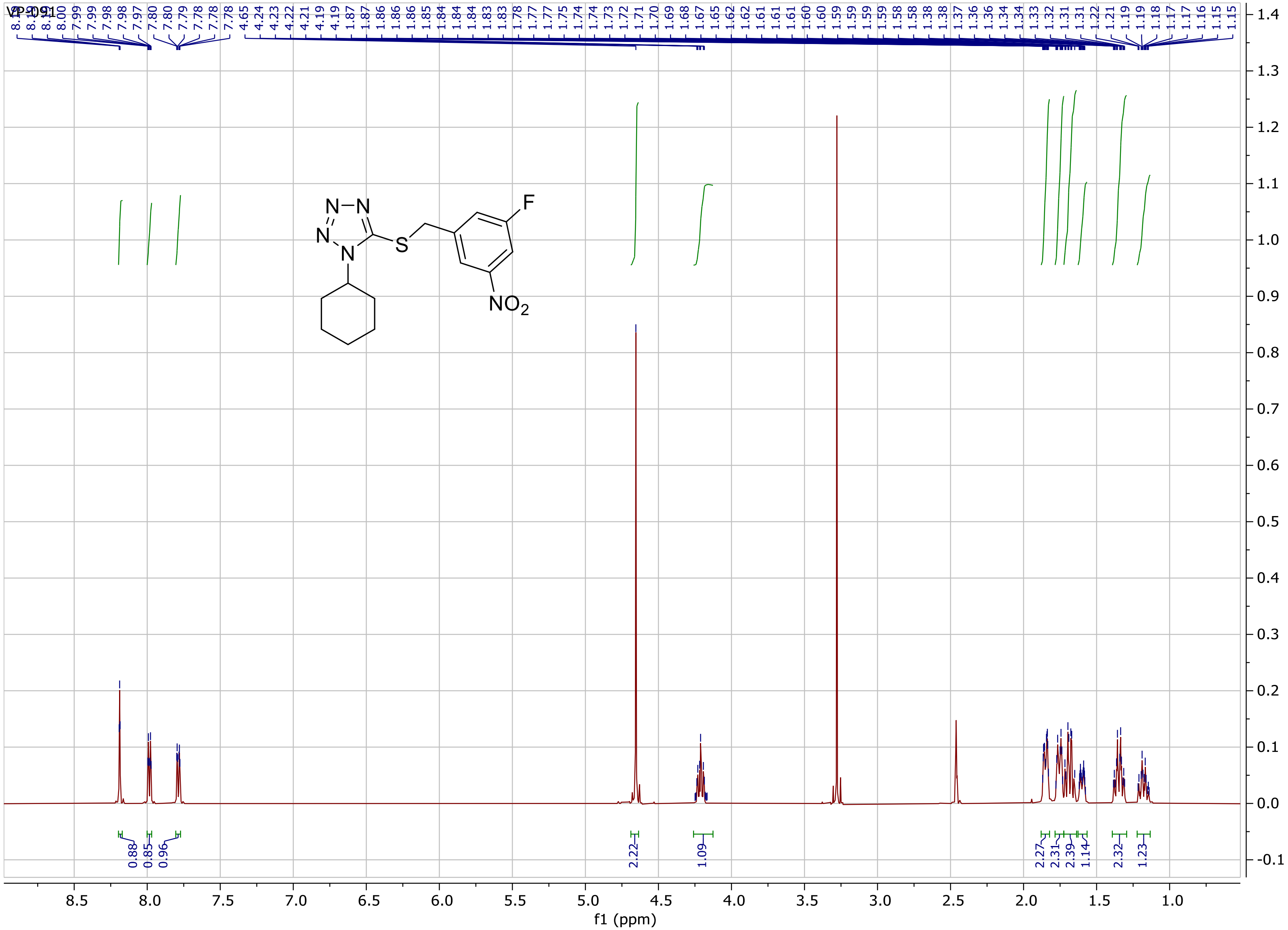

1-Cyclohexyl-5-((3-fluoro-5-nitrobenzyl)sulfanyl)-1H-tetrazole (**54e**): ^{13}C NMR (151 MHz, DMSO-d_6)

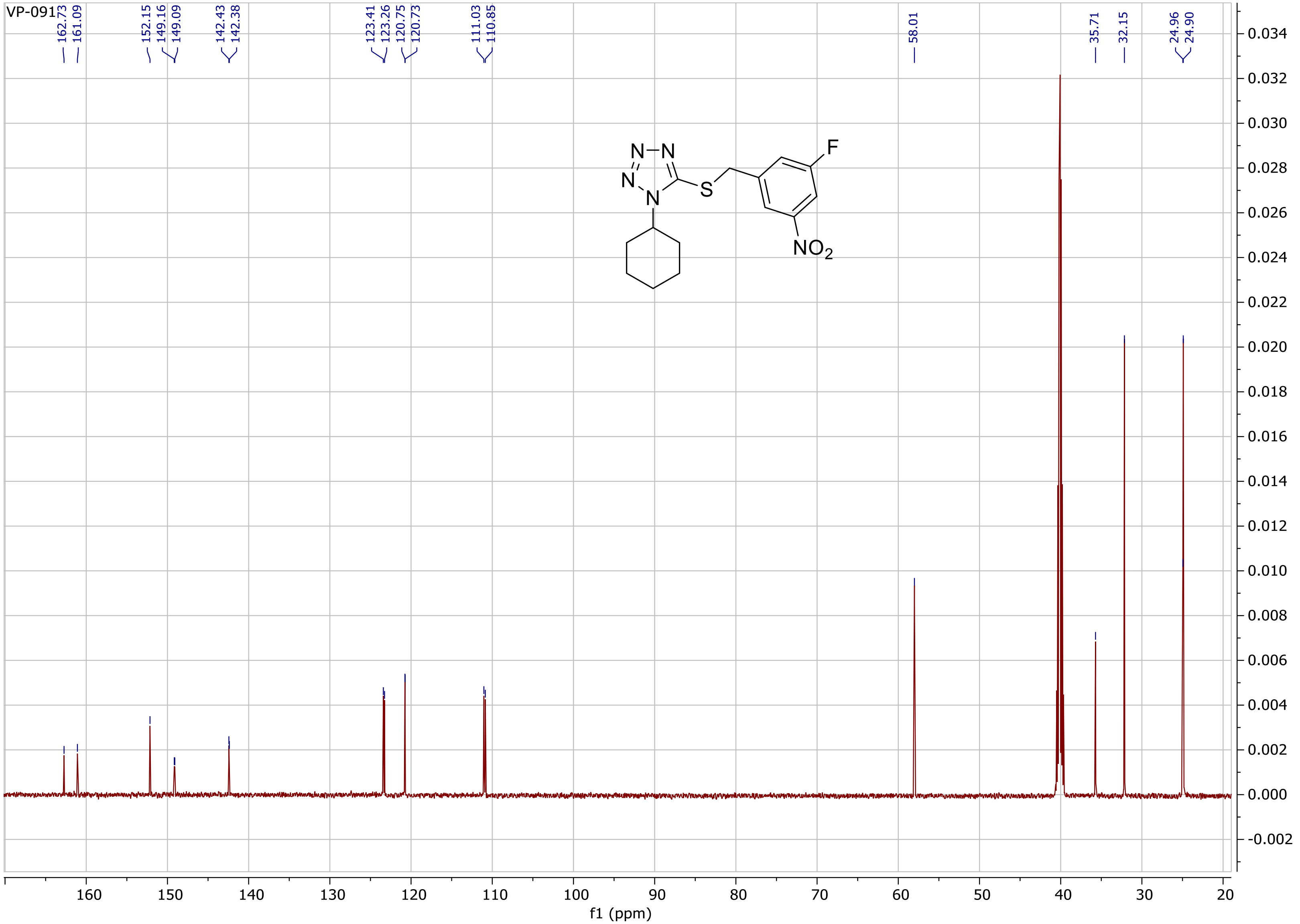

1-Cyclohexyl-5-((3-fluoro-5-nitrobenzyl)sulfanyl)-1H-tetrazole (**54e**):

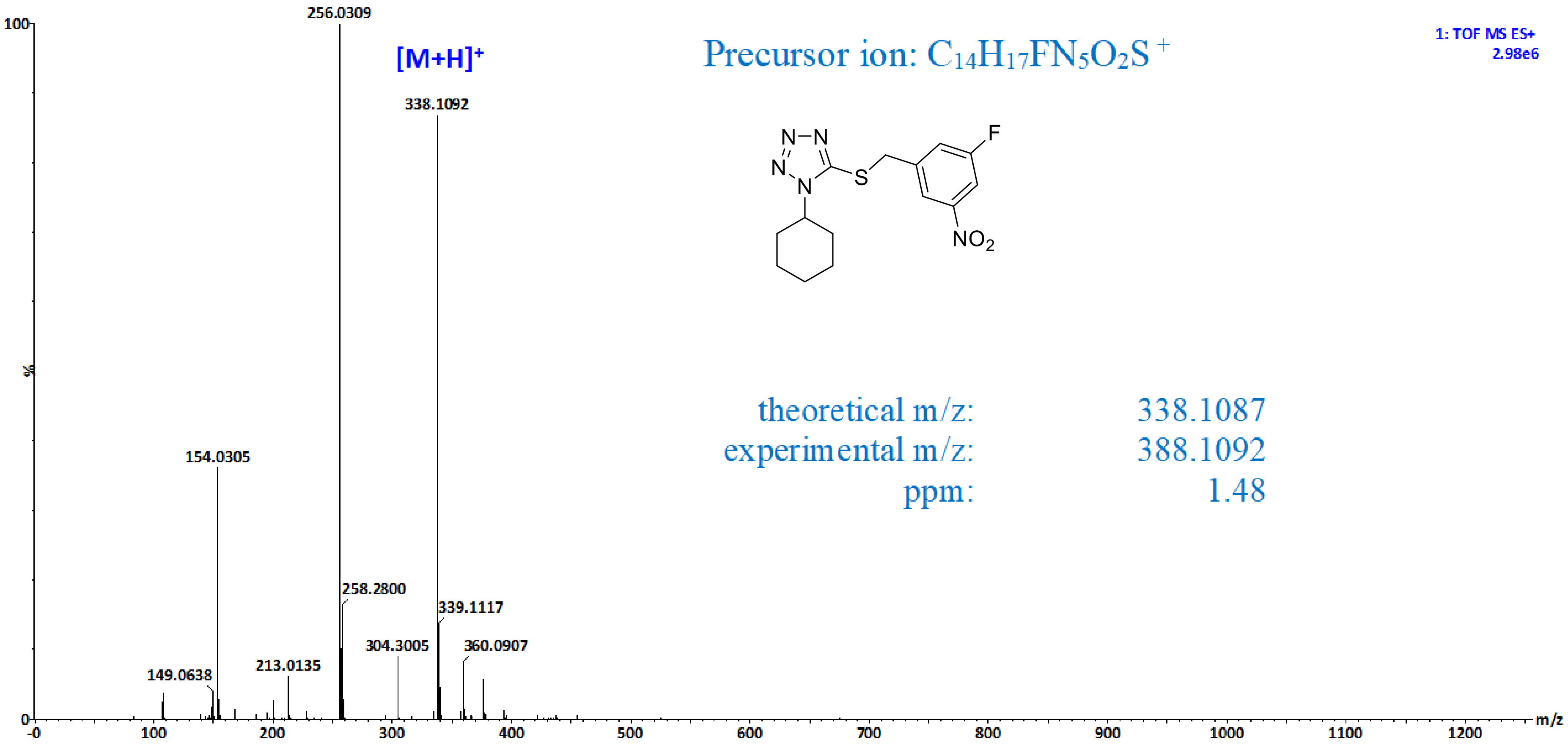

5-((3-Bromo-5-nitrobenzyl)sulfanyl)-1-phenyl-1H-tetrazole (**55a**): ^{1}H NMR (500 MHz, DMSO-d_6)

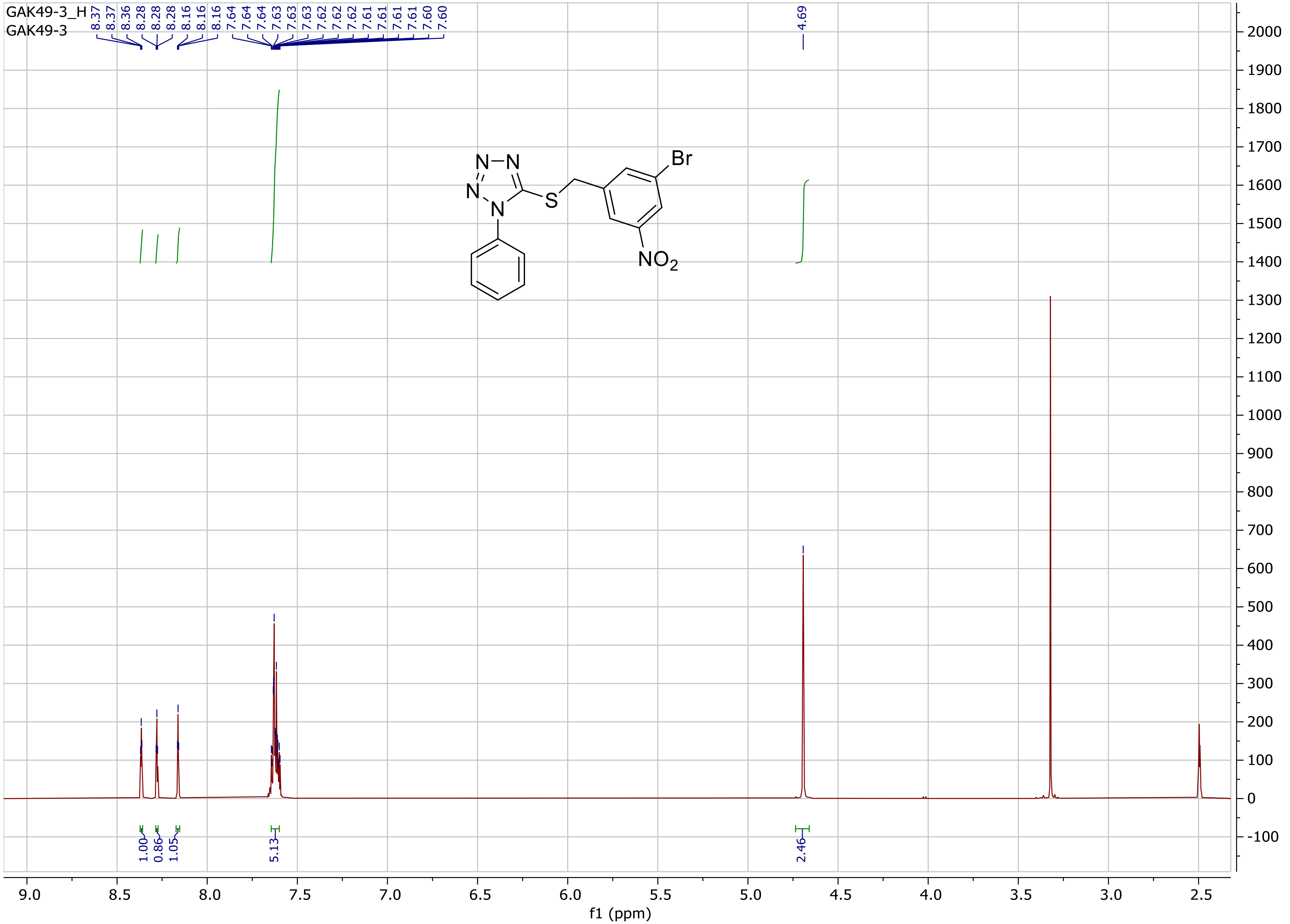

5-((3-Bromo-5-nitrobenzyl)sulfanyl)-1-phenyl-1H-tetrazole (**55a**): ^{13}C NMR (126 MHz, DMSO-d_6)

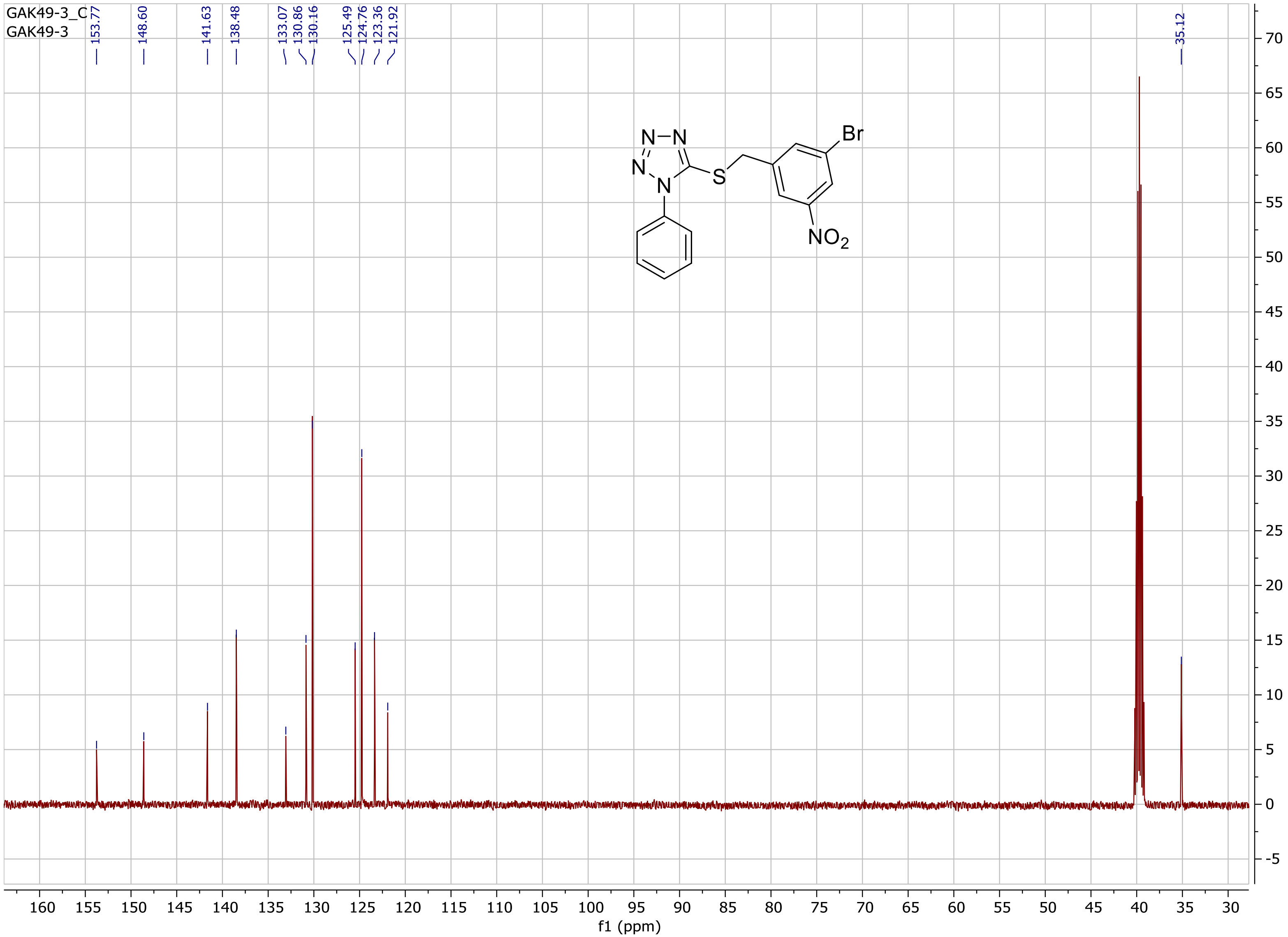

5-((3-Bromo-5-nitrobenzyl)sulfanyl)-1-(4-methoxyphenyl)-1H-tetrazole (**55b**): ^{1}H NMR (600 MHz, DMSO-d_6)

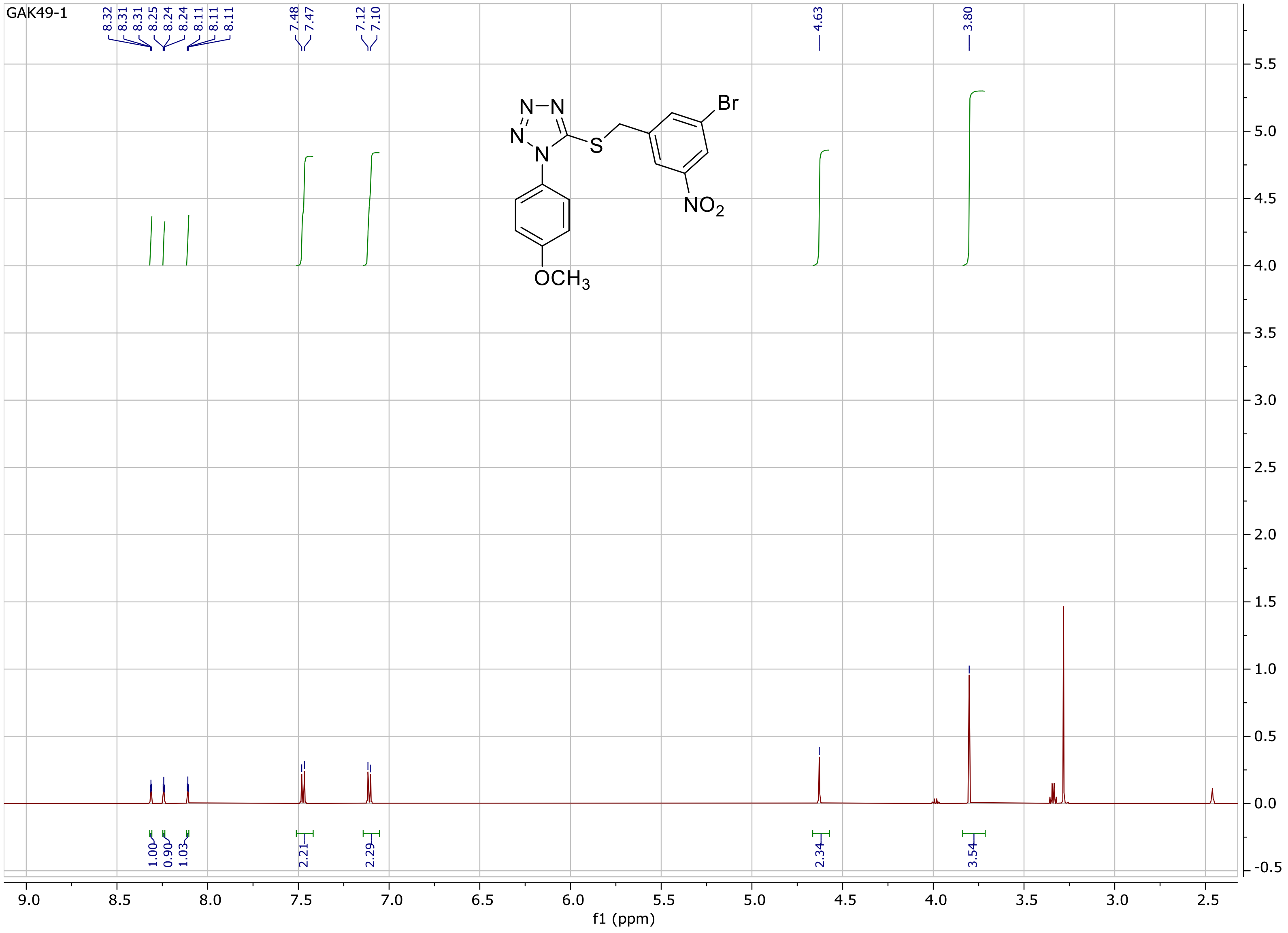

5-((3-Bromo-5-nitrobenzyl)sulfanyl)-1-(4-methoxyphenyl)-1H-tetrazole (**55b**): ^{13}C NMR (151 MHz, DMSO-d_6)

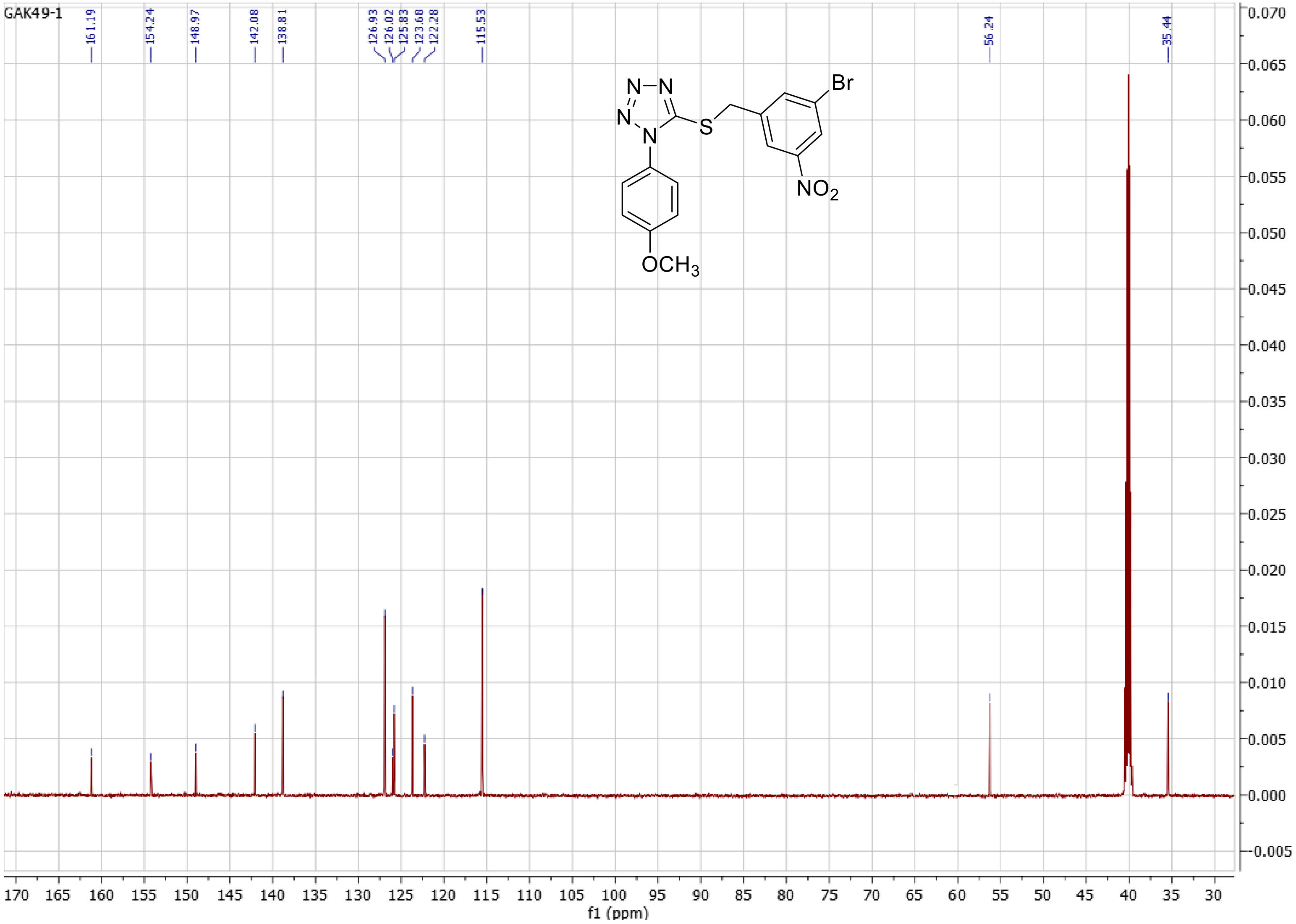

5-((3-Bromo-5-nitrobenzyl)sulfanyl)-1-(4-chlorophenyl)-1H-tetrazole (**55c**): ^{1}H NMR (600 MHz, DMSO-d_6)

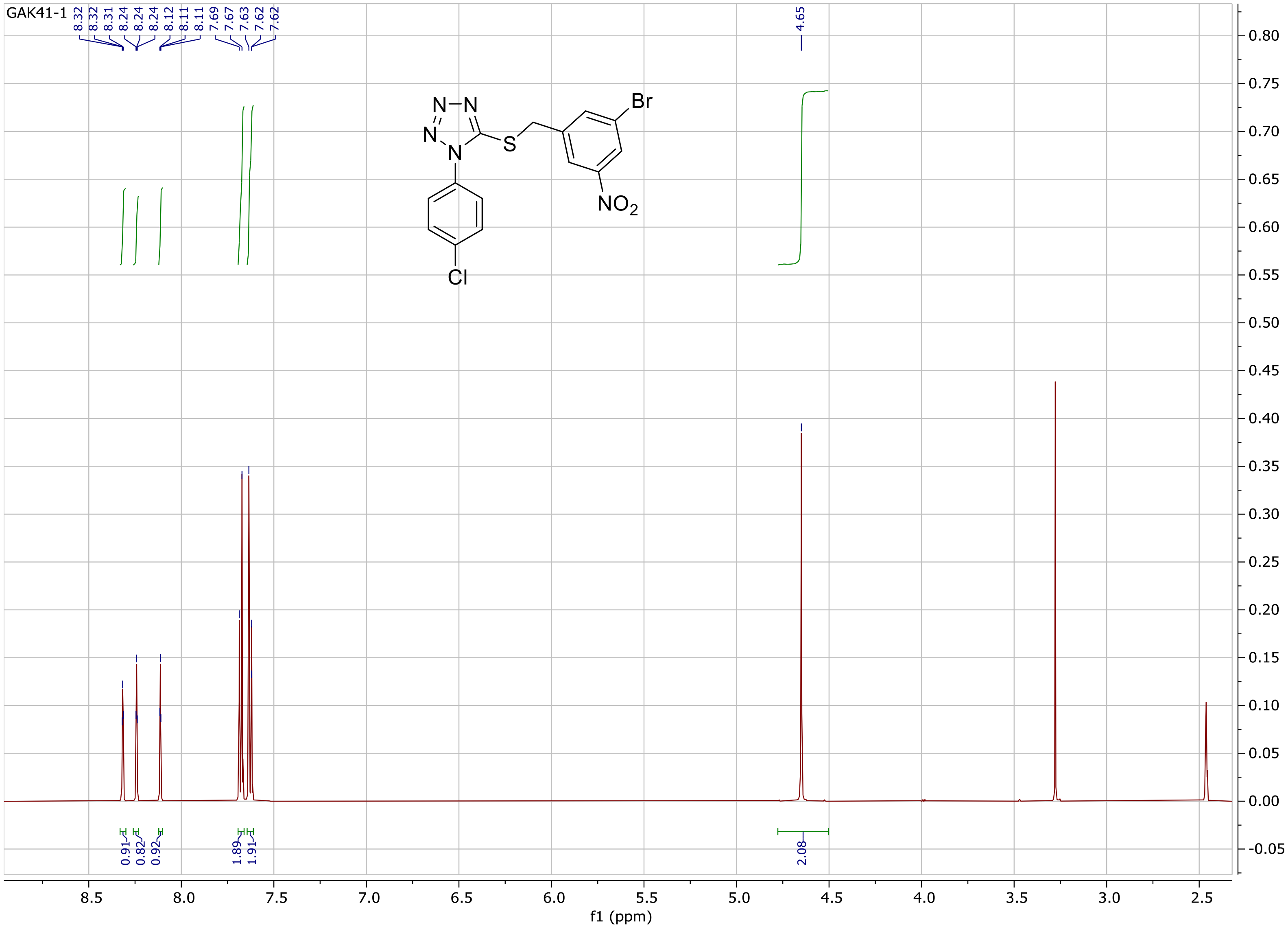

5-((3-Bromo-5-nitrobenzyl)sulfanyl)-1-(4-chlorophenyl)-1H-tetrazole (**55c**): ^{13}C NMR (151 MHz, DMSO-d_6)

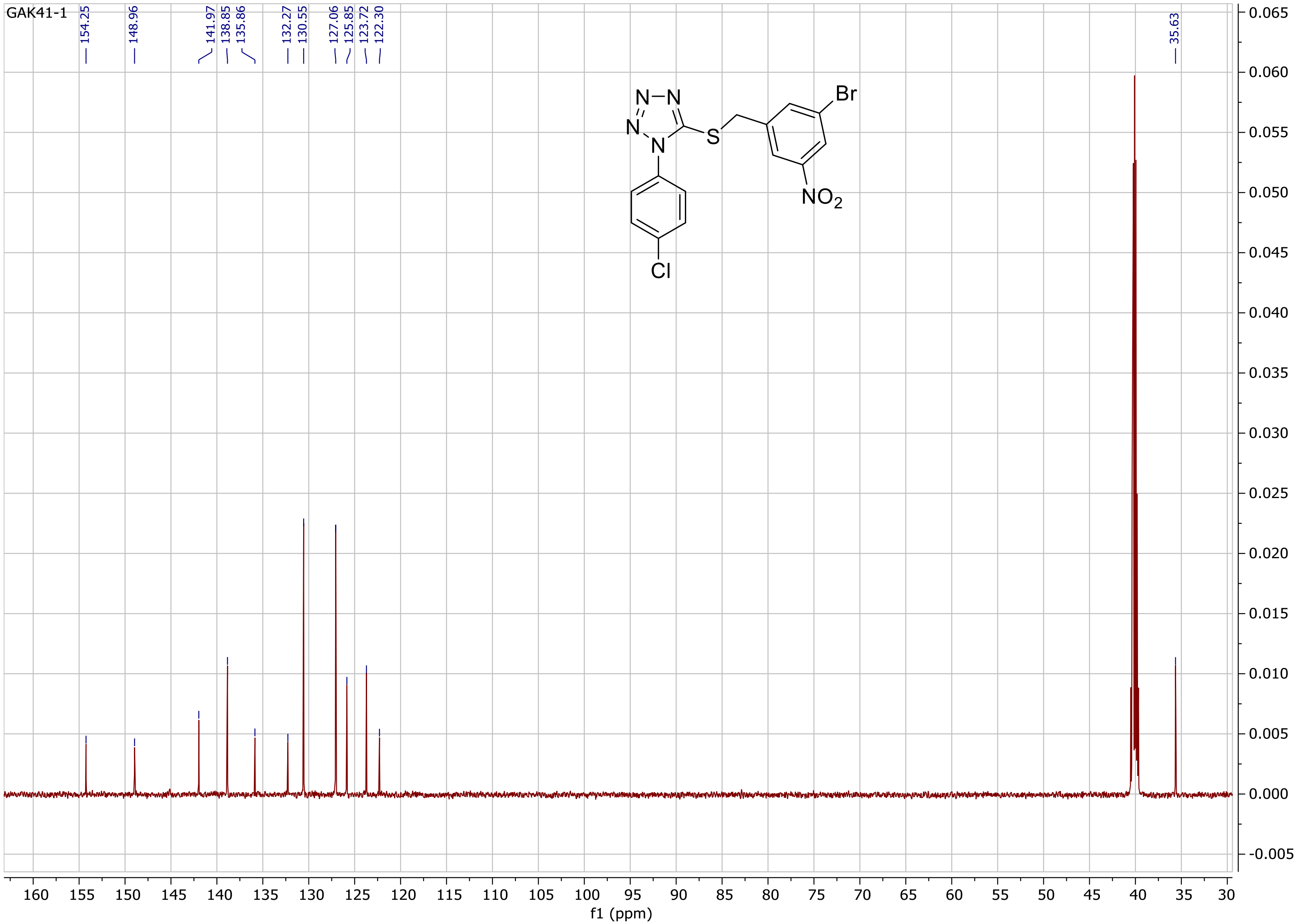

5-((3-Bromo-5-nitrobenzyl)sulfanyl)-1-(4-bromophenyl)-1H-tetrazole (**55d**): ^{1}H NMR (600 MHz, DMSO-d_6)

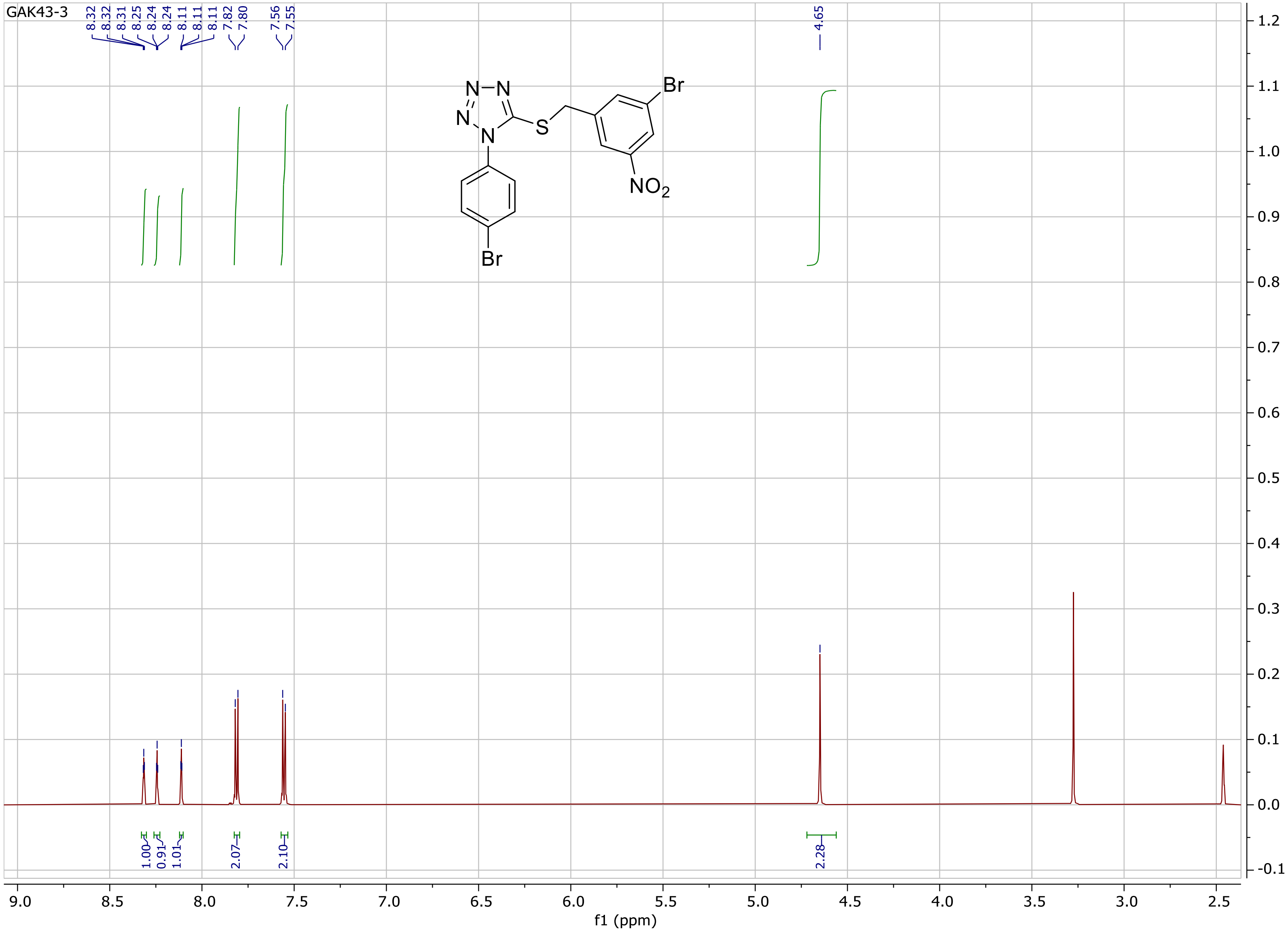

5-((3-Bromo-5-nitrobenzyl)sulfanyl)-1-(4-bromophenyl)-1H-tetrazole (**55d**): ^{13}C NMR (151 MHz, DMSO-d_6)

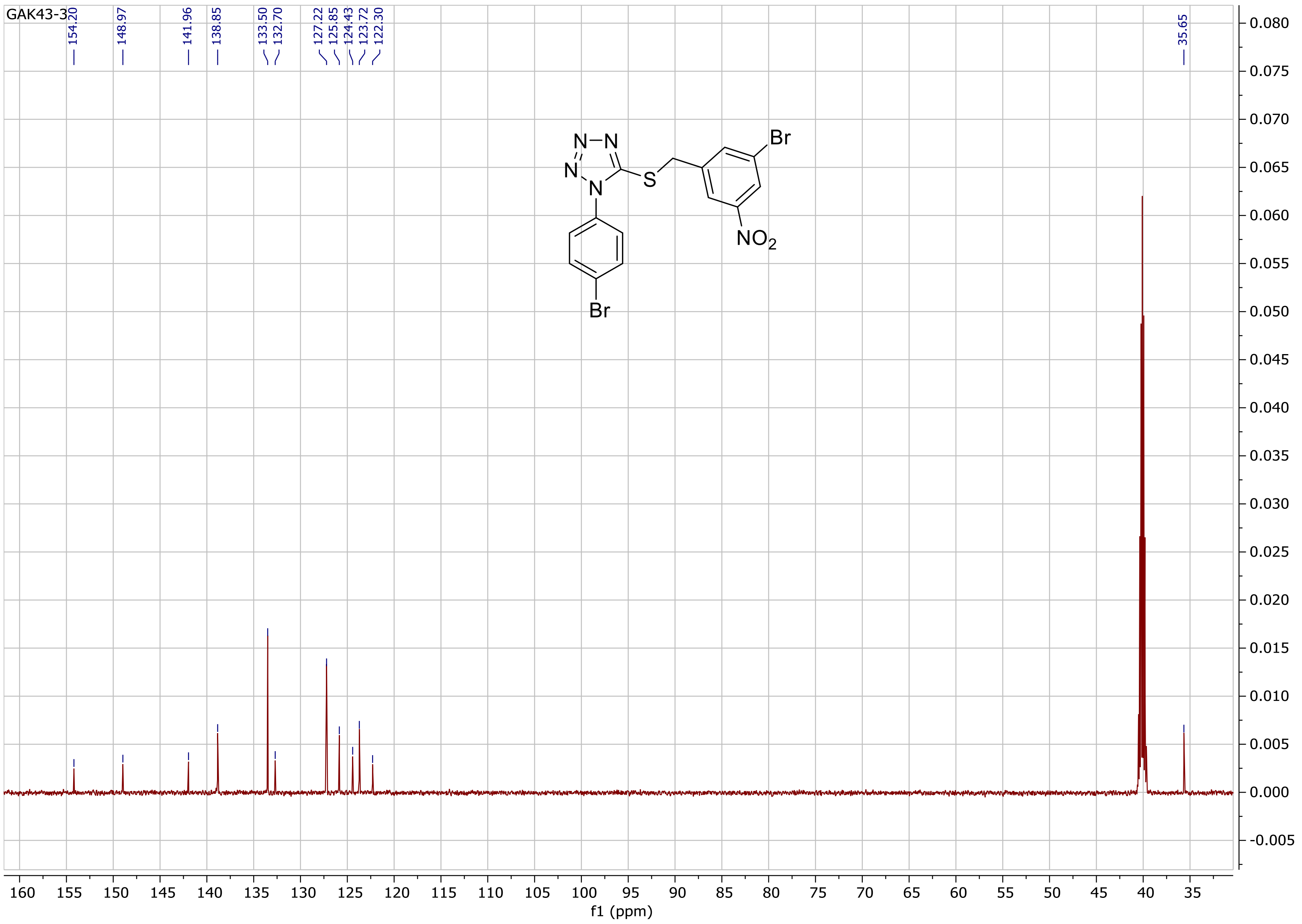

5-((3-Bromo-5-nitrobenzyl)sulfanyl)-1-cyclohexyl-1H-tetrazole (**55e**): ^{1}H NMR (600 MHz, DMSO-d_6)

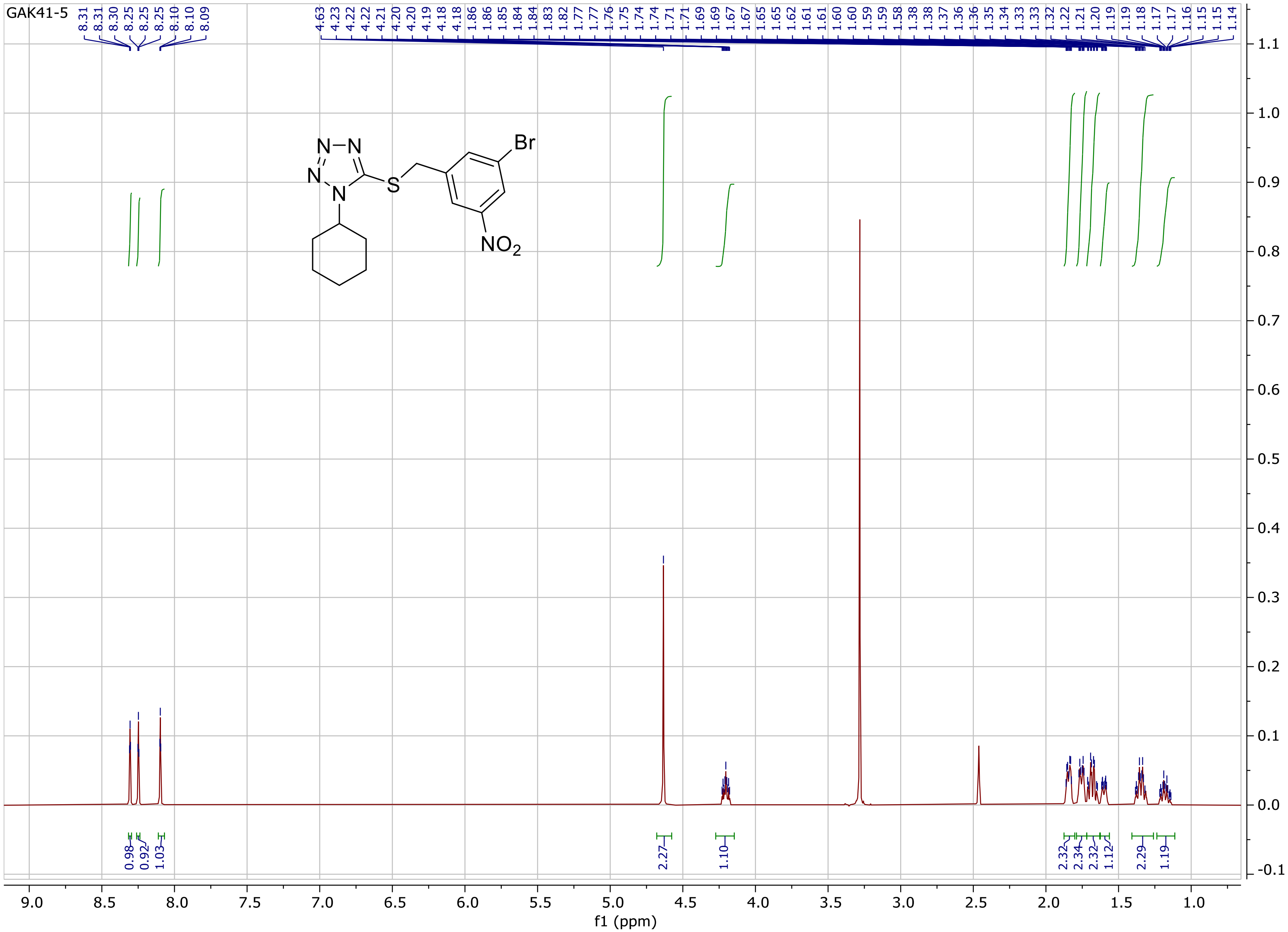

5-((3-Bromo-5-nitrobenzyl)sulfanyl)-1-cyclohexyl-1H-tetrazole (**55e**): ^{13}C NMR (151 MHz, DMSO-d_6)

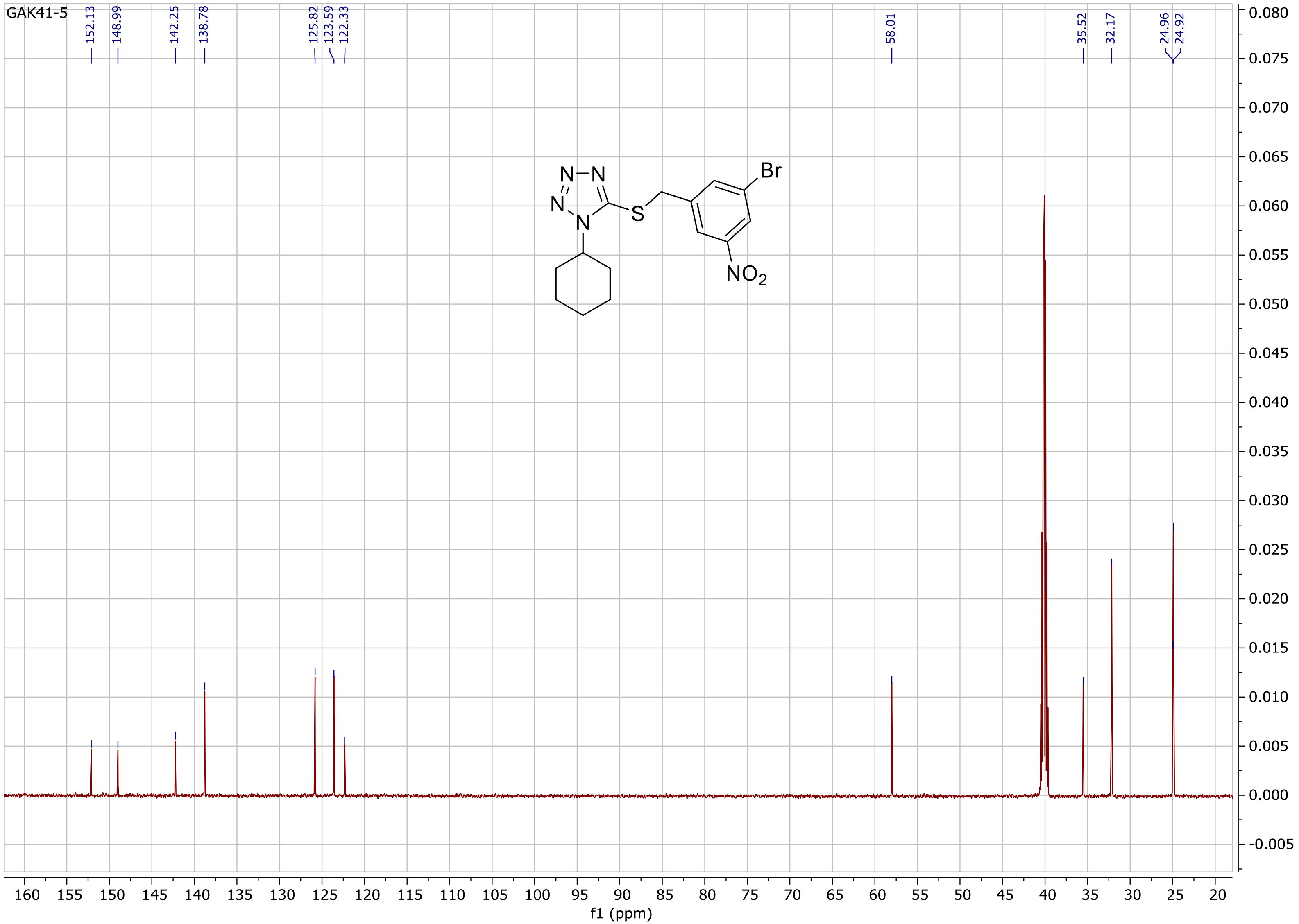

5-((3-Cyano-5-nitrobenzyl)sulfanyl)-1-phenyl-1H-tetrazole (**56a**): ^{1}H NMR (600 MHz, DMSO-d_6)

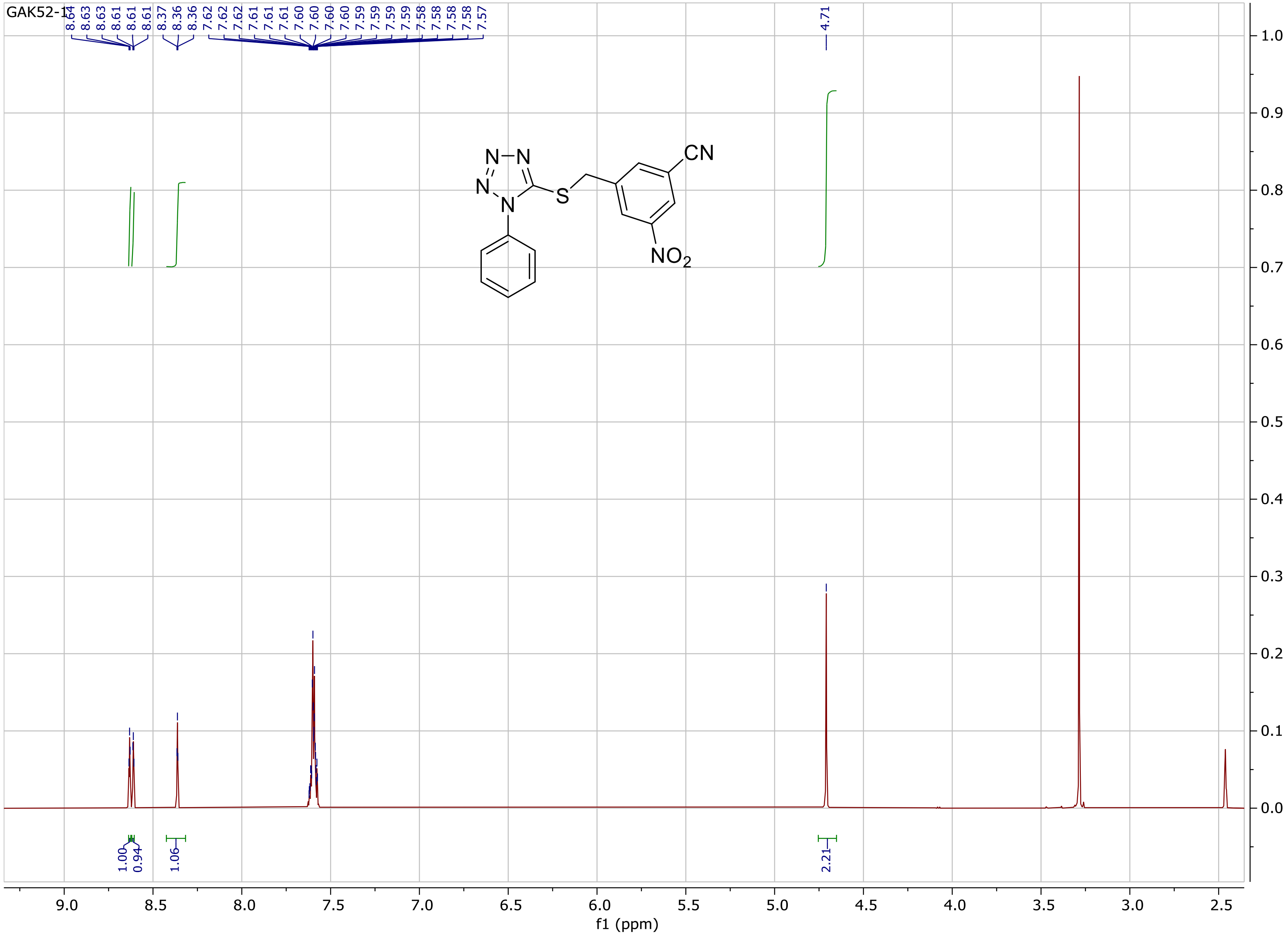

5-((3-Cyano-5-nitrobenzyl)sulfanyl)-1-phenyl-1H-tetrazole (**56a**): ^{13}C NMR (151 MHz, DMSO-d_6)

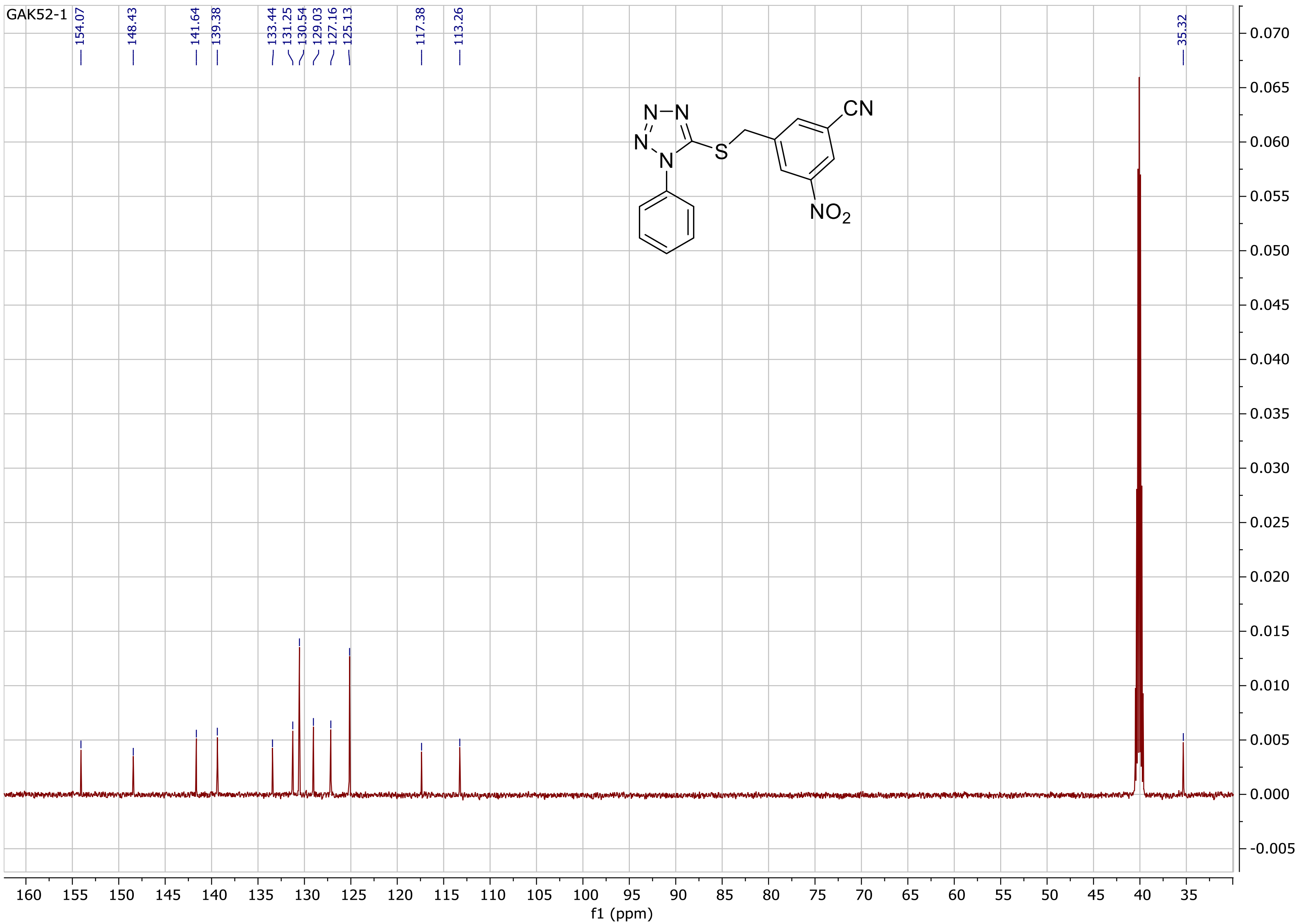

5-((3-Cyano-5-nitrobenzyl)sulfanyl)-1-(4-methoxyphenyl)-1H-tetrazole (**56b**): ^{1}H NMR (600 MHz, DMSO-d_6)

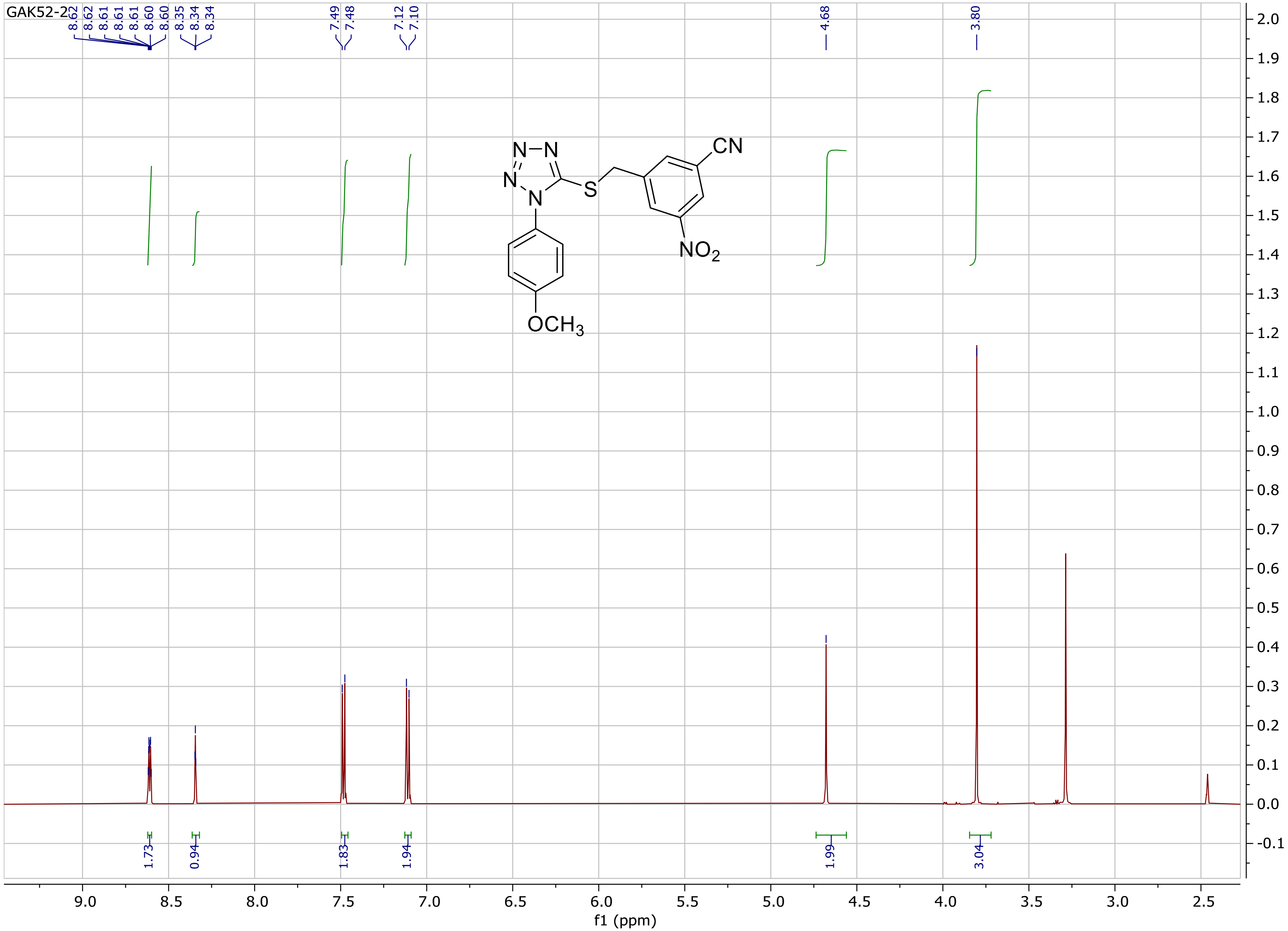

5-((3-Cyano-5-nitrobenzyl)sulfanyl)-1-(4-methoxyphenyl)-1H-tetrazole (**56b**): ^{13}C NMR (151 MHz, DMSO-d_6)

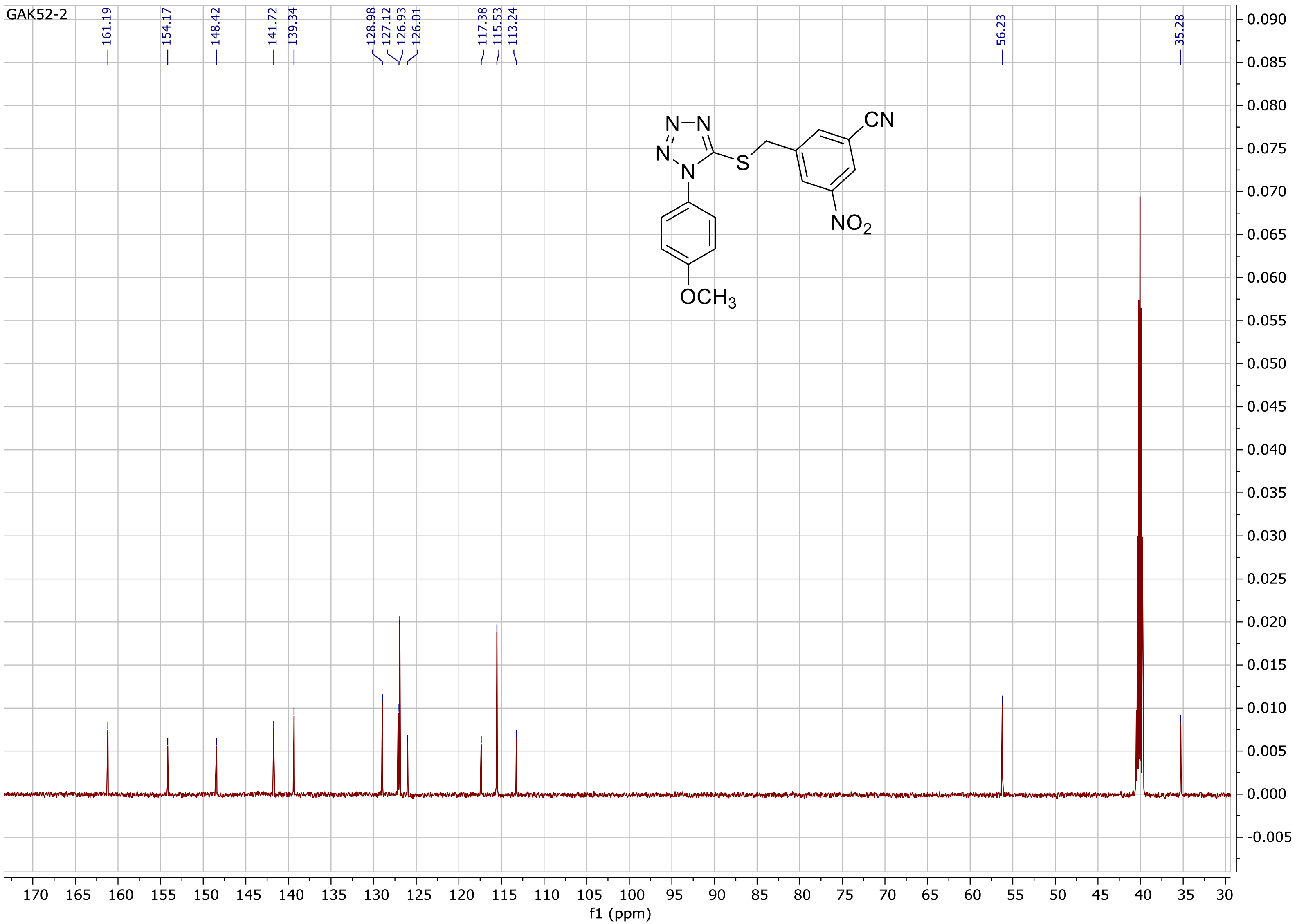

1-(4-Chlorophenyl)-5-((3-cyano-5-nitrobenzyl)sulfanyl)-1H-tetrazole (**56c**): ^{1}H NMR (600 MHz, DMSO-d_6)

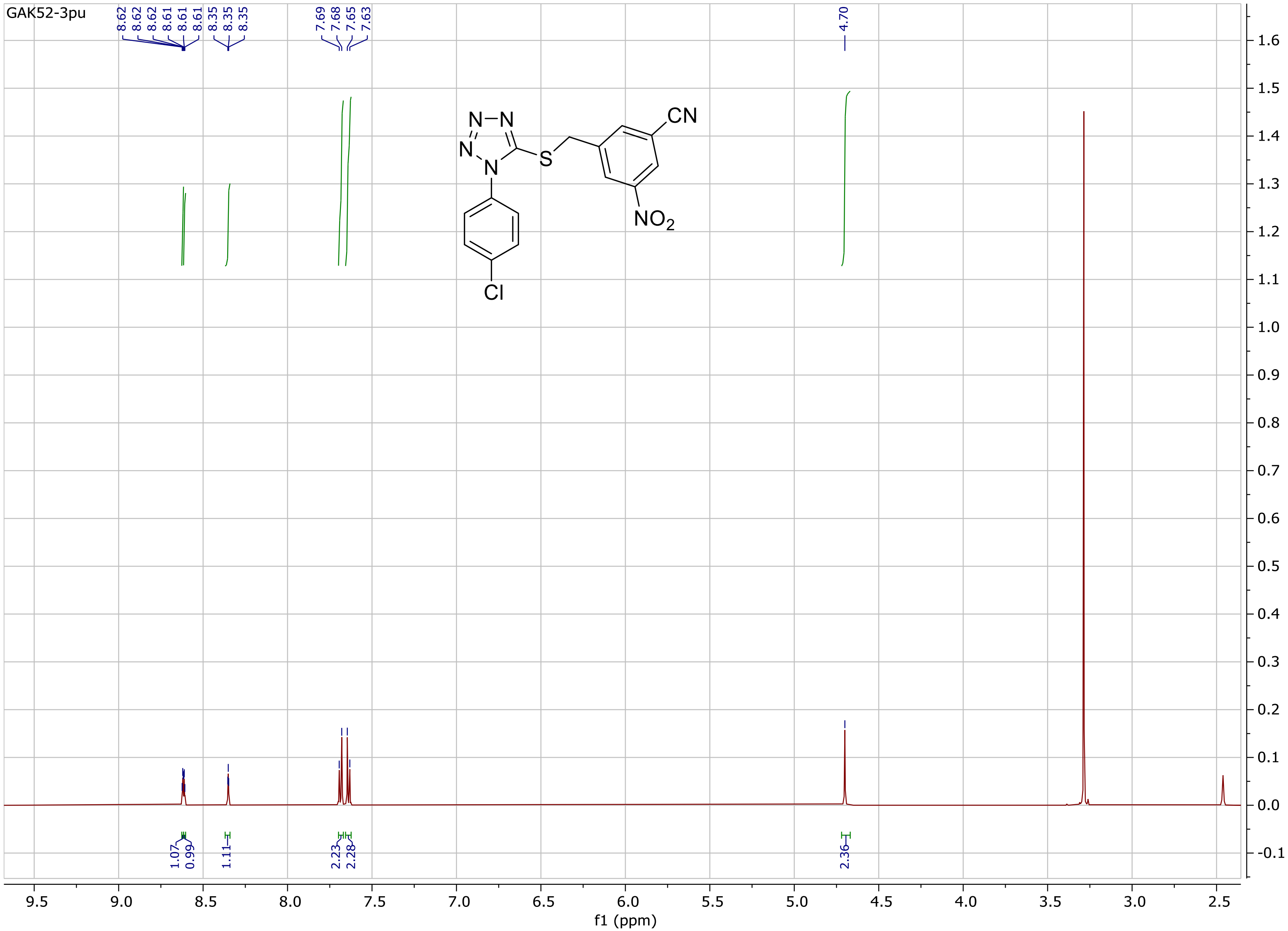

1-(4-Chlorophenyl)-5-((3-cyano-5-nitrobenzyl)sulfanyl)-1H-tetrazole (**56c**): ^{13}C NMR (151 MHz, DMSO-d_6)

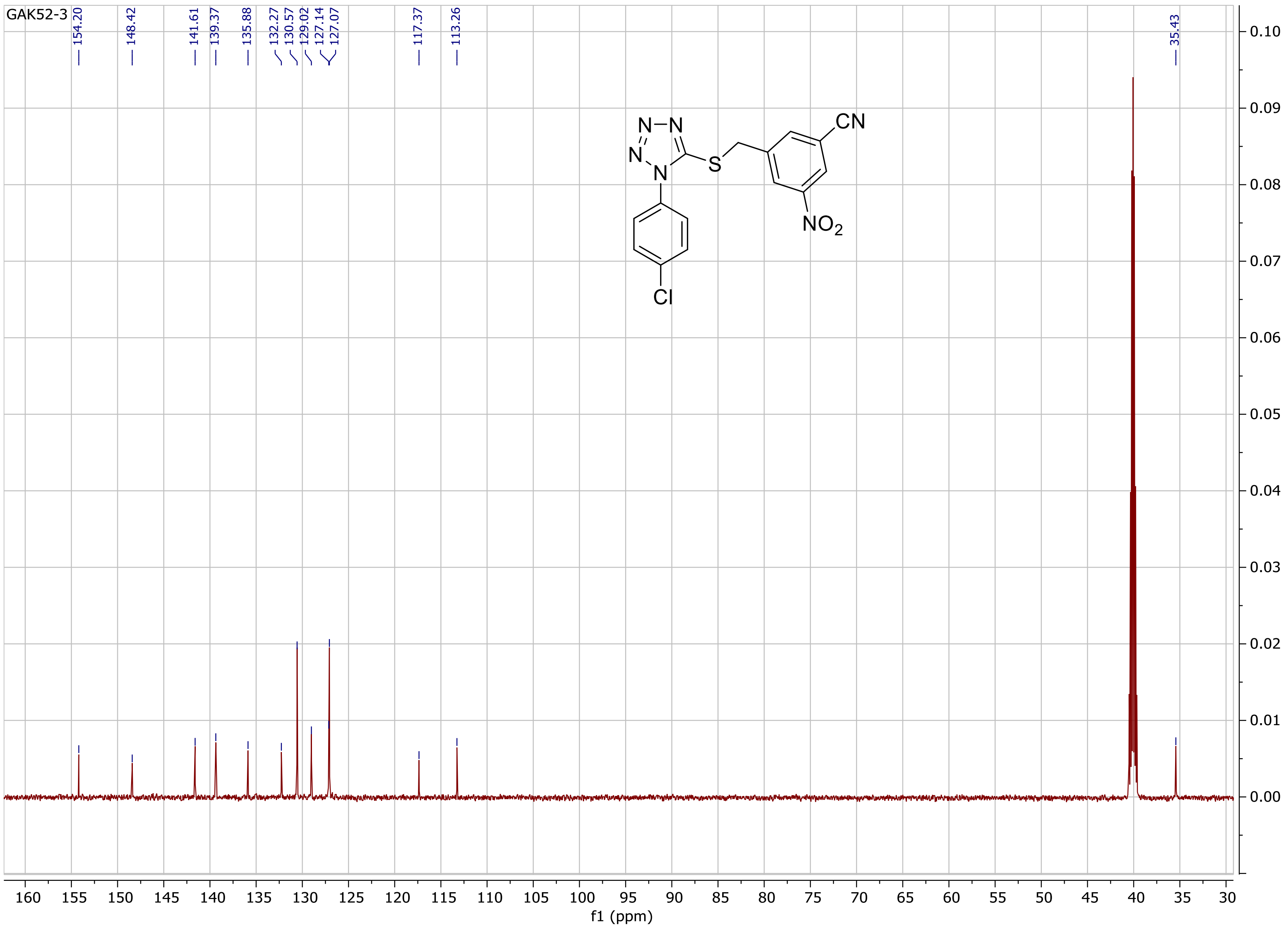

1-(4-Bromophenyl)-5-((3-cyano-5-nitrobenzyl)sulfanyl)-1H-tetrazole (**56d**): ^{1}H NMR (600 MHz, DMSO-d_6)

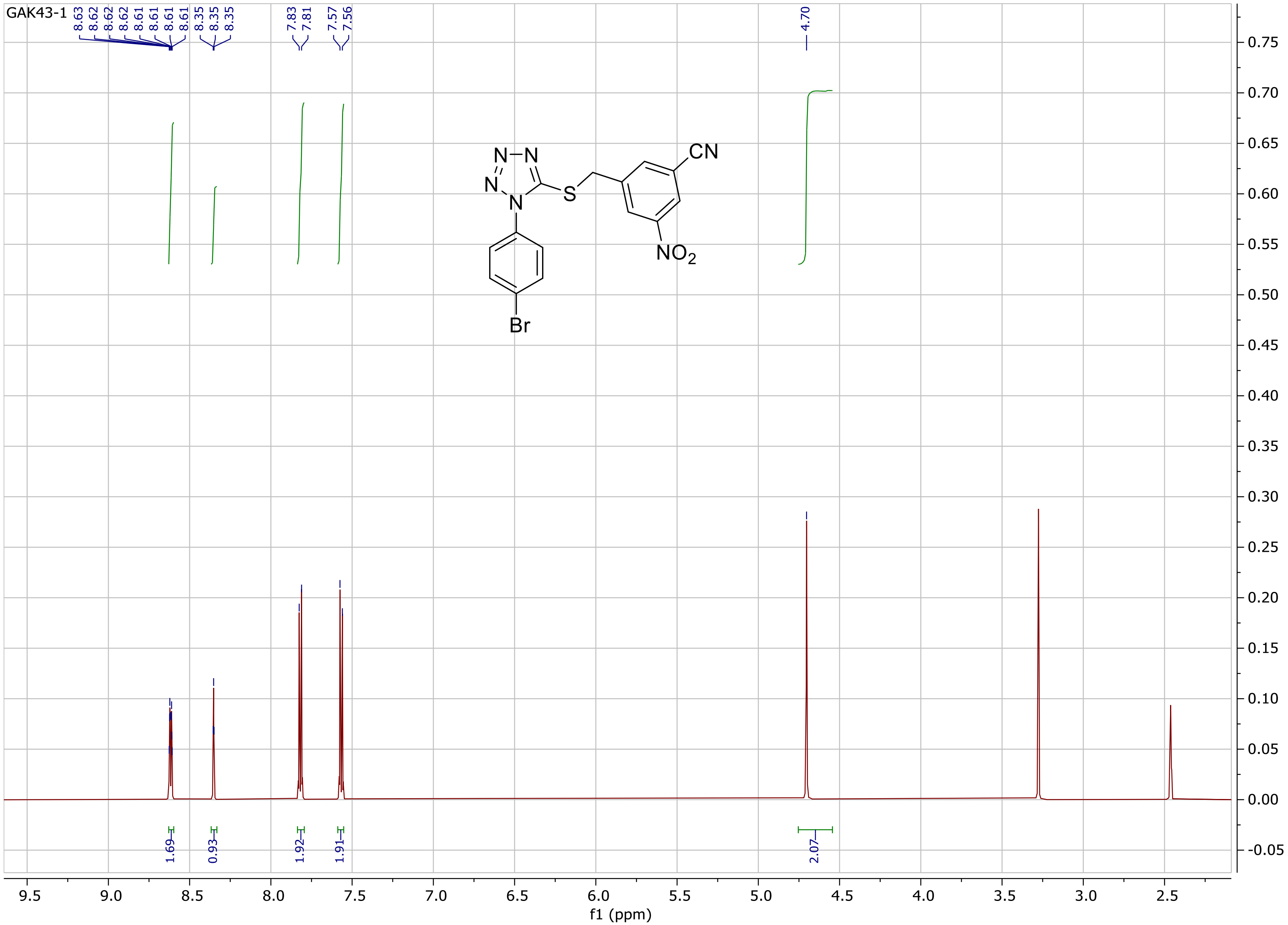

1-(4-Bromophenyl)-5-((3-cyano-5-nitrobenzyl)sulfanyl)-1H-tetrazole (**56d**): ^{13}C NMR (151 MHz, DMSO-d_6)

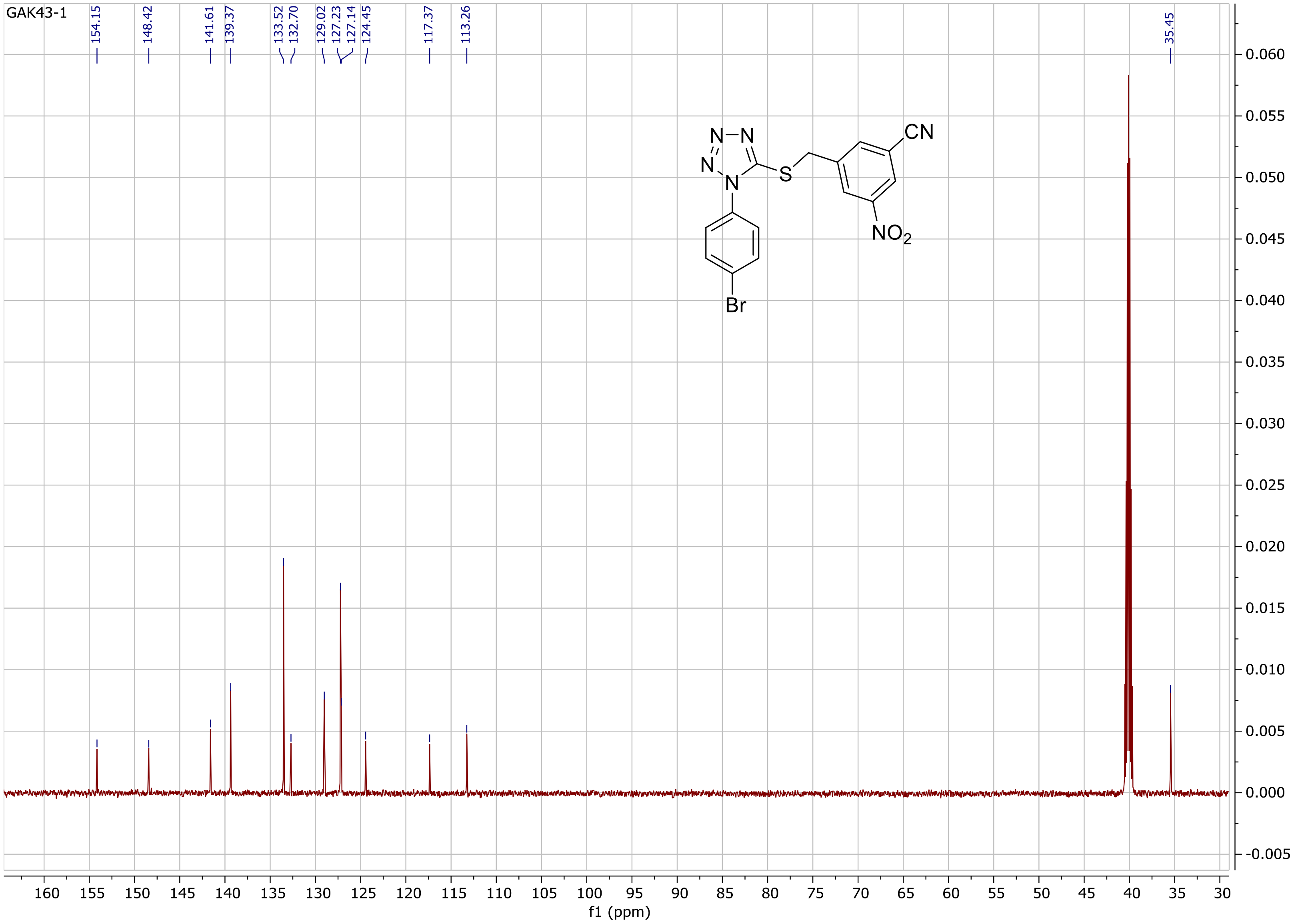

1-Cyclohexyl-5-((3-cyano-5-nitrobenzyl)sulfanyl)-1H-tetrazole (**56e**): ^{1}H NMR (600 MHz, DMSO-d_6)

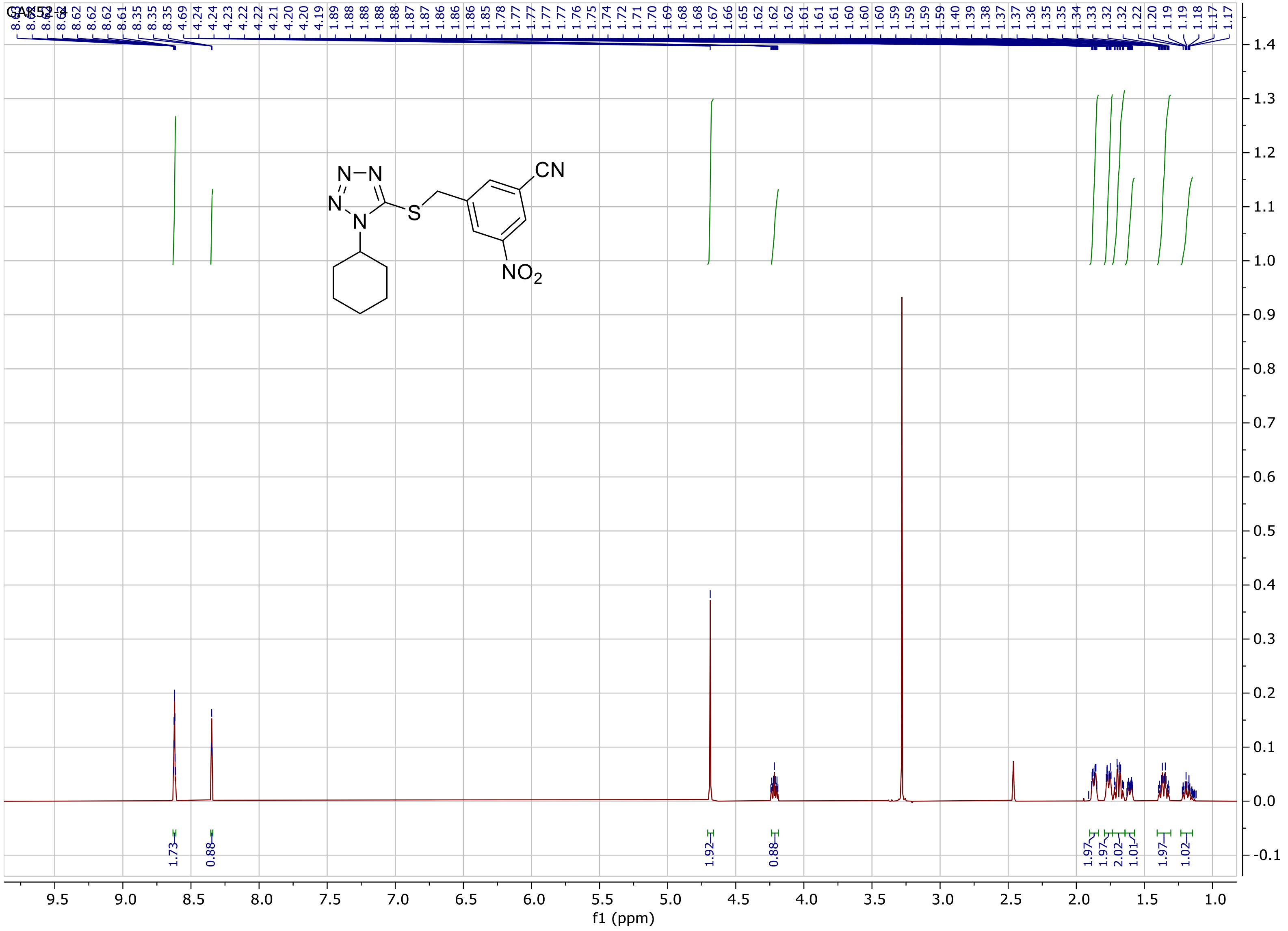

1-Cyclohexyl-5-((3-cyano-5-nitrobenzyl)sulfanyl)-1H-tetrazole (**56e**): ^{13}C NMR (151 MHz, DMSO-d_6)

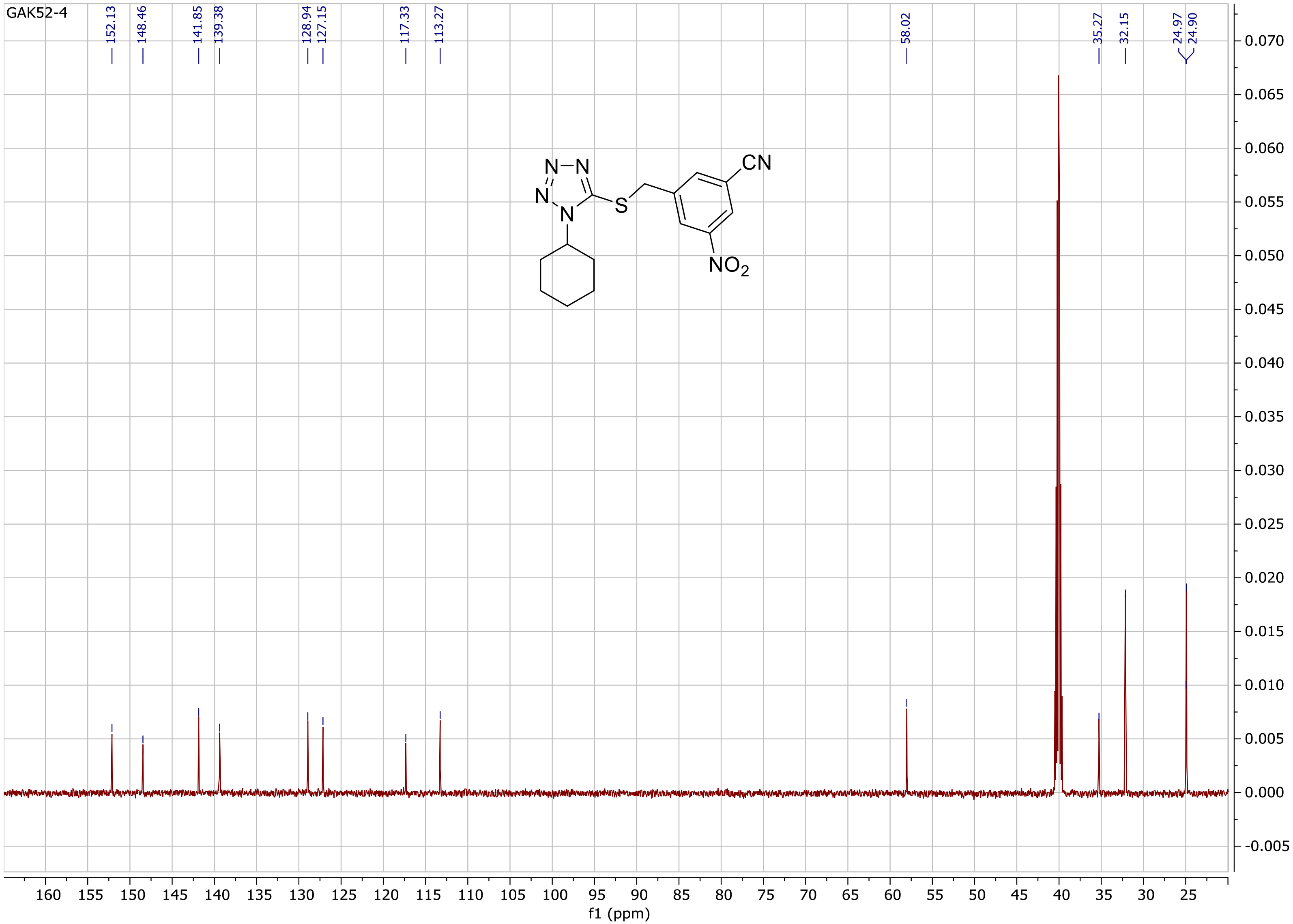

2-((3-Nitro-5-(trifluoromethyl)benzyl)sulfanyl)-5-phenyl-1,3,4-oxadiazole (**57a**): ^{1}H NMR (600 MHz, DMSO-d_6)

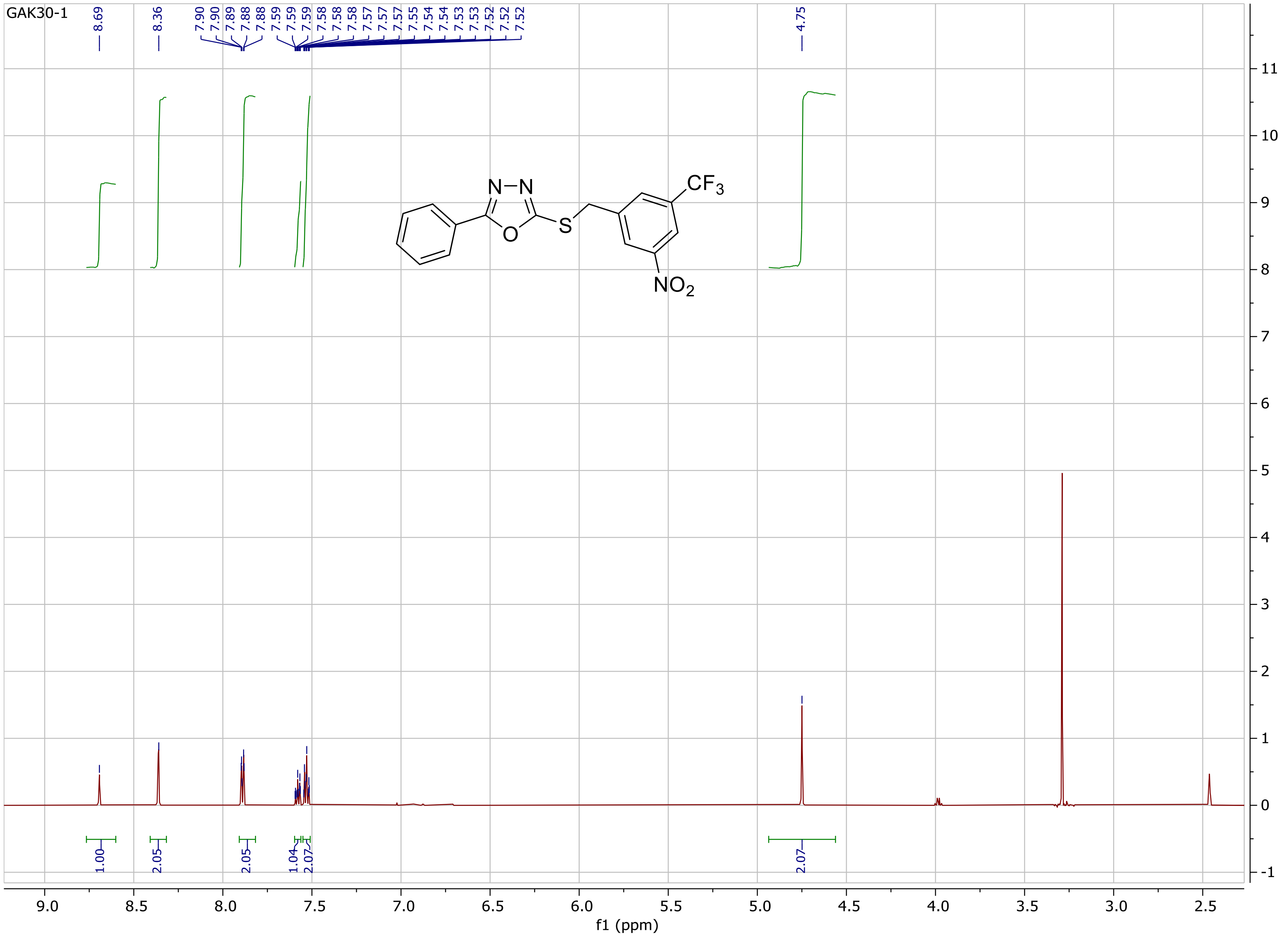

2-((3-Nitro-5-(trifluoromethyl)benzyl)sulfanyl)-5-phenyl-1,3,4-oxadiazole (**57a**): ^{13}C NMR (151 MHz, DMSO-d_6)

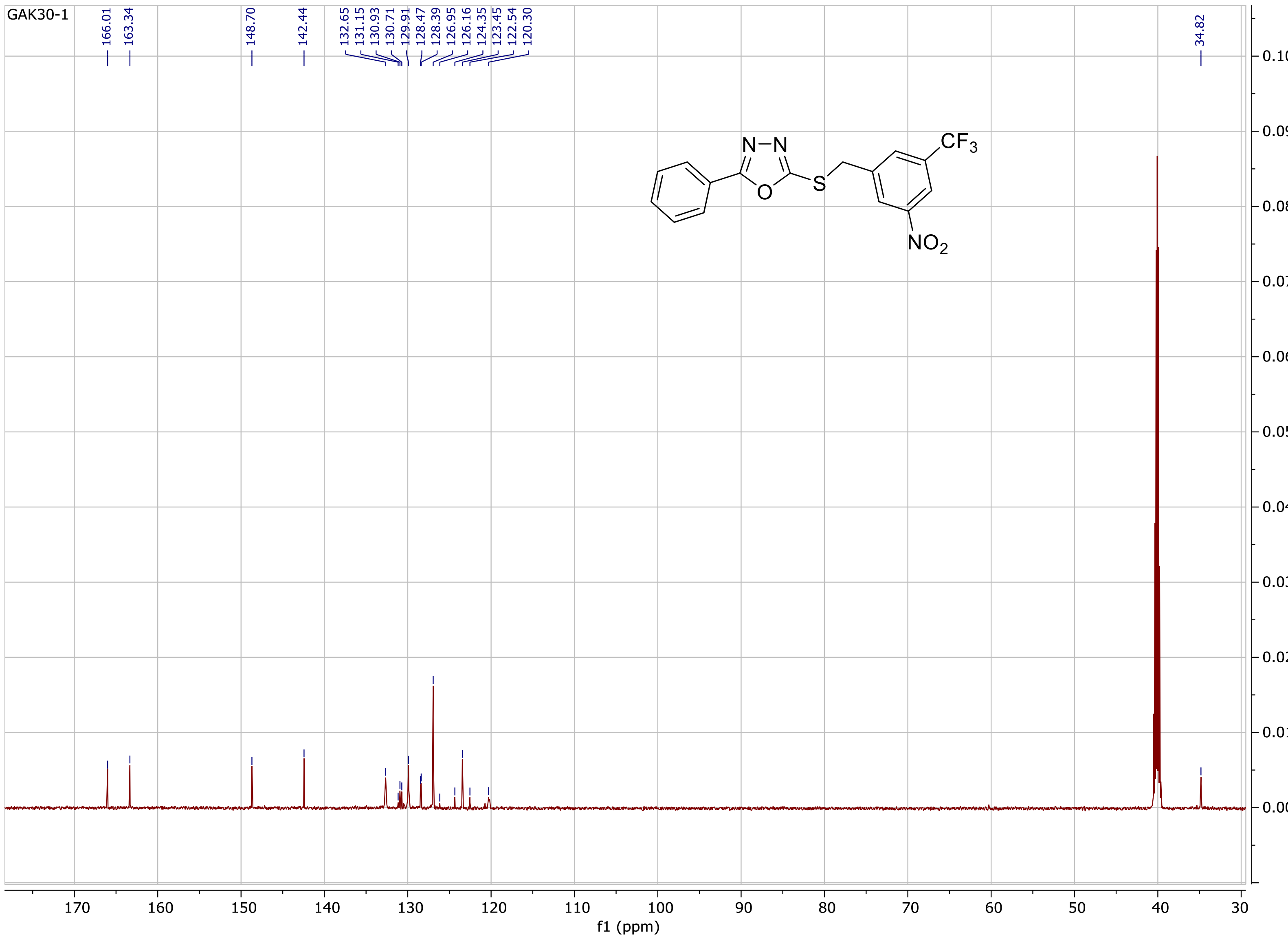

2-((3-Nitro-5-(trifluoromethyl)benzyl)sulfanyl)-5-phenyl-1,3,4-oxadiazole (**57a**):

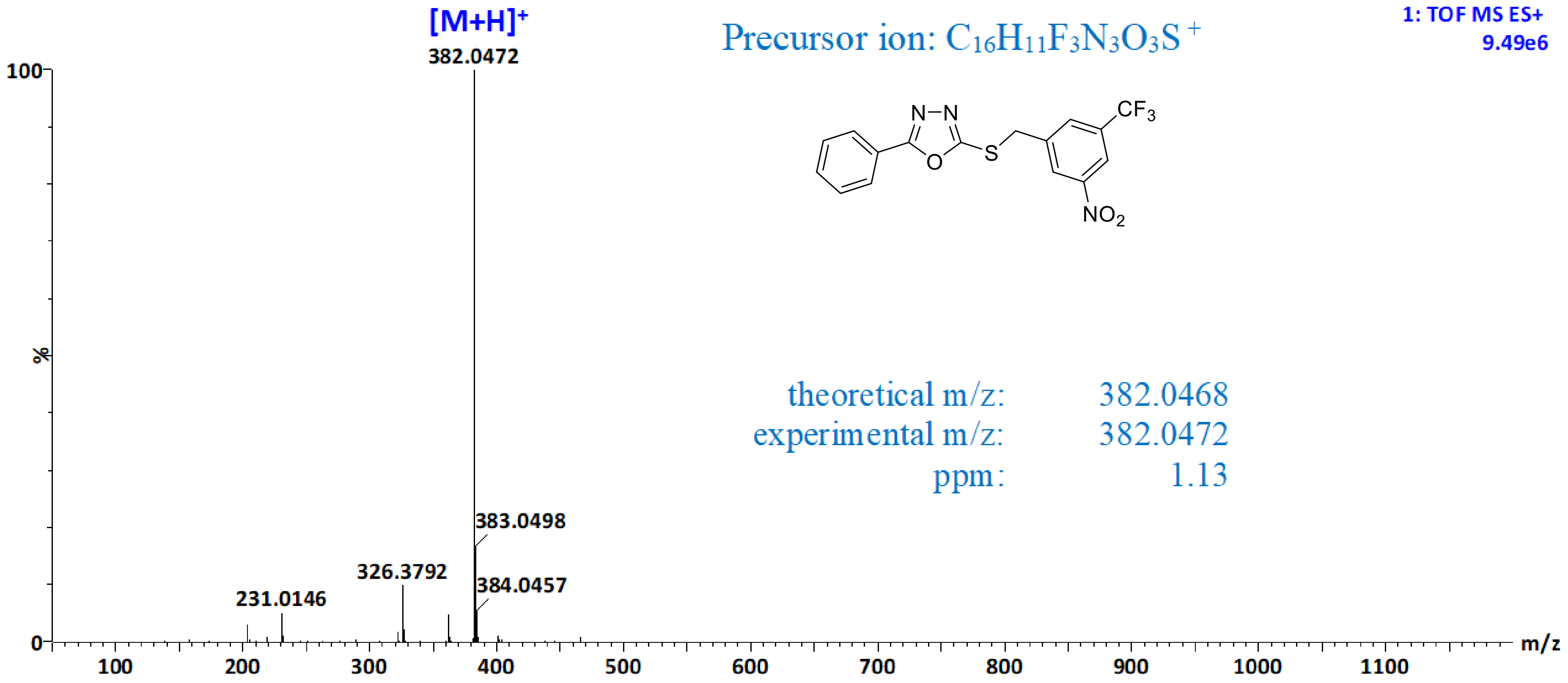

2-(4-Methoxyphenyl)-5-((3-nitro-5-(trifluoromethyl)benzyl)sulfanyl)-1,3,4-oxadiazole (**57b**): ^{1}H NMR (500 MHz, DMSO-d_6)

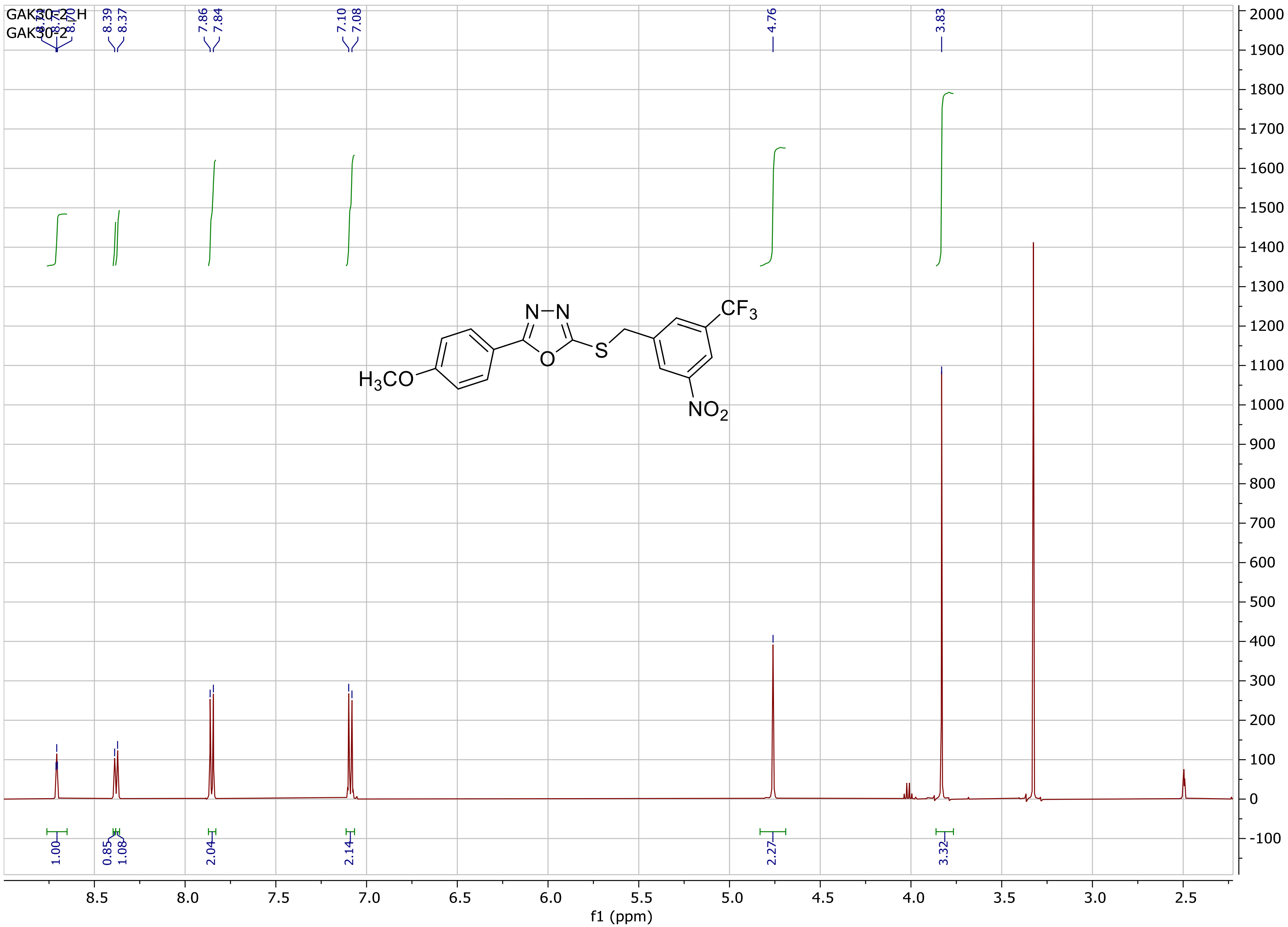

2-(4-Methoxyphenyl)-5-((3-nitro-5-(trifluoromethyl)benzyl)sulfanyl)-1,3,4-oxadiazole (**57b**): ^{13}C NMR (126 MHz, DMSO-d_6)

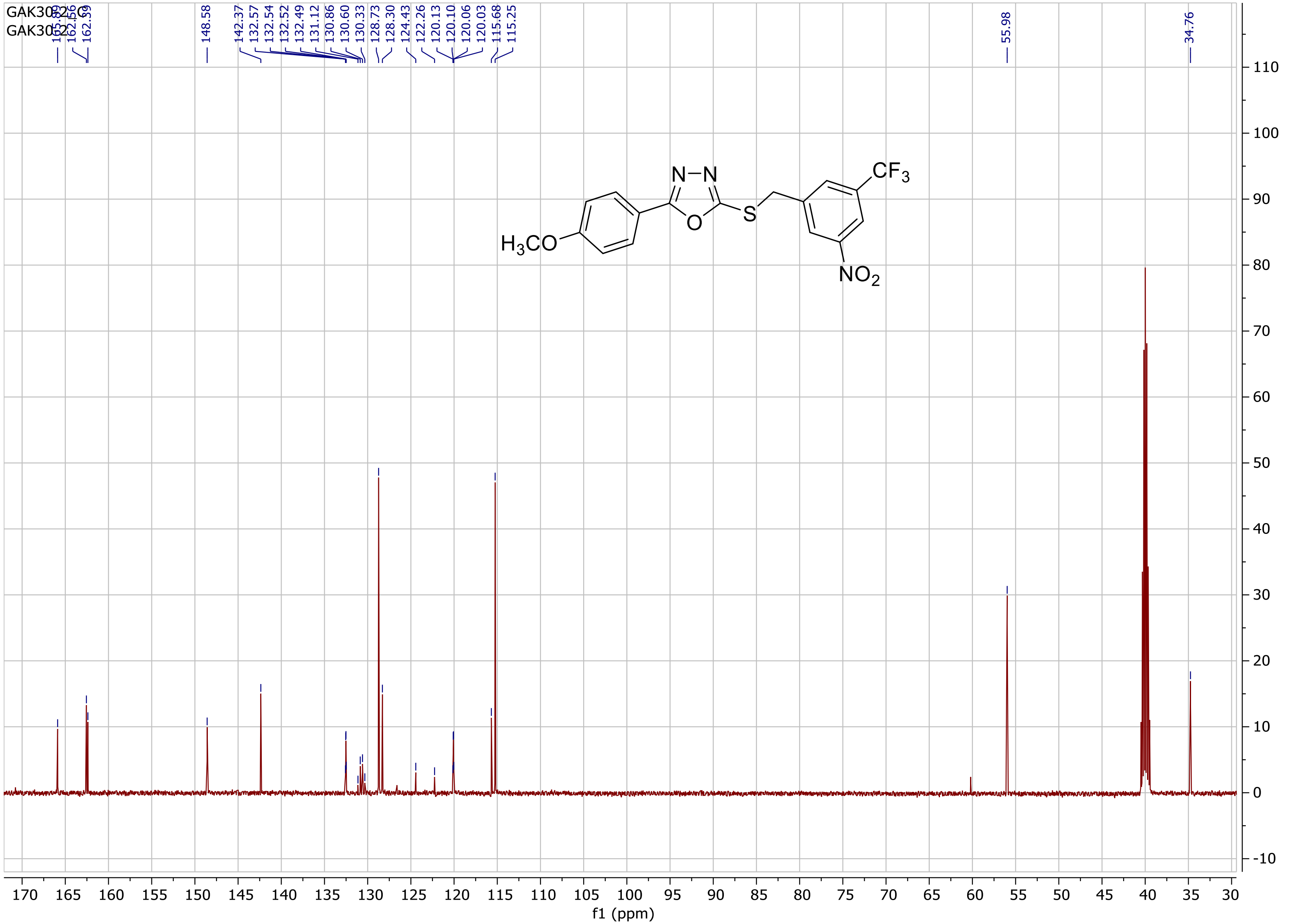

2-(4-Methoxyphenyl)-5-((3-nitro-5-(trifluoromethyl)benzyl)sulfanyl)-1,3,4-oxadiazole (**57b**):

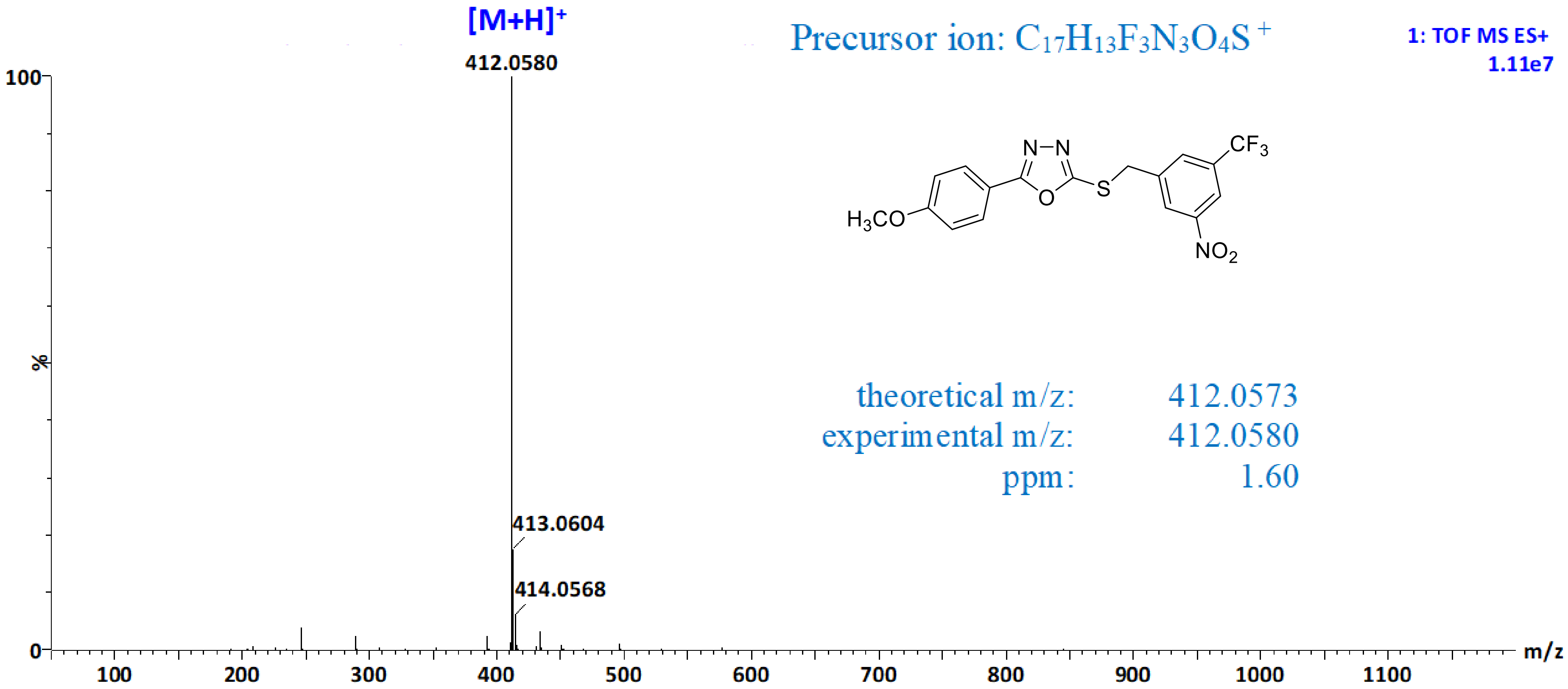

2-(4-Chlorophenyl)-5-((3-nitro-5-(trifluoromethyl)benzyl)sulfanyl)-1,3,4-oxadiazole (**57c**): ^{1}H NMR (500 MHz, DMSO-d_6)

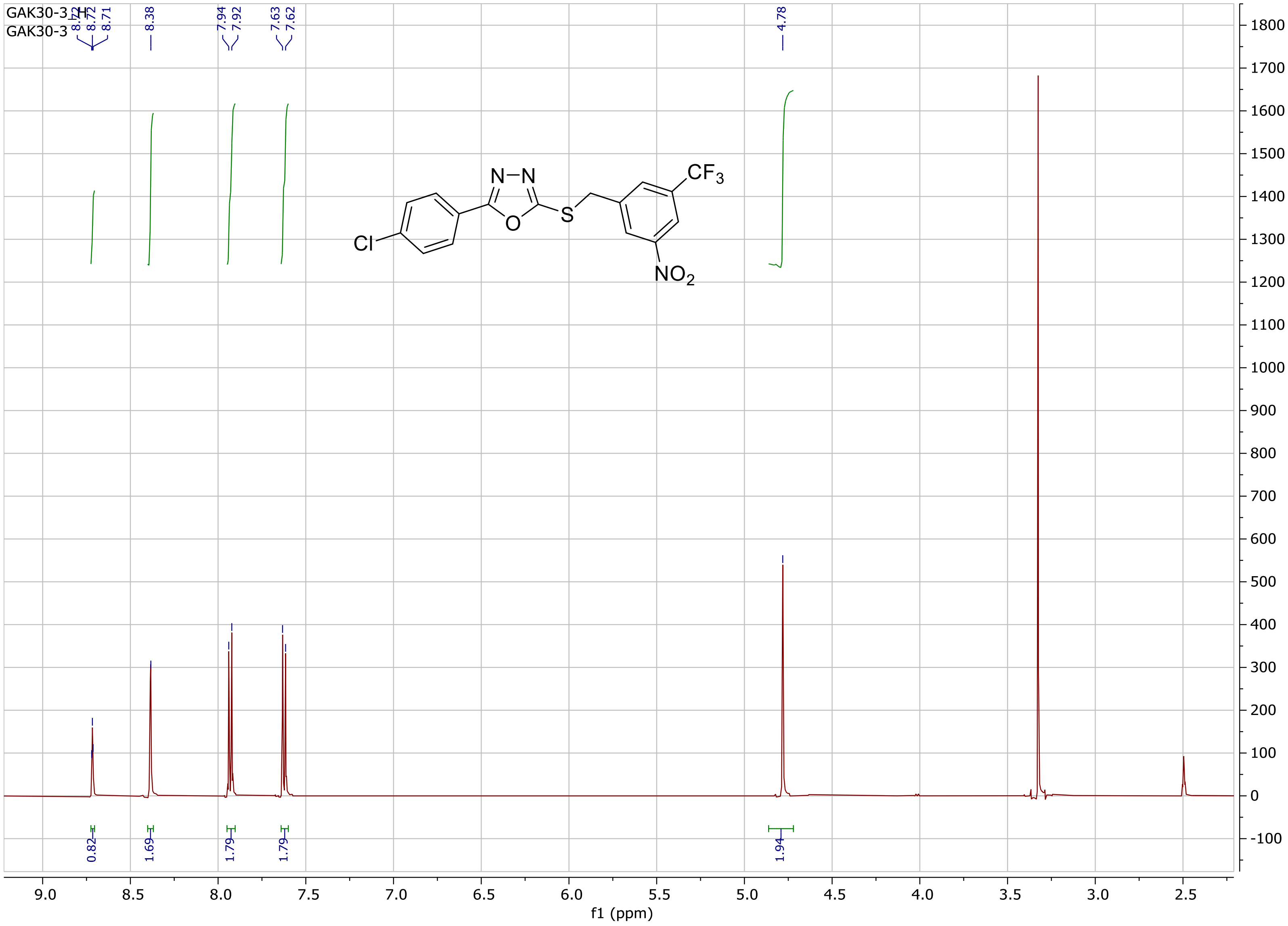

2-(4-Chlorophenyl)-5-((3-nitro-5-(trifluoromethyl)benzyl)sulfanyl)-1,3,4-oxadiazole (**57c**): ^{13}C NMR (126 MHz, DMSO-d_6)

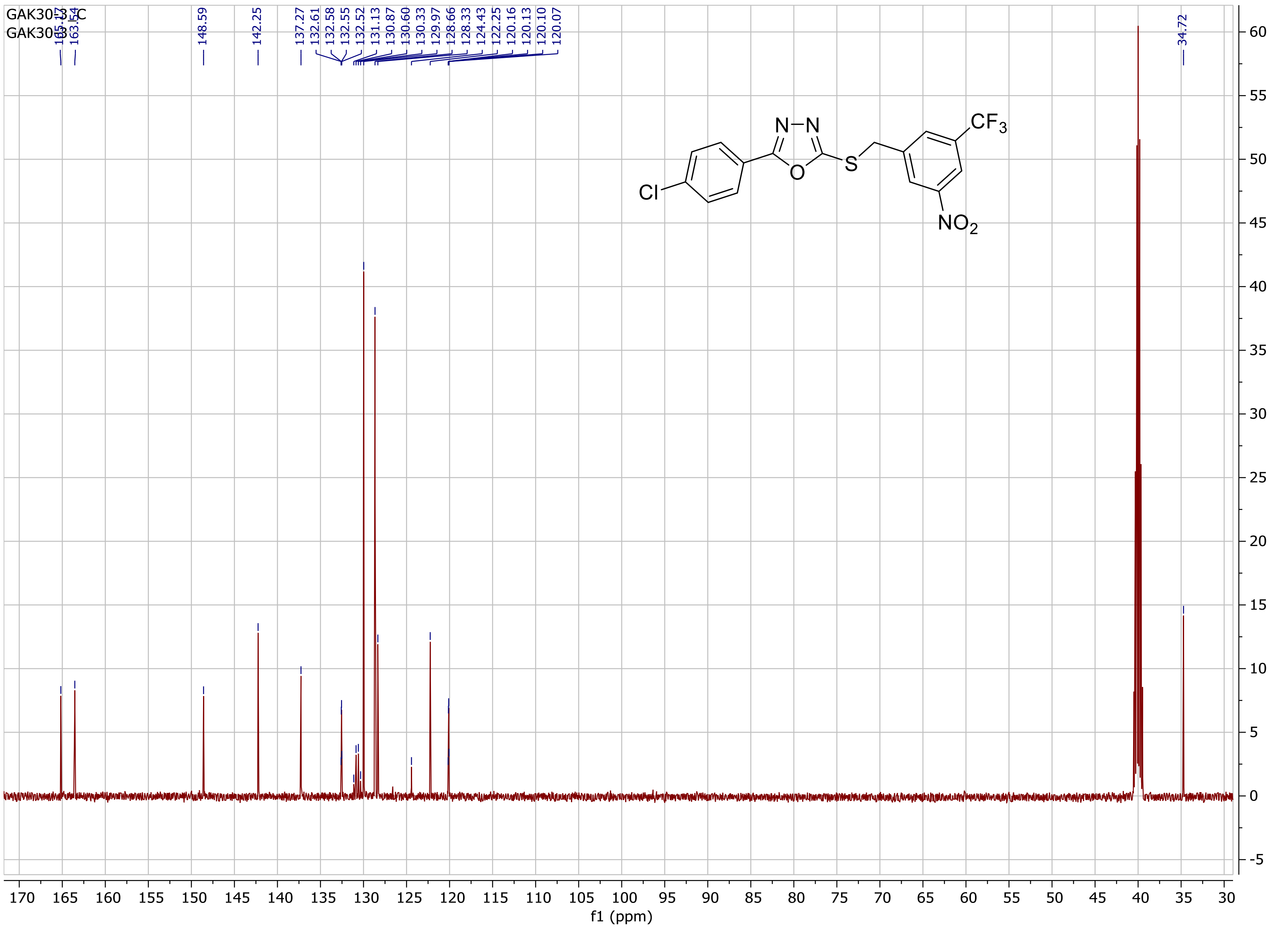

2-(4-Chlorophenyl)-5-((3-nitro-5-(trifluoromethyl)benzyl)sulfanyl)-1,3,4-oxadiazole (**57c**):

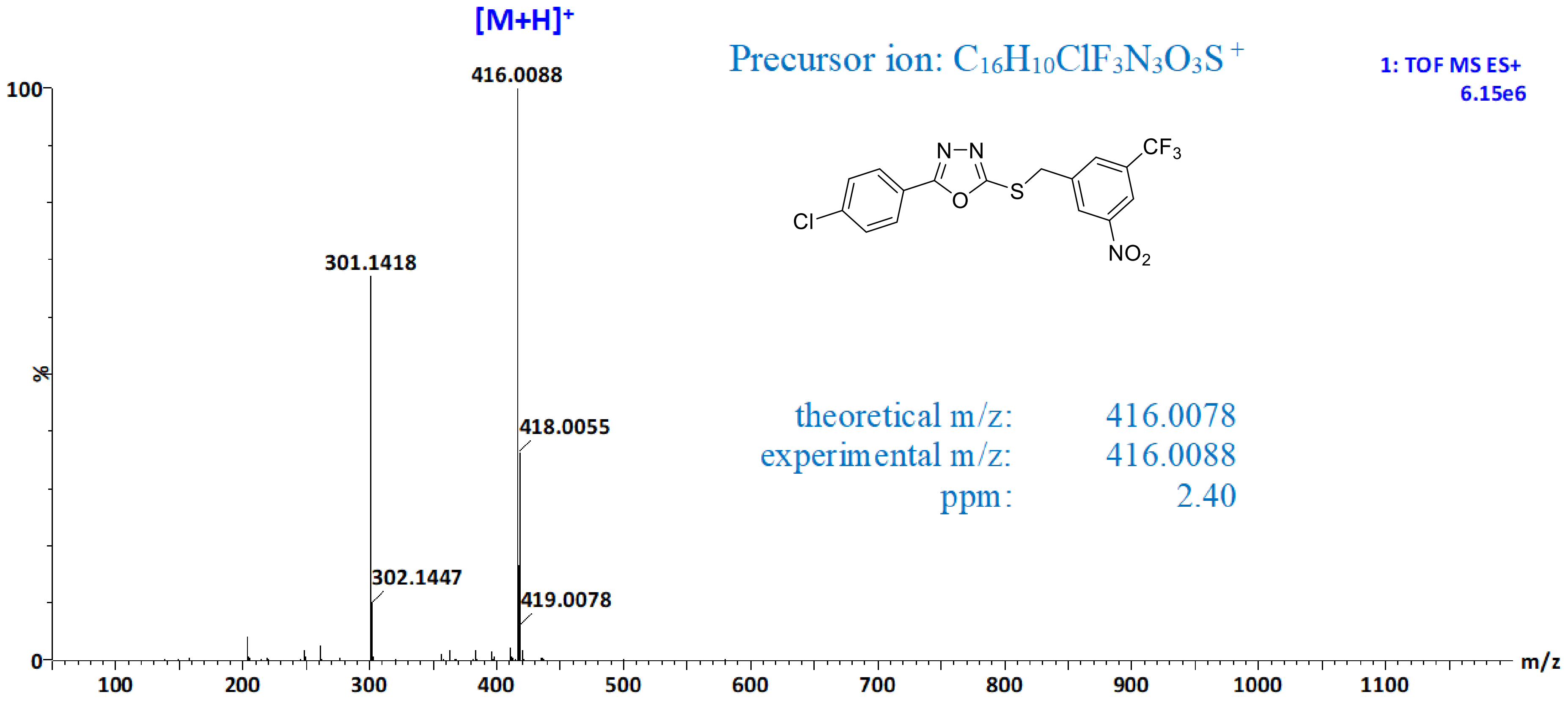

2-(4-Bromophenyl)-5-((3-nitro-5-(trifluoromethyl)benzyl)sulfanyl)-1,3,4-oxadiazole (**57d**): ^{1}H NMR (500 MHz, DMSO-d_6)

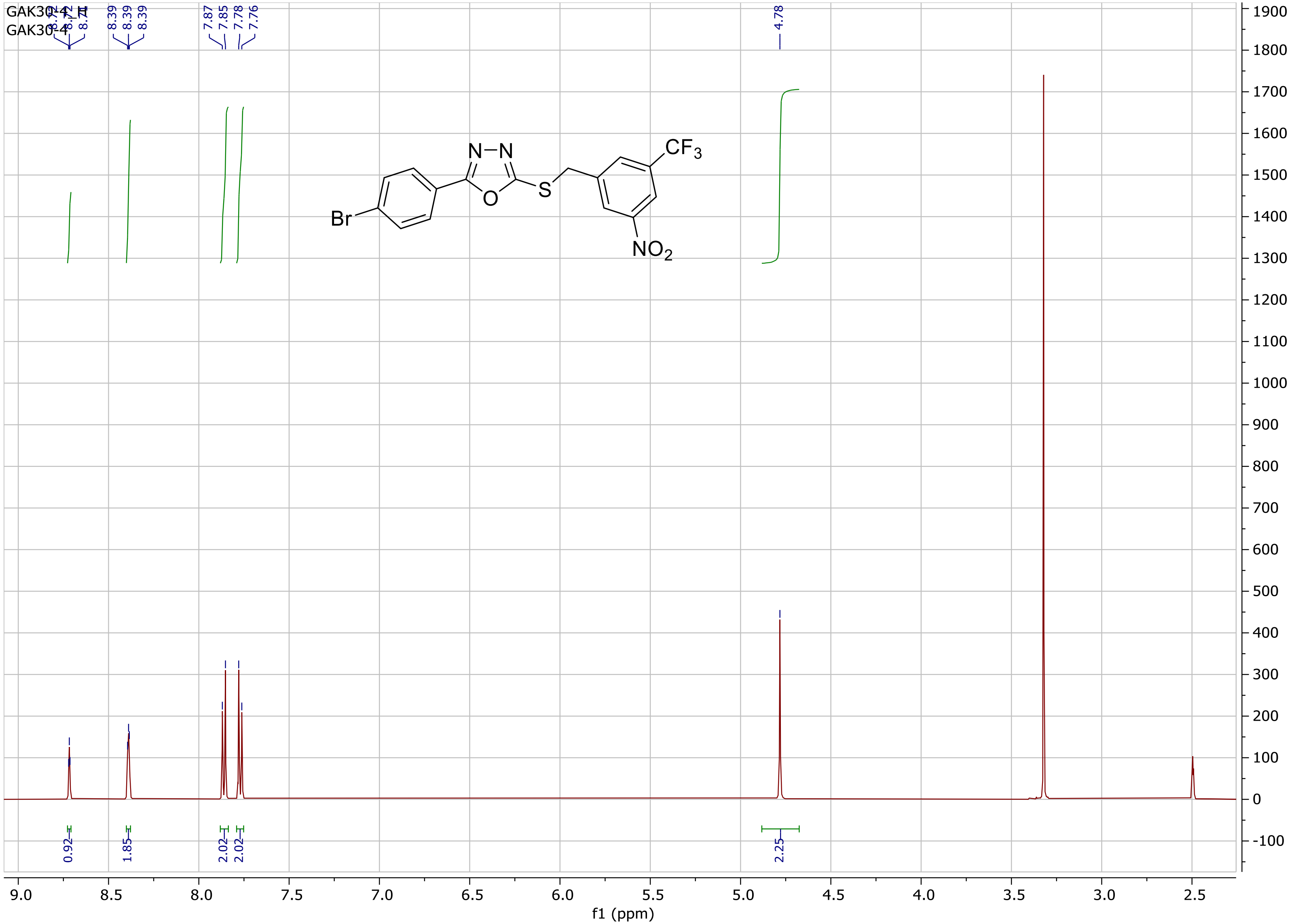

2-(4-Bromophenyl)-5-((3-nitro-5-(trifluoromethyl)benzyl)sulfanyl)-1,3,4-oxadiazole (**57d**): ^{13}C NMR (126 MHz, DMSO-d_6)

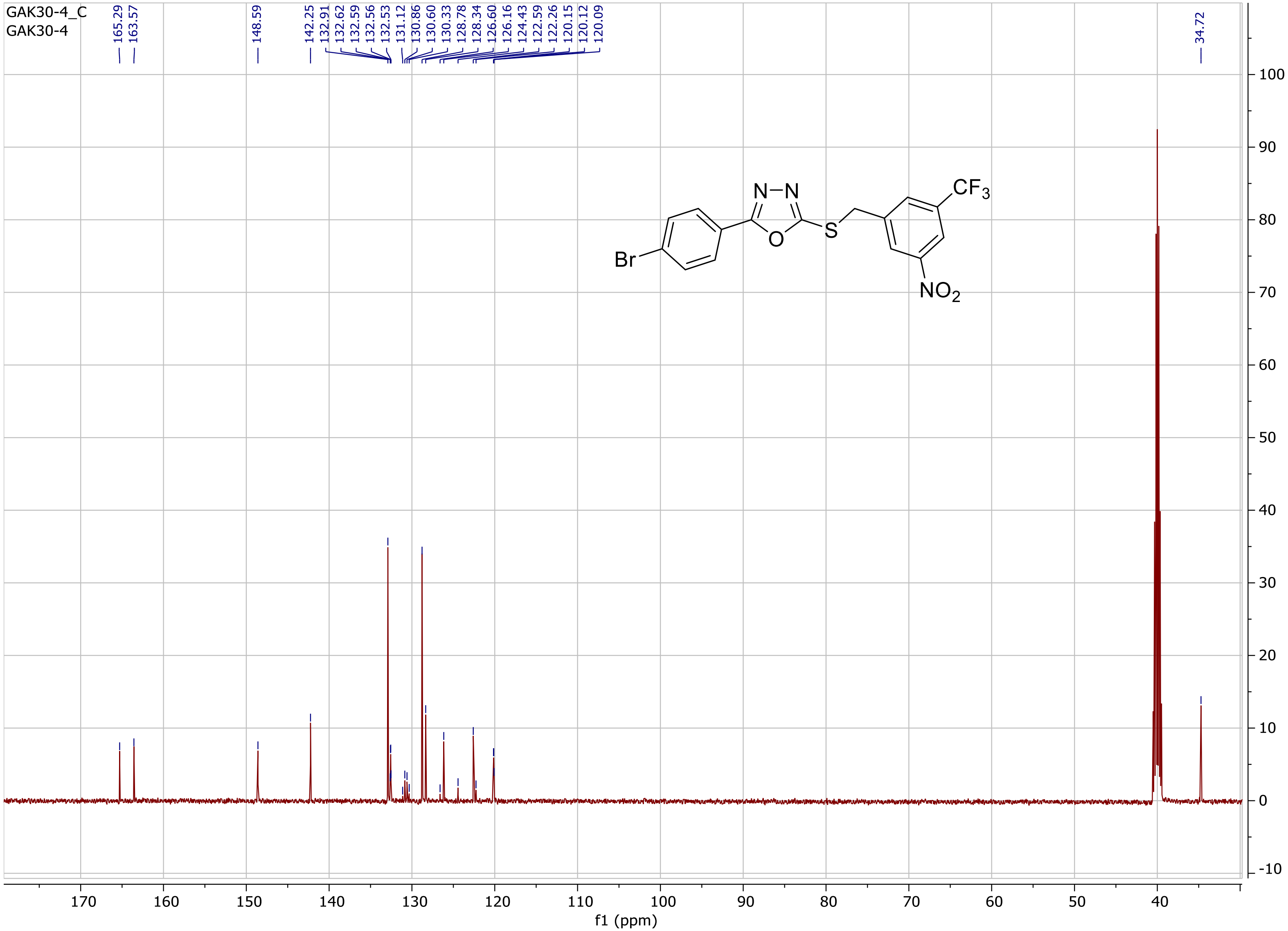

2-(4-Bromophenyl)-5-((3-nitro-5-(trifluoromethyl)benzyl)sulfanyl)-1,3,4-oxadiazole (**57d**):

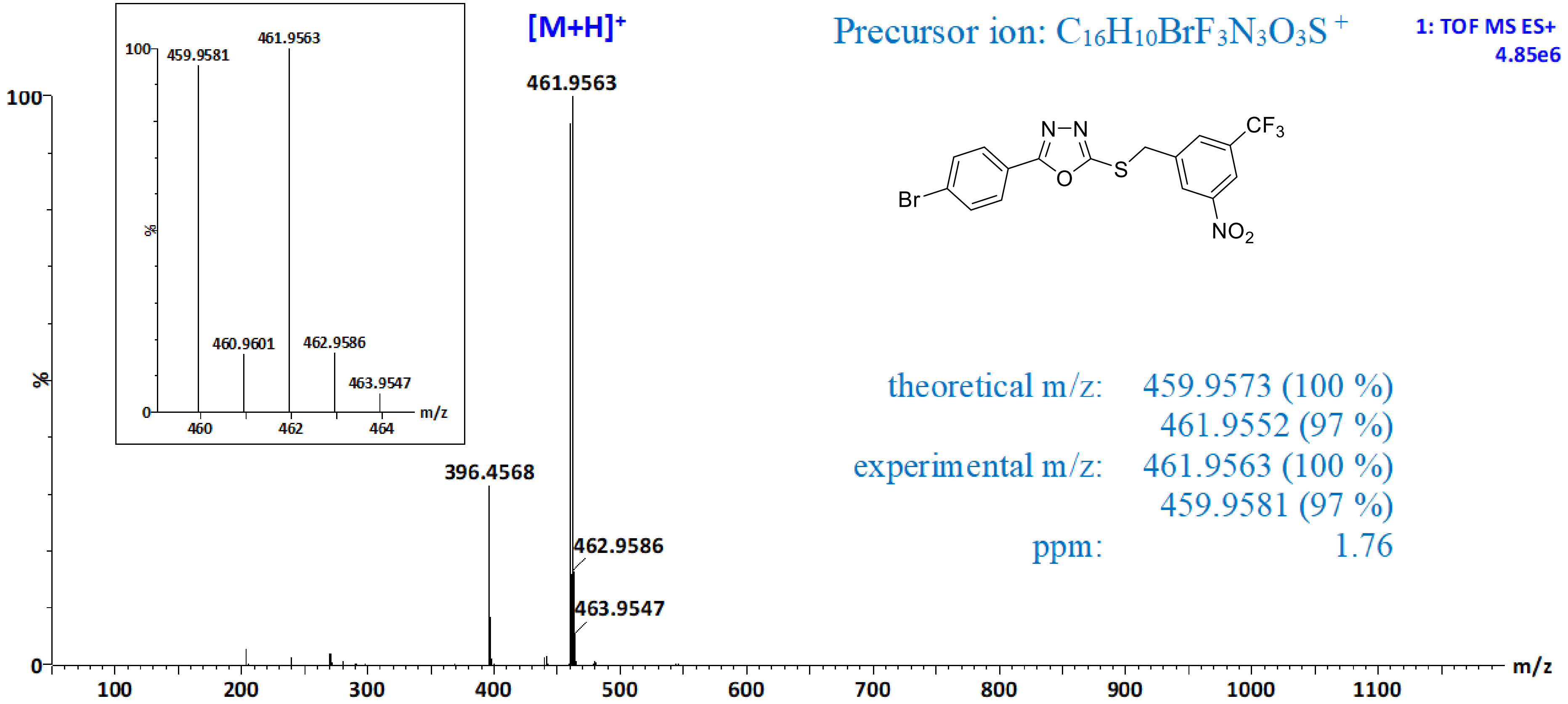

2-Cyclohexyl-5-((3-nitro-5-(trifluoromethyl)benzyl)sulfanyl)-1,3,4-oxadiazole (**57e**): ^{1}H NMR (500 MHz, DMSO-d_6)

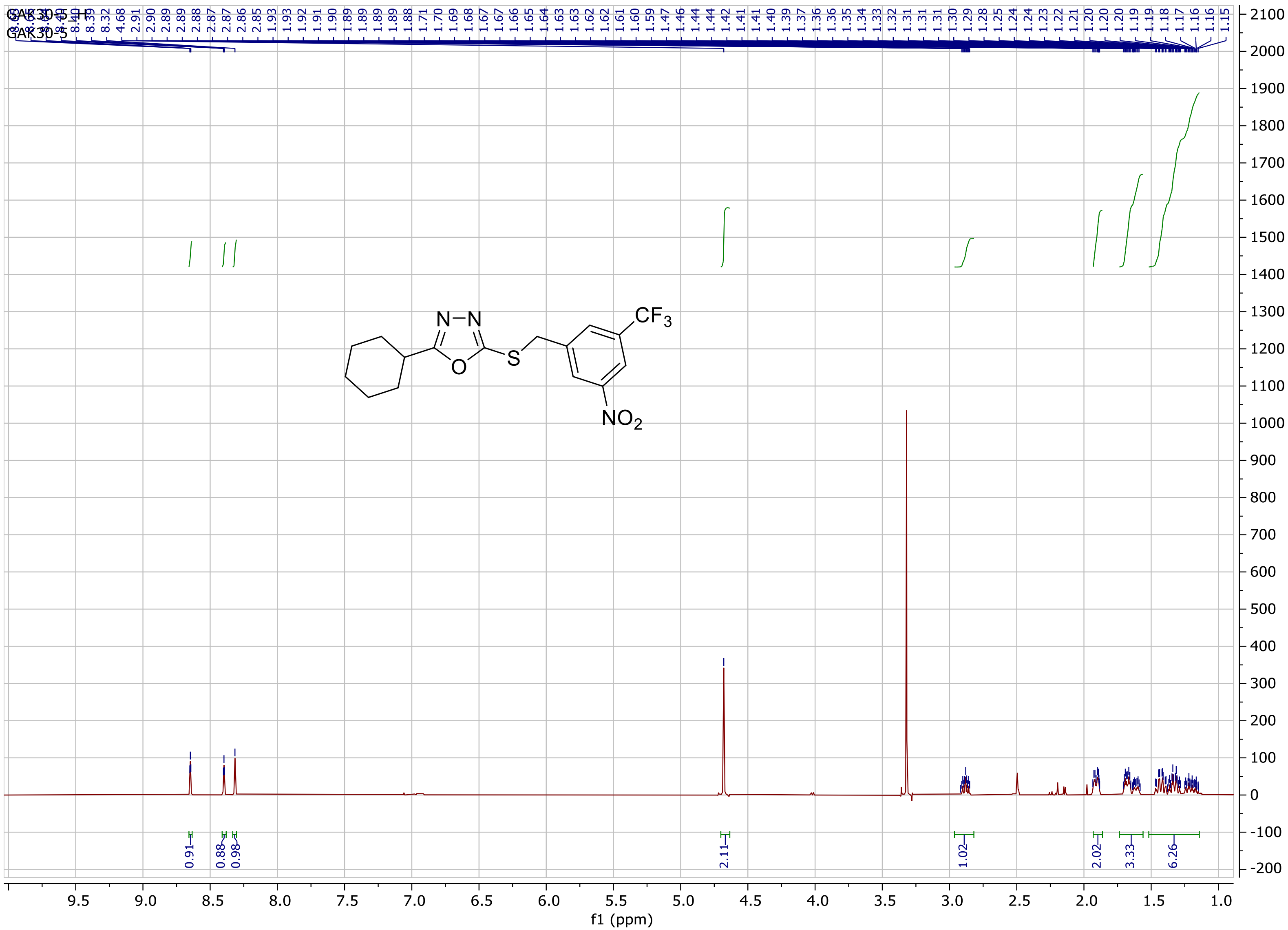

2-Cyclohexyl-5-((3-nitro-5-(trifluoromethyl)benzyl)sulfanyl)-1,3,4-oxadiazole (**57e**): ^{13}C NMR (126 MHz, DMSO-d_6)

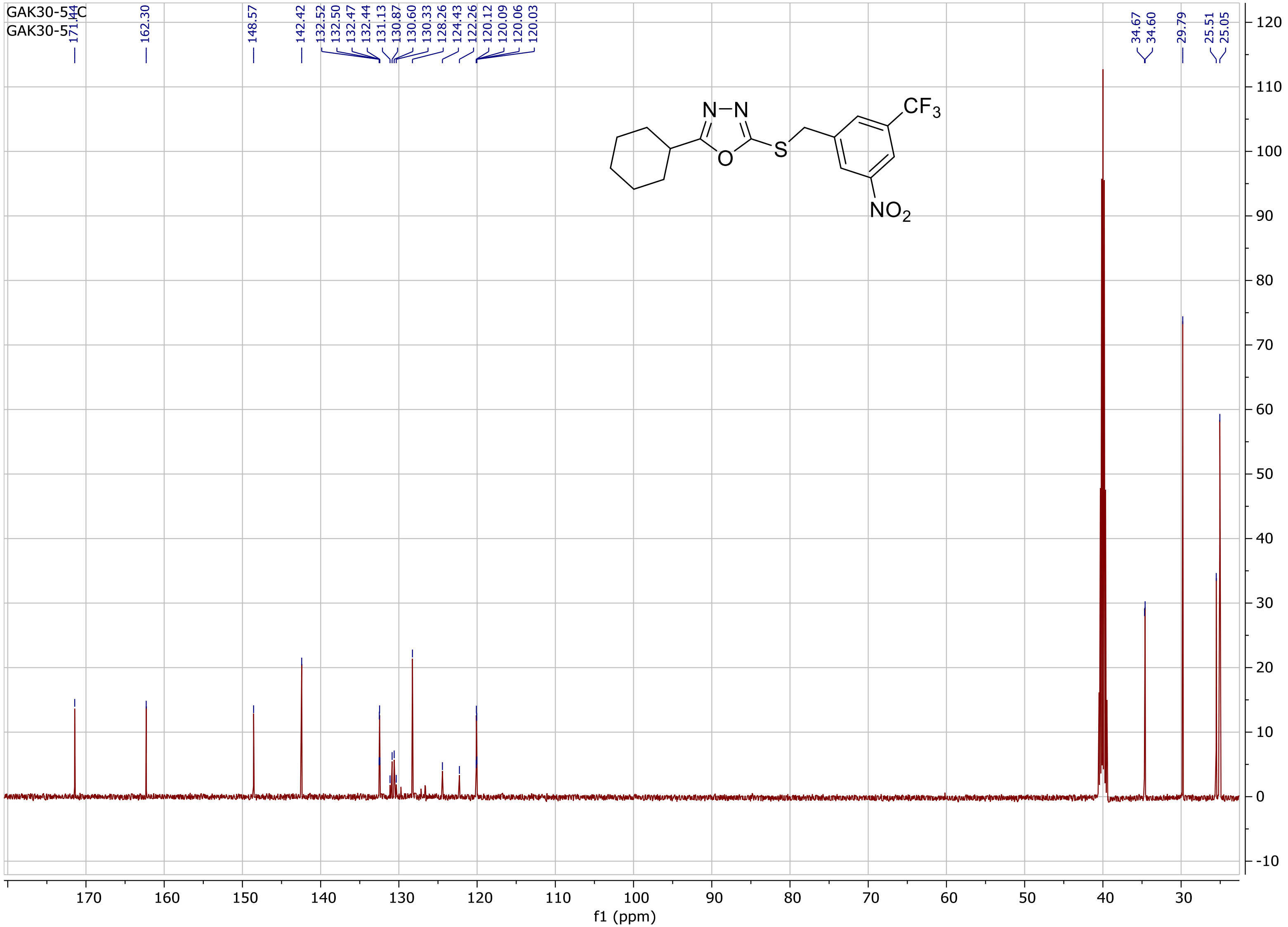

2-Cyclohexyl-5-((3-nitro-5-(trifluoromethyl)benzyl)sulfanyl)-1,3,4-oxadiazole (**57e**):

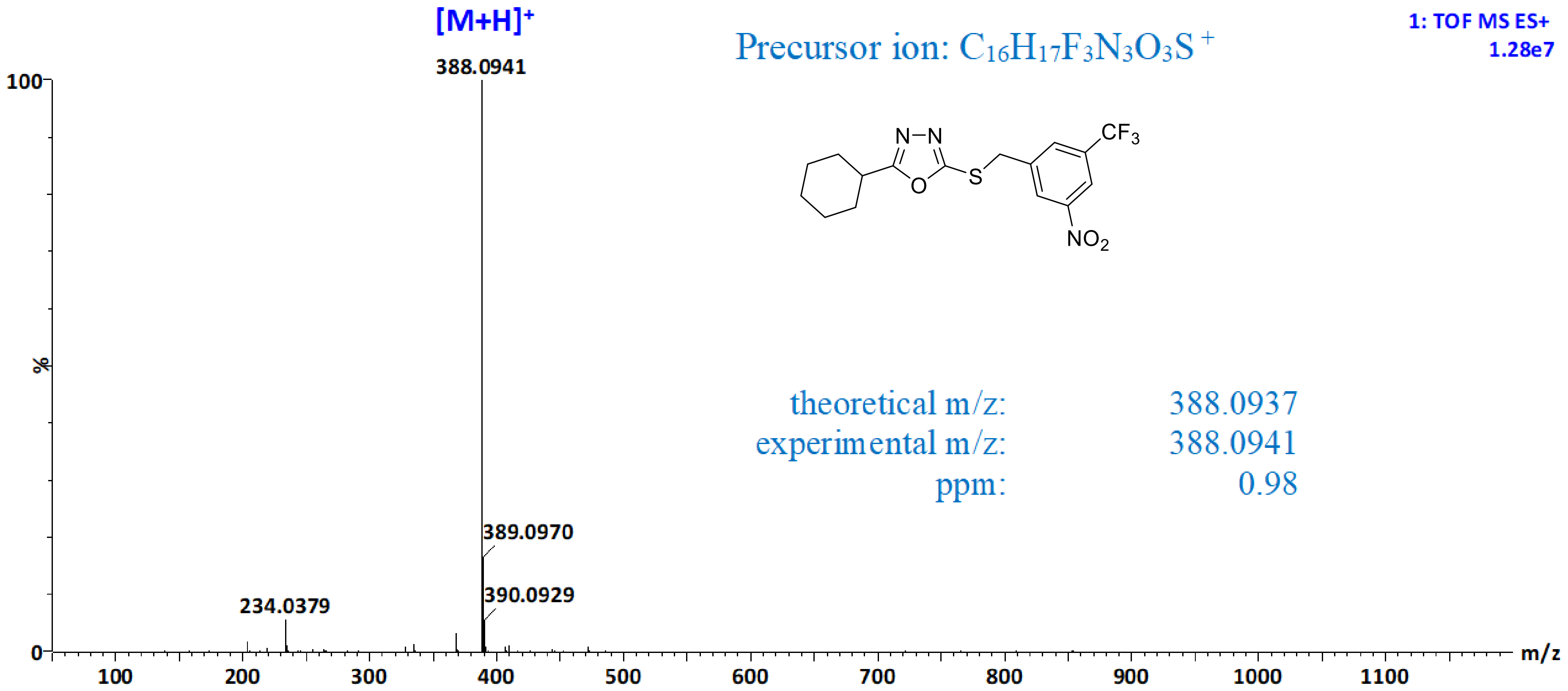

2-((3-Chloro-5-nitrobenzyl)sulfanyl)-5-phenyl-1,3,4-oxadiazole (**58a**): ^{1}H NMR (500 MHz, DMSO-d_6)

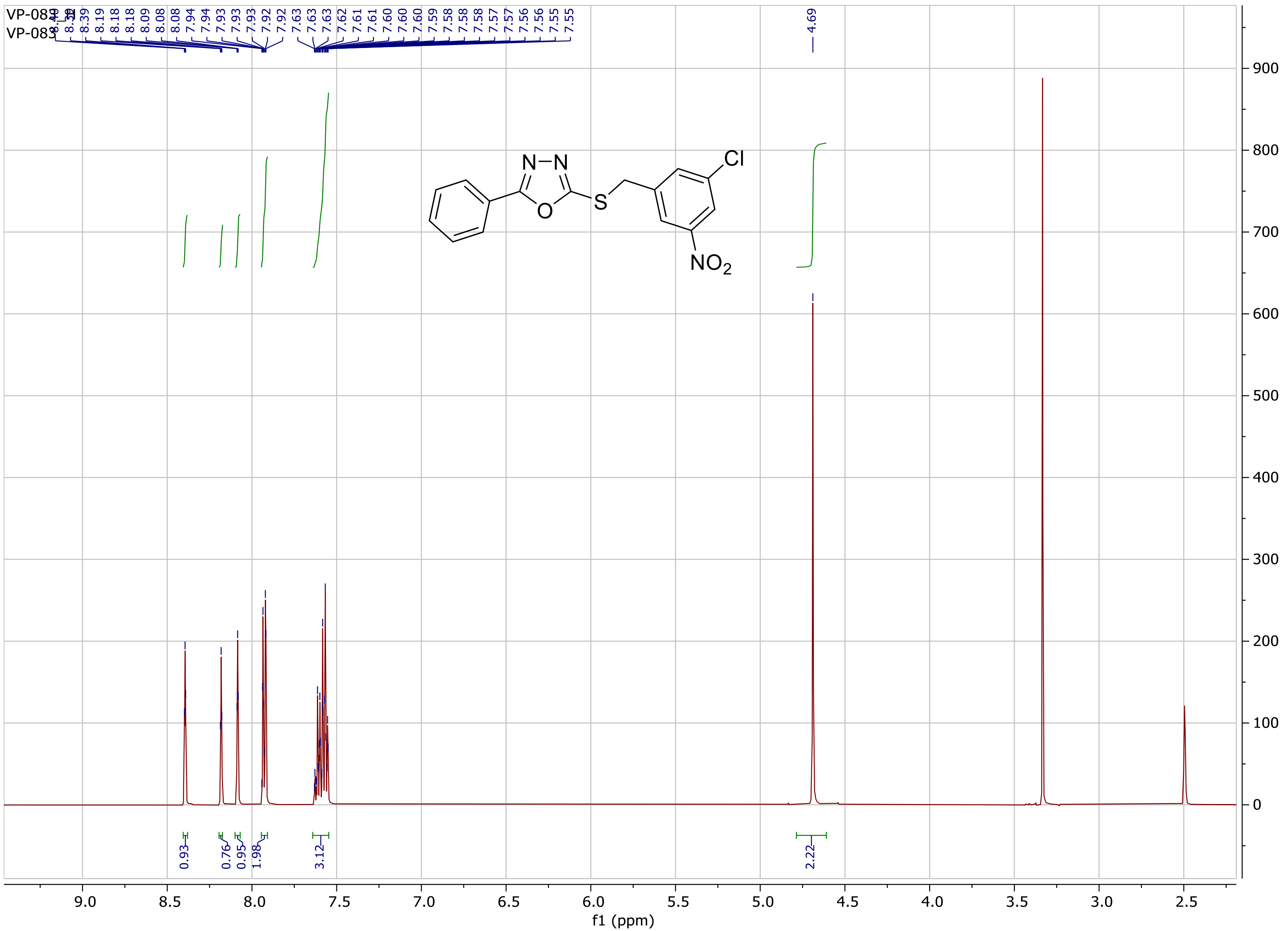

2-((3-Chloro-5-nitrobenzyl)sulfanyl)-5-phenyl-1,3,4-oxadiazole (**58a**): ^{13}C NMR (126 MHz, DMSO-d_6)

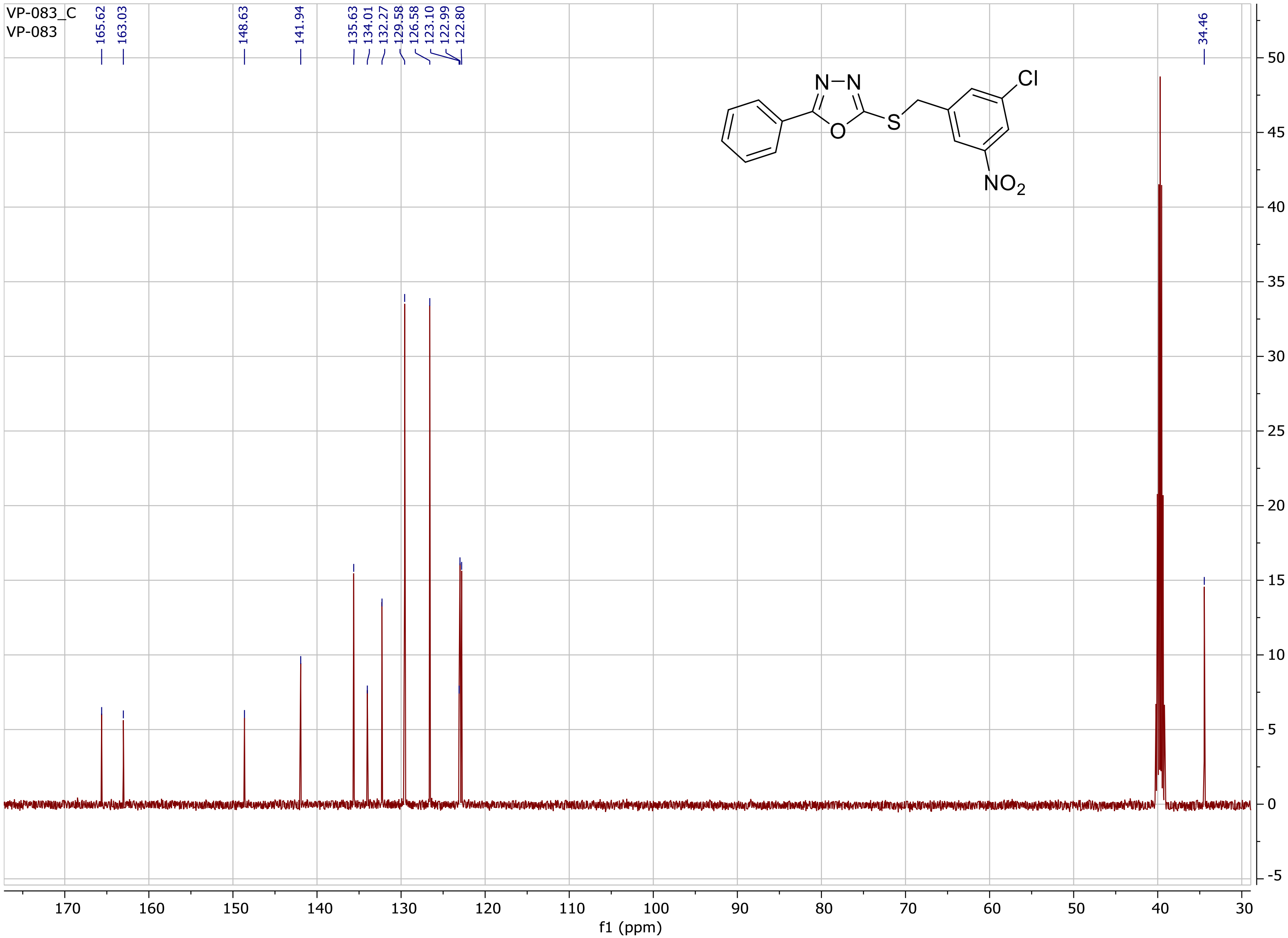

2-((3-Chloro-5-nitrobenzyl)sulfanyl)-5-(4-methoxyphenyl)-1,3,4-oxadiazole (**58b**): ^{1}H NMR (600 MHz, DMSO-d_6)

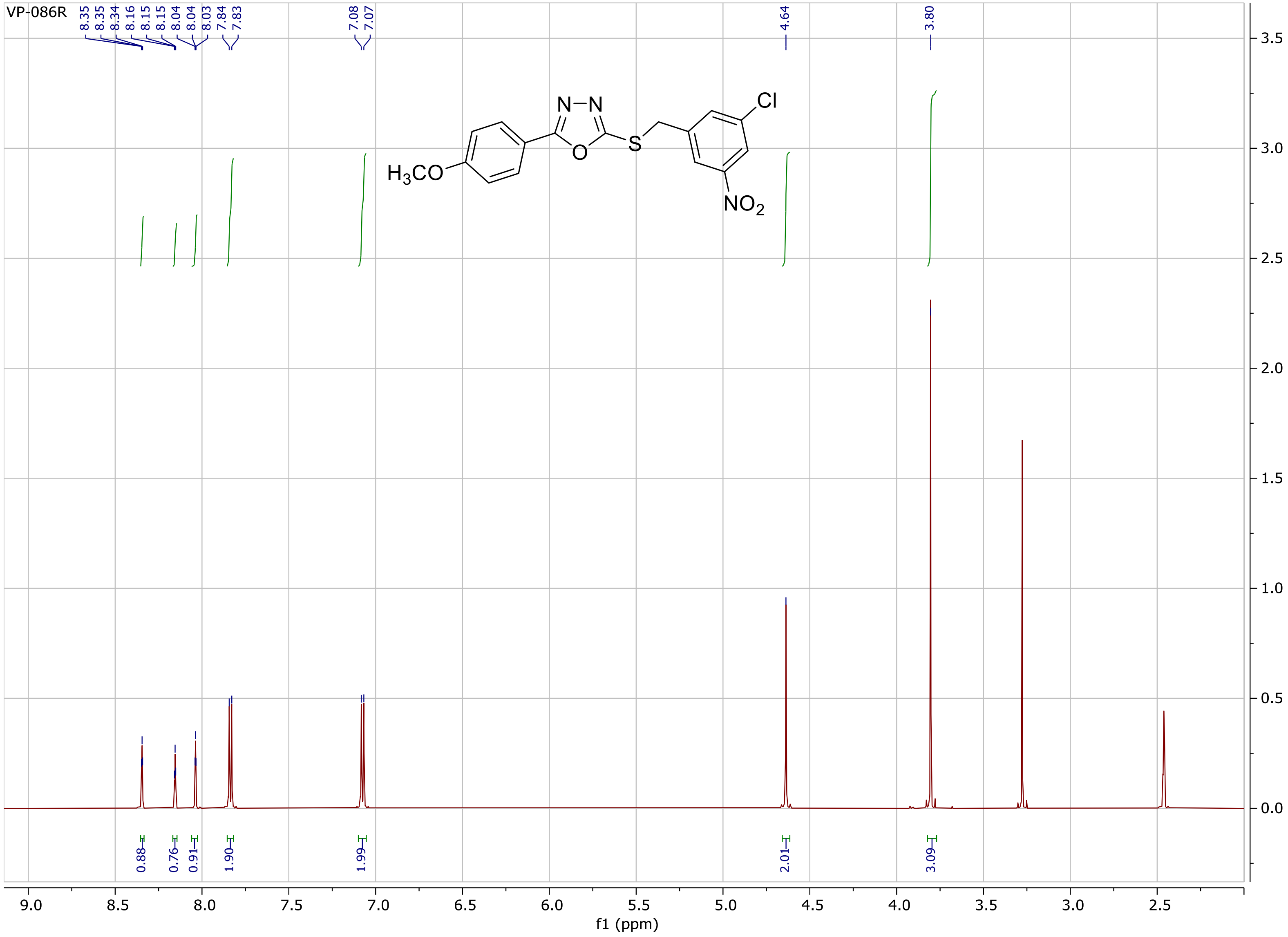

2-((3-Chloro-5-nitrobenzyl)sulfanyl)-5-(4-methoxyphenyl)-1,3,4-oxadiazole (**58b**): ^{13}C NMR (151 MHz, DMSO-d_6)

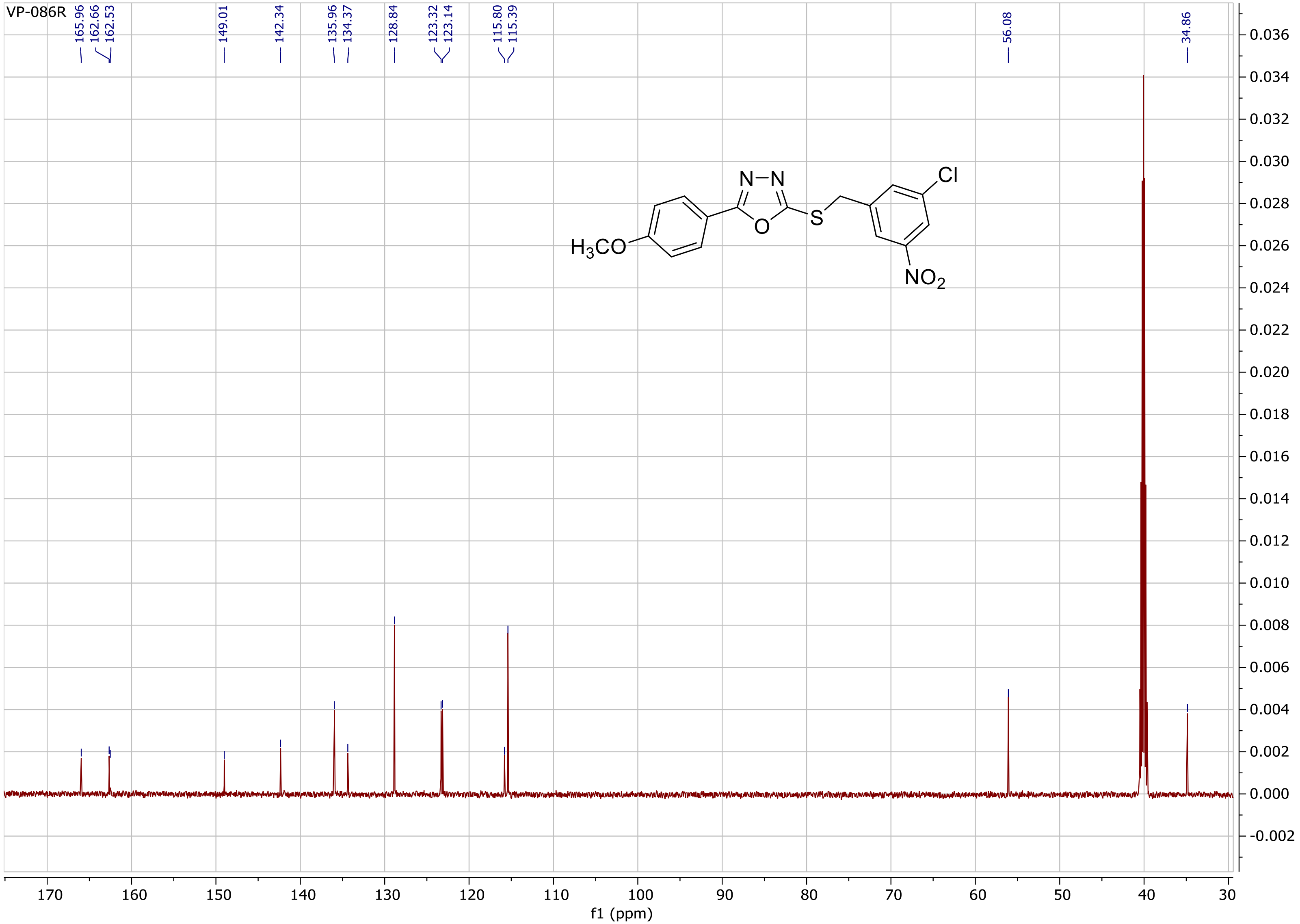

2-((3-Chloro-5-nitrobenzyl)sulfanyl)-5-(4-chlorophenyl)-1,3,4-oxadiazole (**58c**): ^{1}H NMR (500 MHz, DMSO-d_6)

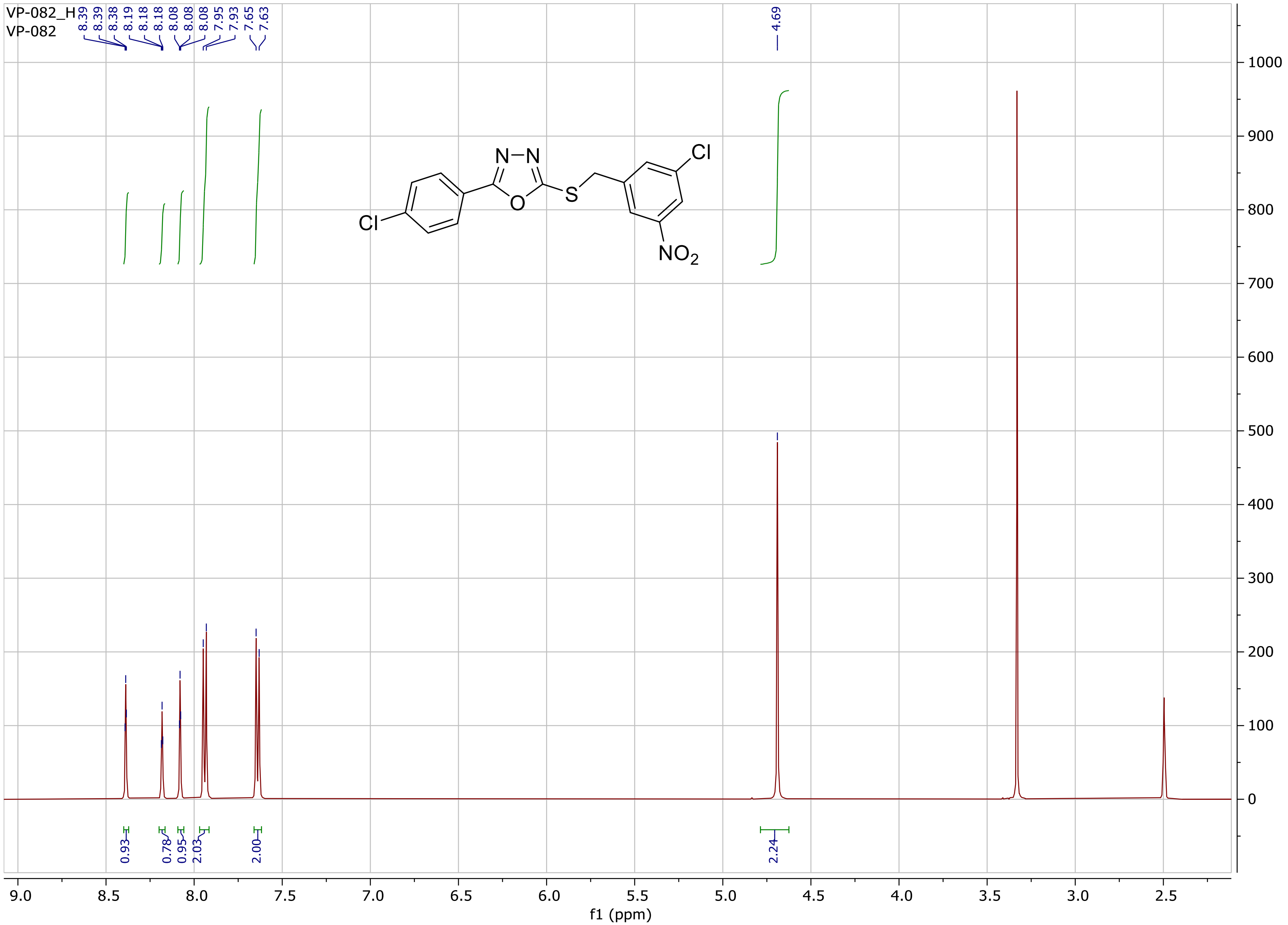

2-((3-Chloro-5-nitrobenzyl)sulfanyl)-5-(4-chlorophenyl)-1,3,4-oxadiazole (**58c**): ^{13}C NMR (126 MHz, DMSO-d_6)

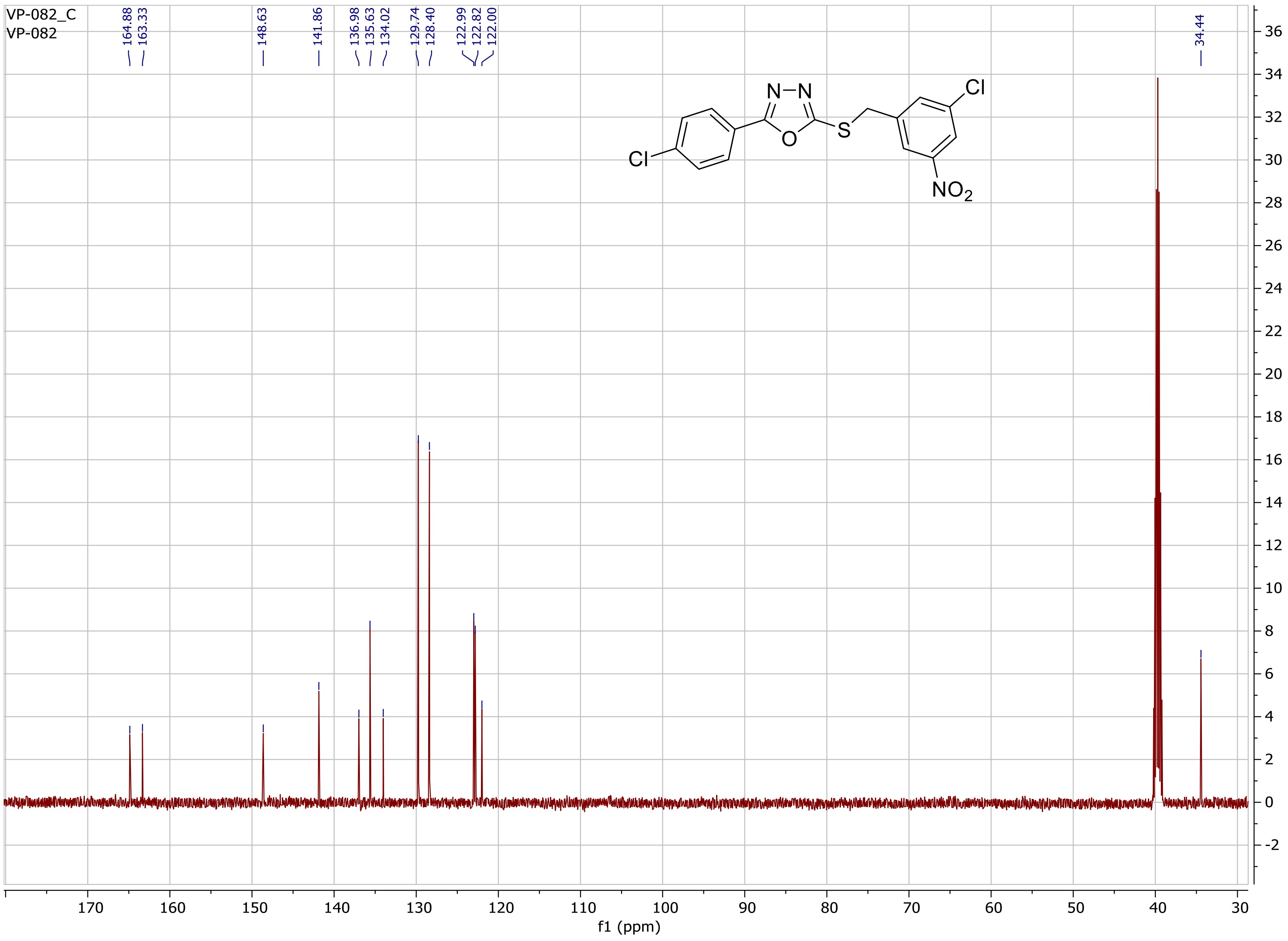

5-(4-Bromophenyl)-2-((3-Chloro-5-nitrobenzyl)sulfanyl)-1,3,4-oxadiazole (**58d**): ^{1}H NMR (600 MHz, DMSO-d_6)

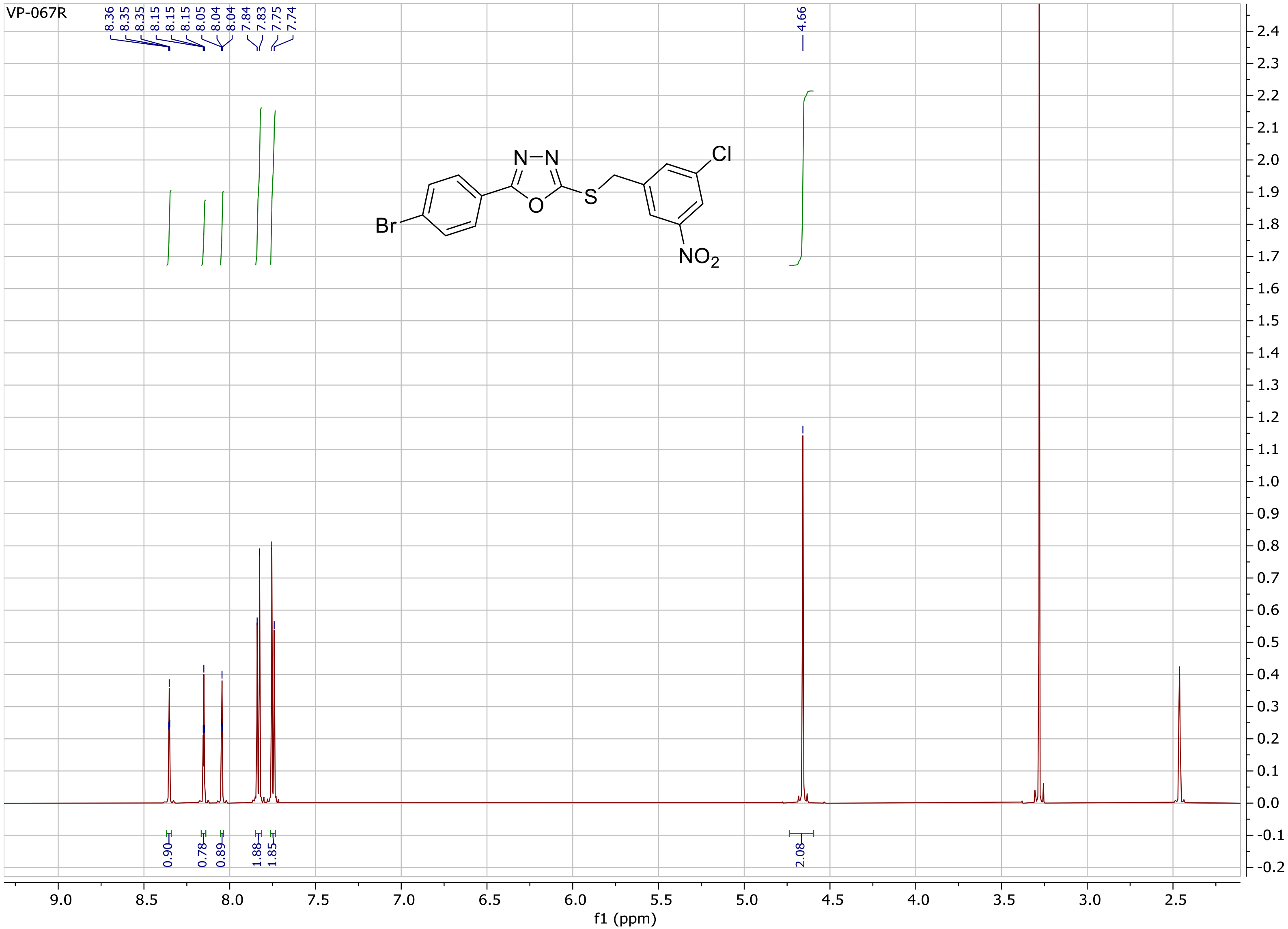

5-(4-Bromophenyl)-2-((3-Chloro-5-nitrobenzyl)sulfanyl)-1,3,4-oxadiazole (**58d**): ^{13}C NMR (151 MHz, DMSO-d_6)

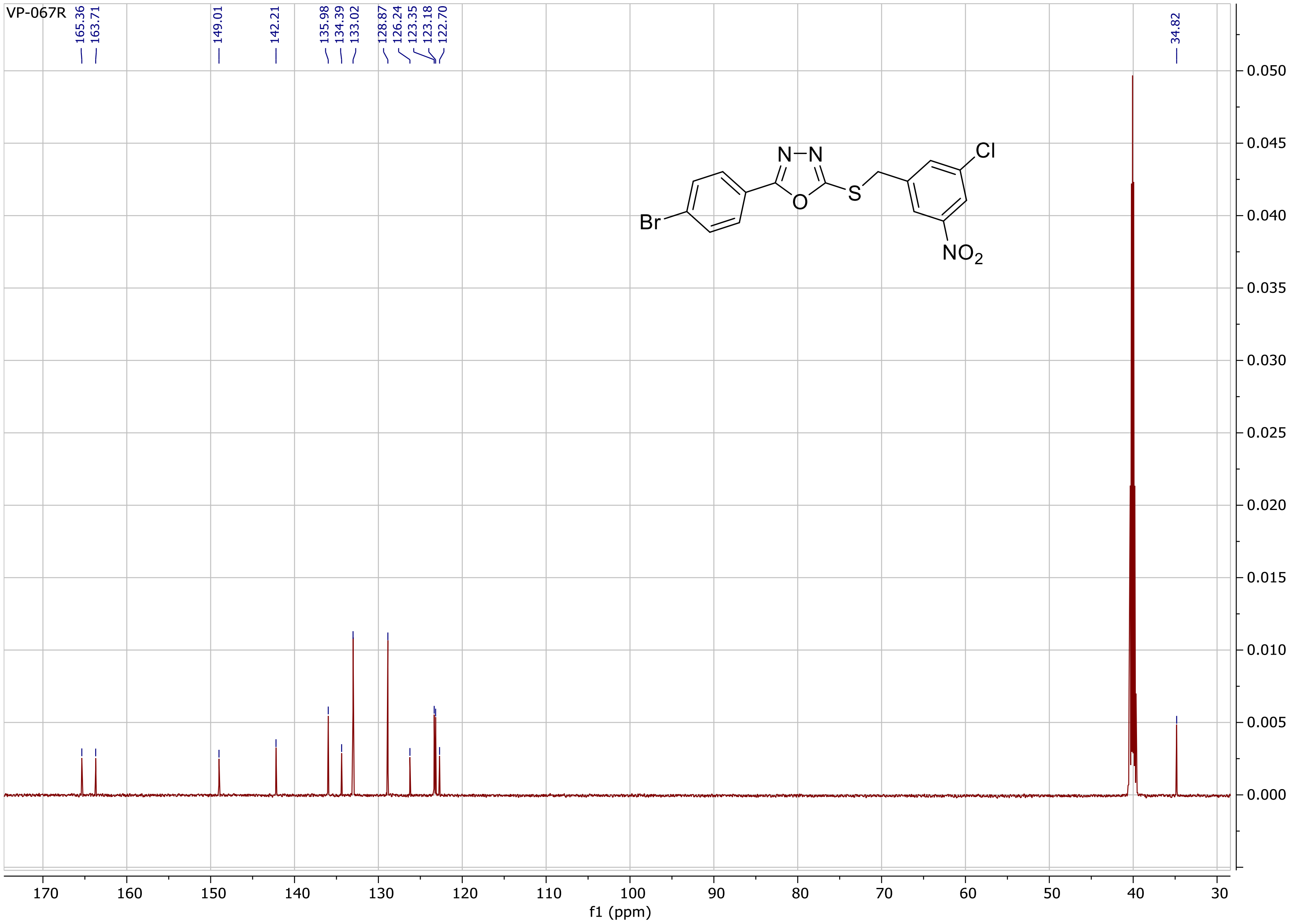

2-((3-Chloro-5-nitrobenzyl)sulfanyl)-5-cyclohexyl-1,3,4-oxadiazole (**58e**): ^{1}H NMR (500 MHz, DMSO-d_6)

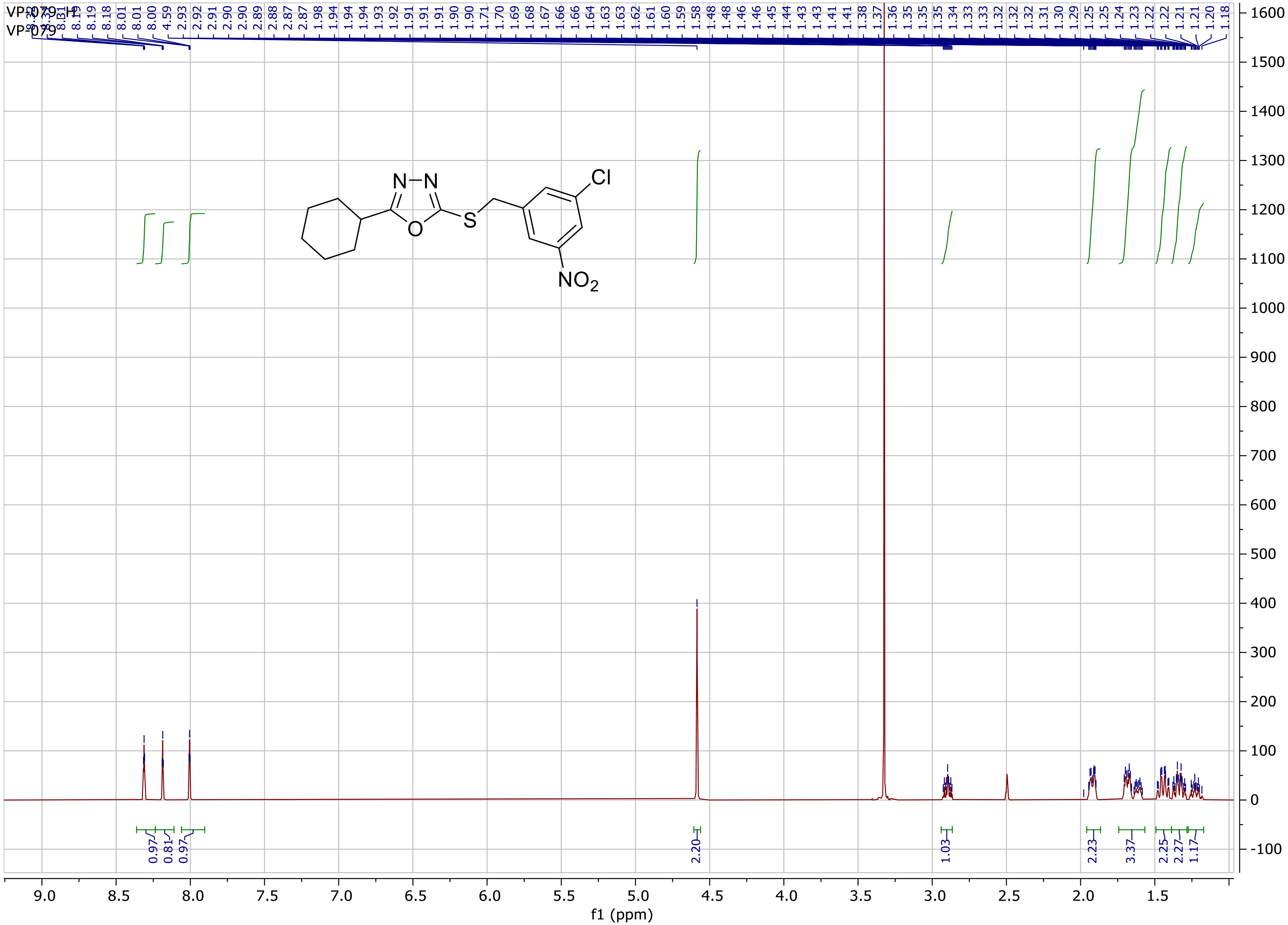

2-((3-Chloro-5-nitrobenzyl)sulfanyl)-5-cyclohexyl-1,3,4-oxadiazole (**58e**): ^{13}C NMR (126 MHz, DMSO-d_6)

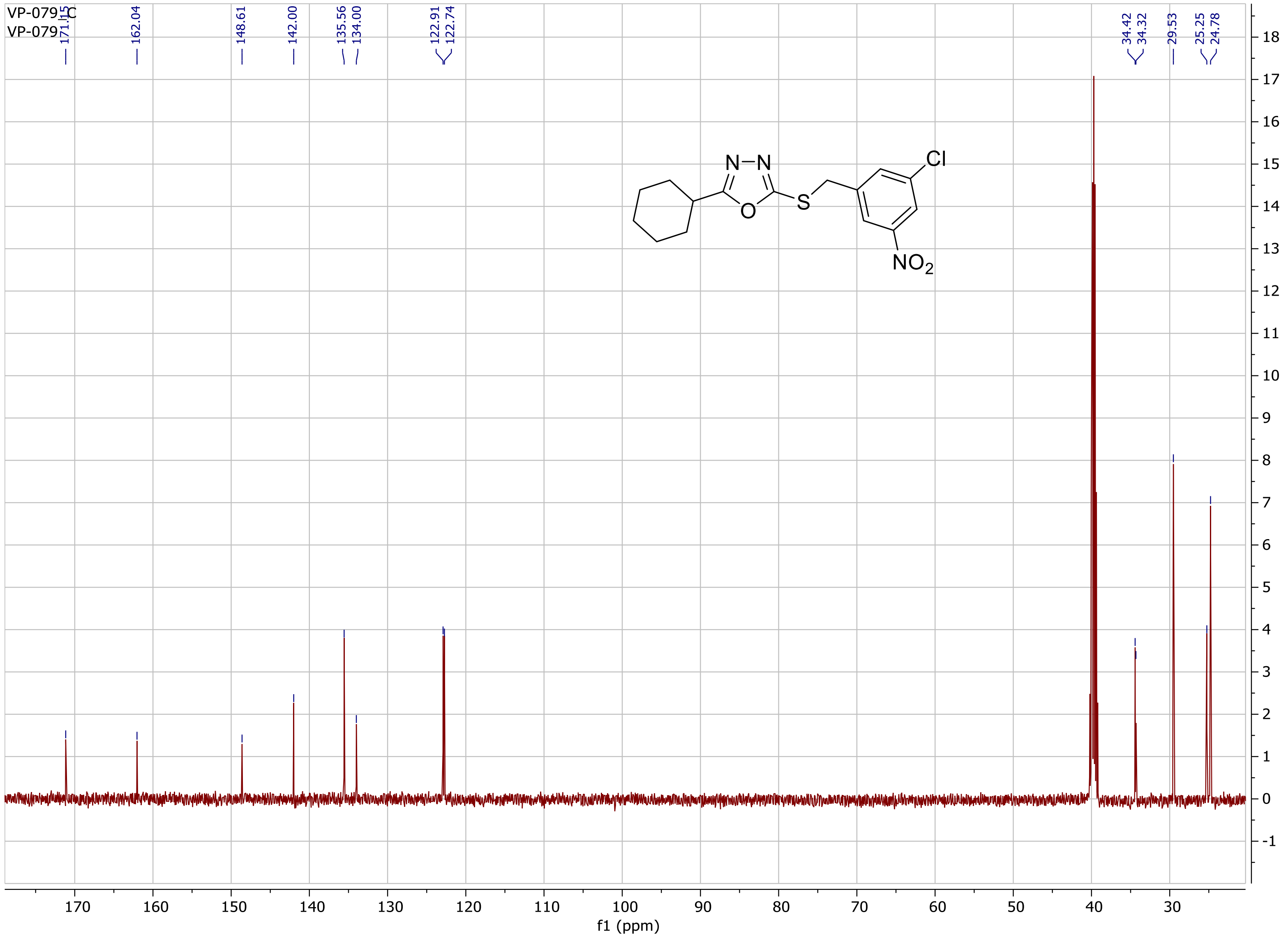

2-((3-Fluoro-5-nitrobenzyl)sulfanyl)-5-phenyl-1,3,4-oxadiazole (**59a**): ^{1}H NMR (600 MHz, DMSO-d_6)

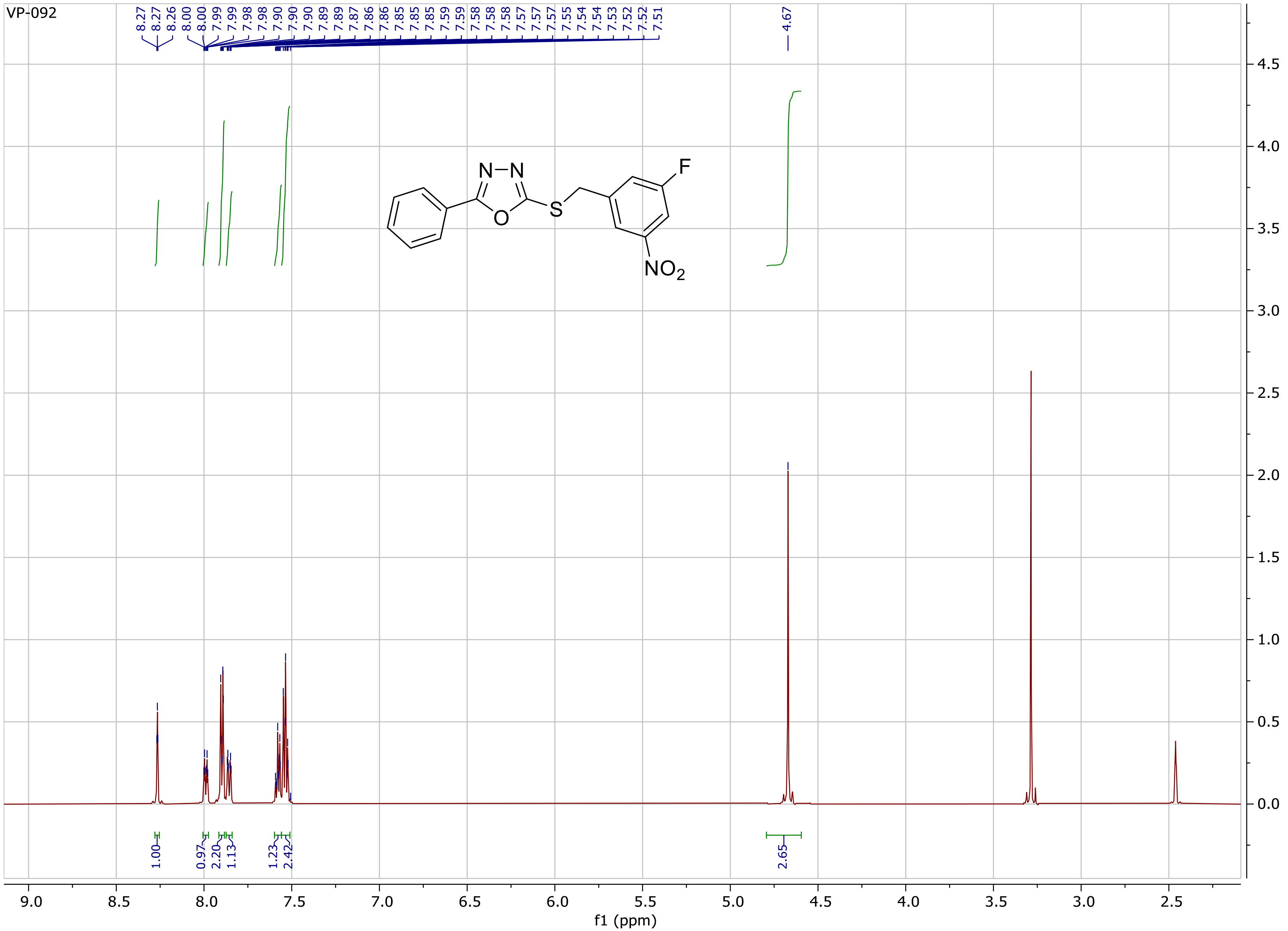

2-((3-Fluoro-5-nitrobenzyl)sulfanyl)-5-phenyl-1,3,4-oxadiazole (**59a**): ^{13}C NMR (151 MHz, DMSO-d_6)

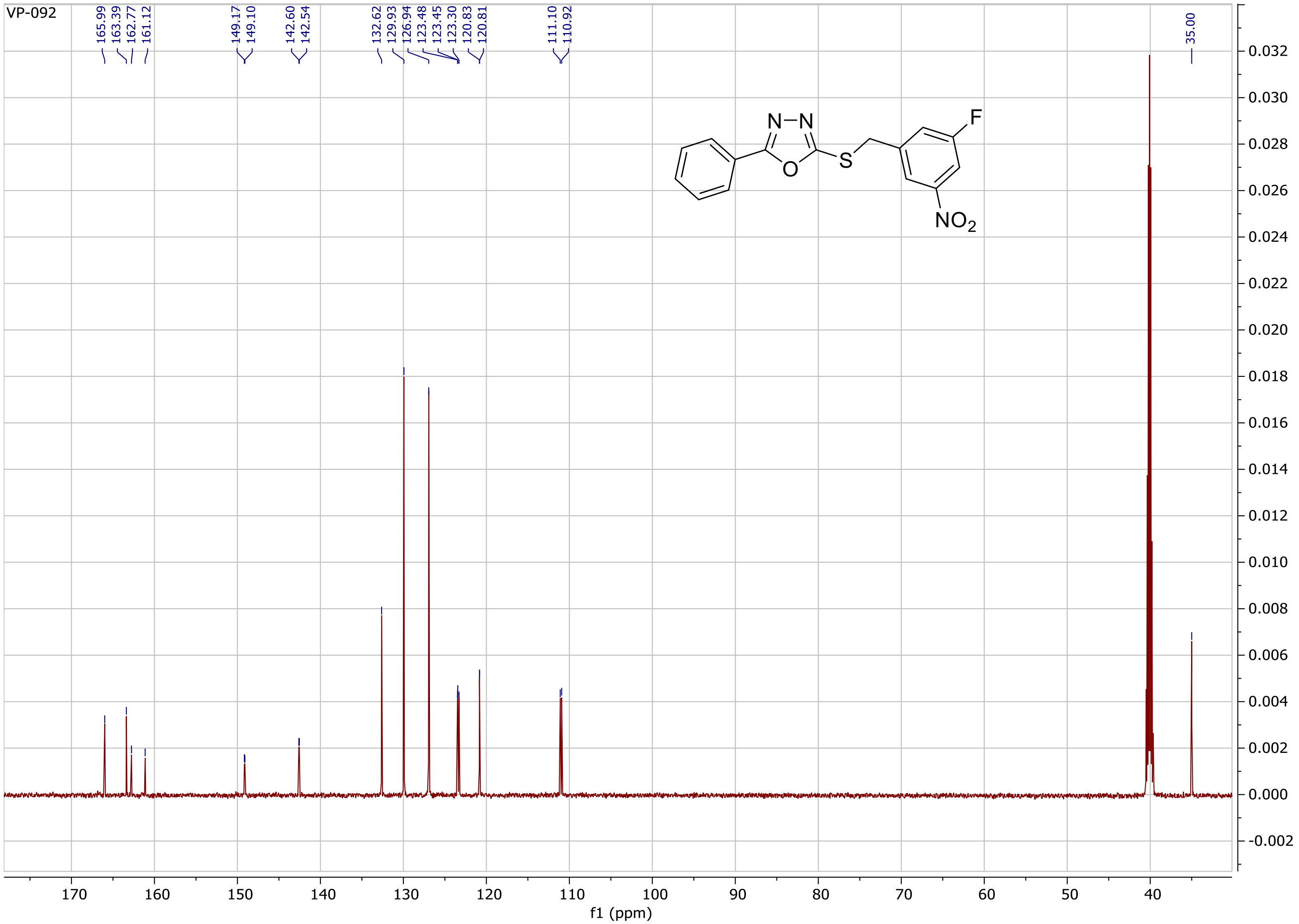

2-((3-Fluoro-5-nitrobenzyl)sulfanyl)-5-phenyl-1,3,4-oxadiazole (**59a**):

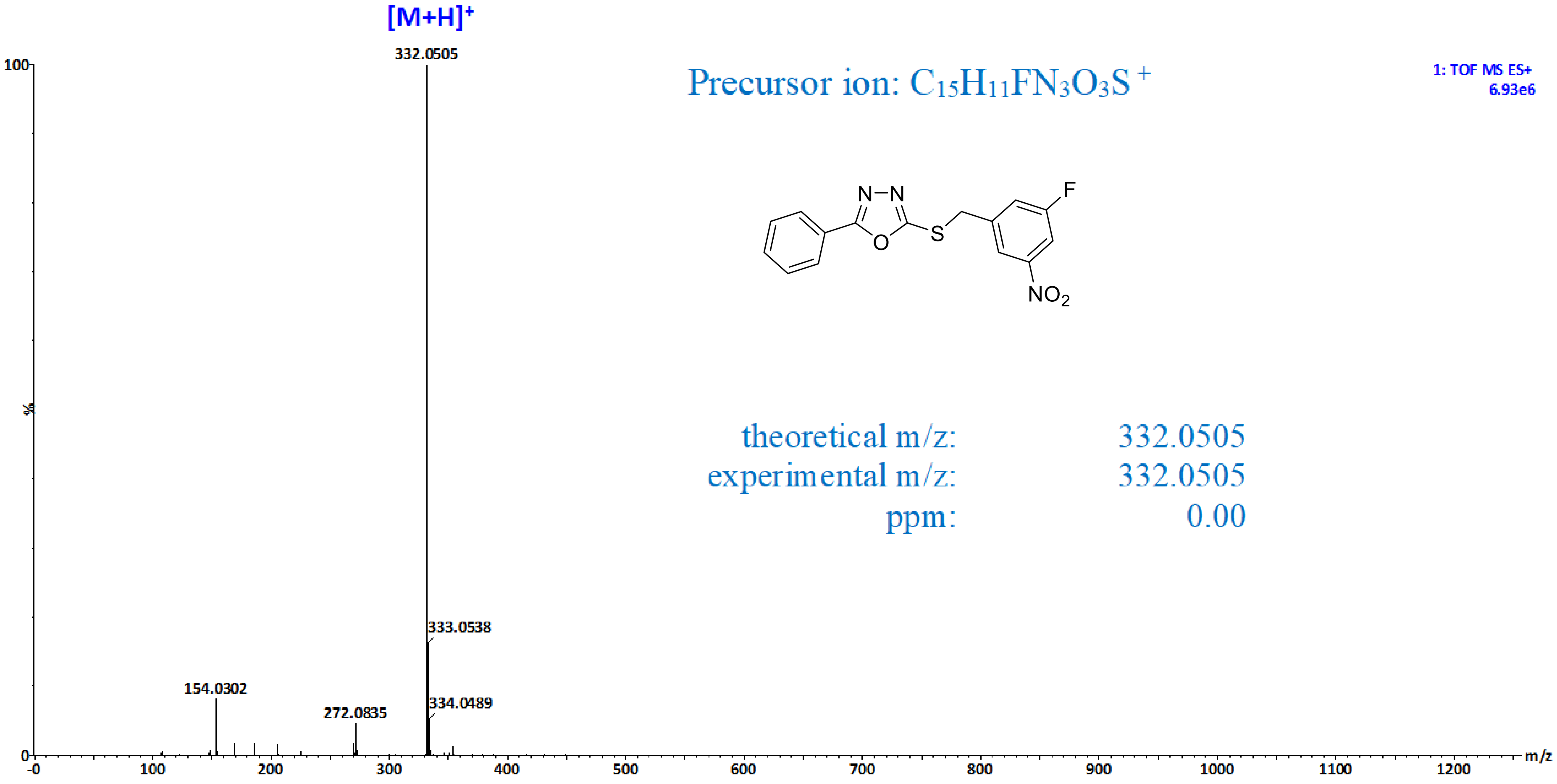

2-((3-Fluoro-5-nitrobenzyl)sulfanyl)-5-(4-methoxyphenyl)-1,3,4-oxadiazole (**59b**): ^{1}H NMR (600 MHz, DMSO-d_6)

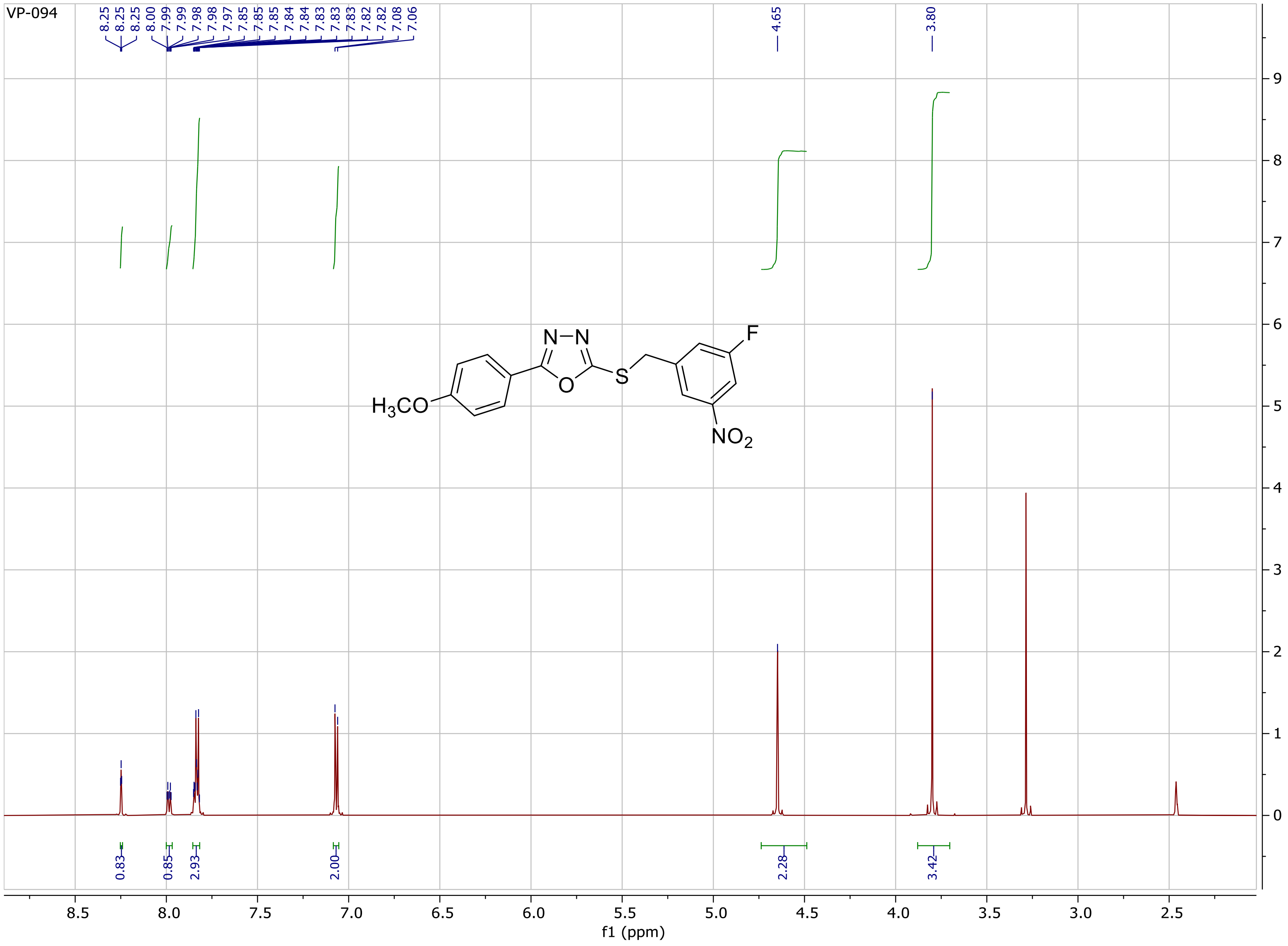

2-((3-Fluoro-5-nitrobenzyl)sulfanyl)-5-(4-methoxyphenyl)-1,3,4-oxadiazole (**59b**): ^{13}C NMR (151 MHz, DMSO-d_6)

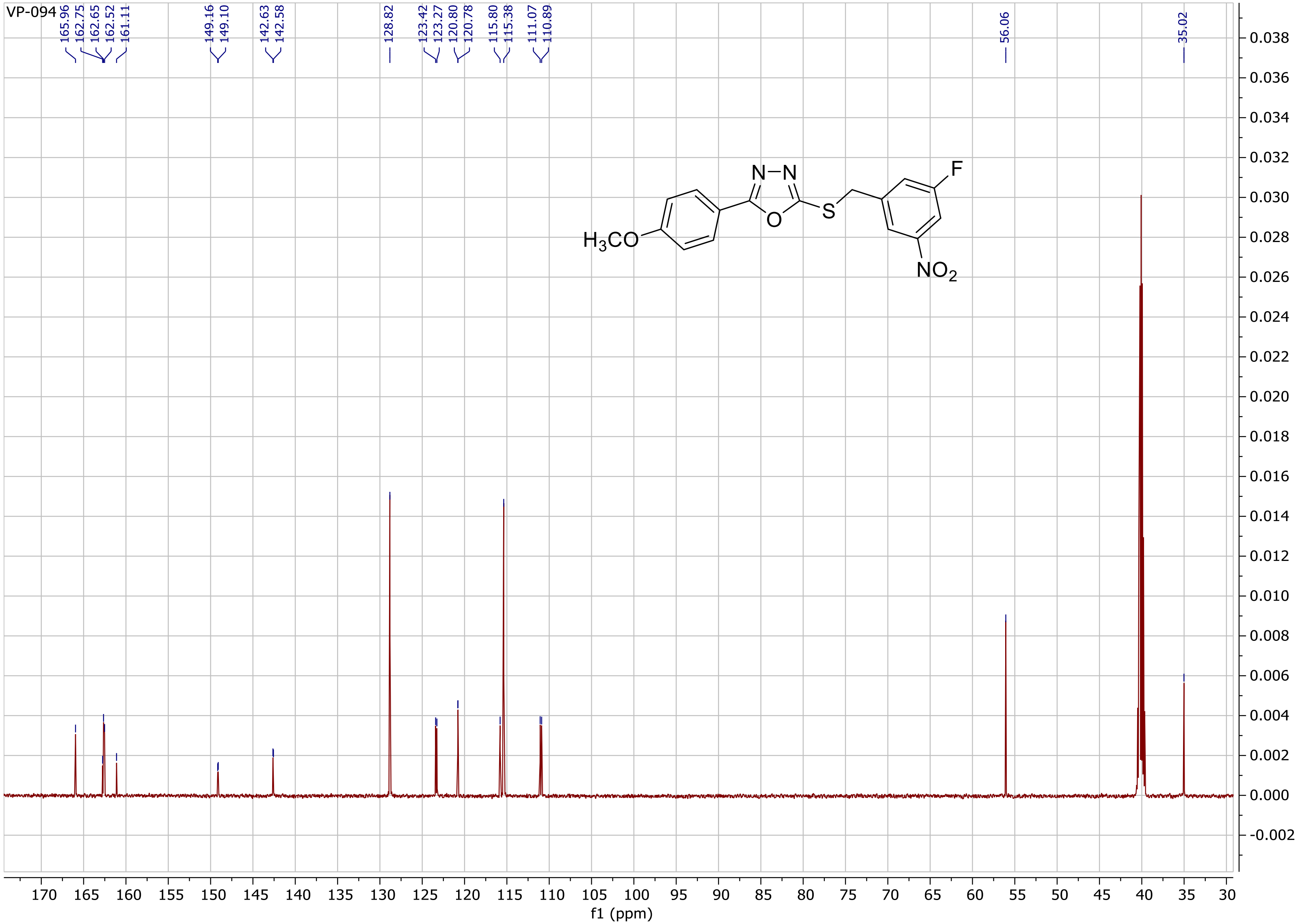

2-((3-Fluoro-5-nitrobenzyl)sulfanyl)-5-(4-methoxyphenyl)-1,3,4-oxadiazole (**59b**):

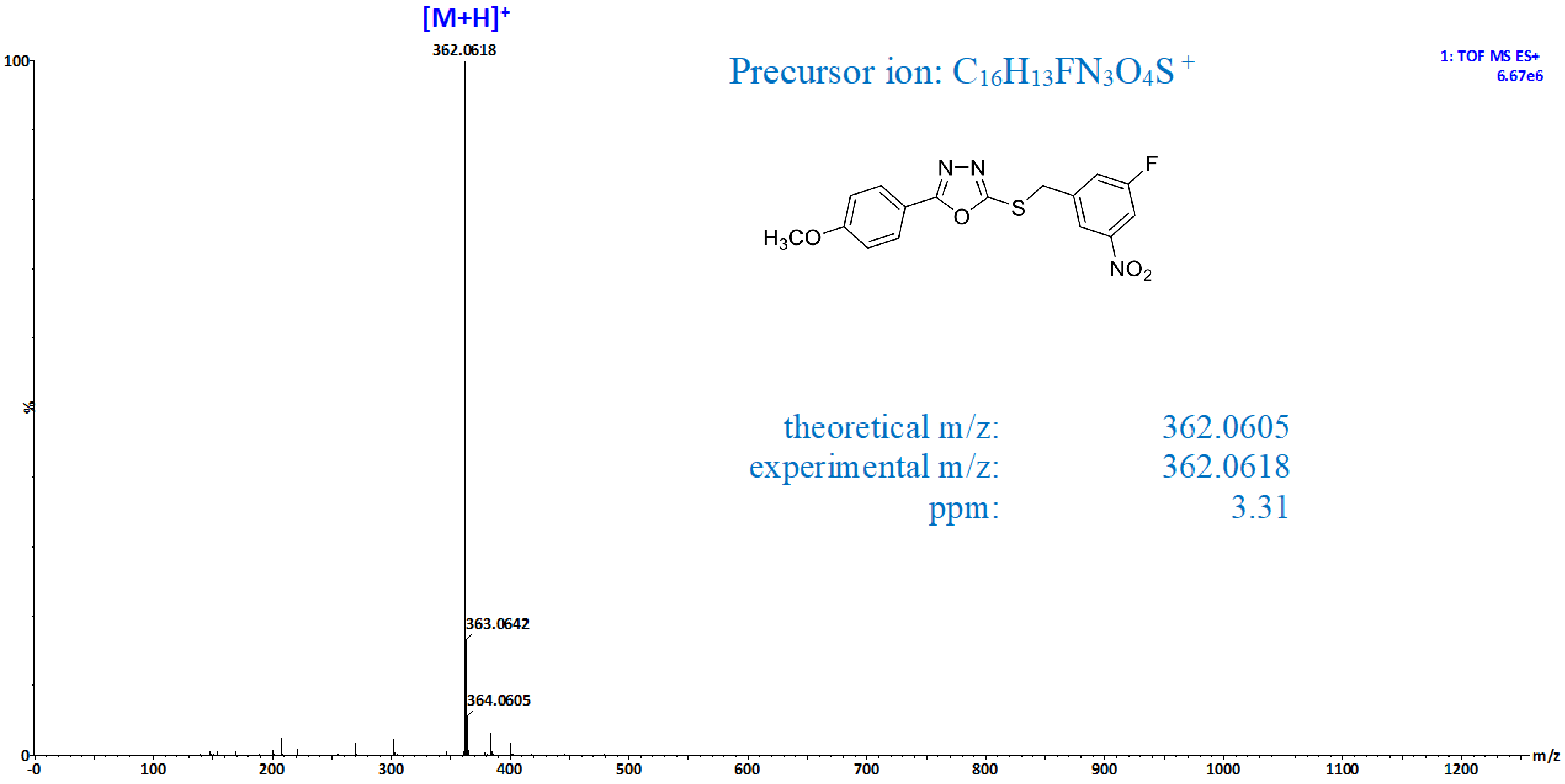

2-(4-Chlorophenyl)-5-((3-fluoro-5-nitrobenzyl)sulfanyl)-1,3,4-oxadiazole (**59c**): ^{1}H NMR (600 MHz, DMSO-d_6)

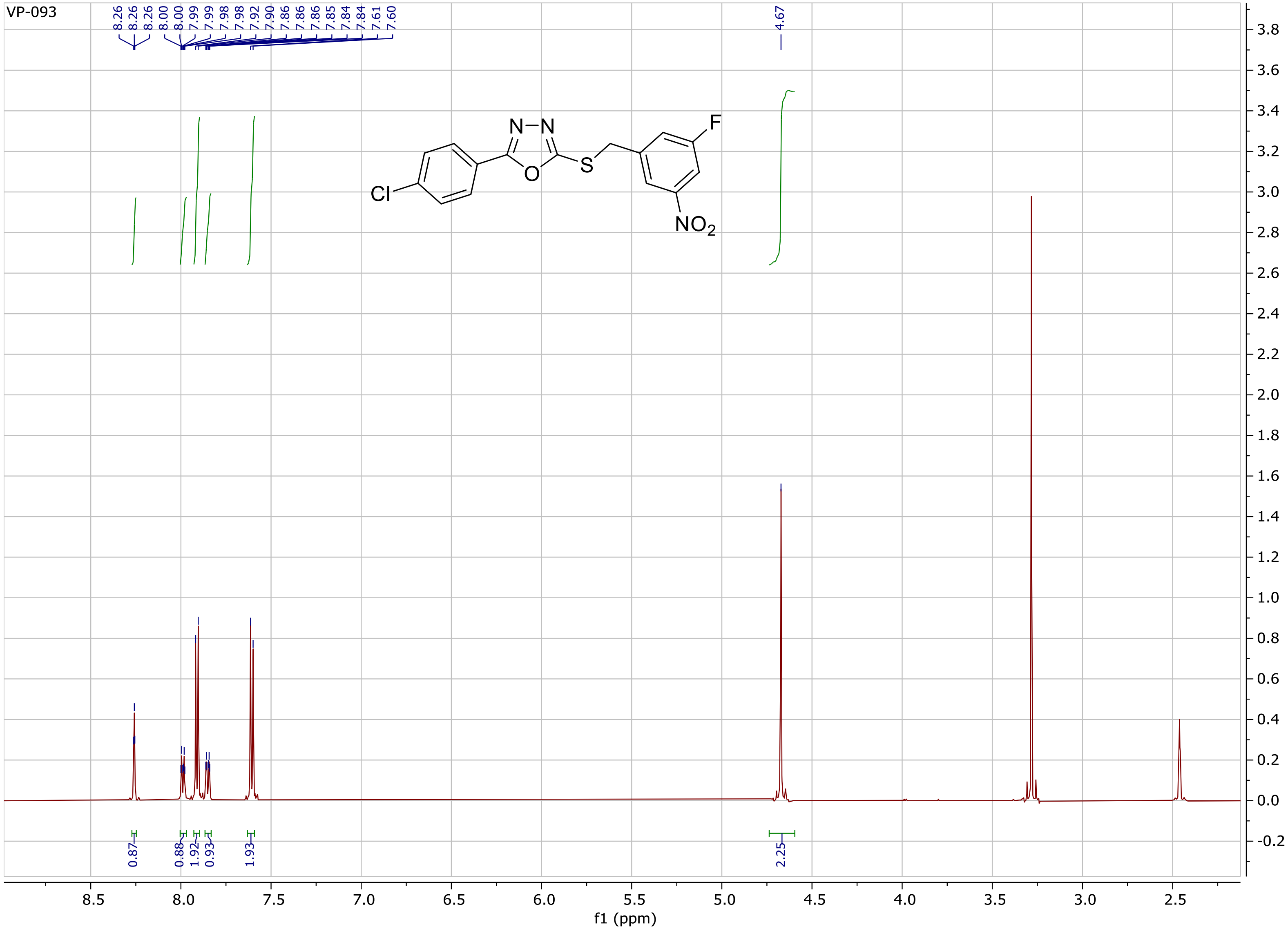

2-(4-Chlorophenyl)-5-((3-fluoro-5-nitrobenzyl)sulfanyl)-1,3,4-oxadiazole (**59c**): ^{13}C NMR (151 MHz, DMSO-d_6)

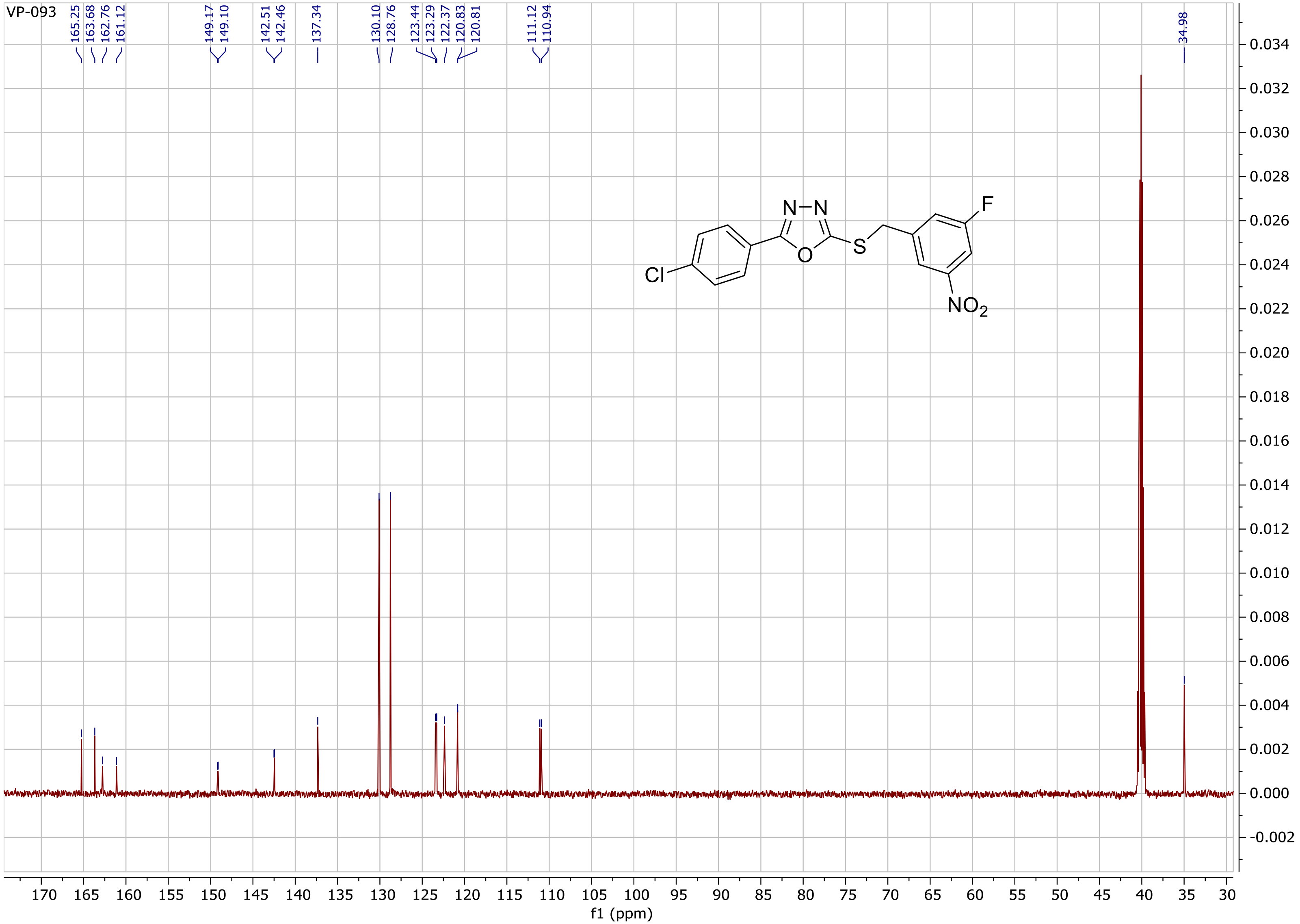

2-(4-Chlorophenyl)-5-((3-fluoro-5-nitrobenzyl)sulfanyl)-1,3,4-oxadiazole (**59c**):

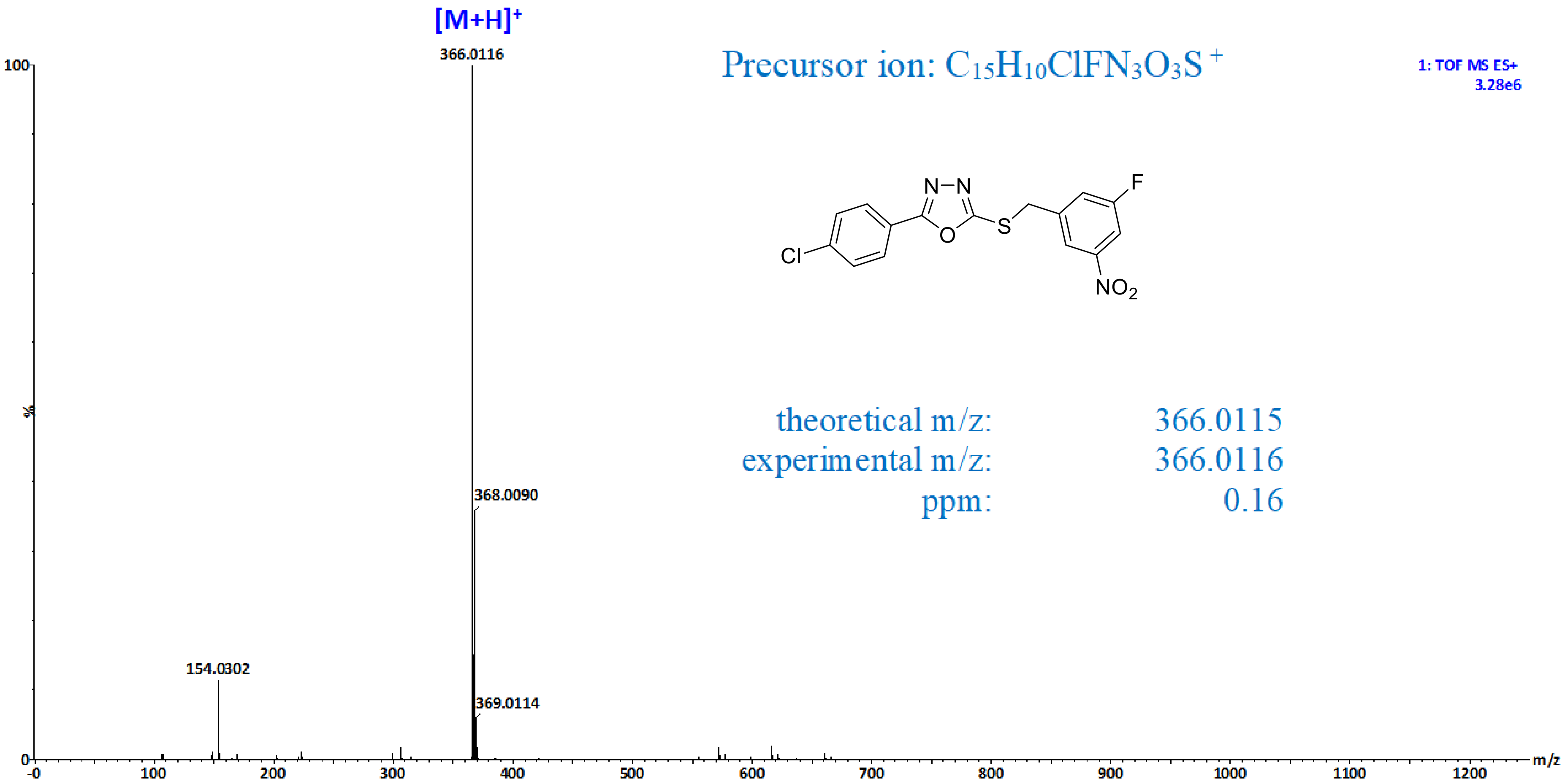

2-(4-Bromophenyl)-5-((3-fluoro-5-nitrobenzyl)sulfanyl)-1,3,4-oxadiazole (**59d**): ^{1}H NMR (500 MHz, DMSO-d_6)

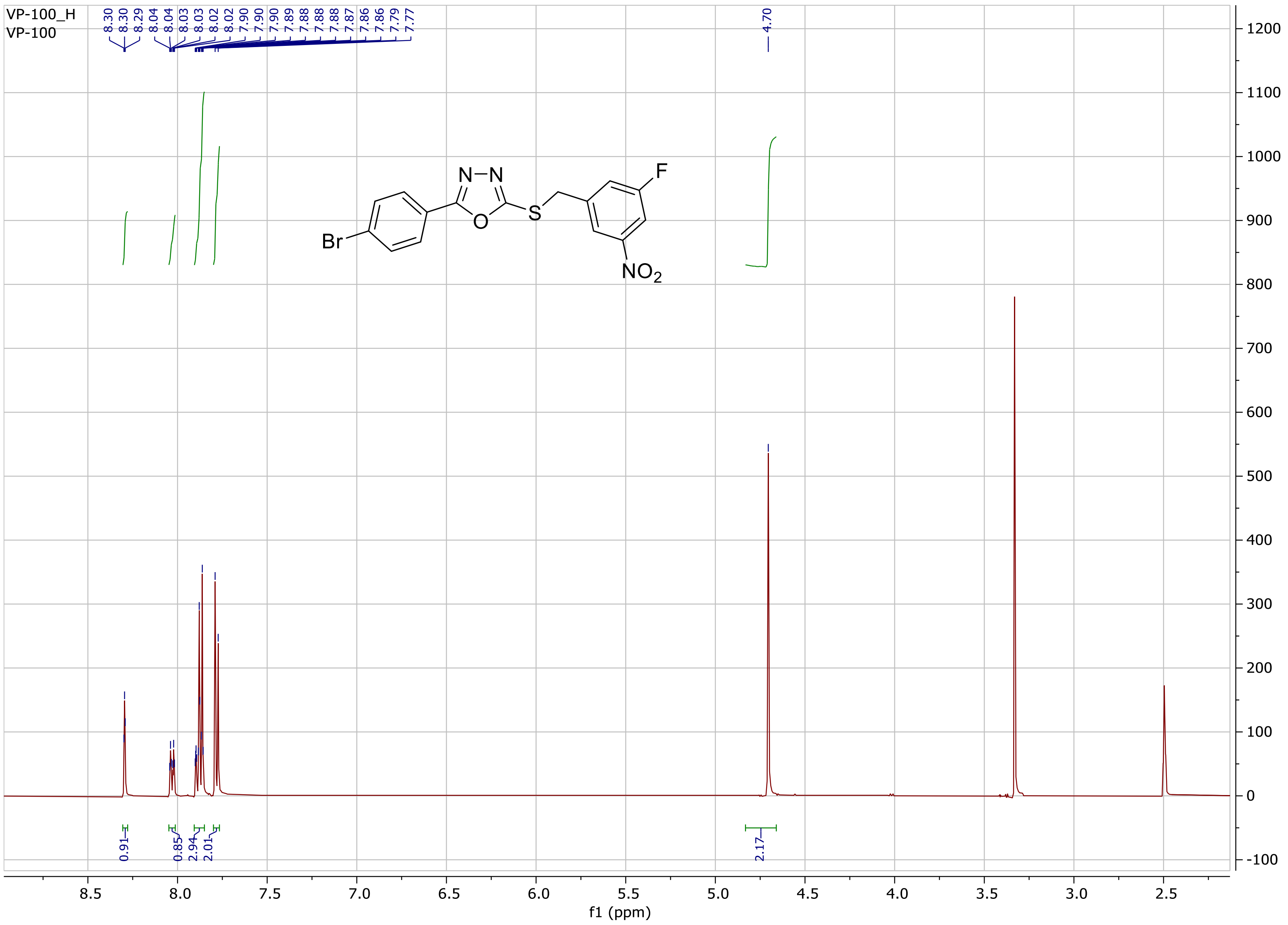

2-(4-Bromophenyl)-5-((3-fluoro-5-nitrobenzyl)sulfanyl)-1,3,4-oxadiazole (**59d**): ^{13}C NMR (126 MHz, DMSO-d_6)

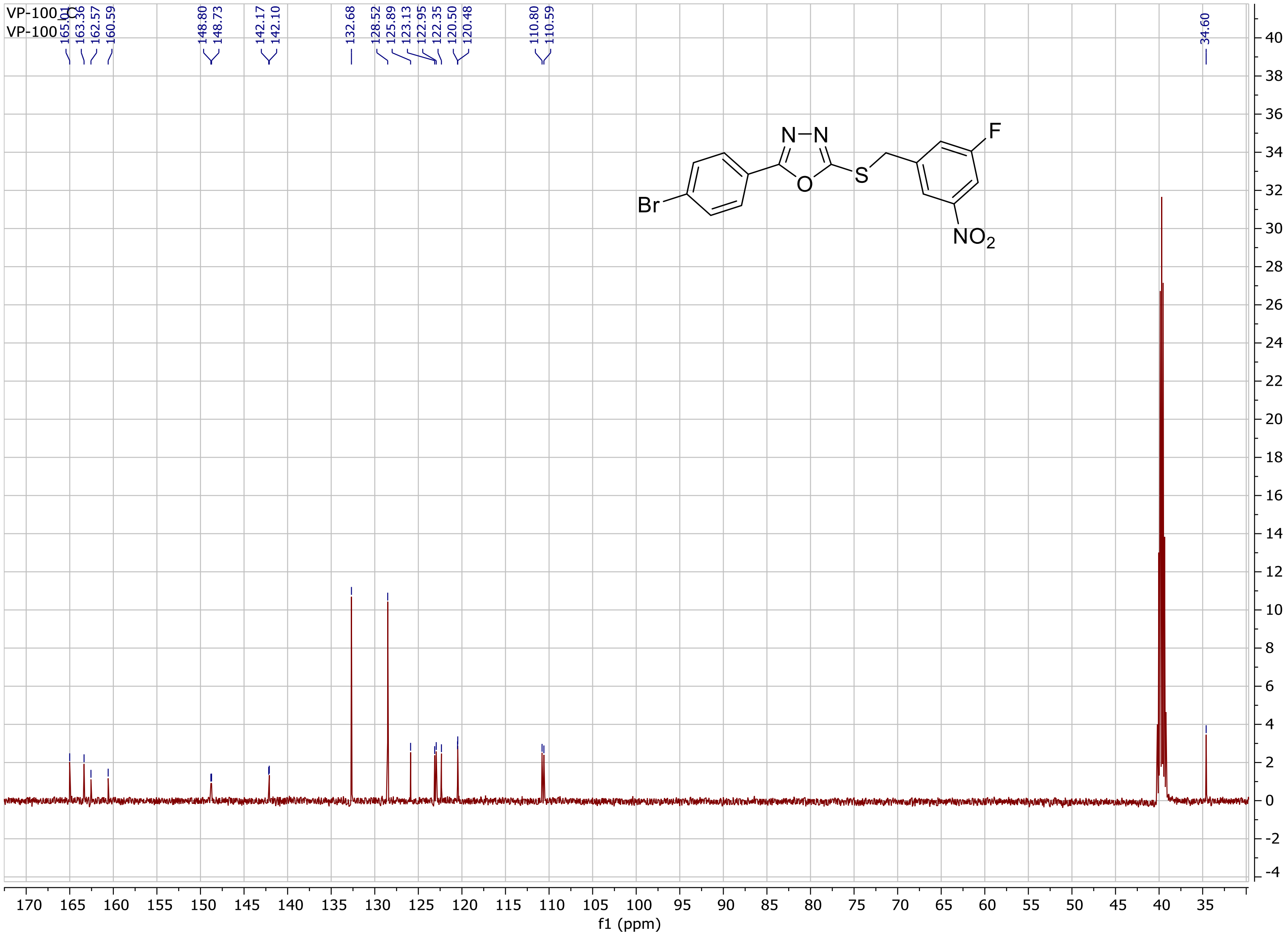

2-(4-Bromophenyl)-5-((3-fluoro-5-nitrobenzyl)sulfanyl)-1,3,4-oxadiazole (**59d**):

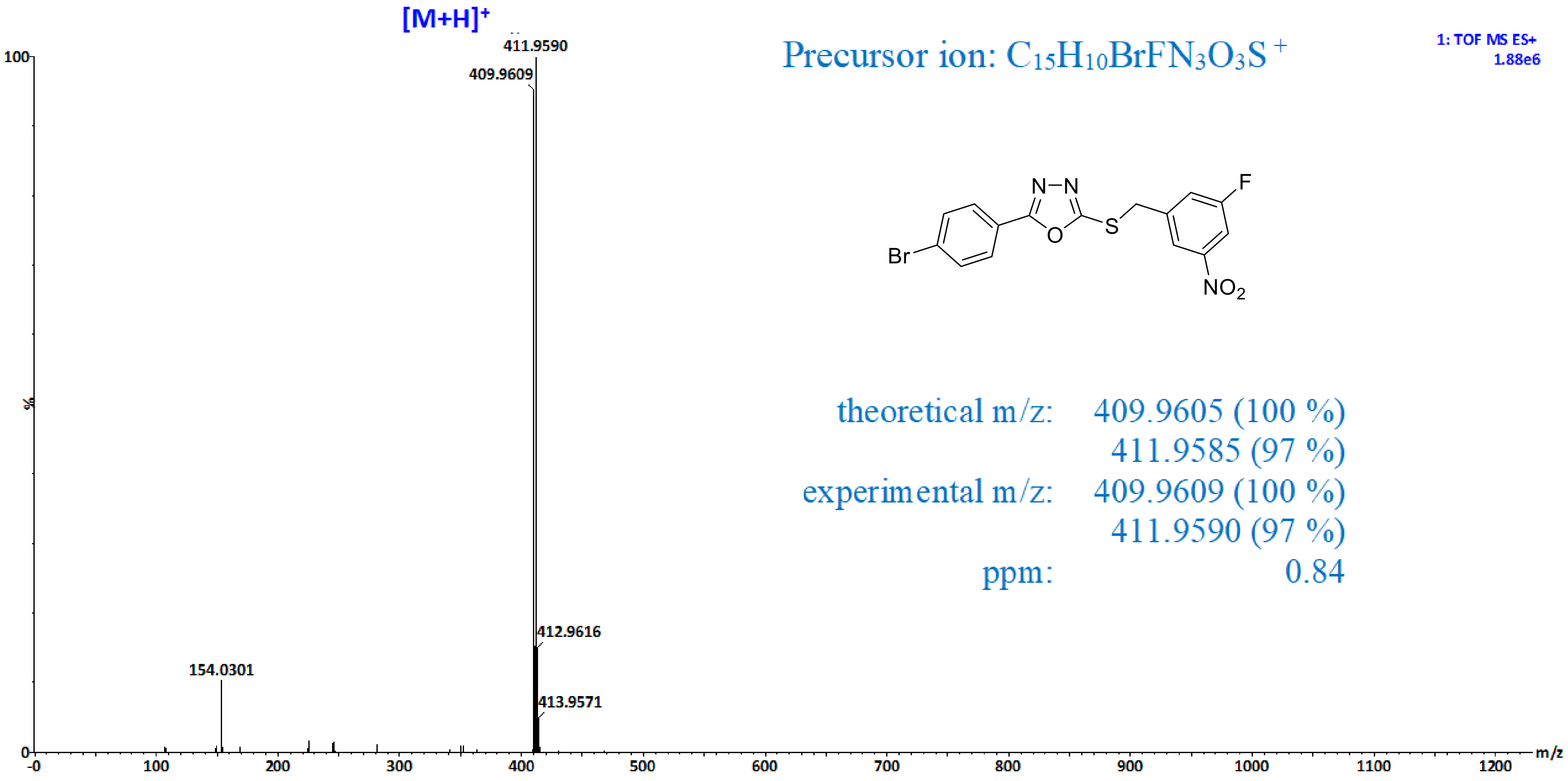

2-Cyclohexyl-5-((3-fluoro-5-nitrobenzyl)sulfanyl)-1,3,4-oxadiazole (**59e**): ^{1}H NMR (500 MHz, DMSO-d_6)

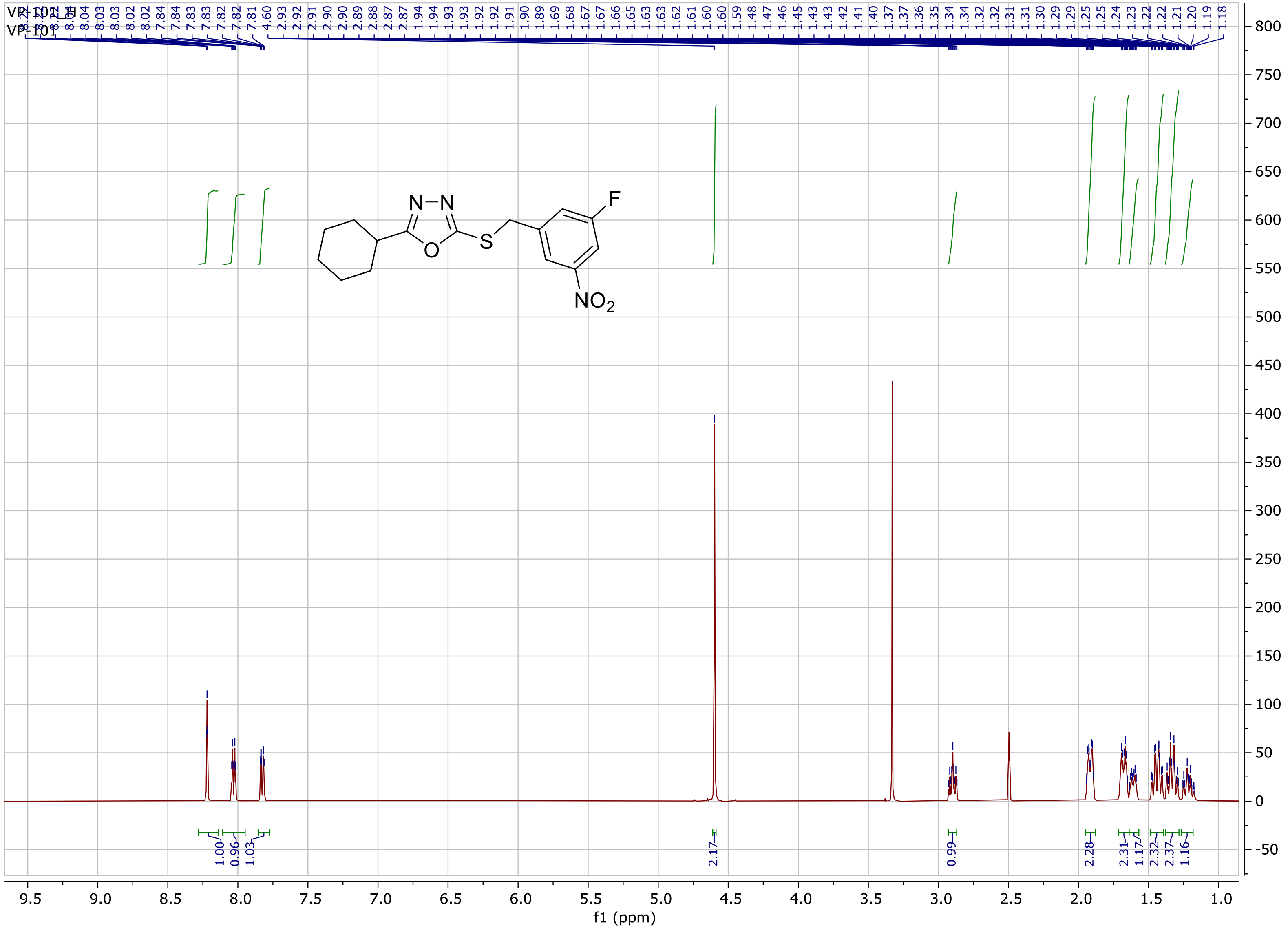

2-Cyclohexyl-5-((3-fluoro-5-nitrobenzyl)sulfanyl)-1,3,4-oxadiazole (**59e**): ^{13}C NMR (126 MHz, DMSO-d_6)

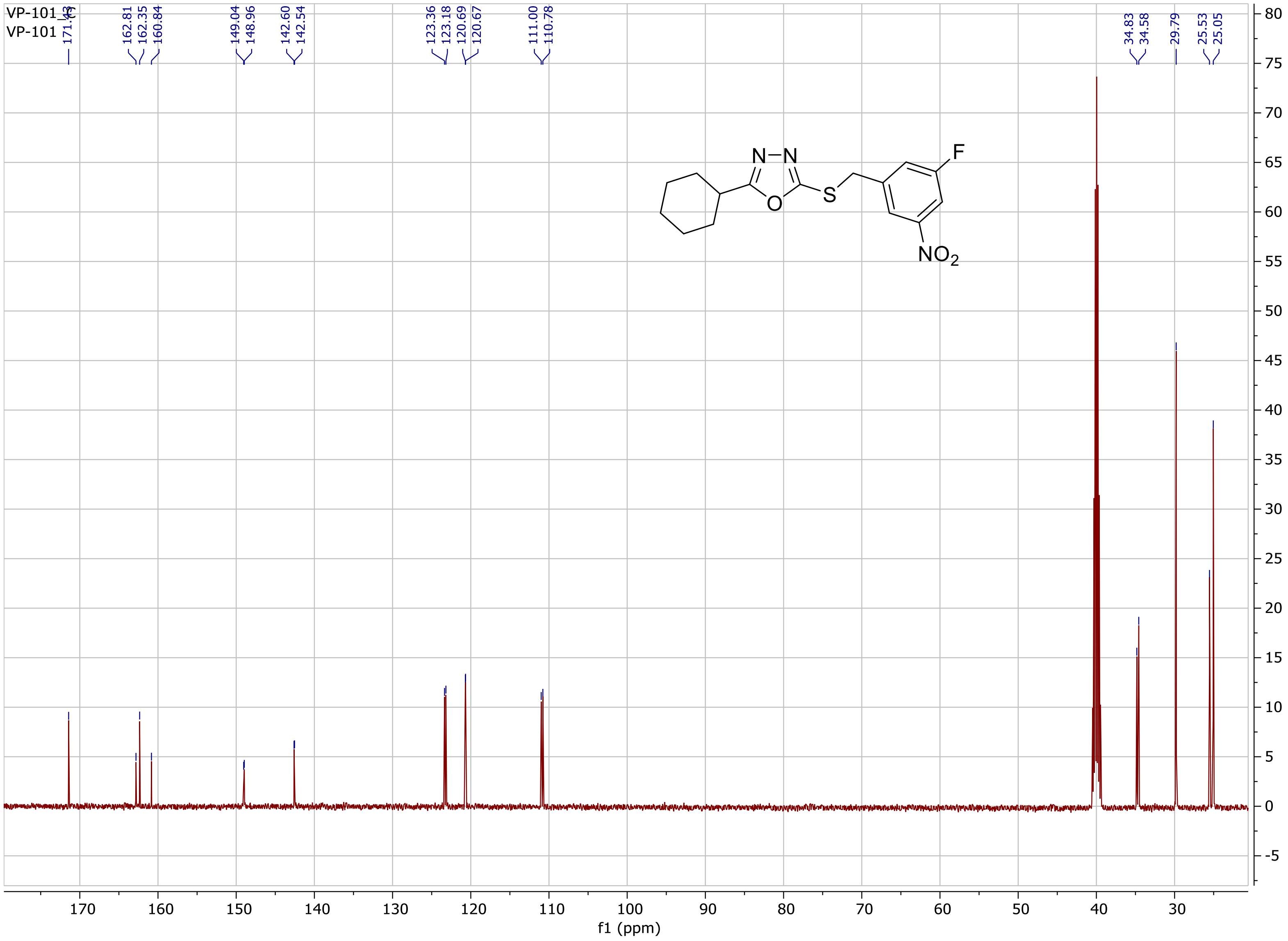

2-Cyclohexyl-5-((3-fluoro-5-nitrobenzyl)sulfanyl)-1,3,4-oxadiazole (**59e**):

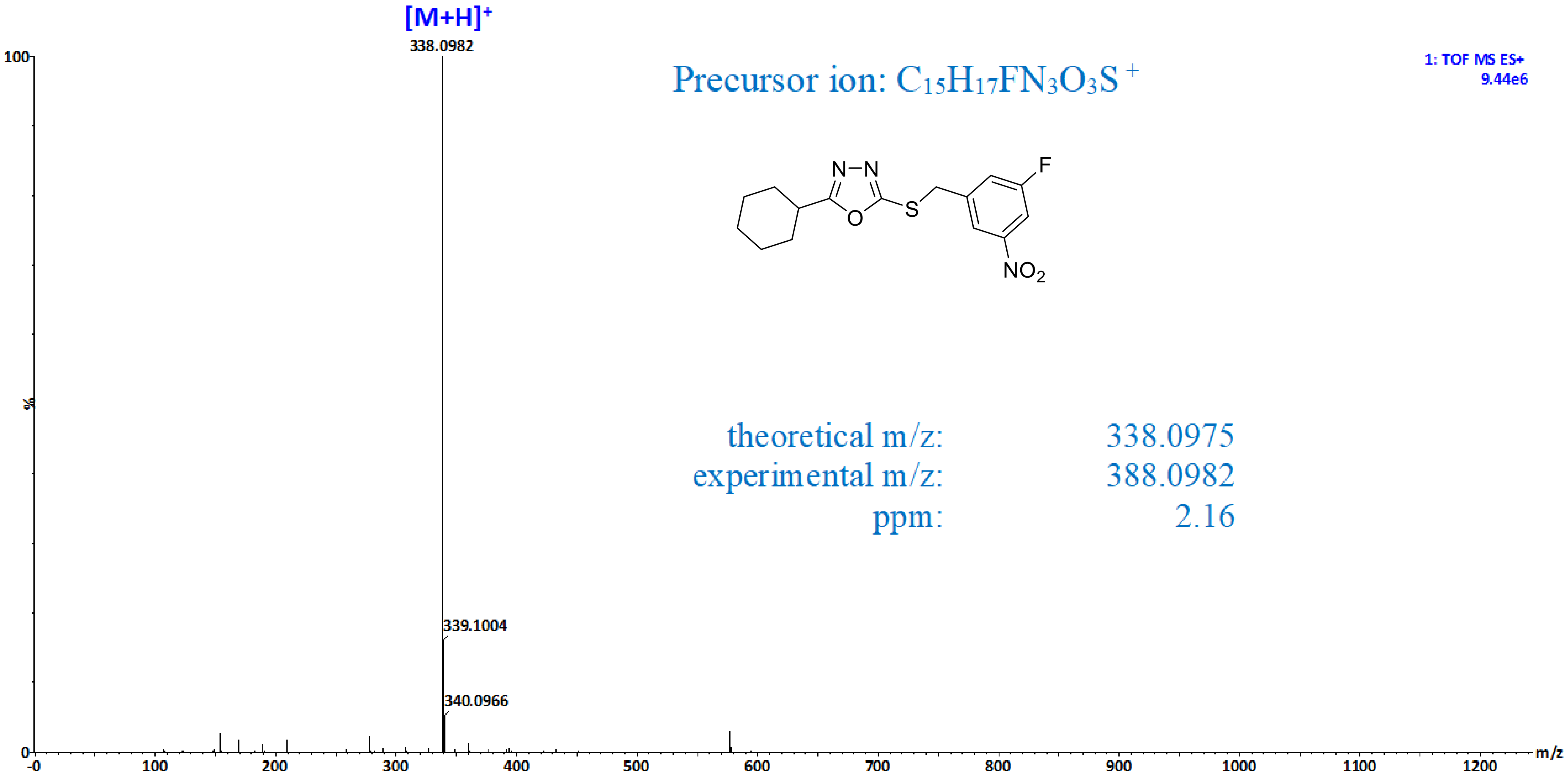

2-((3-Bromo-5-nitrobenzyl)sulfanyl)-5-phenyl-1,3,4-oxadiazole (**60a**): ^{1}H NMR (500 MHz, DMSO-d_6)

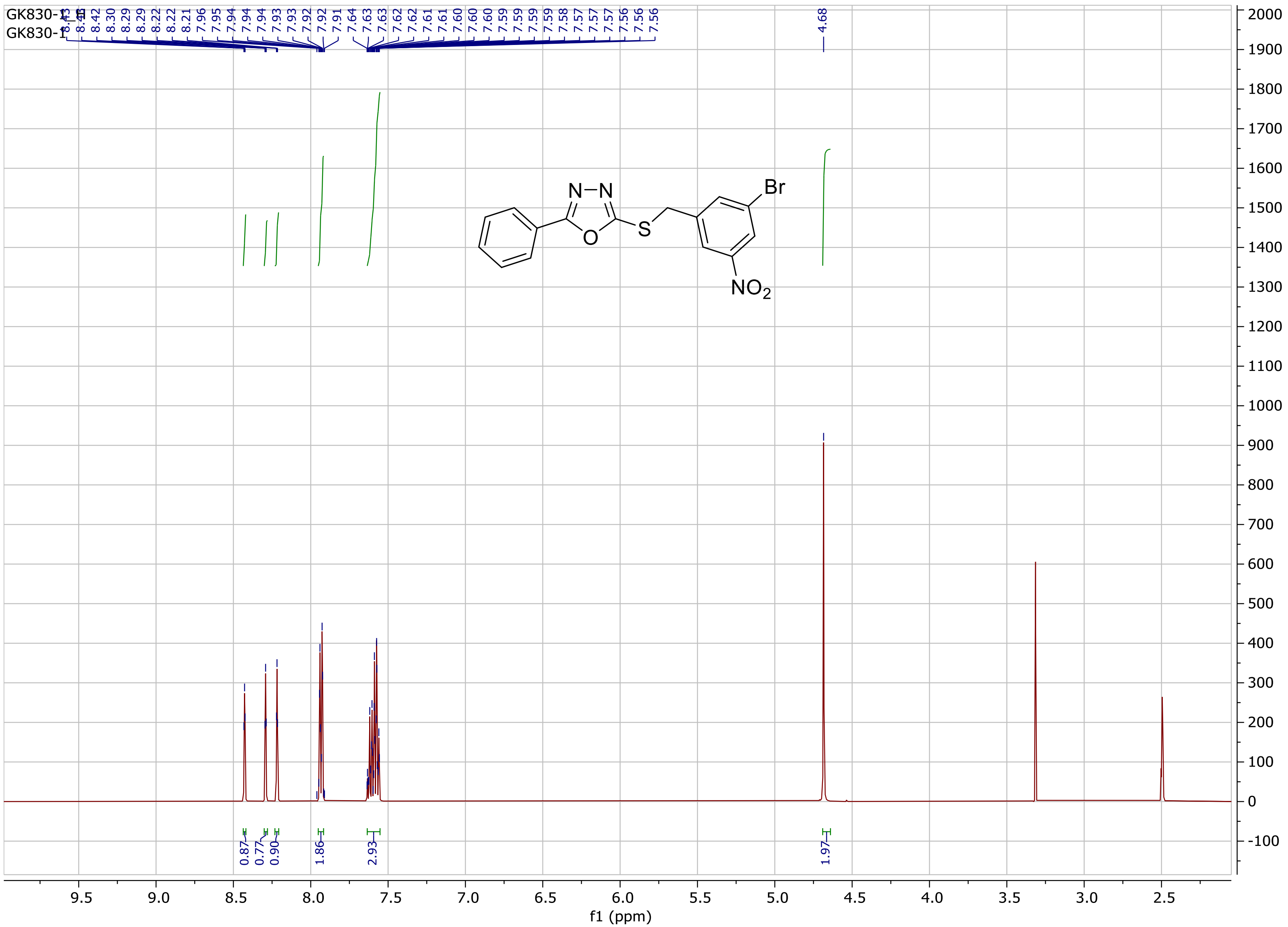

2-((3-Bromo-5-nitrobenzyl)sulfanyl)-5-phenyl-1,3,4-oxadiazole (**60a**): ^{13}C NMR (126 MHz, DMSO-d_6)

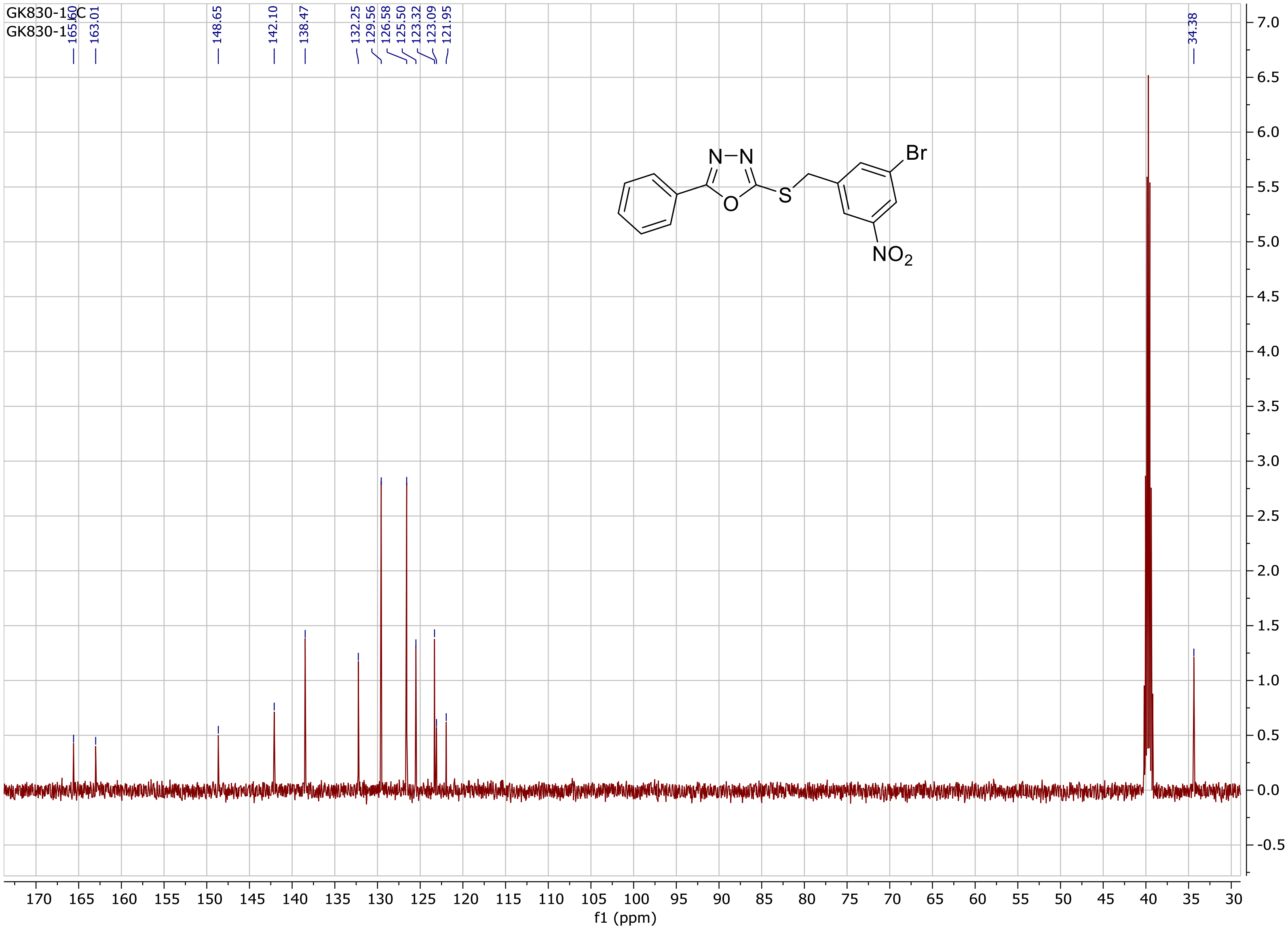

2-((3-Bromo-5-nitrobenzyl)sulfanyl)-5-(4-methoxyphenyl)-1,3,4-oxadiazole (**60b**): ^{1}H NMR (500 MHz, DMSO-d_6)

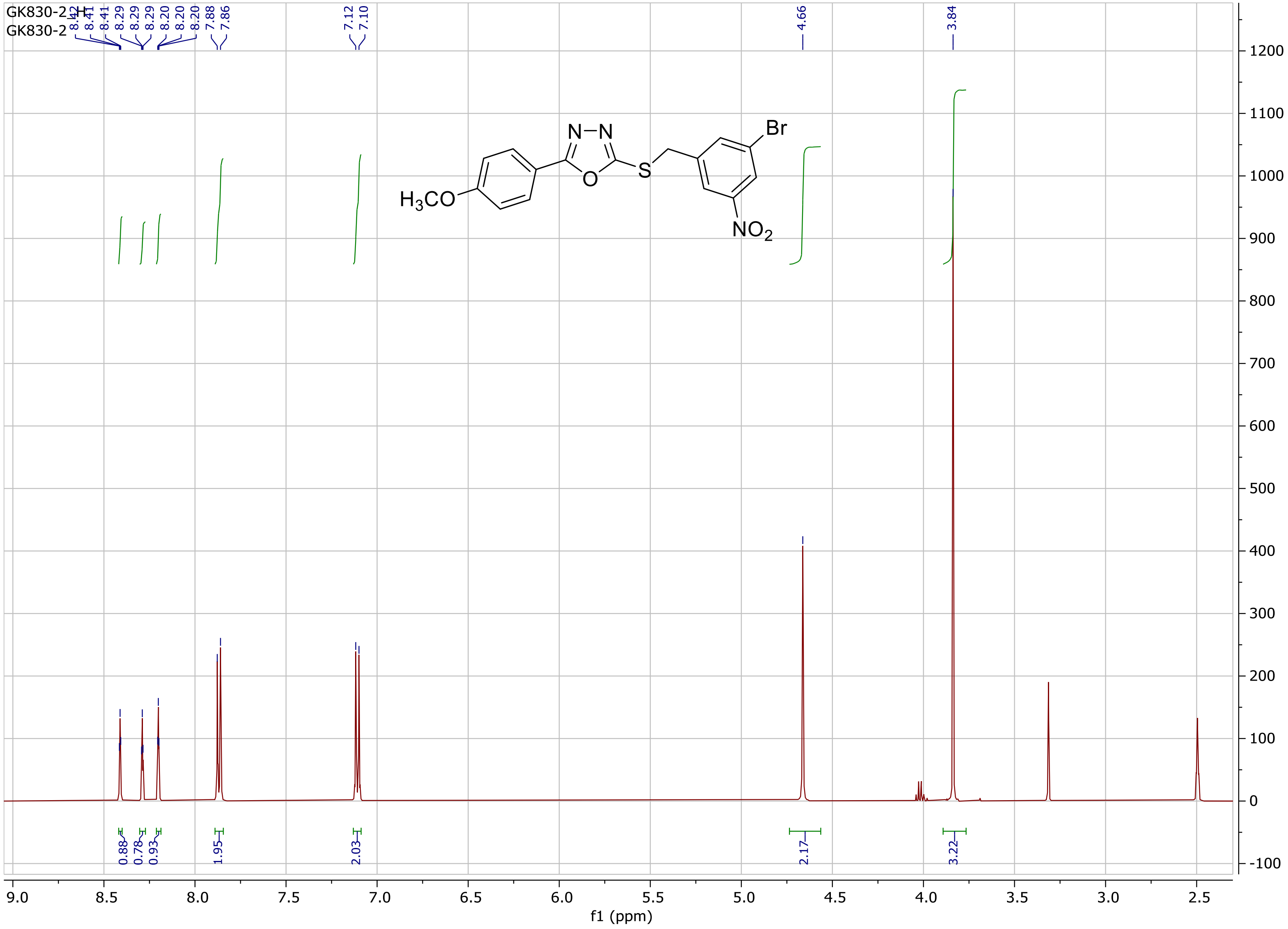

2-((3-Bromo-5-nitrobenzyl)sulfanyl)-5-(4-methoxyphenyl)-1,3,4-oxadiazole (**60b**): ^{13}C NMR (126 MHz, DMSO-d_6)

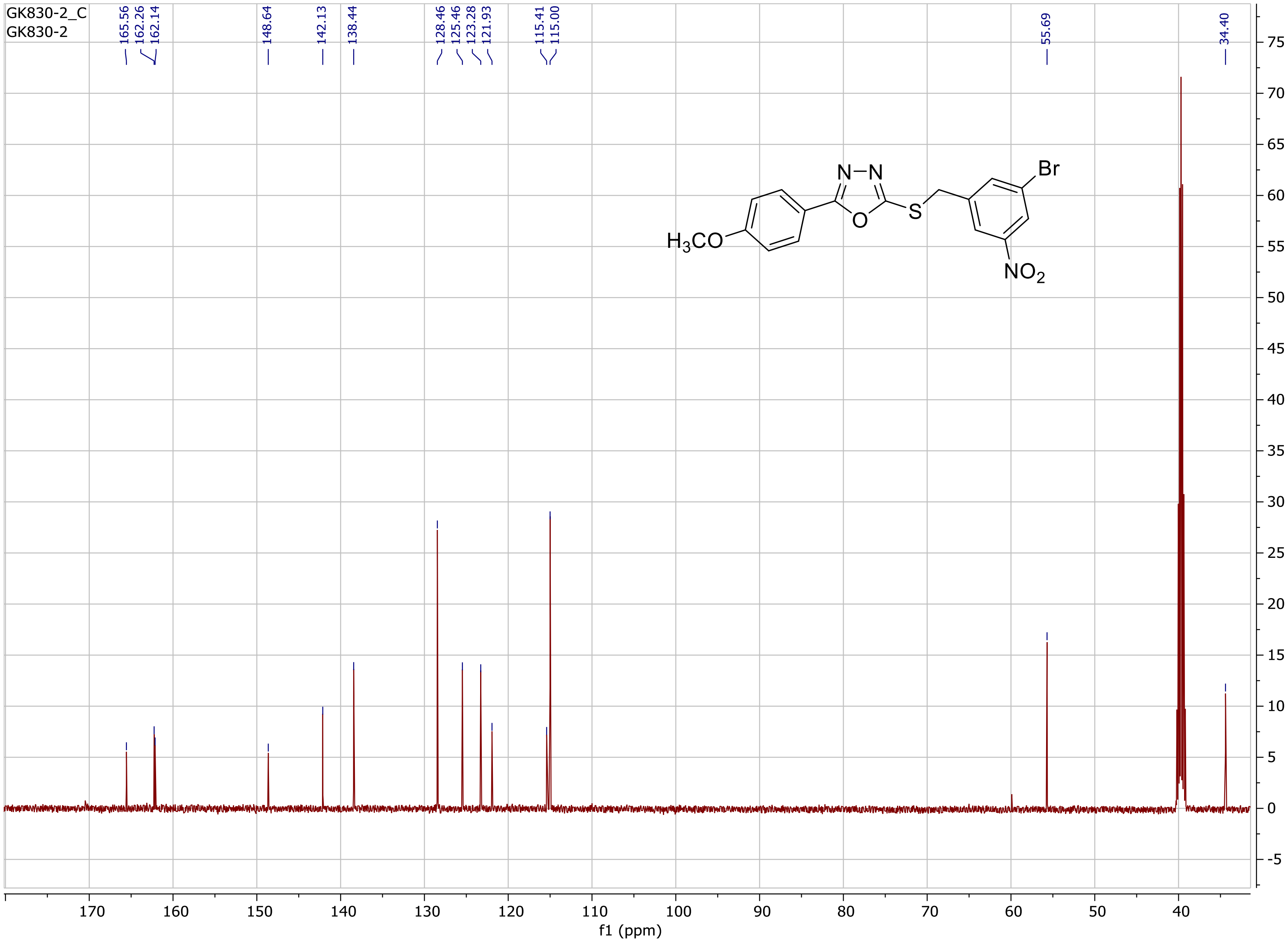

2-((3-Bromo-5-nitrobenzyl)sulfanyl)-5-(4-chlorophenyl)-1,3,4-oxadiazole (**60c**): ^{1}H NMR (500 MHz, DMSO-d_6)

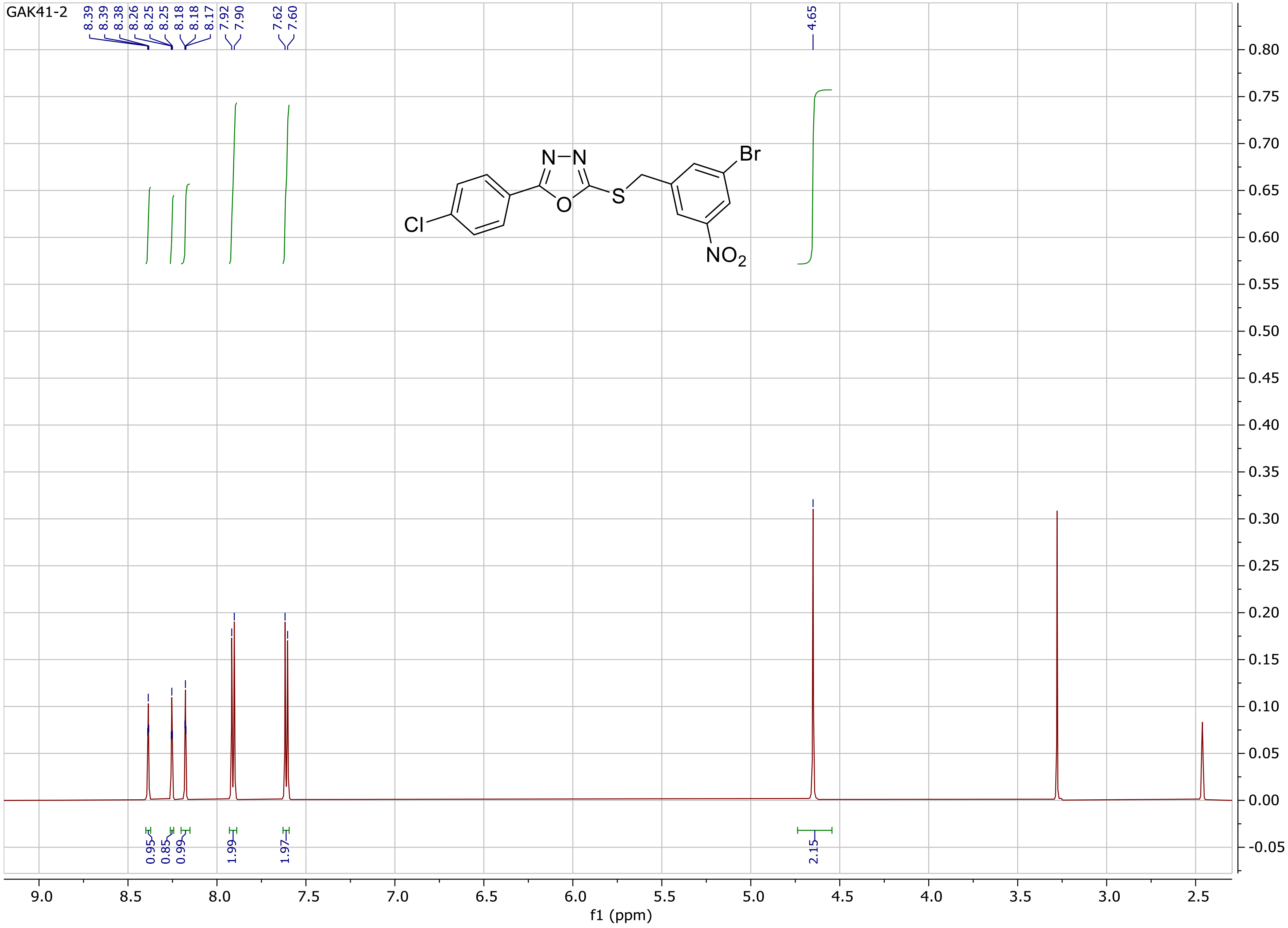

2-((3-Bromo-5-nitrobenzyl)sulfanyl)-5-(4-chlorophenyl)-1,3,4-oxadiazole (**60c**): ^{13}C NMR (126 MHz, DMSO-d_6)

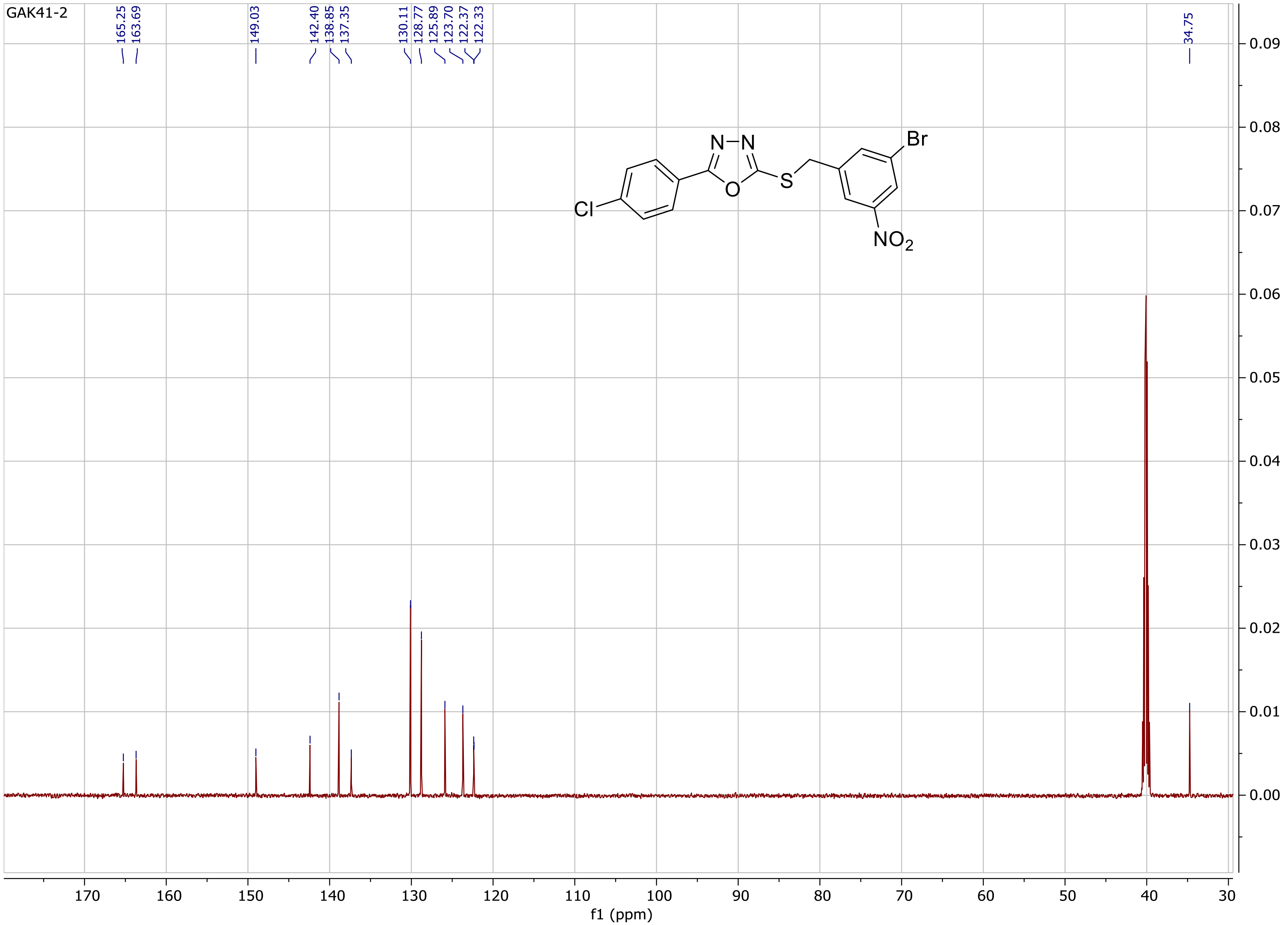

2-((3-Bromo-5-nitrobenzyl)sulfanyl)-5-(4-bromophenyl)-1,3,4-oxadiazole (**60d**): ^{1}H NMR (600 MHz, DMSO-d_6)

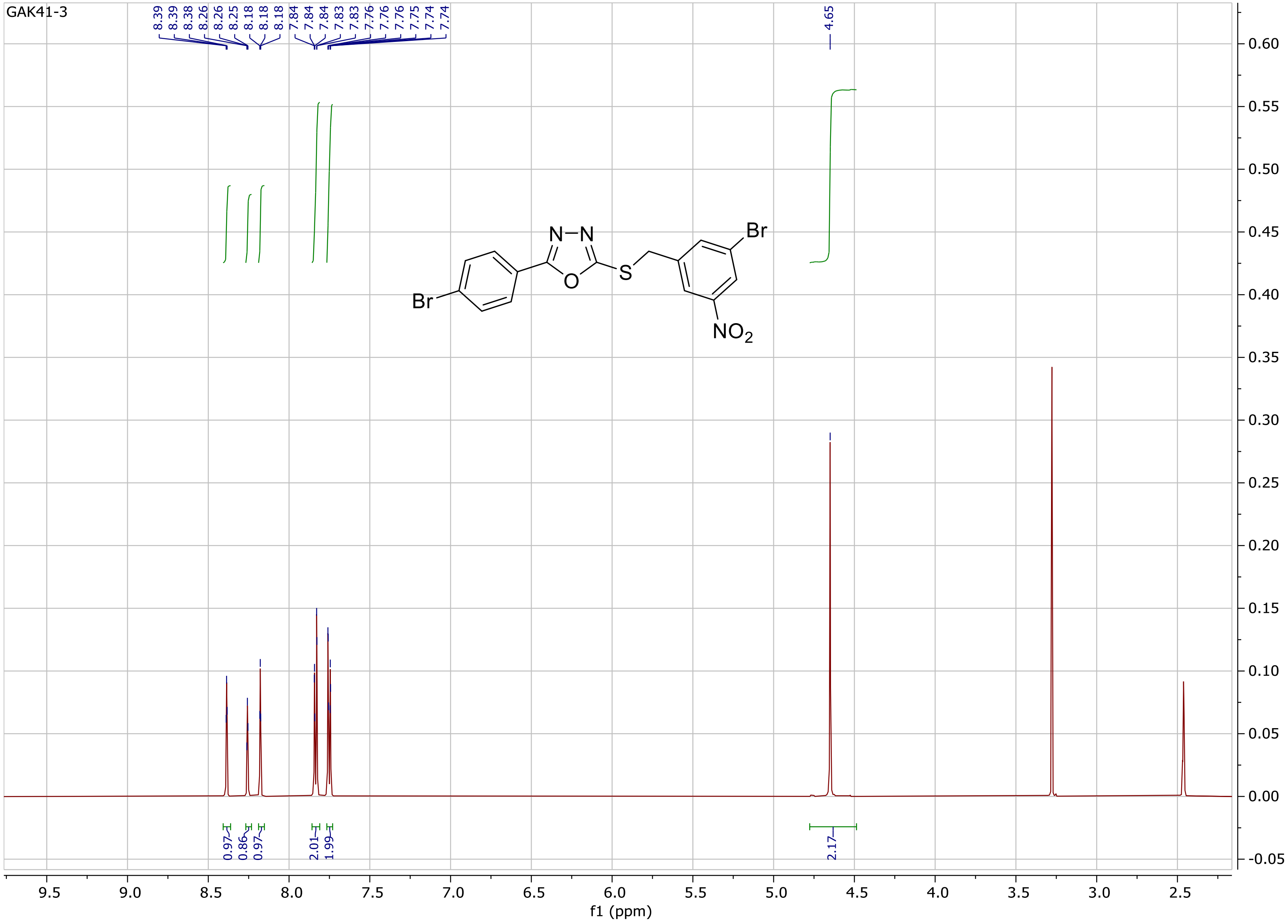

2-((3-Bromo-5-nitrobenzyl)sulfanyl)-5-(4-bromophenyl)-1,3,4-oxadiazole (**60d**): ^{13}C NMR (151 MHz, DMSO-d_6)

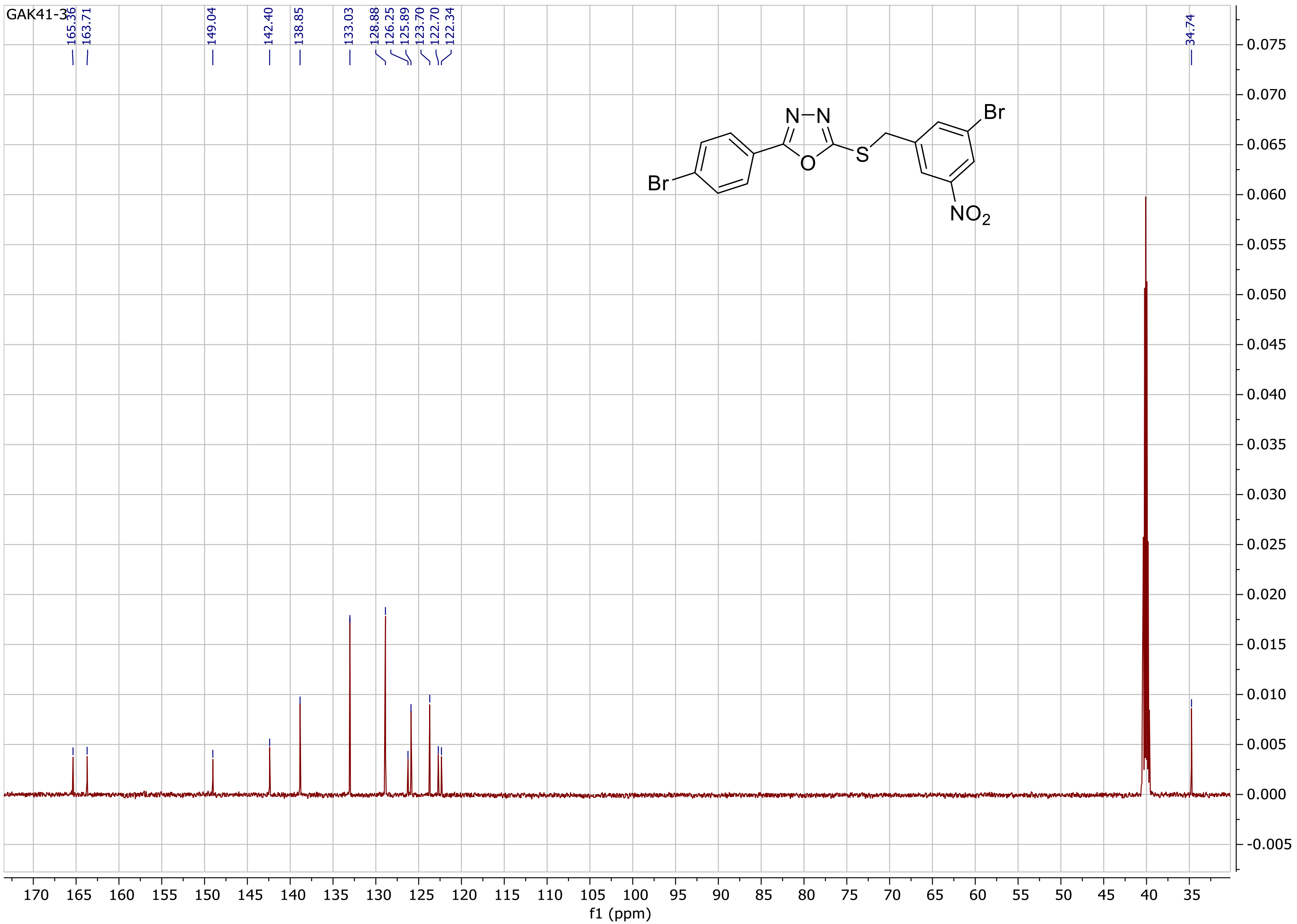

2-((3-Bromo-5-nitrobenzyl)sulfanyl)-5-cyclohexyl-1,3,4-oxadiazole (**60e**): ^{1}H NMR (600 MHz, DMSO-d_6)

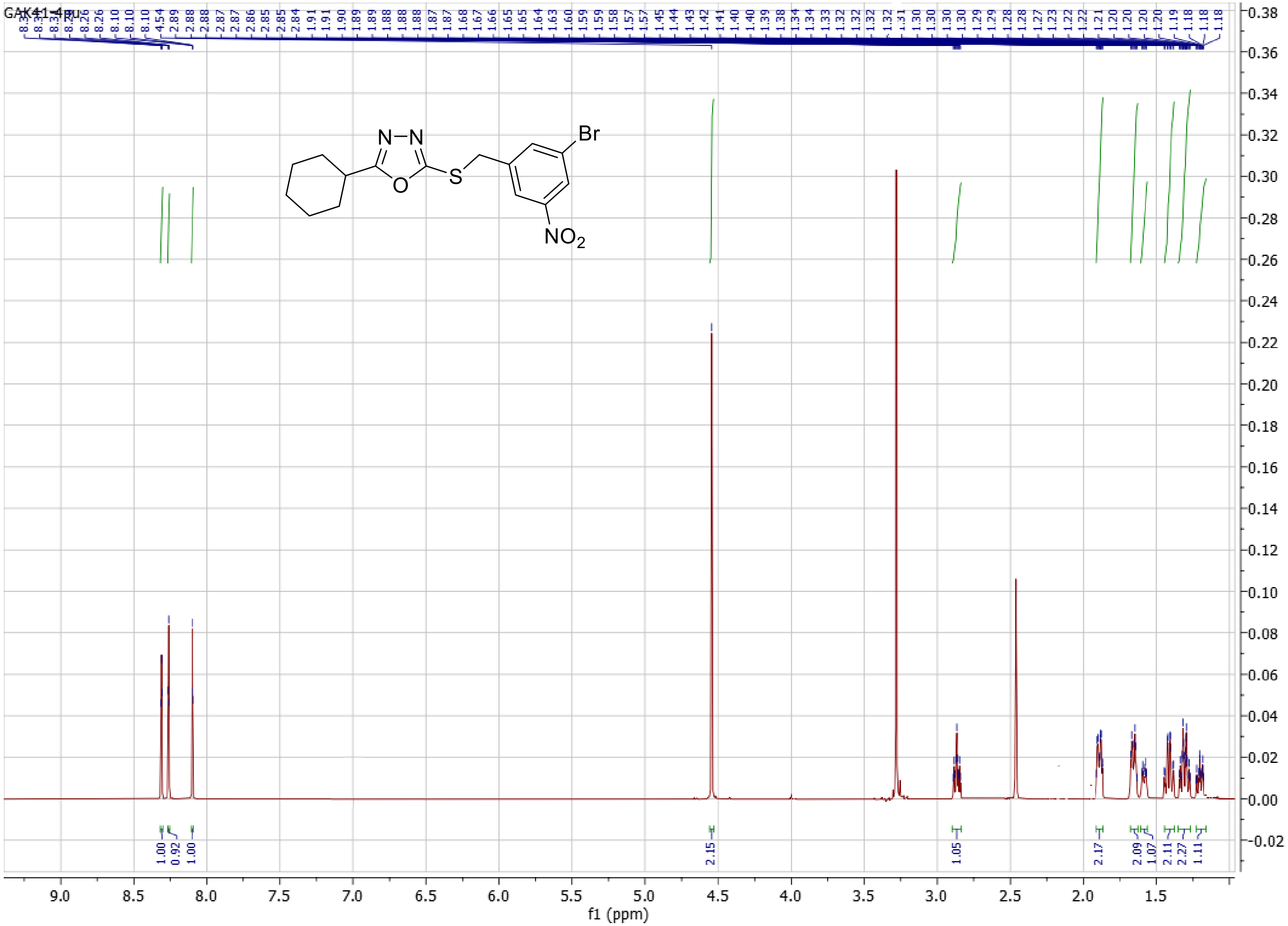

2-((3-Bromo-5-nitrobenzyl)sulfanyl)-5-cyclohexyl-1,3,4-oxadiazole (**60e**): ^{13}C NMR (151 MHz, DMSO-d_6)

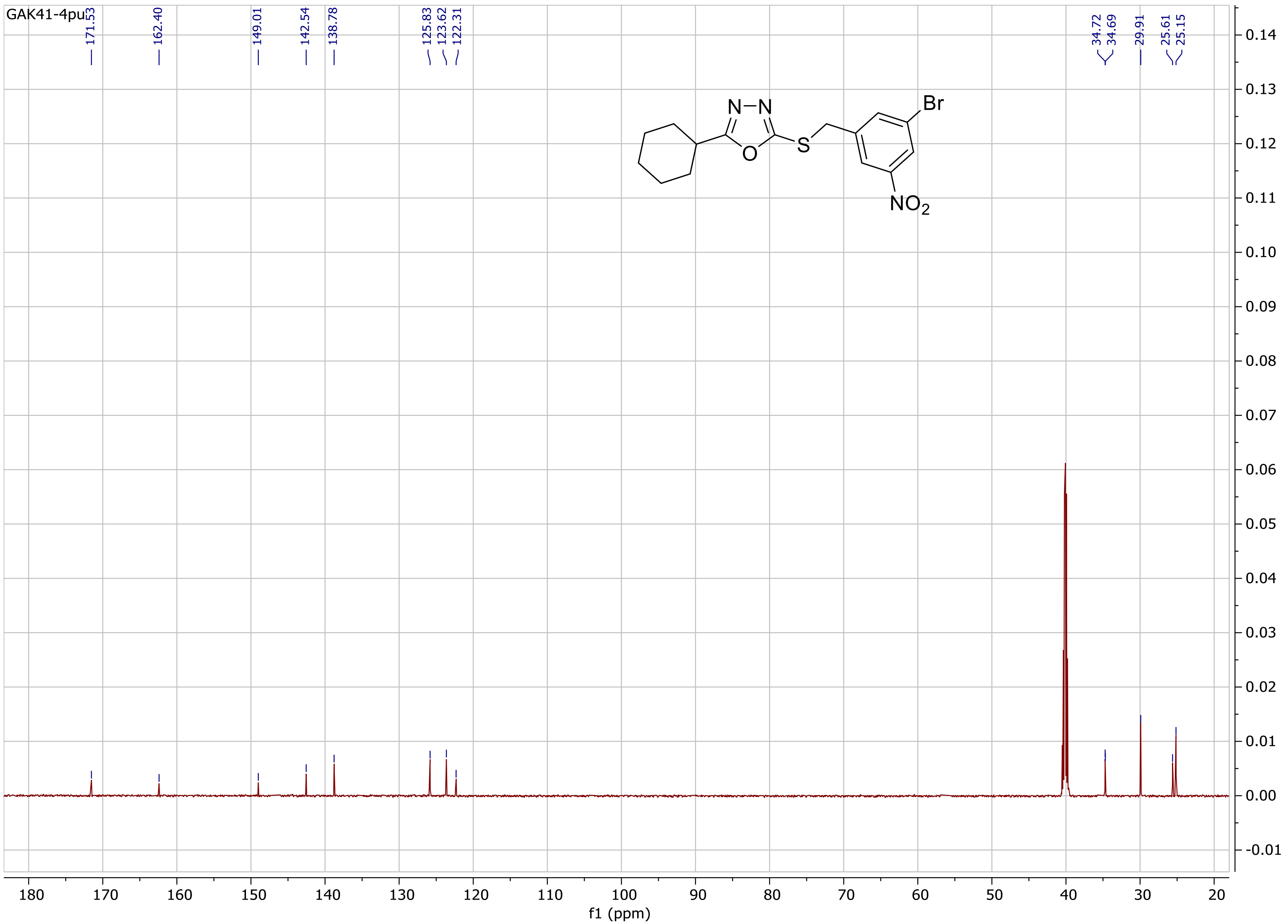

2-((3-Cyano-5-nitrobenzyl)sulfanyl)-5-phenyl-1,3,4-oxadiazole (**61a**): ^{1}H NMR (600 MHz, DMSO-d_6)

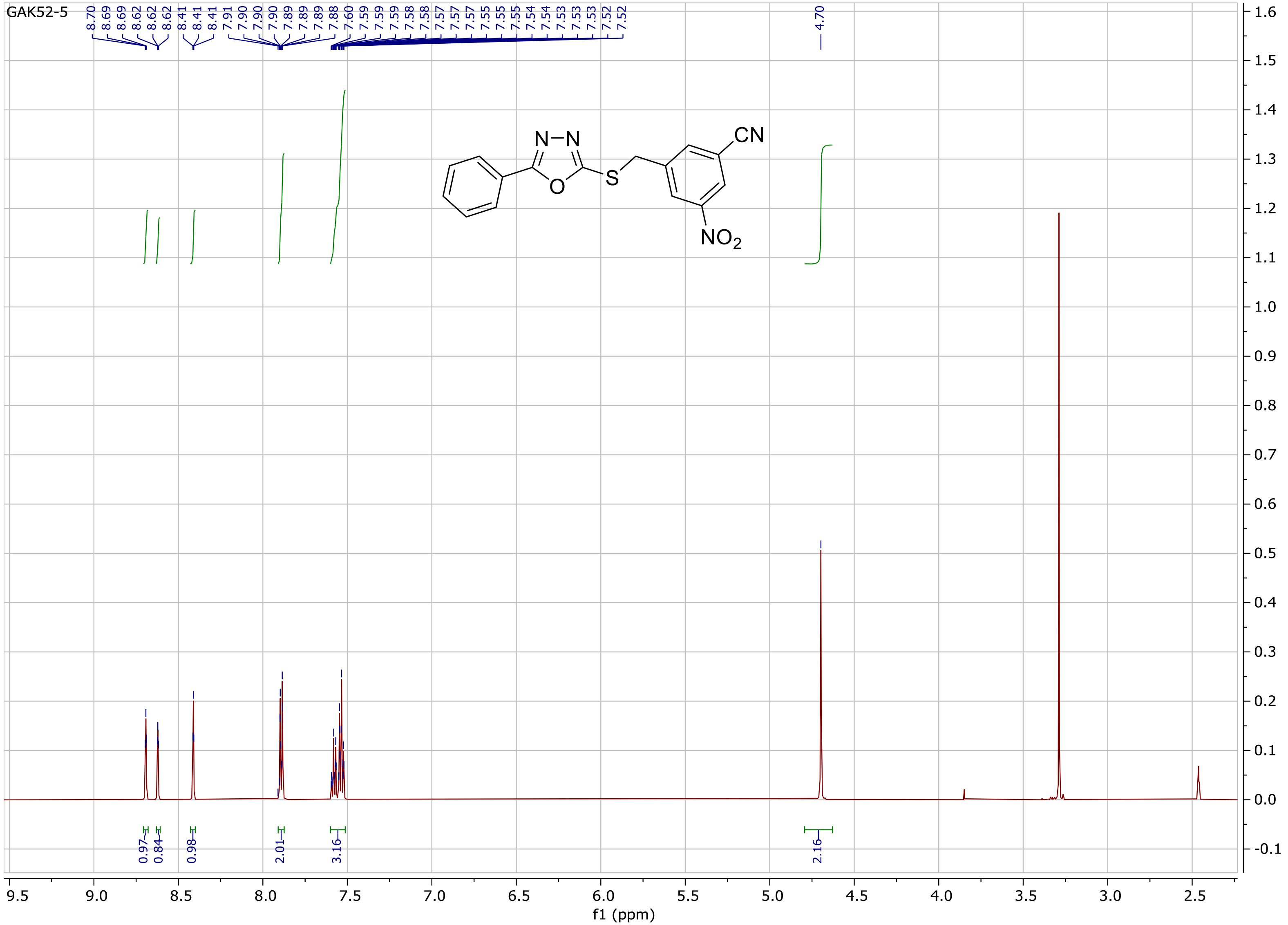

2-((3-Cyano-5-nitrobenzyl)sulfanyl)-5-phenyl-1,3,4-oxadiazole (**61a**): ^{13}C NMR (151 MHz, DMSO-d_6)

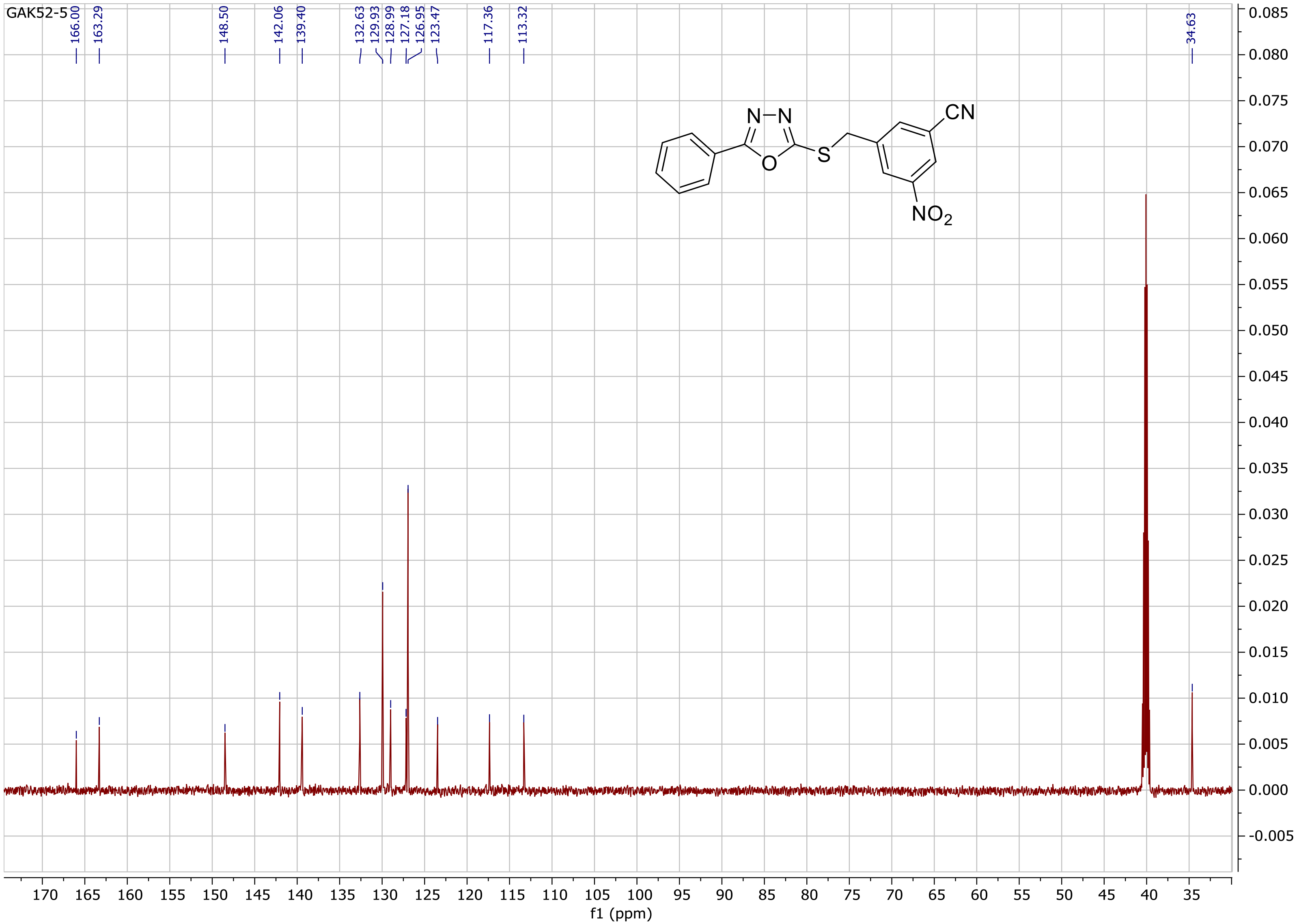

2-((3-Cyano-5-nitrobenzyl)sulfanyl)-5-(4-methoxyphenyl)-1,3,4-oxadiazole (**61b**): ^{1}H NMR (600 MHz, DMSO-d_6)

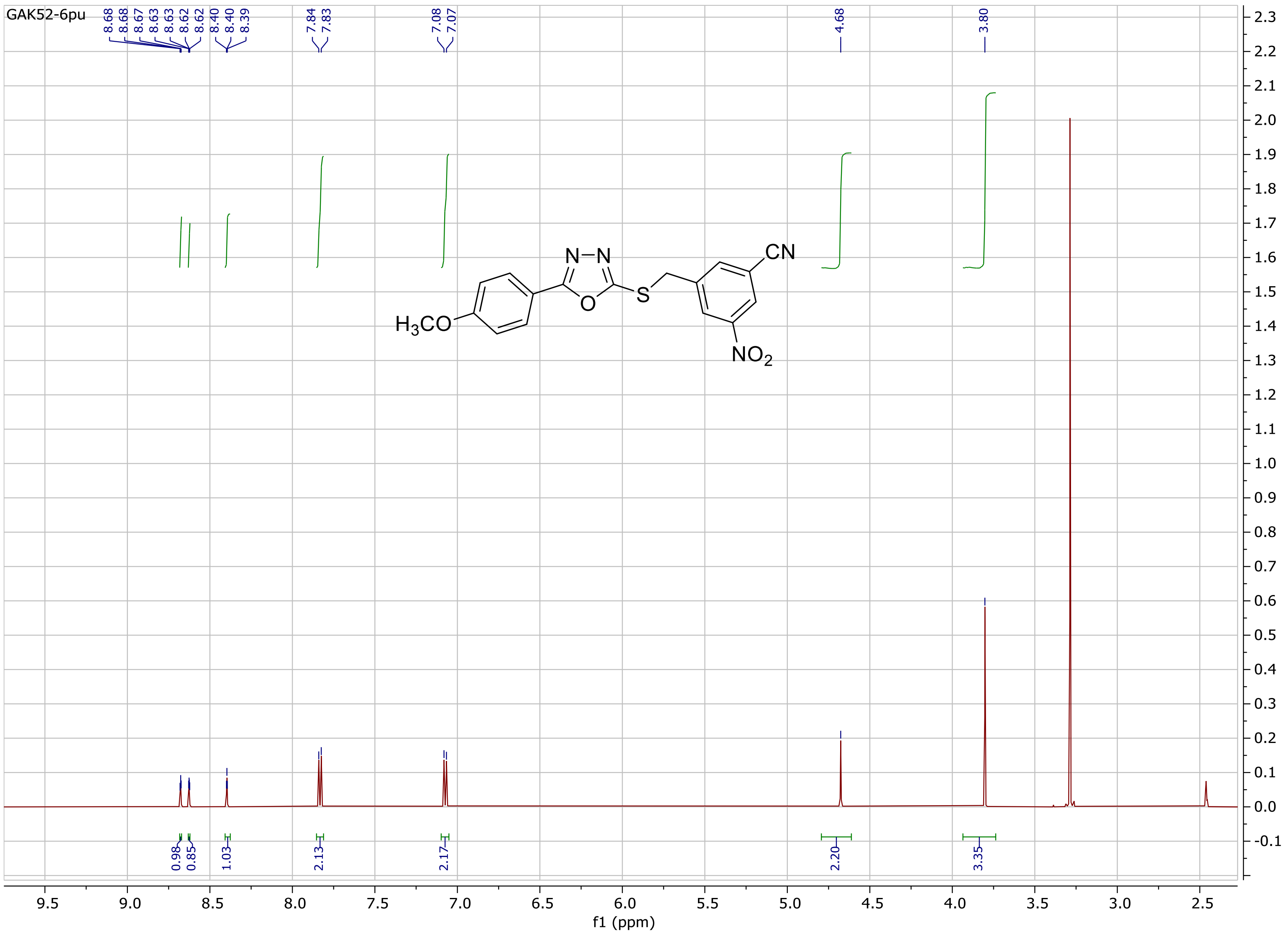

2-((3-Cyano-5-nitrobenzyl)sulfanyl)-5-(4-methoxyphenyl)-1,3,4-oxadiazole (**61b**): ^{13}C NMR (151 MHz, DMSO-d_6)

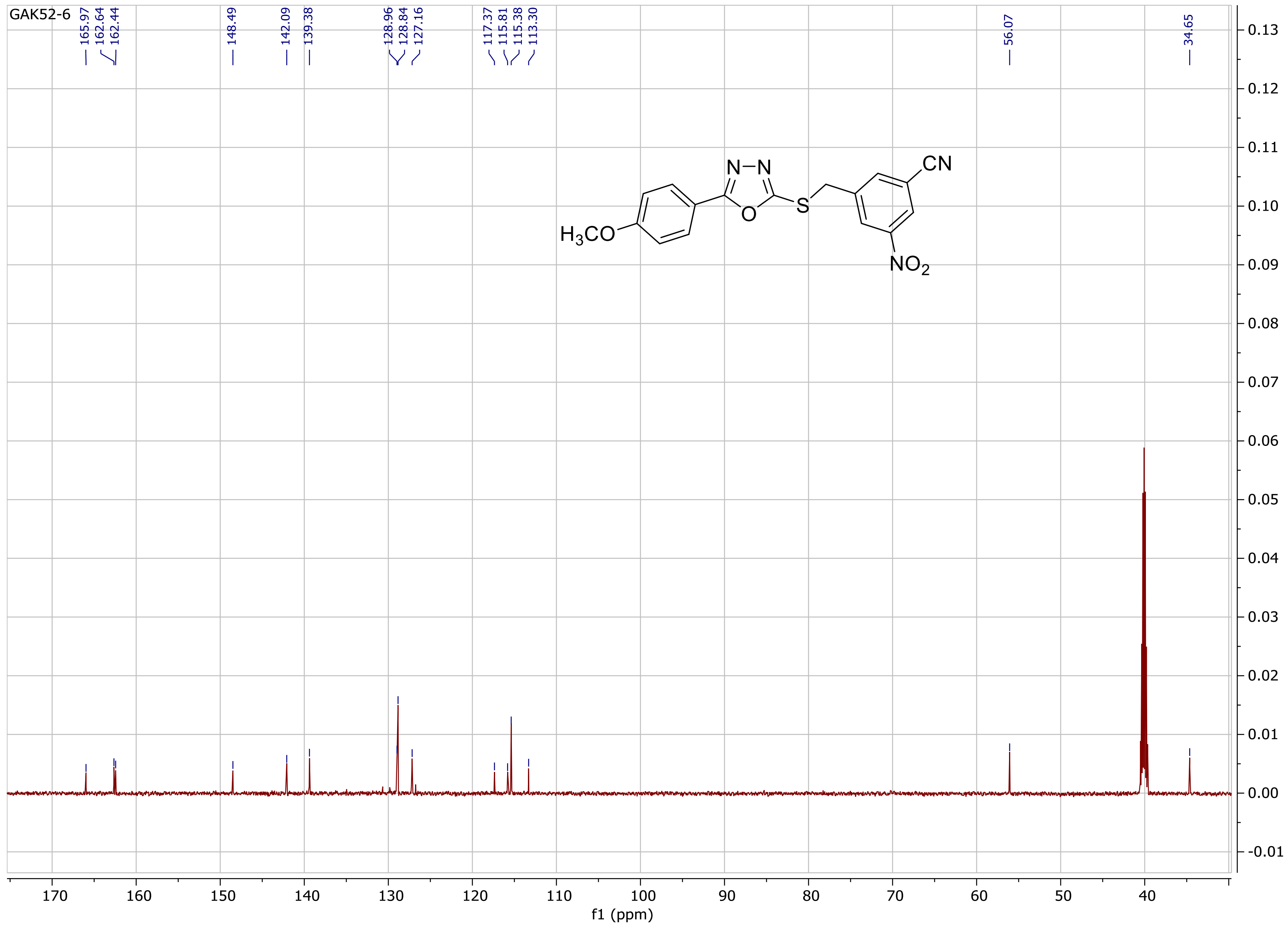

5-(4-Chlorophenyl)-2-((3-cyano-5-nitrobenzyl)sulfanyl)-1,3,4-oxadiazole (**61c**): ^{1}H NMR (500 MHz, DMSO-d_6)

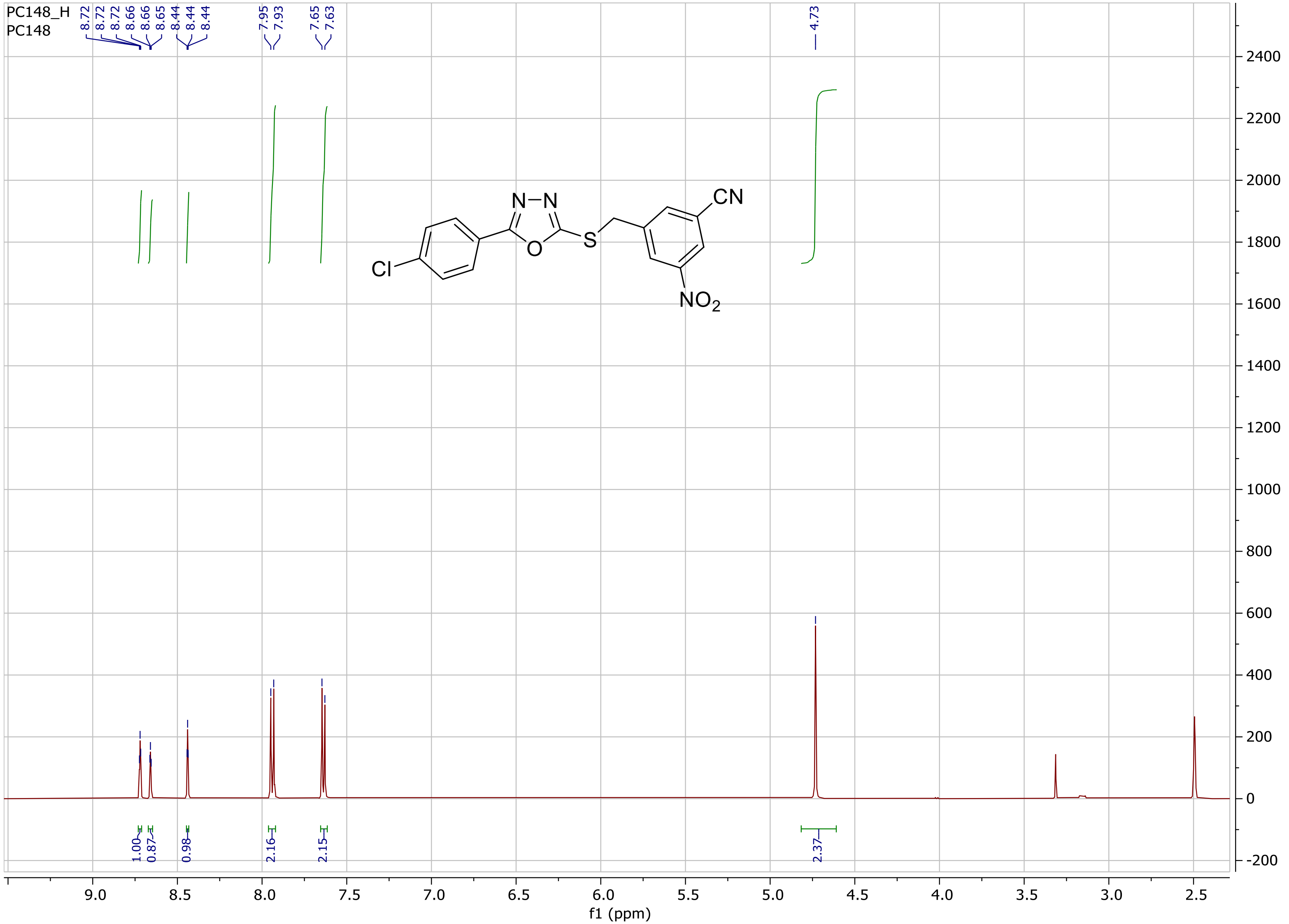

5-(4-Chlorophenyl)-2-((3-cyano-5-nitrobenzyl)sulfanyl)-1,3,4-oxadiazole (**61c**): ^{13}C NMR (126 MHz, DMSO-d_6)

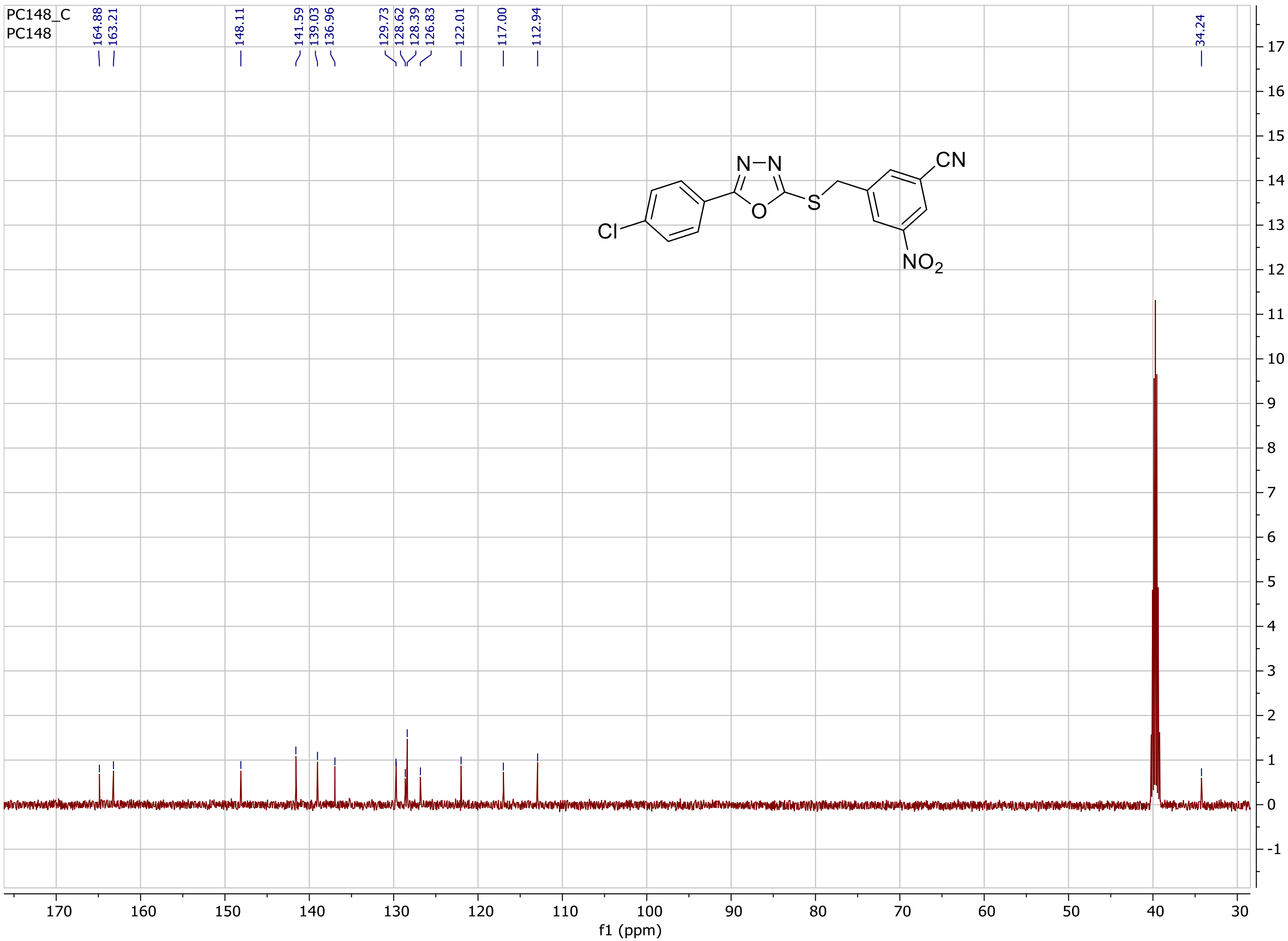

2-(4-Bromophenyl)-5-((3-cyano-5-nitrobenzyl)sulfanyl)-1,3,4-oxadiazole (**61d**): ^{1}H NMR (500 MHz, $CDCl_3$)

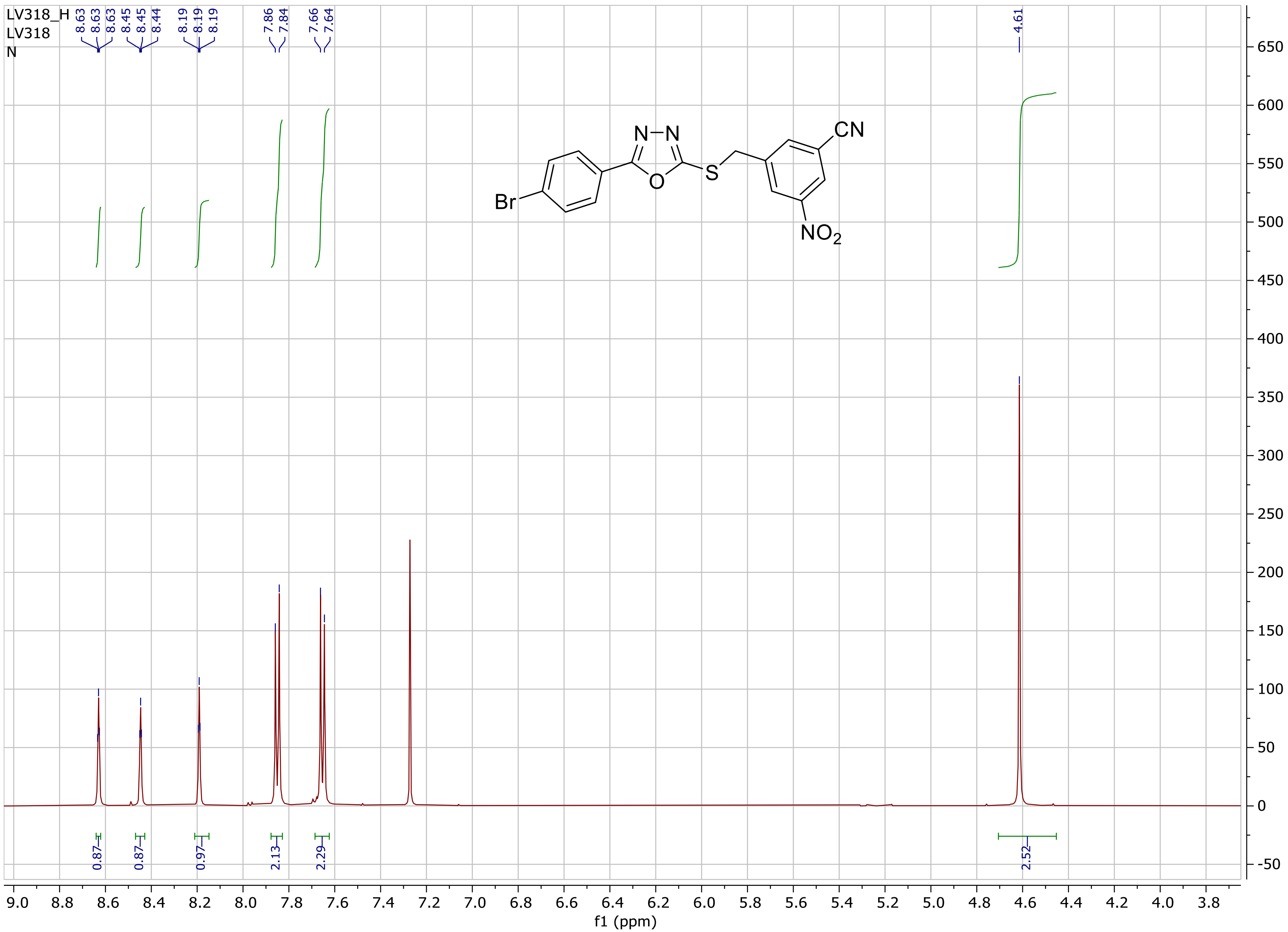

2-(4-Bromophenyl)-5-((3-cyano-5-nitrobenzyl)sulfanyl)-1,3,4-oxadiazole (**61d**): ^{13}C NMR (126 MHz, $CDCl_3$)

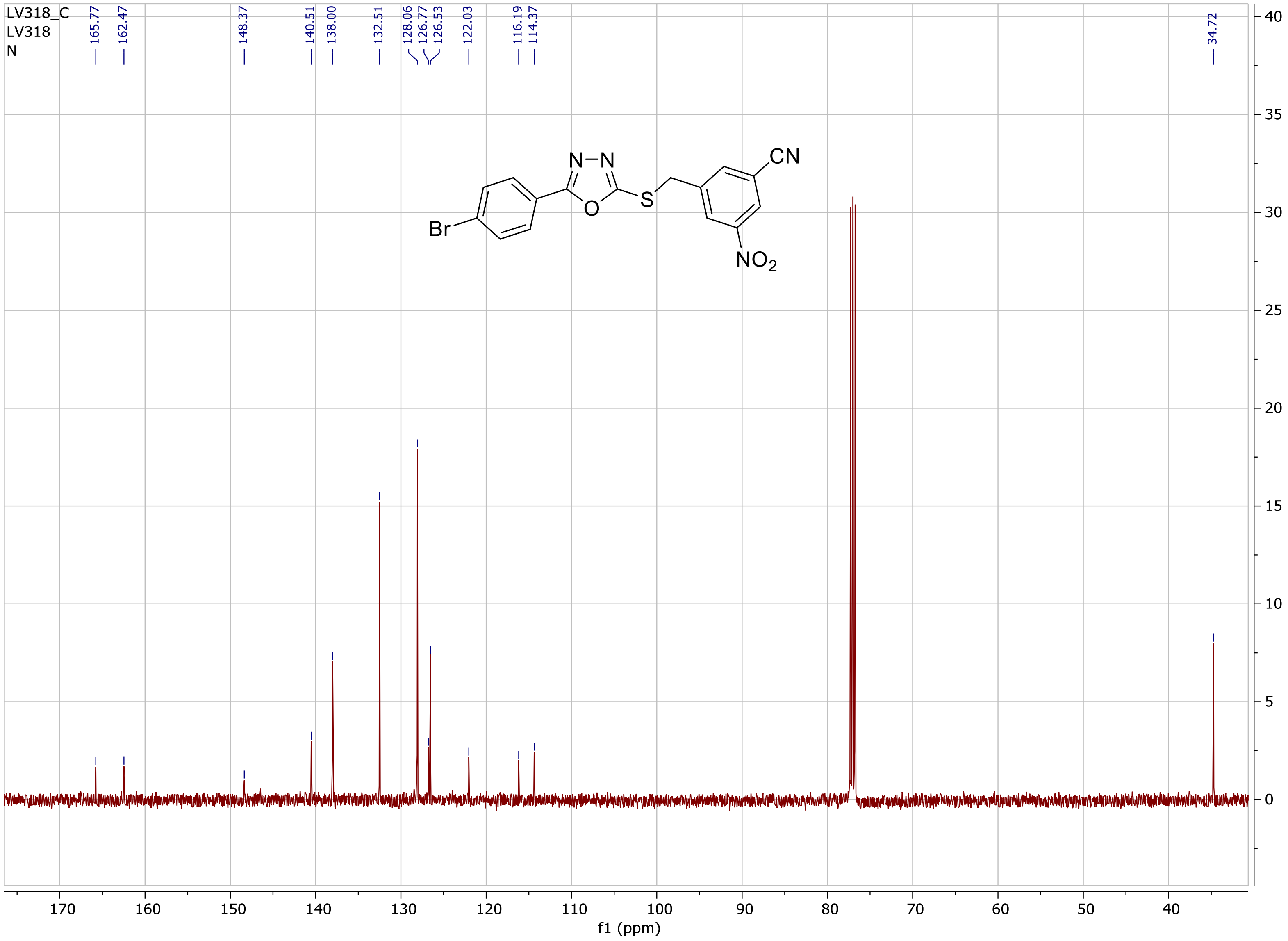

2-((3-Cyano-5-nitrobenzyl)sulfanyl)-5-cyclohexyl-1,3,4-oxadiazole (**61e**): ^{1}H NMR (600 MHz, DMSO-*d*$_6$)

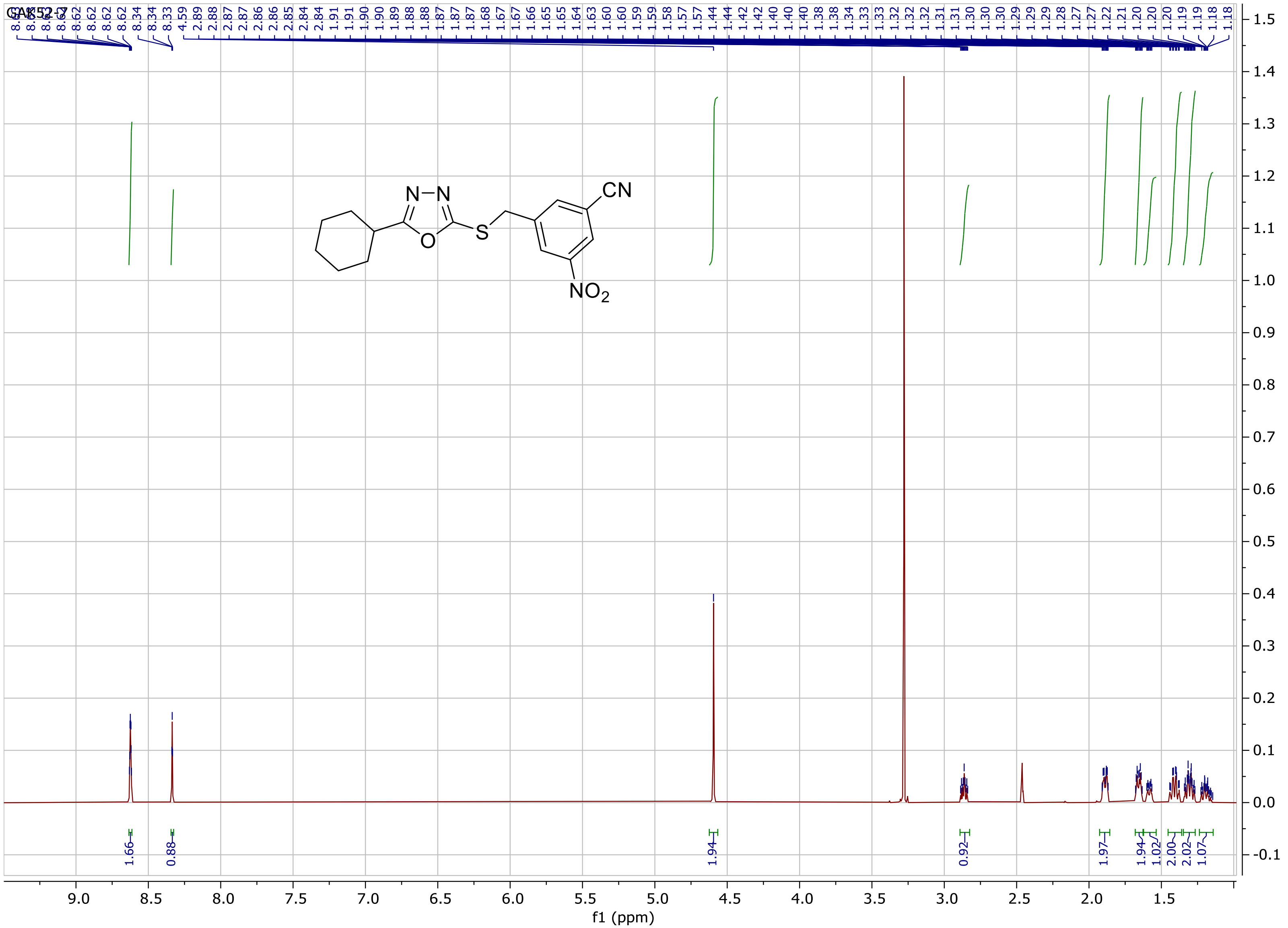

2-((3-Cyano-5-nitrobenzyl)sulfanyl)-5-cyclohexyl-1,3,4-oxadiazole (**61e**): ^{13}C NMR (151 MHz, DMSO-d_6)

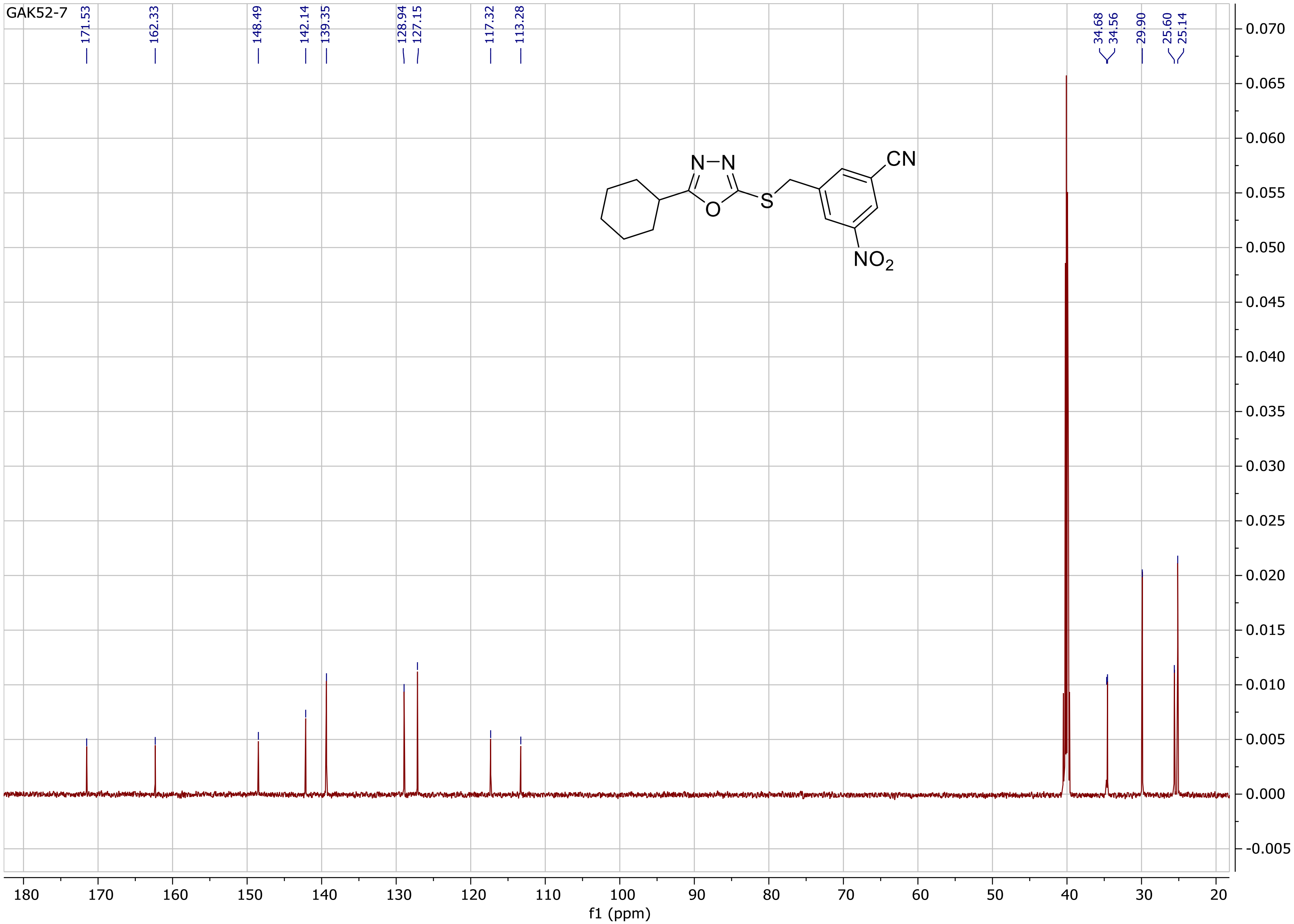

2-((3-(Methoxycarbonyl)-5-nitrobenzyl)sulfanyl)-5-phenyl-1,3,4-oxadiazole (**62a**): ^{1}H NMR (500 MHz, DMSO-d_6)

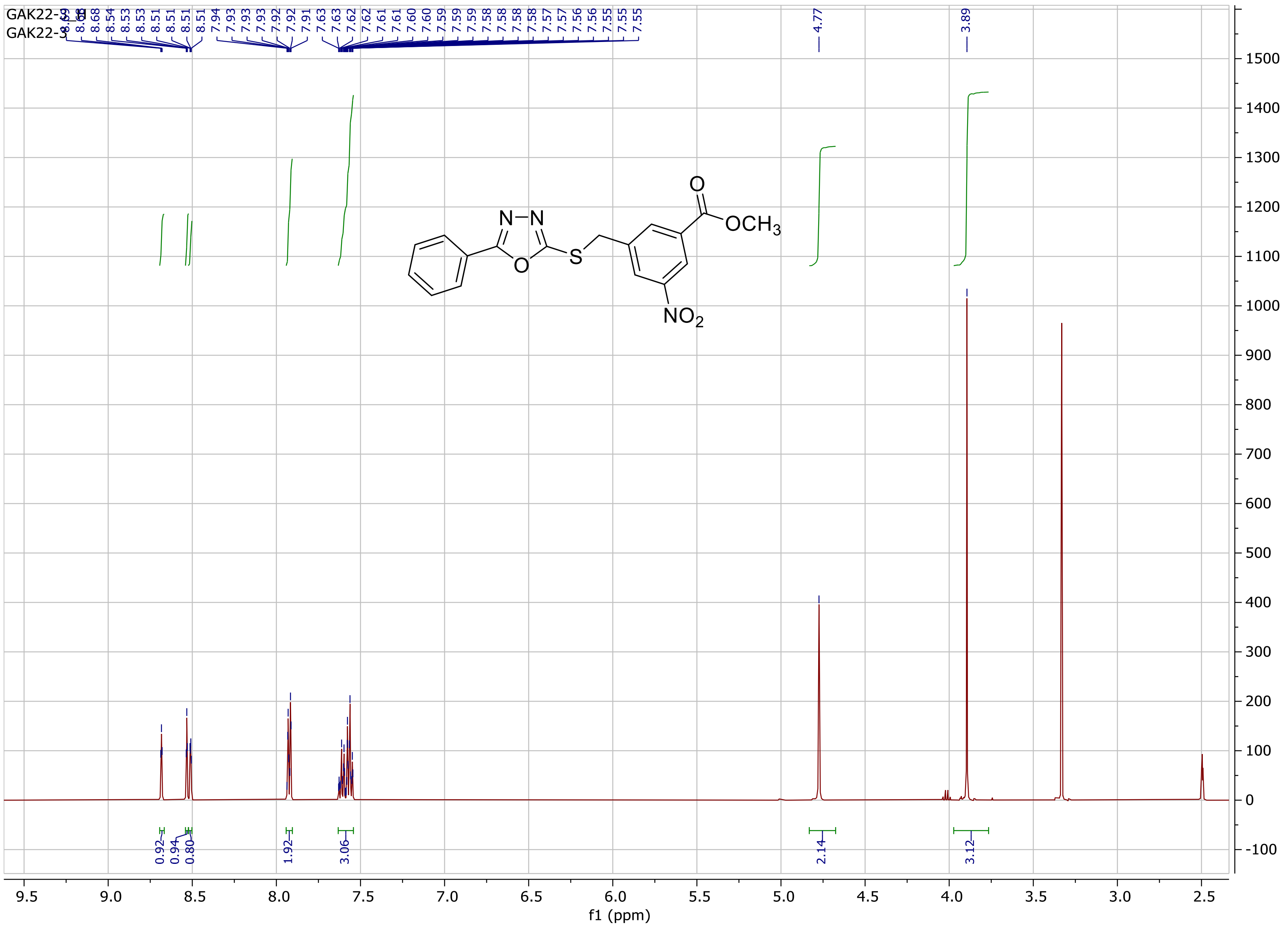

2-((3-(Methoxycarbonyl)-5-nitrobenzyl)sulfanyl)-5-phenyl-1,3,4-oxadiazole (**62a**): ^{13}C NMR (126 MHz, DMSO-d_6)

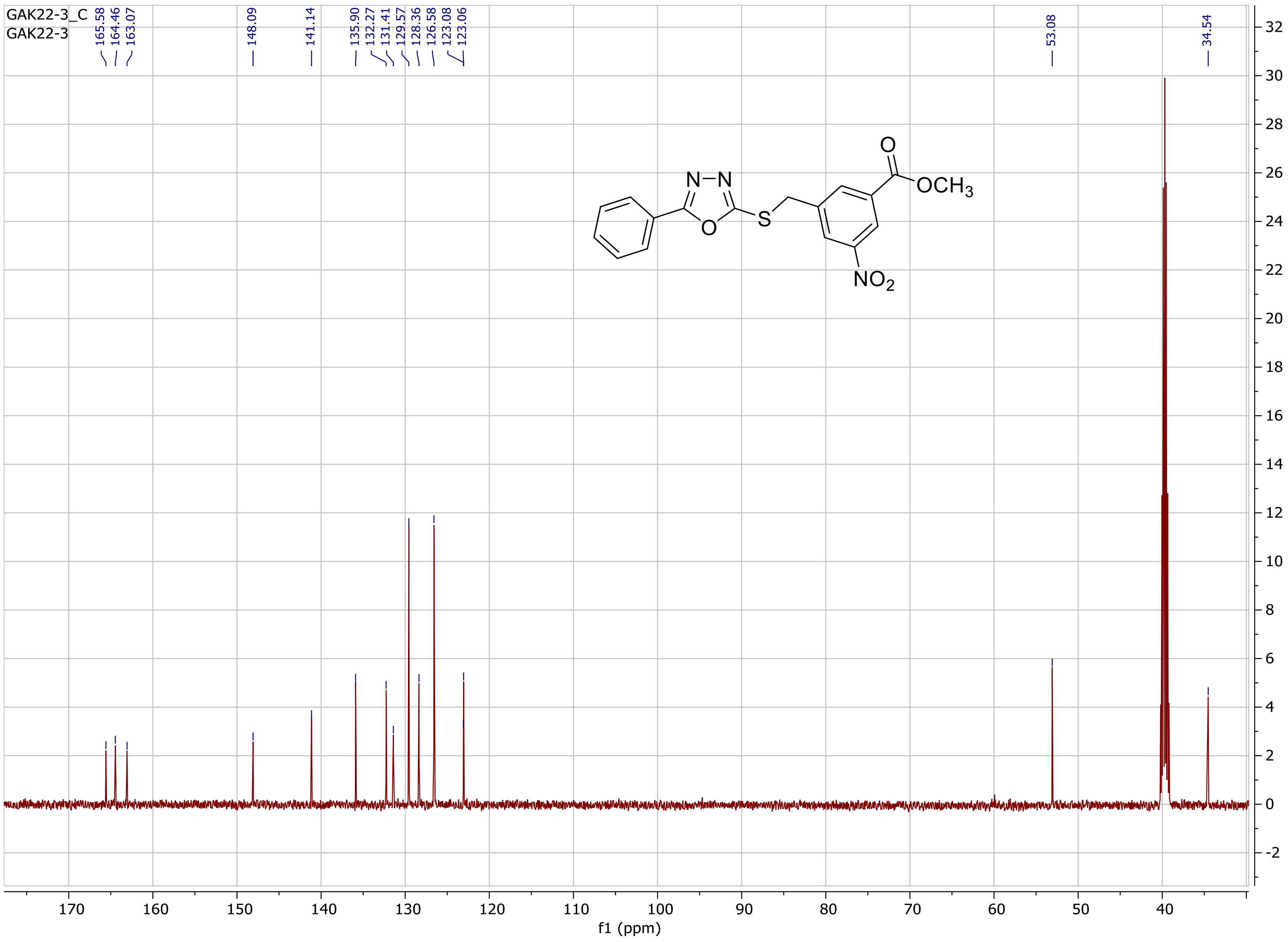

2-((3-(Methoxycarbonyl)-5-nitrobenzyl)sulfanyl)-5-(4-methoxyphenyl)-1,3,4-oxadiazole (**62b**): ^{1}H NMR (500 MHz, DMSO-d_6)

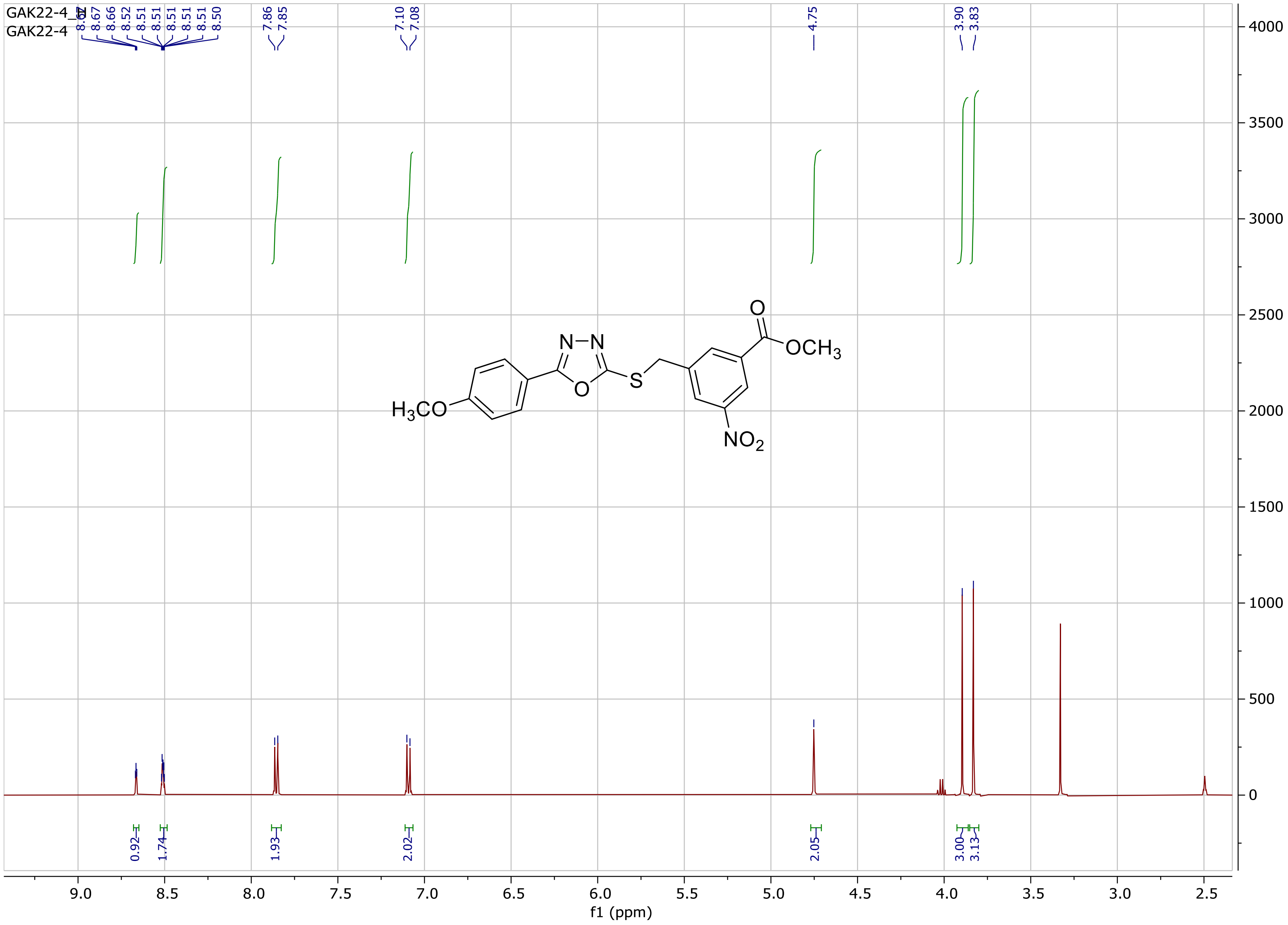

2-((3-(Methoxycarbonyl)-5-nitrobenzyl)sulfanyl)-5-(4-methoxyphenyl)-1,3,4-oxadiazole (**62b**): ^{13}C NMR (126 MHz, DMSO-d_6)

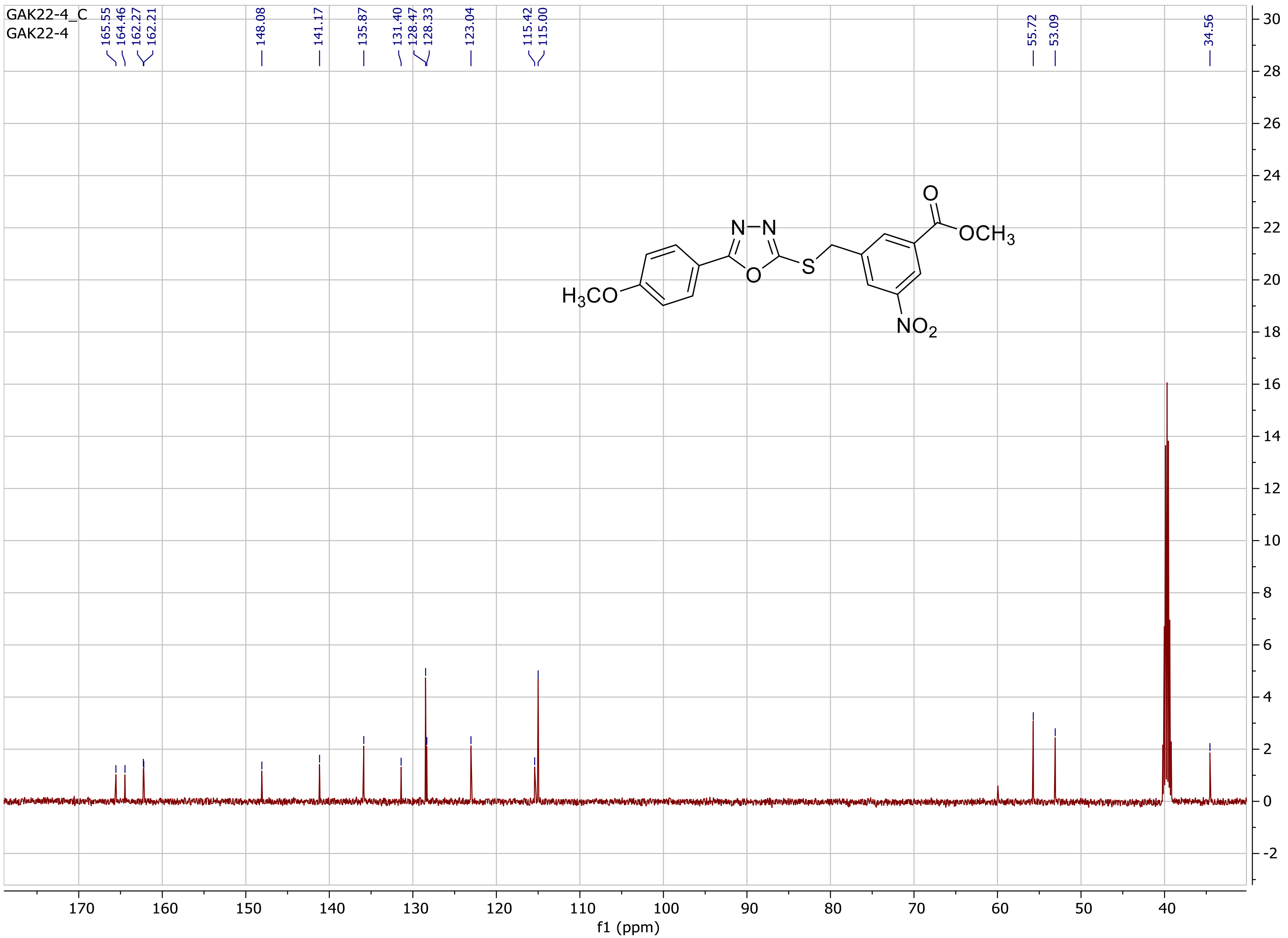

2-(4-Chlorophenyl)-5-((3-(methoxycarbonyl)-5-nitrobenzyl)sulfanyl)-1,3,4-oxadiazole (**62c**): ^{1}H NMR (500 MHz, DMSO-d_6)

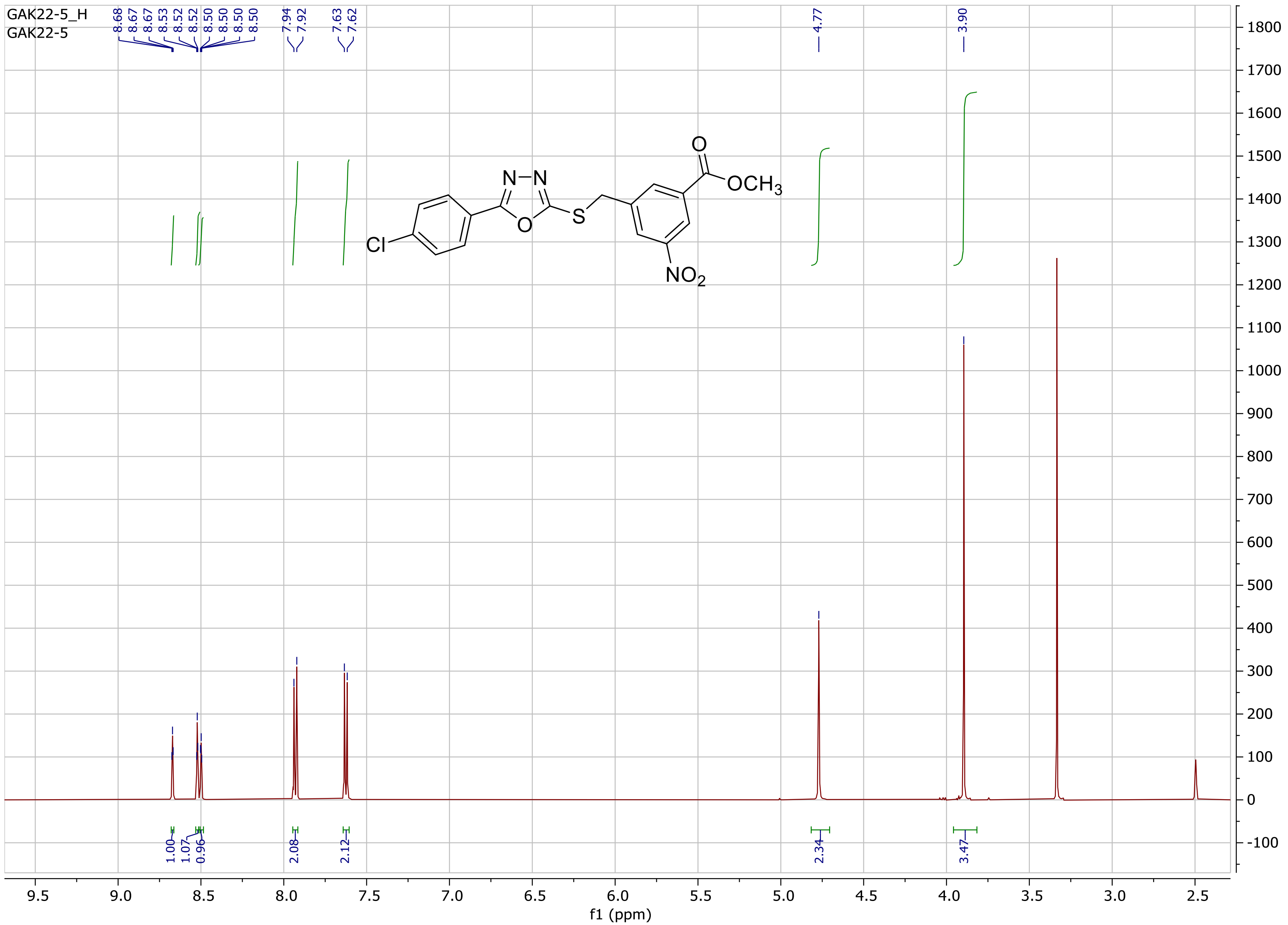

2-(4-Chlorophenyl)-5-((3-(methoxycarbonyl)-5-nitrobenzyl)sulfanyl)-1,3,4-oxadiazole (**62c**): ^{13}C NMR (126 MHz, DMSO-d_6)

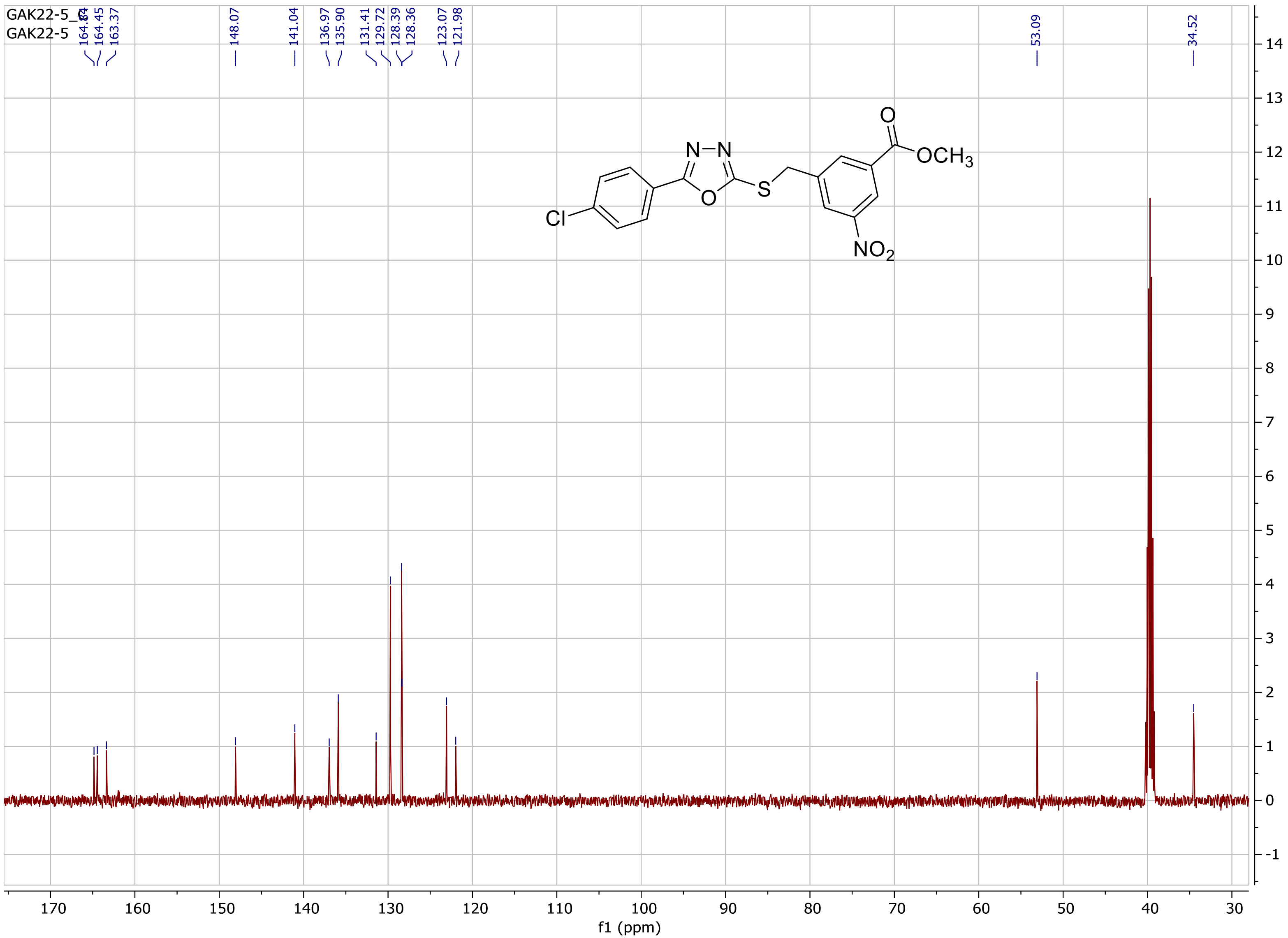

2-(4-Bromophenyl)-5-((3-(methoxycarbonyl)-5-nitrobenzyl)sulfanyl)-1,3,4-oxadiazole (**62d**): ^{1}H NMR (500 MHz, DMSO-d_6)

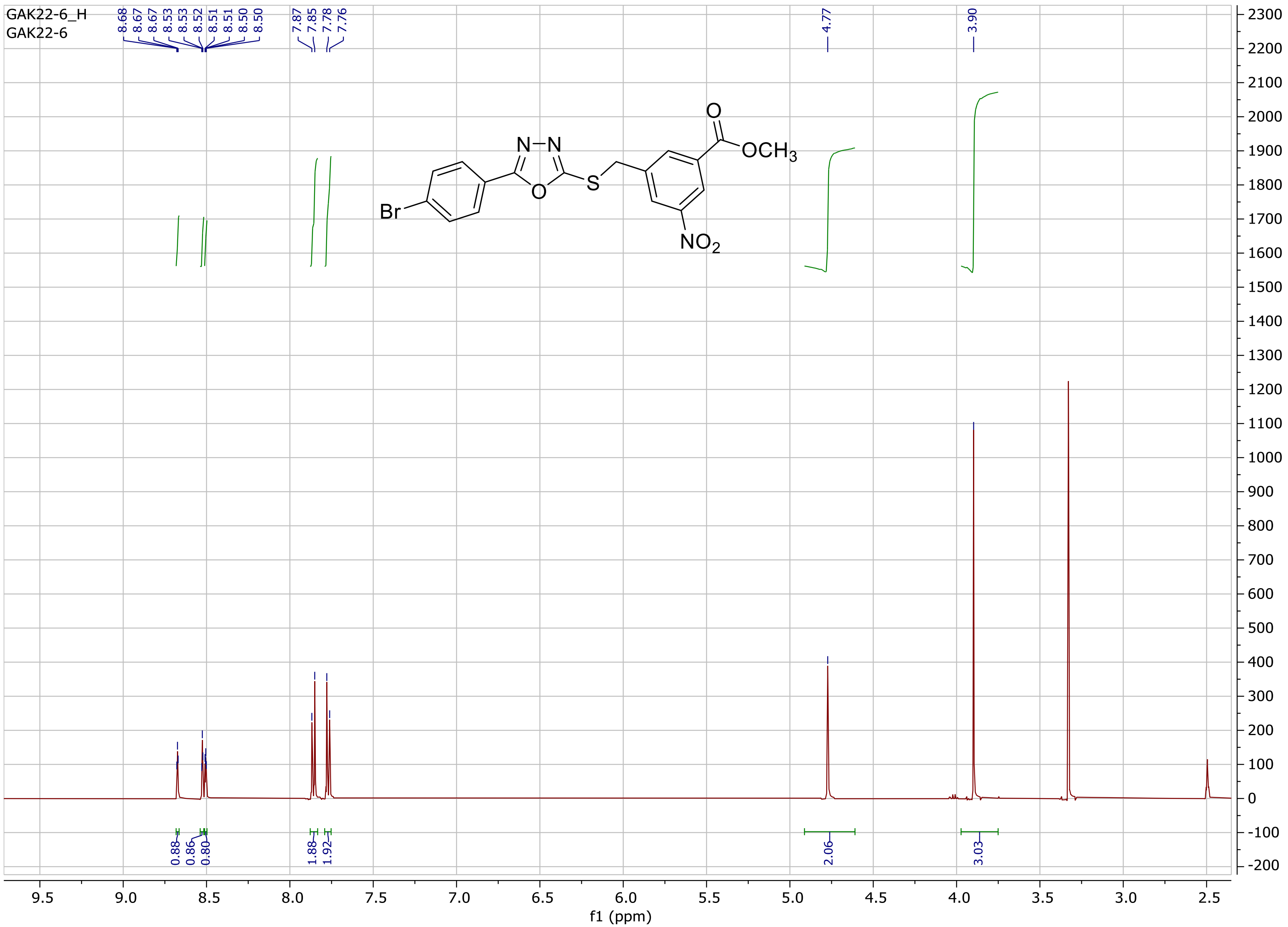

2-(4-Bromophenyl)-5-((3-(methoxycarbonyl)-5-nitrobenzyl)sulfanyl)-1,3,4-oxadiazole (**62d**): ^{13}C NMR (126 MHz, DMSO-d_6)

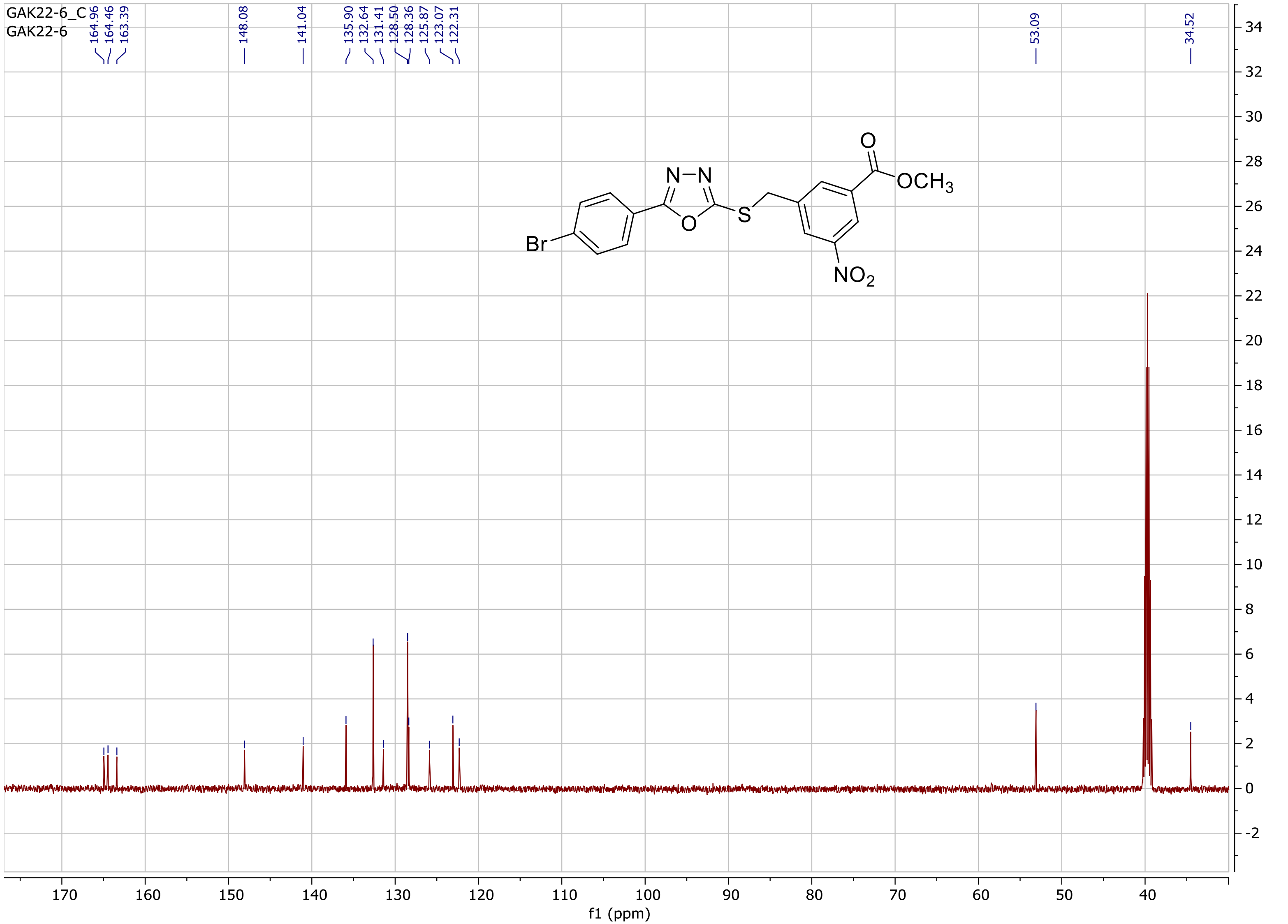

2-Cyclohexyl-5-((3-(methoxycarbonyl)-5-nitrobenzyl)sulfanyl)-1,3,4-oxadiazole (**62e**): ^{1}H NMR (500 MHz, DMSO-d_6)

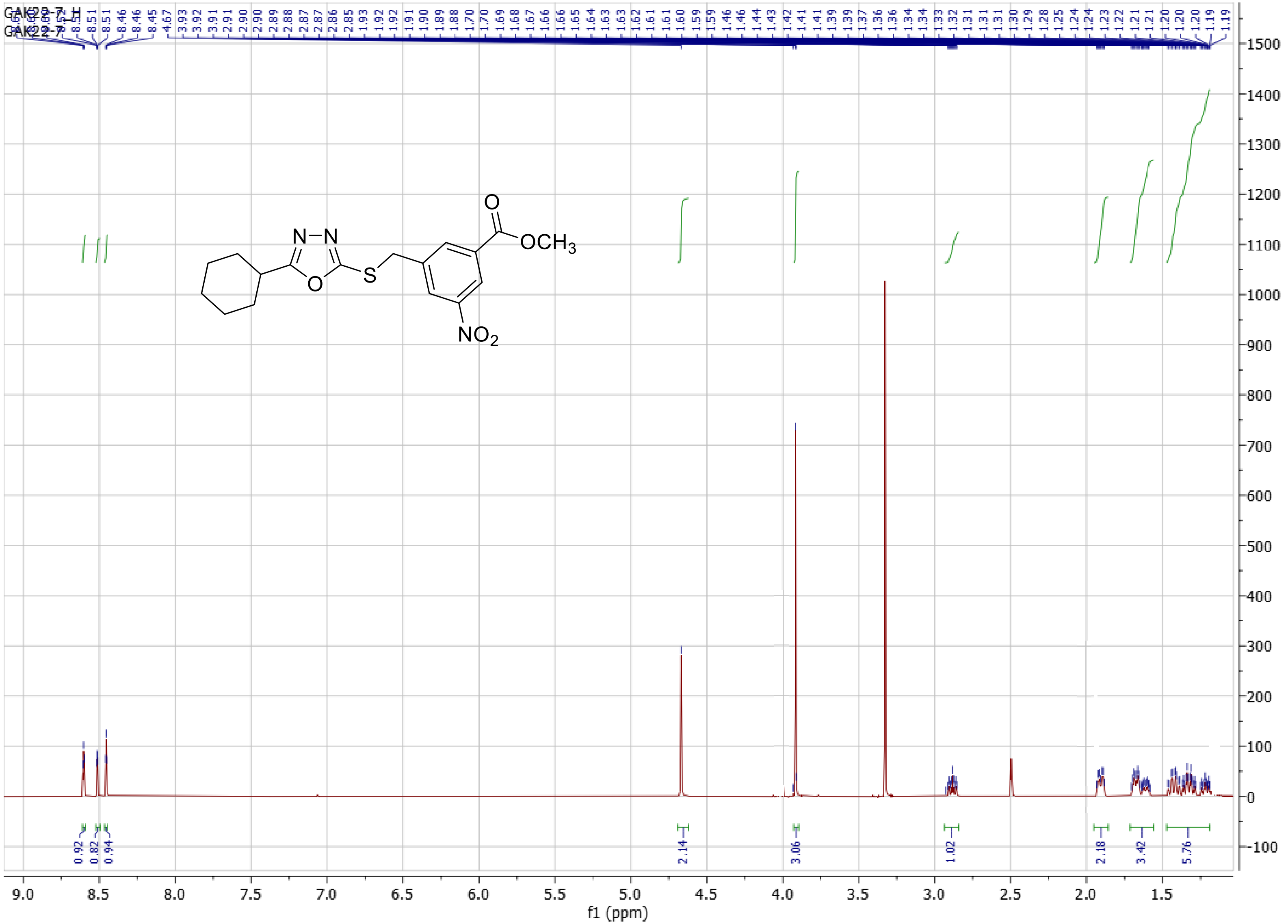

2-Cyclohexyl-5-((3-(methoxycarbonyl)-5-nitrobenzyl)sulfanyl)-1,3,4-oxadiazole (**62e**): ^{13}C NMR (126 MHz, DMSO-d_6)

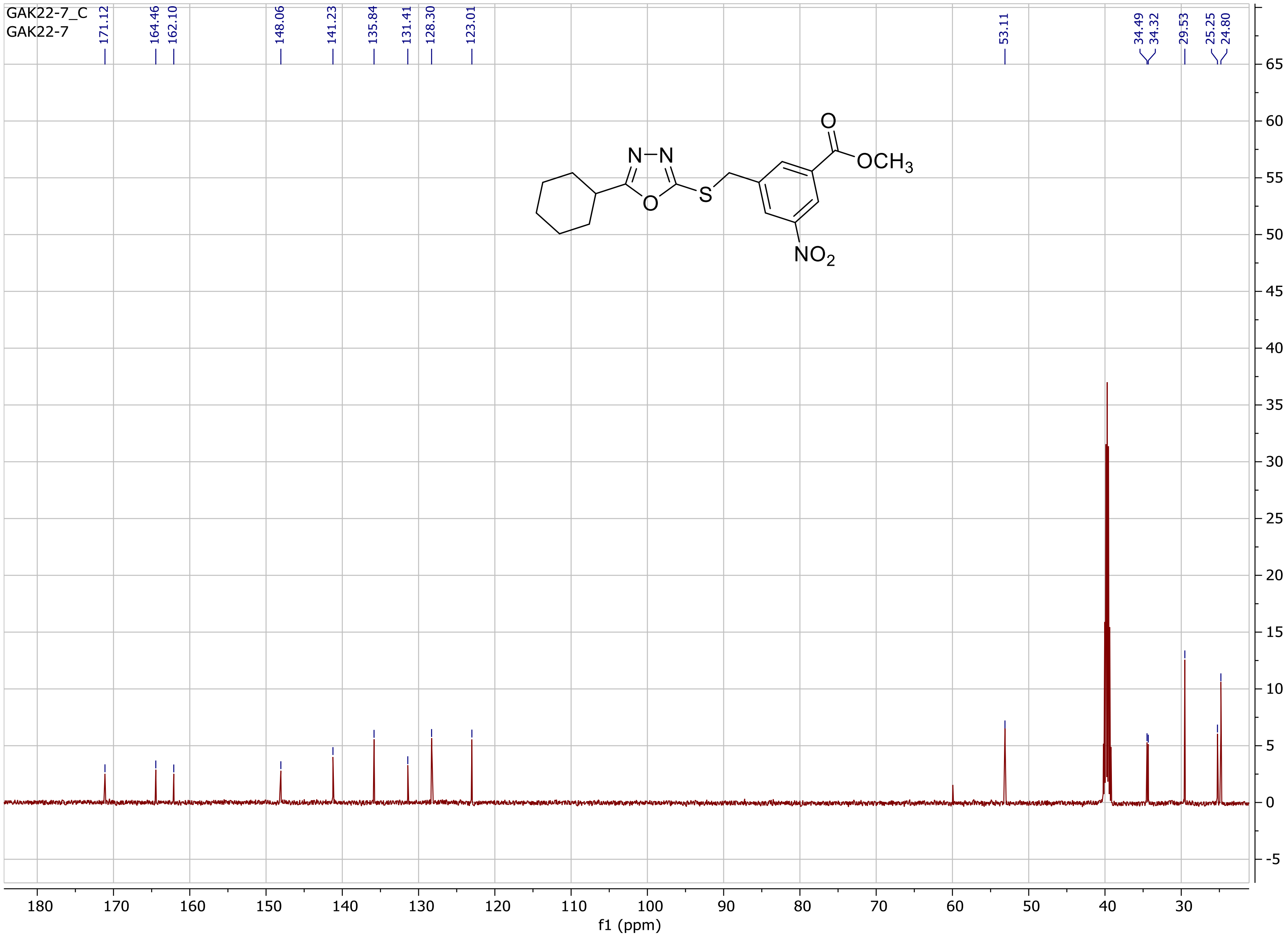

2-Cyclohexyl-5-((3-(methoxycarbonyl)-5-nitrobenzyl)sulfanyl)-1,3,4-oxadiazole (**62e**):

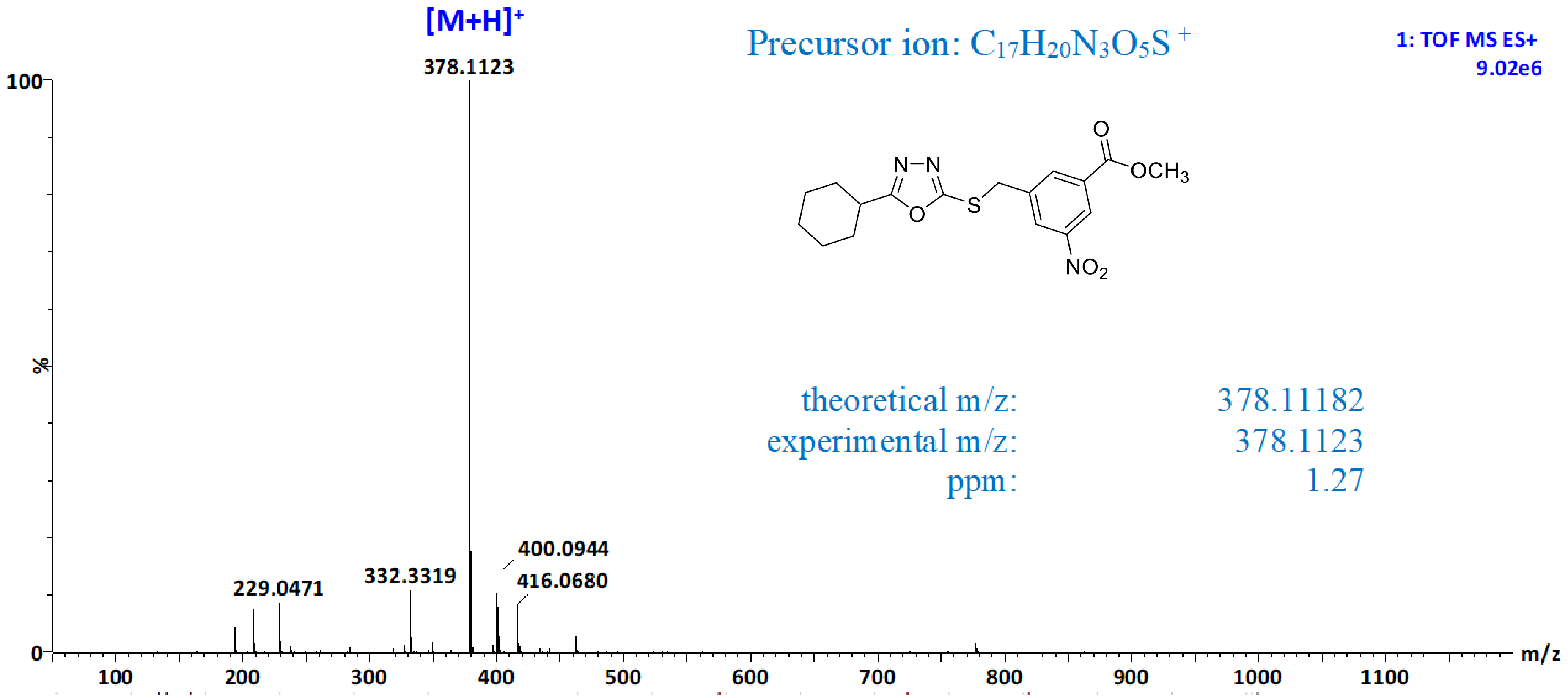

2-((3-(Carbamoyl)-5-nitrobenzyl)sulfanyl)-5-phenyl-1,3,4-oxadiazole (**63a**): ^{1}H NMR (600 MHz, DMSO-d_6)

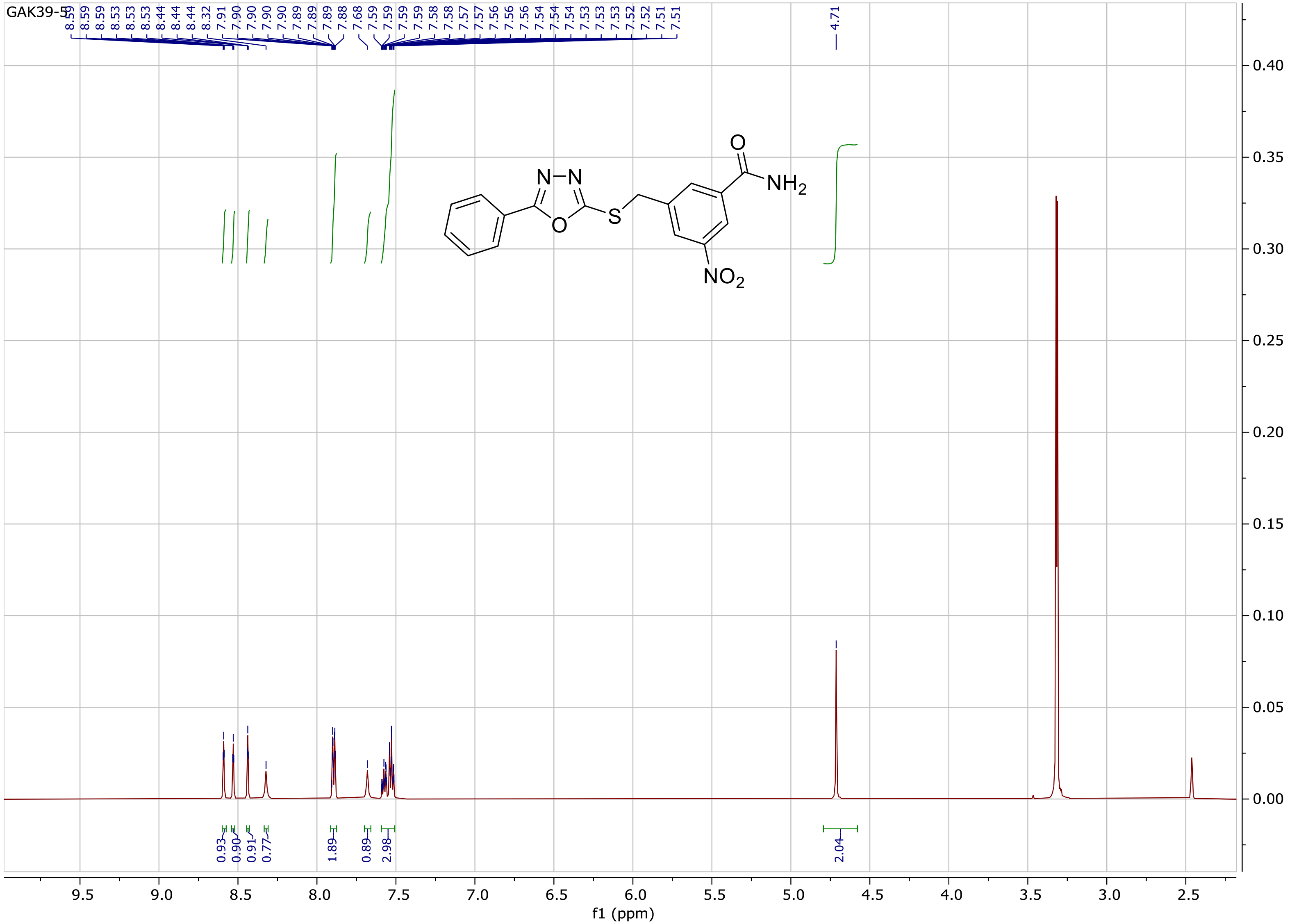

2-((3-(Carbamoyl)-5-nitrobenzyl)sulfanyl)-5-phenyl-1,3,4-oxadiazole (**63a**): ^{13}C NMR (151 MHz, DMSO-d_6)

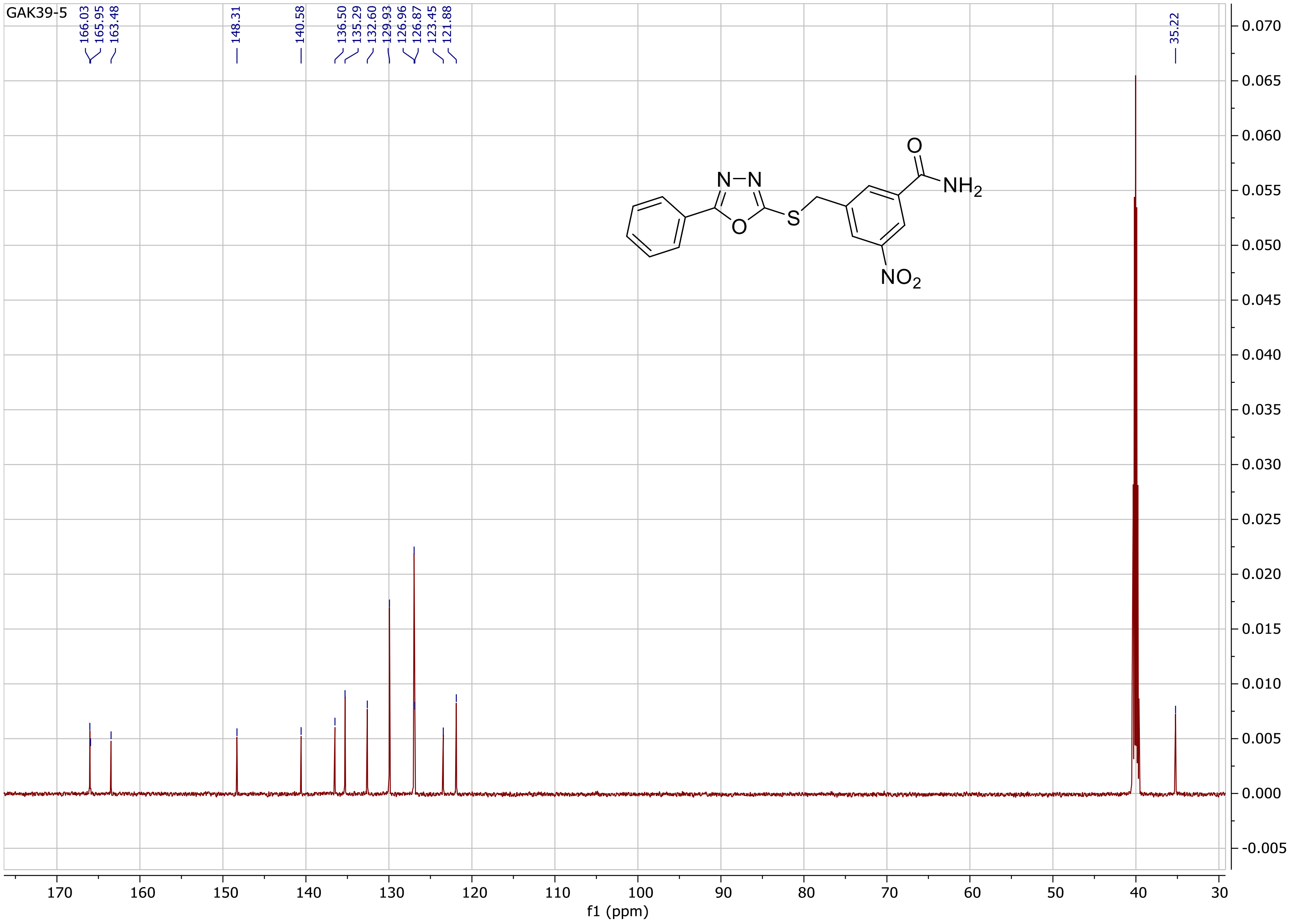

2-((3-(Carbamoyl) -5-nitrobenzyl)sulfanyl)-5-(4-methoxyphenyl)-1,3,4-oxadiazole (**63b**): ^{1}H NMR (600 MHz, DMSO-d_6)

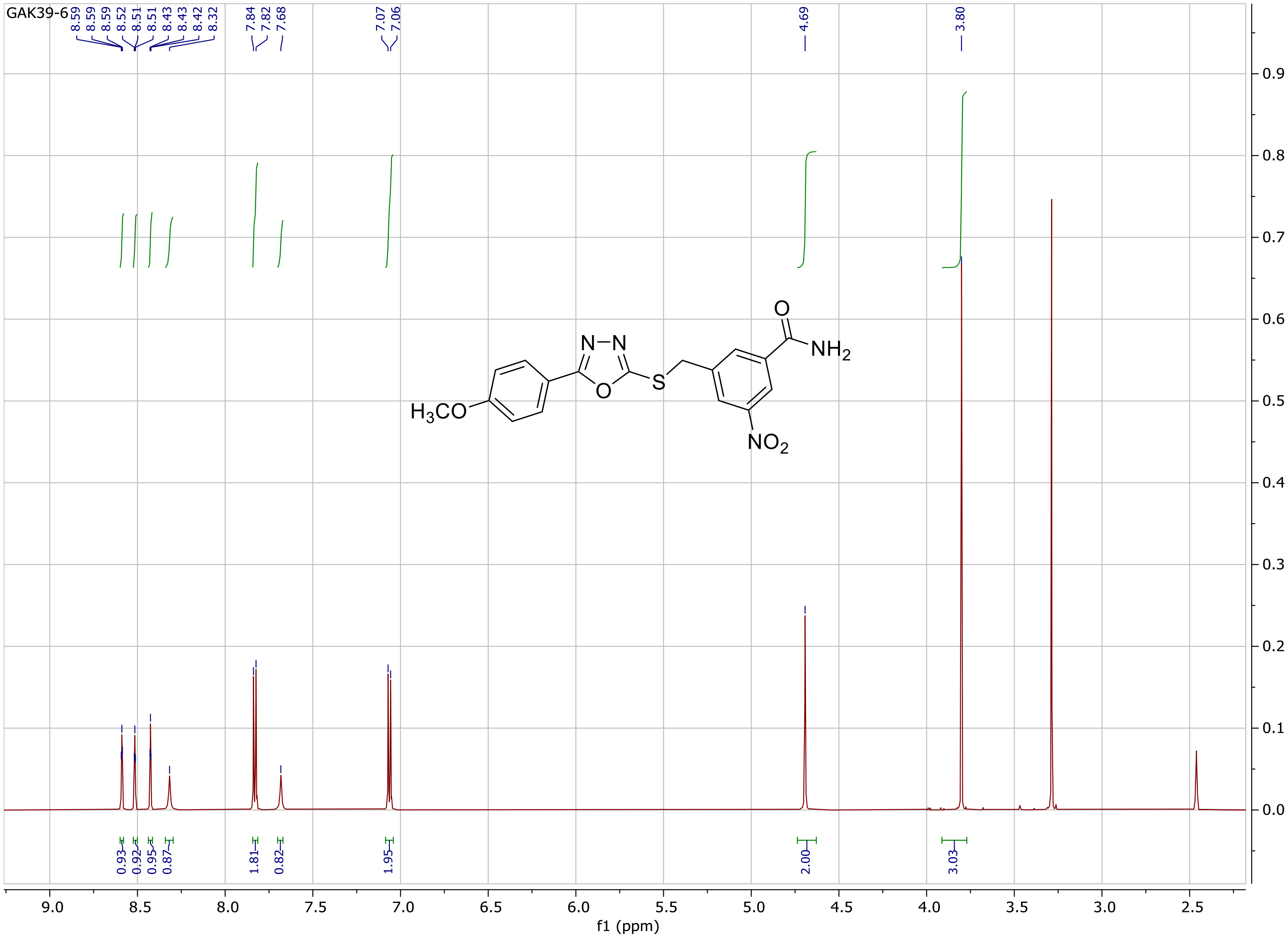

2-((3-(Carbamoyl)-5-nitrobenzyl)sulfanyl)-5-(4-methoxyphenyl)-1,3,4-oxadiazole (**63b**): ^{13}C NMR (151 MHz, DMSO-d_6)

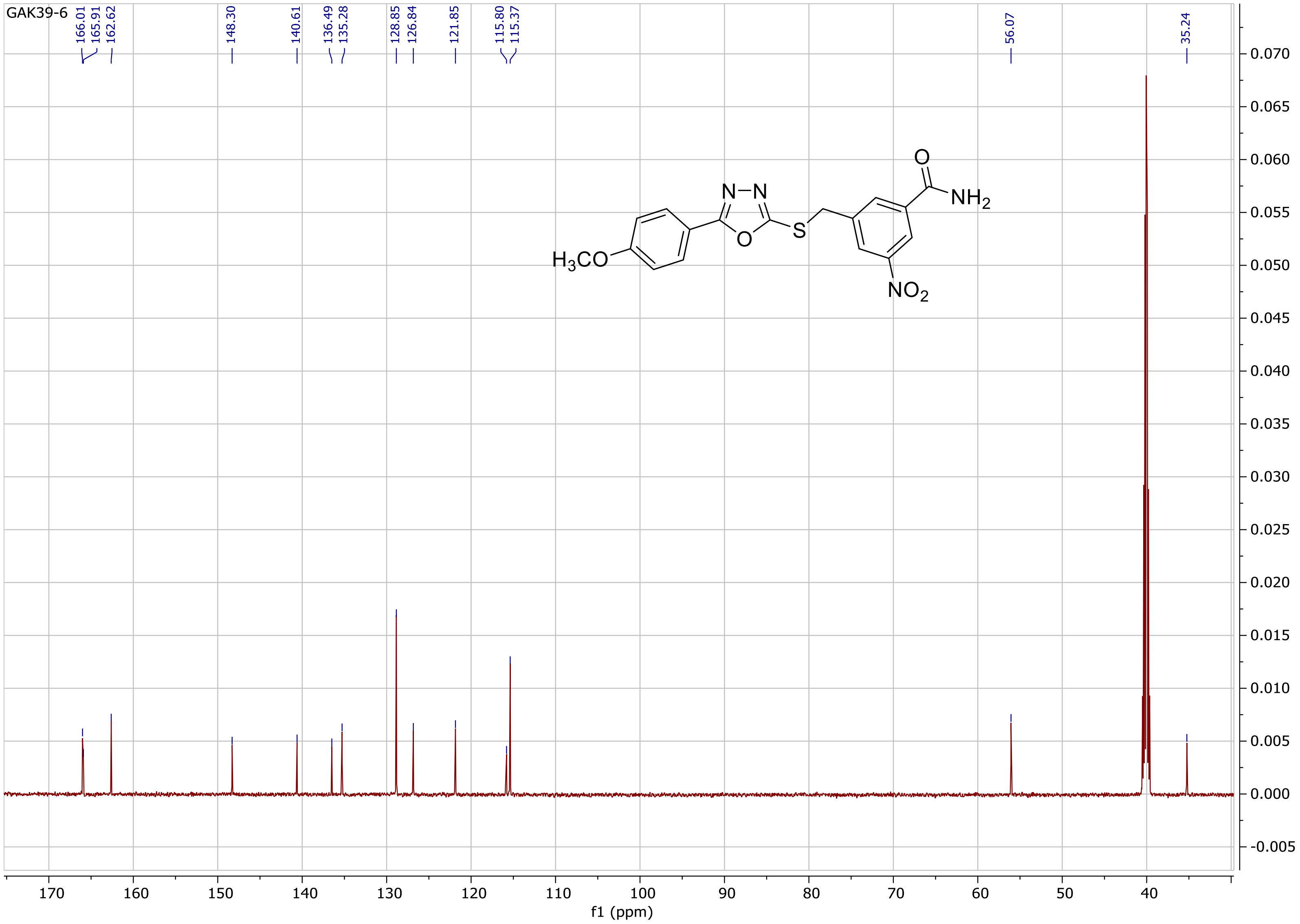

2-((3-(Carbamoyl)-5-nitrobenzyl)sulfanyl)-5-(4-chlorophenyl)-1,3,4-oxadiazole (**63c**): ^{1}H NMR (600 MHz, DMSO-d_6)

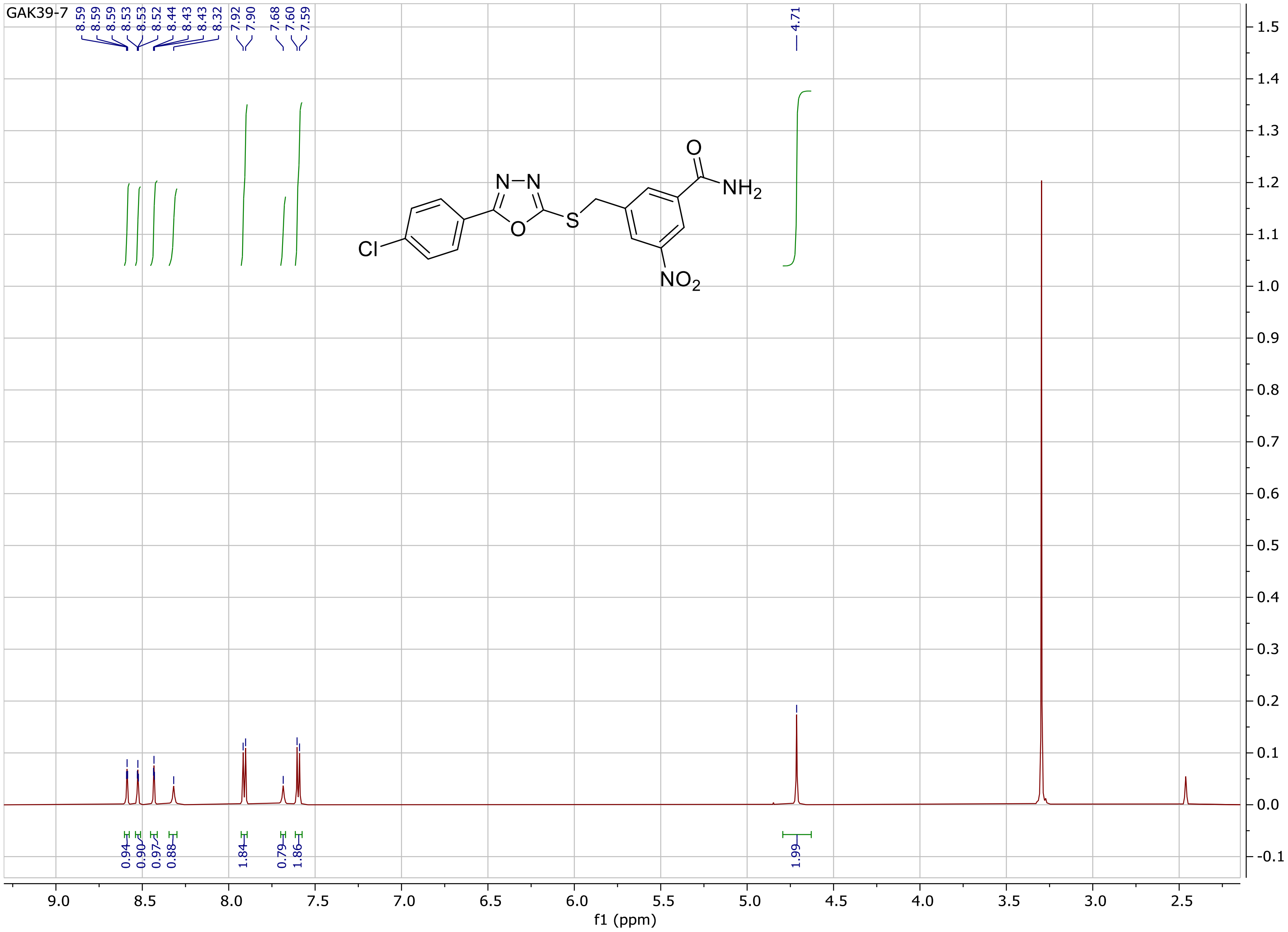

2-((3-(Carbamoyl)-5-nitrobenzyl)sulfanyl)-5-(4-chlorophenyl)-1,3,4-oxadiazole (**63c**): ^{13}C NMR (151 MHz, DMSO-d_6)

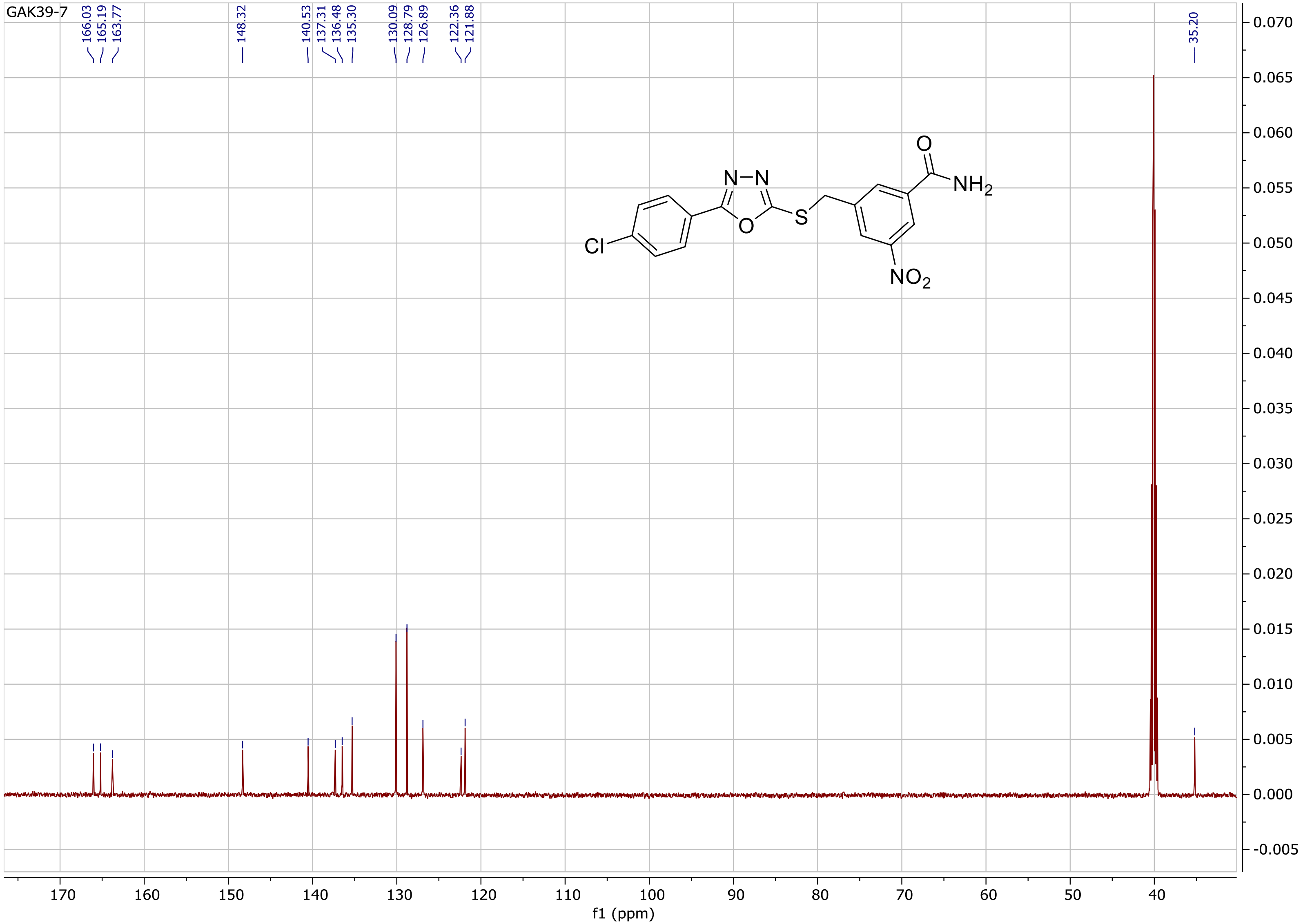

5-(4-Bromophenyl)-2-((3-(carbamoyl)-5-nitrobenzyl)sulfanyl)-1,3,4-oxadiazole (**63d**): ^{1}H NMR (600 MHz, DMSO-*d*$_6$)

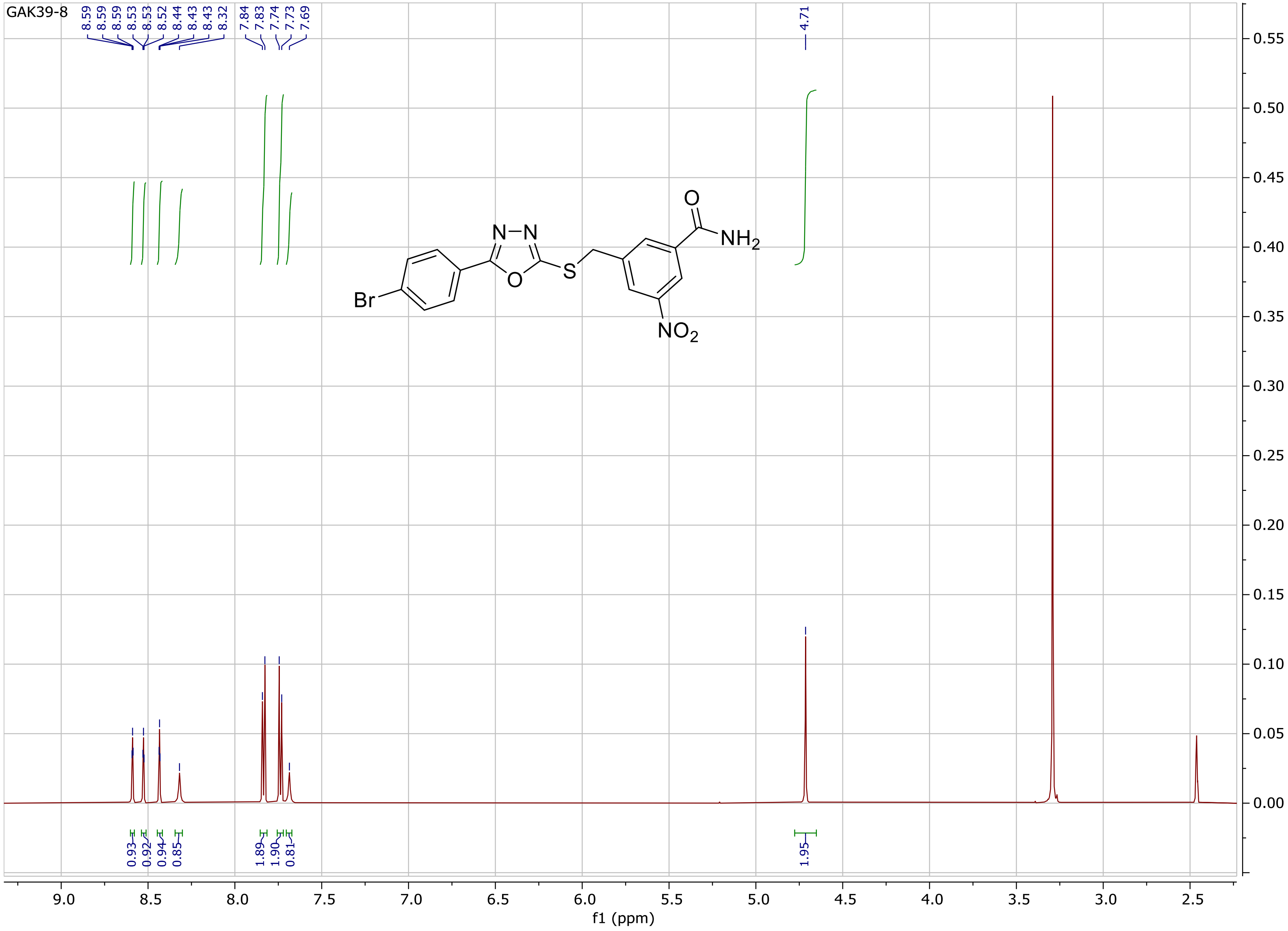

5-(4-Bromophenyl)-2-((3-(carbamoyl)-5-nitrobenzyl)sulfanyl)-1,3,4-oxadiazole (**63d**): ^{13}C NMR (151 MHz, DMSO-d_6)

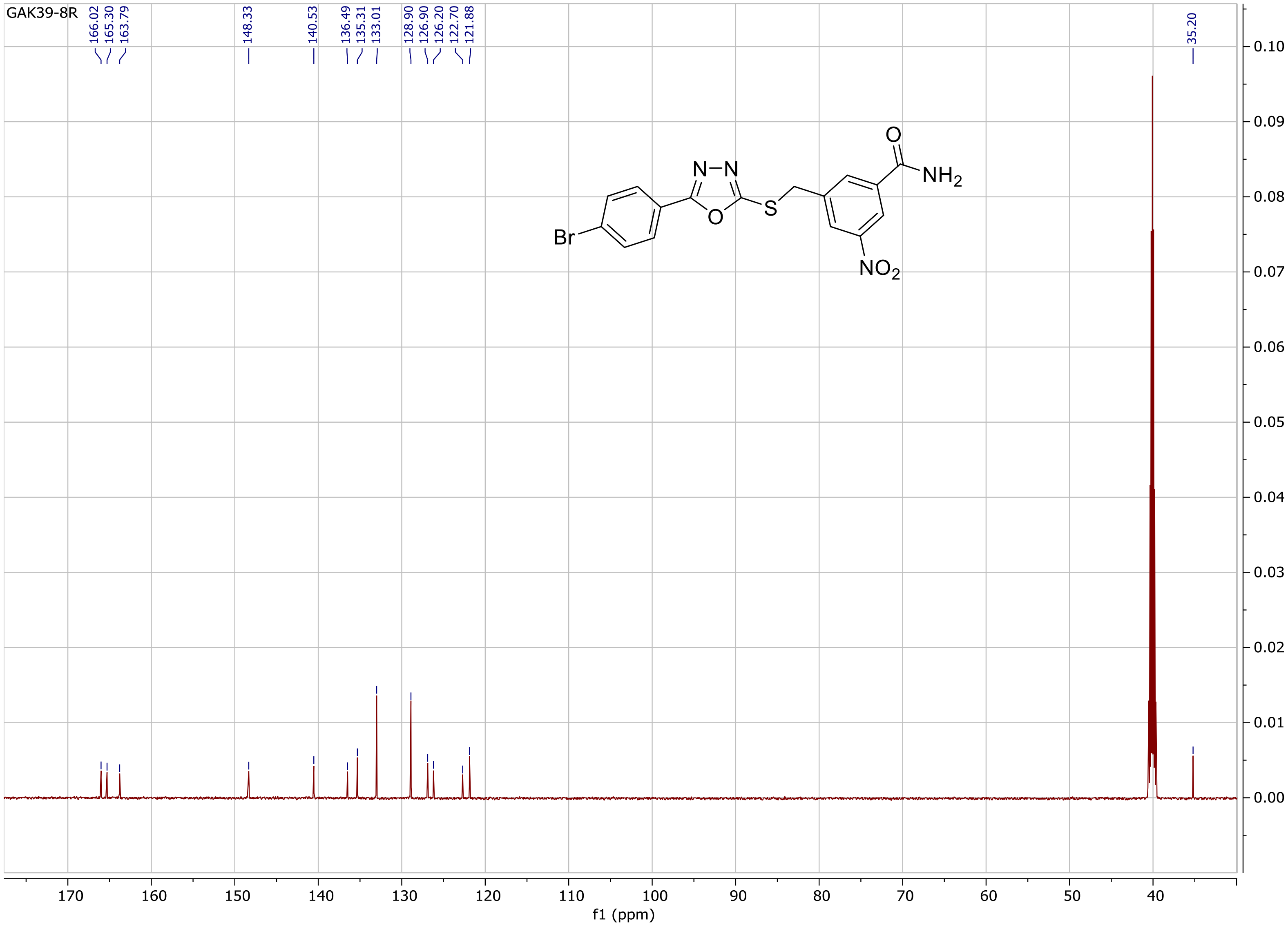

5-((3-(Carbamoyl)-5-nitrobenzyl)sulfanyl)-2-cyclohexyl-1,3,4-oxadiazole (**63e**): ^{1}H NMR (600 MHz, DMSO-d_6)

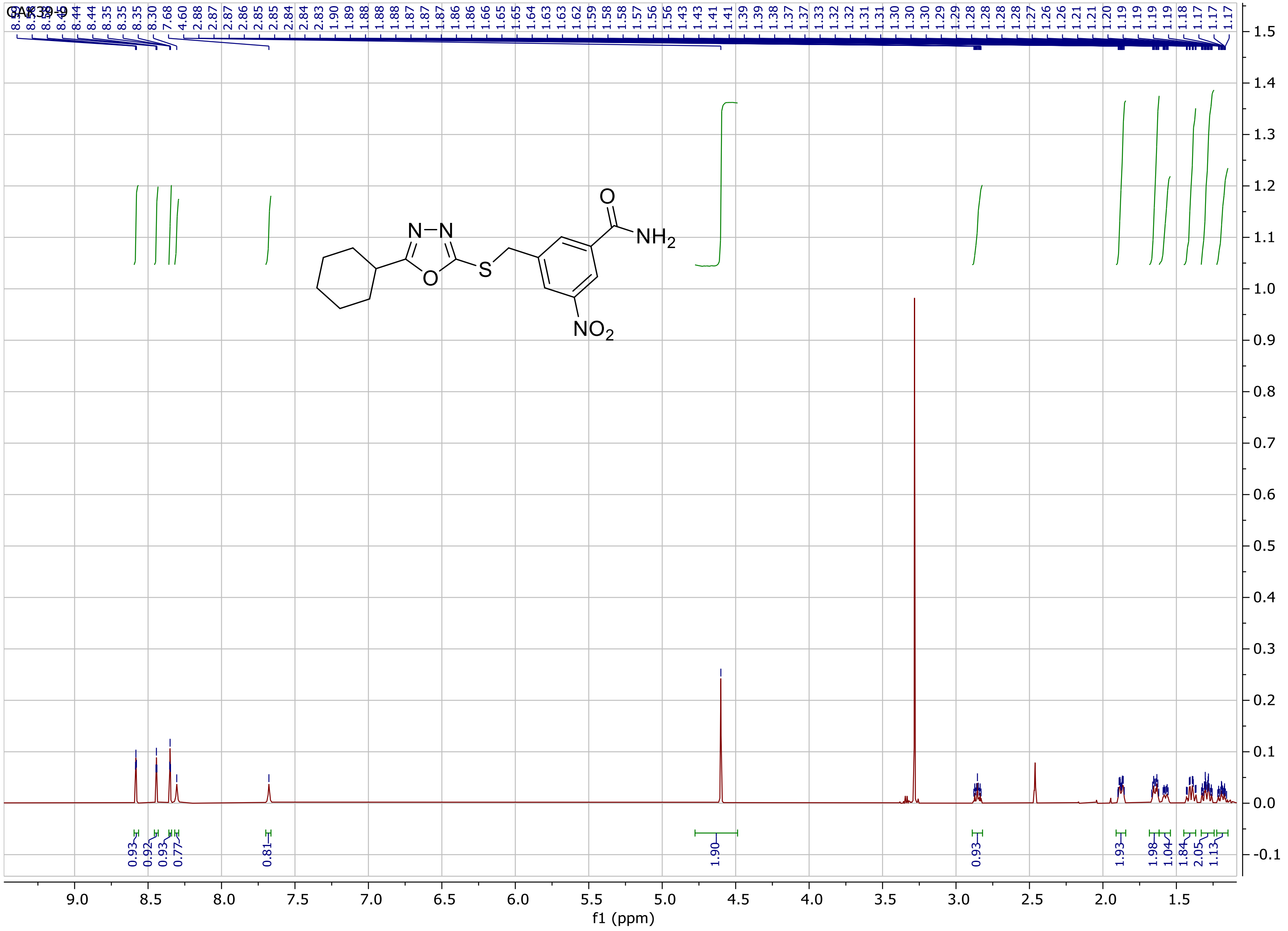

5-((3-(Carbamoyl)-5-nitrobenzyl)sulfanyl)-2-cyclohexyl-1,3,4-oxadiazole (**63e**): ^{13}C NMR (151 MHz, DMSO-d_6)

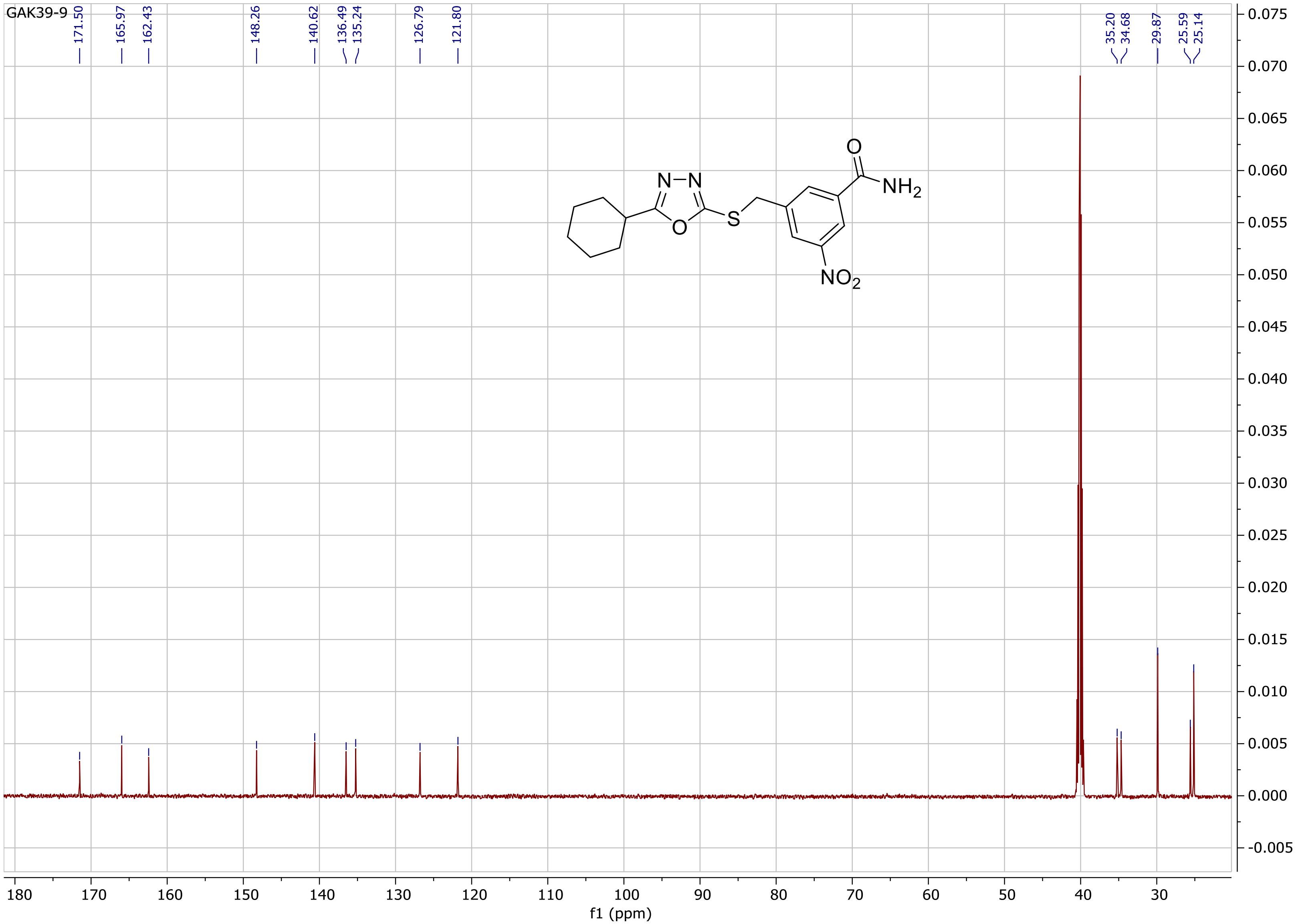

2-((3-(N-benzylcarbamoyl)-5-nitrobenzyl)sulfanyl)-5-phenyl-1,3,4-oxadiazole (**64a**): ^{1}H NMR (600 MHz, DMSO-d_6)

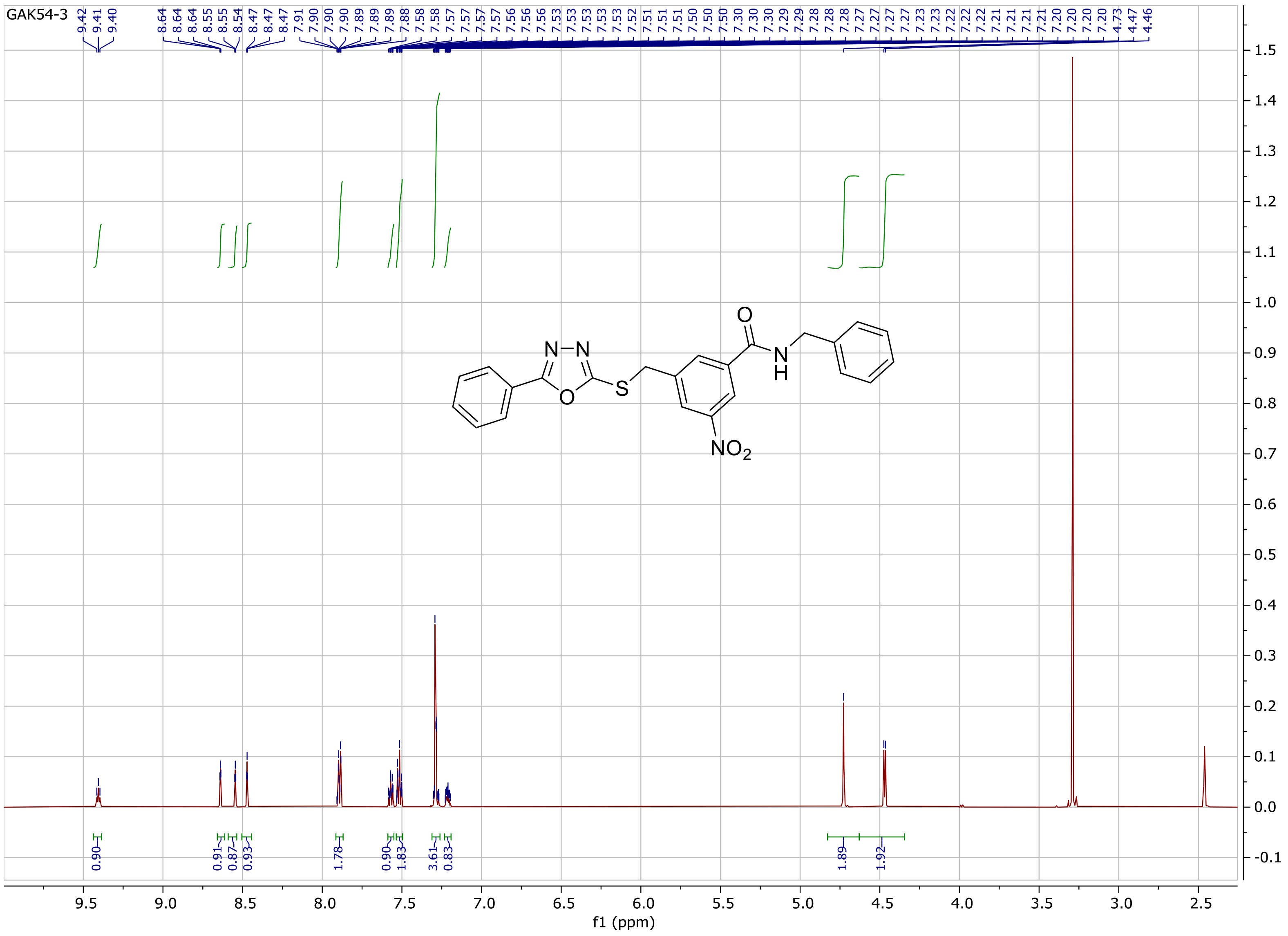

2-((3-(N-benzylcarbamoyl)-5-nitrobenzyl)sulfanyl)-5-phenyl-1,3,4-oxadiazole (**64a**): ^{13}C NMR (151 MHz, DMSO-d_6)

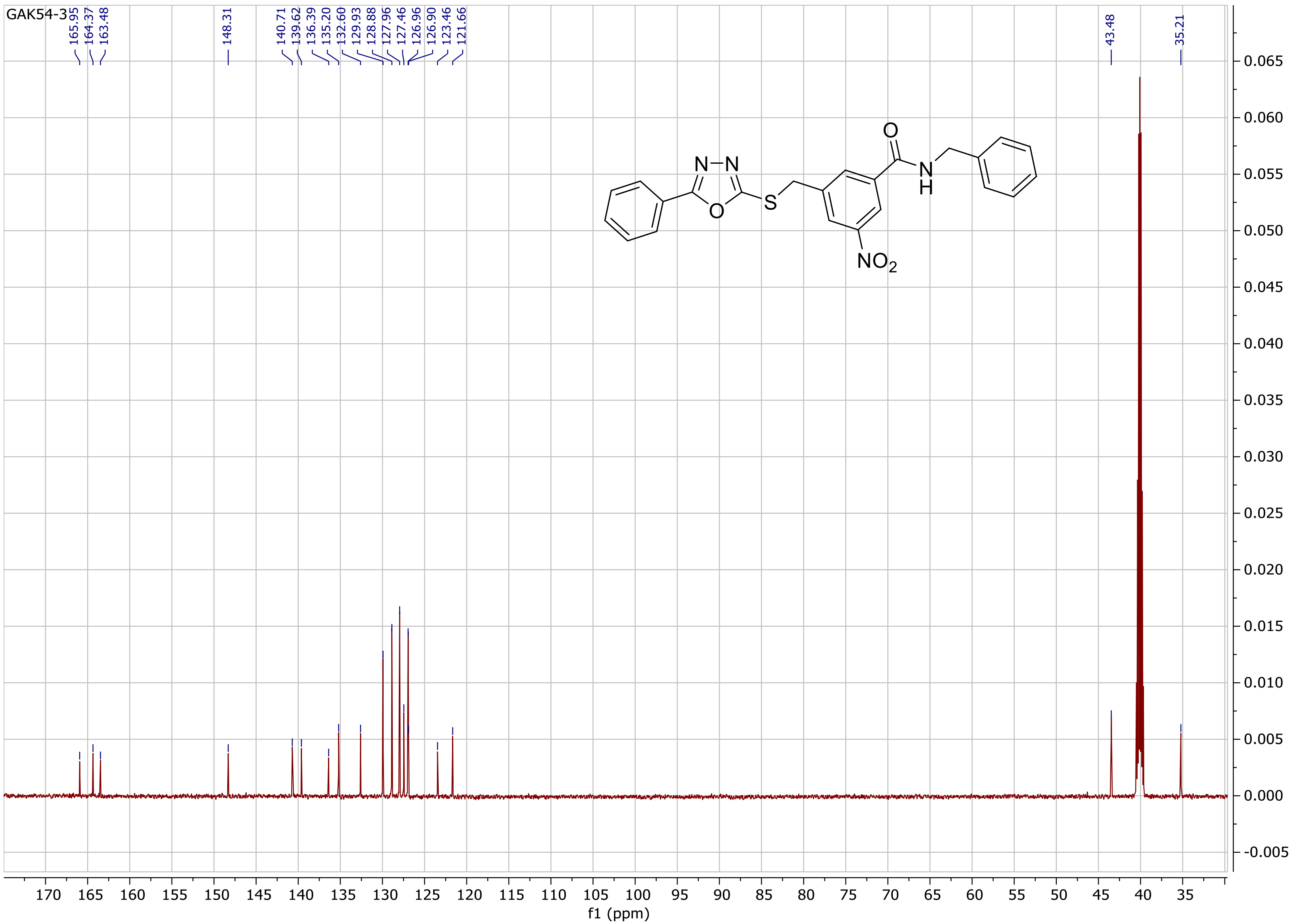

2-((3-(N-benzylcarbamoyl)-5-nitrobenzyl)sulfanyl)-5-(4-methoxyphenyl)-1,3,4-oxadiazole (**64b**): ^{1}H NMR (500 MHz, DMSO-d_6)

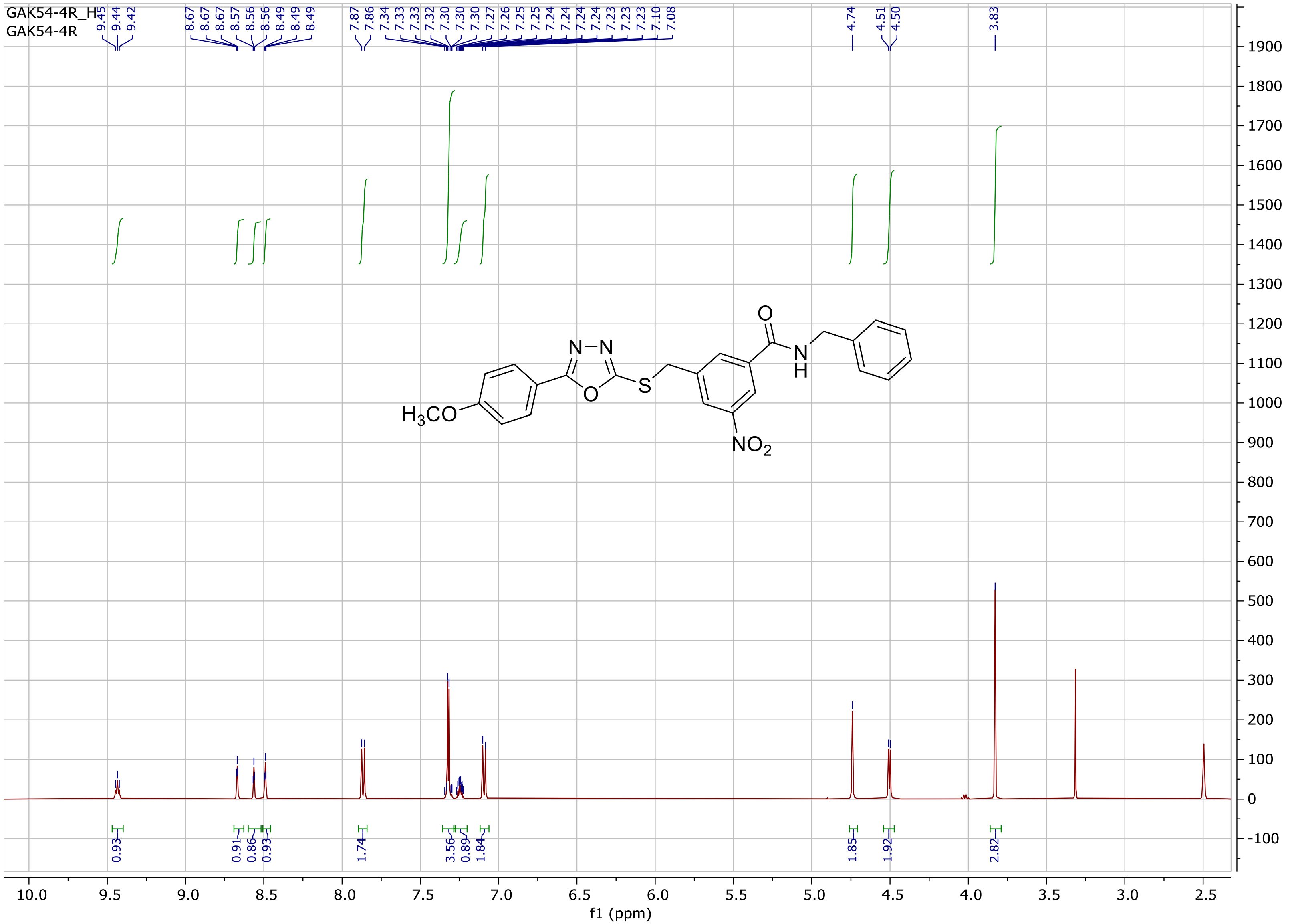

2-((3-(N-benzylcarbamoyl)-5-nitrobenzyl)sulfanyl)-5-(4-methoxyphenyl)-1,3,4-oxadiazole (**64b**): ^{13}C NMR (126 MHz, DMSO-d_6)

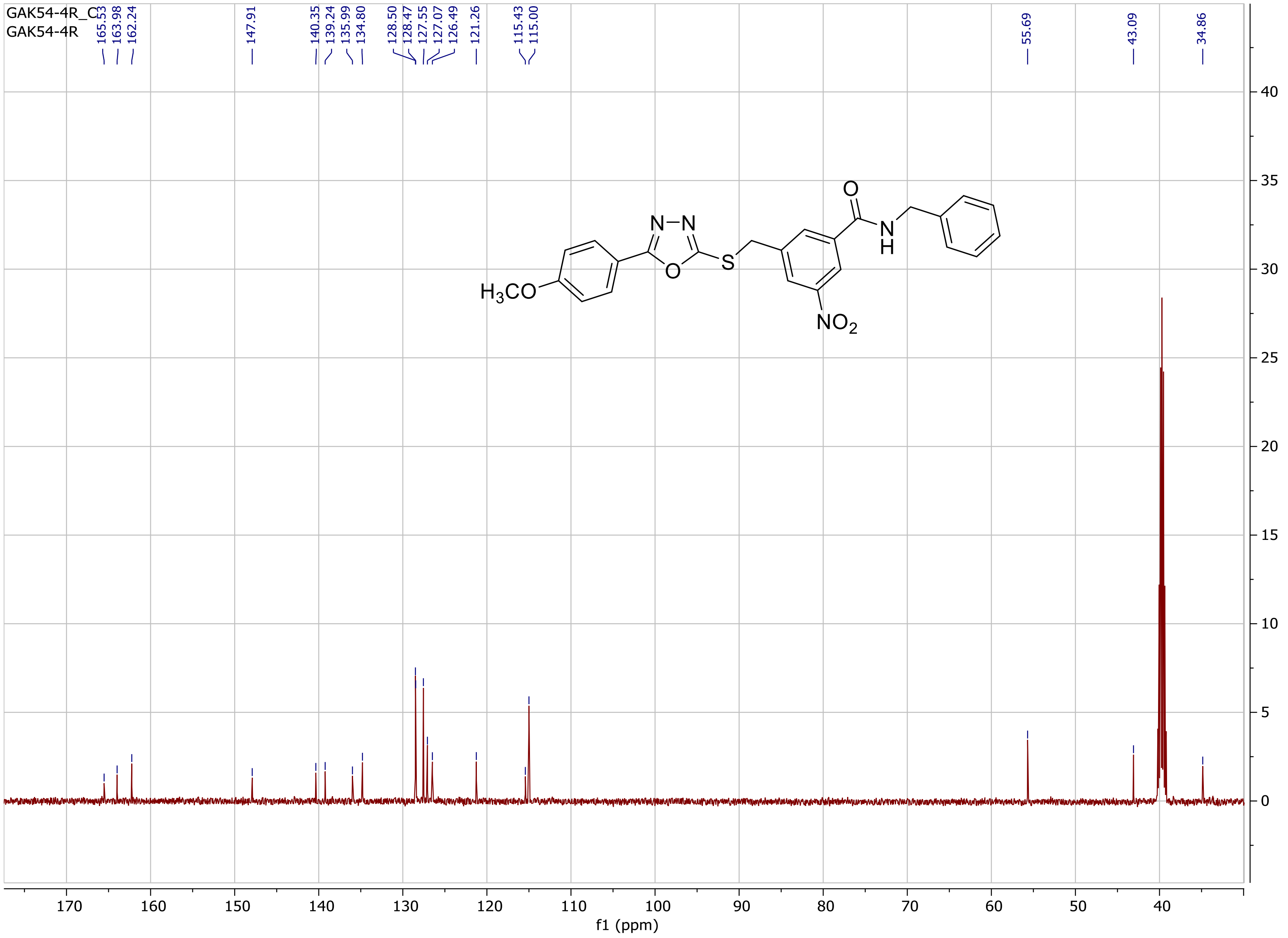

2-((3-(N-benzylcarbamoyl)-5-nitrobenzyl)sulfanyl)-5-(4-chlorophenyl)-1,3,4-oxadiazole (**64c**): ^{1}H NMR (600 MHz, DMSO-d_6)

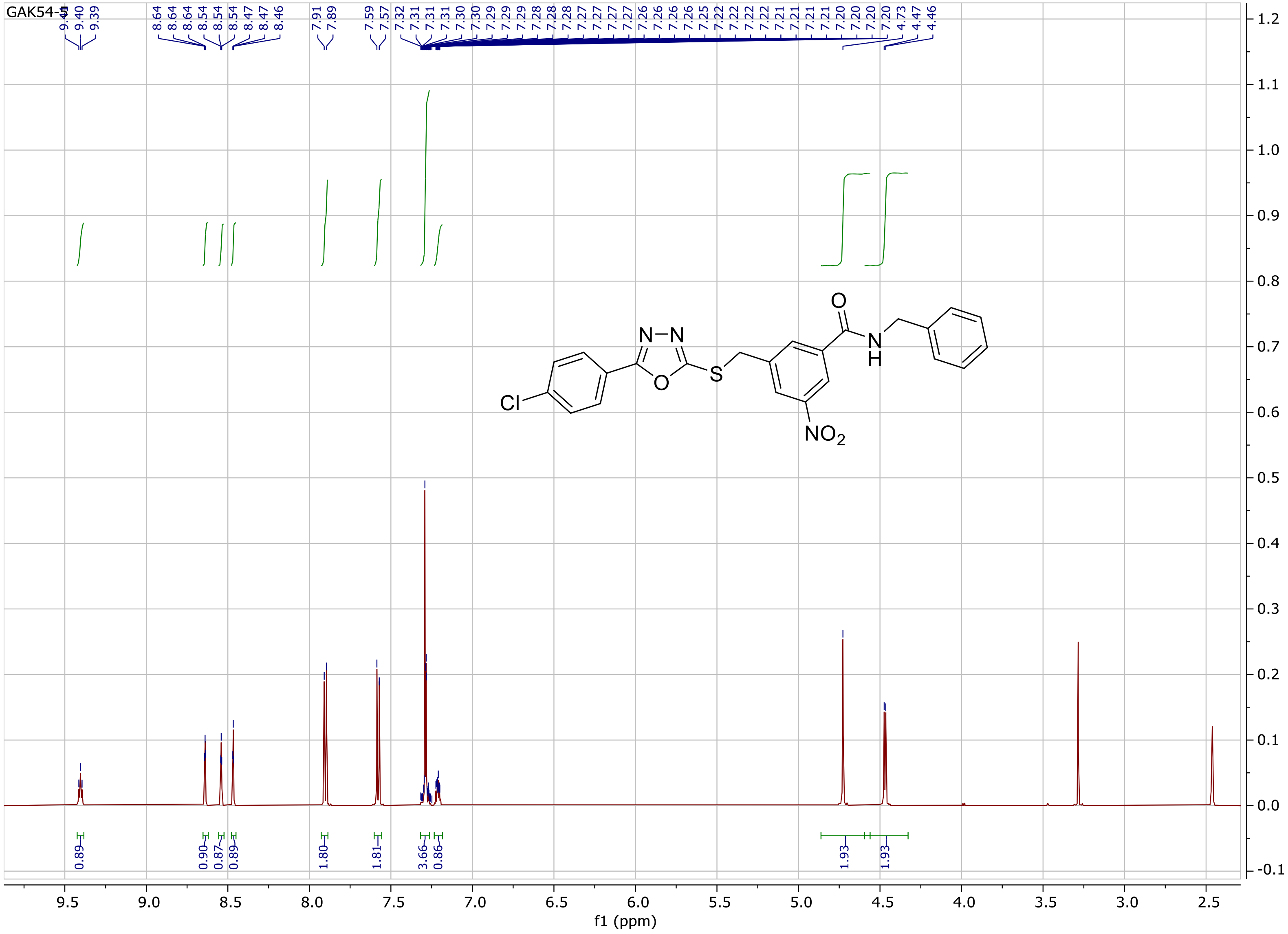

2-((3-(N-benzylcarbamoyl)-5-nitrobenzyl)sulfanyl)-5-(4-chlorophenyl)-1,3,4-oxadiazole (**64c**): ^{13}C NMR (151 MHz, DMSO-d_6)

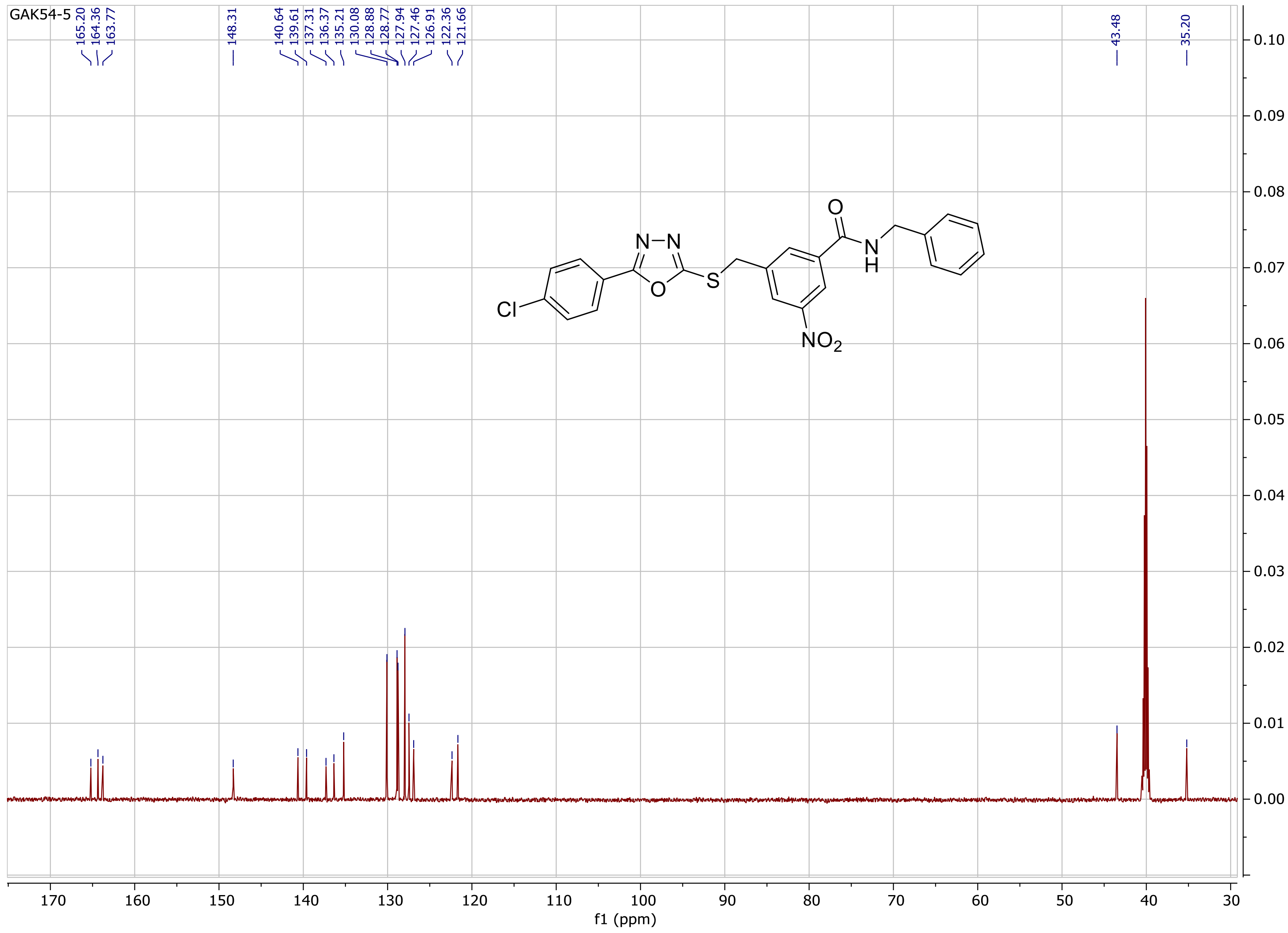

2-((3-(N-benzylcarbamoyl)-5-nitrobenzyl)sulfanyl)-5-(4-bromophenyl)-1,3,4-oxadiazole (**64d**): ^{1}H NMR (600 MHz, DMSO-d_6)

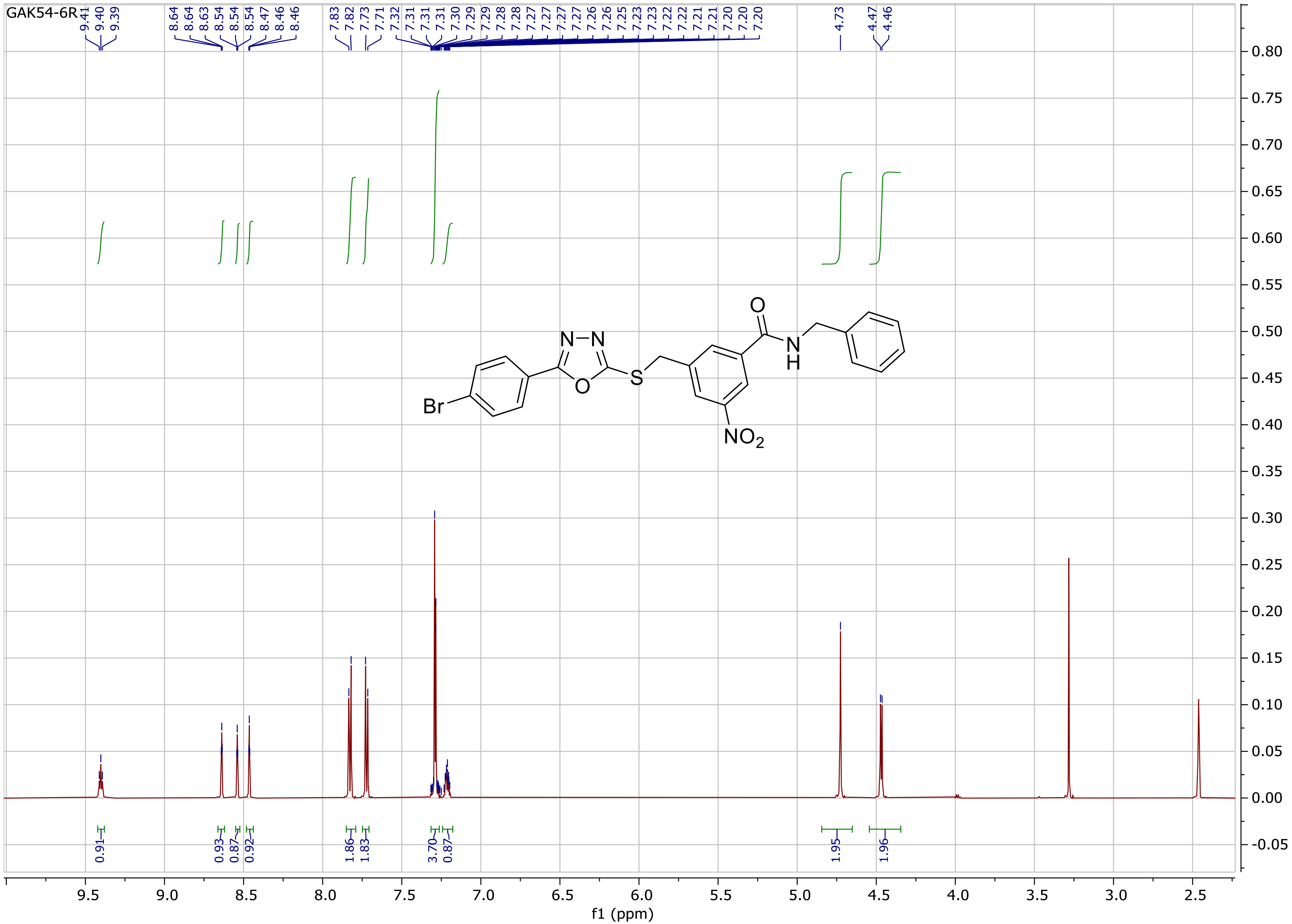

2-((3-(N-benzylcarbamoyl)-5-nitrobenzyl)sulfanyl)-5-(4-bromophenyl)-1,3,4-oxadiazole (**64d**): ^{13}C NMR (151 MHz, DMSO-d_6)

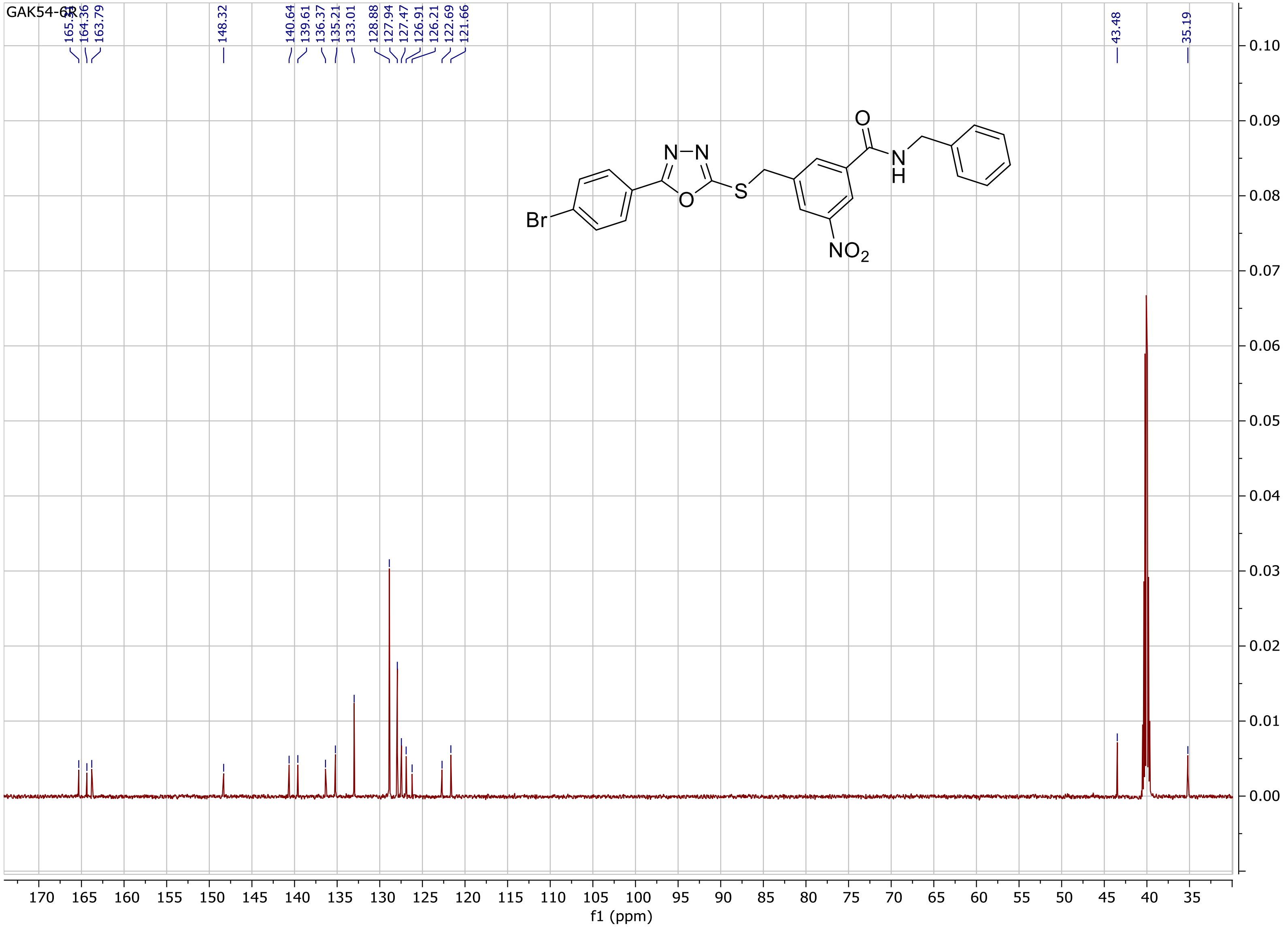

2-((3-(N-benzylcarbamoyl)-5-nitrobenzyl)sulfanyl)-5-cyclohexyl-1,3,4-oxadiazole (**64e**): ^{1}H NMR (600 MHz, DMSO-d_6)

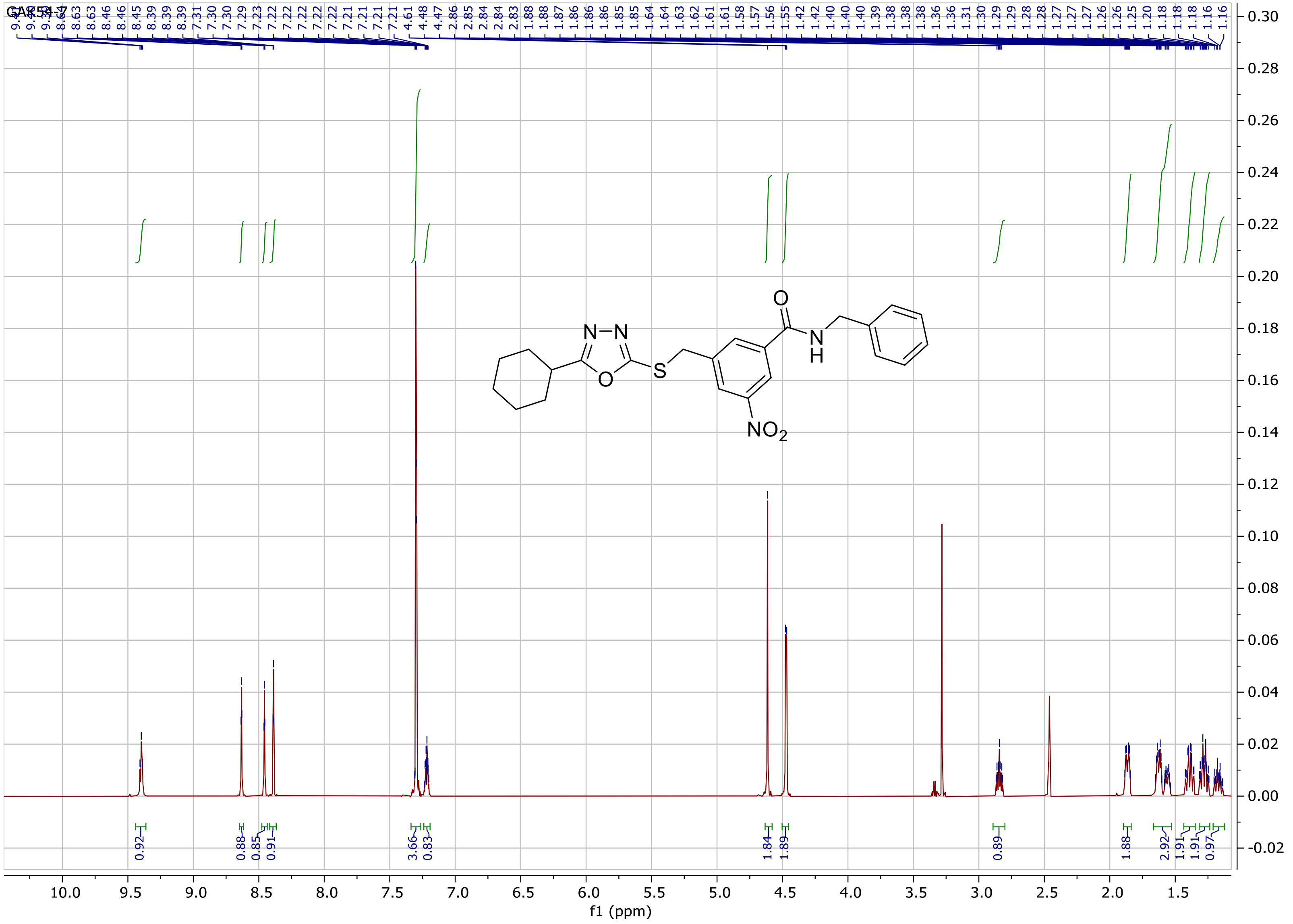

2-((3-(N-benzylcarbamoyl)-5-nitrobenzyl)sulfanyl)-5-cyclohexyl-1,3,4-oxadiazole (**64e**): ^{13}C NMR (151 MHz, DMSO-d_6)

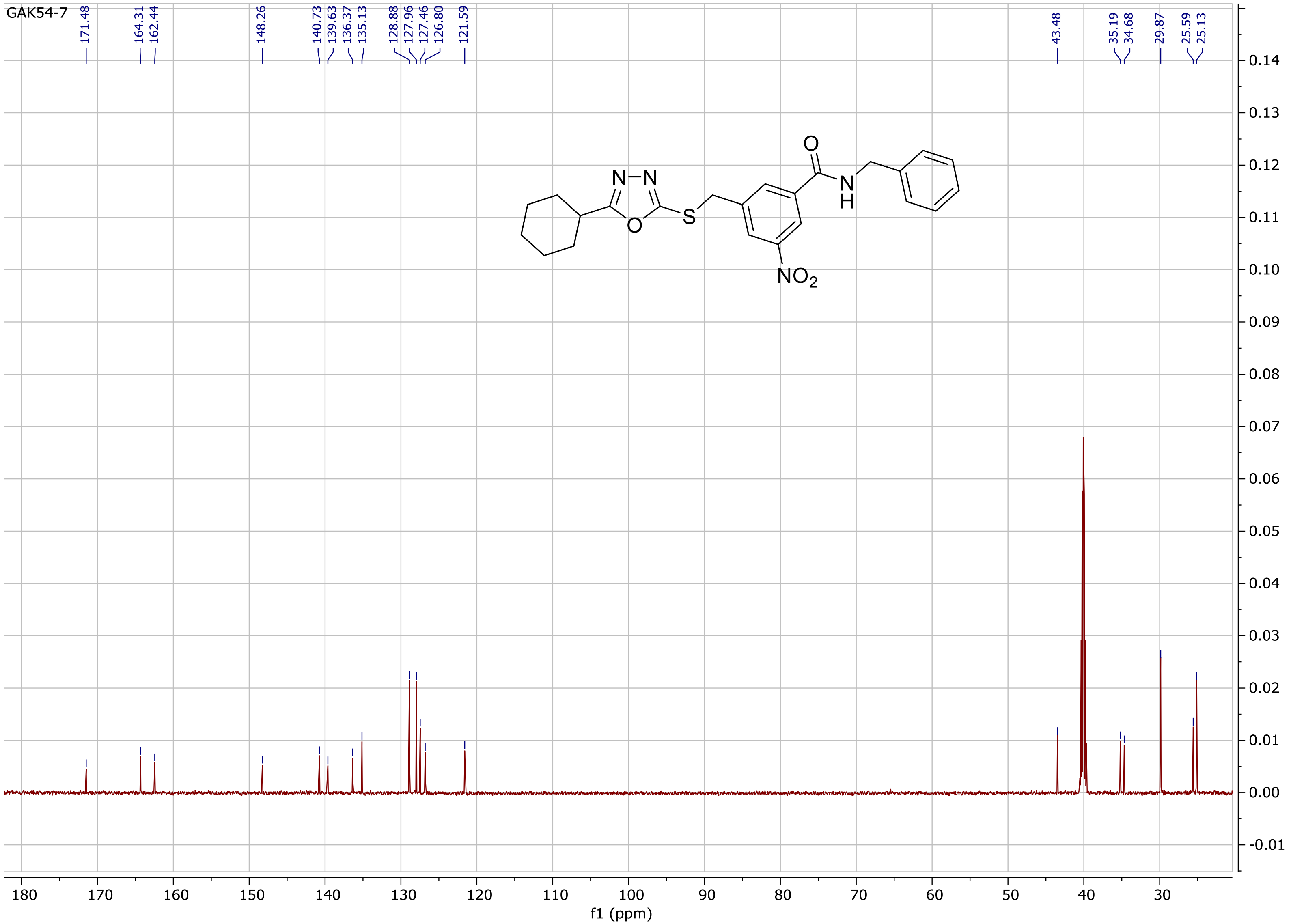

2-((3-Nitro-5-(1H-pyrrol-1-yl)benzyl)sulfanyl)-5-phenyl-1,3,4-oxadiazole (**65a**): ^{1}H NMR (600 MHz, DMSO-d_6)

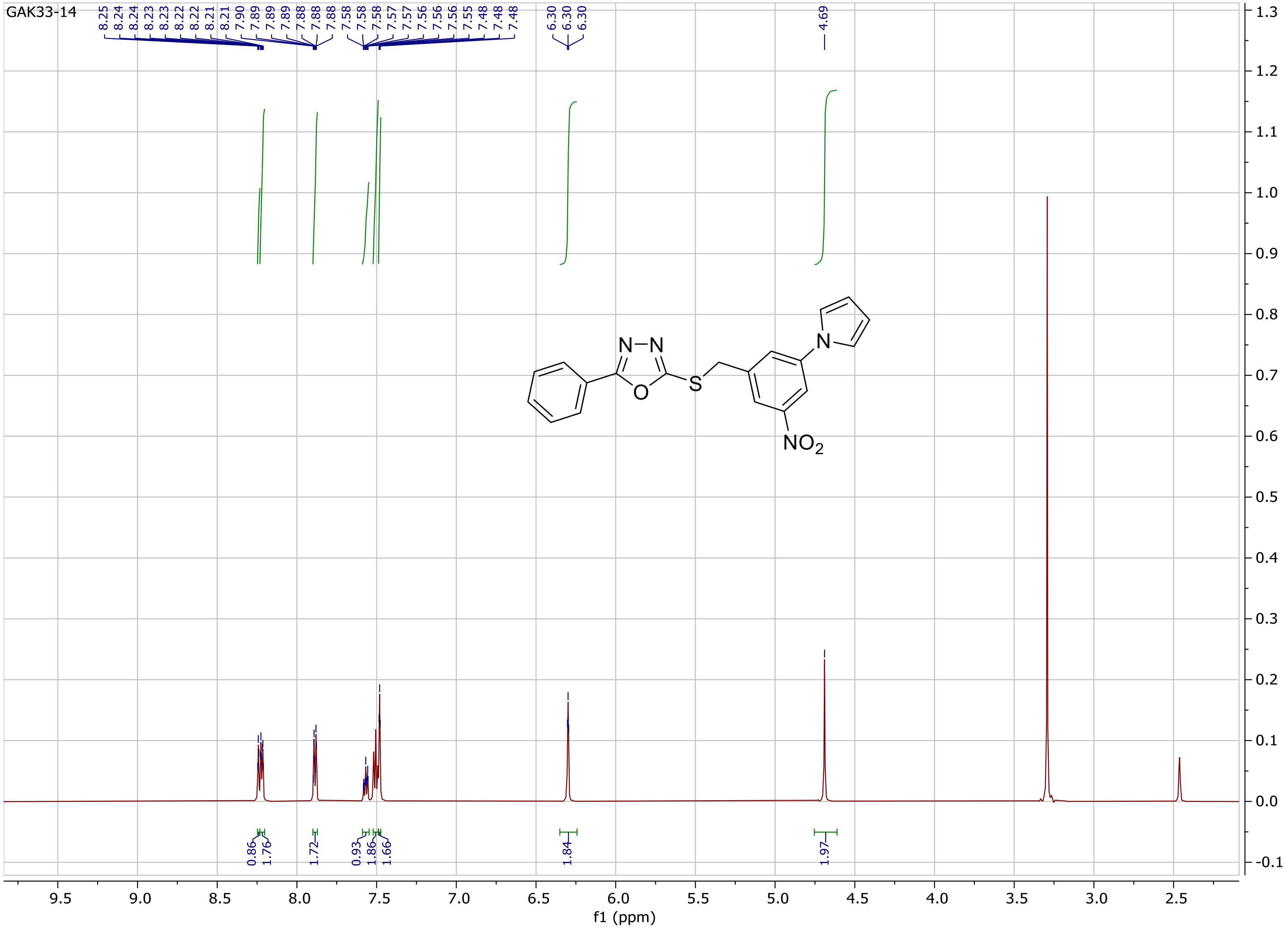

2-((3-Nitro-5-(1H-pyrrol-1-yl)benzyl)sulfanyl)-5-phenyl-1,3,4-oxadiazole (**65a**): ^{13}C NMR (151 MHz, DMSO-d_6)

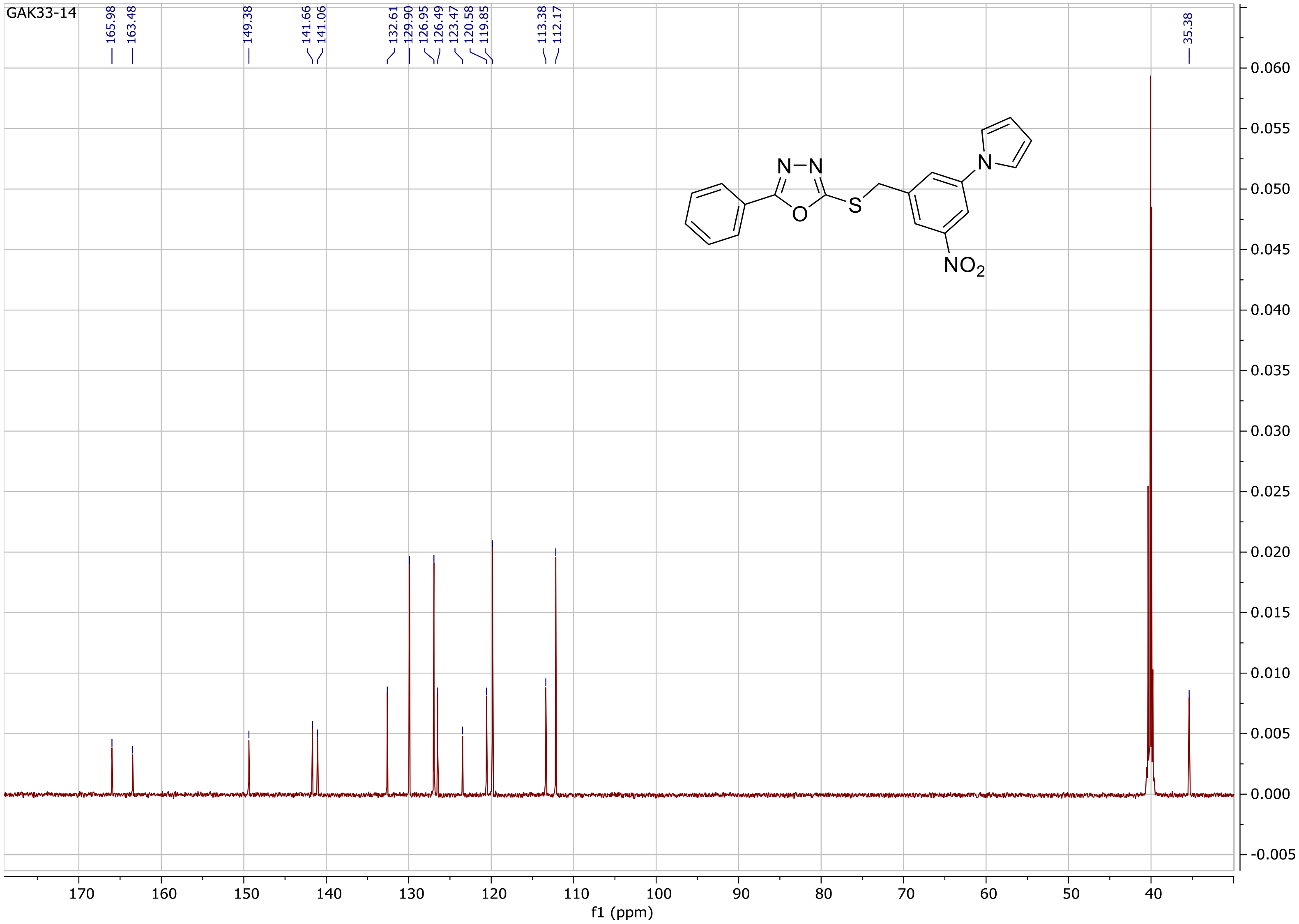

2-(4-Methoxyphenyl)-5-((3-nitro-5-(1H-pyrrol-1-yl)benzyl)sulfanyl)-1,3,4-oxadiazole (**65b**): ^{1}H NMR (600 MHz, DMSO-d_6)

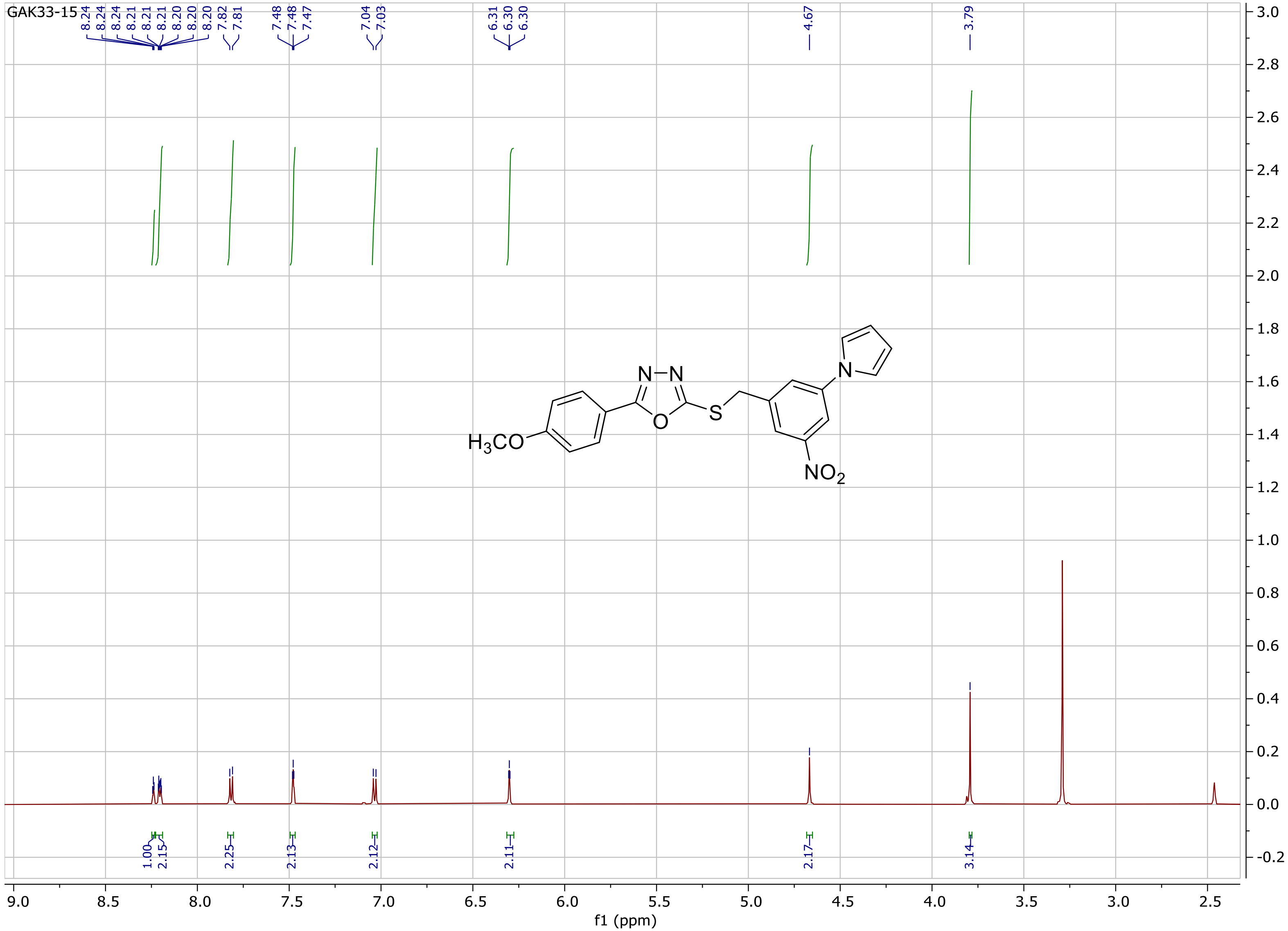

2-(4-Methoxyphenyl)-5-((3-nitro-5-(1H-pyrrol-1-yl)benzyl)sulfanyl)-1,3,4-oxadiazole (**65b**): ^{13}C NMR (151 MHz, DMSO-d_6)

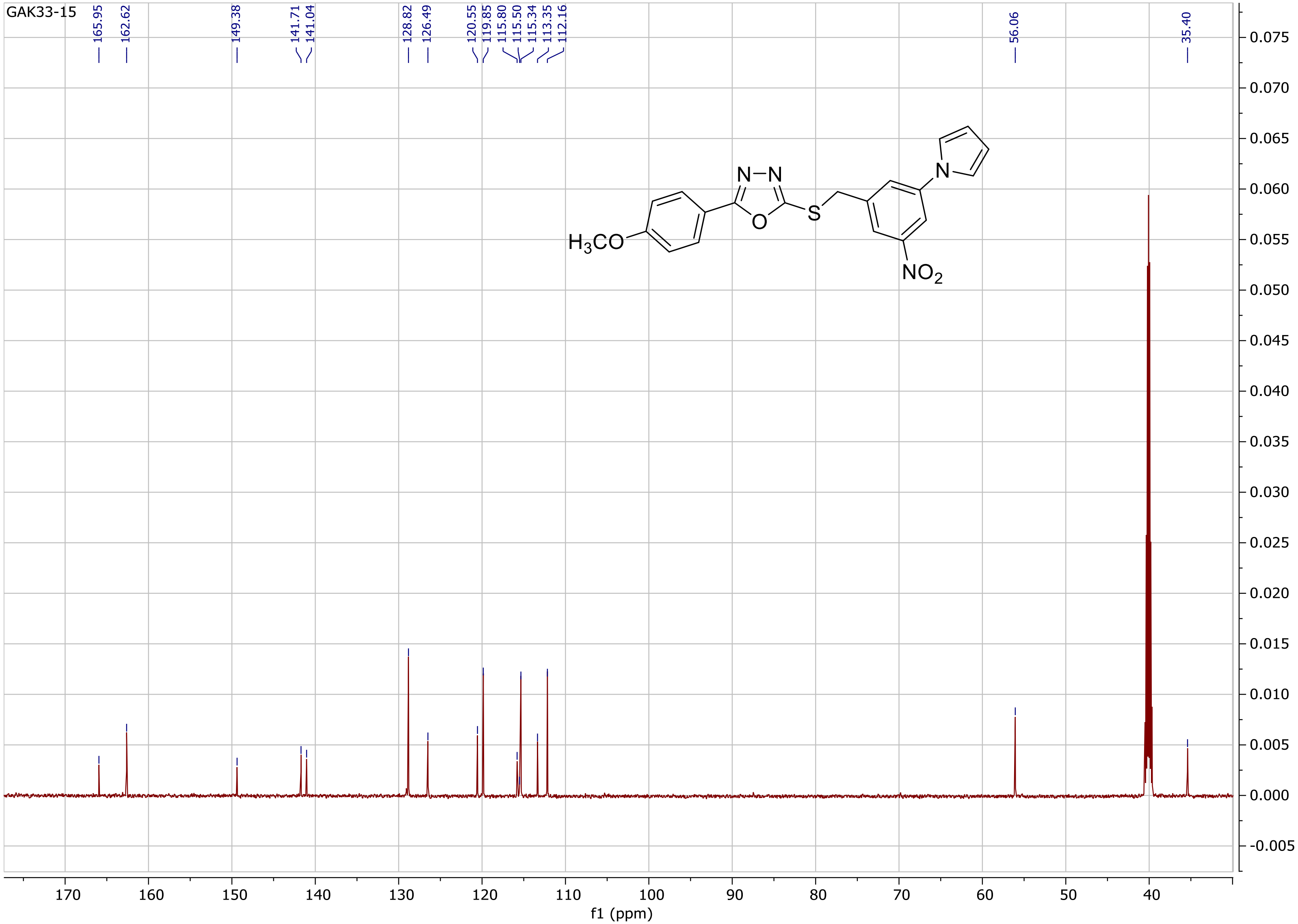

2-(4-Chlorophenyl)-5-((3-nitro-5-(1H-pyrrol-1-yl)benzyl)sulfanyl)-1,3,4-oxadiazole (**65c**): ^{1}H NMR (600 MHz, DMSO-d_6)

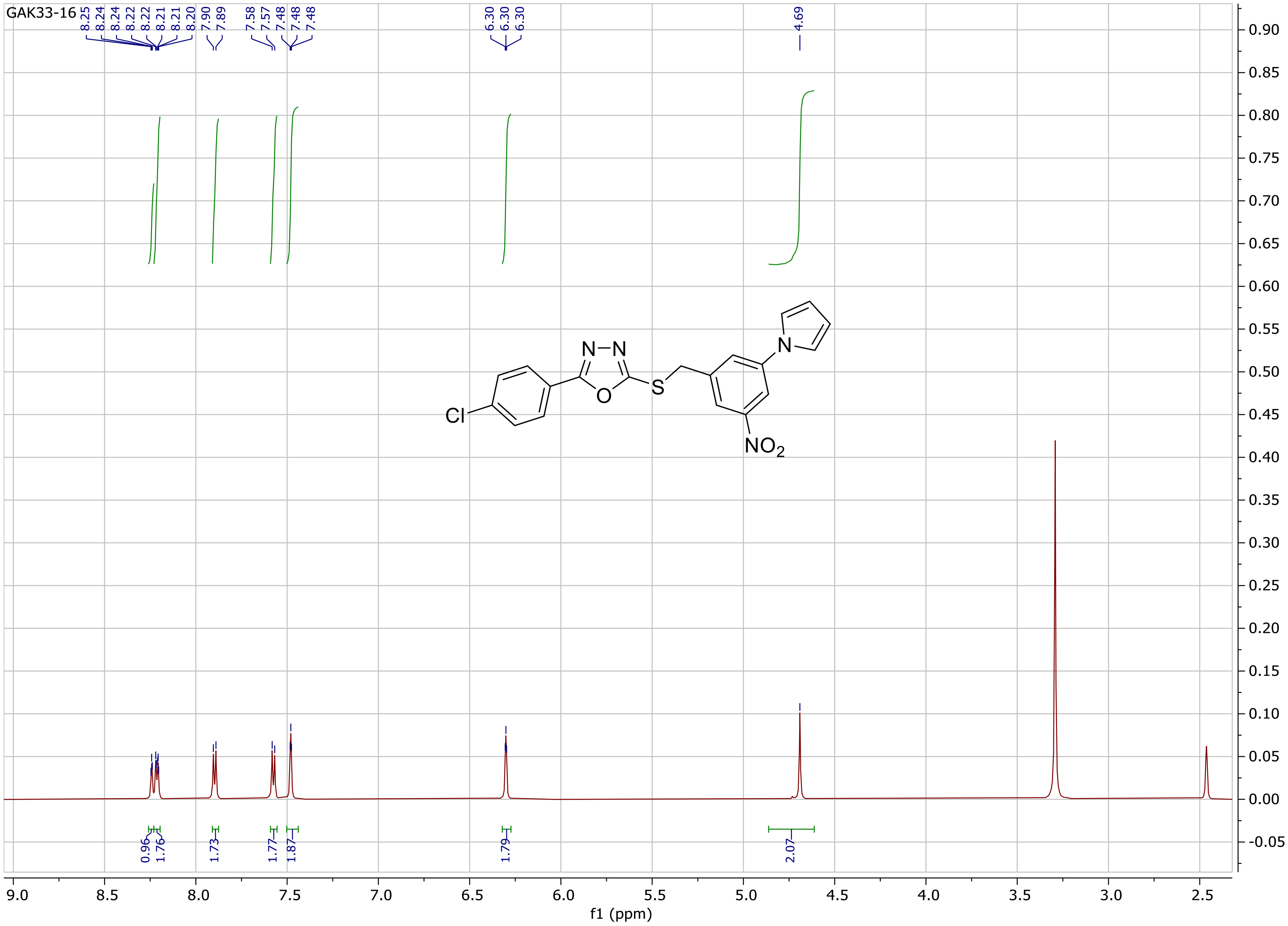

2-(4-Chlorophenyl)-5-((3-nitro-5-(1H-pyrrol-1-yl)benzyl)sulfanyl)-1,3,4-oxadiazole (**65c**): ^{13}C NMR (151 MHz, DMSO-d_6)

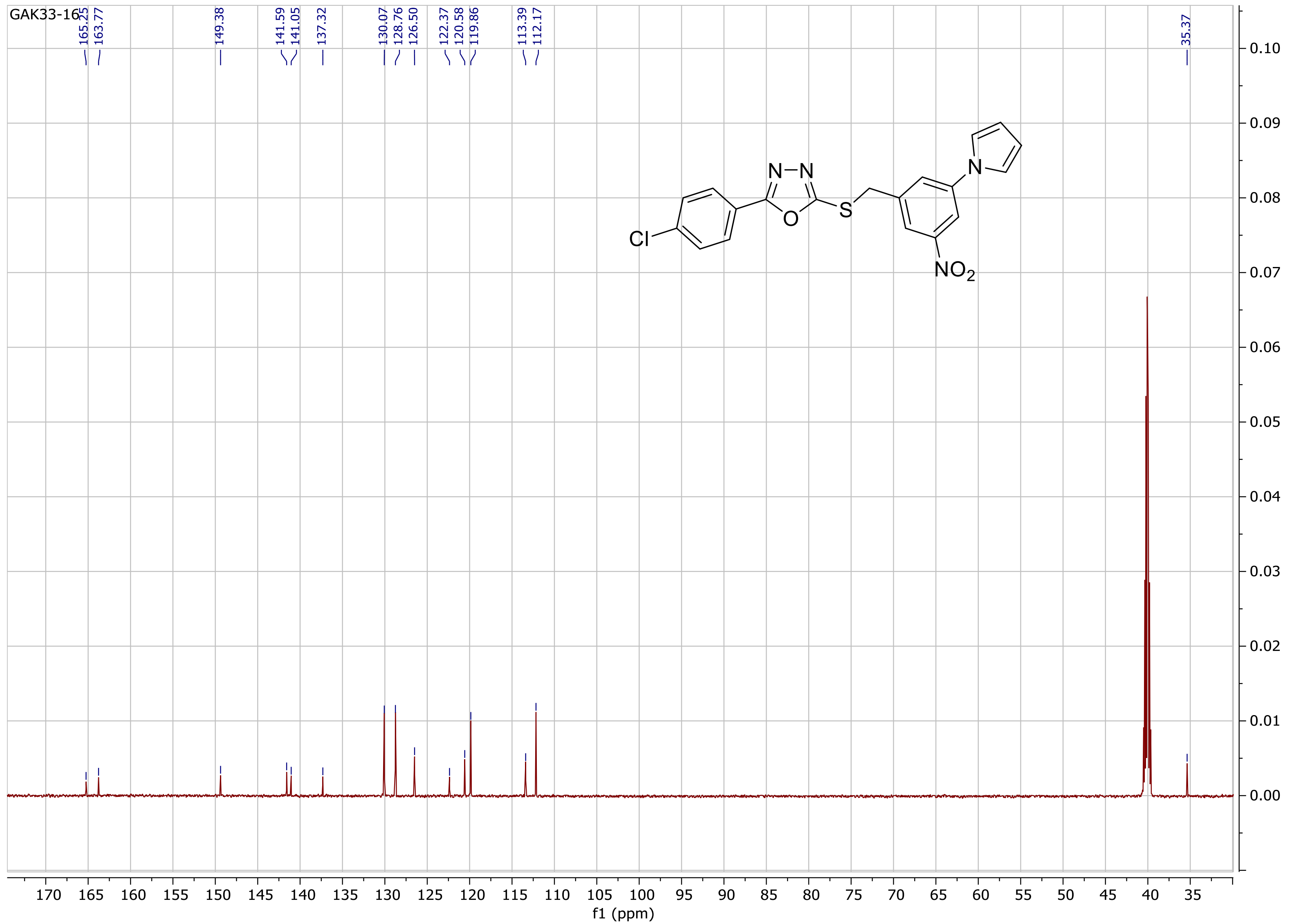

2-(4-Bromophenyl)-5-((3-nitro-5-(1H-pyrrol-1-yl)benzyl)sulfanyl)-1,3,4-oxadiazole (**65d**): ^{1}H NMR (500 MHz, DMSO-d_6)

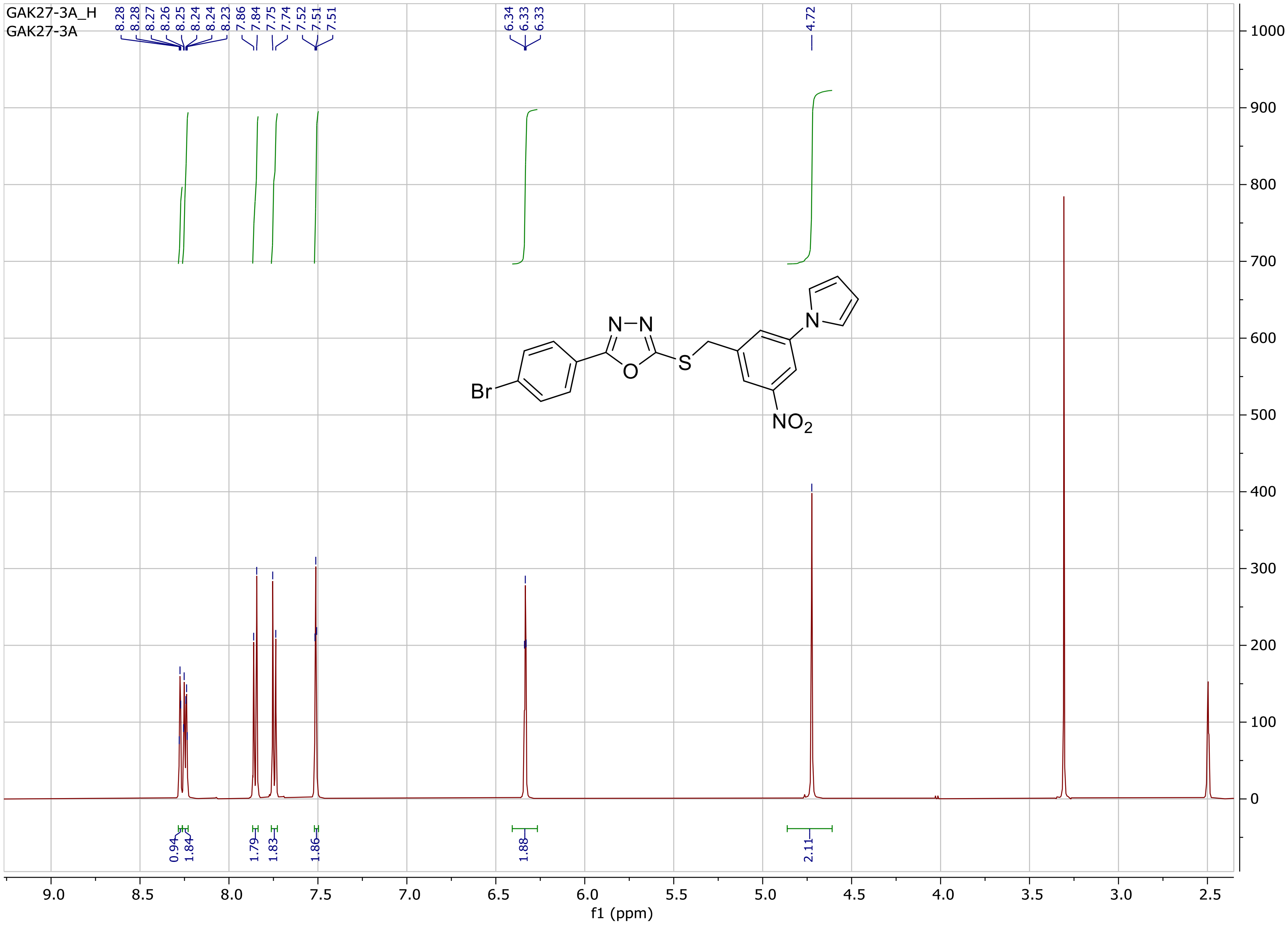

2-(4-Bromophenyl)-5-((3-nitro-5-(1H-pyrrol-1-yl)benzyl)sulfanyl)-1,3,4-oxadiazole (**65d**): ^{13}C NMR (126 MHz, DMSO-d_6)

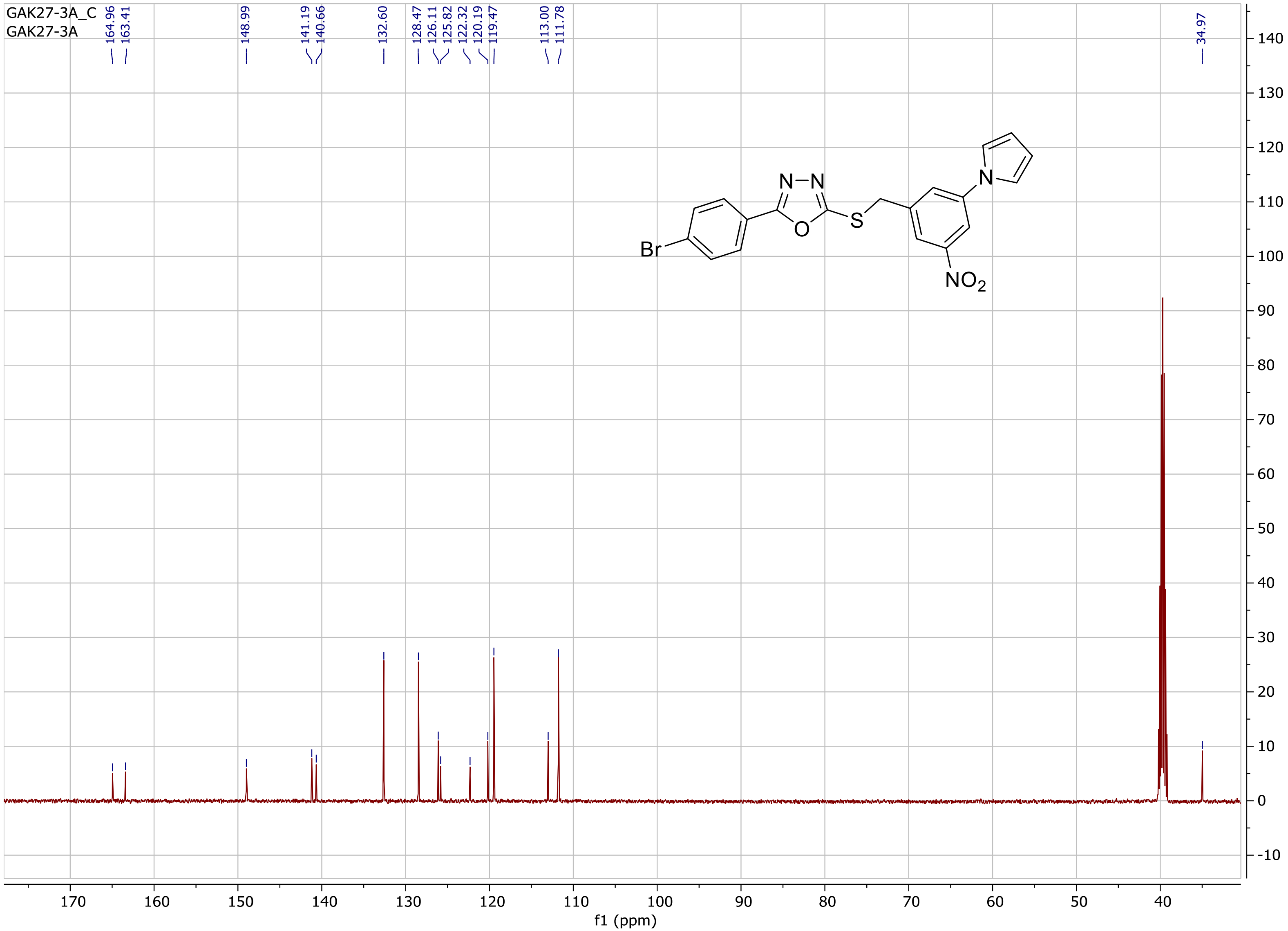

2-Cyclohexyl-5-((3-nitro-5-(1H-pyrrol-1-yl)benzyl)sulfanyl)-1,3,4-oxadiazole (**65e**): ^{1}H NMR (600 MHz, DMSO-d_6)

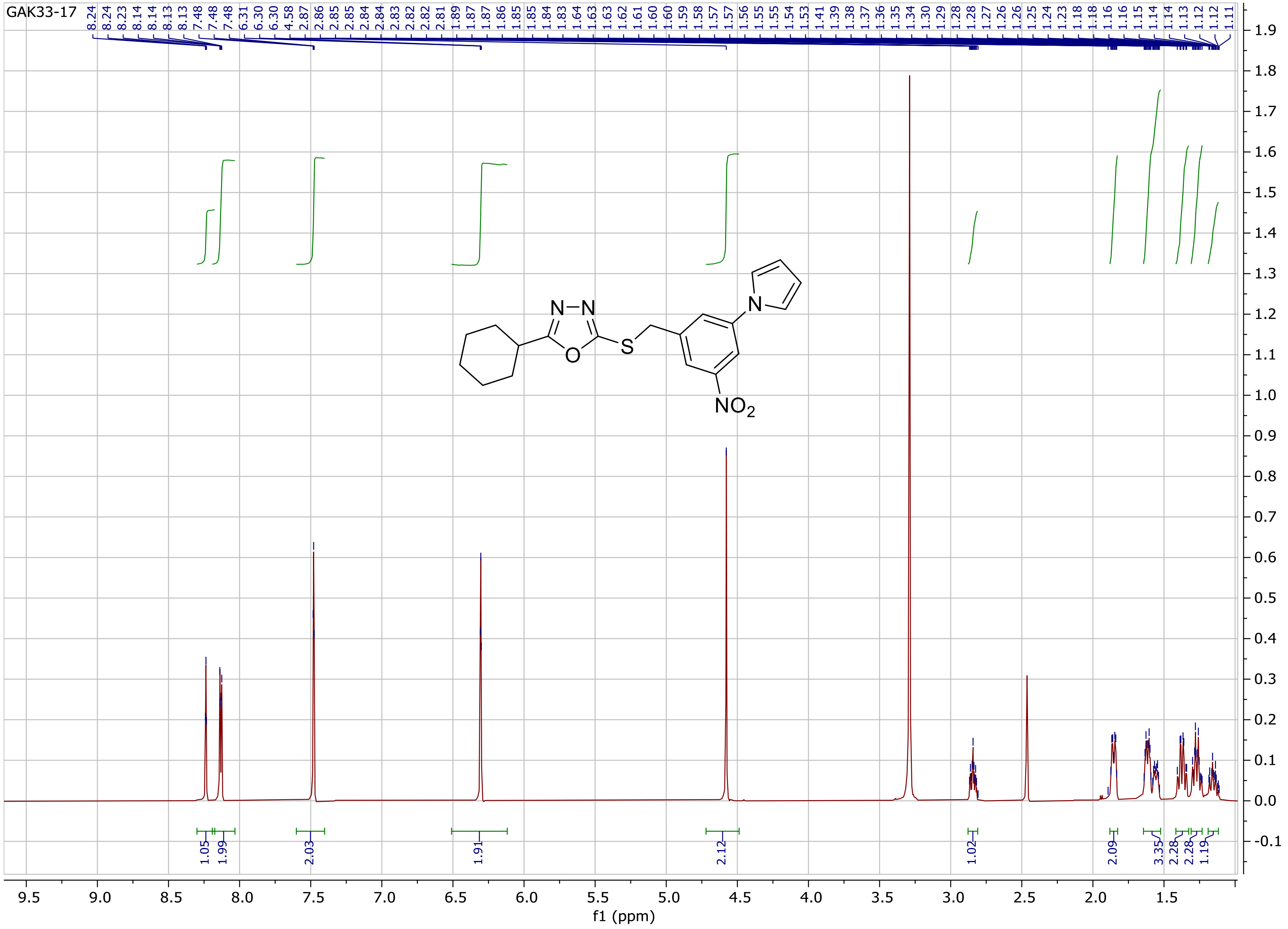

2-Cyclohexyl-5-((3-nitro-5-(1H-pyrrol-1-yl)benzyl)sulfanyl)-1,3,4-oxadiazole (**65e**): ^{13}C NMR (151 MHz, DMSO-d_6)

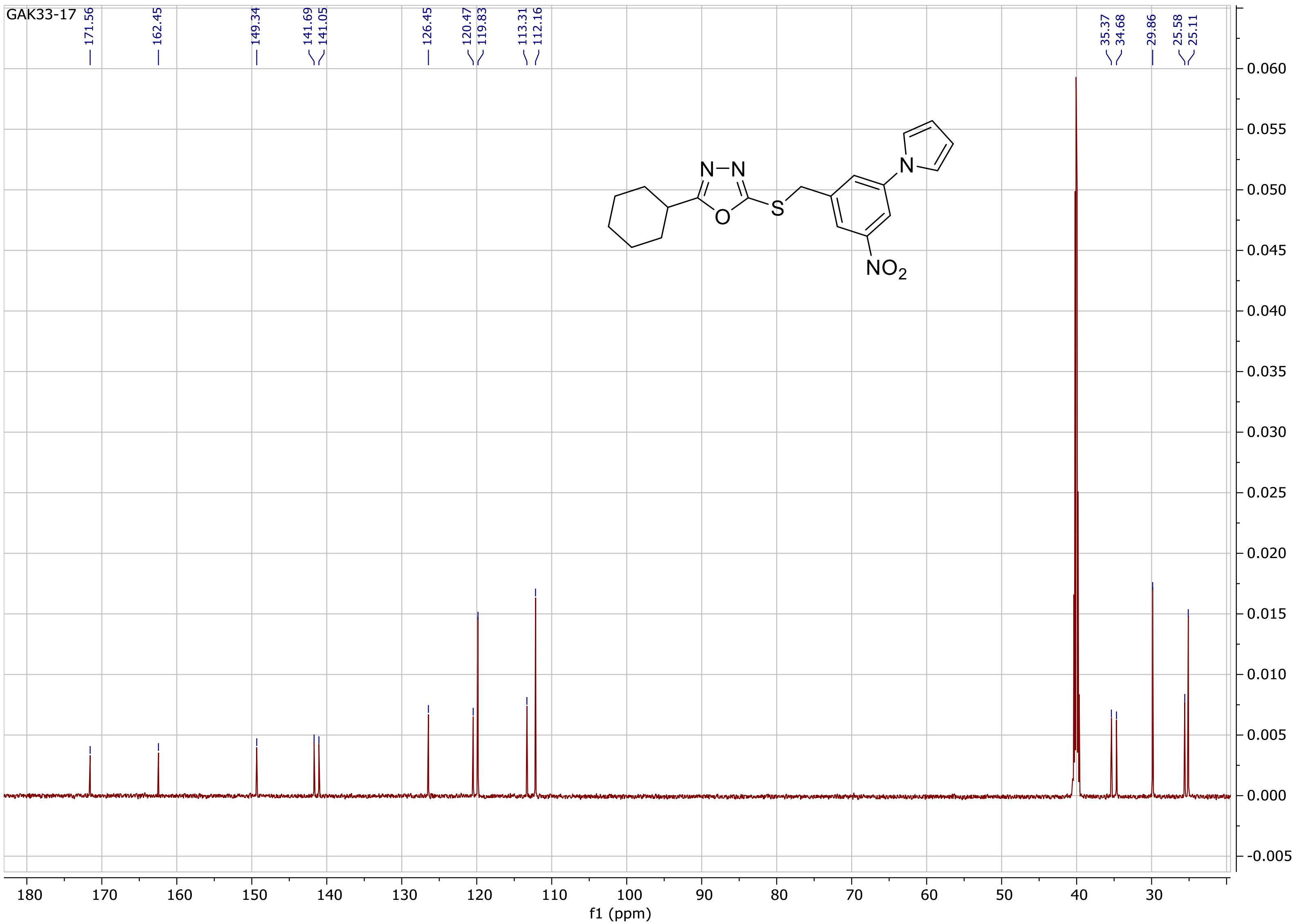

5-((3,4-Dinitrobenzyl)sulfanyl)-1-phenyl-1H-tetrazole (**66a**): ^{1}H NMR (600 MHz, DMSO-d_6)

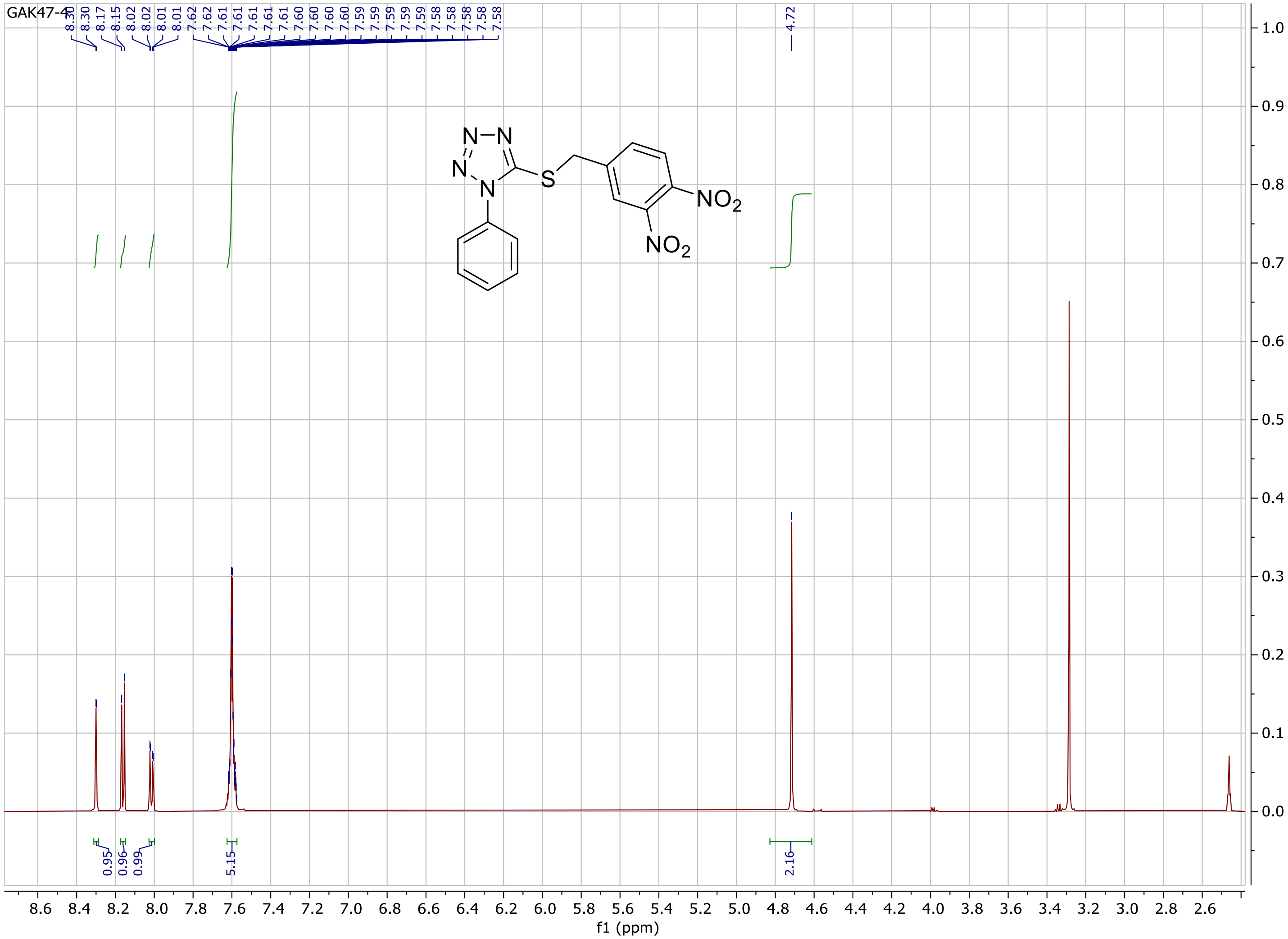

5-((3,4-Dinitrobenzyl)sulfanyl)-1-phenyl-1H-tetrazole (**66a**): ^{13}C NMR (151 MHz, DMSO-d_6)

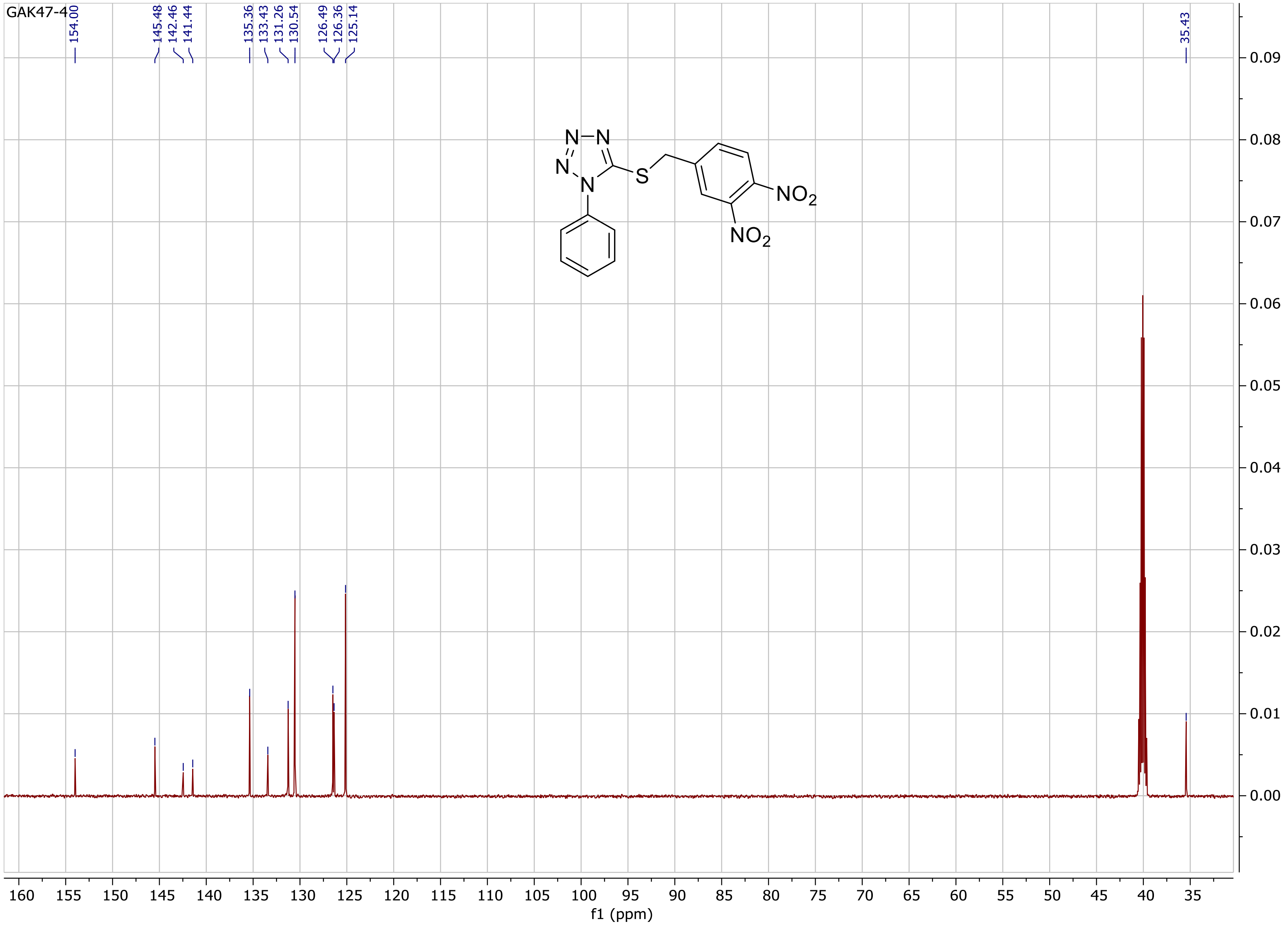

5-((3,4-Dinitrobenzyl)sulfanyl)-1-(4-methoxyphenyl)-1H-tetrazole (**66b**): ^{1}H NMR (600 MHz, DMSO-d_6)

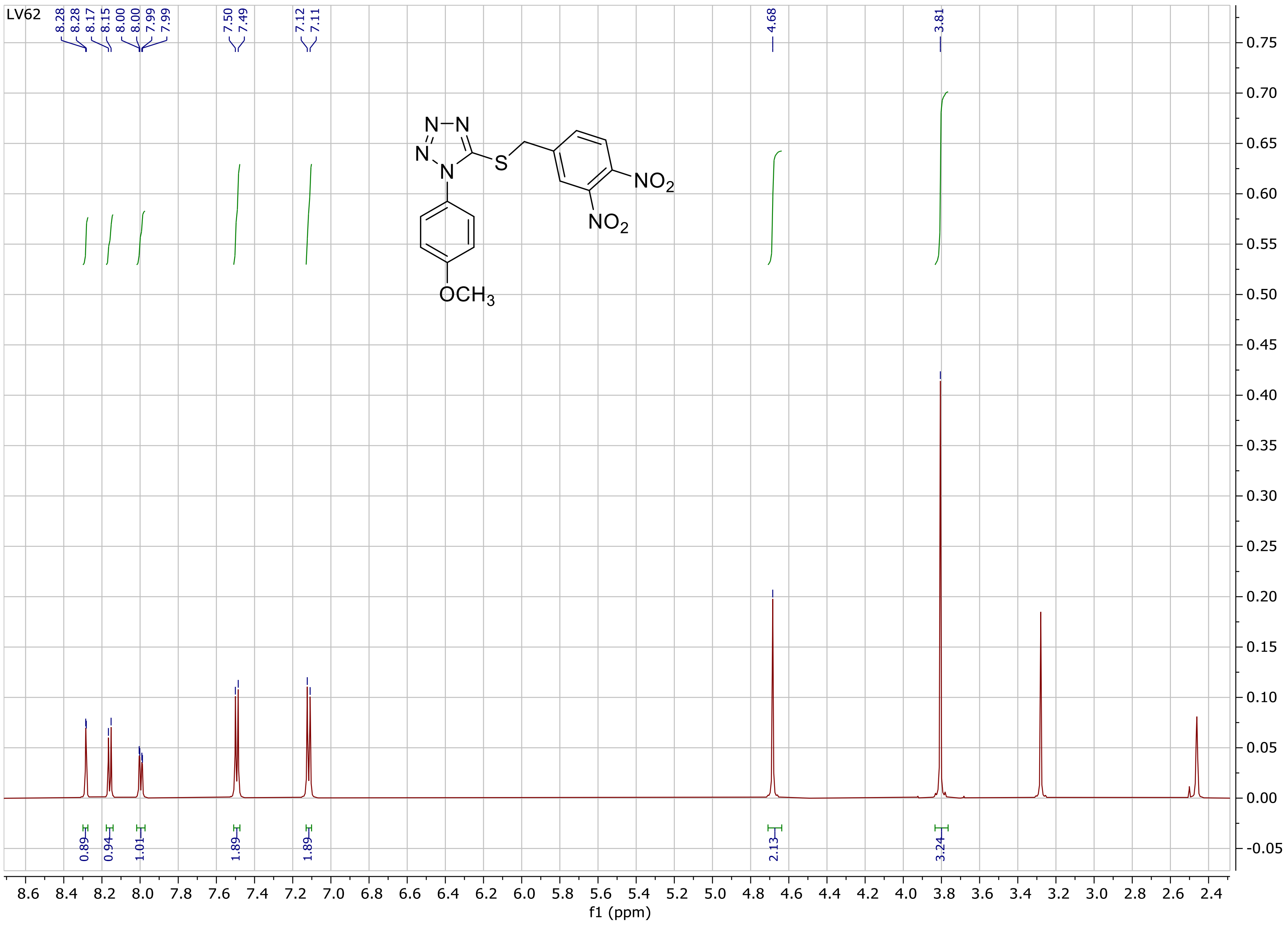

5-((3,4-Dinitrobenzyl)sulfanyl)-1-(4-methoxyphenyl)-1H-tetrazole (**66b**): ^{13}C NMR (151 MHz, DMSO-d_6)

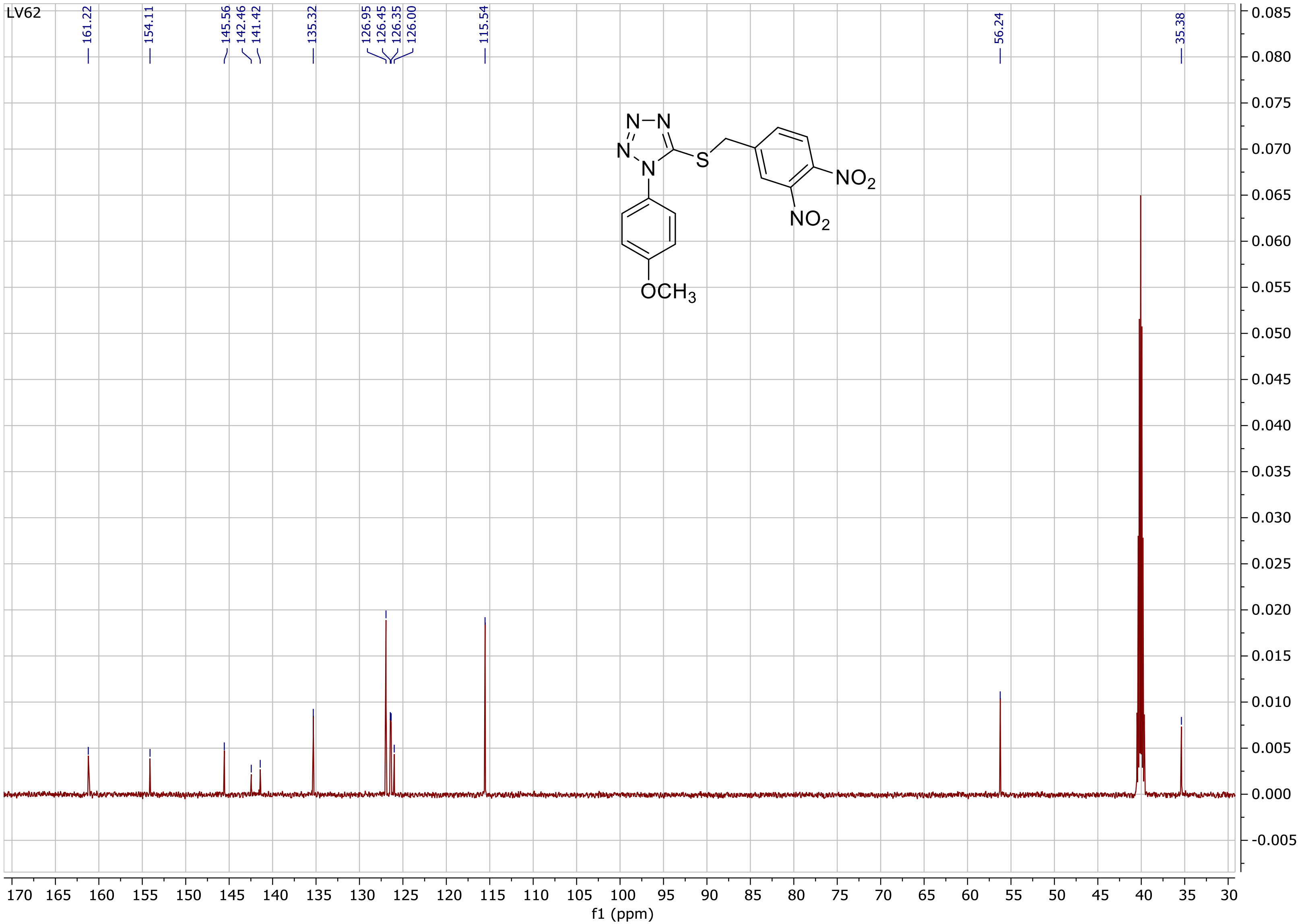

1-(4-Chlorophenyl)-5-((3,4-dinitrobenzyl)sulfanyl)-1H-tetrazole (**66c**): ^{1}H NMR (600 MHz, DMSO-d_6)

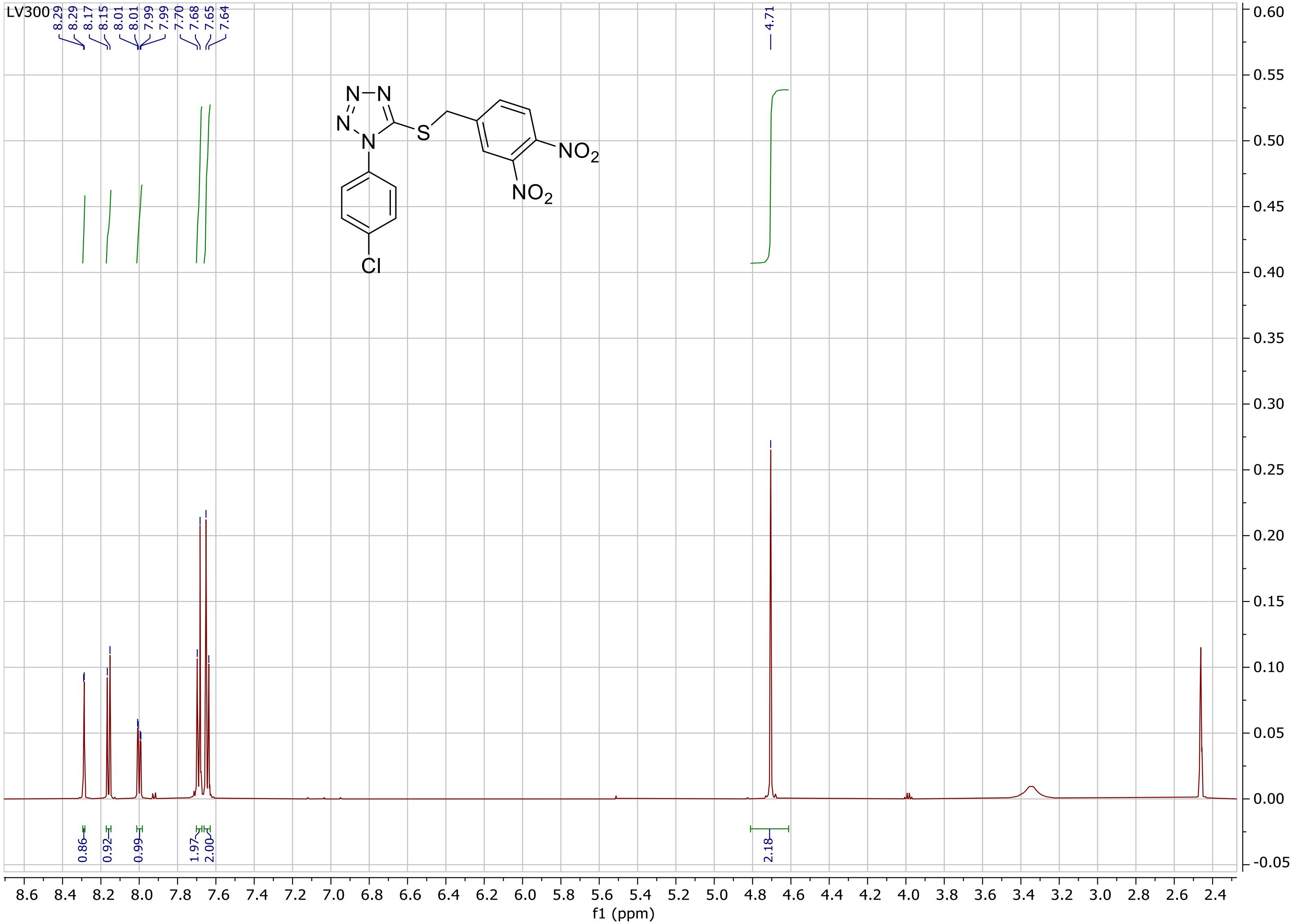

1-(4-Chlorophenyl)-5-((3,4-dinitrobenzyl)sulfanyl)-1H-tetrazole (**66c**): ^{13}C NMR (151 MHz, DMSO-d_6)

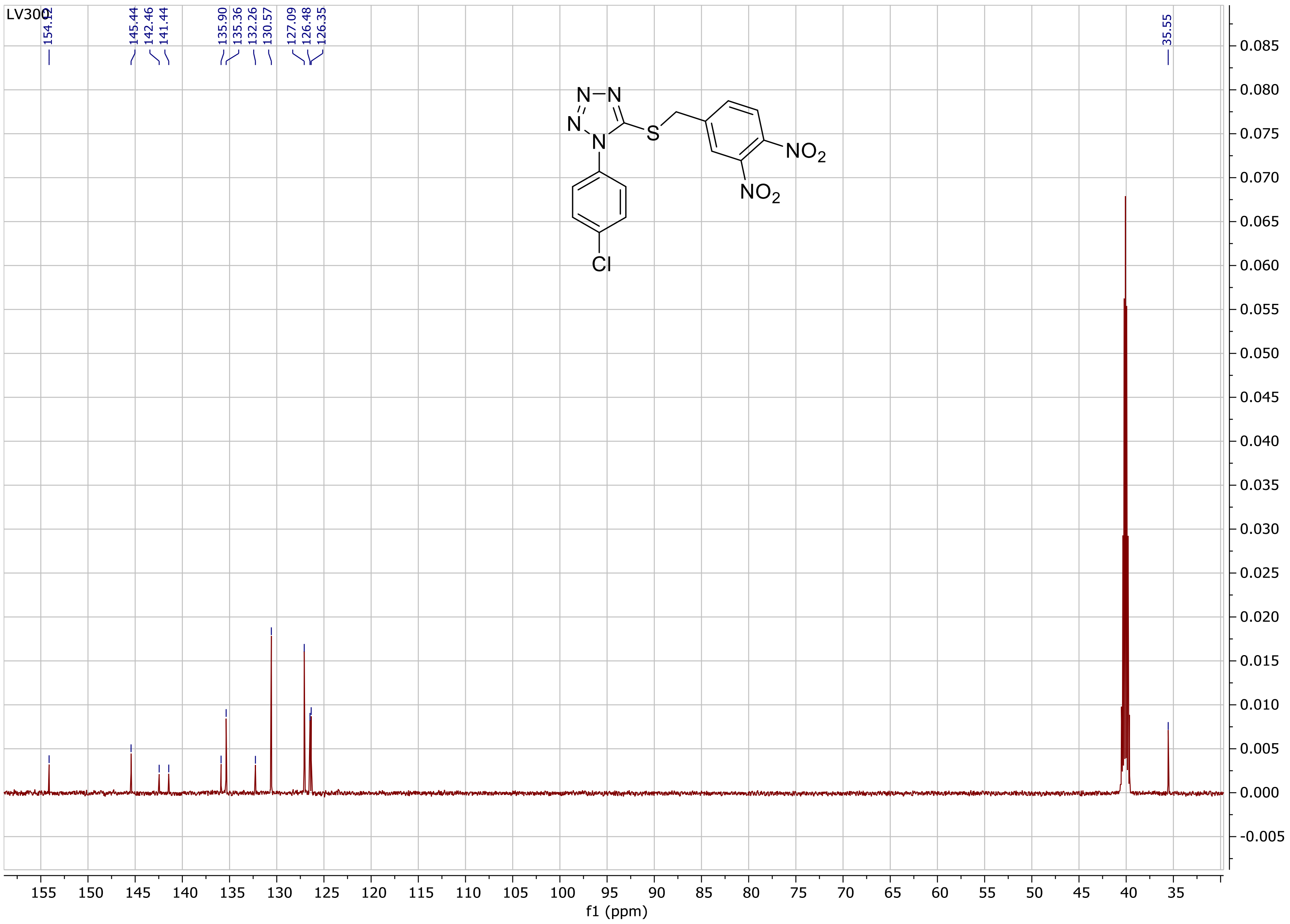

1-(4-Bromophenyl)-5-((3,4-dinitrobenzyl)sulfanyl)-1H-tetrazole (**66d**): ^{1}H NMR (600 MHz, DMSO-d_6)

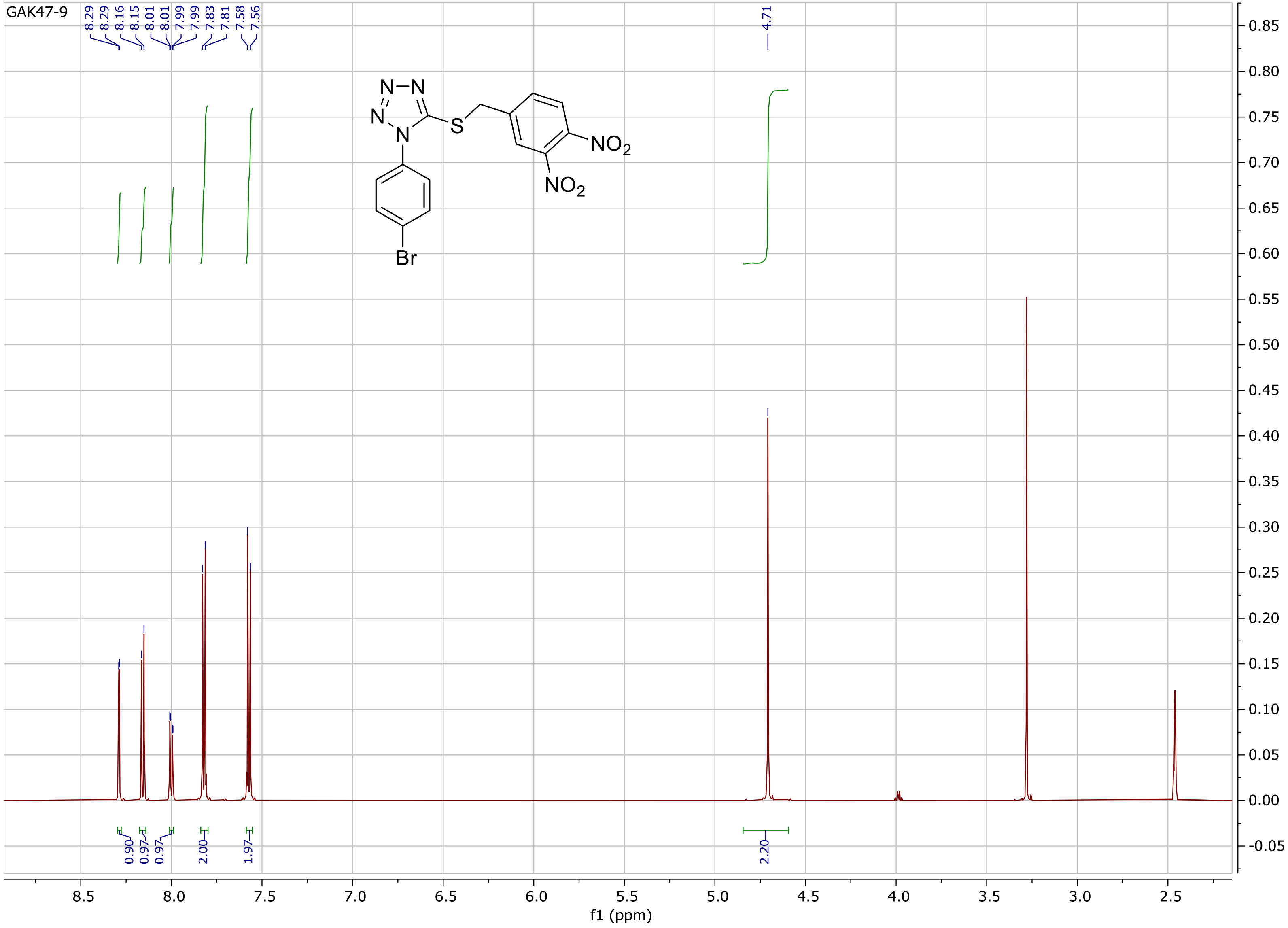

1-(4-Bromophenyl)-5-((3,4-dinitrobenzyl)sulfanyl)-1H-tetrazole (**66d**): ^{13}C NMR (151 MHz, DMSO-d_6)

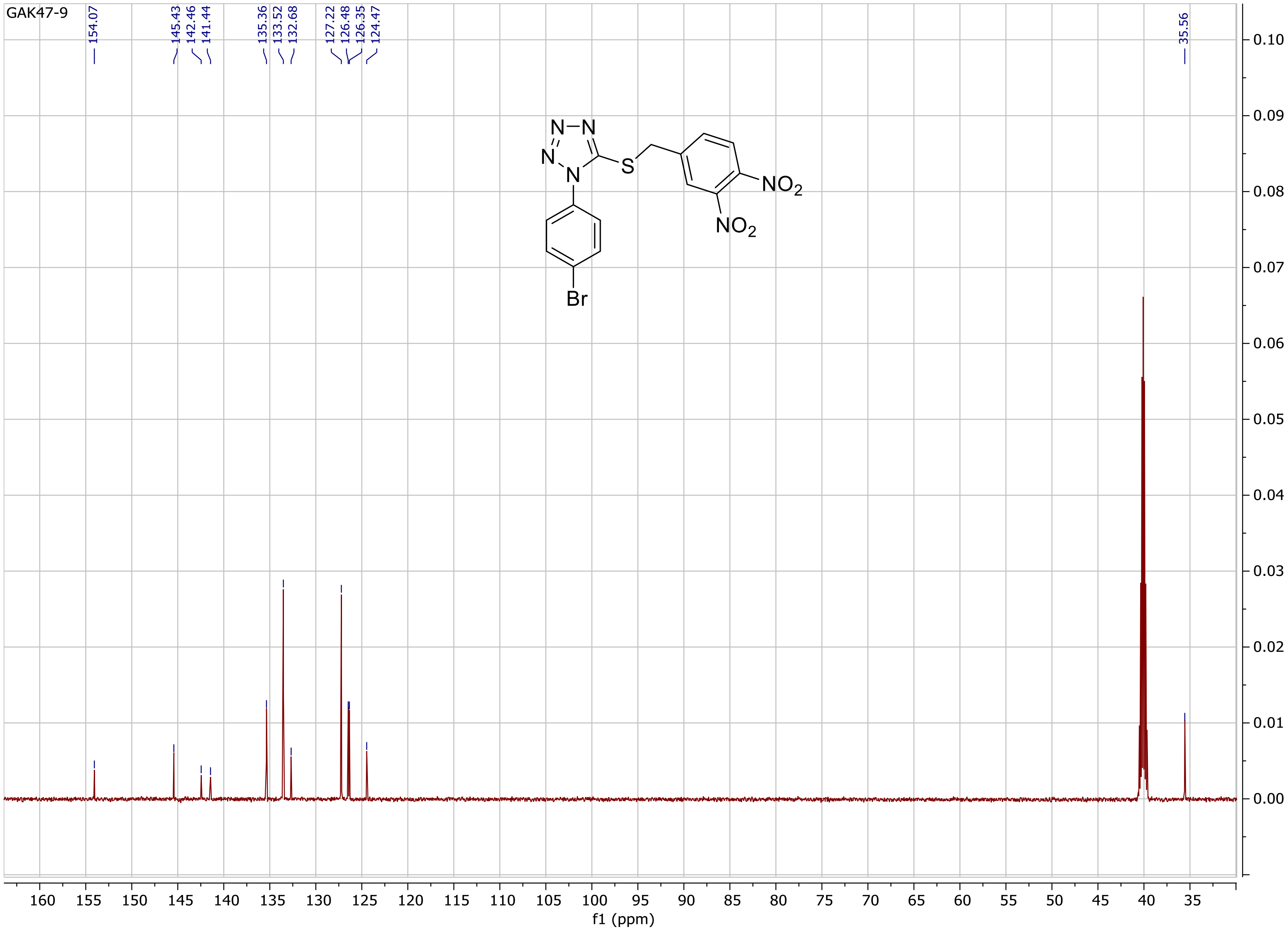

1-Cyclohexyl-5-((3,4-dinitrobenzyl)sulfanyl)-1H-tetrazole (**66e**): ^{1}H NMR (600 MHz, DMSO-d_6)

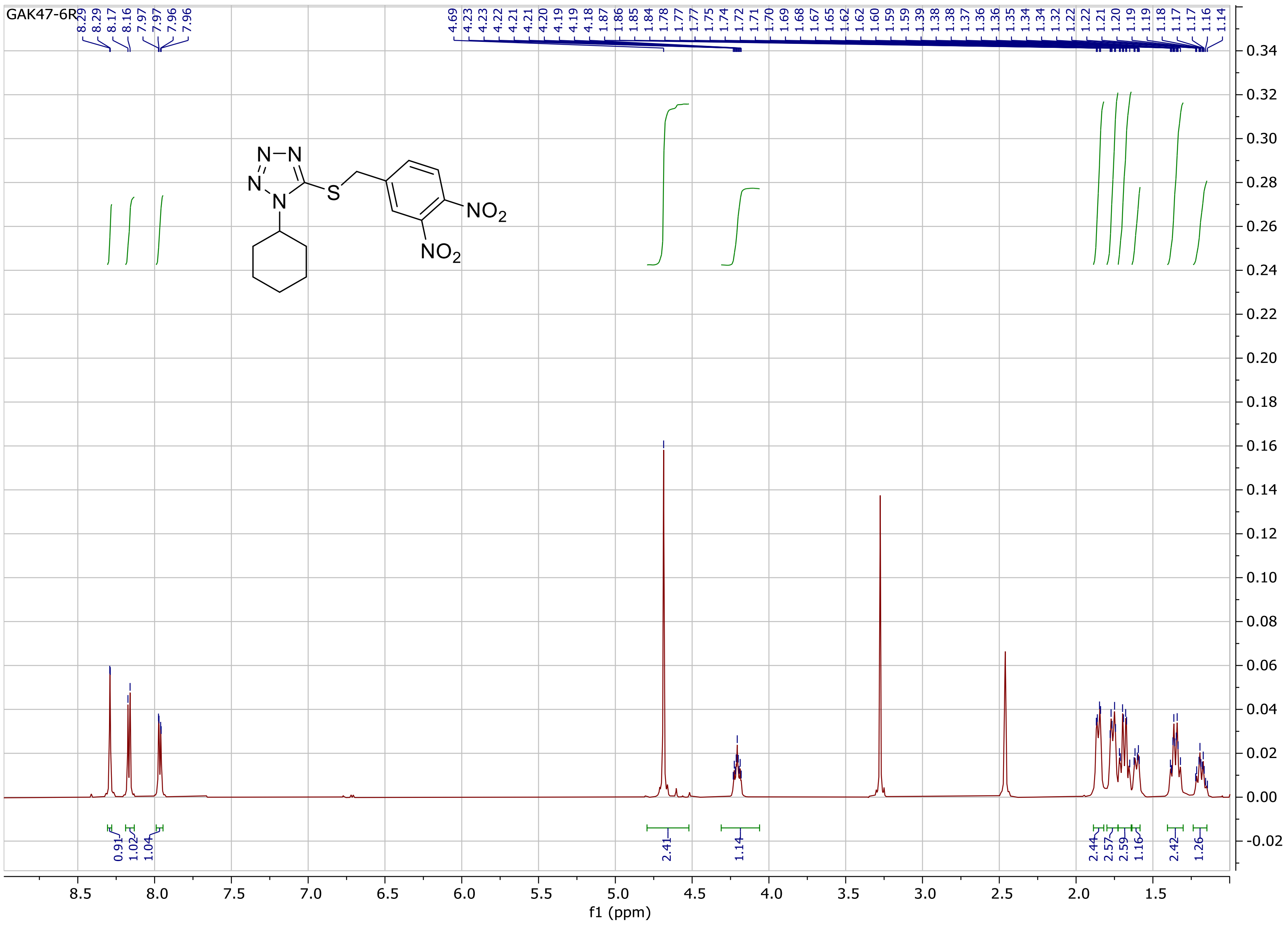

1-Cyclohexyl-5-((3,4-dinitrobenzyl)sulfanyl)-1H-tetrazole (**66e**): ^{13}C NMR (151 MHz, DMSO-d_6)

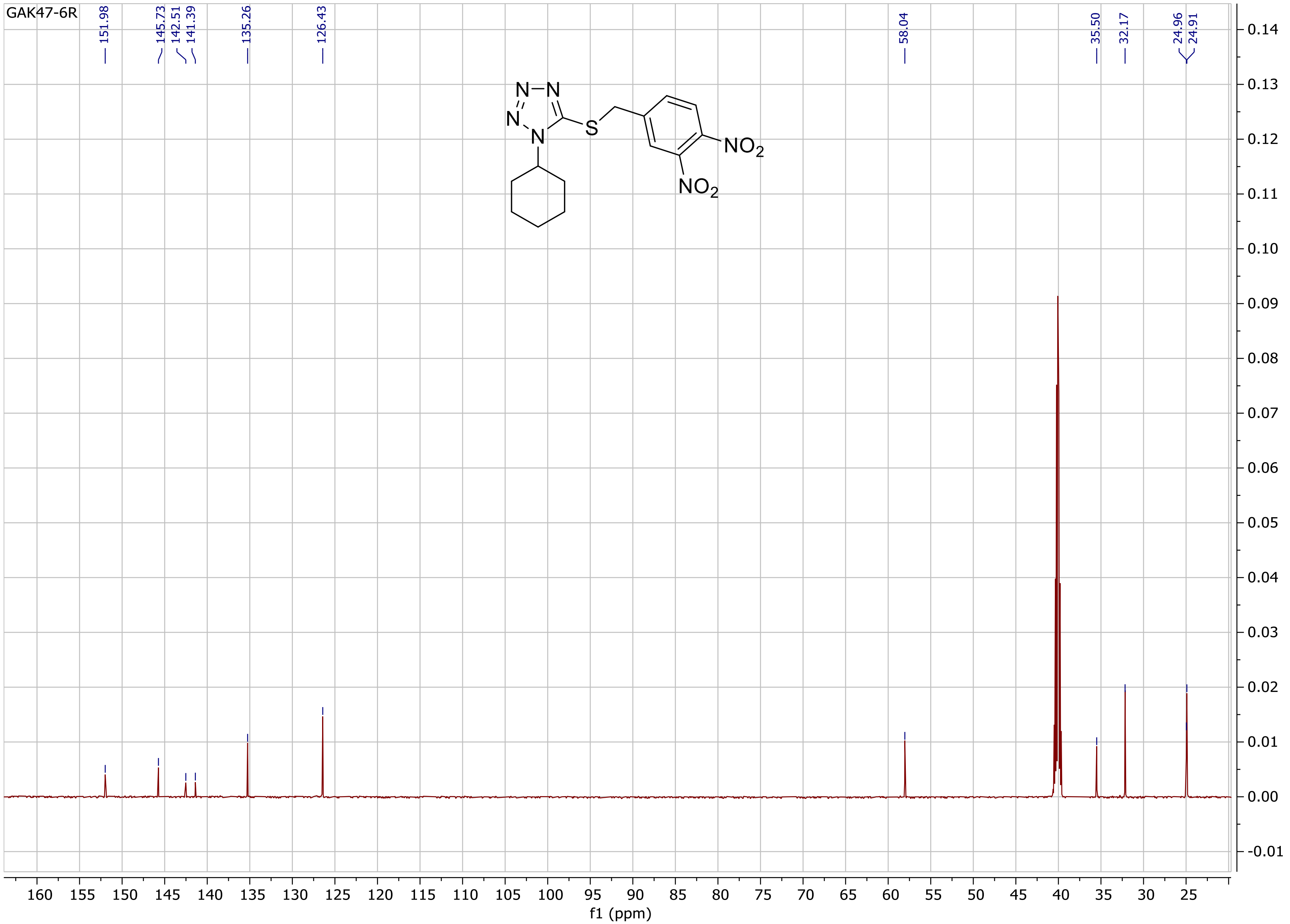

5-((2,5-Dinitrobenzyl)sulfanyl)-1-phenyl-1H-tetrazole (**67a**): ^{1}H NMR (500 MHz, DMSO-d_6)

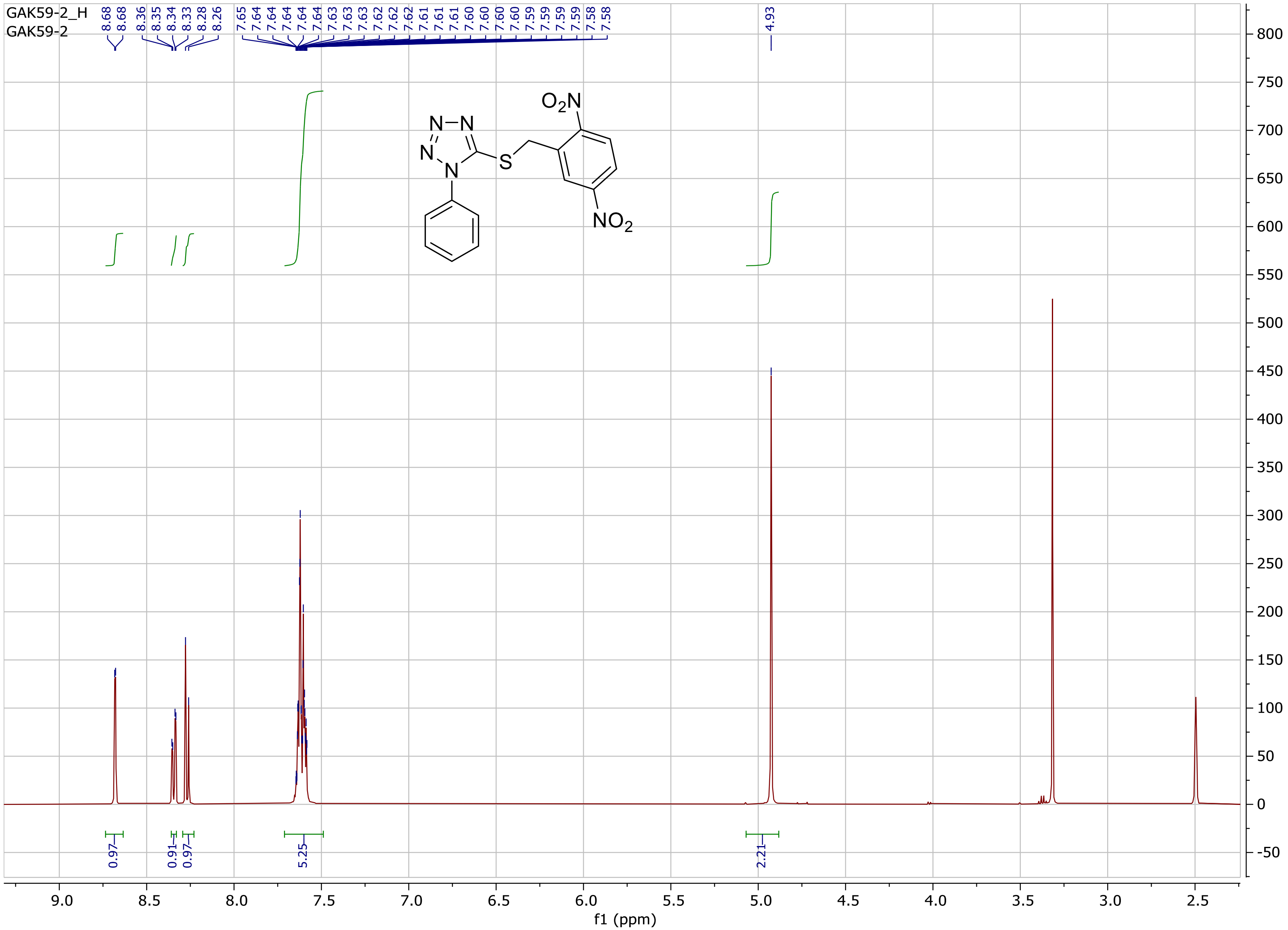

5-((2,5-Dinitrobenzyl)sulfanyl)-1-phenyl-1H-tetrazole (**67a**): ^{13}C NMR (126 MHz, DMSO-d_6)

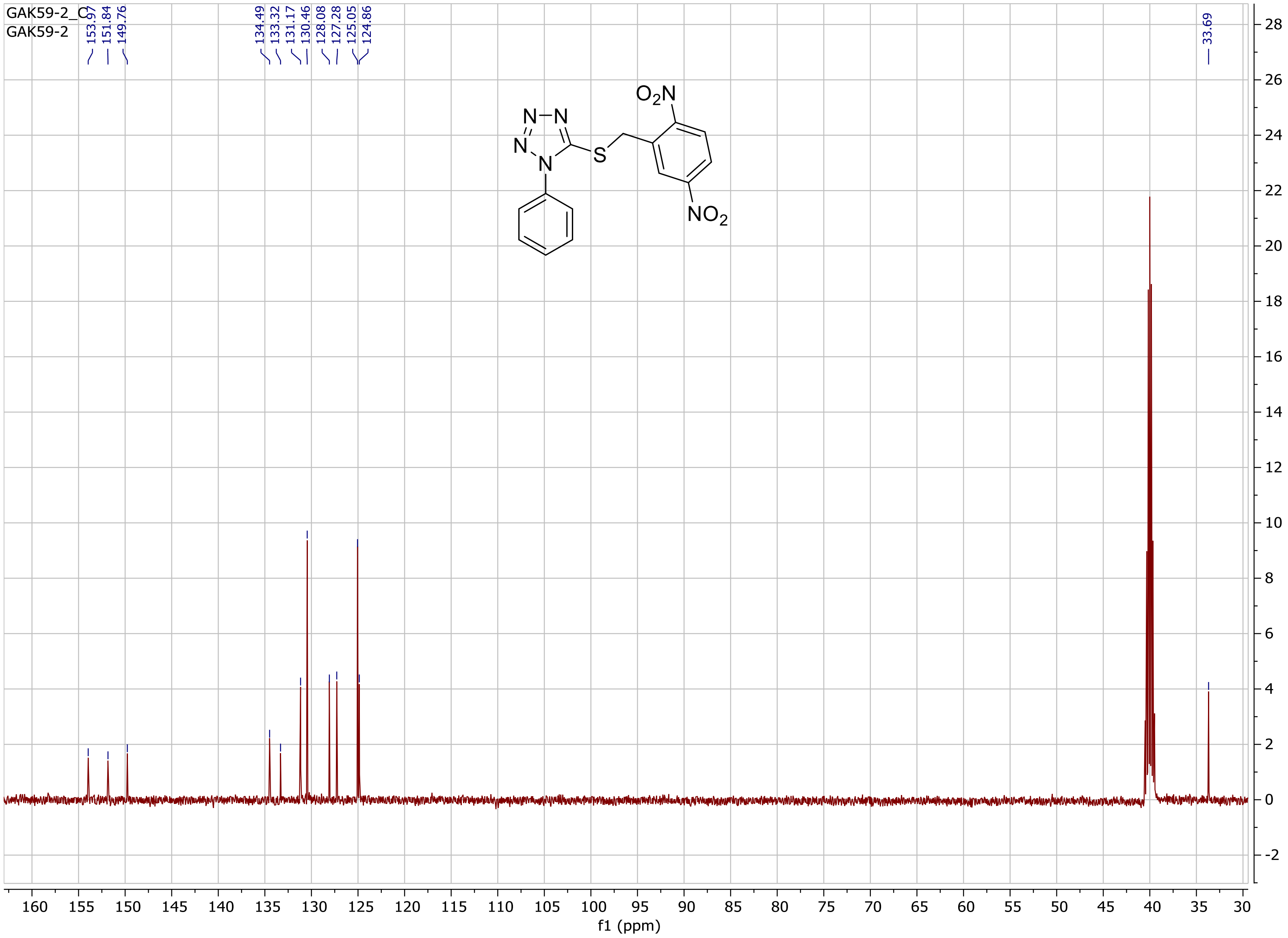

5-((2,5-Dinitrobenzyl)sulfanyl)-1-phenyl-1H-tetrazole (**67a**): HPLC trace

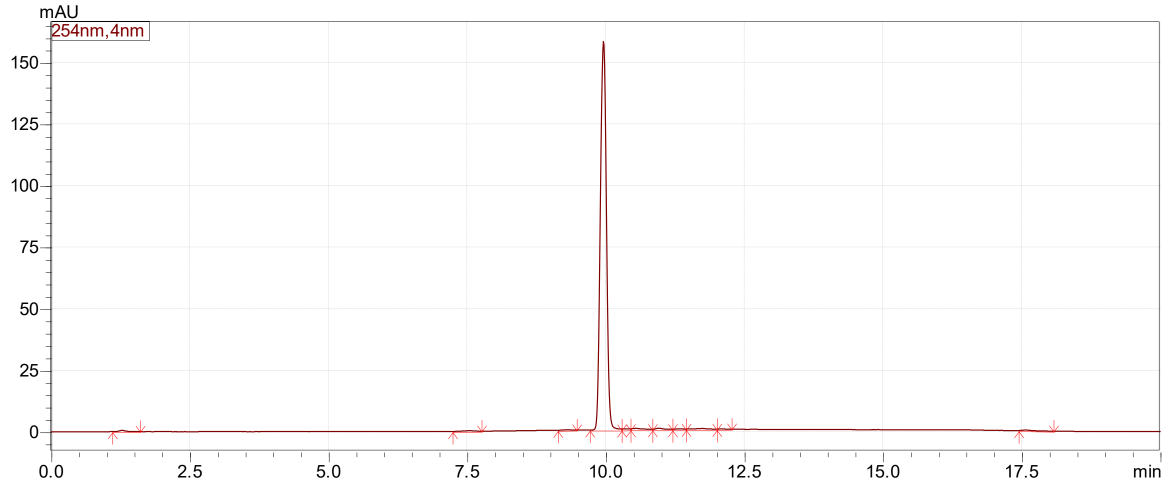

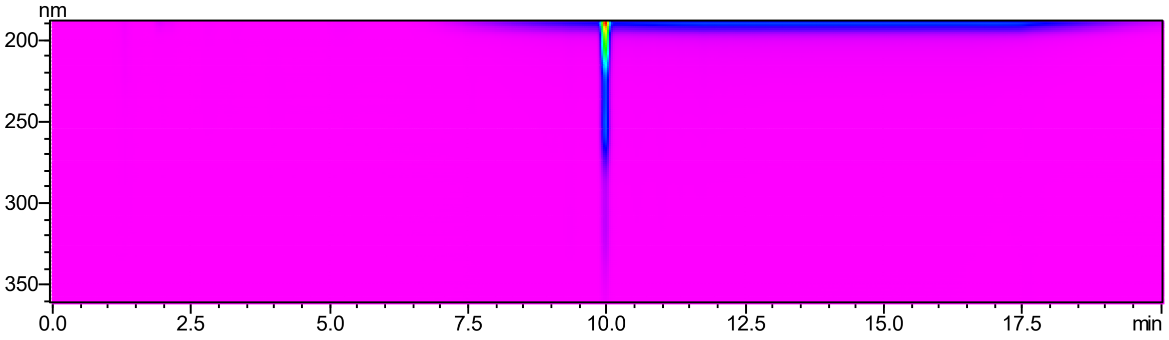

Main peak retention time: 9.965 min with 96.275 % of area under the peak at 254 nm.

5-(2,5-Dinitrobenzyl)sulfanyl)-1-(4-methoxyphenyl)-1H-tetrazole (**67b**): ^{1}H NMR (500 MHz, DMSO-d_6)

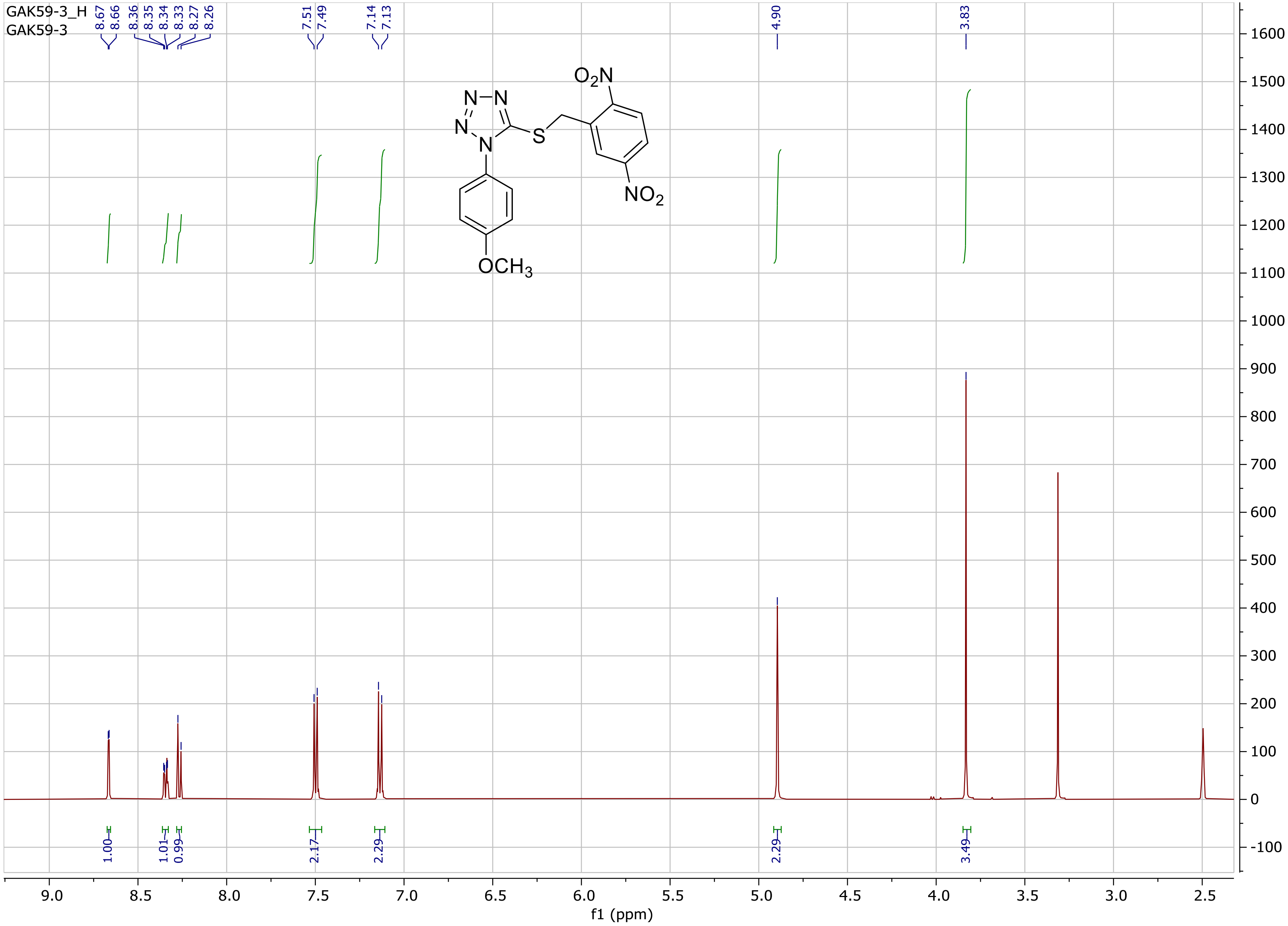

5-(2,5-Dinitrobenzyl)sulfanyl)-1-(4-methoxyphenyl)-1H-tetrazole (**67b**): ^{13}C NMR (126 MHz, DMSO-d_6)

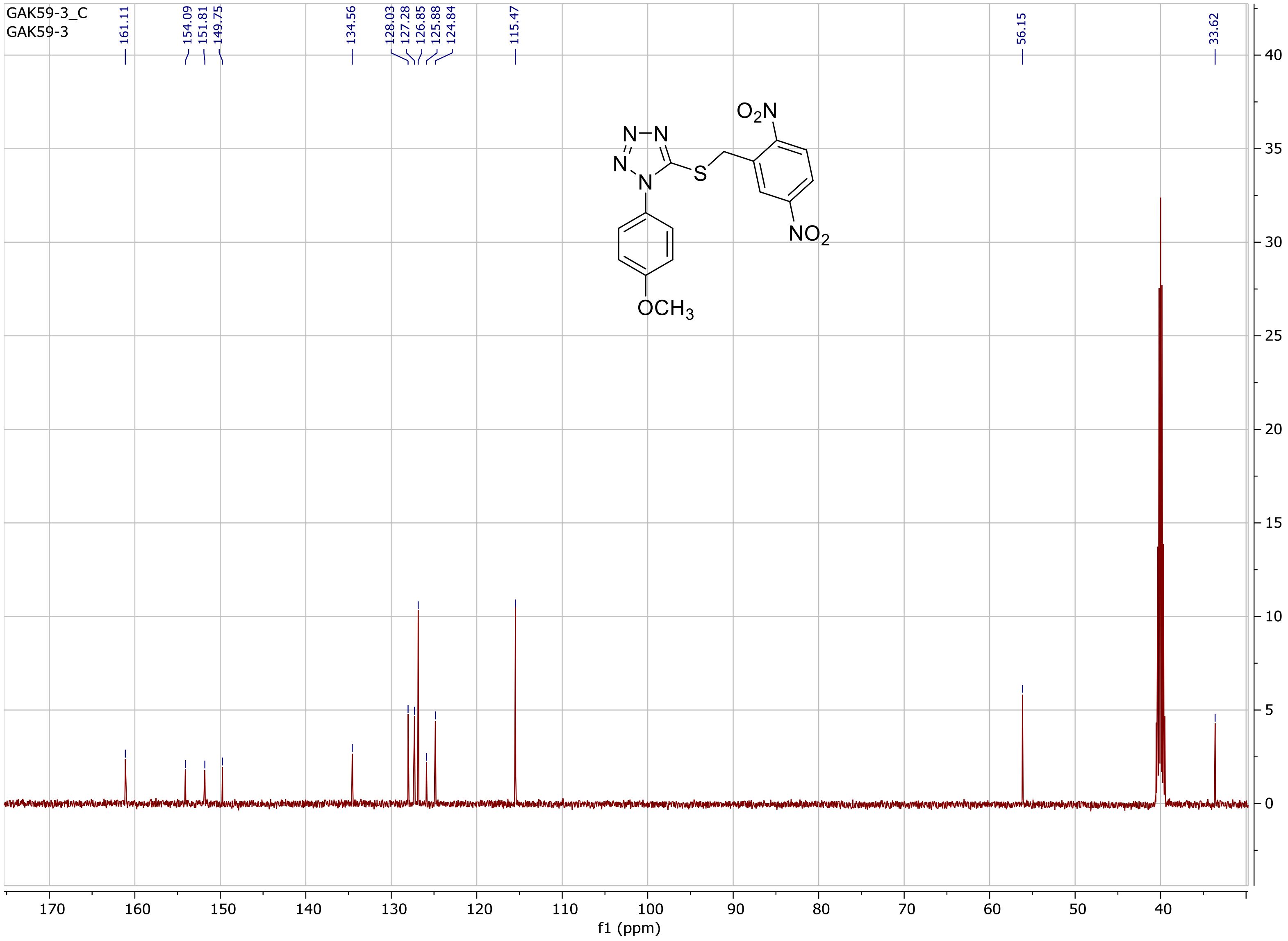

5-(2,5-Dinitrobenzyl)sulfanyl)-1-(4-methoxyphenyl)-1H-tetrazole (**67b**): HPLC trace

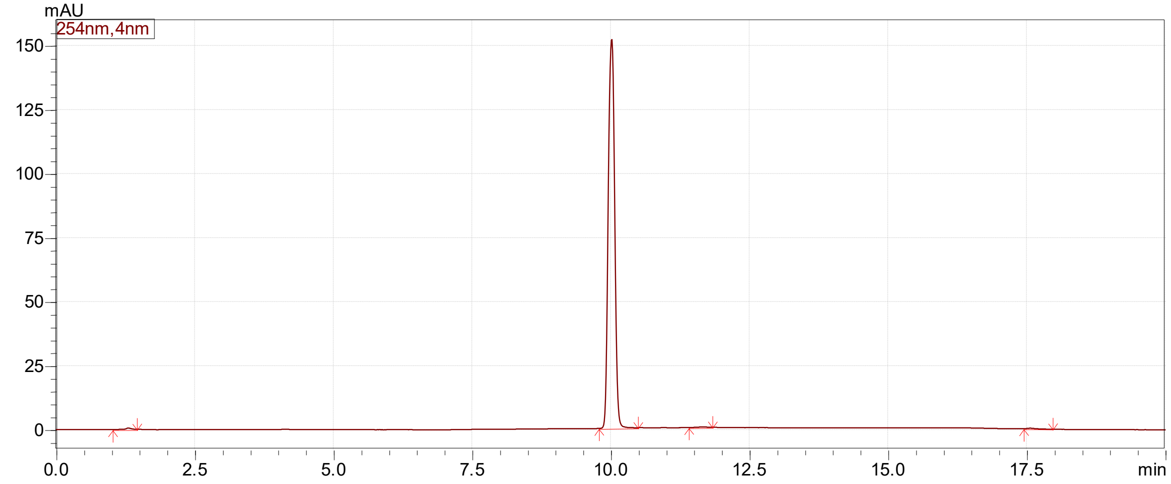

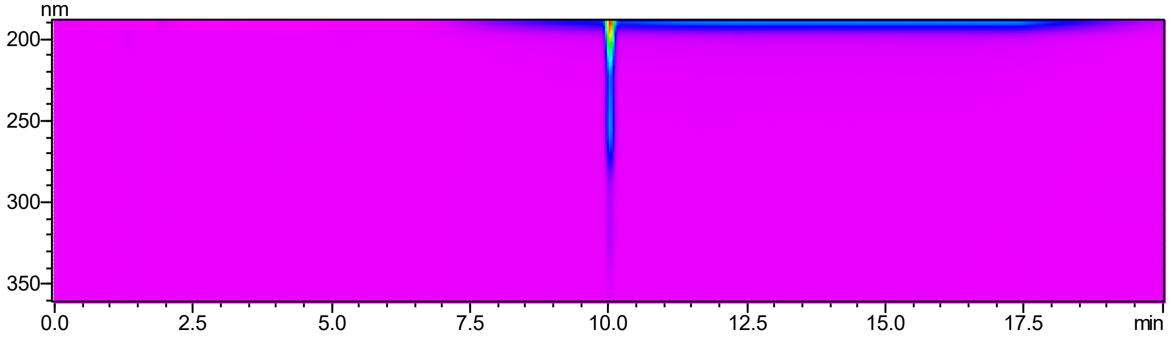

Main peak retention time: 10.022 min with 99.173 % of area under the peak at 254 nm.

1-(4-Chlorophenyl)-5-((2,5-dinitrobenzyl)sulfanyl)-1H-tetrazole (**67c**): ^{1}H NMR (500 MHz, DMSO-d_6)

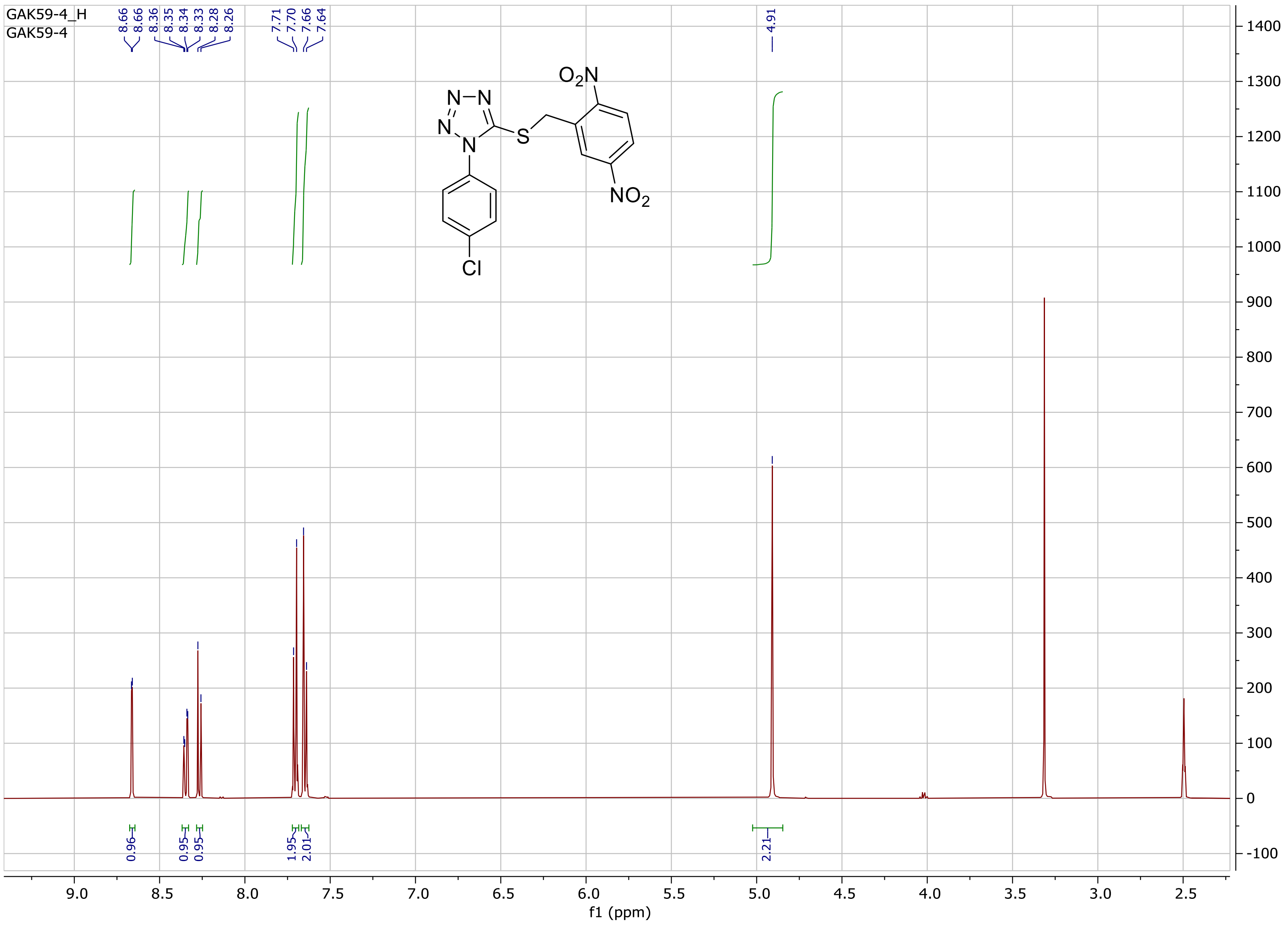

1-(4-Chlorophenyl)-5-((2,5-dinitrobenzyl)sulfanyl)-1H-tetrazole (**67c**): ^{13}C NMR (126 MHz, DMSO-d_6)

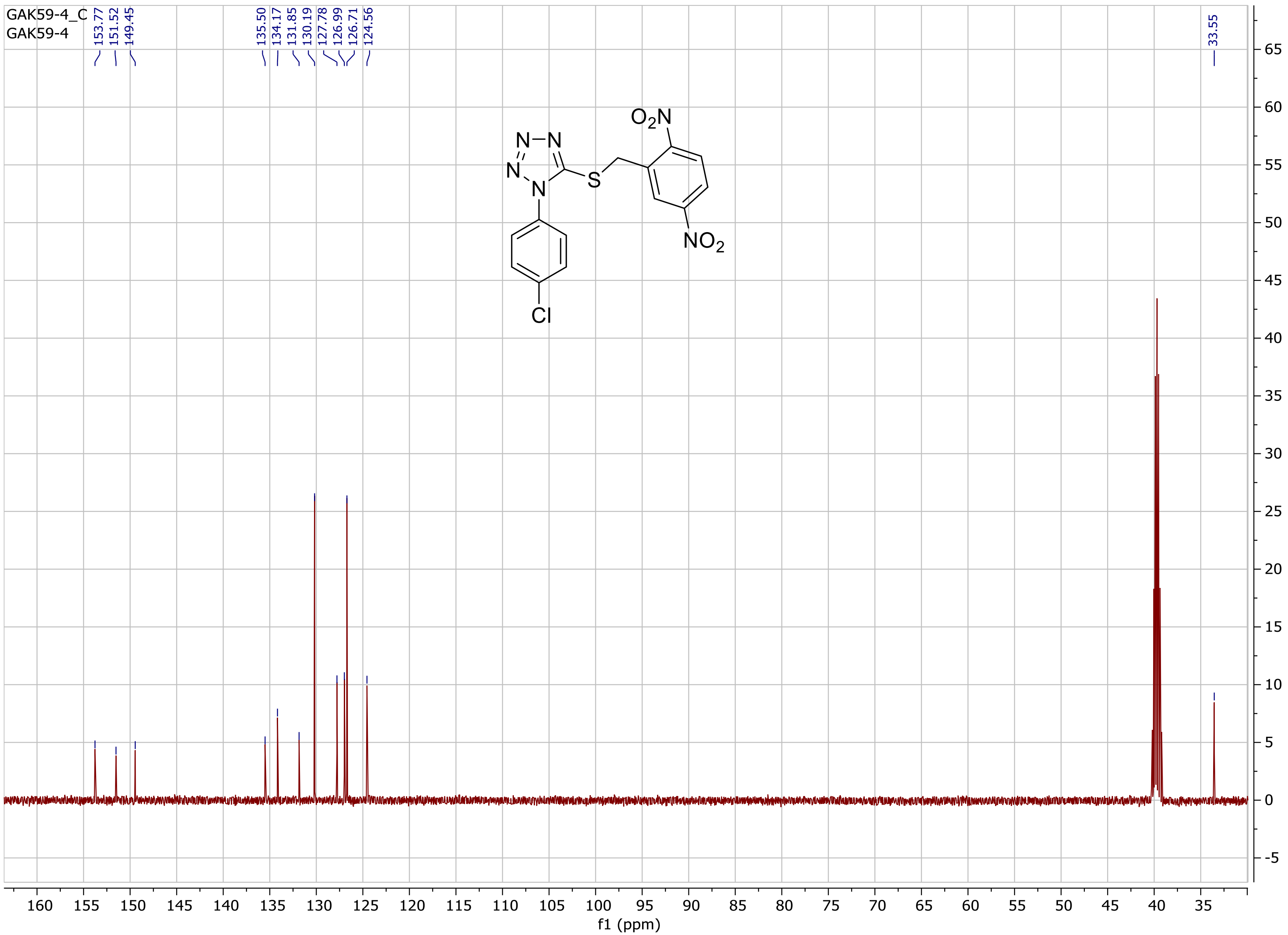

1-(4-Chlorophenyl)-5-((2,5-dinitrobenzyl)sulfanyl)-1H-tetrazole (**67c**): HPLC trace

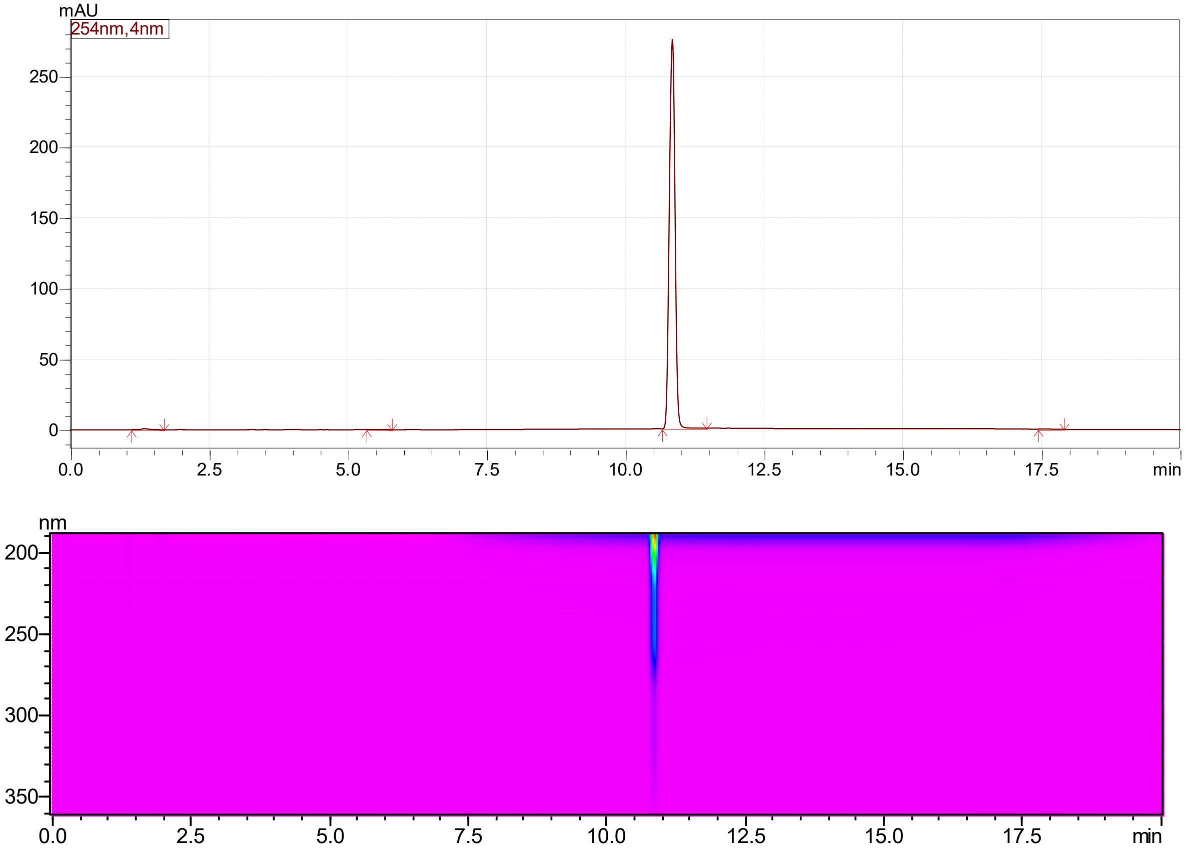

Main peak retention time: 10.850 min with 99.408 % of area under the peak at 254 nm.

1-(4-Bromophenyl)-5-((2,5-dinitrobenzyl)sulfanyl)-1H-tetrazole (**67d**): ^{1}H NMR (600 MHz, DMSO-d_6)

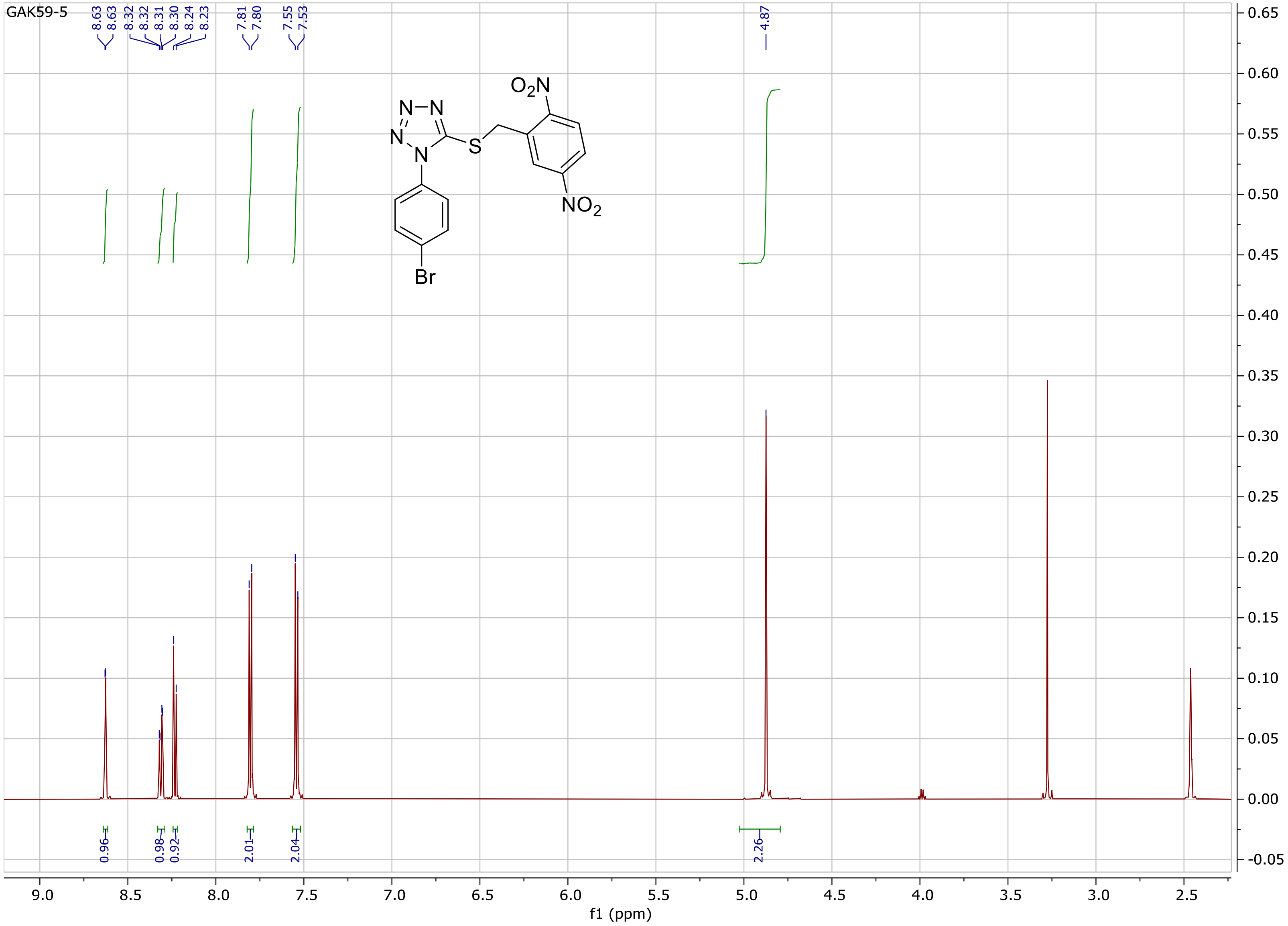

1-(4-Bromophenyl)-5-((2,5-dinitrobenzyl)sulfanyl)-1H-tetrazole (**67d**): ^{13}C NMR (151 MHz, DMSO-d_6)

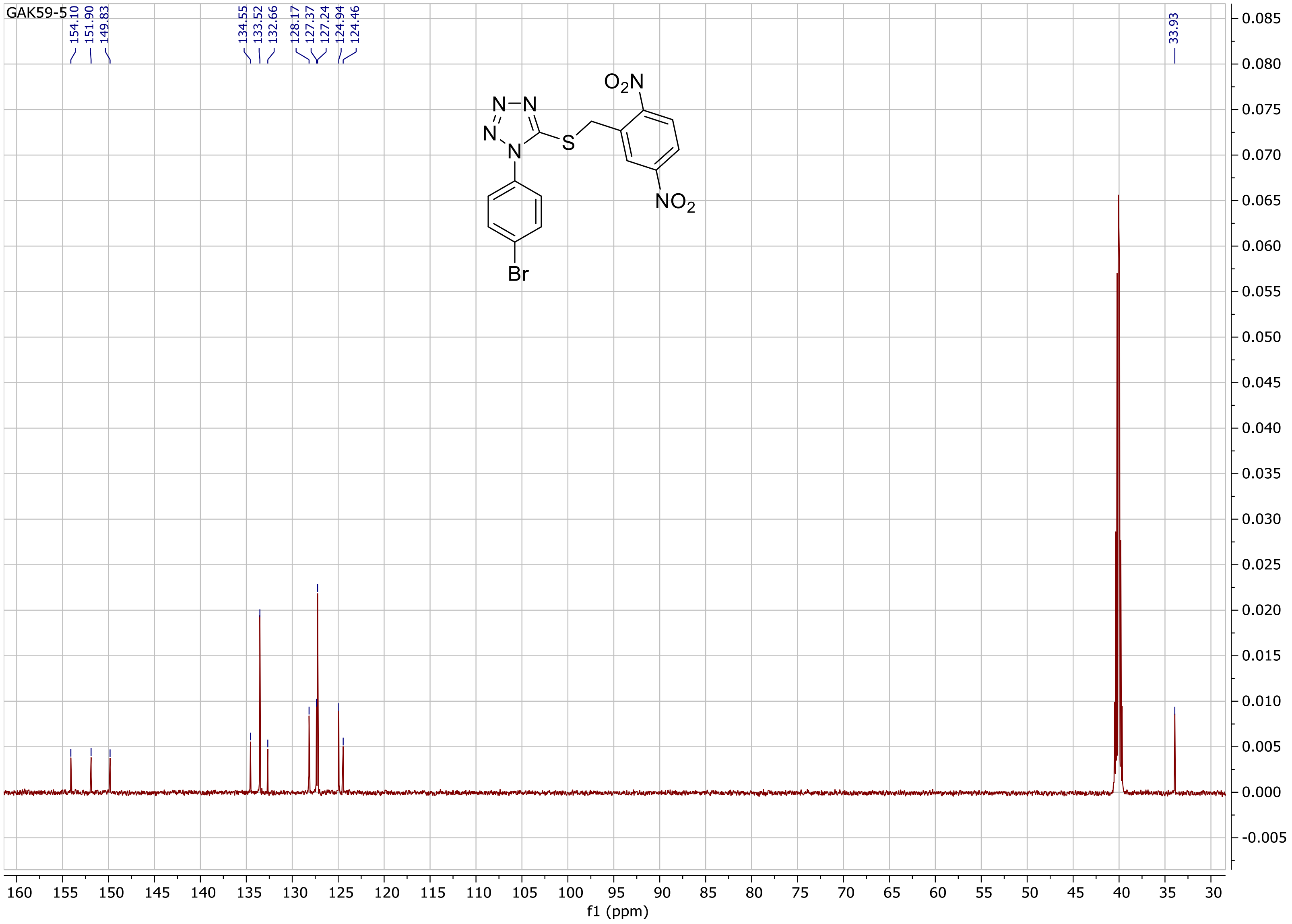

1-(4-Bromophenyl)-5-((2,5-dinitrobenzyl)sulfanyl)-1H-tetrazole (**67d**): HPLC trace

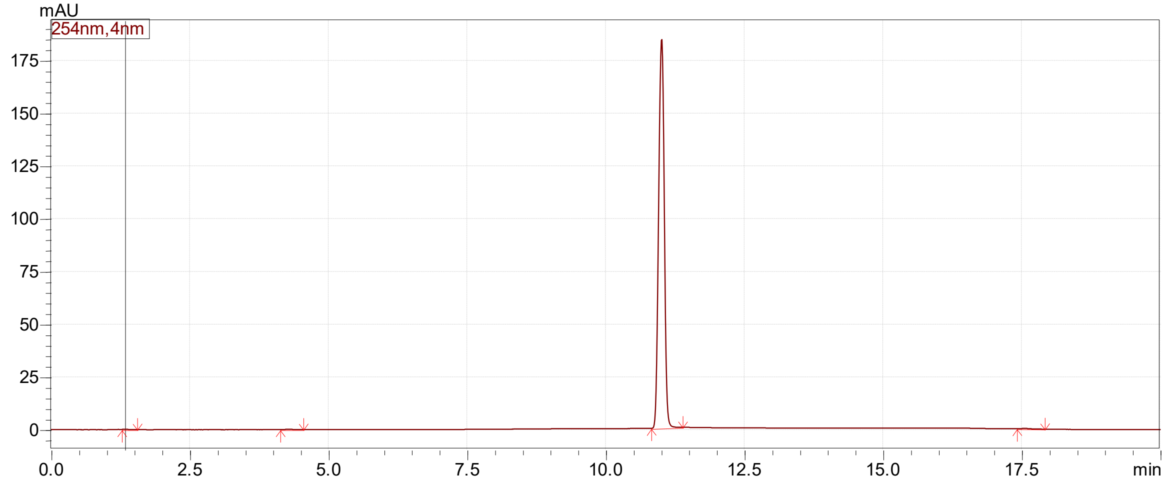

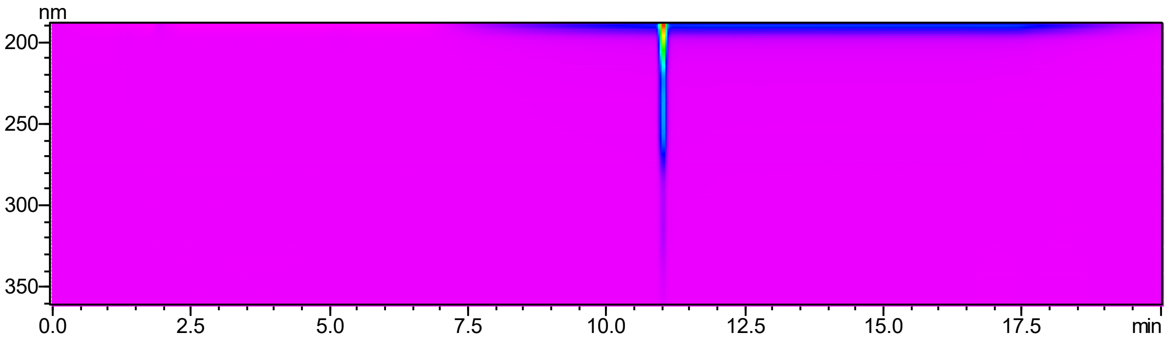

Main peak retention time: 11.014 min with 99.556 % of area under the peak at 254 nm.

1-Cyclohexyl-5-((2,5-dinitrobenzyl)sulfanyl)-1H-tetrazole (**67e**): ^{1}H NMR (600 MHz, DMSO-d_6)

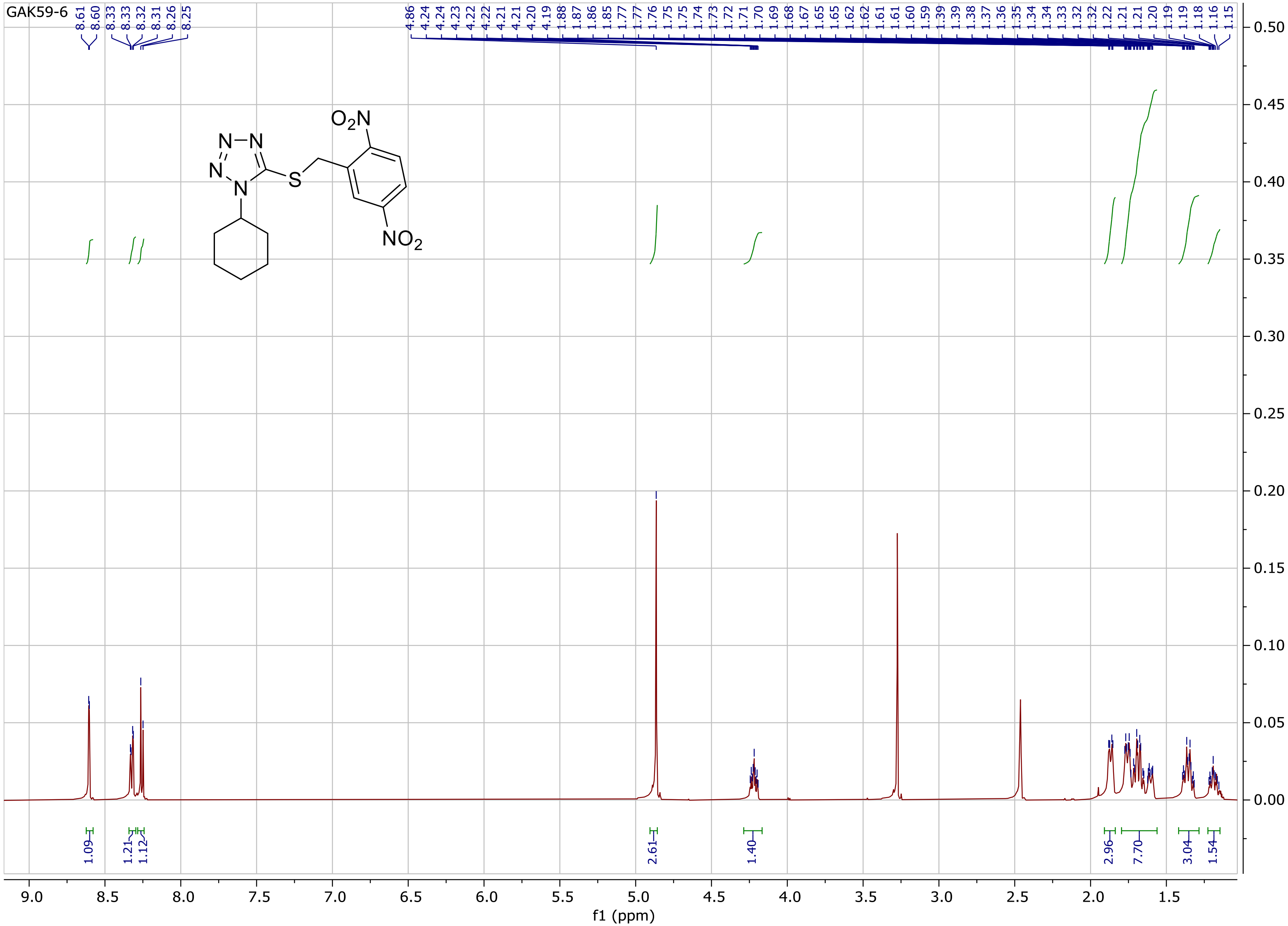

1-Cyclohexyl-5-((2,5-dinitrobenzyl)sulfanyl)-1H-tetrazole (**67e**): ^{13}C NMR (151 MHz, DMSO-d_6)

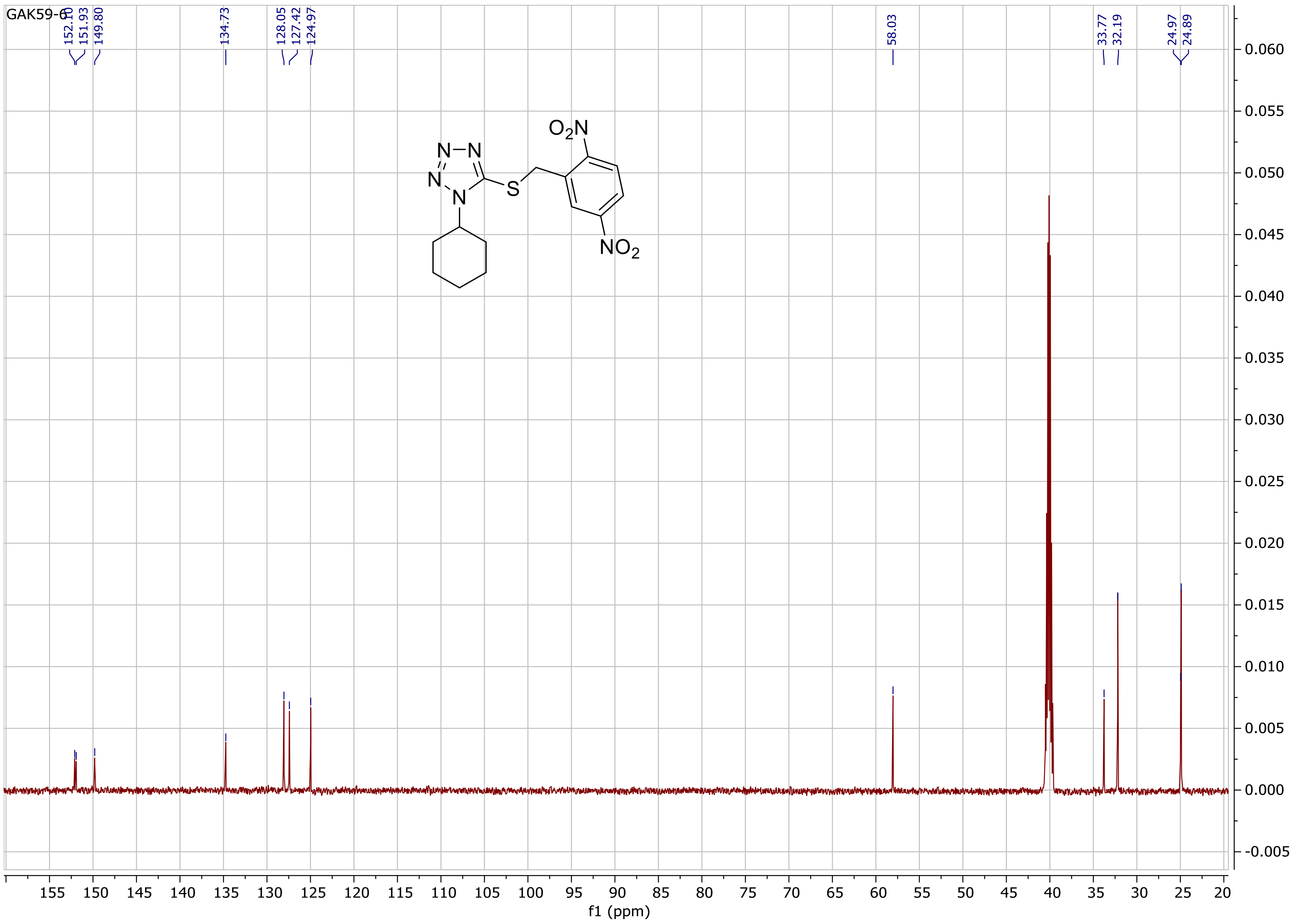

1-Cyclohexyl-5-((2,5-dinitrobenzyl)sulfanyl)-1H-tetrazole (**67e**): HPLC trace

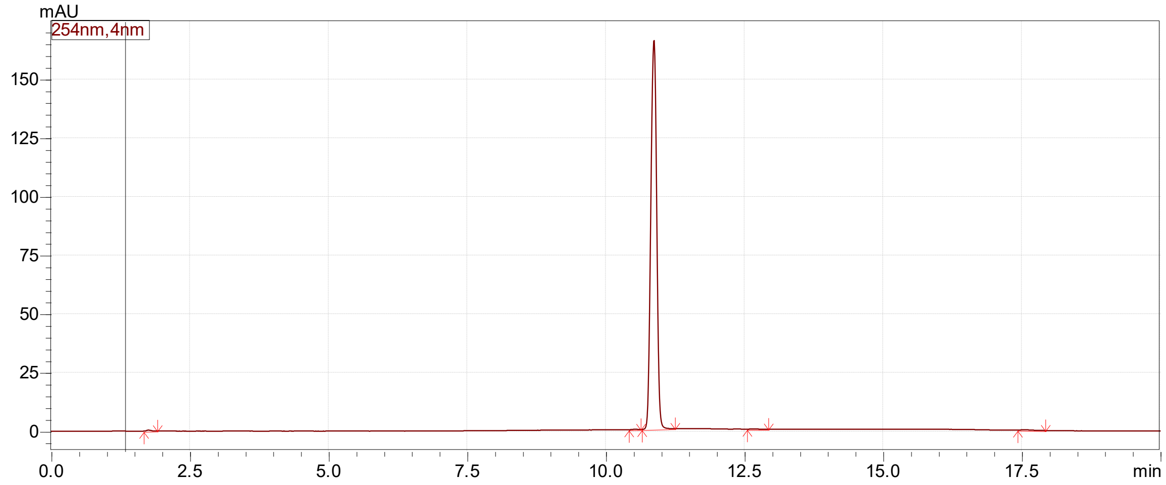

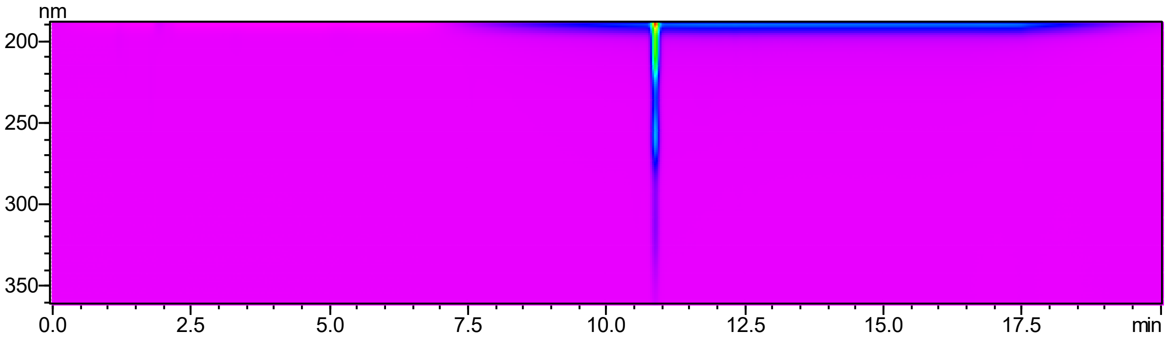

Main peak retention time: 10.875 min with 99.420 % of area under the peak at 254 nm.

2-((3,4-Dinitrobenzyl)sulfanyl)-5-phenyl-1,3,4-oxadiazole (**68a**): ^{1}H NMR (600 MHz, DMSO-d_6)

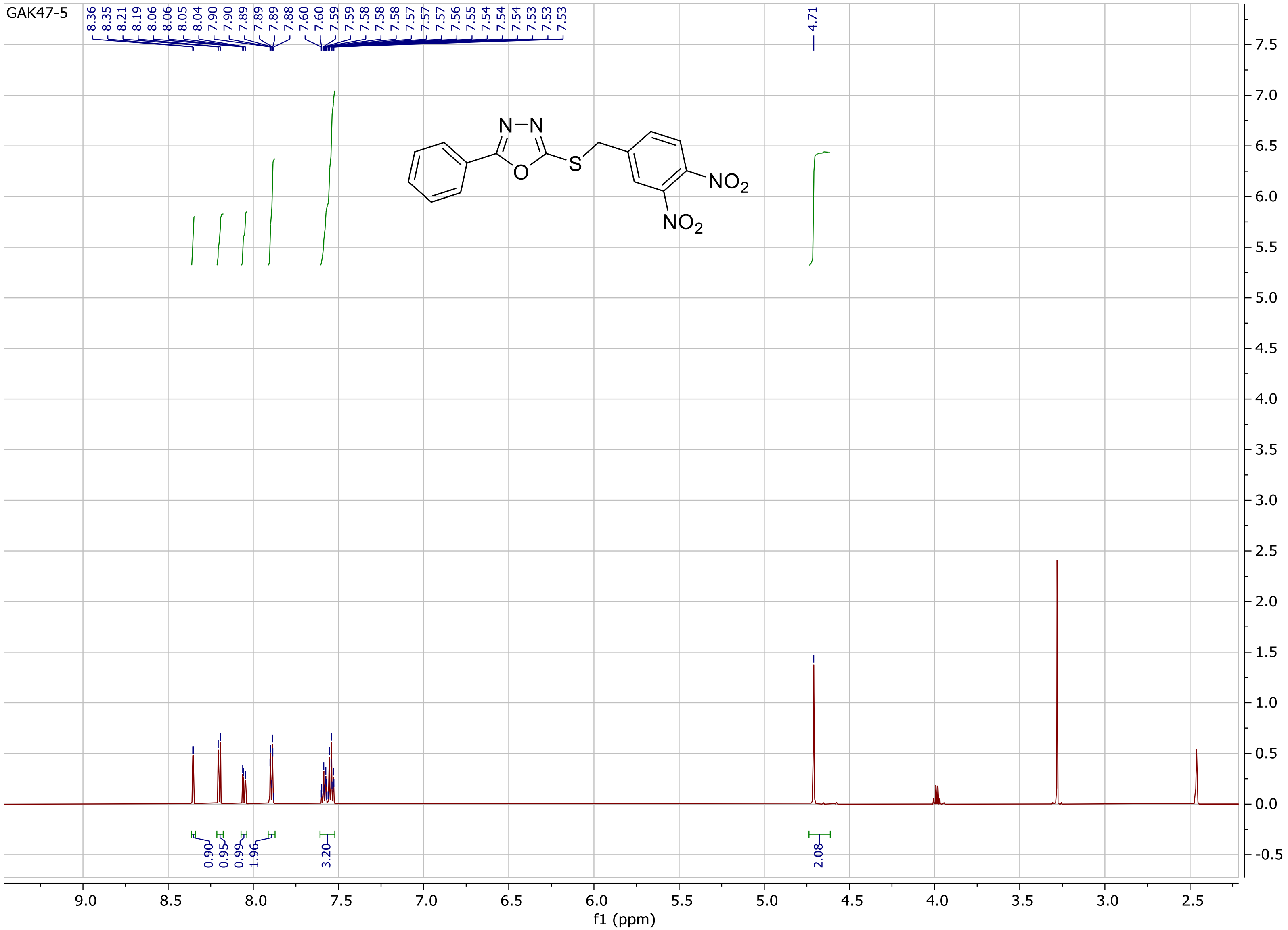

2-((3,4-Dinitrobenzyl)sulfanyl)-5-phenyl-1,3,4-oxadiazole (**68a**): ^{13}C NMR (151 MHz, DMSO-d_6)

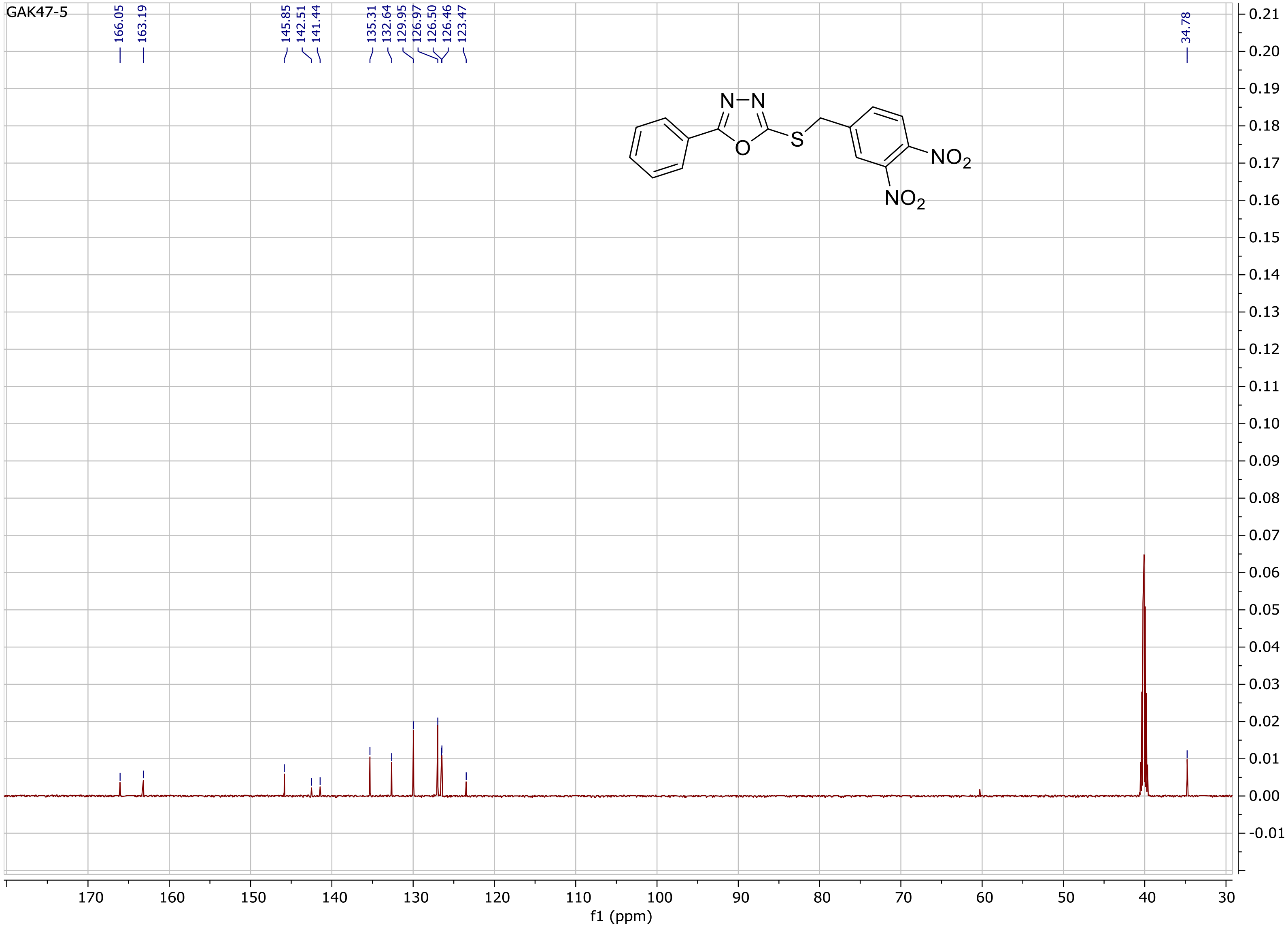

2-((3,4-Dinitrobenzyl)sulfanyl)-5-(4-methoxyphenyl)-1,3,4-oxadiazole (**68b**): ^{1}H NMR (600 MHz, DMSO-d_6)

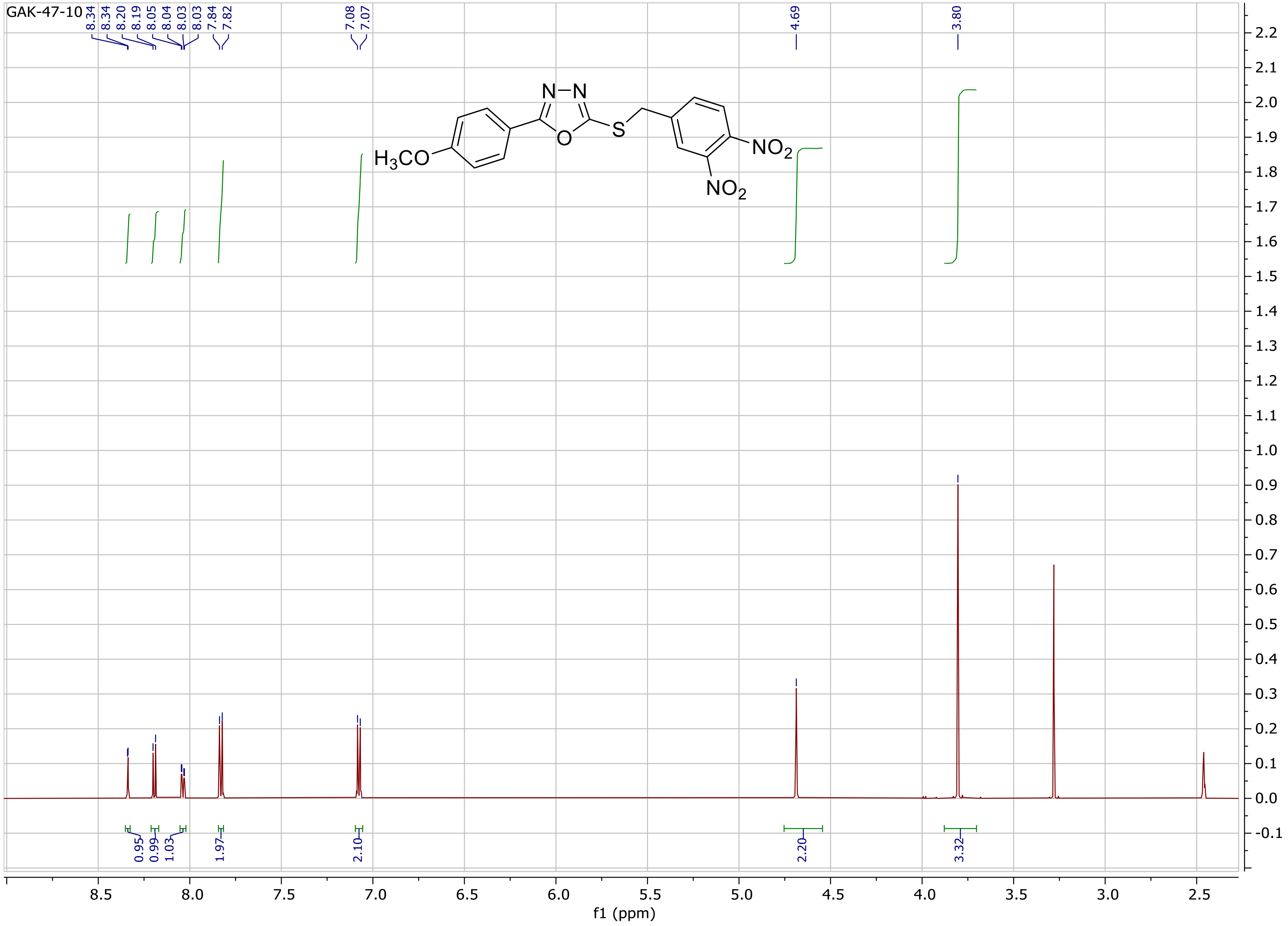

2-((3,4-Dinitrobenzyl)sulfanyl)-5-(4-methoxyphenyl)-1,3,4-oxadiazole (**68b**): ^{13}C NMR (151 MHz, DMSO-d_6)

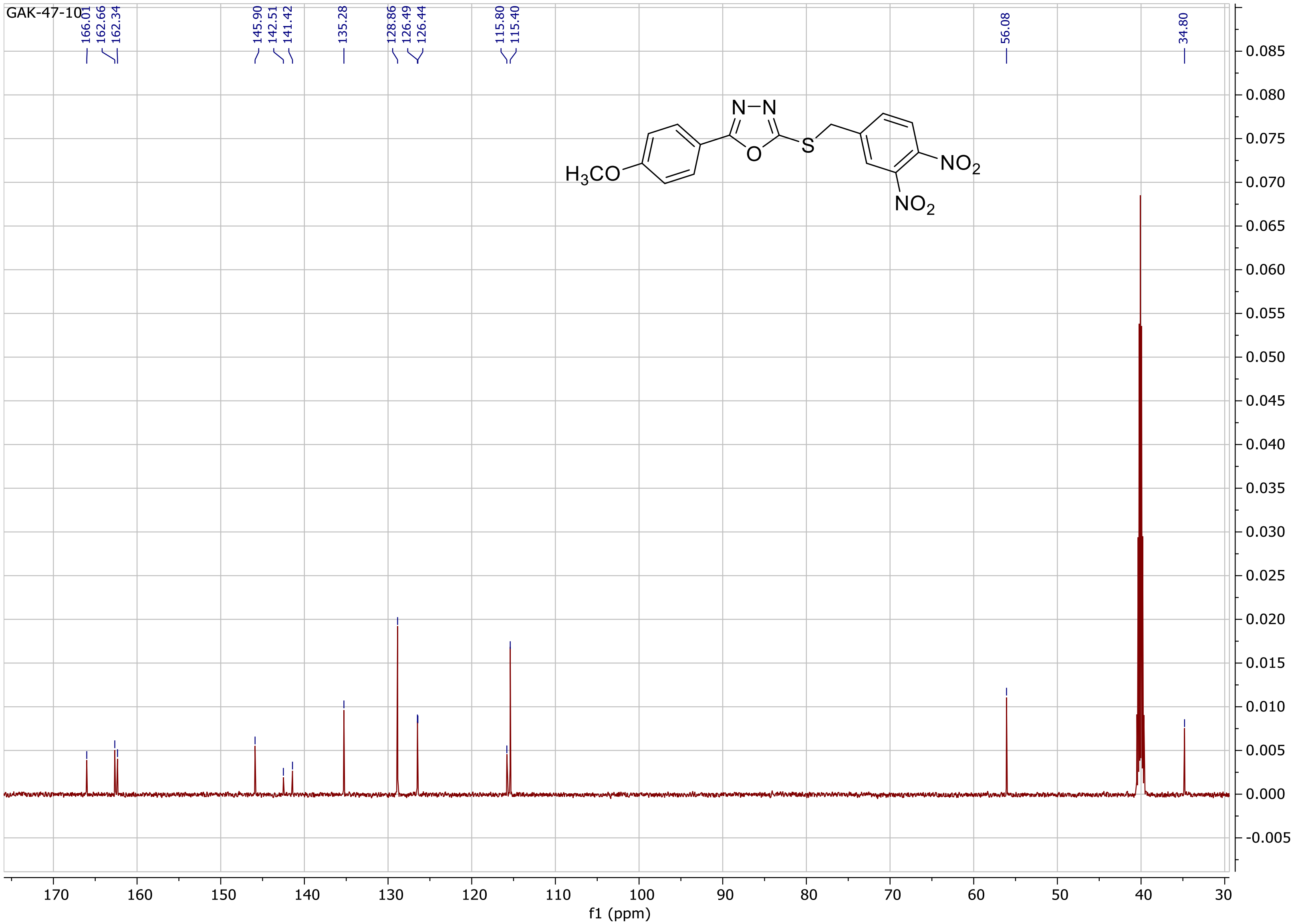

2-(4-Chlorophenyl)-5-((3,4-dinitrobenzyl)sulfanyl)-1,3,4-oxadiazole (**68c**): ^{1}H NMR (600 MHz, DMSO-d_6)

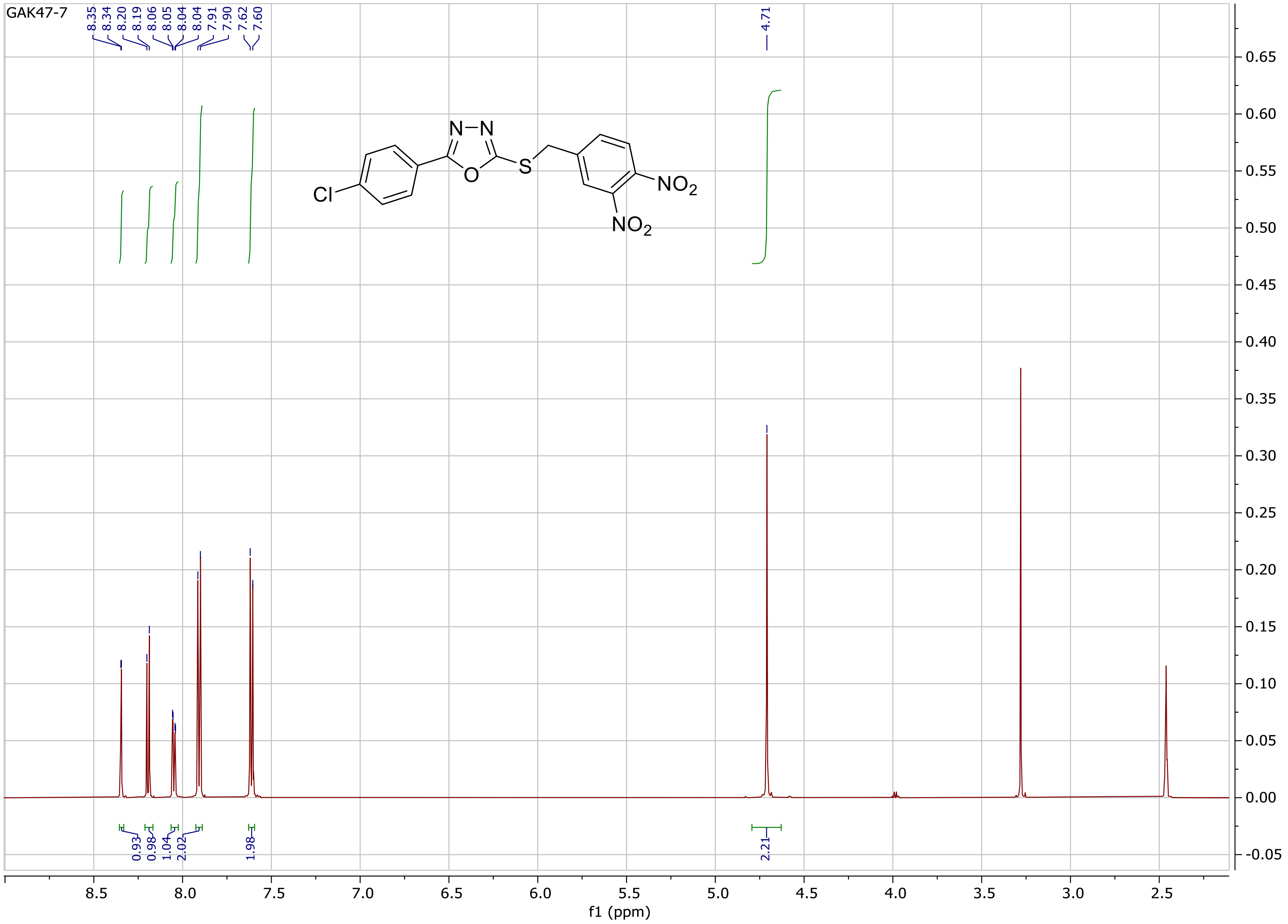

2-(4-Chlorophenyl)-5-((3,4-dinitrobenzyl)sulfanyl)-1,3,4-oxadiazole (**68c**): ^{13}C NMR (151 MHz, DMSO-d_6)

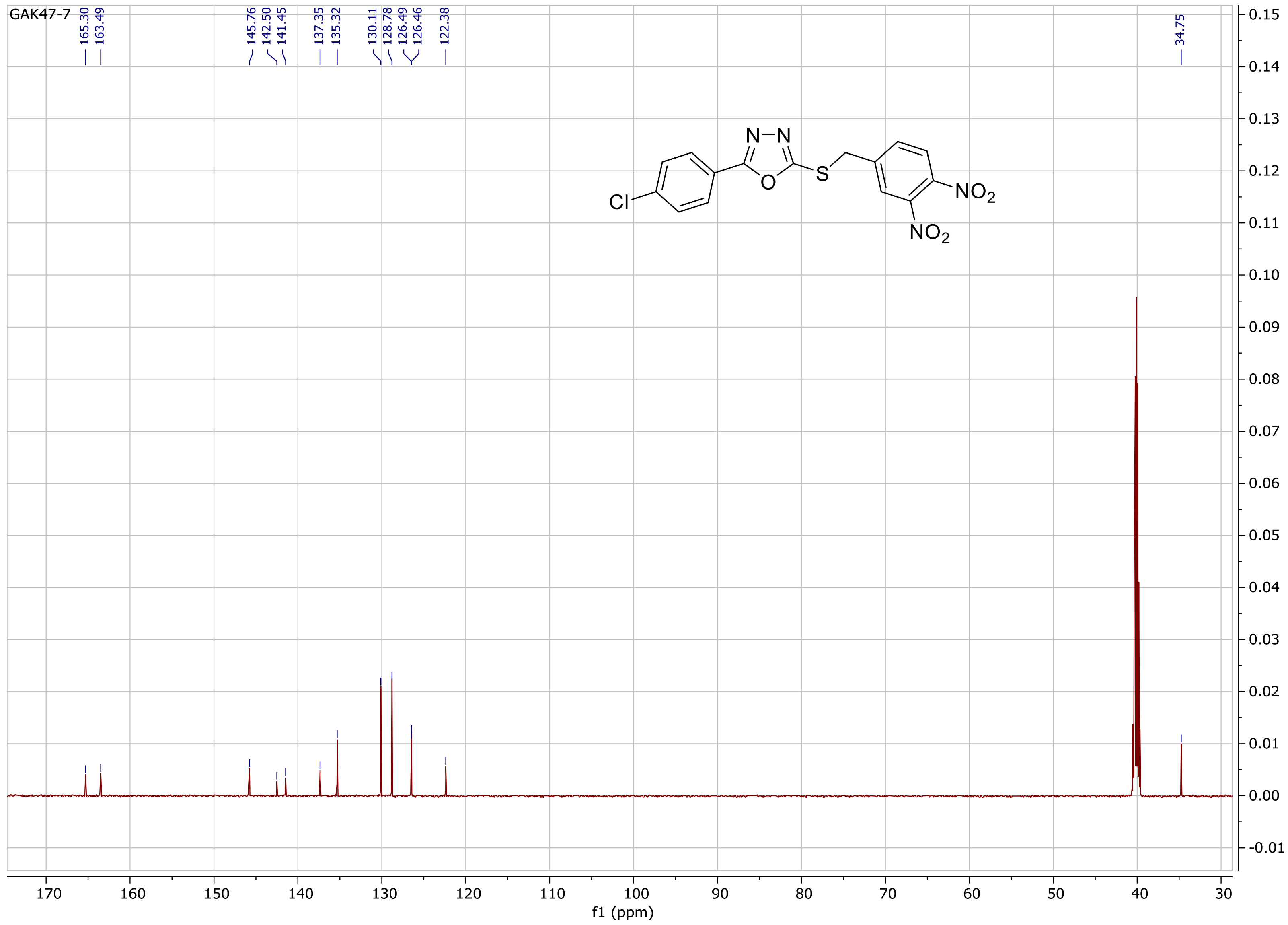

2-(4-Bromophenyl)-5-((3,4-dinitrobenzyl)sulfanyl)-1,3,4-oxadiazole (**68d**): ^{1}H NMR (600 MHz, DMSO-d_6)

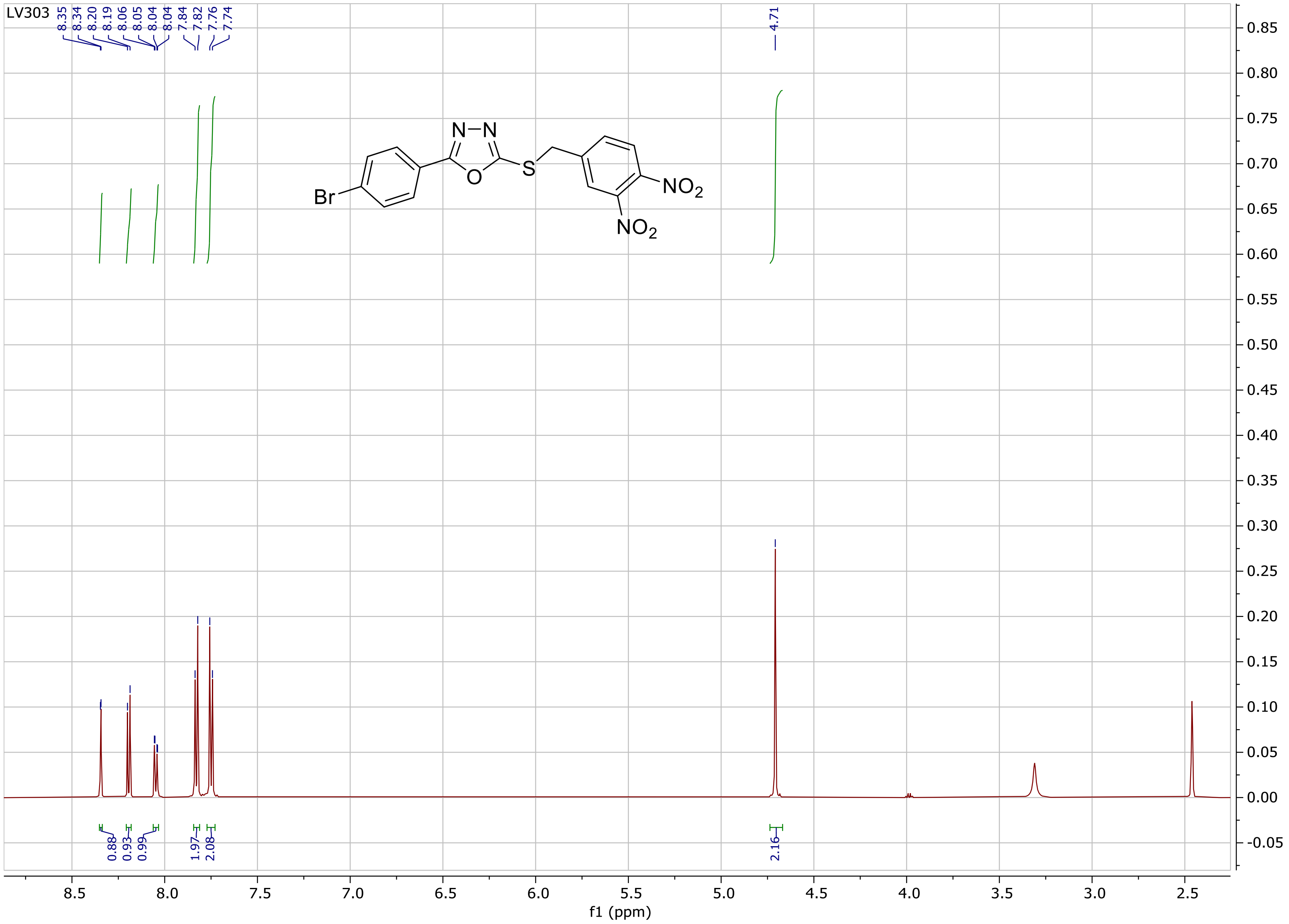

2-(4-Bromophenyl)-5-((3,4-dinitrobenzyl)sulfanyl)-1,3,4-oxadiazole (**68d**): ^{13}C NMR (151 MHz, DMSO-d_6)

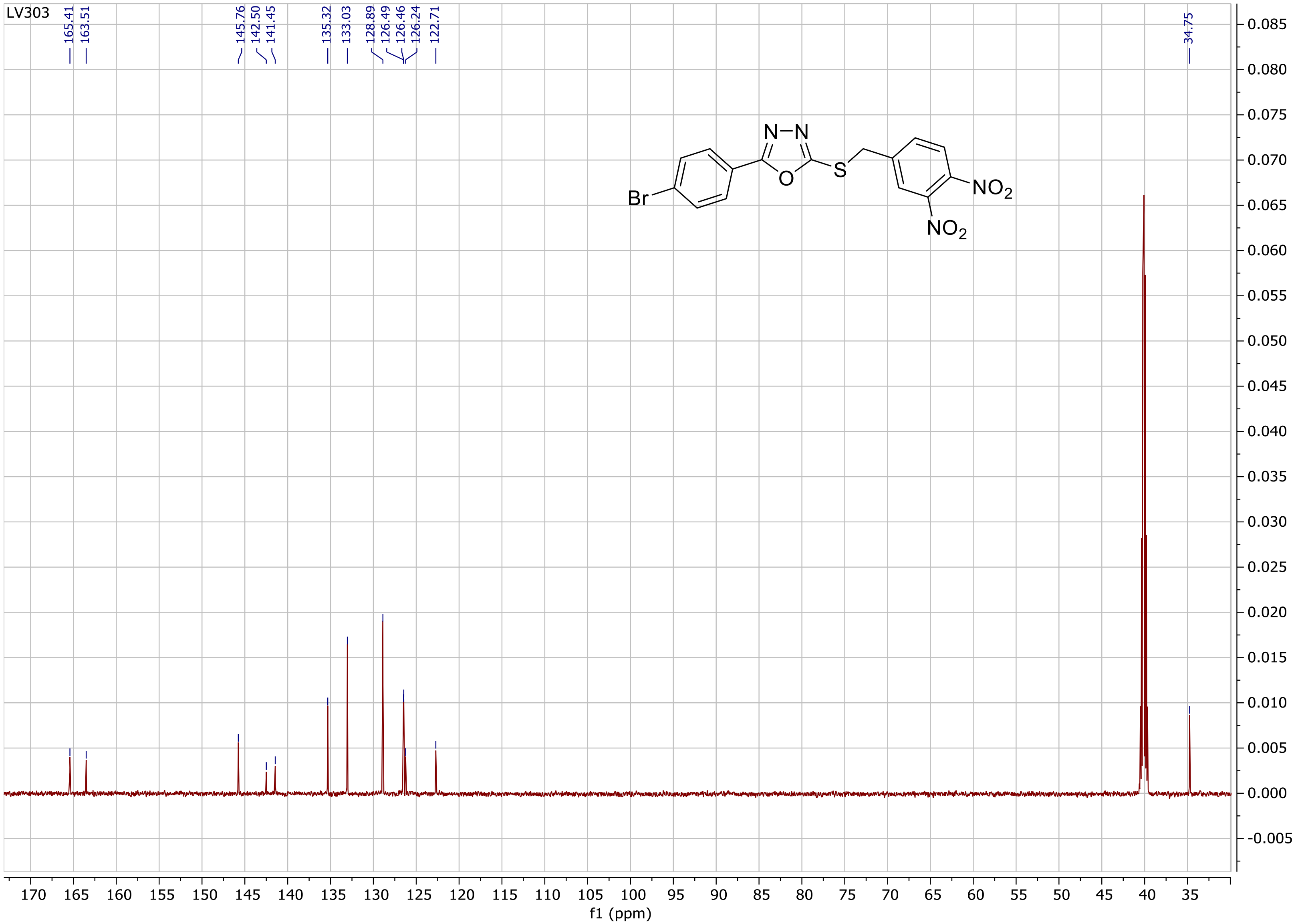

2-Cyclohexyl-5-((3,4-dinitrobenzyl)sulfanyl)-1,3,4-oxadiazole (**68e**): ^{1}H NMR (600 MHz, DMSO-d_6)

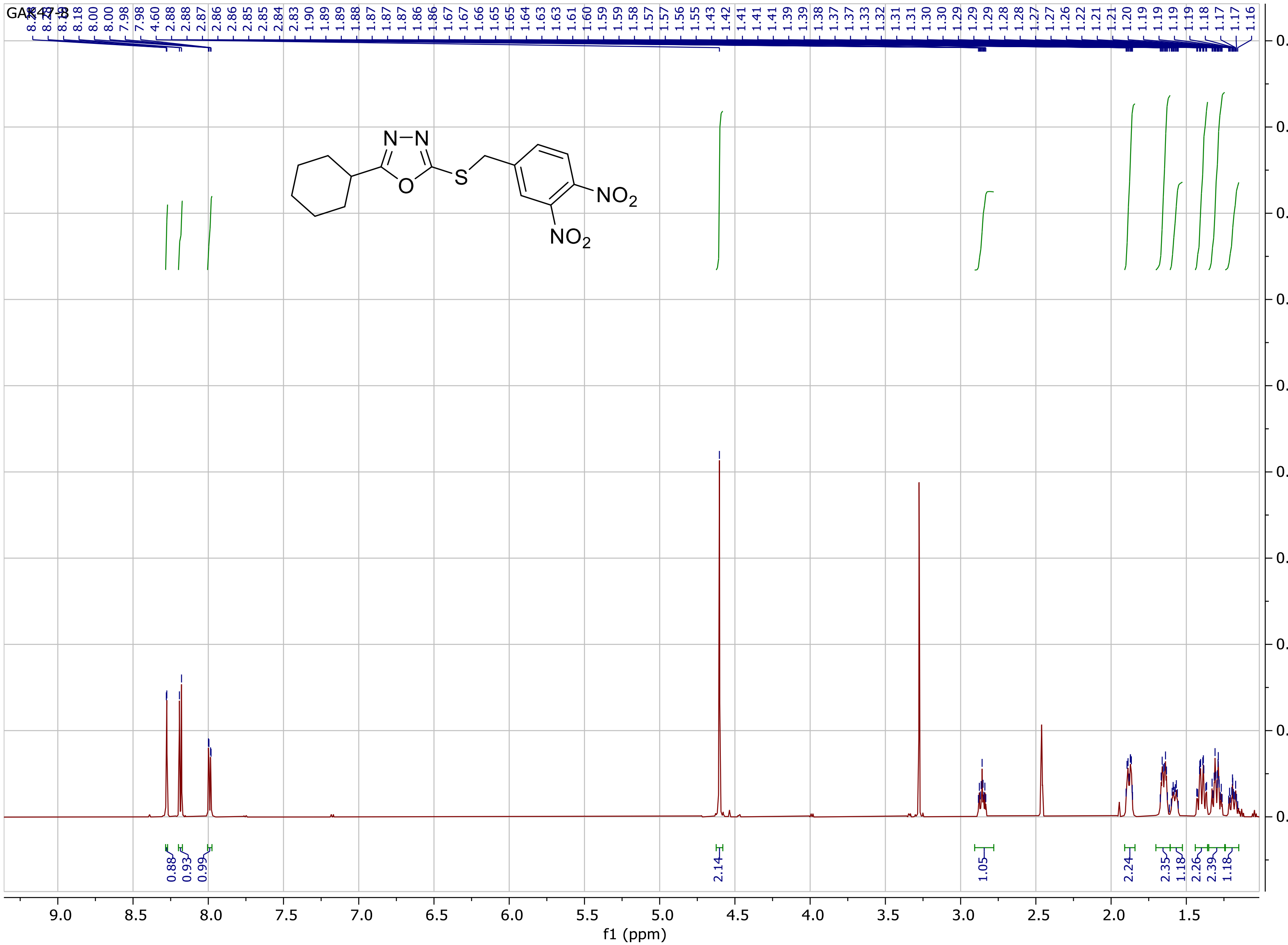

2-Cyclohexyl-5-((3,4-dinitrobenzyl)sulfanyl)-1,3,4-oxadiazole (**68e**): ^{13}C NMR (151 MHz, DMSO-d_6)

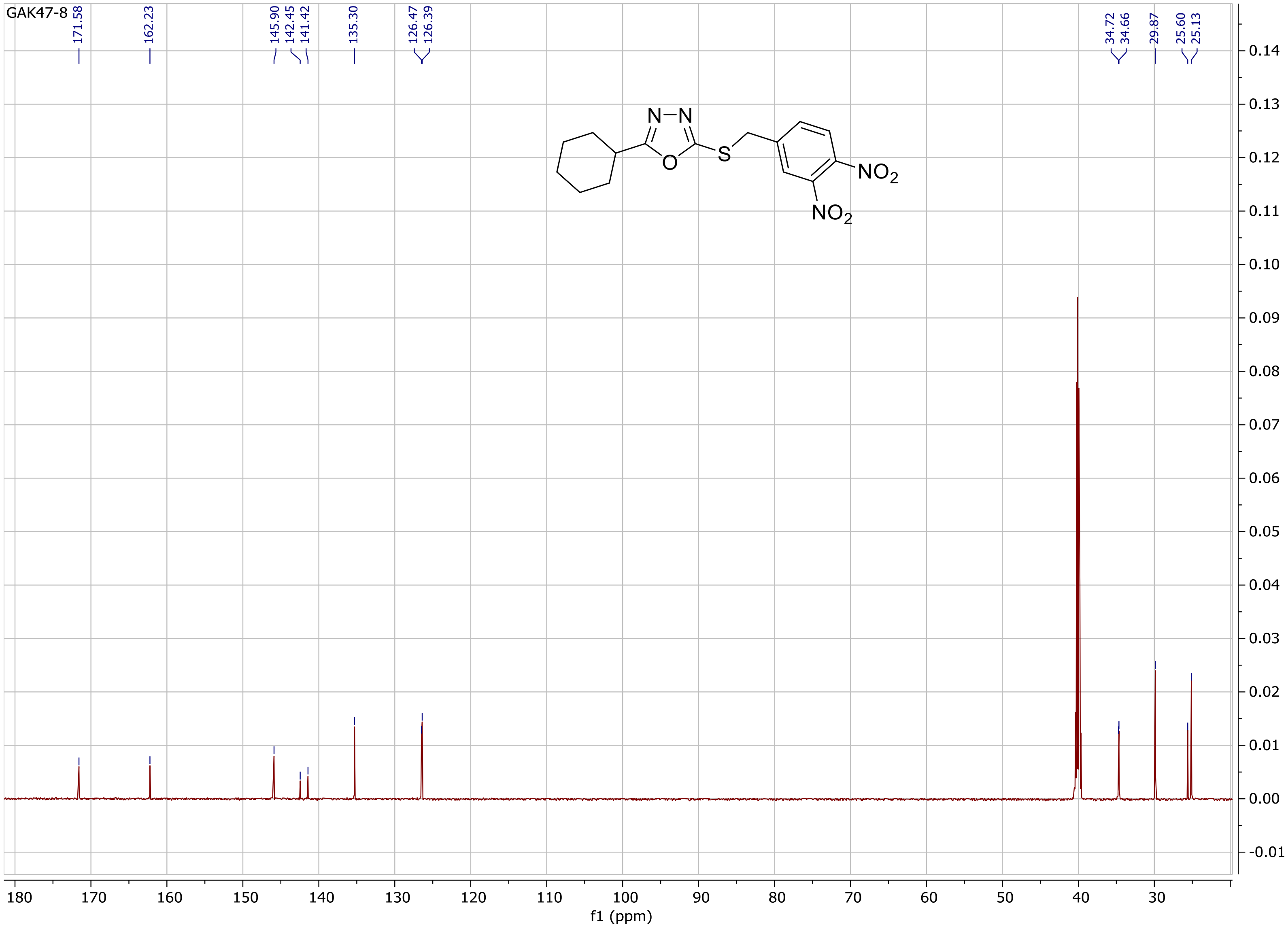

2-((2,5-Dinitrobenzyl)sulfanyl)-5-phenyl-1,3,4-oxadiazole (**69a**): ^{1}H NMR (600 MHz, DMSO-d_6)

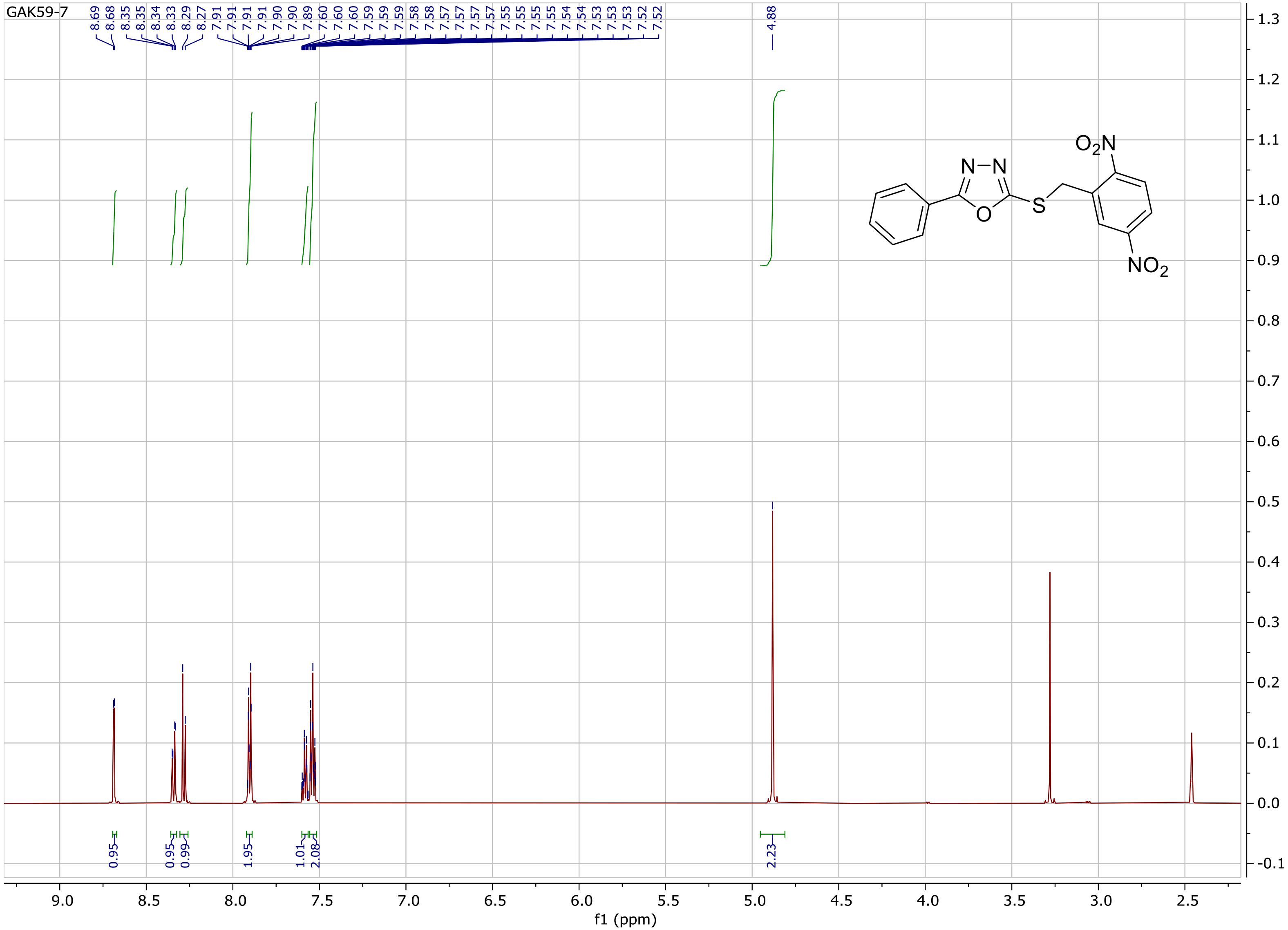

2-((2,5-Dinitrobenzyl)sulfanyl)-5-phenyl-1,3,4-oxadiazole (**69a**): ^{13}C NMR (151 MHz, DMSO-d_6)

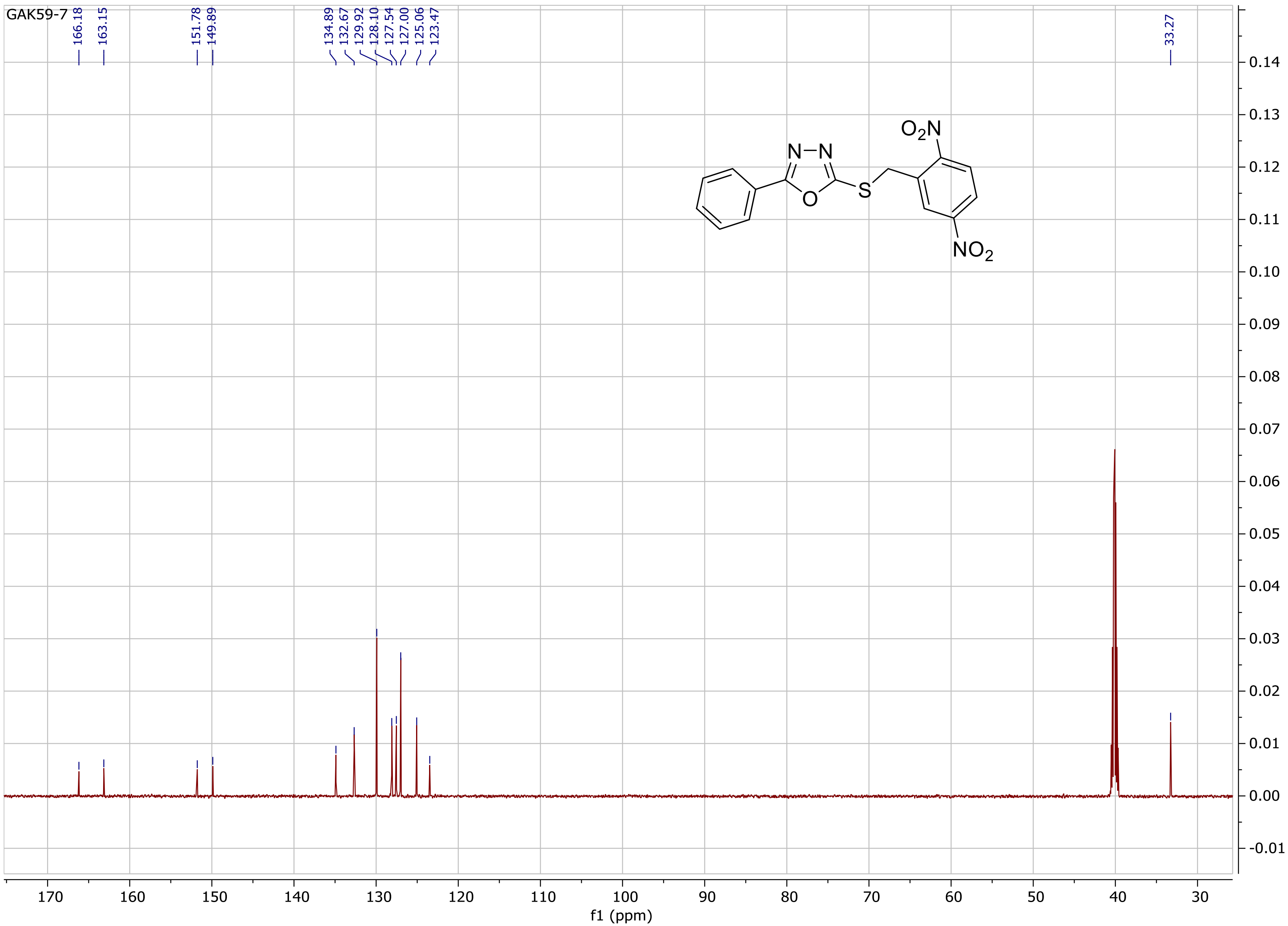

2-((2,5-Dinitrobenzyl)sulfanyl)-5-phenyl-1,3,4-oxadiazole (**69a**): HPLC trace

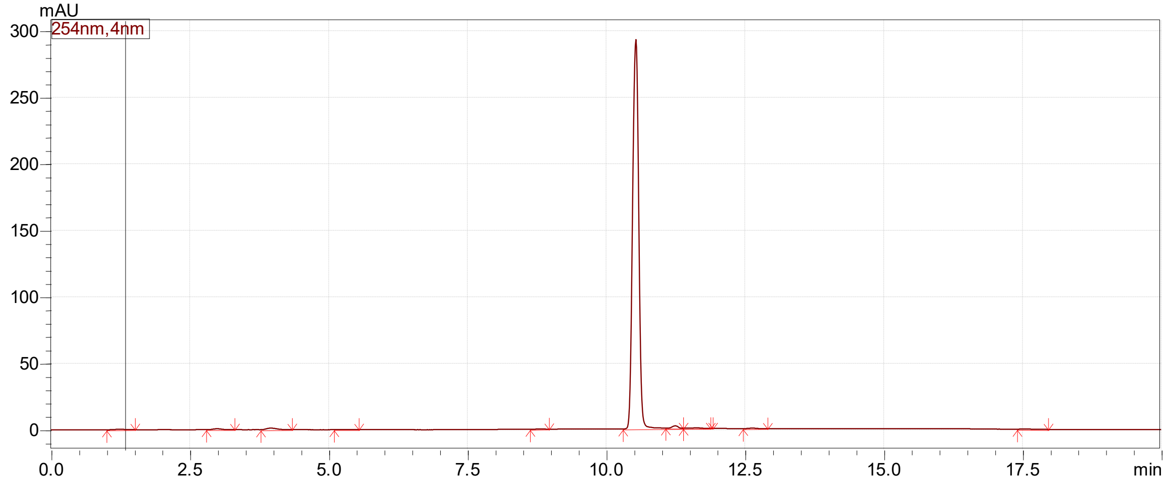

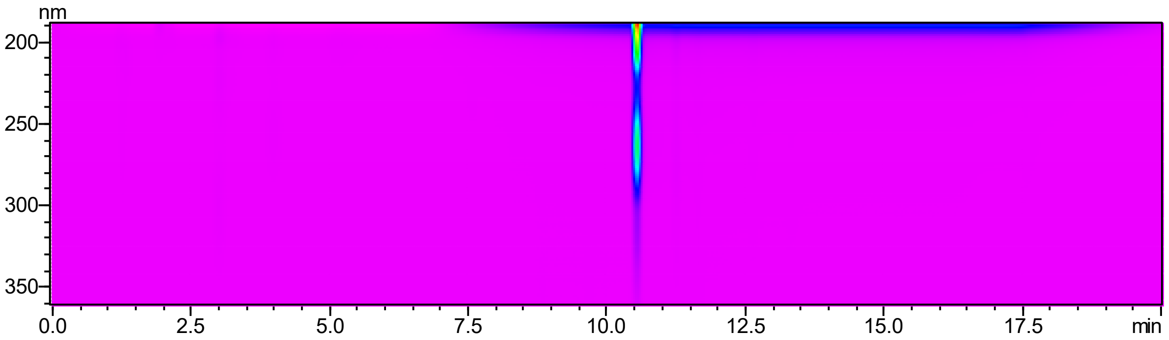

Main peak retention time: 10.538 min with 97.635 % of area under the peak at 254 nm.

2-((2,5-Dinitrobenzyl)sulfanyl)-5-(4-methoxyphenyl)-1,3,4-oxadiazole (**69b**): ^{1}H NMR (600 MHz, DMSO-d_6)

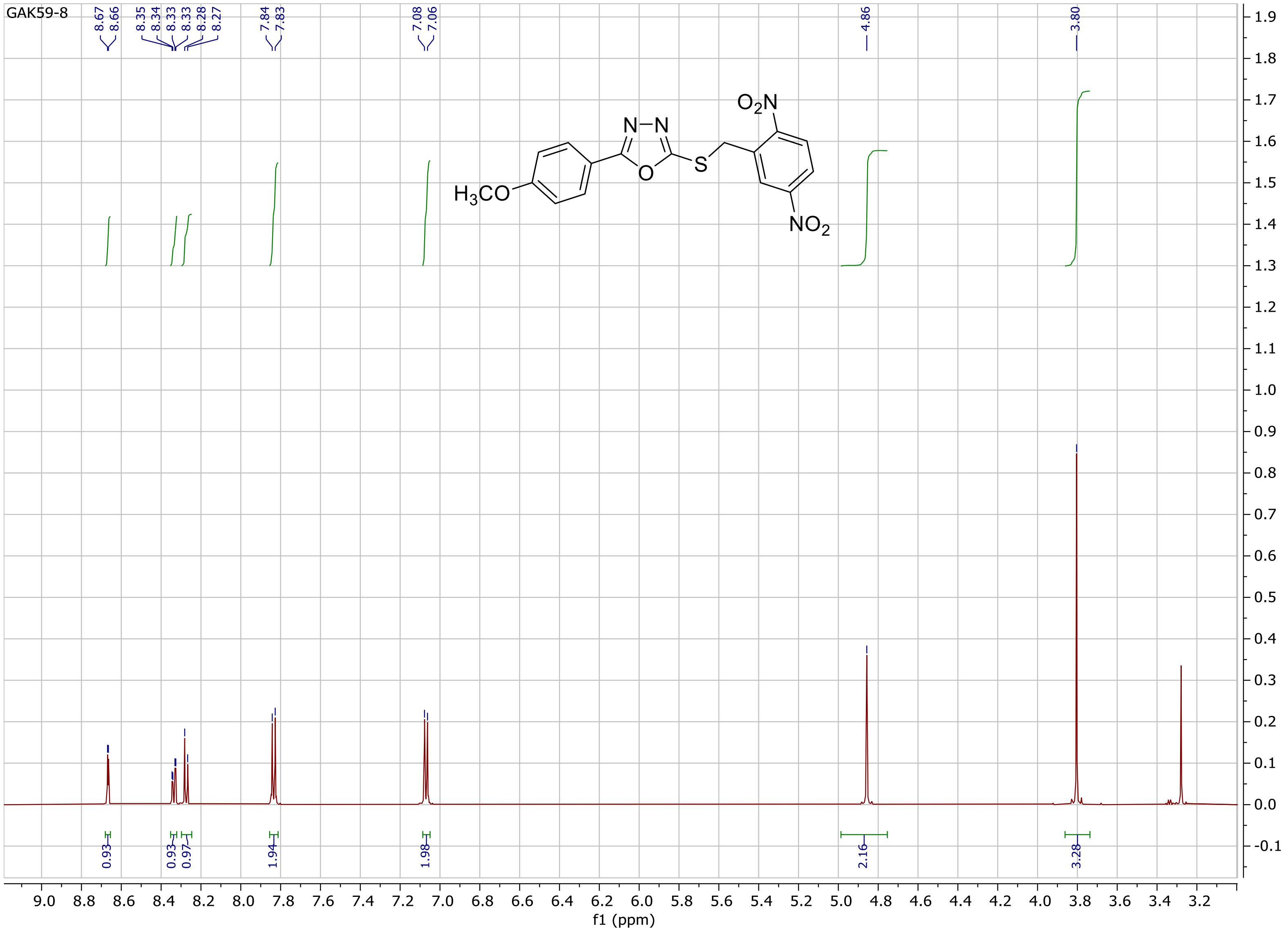

2-((2,5-Dinitrobenzyl)sulfanyl)-5-(4-methoxyphenyl)-1,3,4-oxadiazole (**69b**): ^{13}C NMR (151 MHz, DMSO-d_6)

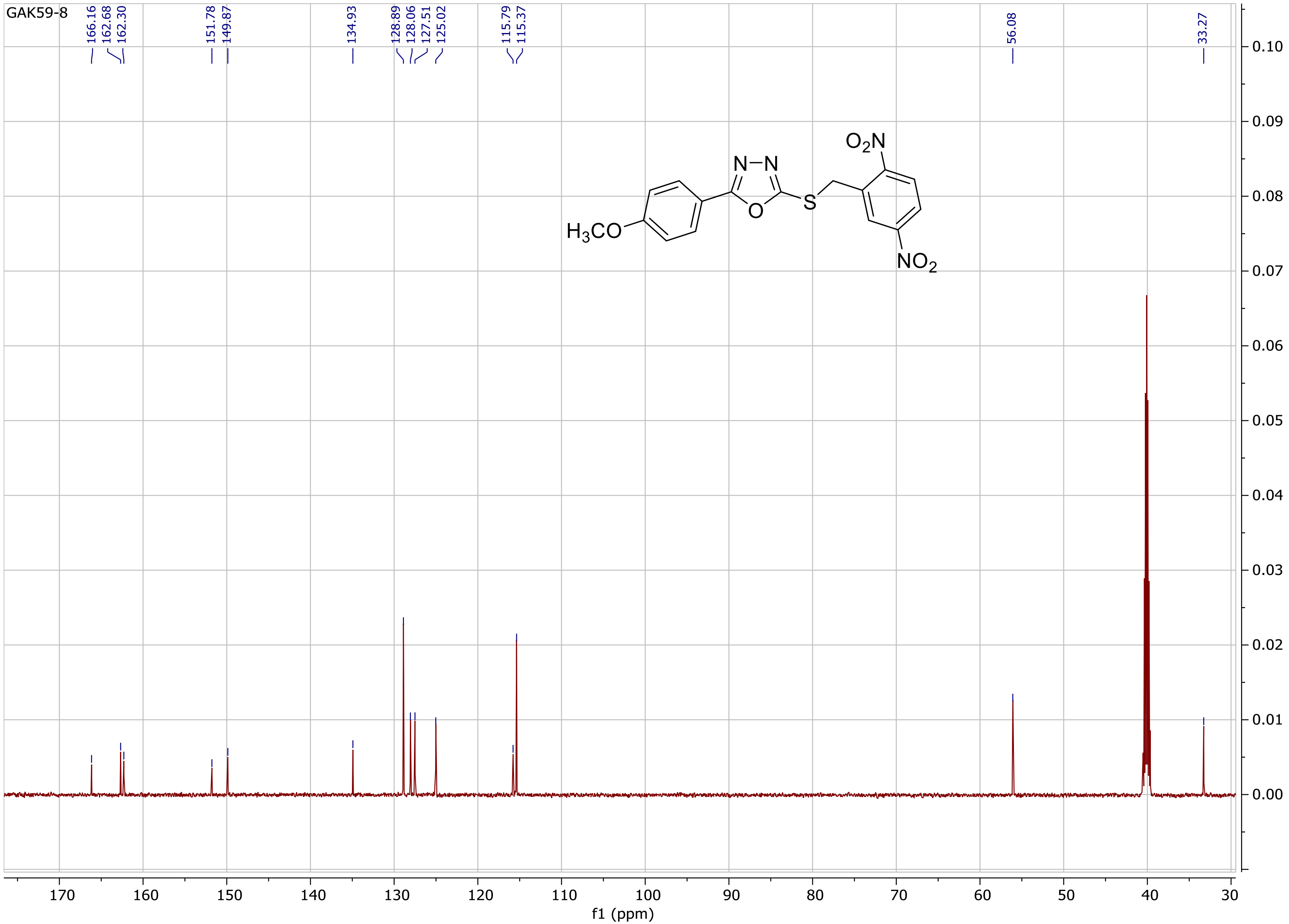

2-((2,5-Dinitrobenzyl)sulfanyl)-5-(4-methoxyphenyl)-1,3,4-oxadiazole (**69b**): HPLC trace

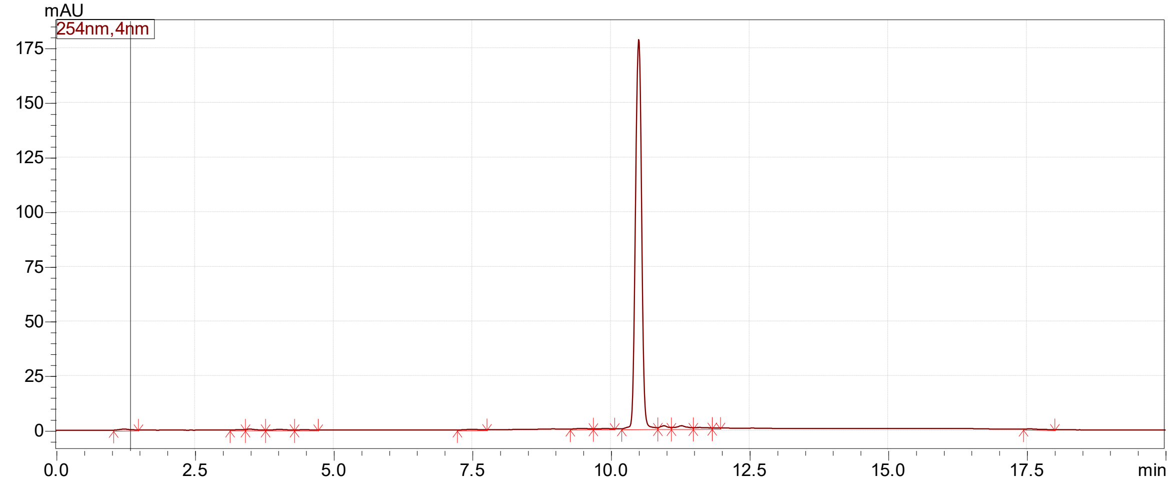

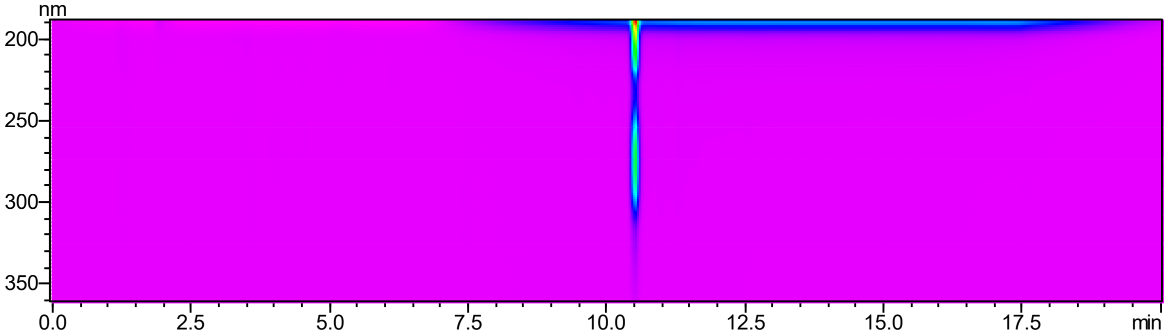

Main peak retention time: 10.510 min with 95.344 % of area under the peak at 254 nm.

2-(4-Chlorophenyl)-5-((2,5-dinitrobenzyl)sulfanyl)-1,3,4-oxadiazole (**69c**): ^{1}H NMR (500 MHz, DMSO-d_6)

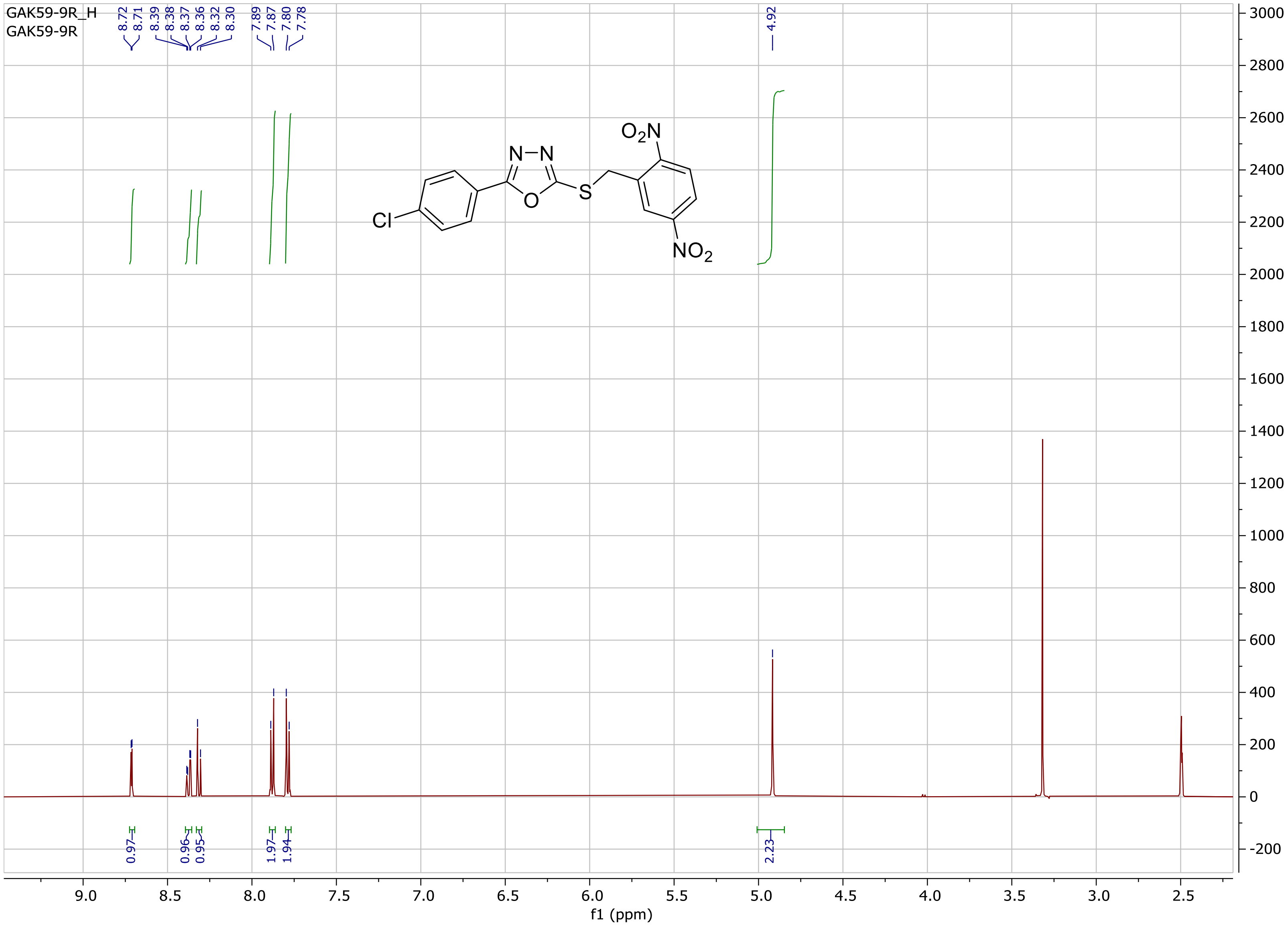

2-(4-Chlorophenyl)-5-((2,5-dinitrobenzyl)sulfanyl)-1,3,4-oxadiazole (**69c**): ^{13}C NMR (126 MHz, DMSO-d_6)

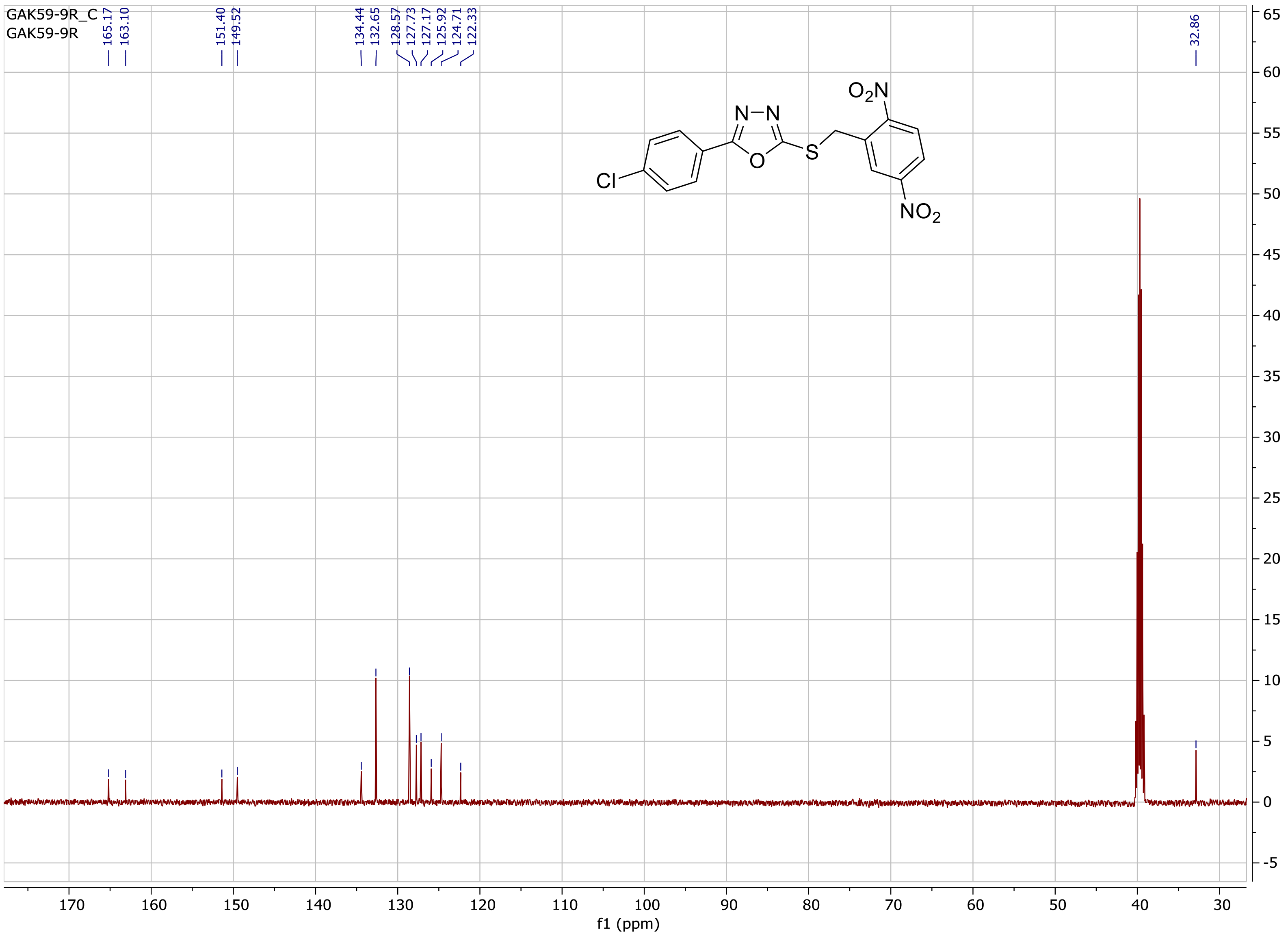

2-(4-Chlorophenyl)-5-((2,5-dinitrobenzyl)sulfanyl)-1,3,4-oxadiazole (**69c**): HPLC trace

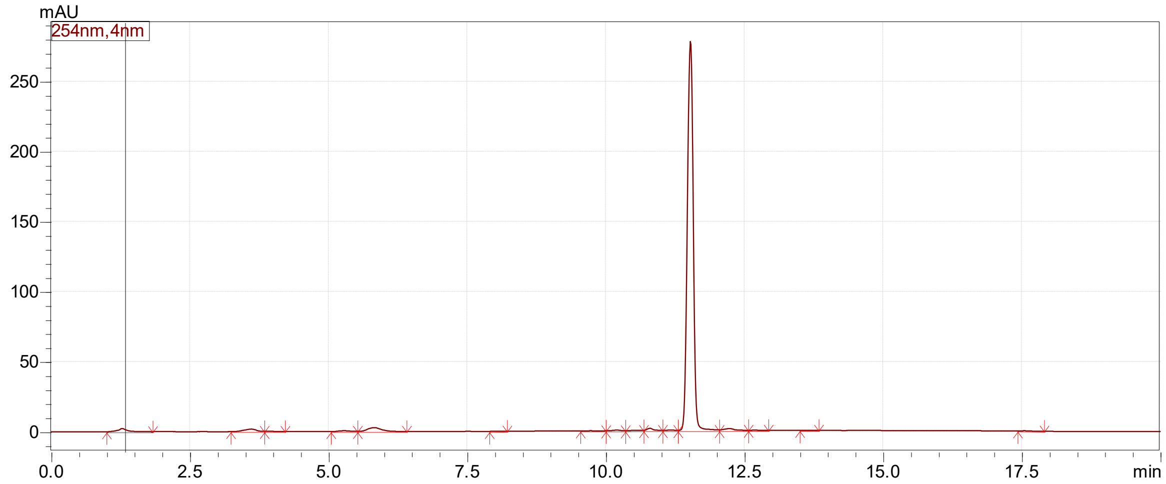

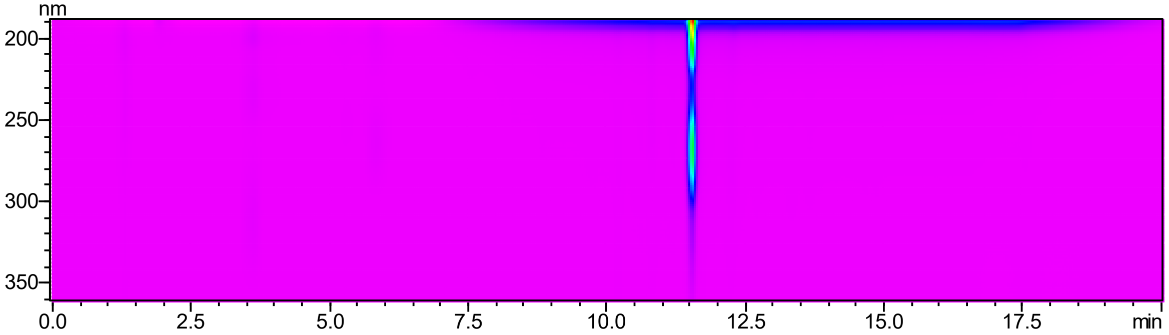

Main peak retention time: 11.534 min with 95.954 % of area under the peak at 254 nm.

2-(4-Bromophenyl)-5-((2,5-dinitrobenzyl)sulfanyl)-1,3,4-oxadiazole (**69d**): ^{1}H NMR (600 MHz, DMSO-d_6)

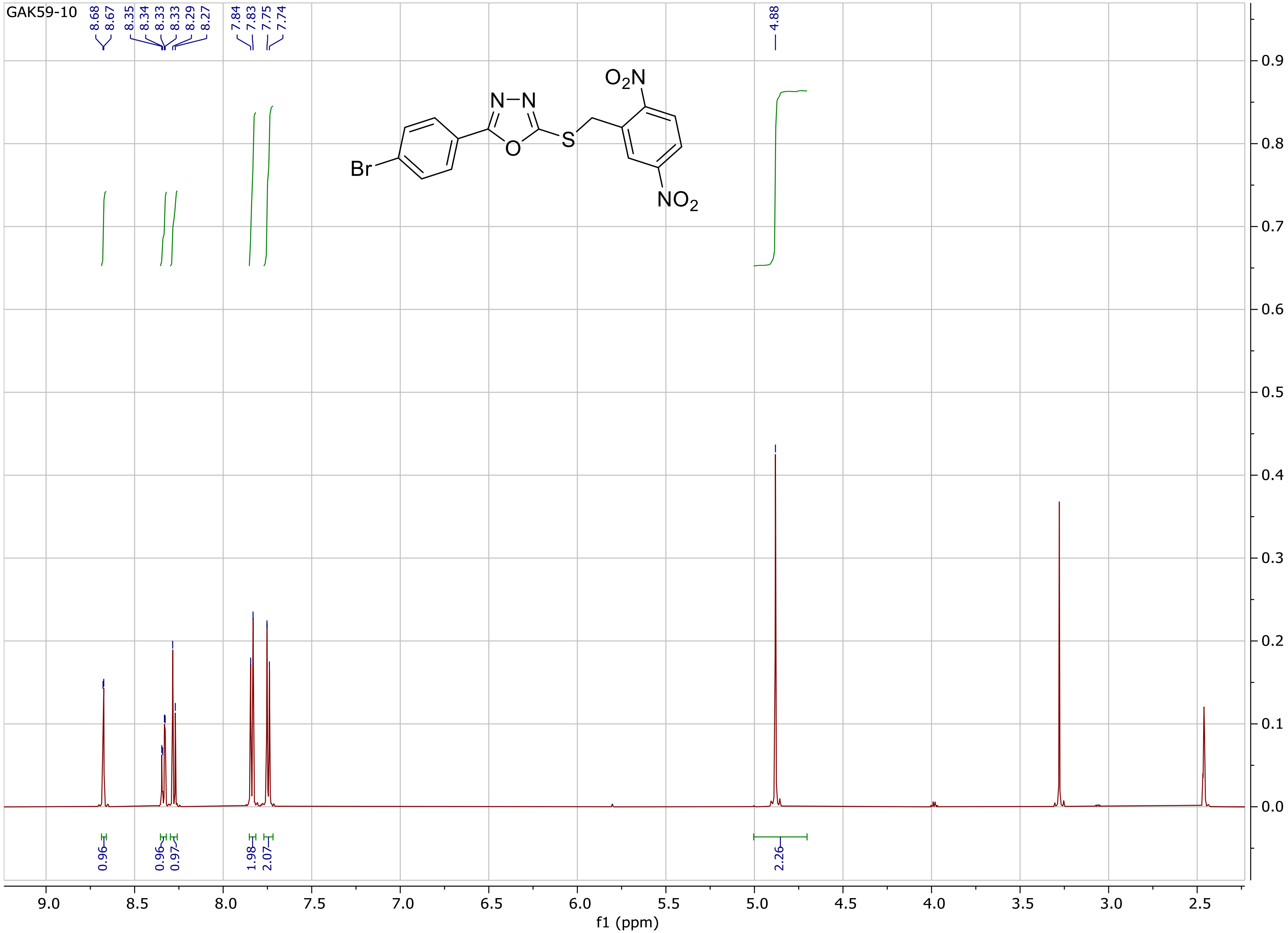

2-(4-Bromophenyl)-5-((2,5-dinitrobenzyl)sulfanyl)-1,3,4-oxadiazole (**69d**): ^{13}C NMR (151 MHz, DMSO-d_6)

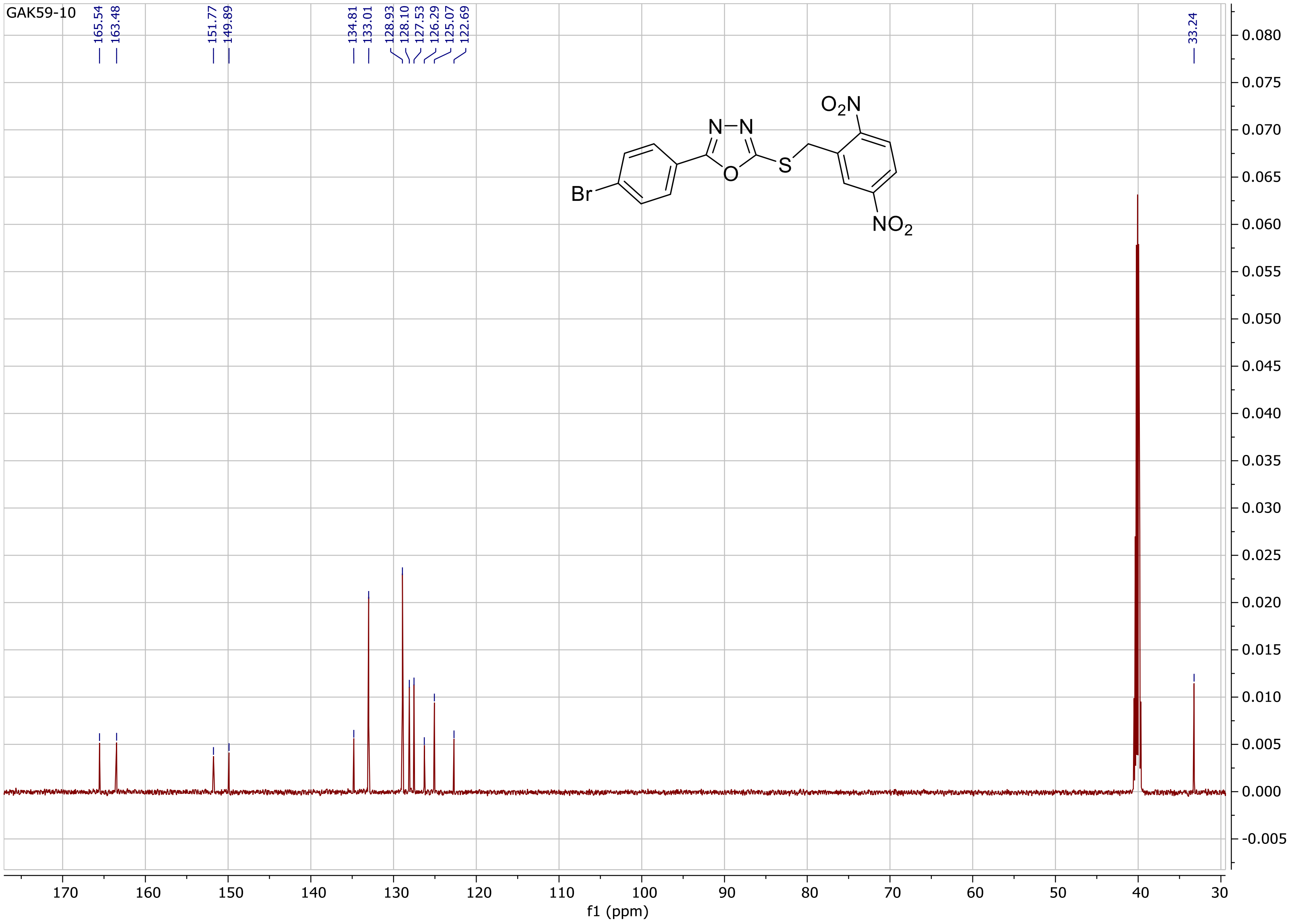

2-(4-Bromophenyl)-5-((2,5-dinitrobenzyl)sulfanyl)-1,3,4-oxadiazole (**69d**): HPLC trace

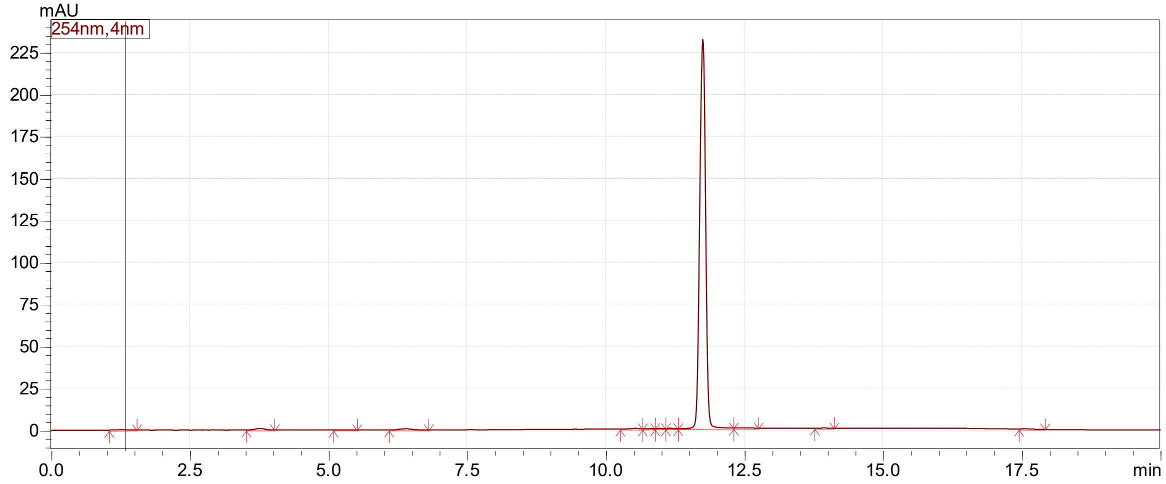

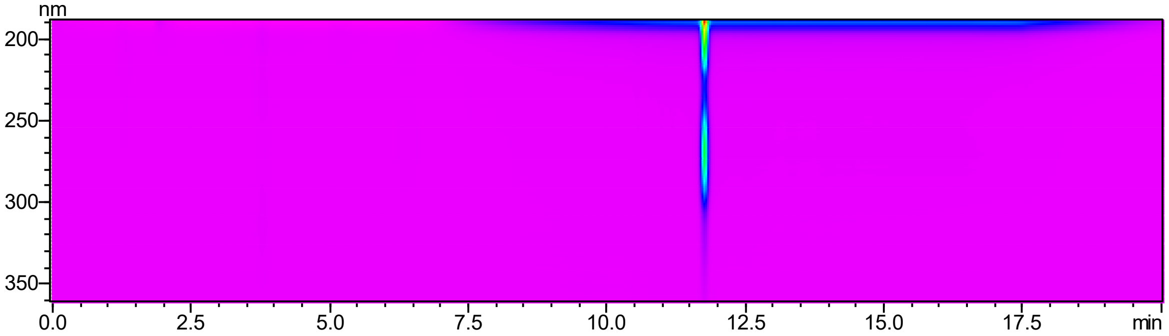

Main peak retention time: 11.757 min with 97.239 % of area under the peak at 254 nm.

2-Cyclohexyl-5-((2,5-dinitrobenzyl)sulfanyl)-1,3,4-oxadiazole (**69e**): ^{1}H NMR (600 MHz, DMSO-d_6)

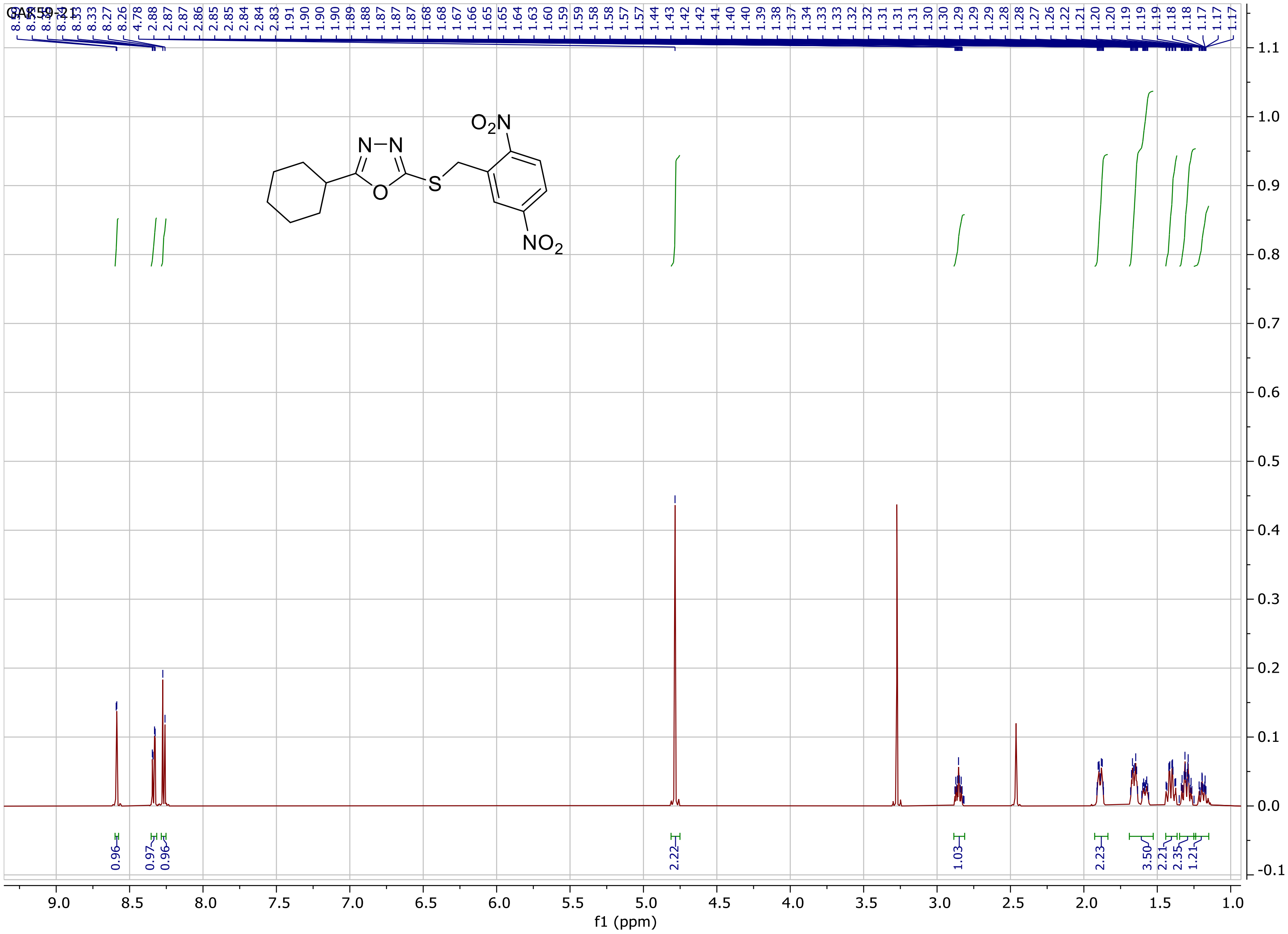

2-Cyclohexyl-5-((2,5-dinitrobenzyl)sulfanyl)-1,3,4-oxadiazole (**69e**): ^{13}C NMR (151 MHz, DMSO-d_6)

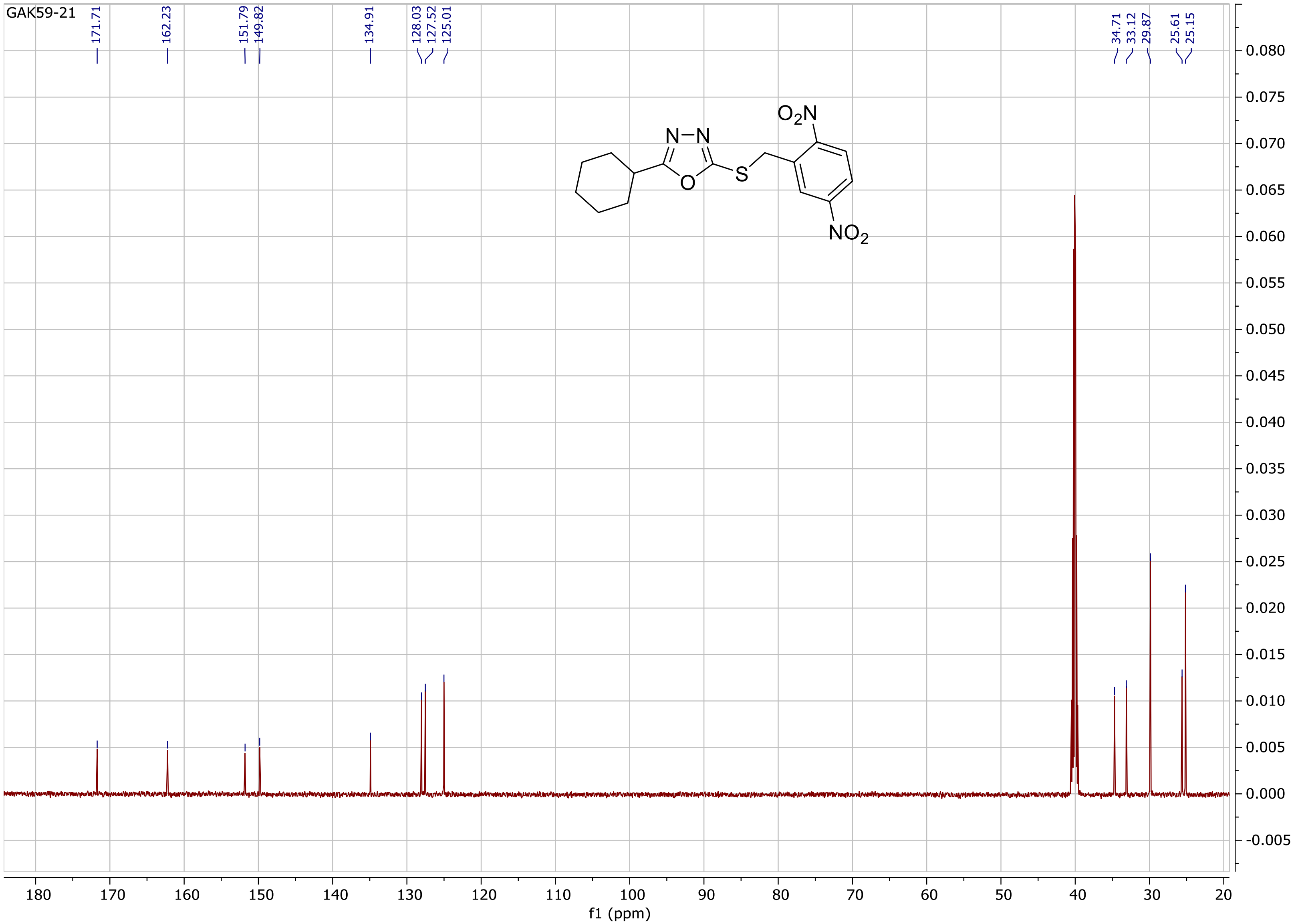

2-Cyclohexyl-5-((2,5-dinitrobenzyl)sulfanyl)-1,3,4-oxadiazole (**69e**): HPLC trace

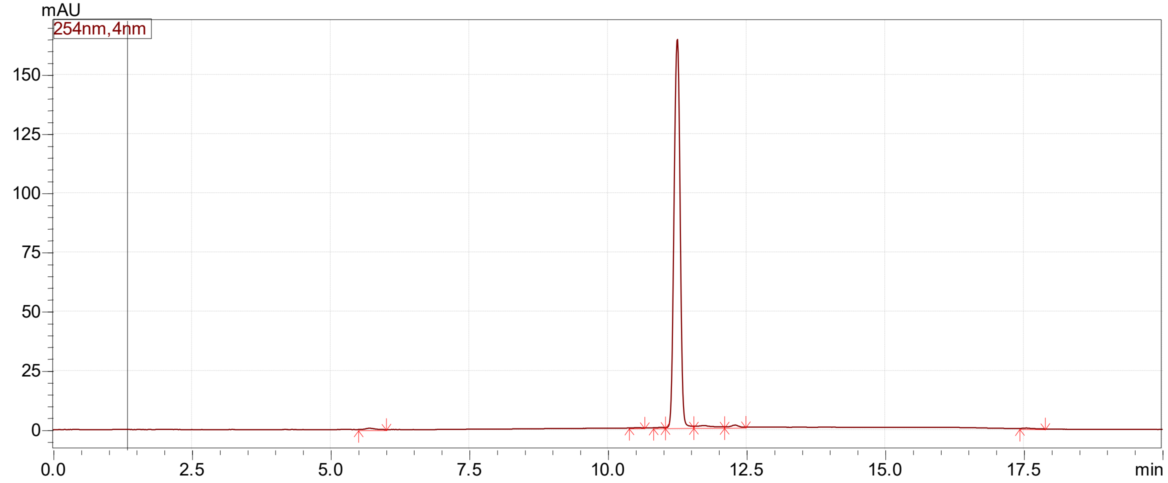

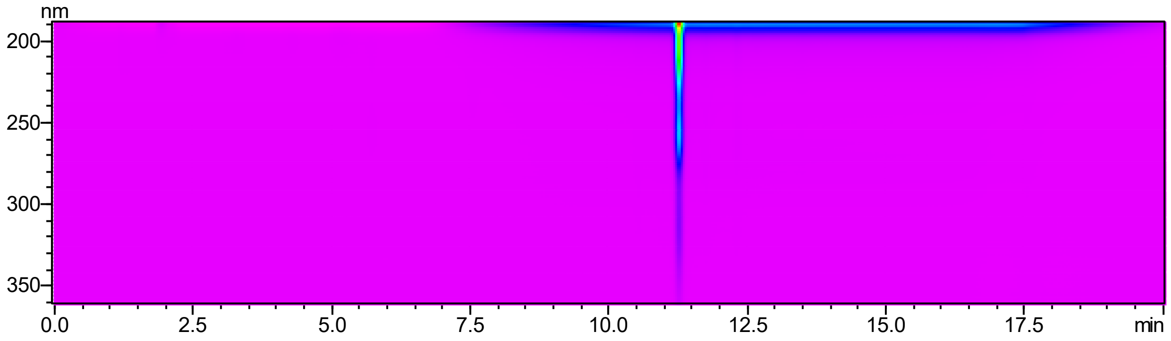

Main peak retention time: 11.260 min with 97.325 % of area under the peak at 254 nm.

5-((2-Nitro-5-(trifluoromethyl)benzyl)sulfanyl)-1-phenyl-1H-tetrazole (**70a**): ^{1}H NMR (600 MHz, DMSO-d_6)

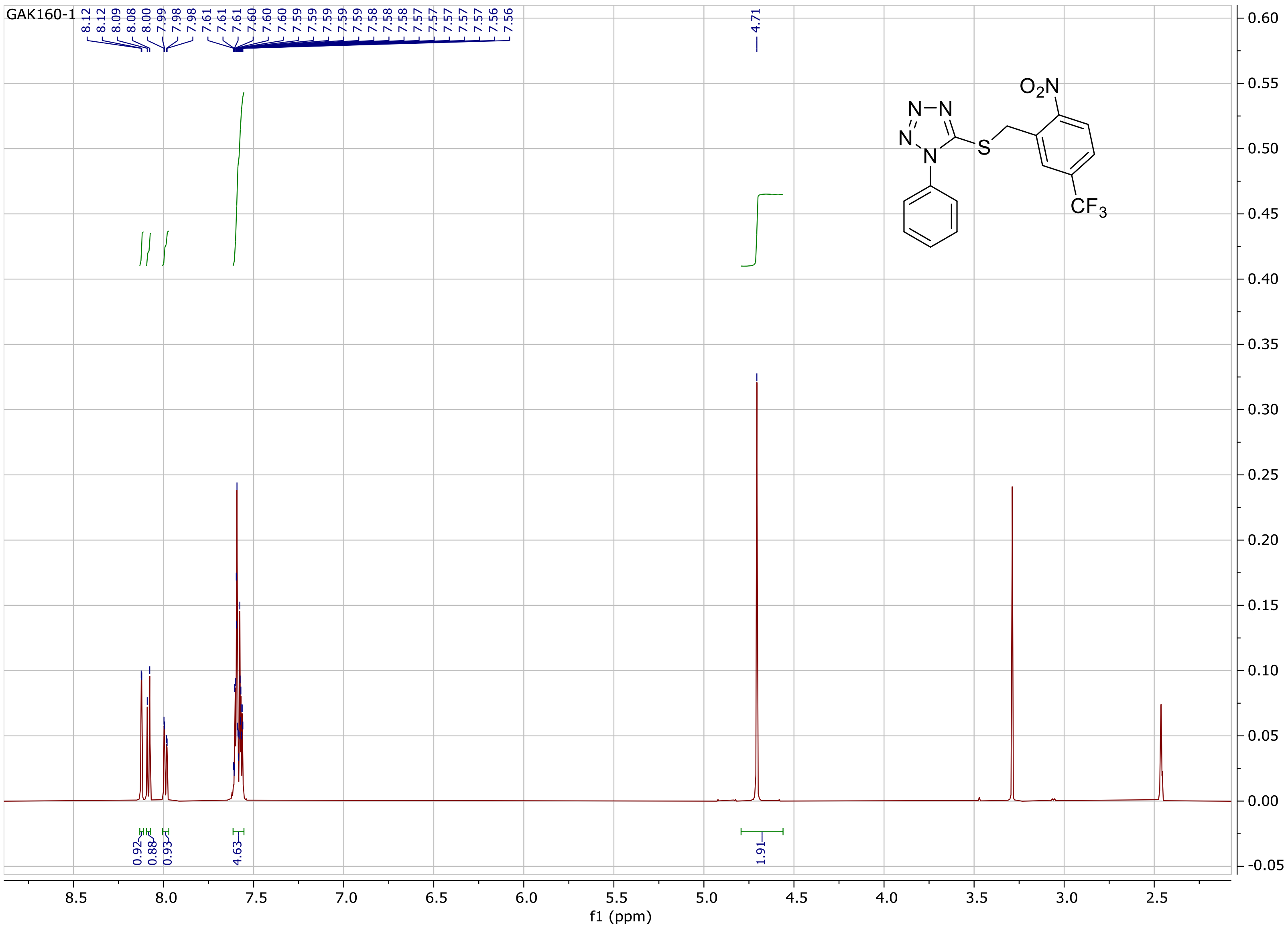

5-((2-Nitro-5-(trifluoromethyl)benzyl)sulfanyl)-1-phenyl-1H-tetrazole (**70a**): ^{13}C NMR (151 MHz, DMSO-d_6)

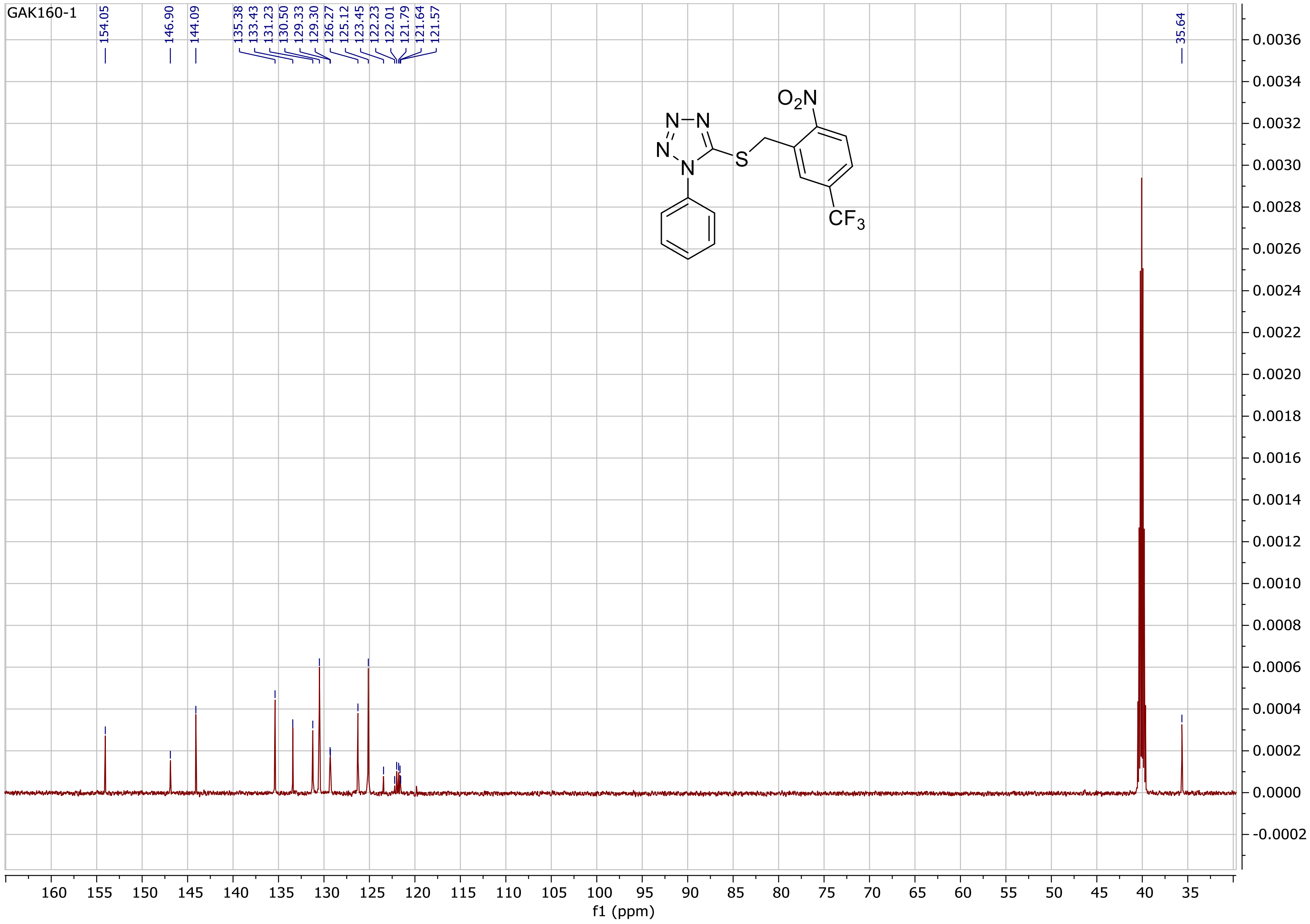

5-((2-Nitro-5-(trifluoromethyl)benzyl)sulfanyl)-1-phenyl-1H-tetrazole (**70a**):

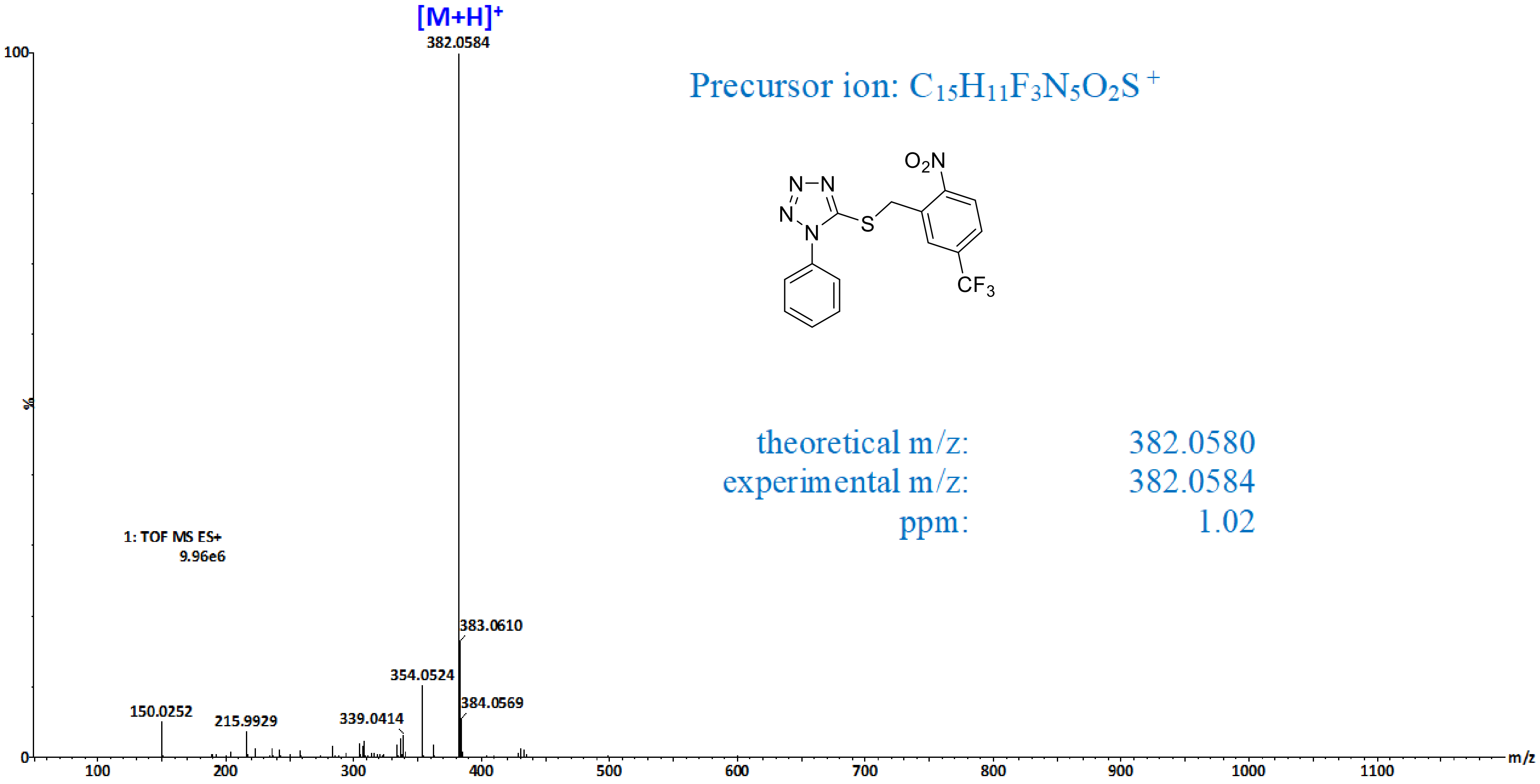

1-(4-Methoxyphenyl)-5-((2-nitro-5-(trifluoromethyl)benzyl)sulfanyl)-1H-tetrazole (**70b**): ^{1}H NMR (600 MHz, DMSO-d_6)

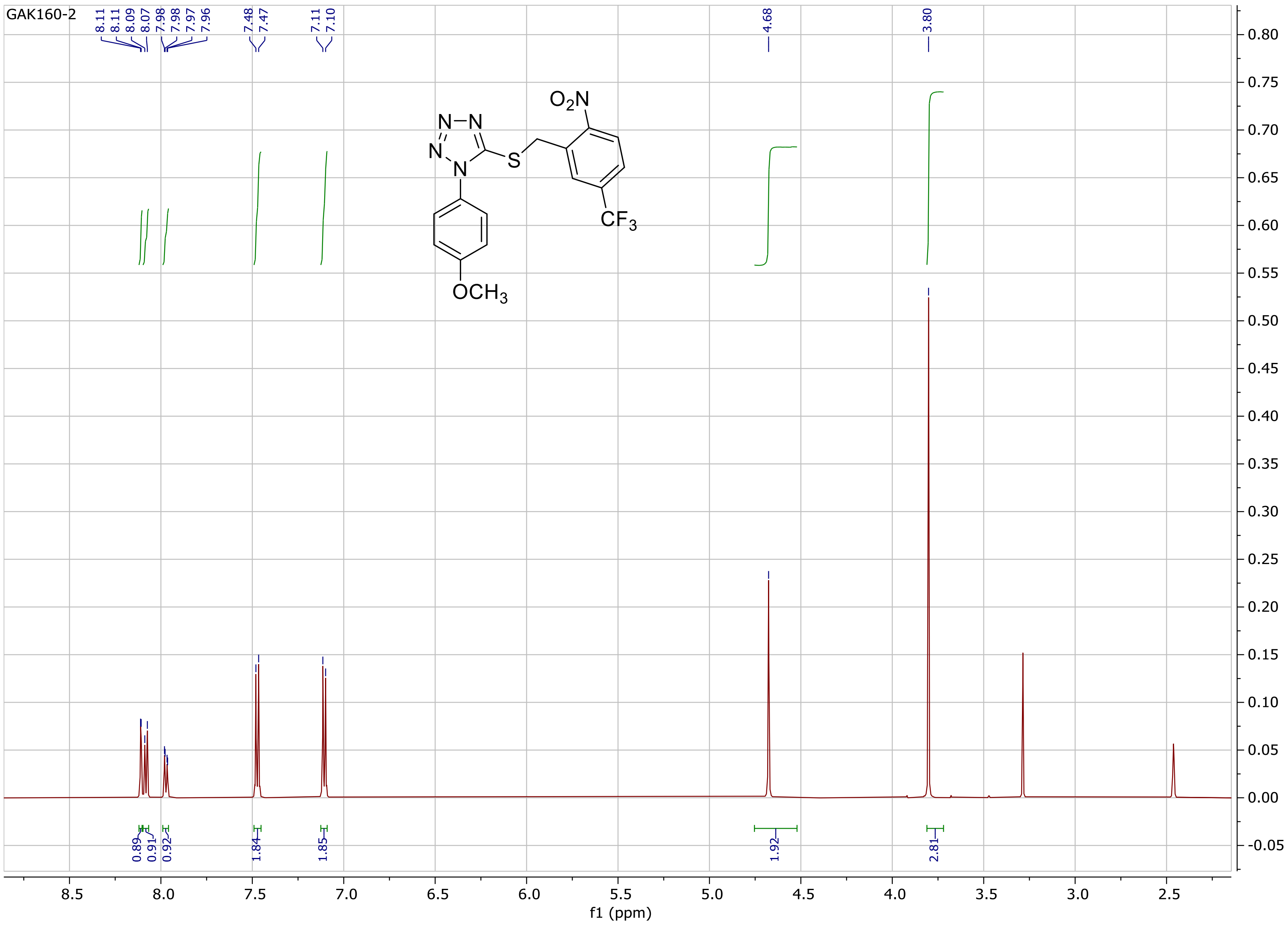

1-(4-Methoxyphenyl)-5-((2-nitro-5-(trifluoromethyl)benzyl)sulfanyl)-1H-tetrazole (**70b**): ^{13}C NMR (151 MHz, DMSO-d_6)

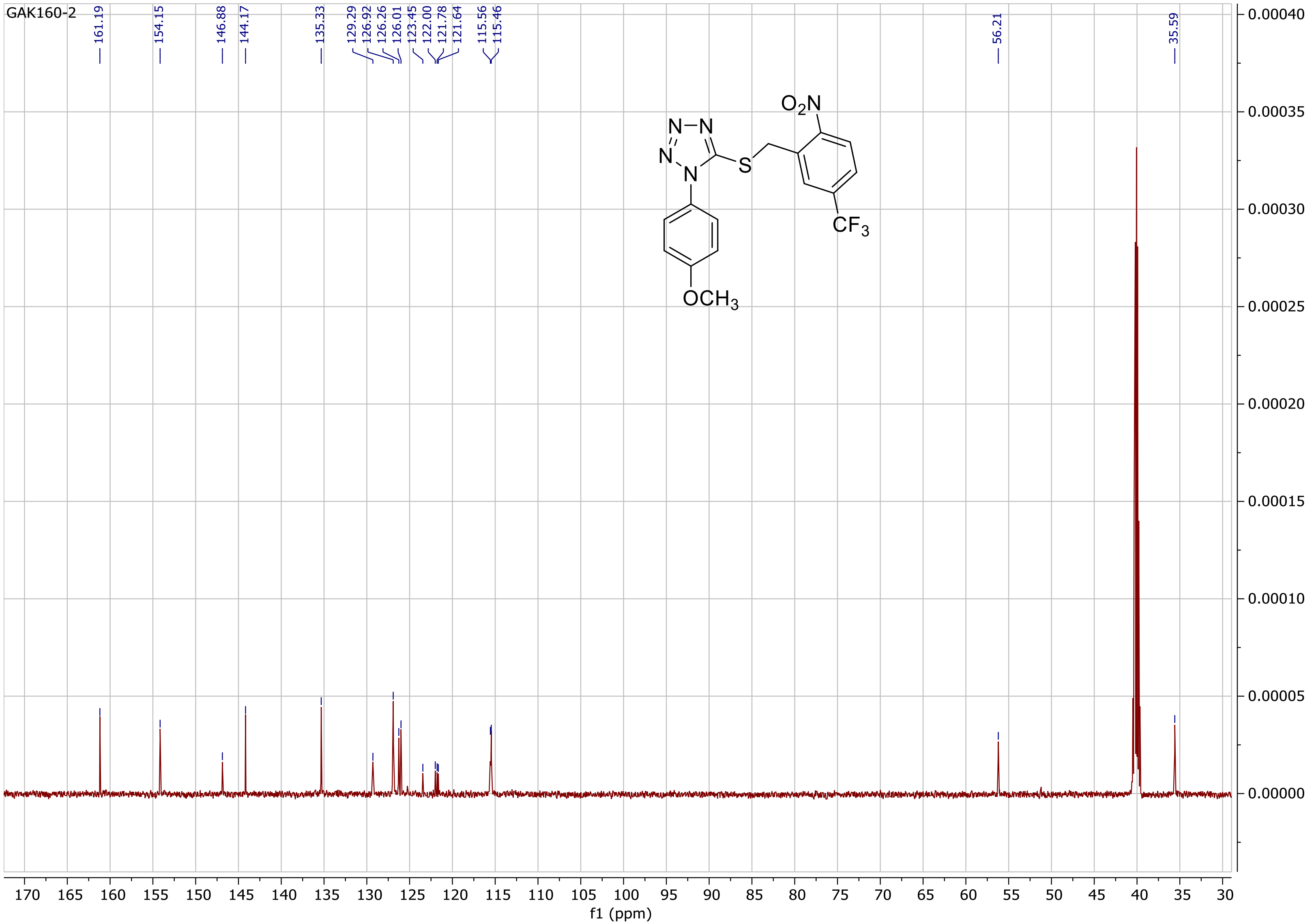

1-(4-Methoxyphenyl)-5-((2-nitro-5-(trifluoromethyl)benzyl)sulfanyl)-1H-tetrazole (**70b**):

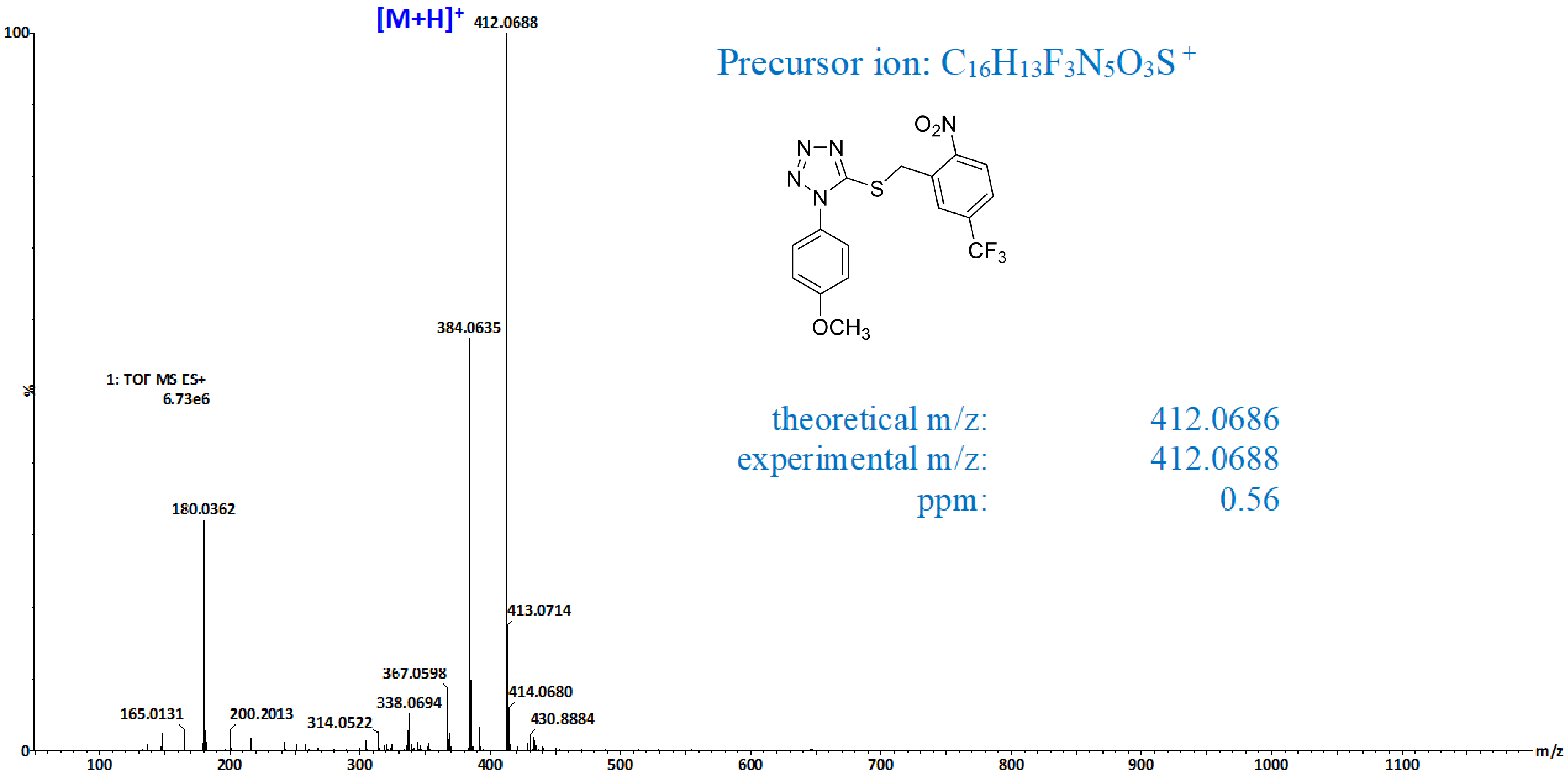

1-(4-Chlorophenyl)-5-((2-nitro-5-(trifluoromethyl)benzyl)sulfanyl)-1H-tetrazole (**70c**): ^{1}H NMR (600 MHz, DMSO-d_6)

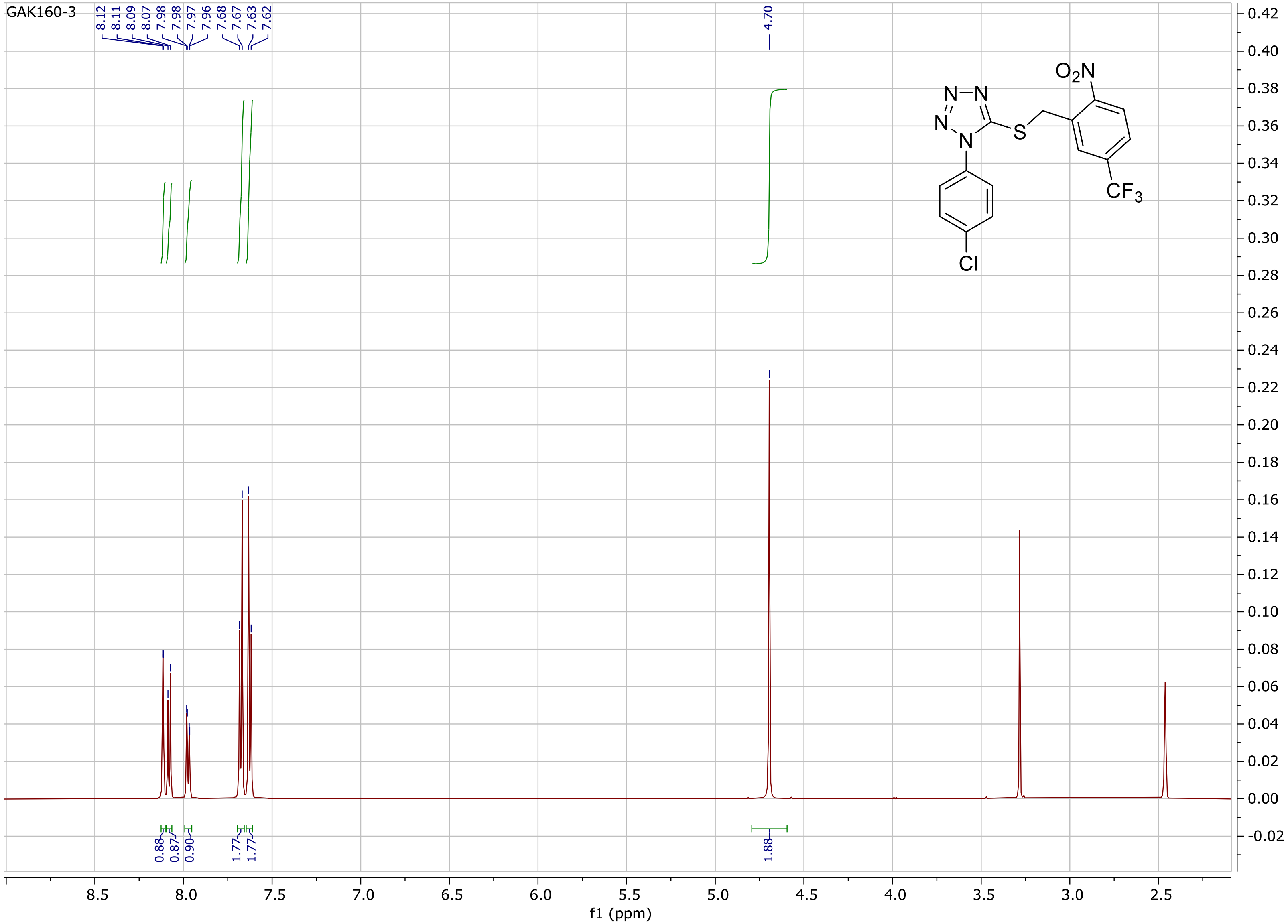

1-(4-Chlorophenyl)-5-((2-nitro-5-(trifluoromethyl)benzyl)sulfanyl)-1H-tetrazole (**70c**): ^{13}C NMR (151 MHz, DMSO-d_6)

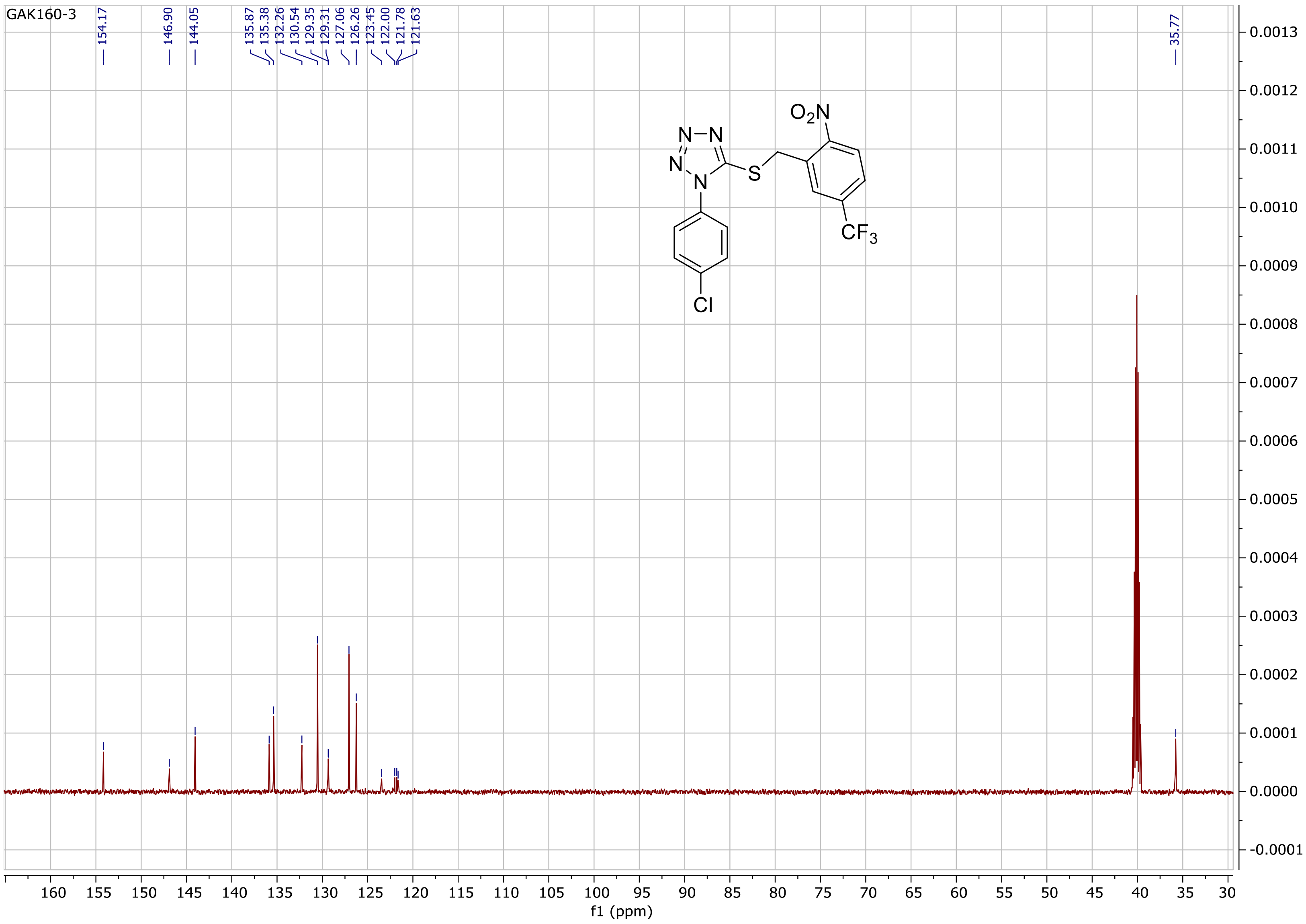

1-(4-Chlorophenyl)-5-((2-nitro-5-(trifluoromethyl)benzyl)sulfanyl)-1H-tetrazole (**70c**):

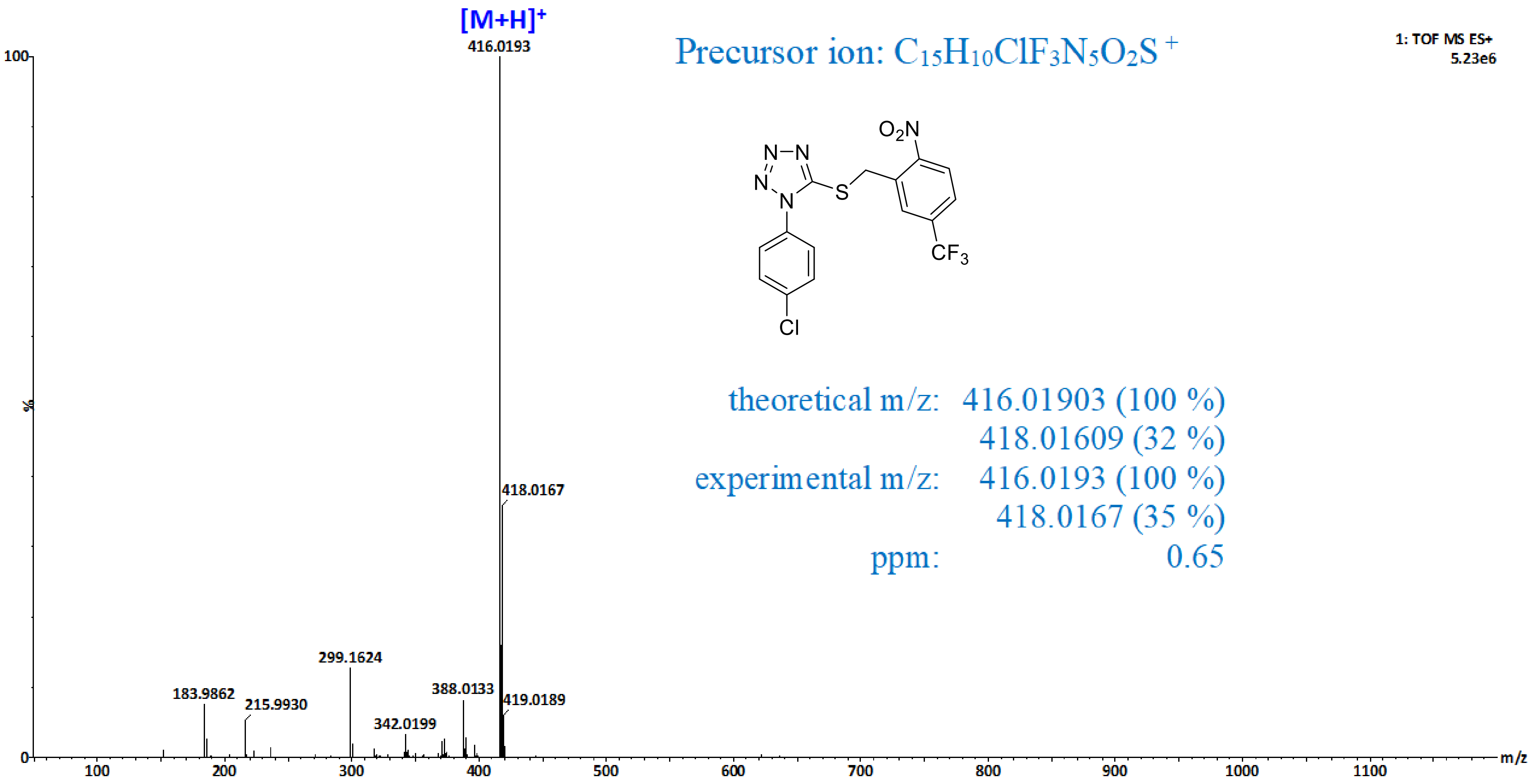

1-(4-Bromophenyl)-5-((2-nitro-5-(trifluoromethyl)benzyl)sulfanyl)-1H-tetrazole (**70d**): ^{1}H NMR (500 MHz, DMSO-d_6)

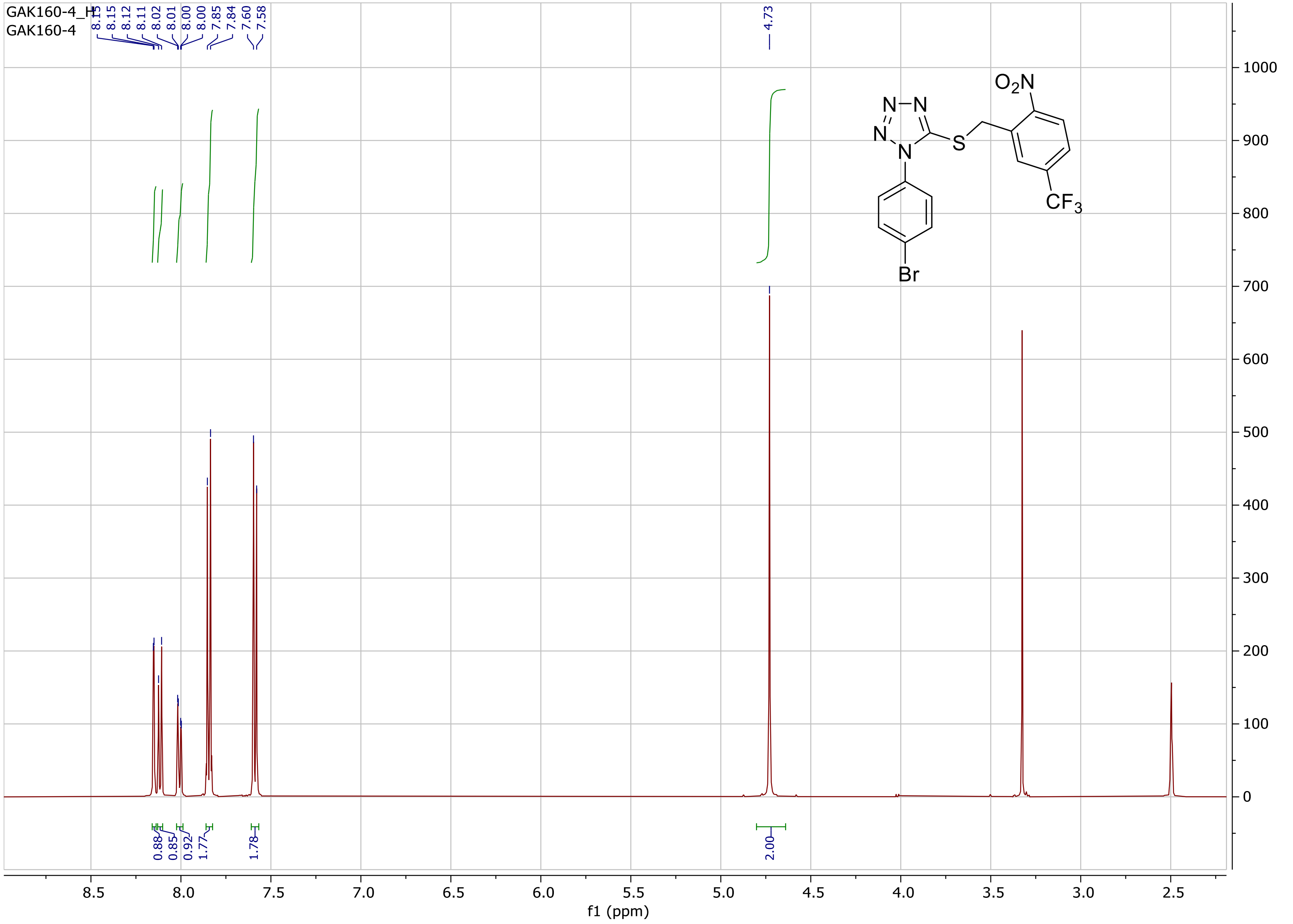

1-(4-Bromophenyl)-5-((2-nitro-5-(trifluoromethyl)benzyl)sulfanyl)-1H-tetrazole (**70d**): ^{13}C NMR (126 MHz, DMSO-d_6)

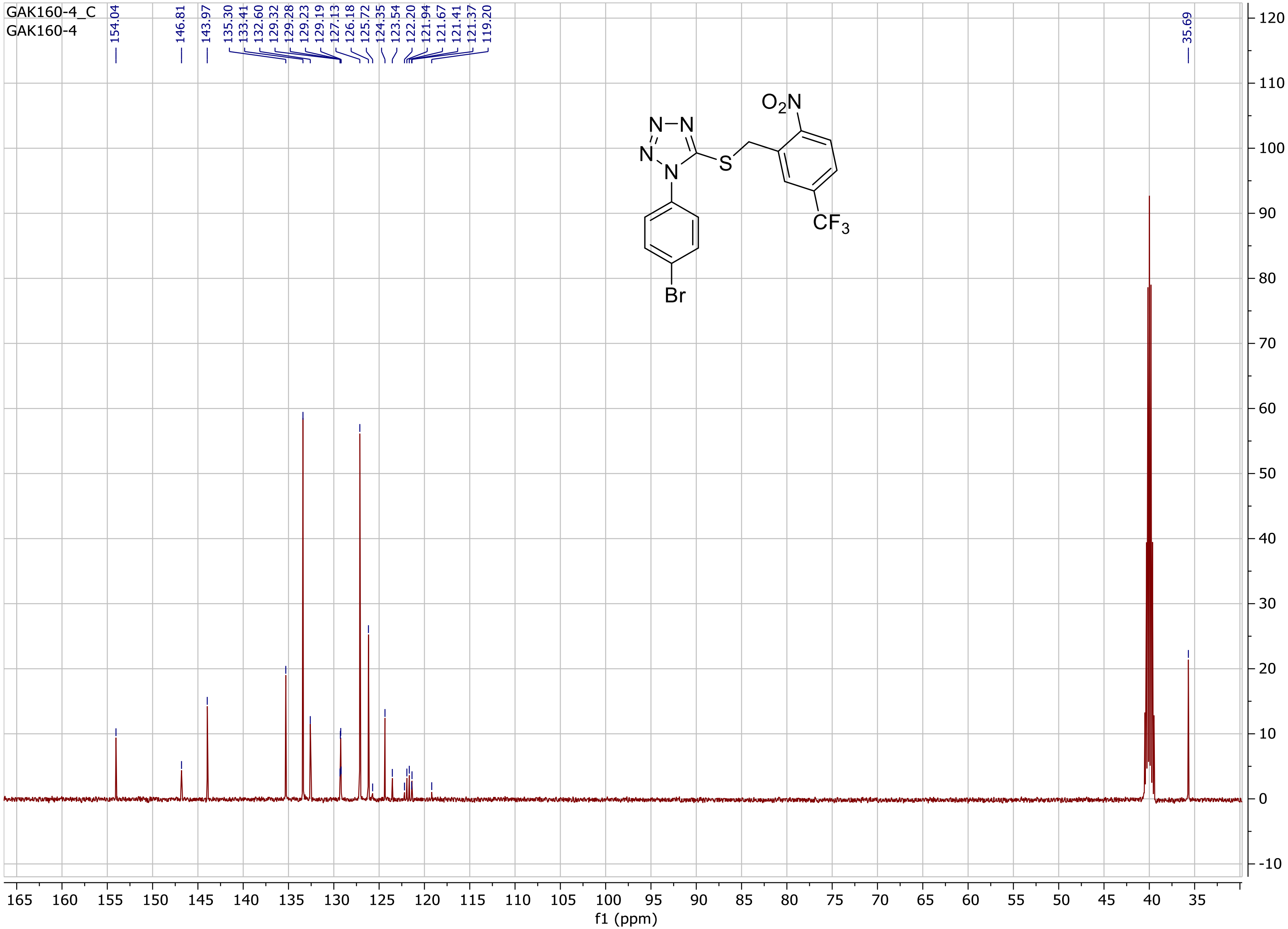

1-(4-Bromophenyl)-5-((2-nitro-5-(trifluoromethyl)benzyl)sulfanyl)-1H-tetrazole (**70d**):

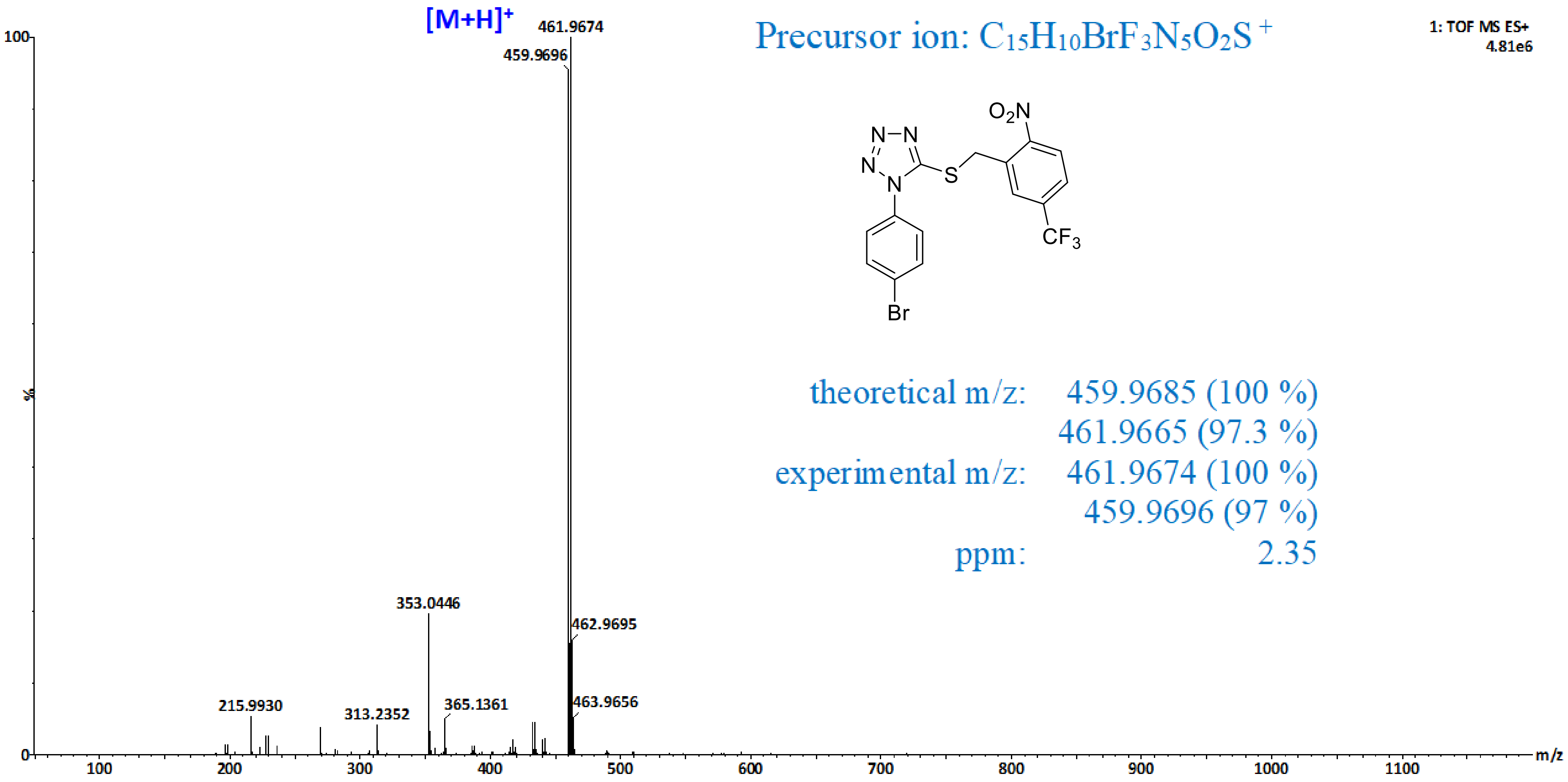

1-Cyclohexyl-5-((2-nitro-5-(trifluoromethyl)benzyl)sulfanyl)-1H-tetrazole (**70e**): ^{1}H NMR (500 MHz, DMSO-d_6)

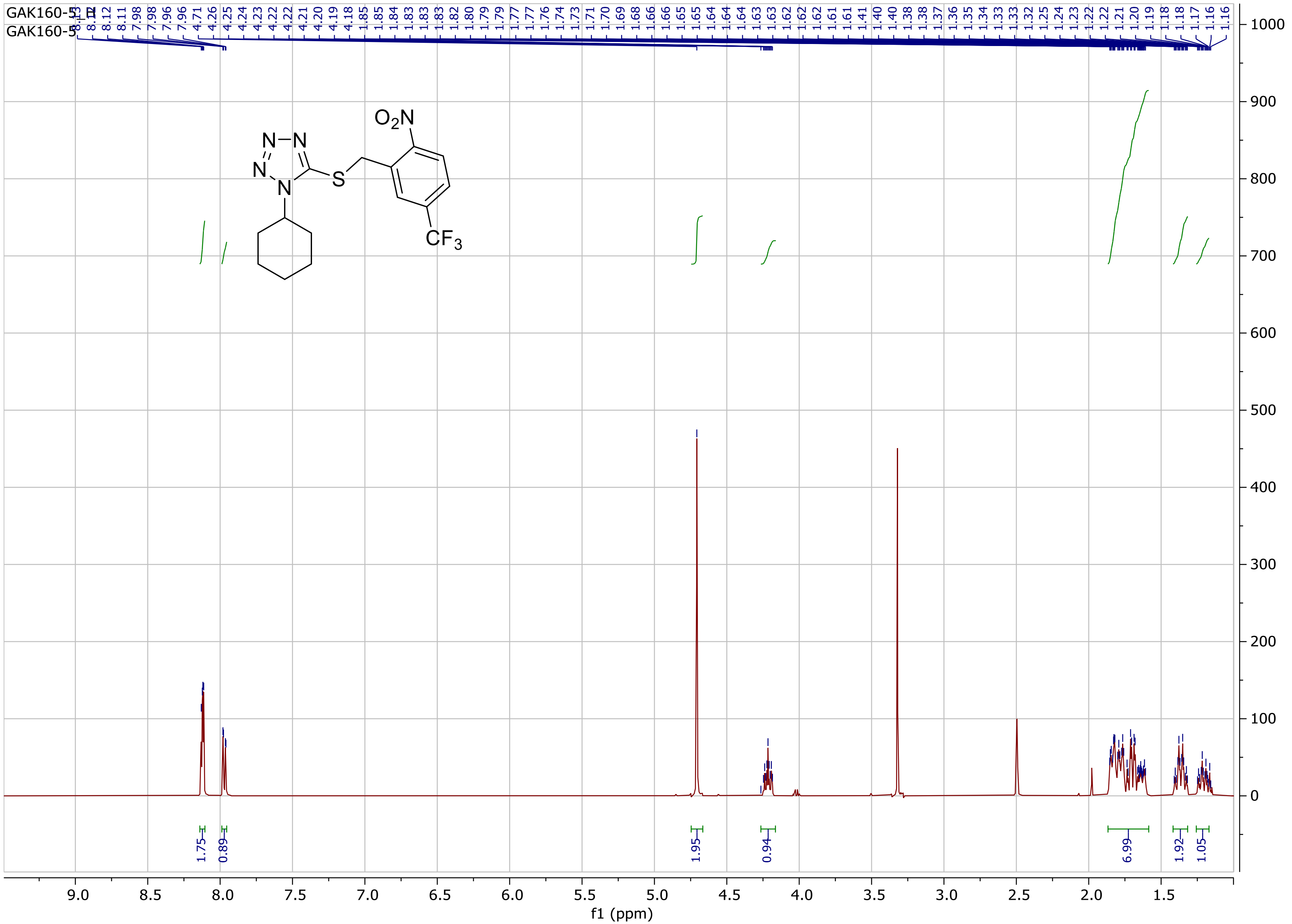

1-Cyclohexyl-5-((2-nitro-5-(trifluoromethyl)benzyl)sulfanyl)-1H-tetrazole (**70e**): ^{13}C NMR (126 MHz, DMSO-d_6)

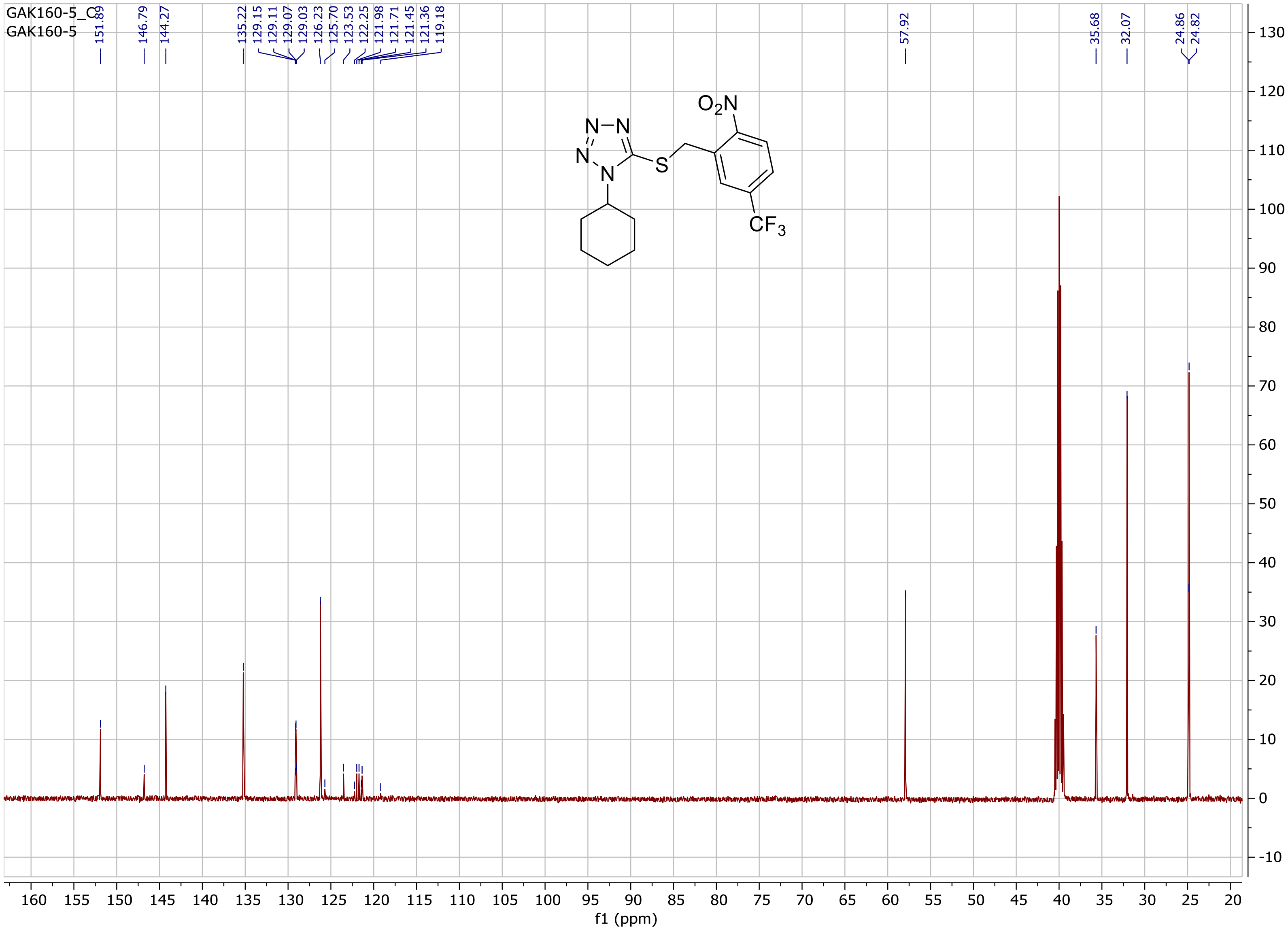

1-Cyclohexyl-5-((2-nitro-5-(trifluoromethyl)benzyl)sulfanyl)-1H-tetrazole (**70e**):

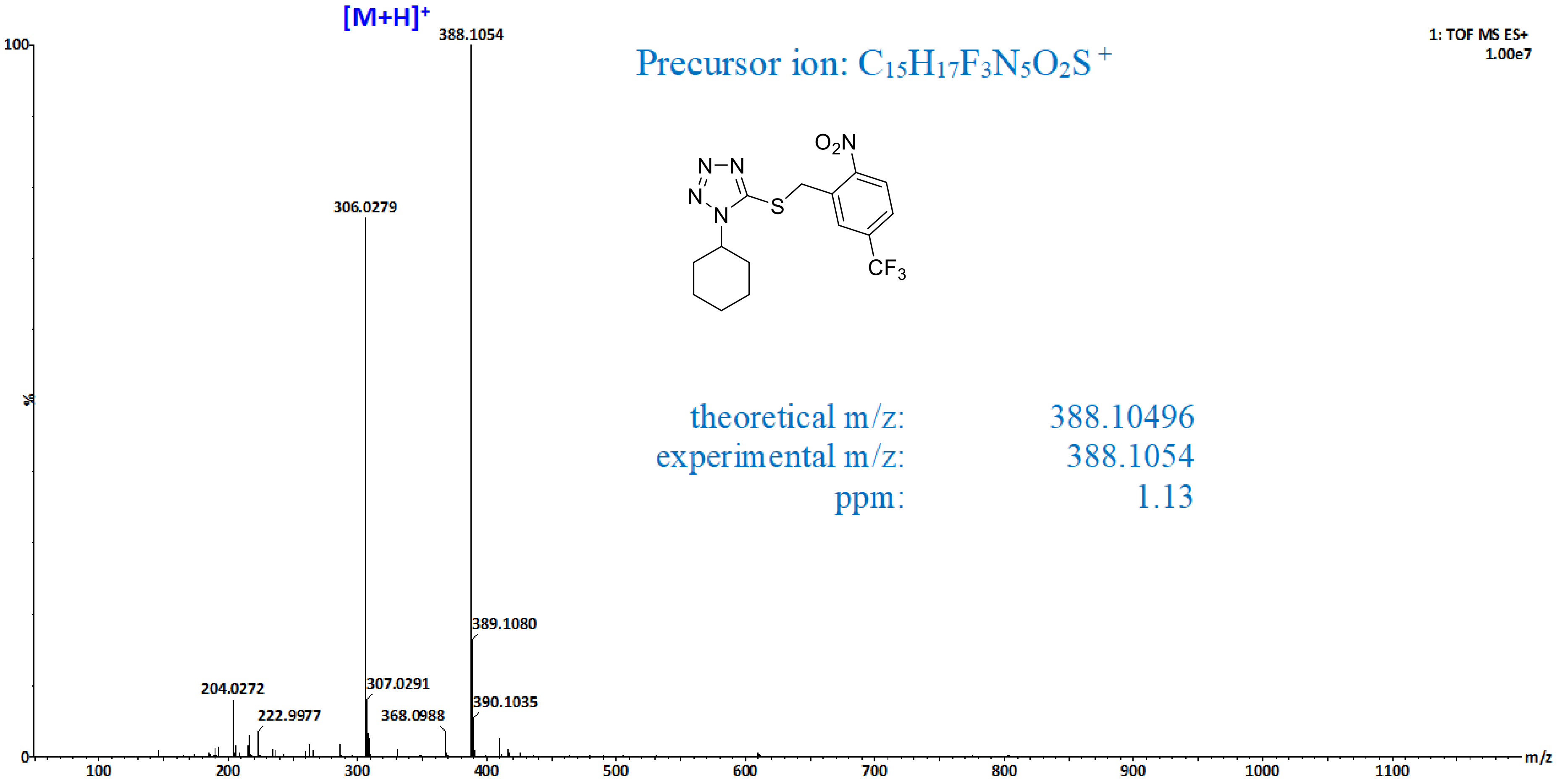

5-((5-Nitro-2-(trifluoromethyl)benzyl)sulfanyl)-1-phenyl-1H-tetrazole (**71a**): ^{1}H NMR (600 MHz, DMSO-d_6)

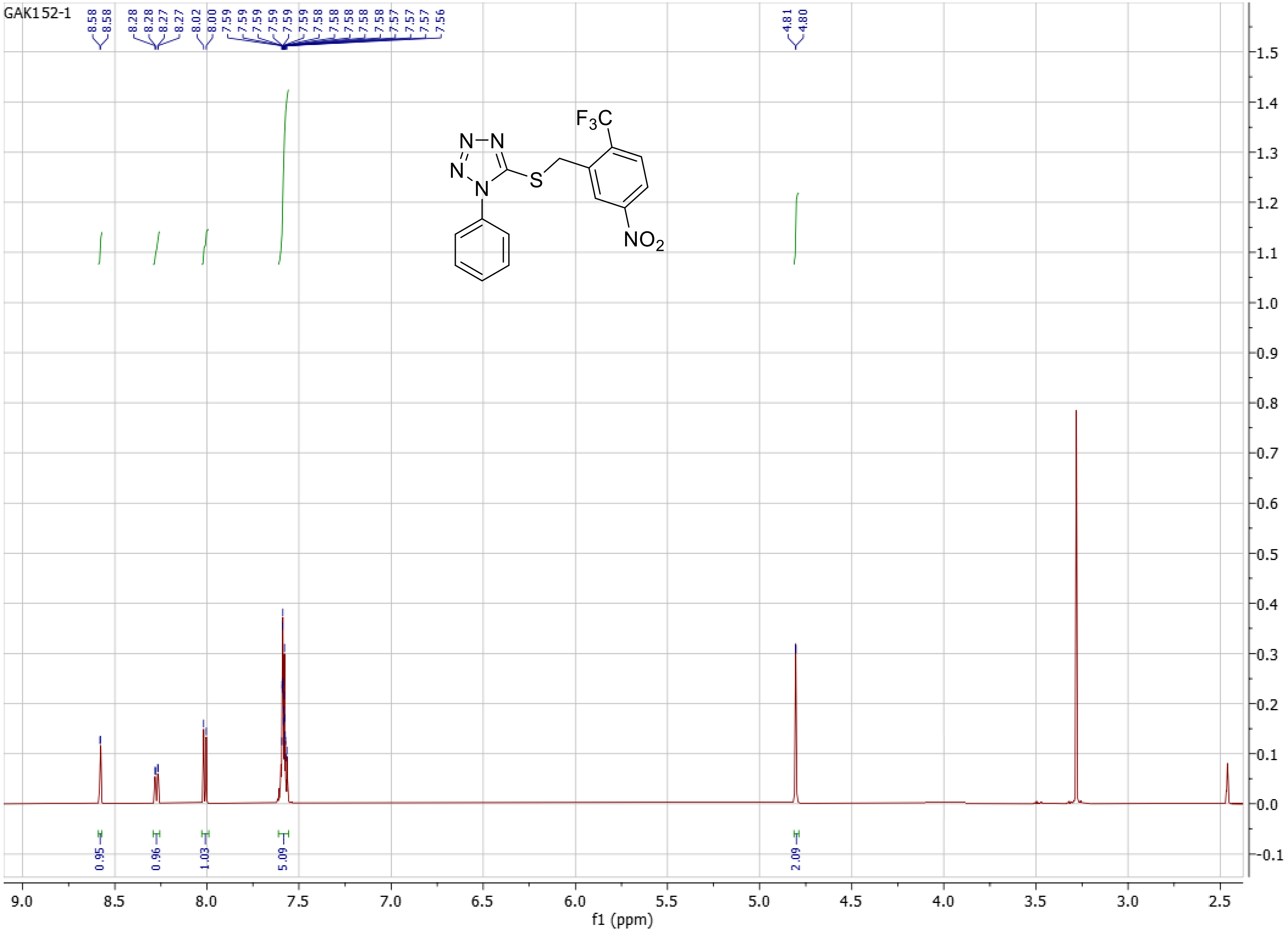

5-((5-Nitro-2-(trifluoromethyl)benzyl)sulfanyl)-1-phenyl-1H-tetrazole (**71a**): ^{13}C NMR (151 MHz, DMSO-d_6)

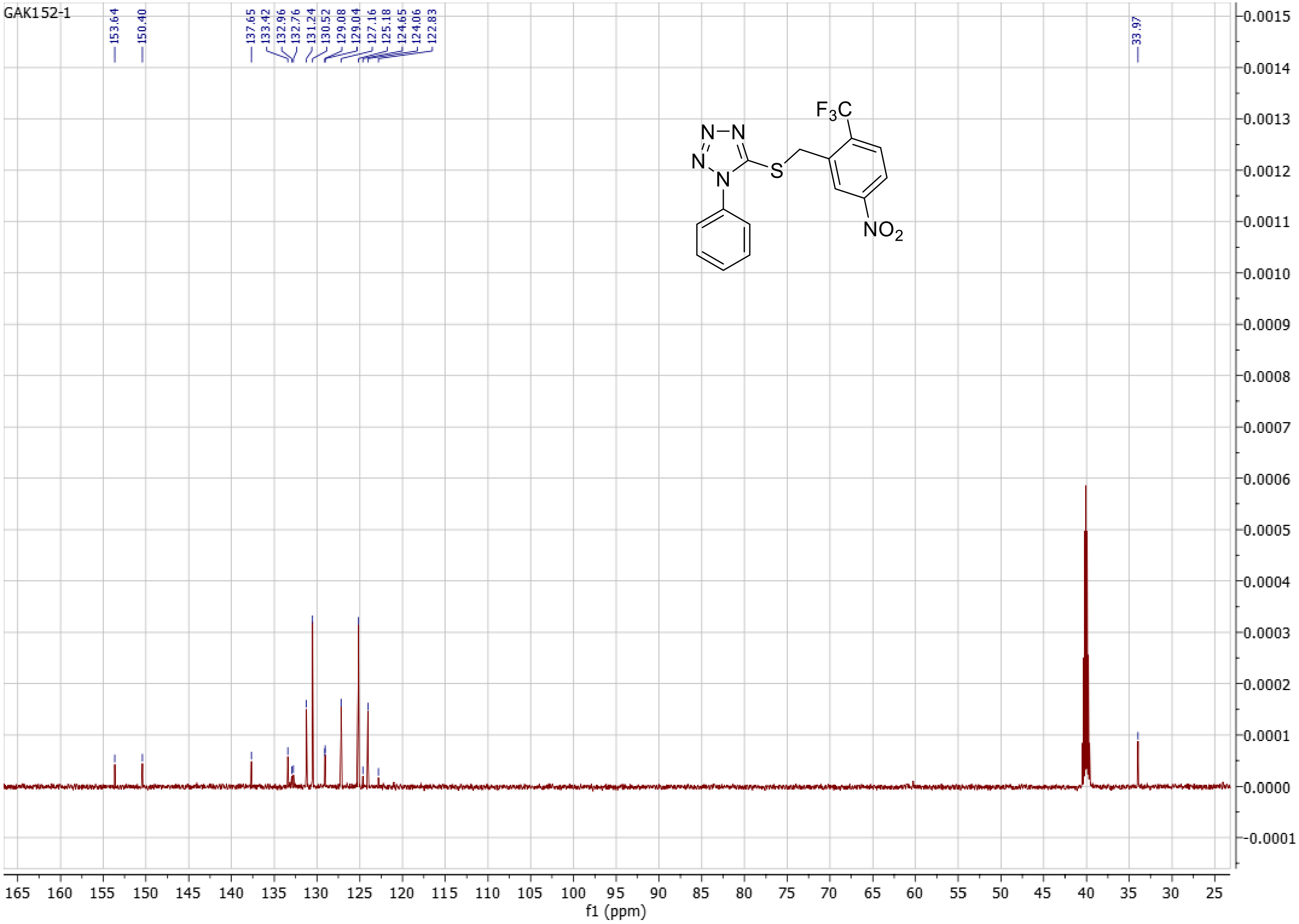

5-((5-Nitro-2-(trifluoromethyl)benzyl)sulfanyl)-1-phenyl-1H-tetrazole (**71a**):

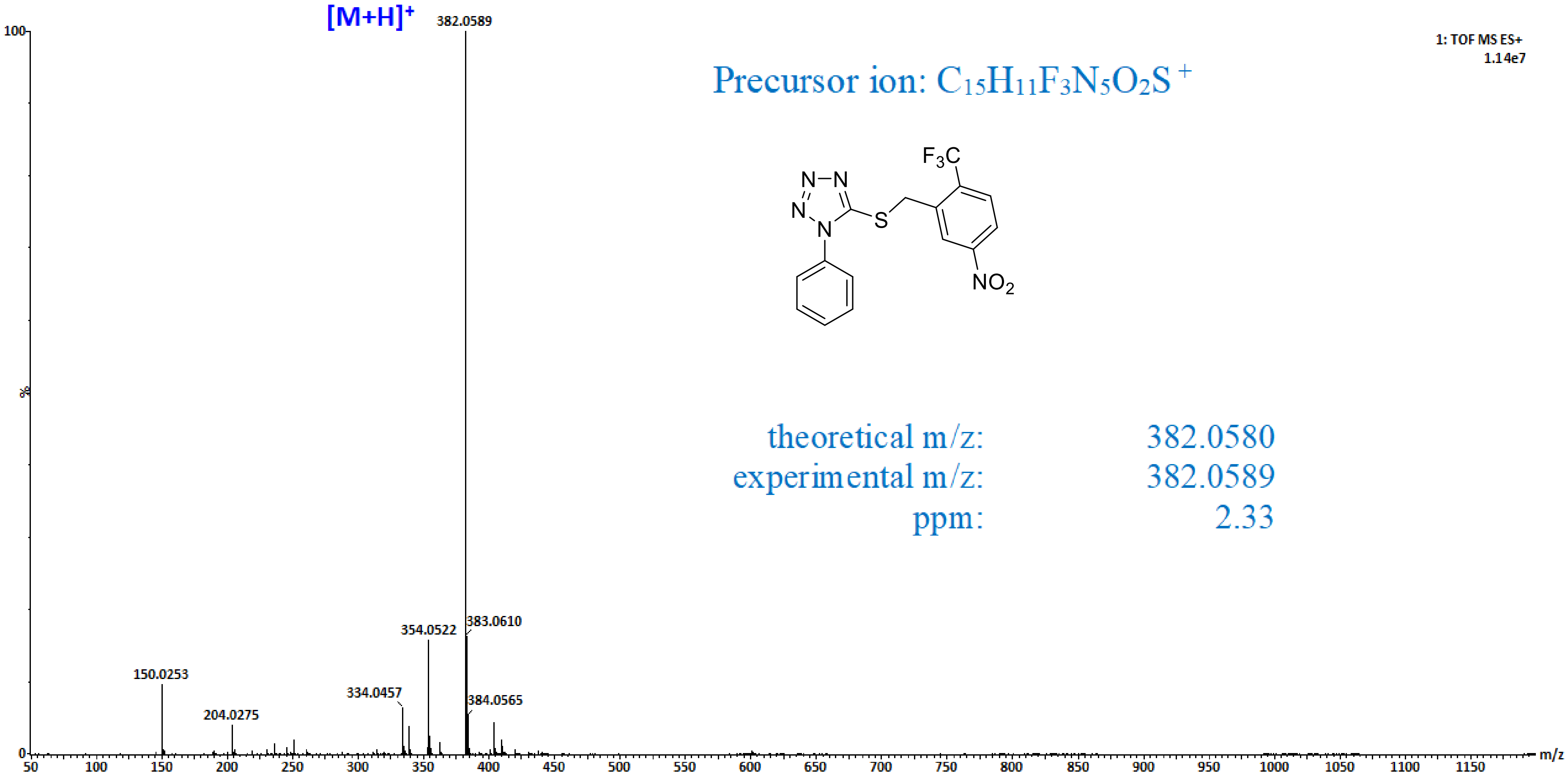

1-(4-Methoxyphenyl)-5-((5-nitro-2-(trifluoromethyl)benzyl)sulfanyl)-1H-tetrazole (**71b**): ^{1}H NMR (600 MHz, DMSO-d_6)

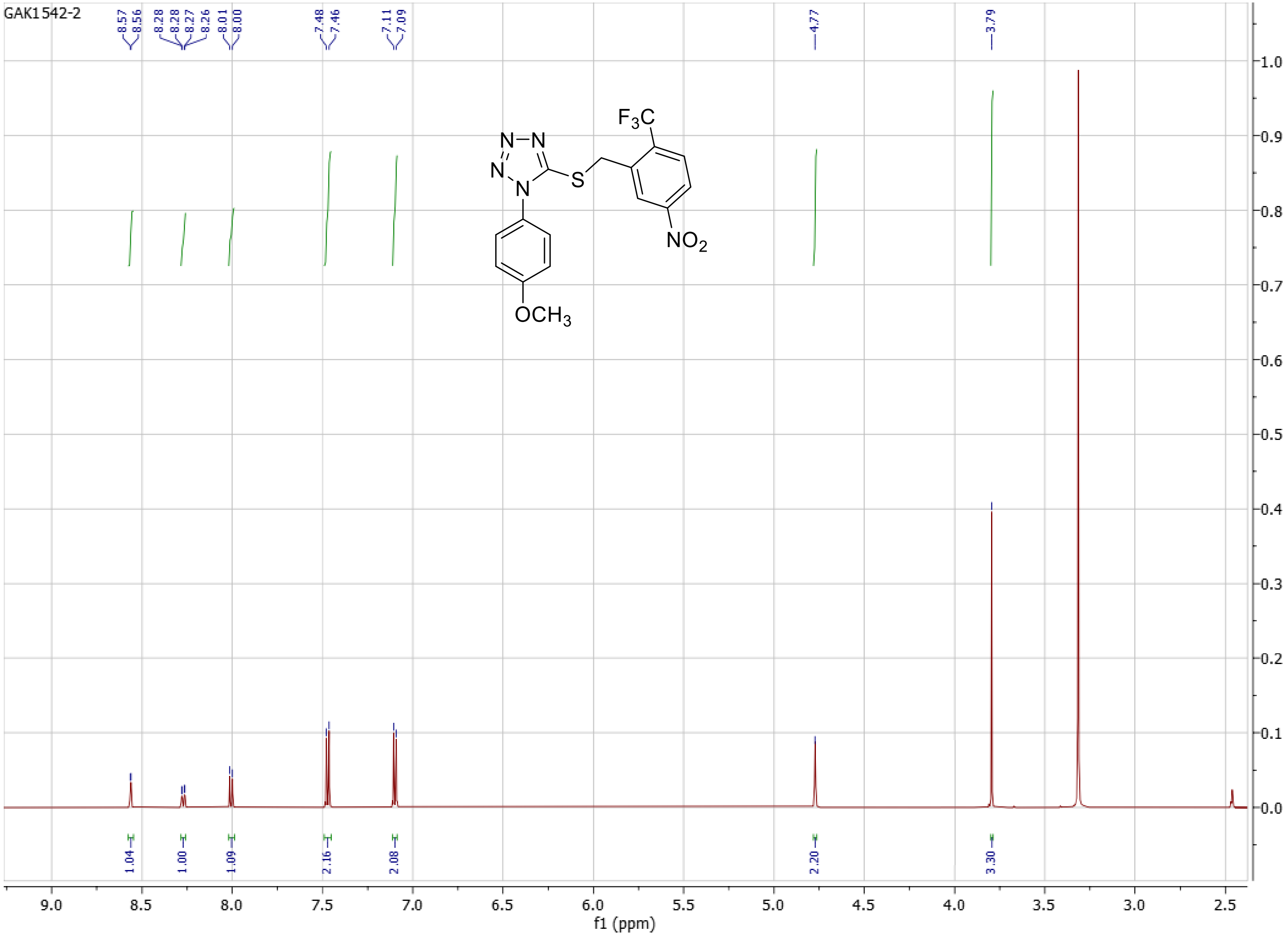

1-(4-Methoxyphenyl)-5-((5-nitro-2-(trifluoromethyl)benzyl)sulfanyl)-1H-tetrazole (**71b**): ^{13}C NMR (151 MHz, DMSO-d_6)

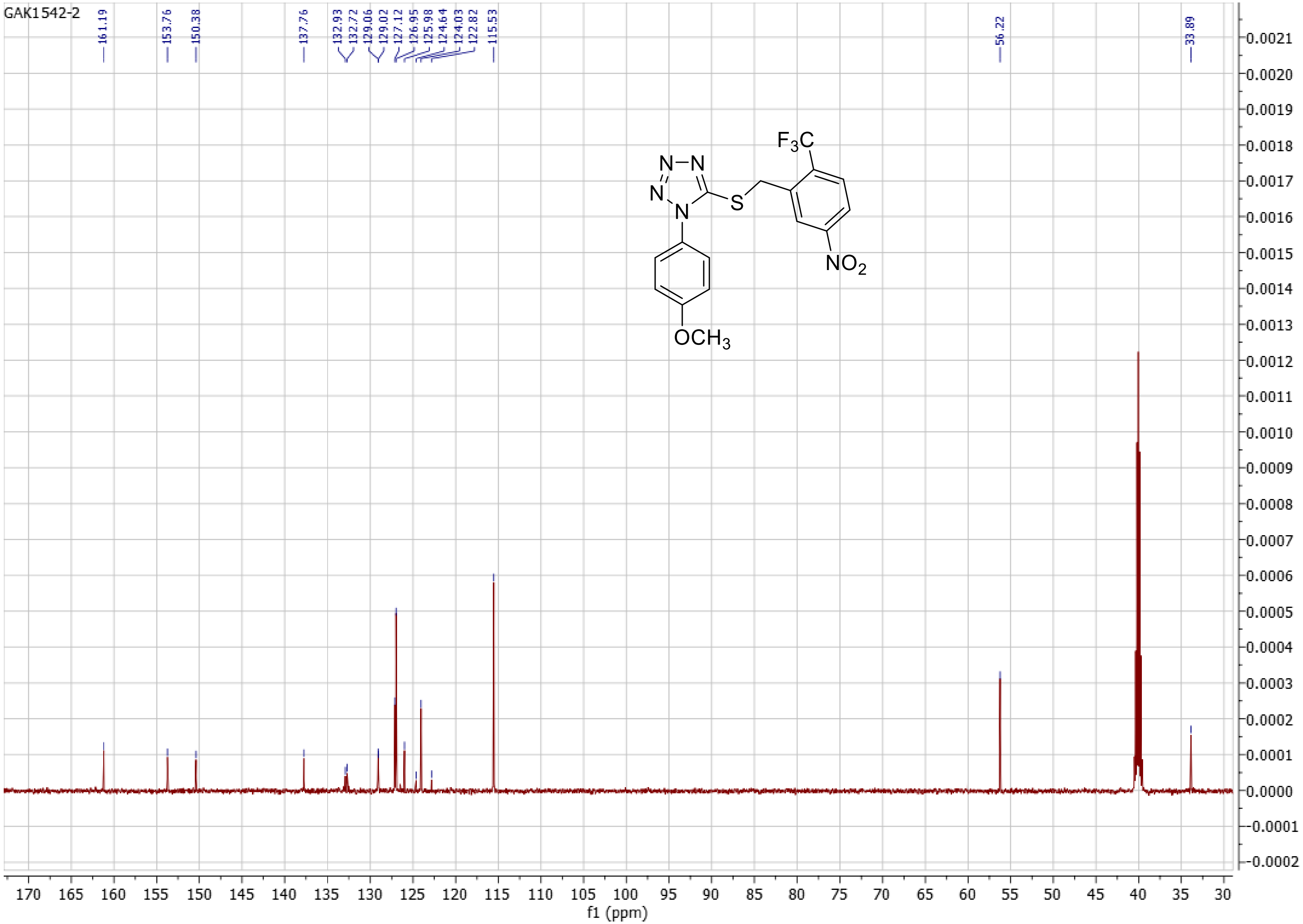

1-(4-Methoxyphenyl)-5-((5-nitro-2-(trifluoromethyl)benzyl)sulfanyl)-1H-tetrazole (**71b**):

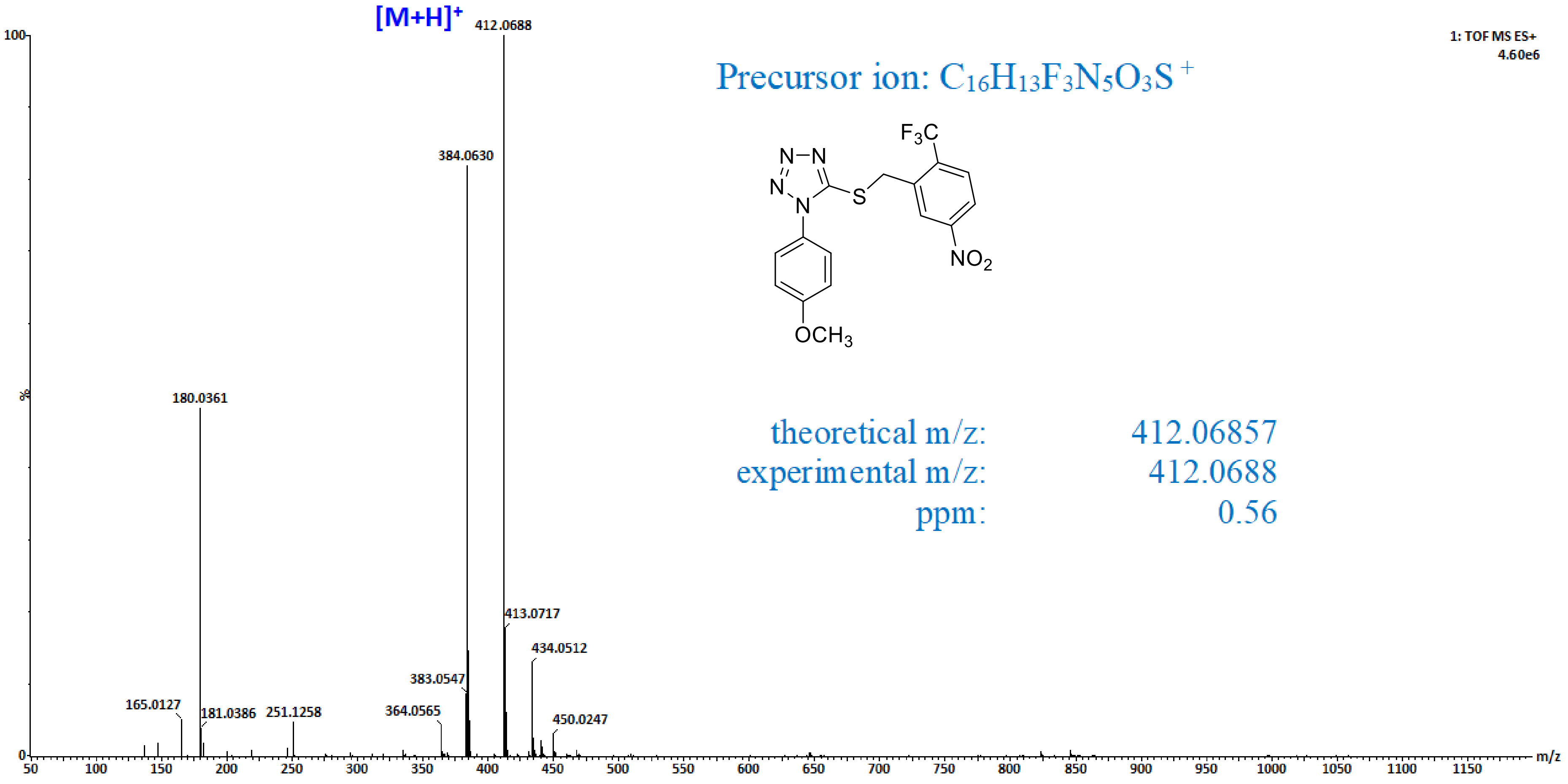

1-(4-Chlorophenyl)-5-((5-nitro-2-(trifluoromethyl)benzyl)sulfanyl)-1H-tetrazole (**71c**): ^{1}H NMR (500 MHz, DMSO-d_6)

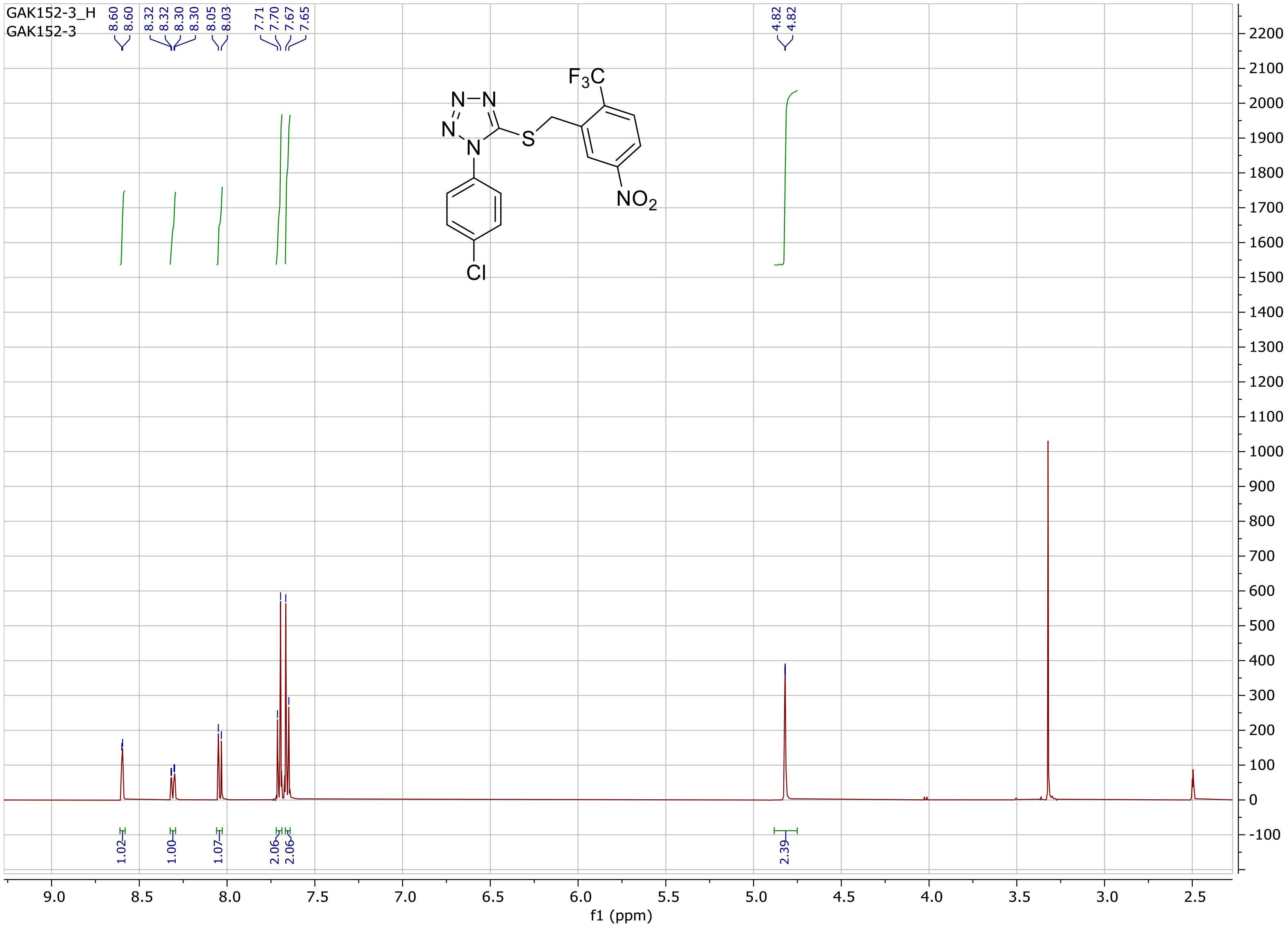

1-(4-Chlorophenyl)-5-((5-nitro-2-(trifluoromethyl)benzyl)sulfanyl)-1H-tetrazole (**71c**): ^{13}C NMR (126 MHz, DMSO-d_6)

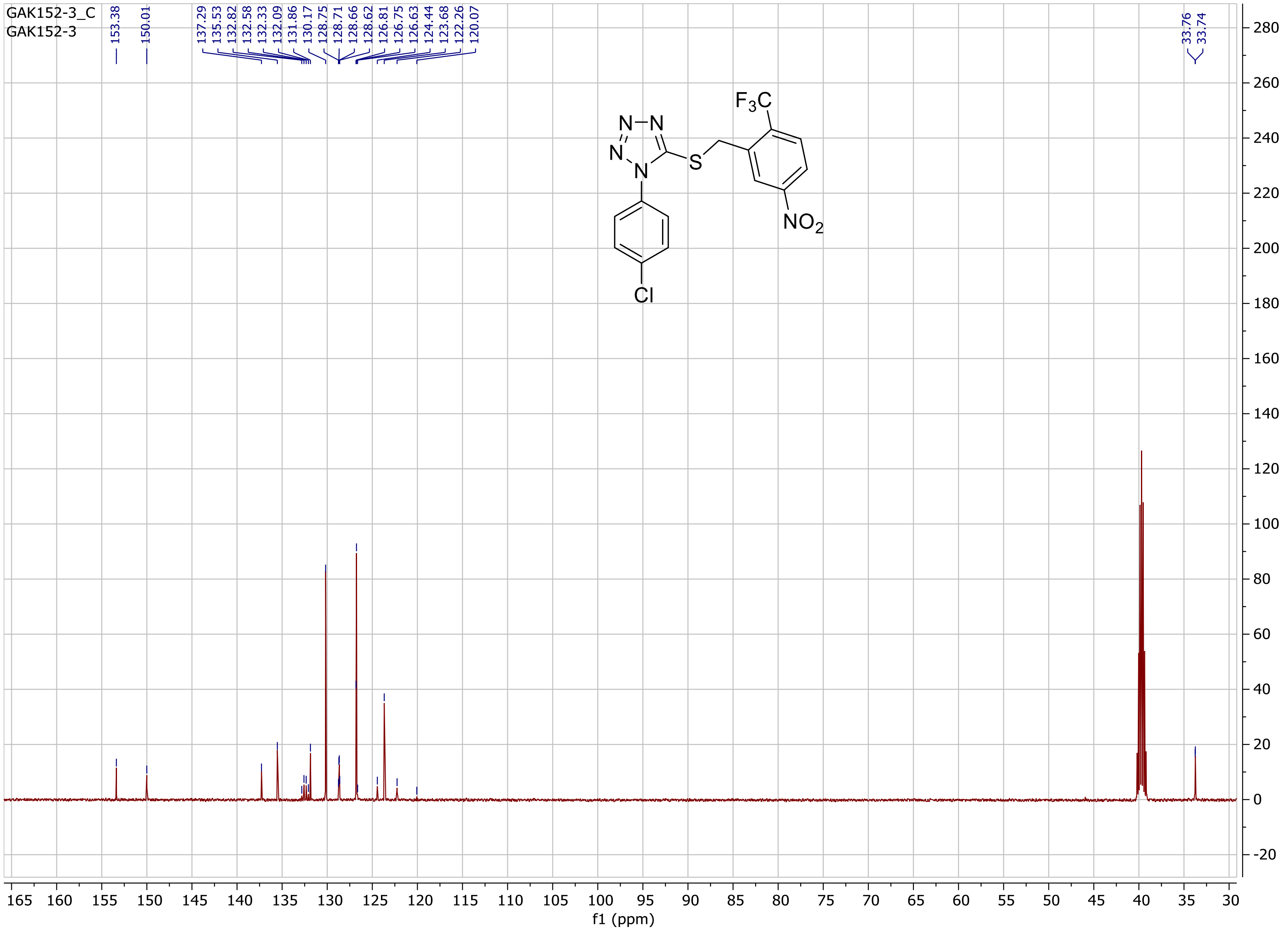

1-(4-Chlorophenyl)-5-((5-nitro-2-(trifluoromethyl)benzyl)sulfanyl)-1H-tetrazole (**71c**):

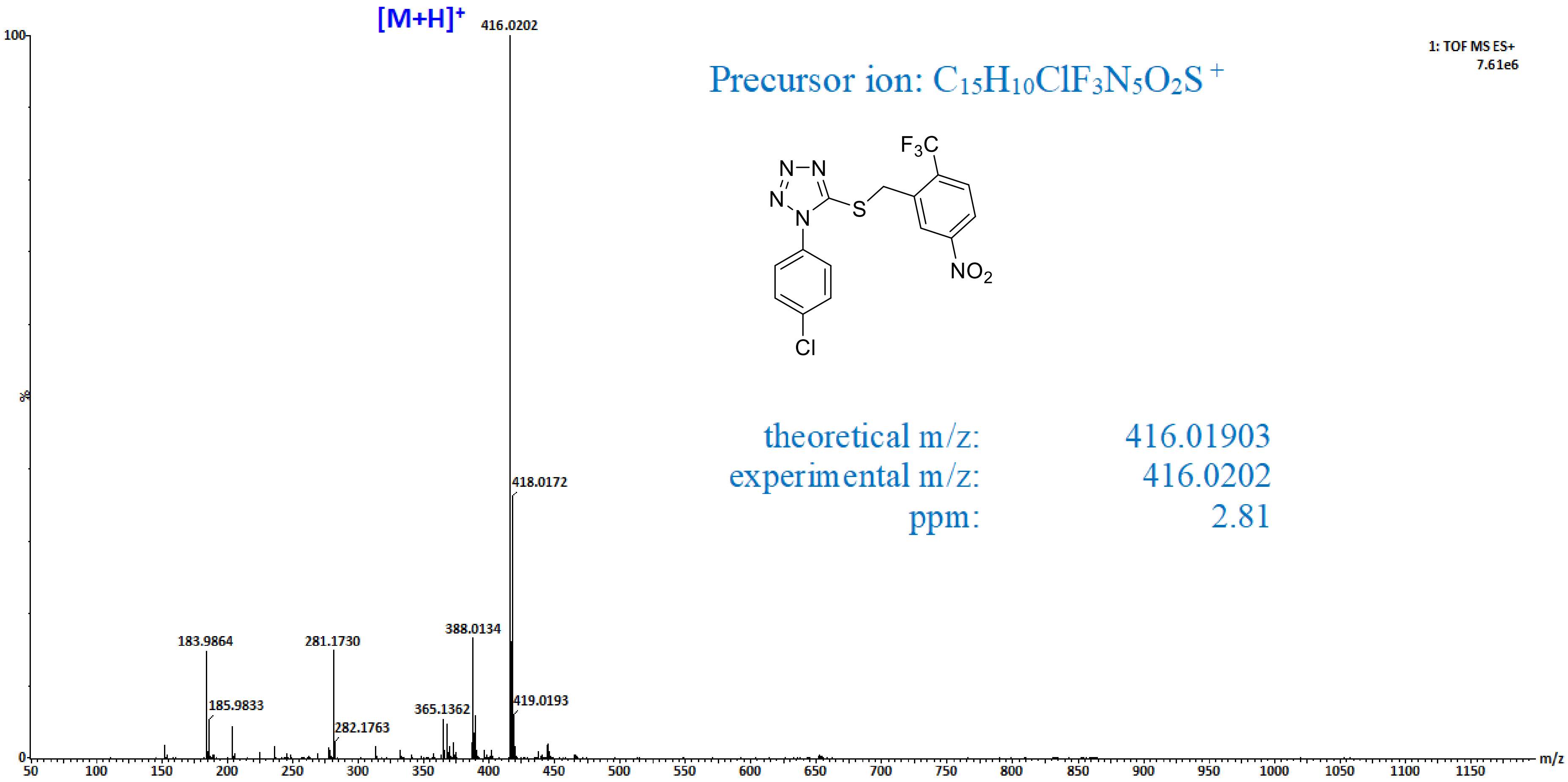

1-(4-Bromophenyl)-5-((5-nitro-2-(trifluoromethyl)benzyl)sulfanyl)-1H-tetrazole (**71d**): ^{1}H NMR (500 MHz, DMSO-d_6)

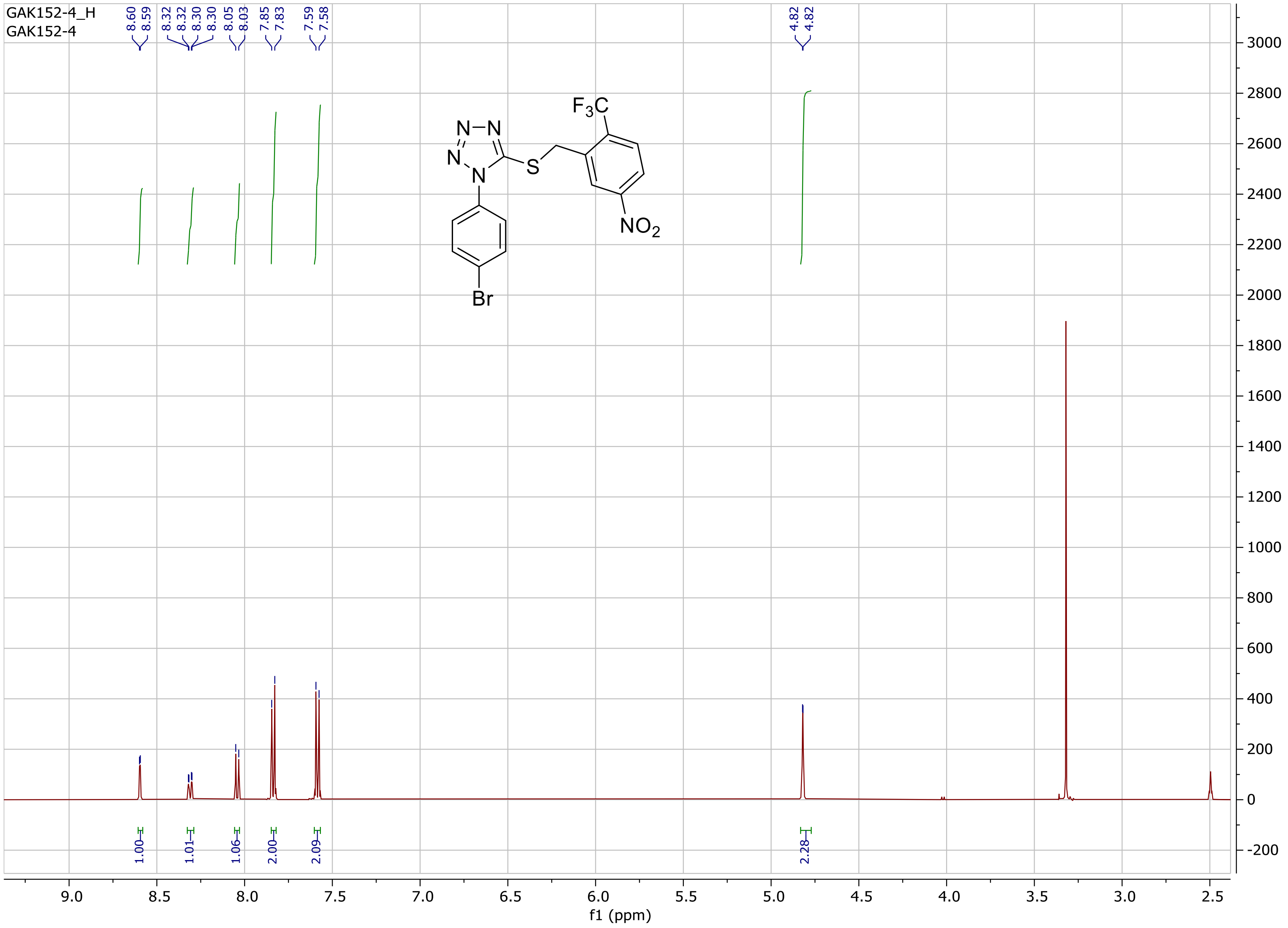

1-(4-Bromophenyl)-5-((5-nitro-2-(trifluoromethyl)benzyl)sulfanyl)-1H-tetrazole (**71d**): ^{13}C NMR (126 MHz, DMSO-d_6)

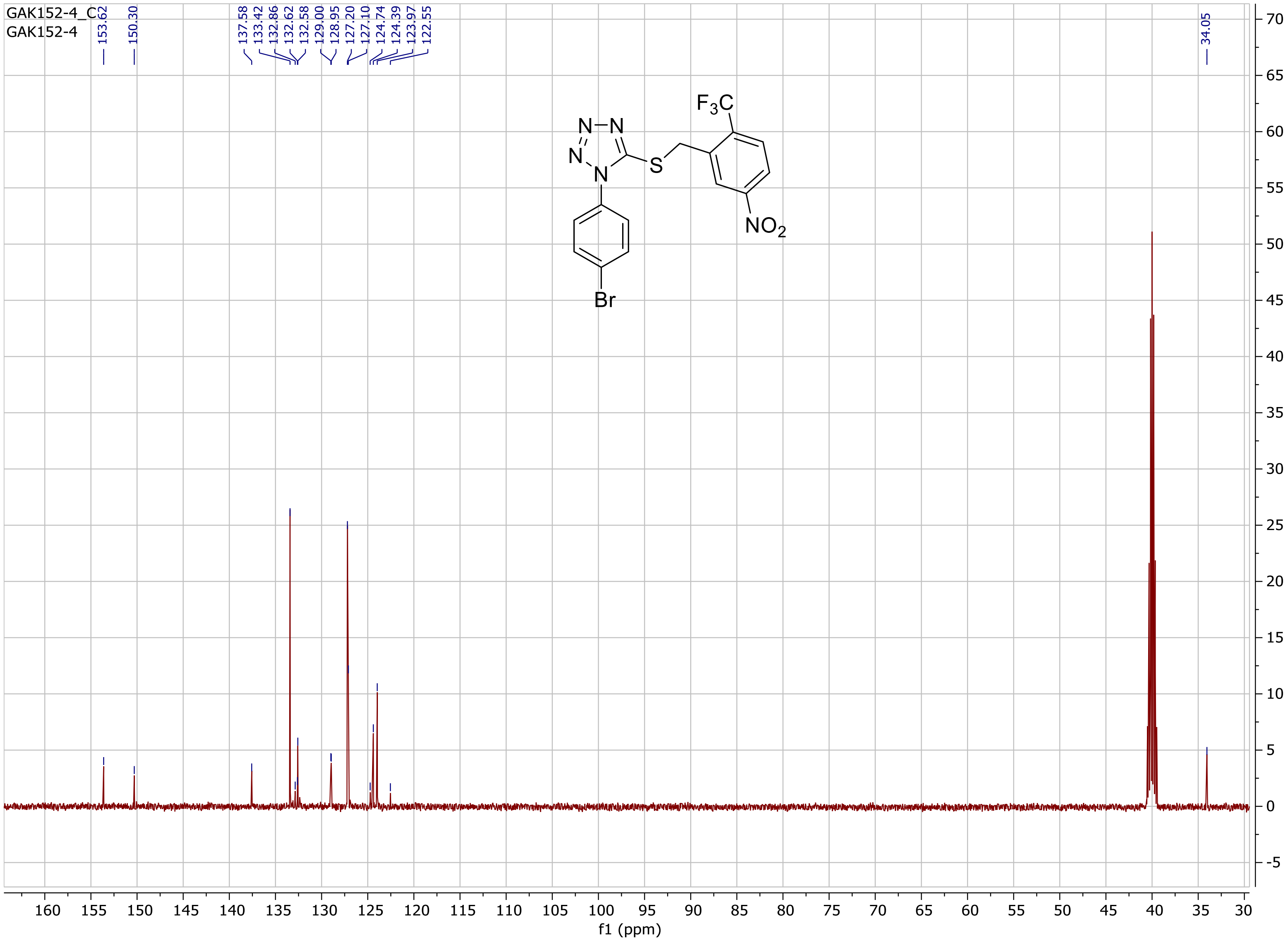

1-(4-Bromophenyl)-5-((5-nitro-2-(trifluoromethyl)benzyl)sulfanyl)-1H-tetrazole (**71d**):

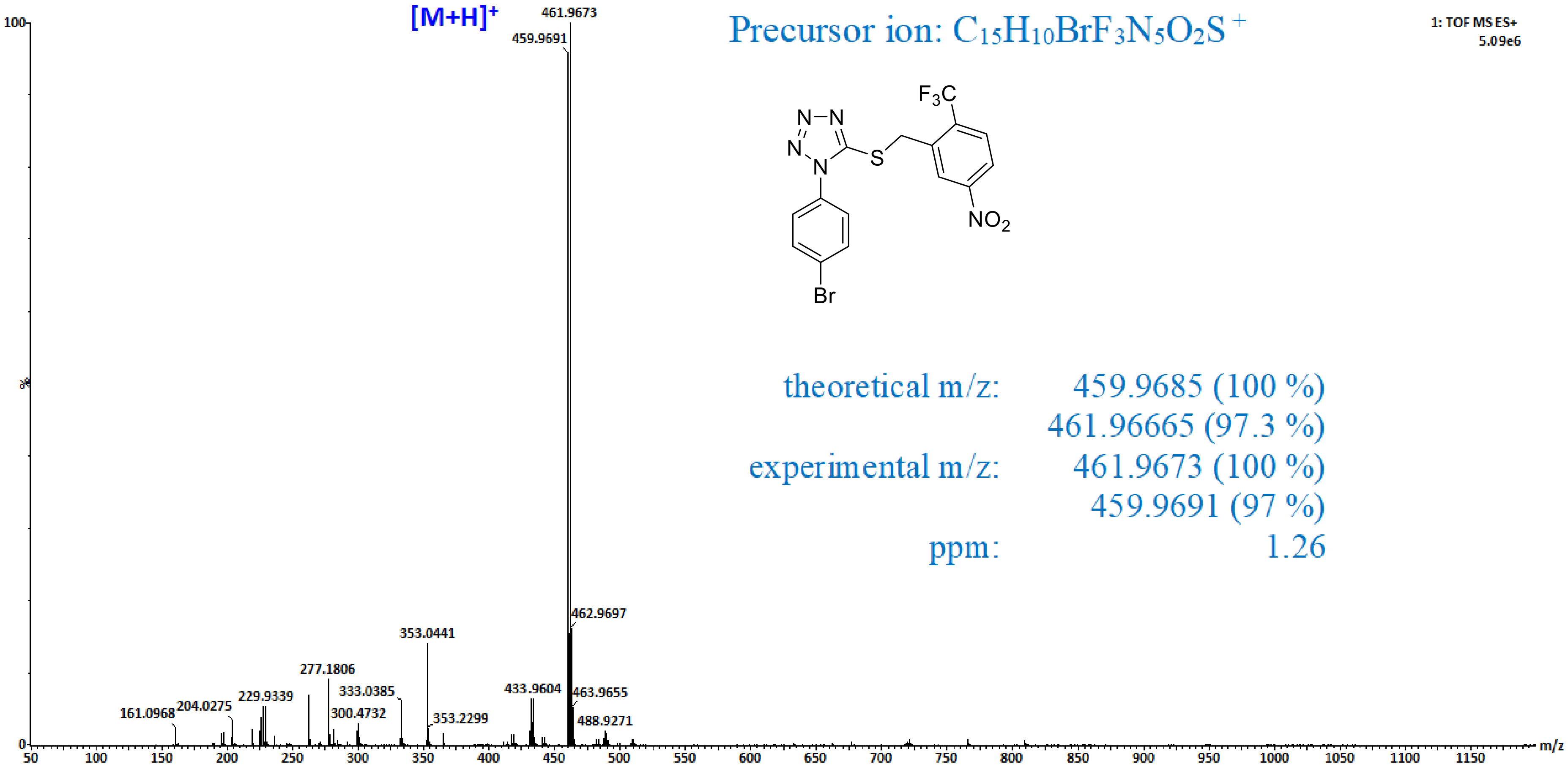

1-Cyclohexyl-5-((5-nitro-2-(trifluoromethyl)benzyl)sulfanyl)-1H-tetrazole (**71e**): ^{1}H NMR (500 MHz, DMSO-d_6)

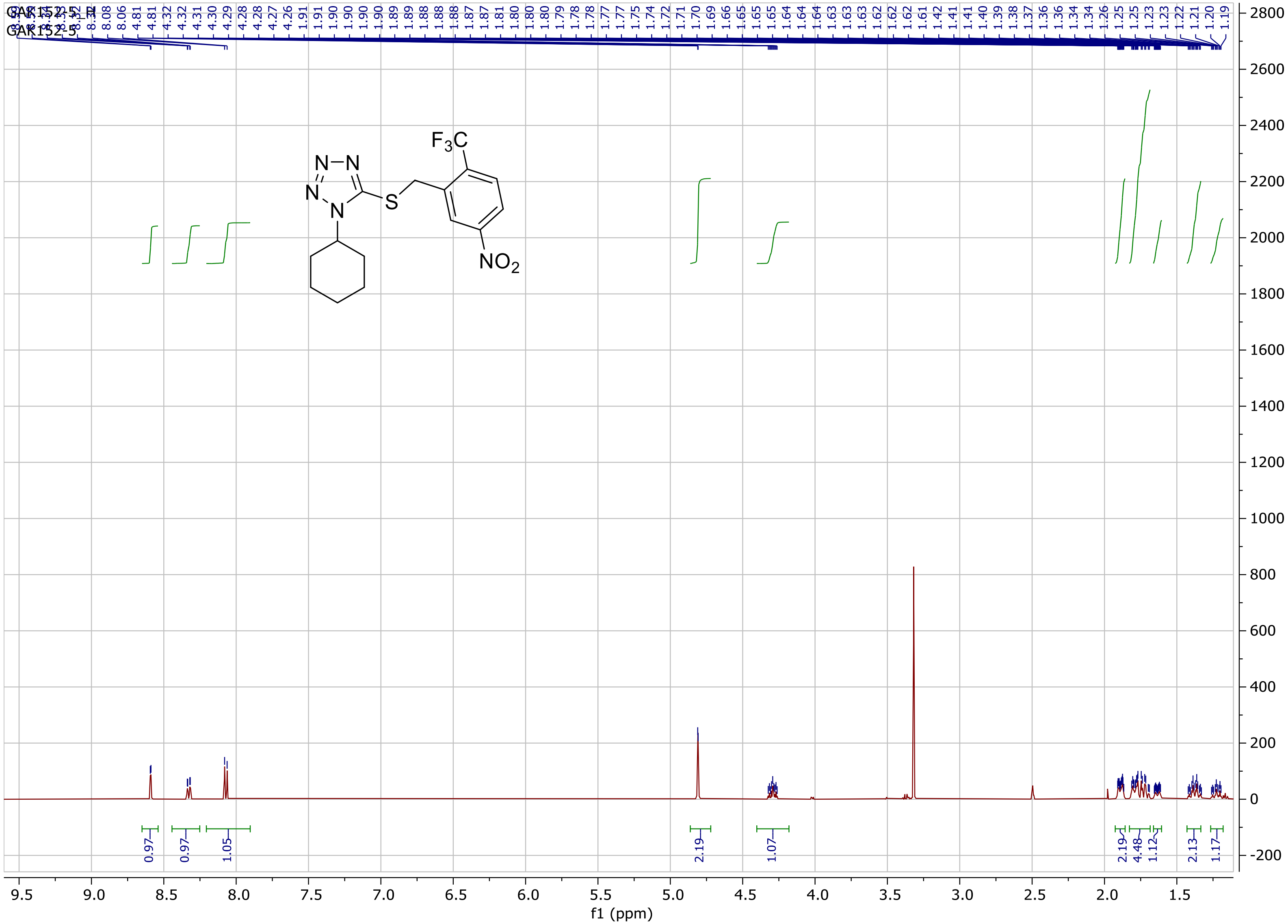

1-Cyclohexyl-5-((5-nitro-2-(trifluoromethyl)benzyl)sulfanyl)-1H-tetrazole (**71e**): ^{13}C NMR (126 MHz, DMSO-d_6)

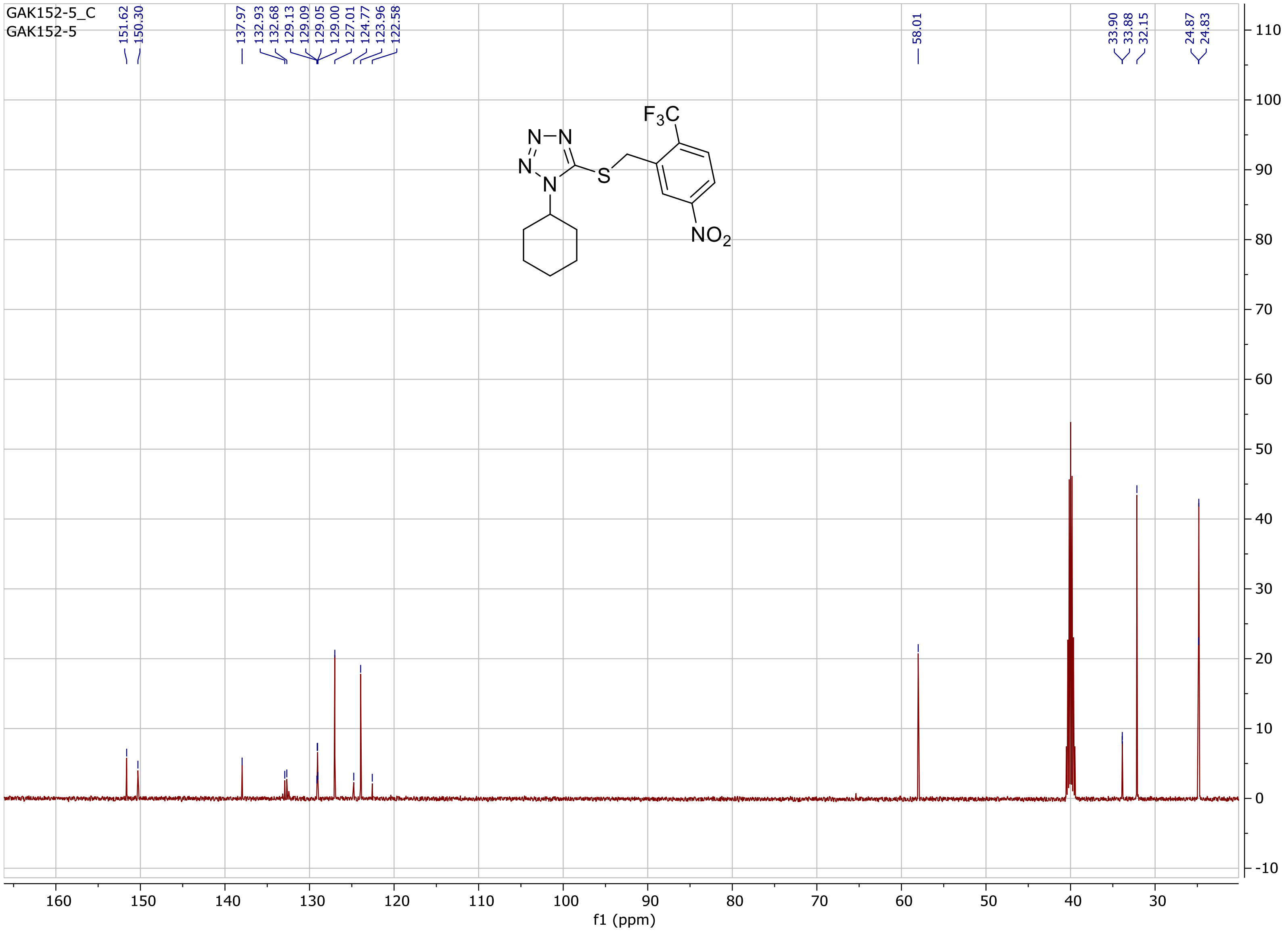

1-Cyclohexyl-5-((5-nitro-2-(trifluoromethyl)benzyl)sulfanyl)-1H-tetrazole (**71e**):

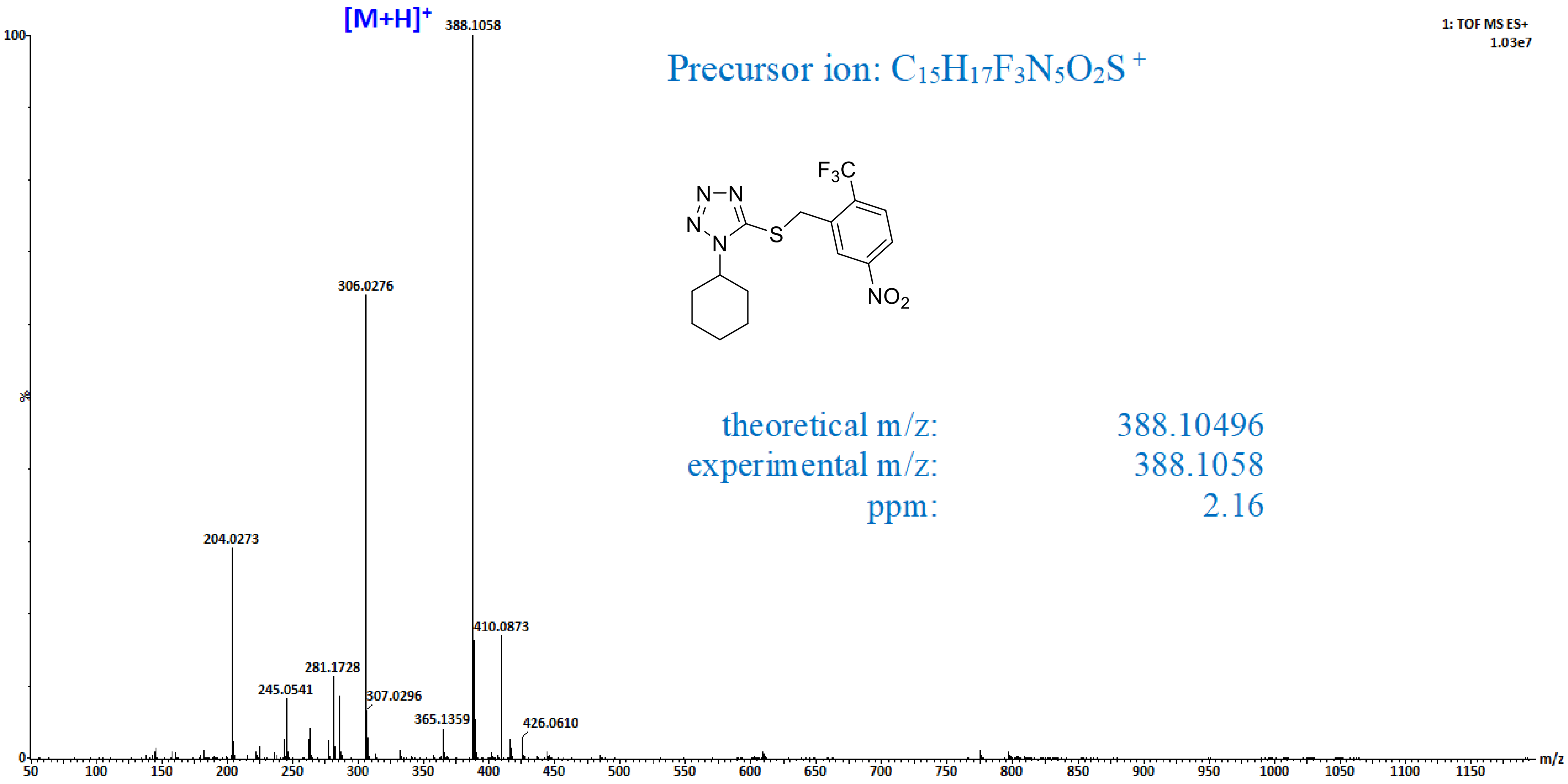

5-((2-Nitro-5-(trifluoromethyl)benzyl)sulfanyl)-2-phenyl-1,3,4-oxadiazole (**72a**): ^{1}H NMR (500 MHz, DMSO-d_6)

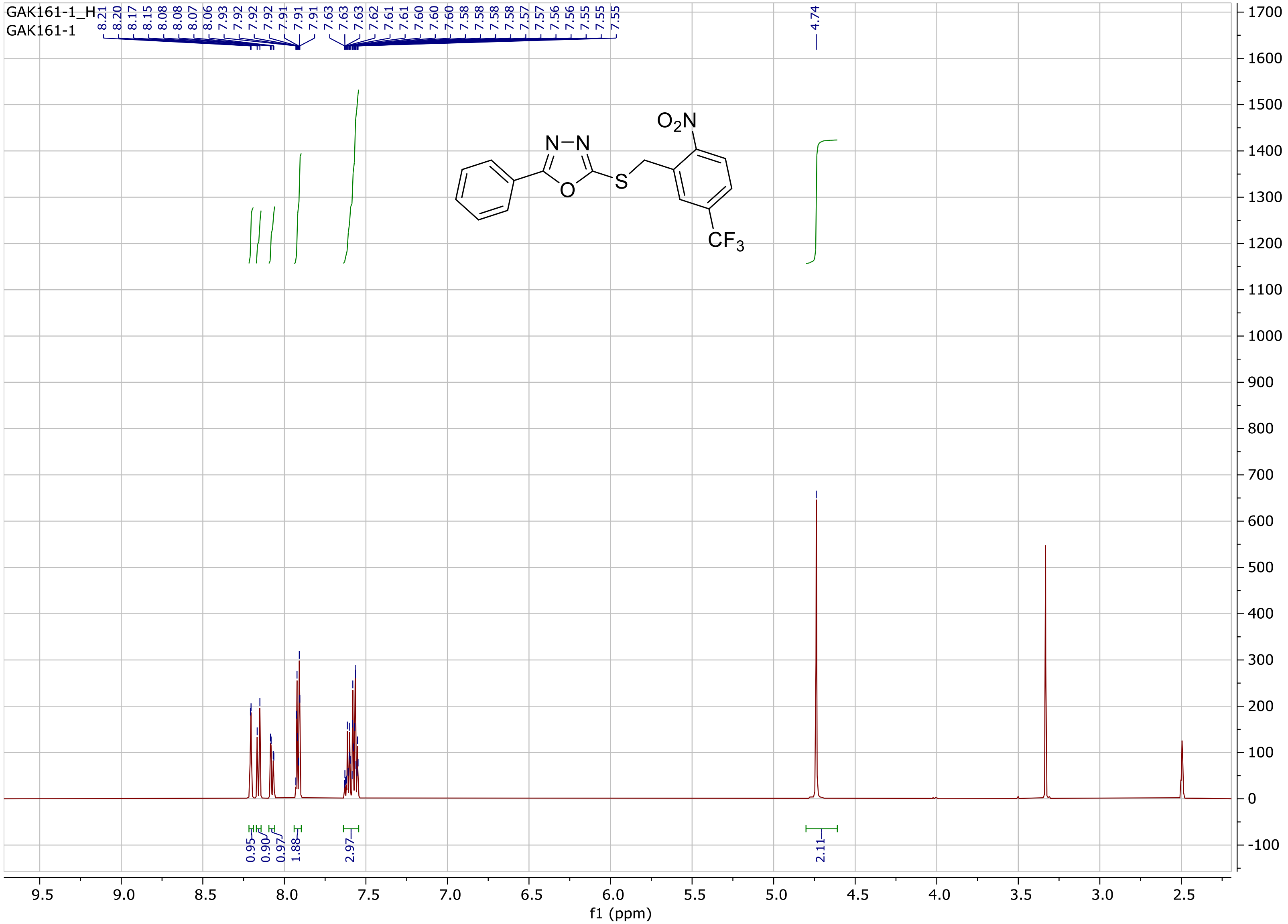

5-((2-Nitro-5-(trifluoromethyl)benzyl)sulfanyl)-2-phenyl-1,3,4-oxadiazole (**72a**): ^{13}C NMR (126 MHz, DMSO-d_6)

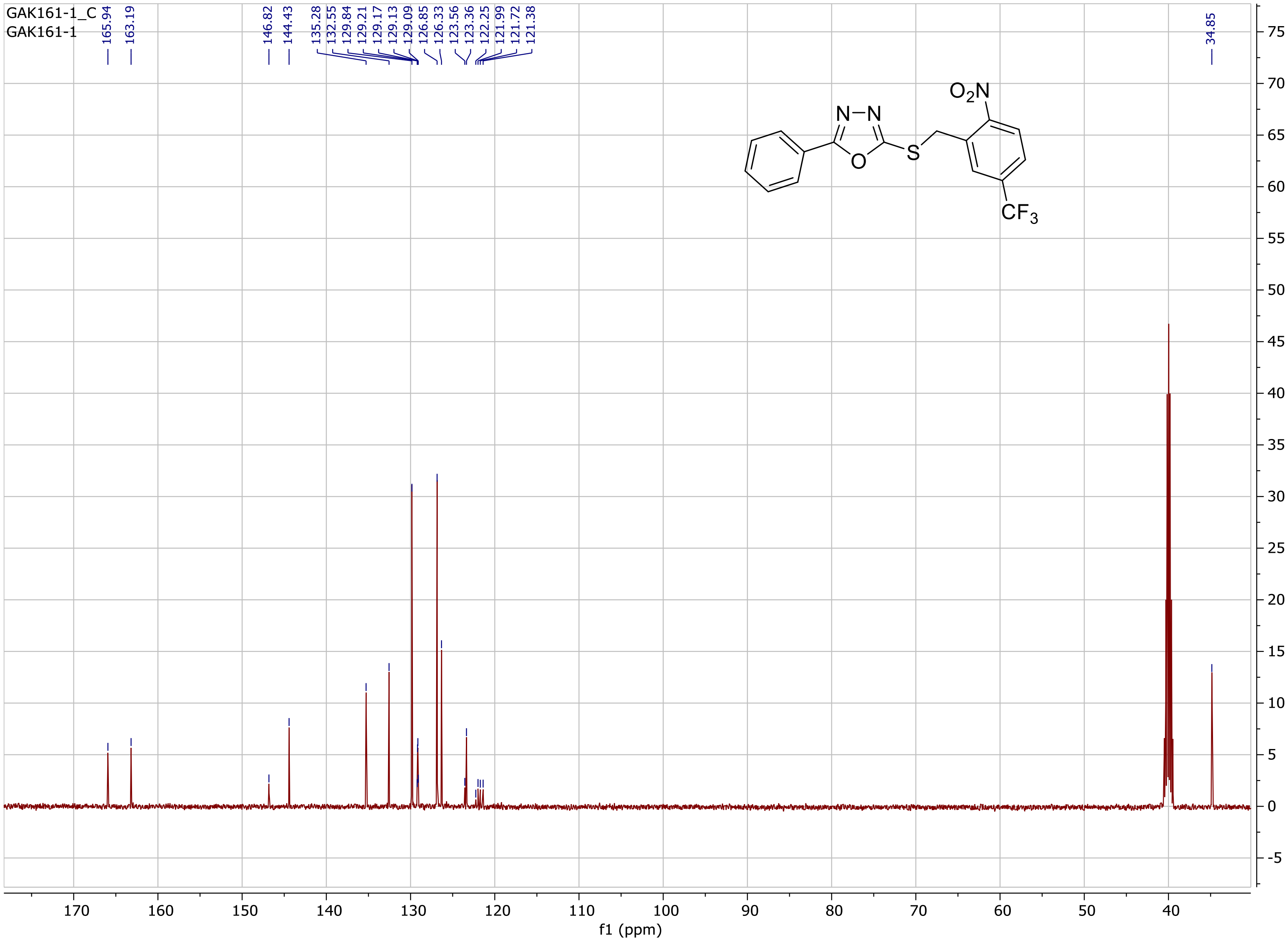

5-((2-Nitro-5-(trifluoromethyl)benzyl)sulfanyl)-2-phenyl-1,3,4-oxadiazole (**72a**):

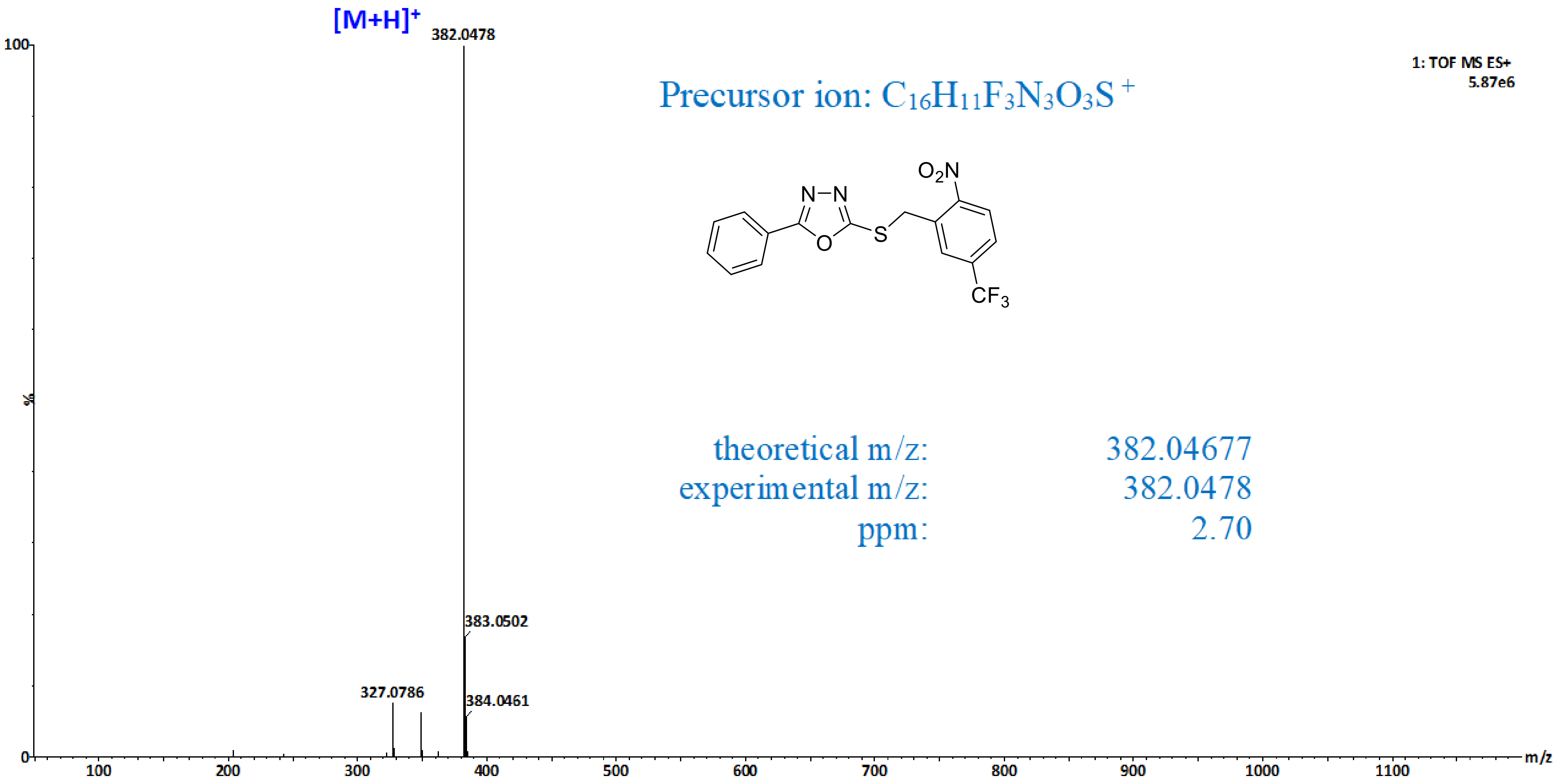

2-(4-Methoxyphenyl)-5-((2-nitro-5-(trifluoromethyl)benzyl)sulfanyl)-2-phenyl-1,3,4-oxadiazole (**72b**): ^{1}H NMR (600 MHz, DMSO-d_6)

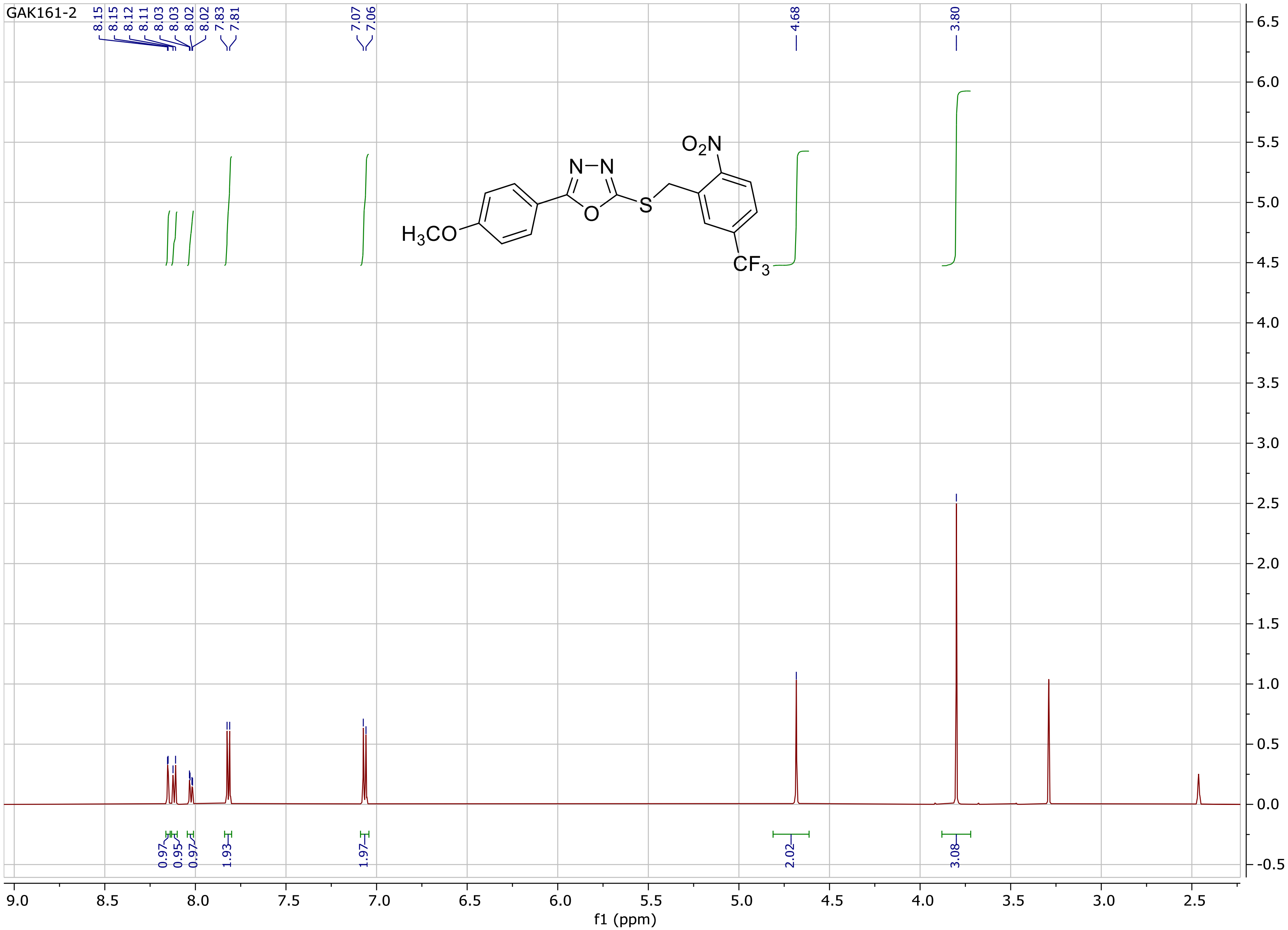

2-(4-Methoxyphenyl)-5-((2-nitro-5-(trifluoromethyl)benzyl)sulfanyl)-2-phenyl-1,3,4-oxadiazole (**72b**): ^{13}C NMR (151 MHz, DMSO-*d*$_6$)

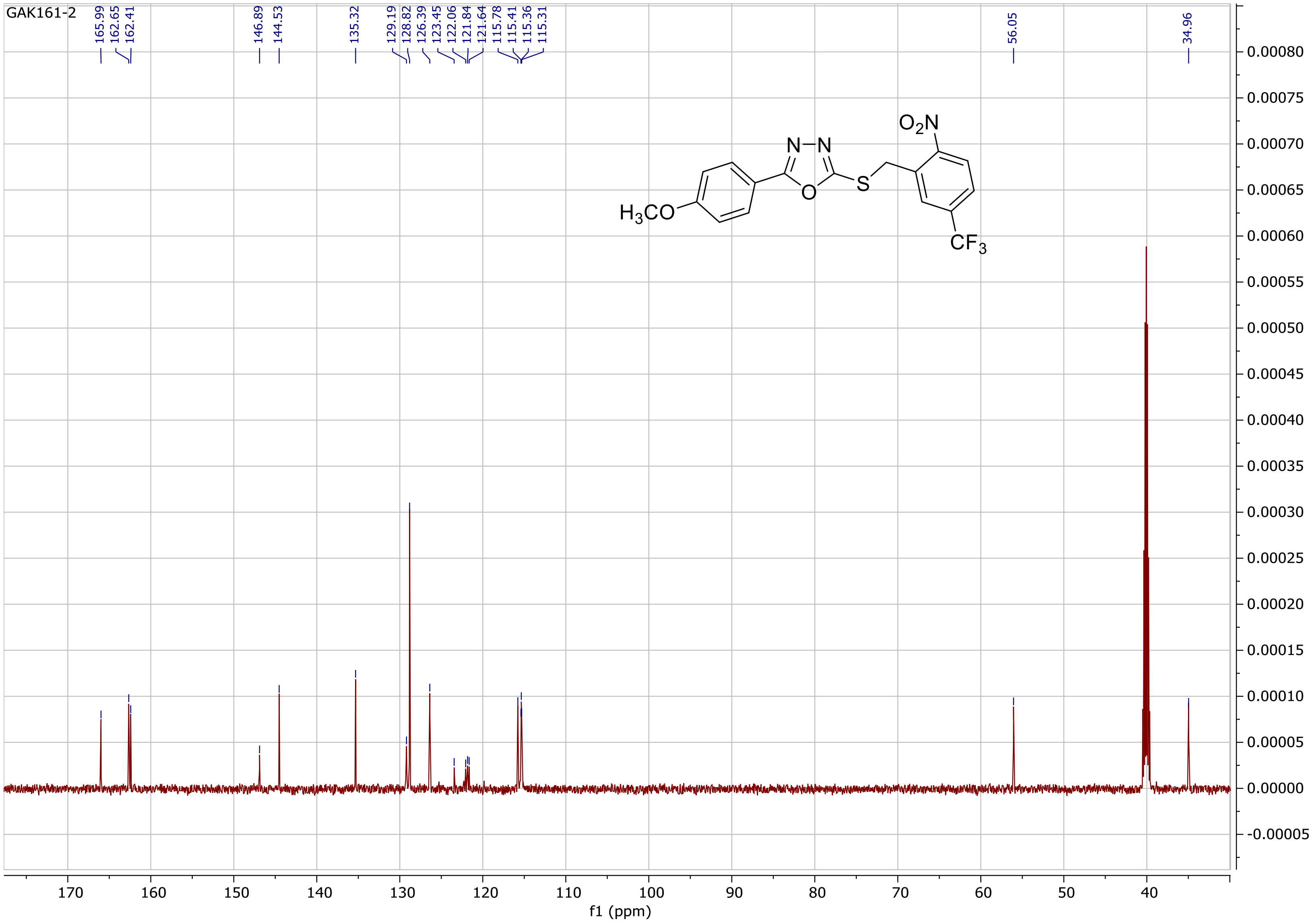

2-(4-Methoxyphenyl)-5-((2-nitro-5-(trifluoromethyl)benzyl)sulfanyl)-2-phenyl-1,3,4-oxadiazole (**72b**):

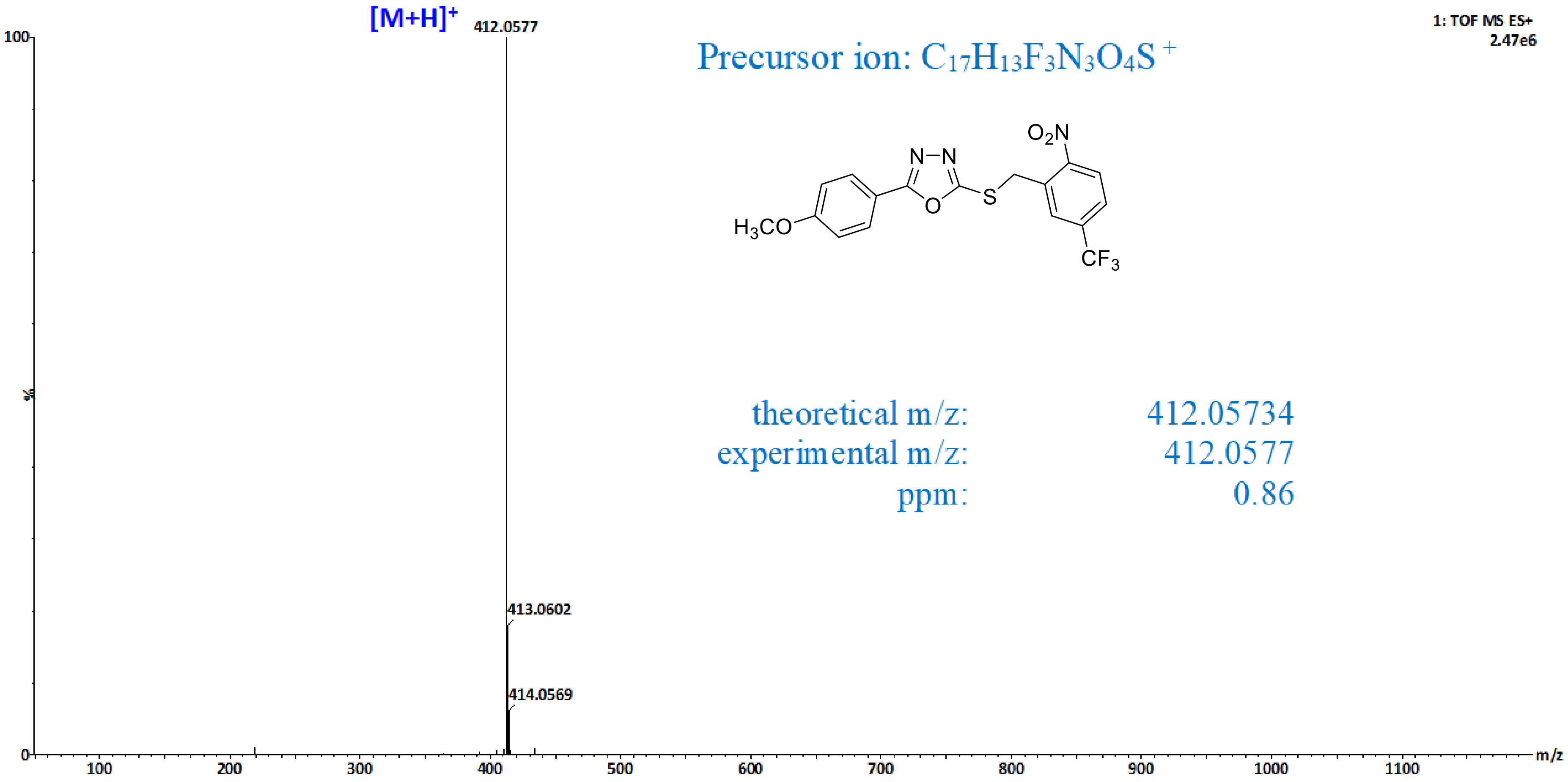

2-(4-Chlorophenyl)-5-((2-nitro-5-(trifluoromethyl)benzyl)sulfanyl)-2-phenyl-1,3,4-oxadiazole (**72c**): ^{1}H NMR (500 MHz, DMSO-d_6)

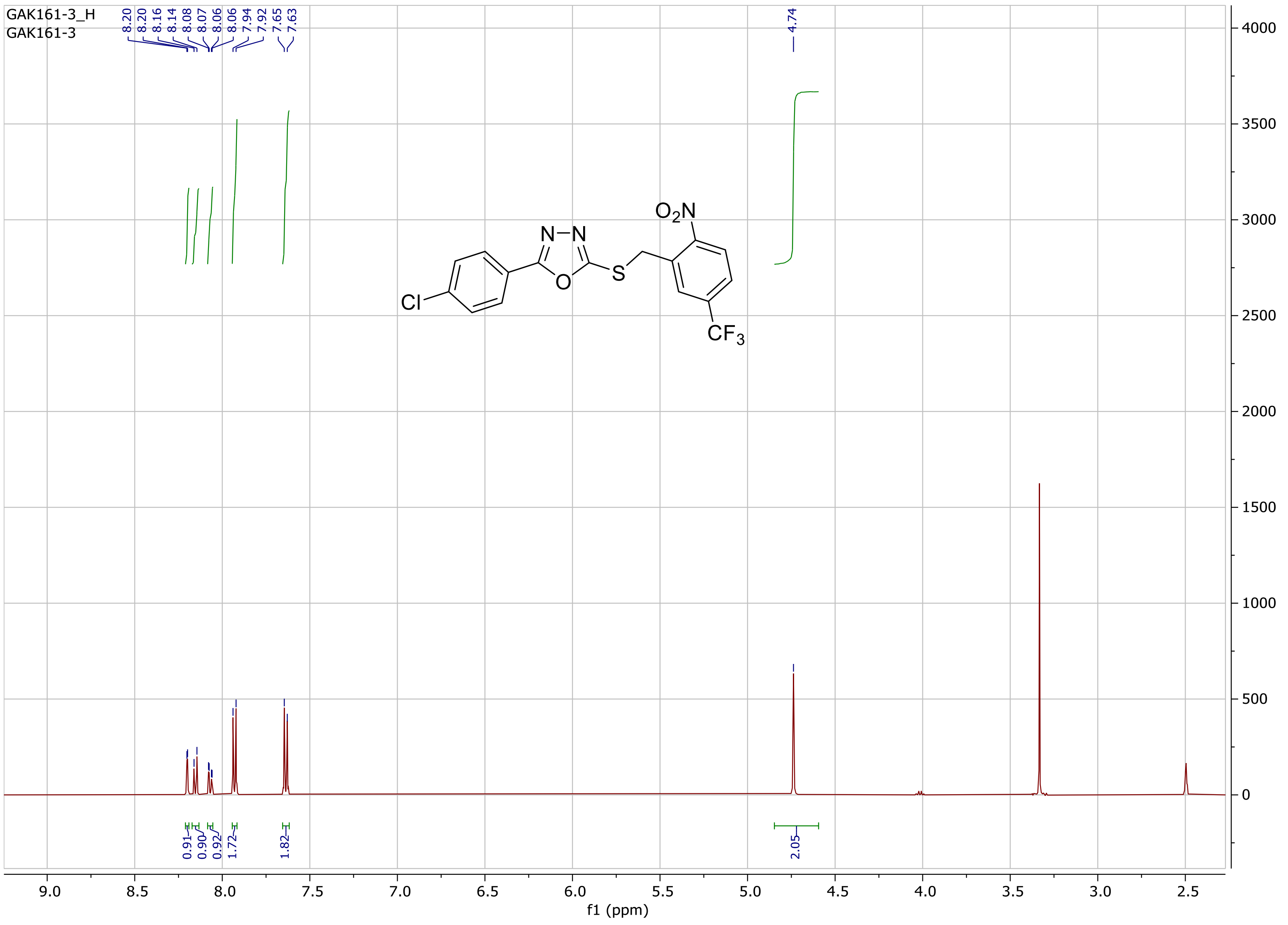

2-(4-Chlorophenyl)-5-((2-nitro-5-(trifluoromethyl)benzyl)sulfanyl)-2-phenyl-1,3,4-oxadiazole (**72c**): ^{13}C NMR (126 MHz, DMSO-d_6)

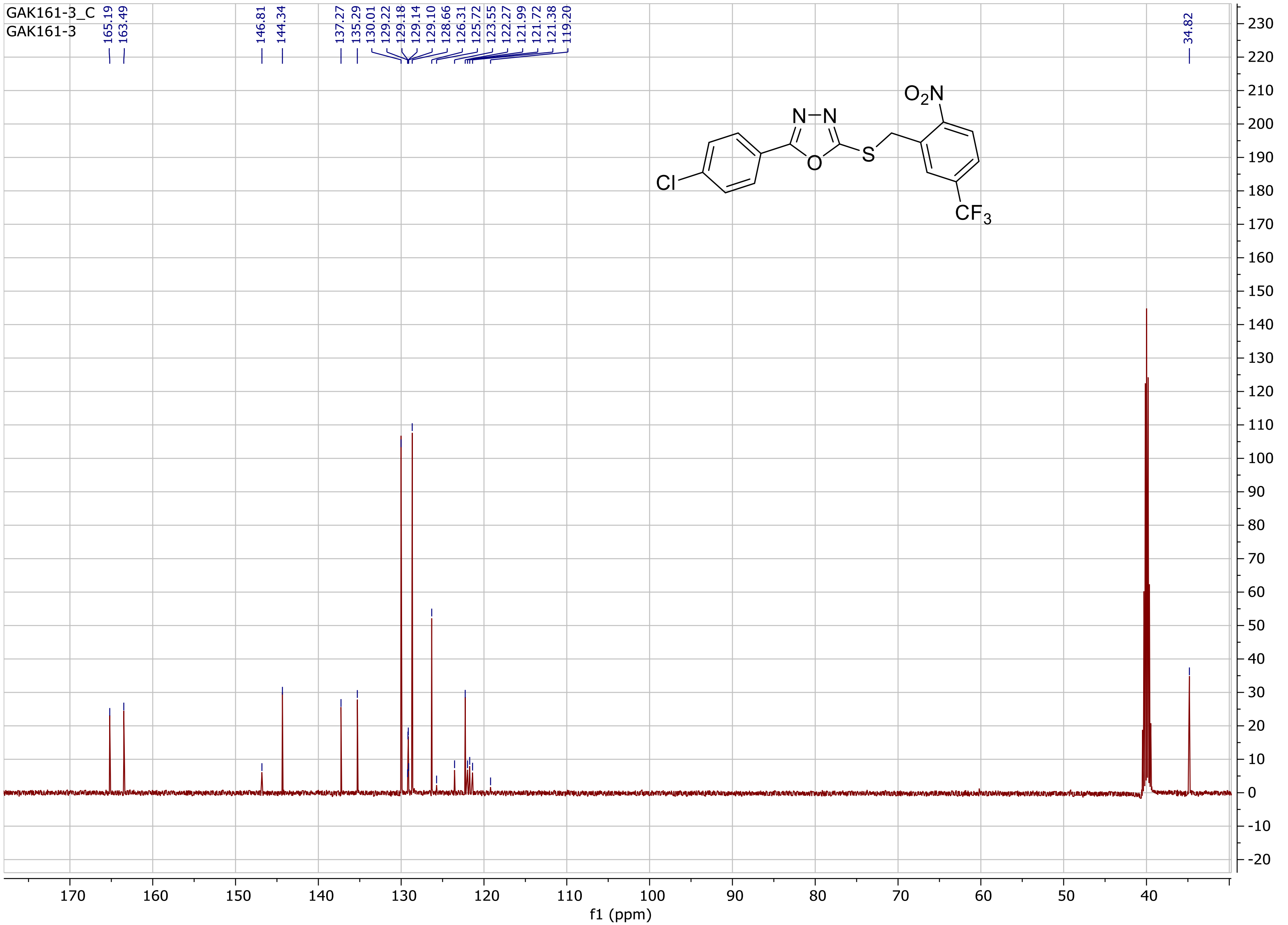

2-(4-Chlorophenyl)-5-((2-nitro-5-(trifluoromethyl)benzyl)sulfanyl)-2-phenyl-1,3,4-oxadiazole (**72c**):

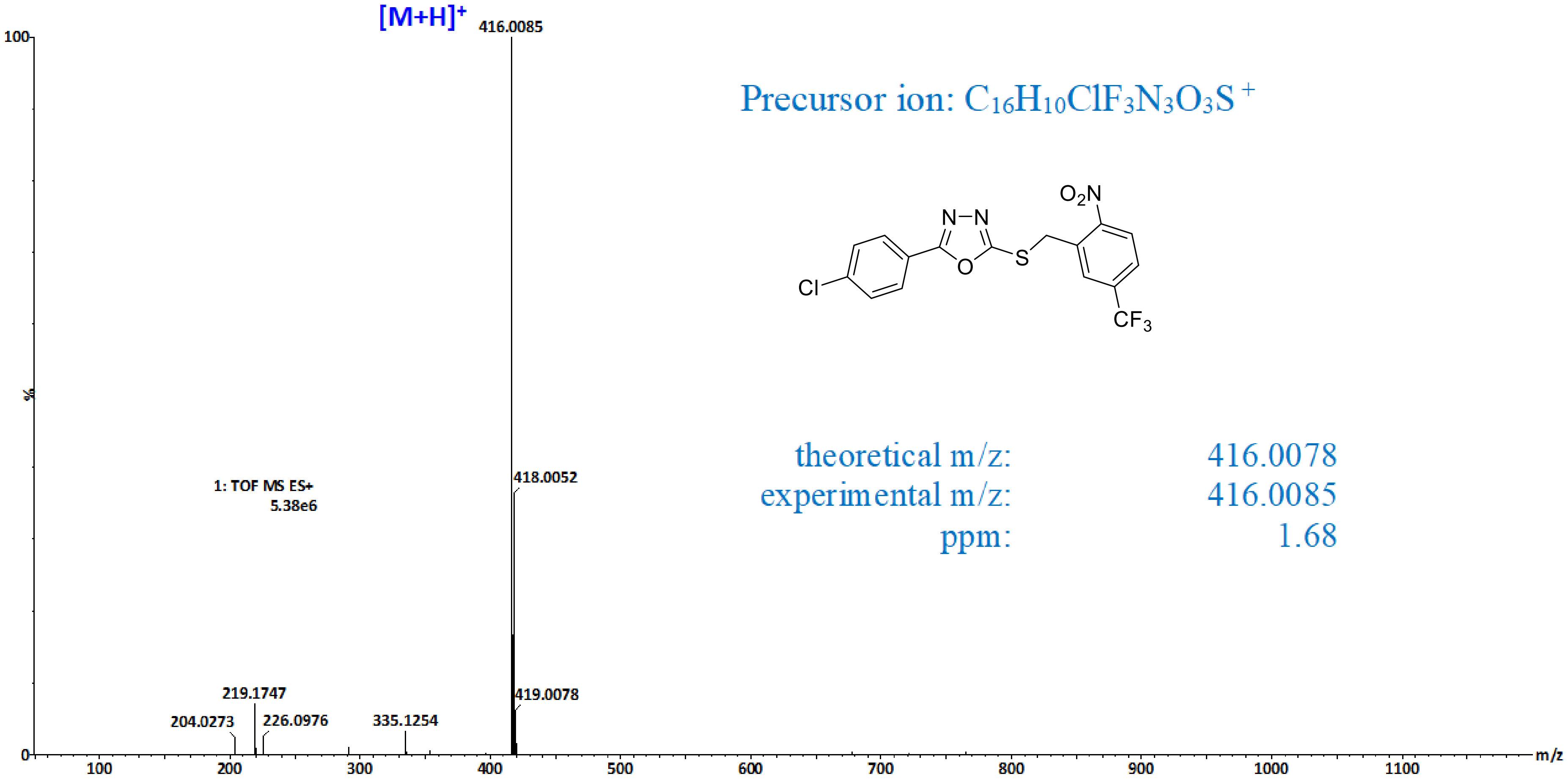

2-(4-Bromophenyl)-5-((2-nitro-5-(trifluoromethyl)benzyl)sulfanyl)-2-phenyl-1,3,4-oxadiazole (**72d**): ^{1}H NMR (500 MHz, DMSO-d_6)

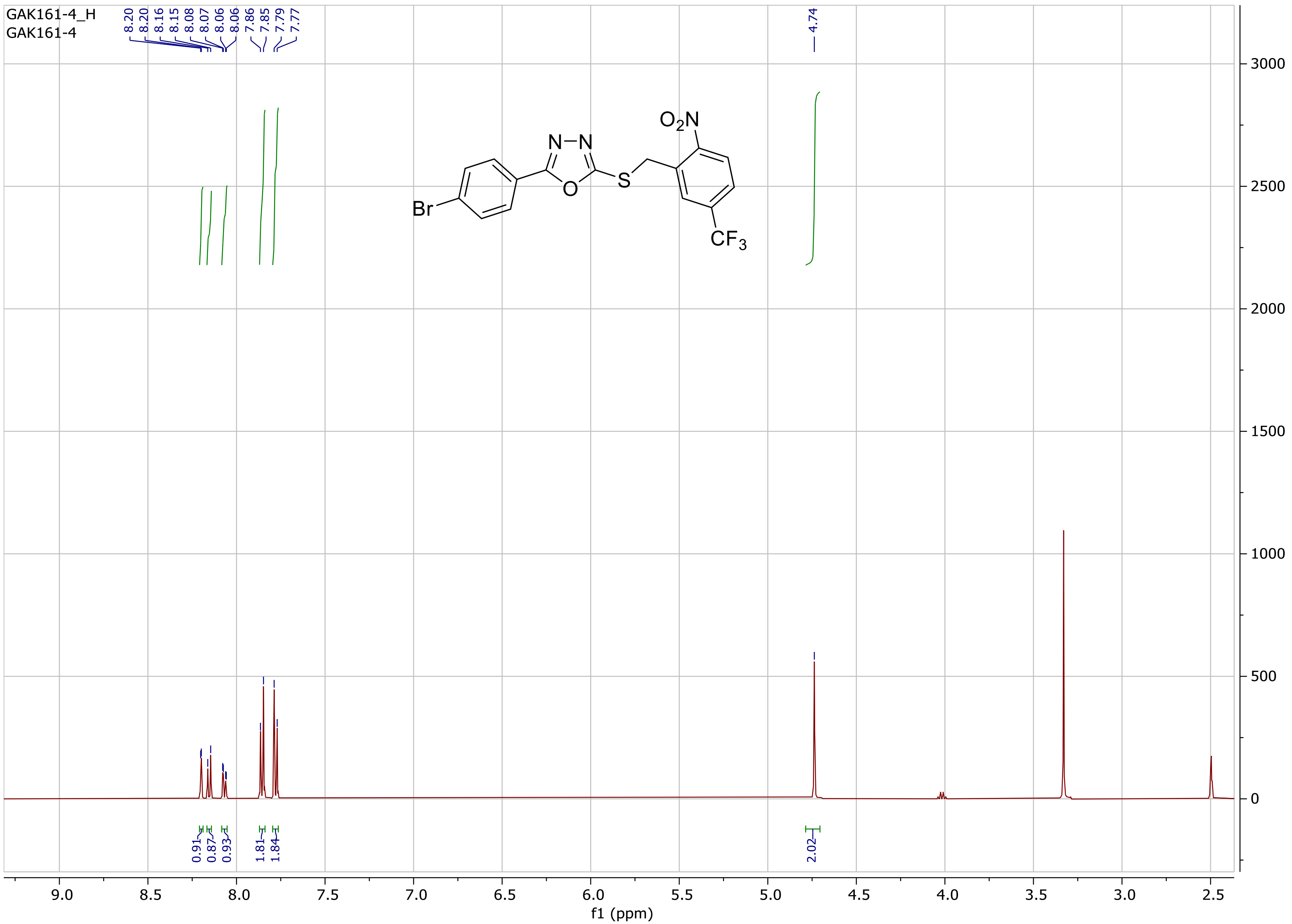

2-(4-Bromophenyl)-5-((2-nitro-5-(trifluoromethyl)benzyl)sulfanyl)-2-phenyl-1,3,4-oxadiazole (**72d**): ^{13}C NMR (126 MHz, DMSO-d_6)

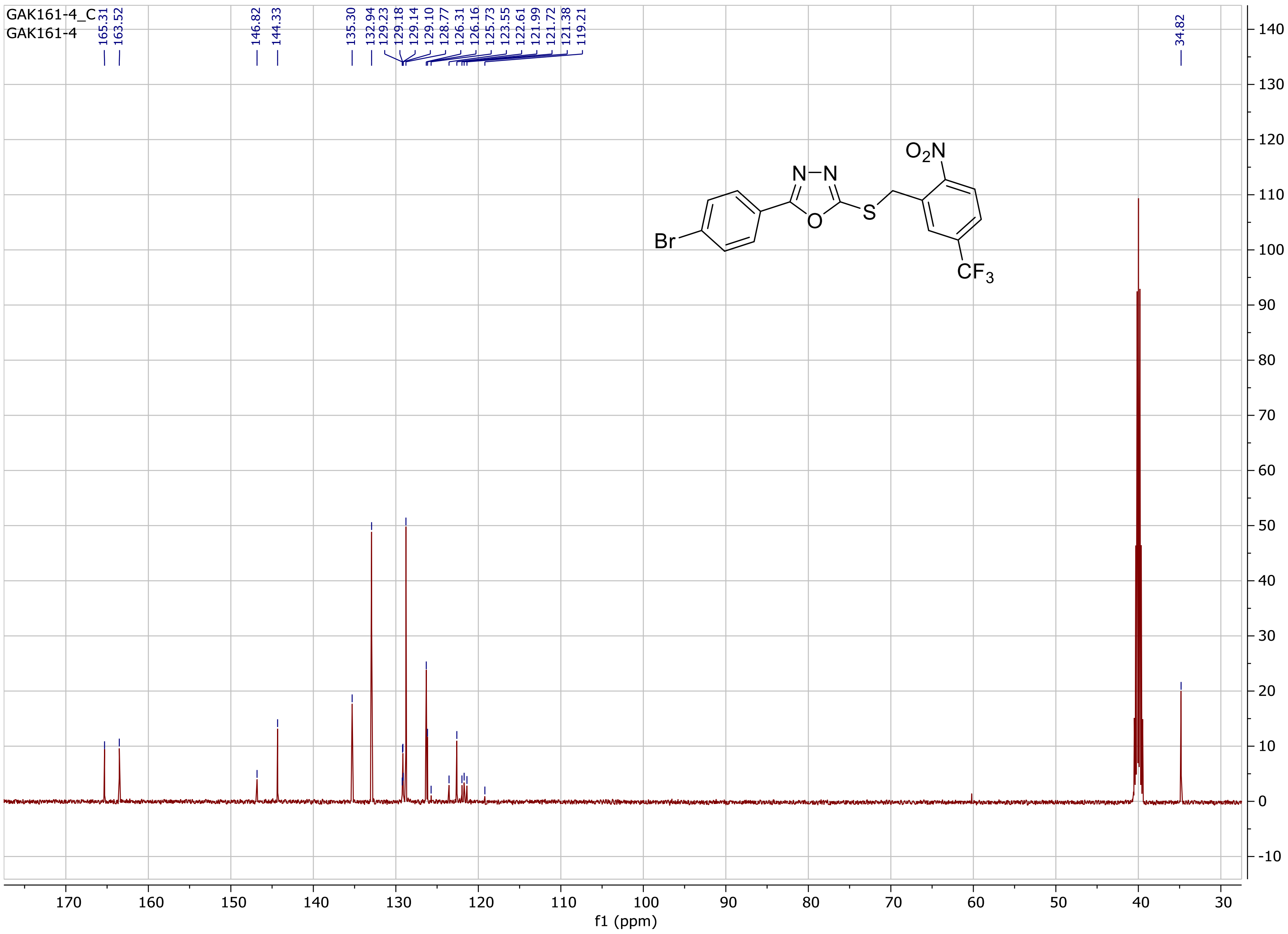

2-(4-Bromophenyl)-5-((2-nitro-5-(trifluoromethyl)benzyl)sulfanyl)-2-phenyl-1,3,4-oxadiazole (**72d**):

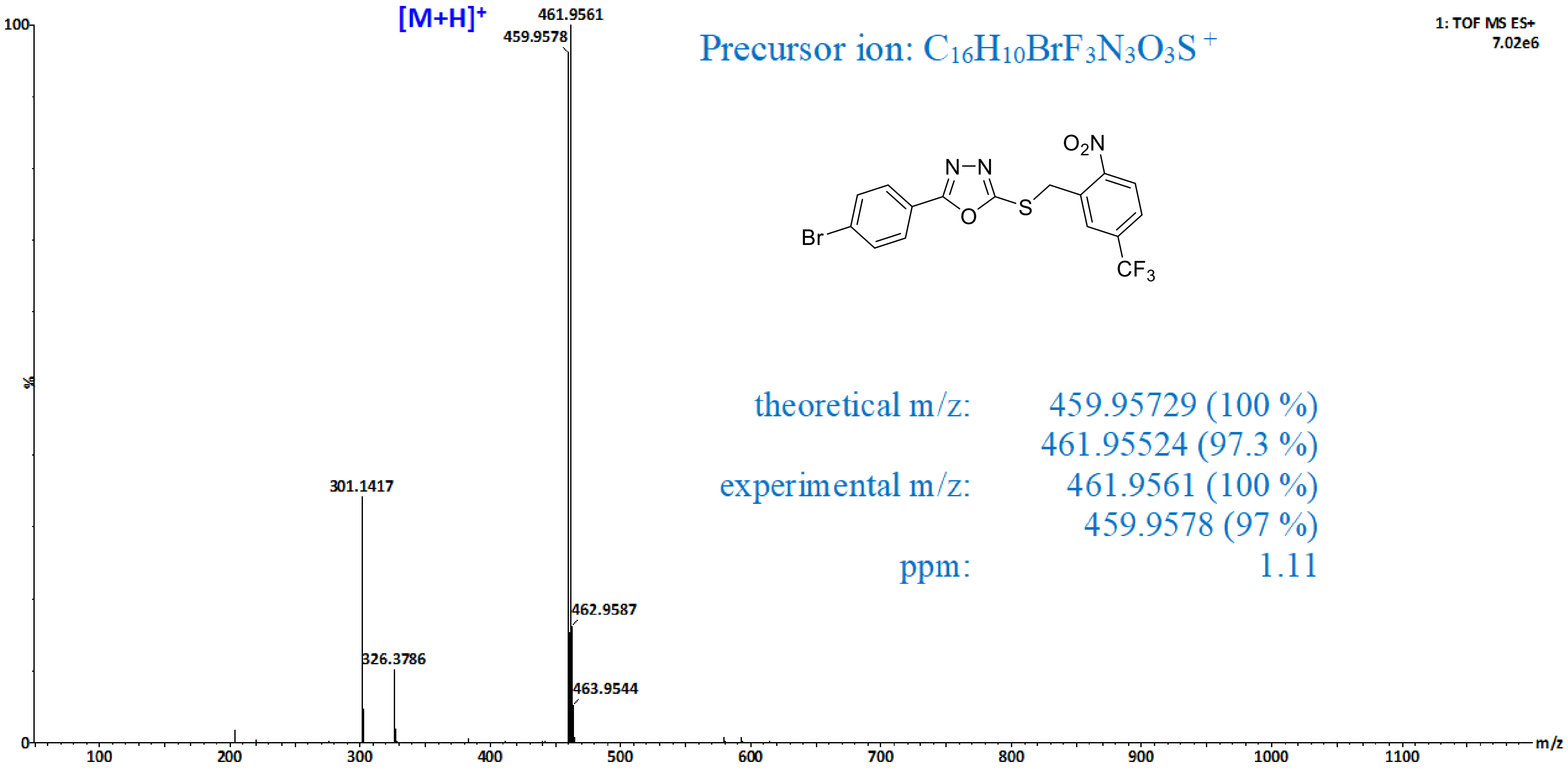

2-Cyclohexyl-5-((2-nitro-5-(trifluoromethyl)benzyl)sulfanyl)-2-phenyl-1,3,4-oxadiazole (**72e**): ^{1}H NMR (500 MHz, DMSO-d_6)

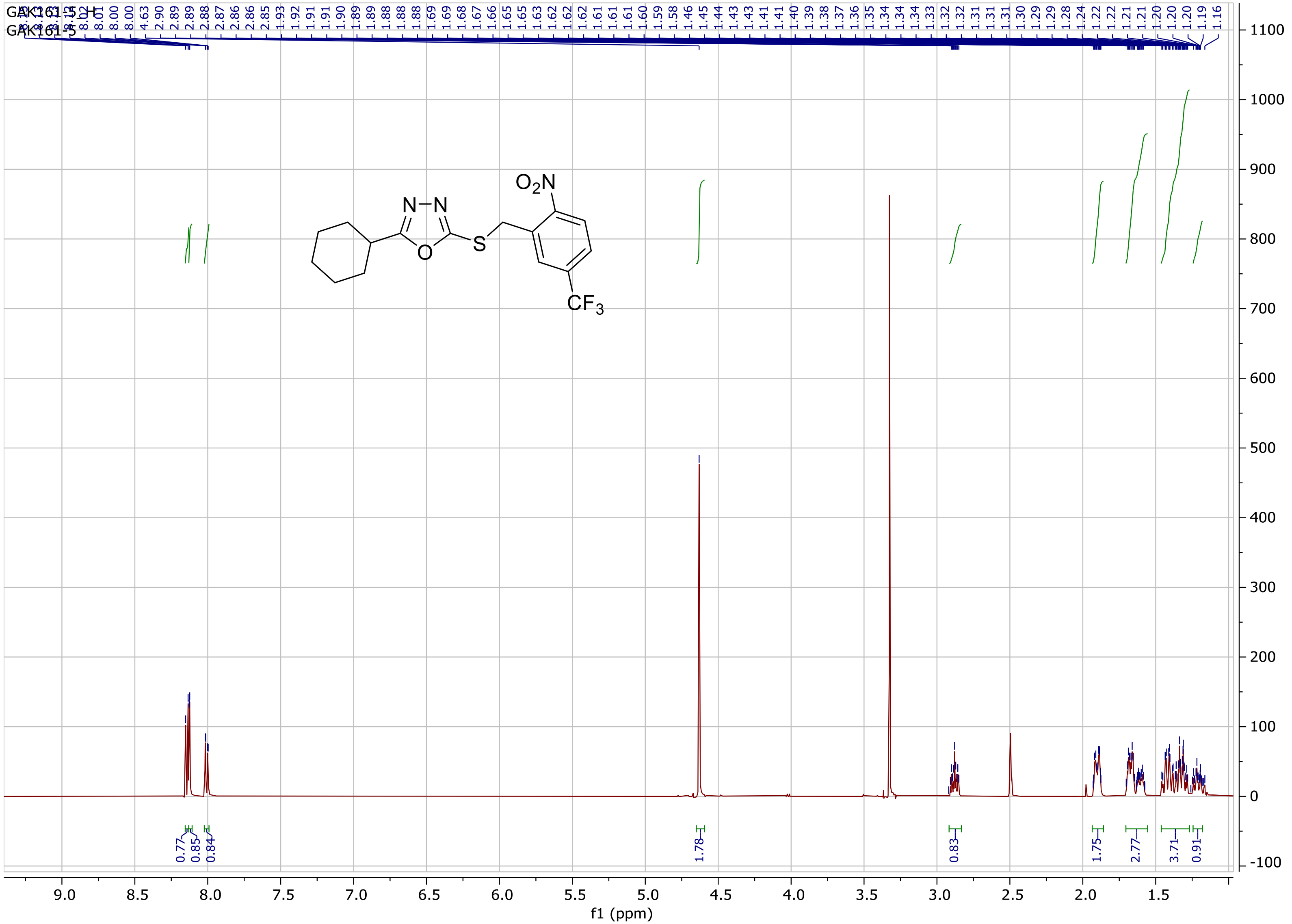

2-Cyclohexyl-5-((2-nitro-5-(trifluoromethyl)benzyl)sulfanyl)-2-phenyl-1,3,4-oxadiazole (**72e**): ^{13}C NMR (126 MHz, DMSO-d_6)

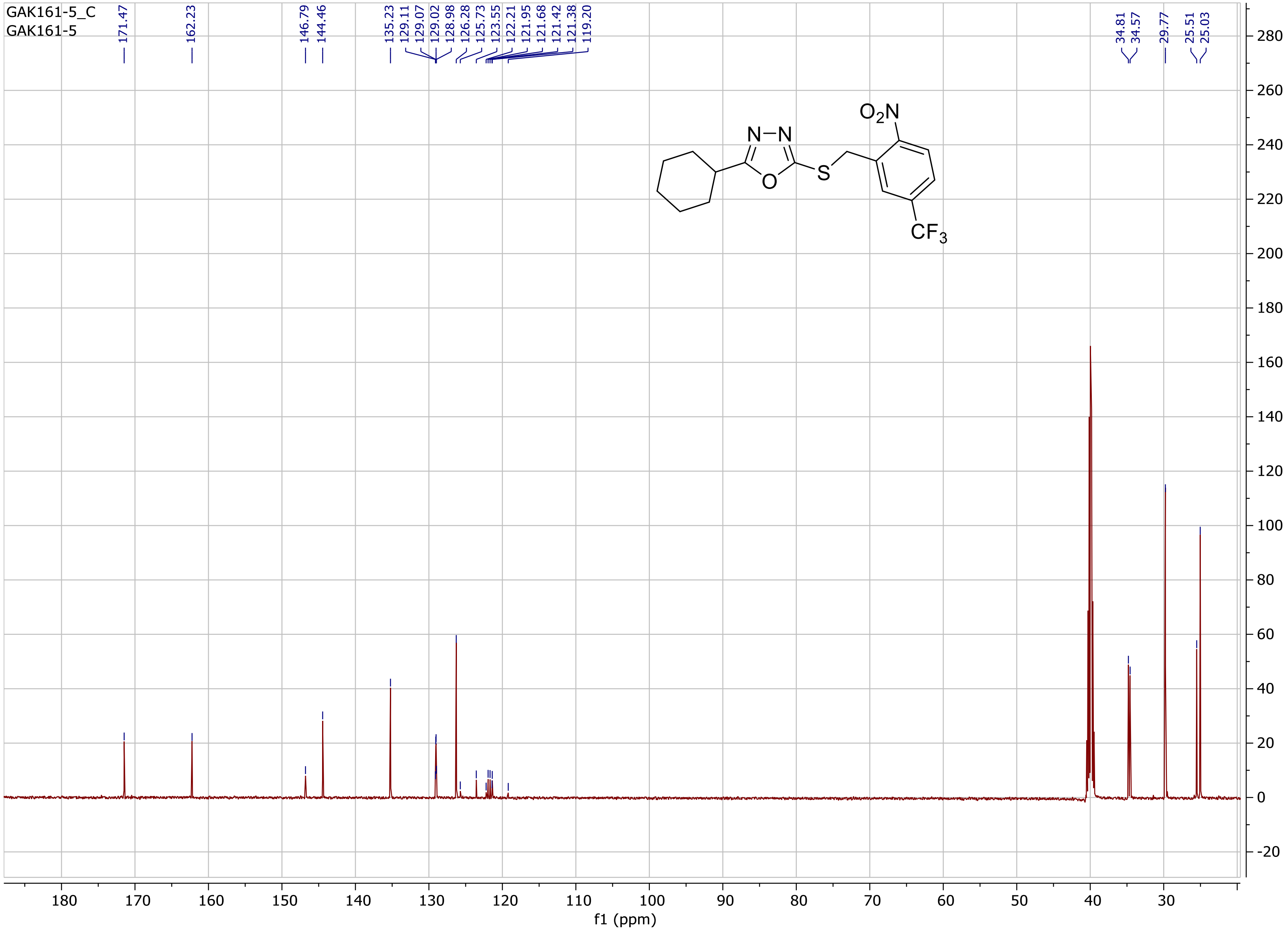

2-Cyclohexyl-5-((2-nitro-5-(trifluoromethyl)benzyl)sulfanyl)-2-phenyl-1,3,4-oxadiazole (**72e**):

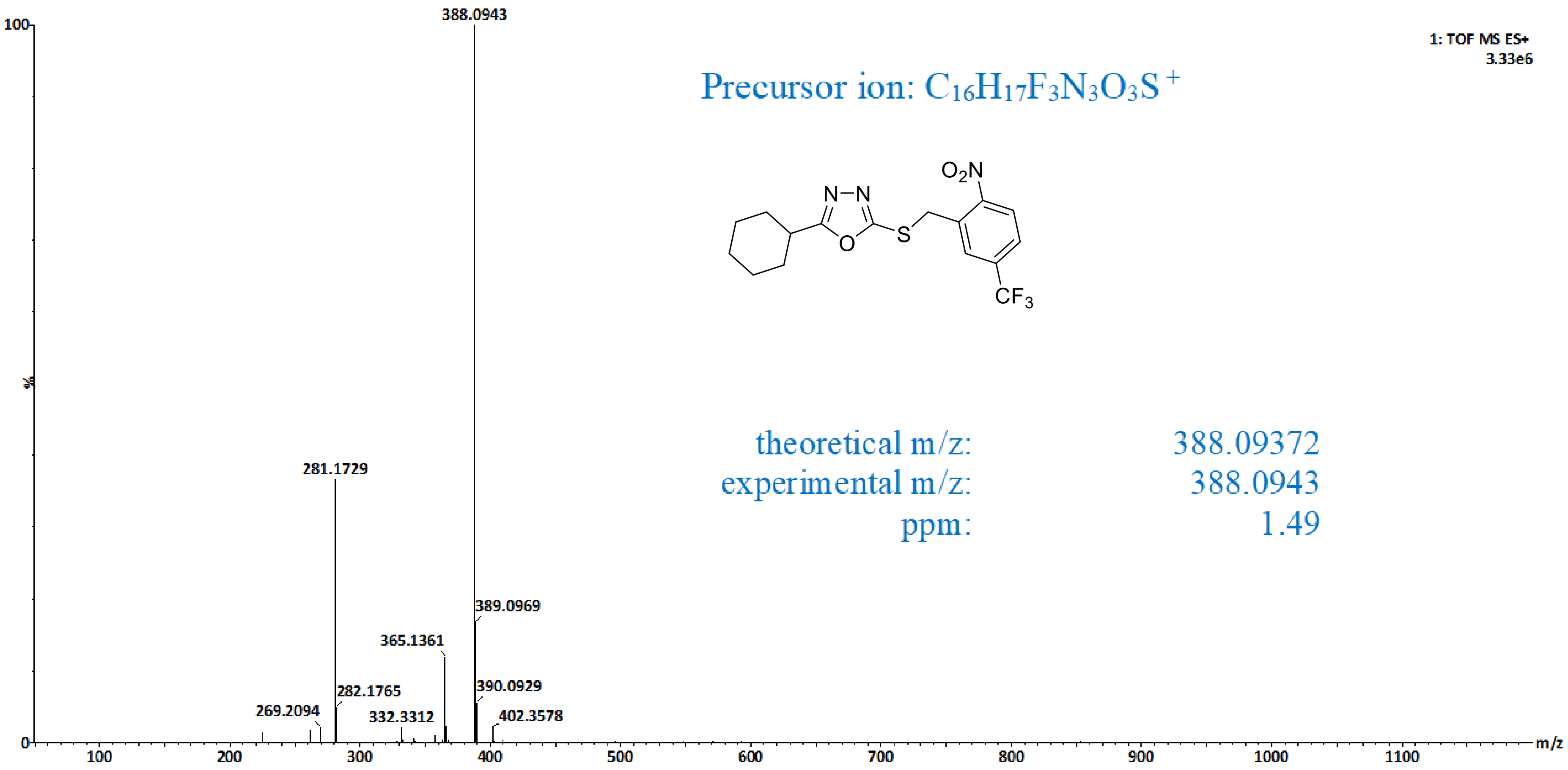

5-((5-Nitro-2-(trifluoromethyl)benzyl)sulfanyl)-2-phenyl-1,3,4-oxadiazole (**73a**): ^{1}H NMR (500 MHz, DMSO-d_6)

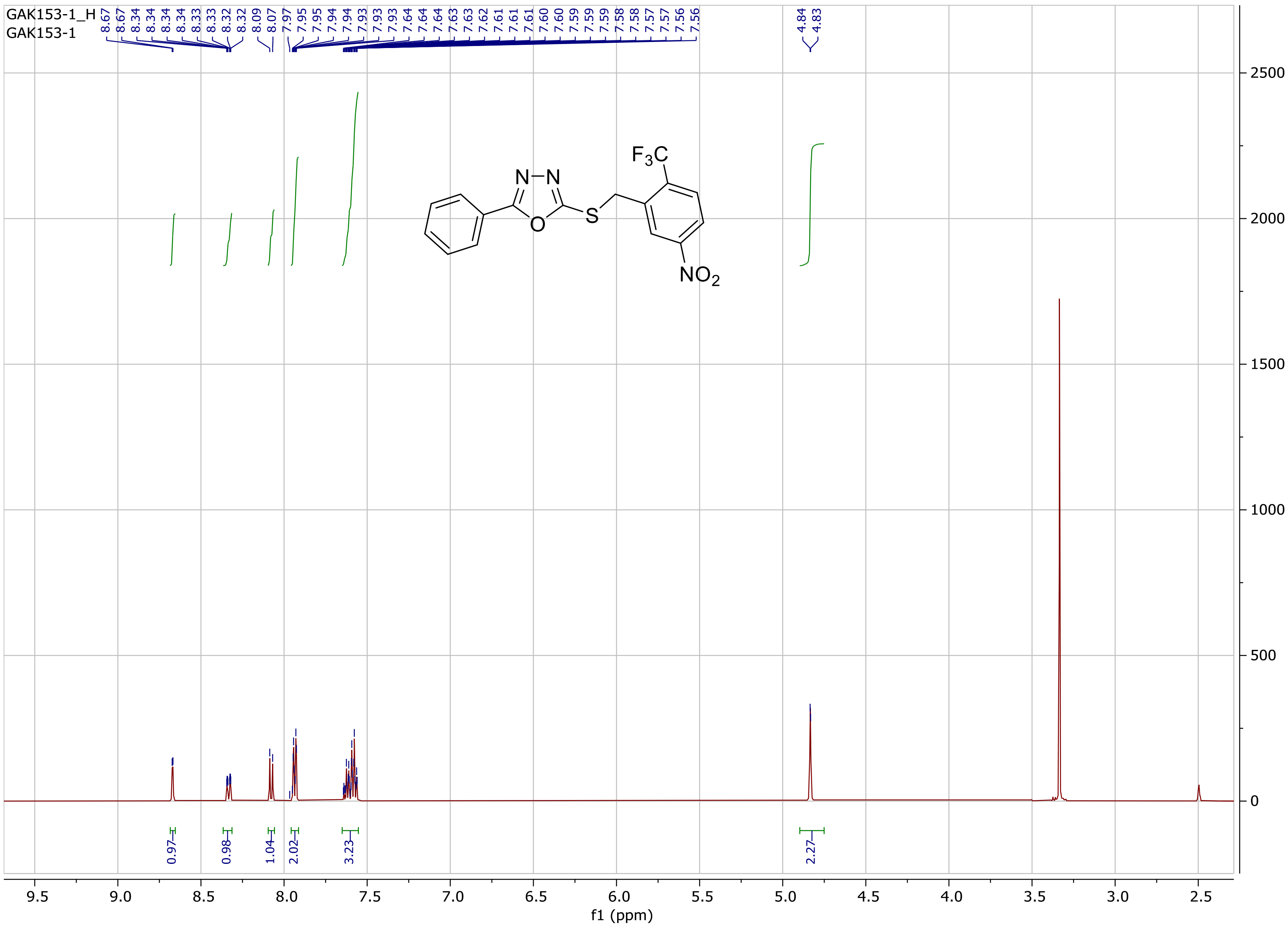

5-((5-Nitro-2-(trifluoromethyl)benzyl)sulfanyl)-2-phenyl-1,3,4-oxadiazole (**73a**): ^{13}C NMR (126 MHz, DMSO-d_6)

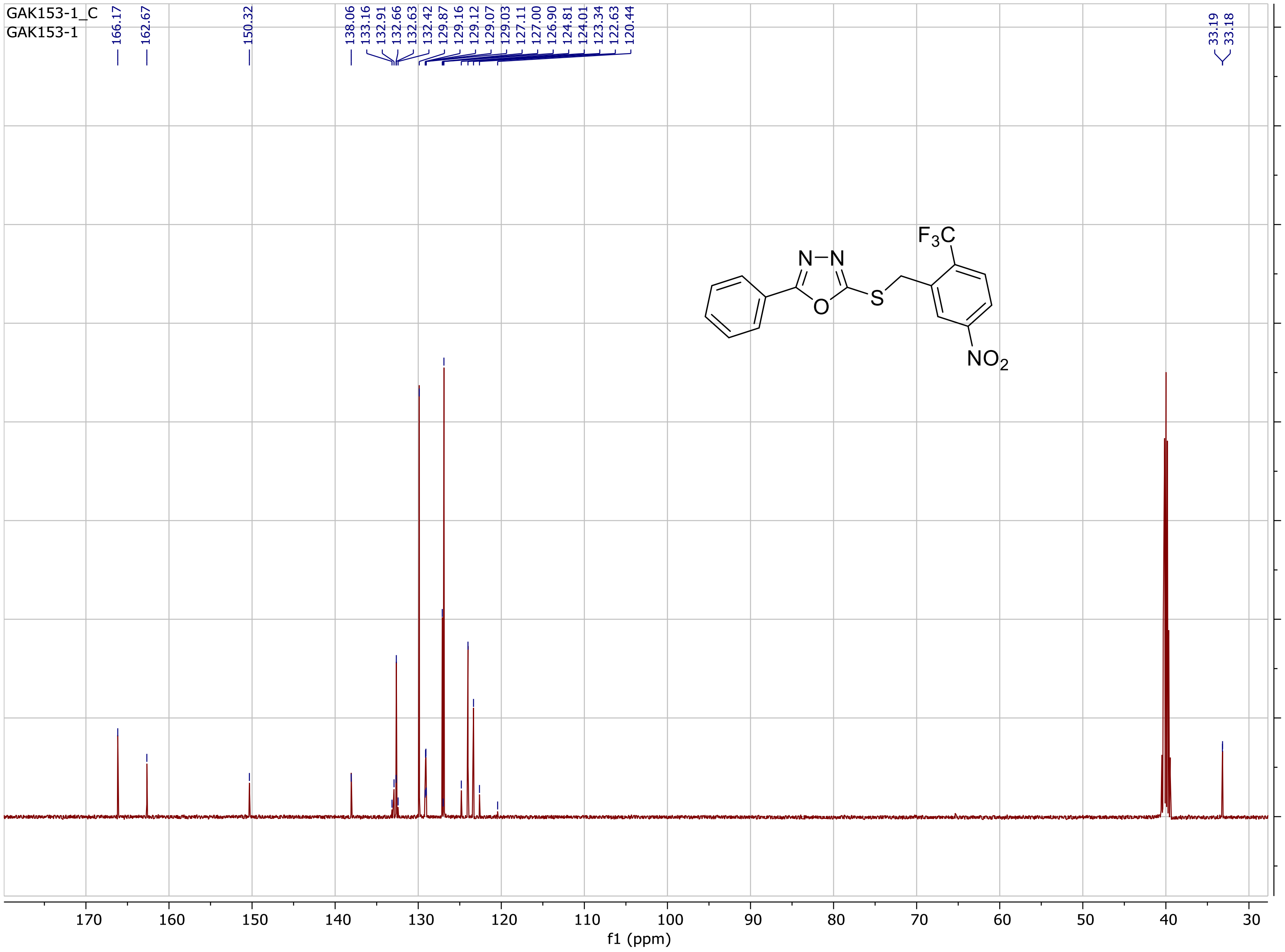

5-((5-Nitro-2-(trifluoromethyl)benzyl)sulfanyl)-2-phenyl-1,3,4-oxadiazole (**73a**):

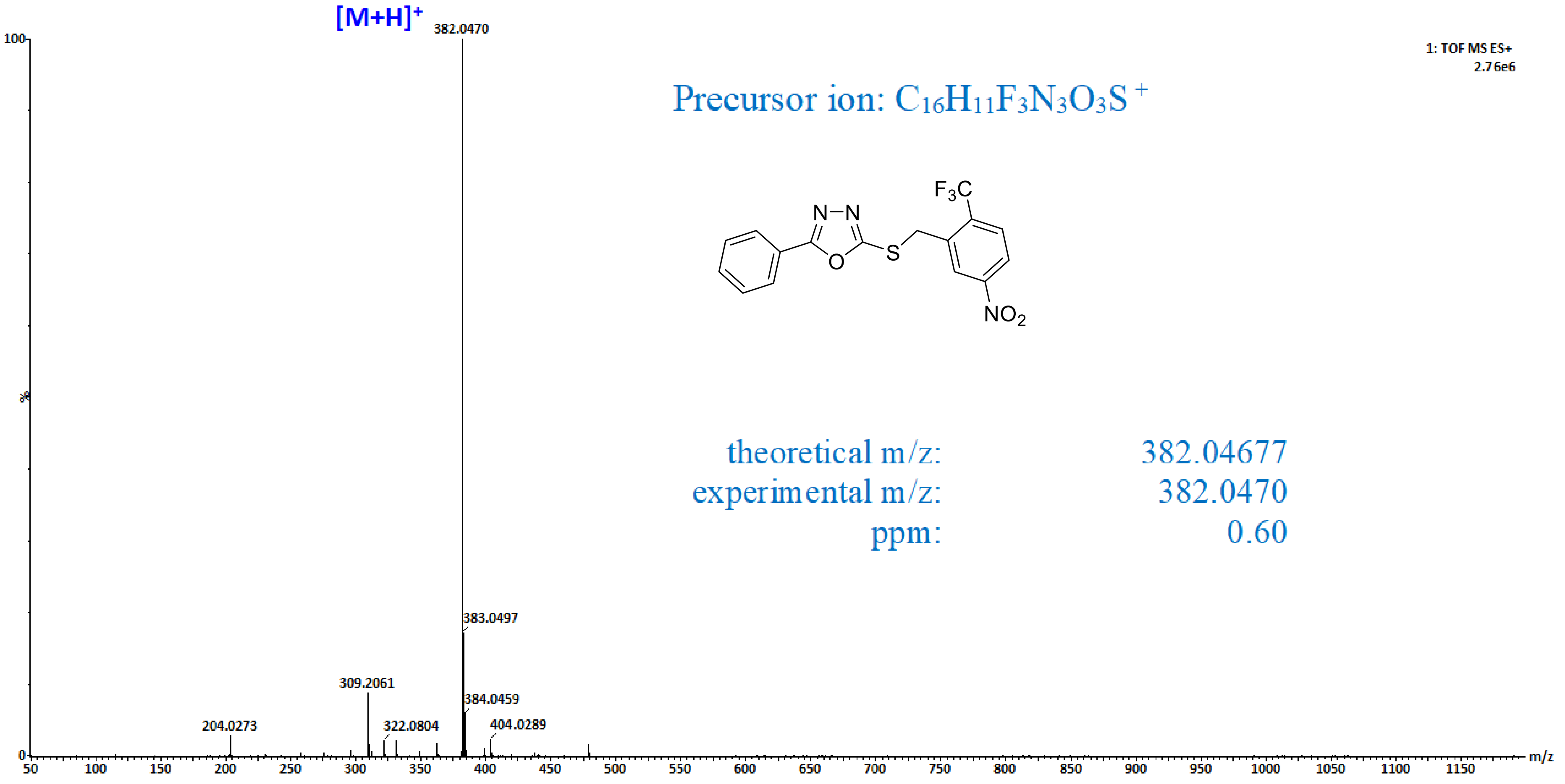

2-(4-Methoxyphenyl)-5-((5-nitro-2-(trifluoromethyl)benzyl)sulfanyl)-1,3,4-oxadiazole (**73b**): ^{1}H NMR (600 MHz, DMSO-d_6)

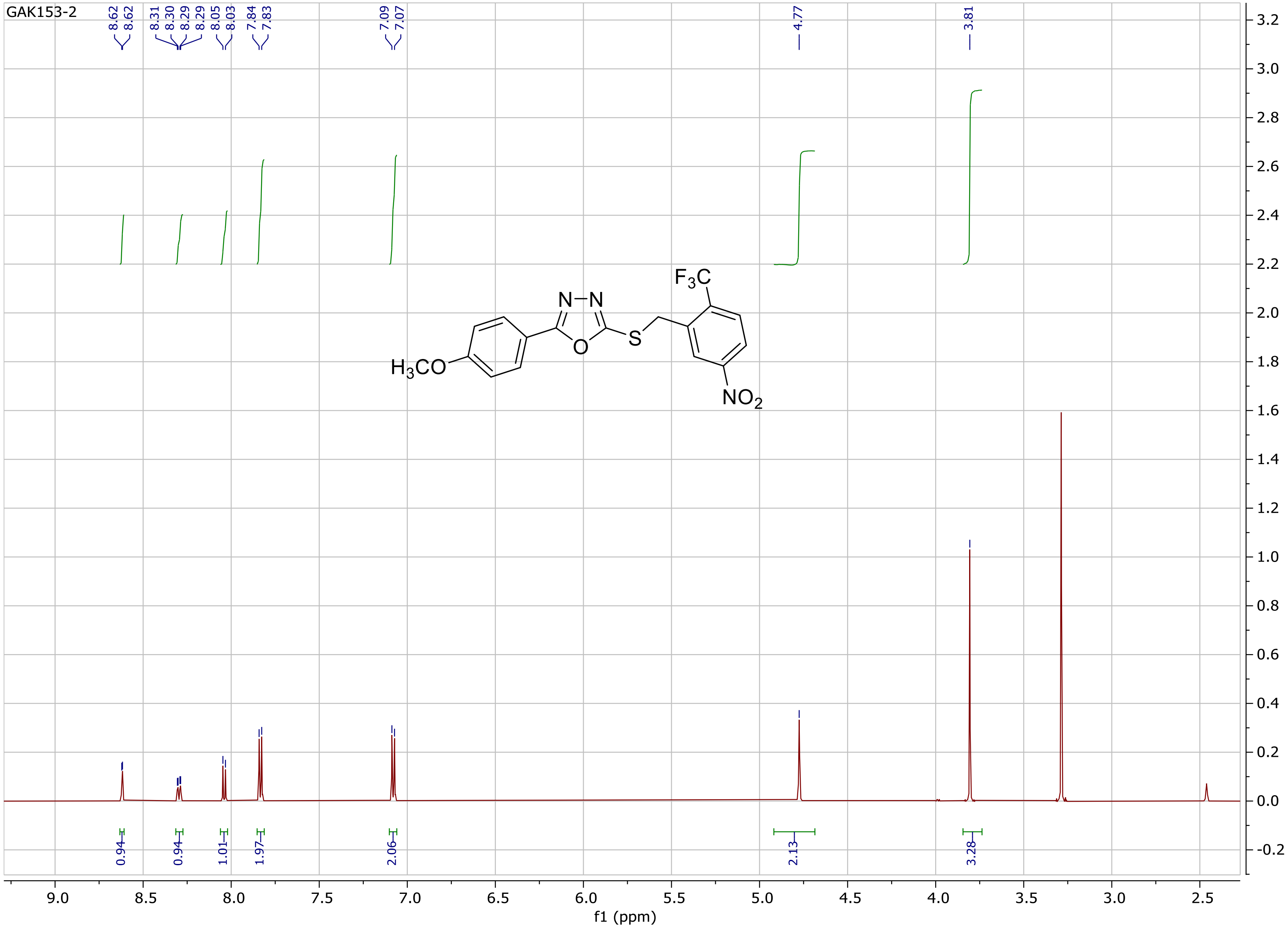

2-(4-Methoxyphenyl)-5-((5-nitro-2-(trifluoromethyl)benzyl)sulfanyl)-1,3,4-oxadiazole (**73b**): ^{13}C NMR (151 MHz, DMSO-d_6)

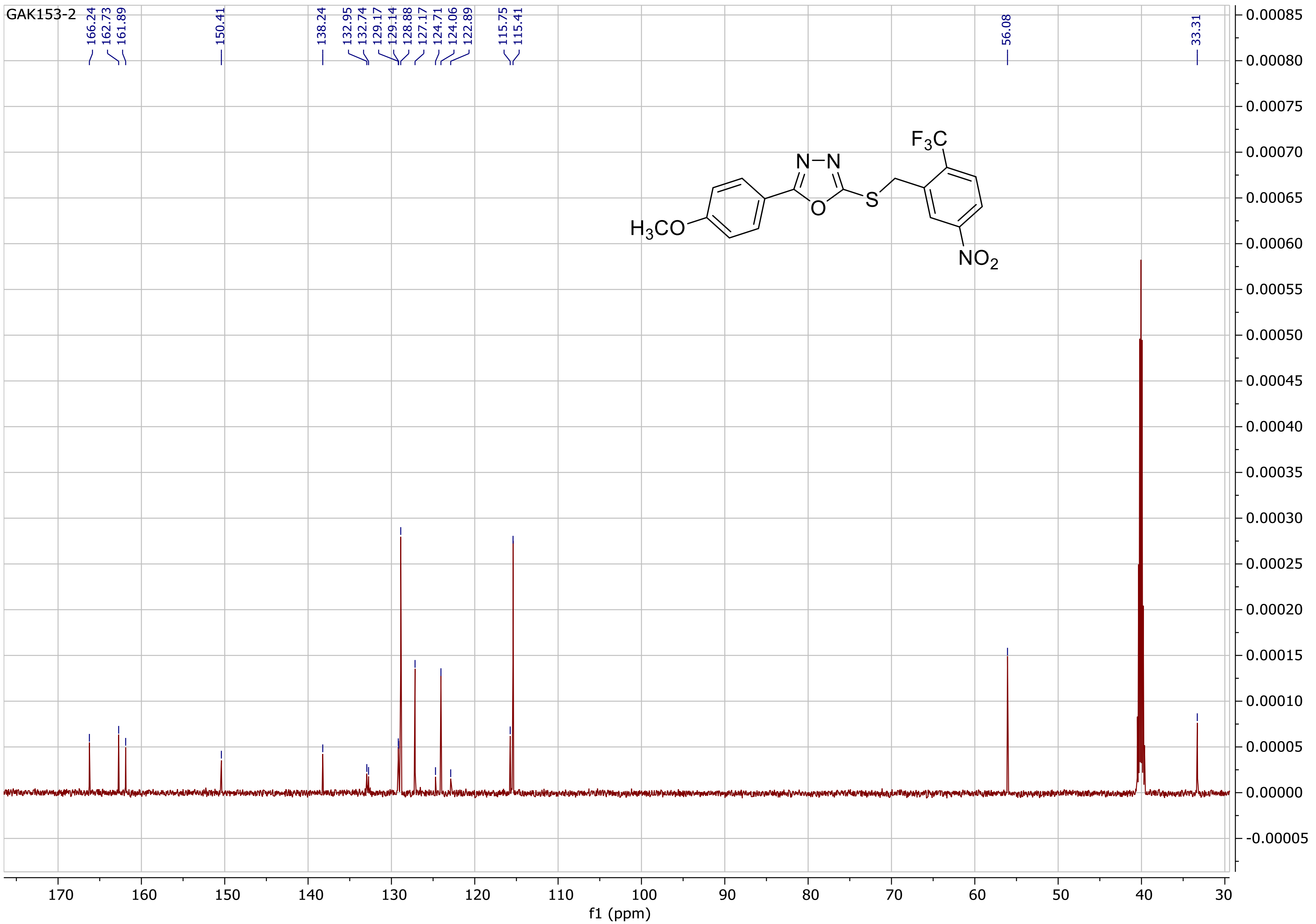

2-(4-Methoxyphenyl)-5-((5-nitro-2-(trifluoromethyl)benzyl)sulfanyl)-1,3,4-oxadiazole (**73b**):

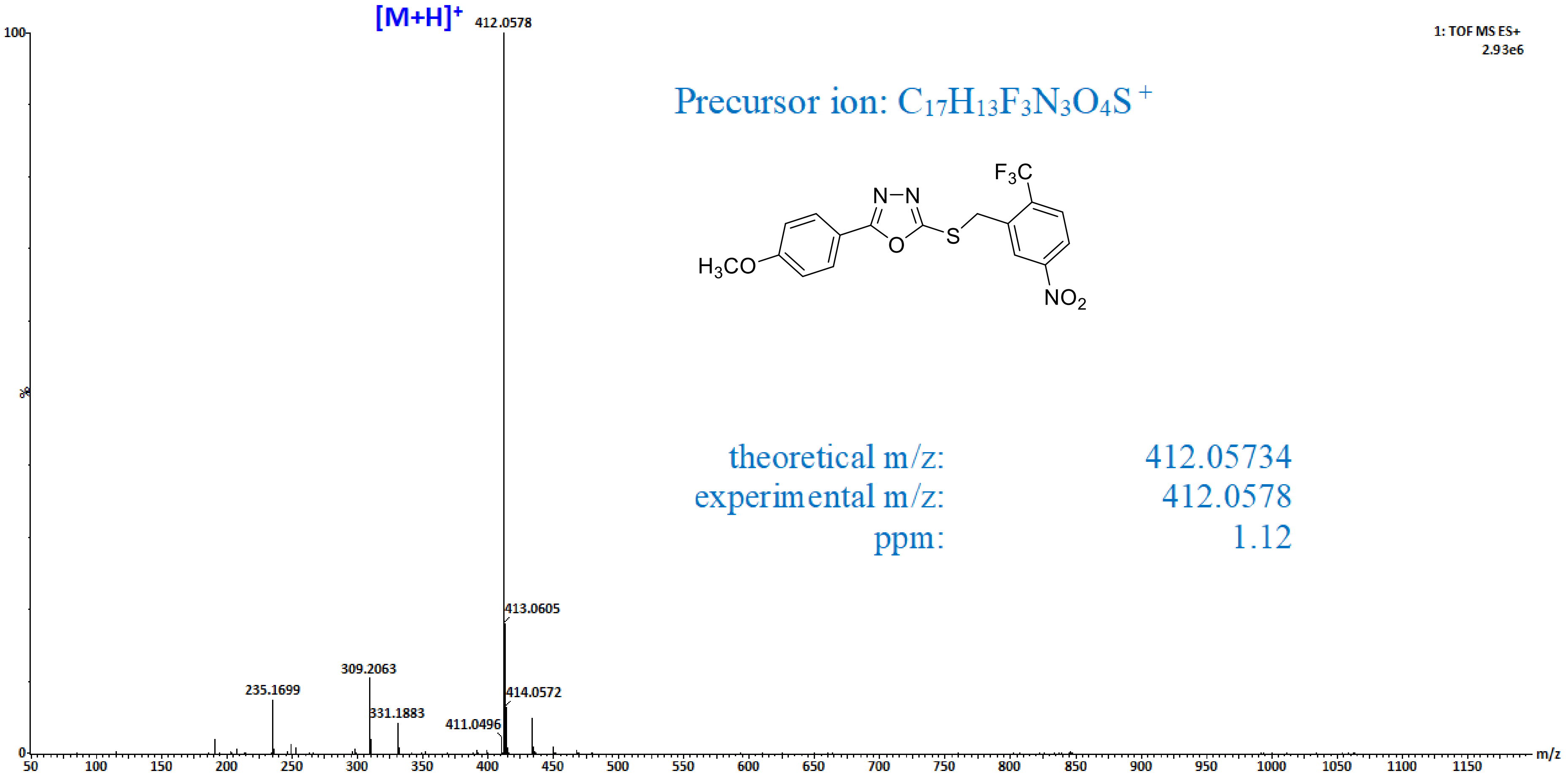

2-(4-Chlorophenyl)-5-((5-nitro-2-(trifluoromethyl)benzyl)sulfanyl)-1,3,4-oxadiazole (**73c**): ^{1}H NMR (500 MHz, DMSO-d_6)

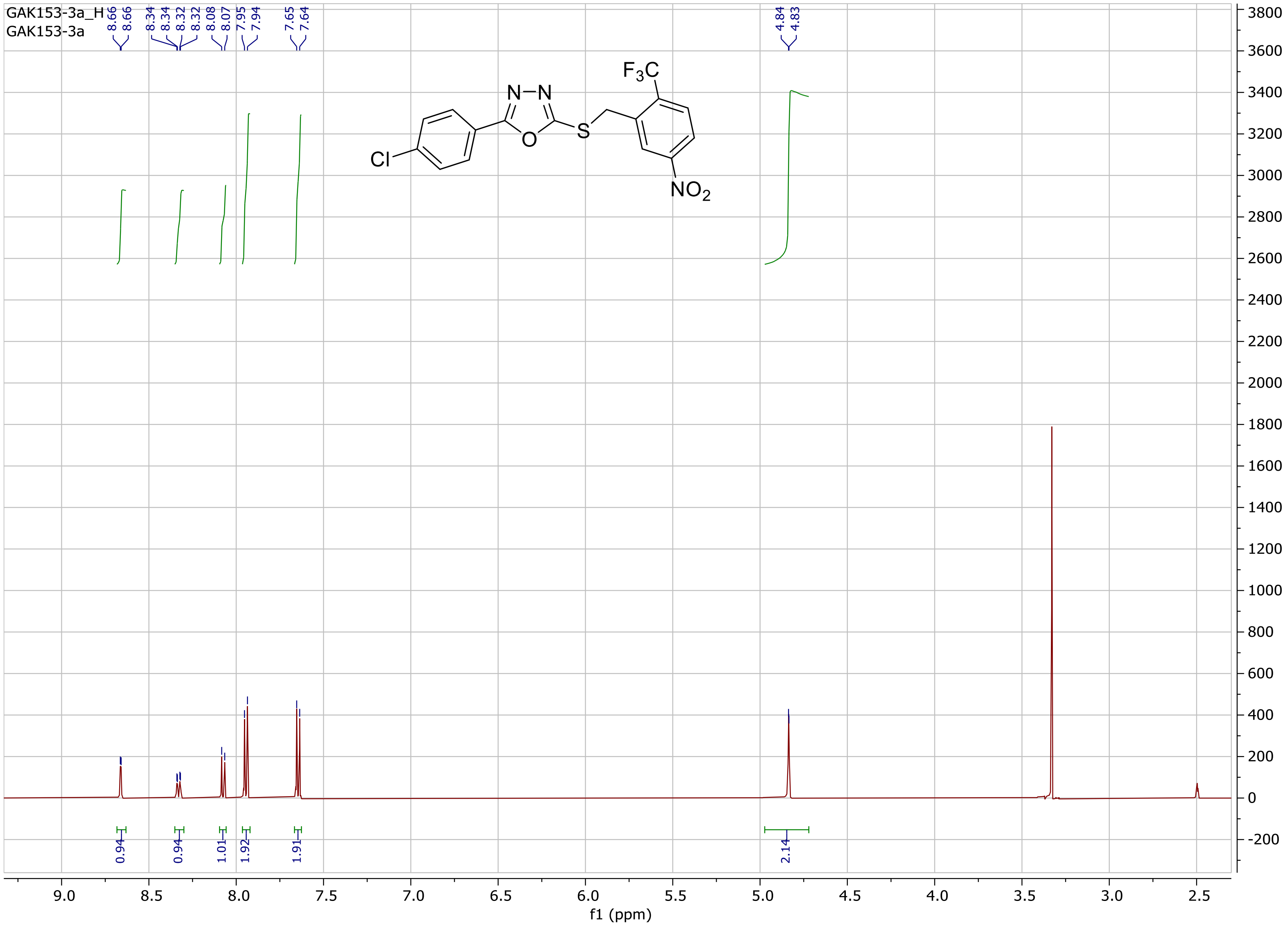

2-(4-Chlorophenyl)-5-((5-nitro-2-(trifluoromethyl)benzyl)sulfanyl)-1,3,4-oxadiazole (**73c**): ^{13}C NMR (126 MHz, DMSO-d_6)

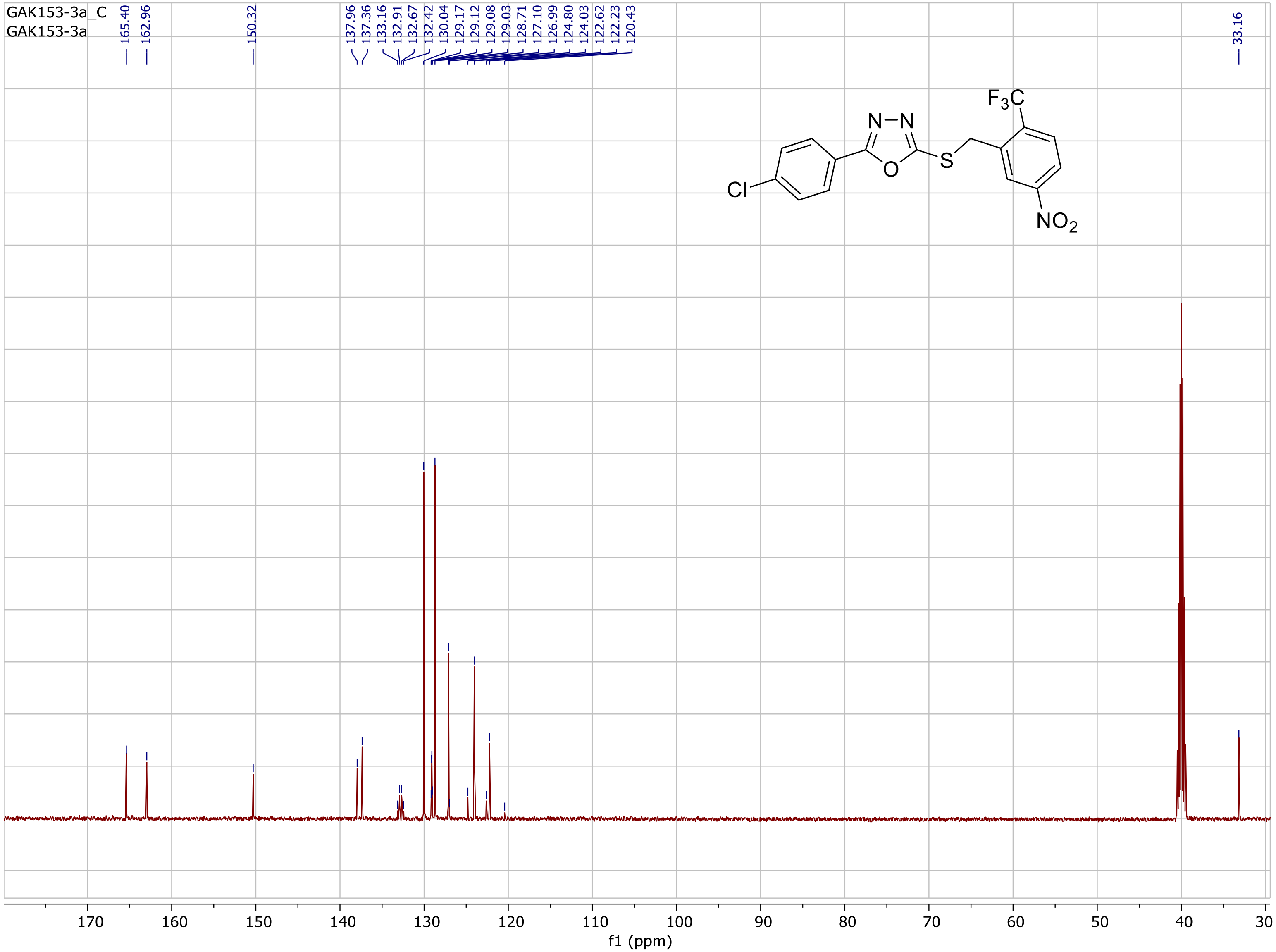

2-(4-Chlorophenyl)-5-((5-nitro-2-(trifluoromethyl)benzyl)sulfanyl)-1,3,4-oxadiazole (**73c**):

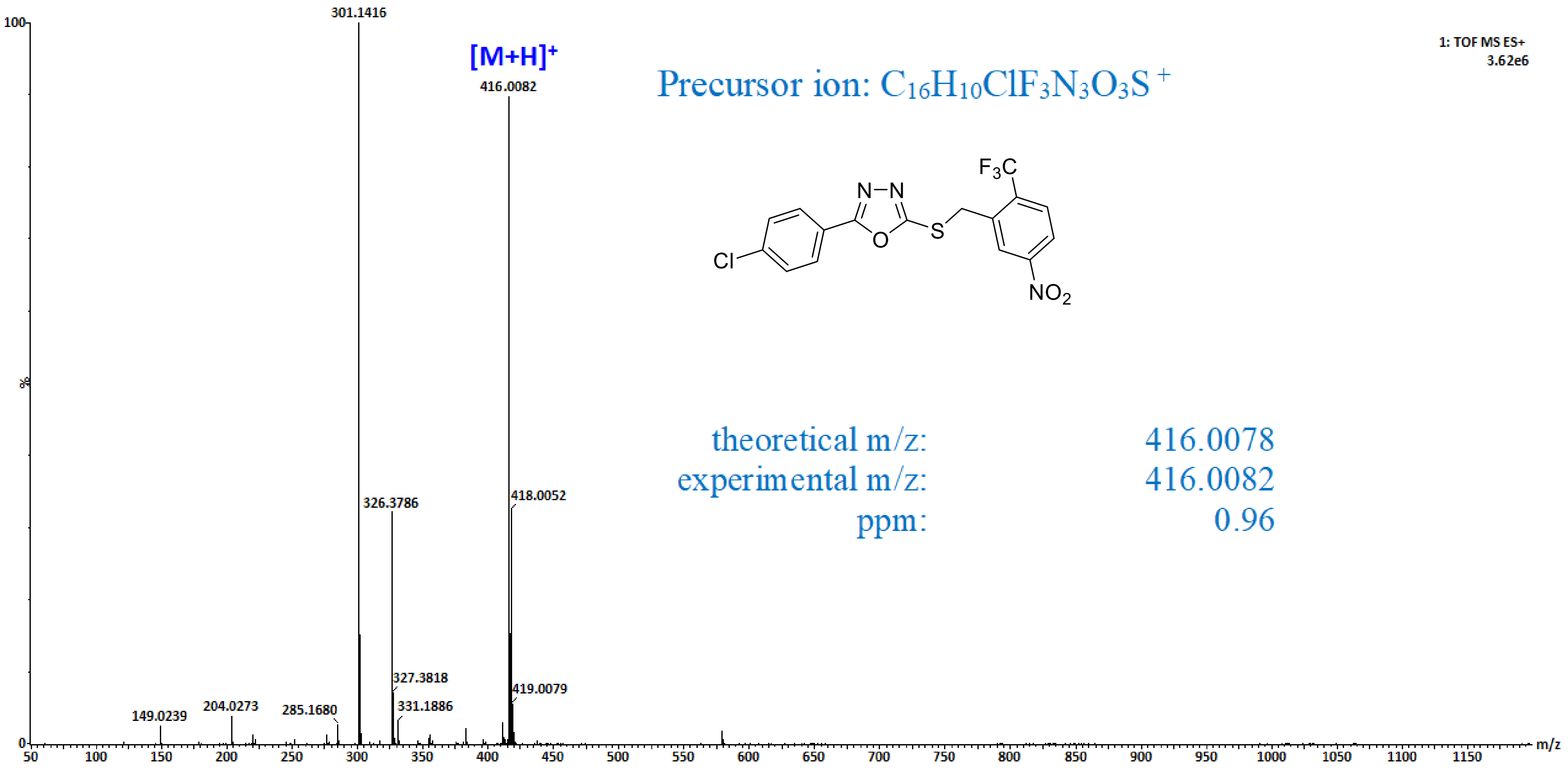

2-(4-Bromophenyl)-5-((5-nitro-2-(trifluoromethyl)benzyl)sulfanyl)-1,3,4-oxadiazole (**73d**): ^{1}H NMR (500 MHz, DMSO-d_6)

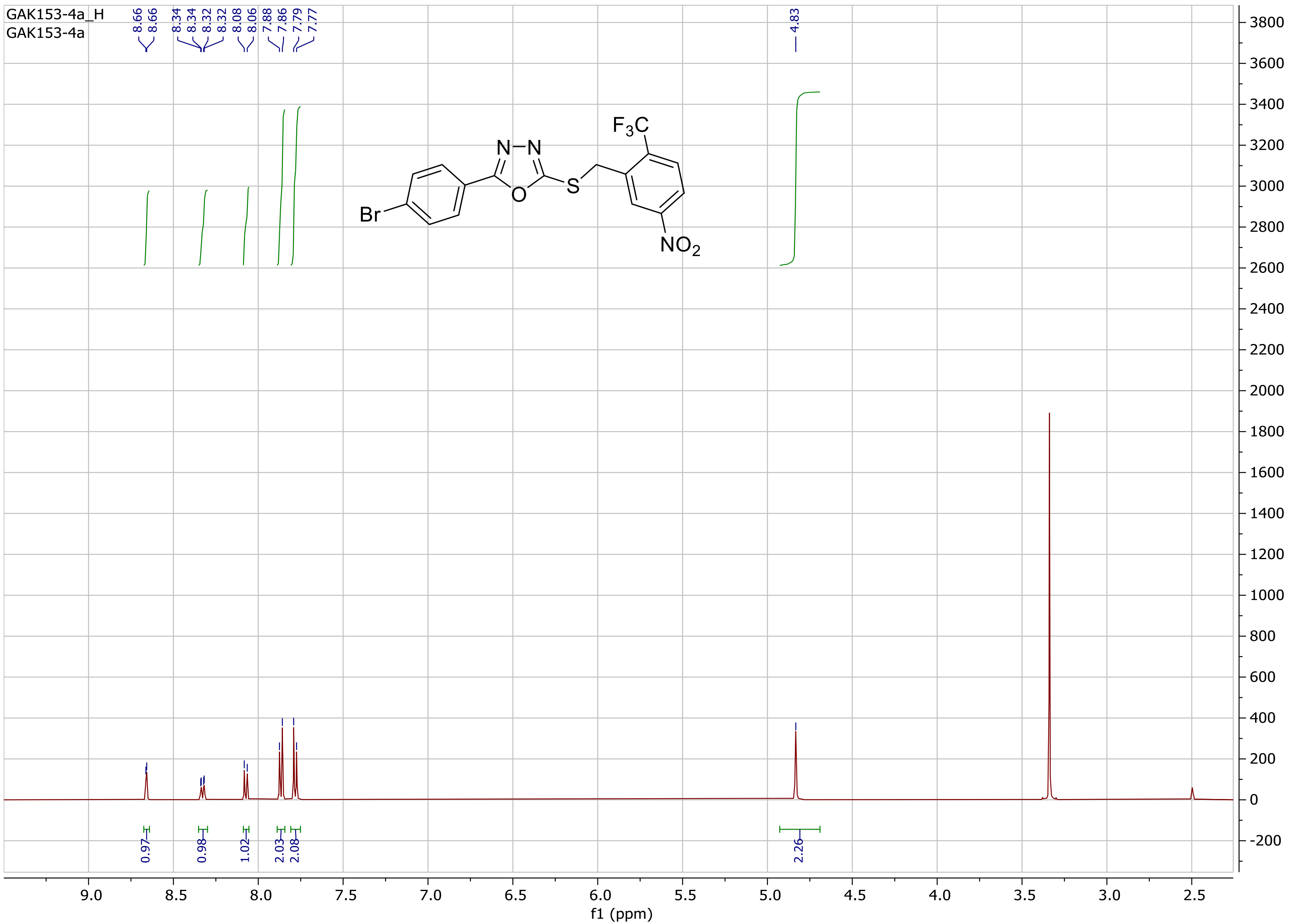

2-(4-Bromophenyl)-5-((5-nitro-2-(trifluoromethyl)benzyl)sulfanyl)-1,3,4-oxadiazole (**73d**): ^{13}C NMR (126 MHz, DMSO-d_6)

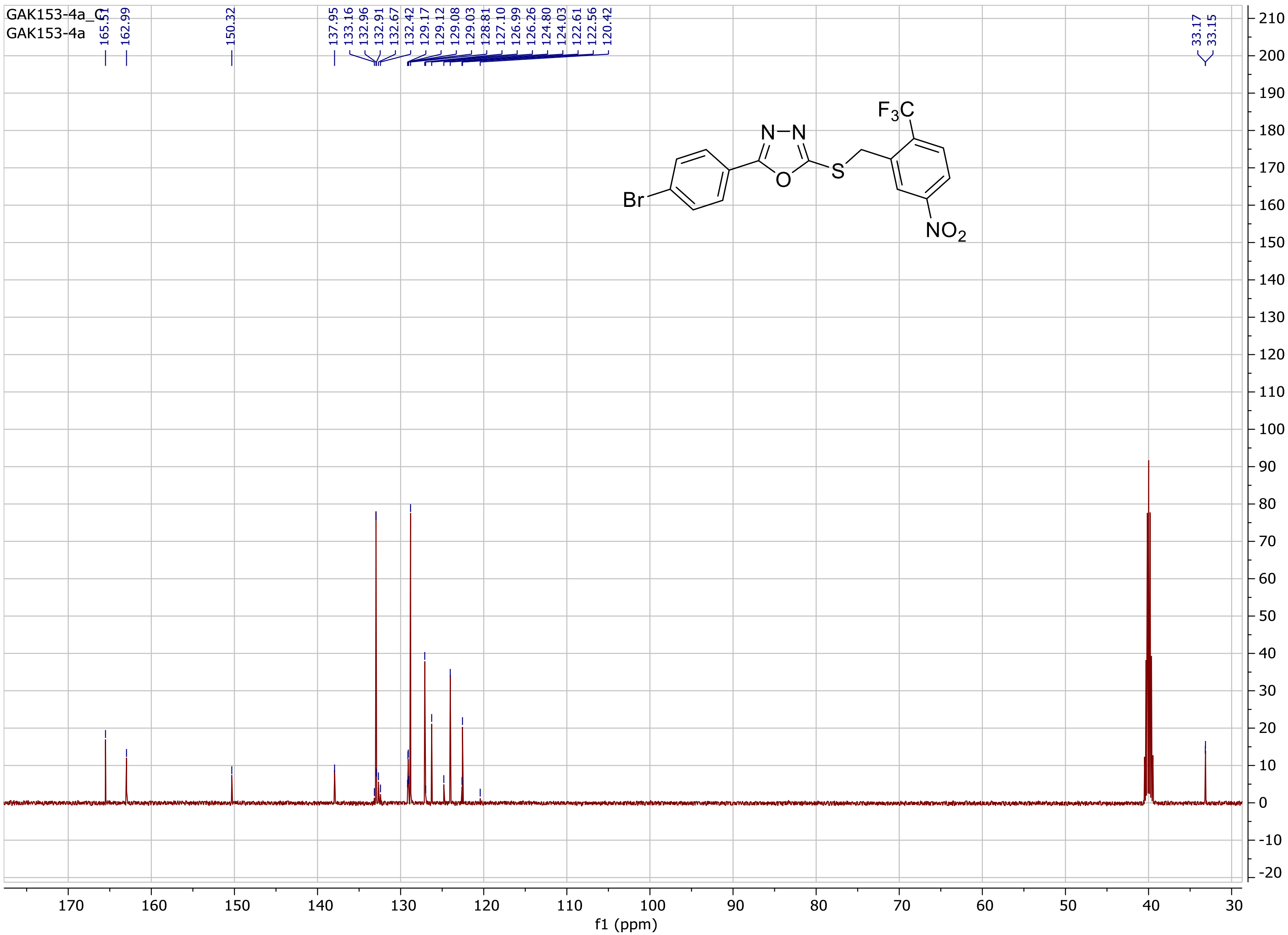

2-(4-Bromophenyl)-5-((5-nitro-2-(trifluoromethyl)benzyl)sulfanyl)-1,3,4-oxadiazole (**73d**):

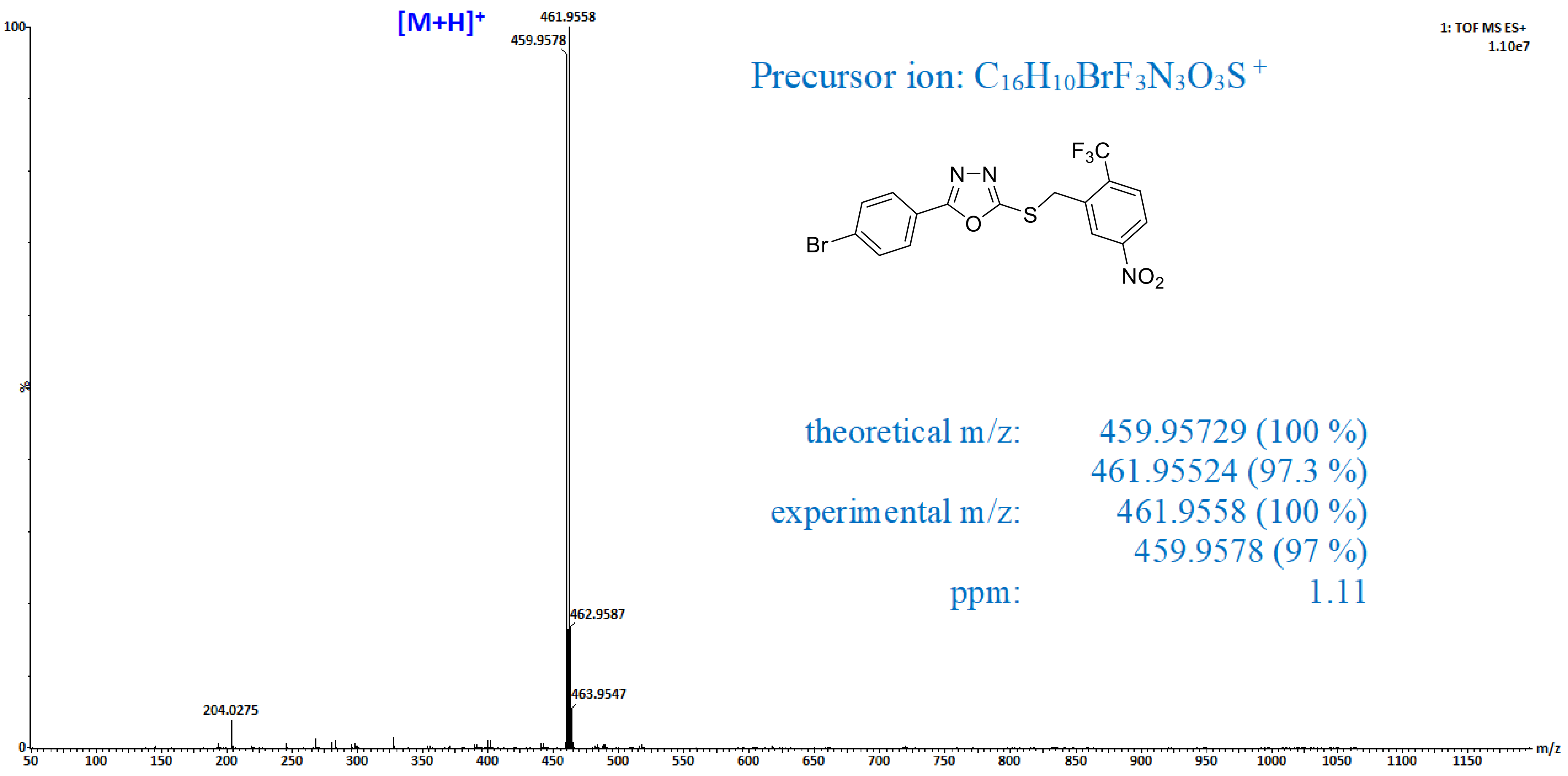

2-Cyclohexyl-5-((5-nitro-2-(trifluoromethyl)benzyl)sulfanyl)-1,3,4-oxadiazole (**73e**): ^{1}H NMR (500 MHz, DMSO-d_6)

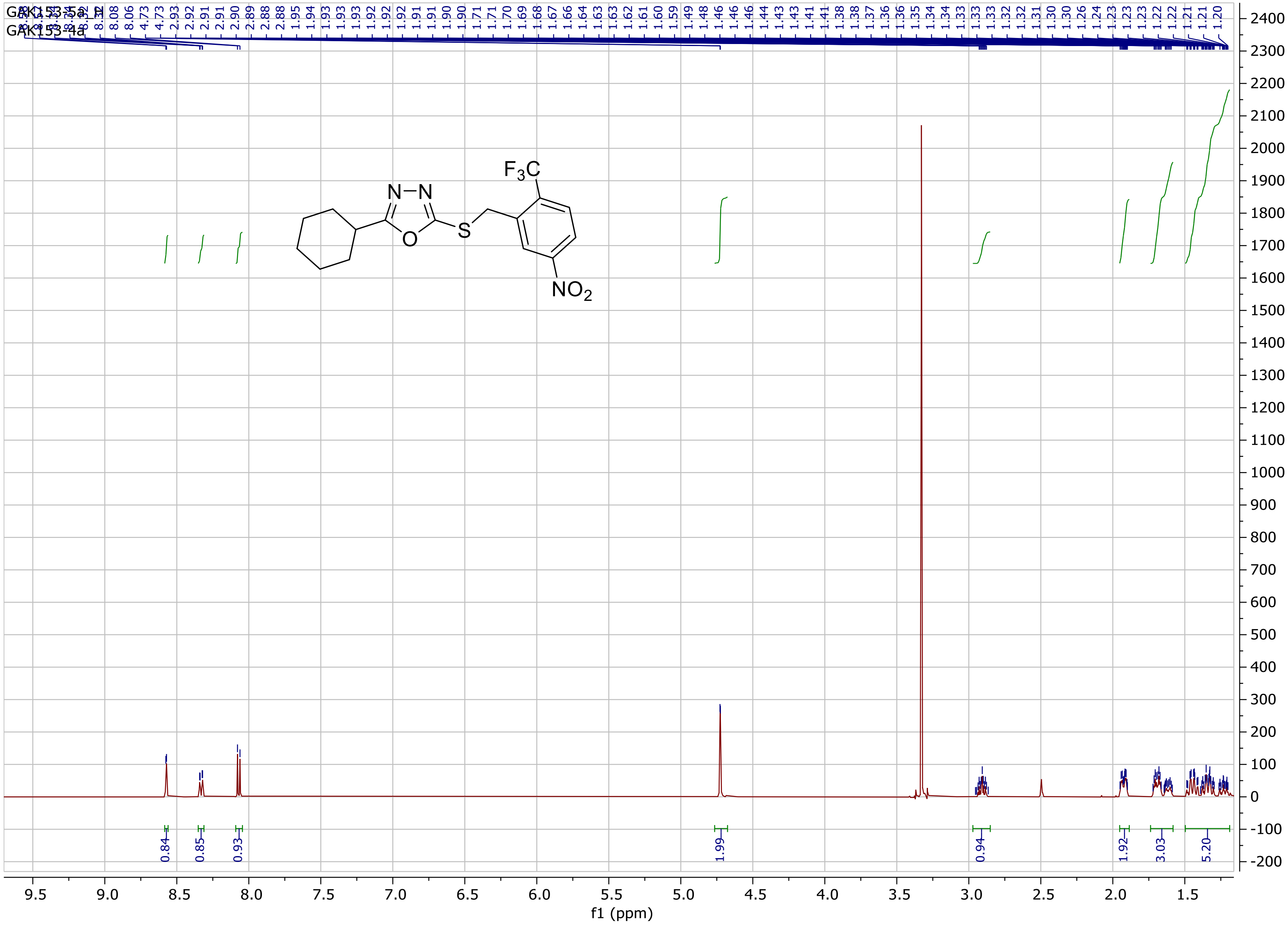

2-Cyclohexyl-5-((5-nitro-2-(trifluoromethyl)benzyl)sulfanyl)-1,3,4-oxadiazole (**73e**): ^{13}C NMR (126 MHz, DMSO-d_6)

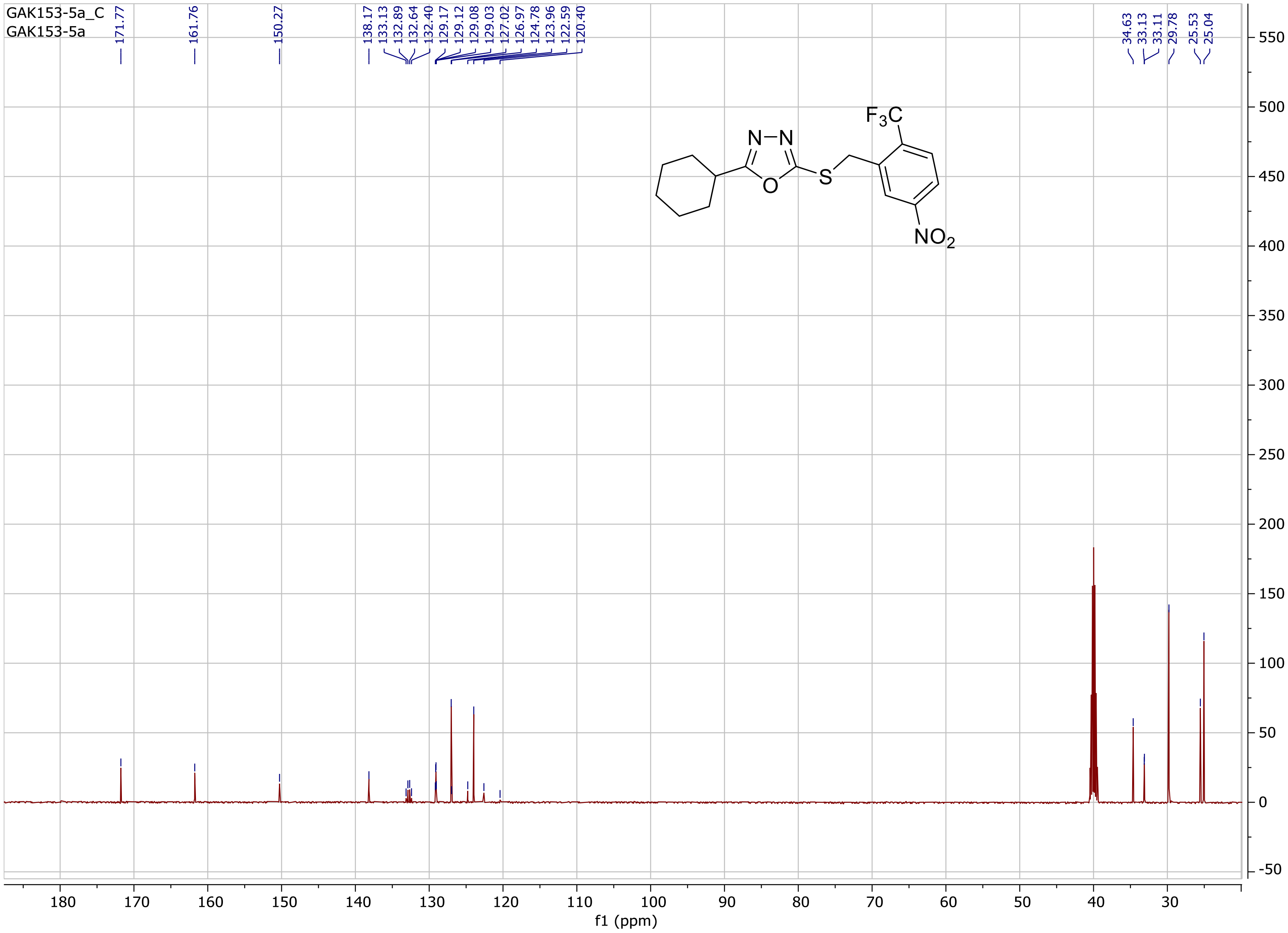

2-Cyclohexyl-5-((5-nitro-2-(trifluoromethyl)benzyl)sulfanyl)-1,3,4-oxadiazole (**73e**):

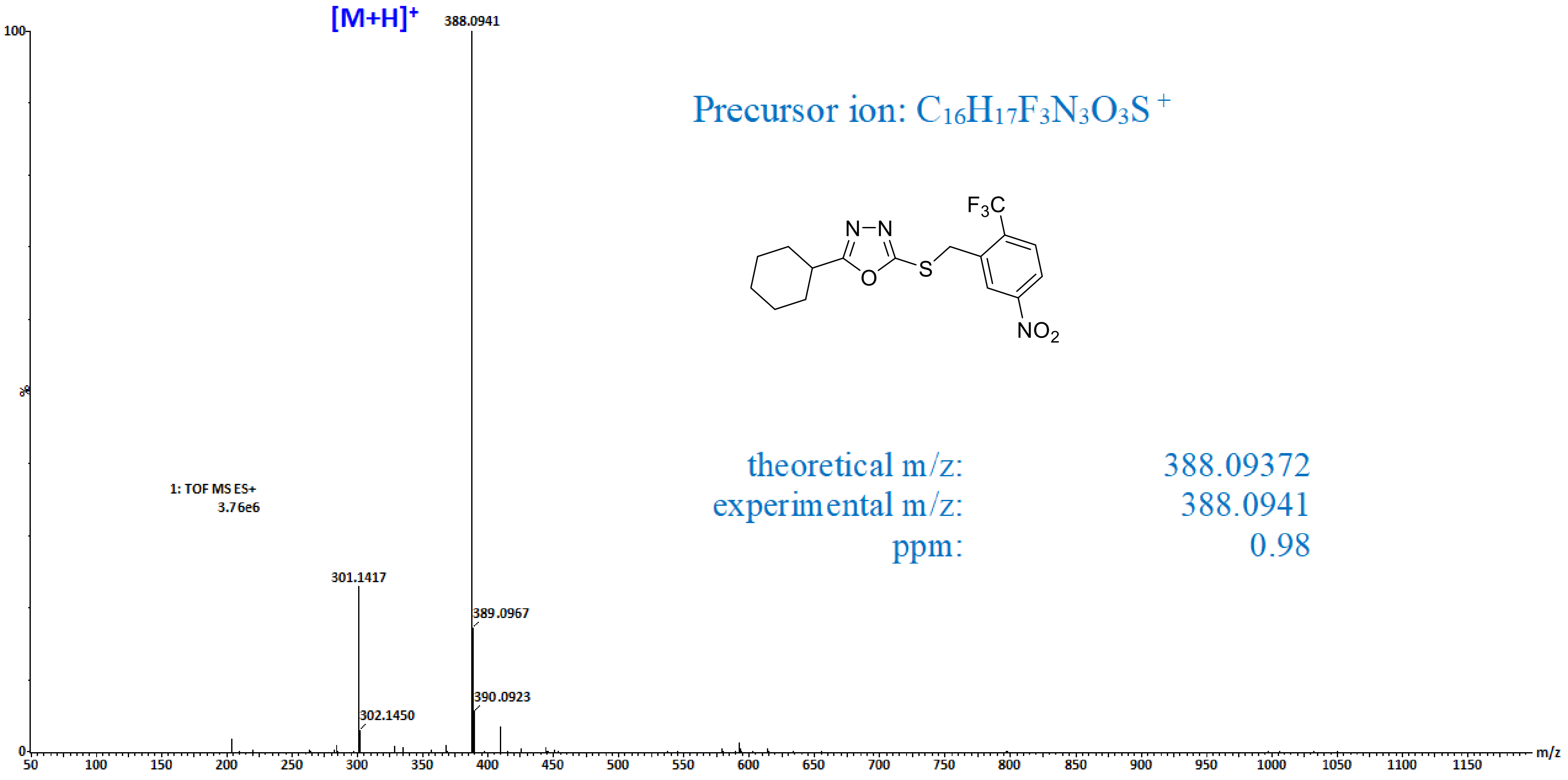

5-((4-Methoxy-3,5-dinitrobenzyl)sulfanyl)-1-phenyl-1H-tetrazole (**74a**): ^{1}H NMR (600 MHz, DMSO-d_6)

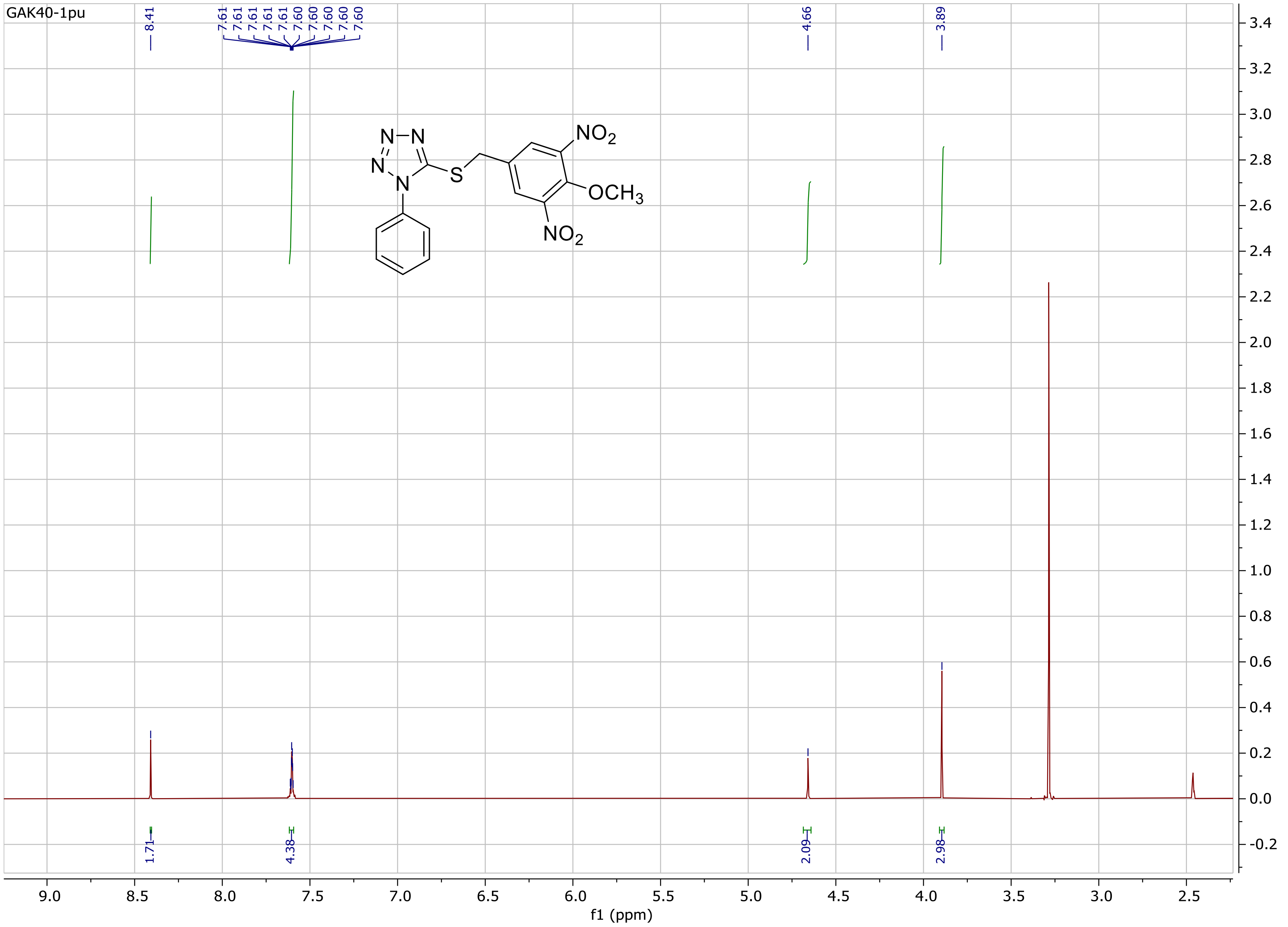

5-((4-Methoxy-3,5-dinitrobenzyl)sulfanyl)-1-phenyl-1H-tetrazole (**74a**): ^{13}C NMR (151 MHz, DMSO-d_6)

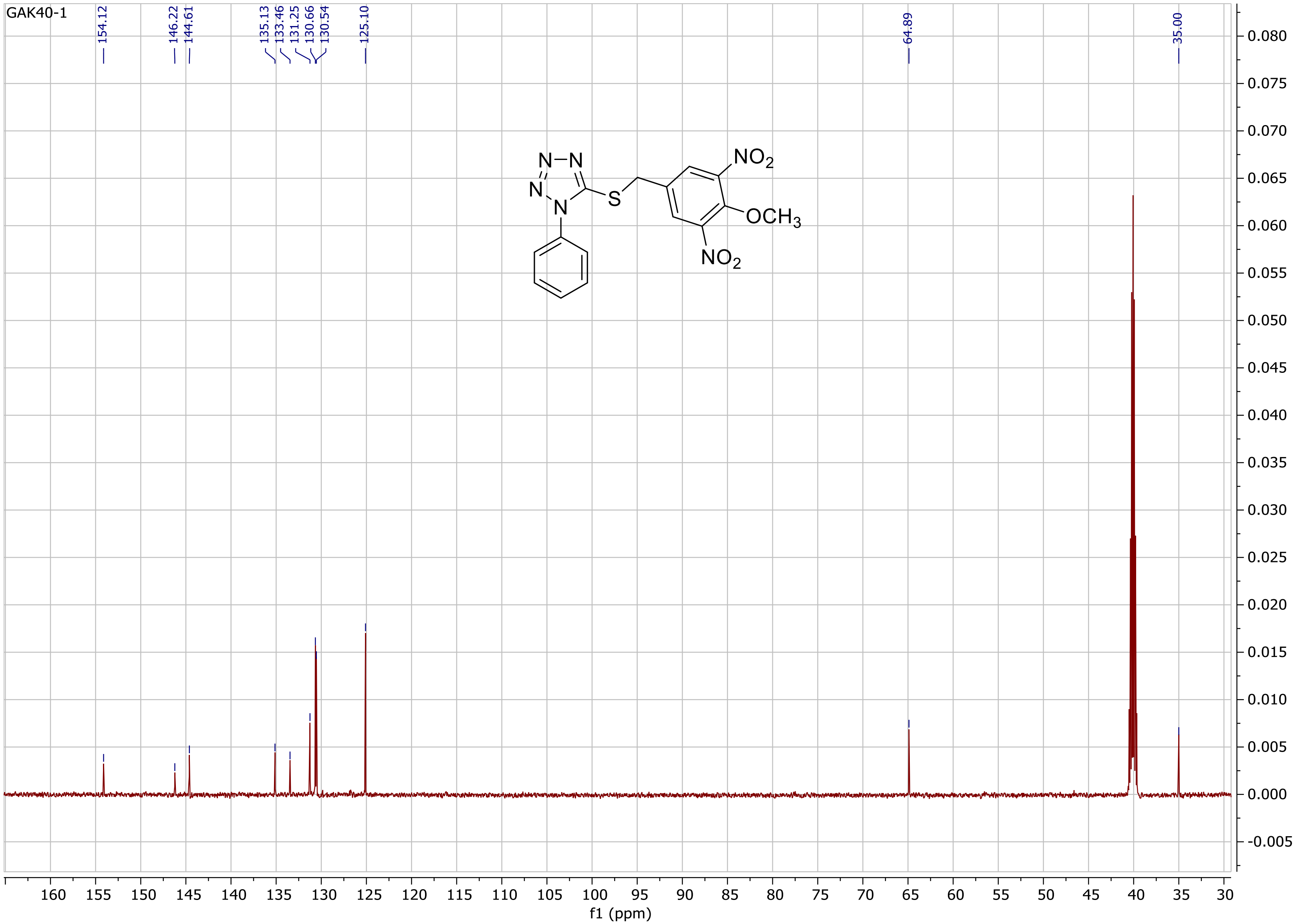

5-((4-Methoxy-3,5-dinitrobenzyl)sulfanyl)-1-(4-methoxyphenyl)-1H-tetrazole (**74b**): ^{1}H NMR (600 MHz, DMSO-d_6)

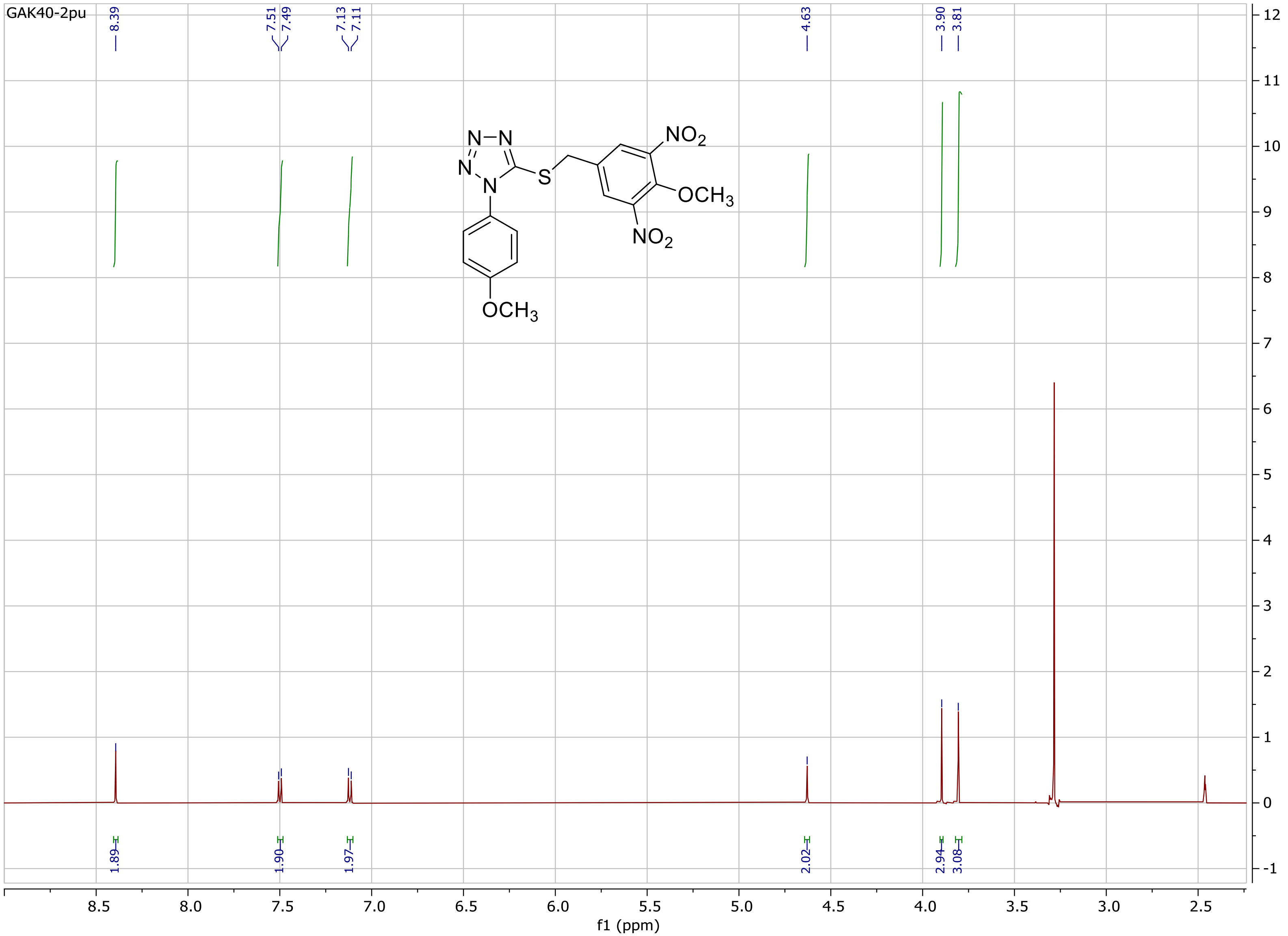

5-((4-Methoxy-3,5-dinitrobenzyl)sulfanyl)-1-(4-methoxyphenyl)-1H-tetrazole (**74b**): ^{13}C NMR (151 MHz, DMSO-d_6)

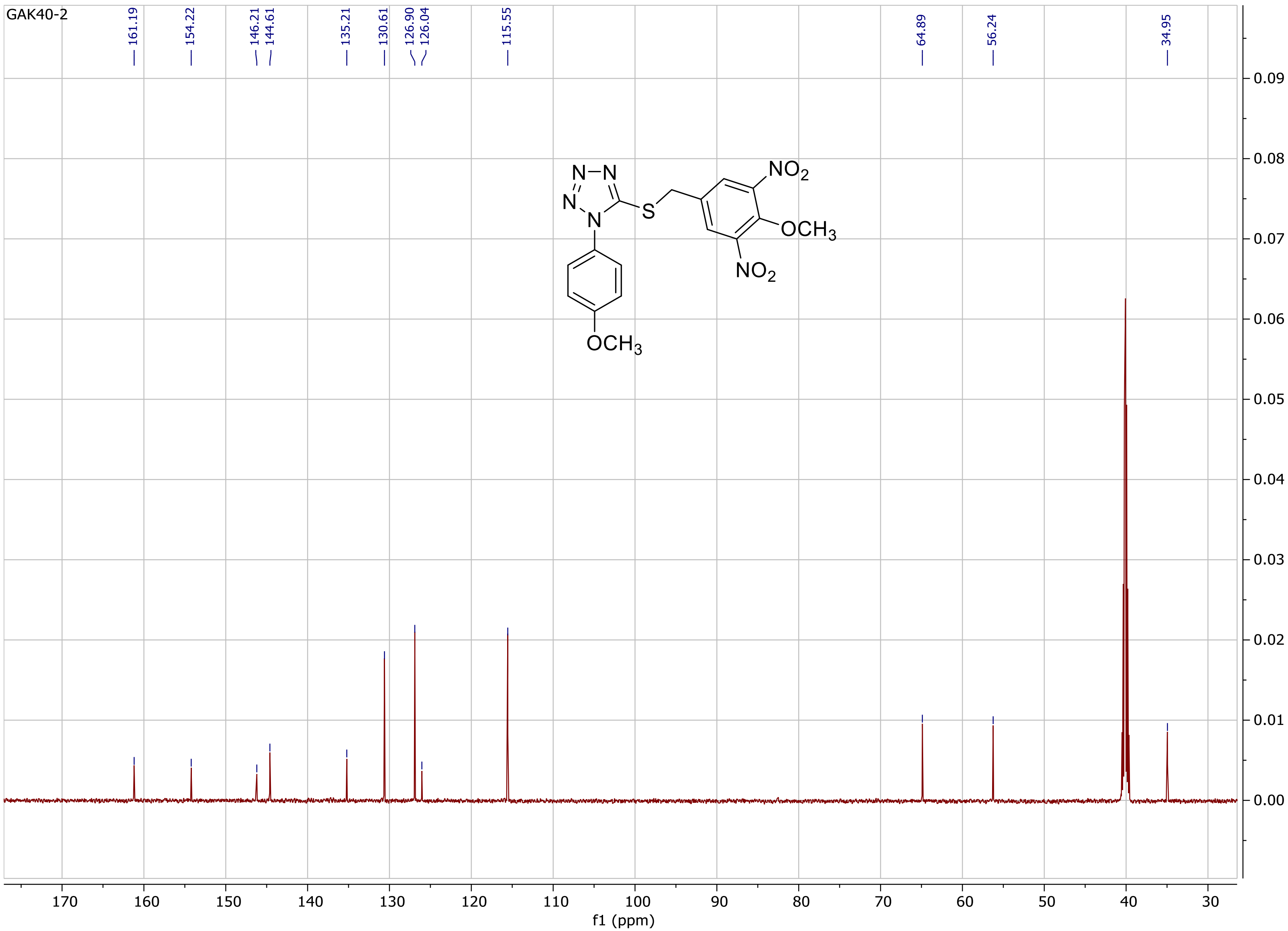

1-(4-Chlorophenyl)-5-((4-methoxy-3,5-dinitrobenzyl)sulfanyl)-1H-tetrazole (**74c**): ^{1}H NMR (600 MHz, DMSO-d_6)

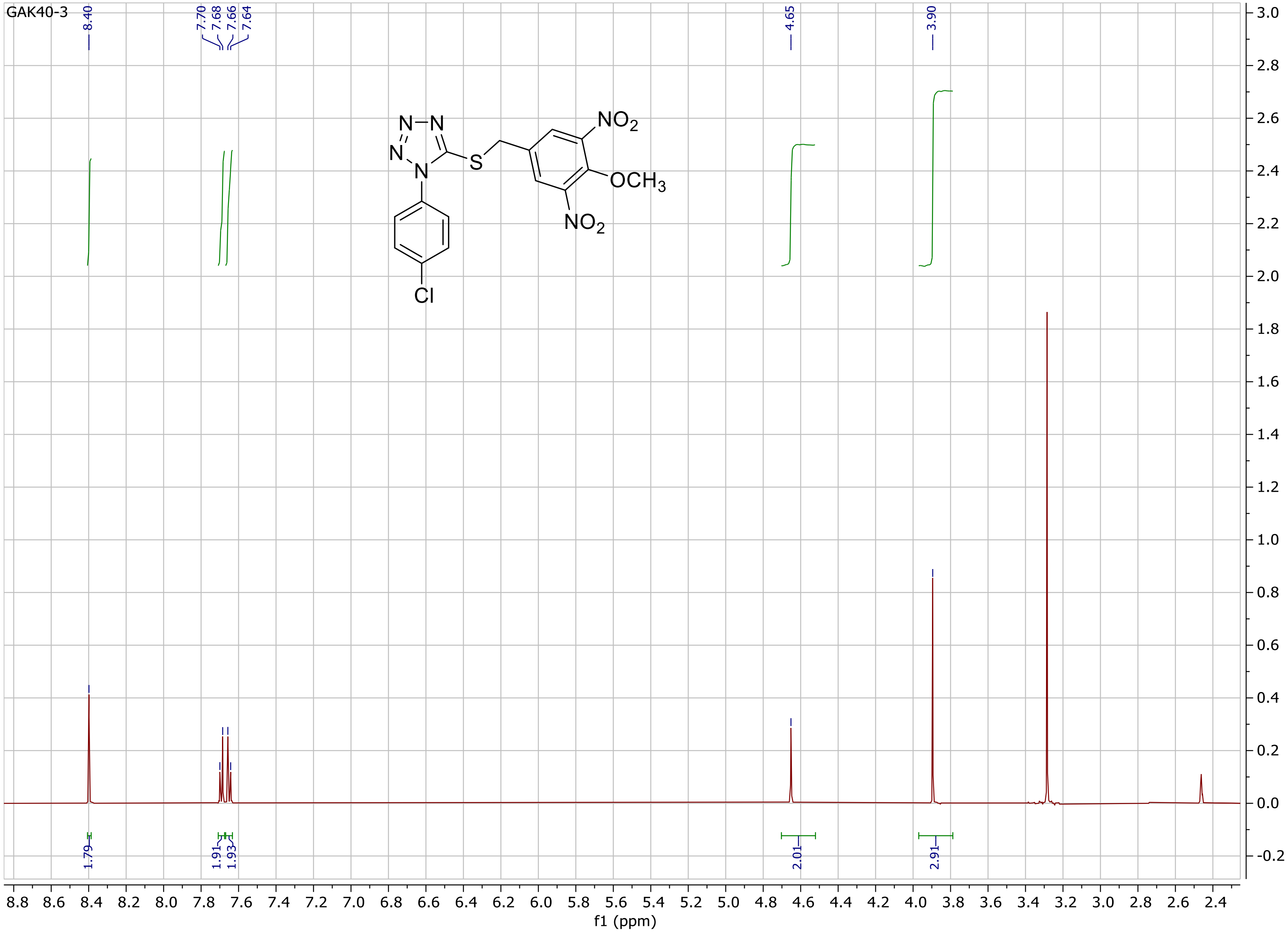

1-(4-Chlorophenyl)-5-((4-methoxy-3,5-dinitrobenzyl)sulfanyl)-1H-tetrazole (**74c**): ^{13}C NMR (151 MHz, DMSO-d_6)

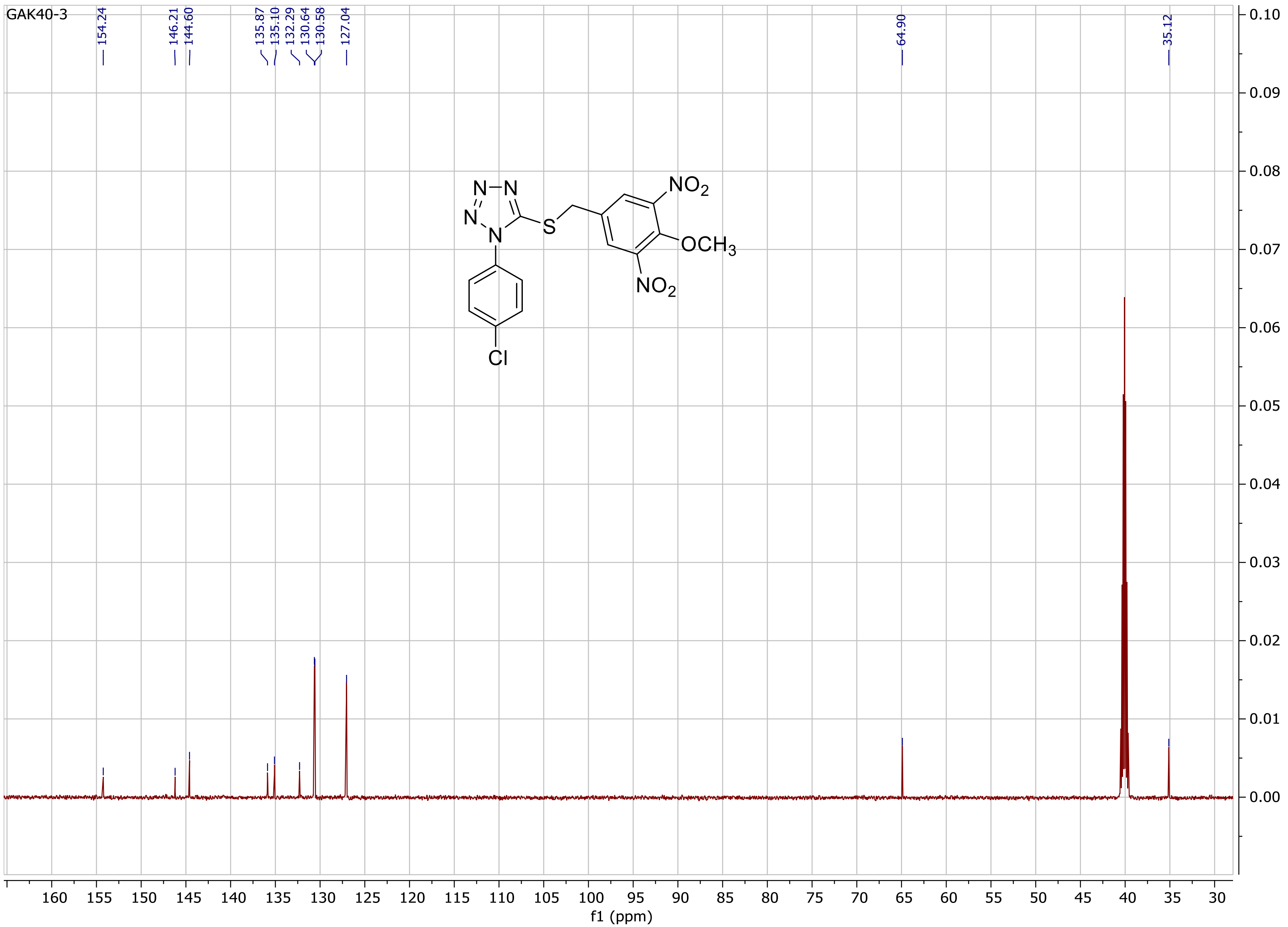

1-(4-Bromophenyl)-5-((4-methoxy-3,5-dinitrobenzyl)sulfanyl)-1H-tetrazole (**74d**): ^{1}H NMR (600 MHz, DMSO-d_6)

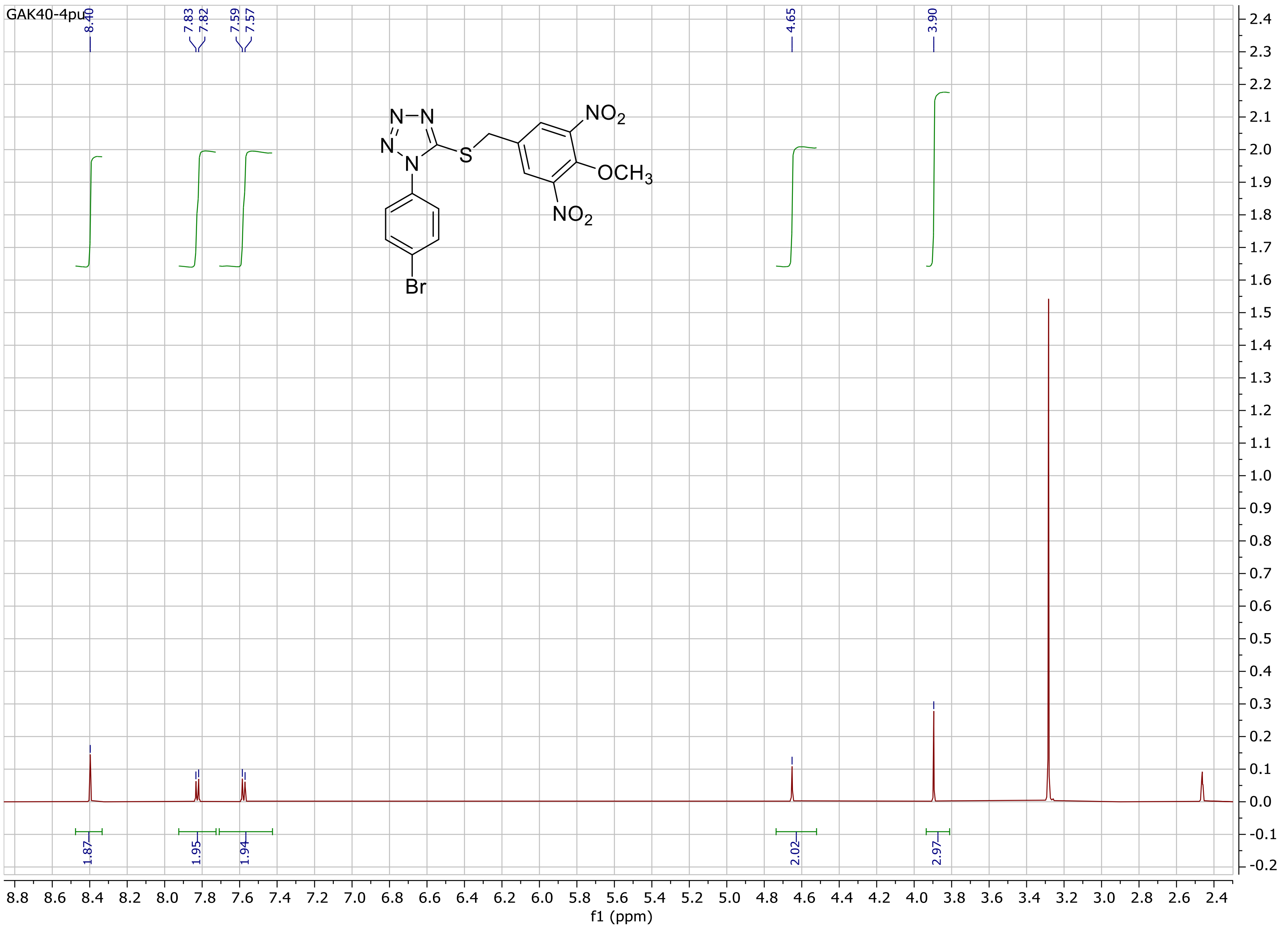

1-(4-Bromophenyl)-5-((4-methoxy-3,5-dinitrobenzyl)sulfanyl)-1H-tetrazole (**74d**): ^{13}C NMR (151 MHz, DMSO-*d*$_6$)

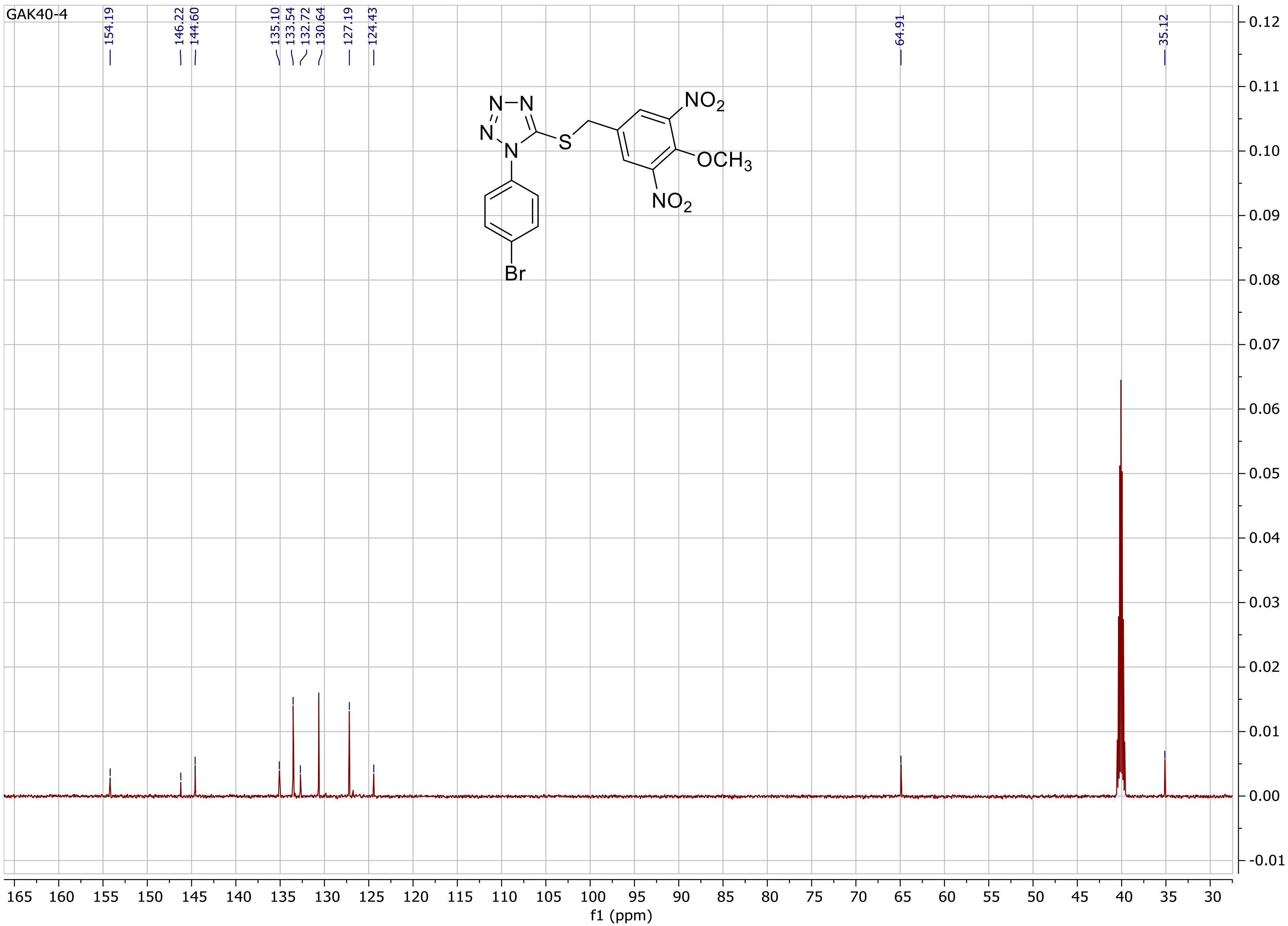

1-Cyclohexyl-5-((4-methoxy-3,5-dinitrobenzyl)sulfanyl)-1H-tetrazole (**74e**): ^{1}H NMR (600 MHz, DMSO-d_6)

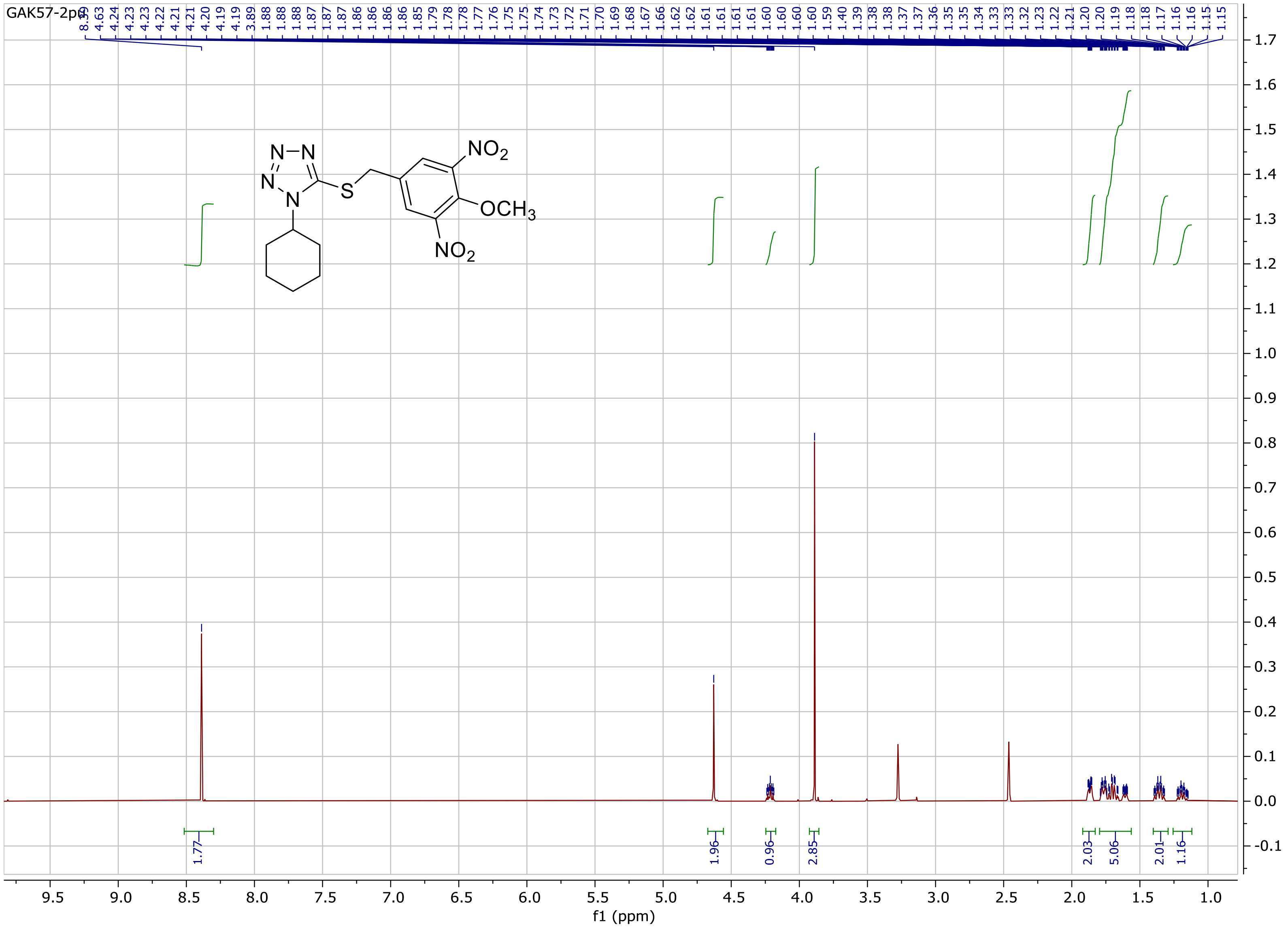

1-Cyclohexyl-5-((4-methoxy-3,5-dinitrobenzyl)sulfanyl)-1H-tetrazole (**74e**): ^{13}C NMR (126 MHz, DMSO-d_6)

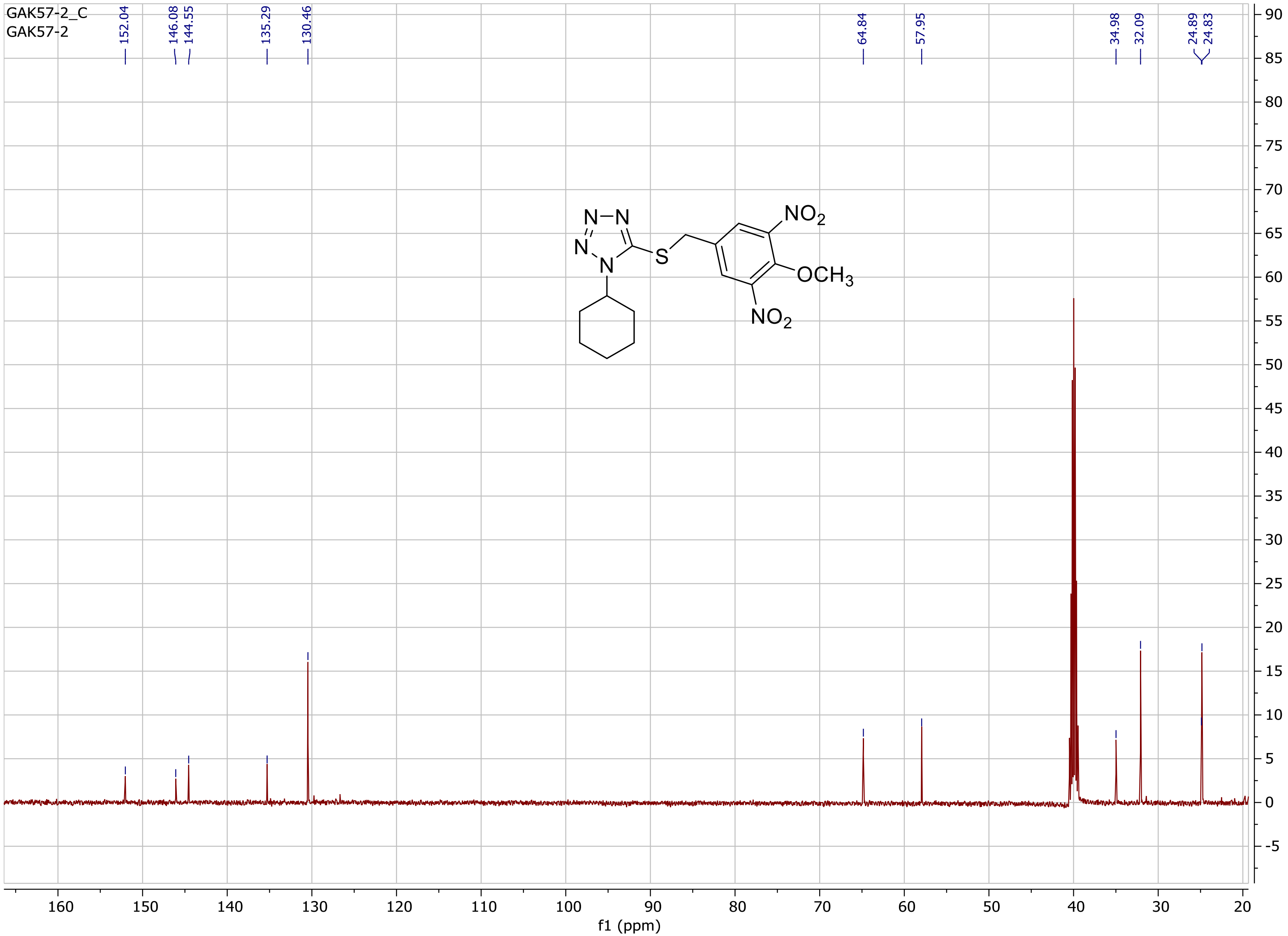

1-Cyclohexyl-5-((4-methoxy-3,5-dinitrobenzyl)sulfanyl)-1H-tetrazole (**74e**):

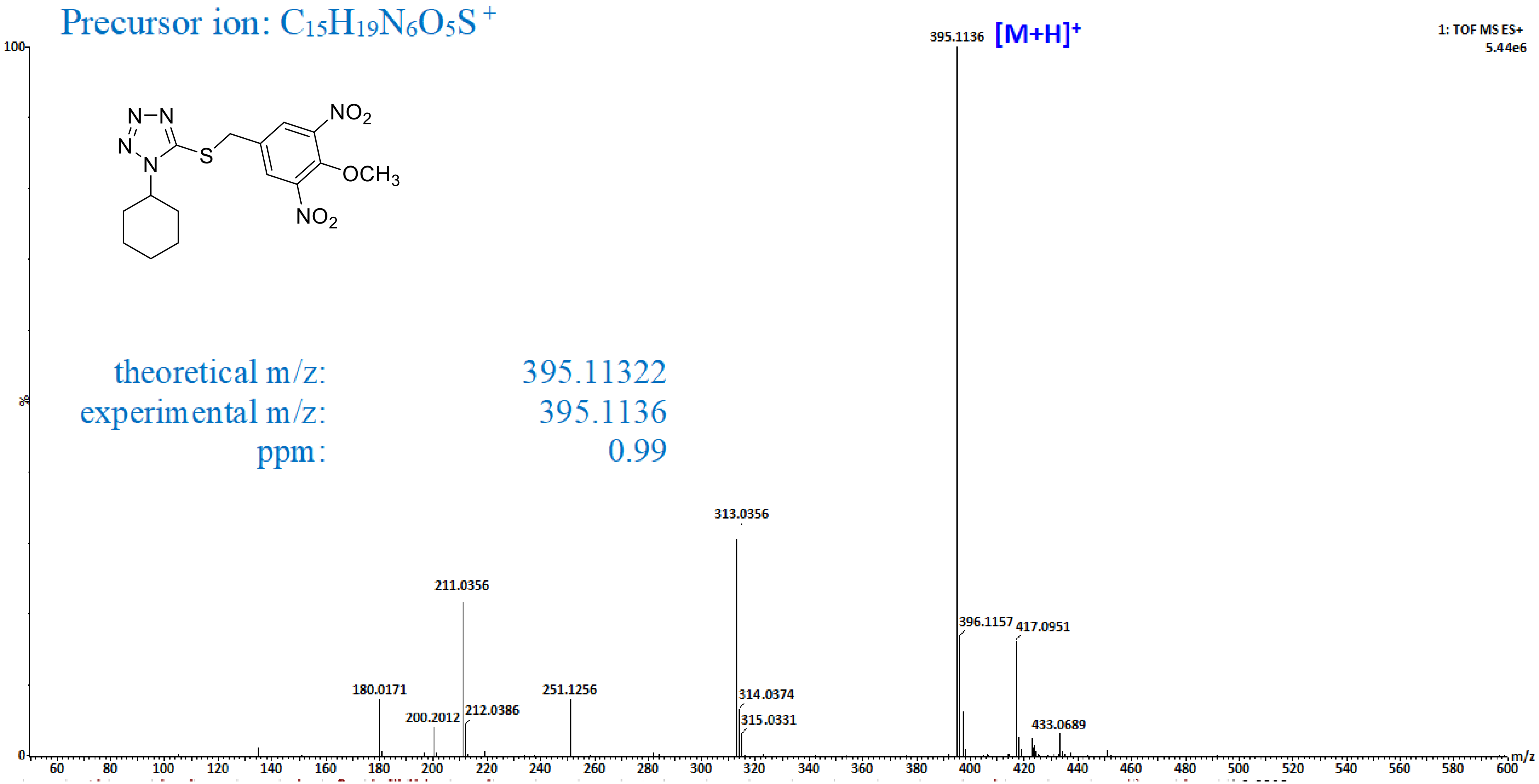

5-((2-Methoxy-3,5-dinitrobenzyl)sulfanyl)-1-phenyl-1H-tetrazole (**75a**): ^{1}H NMR (500 MHz, DMSO-d_6)

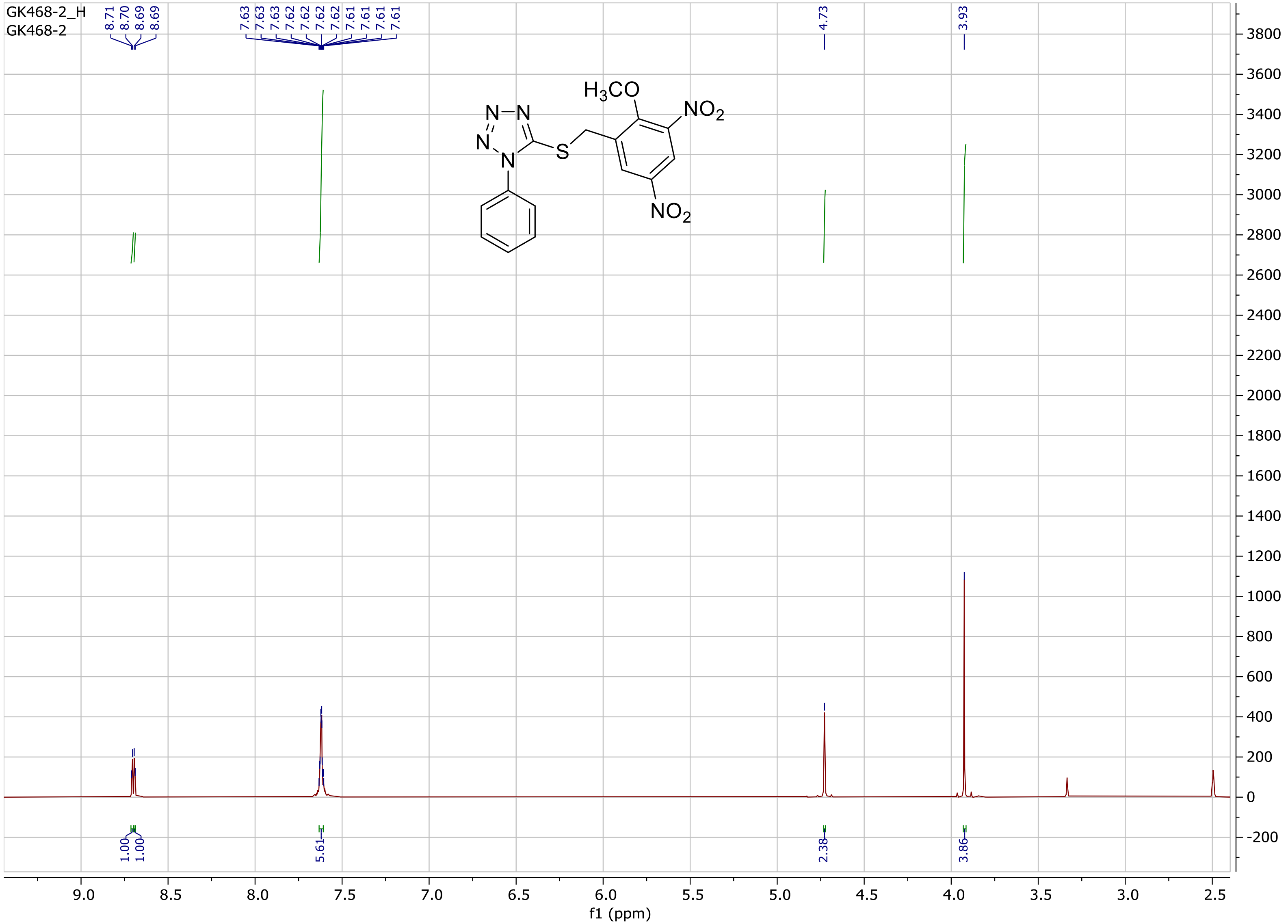

5-((2-Methoxy-3,5-dinitrobenzyl)sulfanyl)-1-phenyl-1H-tetrazole (**75a**): ^{13}C NMR (126 MHz, DMSO-d_6)

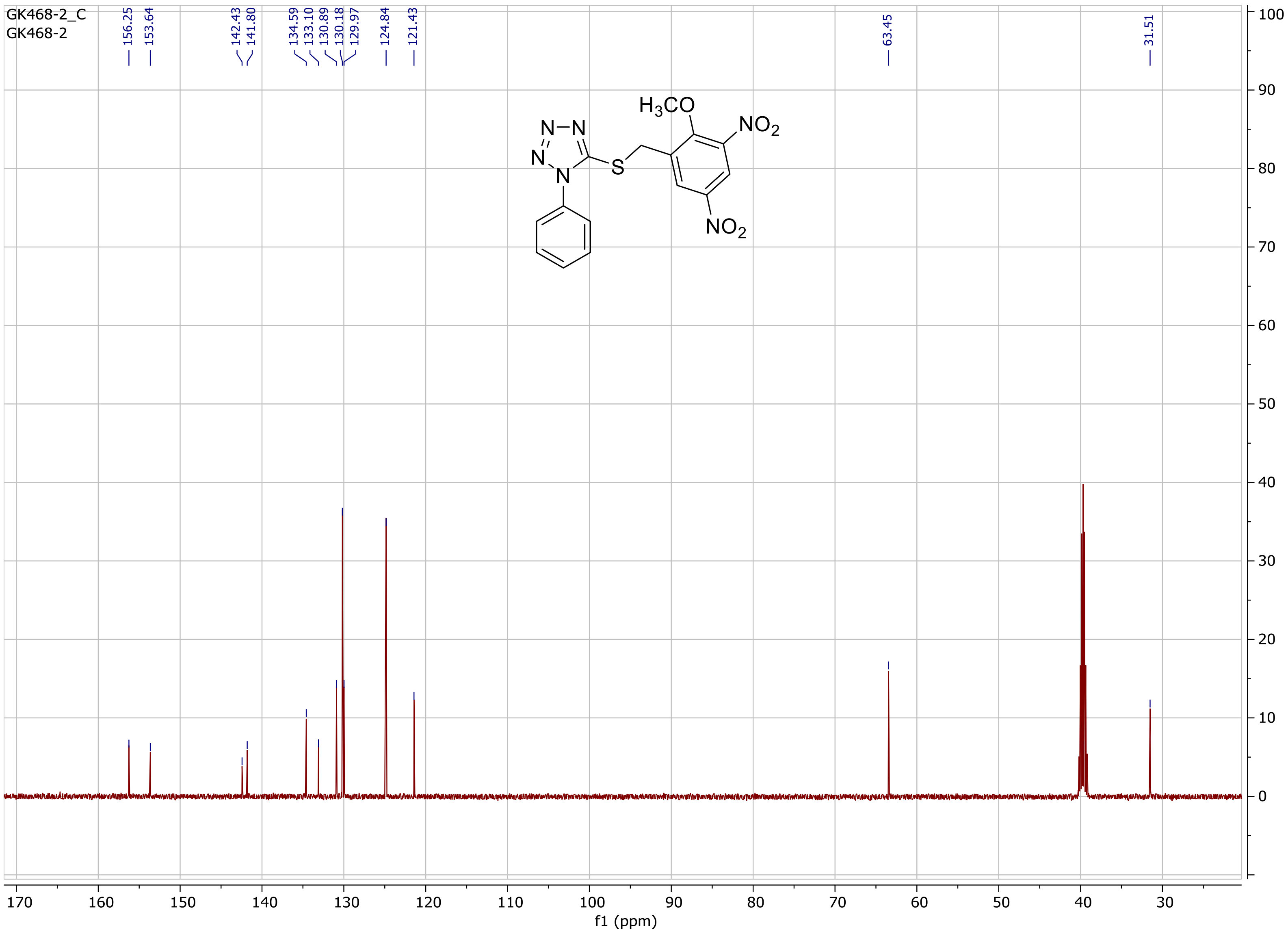

5-((2-Methoxy-3,5-dinitrobenzyl)sulfanyl)-1-(4-methoxyphenyl)-1H-tetrazole (**75b**): ^{1}H NMR (500 MHz, DMSO-d_6)

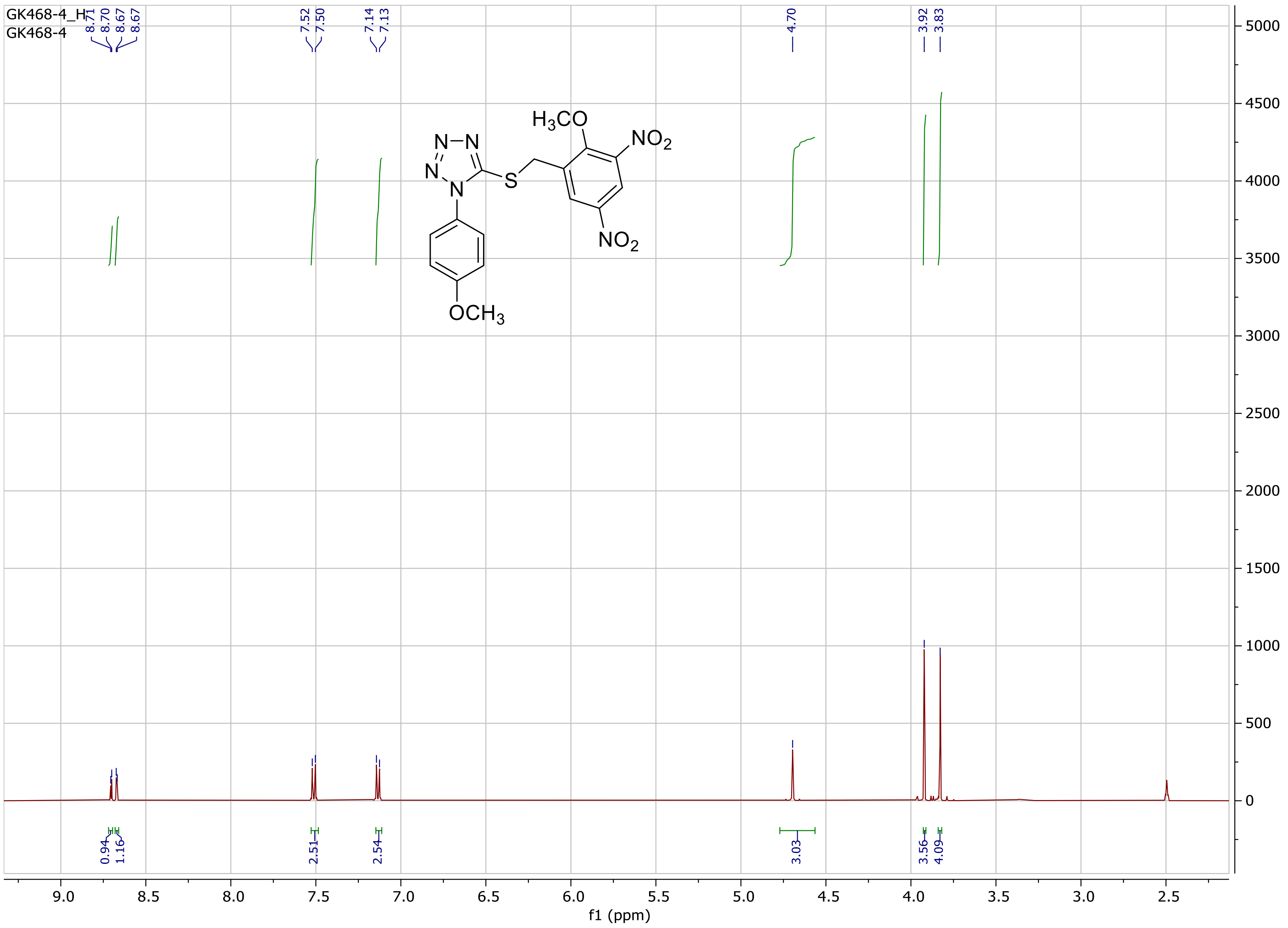

5-((2-Methoxy-3,5-dinitrobenzyl)sulfanyl)-1-(4-methoxyphenyl)-1H-tetrazole (**75b**): ^{13}C NMR (126 MHz, DMSO-d_6)

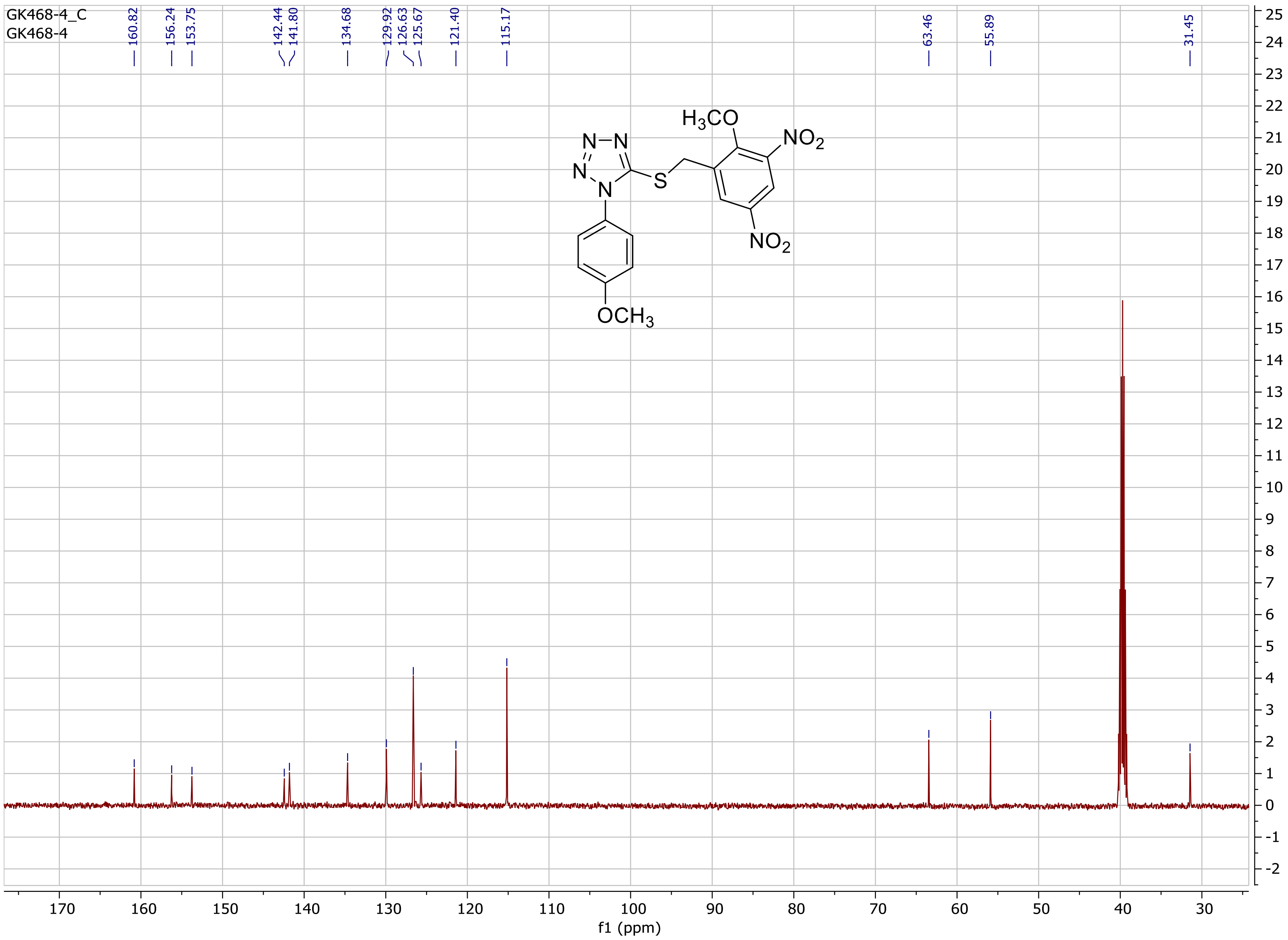

1-(4-Chlorophenyl)-5-((2-methoxy-3,5-dinitrobenzyl)sulfanyl)-1H-tetrazole (**75c**): ^{1}H NMR (500 MHz, DMSO-d_6)

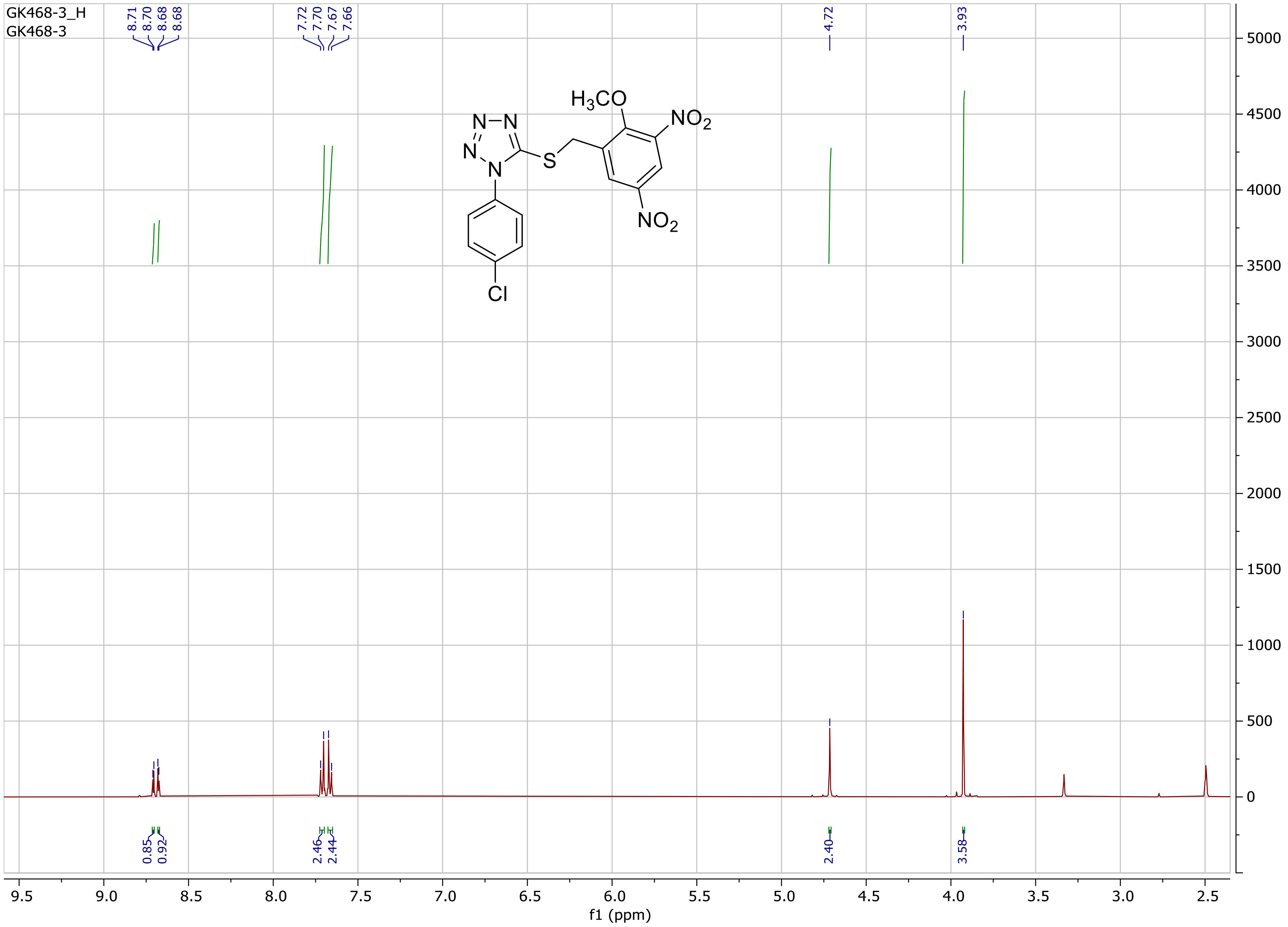

1-(4-Chlorophenyl)-5-((2-methoxy-3,5-dinitrobenzyl)sulfanyl)-1H-tetrazole (**75c**): ^{13}C NMR (126 MHz, DMSO-d_6)

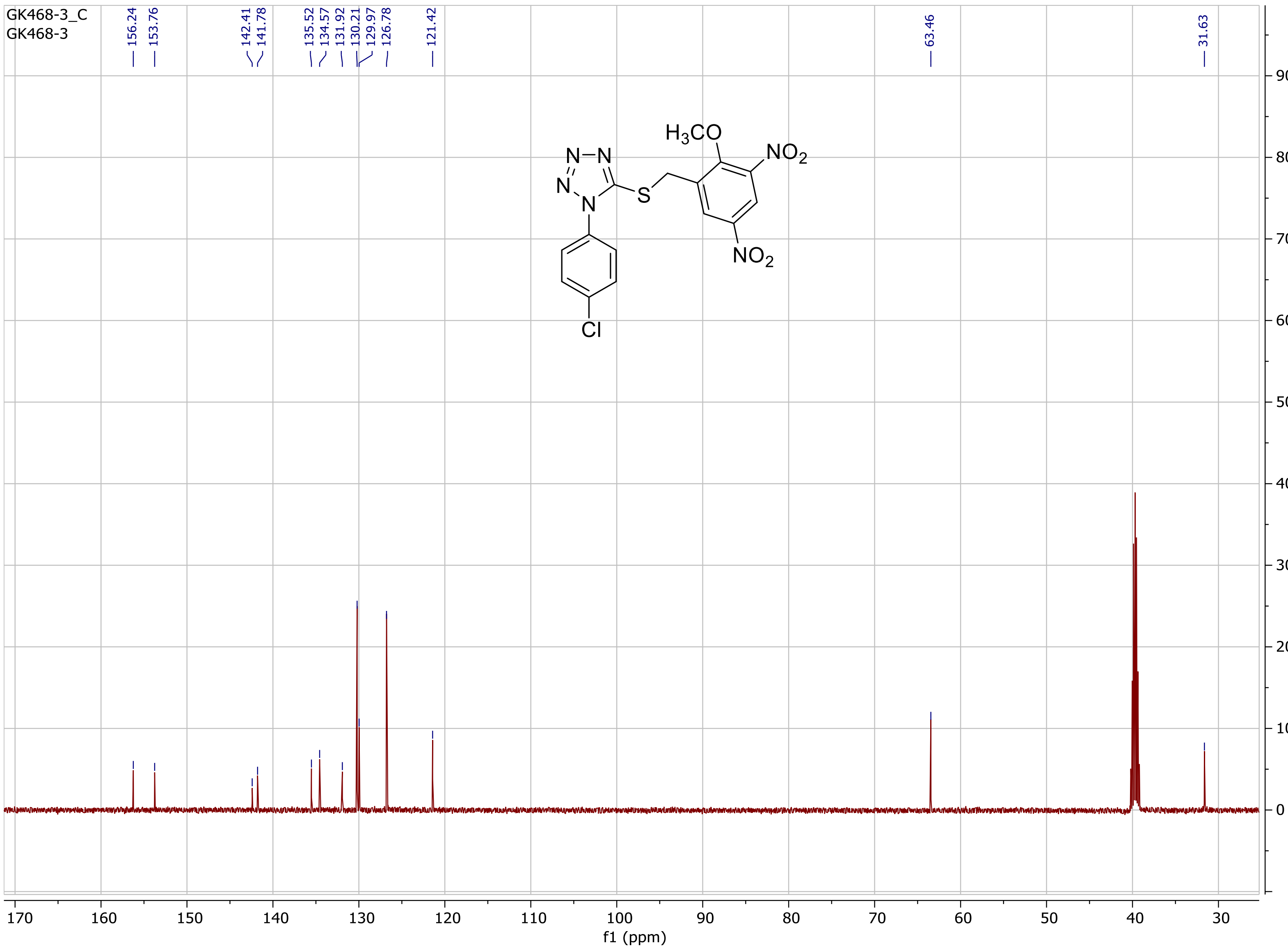

1-(4-Bromophenyl)-5-((2-methoxy-3,5-dinitrobenzyl)sulfanyl)-1H-tetrazole (**75d**): ^{1}H NMR (500 MHz, DMSO-d_6)

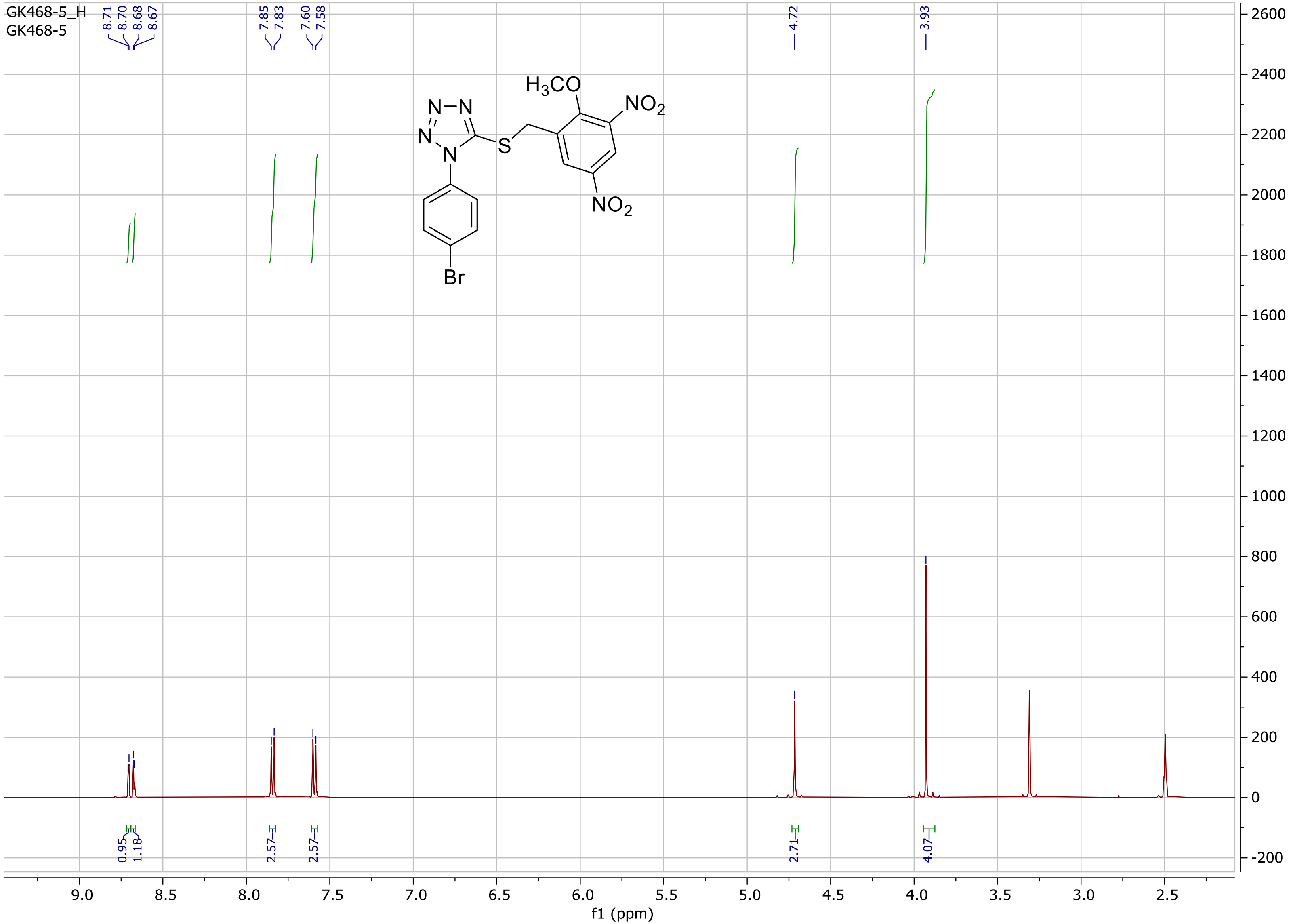

1-(4-Bromophenyl)-5-((2-methoxy-3,5-dinitrobenzyl)sulfanyl)-1H-tetrazole (**75d**): ^{13}C NMR (126 MHz, DMSO-d_6)

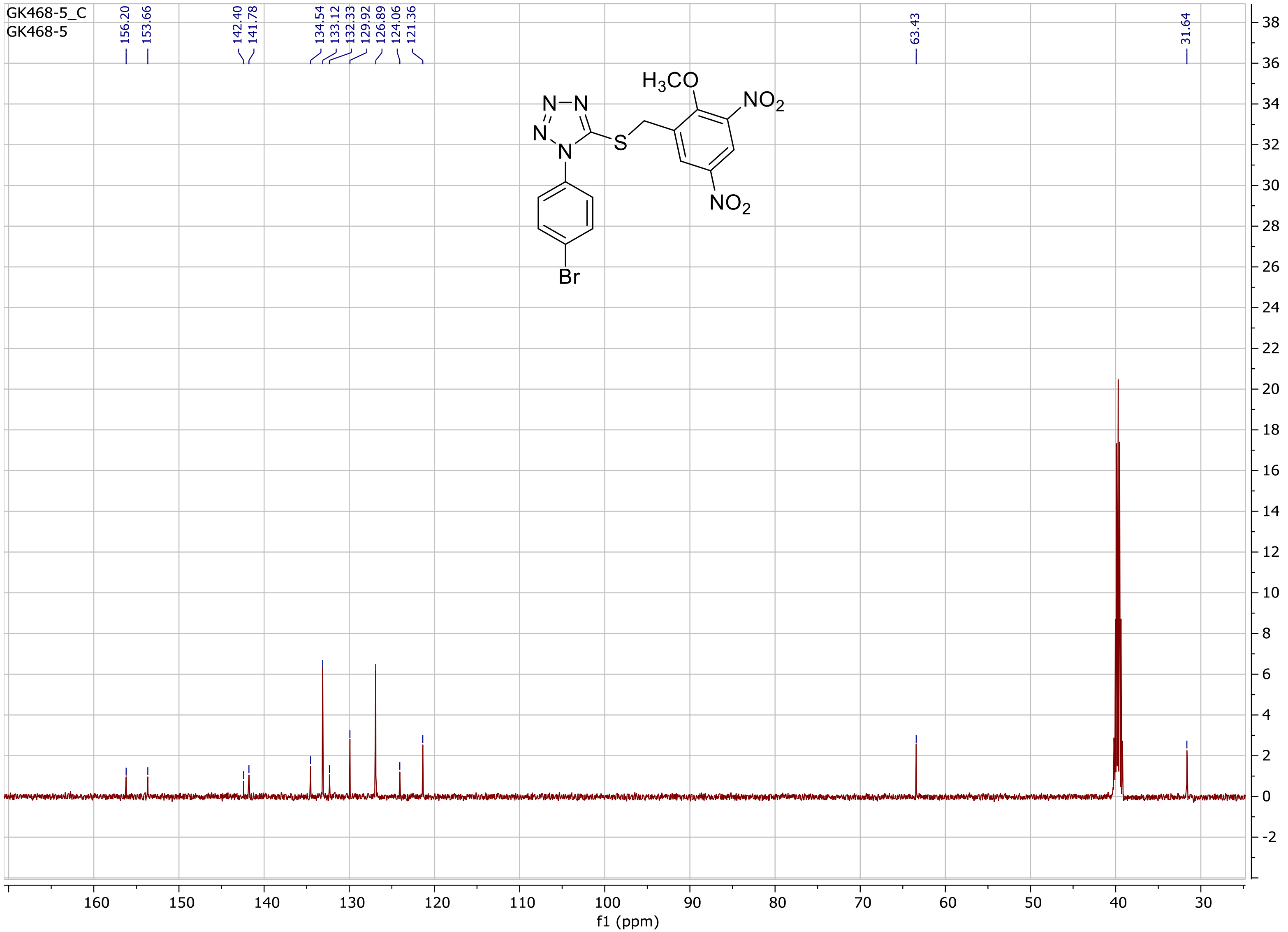

1-Cyclohexyl-5-((2-methoxy-3,5-dinitrobenzyl)sulfanyl)-1H-tetrazole (**75e**): ^{1}H NMR (600 MHz, DMSO-d_6)

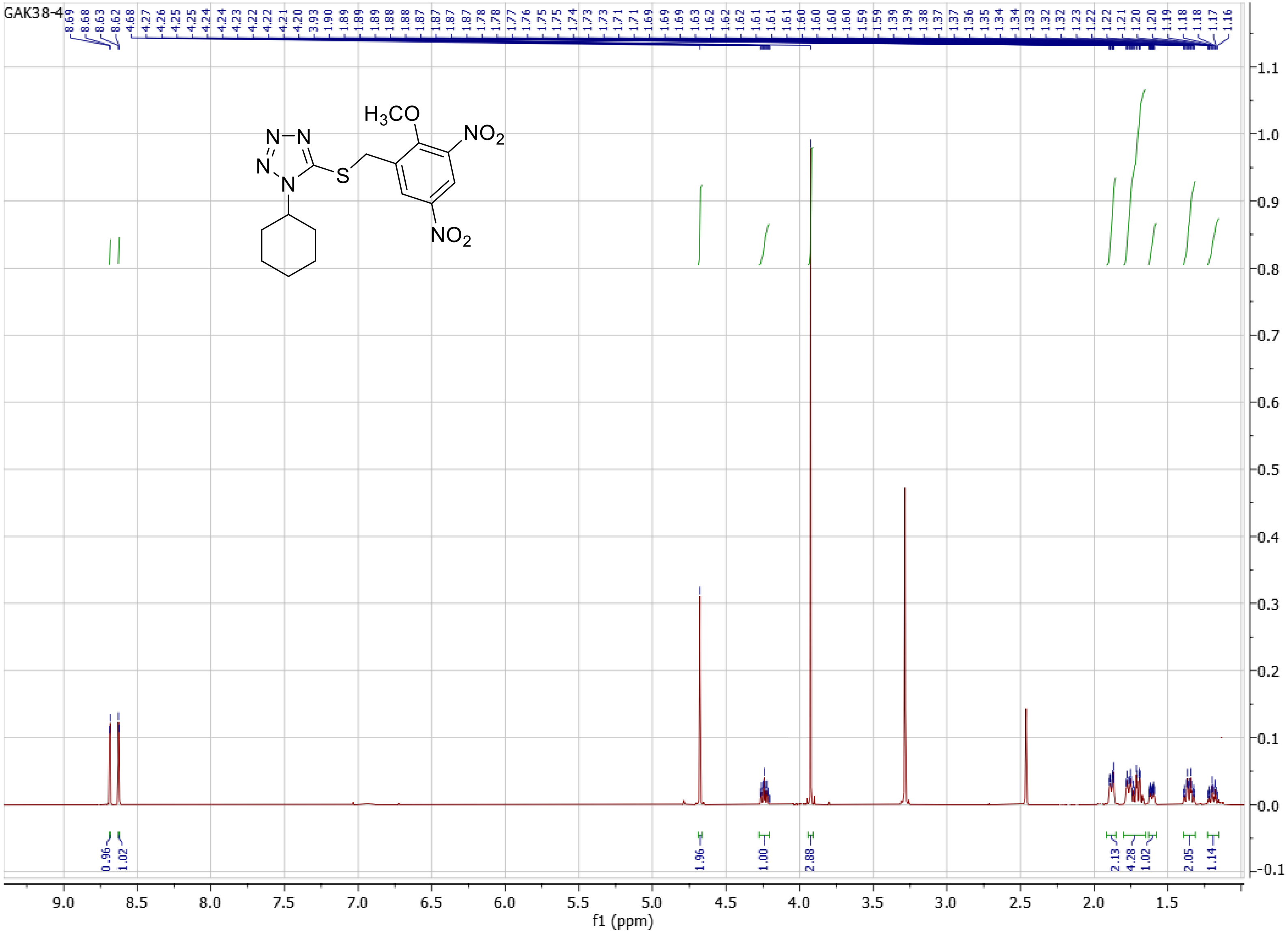

1-Cyclohexyl-5-((2-methoxy-3,5-dinitrobenzyl)sulfanyl)-1H-tetrazole (**75e**): ^{13}C NMR (151 MHz, DMSO-d_6)

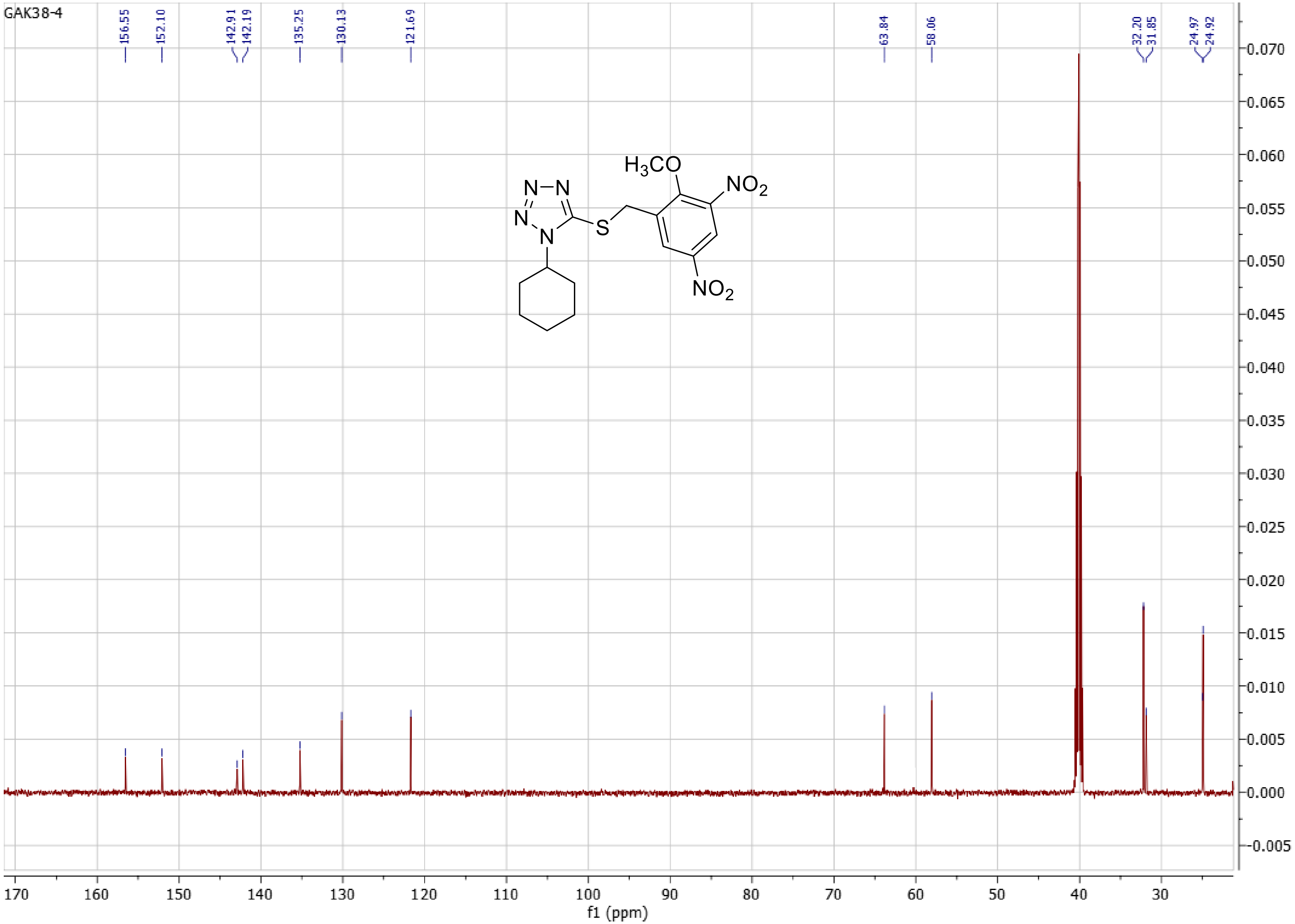

5-((4-Methyl-3,5-dinitrobenzyl)sulfanyl)-1-phenyl-1H-tetrazole (**76a**): ^{1}H NMR (500 MHz, Acetone-d_6)

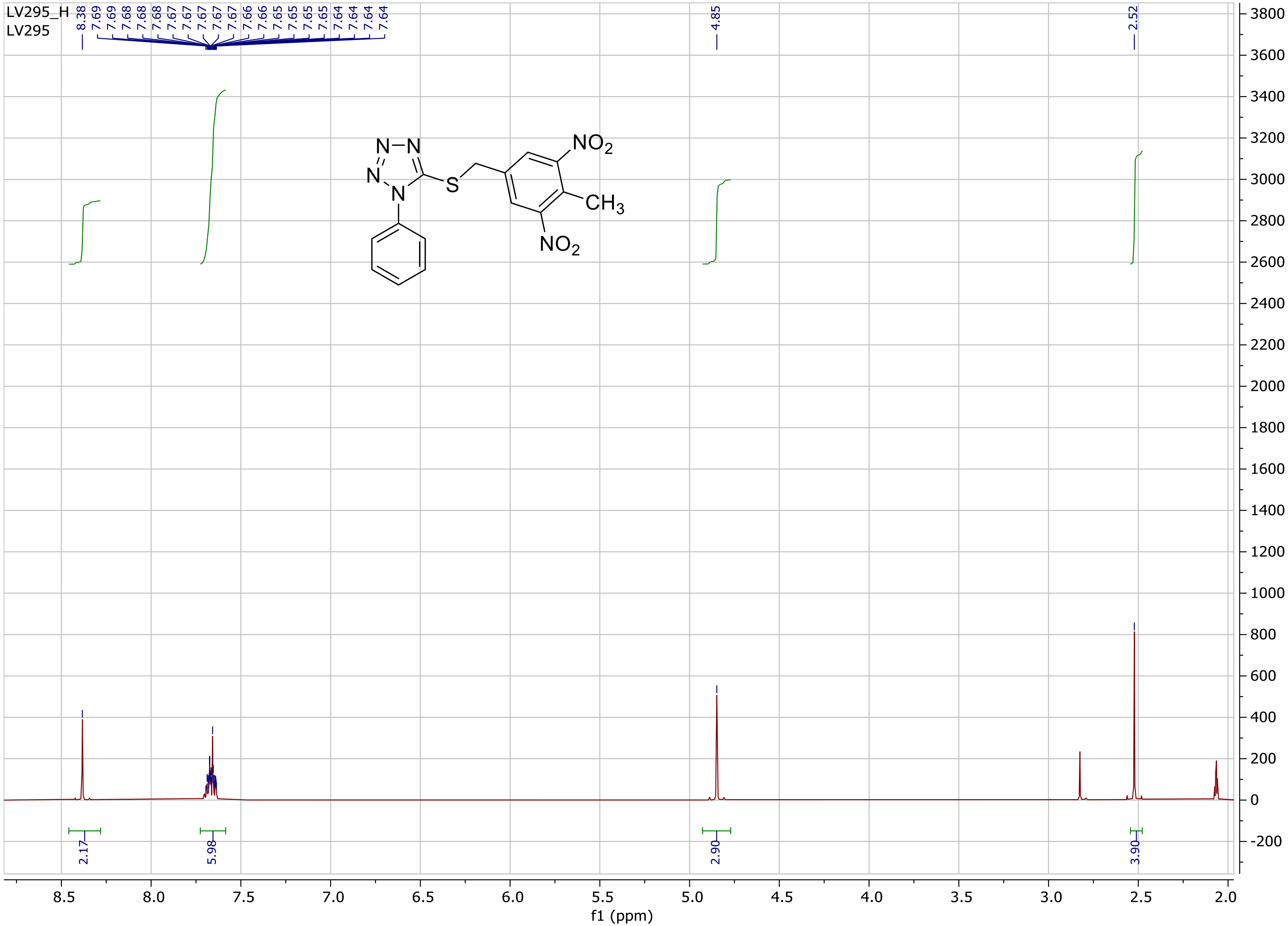

5-((4-Methyl-3,5-dinitrobenzyl)sulfanyl)-1-phenyl-1H-tetrazole (**76a**): ^{13}C NMR (126 MHz, Acetone-d_6)

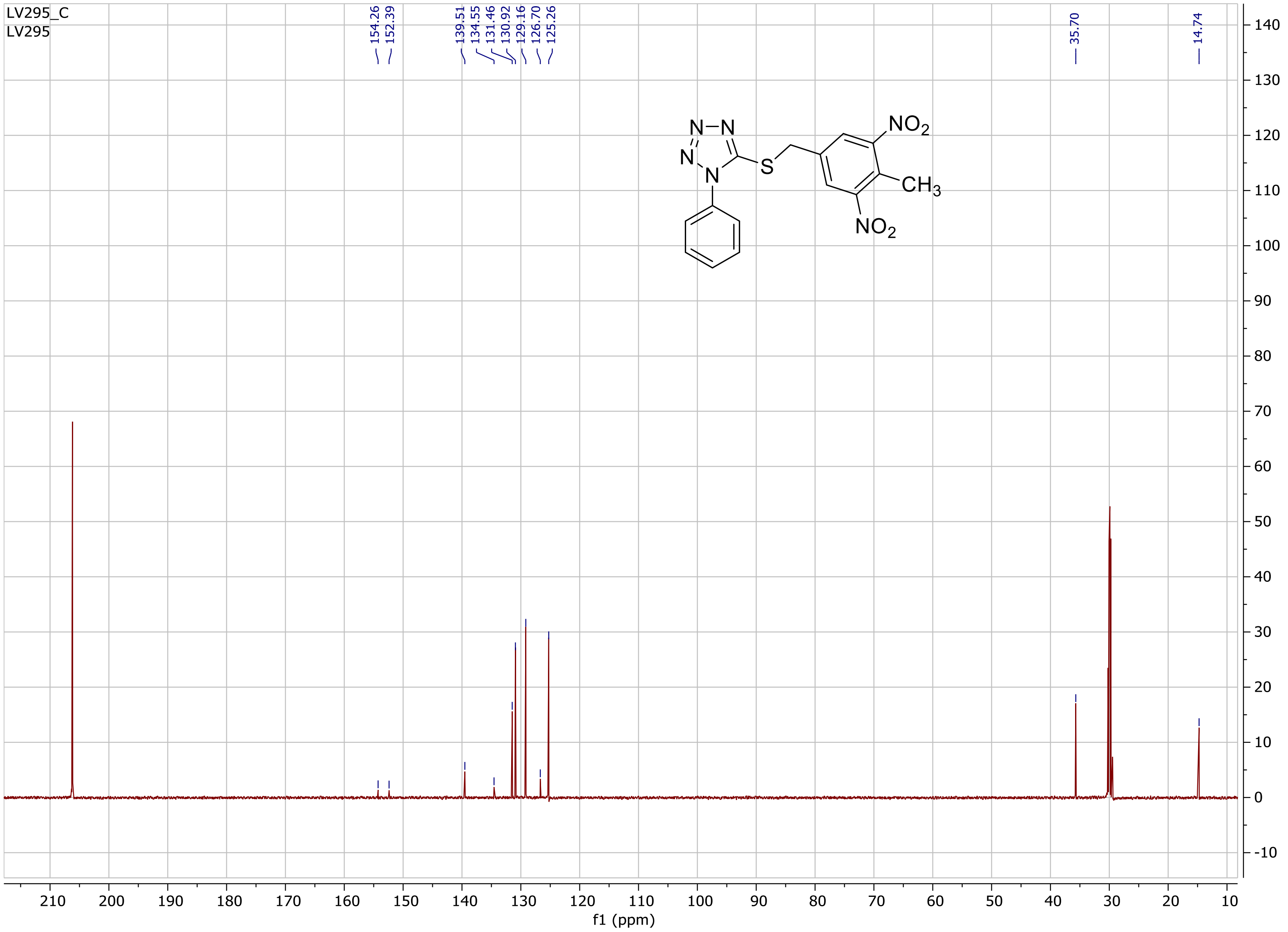

1-(4-Methoxyphenyl)-5-((4-methyl-3,5-dinitrobenzyl)sulfanyl)-1H-tetrazole (**76b**): ^{1}H NMR (600 MHz, DMSO-d_6)

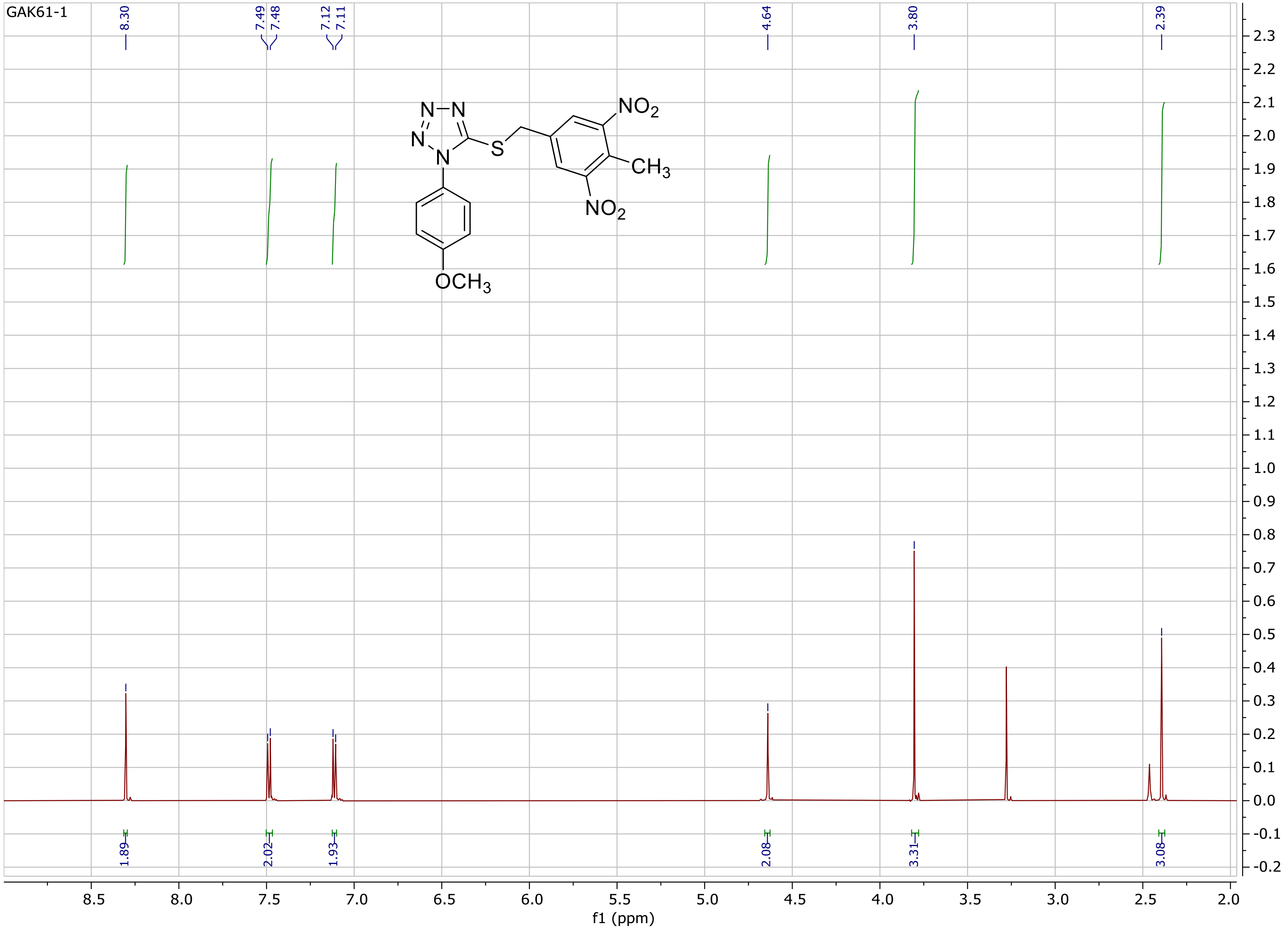

1-(4-Methoxyphenyl)-5-((4-methyl-3,5-dinitrobenzyl)sulfanyl)-1H-tetrazole (**76b**): ^{13}C NMR (151 MHz, DMSO-d_6)

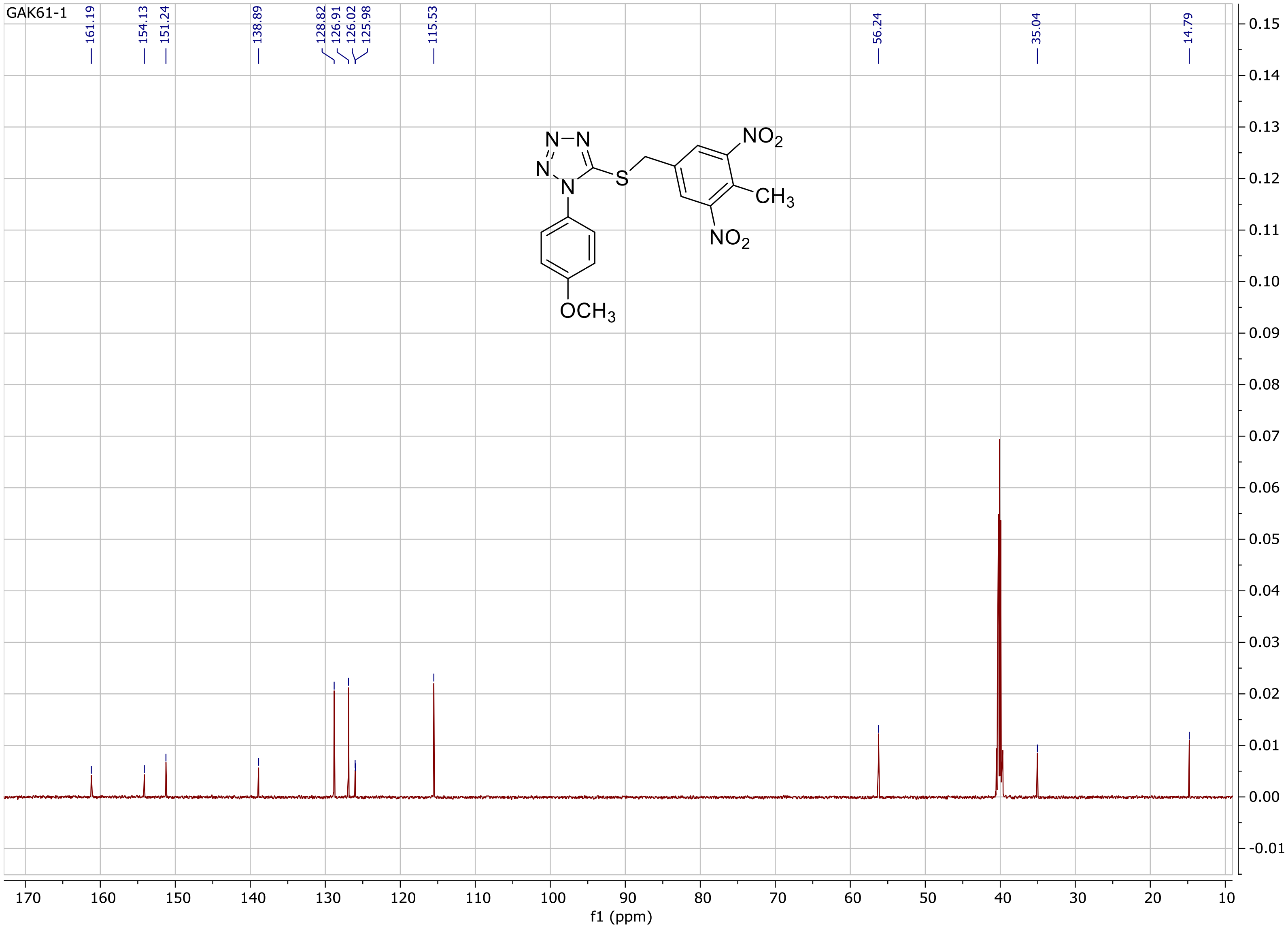

1-(4-Chlorophenyl)-5-((4-methyl-3,5-dinitrobenzyl)sulfanyl(-1H-tetrazole (**76c**): ^{1}H NMR (600 MHz, DMSO-d_6)

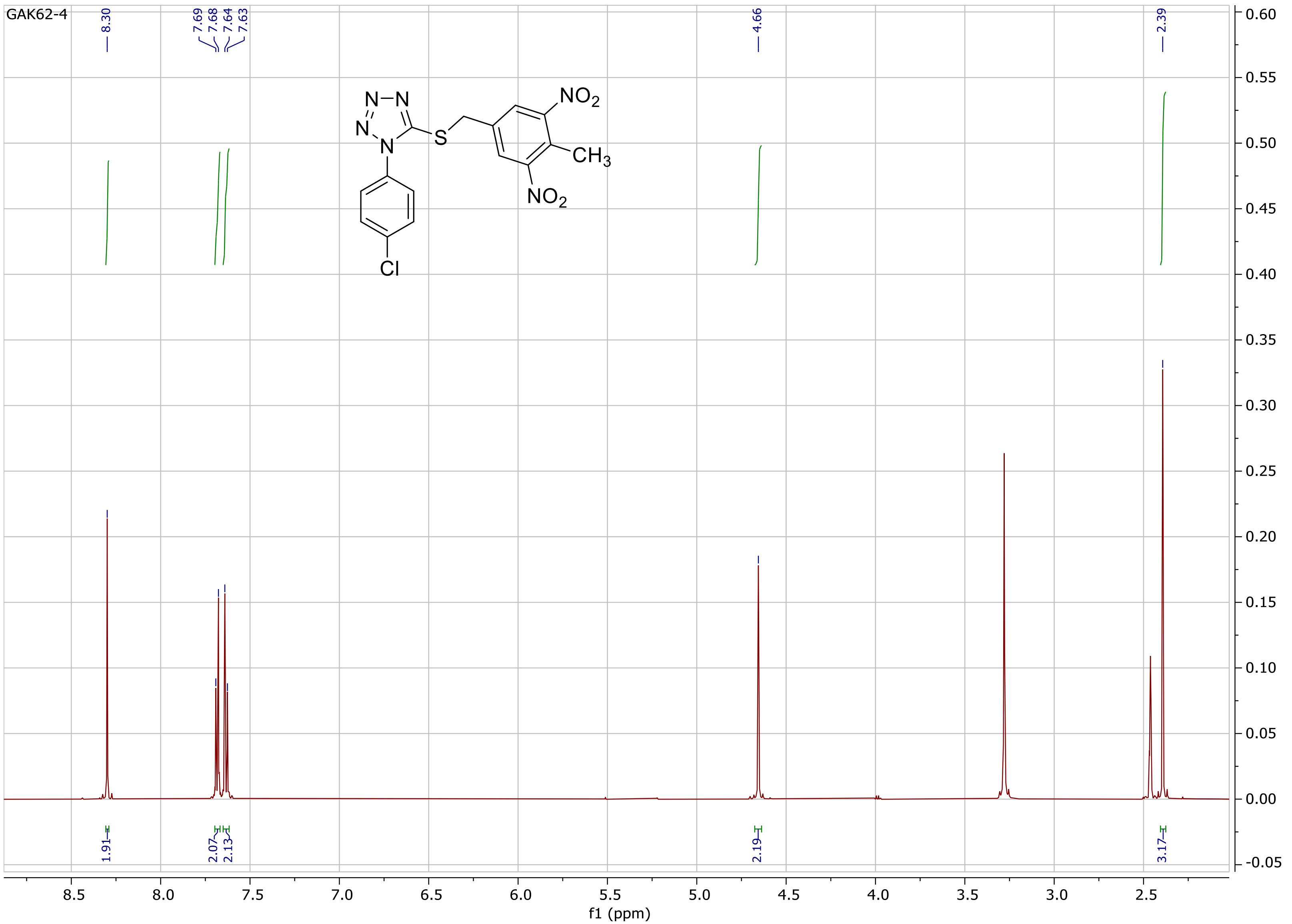

1-(4-Chlorophenyl)-5-((4-methyl-3,5-dinitrobenzyl)sulfanyl(-1H-tetrazole (**76c**): ^{13}C NMR (151 MHz, DMSO-d_6)

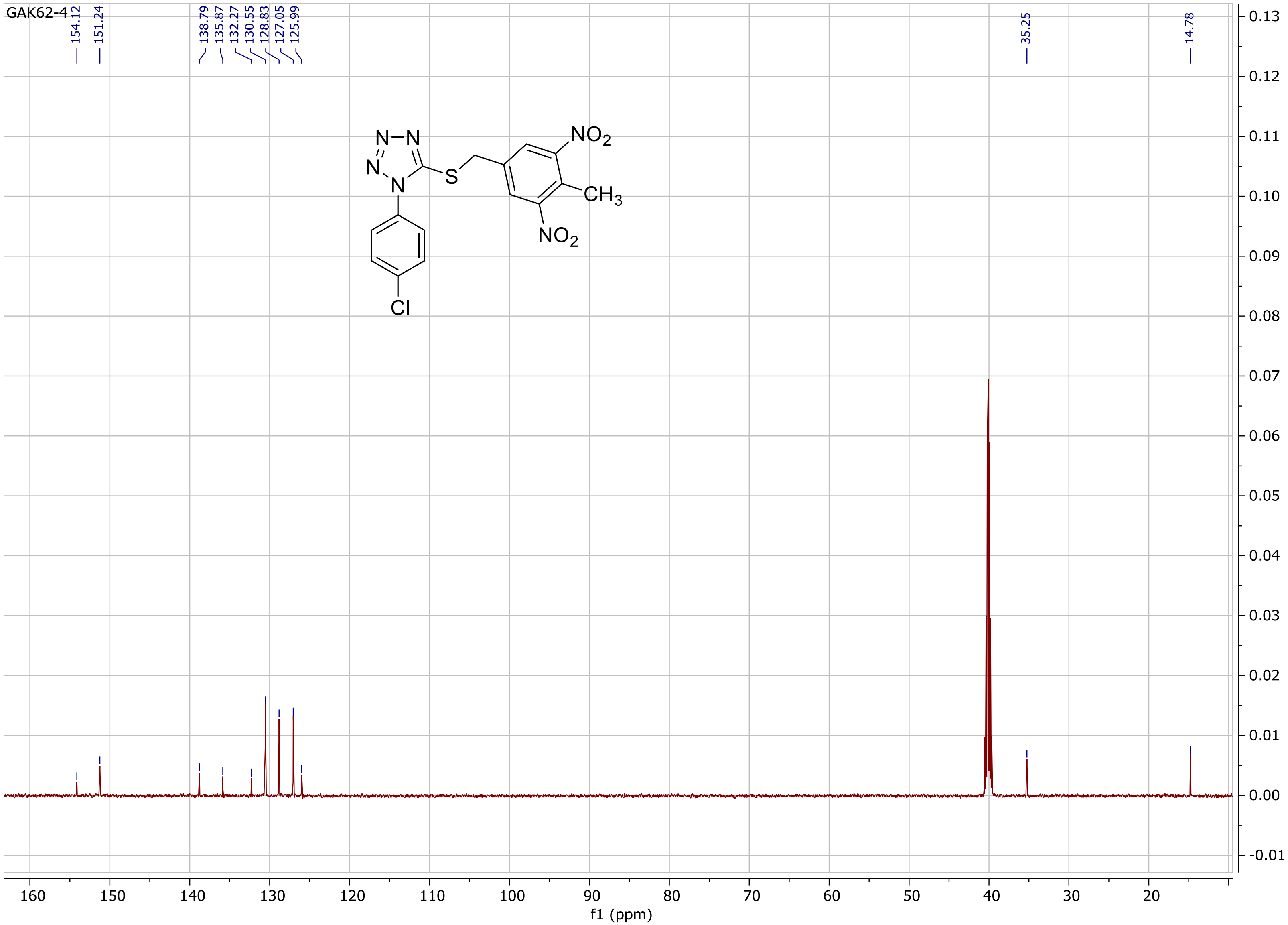

1-(4-Bromophenyl)-5-((4-methyl-3,5-dinitrobenzyl)sulfanyl)-1H-tetrazole (**76d**): ^{1}H NMR (500 MHz, Acetone-d_6)

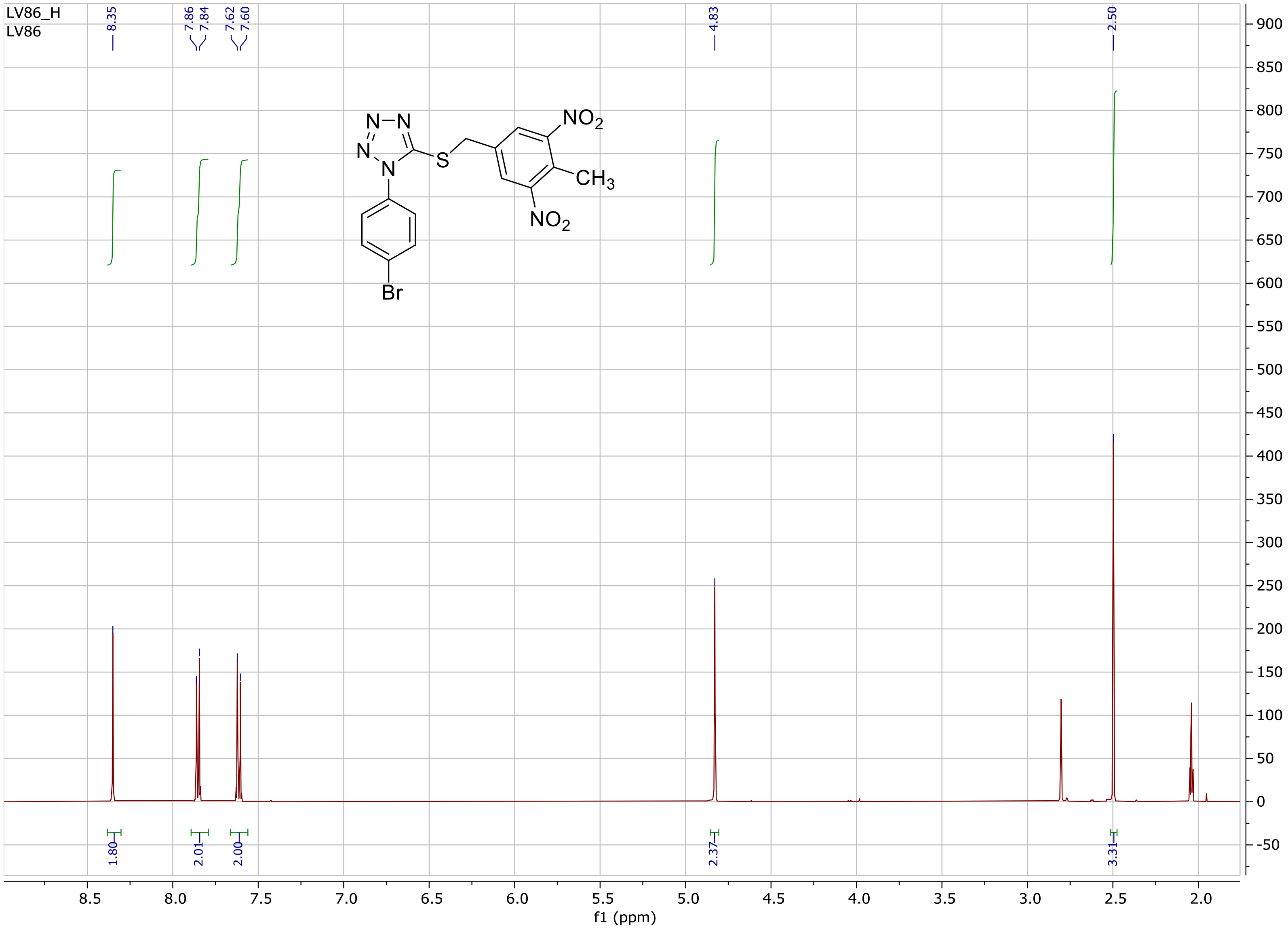

1-(4-Bromophenyl)-5-((4-methyl-3,5-dinitrobenzyl)sulfanyl)-1H-tetrazole (**76d**): ^{13}C NMR (126 MHz, Acetone-d_6)

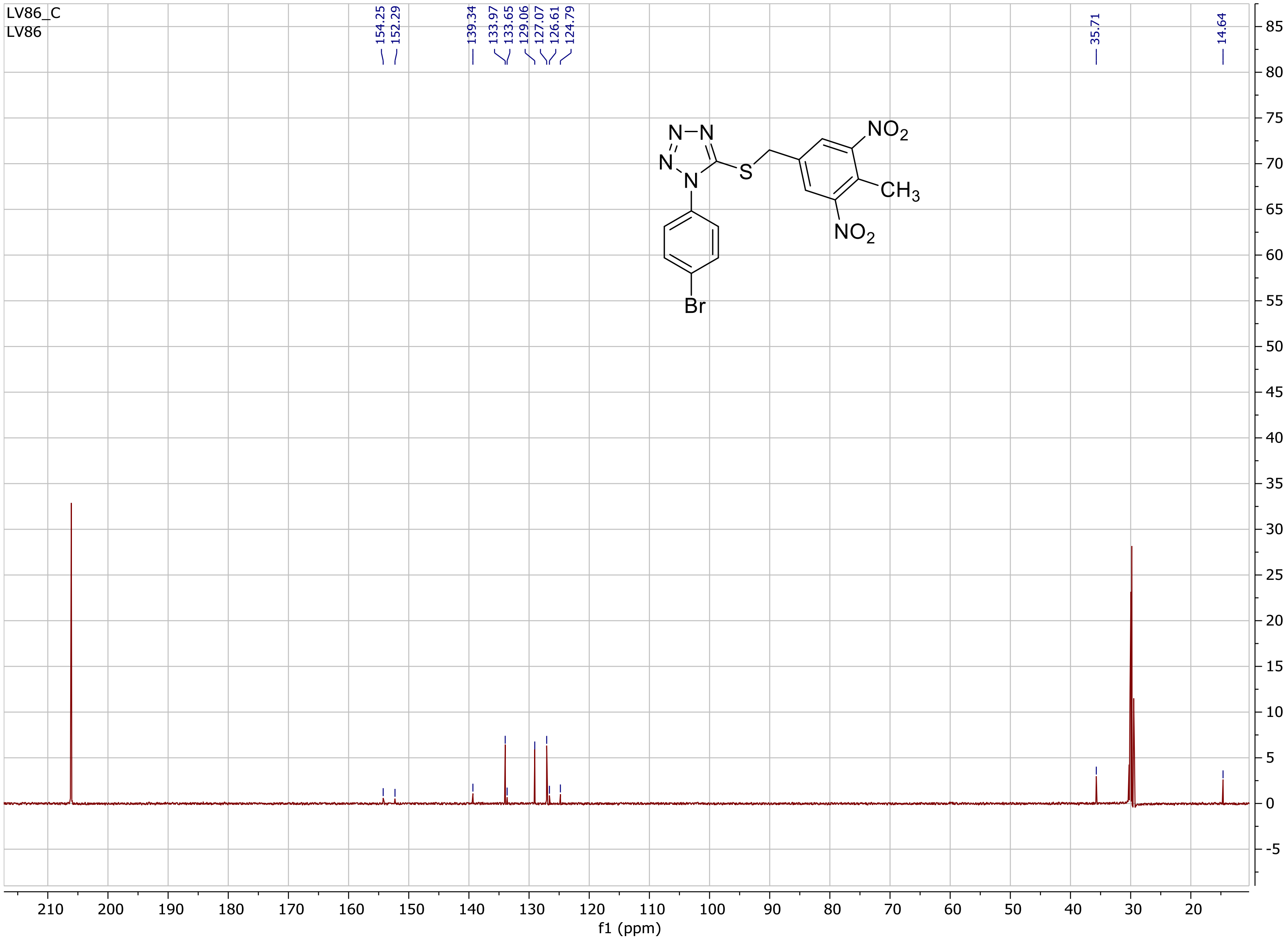

1-Cyclohexyl-5-((4-methyl-3,5-dinitrobenzyl)sulfanyl)-1H-tetrazole (**76e**): ^{1}H NMR (600 MHz, DMSO-d_6)

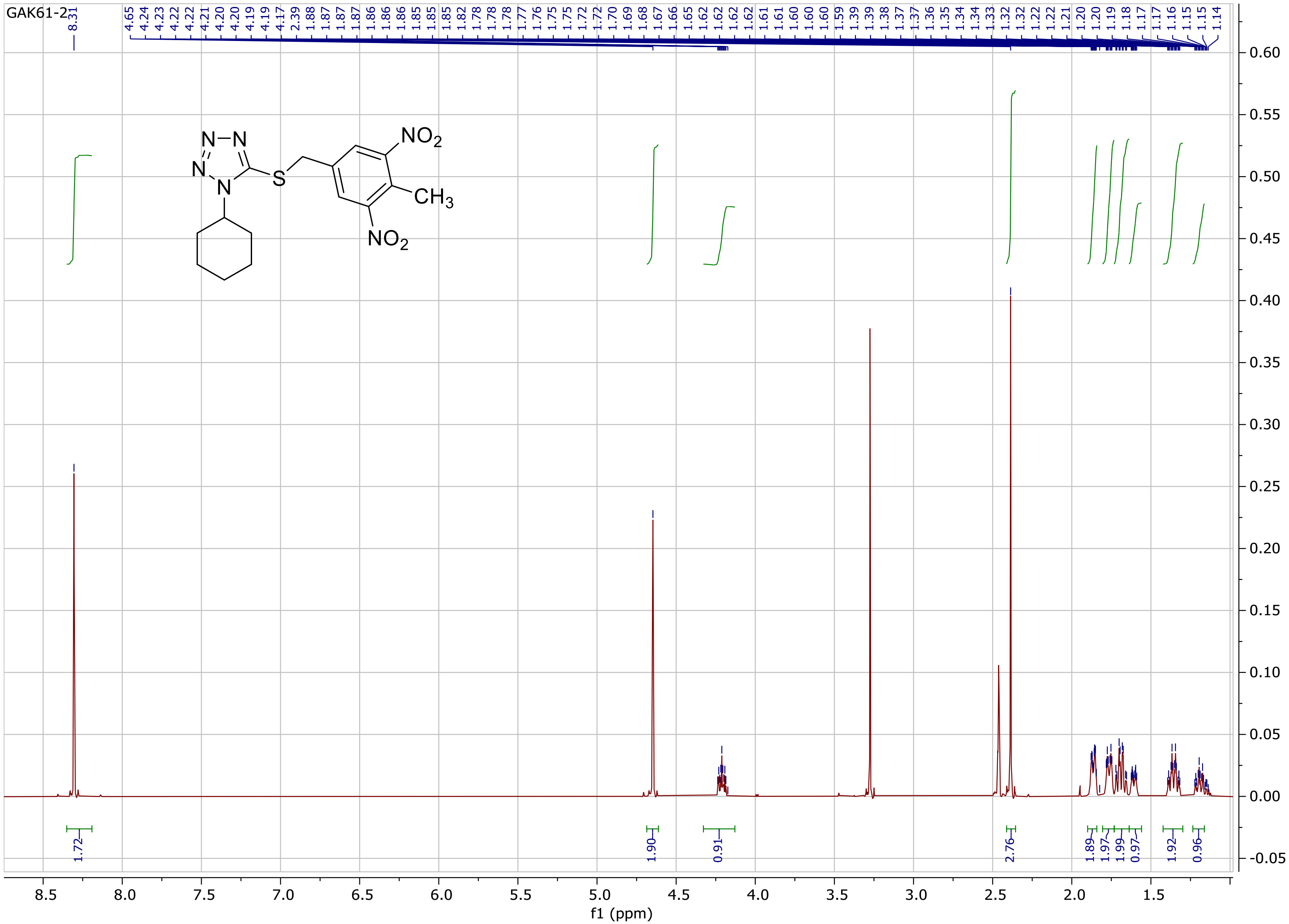

1-Cyclohexyl-5-((4-methyl-3,5-dinitrobenzyl)sulfanyl)-1H-tetrazole (**76e**): ^{13}C NMR (151 MHz, DMSO-d_6)

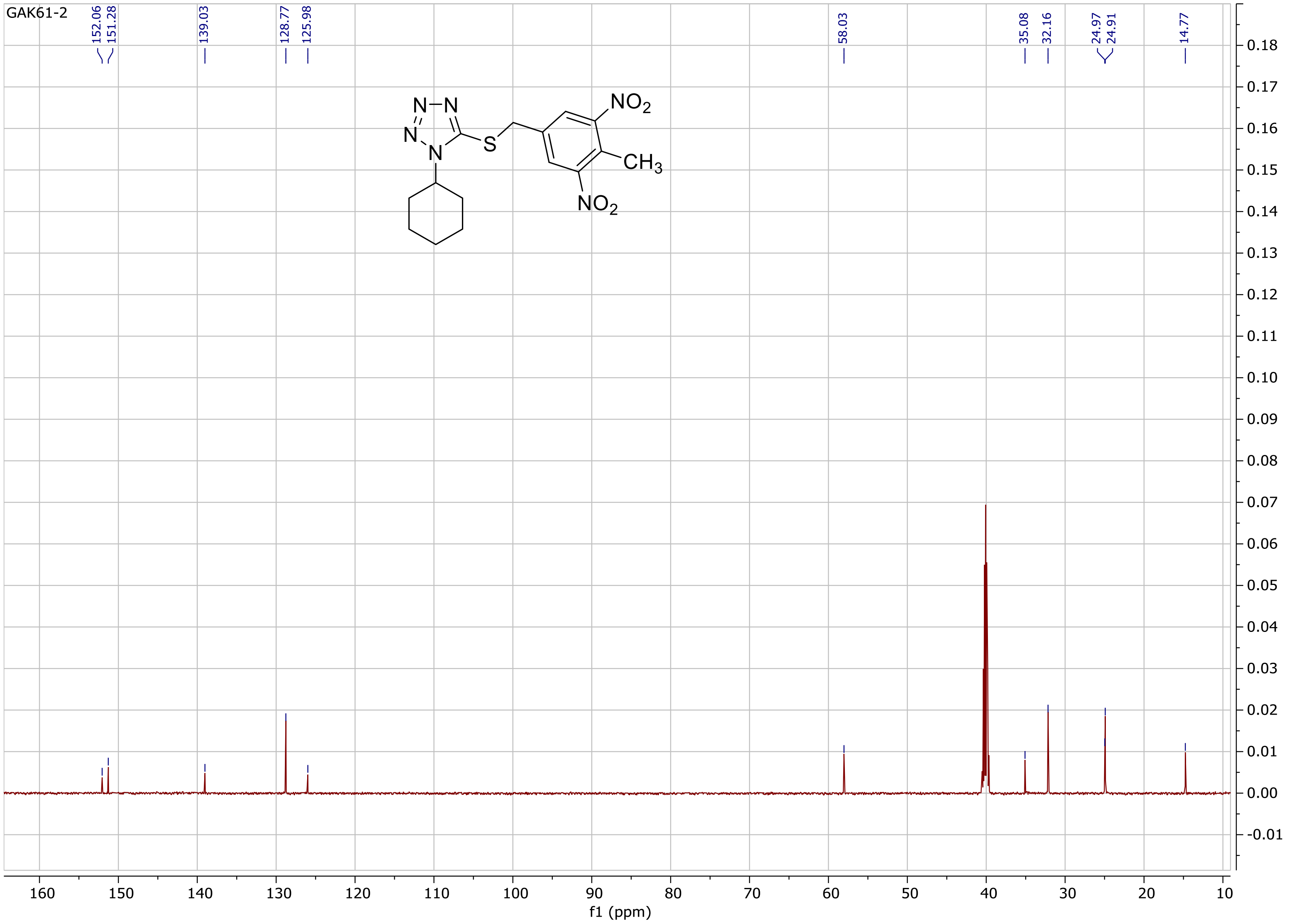

5-((2-Methyl-3,5-dinitrobenzyl)sulfanyl)-1-phenyl-1H-tetrazole (**77a**): ^{1}H NMR (600 MHz, DMSO-d_6)

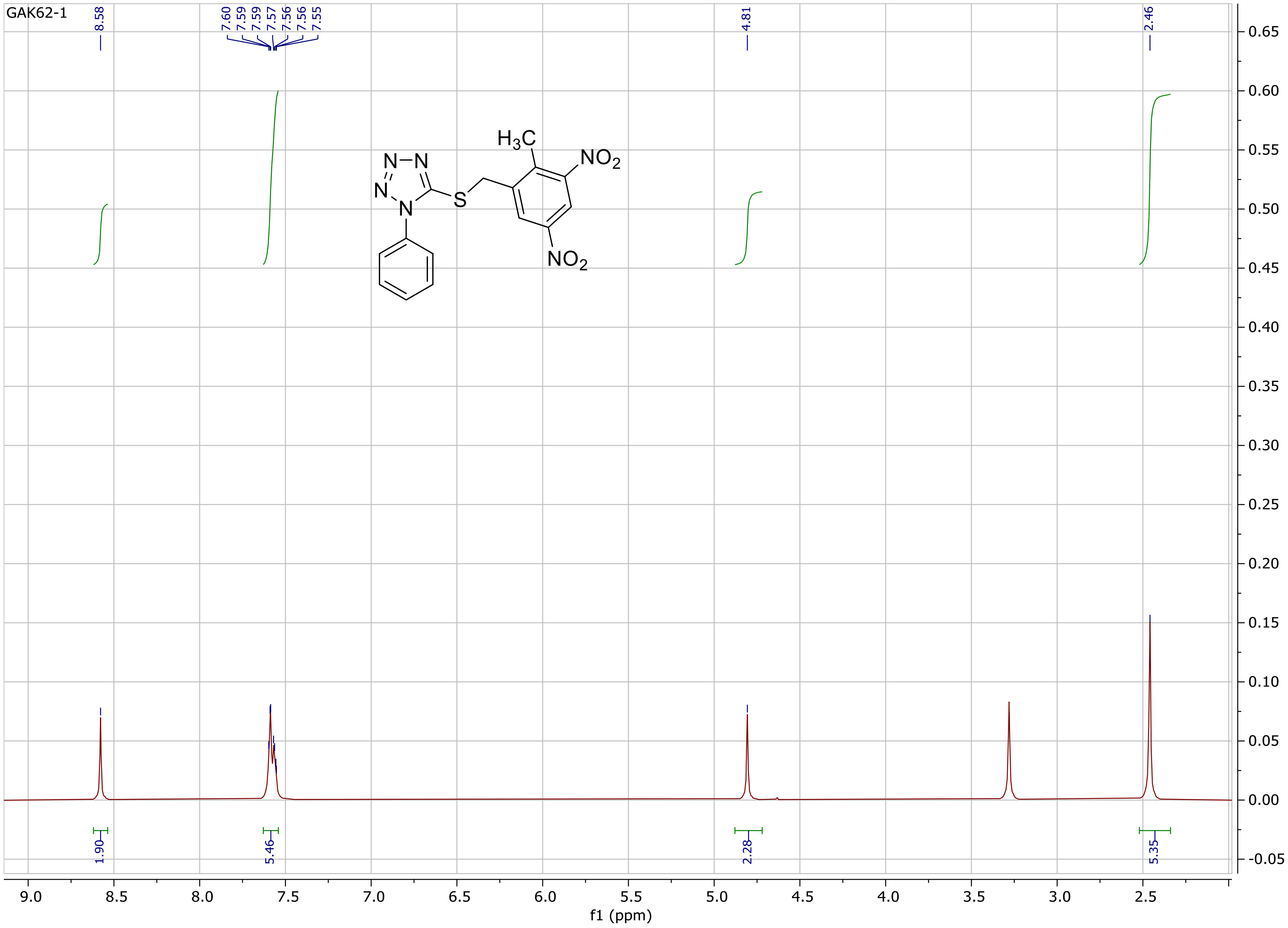

5-((2-Methyl-3,5-dinitrobenzyl)sulfanyl)-1-phenyl-1H-tetrazole (**77a**): ^{13}C NMR (151 MHz, DMSO-d_6)

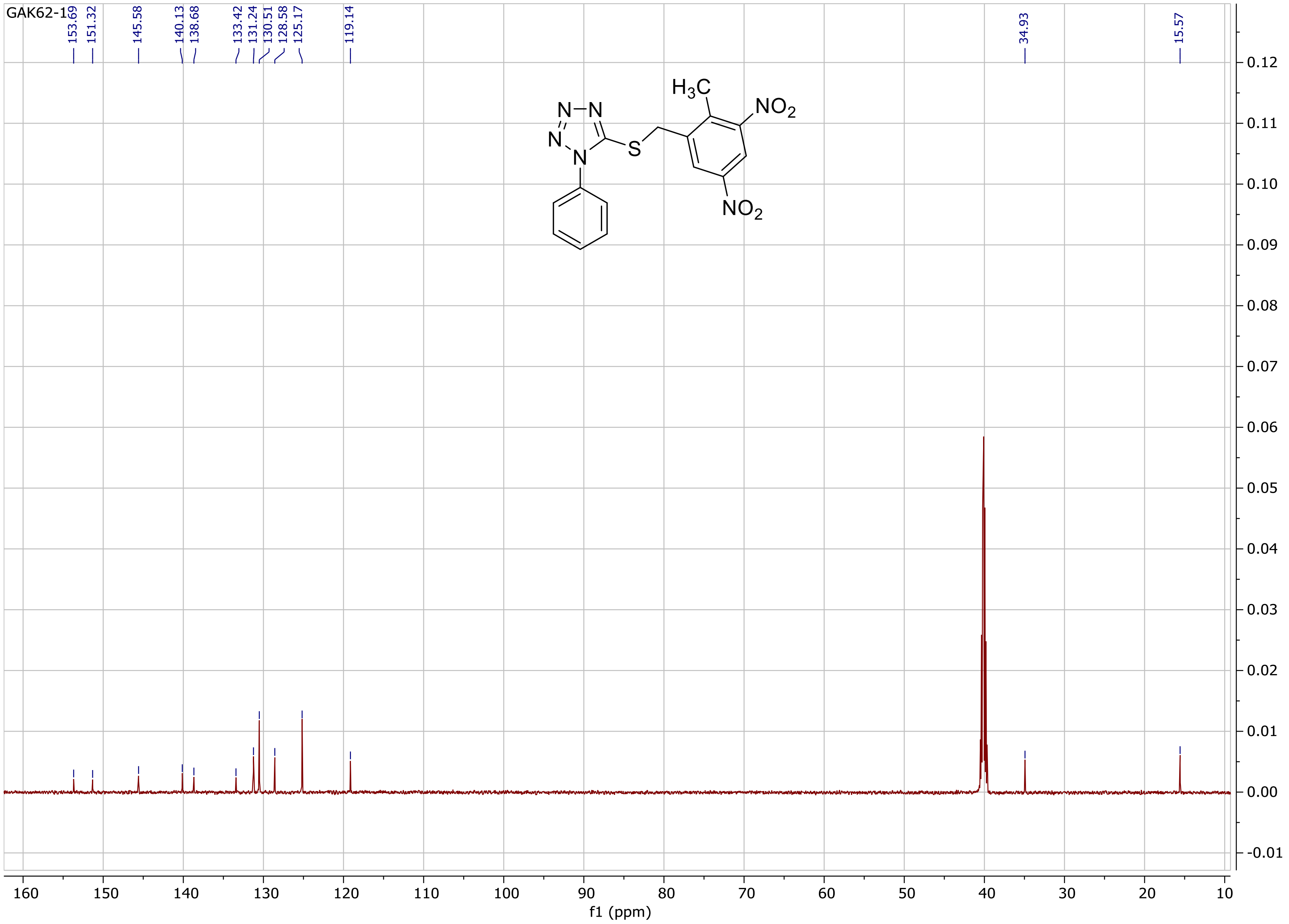

1-(4-Methoxyphenyl)-5-((2-methyl-3,5-dinitrobenzyl)sulfanyl)-1H-tetrazole (**77b**): ^{1}H NMR (600 MHz, DMSO-d_6)

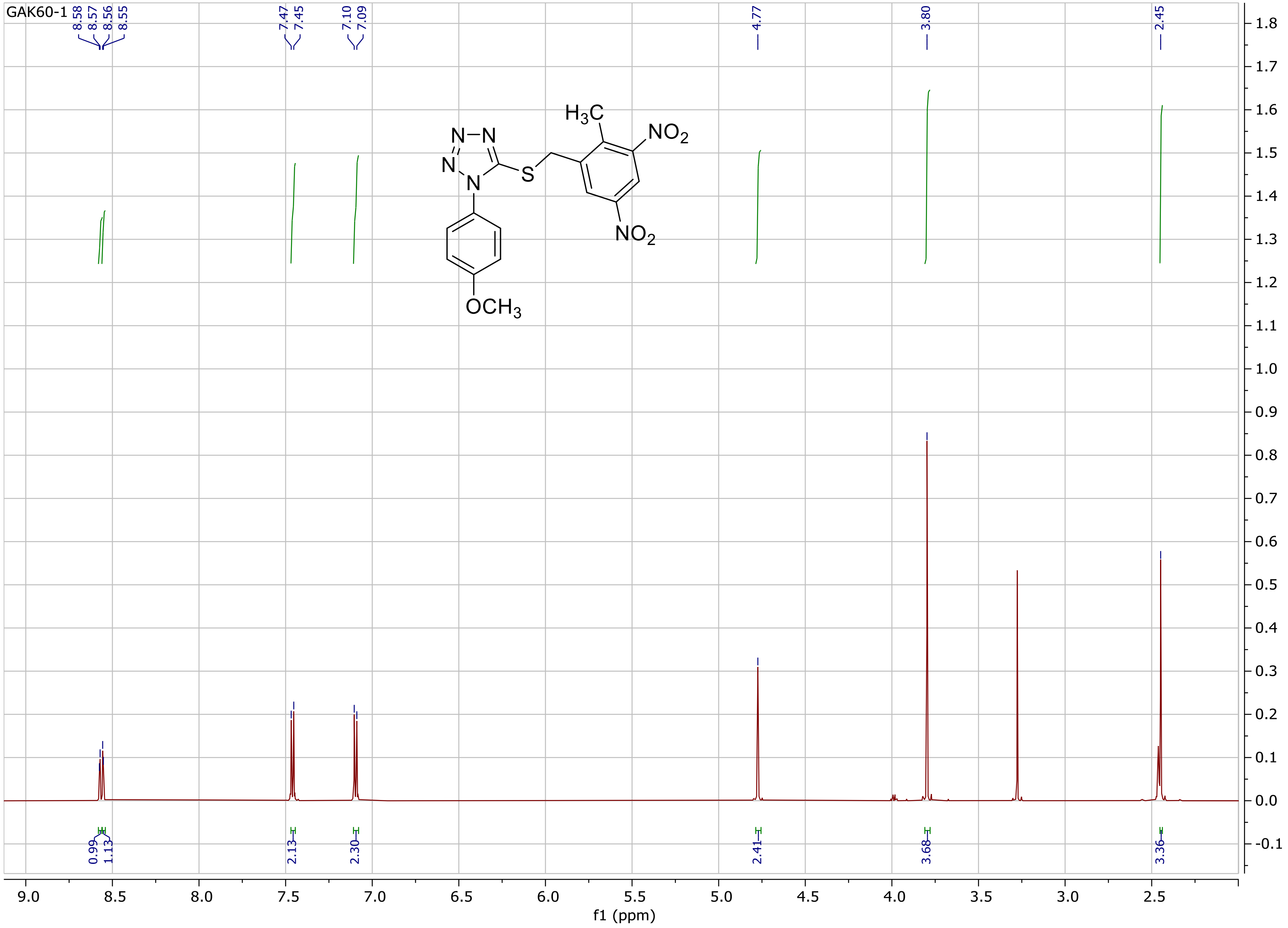

1-(4-Methoxyphenyl)-5-((2-methyl-3,5-dinitrobenzyl)sulfanyl)-1H-tetrazole (**77b**): ^{13}C NMR (151 MHz, DMSO-d_6)

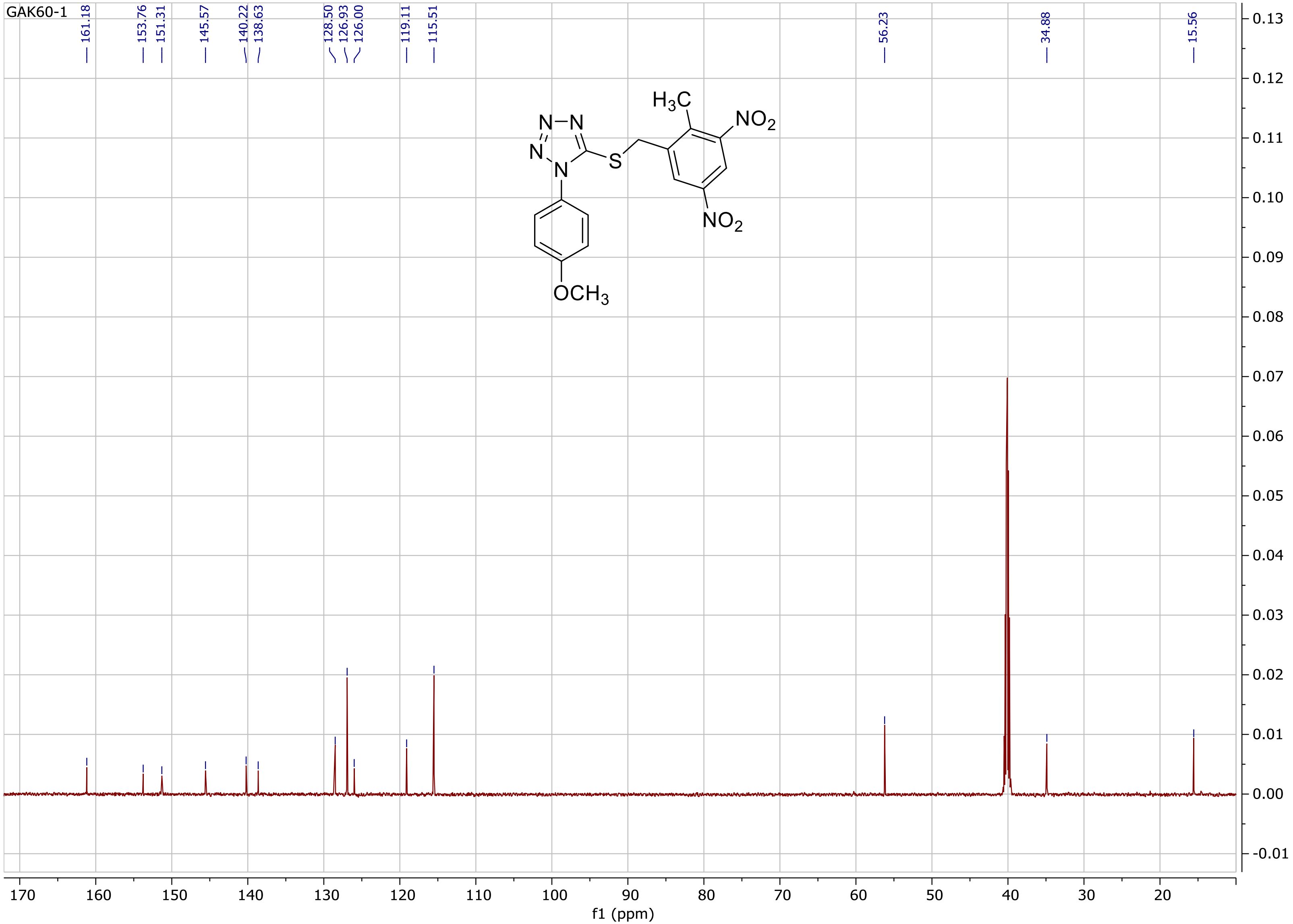

1-(4-Chlorophenyl)-5-((2-methyl-3,5-dinitrobenzyl)sulfanyl)-1H-tetrazole (**77c**): ^{1}H NMR (500 MHz, DMSO-d_6)

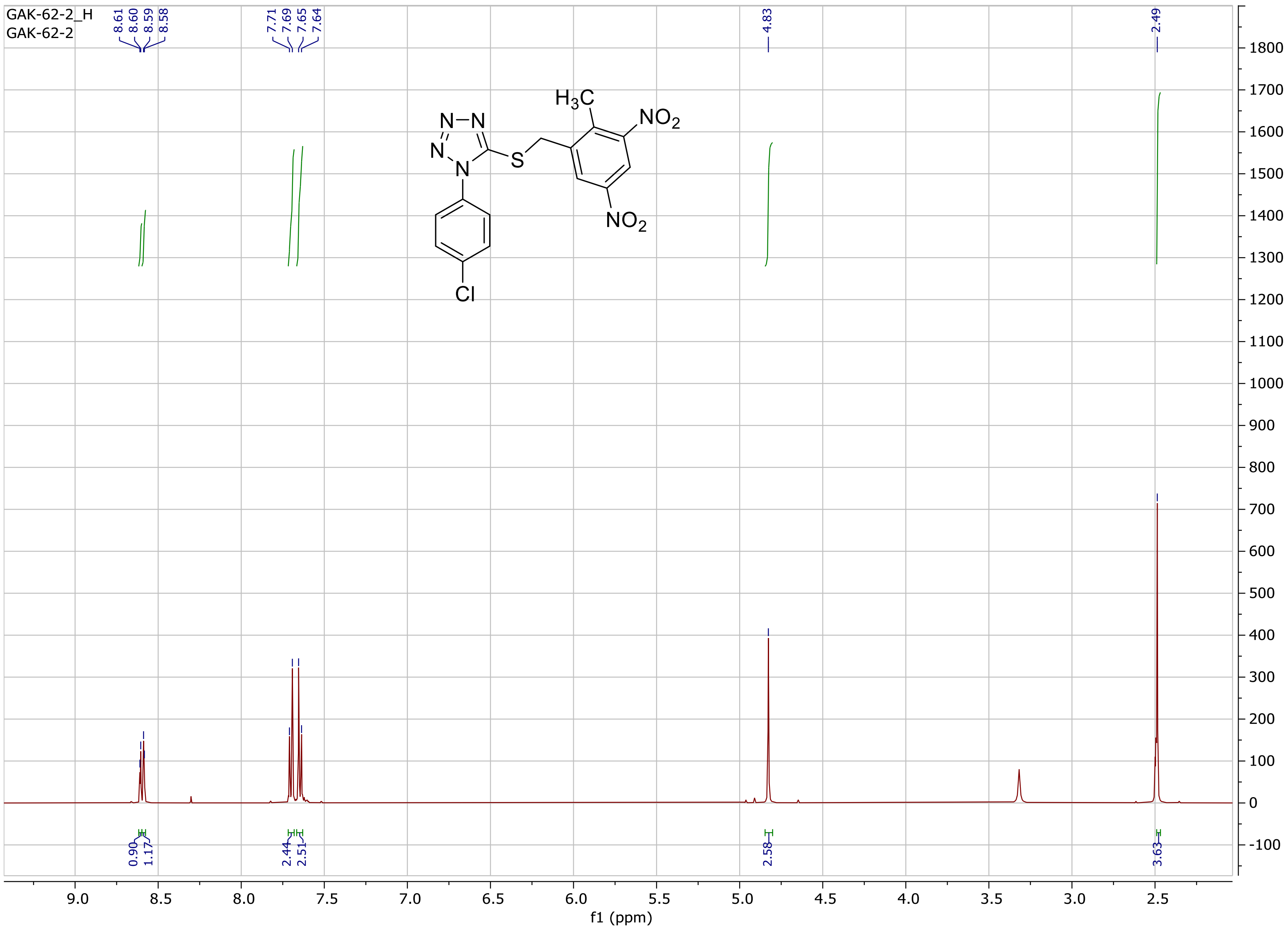

1-(4-Chlorophenyl)-5-((2-methyl-3,5-dinitrobenzyl)sulfanyl)-1H-tetrazole (**77c**): ^{13}C NMR (126 MHz, DMSO-d_6)

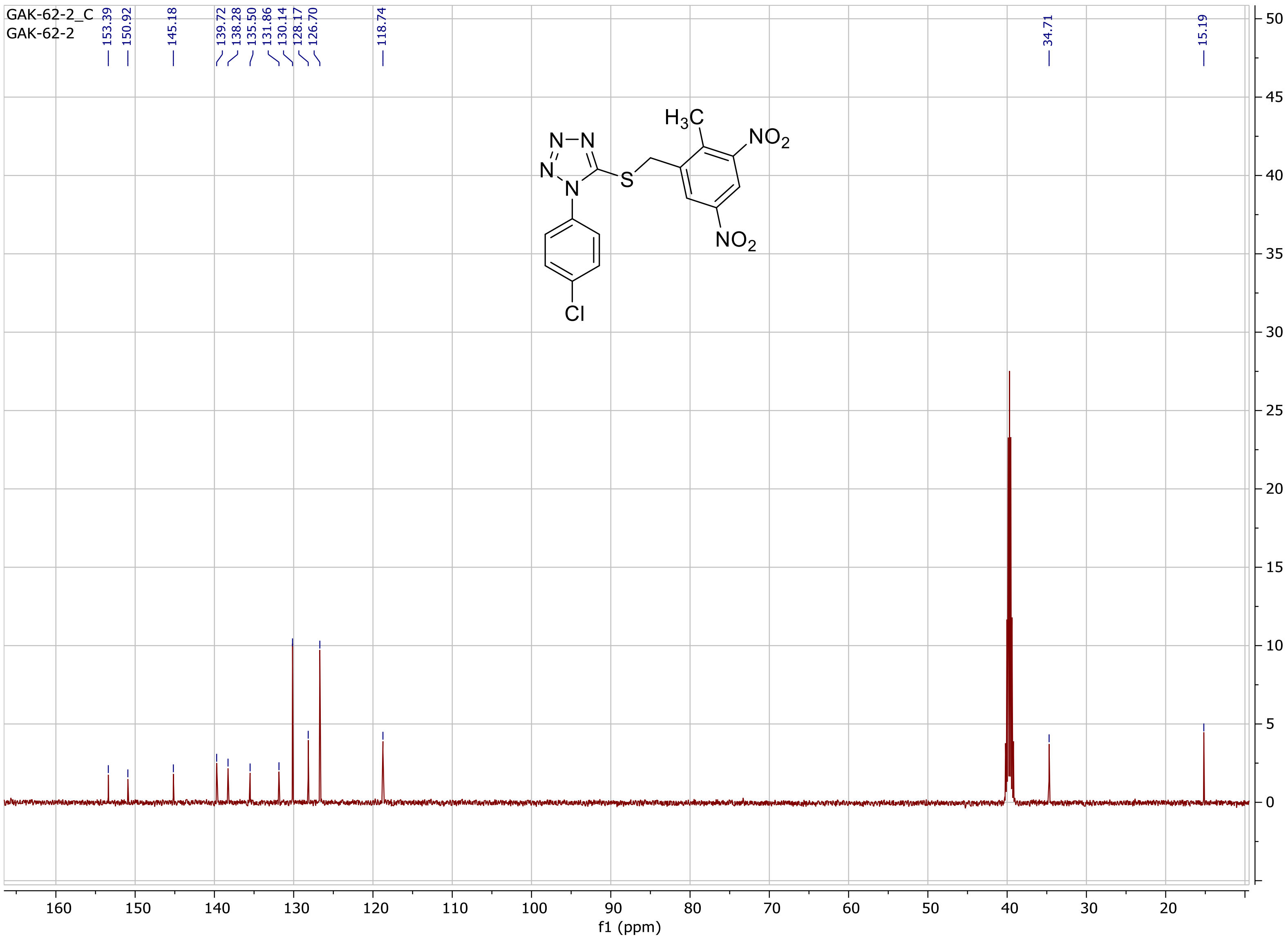

1-(4-Bromophenyl)-5-((2-methyl-3,5-dinitrobenzyl)sulfanyl)-1H-tetrazole (**77d**): ^{1}H NMR (600 MHz, DMSO-d_6)

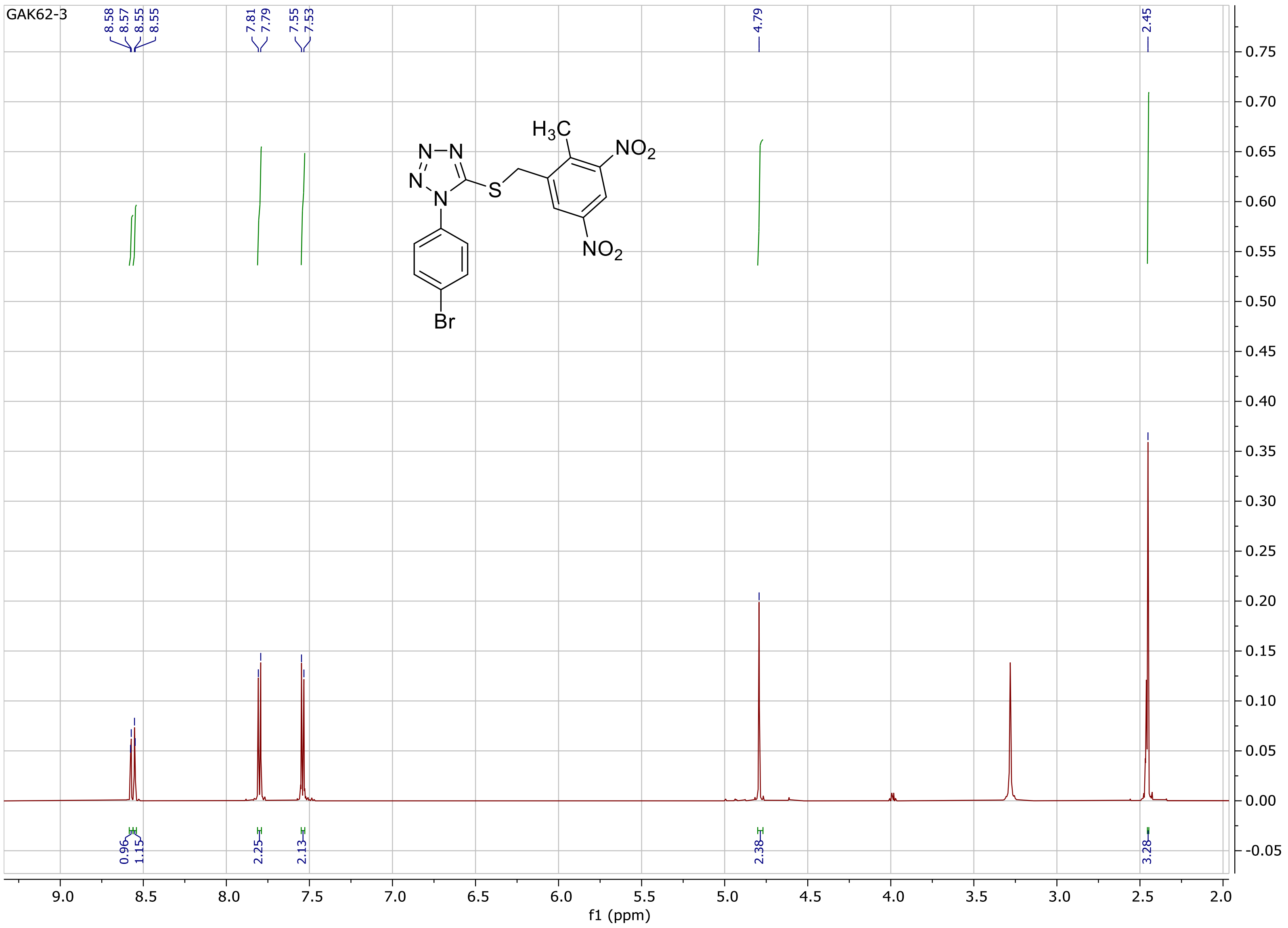

1-(4-Bromophenyl)-5-((2-methyl-3,5-dinitrobenzyl)sulfanyl)-1H-tetrazole (**77d**): ^{13}C NMR (151 MHz, DMSO-d_6)

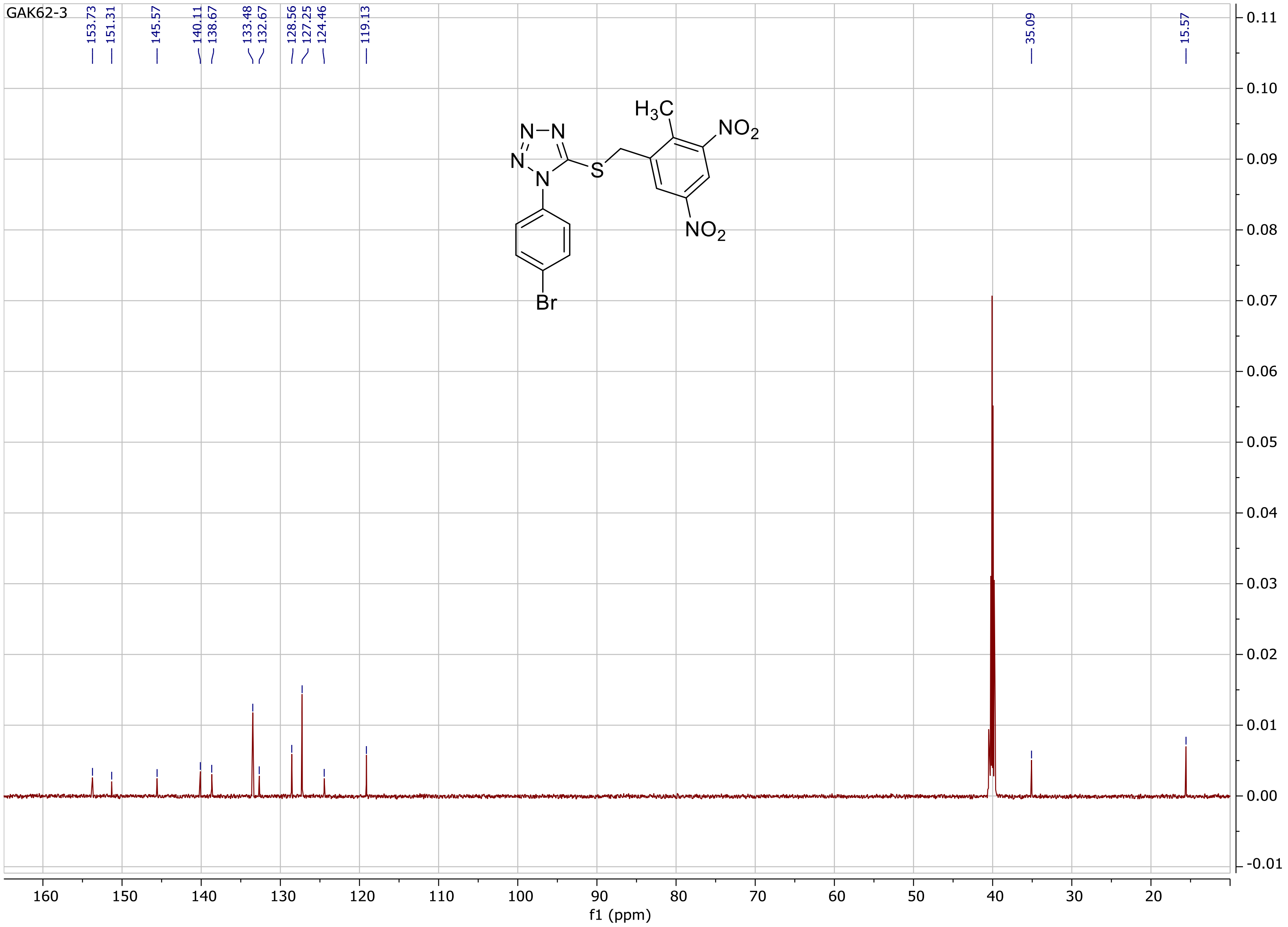

1-Cyclohexyl-5-((2-methyl-3,5-dinitrobenzyl)sulfanyl)-1H-tetrazole (**77e**): ^{1}H NMR (600 MHz, DMSO-d_6)

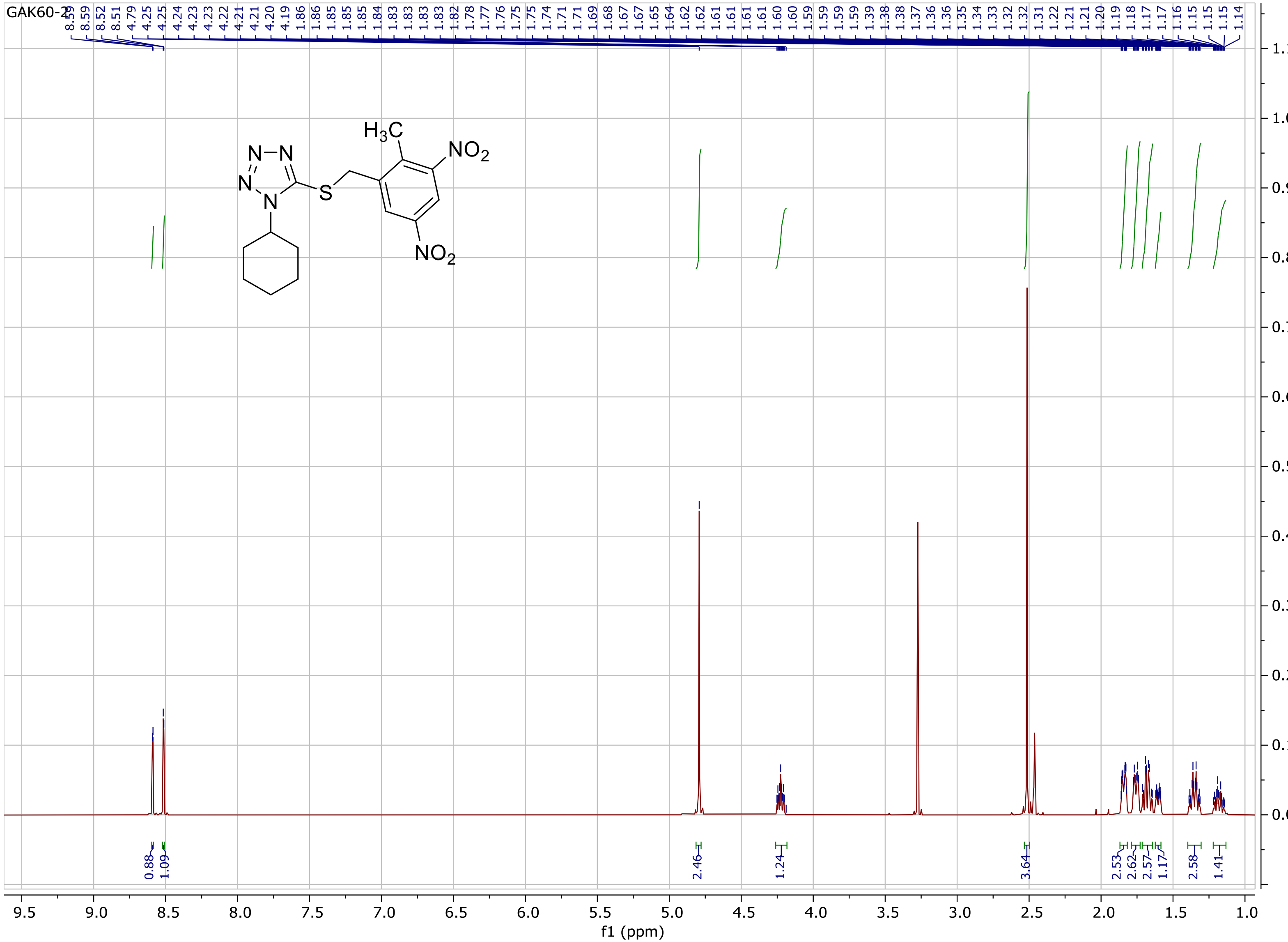

1-Cyclohexyl-5-((2-methyl-3,5-dinitrobenzyl)sulfanyl)-1H-tetrazole (**77e**): ^{13}C NMR (151 MHz, DMSO-d_6)

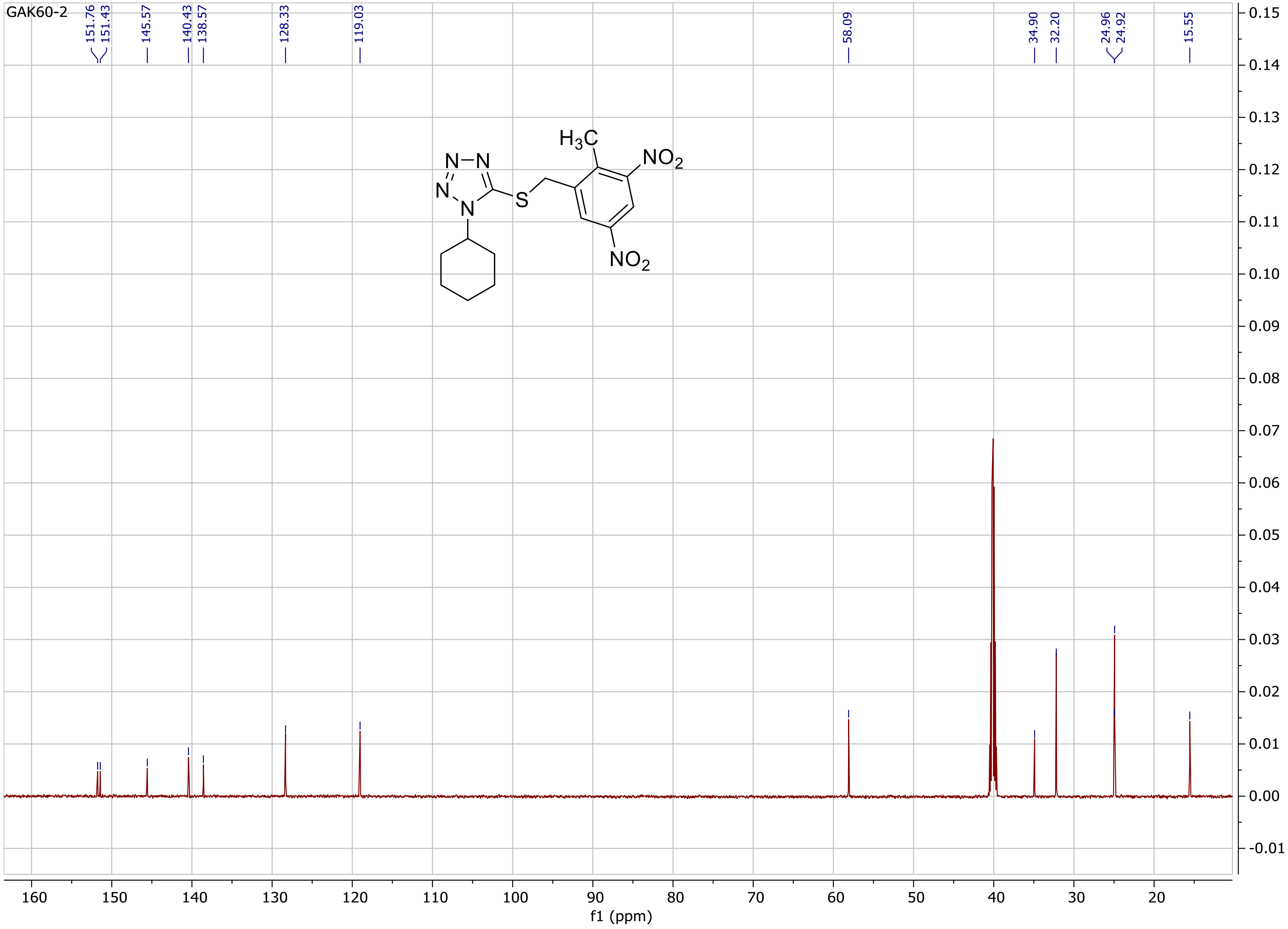

2-((4-Methoxy-3,5-dinitrobenzyl)sulfanyl)-5-phenyl-1,3,4-oxadiazole (**78a**): ^{1}H NMR (500 MHz, Acetone-d_6)

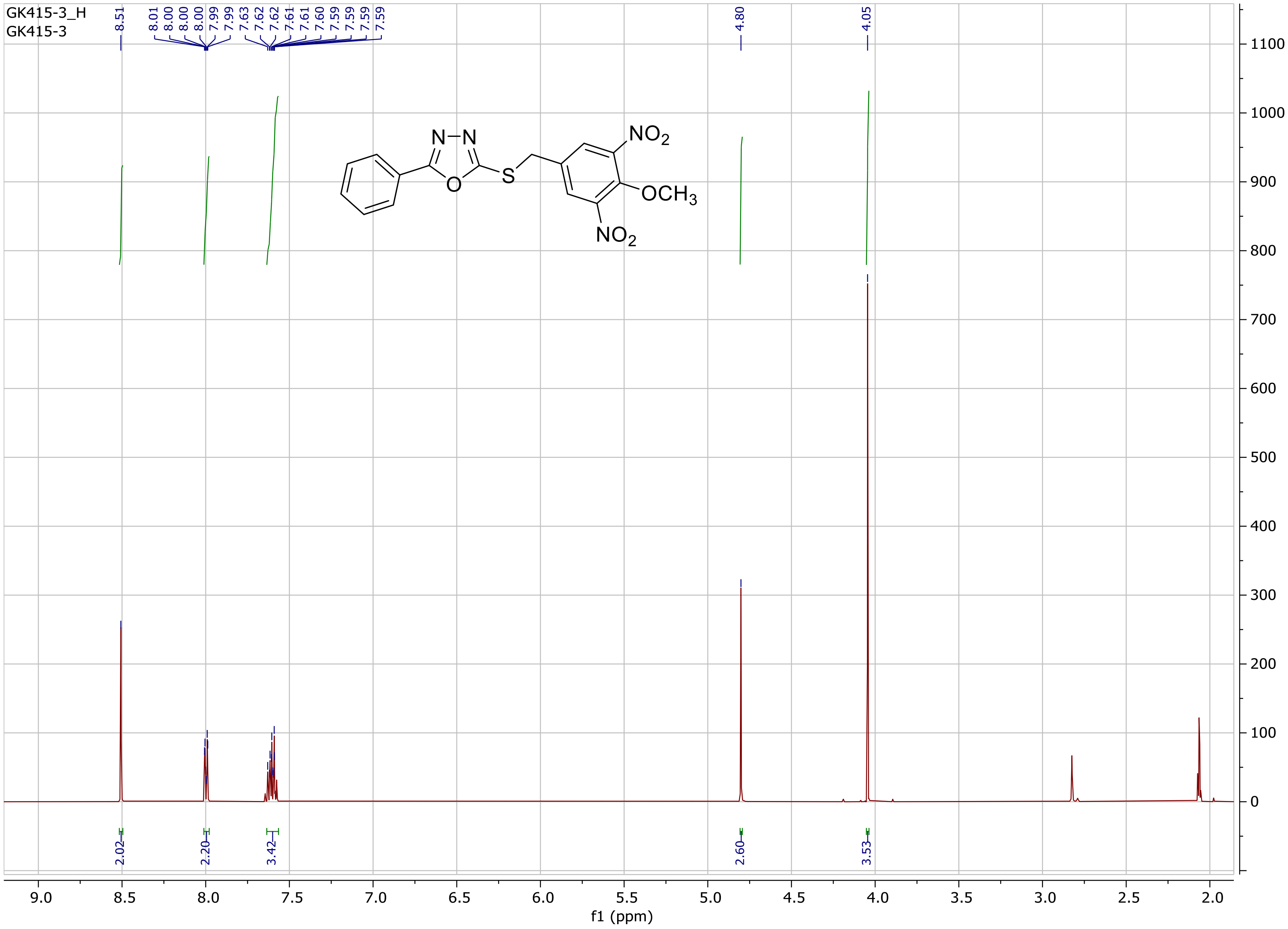

2-((4-Methoxy-3,5-dinitrobenzyl)sulfanyl)-5-phenyl-1,3,4-oxadiazole (**78a**): ^{13}C NMR (126 MHz, Acetone-d_6)

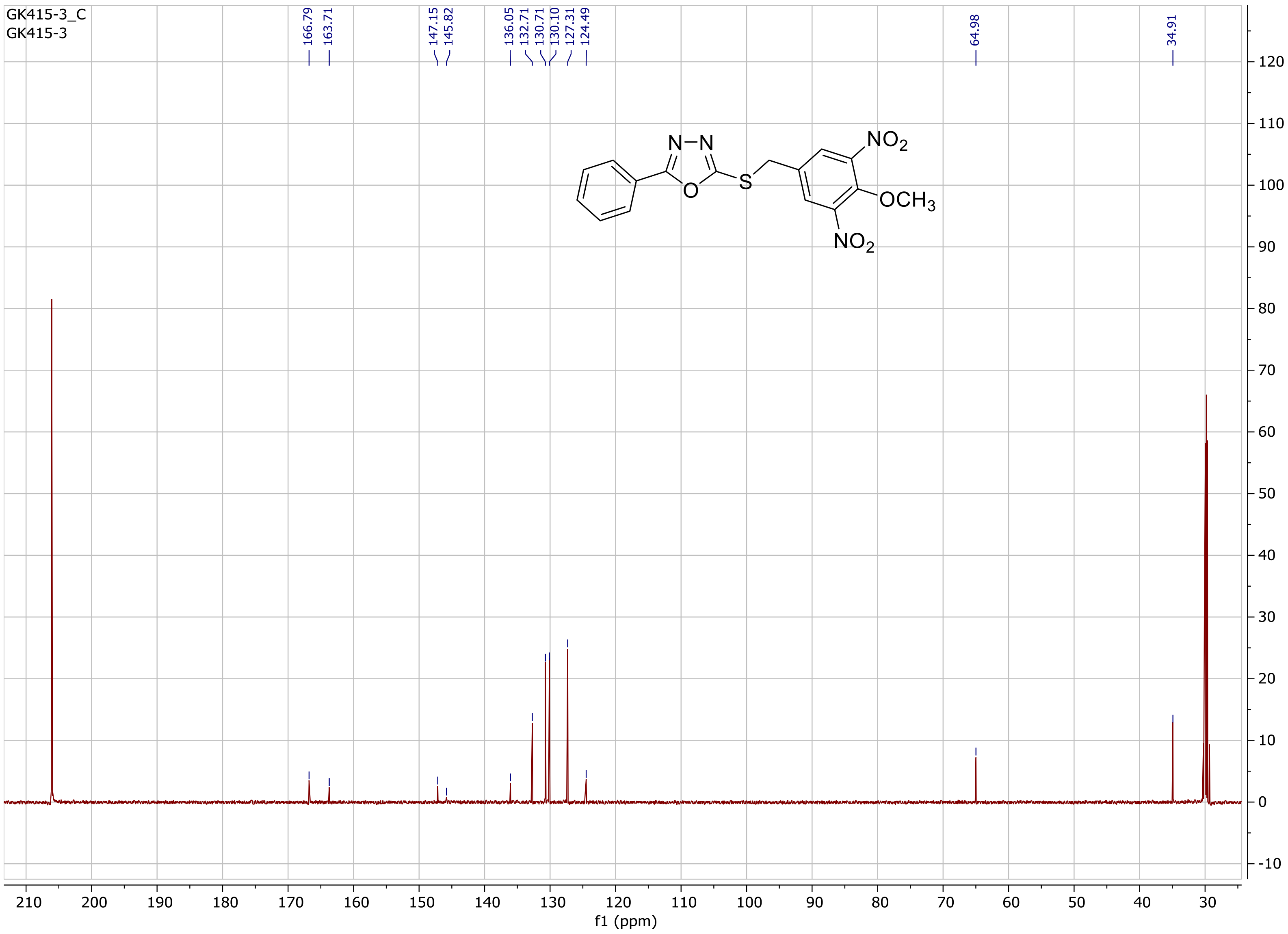

2-((4-Methoxy-3,5-dinitrobenzyl)sulfanyl)-5-(4-methoxyphenyl)-1,3,4-oxadiazole (**78b**): ^{1}H NMR (500 MHz, DMSO-d_6)

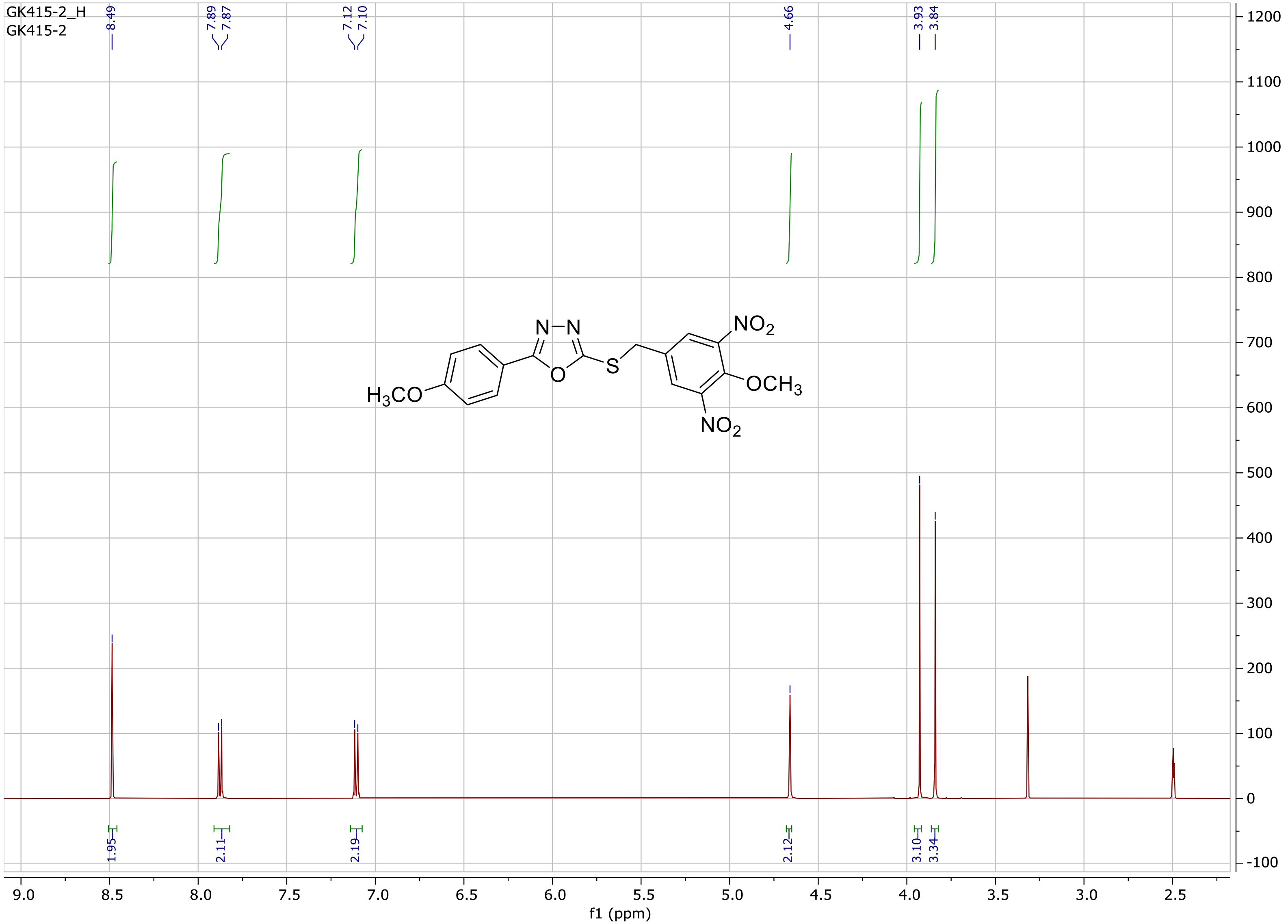

2-((4-Methoxy-3,5-dinitrobenzyl)sulfanyl)-5-(4-methoxyphenyl)-1,3,4-oxadiazole (**78b**): ^{13}C NMR (126 MHz, DMSO-d_6)

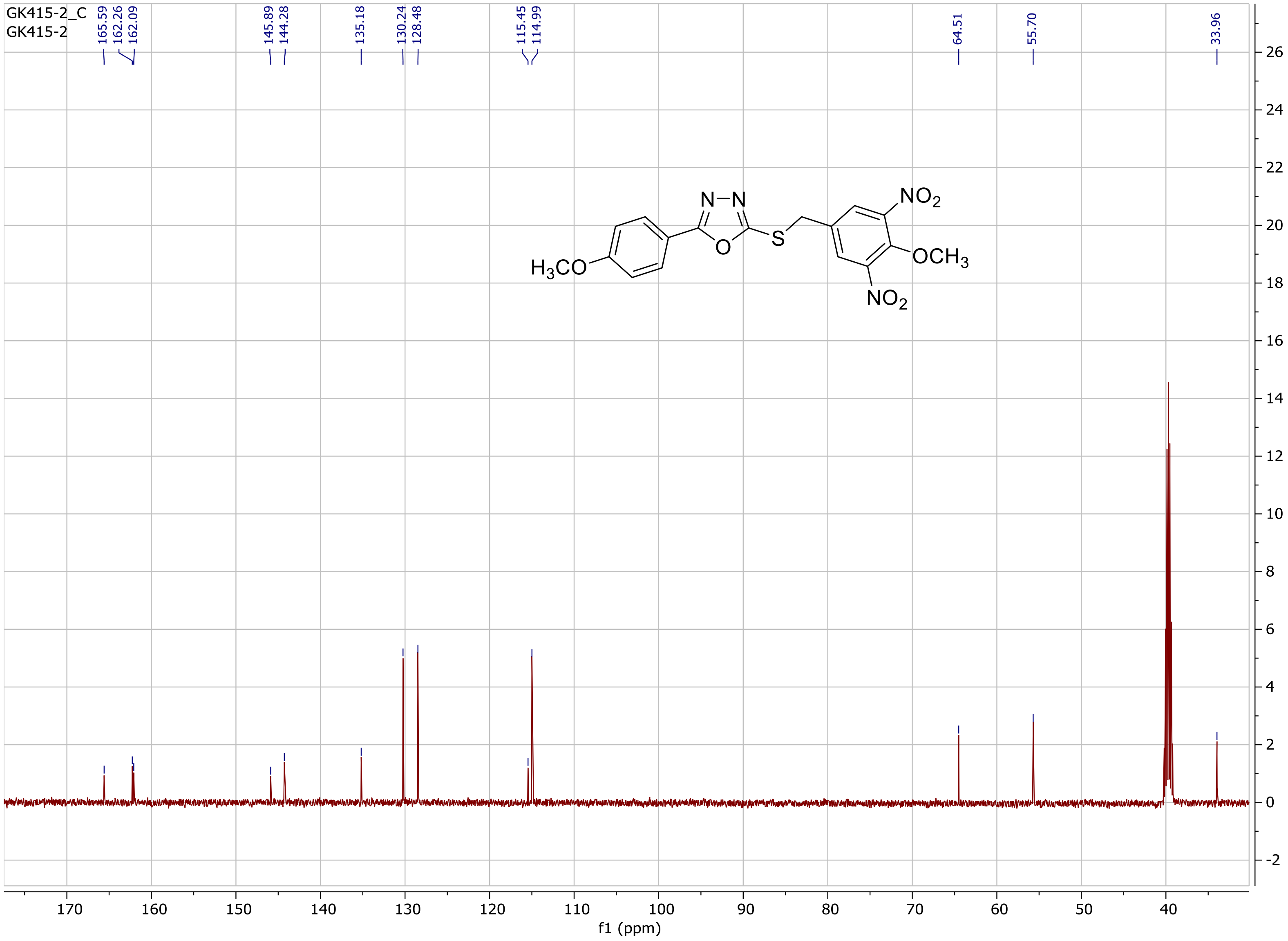

2-(4-Chlorophenyl)-5-((4-methoxy-3,5-dinitrobenzyl)sulfanyl)-1,3,4-oxadiazole (**78c**): ^{1}H NMR (500 MHz, Acetone-d_6)

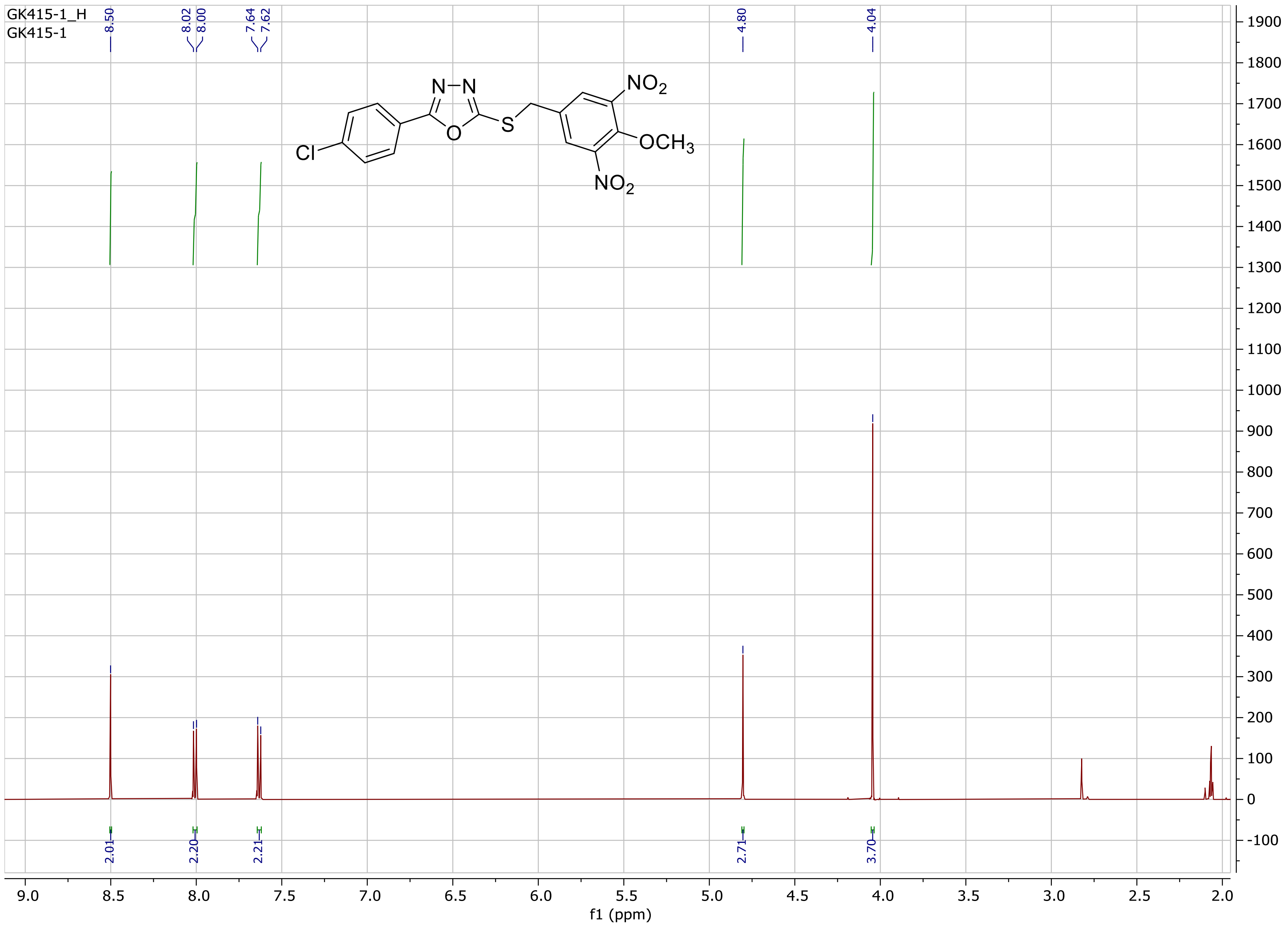

2-(4-Chlorophenyl)-5-((4-methoxy-3,5-dinitrobenzyl)sulfanyl)-1,3,4-oxadiazole (**78c**): ^{13}C NMR (126 MHz, Acetone-d_6)

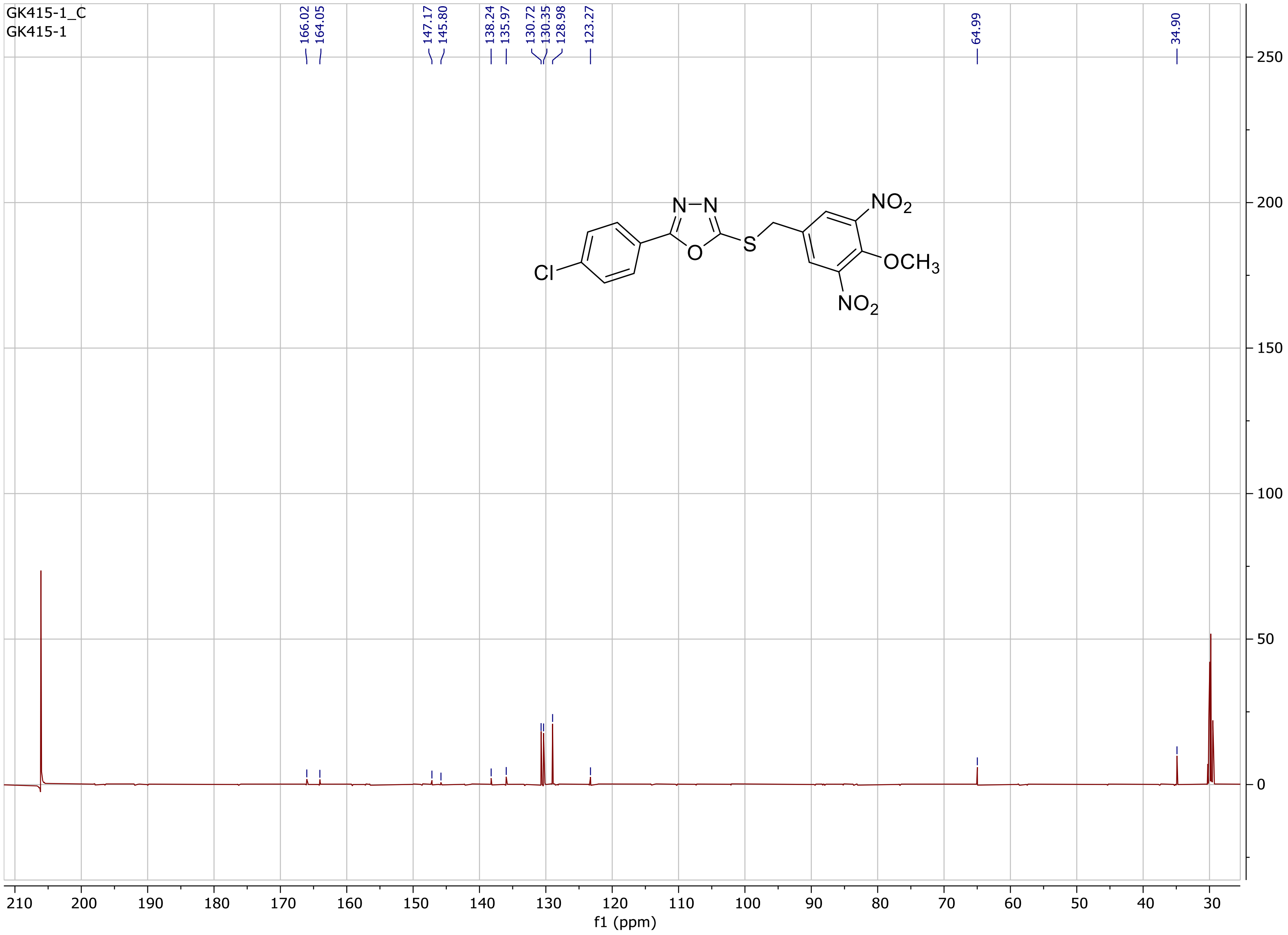

2-(4-Bromophenyl)-5-((4-methoxy-3,5-dinitrobenzyl)sulfanyl)-1,3,4-oxadiazole (**78d**): ^{1}H NMR (500 MHz, DMSO-d_6)

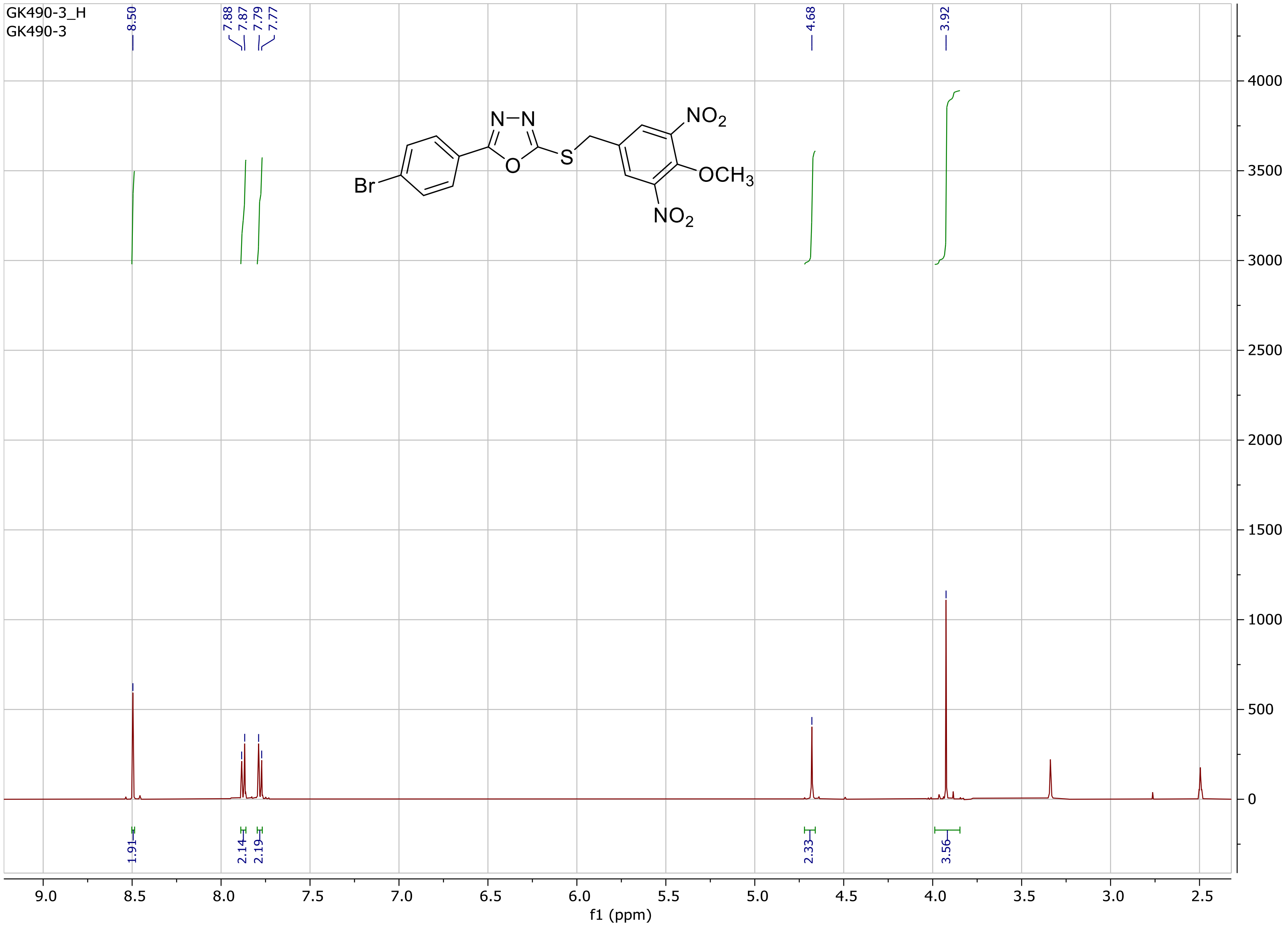

2-(4-Bromophenyl)-5-((4-methoxy-3,5-dinitrobenzyl)sulfanyl)-1,3,4-oxadiazole (**78d**): ^{13}C NMR (126 MHz, DMSO-d_6)

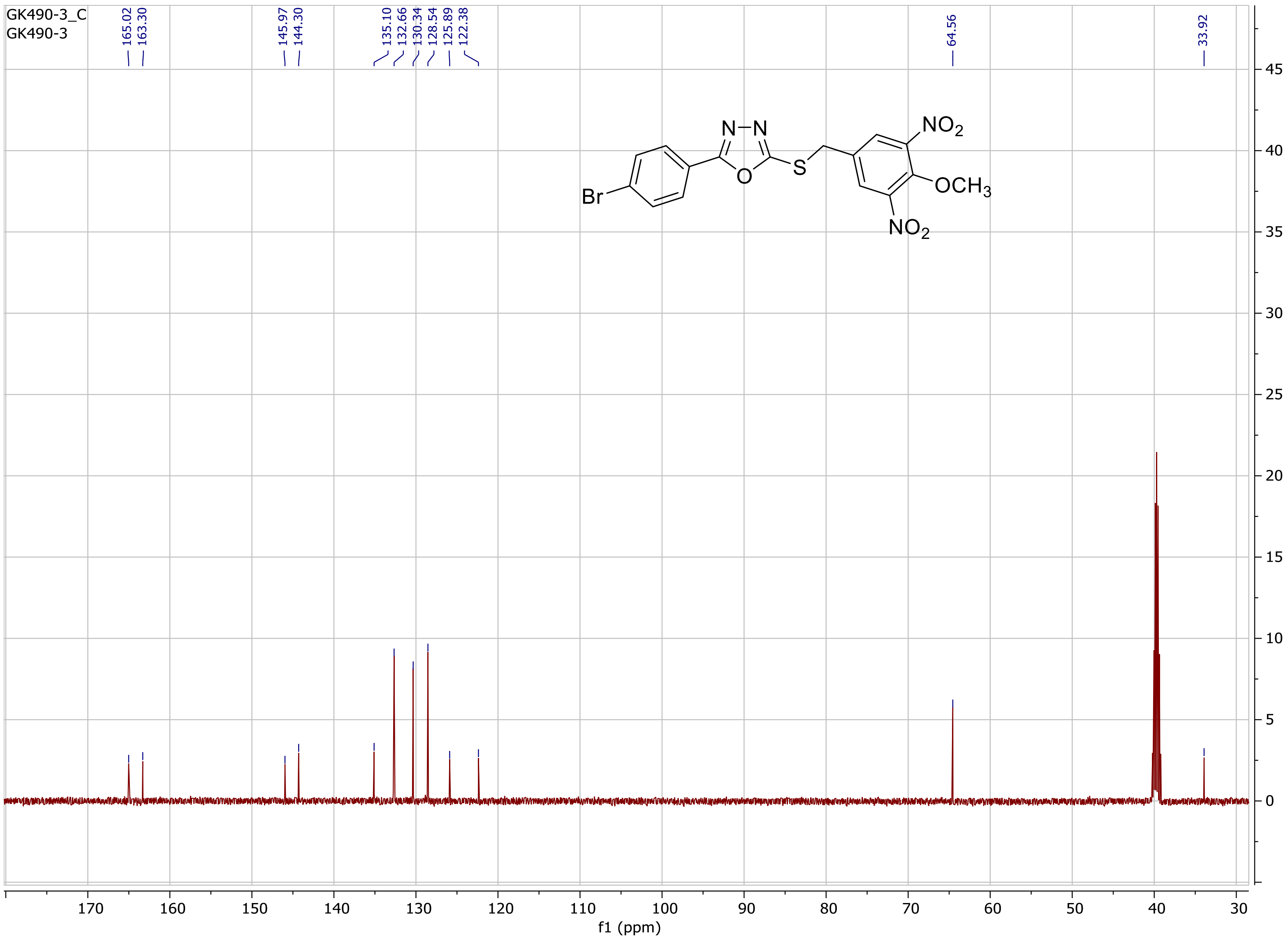

2-Cyclohexyl-5-((4-methoxy-3,5-dinitrobenzyl)sulfanyl)-1,3,4-oxadiazole (**78e**): ^{1}H NMR (600 MHz, DMSO-d_6)

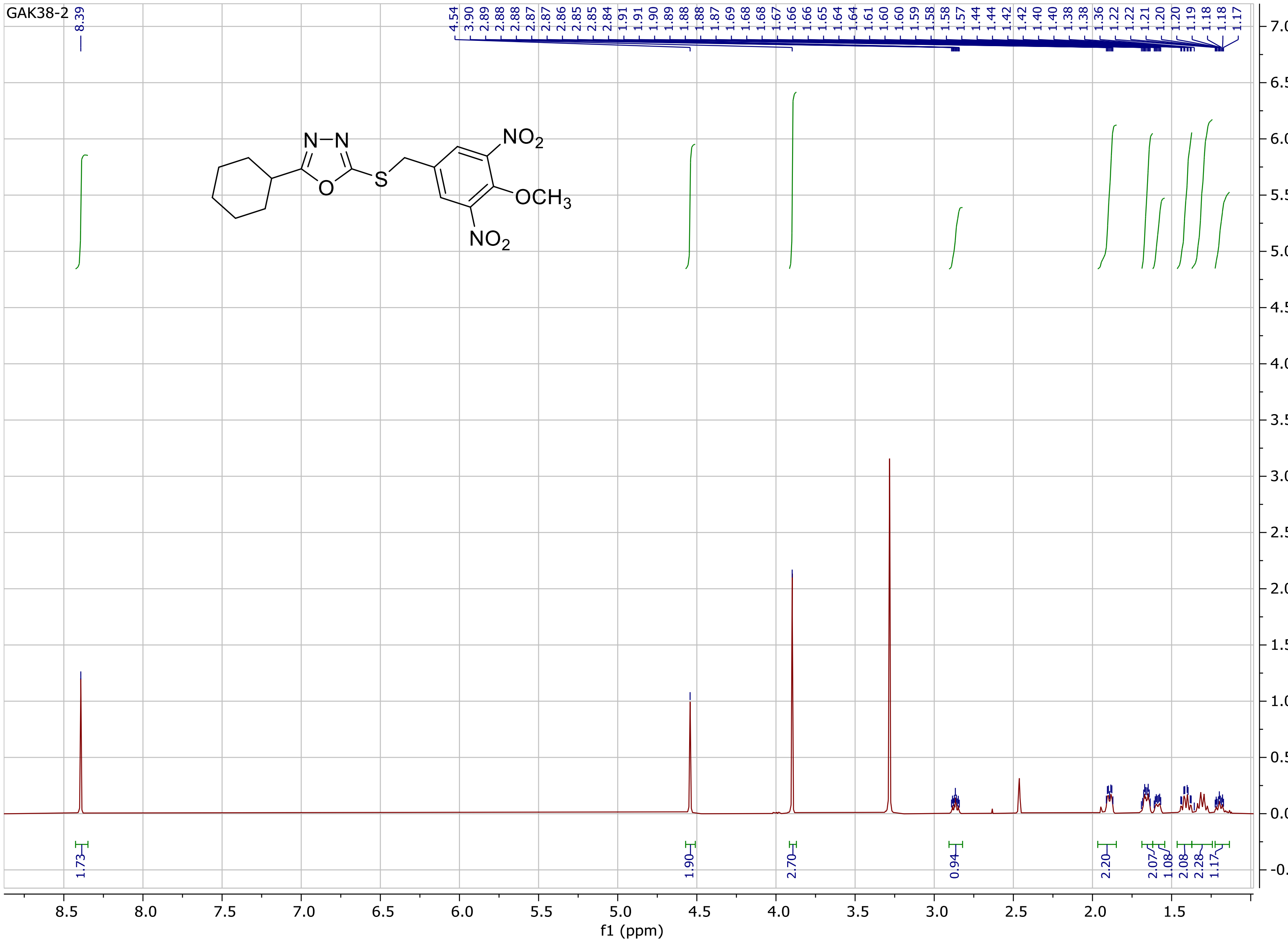

2-Cyclohexyl-5-((4-methoxy-3,5-dinitrobenzyl)sulfanyl)-1,3,4-oxadiazole (**78e**): ^{13}C NMR (151 MHz, DMSO-d_6)

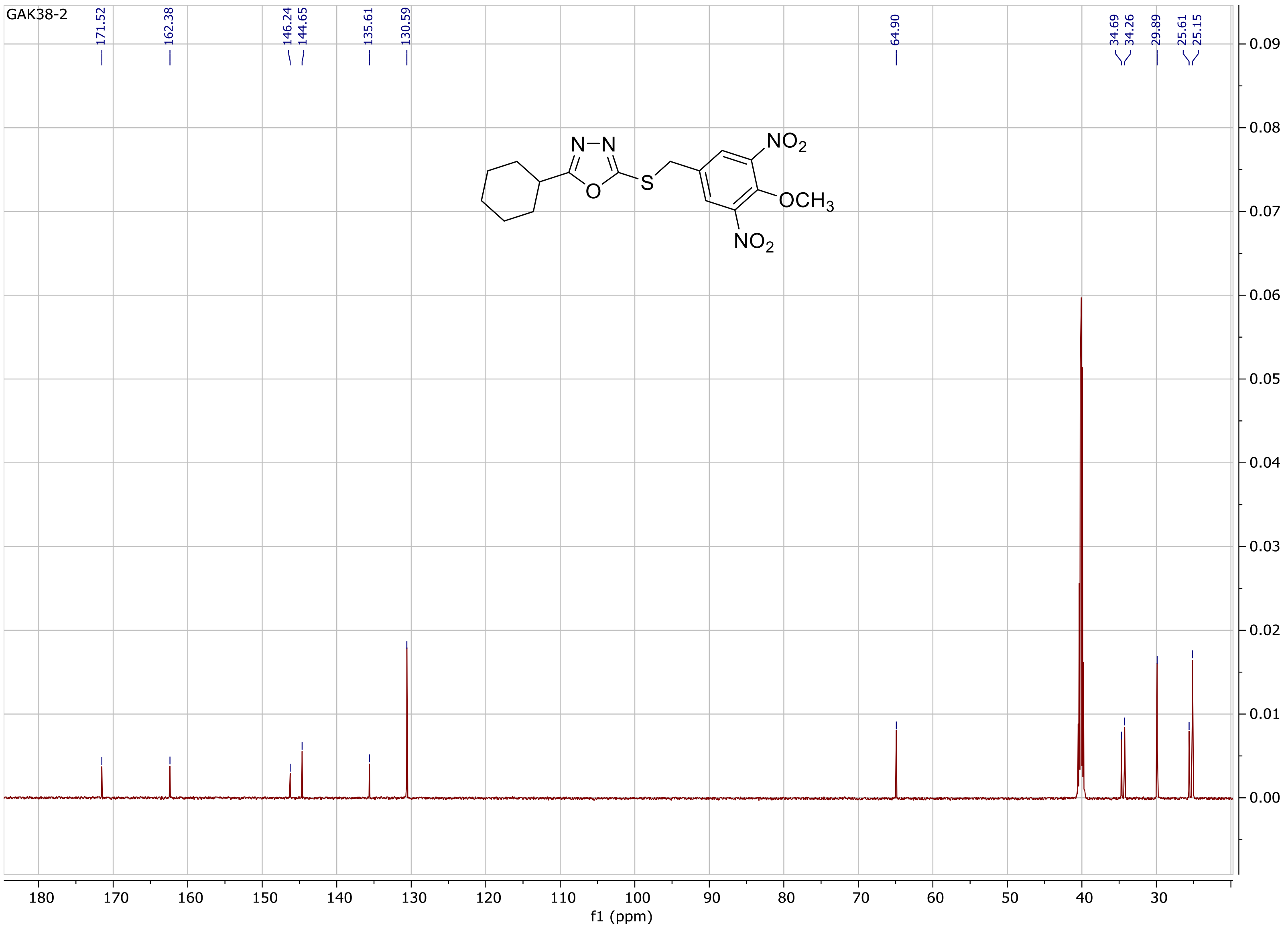

2-((2-Methoxy-3,5-dinitrobenzyl)sulfanyl)-5-phenyl-1,3,4-oxadiazole (**79a**): ^{1}H NMR (500 MHz, Acetone-d_6)

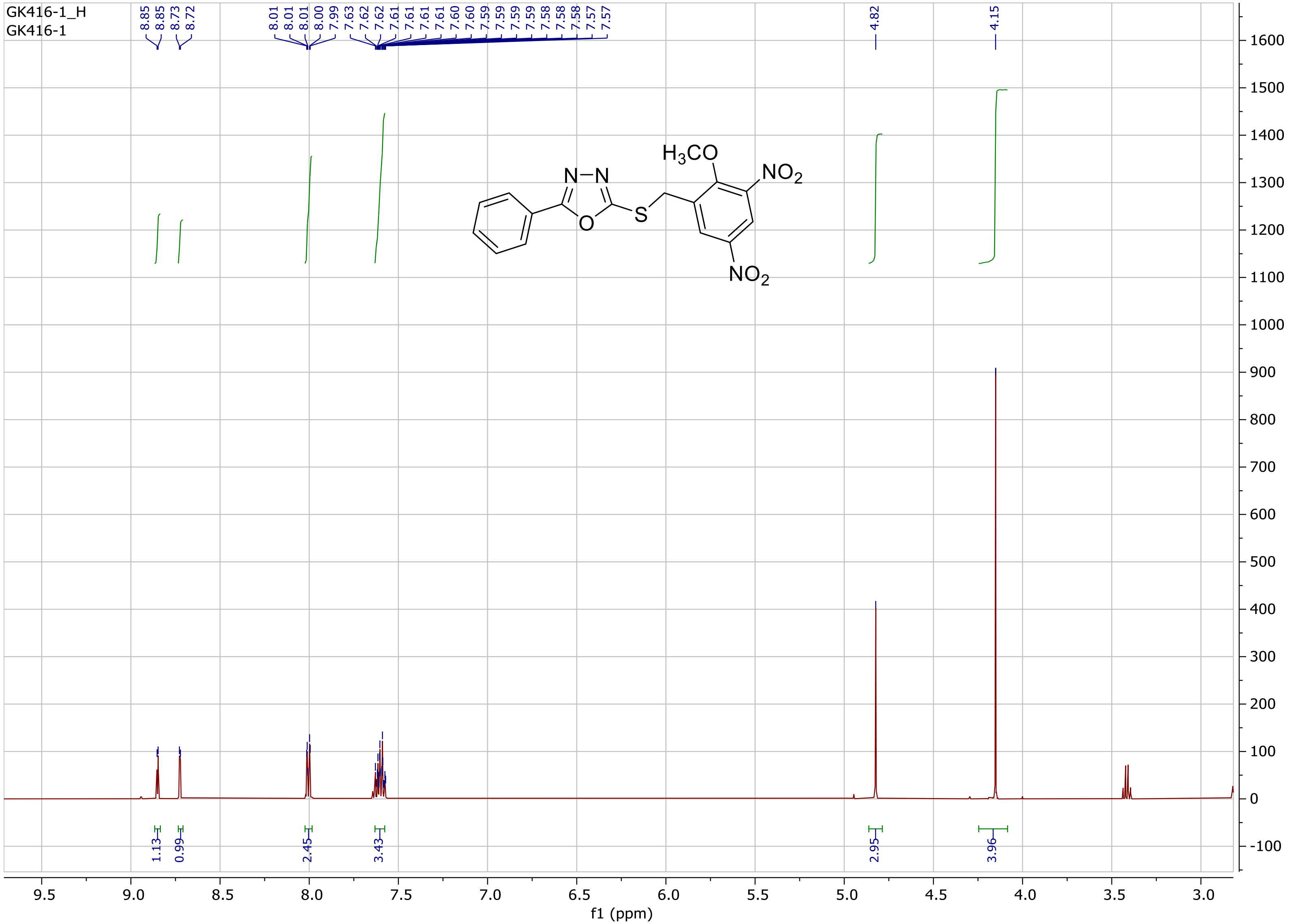

2-((2-Methoxy-3,5-dinitrobenzyl)sulfanyl)-5-phenyl-1,3,4-oxadiazole (**79a**): ^{13}C NMR (126 MHz, Acetone-d_6)

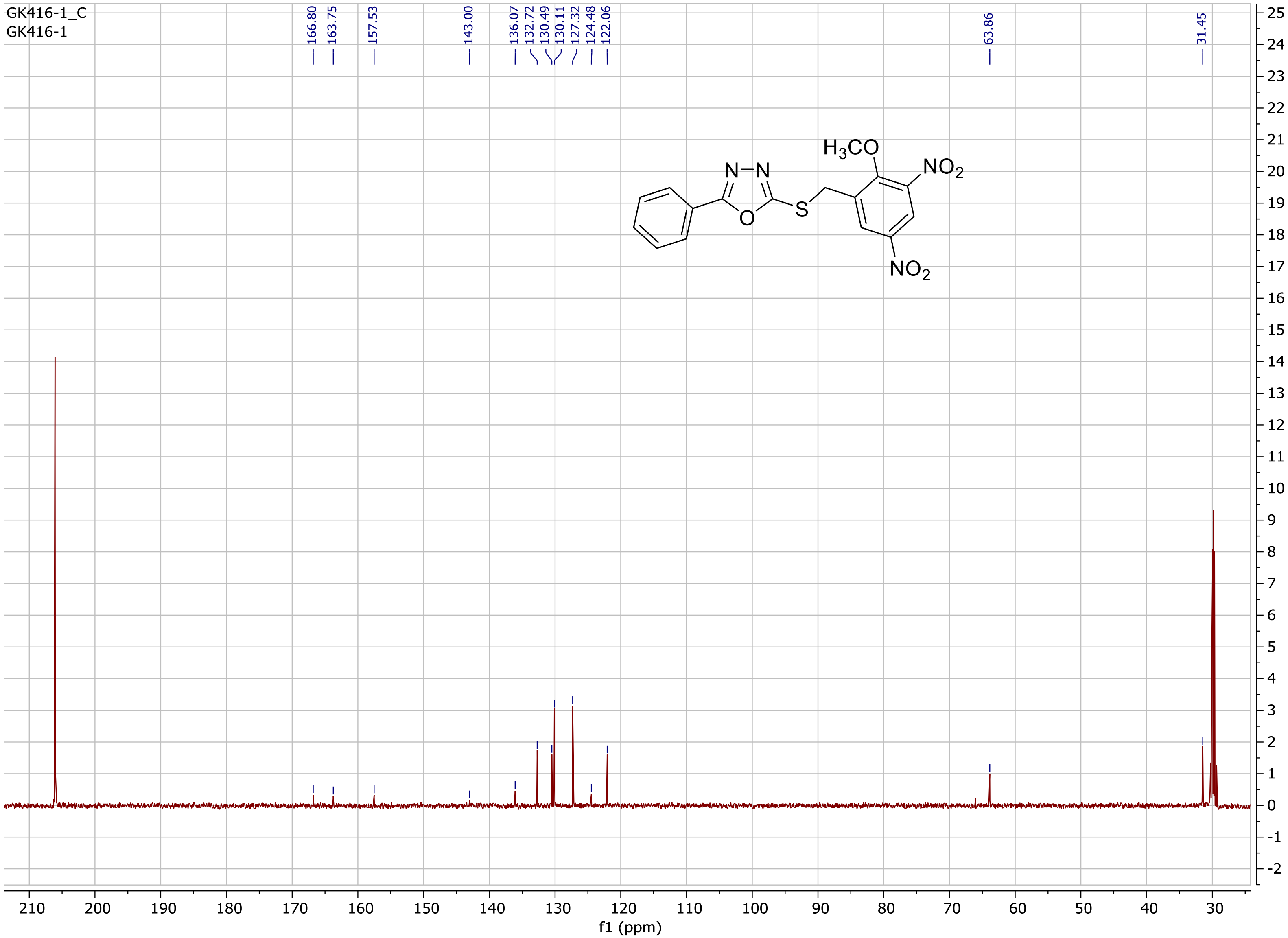

2-((2-Methoxy-3,5-dinitrobenzyl)sulfanyl)-5-(4-methoxyphenyl)-1,3,4-oxadiazole (**79b**): ^{1}H NMR (500 MHz, Acetone-d_6)

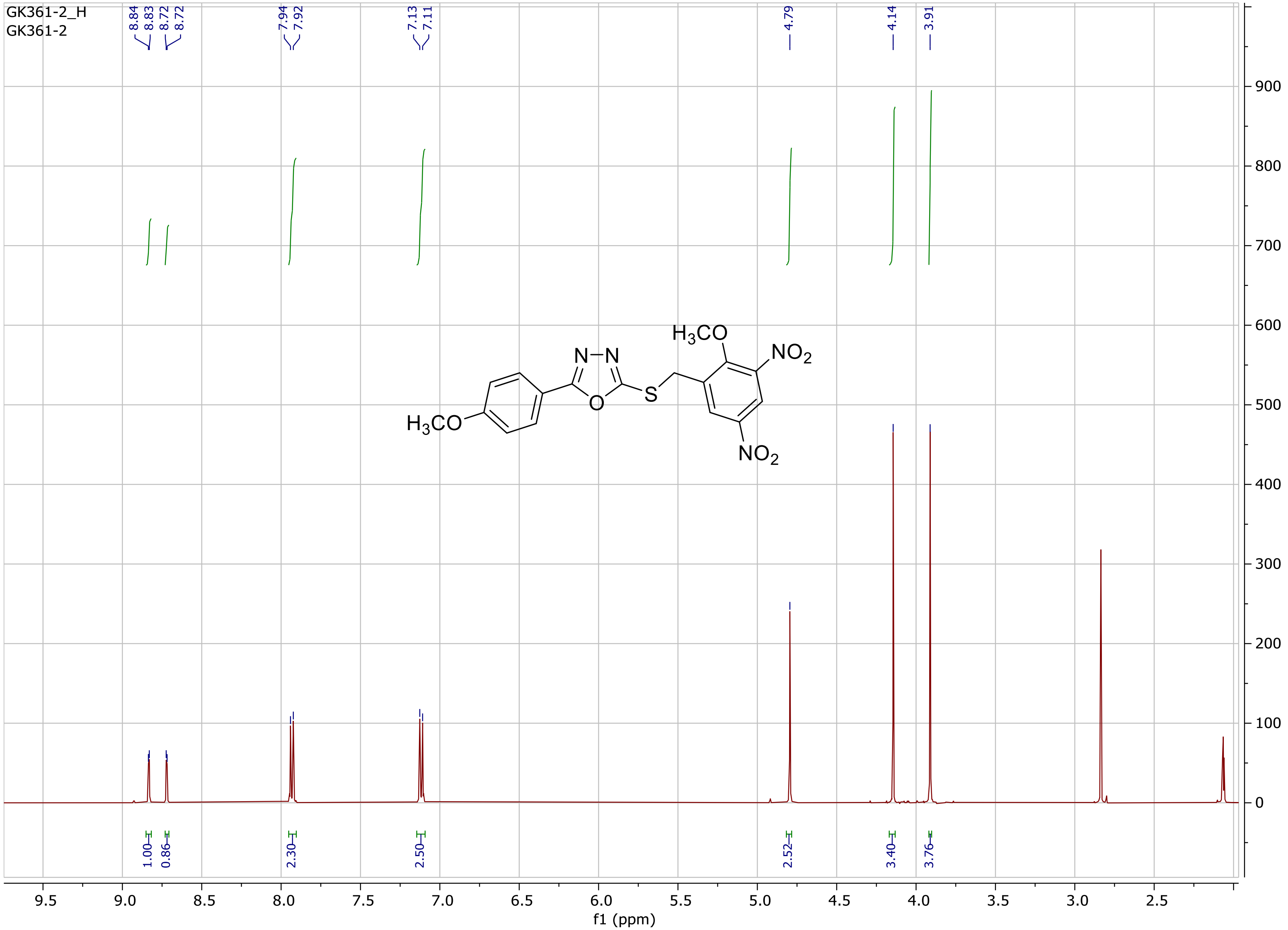

2-((2-Methoxy-3,5-dinitrobenzyl)sulfanyl)-5-(4-methoxyphenyl)-1,3,4-oxadiazole (**79b**): ^{13}C NMR (126 MHz, Acetone-d_6)

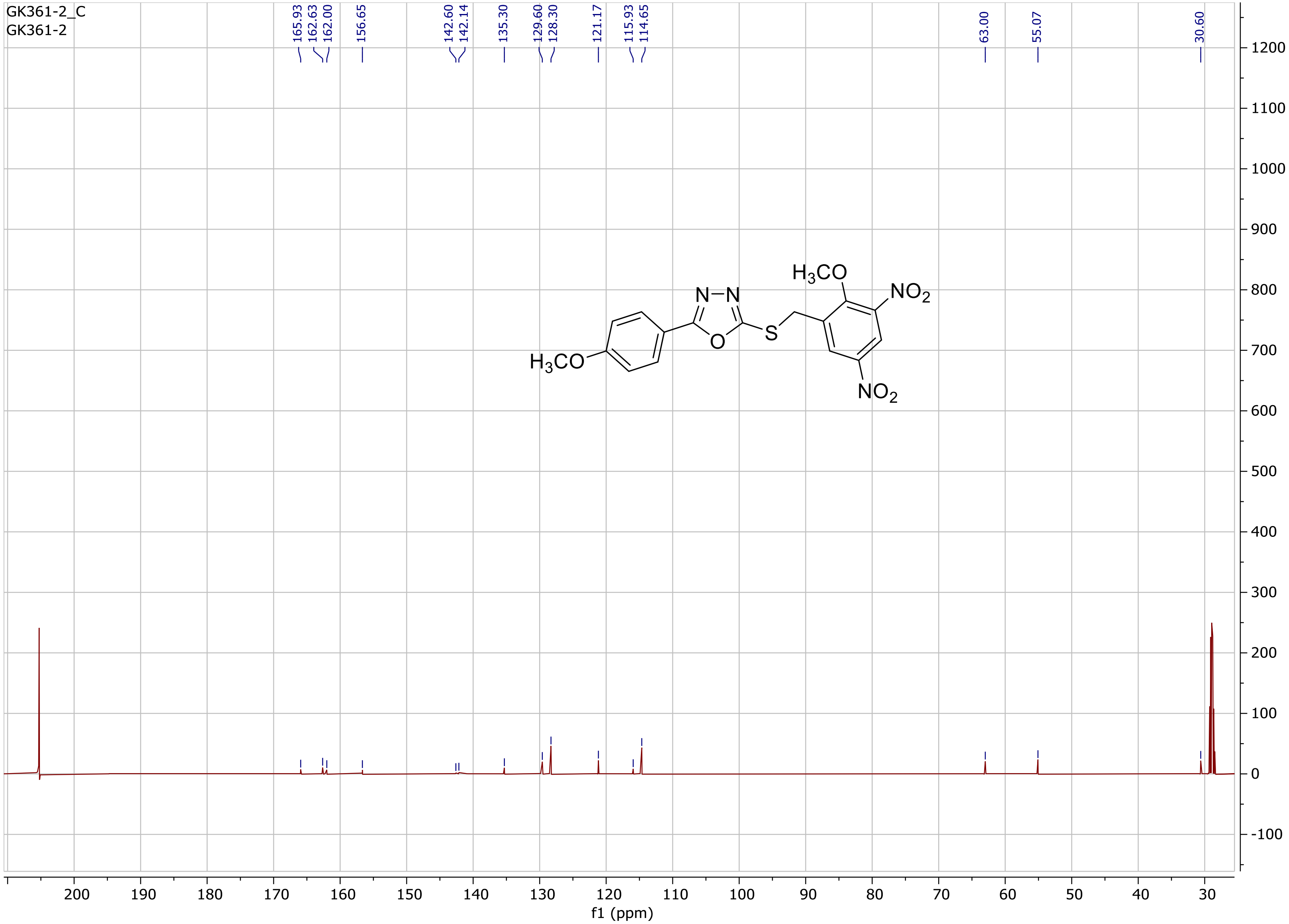

2-(4-Chlorophenyl)-5-((2-methoxy-3,5-dinitrobenzyl)sulfanyl)-1,3,4-oxadiazole (**79c**): ^{1}H NMR (500 MHz, Acetone-d_6)

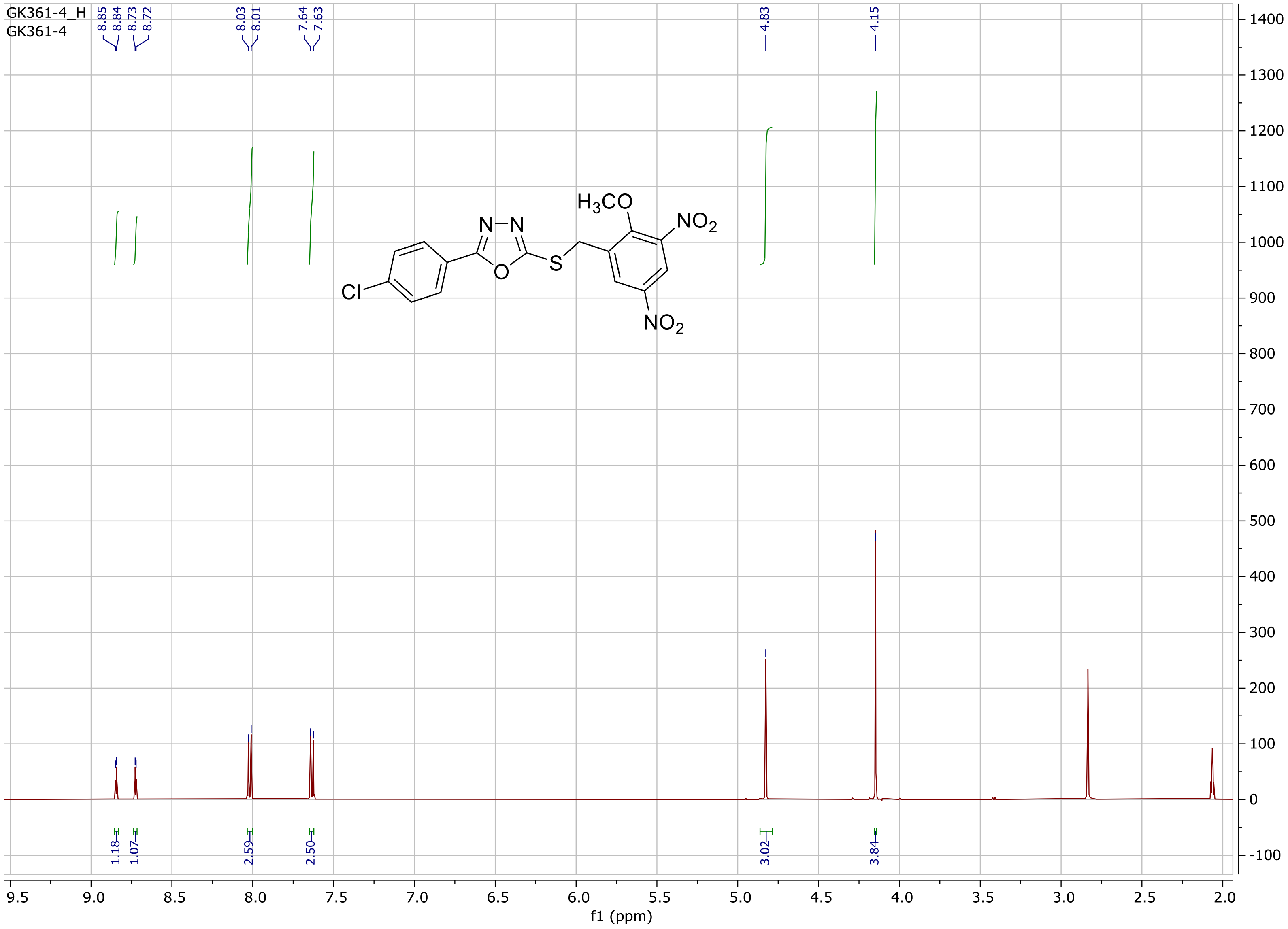

2-(4-Chlorophenyl)-5-((2-methoxy-3,5-dinitrobenzyl)sulfanyl)-1,3,4-oxadiazole (**79c**): ^{13}C NMR (126 MHz, Acetone-d_6)

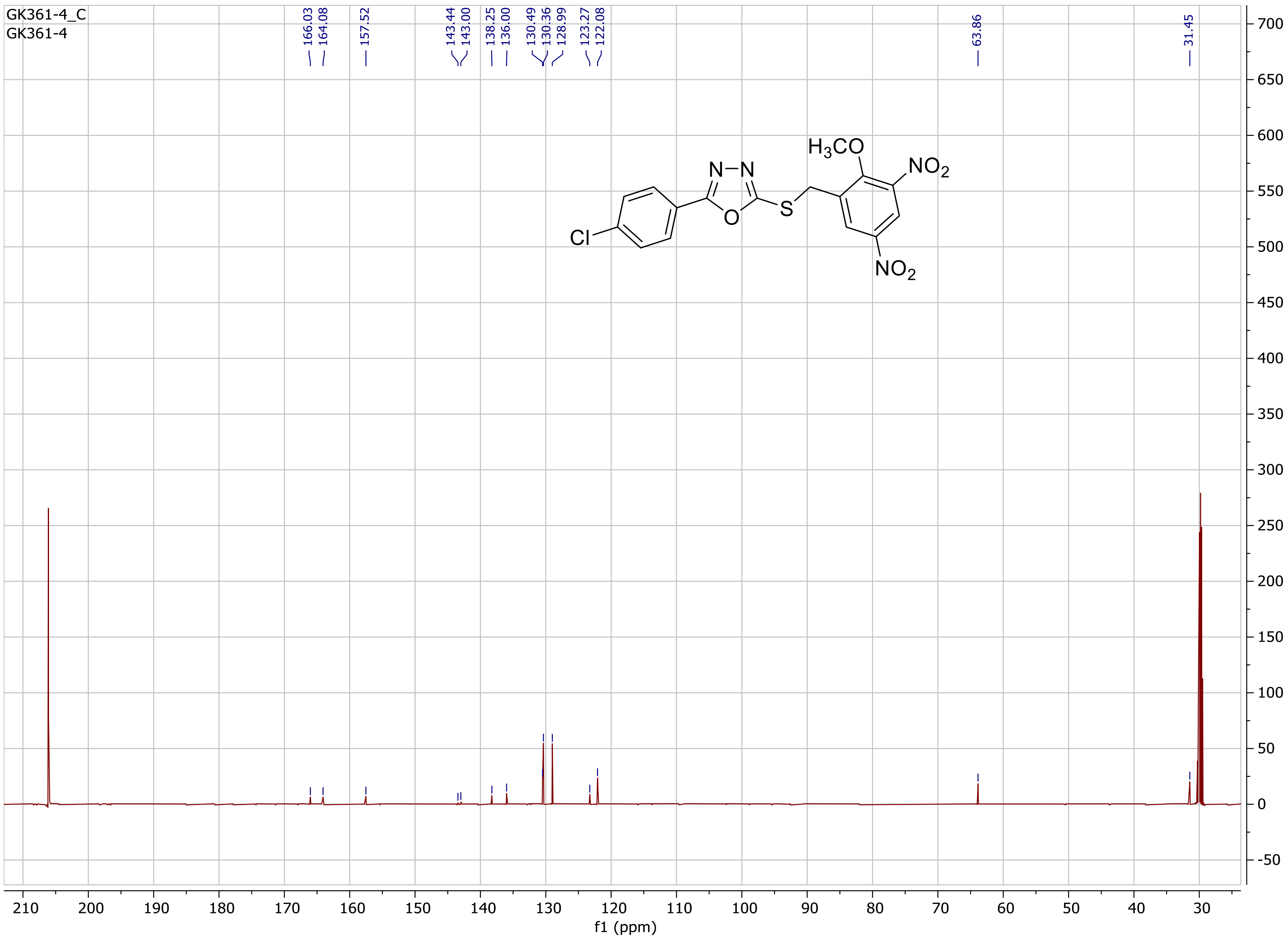

2-(4-Bromophenyl)-5-((2-methoxy-3,5-dinitrobenzyl)sulfanyl)-1,3,4-oxadiazole (**79d**): ^{1}H NMR (500 MHz, DMSO-d_6)

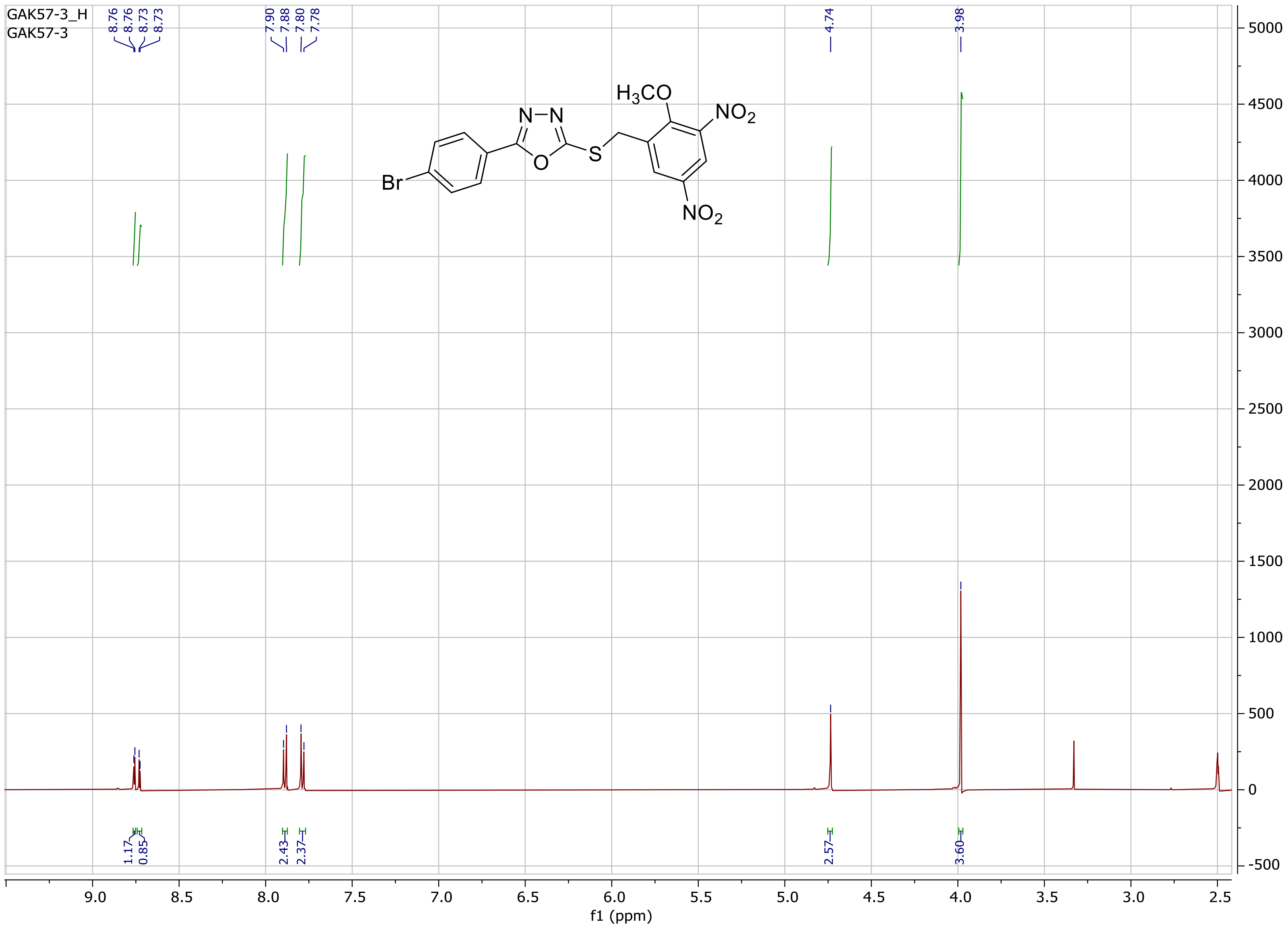

2-(4-Bromophenyl)-5-((2-methoxy-3,5-dinitrobenzyl)sulfanyl)-1,3,4-oxadiazole (**79d**): ^{13}C NMR (126 MHz, DMSO-d_6)

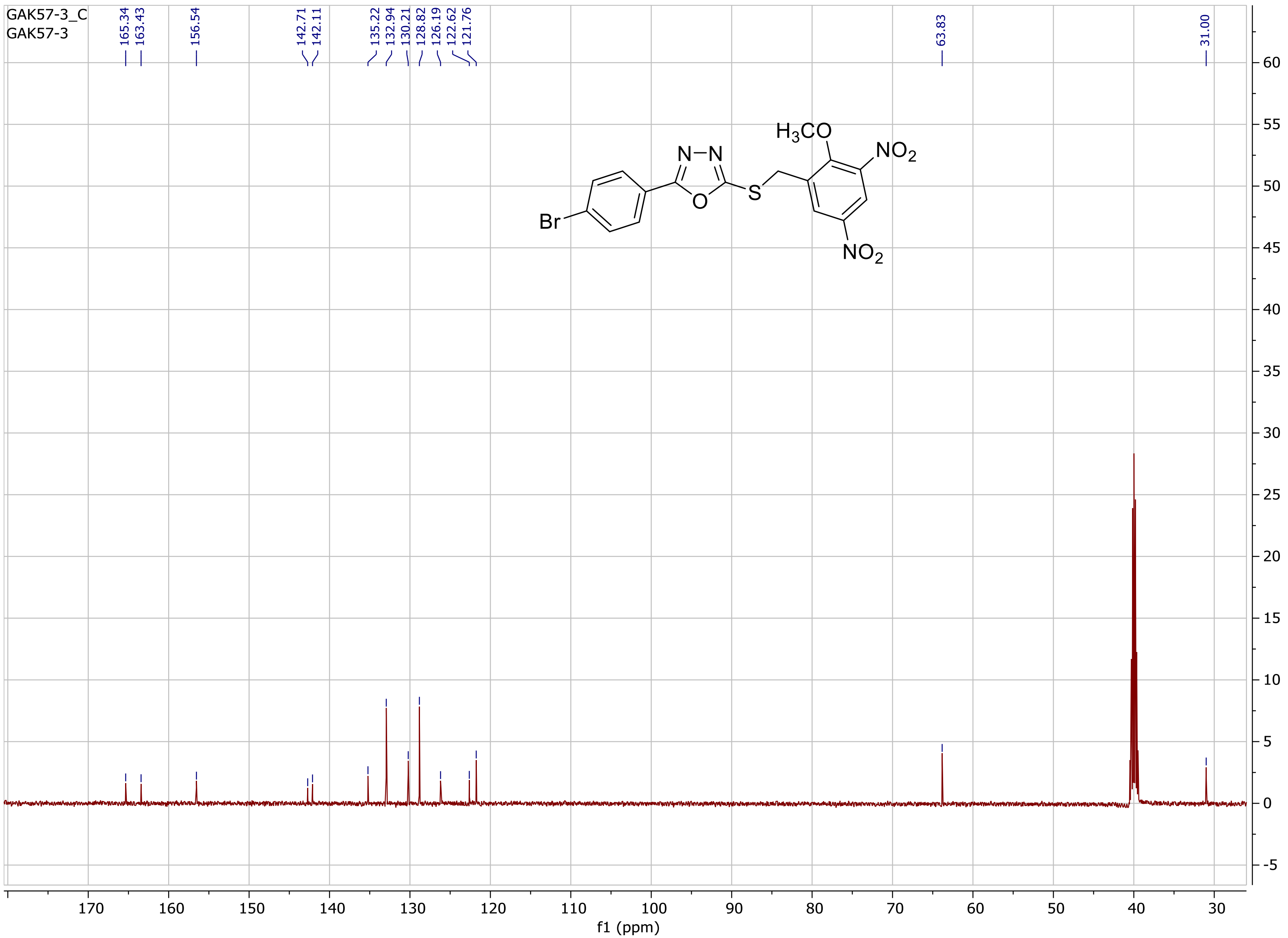

2-Cyclohexyl-5-((2-methoxy-3,5-dinitrobenzyl)sulfanyl)-1,3,4-oxadiazole (**79e**): ^{1}H NMR (600 MHz, DMSO-d_6)

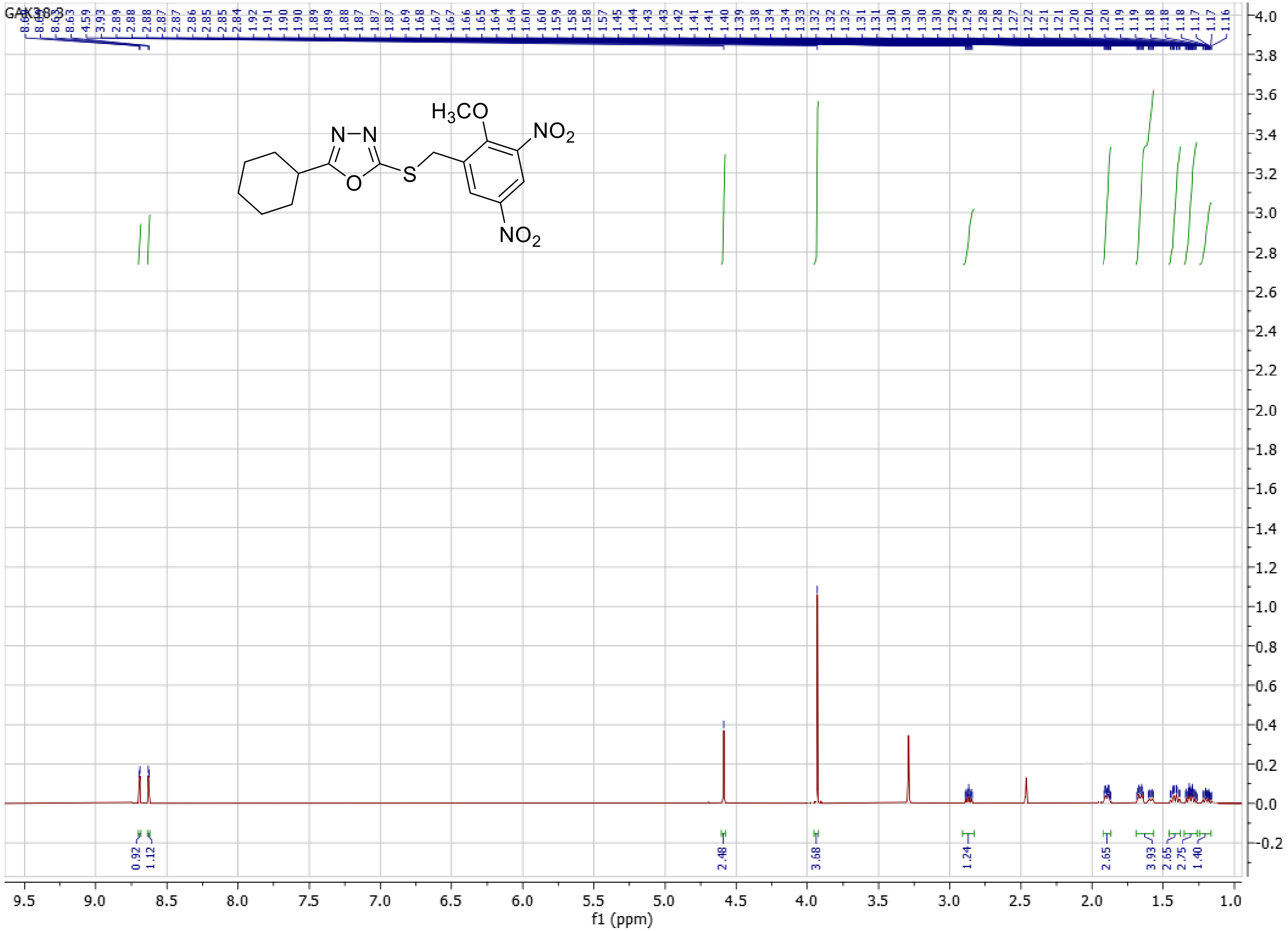

2-Cyclohexyl-5-((2-methoxy-3,5-dinitrobenzyl)sulfanyl)-1,3,4-oxadiazole (**79e**): ^{13}C NMR (151 MHz, DMSO-d_6)

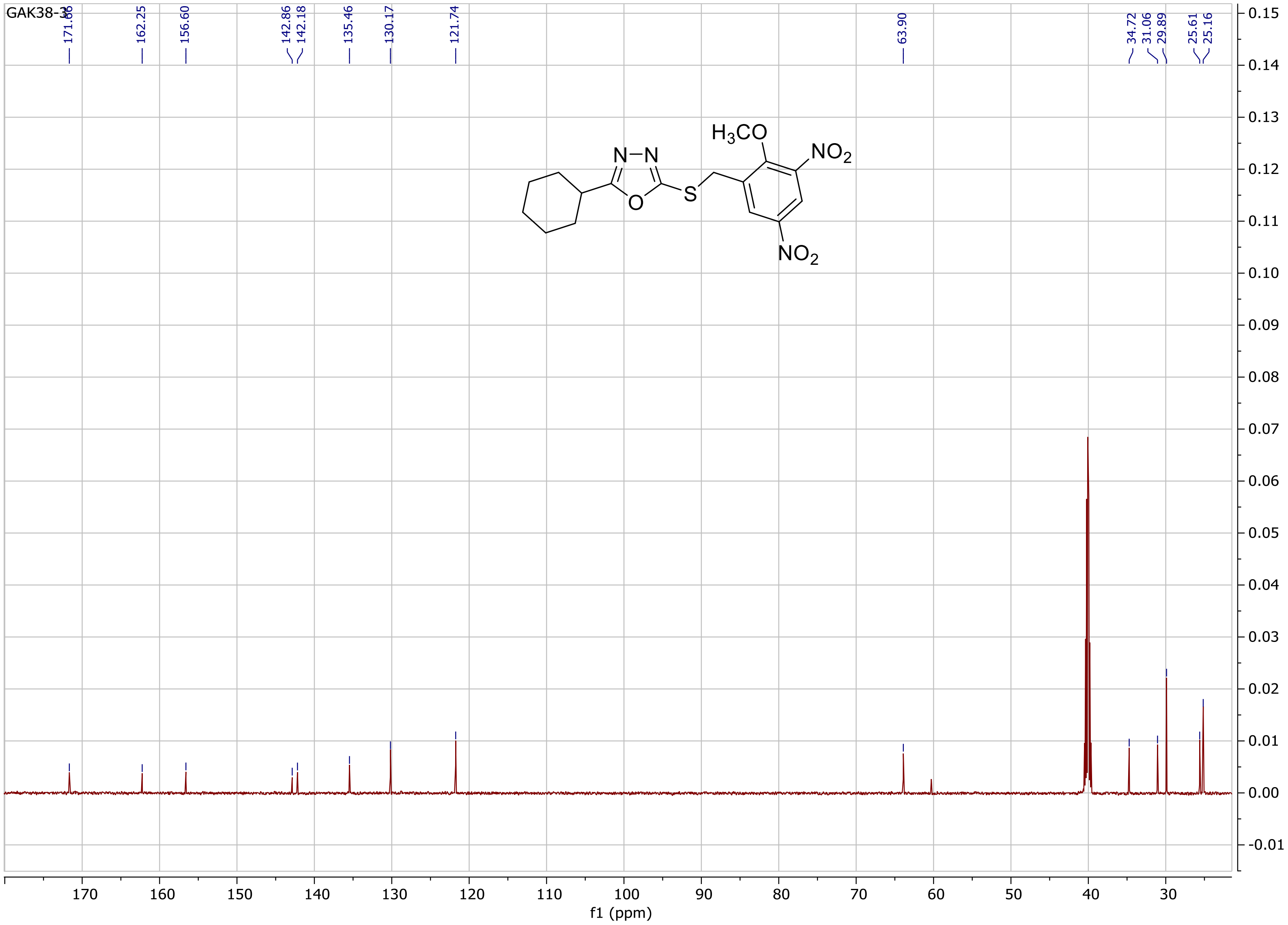

2-Cyclohexyl-5-((2-methoxy-3,5-dinitrobenzyl)sulfanyl)-1,3,4-oxadiazole (**79e**):

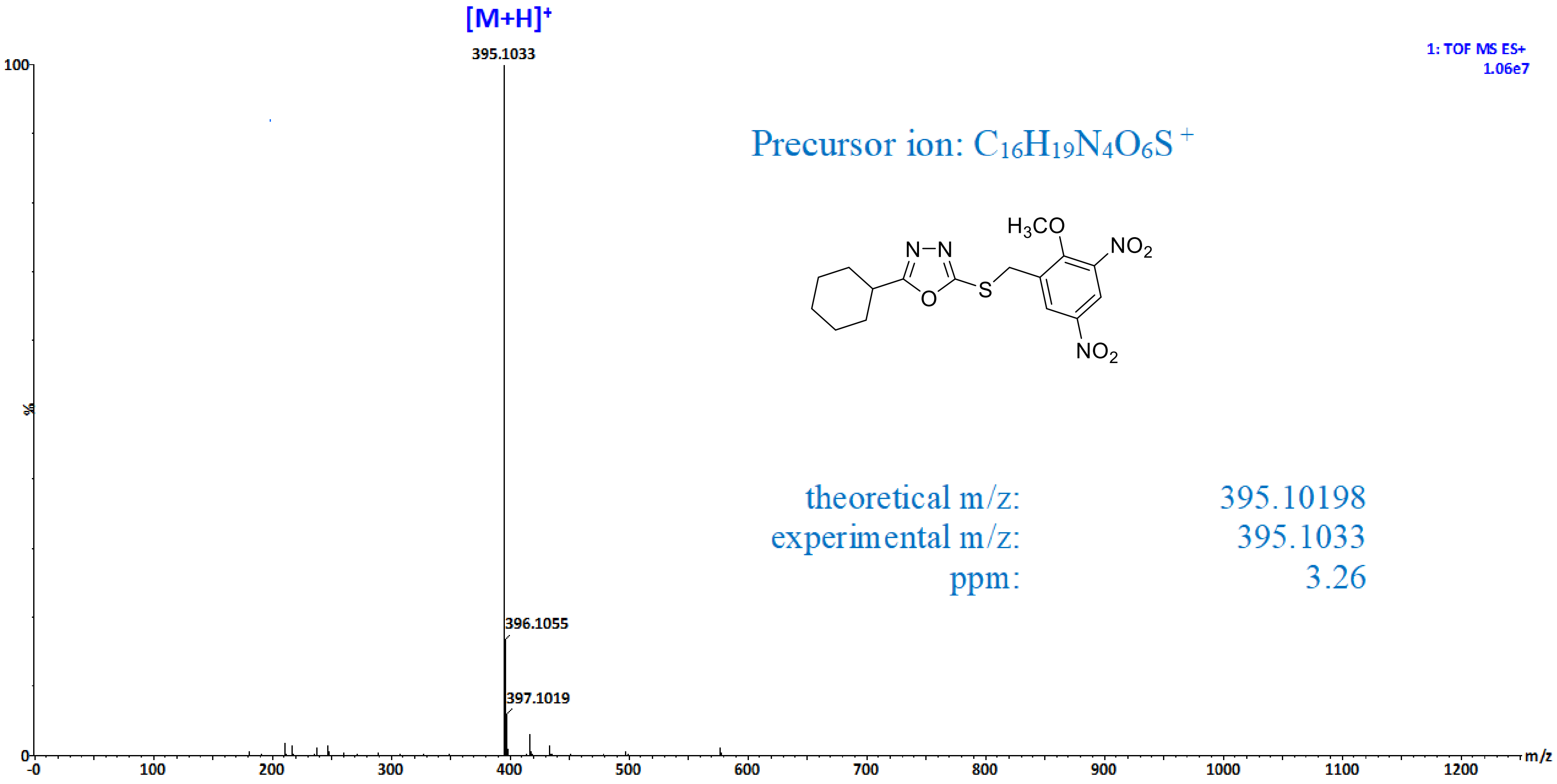

2-((4-Methyl-3,5-dinitrobenzyl)sulfanyl)-5-phenyl-1,3,4-oxadiazole (**80a**): ^{1}H NMR (500 MHz, Acetone-d_6)

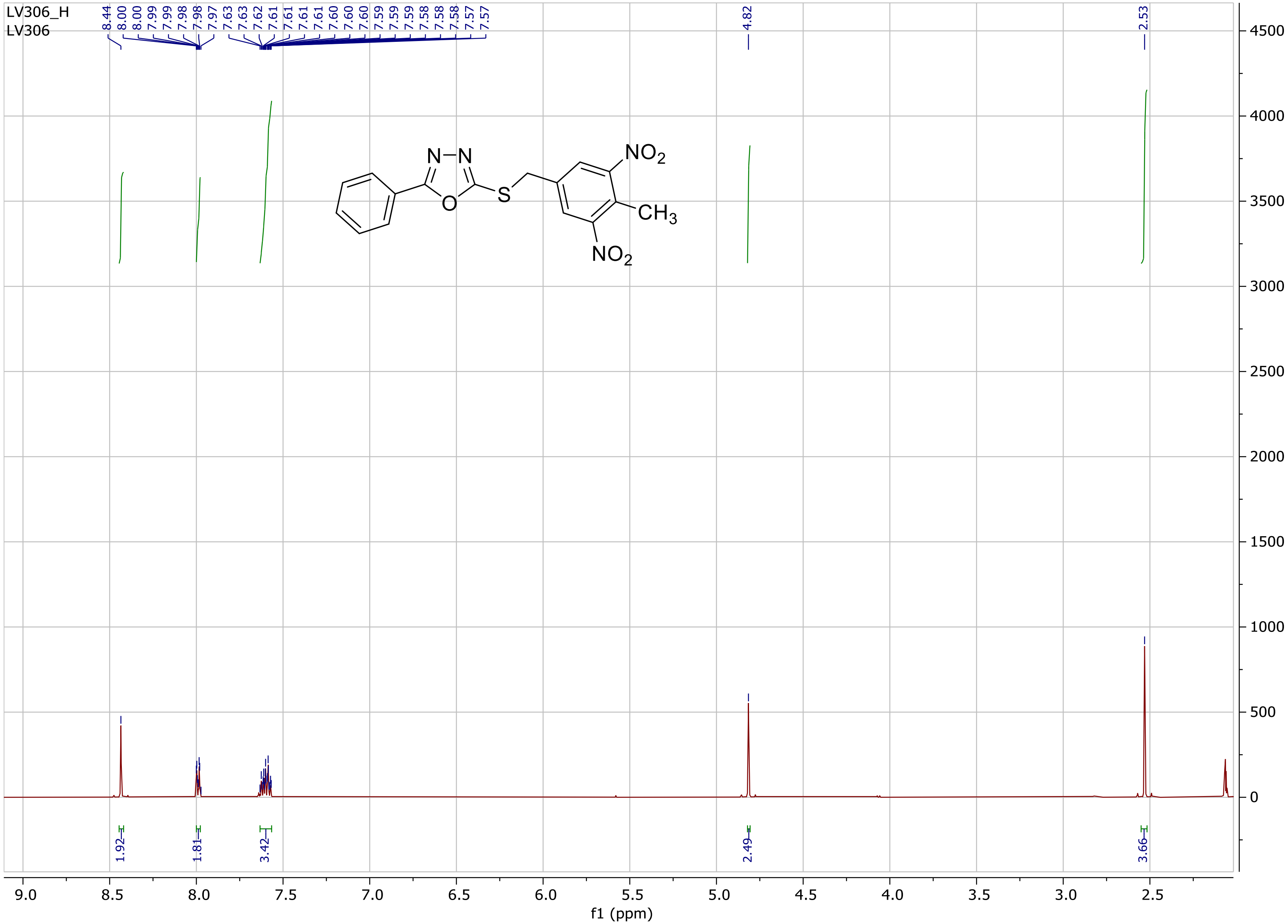

2-((4-Methyl-3,5-dinitrobenzyl)sulfanyl)-5-phenyl-1,3,4-oxadiazole (**80a**): ^{13}C NMR (126 MHz, Acetone-d_6)

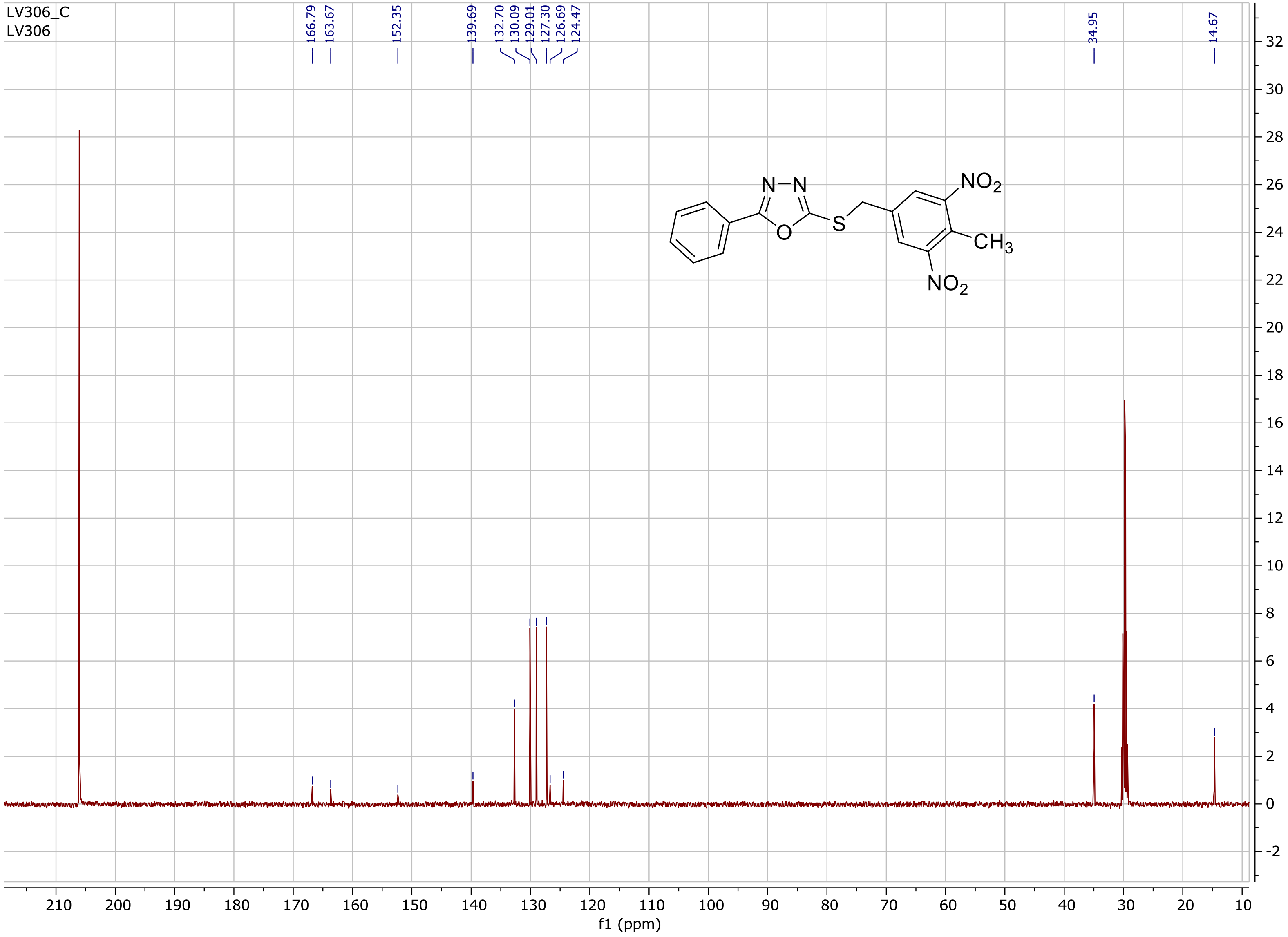

5-(4-Methoxyphenyl)-2-((4-methyl-3,5-dinitrobenzyl)sulfanyl)-1,3,4-oxadiazole (**80b**): ^{1}H NMR (600 MHz, DMSO-d_6)

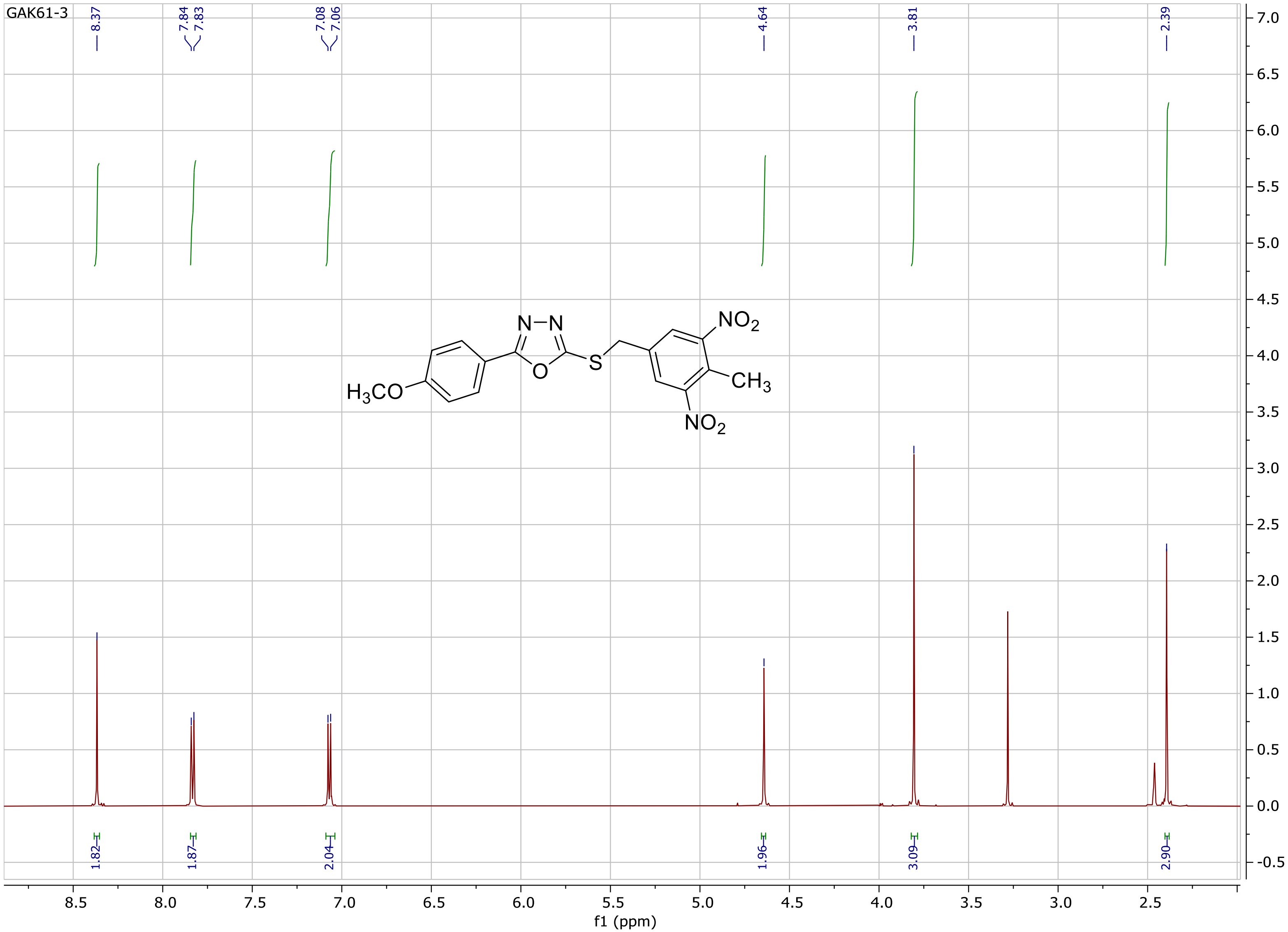

5-(4-Methoxyphenyl)-2-((4-methyl-3,5-dinitrobenzyl)sulfanyl)-1,3,4-oxadiazole (**80b**): ^{13}C NMR (151 MHz, DMSO-d_6)

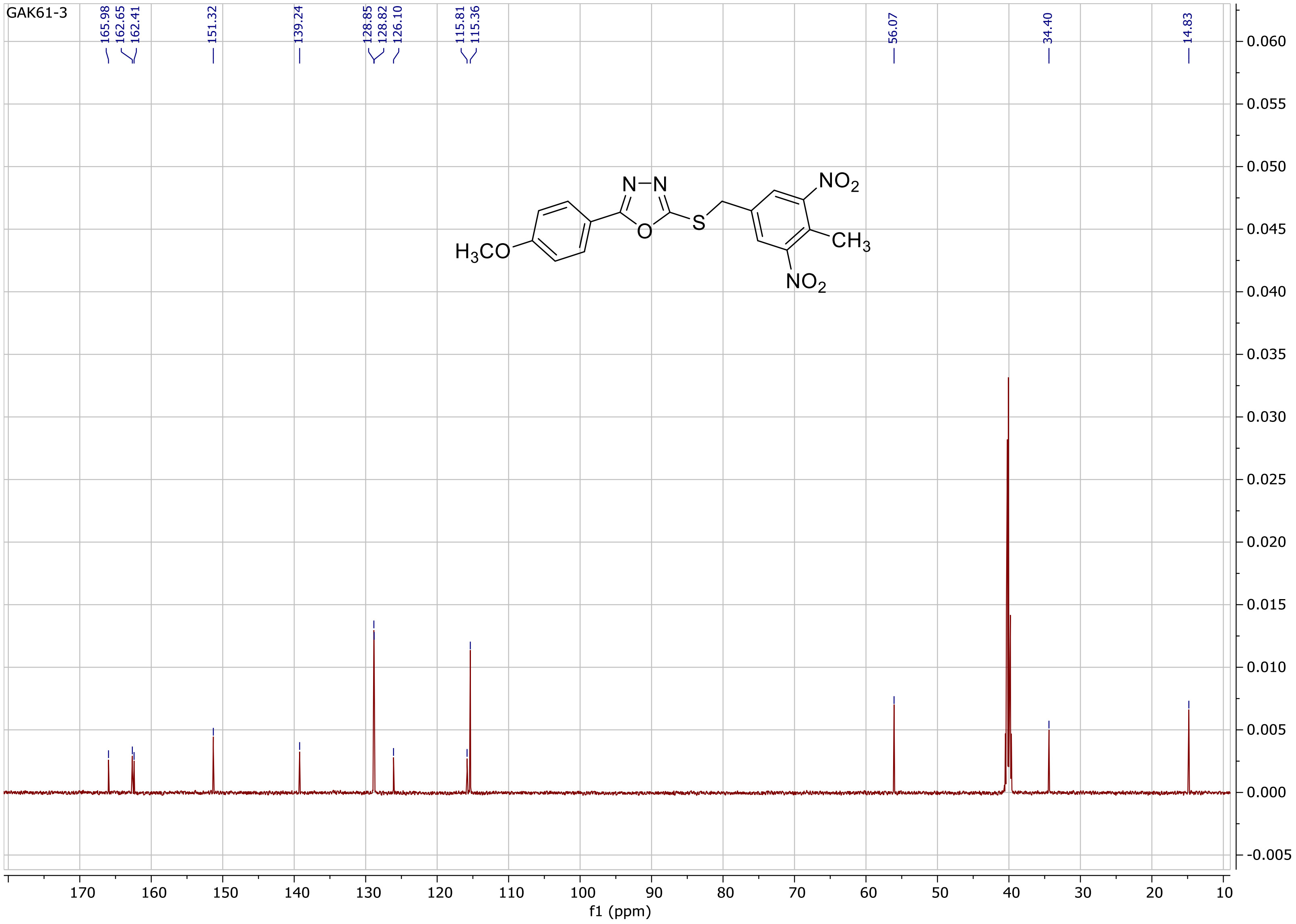

2-(4-Chlorophenyl)-5-((4-methyl-3,5-dinitrobenzyl)sulfanyl)-1,3,4-oxadiazole (**80c**): ^{1}H NMR (500 MHz, Acetone-d_6)

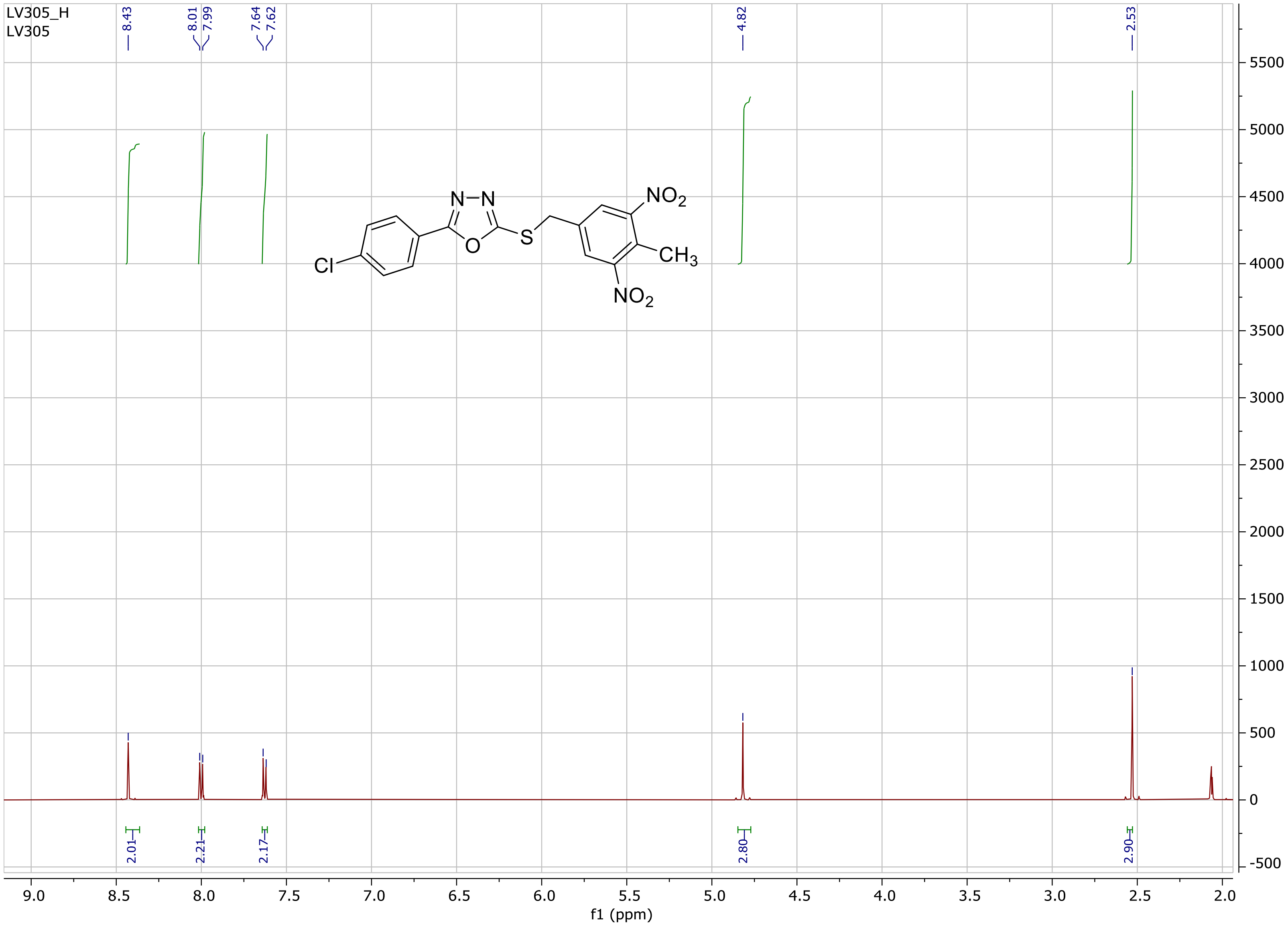

2-(4-Chlorophenyl)-5-((4-methyl-3,5-dinitrobenzyl)sulfanyl)-1,3,4-oxadiazole (**80c**): ^{13}C NMR (126 MHz, Acetone-d_6)

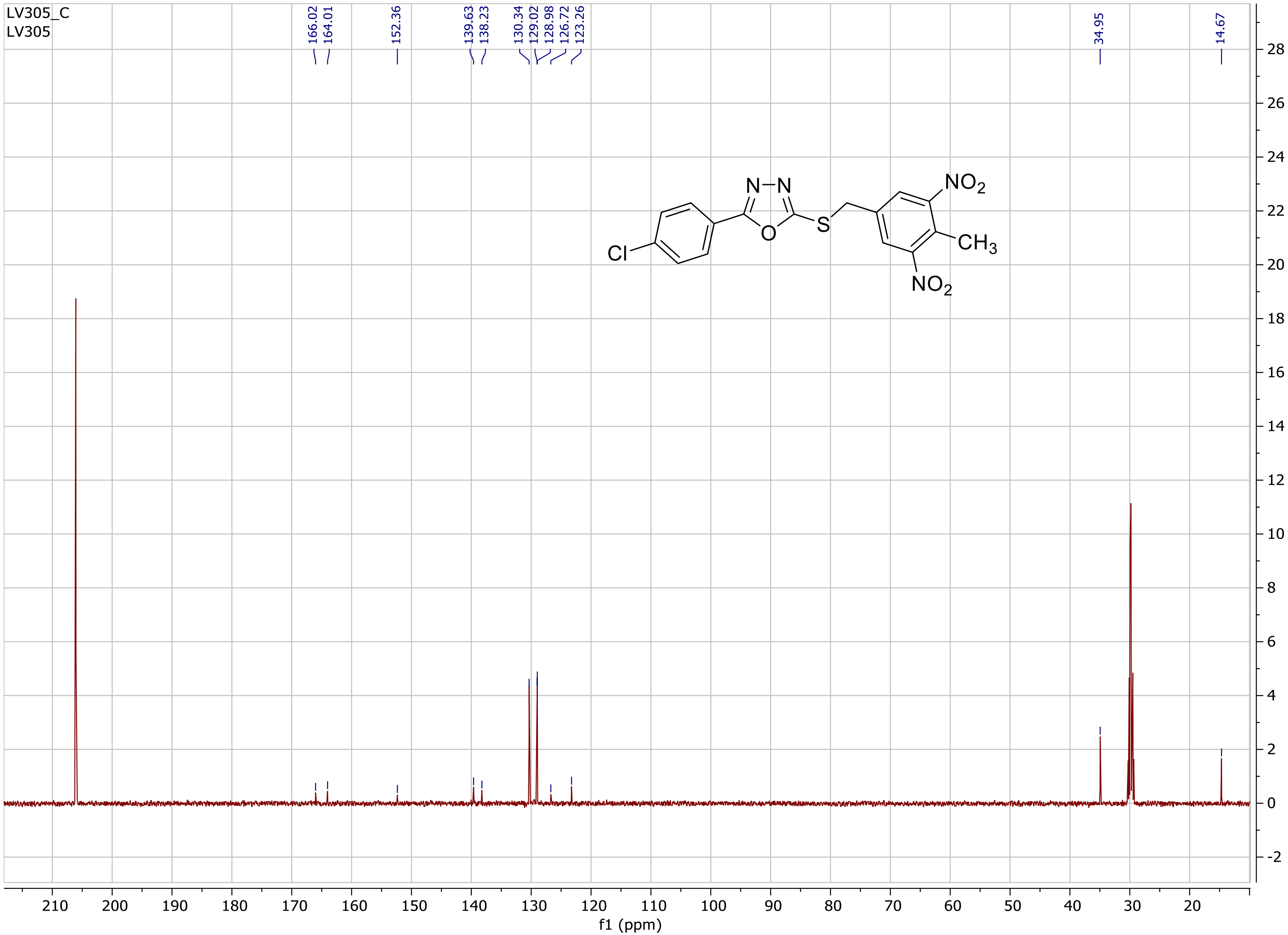

2-(4-Bromophenyl)-5-((4-methyl-3,5-dinitrobenzyl)sulfanyl)-1,3,4-oxadiazole (**80d**): ^{1}H NMR (600 MHz, DMSO-d_6)

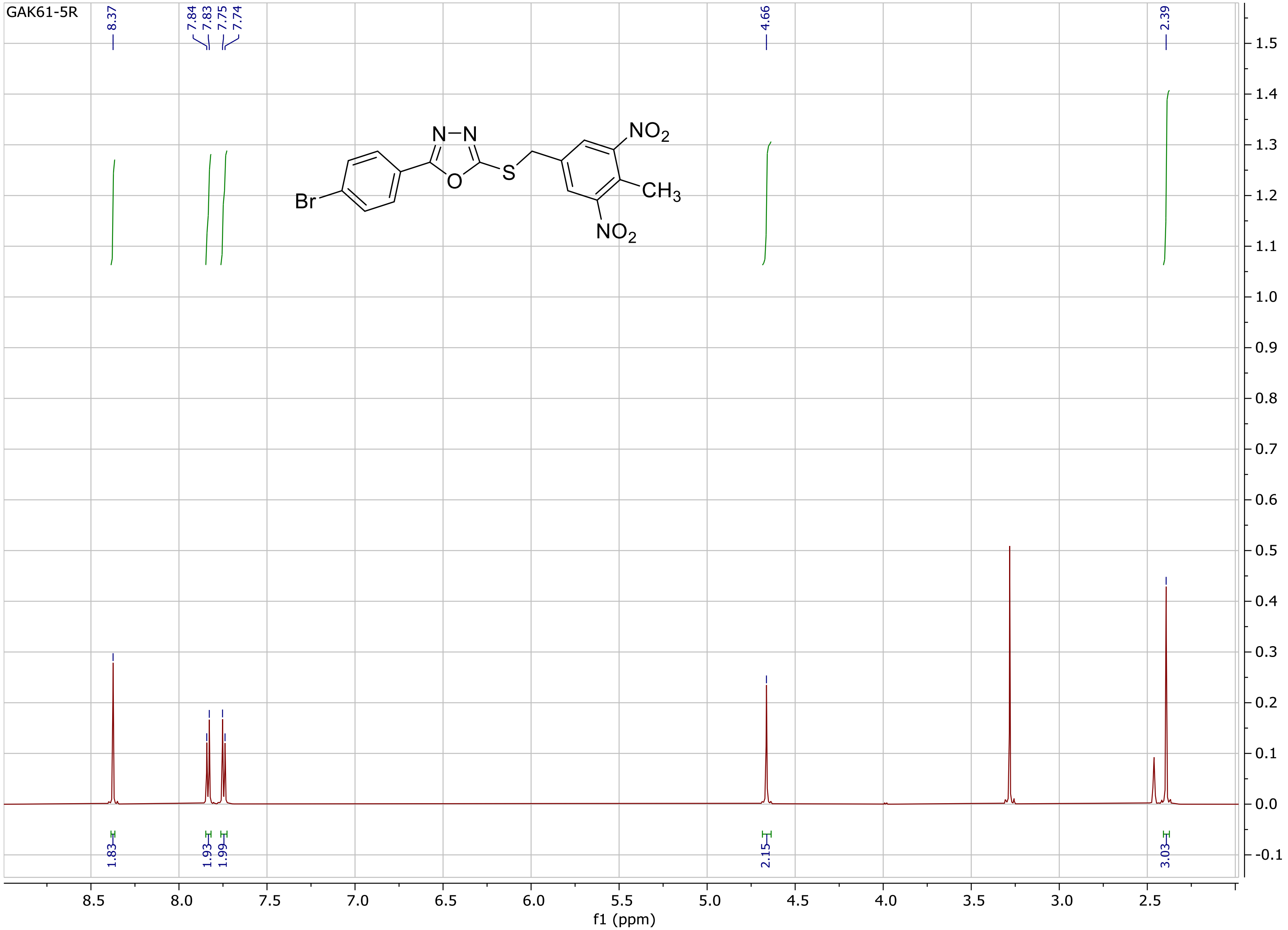

2-(4-Bromophenyl)-5-((4-methyl-3,5-dinitrobenzyl)sulfanyl)-1,3,4-oxadiazole (**80d**): ^{13}C NMR (126 MHz, DMSO-d_6)

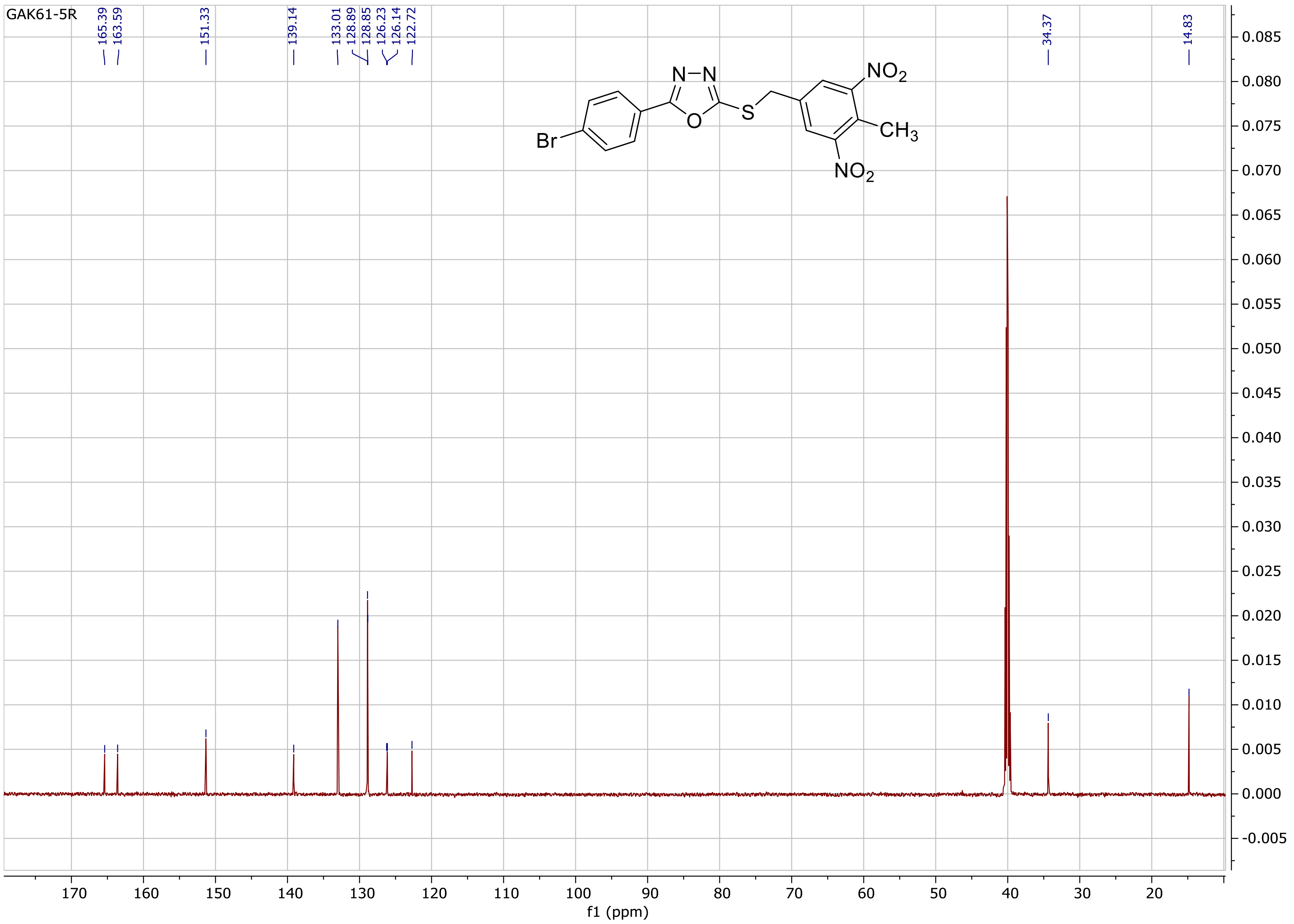

2-Cyclohexyl-5-((4-methyl-3,5-dinitrobenzyl)sulfanyl)-1,3,4-oxadiazole (**80e**): ^{1}H NMR (600 MHz, DMSO-d_6)

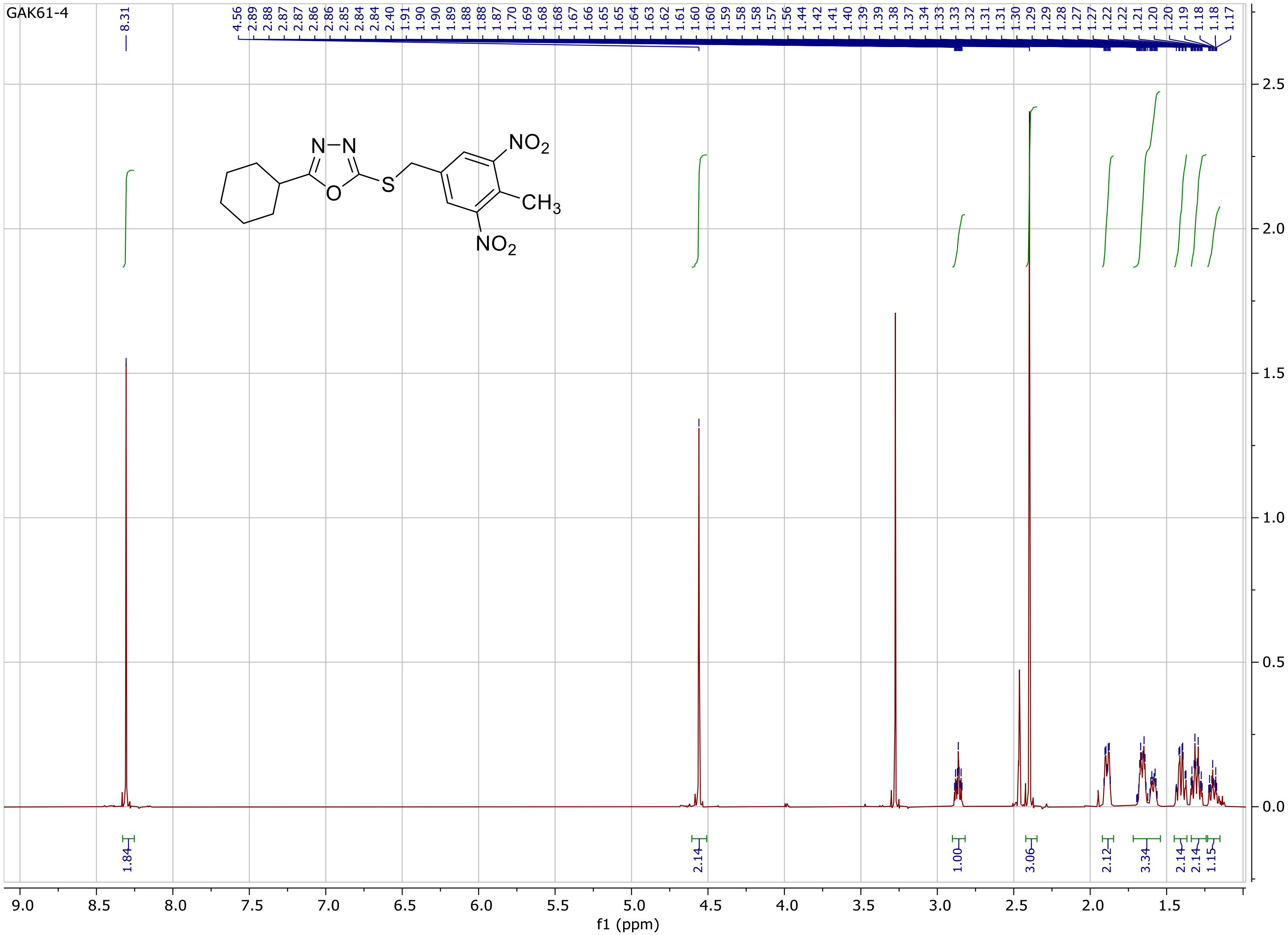

2-Cyclohexyl-5-((4-methyl-3,5-dinitrobenzyl)sulfanyl)-1,3,4-oxadiazole (**80e**): ^{13}C NMR (126 MHz, DMSO-d_6)

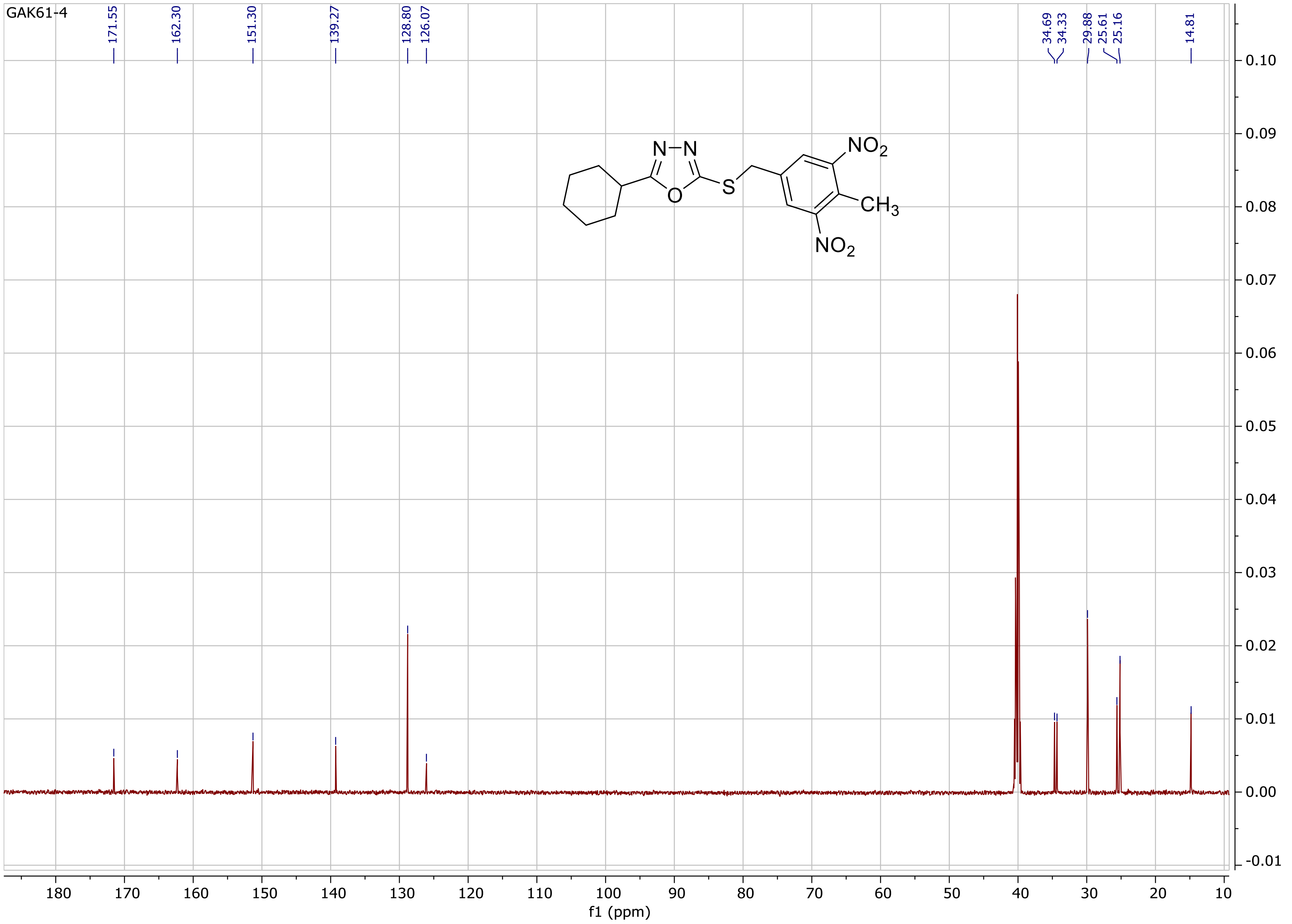

2-((2-Methyl-3,5-dinitrobenzyl)sulfanyl)-5-phenyl-1,3,4-oxadiazole (**81a**): ^{1}H NMR (500 MHz, $CDCl_3$)

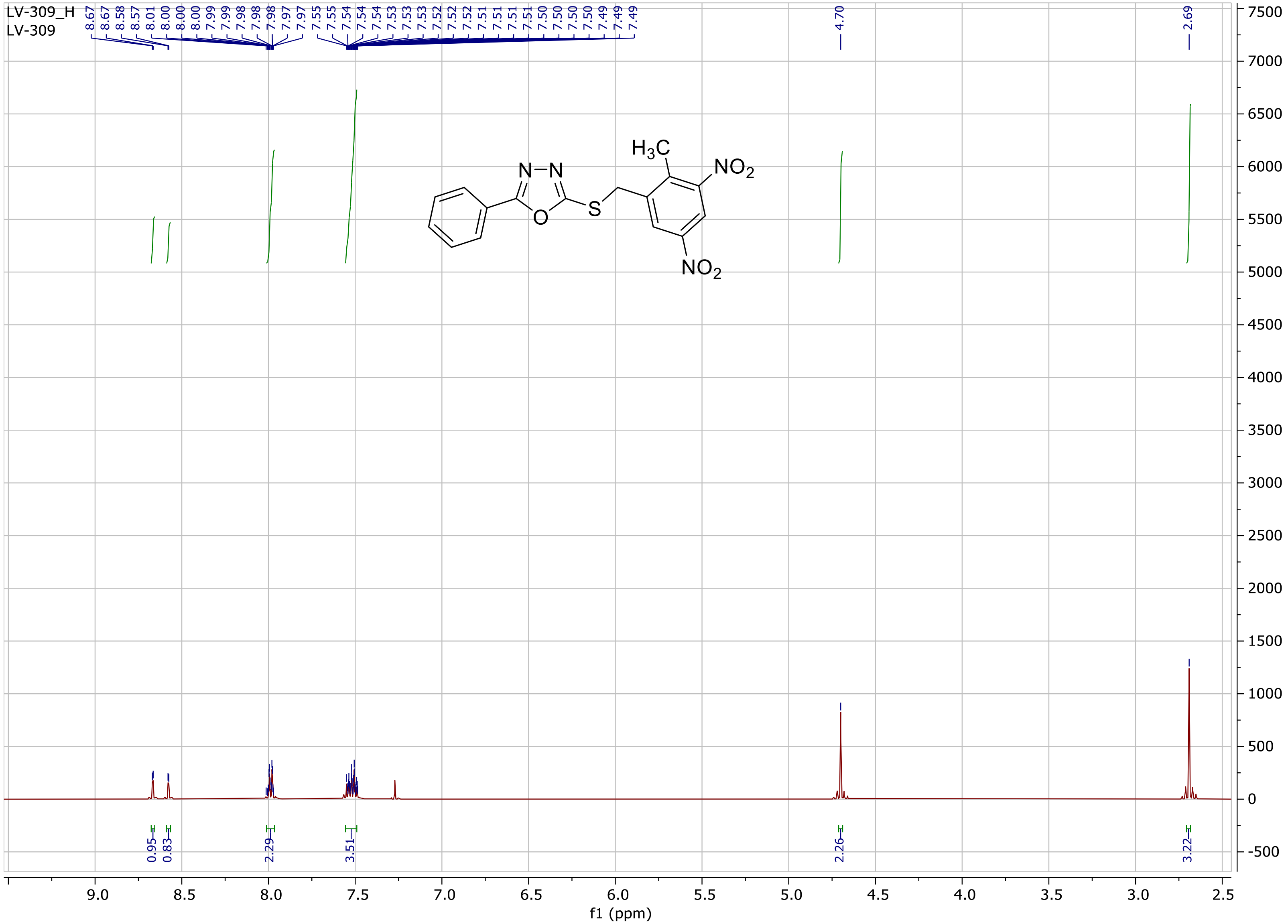

2-((2-Methyl-3,5-dinitrobenzyl)sulfanyl)-5-phenyl-1,3,4-oxadiazole (**81a**): ^{13}C NMR (126 MHz, $CDCl_3$)

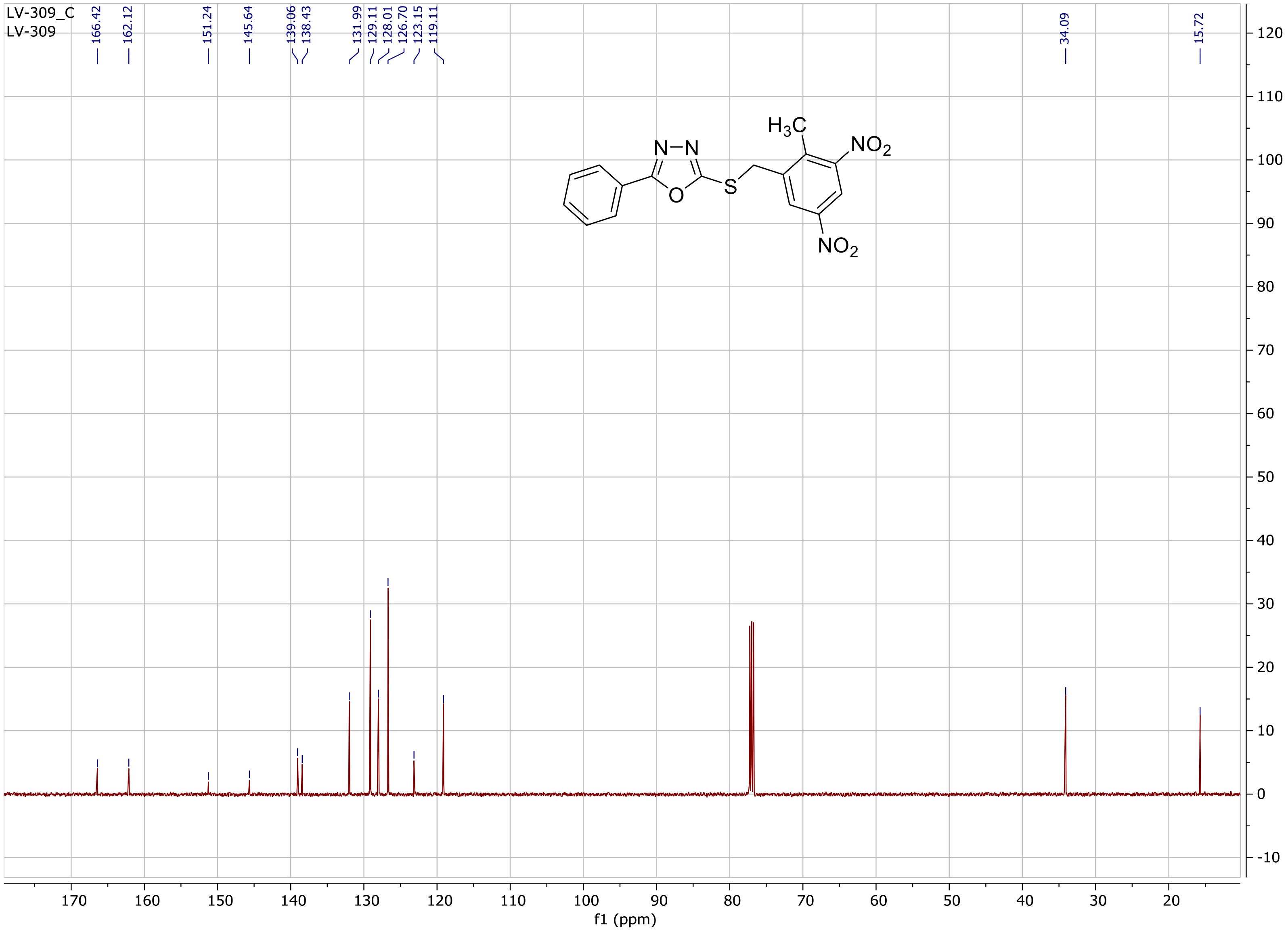

5-(4-Methoxyphenyl)-2-((2-methyl-3,5-dinitrobenzyl)sulfanyl)-1,3,4-oxadiazole (**81b**): ^{1}H NMR (500 MHz, Acetone-d_6)

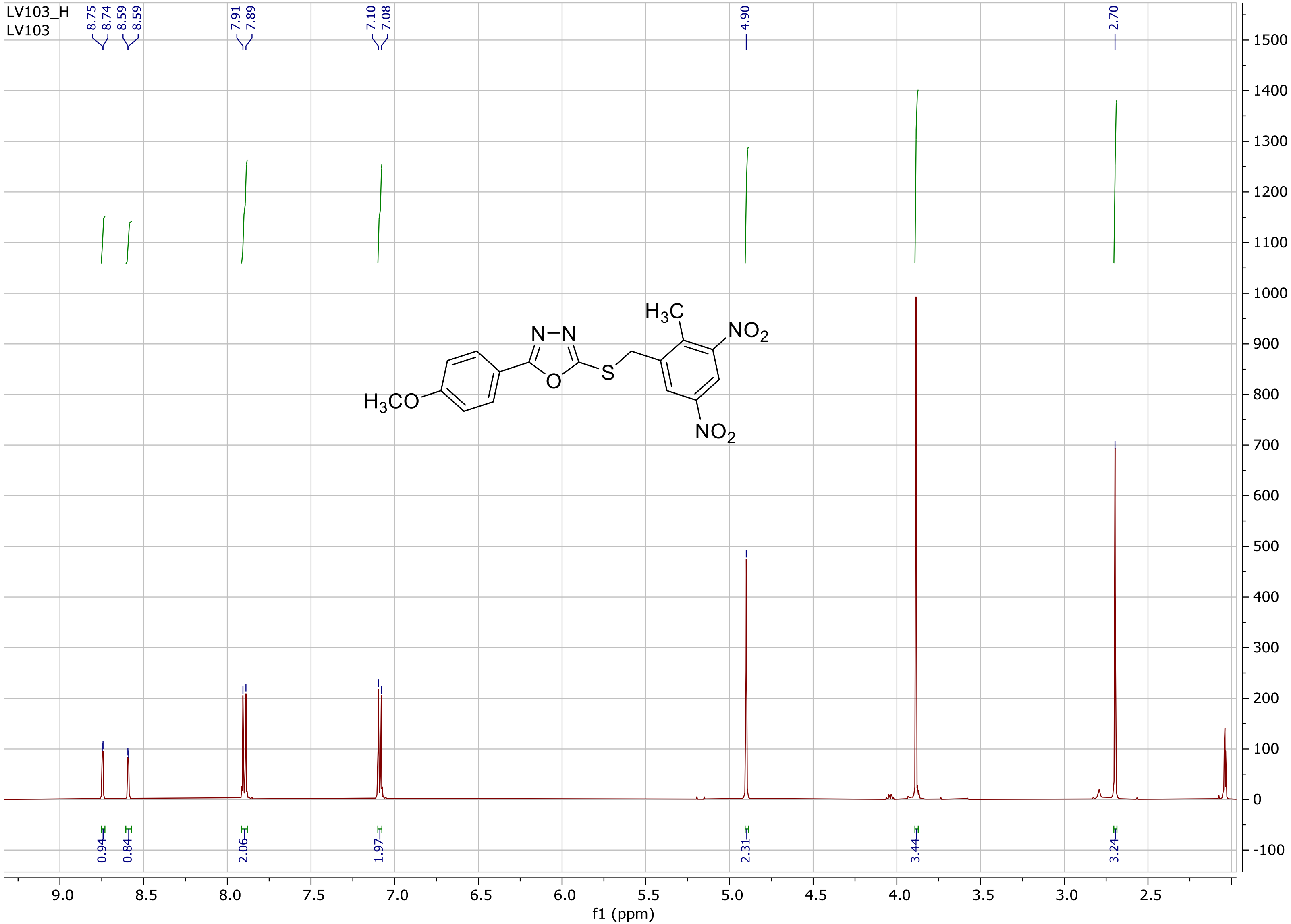

5-(4-Methoxyphenyl)-2-((2-methyl-3,5-dinitrobenzyl)sulfanyl)-1,3,4-oxadiazole (**81b**): ^{13}C NMR (126 MHz, Acetone-d_6)

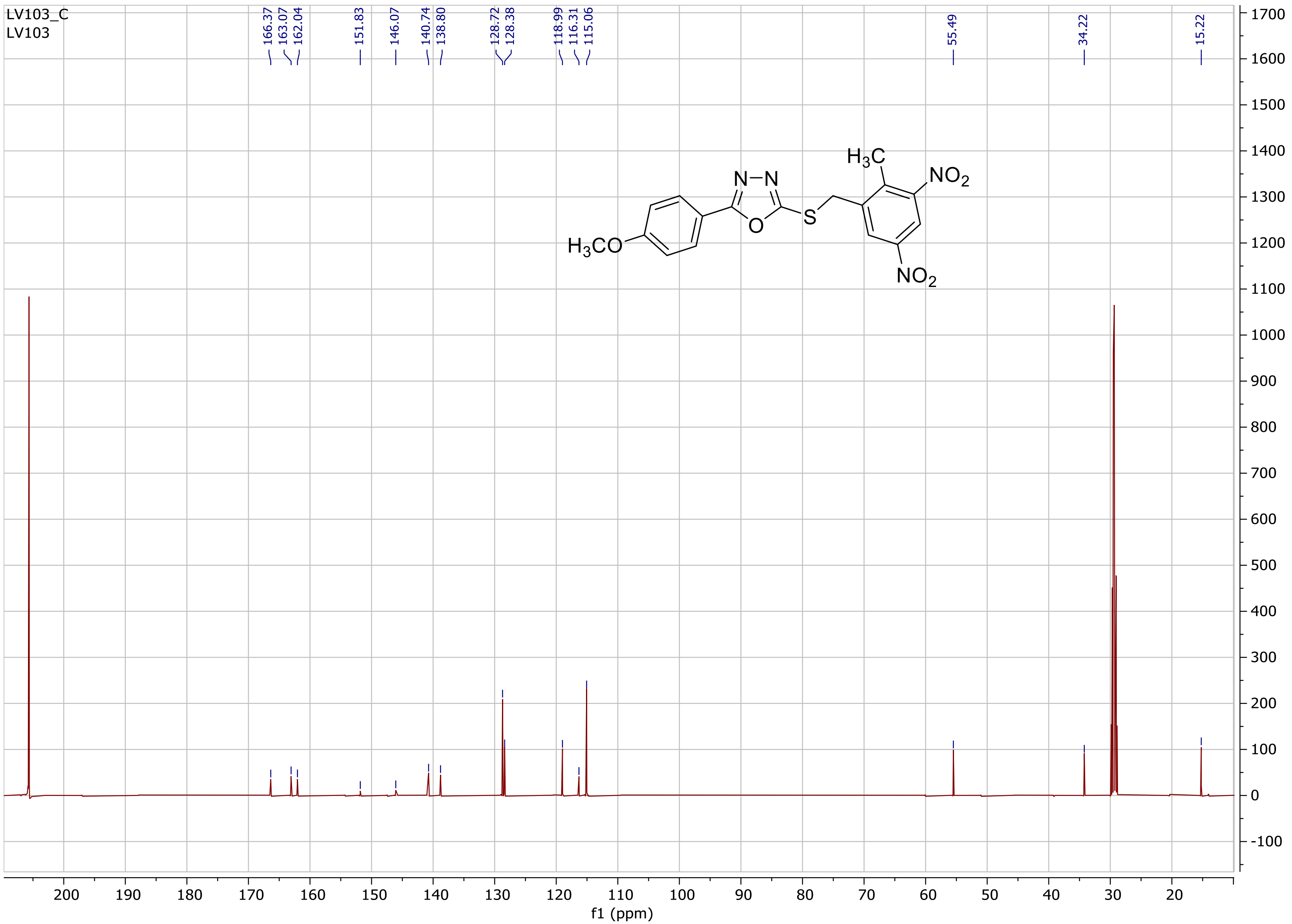

2-(4-Chlorophenyl)-5-((2-methyl-3,5-dinitrobenzyl)sulfanyl)-1,3,4-oxadiazole (**81c**): ^{1}H NMR (500 MHz, $CDCl_3$)

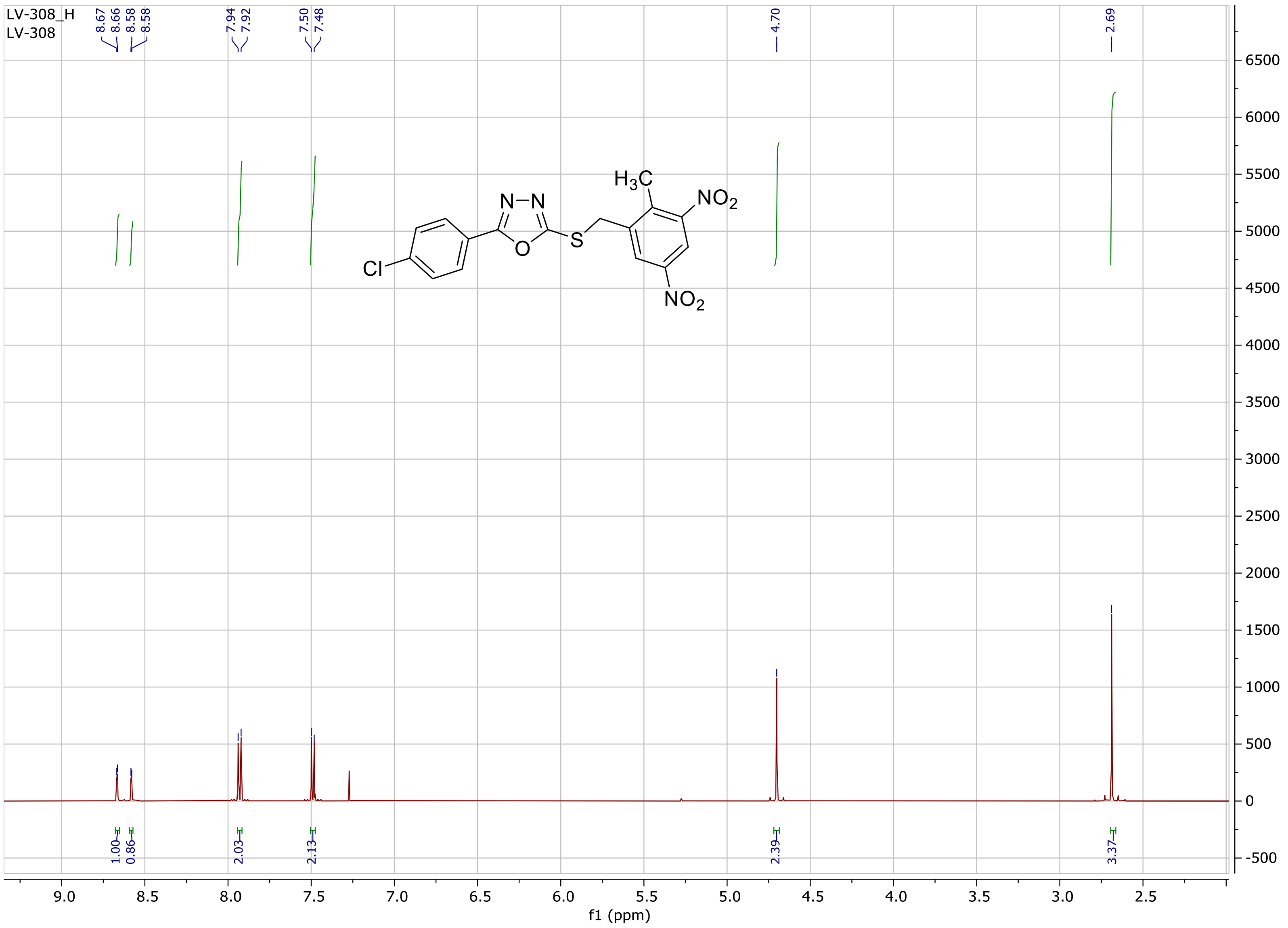

2-(4-Chlorophenyl)-5-((2-methyl-3,5-dinitrobenzyl)sulfanyl)-1,3,4-oxadiazole (**81c**): ^{13}C NMR (126 MHz, $CDCl_3$)

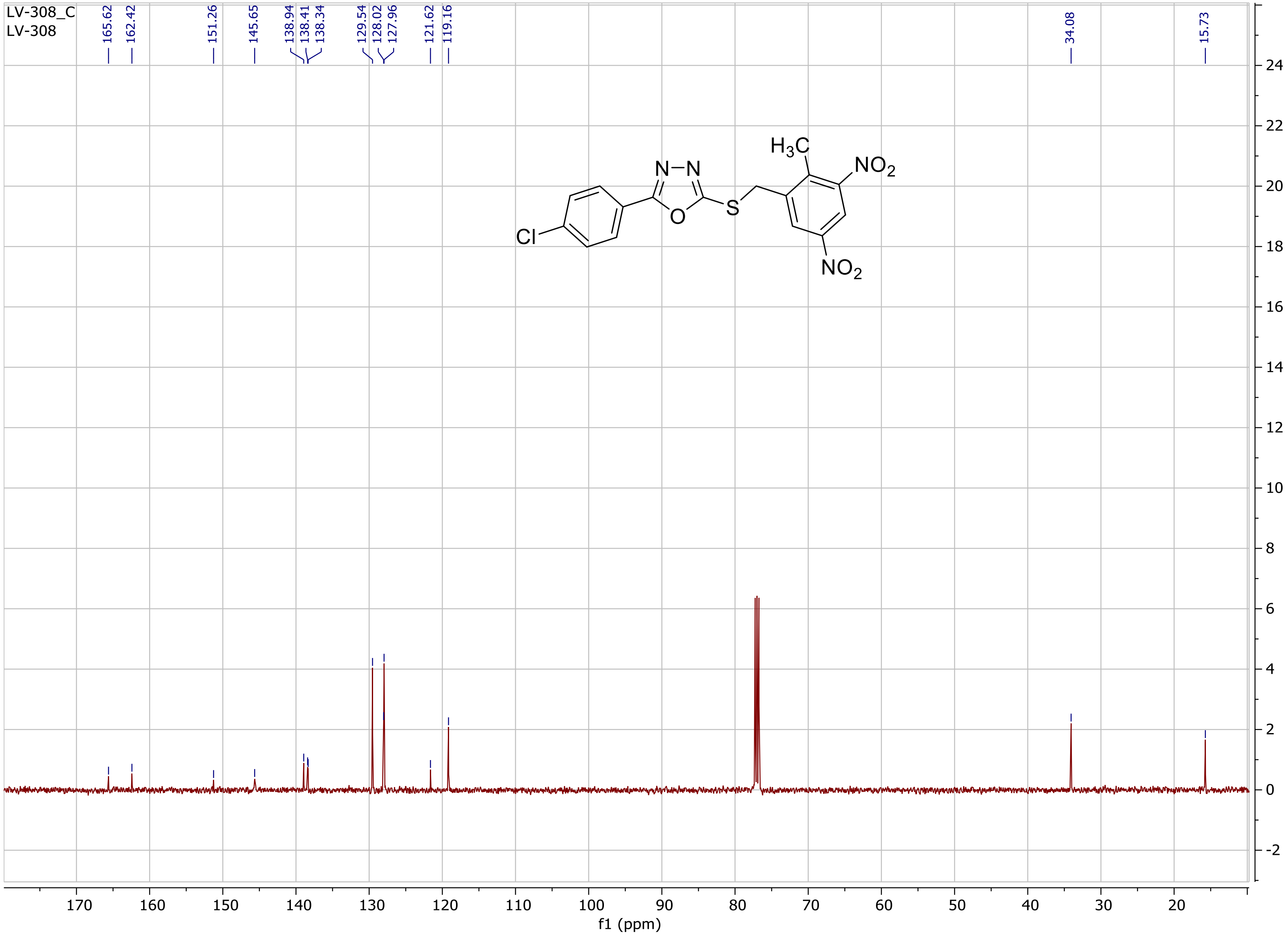

2-(4-Bromophenyl)-5-((2-methyl-3,5-dinitrobenzyl)sulfanyl)-1,3,4-oxadiazole (**81d**): ^{1}H NMR (500 MHz, $CDCl_3$)

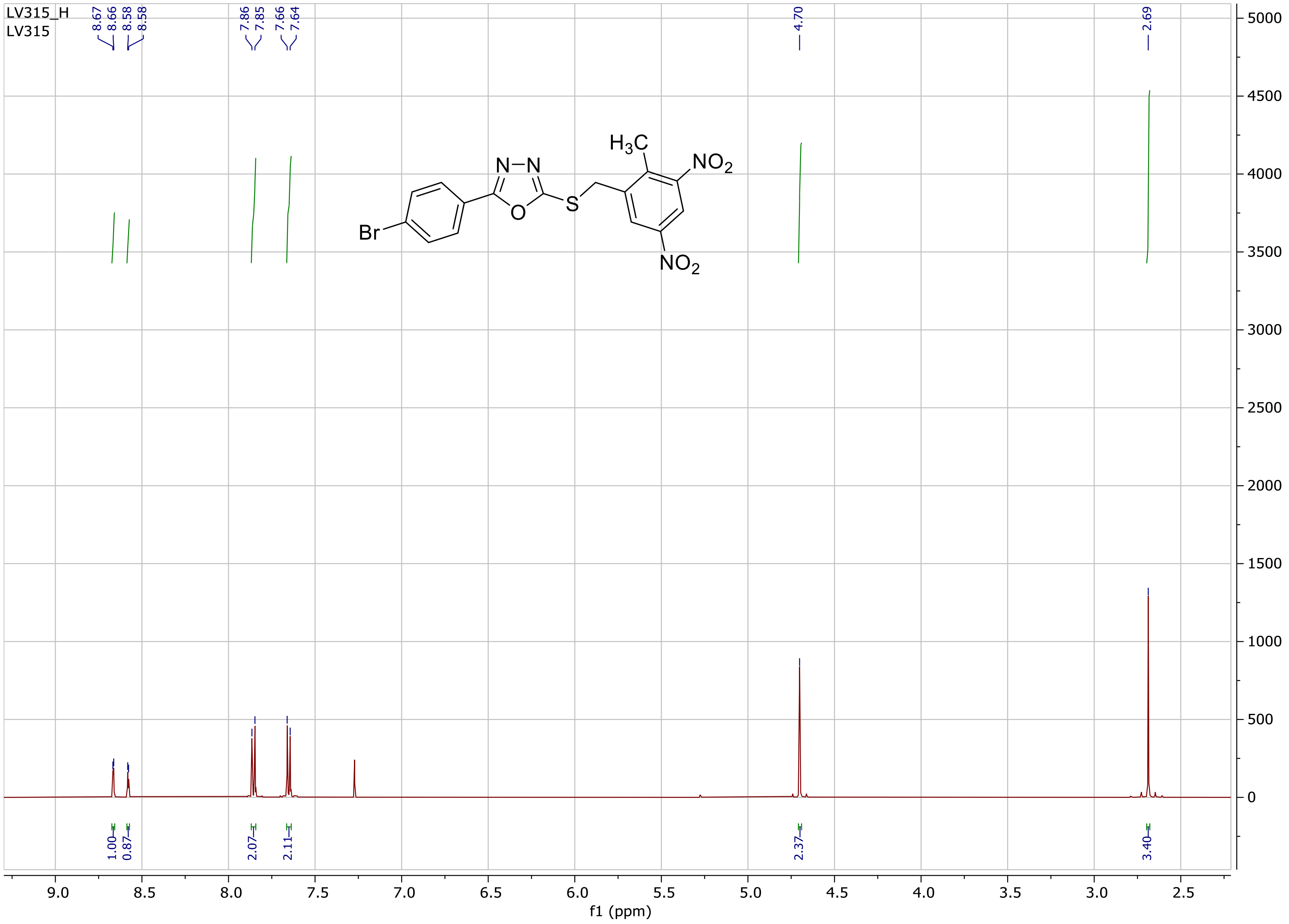

2-(4-Bromophenyl)-5-((2-methyl-3,5-dinitrobenzyl)sulfanyl)-1,3,4-oxadiazole (**81d**): ^{13}C NMR (126 MHz, CDCl$_3$)

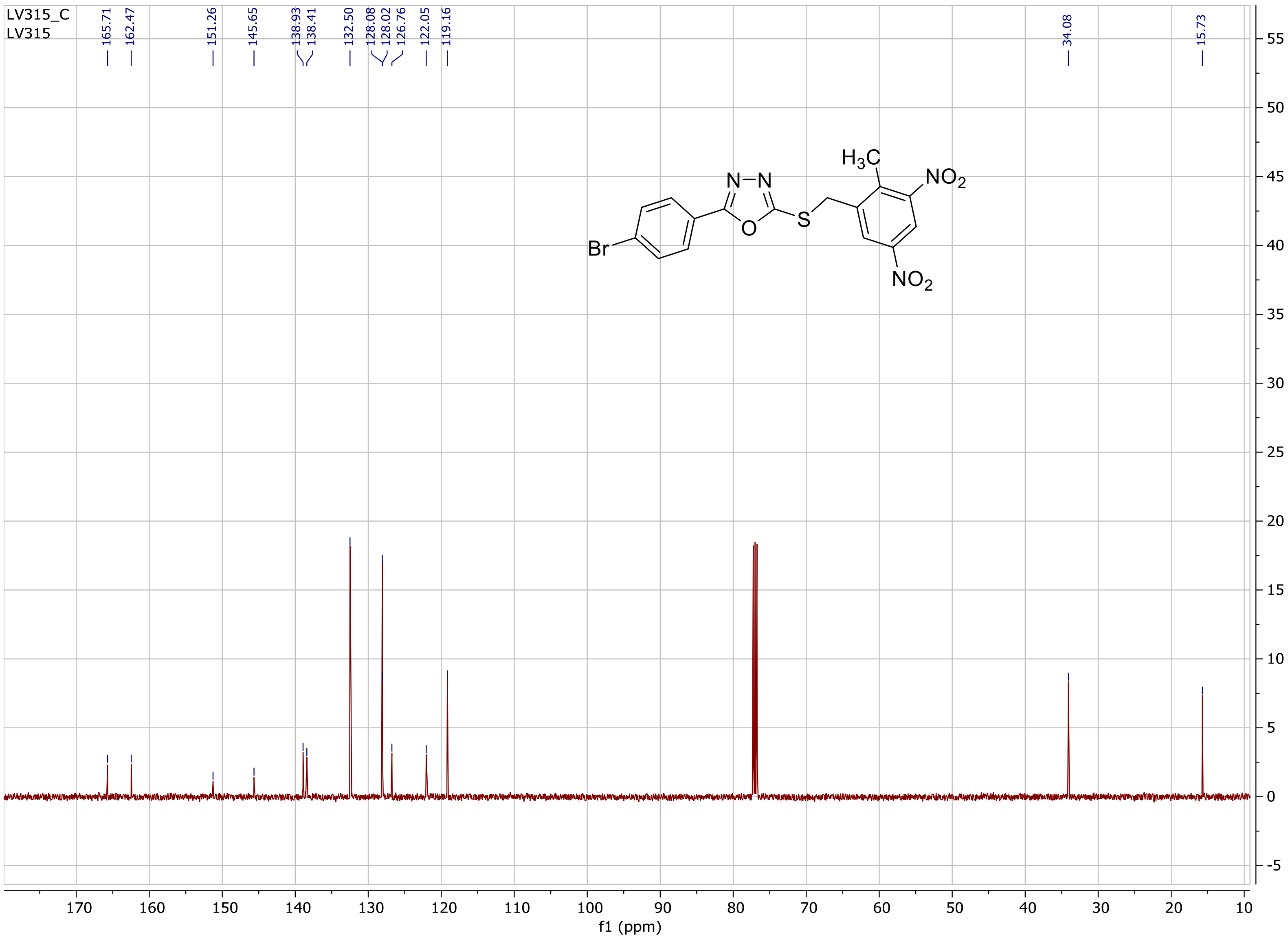

2-Cyclohexyl-5-((2-methyl-3,5-dinitrobenzyl)sulfanyl)-1,3,4-oxadiazole (**81e**): ^{1}H NMR (600 MHz, DMSO-d_6)

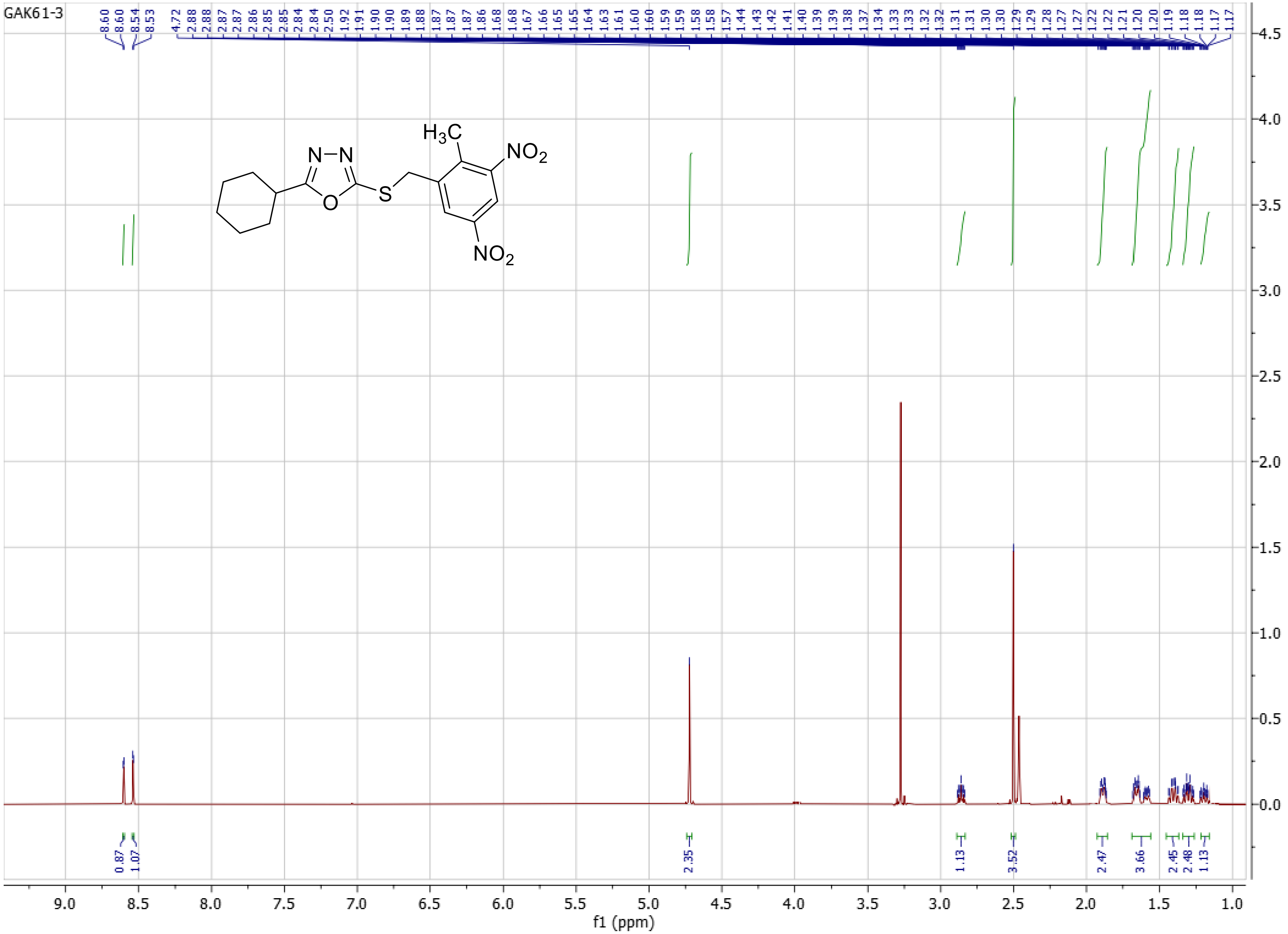

2-Cyclohexyl-5-((2-methyl-3,5-dinitrobenzyl)sulfanyl)-1,3,4-oxadiazole (**81e**): ^{13}C NMR (151 MHz, DMSO-*d*$_6$)

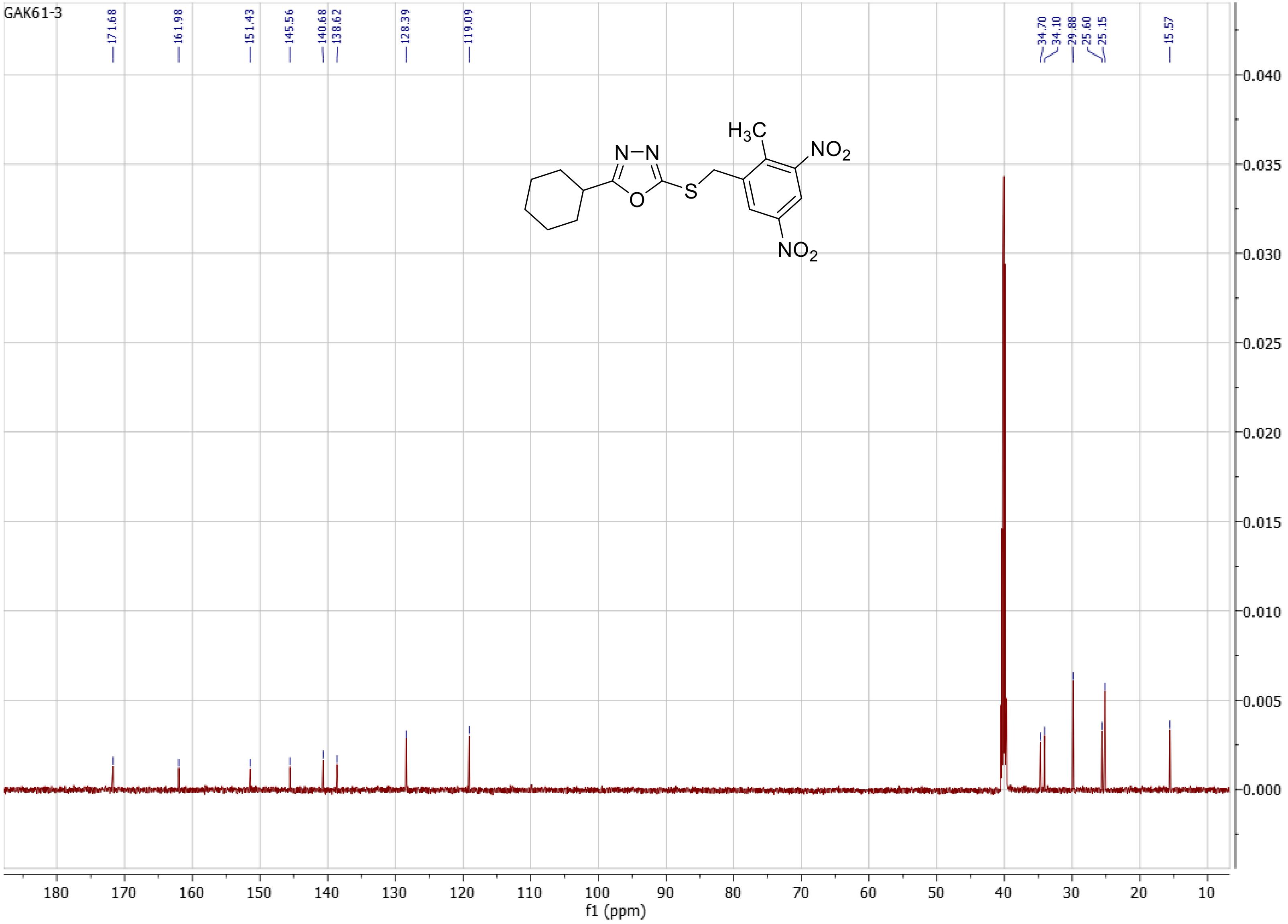

2-Cyclohexyl-5-((2-methyl-3,5-dinitrobenzyl)sulfanyl)-1,3,4-oxadiazole (**81e**):

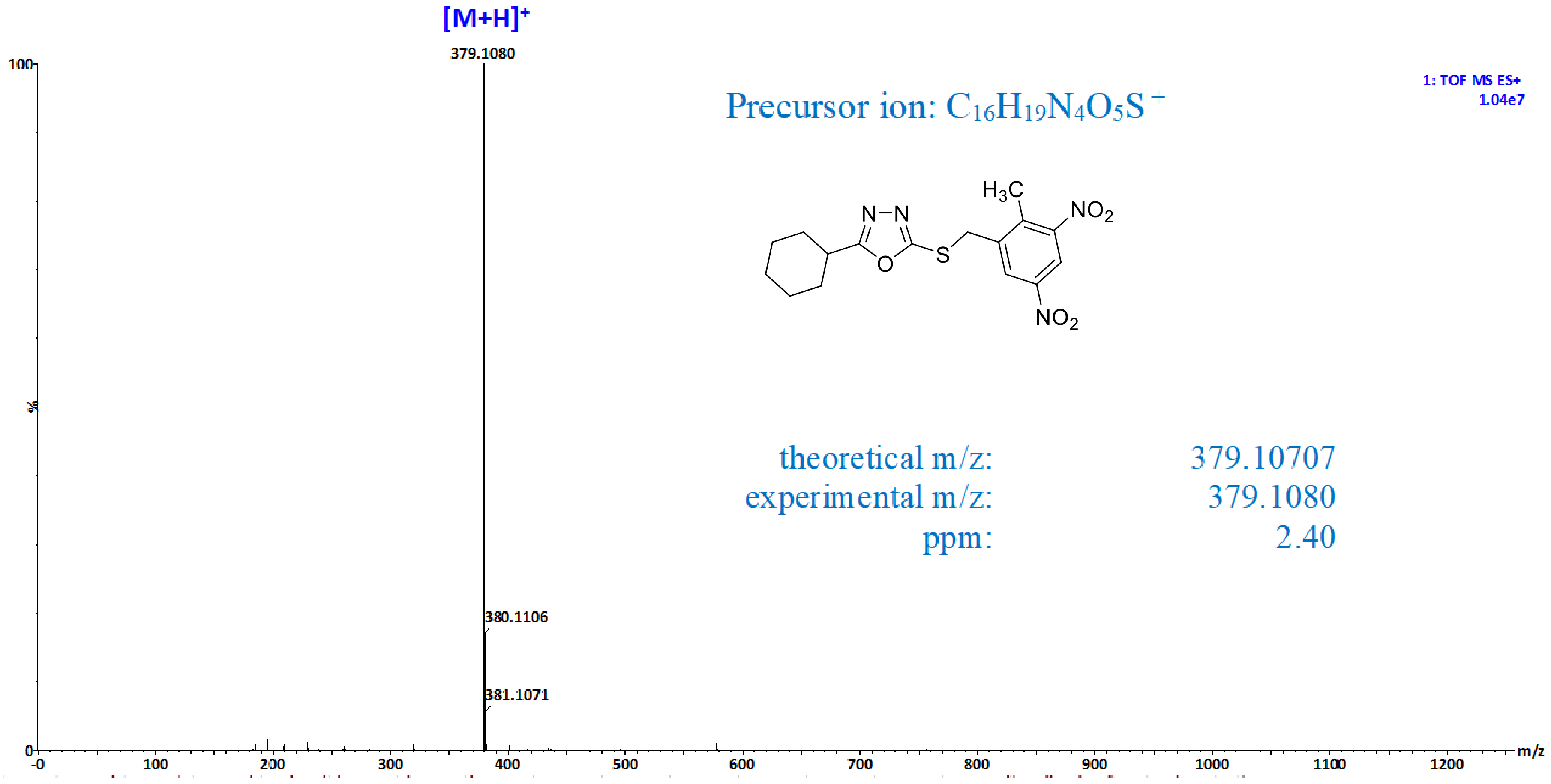

2-((5-Nitropyridin-3-yl)methylsulfanyl)-5-phenyl-1,3,4-oxadiazole (**82a**): ^{1}H NMR (600 MHz, DMSO-d_6)

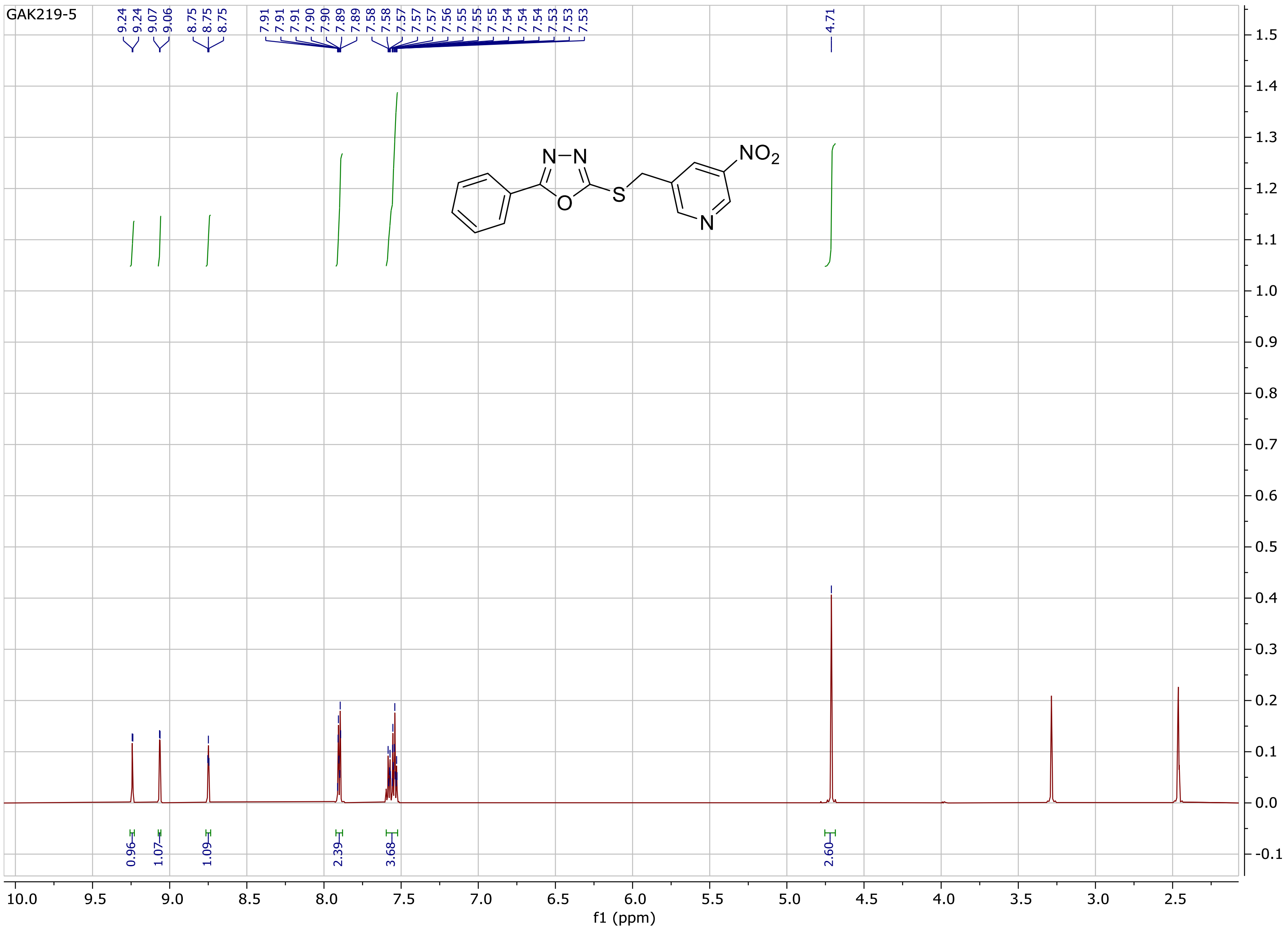

2-((5-Nitropyridin-3-yl)methylsulfanyl)-5-phenyl-1,3,4-oxadiazole (**82a**): ^{13}C NMR (151 MHz, DMSO-d_6)

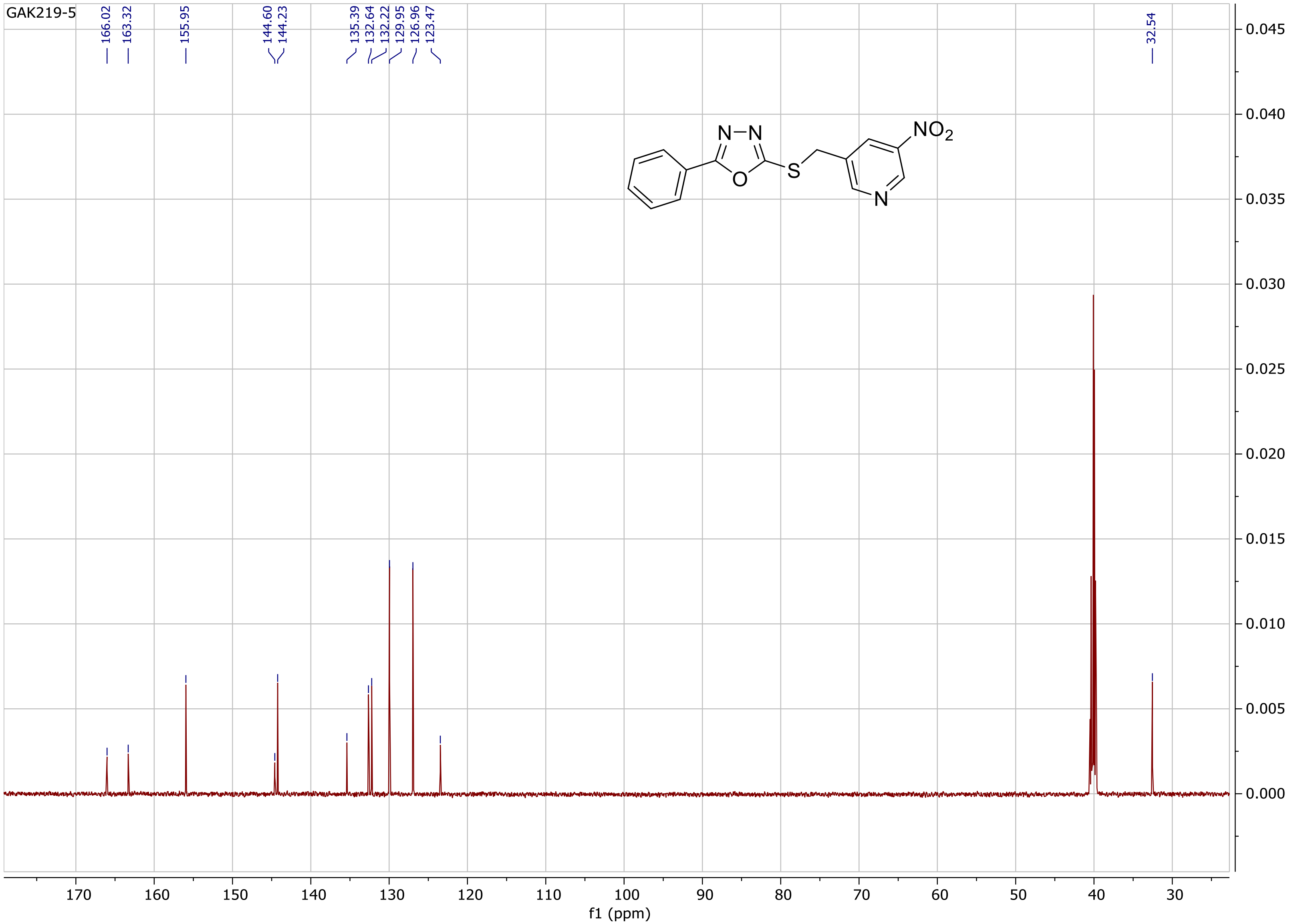

2-(4-Methoxyphenyl)-5-((5-nitropyridin-3-yl)methylsulfanyl)-1,3,4-oxadiazole (**82b**): ^{1}H NMR (600 MHz, DMSO-*d*$_6$)

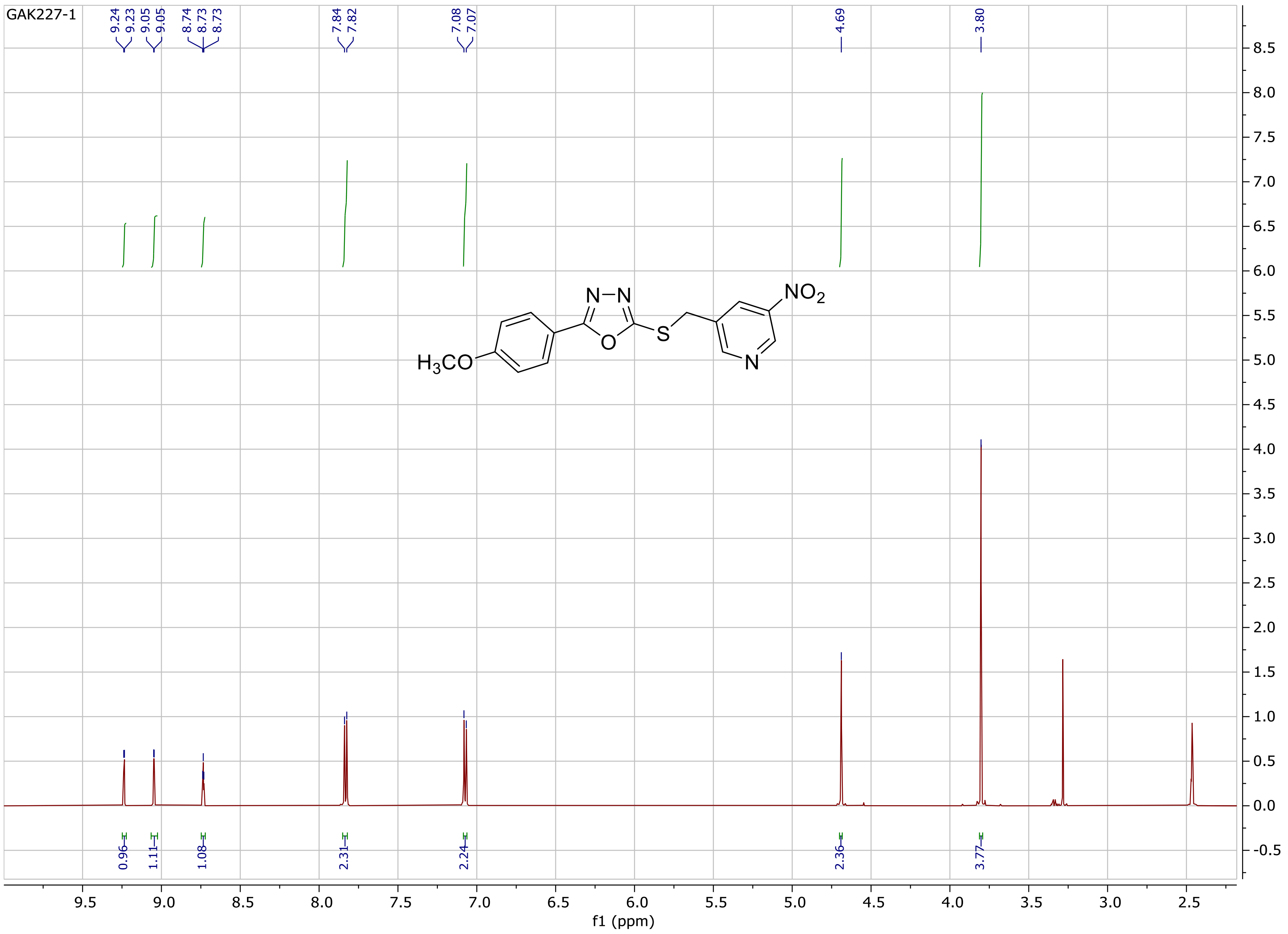

2-(4-Methoxyphenyl)-5-((5-nitropyridin-3-yl)methylsulfanyl)-1,3,4-oxadiazole (**82b**): ^{13}C NMR (151 MHz, DMSO-d_6)

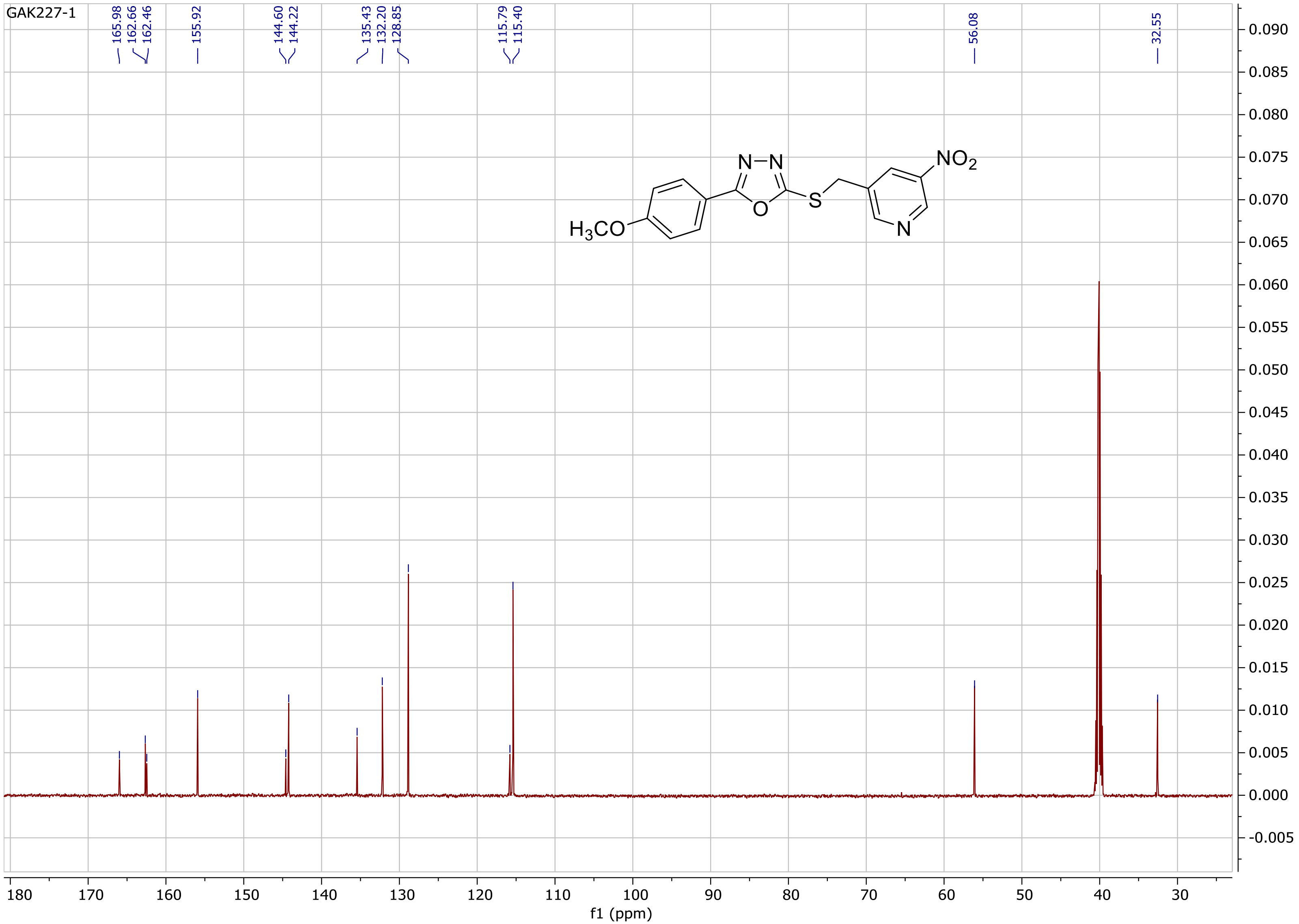

2-(4-Chlorophenyl)-5-((5-nitropyridin-3-yl)methylsulfanyl)-1,3,4-oxadiazole (**82c**): ^{1}H NMR (600 MHz, DMSO-d_6)

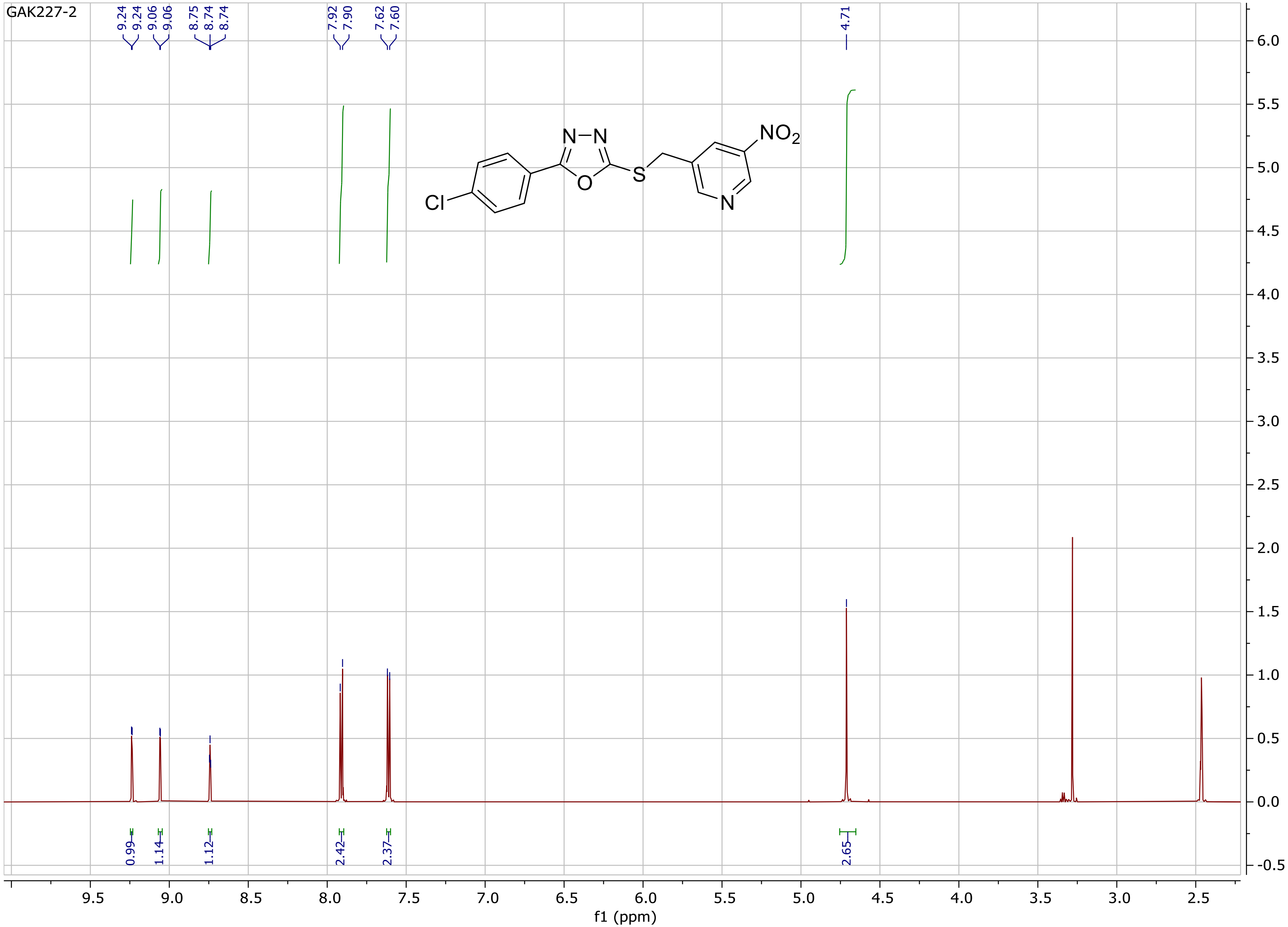

2-(4-Chlorophenyl)-5-((5-nitropyridin-3-yl)methylsulfanyl)-1,3,4-oxadiazole (**82c**): ^{13}C NMR (151 MHz, DMSO-d_6)

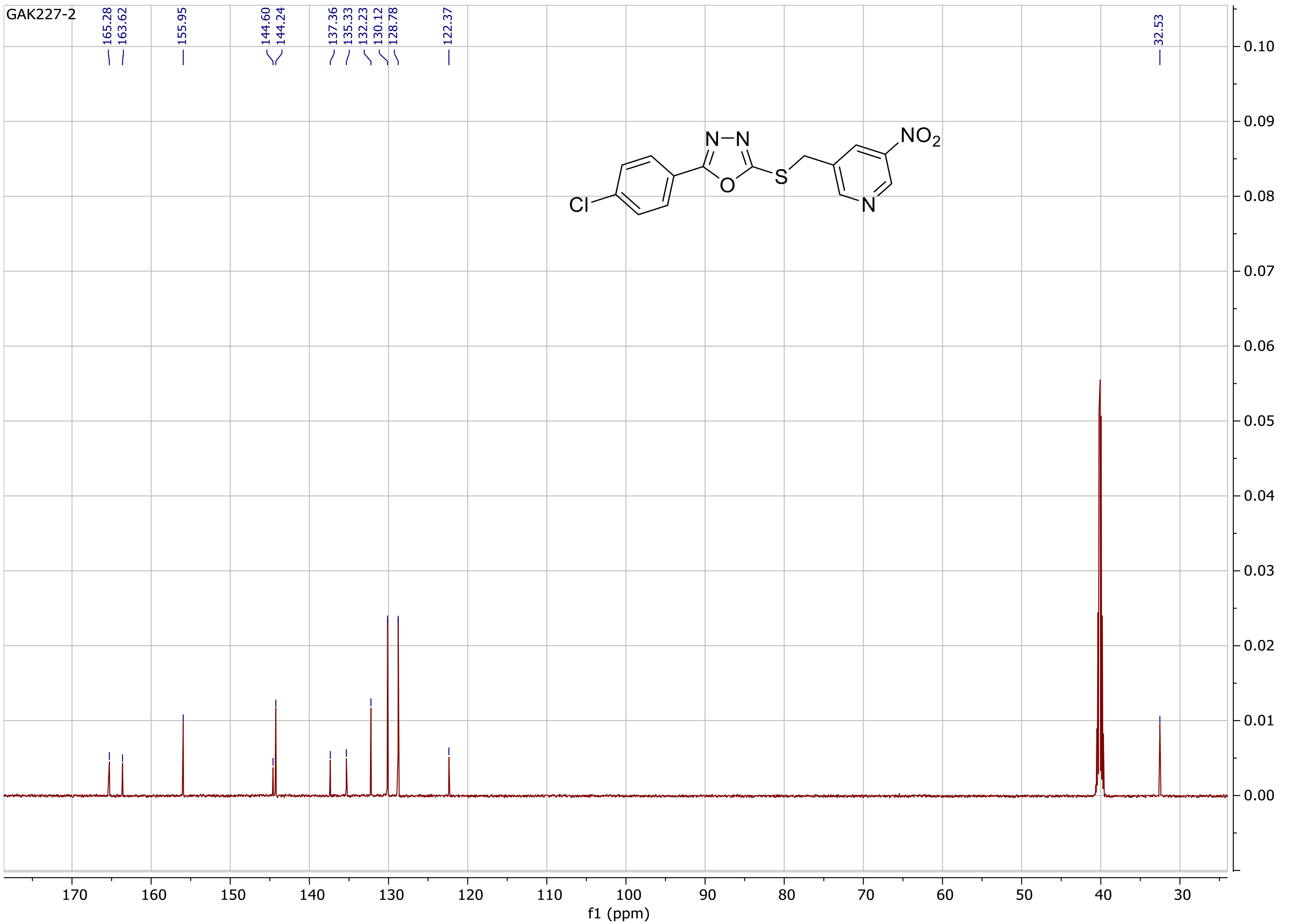

2-(4-Bromophenyl)-5-((5-nitropyridin-3-yl)methylsulfanyl)-1,3,4-oxadiazole (**82d**): ^{1}H NMR (600 MHz, DMSO-d_6)

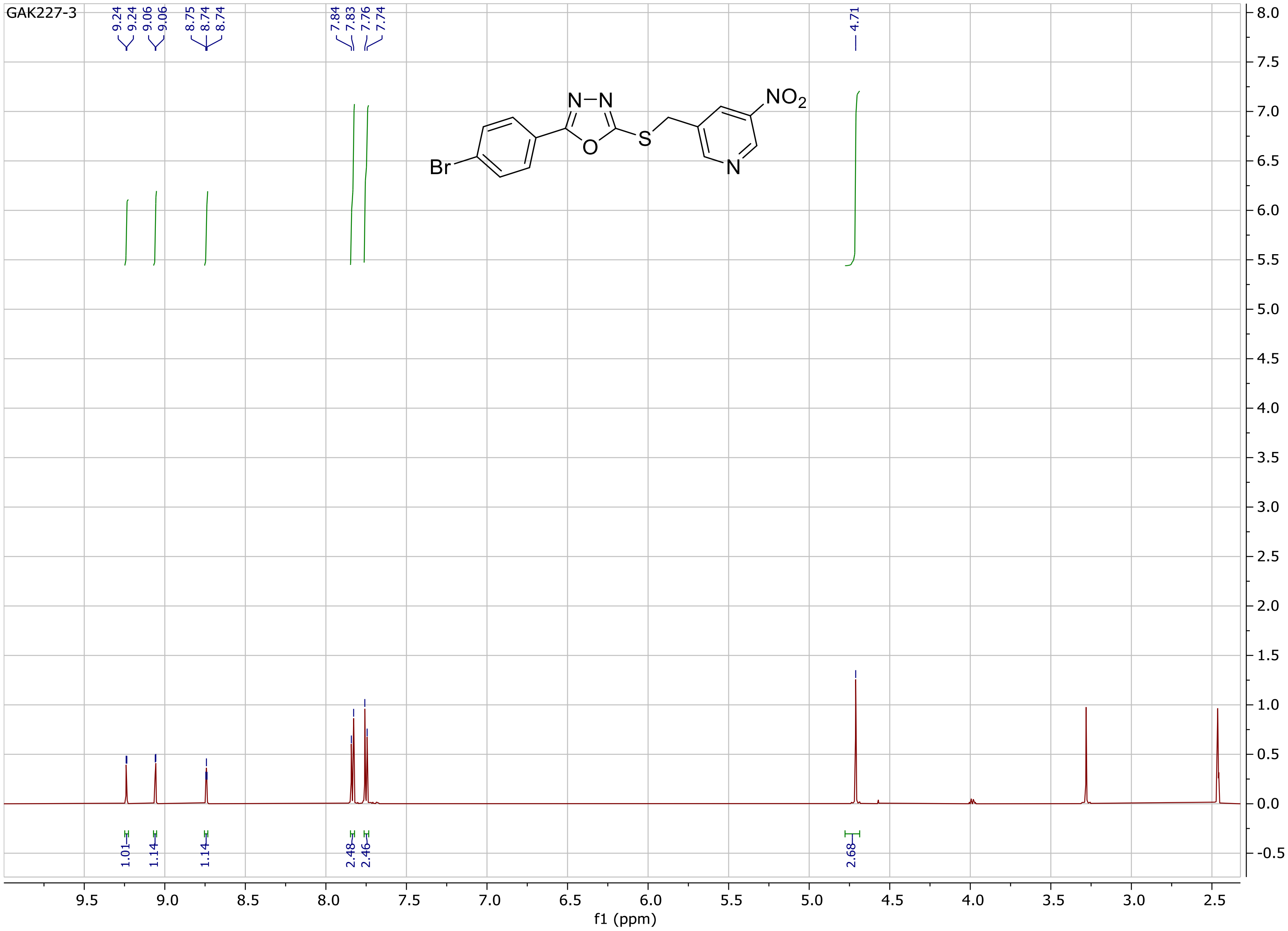

2-(4-Bromophenyl)-5-((5-nitropyridin-3-yl)methylsulfanyl)-1,3,4-oxadiazole (**82d**): ^{13}C NMR (151 MHz, DMSO-d_6)

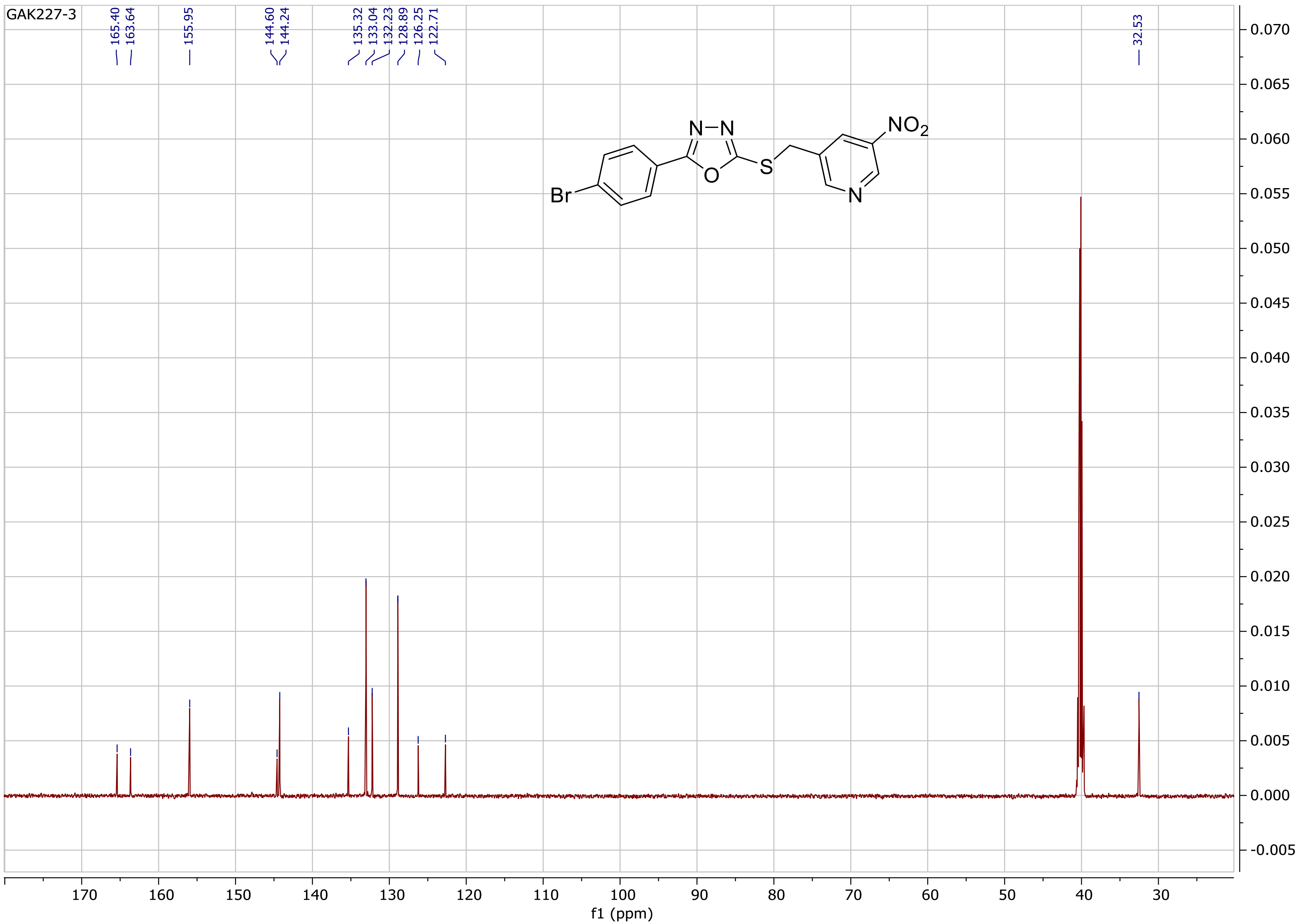

2-Cyclohexyl-5-((5-nitropyridin-3-yl)methylsulfanyl)-1,3,4-oxadiazole (**82e**): ^{1}H NMR (600 MHz, DMSO-d_6)

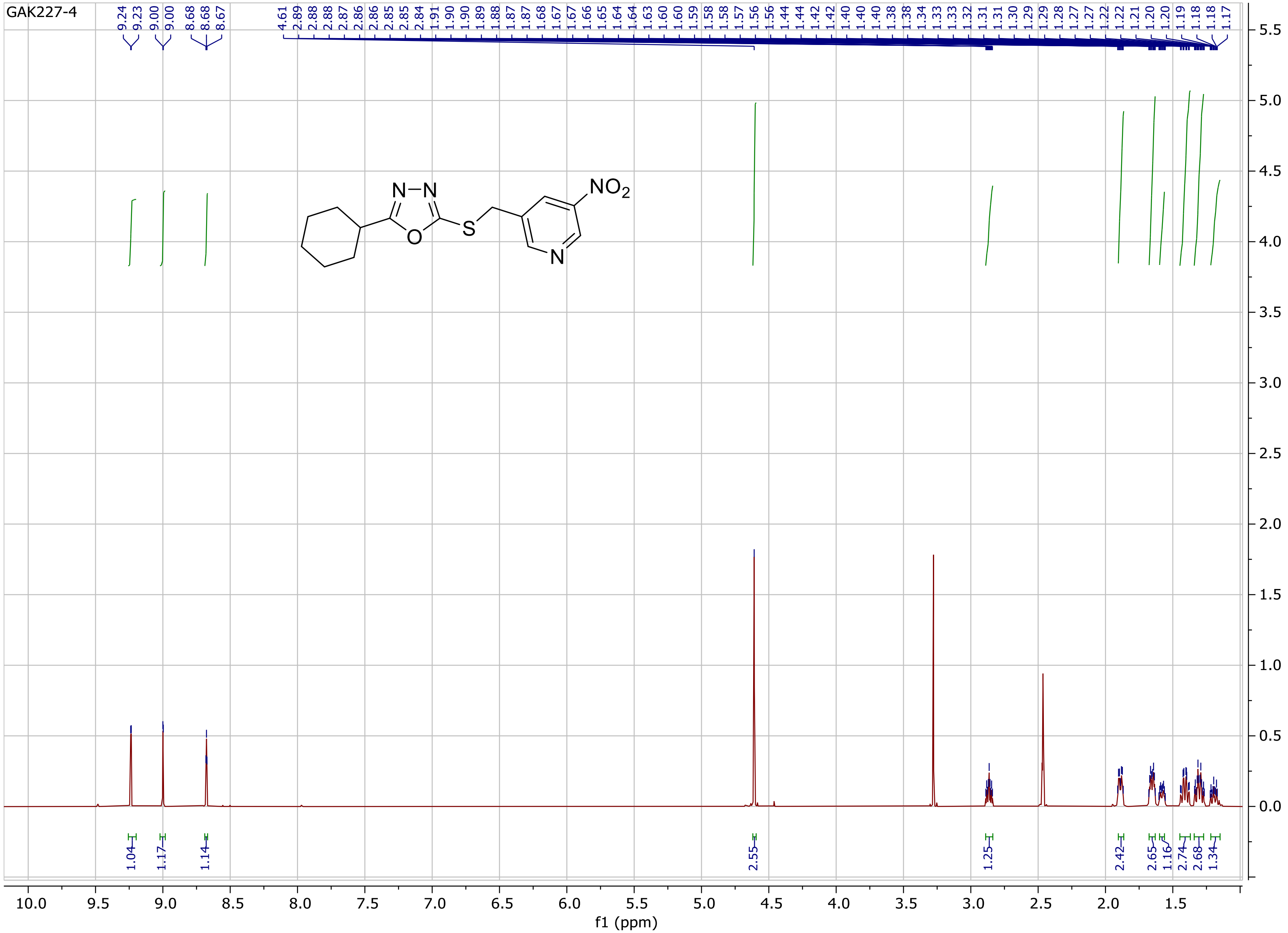

2-Cyclohexyl-5-((5-nitropyridin-3-yl)methylsulfanyl)-1,3,4-oxadiazole (**82e**): ^{13}C NMR (151 MHz, DMSO-d_6)

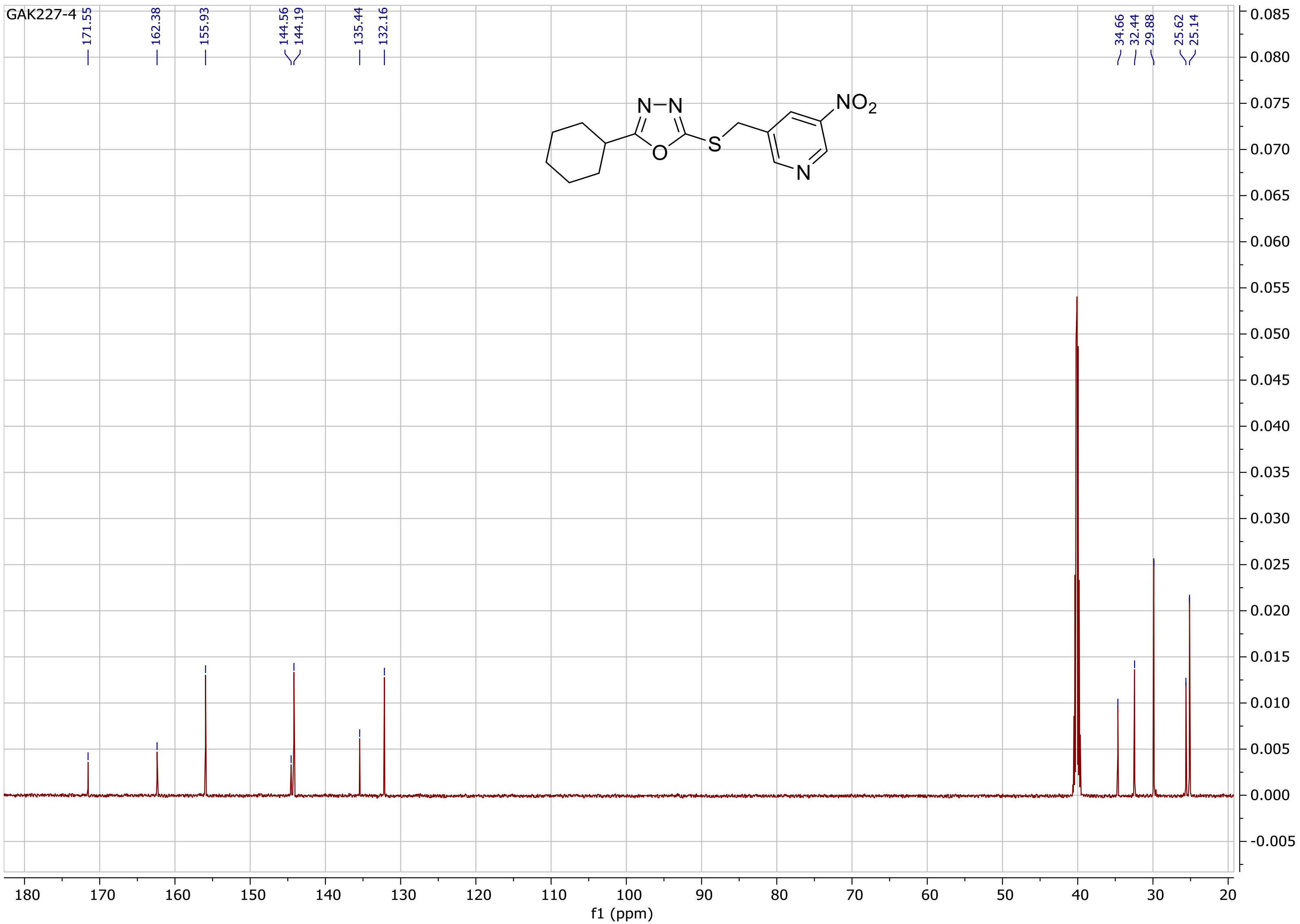

5-((5-Nitrofuran-2-yl)methylsulfanyl)-5-phenyl-1,3,4-oxadiazole (**83a**): ^{1}H NMR (500 MHz, Acetone-d_6)

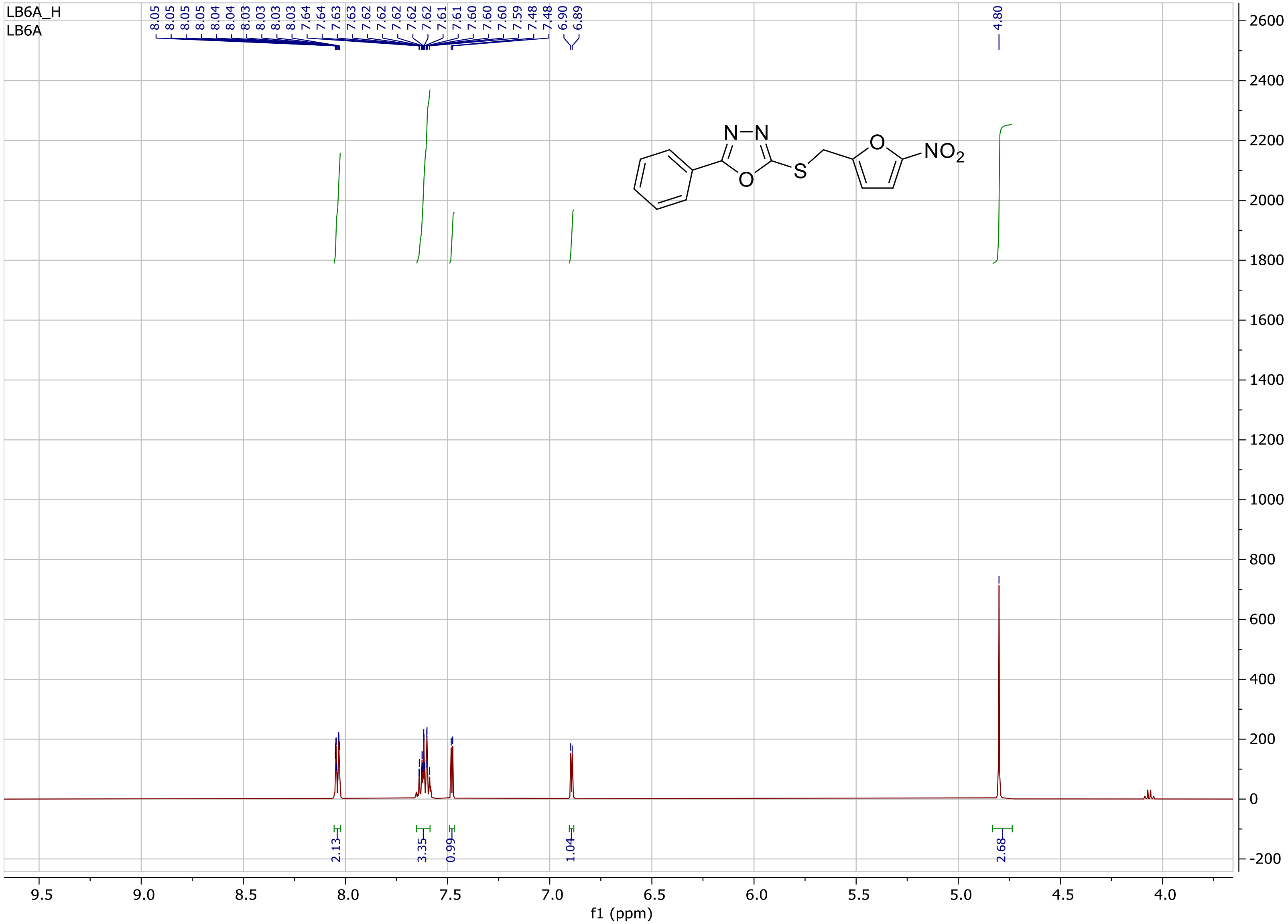

5-((5-Nitrofuran-2-yl)methylsulfanyl)-5-phenyl-1,3,4-oxadiazole (**83a**): ^{13}C NMR (126 MHz, Acetone-d_6)

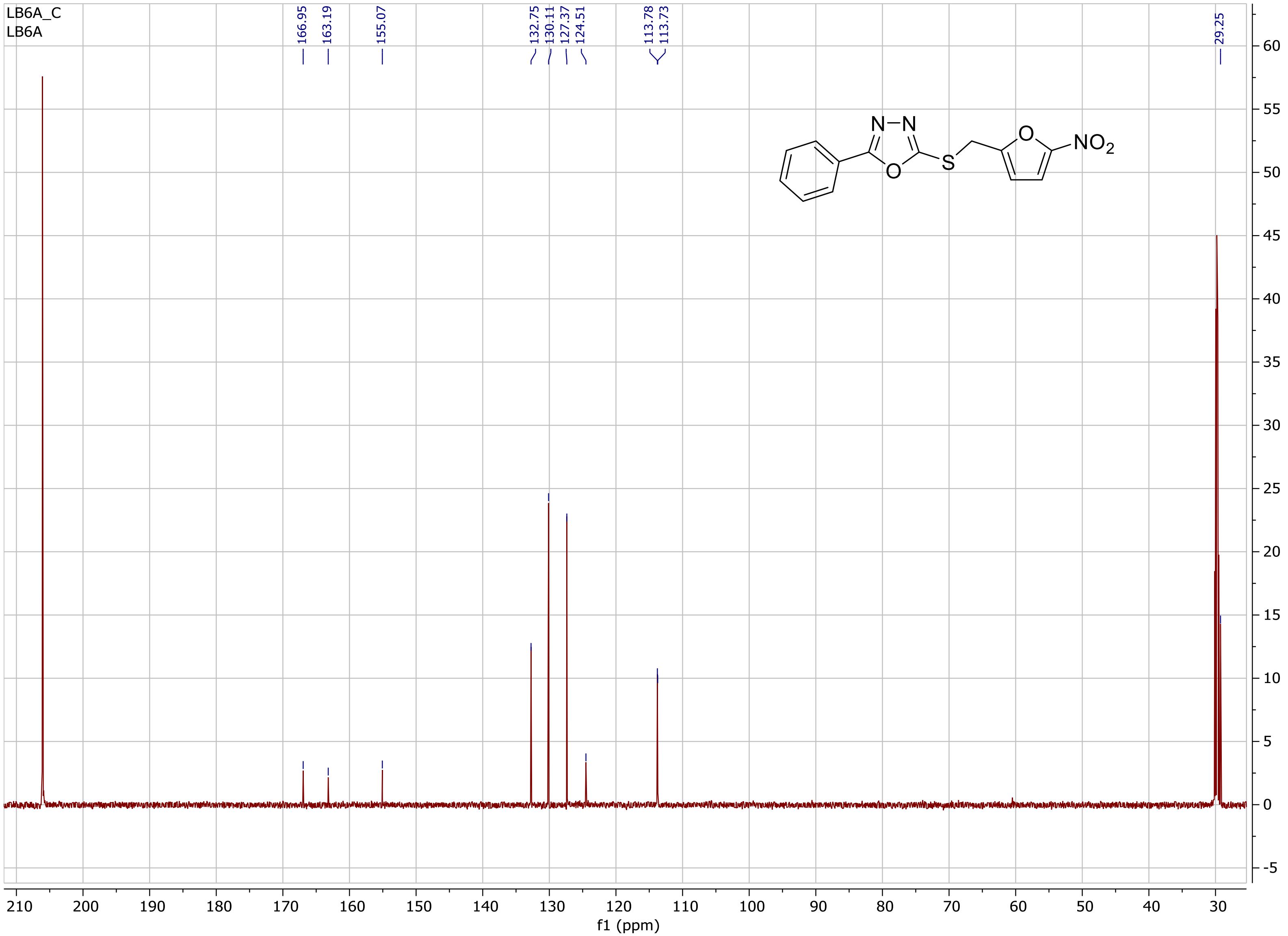

2-(4-Methoxyphenyl)-5-((5-nitrofuran-2-yl)methylsulfanyl)-1,3,4-oxadiazole (**83b**): ^{1}H NMR (500 MHz, Acetone-d_6)

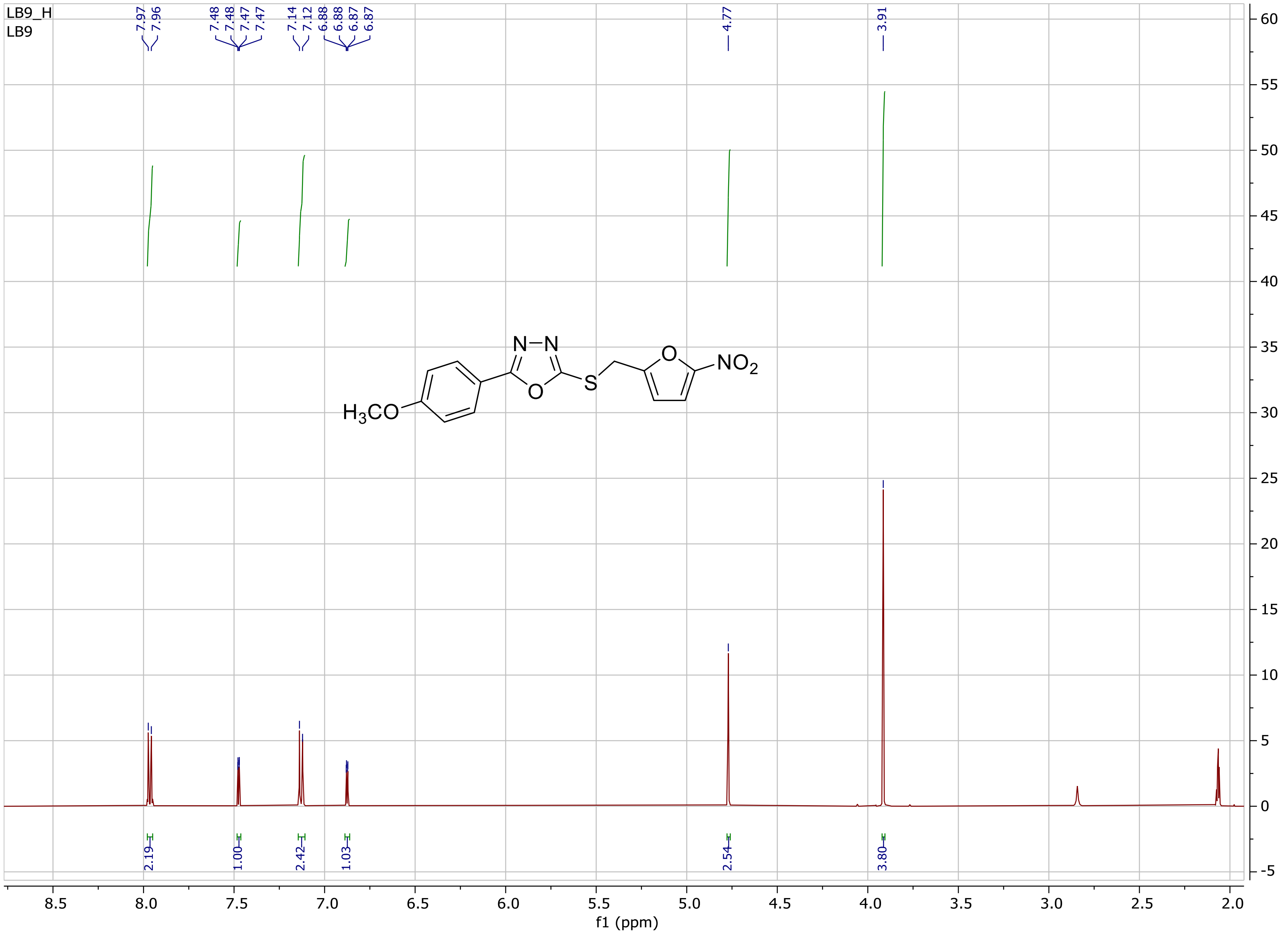

2-(4-Methoxyphenyl)-5-((5-nitrofuran-2-yl)methylsulfanyl)-1,3,4-oxadiazole (**83b**): ^{13}C NMR (126 MHz, Acetone-d_6)

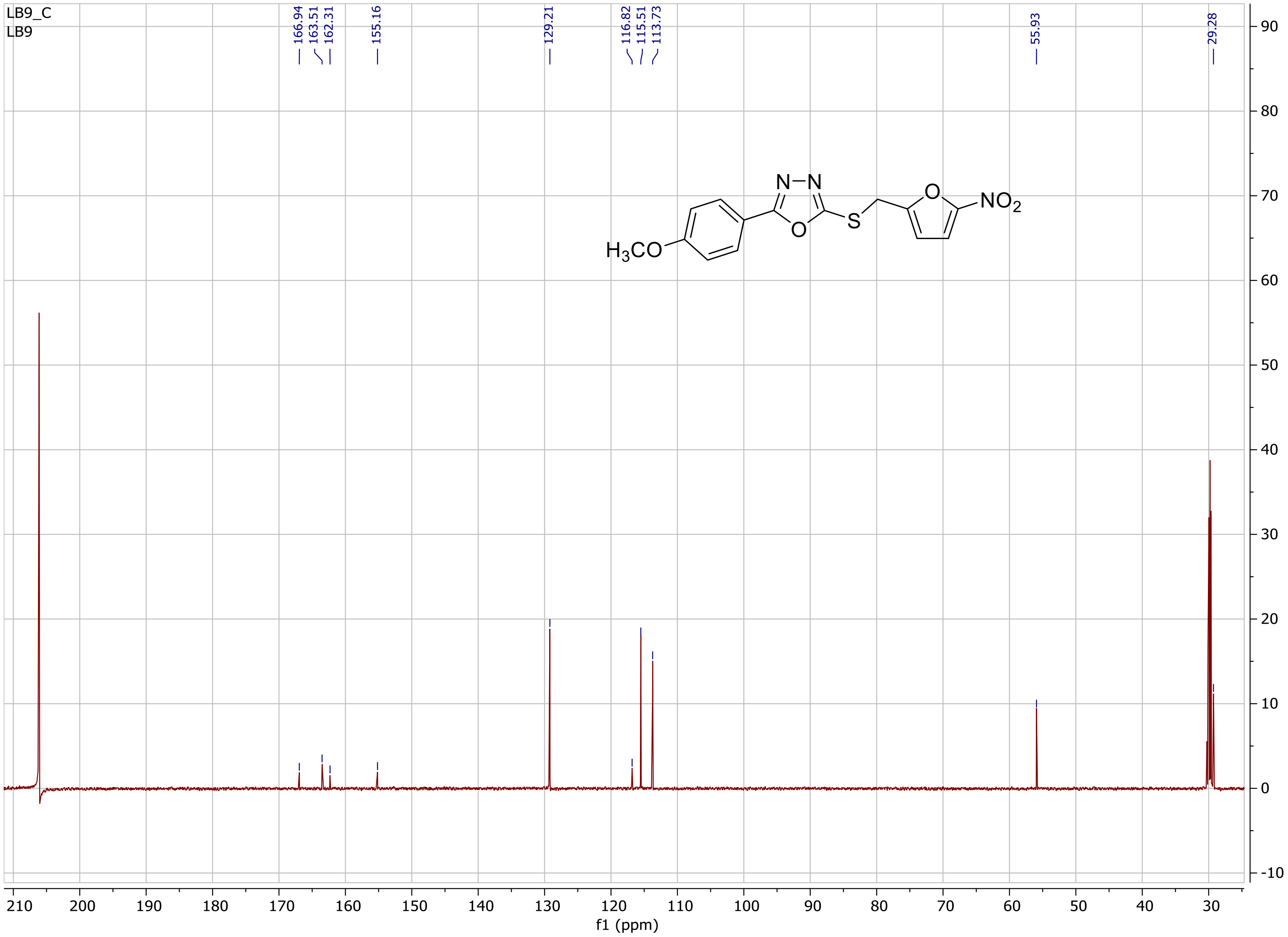

2-(4-Chlorophenyl)-5-((5-nitrofuran-2-yl)methylsulfanyl)-1,3,4-oxadiazole (**83c**): ^{1}H NMR (500 MHz, Acetone-d_6)

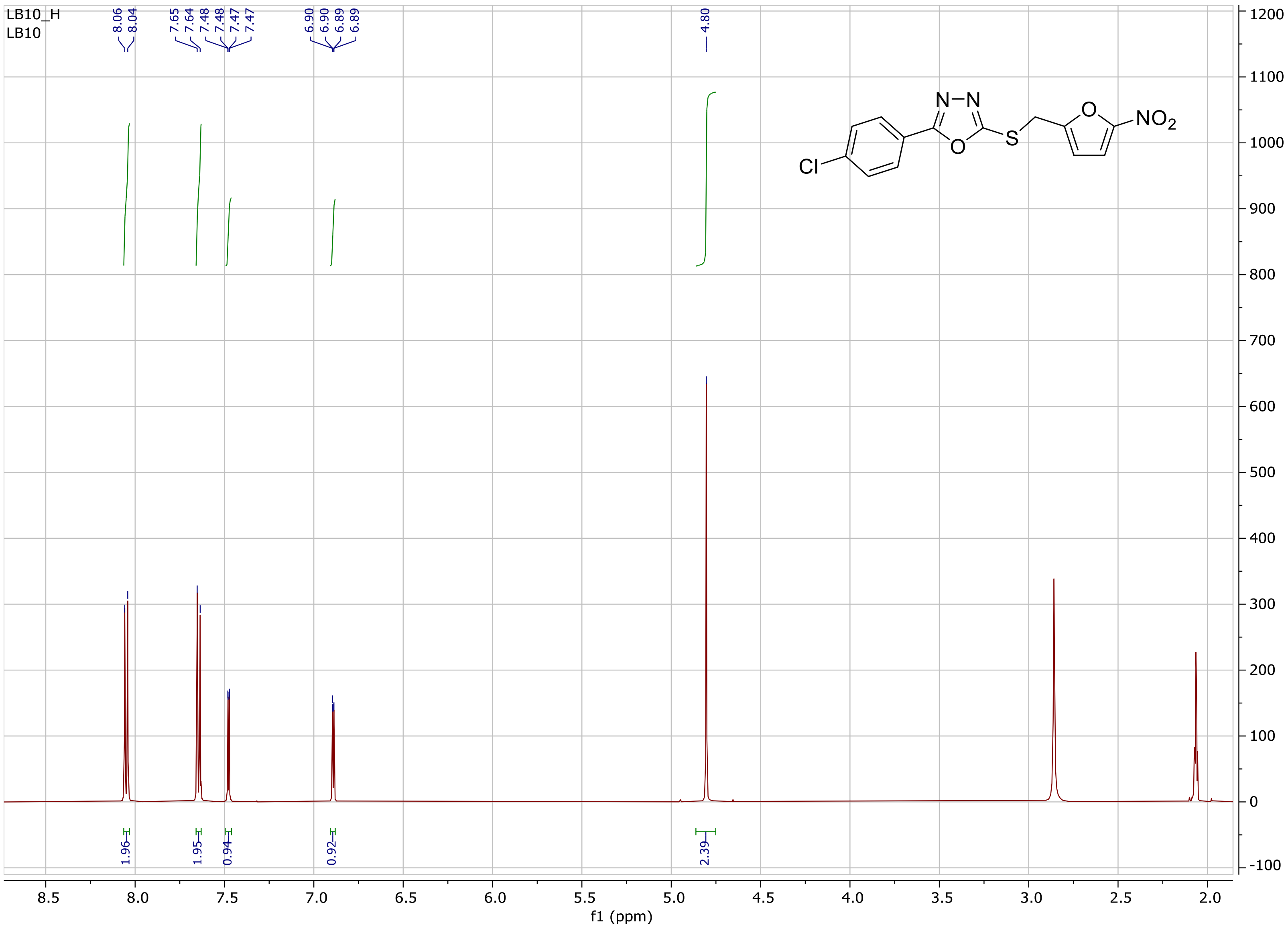

2-(4-Chlorophenyl)-5-((5-nitrofuran-2-yl)methylsulfanyl)-1,3,4-oxadiazole (**83c**): ^{13}C NMR (126 MHz, Acetone-d_6)

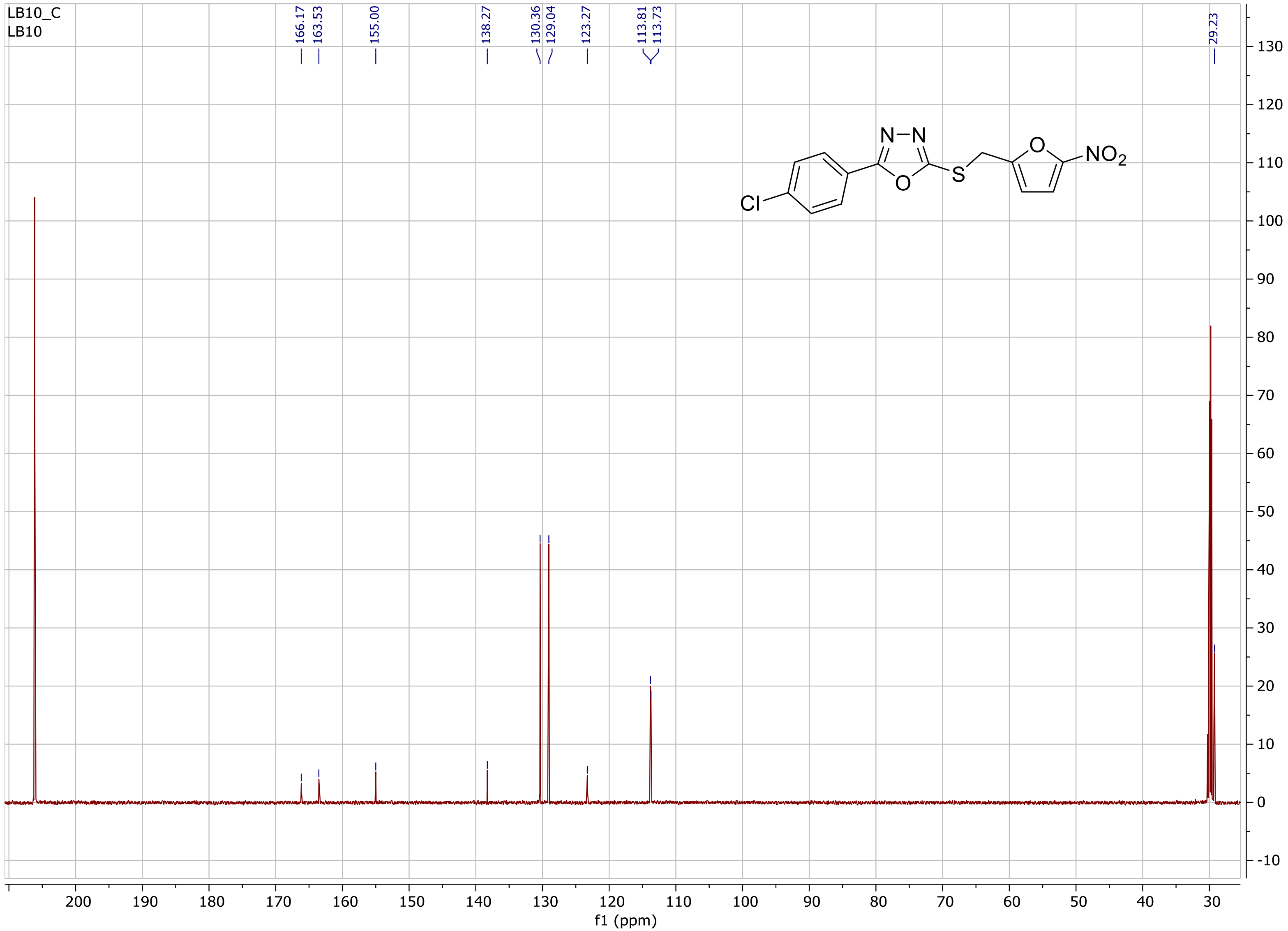

2-(4-Bromophenyl)-5-((5-nitrofuran-2-yl)methylsulfanyl)-1,3,4-oxadiazole (**83d**): ^{1}H NMR (500 MHz, Acetone-d_6)

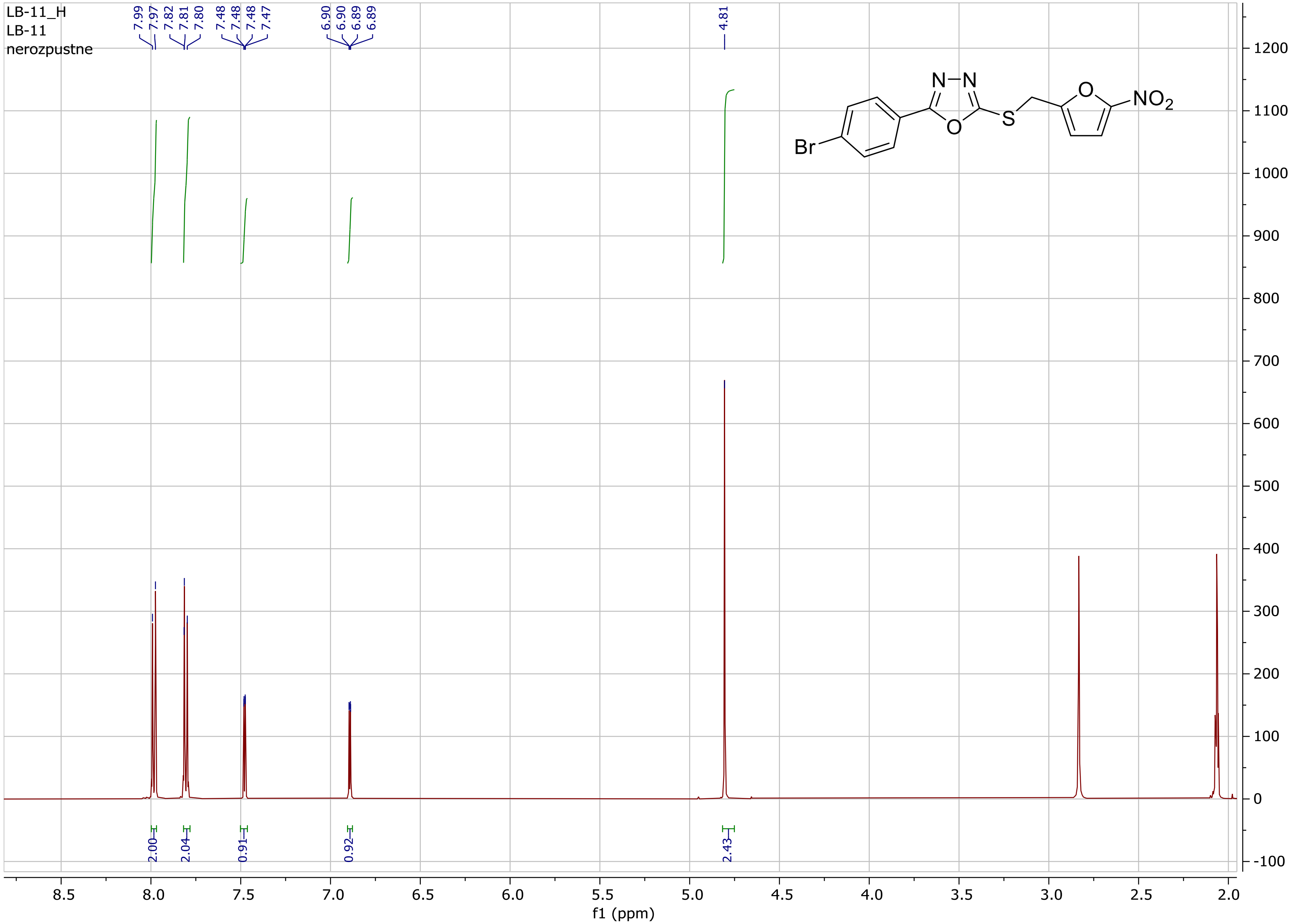

2-(4-Bromophenyl)-5-((5-nitrofuran-2-yl)methylsulfanyl)-1,3,4-oxadiazole (**83d**): ^{13}C NMR (126 MHz, Acetone-d_6)

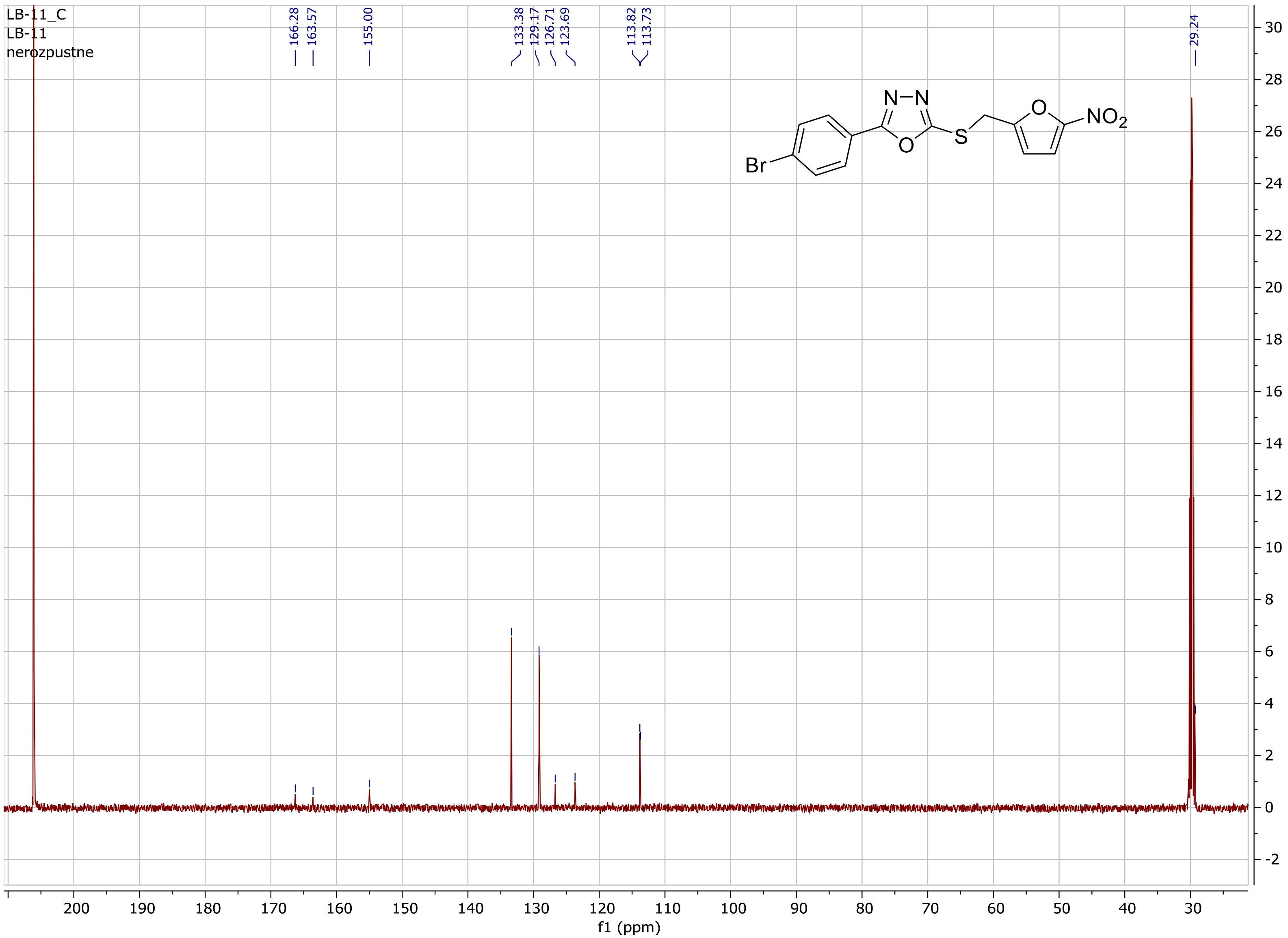

2-Cyclohexyl-5-((5-nitrofuran-2-yl)methylsulfanyl)-1,3,4-oxadiazole (**83e**): ^{1}H NMR (600 MHz, Acetone-d_6)

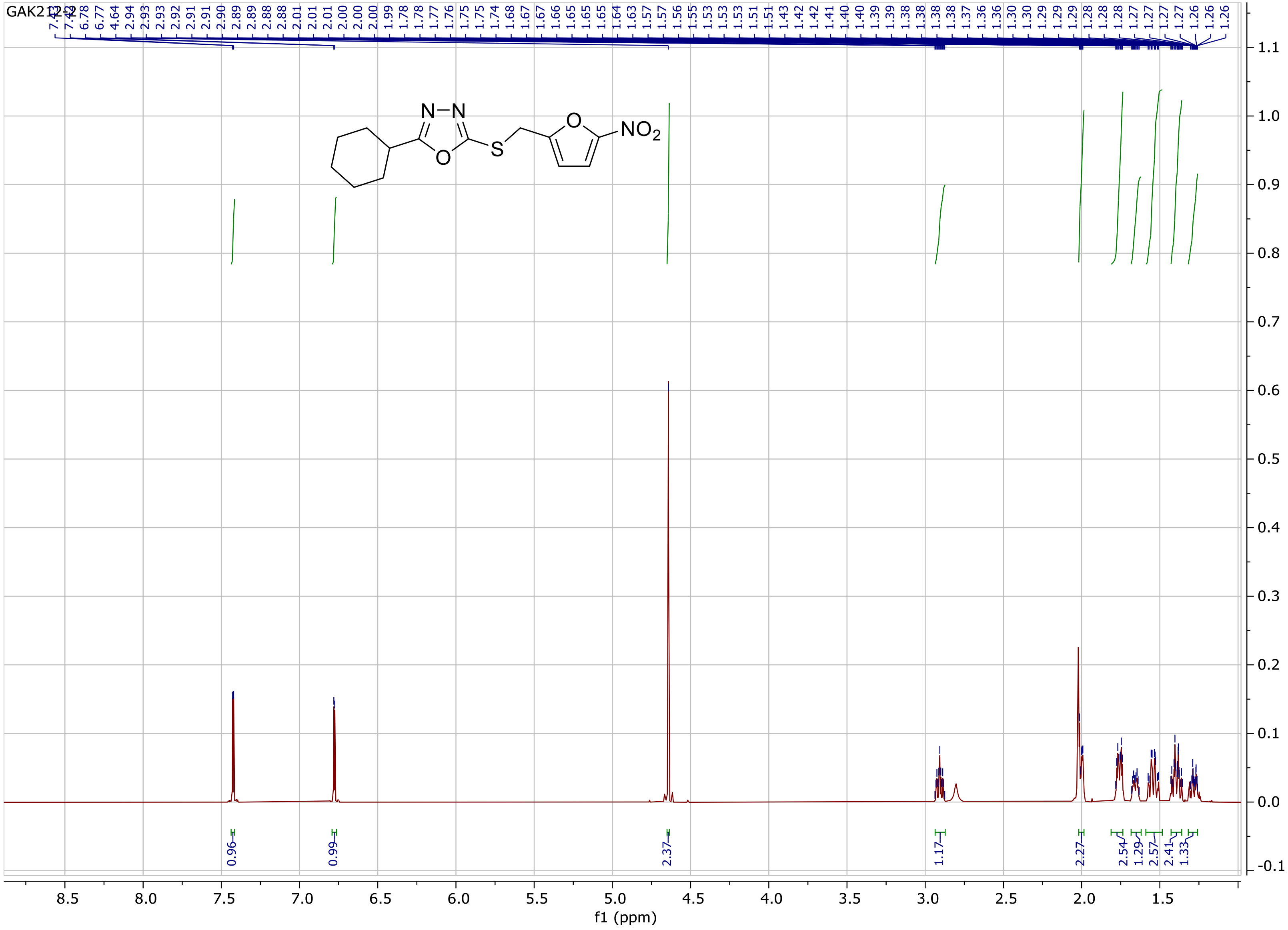

2-Cyclohexyl-5-((5-nitrofuran-2-yl)methylsulfanyl)-1,3,4-oxadiazole (**83e**): ^{13}C NMR (151 MHz, Acetone-d_6)

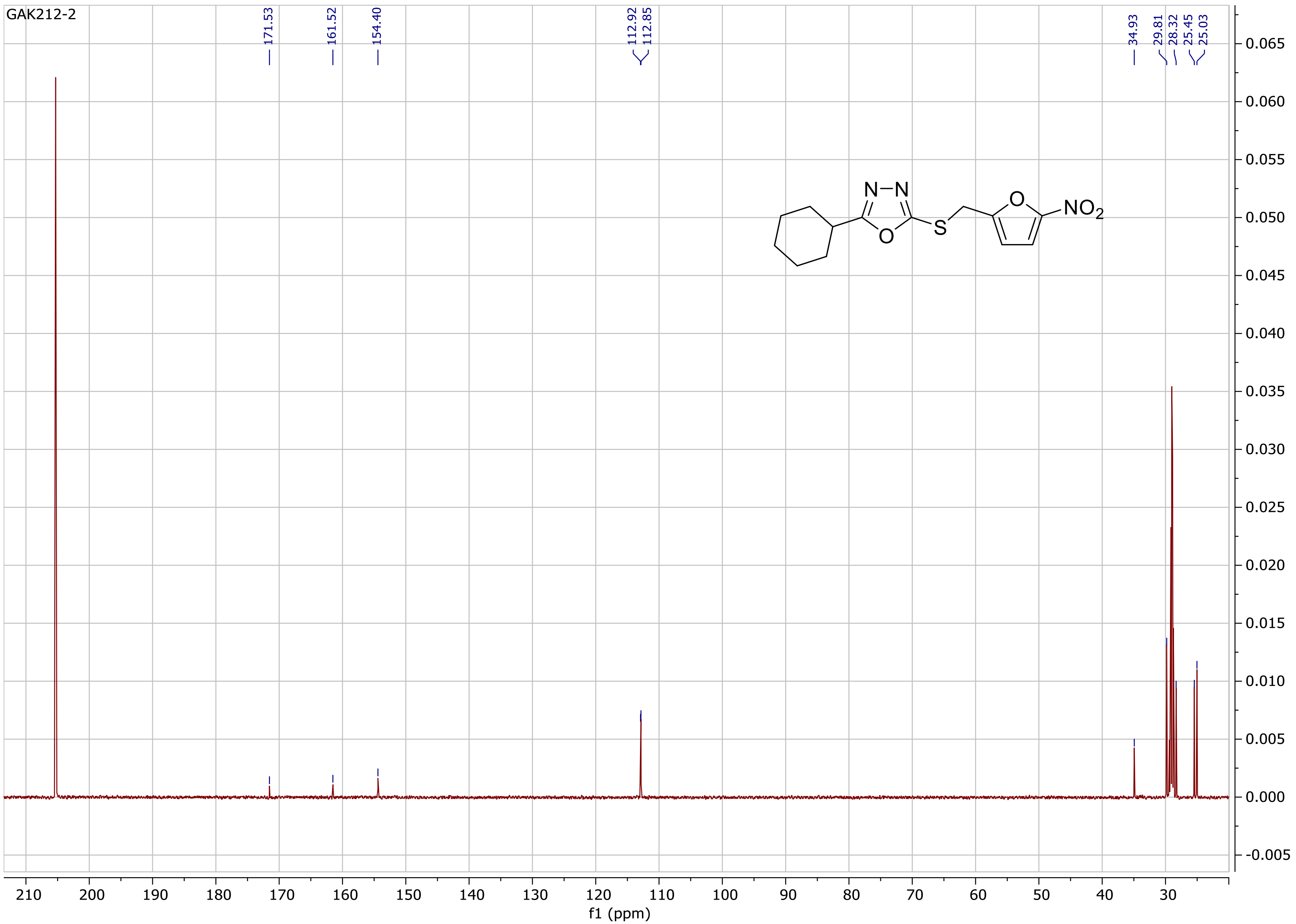

2-Cyclohexyl-5-((5-nitrofuran-2-yl)methylsulfanyl)-1,3,4-oxadiazole (**83e**):

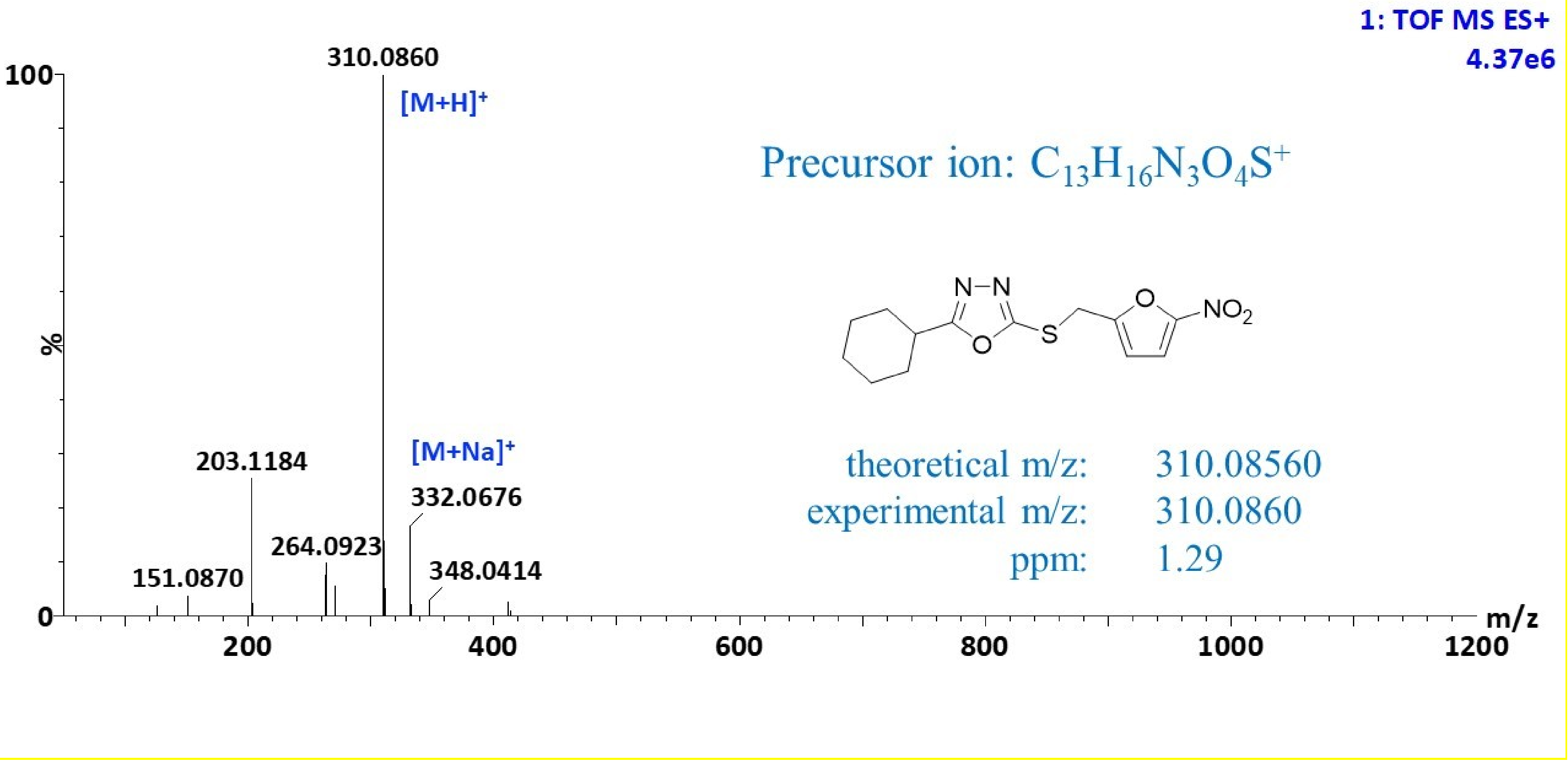

Supplement: Supplementary file 1 — jm3c00925_si_001.pdf [file jm3c00925_si_001.pdf]
